# Supplementary material for: Wheat individual grain-size variance originates from crop development and from specific genetic determinism
Source: PLoS One. 2020 Mar 26;15(3):e0230689. doi: 10.1371/journal.pone.0230689 (PMC7098578; doi:10.1371/journal.pone.0230689)
Supplement: S1 Table — Wheat varieties tested, year of release (YR) and geographical origin (Country). (PDF) [file pone.0230689.s001.pdf]

Name	Country	YR
Apache	FRA	1998
Altigo	FRA	2007
Bermude	FRA	2007
Premio	FRA	2007
Einstein	GBR	2002
KWS Podium	GBR	2009
Ambition	DNK	2004
Lear	GBR	2007
Alchemy	GBR	2005
Amundsen	FRA	2007
Galvano	DNK	2008
Istabraq	GBR	2003
JB Diego	GBR	2006
Manager	FRA	2005
Oxebo	FRA	2009
Pajero	BEL	1995
SY Tolbiac	FRA	2011
Pierrot	DNK	2009
Samurai	FRA	2004
Fairplay	FRA	2011
Selekt	FRA	2006
Viscount	GBR	2007
Warrior	GBR	2009
Xi19	GBR	2001
Antonius	AUT	2006
Ronsard	FRA	2011
Ephoros	DEU	2004
Expert	FRA	2007
Flaubert	FRA	2009
Gladiator	GBR	2002
Glasgow	GBR	2004
Iridium	FRA	2006
Kalahari	DEU	2010
Kalystar	FRA	2010
Koreli	FRA	2006
Marksman	GBR	2006
Pepidor	FRA	2006
Perfector	FRA	2003
Pireneo	AUT	2005
Bergamo	FRA	2011
Racine	FRA	2010
Sankara	FRA	2003
Scor	FRA	2008
Sogood	FRA	2005
Sponsor	FRA	1994
SY Epson	GBR	2011
Trapez	FRA	2009
Allez y	FRA	2010
Aristote	FRA	2010

Attitude	FRA	2009
Attlass	DEU	2005
Barok	FRA	2008
Boisseau	FRA	2007
Boregar	FRA	2007
Camp Remy	FRA	1980
Ch Nara	CHE	2009
Chevalier	AUT	2006
Cordiale	GBR	2003
Crousty	SWE	1995
Dialog	FRA	2007
Dinosor	FRA	2005
Folklor	FRA	2010
Instinct	FRA	2005
Marcelin	FRA	2009
Papageno	AUT	2006
Phare	FRA	2007
Player	FRA	2009
Renan	FRA	1989
Sebasto	FRA	2007
Solution	FRA	2007
Tapidor	FRA	2001
Toisondor	FRA	2003
Odyssee	FRA	2011
Aldric	FRA	2006
Alixan	FRA	2005
Ambello	FRA	2010
Bagou	FRA	2007
Biancor	FRA	2010
Caphorn	FRA	2000
Catalan	FRA	2002
Compil	FRA	2010
Fluor	FRA	2010
Galactic	FRA	2007
Hekto	FRA	2009
Karillon	FRA	2010
Lord	FRA	2008
Laurier	FRA	2011
Orvantis	FRA	2000
Pakito	FRA	2010
Prevert	FRA	2009
Blini	MAR	2012
Rustic	BEL	2005
Sokal	FRA	2011
Sorrial	FRA	2008
Sweet	FRA	2010
Swinggy	FRA	2008
SY Mattis	FRA	2010
Figaro	FRA	2011
Aligator	FRA	2009

Altamira	FRA	2009
Amador	FRA	2009
Aprilio	FRA	2010
Aramis	FRA	2009
Arezzo	FRA	2007
Arkeos	FRA	2010
Arlequin	FRA	2007
Athlon	FRA	2009
Aubusson	FRA	2001
Bastide	FRA	2002
Buenno	FRA	2007
SY Moisson	FRA	2011
Celestin	FRA	2009
Epidoc	FRA	2005
Euclide	FRA	2006
Farinelli	FRA	2010
Oregrain	FRA	2011
Cellule	FRA	2011
Flamenko	FRA	2010
Galpino	ESP	2010
Goncourt	FRA	2008
Graindor	FRA	2005
Hisseo	ITA	2010
Illico	FRA	2009
Innov	FRA	2005
Isengrain	FRA	1997
MH 09-17	FRA	NA
Musik	FRA	2010
Ascott	FRA	2011
Paledor	FRA	2004
Rubisko	FRA	2011
Saint Ex	FRA	2010
Scenario	FRA	2010
Soissons	FRA	1987
Solehio	FRA	2008
SY Alteo	FRA	2010
Tremie	FRA	1992
Tulip	FRA	2010
Uski	FRA	2008
Accor	FRA	2006
Accroc	FRA	2009
Acienda	FRA	2004
Adhoc	FRA	2010
Altria	FRA	1996
Andalou	FRA	2001
Autan	FRA	2000
Azimut	FRA	2004
Bologna	ESP	2001
Cezanne	FRA	1998
Courtot	FRA	1974

Eureka	FRA	1992
Exotic	FRA	2005
Galopain	FRA	2009
Garcia	FRA	2005
Jaguar	ITA	2009
Miroir	FRA	2010
Artdeco	FRA	2011
PR22R58	ITA	2002
Primo	ITA	2009
Quality	ITA	2002
Rimbaud	FRA	2009
Royssac	FRA	2002
Sirtaki	ITA	2007
Soledad	ITA	2008
Sollario	FRA	2007
Basmati	ITA	2011
Tigre	POR	1996
Calisol	FRA	2012
Isidor	FRA	2002
Ardelor	FRA	2010
Deucendeu	FRA	1997
Eperon	FRA	2012
Behert	FRA	1995
Blason	FRA	1977
Genesis	FRA	1992
Grenier	FRA	1992
Amifort	FRA	1992
Sofolk CS	FRA	2014
Aigle	FRA	2014
Aplomb	FRA	2014
Armada	FRA	1978
Bonifacio	FRA	2011
Calabro	FRA	2011
Calumet	FRA	2013
Sothys CS	FRA	2014
RGT Mondio	FRA	2014
Picador	FRA	2006
Audace	FRA	1996
Sherlock	FRA	2014
Auckland	FRA	2014
Element	AUT	2006
Foxyl	FRA	2014
Lavoisier	FRA	2013
Lennox	FRA	2012
Nemo	FRA	2014
Plainedor	FRA	2009
Quatuor	FRA	2002
RGT Venezio	FRA	2013
Diamento	FRA	2012
Syllon	FRA	2013

Descartes	FRA	2013
Collector	FRA	2014
Belepi	FRA	2012
Chevron	FRA	2009
Contrefor	FRA	2010
Fructidor	FRA	2013
Ghayta	FRA	2012
Granamax	FRA	2013
Hendrix	FRA	2011
Kundera	FRA	2013
Lyrik	FRA	2011
Minotor	FRA	2007
RGT Tekno	FRA	2014
Skerzzo	FRA	2011
Triomph	FRA	2014
Orfield	FRA	1998
Gallixe	FRA	2014
Gotik	FRA	2014
Grapeli	FRA	2012
Noblesko	FRA	2011
Phileas	FRA	2014
Quebon	FRA	2004
RGT Texaco	FRA	2014
Terroir	FRA	2012
Baltimor	FRA	1998
Mandragor	FRA	2012
Popeye	FRA	2014
Costello	FRA	2014
Paindor	FRA	1996
