# Supplementary material for: Wheat individual grain-size variance originates from crop development and from specific genetic determinism
Source: PLoS One. 2020 Mar 26;15(3):e0230689. doi: 10.1371/journal.pone.0230689 (PMC7098578; doi:10.1371/journal.pone.0230689)
Supplement: S2 Table — Wheat varieties tested, year of release (YR) and geographical origin (Country). (PDF) [file pone.0230689.s002.pdf]

Name	Country	YR
A_15	AFG	NA
KLEIN_32	ARG	1932
KL_ESTRELLA	ARG	1995
SWM89Y074	ARM	NA
ATUT_II	AUT	1974
DANUBIUS	AUT	1974
KRISTALL	AUT	1983
NEUHOF_1	AUT	1969
OENUS	AUT	1977
P4523_80	AUT	NA
PROBSTDORF	AUT	1982
PROBSTDORF	AUT	1967
PROBSTDORF	AUT	1969
PROTEKTOR	AUT	1974
RINNER_WIN	AUT	1972
VERBESSERTE	AUT	1962
REICHERSBER	AUT	1955
FERDINAND	AUT	1982
CAPO	AUT	1990
PROBSTDORF	AUT	1963
ALBATROS	BEL	1950
ALTER_DE_G	BEL	NA
PRECOCE_DE	BEL	1930
RENVAL	BEL	1977
CAPITAINE	BEL	1982
2109_36	BGR	1976
2678_6	BGR	NA
2838_39	BGR	NA
KREMENA	BGR	1978
K14	BGR	NA
KRAPETZ	BGR	1982
SADOVO_SUF	BGR	1979
SADOVO_1	BGR	1972
TRAYANA	BGR	1981
830_22	BGR	NA
HARUS	CAN	1985
CDC_CLAIR	CAN	1997
ARINA	CHE	1981
BERNINA	CHE	1983
CH73185	CHE	1980
CH73241	CHE	1979
CH73425	CHE	1979
CH73641	CHE	1982
FERMO	CHE	1978
TAMBO	CHE	1985
CAMINO	CHE	1993
IWWRN_85_1	CHL	NA
CAR1608	CHL	NA
QUENOA_INI	CHL	NA

DANUBIA	CZE	1984
OSKA	CZE	1971
BRANKA	CZE	1988
SOLARIS	CZE	1976
BLAVA	CZE	1992
ALKA	CZE	1995
BRUTA	CZE	1994
MONA	CZE	1994
SIRIA	CZE	1994
SIDA	CZE	1993
SIMONA	CZE	1991
SOFIA	CZE	1985
VLADA	CZE	1990
REGINA	CZE	1981
ALMUS	DEU	1973
CAROLUS	DEU	1985
DISPONENT	DEU	1980
GRANADA	DEU	1980
HOCHLAND	DEU	1955
HOLZAPFELS_	DEU	1917
TENOR	DEU	1960
TRUBILO	DEU	1948
TRANE	DEU	1994
PETRUS	DEU	1996
BOVICTUS	DEU	1993
RONOS	DEU	1988
BONTARIS	DEU	1990
MILLER	DNK	2001
MANERO	ESP	NA
SAN_RAFAEL	ESP	1964
SANSA	ESP	1988
VAKKA	FIN	1953
DI37_12_2	FRA	NA
DI50_12	FRA	NA
ABEL	FRA	1961
ABO	FRA	1977
ALCY_CAMBI	FRA	1956
ALTERNA_TO	FRA	1957
ALTO	FRA	1975
AUBE	FRA	1960
BALI	FRA	1975
BIZEL	FRA	1966
BRACO	FRA	1977
CAPET	FRA	1989
CEREALOR	FRA	1964
CHAMBORD	FRA	1960
CORSODOR	FRA	1987
COTE_D_OR	FRA	1937
COURTAL	FRA	1943
DECLIC	FRA	1985

DI7003_1_12	FRA	NA
DI7202_103	FRA	NA
DI7210_15_1	FRA	NA
ECRIN	FRA	1985
ESPOIR	FRA	1950
FANION	FRA	1978
FOISON	FRA	1985
FROIDURE	FRA	1960
GELPA	FRA	1967
GERMINAL	FRA	1969
GH126	FRA	NA
HEURTEBISE	FRA	1954
HUGO	FRA	1988
LANGUEDOC	FRA	1962
MONTJOIE	FRA	1969
NECTAR	FRA	1985
PRESTIGE	FRA	1969
PRIMEPI	FRA	1962
PROQUAL	FRA	1959
RALLYE	FRA	1963
RIGOUDI	FRA	1966
TARASQUE	FRA	1981
TORRIL	FRA	1989
ULM	FRA	1983
MAXIMUM_(	FRA	1936
AJAX	FRA	1994
BOURGOGNE	FRA	1995
NOBLET	FRA	1995
ORMIL	FRA	1996
VIENOY	FRA	1994
BEHERT	FRA	1994
TALDOR	FRA	1997
ASVM4_BEAU	FRA	1994
COMTAL	FRA	1973
CORIN	GBR	1976
CWW1857_1	GBR	1980
FLORIO	GBR	1993
HOLDFAST	GBR	1936
PAGEANT	GBR	1981
STETSON	GBR	1981
MADRIGAL	GBR	1995
MALACCA	GBR	1997
SIROKA	HRV	1984
ZG_IPK_4387	HRV	NA
ZG1004_82	HRV	1982
ZG7865_83	HRV	1983
CERERA	HRV	1993
ZITARKA_1	HRV	1985
ZG3073_84	HRV	1984
ARPADHALOI	HUN	NA

HERKULES	HUN	NA
GK_CSUROS	HUN	1991
GK_KALAKA	HUN	NA
HATVANI_40_	HUN	NA
KARCAGI_149	HUN	1967
MV03_86	HUN	1986
MV06_80	HUN	1980
MV17_86	HUN	1986
MV23_77	HUN	1978
MV30_86	HUN	1986
MV33_86	HUN	1986
MV119_88	HUN	1988
GK_SAGVARI_	HUN	NA
MV_MAGVAS	HUN	1998
MV_MARTIN_	HUN	1998
GK_ZUGOLY	HUN	1994
MV233_99	HUN	1999
MV309_99	HUN	1999
MV314_99	HUN	1999
MV_EMMA	HUN	1994
MV_VEKNI	HUN	NA
OWL_Col_No	IRN	NA
AUTONOMIA	ITA	1938
COLORBEN_4	ITA	1966
F51	ITA	1961
IDRA	ITA	1994
JACOMETTI_4	ITA	1966
OVEST	ITA	1961
ROMA	ITA	1935
SALTO	ITA	1939
SAN_MAURO	ITA	1990
SALMONE	ITA	1980
LORETO	ITA	1982
DEMAR_4	ITA	1965
HOKUEI	JPN	1965
HOROSHIRI_K	JPN	1974
MUTSUBENK	JPN	1950
TAISETSU_KO	JPN	1990
BONNY	KEN	1966
BERMET	KGZ	1998
ERYTHROSPE	KGZ	1979
TILEK	KGZ	2001
ALMA	LTU	NA
OBRII_DNEST	MDA	NA
SKOPJANKA	MKD	1982
APOLLO	NLD	1958
BEAUBOURG	NLD	1993
SEMPER	NLD	1998
RIDA	NOR	1977
PEGASUS	NZL	1985

KOTARE	NZL	1984
DANKOWSKA	POL	1920
JANA	POL	1975
LUNA	POL	1970
ZETA	POL	1978
DACIA	ROU	1971
FAVORIT	ROU	1970
LOVRIN_29	ROU	1975
MOLDOVA	ROU	1970
TURDA_95	ROU	1995
ANIVERSAR	ROU	1986
MAGISTRAL	ROU	1998
DRUZHBA	RUS	1981
ERSHOVSKAJ/	RUS	1973
KRASNODARS	RUS	1973
BELGORODSK	RUS	1997
PAMYATI_FE	RUS	1993
ERMAK	RUS	2001
PALPICH	RUS	2004
SELYANKA	RUS	2002
MALYSA	SVK	1998
RUBIS	SWE	1962
TIMMO	SWE	1982
ES14_SITTA_	TCA	NA
308_02_2_W	TCA	NA
BILINMIYEN9	TCA	NA
TCI962395	TCA	NA
AKULA_BONI	TCA	NA
BONITO__KA	TCA	NA
GONCHA	TKM	NA
GARAGUM	TKM	2000
WWL860104	TUR	NA
CIT89073	TUR	NA
GEREK79	TUR	1979
TCI960735	TUR	NA
LUTESCENS_(	UKR	1984
LUTESCENS_(	UKR	1985
LUTESCENS_E	UKR	1984
MIRONOVSK/	UKR	1960
OBRII	UKR	1983
VESELOPODC	UKR	1938
DONCHANKA	UKR	1995
LUTESCENS_7	UKR	1991
DONETSKAYA	UKR	1990
MIRONOVSK/	UKR	1995
MIRONOVSK/	UKR	1992
MIRLEBEN	UKR	1993
FEDOROVKA	UKR	1993
ODESKA_161	UKR	1995
ODESKA_267	UKR	1997

MIRONIVSKA	UKR	1999
KHVYLIA	UKR	1995
ARGEE	USA	1976
BENNI	USA	1980
BOLAL	USA	1970
BUCKSKIN	USA	1973
CARSON	USA	1986
CHARCOAL_E	USA	1977
CHARMANY	USA	1984
COLT	USA	1983
COMPACT	USA	1974
DOWNY	USA	1976
HIPLAINS	USA	1973
MCNAIR_158	USA	1973
MCNAIR_701	USA	1973
MONON	USA	1959
NUGAINES	USA	1965
OMAR	USA	1955
PAYNE	USA	1977
REDLAND	USA	1986
ROSE	USA	1981
SALUDA	USA	1983
SCOUT_66	USA	1967
SENTINEL	USA	1973
STACY	USA	1980
STADLER	USA	1964
STODDARD	USA	1973
TAM_108	USA	1984
TECUMSEH	USA	1974
UTE	USA	1983
WHEELER	USA	1980
YORKSTAR	USA	1968
NORWIN	USA	1984
ARTHUR	USA	1968
ROD	USA	1992
MACNAIR181	USA	1981
LONGHORN	USA	1991
PRONGHORN	USA	1996
MCGUIRE	USA	1996
RAMPART	USA	1996
GR915	USA	1991
P29	USA	1996
PROWERS_9	USA	1999
TAMEX	USA	1980
COUGAR	USA	1999
PAT	USA	2001
EDWIN	USA	1999
ADENA	USA	1984
HOSAR	USA	1930
SUSQUEHANI	USA	1988

MUSTANG	USA	1983
ALBIDUM	UZB	1974
AGROUNIA	YUG	1987
NS_RANA_1	YUG	1975
SUTJESKA	YUG	1979
ZITNICA	YUG	1982
UNA	YUG	1983
CRVENA_ZVE	YUG	1967
