# Supplementary figures and images for: Wheat individual grain-size variance originates from crop development and from specific genetic determinism
Source: PLoS One. 2020 Mar 26;15(3):e0230689. doi: 10.1371/journal.pone.0230689 (PMC7098578; doi:10.1371/journal.pone.0230689)

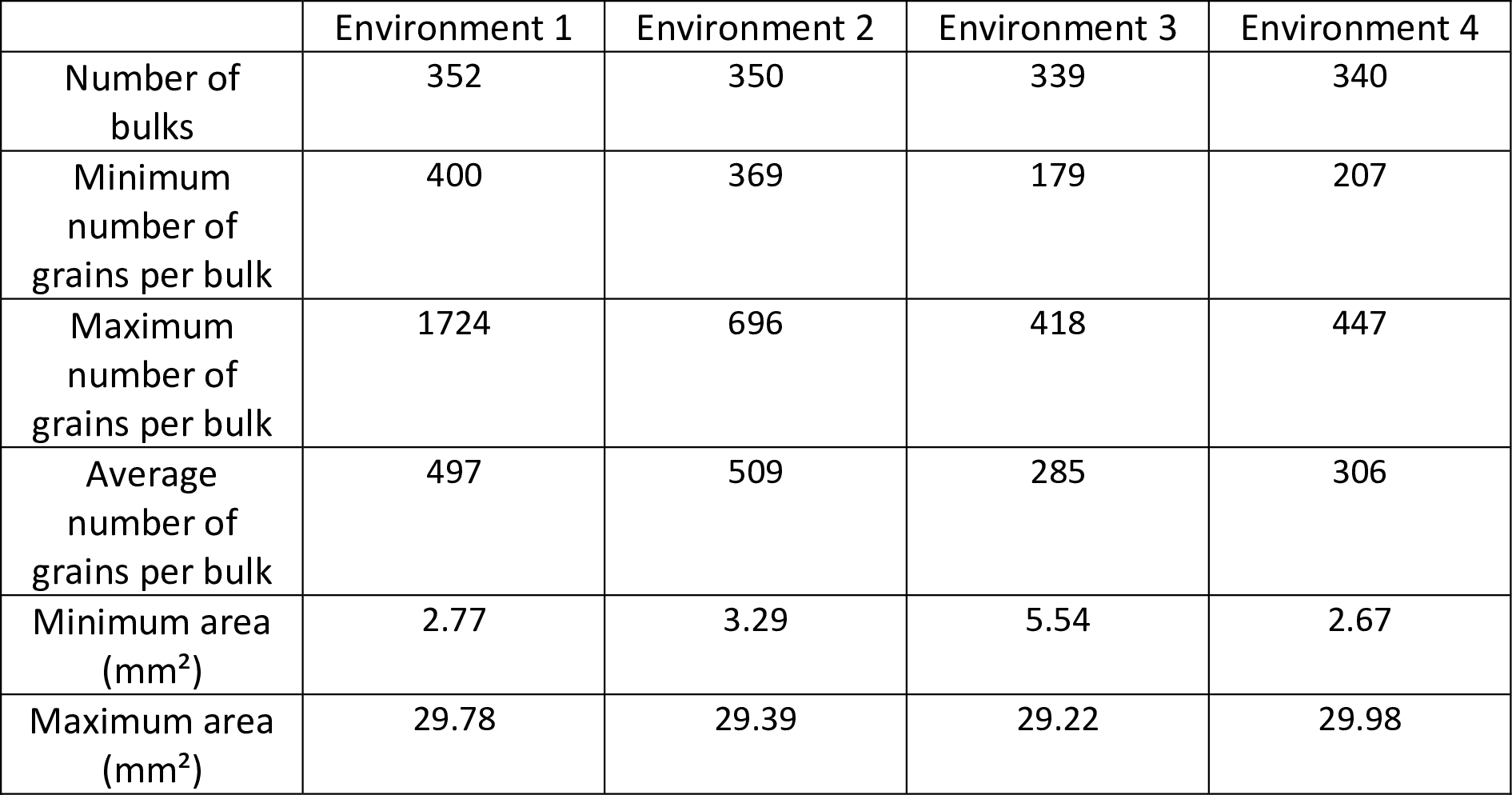

Supplement: S3 Table — For each environment, descriptive statistics were calculated for all grains composing the bulk samples harvested on each micro-plot. E1 (well-watered, 2016), E2 (water-deficit, 2016), E3 (well-watered, 2017) and E4 (water-deficit, 2017). (TIF) [file pone.0230689.s003.tif]

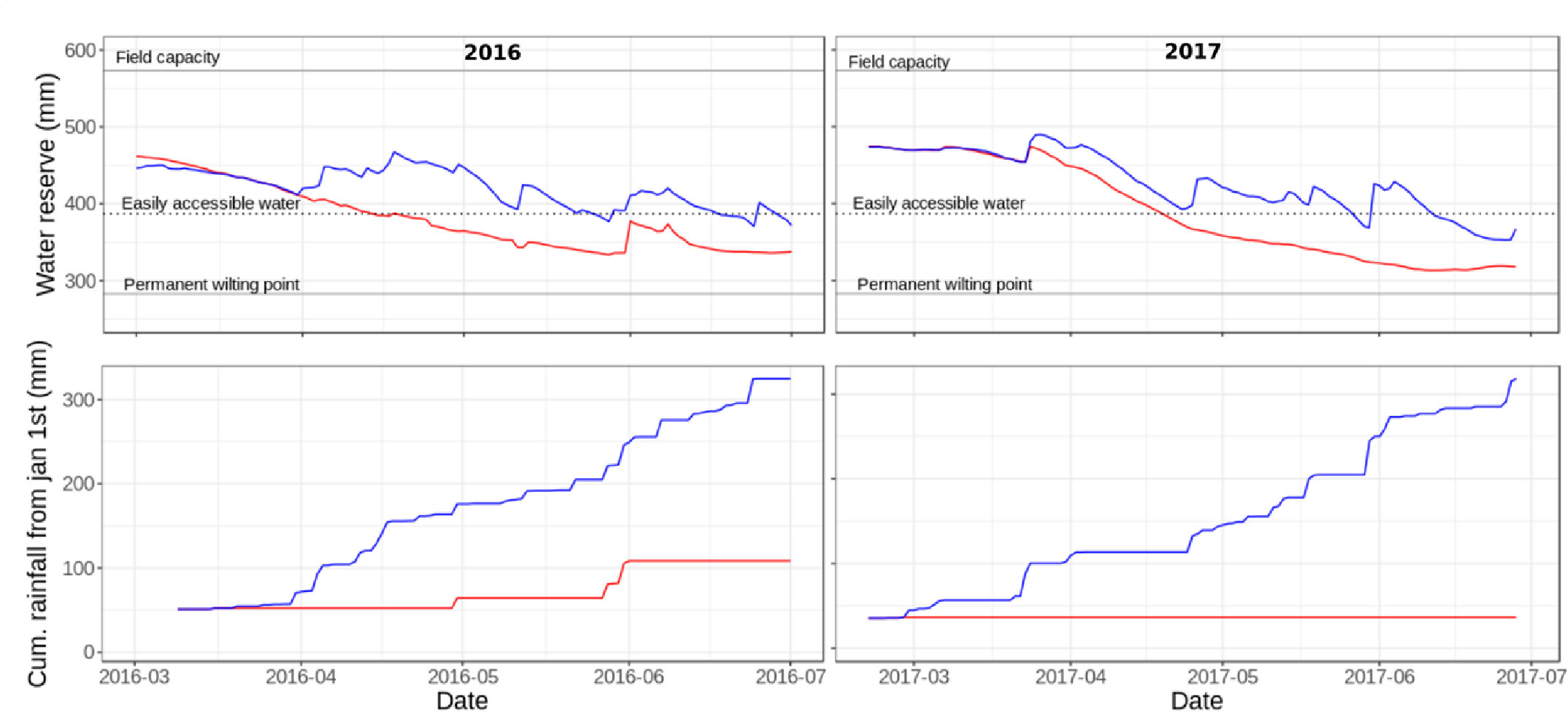

Supplement: S1 Fig — Soil field capacity, limit of easily accessible water and permanent wilting point were calculated from soil water retention characteristics determined over the soil profile. (TIF) [file pone.0230689.s007.tif]

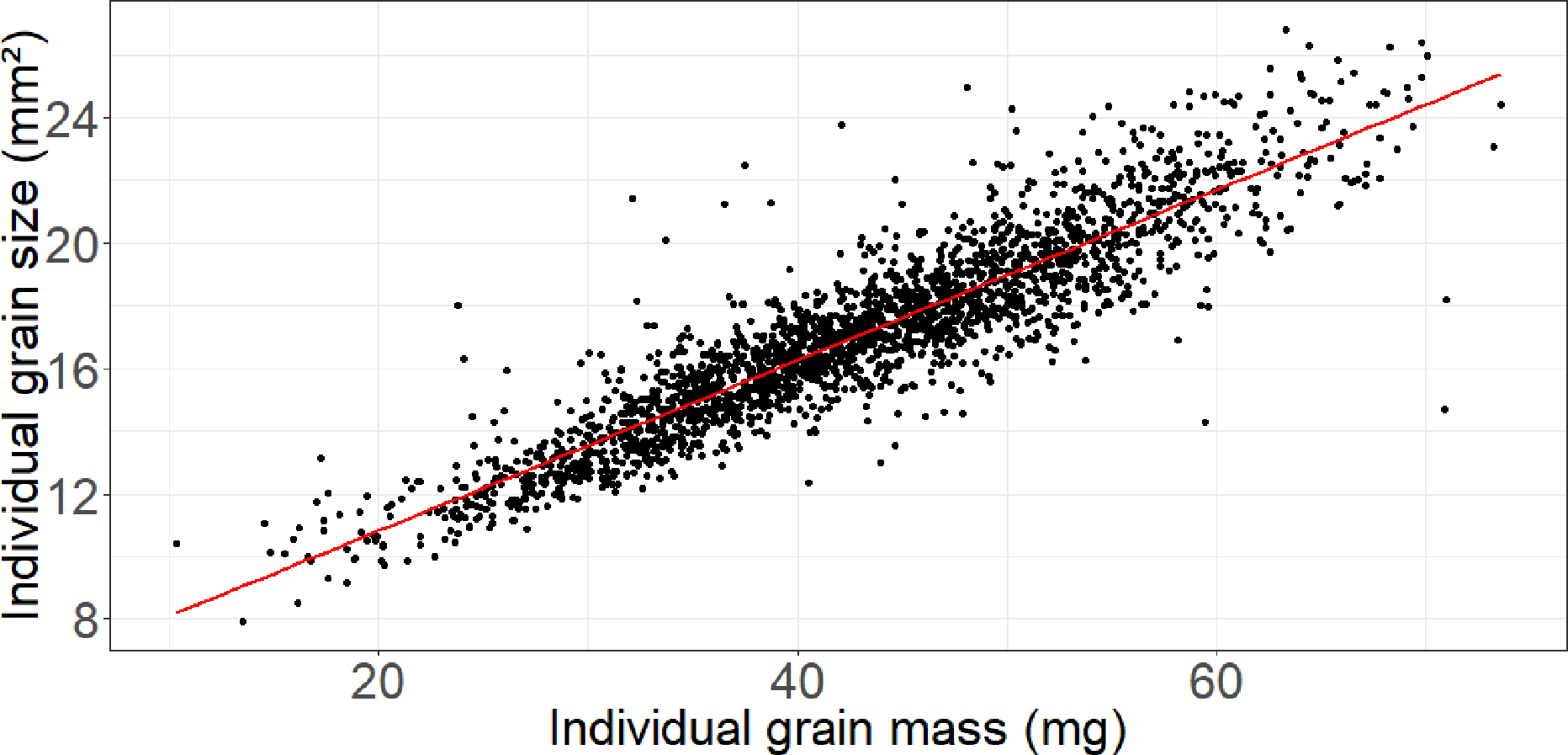

Supplement: S2 Fig — The red diagonal represents the linear regression between individual grain projected area and individual grain mass (R2 = 0.83). (TIF) [file pone.0230689.s008.tif]

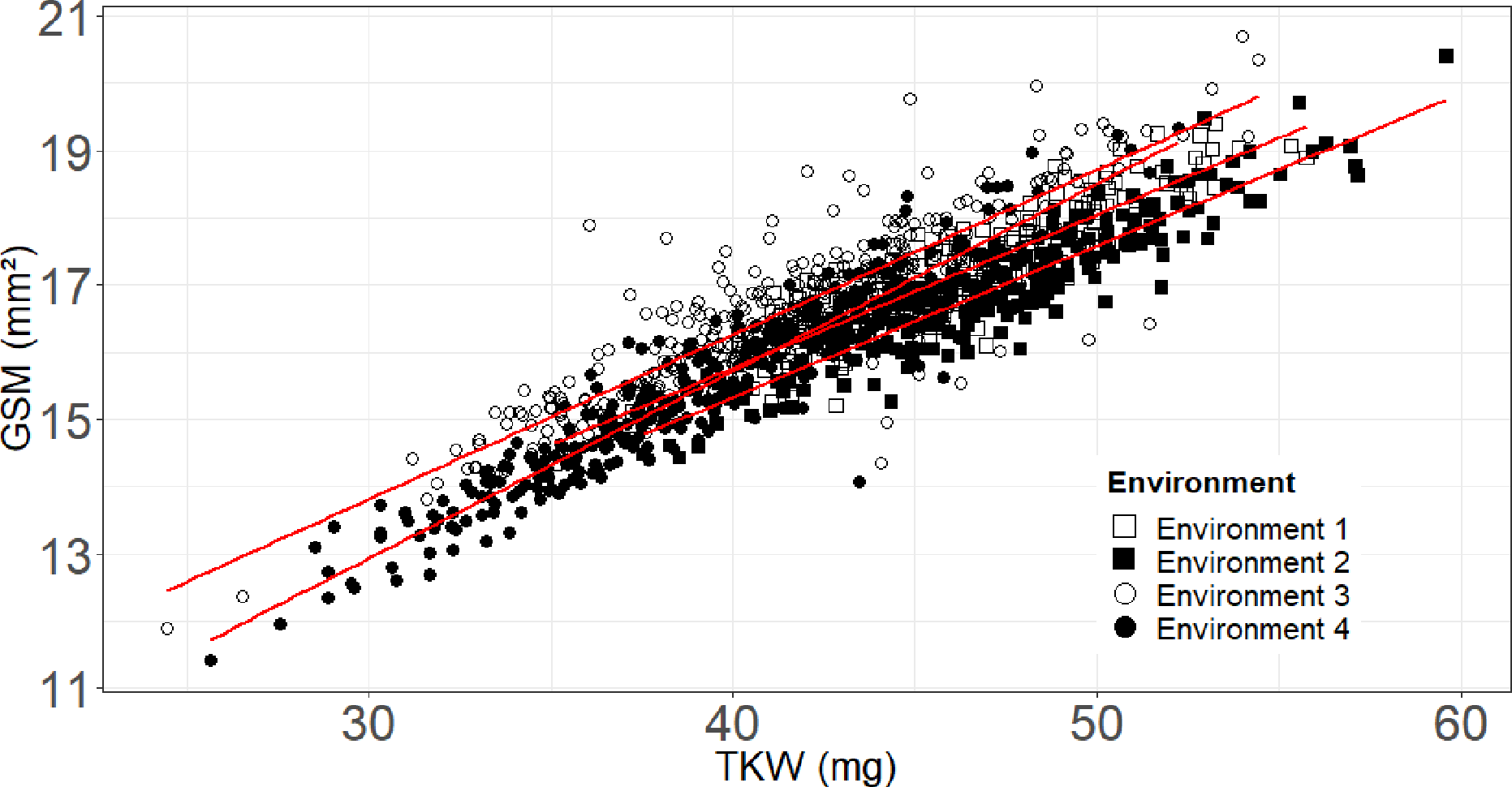

Supplement: S3 Fig — The red diagonal represents the linear regressions between GSM and TKW for each of four environments. E1 (well-watered, 2016): R2 = 0.81; E2 (water-deficit, 2016): R2 = 0.83; E3 (well-watered, 2017): R2 = 0.78; E4 (water-deficit, 2017): R2 = 0.89. (TIF) [file pone.0230689.s009.tif]

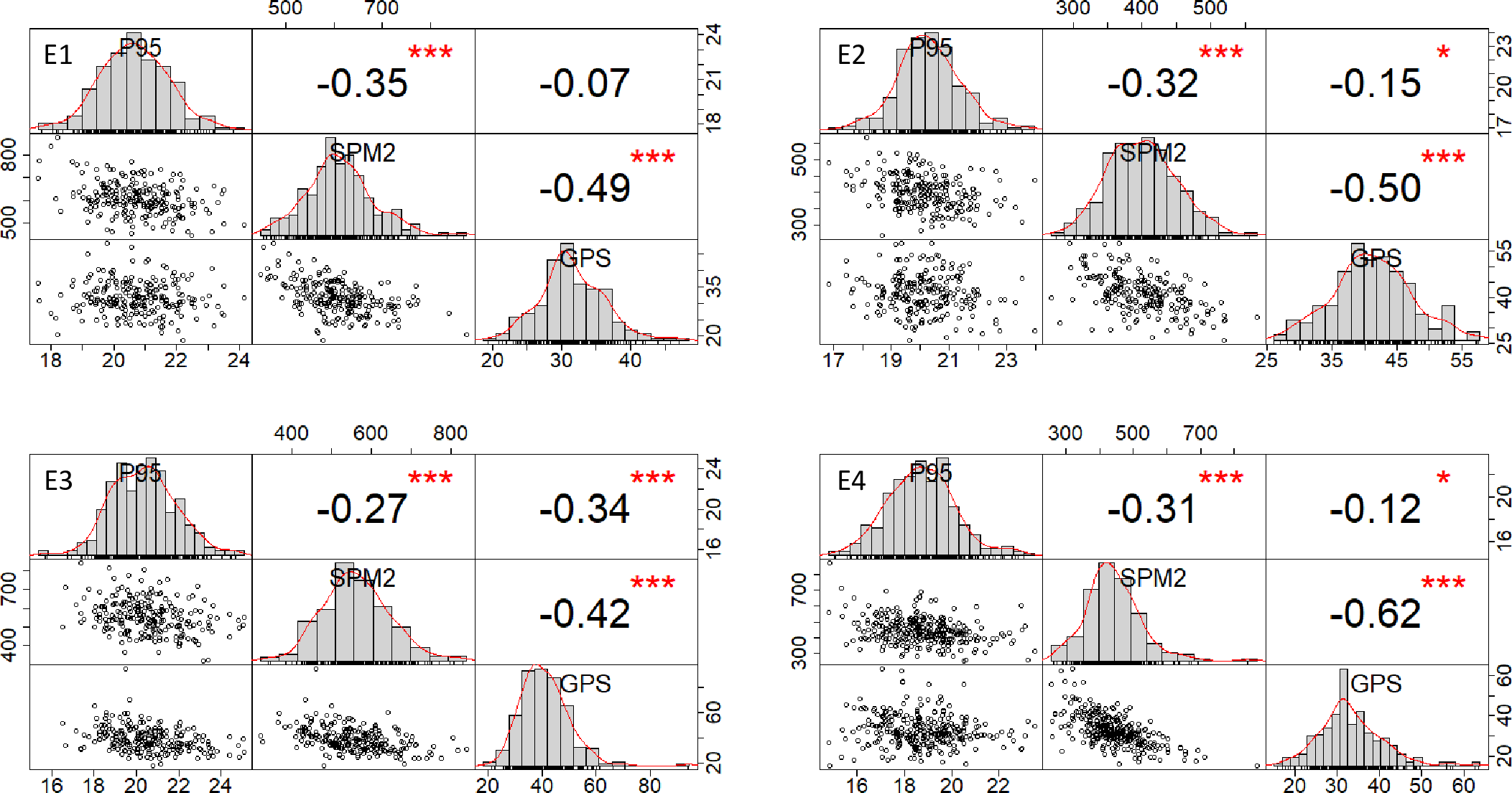

Supplement: S4 Fig — The diagonal panel shows histograms of each trait. The lower and upper triangular panels show, respectively, the scatter plot and the Pearson correlation coefficient between the two traits. P95: 95th percentile of grain sizes, SPM2: number of spikes per m2, GPS: number of grains per spike. E1 (well-watered, 2016), E2 (water-deficit, 2016), E3 (well-watered, 2017) and E4 (water-deficit, 2017). ‘.’: P-value<0.1; ‘*’: P-value<0.05; ‘**’: P-value<0.01; ‘***’: P-value<0.001. (TIF) [file pone.0230689.s010.tif]
