# Supplementary material for: Wheat individual grain-size variance originates from crop development and from specific genetic determinism
Source: PLoS One. 2020 Mar 26;15(3):e0230689. doi: 10.1371/journal.pone.0230689 (PMC7098578; doi:10.1371/journal.pone.0230689)
Supplement: S4 Table — We identify and report the characteristics of each chromosomal zone of interest (64 QTL associated with grain size variance (GSV)). (1)Number of SNP associated with a trait in this chromosomal zone, (2)Maximum LOD score on the significant associated SNP, (3) Physical position on the chromosome (Chr) [36] (4)Minor allele frequency (MAF), (4)Expressed in terms of the absolute value for each trait. E1 (well-watered, 2016), E2 (water-deficit, 2016), E3 (well-watered, 2017) and E4 (water-deficit, 2017). (PDF) [file pone.0230689.s004.pdf]

Zone	Chr	Trait	Env	Nb.SNP[(1)]	LOD.score.max[(2)]	Position[(3)]		MAF[(4)]		Effect[(5)]	
						Min	Max	Mean	SD	Mean	SD
1	1A	GSV	E1	70	4.05	0.00E+00	5.53E+08	0.20	0.09	-0.47	0.11
1	1A	SPM2	E1	17	4.54	0.00E+00	5.53E+08	0.27	0.08	-5.40	20.30
1	1A	GPS	E1	3	3.14	0.00E+00	5.53E+08	0.10	0.00	-1.89	0.00
1	1A	GPM2	E1	23	3.82	0.00E+00	5.53E+08	0.27	0.03	-684.46	37.92
1	1A	TKW	E1	10	3.69	0.00E+00	5.53E+08	0.26	0.00	0.98	0.04
2	2A	GSV	E1	13	3.35	8.02E+07	9.00E+07	0.10	0.01	-0.58	0.02
2	2A	GPM2	E1	50	4.12	8.02E+07	9.00E+07	0.19	0.06	-824.81	245.82
3	2A	GSV	E1	18	3.89	7.33E+08	7.35E+08	0.19	0.06	0.47	0.08
4	2B	GSV	E1	7	3.53	2.29E+07	7.88E+08	0.26	0.15	-0.08	0.46
4	2B	SPM2	E1	12	4.47	2.29E+07	7.88E+08	0.20	0.05	20.55	13.99
4	2B	GPS	E1	32	4.04	2.29E+07	7.88E+08	0.39	0.02	1.14	0.43
4	2B	GPM2	E1	3	3.09	2.29E+07	7.88E+08	0.09	0.03	-1017.11	138.37
4	2B	TKW	E1	82	4.28	2.29E+07	7.88E+08	0.31	0.08	0.67	0.76
5	2B	GSV	E1	14	4.24	7.76E+08	7.78E+08	0.25	0.05	0.42	0.03
5	2B	GPM2	E1	1	3.31	7.76E+08	7.78E+08	0.49	NA	-606.90	NA
6	3A	GSV	E1	95	4.08	3.59E+07	3.88E+07	0.48	0.01	0.36	0.07
7	3B	GSV	E1	55	3.40	5.77E+08	8.09E+08	0.17	0.06	-0.47	0.08
7	3B	SPM2	E1	211	5.59	5.77E+08	8.09E+08	0.41	0.03	-19.91	1.15
7	3B	GPM2	E1	88	4.54	5.77E+08	8.09E+08	0.30	0.06	-676.04	48.58
7	3B	GPS	E1	1	3.37	5.77E+08	8.09E+08	0.12	NA	1.79	NA
7	3B	TKW	E1	28	5.48	5.77E+08	8.09E+08	0.33	0.07	-0.97	0.17
8	4A	GSV	E1	237	4.53	6.27E+08	6.88E+08	0.10	0.02	0.63	0.07
9	5A	GSV	E1	8	3.28	6.53E+08	6.58E+08	0.18	0.03	-0.45	0.03
9	5A	TKW	E1	1	3.26	6.53E+08	6.58E+08	0.07	NA	-1.66	NA
10	5D	GSV	E1	161	4.28	4.04E+07	3.48E+08	0.43	0.10	0.10	0.35
10	5D	GPS	E1	26	3.51	4.04E+07	3.48E+08	0.45	0.09	-0.58	1.03
11	6A	GSV	E1	86	4.36	6.14E+08	6.17E+08	0.45	0.02	0.34	0.11
12	6B	GSV	E1	293	4.61	2.90E+07	5.42E+08	0.19	0.05	-0.42	0.14
12	6B	GPM2	E1	106	4.54	2.90E+07	5.42E+08	0.12	0.08	-950.71	625.41
12	6B	TKW	E1	1	3.06	2.90E+07	5.42E+08	0.05	NA	-1.90	NA
13	7A	GSV	E1	19	4.32	1.44E+07	1.70E+07	0.43	0.02	-0.38	0.02
14	7A	GSV	E1	11	3.95	2.02E+07	2.39E+07	0.08	0.01	-0.68	0.06
15	7A	GSV	E1	76	6.64	6.40E+08	6.55E+08	0.16	0.03	-0.65	0.07
15	7A	TKW	E1	8	3.97	6.40E+08	6.55E+08	0.23	0.01	-1.13	0.07
16	7D	GSV	E1	6	4.04	3.98E+08	5.00E+08	0.12	0.04	-0.56	0.09
16	7D	TKW	E1	1	3.45	3.98E+08	5.00E+08	0.18	NA	-1.18	NA
17	1A	GSV	E2	70	4.42	2.43E+07	4.02E+07	0.21	0.05	-0.35	0.04
17	1A	SPM2	E2	1	3.17	2.43E+07	4.02E+07	0.47	NA	11.93	NA
18	1A	GSV	E2	15	4.14	5.83E+08	5.86E+08	0.12	0.02	-0.46	0.04
19	2B	GSV	E2	19	3.93	2.49E+07	2.67E+07	0.19	0.01	-0.37	0.01
20	2B	GSV	E2	3	4.55	7.96E+08	7.97E+08	0.11	0.00	-0.49	0.04
21	2B	GSV	E2	4	4.72	7.94E+08	7.96E+08	0.21	0.05	-0.41	0.05
22	3A	GSV	E2	27	3.41	3.59E+07	3.87E+07	0.49	0.00	0.28	0.00
23	3A	GSV	E2	7	3.70	4.41E+08	5.66E+08	0.13	0.01	-0.42	0.02
23	3A	TKW	E2	64	4.04	4.41E+08	5.66E+08	0.18	0.04	-1.21	0.32
23	3A	GPM2	E2	9	4.07	4.41E+08	5.66E+08	0.08	0.03	-1034.82	268.01
24	3A	GSV	E2	48	3.36	7.09E+08	7.11E+08	0.33	0.04	0.30	0.02
24	3A	SPM2	E2	41	3.42	7.09E+08	7.11E+08	0.22	0.00	-14.03	0.27

25	3A	GSV	E2	8	3.85	6.86E+08	6.89E+08	0.46	0.03	-0.21	0.22
26	3D	GSV	E2	8	3.90	3.96E+08	4.15E+08	0.38	0.03	0.30	0.02
27	3D	GSV	E2	20	3.58	5.93E+08	6.00E+08	0.20	0.01	-0.35	0.01
28	4A	GSV	E2	8	3.56	6.15E+08	6.17E+08	0.30	0.05	-0.32	0.01
29	5B	GSV	E2	9	3.47	5.59E+08	5.64E+08	0.31	0.01	0.31	0.01
30	5B	GSV	E2	7	3.98	5.81E+08	5.84E+08	0.36	0.01	-0.30	0.02
31	6A	GSV	E2	40	4.13	8.99E+06	1.56E+07	0.12	0.01	-0.48	0.02
31	6A	GPM2	E2	7	3.47	8.99E+06	1.56E+07	0.05	0.01	-1173.09	88.37
32	6B	GSV	E2	12	4.60	2.99E+07	3.36E+07	0.13	0.05	-0.47	0.07
32	6B	TKW	E2	2	3.67	2.99E+07	3.36E+07	0.23	0.23	-0.40	2.13
33	7A	GSV	E2	55	3.41	7.55E+05	2.81E+07	0.48	0.01	0.24	0.14
33	7A	TKW	E2	7	3.53	7.55E+05	2.81E+07	0.36	0.10	-1.02	0.13
34	7A	GSV	E2	75	5.87	6.38E+08	6.60E+08	0.15	0.04	-0.45	0.06
34	7A	SPM2	E2	8	3.73	6.38E+08	6.60E+08	0.14	0.02	17.33	0.25
34	7A	TKW	E2	16	4.20	6.38E+08	6.60E+08	0.21	0.02	-1.25	0.07
35	7A	GSV	E2	16	3.44	6.80E+08	6.85E+08	0.44	0.08	-0.25	0.17
36	7B	GSV	E2	19	4.39	0.00E+00	6.94E+08	0.40	0.00	0.34	0.01
36	7B	SPM2	E2	3	4.05	0.00E+00	6.94E+08	0.14	0.10	20.47	5.00
36	7B	TKW	E2	1	3.04	0.00E+00	6.94E+08	0.29	NA	1.05	NA
36	7B	GPM2	E2	6	5.17	0.00E+00	6.94E+08	0.15	0.12	-861.07	330.04
37	1B	GSV	E3	65	3.93	0.00E+00	2.68E+08	0.20	0.04	0.57	0.05
37	1B	GPS	E3	1	3.07	0.00E+00	2.68E+08	0.21	NA	-2.37	NA
37	1B	SPM2	E3	88	4.15	0.00E+00	2.68E+08	0.28	0.04	-23.87	5.11
37	1B	TKW	E3	3	3.75	0.00E+00	2.68E+08	0.19	0.06	1.26	0.23
38	1B	GSV	E3	7	4.65	5.25E+08	5.33E+08	0.37	0.03	-0.46	0.03
39	2A	GSV	E3	34	3.71	4.37E+07	4.92E+07	0.35	0.04	-0.41	0.02
40	4A	GSV	E3	5	3.49	1.92E+08	6.35E+08	0.29	0.00	0.46	0.01
40	4A	SPM2	E3	1	3.22	1.92E+08	6.35E+08	0.10	NA	-32.27	NA
40	4A	TKW	E3	4	3.24	1.92E+08	6.35E+08	0.33	0.00	1.05	0.01
41	4A	GSV	E3	4	4.04	5.95E+08	6.06E+08	0.36	0.14	0.19	0.49
41	4A	GPS	E3	1	3.75	5.95E+08	6.06E+08	0.08	NA	3.99	NA
42	4B	GSV	E3	5	4.04	1.39E+07	1.74E+07	0.25	0.04	0.52	0.03
42	4B	TKW	E3	3	3.42	1.39E+07	1.74E+07	0.24	0.00	-1.05	0.01
43	5A	GSV	E3	7	4.11	6.78E+08	6.81E+08	0.43	0.01	-0.42	0.03
44	5D	GSV	E3	5	3.20	5.41E+08	5.46E+08	0.40	0.00	-0.39	0.01
45	6A	GSV	E3	120	4.33	5.62E+08	5.72E+08	0.10	0.00	0.67	0.03
46	7B	GSV	E3	5	3.31	6.85E+08	6.92E+08	0.23	0.01	-0.45	0.01
46	7B	GPM2	E3	3	3.02	6.85E+08	6.92E+08	0.39	0.00	-900.82	0.00
46	7B	TKW	E3	1	3.04	6.85E+08	6.92E+08	0.40	NA	-0.83	NA
47	7D	GSV	E3	4	3.18	1.01E+08	1.15E+08	0.33	0.03	0.42	0.01
48	1A	GSV	E4	5	3.59	5.16E+08	5.18E+08	0.25	0.01	-0.28	0.02
49	1A	GSV	E4	4	3.50	5.20E+08	5.22E+08	0.36	0.00	0.25	0.00
50	1A	GSV	E4	3	3.26	5.78E+08	5.81E+08	0.09	0.01	0.39	0.02
51	1B	GSV	E4	2159	7.08	0.00E+00	5.65E+08	0.20	0.03	0.42	0.07
51	1B	GPS	E4	10	4.36	0.00E+00	5.65E+08	0.20	0.17	1.53	2.16
52	2A	GSV	E4	5	3.57	4.89E+08	6.53E+08	0.19	0.09	-0.30	0.04
52	2A	GPS	E4	1	3.22	4.89E+08	6.53E+08	0.38	NA	-1.57	NA
53	2D	GSV	E4	9	3.19	6.20E+07	8.44E+07	0.51	0.00	-0.22	0.00
54	2D	GSV	E4	6	3.23	6.83E+07	9.09E+07	0.28	0.00	-0.25	0.00
54	2D	TKW	E4	7	4.39	6.83E+07	9.09E+07	0.27	0.00	-1.10	0.02

55	2D	GSV	E4	15	4.24	1.91E+08	3.93E+08	0.20	0.10	0.25	0.19
56	5B	GSV	E4	6	3.31	3.96E+07	1.20E+08	0.10	0.01	-0.36	0.01
57	5B	GSV	E4	10	3.55	2.63E+08	2.95E+08	0.17	0.01	0.31	0.01
58	6A	GSV	E4	3	3.18	5.65E+08	5.71E+08	0.09	0.00	0.37	0.01
59	6B	GSV	E4	9	3.61	4.81E+08	6.52E+08	0.34	0.06	-0.25	0.02
59	6B	SPM2	E4	38	3.79	4.81E+08	6.52E+08	0.25	0.01	20.03	0.99
60	6D	GSV	E4	52	4.08	1.27E+08	2.93E+08	0.27	0.01	0.27	0.01
61	2D	GSV	E4	1	4.82	2.91E+07	5.11E+07	0.19	NA	0.39	NA
62	6B	SPM2	E2	1	3.10	8.06E+07	1.18E+08	0.08	NA	21.27	NA
62	6B	TKW	E2	22	3.74	8.06E+07	1.18E+08	0.15	0.01	-1.39	0.04
63	6B	GSV	E3	3	3.15	6.11E+08	6.12E+08	0.28	0.00	0.42	0.01
63	6A	SPM2	E3	1	3.08	6.11E+08	6.12E+08	0.11	NA	-30.20	NA
64	7A	GSV	E4	4	3.26	7.12E+08	7.14E+08	0.42	0.05	-0.11	0.22
64	7A	TKW	E4	1	3.29	7.12E+08	7.14E+08	0.43	NA	-0.84	NA
