# Supplementary material for: Wheat individual grain-size variance originates from crop development and from specific genetic determinism
Source: PLoS One. 2020 Mar 26;15(3):e0230689. doi: 10.1371/journal.pone.0230689 (PMC7098578; doi:10.1371/journal.pone.0230689)
Supplement: S5 Table — For each environment, the broad sense heritabilities (see Materials and methods) of all yield components were calculated and compared between environments. GSV: Grain size variance, SPM2: number of spikes per m2, GPS: number of grains per spike, GPM2: number of grains/m2, TKW: thousand kernel weight (g at 15% moisture content), GY: grain yield (t/ha at 15% moisture content). E1 (well-watered, 2016), E2 (water-deficit, 2016), E3 (well-watered, 2017) and E4 (water-deficit, 2017). (PDF) [file pone.0230689.s005.pdf]

	SPM2	GPS	GPM2	TKW	GSV	GY
Environment 1	0.60	0.55	0.82	0.91	0.85	0.77
Environment 2	0.61	0.51	0.76	0.92	0.82	0.71
Environment 3	0.48	0.52	0.85	0.96	0.69	0.80
Environment 4	0.58	0.80	0.75	0.91	0.52	0.74
