# Supplementary material for: Wheat individual grain-size variance originates from crop development and from specific genetic determinism
Source: PLoS One. 2020 Mar 26;15(3):e0230689. doi: 10.1371/journal.pone.0230689 (PMC7098578; doi:10.1371/journal.pone.0230689)
Supplement: S6 Table — Characteristics (Environment (Env), Trait, Chromosome (Chr), Name of the SNP (SNP), Physical position, MAF, Effect, LOD score and Percentage of the total variance of each trait explained by the significant SNP) of each significant SNP are identified and reported. (1) Physical position on the chromosome (Chr) [36], (2)Minor allele frequency (MAF), (3)Expressed in terms of the absolute value for each trait, (4) LOD score on the significant associated SNP, (5) Percentage of the total variance of each trait explained (r2). E1 (well-watered, 2016), E2 (water-deficit, 2016), E3 (well-watered, 2017) and E4 (water-deficit, 2017). (PDF) [file pone.0230689.s006.pdf]

Env	Trait	Chr	SNP	Position[(1)]	MAF[(2)]	Effect[(3)]	LOD[(4)]	$R^{2(5)}$
E1	SPM2	1A	AX.89382044	2.31E+07	0.50	-16.77	3.20	0.05
E1	GY	1A	AX.89365795	3.78E+06	0.08	-4.72	3.77	0.07
E1	GY	1A	AX.89438586	5.55E+08	0.34	-2.55	3.46	0.06
E1	SPM2	1A	AX.89592483	4.84E+08	0.34	-17.31	3.11	0.05
E1	GY	1A	AX.89654016	5.53E+08	0.45	-2.50	3.62	0.06
E1	SPM2	1A	AX.89592294	4.95E+08	0.20	-22.22	3.55	0.06
E1	GY	1A	AX.89524501	5.04E+08	0.26	-2.61	3.15	0.05
E1	SPM2	1A	AX.89449109	2.31E+07	0.50	-16.77	3.20	0.05
E1	GY	1A	AX.89488071	5.92E+08	0.43	2.32	3.14	0.05
E1	GY	1A	AX.89451237	5.92E+08	0.43	2.32	3.14	0.05
E1	GY	1A	AX.89488071	5.92E+08	0.43	2.32	3.14	0.05
E1	SPM2	1A	AX.89695936	3.94E+08	0.22	24.81	4.54	0.08
E1	GY	1A	AX.89484374	3.61E+06	0.07	-4.83	3.73	0.07
E1	GY	1A	AX.89644738	2.48E+06	0.07	-4.61	3.45	0.05
E1	GY	1A	AX.89699261	3.78E+06	0.06	-5.19	3.36	0.06
E1	SPM2	1A	AX.89312211	4.92E+08	0.19	-23.34	3.69	0.07
E1	GY	1A	AX.89540921	5.57E+08	0.06	-4.73	3.06	0.05
E1	GY	1A	AX.89540921	5.57E+08	0.06	-4.73	3.06	0.05
E1	GY	1A	AX.89484374	3.61E+06	0.07	-4.83	3.73	0.07
E1	SPM2	1A	AX.89311190	4.87E+08	0.34	-17.31	3.11	0.05
E1	GY	1A	AX.89334798	4.50E+08	0.08	-5.28	4.80	0.08
E1	GY	1A	AX.89699261	3.78E+06	0.06	-5.19	3.36	0.06
E1	GY	1A	AX.89485591	5.04E+08	0.20	-3.47	4.44	0.08
E1	GY	1A	AX.89524501	5.04E+08	0.26	-2.61	3.15	0.05
E1	GY	1A	AX.89322672	5.72E+08	0.07	-4.55	3.04	0.05
E1	GY	1A	AX.89619043	5.92E+08	0.43	2.30	3.11	0.05
E1	GY	1A	AX.89491333	3.61E+06	0.08	-4.40	3.50	0.06
E1	GY	1A	AX.89491333	3.61E+06	0.08	-4.40	3.50	0.06
E1	SPM2	1A	AX.89356729	5.17E+08	0.31	17.56	3.05	0.05
E1	GY	1A	AX.89654016	5.53E+08	0.45	-2.50	3.62	0.06
E1	SPM2	1A	AX.89362965	5.03E+06	0.08	32.89	3.68	0.05
E1	GY	1A	AX.89495233	5.75E+08	0.06	-4.73	3.05	0.05
E1	GY	1A	AX.89644738	2.48E+06	0.07	-4.61	3.45	0.05
E1	GY	1A	AX.89478953	2.48E+06	0.08	-4.31	3.22	0.05
E1	GY	1A	AX.89605611	2.38E+06	0.08	-4.31	3.22	0.05
E1	GY	1A	AX.89577136	4.74E+08	0.07	-4.40	3.19	0.06
E1	GY	1A	AX.89485591	5.04E+08	0.20	-3.47	4.44	0.08
E1	GY	1A	AX.89590002	3.76E+06	0.08	-4.58	3.74	0.07
E1	GY	1A	AX.89438586	5.55E+08	0.34	-2.55	3.46	0.06
E1	GY	1A	AX.89391296	3.61E+06	0.08	-4.69	3.73	0.07
E1	GY	1A	AX.89391296	3.61E+06	0.08	-4.69	3.73	0.07
E1	SPM2	1A	AX.89490533	2.31E+07	0.50	-16.77	3.20	0.05
E1	GY	1A	AX.89593842	5.72E+08	0.07	-4.55	3.04	0.05
E1	SPM2	1A	AX.89721814	4.87E+08	0.33	-19.05	3.62	0.06
E1	GY	1A	AX.89453261	5.57E+08	0.06	-4.73	3.06	0.05
E1	GY	1A	AX.89453261	5.57E+08	0.06	-4.73	3.06	0.05
E1	GY	1A	AX.89740285	4.61E+08	0.07	-5.31	4.42	0.08
E1	GY	1A	AX.89619043	5.92E+08	0.43	2.30	3.11	0.05
E1	SPM2	1A	AX.89504713	5.17E+08	0.32	17.51	3.05	0.05

E1	GY	1A	AX.89478953	2.48E+06	0.08	-4.31	3.22	0.05
E1	GY	1A	AX.89608660	4.50E+08	0.08	-5.28	4.80	0.09
E1	SPM2	1A	AX.89764618	3.93E+08	0.18	25.14	4.12	0.08
E1	GY	1A	AX.89338310	2.93E+06	0.07	-4.45	3.25	0.05
E1	GY	1A	AX.89658648	5.42E+08	0.37	-2.89	4.44	0.07
E1	GY	1A	AX.89528878	4.61E+08	0.10	-4.84	4.65	0.09
E1	SPM2	1A	AX.89487050	4.87E+08	0.34	-17.31	3.11	0.05
E1	GY	1A	AX.89634735	3.76E+06	0.08	-4.72	3.77	0.07
E1	GY	1A	AX.89451237	5.92E+08	0.43	2.32	3.14	0.05
E1	GY	1A	AX.89495233	5.75E+08	0.06	-4.73	3.05	0.05
E1	GY	1A	AX.89605611	2.38E+06	0.08	-4.31	3.22	0.05
E1	GY	1A	AX.89593842	5.72E+08	0.07	-4.55	3.04	0.05
E1	GY	1A	AX.89474547	5.92E+08	0.43	2.32	3.14	0.05
E1	GY	1A	AX.89619783	4.58E+08	0.10	-4.84	4.65	0.09
E1	GY	1A	AX.89619783	4.58E+08	0.10	-4.84	4.65	0.09
E1	SPM2	1A	AX.89589493	4.95E+08	0.18	-24.44	3.93	0.07
E1	GY	1A	AX.89398992	3.61E+06	0.07	-4.83	3.73	0.07
E1	GY	1A	AX.89398992	3.61E+06	0.07	-4.83	3.73	0.07
E1	GY	1A	AX.89318842	3.61E+06	0.07	-4.83	3.73	0.07
E1	SPM2	1A	AX.89720387	4.87E+08	0.34	-17.31	3.11	0.05
E1	SPM2	1A	AX.89403547	4.85E+08	0.34	-17.31	3.11	0.05
E1	GY	1A	AX.89757813	4.75E+08	0.09	-4.58	4.07	0.08
E1	GY	1A	AX.89757813	4.75E+08	0.09	-4.58	4.07	0.08
E1	SPM2	1A	AX.89694178	3.95E+08	0.16	23.00	3.28	0.06
E1	GY	1A	AX.89520705	5.53E+08	0.45	-2.71	4.15	0.07
E1	GY	1A	AX.89322672	5.72E+08	0.07	-4.55	3.04	0.05
E1	GY	1A	AX.89634735	3.76E+06	0.08	-4.72	3.77	0.07
E1	SPM2	1A	AX.89548200	4.95E+08	0.18	-24.44	3.93	0.07
E1	GY	1A	AX.89334798	4.50E+08	0.08	-5.28	4.80	0.08
E1	GPS	1A	AX.89760863	5.91E+08	0.50	1.21	3.57	0.06
E1	GPS	1A	AX.89346156	5.91E+08	0.50	1.21	3.57	0.06
E1	GPS	1A	AX.89750662	4.61E+08	0.10	-1.89	3.14	0.05
E1	GPS	1A	AX.89451237	5.92E+08	0.43	1.22	3.55	0.06
E1	GY	1A	AX.89365795	3.78E+06	0.08	-4.72	3.77	0.07
E1	GPS	1A	AX.89384857	5.94E+08	0.32	1.22	3.18	0.06
E1	GPS	1A	AX.89474547	5.92E+08	0.43	1.22	3.55	0.06
E1	GY	1A	AX.89747039	4.75E+08	0.10	-4.72	4.62	0.09
E1	GPS	1A	AX.89386468	5.91E+08	0.50	1.21	3.57	0.06
E1	GY	1A	AX.89577136	4.74E+08	0.07	-4.40	3.19	0.06
E1	GY	1A	AX.89590002	3.76E+06	0.08	-4.58	3.74	0.07
E1	GPS	1A	AX.89533756	5.91E+08	0.44	1.11	3.02	0.05
E1	GPS	1A	AX.89391152	5.91E+08	0.49	1.20	3.52	0.06
E1	GY	1A	AX.89495866	4.50E+08	0.08	-5.28	4.80	0.09
E1	GY	1A	AX.89454402	4.50E+08	0.08	-5.28	4.80	0.09
E1	GY	1A	AX.89454402	4.50E+08	0.08	-5.28	4.80	0.09
E1	GY	1A	AX.89608660	4.50E+08	0.08	-5.28	4.80	0.09
E1	GY	1A	AX.89338310	2.93E+06	0.07	-4.45	3.25	0.05
E1	GY	1A	AX.89528878	4.61E+08	0.10	-4.84	4.65	0.09
E1	GPS	1A	AX.89614198	5.91E+08	0.50	1.21	3.57	0.06
E1	GY	1A	AX.89318842	3.61E+06	0.07	-4.83	3.73	0.07

E1	GY	1A	AX.89750662	4.61E+08	0.10	-4.84	4.65	0.09
E1	GY	1A	AX.89740285	4.61E+08	0.07	-5.31	4.42	0.08
E1	GY	1A	AX.89608289	2.98E+06	0.07	-4.45	3.25	0.05
E1	GY	1A	AX.89516165	4.50E+08	0.10	-4.59	4.24	0.08
E1	GY	1A	AX.89747039	4.75E+08	0.10	-4.72	4.62	0.09
E1	SPM2	1A	AX.89418654	2.31E+07	0.49	16.53	3.12	0.05
E1	SPM2	1A	AX.89421925	4.87E+08	0.34	-17.31	3.11	0.05
E1	GPM2	1A	AX.89451199	4.88E+08	0.26	-669.96	3.05	0.05
E1	GPS	1A	AX.89619043	5.92E+08	0.43	1.20	3.43	0.06
E1	GPM2	1A	AX.89407832	4.88E+08	0.25	-674.26	3.02	0.05
E1	GPS	1A	AX.89631871	5.91E+08	0.44	1.11	3.02	0.05
E1	GY	1A	AX.89474547	5.92E+08	0.43	2.32	3.14	0.05
E1	GY	1A	AX.89652711	3.61E+06	0.07	-4.83	3.73	0.07
E1	GY	1A	AX.89652711	3.61E+06	0.07	-4.83	3.73	0.07
E1	SPM2	1A	AX.89581416	5.16E+08	0.31	17.48	3.01	0.05
E1	GY	1A	AX.89658648	5.42E+08	0.37	-2.89	4.44	0.07
E1	GPM2	1A	AX.89614198	5.91E+08	0.50	595.98	3.12	0.05
E1	GPS	1A	AX.89378347	5.78E+08	0.25	1.40	3.59	0.06
E1	GY	1A	AX.89750662	4.61E+08	0.10	-4.84	4.65	0.09
E1	GPM2	1A	AX.89365920	4.88E+08	0.25	-674.26	3.02	0.05
E1	GPS	1A	AX.86164505	5.92E+08	0.43	1.15	3.21	0.05
E1	GPM2	1A	AX.89530045	4.84E+08	0.31	-649.44	3.17	0.05
E1	GPS	1A	AX.89705511	5.91E+08	0.49	1.24	3.69	0.06
E1	GPM2	1A	AX.89509754	4.88E+08	0.25	-674.26	3.02	0.05
E1	GPS	1A	AX.89674096	5.94E+08	0.32	1.22	3.18	0.06
E1	GY	1A	AX.89520705	5.53E+08	0.45	-2.71	4.15	0.07
E1	GPS	1A	AX.89382888	5.94E+08	0.32	1.22	3.18	0.06
E1	GPS	1A	AX.89528878	4.61E+08	0.10	-1.89	3.14	0.05
E1	GPS	1A	AX.89488071	5.92E+08	0.43	1.22	3.55	0.06
E1	GPS	1A	AX.89482831	5.92E+08	0.49	-1.18	3.42	0.06
E1	GPS	1A	AX.89383456	5.93E+08	0.32	1.22	3.18	0.06
E1	GY	1A	AX.89760283	4.76E+08	0.20	-2.86	3.10	0.05
E1	GY	1A	AX.89760283	4.76E+08	0.20	-2.86	3.10	0.05
E1	GPS	1A	AX.89619783	4.58E+08	0.10	-1.89	3.14	0.05
E1	GY	1A	AX.89516165	4.50E+08	0.10	-4.59	4.24	0.08
E1	GPM2	1A	AX.89632460	4.88E+08	0.26	-669.96	3.05	0.05
E1	GY	1A	AX.89761921	4.77E+08	0.20	-3.14	3.67	0.06
E1	GY	1A	AX.89761921	4.77E+08	0.20	-3.14	3.67	0.06
E1	GPM2	1A	AX.89581657	4.88E+08	0.27	-694.82	3.27	0.05
E1	GPM2	1A	AX.89681974	4.84E+08	0.31	-649.44	3.17	0.05
E1	GPM2	1A	AX.89751748	4.86E+08	0.27	-716.25	3.51	0.06
E1	GPM2	1A	AX.89678176	4.88E+08	0.25	-674.26	3.02	0.05
E1	GPM2	1A	AX.89526150	4.82E+08	0.31	-668.47	3.30	0.06
E1	GPM2	1A	AX.89705511	5.91E+08	0.49	597.01	3.13	0.05
E1	GPM2	1A	AX.89472485	4.88E+08	0.25	-674.26	3.02	0.05
E1	GPM2	1A	AX.89618201	4.82E+08	0.31	-668.47	3.30	0.06
E1	GPM2	1A	AX.89485591	5.04E+08	0.20	-811.46	3.66	0.07
E1	GPS	1A	AX.89697409	5.93E+08	0.32	1.19	3.07	0.05
E1	GPS	1A	AX.89380462	5.94E+08	0.32	1.22	3.18	0.06
E1	GPM2	1A	AX.89644137	4.88E+08	0.26	-717.80	3.43	0.05

E1	GPM2	1A	AX.89716972	4.88E+08	0.25	-674.26	3.02	0.05
E1	GPM2	1A	AX.89613533	4.88E+08	0.27	-694.82	3.27	0.05
E1	GPM2	1A	AX.89760863	5.91E+08	0.50	595.98	3.12	0.05
E1	GPS	1A	AX.89749145	5.91E+08	0.49	1.18	3.42	0.06
E1	GPS	1A	AX.89709616	5.93E+08	0.32	1.22	3.18	0.06
E1	GY	1A	AX.89495866	4.50E+08	0.08	-5.28	4.80	0.09
E1	TKW	1A	AX.89584068	2.16E+07	0.15	-1.18	3.06	0.05
E1	GPM2	1A	AX.89631167	4.88E+08	0.25	-674.26	3.02	0.05
E1	GPS	1A	AX.89578979	5.93E+08	0.32	1.22	3.18	0.06
E1	TKW	1A	AX.89451199	4.88E+08	0.26	0.97	3.16	0.05
E1	TKW	1A	AX.89759652	1.64E+07	0.19	-1.09	3.14	0.05
E1	GPM2	1A	AX.89495233	5.75E+08	0.06	-1393.11	3.75	0.06
E1	GPM2	1A	AX.89332564	4.88E+08	0.25	-674.26	3.02	0.05
E1	GPM2	1A	AX.89386468	5.91E+08	0.50	595.98	3.12	0.05
E1	TKW	1A	AX.89366930	4.88E+08	0.26	1.06	3.69	0.06
E1	TKW	1A	AX.89632460	4.88E+08	0.26	0.97	3.16	0.05
E1	GY	1A	AX.89608289	2.98E+06	0.07	-4.45	3.25	0.05
E1	TKW	1A	AX.89644137	4.88E+08	0.26	1.00	3.32	0.06
E1	TKW	1A	AX.89508718	1.16E+07	0.10	-1.55	3.59	0.06
E1	TKW	1A	AX.89628186	2.41E+07	0.31	0.90	3.04	0.05
E1	GPM2	1A	AX.89346156	5.91E+08	0.50	595.98	3.12	0.05
E1	GPM2	1A	AX.89366930	4.88E+08	0.26	-764.38	3.82	0.06
E1	GPM2	1A	AX.89532336	4.84E+08	0.31	-649.44	3.17	0.05
E1	TKW	1A	AX.89772366	4.82E+08	0.26	1.04	3.53	0.06
E1	TKW	1A	AX.89590906	1.41E+07	0.50	-0.83	3.02	0.05
E1	TKW	1A	AX.89535322	5.93E+08	0.33	-0.92	3.23	0.06
E1	TKW	1A	AX.89751748	4.86E+08	0.27	0.95	3.15	0.05
E1	TKW	1A	AX.89723195	5.93E+08	0.31	-0.90	3.06	0.05
E1	GPM2	1A	AX.89473144	4.88E+08	0.25	-674.26	3.02	0.05
E1	GPM2	1A	AX.89748675	4.84E+08	0.31	-649.44	3.17	0.05
E1	TKW	1A	AX.89588392	1.16E+07	0.06	-1.77	3.11	0.05
E1	TKW	1A	AX.89677919	2.41E+07	0.31	0.90	3.04	0.05
E1	TKW	1A	AX.89613533	4.88E+08	0.27	0.97	3.16	0.05
E1	TKW	1A	AX.89581657	4.88E+08	0.27	0.97	3.16	0.05
E1	TKW	1A	AX.89483593	5.73E+08	0.05	-1.94	3.17	0.05
E1	TKW	1A	AX.89343200	4.88E+08	0.27	0.94	3.03	0.05
E1	TKW	1A	AX.89346508	2.17E+07	0.16	-1.13	3.06	0.05
E1	TKW	1A	AX.89389331	4.88E+08	0.27	0.94	3.01	0.05
E2	GY	1A	AX.89492546	5.03E+08	0.39	-2.02	3.29	0.04
E2	TKW	1A	AX.89429909	5.73E+08	0.07	1.85	3.30	0.06
E2	SPM2	1A	AX.89386026	2.53E+08	0.07	25.80	3.54	0.06
E2	GY	1A	AX.89330607	5.40E+08	0.22	-2.64	3.92	0.07
E2	GY	1A	AX.89401575	5.03E+08	0.39	-1.97	3.15	0.04
E2	GY	1A	AX.89757813	4.75E+08	0.09	-3.56	3.56	0.06
E2	GY	1A	AX.89760710	5.05E+08	0.32	-2.14	3.30	0.05
E2	GY	1A	AX.89761921	4.77E+08	0.20	-2.39	3.12	0.05
E2	GY	1A	AX.89761921	4.77E+08	0.20	-2.39	3.12	0.05
E2	GY	1A	AX.89380336	5.72E+08	0.07	-3.86	3.12	0.04
E2	GY	1A	AX.89356842	5.04E+08	0.33	-2.09	3.22	0.05
E2	SPM2	1A	AX.89547384	2.49E+08	0.42	12.22	3.24	0.06

E2	GY	1A	AX.89369571	5.05E+08	0.33	-2.11	3.24	0.05
E2	GY	1A	AX.89334474	5.05E+08	0.33	-2.11	3.24	0.05
E2	GY	1A	AX.89334474	5.05E+08	0.33	-2.11	3.24	0.05
E2	GY	1A	AX.89334798	4.50E+08	0.08	-3.39	3.02	0.03
E2	GY	1A	AX.89334798	4.50E+08	0.08	-3.39	3.02	0.03
E2	SPM2	1A	AX.89418526	3.77E+08	0.07	26.28	3.87	0.07
E2	GY	1A	AX.89763732	4.76E+08	0.25	-2.30	3.29	0.05
E2	GY	1A	AX.89763732	4.76E+08	0.25	-2.30	3.29	0.05
E2	GY	1A	AX.89617974	5.04E+08	0.23	-2.26	3.09	0.05
E2	GY	1A	AX.89692058	5.05E+08	0.15	-2.82	3.44	0.06
E2	GY	1A	AX.89692058	5.05E+08	0.15	-2.82	3.44	0.06
E2	GY	1A	AX.89336298	4.74E+08	0.40	-1.92	3.03	0.04
E2	SPM2	1A	AX.89736079	1.71E+08	0.21	14.67	3.17	0.05
E2	GY	1A	AX.89383288	5.68E+08	0.07	-3.74	3.27	0.05
E2	SPM2	1A	AX.89515342	3.06E+07	0.47	11.93	3.17	0.06
E2	GY	1A	AX.89338125	5.05E+08	0.33	-2.11	3.24	0.05
E2	GY	1A	AX.89681579	5.05E+08	0.15	-2.82	3.44	0.06
E2	GY	1A	AX.89445577	5.05E+08	0.15	-2.82	3.44	0.06
E2	GY	1A	AX.89445577	5.05E+08	0.15	-2.82	3.44	0.06
E2	GY	1A	AX.89351069	5.05E+08	0.33	-2.11	3.24	0.05
E2	GY	1A	AX.89722818	5.05E+08	0.33	-2.28	3.70	0.06
E2	SPM2	1A	AX.89651896	3.02E+08	0.07	23.71	3.07	0.05
E2	GY	1A	AX.89457045	5.05E+08	0.33	-2.11	3.24	0.05
E2	GY	1A	AX.89376432	5.05E+08	0.33	-2.11	3.24	0.05
E2	GY	1A	AX.89376432	5.05E+08	0.33	-2.11	3.24	0.05
E2	GY	1A	AX.89483373	5.40E+08	0.21	-2.35	3.14	0.05
E2	GY	1A	AX.89483373	5.40E+08	0.21	-2.35	3.14	0.05
E2	GY	1A	AX.89729774	5.03E+08	0.39	-2.01	3.29	0.05
E2	GY	1A	AX.89729774	5.03E+08	0.39	-2.01	3.29	0.05
E2	GY	1A	AX.89506699	5.05E+08	0.15	-2.82	3.44	0.06
E2	GY	1A	AX.89506699	5.05E+08	0.15	-2.82	3.44	0.06
E2	GY	1A	AX.89519840	5.05E+08	0.33	-2.11	3.24	0.05
E2	GY	1A	AX.89519840	5.05E+08	0.33	-2.11	3.24	0.05
E2	GY	1A	AX.89496603	5.40E+08	0.21	-2.35	3.14	0.05
E2	GY	1A	AX.89332707	5.01E+08	0.39	-2.01	3.29	0.05
E2	GY	1A	AX.89332707	5.01E+08	0.39	-2.01	3.29	0.05
E2	GY	1A	AX.89700744	5.03E+08	0.39	-2.01	3.29	0.05
E2	GY	1A	AX.89700744	5.03E+08	0.39	-2.01	3.29	0.05
E2	GY	1A	AX.89380336	5.72E+08	0.07	-3.86	3.12	0.04
E2	GY	1A	AX.89416172	5.05E+08	0.32	-2.04	3.04	0.04
E2	GY	1A	AX.89416172	5.05E+08	0.32	-2.04	3.04	0.04
E2	GY	1A	AX.89416233	5.03E+08	0.39	-2.02	3.29	0.04
E2	GY	1A	AX.89416233	5.03E+08	0.39	-2.02	3.29	0.04
E2	GY	1A	AX.89380746	5.68E+08	0.07	-3.74	3.27	0.05
E2	GY	1A	AX.89380746	5.68E+08	0.07	-3.74	3.27	0.05
E2	GY	1A	AX.89345545	5.05E+08	0.33	-2.11	3.24	0.05
E2	GY	1A	AX.89345545	5.05E+08	0.33	-2.11	3.24	0.05
E2	GY	1A	AX.89579559	5.41E+08	0.20	-2.45	3.22	0.05
E2	GY	1A	AX.89579559	5.41E+08	0.20	-2.45	3.22	0.05
E2	GY	1A	AX.89312999	5.68E+08	0.07	-3.74	3.27	0.05

E2	GY	1A	AX.89312999	5.68E+08	0.07	-3.74	3.27	0.05
E2	GY	1A	AX.89524262	5.04E+08	0.15	-2.82	3.44	0.06
E2	GY	1A	AX.89524262	5.04E+08	0.15	-2.82	3.44	0.06
E2	GY	1A	AX.89739968	5.05E+08	0.33	-2.11	3.24	0.05
E2	GY	1A	AX.89739968	5.05E+08	0.33	-2.11	3.24	0.05
E2	GY	1A	AX.89777735	5.05E+08	0.32	-2.32	3.82	0.06
E2	GY	1A	AX.89777735	5.05E+08	0.32	-2.32	3.82	0.06
E2	GY	1A	AX.89740285	4.61E+08	0.07	-3.94	3.57	0.06
E2	GY	1A	AX.89740285	4.61E+08	0.07	-3.94	3.57	0.06
E2	GY	1A	AX.89594667	5.05E+08	0.33	-2.11	3.24	0.05
E2	GY	1A	AX.89594667	5.05E+08	0.33	-2.11	3.24	0.05
E2	GY	1A	AX.89382897	5.40E+08	0.21	-2.35	3.09	0.05
E2	GY	1A	AX.89382897	5.40E+08	0.21	-2.35	3.09	0.05
E2	GY	1A	AX.89383288	5.68E+08	0.07	-3.74	3.27	0.05
E2	GY	1A	AX.89431881	5.40E+08	0.22	-2.69	4.08	0.07
E2	GY	1A	AX.89431881	5.40E+08	0.22	-2.69	4.08	0.07
E2	GY	1A	AX.89741832	5.05E+08	0.32	-2.32	3.82	0.06
E2	GY	1A	AX.89741832	5.05E+08	0.32	-2.32	3.82	0.06
E2	GY	1A	AX.89384318	5.06E+08	0.30	-2.16	3.24	0.05
E2	GY	1A	AX.89384318	5.06E+08	0.30	-2.16	3.24	0.05
E2	GY	1A	AX.89653980	5.05E+08	0.30	-2.16	3.24	0.05
E2	GY	1A	AX.89653980	5.05E+08	0.30	-2.16	3.24	0.05
E2	GY	1A	AX.89397127	5.05E+08	0.33	-2.11	3.24	0.05
E2	GY	1A	AX.89527179	5.05E+08	0.33	-2.11	3.24	0.05
E2	GY	1A	AX.89527179	5.05E+08	0.33	-2.11	3.24	0.05
E2	GY	1A	AX.89743474	5.05E+08	0.33	-2.14	3.34	0.05
E2	GY	1A	AX.89743474	5.05E+08	0.33	-2.14	3.34	0.05
E2	GY	1A	AX.89317237	5.40E+08	0.20	-2.37	3.08	0.05
E2	GY	1A	AX.89317237	5.40E+08	0.20	-2.37	3.08	0.05
E2	GY	1A	AX.89499443	5.40E+08	0.22	-2.69	4.08	0.07
E2	GY	1A	AX.89499443	5.40E+08	0.22	-2.69	4.08	0.07
E2	GY	1A	AX.89706884	5.05E+08	0.33	-2.11	3.24	0.05
E2	GY	1A	AX.89706884	5.05E+08	0.33	-2.11	3.24	0.05
E2	GY	1A	AX.89351069	5.05E+08	0.33	-2.11	3.24	0.05
E2	GY	1A	AX.89628879	5.05E+08	0.33	-2.11	3.24	0.05
E2	GY	1A	AX.89628879	5.05E+08	0.33	-2.11	3.24	0.05
E2	GY	1A	AX.89598827	5.05E+08	0.32	-2.14	3.30	0.05
E2	GY	1A	AX.89598827	5.05E+08	0.32	-2.14	3.30	0.05
E2	GY	1A	AX.89457045	5.05E+08	0.33	-2.11	3.24	0.05
E2	GY	1A	AX.89744714	5.05E+08	0.33	-2.11	3.24	0.05
E2	GY	1A	AX.89744714	5.05E+08	0.33	-2.11	3.24	0.05
E2	GY	1A	AX.89387877	5.05E+08	0.32	-2.32	3.82	0.06
E2	GY	1A	AX.89387877	5.05E+08	0.32	-2.32	3.82	0.06
E2	GY	1A	AX.89673050	5.40E+08	0.22	-2.69	4.08	0.07
E2	GY	1A	AX.89673050	5.40E+08	0.22	-2.69	4.08	0.07
E2	GY	1A	AX.89636183	5.05E+08	0.30	-2.16	3.24	0.05
E2	GY	1A	AX.89636183	5.05E+08	0.30	-2.16	3.24	0.05
E2	GY	1A	AX.89757813	4.75E+08	0.09	-3.56	3.56	0.06
E2	GY	1A	AX.89747039	4.75E+08	0.10	-3.22	3.22	0.06
E2	GY	1A	AX.89747039	4.75E+08	0.10	-3.22	3.22	0.06

E2	GY	1A	AX.89495866	4.50E+08	0.08	-3.39	3.02	0.05
E2	GY	1A	AX.89495866	4.50E+08	0.08	-3.39	3.02	0.05
E2	GY	1A	AX.89321558	5.05E+08	0.33	-2.11	3.24	0.05
E2	GY	1A	AX.89321558	5.05E+08	0.33	-2.11	3.24	0.05
E2	TKW	1A	AX.89370608	2.09E+07	0.26	-1.12	3.52	0.07
E2	GY	1A	AX.89321838	5.05E+08	0.33	-2.11	3.24	0.05
E2	GY	1A	AX.89321838	5.05E+08	0.33	-2.11	3.24	0.05
E2	GY	1A	AX.89460271	5.40E+08	0.21	-2.35	3.09	0.05
E2	GY	1A	AX.89460271	5.40E+08	0.21	-2.35	3.09	0.05
E2	GY	1A	AX.89496603	5.40E+08	0.21	-2.35	3.14	0.05
E2	GY	1A	AX.89426368	5.46E+08	0.05	-4.39	3.14	0.04
E2	GY	1A	AX.89484669	5.03E+08	0.38	-2.11	3.52	0.05
E2	GY	1A	AX.89484669	5.03E+08	0.38	-2.11	3.52	0.05
E2	GY	1A	AX.89322580	5.05E+08	0.33	-2.11	3.24	0.05
E2	GY	1A	AX.89322580	5.05E+08	0.33	-2.11	3.24	0.05
E2	GY	1A	AX.89676171	5.05E+08	0.33	-2.11	3.24	0.05
E2	GY	1A	AX.89676171	5.05E+08	0.33	-2.11	3.24	0.05
E2	GY	1A	AX.89323937	5.03E+08	0.39	-2.02	3.29	0.04
E2	GY	1A	AX.89323937	5.03E+08	0.39	-2.02	3.29	0.04
E2	GY	1A	AX.89392808	5.68E+08	0.07	-4.67	4.31	0.07
E2	GY	1A	AX.89392808	5.68E+08	0.07	-4.67	4.31	0.07
E2	GY	1A	AX.89750332	5.05E+08	0.33	-2.11	3.24	0.05
E2	GY	1A	AX.89750332	5.05E+08	0.33	-2.11	3.24	0.05
E2	GY	1A	AX.89358873	5.04E+08	0.26	-2.23	3.15	0.05
E2	GY	1A	AX.89358873	5.04E+08	0.26	-2.23	3.15	0.05
E2	GY	1A	AX.89654297	5.03E+08	0.39	-2.01	3.29	0.05
E2	GY	1A	AX.89654297	5.03E+08	0.39	-2.01	3.29	0.05
E2	GY	1A	AX.89629427	5.37E+08	0.05	-4.59	3.42	0.04
E2	GY	1A	AX.89629427	5.37E+08	0.05	-4.59	3.42	0.04
E2	GY	1A	AX.89326278	5.01E+08	0.39	-2.01	3.29	0.05
E2	GY	1A	AX.89326278	5.01E+08	0.39	-2.01	3.29	0.05
E2	GY	1A	AX.89336298	4.74E+08	0.40	-1.92	3.03	0.04
E2	GY	1A	AX.89431899	5.05E+08	0.33	-2.11	3.24	0.05
E2	GY	1A	AX.89431899	5.05E+08	0.33	-2.11	3.24	0.05
E2	GY	1A	AX.89465558	5.40E+08	0.21	-2.35	3.14	0.05
E2	GY	1A	AX.89397127	5.05E+08	0.33	-2.11	3.24	0.05
E2	GY	1A	AX.89537351	5.03E+08	0.39	-2.10	3.52	0.05
E2	GY	1A	AX.89537351	5.03E+08	0.39	-2.10	3.52	0.05
E2	GY	1A	AX.89327850	5.05E+08	0.20	-2.52	3.39	0.05
E2	GY	1A	AX.89327850	5.05E+08	0.20	-2.52	3.39	0.05
E2	GY	1A	AX.89398098	5.01E+08	0.39	-2.01	3.29	0.05
E2	GY	1A	AX.89398098	5.01E+08	0.39	-2.01	3.29	0.05
E2	GY	1A	AX.89398334	5.05E+08	0.32	-2.32	3.82	0.06
E2	GY	1A	AX.89398334	5.05E+08	0.32	-2.32	3.82	0.06
E2	GY	1A	AX.89538619	5.05E+08	0.33	-2.14	3.34	0.05
E2	GY	1A	AX.89538619	5.05E+08	0.33	-2.14	3.34	0.05
E2	GY	1A	AX.89492546	5.03E+08	0.39	-2.02	3.29	0.04
E2	GY	1A	AX.89375325	5.03E+08	0.39	-2.01	3.29	0.05
E2	GY	1A	AX.89363858	5.05E+08	0.33	-2.11	3.24	0.05
E2	GY	1A	AX.89570128	5.04E+08	0.38	-2.14	3.62	0.05

E2	GY	1A	AX.89364537	5.05E+08	0.32	-2.25	3.59	0.05
E2	GY	1A	AX.89364537	5.05E+08	0.32	-2.25	3.59	0.05
E2	GPM2	1A	AX.89429909	5.73E+08	0.07	-1224.30	4.47	0.08
E2	GY	1A	AX.89330607	5.40E+08	0.22	-2.64	3.92	0.07
E2	GY	1A	AX.89684853	5.40E+08	0.21	-2.35	3.09	0.05
E2	GY	1A	AX.89684853	5.40E+08	0.21	-2.35	3.09	0.05
E2	GY	1A	AX.89401575	5.03E+08	0.39	-1.97	3.15	0.04
E2	GY	1A	AX.89484484	5.06E+08	0.30	-2.16	3.24	0.05
E2	GY	1A	AX.89719310	5.06E+08	0.20	-2.34	3.06	0.05
E2	GY	1A	AX.89542027	5.40E+08	0.21	-2.35	3.14	0.05
E2	GY	1A	AX.89542027	5.40E+08	0.21	-2.35	3.14	0.05
E2	GY	1A	AX.89402369	5.04E+08	0.33	-2.09	3.22	0.05
E2	GY	1A	AX.89402369	5.04E+08	0.33	-2.09	3.22	0.05
E2	GY	1A	AX.89736384	5.05E+08	0.33	-2.11	3.24	0.05
E2	GY	1A	AX.89760710	5.05E+08	0.32	-2.14	3.30	0.05
E2	GY	1A	AX.89577707	5.05E+08	0.33	-2.11	3.24	0.05
E2	TKW	1A	AX.89706863	2.17E+07	0.17	-1.24	3.22	0.05
E2	GY	1A	AX.89415545	5.05E+08	0.33	-2.11	3.24	0.05
E2	GY	1A	AX.89521467	5.05E+08	0.33	-2.11	3.24	0.05
E2	GY	1A	AX.89356842	5.04E+08	0.33	-2.09	3.22	0.05
E2	GY	1A	AX.89369571	5.05E+08	0.33	-2.11	3.24	0.05
E2	GY	1A	AX.89600738	5.02E+08	0.39	-2.09	3.49	0.05
E2	GY	1A	AX.89608660	4.50E+08	0.08	-3.39	3.02	0.05
E2	GY	1A	AX.89617974	5.04E+08	0.23	-2.26	3.09	0.05
E2	GY	1A	AX.89465558	5.40E+08	0.21	-2.35	3.14	0.05
E2	GY	1A	AX.89337165	5.05E+08	0.32	-2.38	3.96	0.06
E2	GY	1A	AX.89337165	5.05E+08	0.32	-2.38	3.96	0.06
E2	GY	1A	AX.89655864	5.05E+08	0.33	-2.23	3.55	0.05
E2	GY	1A	AX.89655864	5.05E+08	0.33	-2.23	3.55	0.05
E2	GY	1A	AX.89338125	5.05E+08	0.33	-2.11	3.24	0.05
E2	GY	1A	AX.89681579	5.05E+08	0.15	-2.82	3.44	0.06
E2	GY	1A	AX.89538300	5.01E+08	0.39	-2.01	3.29	0.05
E2	GY	1A	AX.89538300	5.01E+08	0.39	-2.01	3.29	0.05
E2	GY	1A	AX.89722818	5.05E+08	0.33	-2.28	3.70	0.06
E2	GY	1A	AX.89351520	5.05E+08	0.33	-2.14	3.34	0.05
E2	GY	1A	AX.89375325	5.03E+08	0.39	-2.01	3.29	0.05
E2	GPS	1A	AX.89378347	5.78E+08	0.25	1.62	3.43	0.06
E2	GY	1A	AX.89660138	5.05E+08	0.33	-2.14	3.34	0.05
E2	GY	1A	AX.89660138	5.05E+08	0.33	-2.14	3.34	0.05
E2	GY	1A	AX.89648239	5.05E+08	0.33	-2.11	3.24	0.05
E2	GY	1A	AX.89648239	5.05E+08	0.33	-2.11	3.24	0.05
E2	GY	1A	AX.89484484	5.06E+08	0.30	-2.16	3.24	0.05
E2	GY	1A	AX.89507183	5.05E+08	0.33	-2.11	3.24	0.05
E2	GY	1A	AX.89699261	3.78E+06	0.06	-4.27	3.29	0.06
E2	GY	1A	AX.89736384	5.05E+08	0.33	-2.11	3.24	0.05
E2	GY	1A	AX.89736876	5.05E+08	0.24	-2.33	3.30	0.05
E2	GY	1A	AX.89736876	5.05E+08	0.24	-2.33	3.30	0.05
E2	GY	1A	AX.89415545	5.05E+08	0.33	-2.11	3.24	0.05
E2	GY	1A	AX.89521467	5.05E+08	0.33	-2.11	3.24	0.05
E2	GY	1A	AX.89749368	5.05E+08	0.32	-2.32	3.82	0.06

E2	GY	1A	AX.89749368	5.05E+08	0.32	-2.32	3.82	0.06
E2	GY	1A	AX.89639528	5.40E+08	0.21	-2.35	3.14	0.05
E2	GY	1A	AX.89639528	5.40E+08	0.21	-2.35	3.14	0.05
E2	GY	1A	AX.89346530	5.03E+08	0.39	-2.01	3.29	0.05
E2	GY	1A	AX.89346530	5.03E+08	0.39	-2.01	3.29	0.05
E2	GY	1A	AX.89569895	5.05E+08	0.33	-2.11	3.24	0.05
E2	GY	1A	AX.89419058	5.40E+08	0.21	-2.35	3.14	0.05
E2	GY	1A	AX.89419058	5.40E+08	0.21	-2.35	3.14	0.05
E2	GY	1A	AX.89419399	5.03E+08	0.39	-2.02	3.29	0.04
E2	GY	1A	AX.89419399	5.03E+08	0.39	-2.02	3.29	0.04
E2	GY	1A	AX.89454402	4.50E+08	0.08	-3.39	3.02	0.05
E2	GY	1A	AX.89454402	4.50E+08	0.08	-3.39	3.02	0.05
E2	GY	1A	AX.89469304	5.05E+08	0.33	-2.11	3.24	0.05
E2	GY	1A	AX.89469304	5.05E+08	0.33	-2.11	3.24	0.05
E2	TKW	1A	AX.89319322	2.16E+07	0.17	-1.24	3.22	0.05
E2	GY	1A	AX.89744167	5.03E+08	0.39	-2.02	3.29	0.04
E2	GY	1A	AX.89744167	5.03E+08	0.39	-2.02	3.29	0.04
E2	GY	1A	AX.89351520	5.05E+08	0.33	-2.14	3.34	0.05
E2	GY	1A	AX.89363858	5.05E+08	0.33	-2.11	3.24	0.05
E2	GY	1A	AX.89570128	5.04E+08	0.38	-2.14	3.62	0.05
E2	TKW	1A	AX.89494091	2.16E+07	0.15	-1.34	3.44	0.06
E2	GY	1A	AX.89682387	5.40E+08	0.21	-2.35	3.14	0.05
E2	GY	1A	AX.89682387	5.40E+08	0.21	-2.35	3.14	0.05
E2	TKW	1A	AX.89574309	2.16E+07	0.16	-1.32	3.42	0.06
E2	GY	1A	AX.89635890	5.05E+08	0.29	-2.11	3.08	0.05
E2	TKW	1A	AX.89440584	2.16E+07	0.21	-1.19	3.45	0.06
E2	GY	1A	AX.89638843	4.93E+08	0.37	-1.96	3.08	0.05
E2	GY	1A	AX.89638843	4.93E+08	0.37	-1.96	3.08	0.05
E2	GY	1A	AX.89507183	5.05E+08	0.33	-2.11	3.24	0.05
E2	GY	1A	AX.89632269	5.03E+08	0.39	-2.02	3.29	0.04
E2	GY	1A	AX.89632269	5.03E+08	0.39	-2.02	3.29	0.04
E2	GY	1A	AX.89699261	3.78E+06	0.06	-4.27	3.29	0.06
E2	GY	1A	AX.89426368	5.46E+08	0.05	-4.39	3.14	0.04
E2	GY	1A	AX.89577707	5.05E+08	0.33	-2.11	3.24	0.05
E2	GY	1A	AX.89535903	5.05E+08	0.15	-2.82	3.44	0.06
E2	GY	1A	AX.89600738	5.02E+08	0.39	-2.09	3.49	0.05
E2	GY	1A	AX.89738587	5.01E+08	0.39	-2.01	3.29	0.05
E2	GY	1A	AX.89409765	5.74E+08	0.18	-3.01	4.26	0.08
E2	GY	1A	AX.89409765	5.74E+08	0.18	-3.01	4.26	0.08
E2	GY	1A	AX.89657762	5.04E+08	0.39	-2.02	3.29	0.04
E2	GY	1A	AX.89485112	5.40E+08	0.21	-2.35	3.14	0.05
E2	GY	1A	AX.89485112	5.40E+08	0.21	-2.35	3.14	0.05
E2	GY	1A	AX.89569895	5.05E+08	0.33	-2.11	3.24	0.05
E2	GY	1A	AX.89535903	5.05E+08	0.15	-2.82	3.44	0.06
E2	GY	1A	AX.89570710	5.05E+08	0.33	-2.28	3.70	0.06
E2	GY	1A	AX.89570710	5.05E+08	0.33	-2.28	3.70	0.06
E2	GY	1A	AX.89537350	5.02E+08	0.39	-2.01	3.29	0.05
E2	GY	1A	AX.89537350	5.02E+08	0.39	-2.01	3.29	0.05
E2	GY	1A	AX.89765317	5.05E+08	0.33	-2.11	3.24	0.05
E2	GY	1A	AX.89657762	5.04E+08	0.39	-2.02	3.29	0.04

E2	TKW	1A	AX.89584068	2.16E+07	0.15	-1.41	3.67	0.06
E2	GY	1A	AX.89700128	5.03E+08	0.39	-2.02	3.29	0.04
E2	TKW	1A	AX.89578895	2.31E+07	0.50	0.91	3.07	0.05
E2	GY	1A	AX.89635890	5.05E+08	0.29	-2.11	3.08	0.05
E2	GY	1A	AX.89719310	5.06E+08	0.20	-2.34	3.06	0.05
E2	TKW	1A	AX.89714226	4.81E+08	0.06	-1.88	3.03	0.05
E2	GY	1A	AX.89414928	5.03E+08	0.39	-2.02	3.29	0.04
E2	GY	1A	AX.89760969	5.05E+08	0.33	-2.11	3.24	0.05
E2	TKW	1A	AX.89408380	2.16E+07	0.20	1.15	3.14	0.06
E2	TKW	1A	AX.89380066	4.77E+08	0.22	-1.11	3.10	0.05
E2	GY	1A	AX.89608660	4.50E+08	0.08	-3.39	3.02	0.05
E2	TKW	1A	AX.89448839	2.16E+07	0.15	-1.34	3.44	0.06
E2	GY	1A	AX.89700128	5.03E+08	0.39	-2.02	3.29	0.04
E2	TKW	1A	AX.89666034	2.17E+07	0.15	-1.30	3.13	0.05
E2	GY	1A	AX.89760969	5.05E+08	0.33	-2.11	3.24	0.05
E2	GY	1A	AX.89730577	5.40E+08	0.21	-2.35	3.14	0.05
E2	GY	1A	AX.89487373	5.03E+08	0.39	-2.01	3.29	0.05
E2	GY	1A	AX.89487373	5.03E+08	0.39	-2.01	3.29	0.05
E2	GY	1A	AX.89596164	5.05E+08	0.32	-2.13	3.26	0.05
E2	TKW	1A	AX.89728010	2.17E+07	0.15	-1.29	3.10	0.05
E2	TKW	1A	AX.89463679	2.17E+07	0.15	-1.26	3.09	0.05
E2	GY	1A	AX.89765317	5.05E+08	0.33	-2.11	3.24	0.05
E2	GY	1A	AX.89414928	5.03E+08	0.39	-2.02	3.29	0.04
E2	GY	1A	AX.89738587	5.01E+08	0.39	-2.01	3.29	0.05
E2	TKW	1A	AX.89750504	2.09E+07	0.26	-1.11	3.47	0.07
E2	TKW	1A	AX.89542340	2.09E+07	0.26	-1.10	3.38	0.06
E2	TKW	1A	AX.89557788	2.16E+07	0.19	1.19	3.23	0.06
E2	TKW	1A	AX.89346508	2.17E+07	0.16	-1.32	3.51	0.06
E2	TKW	1A	AX.89602125	2.17E+07	0.15	-1.34	3.44	0.06
E2	GY	1A	AX.89697471	5.05E+08	0.33	-2.11	3.24	0.05
E2	GY	1A	AX.89596164	5.05E+08	0.32	-2.13	3.26	0.05
E2	TKW	1A	AX.89655153	2.16E+07	0.15	-1.34	3.44	0.06
E2	TKW	1A	AX.89590906	1.41E+07	0.50	-0.94	3.24	0.06
E2	GY	1A	AX.89730577	5.40E+08	0.21	-2.35	3.14	0.05
E2	TKW	1A	AX.89418654	2.31E+07	0.49	-0.99	3.56	0.06
E2	TKW	1A	AX.89366384	2.17E+07	0.17	-1.24	3.22	0.05
E2	TKW	1A	AX.89476670	4.81E+08	0.24	-1.09	3.22	0.05
E2	GY	1A	AX.89697471	5.05E+08	0.33	-2.11	3.24	0.05
E2	TKW	1A	AX.89534876	2.17E+07	0.15	-1.31	3.22	0.05
E2	TKW	1A	AX.89378885	2.16E+07	0.15	-1.34	3.44	0.06
E2	TKW	1A	AX.89407344	2.02E+07	0.35	-0.99	3.26	0.06
E2	TKW	1A	AX.89663788	2.16E+07	0.15	-1.34	3.44	0.06
E2	TKW	1A	AX.89534049	4.78E+08	0.22	-1.16	3.34	0.06
E3	GPM2	1A	AX.89464130	4.82E+08	0.26	-969.29	3.01	0.00
E3	GPM2	1A	AX.89703711	4.82E+08	0.26	-991.37	3.06	0.01
E3	GPM2	1A	AX.89632557	4.82E+08	0.26	-991.37	3.06	0.01
E3	GPM2	1A	AX.89557563	4.82E+08	0.21	-1143.48	3.52	0.00
E3	GPM2	1A	AX.89508163	4.82E+08	0.26	-991.37	3.06	0.01
E3	GPM2	1A	AX.89664895	4.82E+08	0.21	-1148.29	3.43	0.00
E3	GPM2	1A	AX.89727551	4.82E+08	0.21	-1151.91	3.51	0.00

E3	GPM2	1A	AX.89755257	4.82E+08	0.21	-1153.39	3.52	0.00
E3	GPM2	1A	AX.89432032	4.82E+08	0.21	-1151.91	3.51	0.00
E3	GPM2	1A	AX.89718387	4.82E+08	0.21	-1151.91	3.51	0.00
E3	GPM2	1A	AX.89364617	4.82E+08	0.21	-1151.91	3.51	0.00
E3	GPM2	1A	AX.89450425	4.82E+08	0.21	-1151.91	3.51	0.00
E3	GPM2	1A	AX.89523174	4.82E+08	0.21	-1151.91	3.51	0.00
E3	GPM2	1A	AX.89375214	5.86E+08	0.10	-1361.62	3.13	0.06
E3	GPM2	1A	AX.89375577	5.87E+08	0.10	-1366.10	3.10	0.06
E3	GPM2	1A	AX.89484684	4.82E+08	0.21	-1214.94	3.79	0.01
E3	GPM2	1A	AX.89443881	4.82E+08	0.21	-1106.15	3.27	0.00
E3	GPM2	1A	AX.89594129	4.82E+08	0.21	-1131.02	3.35	0.00
E3	GPM2	1A	AX.89597359	4.82E+08	0.40	-893.87	3.12	0.00
E3	GPM2	1A	AX.89526819	4.82E+08	0.40	-893.87	3.12	0.00
E3	GPM2	1A	AX.89693123	4.82E+08	0.38	-966.17	3.45	0.00
E3	GPM2	1A	AX.89574228	4.82E+08	0.38	-966.17	3.45	0.00
E3	GPM2	1A	AX.89532401	4.82E+08	0.26	-993.82	3.11	0.01
E3	GPM2	1A	AX.89343225	4.82E+08	0.26	-993.82	3.11	0.01
E3	GY	1A	AX.89693123	4.82E+08	0.38	-3.16	3.31	0.00
E3	GY	1A	AX.89574228	4.82E+08	0.38	-3.16	3.31	0.00
E3	GY	1A	AX.89484684	4.82E+08	0.21	-3.70	3.21	0.01
E3	GY	1A	AX.89392150	5.68E+08	0.18	-3.82	3.23	0.08
E3	GY	1A	AX.89375214	5.86E+08	0.10	-4.78	3.40	0.08
E3	GY	1A	AX.89375577	5.87E+08	0.10	-4.53	3.05	0.08
E3	GPS	1A	AX.89604352	1.24E+07	0.29	2.19	3.26	0.08
E3	GPS	1A	AX.89571090	1.24E+07	0.29	2.12	3.06	0.08
E3	GPS	1A	AX.89355817	1.25E+07	0.28	2.33	3.45	0.10
E3	GPS	1A	AX.89604123	1.39E+07	0.24	2.44	3.42	0.10
E3	GPS	1A	AX.89408581	1.40E+07	0.26	2.59	4.14	0.11
E3	GPS	1A	AX.89507359	1.40E+07	0.27	2.52	3.89	0.10
E3	GPS	1A	AX.89362896	1.40E+07	0.26	2.27	3.17	0.09
E3	GPS	1A	AX.89495208	1.40E+07	0.28	2.65	4.33	0.11
E3	GPS	1A	AX.89662596	1.40E+07	0.27	2.52	3.89	0.10
E3	GPS	1A	AX.89370300	1.41E+07	0.26	2.27	3.17	0.09
E3	GPS	1A	AX.89494107	1.41E+07	0.26	2.27	3.17	0.09
E3	GPS	1A	AX.89588489	1.41E+07	0.17	3.08	3.72	0.11
E3	GPS	1A	AX.89318290	5.64E+08	0.18	2.81	3.47	0.11
E3	GPS	1A	AX.89415841	5.79E+08	0.42	-1.95	3.19	0.07
E3	GPS	1A	AX.89511417	5.83E+08	0.42	1.92	3.01	0.03
E3	GPS	1A	AX.89732974	5.83E+08	0.42	1.92	3.01	0.03
E3	GPS	1A	AX.89434555	5.83E+08	0.42	1.92	3.01	0.03
E3	GPS	1A	AX.89694162	5.83E+08	0.42	1.92	3.01	0.03
E3	SPM2	1A	AX.89510856	2.92E+07	0.32	21.29	3.14	0.05
E3	SPM2	1A	AX.89764618	3.93E+08	0.48	23.08	4.01	0.06
E3	SPM2	1A	AX.89772406	4.03E+08	0.34	25.56	4.47	0.06
E3	SPM2	1A	AX.89438882	4.06E+08	0.46	-25.72	4.60	0.07
E3	SPM2	1A	AX.89764057	4.36E+08	0.16	29.26	3.82	0.03
E3	SPM2	1A	AX.89352646	5.35E+08	0.48	-20.50	3.04	0.05
E3	SPM2	1A	AX.89736206	5.40E+08	0.06	40.75	3.02	0.04
E3	TKW	1A	AX.89733294	5.01E+08	0.14	1.17	3.04	0.00
E4	GPM2	1A	AX.89604123	1.39E+07	0.25	536.11	3.55	0.07

E4	GPM2	1A	AX.89665757	1.40E+07	0.23	518.49	3.20	0.06
E4	GPM2	1A	AX.89520082	1.52E+07	0.27	471.65	3.04	0.06
E4	GPM2	1A	AX.89625350	1.52E+07	0.26	473.99	3.01	0.07
E4	GPM2	1A	AX.89473124	1.53E+07	0.44	-477.22	3.36	0.09
E4	GPS	1A	AX.89678958	1.24E+07	0.40	-1.58	3.05	0.07
E4	GPM2	1A	AX.89486957	1.48E+07	0.27	490.96	3.23	0.07
E4	GPM2	1A	AX.89495221	1.49E+07	0.27	490.96	3.23	0.07
E4	GPM2	1A	AX.89537097	1.51E+07	0.27	475.66	3.08	0.06
E4	GPS	1A	AX.89662264	1.47E+07	0.39	1.69	3.91	0.06
E4	GPS	1A	AX.89399429	1.47E+07	0.38	1.55	3.38	0.05
E4	GPS	1A	AX.89467895	1.47E+07	0.38	1.53	3.29	0.05
E4	GPS	1A	AX.89443156	1.47E+07	0.39	1.46	3.08	0.05
E4	GPS	1A	AX.89520082	1.52E+07	0.27	1.66	3.25	0.06
E4	GPS	1A	AX.89714898	1.52E+07	0.50	-1.52	3.38	0.03
E4	GY	1A	X.89740781_OT	5.84E+08	0.09	2.63	3.18	0.02
E4	GY	1A	X.89615284_OT	5.84E+08	0.10	2.48	3.03	0.02
E4	GY	1A	X.89454122_OT	5.84E+08	0.10	2.68	3.56	0.03
E4	GY	1A	X.89569829_OT	5.84E+08	0.10	2.50	3.07	0.03
E4	SPM2	1A	AX.89438882	4.06E+08	0.43	-18.06	3.36	0.06
E4	TKW	1A	AX.89684335	1.21E+07	0.32	-0.84	3.03	0.05
E4	TKW	1A	AX.89607744	1.24E+07	0.37	-0.91	3.61	0.06
E4	TKW	1A	AX.89618389	1.24E+07	0.42	0.80	3.09	0.03
E4	TKW	1A	AX.89459714	5.89E+08	0.28	-0.94	3.07	0.05
E1	GY	1B	AX.89557668	5.75E+08	0.09	-4.33	3.64	0.06
E1	GY	1B	AX.89402478	5.63E+08	0.48	-2.36	3.25	0.05
E1	GY	1B	AX.89310034	5.50E+08	0.26	-3.07	4.11	0.06
E1	GY	1B	AX.89535186	5.72E+08	0.11	-3.78	3.32	0.05
E1	GY	1B	AX.89531860	5.48E+08	0.19	-3.13	3.50	0.05
E1	GY	1B	AX.89346649	5.44E+08	0.44	-2.29	3.06	0.05
E1	GY	1B	AX.89376997	6.14E+08	0.18	-3.08	3.24	0.05
E1	GY	1B	AX.89518409	5.91E+08	0.26	-2.71	3.27	0.05
E1	GY	1B	AX.89552444	5.43E+08	0.27	-3.68	5.78	0.09
E1	GY	1B	AX.89617894	6.14E+08	0.20	-3.19	3.67	0.06
E1	GY	1B	AX.89537204	6.13E+08	0.17	-3.08	3.11	0.05
E1	GY	1B	AX.89408237	6.14E+08	0.20	-3.19	3.67	0.06
E1	GY	1B	AX.89510480	6.14E+08	0.20	-3.16	3.67	0.06
E1	GY	1B	AX.89679299	6.14E+08	0.20	-3.19	3.67	0.06
E1	GY	1B	AX.89340249	6.14E+08	0.20	-3.19	3.67	0.06
E1	GY	1B	AX.89670926	6.14E+08	0.20	-3.19	3.67	0.06
E1	GY	1B	AX.89586596	5.89E+08	0.27	-2.70	3.35	0.06
E1	GY	1B	AX.89456394	6.15E+08	0.20	-3.18	3.71	0.06
E1	GY	1B	AX.89492642	5.73E+08	0.08	-4.44	3.50	0.05
E1	GY	1B	AX.89345885	5.43E+08	0.44	-2.82	4.39	0.08
E1	GY	1B	AX.89324925	5.43E+08	0.41	-2.68	3.95	0.07
E1	GY	1B	AX.89389412	6.14E+08	0.19	-3.01	3.23	0.05
E1	GY	1B	AX.89428423	5.32E+08	0.18	-3.23	3.50	0.05
E1	GY	1B	AX.89670926	6.14E+08	0.20	-3.19	3.67	0.06
E1	GY	1B	AX.89460697	5.62E+08	0.17	-3.59	4.14	0.06
E1	GY	1B	AX.89463857	5.91E+08	0.25	-2.69	3.20	0.05
E1	GY	1B	AX.89380688	5.90E+08	0.27	-2.70	3.35	0.06

E1	GY	1B	AX.89518107	5.95E+08	0.15	-3.47	3.52	0.05
E1	GY	1B	AX.89414649	5.71E+08	0.09	-4.20	3.32	0.05
E1	GY	1B	AX.89310034	5.50E+08	0.26	-3.07	4.11	0.06
E1	GY	1B	AX.89343729	6.14E+08	0.20	-3.18	3.71	0.06
E1	GY	1B	AX.89324925	5.43E+08	0.41	-2.68	3.95	0.07
E1	GY	1B	AX.89449310	6.15E+08	0.20	-3.18	3.71	0.06
E1	GY	1B	AX.89693737	5.74E+08	0.09	-4.33	3.64	0.06
E1	GY	1B	AX.89470072	5.71E+08	0.09	-4.20	3.32	0.05
E1	GY	1B	AX.89457977	4.74E+08	0.14	-3.52	3.53	0.06
E1	GY	1B	AX.89322211	5.71E+08	0.09	-4.20	3.32	0.05
E1	GY	1B	AX.89520692	5.72E+08	0.11	-3.78	3.32	0.05
E1	GY	1B	AX.89518409	5.91E+08	0.26	-2.71	3.27	0.05
E1	GY	1B	AX.89605695	5.71E+08	0.09	-4.20	3.32	0.05
E1	GY	1B	AX.89310249	5.91E+08	0.10	-4.12	3.60	0.06
E1	GY	1B	AX.89693737	5.74E+08	0.09	-4.33	3.64	0.06
E1	GY	1B	AX.89531860	5.48E+08	0.19	-3.13	3.50	0.05
E1	GY	1B	AX.89526974	6.15E+08	0.20	-3.18	3.71	0.06
E1	GY	1B	AX.89614985	5.73E+08	0.08	-4.44	3.50	0.05
E1	GY	1B	AX.89663827	4.81E+08	0.13	3.35	3.02	0.04
E1	GY	1B	AX.89663827	4.81E+08	0.13	3.35	3.02	0.04
E1	GY	1B	AX.89415762	5.91E+08	0.26	-2.71	3.27	0.05
E1	GY	1B	AX.89310249	5.91E+08	0.10	-4.12	3.60	0.06
E1	GY	1B	AX.89564115	5.76E+08	0.09	-4.33	3.64	0.06
E1	GY	1B	AX.89424158	5.29E+08	0.35	2.36	3.02	0.04
E1	GY	1B	AX.89402478	5.63E+08	0.48	-2.36	3.25	0.05
E1	GY	1B	AX.89336594	6.15E+08	0.20	-3.18	3.71	0.06
E1	GY	1B	AX.89584240	5.43E+08	0.27	-3.68	5.78	0.09
E1	GY	1B	AX.89506992	5.45E+08	0.19	-3.12	3.49	0.05
E1	GY	1B	AX.89401886	5.95E+08	0.14	-3.50	3.50	0.05
E1	GY	1B	AX.89461734	5.49E+08	0.27	-3.07	4.15	0.06
E1	GY	1B	AX.89541920	5.43E+08	0.27	-2.85	3.65	0.05
E1	GY	1B	AX.89376997	6.14E+08	0.18	-3.08	3.24	0.05
E1	GY	1B	AX.89374979	5.47E+08	0.12	-3.52	3.11	0.05
E1	GY	1B	AX.89346649	5.44E+08	0.44	-2.29	3.06	0.05
E1	GY	1B	AX.89507929	5.43E+08	0.44	-2.82	4.39	0.08
E1	GY	1B	AX.89449310	6.15E+08	0.20	-3.18	3.71	0.06
E1	GY	1B	AX.89373658	5.32E+08	0.24	-3.26	4.37	0.07
E1	GY	1B	AX.89347349	5.94E+08	0.14	-3.50	3.50	0.05
E1	GY	1B	AX.89520572	5.47E+08	0.33	-2.52	3.30	0.05
E1	GY	1B	AX.89539367	5.92E+08	0.28	-2.65	3.34	0.05
E1	GY	1B	AX.89340424	5.62E+08	0.28	-2.69	3.42	0.05
E1	GY	1B	AX.89614302	5.62E+08	0.29	-2.70	3.47	0.05
E1	GY	1B	AX.89344300	5.89E+08	0.27	-2.70	3.35	0.06
E1	GY	1B	AX.89336594	6.15E+08	0.20	-3.18	3.71	0.06
E1	GY	1B	AX.89526974	6.15E+08	0.20	-3.18	3.71	0.06
E1	GY	1B	AX.89336833	6.15E+08	0.20	-3.18	3.71	0.06
E1	GY	1B	AX.89510021	5.73E+08	0.08	-4.44	3.50	0.05
E1	GY	1B	AX.89725397	5.63E+08	0.48	-2.36	3.25	0.05
E1	GY	1B	AX.89505604	5.92E+08	0.26	-2.71	3.27	0.05
E1	GY	1B	AX.89401180	5.71E+08	0.09	-4.20	3.32	0.05

E1	GY	1B	AX.89401180	5.71E+08	0.09	-4.20	3.32	0.05
E1	GY	1B	AX.89380569	5.92E+08	0.26	-2.71	3.27	0.05
E1	GY	1B	AX.89530956	5.43E+08	0.44	-2.82	4.39	0.08
E1	GY	1B	AX.89604422	5.91E+08	0.25	-2.69	3.20	0.05
E1	GY	1B	AX.89361588	5.48E+08	0.19	-3.13	3.50	0.05
E1	GY	1B	AX.89515088	5.92E+08	0.26	-2.71	3.27	0.05
E1	SPM2	1B	AX.89606726	4.45E+07	0.44	-19.00	3.92	0.07
E1	GY	1B	AX.89383273	5.91E+08	0.25	-2.69	3.20	0.05
E1	GY	1B	AX.89531457	6.14E+08	0.20	-3.18	3.71	0.06
E1	GY	1B	AX.89361803	5.63E+08	0.48	-2.36	3.25	0.05
E1	GY	1B	AX.89397437	5.93E+08	0.13	-3.89	4.01	0.06
E1	GY	1B	AX.89346018	5.92E+08	0.28	-2.65	3.34	0.05
E1	GY	1B	AX.89498745	5.76E+08	0.09	-4.33	3.64	0.06
E1	GY	1B	AX.89381273	6.15E+08	0.20	-3.18	3.71	0.06
E1	GY	1B	AX.89491706	5.64E+08	0.12	-3.79	3.53	0.05
E1	GY	1B	AX.89491706	5.64E+08	0.12	-3.79	3.53	0.05
E1	GY	1B	AX.89392454	5.95E+08	0.14	-3.50	3.50	0.05
E1	GY	1B	AX.89534408	5.71E+08	0.09	-4.20	3.32	0.05
E1	GY	1B	AX.89743822	5.48E+08	0.19	-3.13	3.50	0.05
E1	GY	1B	AX.89572193	5.72E+08	0.09	-4.20	3.32	0.05
E1	GY	1B	AX.89339341	4.30E+08	0.39	2.57	3.60	0.07
E1	GY	1B	AX.89588573	5.91E+08	0.25	-2.69	3.20	0.05
E1	GY	1B	AX.89414649	5.71E+08	0.09	-4.20	3.32	0.05
E1	GY	1B	AX.89456394	6.15E+08	0.20	-3.18	3.71	0.06
E1	GY	1B	AX.89504198	6.14E+08	0.19	-2.97	3.21	0.05
E1	GY	1B	AX.89399318	5.95E+08	0.15	-3.72	3.98	0.06
E1	GY	1B	AX.89339603	6.13E+08	0.14	-3.73	3.91	0.06
E1	GY	1B	AX.89317797	5.95E+08	0.14	-3.50	3.50	0.05
E1	GY	1B	AX.89317797	5.95E+08	0.14	-3.50	3.50	0.05
E1	GY	1B	AX.89484632	5.73E+08	0.09	-4.93	4.38	0.07
E1	GY	1B	AX.89671300	5.43E+08	0.46	-2.72	4.15	0.08
E1	GY	1B	AX.89621777	5.43E+08	0.43	-2.62	3.84	0.07
E1	GY	1B	AX.89609372	5.73E+08	0.08	-4.44	3.50	0.05
E1	GY	1B	AX.89614302	5.62E+08	0.29	-2.70	3.47	0.05
E1	GY	1B	AX.89460697	5.62E+08	0.17	-3.59	4.14	0.06
E1	GY	1B	AX.89721599	5.94E+08	0.15	-3.72	3.98	0.06
E1	GY	1B	AX.89347943	5.46E+08	0.33	-2.57	3.42	0.05
E1	GY	1B	AX.89446794	5.50E+08	0.27	-2.77	3.54	0.05
E1	GY	1B	AX.89457977	4.74E+08	0.14	-3.52	3.53	0.06
E1	GY	1B	AX.89635557	5.32E+08	0.20	-3.18	3.65	0.06
E1	GY	1B	AX.89635557	5.32E+08	0.20	-3.18	3.65	0.06
E1	GY	1B	AX.89587950	5.92E+08	0.26	-2.71	3.27	0.05
E1	GY	1B	AX.89564115	5.76E+08	0.09	-4.33	3.64	0.06
E1	GY	1B	AX.89466319	5.71E+08	0.11	-3.78	3.32	0.05
E1	GY	1B	AX.89564492	5.71E+08	0.09	-4.20	3.32	0.05
E1	GY	1B	AX.89466421	5.91E+08	0.26	-2.70	3.25	0.05
E1	GY	1B	AX.89644425	5.71E+08	0.09	-4.20	3.32	0.05
E1	GY	1B	AX.89397557	5.94E+08	0.15	-3.69	4.00	0.06
E1	GY	1B	AX.89342515	6.14E+08	0.20	-3.19	3.67	0.06
E1	GY	1B	AX.89498745	5.76E+08	0.09	-4.33	3.64	0.06

E1	GY	1B	AX.89412705	5.95E+08	0.15	-3.72	3.98	0.06
E1	GY	1B	AX.89715167	5.76E+08	0.20	-3.14	3.63	0.06
E1	GY	1B	AX.89589298	5.95E+08	0.15	-3.72	3.98	0.06
E1	GY	1B	AX.89541910	5.53E+08	0.06	-5.22	3.57	0.06
E1	GY	1B	AX.89389412	6.14E+08	0.19	-3.01	3.23	0.05
E1	GY	1B	AX.89374979	5.47E+08	0.12	-3.52	3.11	0.05
E1	GY	1B	AX.89414699	5.95E+08	0.15	-3.69	4.00	0.06
E1	GY	1B	AX.89528405	5.89E+08	0.12	-4.11	4.06	0.08
E1	GY	1B	AX.89485879	6.15E+08	0.20	-3.18	3.71	0.06
E1	GY	1B	AX.89426148	5.50E+08	0.29	-2.67	3.41	0.05
E1	GY	1B	AX.89426148	5.50E+08	0.29	-2.67	3.41	0.05
E1	GY	1B	AX.89562690	5.73E+08	0.08	-4.58	3.53	0.06
E1	GY	1B	AX.89524937	6.13E+08	0.14	-3.73	3.91	0.06
E1	GY	1B	AX.89621777	5.43E+08	0.43	-2.62	3.84	0.07
E1	GY	1B	AX.89573650	6.15E+08	0.20	-3.18	3.71	0.06
E1	GY	1B	AX.89472916	4.29E+08	0.39	2.57	3.60	0.07
E1	GY	1B	AX.89415540	5.90E+08	0.27	-2.70	3.35	0.06
E1	GY	1B	AX.89574388	5.62E+08	0.17	-3.62	4.12	0.06
E1	GY	1B	AX.89614985	5.73E+08	0.08	-4.44	3.50	0.05
E1	GY	1B	AX.89676422	5.65E+08	0.14	-3.60	3.59	0.06
E1	GY	1B	AX.89461734	5.49E+08	0.27	-3.07	4.15	0.06
E1	GY	1B	AX.89428423	5.32E+08	0.18	-3.23	3.50	0.05
E1	SPM2	1B	AX.89645430	4.14E+07	0.10	29.82	3.64	0.07
E1	GY	1B	AX.89392454	5.95E+08	0.14	-3.50	3.50	0.05
E1	GY	1B	AX.89342243	6.15E+08	0.20	-3.18	3.71	0.06
E1	GY	1B	AX.89405354	5.28E+08	0.35	2.36	3.02	0.04
E1	GY	1B	AX.89346018	5.92E+08	0.28	-2.65	3.34	0.05
E1	GY	1B	AX.89459113	5.63E+08	0.48	-2.36	3.25	0.05
E1	GY	1B	AX.89459113	5.63E+08	0.48	-2.36	3.25	0.05
E1	GY	1B	AX.89484632	5.73E+08	0.09	-4.93	4.38	0.07
E1	GY	1B	AX.89715167	5.76E+08	0.20	-3.14	3.63	0.06
E1	GY	1B	AX.89616764	6.14E+08	0.20	-3.19	3.67	0.06
E1	GY	1B	AX.89346098	5.62E+08	0.17	-3.59	4.14	0.06
E1	GY	1B	AX.89346098	5.62E+08	0.17	-3.59	4.14	0.06
E1	GY	1B	AX.89452054	6.15E+08	0.20	-3.19	3.67	0.06
E1	GY	1B	AX.89452054	6.15E+08	0.20	-3.19	3.67	0.06
E1	GY	1B	AX.89334781	5.96E+08	0.15	-3.72	3.98	0.06
E1	GY	1B	AX.89541910	5.53E+08	0.06	-5.22	3.57	0.06
E1	GY	1B	AX.89495626	5.48E+08	0.19	-3.13	3.50	0.05
E1	GY	1B	AX.89561467	5.48E+08	0.20	-3.03	3.36	0.05
E1	GY	1B	AX.89534408	5.71E+08	0.09	-4.20	3.32	0.05
E1	GY	1B	AX.89690662	5.94E+08	0.15	-3.47	3.52	0.05
E1	GY	1B	AX.89339341	4.30E+08	0.39	2.57	3.60	0.07
E1	GY	1B	AX.89496313	6.14E+08	0.20	-3.18	3.71	0.06
E1	GY	1B	AX.89309966	5.48E+08	0.19	-3.13	3.50	0.05
E1	GY	1B	AX.89678864	5.43E+08	0.44	-2.82	4.39	0.08
E1	GY	1B	AX.89678864	5.43E+08	0.44	-2.82	4.39	0.08
E1	GY	1B	AX.89355593	5.89E+08	0.27	-2.70	3.35	0.06
E1	GY	1B	AX.89322211	5.71E+08	0.09	-4.20	3.32	0.05
E1	GY	1B	AX.89395127	6.54E+08	0.12	-3.88	3.58	0.07

E1	GY	1B	AX.89667223	6.14E+08	0.20	-3.19	3.67	0.06
E1	GY	1B	AX.89765386	5.44E+08	0.44	-2.33	3.16	0.05
E1	GY	1B	AX.89442364	5.48E+08	0.19	-3.13	3.50	0.05
E1	GY	1B	AX.89486405	5.94E+08	0.15	-3.72	3.98	0.06
E1	GY	1B	AX.89637878	5.89E+08	0.27	-2.70	3.35	0.06
E1	GY	1B	AX.89355729	5.94E+08	0.15	-3.47	3.52	0.05
E1	GY	1B	AX.89536084	5.46E+08	0.33	-2.57	3.42	0.05
E1	GY	1B	AX.89314421	5.92E+08	0.26	-2.71	3.27	0.05
E1	GY	1B	AX.89415657	6.32E+08	0.07	-6.66	5.82	0.10
E1	GY	1B	AX.89415657	6.32E+08	0.07	-6.66	5.82	0.10
E1	GY	1B	AX.89336833	6.15E+08	0.20	-3.18	3.71	0.06
E1	GY	1B	AX.89465165	5.49E+08	0.27	-3.07	4.15	0.06
E1	GY	1B	AX.89360777	6.14E+08	0.20	-3.19	3.67	0.06
E1	GY	1B	AX.89536555	6.15E+08	0.20	-3.18	3.71	0.06
E1	GY	1B	AX.89454580	6.14E+08	0.20	-3.14	3.68	0.06
E1	GY	1B	AX.89655981	5.75E+08	0.09	-4.33	3.64	0.06
E1	GY	1B	AX.89655981	5.75E+08	0.09	-4.33	3.64	0.06
E1	GY	1B	AX.89409179	4.73E+08	0.14	-3.52	3.53	0.06
E1	GY	1B	AX.89409179	4.73E+08	0.14	-3.52	3.53	0.06
E1	GY	1B	AX.89361355	4.72E+08	0.14	-3.56	3.51	0.06
E1	GY	1B	AX.89349463	5.48E+08	0.19	-3.13	3.50	0.05
E1	GY	1B	AX.89583789	5.71E+08	0.09	-4.20	3.32	0.05
E1	GY	1B	AX.89432897	6.13E+08	0.15	-3.60	3.75	0.06
E1	GY	1B	AX.89432897	6.13E+08	0.15	-3.60	3.75	0.06
E1	GY	1B	AX.89648440	5.71E+08	0.09	-4.20	3.32	0.05
E1	GY	1B	AX.89771478	6.16E+08	0.20	-3.18	3.71	0.06
E1	GY	1B	AX.89361803	5.63E+08	0.48	-2.36	3.25	0.05
E1	GY	1B	AX.89584240	5.43E+08	0.27	-3.68	5.78	0.09
E1	GY	1B	AX.89381080	5.62E+08	0.16	-3.68	4.17	0.07
E1	GY	1B	AX.89506992	5.45E+08	0.19	-3.12	3.49	0.05
E1	GY	1B	AX.89479897	5.96E+08	0.15	-3.72	3.98	0.06
E1	GY	1B	AX.89609372	5.73E+08	0.08	-4.44	3.50	0.05
E1	GY	1B	AX.89317078	6.14E+08	0.20	-3.19	3.67	0.06
E1	GY	1B	AX.89338664	5.62E+08	0.28	-2.82	3.69	0.05
E1	GY	1B	AX.89421570	6.14E+08	0.16	-3.17	3.22	0.05
E1	GY	1B	AX.89754639	5.95E+08	0.14	-3.50	3.50	0.05
E1	GY	1B	AX.89656283	5.32E+08	0.24	-3.26	4.37	0.07
E1	GY	1B	AX.89656283	5.32E+08	0.24	-3.26	4.37	0.07
E1	GY	1B	AX.89430295	6.54E+08	0.13	-3.54	3.23	0.06
E1	GY	1B	AX.89328659	4.73E+08	0.15	-3.39	3.39	0.06
E1	GY	1B	AX.89492642	5.73E+08	0.08	-4.44	3.50	0.05
E1	GY	1B	AX.86161856	5.72E+08	0.11	-3.85	3.31	0.05
E1	GY	1B	AX.89550273	5.94E+08	0.15	-3.69	4.00	0.06
E1	GY	1B	AX.89562690	5.73E+08	0.08	-4.58	3.53	0.06
E1	GY	1B	AX.89468862	6.14E+08	0.13	-3.53	3.39	0.05
E1	GY	1B	AX.89468862	6.14E+08	0.13	-3.53	3.39	0.05
E1	GY	1B	AX.89563144	5.72E+08	0.11	-3.78	3.32	0.05
E1	GY	1B	AX.89421818	4.72E+08	0.14	-3.56	3.51	0.06
E1	GY	1B	AX.89586596	5.89E+08	0.27	-2.70	3.35	0.06
E1	GY	1B	AX.89412388	5.48E+08	0.19	-3.13	3.50	0.05

E1	GY	1B	AX.89697293	5.47E+08	0.33	-2.50	3.24	0.05
E1	GY	1B	AX.89722018	5.90E+08	0.27	-2.80	3.54	0.06
E1	GY	1B	AX.89745504	6.14E+08	0.20	-3.18	3.71	0.06
E1	GY	1B	AX.89518107	5.95E+08	0.15	-3.47	3.52	0.05
E1	GY	1B	AX.89587635	6.16E+08	0.20	-3.18	3.71	0.06
E1	GY	1B	AX.89341296	6.54E+08	0.14	-3.31	3.11	0.06
E1	GY	1B	AX.89659812	5.94E+08	0.15	-3.47	3.52	0.05
E1	GY	1B	AX.89466319	5.71E+08	0.11	-3.78	3.32	0.05
E1	GY	1B	AX.89564492	5.71E+08	0.09	-4.20	3.32	0.05
E1	GY	1B	AX.89342243	6.15E+08	0.20	-3.18	3.71	0.06
E1	GY	1B	AX.89485001	5.95E+08	0.15	-3.72	3.98	0.06
E1	GY	1B	AX.89485001	5.95E+08	0.15	-3.72	3.98	0.06
E1	GY	1B	AX.89735423	6.15E+08	0.20	-3.18	3.71	0.06
E1	GY	1B	AX.89530956	5.43E+08	0.44	-2.82	4.39	0.08
E1	GY	1B	AX.89605695	5.71E+08	0.09	-4.20	3.32	0.05
E1	GY	1B	AX.89735505	5.50E+08	0.26	-2.95	3.80	0.06
E1	GY	1B	AX.89402330	5.93E+08	0.13	-3.89	4.01	0.06
E1	GY	1B	AX.89471727	6.16E+08	0.20	-3.18	3.71	0.06
E1	GY	1B	AX.89720333	5.44E+08	0.45	-2.36	3.23	0.05
E1	GY	1B	AX.89528229	6.15E+08	0.20	-3.18	3.71	0.06
E1	GY	1B	AX.89699324	5.64E+08	0.11	-3.58	3.03	0.05
E1	GY	1B	AX.89686897	4.30E+08	0.39	2.58	3.65	0.07
E1	GY	1B	AX.89590119	5.63E+08	0.12	-3.53	3.04	0.05
E1	GY	1B	AX.89602399	5.72E+08	0.09	-4.20	3.32	0.05
E1	GY	1B	AX.89520284	5.76E+08	0.09	-4.33	3.64	0.06
E1	GY	1B	AX.89343729	6.14E+08	0.20	-3.18	3.71	0.06
E1	GY	1B	AX.89310110	5.71E+08	0.11	-3.78	3.32	0.05
E1	GY	1B	AX.89355593	5.89E+08	0.27	-2.70	3.35	0.06
E1	GY	1B	AX.89558501	5.73E+08	0.08	-4.80	3.83	0.06
E1	GY	1B	AX.89671300	5.43E+08	0.46	-2.72	4.15	0.08
E1	GY	1B	AX.89590842	6.15E+08	0.20	-3.18	3.71	0.06
E1	GY	1B	AX.89310641	5.48E+08	0.19	-3.13	3.50	0.05
E1	GY	1B	AX.89344300	5.89E+08	0.27	-2.70	3.35	0.06
E1	GY	1B	AX.89472916	4.29E+08	0.39	2.57	3.60	0.07
E1	GY	1B	AX.89379733	5.72E+08	0.09	-4.20	3.32	0.05
E1	GY	1B	AX.89379733	5.72E+08	0.09	-4.20	3.32	0.05
E1	GY	1B	AX.89580727	5.96E+08	0.15	-3.72	3.98	0.06
E1	GY	1B	AX.89415540	5.90E+08	0.27	-2.70	3.35	0.06
E1	GY	1B	AX.89574388	5.62E+08	0.17	-3.62	4.12	0.06
E1	GY	1B	AX.89344807	6.16E+08	0.20	-3.18	3.71	0.06
E1	GY	1B	AX.89509761	5.43E+08	0.41	-2.57	3.68	0.06
E1	GY	1B	AX.89591657	5.72E+08	0.09	-4.20	3.32	0.05
E1	GY	1B	AX.89450987	5.71E+08	0.09	-4.20	3.32	0.05
E1	GY	1B	AX.89450987	5.71E+08	0.09	-4.20	3.32	0.05
E1	GY	1B	AX.89404365	5.89E+08	0.27	-2.69	3.30	0.06
E1	GY	1B	AX.89404365	5.89E+08	0.27	-2.69	3.30	0.06
E1	GY	1B	AX.89587950	5.92E+08	0.26	-2.71	3.27	0.05
E1	GY	1B	AX.89380569	5.92E+08	0.26	-2.71	3.27	0.05
E1	GY	1B	AX.89627315	5.73E+08	0.08	-4.44	3.50	0.05
E1	GY	1B	AX.89604422	5.91E+08	0.25	-2.69	3.20	0.05

E1	GY	1B	AX.89428761	5.44E+08	0.45	-2.36	3.23	0.05
E1	GY	1B	AX.89357199	5.94E+08	0.15	-3.47	3.52	0.05
E1	GY	1B	AX.89334216	5.76E+08	0.09	-4.33	3.64	0.06
E1	GY	1B	AX.89588573	5.91E+08	0.25	-2.69	3.20	0.05
E1	GY	1B	AX.89357479	5.43E+08	0.45	-2.57	3.73	0.06
E1	GY	1B	AX.89345885	5.43E+08	0.44	-2.82	4.39	0.08
E1	GY	1B	AX.89488197	5.94E+08	0.15	-3.69	4.00	0.06
E1	GY	1B	AX.89605272	6.16E+08	0.20	-3.18	3.71	0.06
E1	GY	1B	AX.89653613	5.44E+08	0.44	-2.45	3.44	0.06
E1	GY	1B	AX.89541920	5.43E+08	0.27	-2.85	3.65	0.05
E1	GY	1B	AX.89463084	6.14E+08	0.20	-3.19	3.67	0.06
E1	GY	1B	AX.89381566	5.73E+08	0.09	-4.93	4.38	0.07
E1	GY	1B	AX.89381776	4.29E+08	0.39	2.57	3.60	0.07
E1	GY	1B	AX.89580989	6.46E+08	0.07	-4.89	3.58	0.06
E1	GY	1B	AX.89629310	6.14E+08	0.19	-3.07	3.39	0.05
E1	GY	1B	AX.89441819	5.89E+08	0.27	-2.70	3.35	0.06
E1	GY	1B	AX.89524464	5.89E+08	0.27	-2.70	3.35	0.06
E1	GY	1B	AX.89347349	5.94E+08	0.14	-3.50	3.50	0.05
E1	GY	1B	AX.89395127	6.54E+08	0.12	-3.88	3.58	0.07
E1	GY	1B	AX.89359791	5.32E+08	0.22	-3.29	4.16	0.06
E1	GY	1B	AX.89524937	6.13E+08	0.14	-3.73	3.91	0.06
E1	GY	1B	AX.89667223	6.14E+08	0.20	-3.19	3.67	0.06
E1	GY	1B	AX.89581957	5.95E+08	0.15	-3.72	3.98	0.06
E1	GY	1B	AX.89765386	5.44E+08	0.44	-2.33	3.16	0.05
E1	GY	1B	AX.89442364	5.48E+08	0.19	-3.13	3.50	0.05
E1	GY	1B	AX.89431355	6.14E+08	0.20	-3.19	3.67	0.06
E1	GY	1B	AX.89460853	5.92E+08	0.26	-2.71	3.27	0.05
E1	GY	1B	AX.89536040	4.73E+08	0.14	-3.52	3.53	0.06
E1	GY	1B	AX.89347943	5.46E+08	0.33	-2.57	3.42	0.05
E1	GY	1B	AX.89765882	6.46E+08	0.07	-4.89	3.58	0.06
E1	GY	1B	AX.89477534	5.62E+08	0.28	-2.83	3.73	0.06
E1	GY	1B	AX.89391459	4.82E+08	0.13	3.35	3.02	0.04
E1	GY	1B	AX.89513960	6.13E+08	0.16	-3.17	3.22	0.05
E1	GY	1B	AX.89383273	5.91E+08	0.25	-2.69	3.20	0.05
E1	GY	1B	AX.89383439	6.14E+08	0.20	-3.19	3.67	0.06
E1	GY	1B	AX.89536494	5.47E+08	0.19	-3.13	3.50	0.05
E1	GY	1B	AX.89360777	6.14E+08	0.20	-3.19	3.67	0.06
E1	GY	1B	AX.89315126	5.91E+08	0.25	-2.69	3.20	0.05
E1	GY	1B	AX.89384148	5.76E+08	0.09	-4.33	3.64	0.06
E1	GY	1B	AX.89443701	6.15E+08	0.20	-3.18	3.71	0.06
E1	GY	1B	AX.89432547	5.73E+08	0.08	-4.44	3.50	0.05
E1	GY	1B	AX.89454719	5.91E+08	0.10	-4.12	3.60	0.06
E1	GY	1B	AX.89596159	5.90E+08	0.26	-2.63	3.14	0.05
E1	GY	1B	AX.89315865	5.71E+08	0.09	-4.20	3.32	0.05
E1	GY	1B	AX.89596373	5.62E+08	0.28	-2.83	3.73	0.06
E1	GY	1B	AX.89515088	5.92E+08	0.26	-2.71	3.27	0.05
E1	GY	1B	AX.89380858	5.94E+08	0.15	-3.69	4.00	0.06
E1	GY	1B	AX.89466421	5.91E+08	0.26	-2.70	3.25	0.05
E1	GY	1B	AX.89502838	6.15E+08	0.20	-3.18	3.71	0.06
E1	GY	1B	AX.89502838	6.15E+08	0.20	-3.18	3.71	0.06

E1	GY	1B	AX.89702421	5.91E+08	0.25	-2.69	3.20	0.05
E1	GY	1B	AX.89397437	5.93E+08	0.13	-3.89	4.01	0.06
E1	GY	1B	AX.89488197	5.94E+08	0.15	-3.69	4.00	0.06
E1	GY	1B	AX.89657292	5.94E+08	0.13	-3.37	3.05	0.05
E1	GY	1B	AX.89657292	5.94E+08	0.13	-3.37	3.05	0.05
E1	GY	1B	AX.89706711	6.15E+08	0.20	-3.18	3.71	0.06
E1	GY	1B	AX.89374635	5.43E+08	0.43	-2.62	3.84	0.07
E1	GY	1B	AX.89374635	5.43E+08	0.43	-2.62	3.84	0.07
E1	GY	1B	AX.89754639	5.95E+08	0.14	-3.50	3.50	0.05
E1	GY	1B	AX.89743822	5.48E+08	0.19	-3.13	3.50	0.05
E1	GY	1B	AX.89492307	5.75E+08	0.09	-4.33	3.64	0.06
E1	GY	1B	AX.89328659	4.73E+08	0.15	-3.39	3.39	0.06
E1	GY	1B	AX.89434131	5.73E+08	0.08	-4.44	3.50	0.05
E1	GY	1B	AX.89670540	5.71E+08	0.09	-4.20	3.32	0.05
E1	GY	1B	AX.89512987	5.71E+08	0.09	-4.20	3.32	0.05
E1	GY	1B	AX.89708163	5.48E+08	0.19	-3.13	3.50	0.05
E1	GY	1B	AX.89539367	5.92E+08	0.28	-2.65	3.34	0.05
E1	GY	1B	AX.89634659	6.15E+08	0.20	-3.18	3.71	0.06
E1	GY	1B	AX.89675930	5.71E+08	0.09	-4.20	3.32	0.05
E1	GY	1B	AX.89675930	5.71E+08	0.09	-4.20	3.32	0.05
E1	GY	1B	AX.89769760	6.15E+08	0.20	-3.18	3.71	0.06
E1	GY	1B	AX.89387161	5.48E+08	0.19	-3.13	3.50	0.05
E1	GY	1B	AX.89446794	5.50E+08	0.27	-2.77	3.54	0.05
E1	GY	1B	AX.89622537	5.71E+08	0.11	-3.78	3.32	0.05
E1	GY	1B	AX.89622537	5.71E+08	0.11	-3.78	3.32	0.05
E1	GY	1B	AX.89412705	5.95E+08	0.15	-3.72	3.98	0.06
E1	GY	1B	AX.89745504	6.14E+08	0.20	-3.18	3.71	0.06
E1	GY	1B	AX.89659812	5.94E+08	0.15	-3.47	3.52	0.05
E1	GY	1B	AX.89608451	5.95E+08	0.15	-3.69	4.00	0.06
E1	GY	1B	AX.89608451	5.95E+08	0.15	-3.69	4.00	0.06
E1	GY	1B	AX.89753324	5.49E+08	0.49	2.42	3.41	0.05
E1	GY	1B	AX.89494755	6.62E+08	0.05	-6.01	3.98	0.06
E1	GY	1B	AX.89494755	6.62E+08	0.05	-6.01	3.98	0.06
E1	GY	1B	AX.89526794	5.90E+08	0.27	-2.70	3.35	0.06
E1	GY	1B	AX.89648440	5.71E+08	0.09	-4.20	3.32	0.05
E1	GY	1B	AX.89320059	5.95E+08	0.15	-3.72	3.98	0.06
E1	GY	1B	AX.89767080	5.45E+08	0.20	-3.19	3.67	0.06
E1	GY	1B	AX.89734677	5.48E+08	0.19	-3.13	3.50	0.05
E1	GY	1B	AX.89342515	6.14E+08	0.20	-3.19	3.67	0.06
E1	GY	1B	AX.89377815	5.62E+08	0.28	-2.82	3.69	0.05
E1	GY	1B	AX.89377815	5.62E+08	0.28	-2.82	3.69	0.05
E1	GY	1B	AX.89467073	5.32E+08	0.07	-5.27	4.29	0.07
E1	GY	1B	AX.89589298	5.95E+08	0.15	-3.72	3.98	0.06
E1	GY	1B	AX.89495626	5.48E+08	0.19	-3.13	3.50	0.05
E1	GY	1B	AX.89735423	6.15E+08	0.20	-3.18	3.71	0.06
E1	GY	1B	AX.89674301	5.62E+08	0.28	-2.83	3.73	0.06
E1	GY	1B	AX.89735505	5.50E+08	0.26	-2.95	3.80	0.06
E1	GY	1B	AX.89589597	6.14E+08	0.20	-3.18	3.71	0.06
E1	GY	1B	AX.89613526	5.72E+08	0.09	-4.20	3.32	0.05
E1	GY	1B	AX.89637173	5.66E+08	0.14	-3.56	3.61	0.06

E1	GY	1B	AX.89496313	6.14E+08	0.20	-3.18	3.71	0.06
E1	GY	1B	AX.89309966	5.48E+08	0.19	-3.13	3.50	0.05
E1	GY	1B	AX.89520284	5.76E+08	0.09	-4.33	3.64	0.06
E1	GY	1B	AX.89390480	5.89E+08	0.27	-2.70	3.35	0.06
E1	GY	1B	AX.89390480	5.89E+08	0.27	-2.70	3.35	0.06
E1	GY	1B	AX.89310110	5.71E+08	0.11	-3.78	3.32	0.05
E1	GY	1B	AX.89712134	5.92E+08	0.26	-2.71	3.27	0.05
E1	GY	1B	AX.89712141	4.30E+08	0.38	2.49	3.40	0.06
E1	GY	1B	AX.89625447	6.15E+08	0.20	-3.18	3.71	0.06
E1	GY	1B	AX.89625447	6.15E+08	0.20	-3.18	3.71	0.06
E1	GY	1B	AX.89520572	5.47E+08	0.33	-2.52	3.30	0.05
E1	GY	1B	AX.89520692	5.72E+08	0.11	-3.78	3.32	0.05
E1	GY	1B	AX.89736664	5.90E+08	0.10	-4.12	3.60	0.06
E1	GY	1B	AX.89724852	5.94E+08	0.15	-3.69	4.00	0.06
E1	GY	1B	AX.89355729	5.94E+08	0.15	-3.47	3.52	0.05
E1	GY	1B	AX.89460853	5.92E+08	0.26	-2.71	3.27	0.05
E1	GY	1B	AX.89333321	5.50E+08	0.26	-3.07	4.11	0.06
E1	GY	1B	AX.89543657	5.96E+08	0.15	-3.72	3.98	0.06
E1	GY	1B	AX.89543657	5.96E+08	0.15	-3.72	3.98	0.06
E1	GY	1B	AX.89676422	5.65E+08	0.14	-3.60	3.59	0.06
E1	GY	1B	AX.89333721	6.13E+08	0.14	-3.73	3.91	0.06
E1	GY	1B	AX.89775428	5.71E+08	0.09	-4.17	3.28	0.05
E1	GY	1B	AX.89356982	6.54E+08	0.13	-3.54	3.23	0.06
E1	GY	1B	AX.89356982	6.54E+08	0.13	-3.54	3.23	0.06
E1	GY	1B	AX.89568217	5.89E+08	0.13	-4.04	4.06	0.07
E1	GY	1B	AX.89357199	5.94E+08	0.15	-3.47	3.52	0.05
E1	GY	1B	AX.89334216	5.76E+08	0.09	-4.33	3.64	0.06
E1	GY	1B	AX.89544768	5.95E+08	0.15	-3.47	3.52	0.05
E1	GY	1B	AX.89767080	5.45E+08	0.20	-3.19	3.67	0.06
E1	GY	1B	AX.89710848	5.95E+08	0.14	-3.50	3.50	0.05
E1	GY	1B	AX.89381080	5.62E+08	0.16	-3.68	4.17	0.07
E1	GY	1B	AX.89346068	6.14E+08	0.20	-3.19	3.67	0.06
E1	GY	1B	AX.89727207	5.94E+08	0.15	-3.69	4.00	0.06
E1	GY	1B	AX.89334781	5.96E+08	0.15	-3.72	3.98	0.06
E1	GY	1B	AX.89381566	5.73E+08	0.09	-4.93	4.38	0.07
E1	GY	1B	AX.89524034	6.46E+08	0.07	-4.89	3.58	0.06
E1	GY	1B	AX.89430167	5.71E+08	0.08	-4.16	3.00	0.05
E1	GY	1B	AX.89507929	5.43E+08	0.44	-2.82	4.39	0.08
E1	GY	1B	AX.89594265	5.76E+08	0.09	-4.33	3.64	0.06
E1	GY	1B	AX.89325626	5.43E+08	0.41	-2.68	3.95	0.07
E1	GY	1B	AX.89325626	5.43E+08	0.41	-2.68	3.95	0.07
E1	GY	1B	AX.89629310	6.14E+08	0.19	-3.07	3.39	0.05
E1	GY	1B	AX.89764735	4.30E+08	0.39	2.57	3.60	0.07
E1	GY	1B	AX.89535186	5.72E+08	0.11	-3.78	3.32	0.05
E1	GY	1B	AX.89751699	5.50E+08	0.26	-3.07	4.11	0.06
E1	GY	1B	AX.89751699	5.50E+08	0.26	-3.07	4.11	0.06
E1	GY	1B	AX.89359652	5.92E+08	0.27	-2.54	3.01	0.04
E1	GY	1B	AX.89703958	6.15E+08	0.20	-3.18	3.71	0.06
E1	GY	1B	AX.89463857	5.91E+08	0.25	-2.69	3.20	0.05
E1	GY	1B	AX.89359791	5.32E+08	0.22	-3.29	4.16	0.06

E1	GY	1B	AX.89765557	6.14E+08	0.20	-3.19	3.67	0.06
E1	GY	1B	AX.89679299	6.14E+08	0.20	-3.19	3.67	0.06
E1	GY	1B	AX.89536040	4.73E+08	0.14	-3.52	3.53	0.06
E1	GY	1B	AX.89500889	6.61E+08	0.15	-3.12	3.01	0.04
E1	GY	1B	AX.89403636	6.14E+08	0.20	-3.18	3.71	0.06
E1	GY	1B	AX.89403636	6.14E+08	0.20	-3.18	3.71	0.06
E1	GY	1B	AX.89490329	5.73E+08	0.08	-4.44	3.50	0.05
E1	GY	1B	AX.89765932	6.16E+08	0.20	-3.18	3.71	0.06
E1	GY	1B	AX.89765932	6.16E+08	0.20	-3.18	3.71	0.06
E1	GY	1B	AX.89725397	5.63E+08	0.48	-2.36	3.25	0.05
E1	GY	1B	AX.89372821	5.91E+08	0.10	-4.12	3.60	0.06
E1	GY	1B	AX.89536555	6.15E+08	0.20	-3.18	3.71	0.06
E1	GY	1B	AX.89315126	5.91E+08	0.25	-2.69	3.20	0.05
E1	GY	1B	AX.89655760	6.46E+08	0.07	-4.89	3.58	0.06
E1	GY	1B	AX.89571554	6.14E+08	0.20	-3.16	3.73	0.06
E1	GY	1B	AX.89571554	6.14E+08	0.20	-3.16	3.73	0.06
E1	GY	1B	AX.89454580	6.14E+08	0.20	-3.14	3.68	0.06
E1	GY	1B	AX.89373054	6.15E+08	0.20	-3.14	3.64	0.06
E1	GY	1B	AX.89361355	4.72E+08	0.14	-3.56	3.51	0.06
E1	GY	1B	AX.89361485	6.15E+08	0.20	-3.18	3.71	0.06
E1	GY	1B	AX.89537204	6.13E+08	0.17	-3.08	3.11	0.05
E1	GY	1B	AX.89627039	5.71E+08	0.15	-3.40	3.40	0.05
E1	GY	1B	AX.89511243	5.45E+08	0.33	-2.44	3.12	0.04
E1	GY	1B	AX.89373658	5.32E+08	0.24	-3.26	4.37	0.07
E1	GY	1B	AX.89714488	5.71E+08	0.08	-4.31	3.33	0.05
E1	GY	1B	AX.89644425	5.71E+08	0.09	-4.20	3.32	0.05
E1	GY	1B	AX.89584485	4.46E+07	0.47	2.36	3.24	0.05
E1	GY	1B	AX.89467073	5.32E+08	0.07	-5.27	4.29	0.07
E1	GY	1B	AX.89503677	6.29E+08	0.08	-4.40	3.45	0.05
E1	GY	1B	AX.89437602	5.91E+08	0.10	-4.12	3.60	0.06
E1	GY	1B	AX.89492307	5.75E+08	0.09	-4.33	3.64	0.06
E1	GY	1B	AX.89467807	5.46E+08	0.34	-2.58	3.47	0.05
E1	GY	1B	AX.89467807	5.46E+08	0.34	-2.58	3.47	0.05
E1	GY	1B	AX.89504198	6.14E+08	0.19	-2.97	3.21	0.05
E1	GY	1B	AX.89434410	5.90E+08	0.25	-2.74	3.27	0.05
E1	GY	1B	AX.89550273	5.94E+08	0.15	-3.69	4.00	0.06
E1	GY	1B	AX.89558501	5.73E+08	0.08	-4.80	3.83	0.06
E1	GY	1B	AX.89340424	5.62E+08	0.28	-2.69	3.42	0.05
E1	GY	1B	AX.89610620	5.93E+08	0.13	-3.93	3.98	0.06
E1	GY	1B	AX.89610620	5.93E+08	0.13	-3.93	3.98	0.06
E1	GY	1B	AX.89683293	5.71E+08	0.09	-4.20	3.32	0.05
E1	GY	1B	AX.89634659	6.15E+08	0.20	-3.18	3.71	0.06
E1	GY	1B	AX.89400218	5.49E+08	0.13	-3.58	3.29	0.05
E1	GY	1B	AX.89387161	5.48E+08	0.19	-3.13	3.50	0.05
E1	GY	1B	AX.89697293	5.47E+08	0.33	-2.50	3.24	0.05
E1	GY	1B	AX.89647056	5.43E+08	0.46	-2.72	4.15	0.08
E1	GY	1B	AX.89400603	5.48E+08	0.19	-3.13	3.50	0.05
E1	GY	1B	AX.89400618	5.96E+08	0.15	-3.72	3.98	0.06
E1	GY	1B	AX.89400618	5.96E+08	0.15	-3.72	3.98	0.06
E1	GY	1B	AX.89722018	5.90E+08	0.27	-2.80	3.54	0.06

E1	GY	1B	AX.89575021	5.72E+08	0.09	-4.20	3.32	0.05
E1	GY	1B	AX.89575021	5.72E+08	0.09	-4.20	3.32	0.05
E1	GY	1B	AX.89759919	5.90E+08	0.27	-2.69	3.30	0.06
E1	GY	1B	AX.89655838	5.90E+08	0.27	-2.69	3.30	0.06
E1	GY	1B	AX.89655838	5.90E+08	0.27	-2.69	3.30	0.06
E1	GY	1B	AX.89660011	6.13E+08	0.14	-3.73	3.91	0.06
E1	GY	1B	AX.89470072	5.71E+08	0.09	-4.20	3.32	0.05
E1	GY	1B	AX.89424158	5.29E+08	0.35	2.36	3.02	0.04
E1	GY	1B	AX.89770837	5.43E+08	0.44	-2.72	4.11	0.07
E1	GY	1B	AX.89694069	6.54E+08	0.14	-3.31	3.11	0.06
E1	GY	1B	AX.89601293	6.14E+08	0.20	-3.19	3.67	0.06
E1	GY	1B	AX.89698290	5.44E+08	0.45	-2.36	3.23	0.05
E1	GY	1B	AX.89589021	6.13E+08	0.14	-3.73	3.91	0.06
E1	GY	1B	AX.89552029	4.30E+08	0.38	2.47	3.37	0.06
E1	GY	1B	AX.89679010	6.16E+08	0.20	-3.18	3.71	0.06
E1	GY	1B	AX.89589368	5.71E+08	0.15	-3.40	3.40	0.05
E1	GY	1B	AX.89674301	5.62E+08	0.28	-2.83	3.73	0.06
E1	GY	1B	AX.89553218	5.62E+08	0.28	-2.67	3.38	0.05
E1	GY	1B	AX.89613020	6.15E+08	0.20	-3.18	3.71	0.06
E1	GY	1B	AX.89613020	6.15E+08	0.20	-3.18	3.71	0.06
E1	GY	1B	AX.89589597	6.14E+08	0.20	-3.18	3.71	0.06
E1	GY	1B	AX.89471968	5.71E+08	0.09	-4.20	3.32	0.05
E1	GY	1B	AX.89637173	5.66E+08	0.14	-3.56	3.61	0.06
E1	GY	1B	AX.89751131	5.71E+08	0.09	-4.20	3.32	0.05
E1	GY	1B	AX.89751131	5.71E+08	0.09	-4.20	3.32	0.05
E1	GY	1B	AX.89472257	5.32E+08	0.20	-3.17	3.68	0.06
E1	GY	1B	AX.89472257	5.32E+08	0.20	-3.17	3.68	0.06
E1	GY	1B	AX.89621545	5.71E+08	0.09	-4.20	3.32	0.05
E1	GY	1B	AX.89621545	5.71E+08	0.09	-4.20	3.32	0.05
E1	GY	1B	AX.89367182	5.95E+08	0.17	-3.45	3.80	0.06
E1	GY	1B	AX.89367182	5.95E+08	0.17	-3.45	3.80	0.06
E1	GY	1B	AX.89367256	5.91E+08	0.27	-2.80	3.54	0.06
E1	GY	1B	AX.89712134	5.92E+08	0.26	-2.71	3.27	0.05
E1	GY	1B	AX.89712141	4.30E+08	0.38	2.49	3.40	0.06
E1	GY	1B	AX.89590842	6.15E+08	0.20	-3.18	3.71	0.06
E1	GY	1B	AX.89486405	5.94E+08	0.15	-3.72	3.98	0.06
E1	GY	1B	AX.89543383	5.94E+08	0.15	-3.47	3.52	0.05
E1	GY	1B	AX.89509761	5.43E+08	0.41	-2.57	3.68	0.06
E1	GY	1B	AX.89770151	6.15E+08	0.20	-3.18	3.71	0.06
E1	GY	1B	AX.89770151	6.15E+08	0.20	-3.18	3.71	0.06
E1	GY	1B	AX.89333721	6.13E+08	0.14	-3.73	3.91	0.06
E1	GY	1B	AX.89510480	6.14E+08	0.20	-3.16	3.67	0.06
E1	GY	1B	AX.89578881	5.45E+08	0.33	-2.57	3.42	0.05
E1	GY	1B	AX.89380858	5.94E+08	0.15	-3.69	4.00	0.06
E1	GY	1B	AX.89771477	6.15E+08	0.20	-3.18	3.71	0.06
E1	GY	1B	AX.89405354	5.28E+08	0.35	2.36	3.02	0.04
E1	GY	1B	AX.89742936	5.89E+08	0.27	-2.70	3.35	0.06
E1	GY	1B	AX.89632144	5.90E+08	0.25	-2.74	3.27	0.05
E1	GY	1B	AX.89605104	5.76E+08	0.09	-4.33	3.64	0.06
E1	GY	1B	AX.89616764	6.14E+08	0.20	-3.19	3.67	0.06

E1	SPM2	1B	AX.89457469	6.41E+08	0.05	-39.29	3.13	0.06
E1	GY	1B	AX.89727207	5.94E+08	0.15	-3.69	4.00	0.06
E1	GY	1B	AX.89561467	5.48E+08	0.20	-3.03	3.36	0.05
E1	GY	1B	AX.89546235	6.14E+08	0.20	-3.18	3.71	0.06
E1	GY	1B	AX.89546341	5.94E+08	0.15	-3.69	4.00	0.06
E1	GY	1B	AX.89760457	5.90E+08	0.25	-2.69	3.20	0.05
E1	GY	1B	AX.89617894	6.14E+08	0.20	-3.19	3.67	0.06
E1	GY	1B	AX.89359652	5.92E+08	0.27	-2.54	3.01	0.04
E1	GY	1B	AX.89581957	5.95E+08	0.15	-3.72	3.98	0.06
E1	GY	1B	AX.89637878	5.89E+08	0.27	-2.70	3.35	0.06
E1	GY	1B	AX.89431323	5.91E+08	0.24	-2.66	3.07	0.04
E1	GY	1B	AX.89431323	5.91E+08	0.24	-2.66	3.07	0.04
E1	GY	1B	AX.89765882	6.46E+08	0.07	-4.89	3.58	0.06
E1	GY	1B	AX.89477534	5.62E+08	0.28	-2.83	3.73	0.06
E1	GY	1B	AX.89513960	6.13E+08	0.16	-3.17	3.22	0.05
E1	GY	1B	AX.89510021	5.73E+08	0.08	-4.44	3.50	0.05
E1	GY	1B	AX.89408237	6.14E+08	0.20	-3.19	3.67	0.06
E1	GY	1B	AX.89465482	5.43E+08	0.41	-2.68	3.95	0.07
E1	GY	1B	AX.89536494	5.47E+08	0.19	-3.13	3.50	0.05
E1	GY	1B	AX.89419135	4.29E+08	0.38	2.49	3.40	0.06
E1	GY	1B	AX.89583603	6.13E+08	0.14	-3.73	3.91	0.06
E1	GY	1B	AX.89583789	5.71E+08	0.09	-4.20	3.32	0.05
E1	GY	1B	AX.89515290	5.32E+08	0.20	-3.17	3.68	0.06
E1	GY	1B	AX.89515290	5.32E+08	0.20	-3.17	3.68	0.06
E1	GY	1B	AX.89702421	5.91E+08	0.25	-2.69	3.20	0.05
E1	GY	1B	AX.89714925	5.71E+08	0.09	-4.20	3.32	0.05
E1	GY	1B	AX.89537771	5.48E+08	0.19	-3.13	3.50	0.05
E1	GY	1B	AX.89338664	5.62E+08	0.28	-2.82	3.69	0.05
E1	GY	1B	AX.89516076	5.50E+08	0.26	-3.07	4.11	0.06
E1	GY	1B	AX.89428640	5.91E+08	0.26	-2.71	3.27	0.05
E1	GY	1B	AX.89428640	5.91E+08	0.26	-2.71	3.27	0.05
E1	GY	1B	AX.89437602	5.91E+08	0.10	-4.12	3.60	0.06
E1	GY	1B	AX.89375029	5.89E+08	0.27	-2.70	3.35	0.06
E1	GY	1B	AX.89680841	5.43E+08	0.25	-3.71	5.55	0.09
E1	GY	1B	AX.89434131	5.73E+08	0.08	-4.44	3.50	0.05
E1	GY	1B	AX.89339603	6.13E+08	0.14	-3.73	3.91	0.06
E1	GY	1B	AX.89434410	5.90E+08	0.25	-2.74	3.27	0.05
E1	GY	1B	AX.86161856	5.72E+08	0.11	-3.85	3.31	0.05
E1	GY	1B	AX.89573521	6.14E+08	0.20	-3.18	3.71	0.06
E1	GY	1B	AX.89573521	6.14E+08	0.20	-3.18	3.71	0.06
E1	GY	1B	AX.89535375	5.49E+08	0.13	-3.58	3.29	0.05
E1	GY	1B	AX.89340249	6.14E+08	0.20	-3.19	3.67	0.06
E1	GY	1B	AX.89386642	5.75E+08	0.09	-4.33	3.64	0.06
E1	GY	1B	AX.89622088	5.91E+08	0.25	-2.69	3.20	0.05
E1	GY	1B	AX.89329223	4.30E+08	0.39	2.57	3.60	0.07
E1	GY	1B	AX.89329223	4.30E+08	0.39	2.57	3.60	0.07
E1	GY	1B	AX.89376184	5.89E+08	0.27	-2.70	3.35	0.06
E1	GY	1B	AX.89490318	6.14E+08	0.20	-3.26	3.83	0.06
E1	GY	1B	AX.89721858	5.89E+08	0.13	-4.04	4.06	0.07
E1	GY	1B	AX.89721912	5.90E+08	0.10	-4.12	3.60	0.06

E1	GY	1B	AX.89721912	5.90E+08	0.10	-4.12	3.60	0.06
E1	GY	1B	AX.89655645	5.44E+08	0.42	-2.51	3.55	0.06
E1	GY	1B	AX.89655645	5.44E+08	0.42	-2.51	3.55	0.06
E1	GY	1B	AX.89705262	6.14E+08	0.20	-3.18	3.71	0.06
E1	GY	1B	AX.89540478	5.74E+08	0.09	-4.33	3.64	0.06
E1	GY	1B	AX.89587635	6.16E+08	0.20	-3.18	3.71	0.06
E1	GY	1B	AX.86166370	6.15E+08	0.20	-3.18	3.71	0.06
E1	GY	1B	AX.89659895	6.14E+08	0.20	-3.19	3.67	0.06
E1	GY	1B	AX.89753324	5.49E+08	0.49	2.42	3.41	0.05
E1	GY	1B	AX.89714920	5.43E+08	0.41	-2.57	3.68	0.06
E1	GY	1B	AX.89714920	5.43E+08	0.41	-2.57	3.68	0.06
E1	GY	1B	AX.89401886	5.95E+08	0.14	-3.50	3.50	0.05
E1	GY	1B	AX.89589021	6.13E+08	0.14	-3.73	3.91	0.06
E1	GY	1B	AX.89519215	5.64E+08	0.12	-3.79	3.53	0.05
E1	GY	1B	AX.89519215	5.64E+08	0.12	-3.79	3.53	0.05
E1	GY	1B	AX.89679010	6.16E+08	0.20	-3.18	3.71	0.06
E1	GY	1B	AX.89756912	4.74E+08	0.14	-3.56	3.51	0.06
E1	GY	1B	AX.89756912	4.74E+08	0.14	-3.56	3.51	0.06
E1	GY	1B	AX.89636906	6.15E+08	0.20	-3.18	3.71	0.06
E1	GY	1B	AX.89636906	6.15E+08	0.20	-3.18	3.71	0.06
E1	GY	1B	AX.89471727	6.16E+08	0.20	-3.18	3.71	0.06
E1	GY	1B	AX.89541312	5.50E+08	0.26	-3.07	4.11	0.06
E1	GY	1B	AX.89541312	5.50E+08	0.26	-3.07	4.11	0.06
E1	GY	1B	AX.89471968	5.71E+08	0.09	-4.20	3.32	0.05
E1	GY	1B	AX.89613526	5.72E+08	0.09	-4.20	3.32	0.05
E1	GY	1B	AX.89577198	5.89E+08	0.27	-2.70	3.35	0.06
E1	GY	1B	AX.89528229	6.15E+08	0.20	-3.18	3.71	0.06
E1	GY	1B	AX.89699324	5.64E+08	0.11	-3.58	3.03	0.05
E1	GY	1B	AX.89624962	6.15E+08	0.20	-3.19	3.67	0.06
E1	GY	1B	AX.89624962	6.15E+08	0.20	-3.19	3.67	0.06
E1	GY	1B	AX.89585539	6.14E+08	0.20	-3.18	3.71	0.06
E1	GY	1B	AX.89414699	5.95E+08	0.15	-3.69	4.00	0.06
E1	GY	1B	AX.89485879	6.15E+08	0.20	-3.18	3.71	0.06
E1	GY	1B	AX.89755321	6.14E+08	0.20	-3.19	3.67	0.06
E1	GY	1B	AX.89675132	5.71E+08	0.09	-4.20	3.32	0.05
E1	GY	1B	AX.89504471	5.95E+08	0.15	-3.69	4.00	0.06
E1	GY	1B	AX.89411589	5.66E+08	0.16	-3.04	3.00	0.04
E1	GY	1B	AX.89310641	5.48E+08	0.19	-3.13	3.50	0.05
E1	GY	1B	AX.89436079	6.15E+08	0.17	-3.09	3.14	0.05
E1	GY	1B	AX.89774250	6.15E+08	0.20	-3.18	3.71	0.06
E1	GY	1B	AX.89774250	6.15E+08	0.20	-3.18	3.71	0.06
E1	GY	1B	AX.89344807	6.16E+08	0.20	-3.18	3.71	0.06
E1	GY	1B	AX.89713215	5.48E+08	0.19	-3.13	3.50	0.05
E1	GY	1B	AX.89415762	5.91E+08	0.26	-2.71	3.27	0.05
E1	GY	1B	AX.89591657	5.72E+08	0.09	-4.20	3.32	0.05
E1	GY	1B	AX.89595965	5.49E+08	0.26	-3.07	4.11	0.06
E1	GY	1B	AX.89595965	5.49E+08	0.26	-3.07	4.11	0.06
E1	GY	1B	AX.89672607	5.93E+08	0.30	-2.66	3.46	0.06
E1	GY	1B	AX.89635603	5.94E+08	0.15	-3.47	3.52	0.05
E1	GY	1B	AX.89380688	5.90E+08	0.27	-2.70	3.35	0.06

E1	GY	1B	AX.89345478	5.89E+08	0.13	-4.04	4.06	0.07
E1	GY	1B	AX.89345478	5.89E+08	0.13	-4.04	4.06	0.07
E1	GY	1B	AX.89544768	5.95E+08	0.15	-3.47	3.52	0.05
E1	GY	1B	AX.89380977	1.78E+07	0.22	-2.82	3.20	0.05
E1	GY	1B	AX.89596577	5.95E+08	0.15	-3.72	3.98	0.06
E1	GY	1B	AX.89346068	6.14E+08	0.20	-3.19	3.67	0.06
E1	GY	1B	AX.89665857	5.74E+08	0.09	-4.33	3.64	0.06
E1	GY	1B	AX.89665857	5.74E+08	0.09	-4.33	3.64	0.06
E1	GY	1B	AX.89653613	5.44E+08	0.44	-2.45	3.44	0.06
E1	GY	1B	AX.89417724	5.94E+08	0.15	-3.47	3.52	0.05
E1	GY	1B	AX.89524034	6.46E+08	0.07	-4.89	3.58	0.06
E1	GY	1B	AX.89557668	5.75E+08	0.09	-4.33	3.64	0.06
E1	GY	1B	AX.89546235	6.14E+08	0.20	-3.18	3.71	0.06
E1	GY	1B	AX.89686858	6.13E+08	0.14	-3.73	3.91	0.06
E1	GY	1B	AX.89500077	4.73E+08	0.14	-3.52	3.53	0.06
E1	GY	1B	AX.89524464	5.89E+08	0.27	-2.70	3.35	0.06
E1	GY	1B	AX.89728530	5.96E+08	0.15	-3.72	3.98	0.06
E1	GY	1B	AX.89728530	5.96E+08	0.15	-3.72	3.98	0.06
E1	GY	1B	AX.89692308	5.95E+08	0.15	-3.72	3.98	0.06
E1	GY	1B	AX.89692308	5.95E+08	0.15	-3.72	3.98	0.06
E1	GY	1B	AX.89372056	5.96E+08	0.15	-3.72	3.98	0.06
E1	GY	1B	AX.89525338	5.73E+08	0.08	-4.58	3.53	0.06
E1	GY	1B	AX.89314421	5.92E+08	0.26	-2.71	3.27	0.05
E1	GY	1B	AX.89391459	4.82E+08	0.13	3.35	3.02	0.04
E1	GY	1B	AX.89454201	6.15E+08	0.20	-3.18	3.71	0.06
E1	GY	1B	AX.89326749	5.71E+08	0.09	-4.20	3.32	0.05
E1	GY	1B	AX.89578393	5.71E+08	0.09	-4.20	3.32	0.05
E1	GY	1B	AX.89693026	5.66E+08	0.14	-3.56	3.61	0.06
E1	GY	1B	AX.89693026	5.66E+08	0.14	-3.56	3.61	0.06
E1	GY	1B	AX.89372821	5.91E+08	0.10	-4.12	3.60	0.06
E1	GY	1B	AX.89465484	5.50E+08	0.26	-3.07	4.11	0.06
E1	GY	1B	AX.89751221	5.32E+08	0.20	-3.18	3.65	0.06
E1	GY	1B	AX.89384148	5.76E+08	0.09	-4.33	3.64	0.06
E1	GY	1B	AX.89749378	6.32E+08	0.08	-4.54	3.63	0.06
E1	GY	1B	AX.89454719	5.91E+08	0.10	-4.12	3.60	0.06
E1	GY	1B	AX.89564257	6.14E+08	0.20	-3.19	3.67	0.06
E1	GY	1B	AX.89564257	6.14E+08	0.20	-3.19	3.67	0.06
E1	GY	1B	AX.89327316	5.95E+08	0.15	-3.72	3.98	0.06
E1	GY	1B	AX.89315865	5.71E+08	0.09	-4.20	3.32	0.05
E1	GY	1B	AX.89526794	5.90E+08	0.27	-2.70	3.35	0.06
E1	GY	1B	AX.89765713	6.21E+08	0.19	-3.20	3.64	0.06
E1	GY	1B	AX.89349463	5.48E+08	0.19	-3.13	3.50	0.05
E1	GY	1B	AX.89586686	5.48E+08	0.19	-3.13	3.50	0.05
E1	GY	1B	AX.89454941	4.30E+08	0.39	2.57	3.60	0.07
E1	GY	1B	AX.89511243	5.45E+08	0.33	-2.44	3.12	0.04
E1	GY	1B	AX.89462493	5.95E+08	0.15	-3.47	3.52	0.05
E1	GY	1B	AX.89640474	5.89E+08	0.13	-4.04	4.06	0.07
E1	GY	1B	AX.89640474	5.89E+08	0.13	-4.04	4.06	0.07
E1	GY	1B	AX.89317078	6.14E+08	0.20	-3.19	3.67	0.06
E1	GY	1B	AX.89421570	6.14E+08	0.16	-3.17	3.22	0.05

E1	GY	1B	AX.89649127	5.73E+08	0.08	-4.42	3.32	0.05
E1	GY	1B	AX.89649127	5.73E+08	0.08	-4.42	3.32	0.05
E1	GY	1B	AX.89528405	5.89E+08	0.12	-4.11	4.06	0.08
E1	GY	1B	AX.89769126	4.72E+08	0.14	-3.52	3.53	0.06
E1	GY	1B	AX.89769126	4.72E+08	0.14	-3.52	3.53	0.06
E1	GY	1B	AX.89670540	5.71E+08	0.09	-4.20	3.32	0.05
E1	GY	1B	AX.89512987	5.71E+08	0.09	-4.20	3.32	0.05
E1	GY	1B	AX.89598726	5.72E+08	0.09	-4.20	3.32	0.05
E1	GY	1B	AX.89598726	5.72E+08	0.09	-4.20	3.32	0.05
E1	GY	1B	AX.89691473	5.96E+08	0.15	-3.72	3.98	0.06
E1	GY	1B	AX.89535375	5.49E+08	0.13	-3.58	3.29	0.05
E1	GY	1B	AX.89573650	6.15E+08	0.20	-3.18	3.71	0.06
E1	GY	1B	AX.89386642	5.75E+08	0.09	-4.33	3.64	0.06
E1	GY	1B	AX.89568032	6.15E+08	0.20	-3.18	3.71	0.06
E1	GY	1B	AX.89625060	5.50E+08	0.13	-3.47	3.13	0.05
E1	GY	1B	AX.89563144	5.72E+08	0.11	-3.78	3.32	0.05
E1	GY	1B	AX.89386960	5.73E+08	0.08	-4.44	3.50	0.05
E1	GY	1B	AX.89376184	5.89E+08	0.27	-2.70	3.35	0.06
E1	GY	1B	AX.89412388	5.48E+08	0.19	-3.13	3.50	0.05
E1	GY	1B	AX.89769760	6.15E+08	0.20	-3.18	3.71	0.06
E1	GY	1B	AX.89400603	5.48E+08	0.19	-3.13	3.50	0.05
E1	GY	1B	AX.89729451	6.14E+08	0.20	-3.19	3.67	0.06
E1	GY	1B	AX.89729451	6.14E+08	0.20	-3.19	3.67	0.06
E1	GY	1B	AX.89468178	5.89E+08	0.13	-4.04	4.06	0.07
E1	GY	1B	AX.89705262	6.14E+08	0.20	-3.18	3.71	0.06
E1	GY	1B	AX.89659701	5.63E+08	0.12	-3.53	3.04	0.05
E1	GY	1B	AX.89530034	5.73E+08	0.09	-4.93	4.38	0.07
E1	GY	1B	AX.89530034	5.73E+08	0.09	-4.93	4.38	0.07
E1	GY	1B	AX.89353327	6.15E+08	0.20	-3.18	3.71	0.06
E1	GY	1B	AX.89600634	5.93E+08	0.13	-3.89	4.01	0.06
E1	GY	1B	AX.89685826	5.73E+08	0.10	-3.96	3.24	0.05
E1	GY	1B	AX.89552444	5.43E+08	0.27	-3.68	5.78	0.09
E1	GY	1B	AX.89771478	6.16E+08	0.20	-3.18	3.71	0.06
E1	GY	1B	AX.89320059	5.95E+08	0.15	-3.72	3.98	0.06
E1	GY	1B	AX.89698290	5.44E+08	0.45	-2.36	3.23	0.05
E1	GY	1B	AX.89722505	5.71E+08	0.09	-4.20	3.32	0.05
E1	GY	1B	AX.89711094	6.14E+08	0.18	-3.29	3.68	0.06
E1	GY	1B	AX.89711094	6.14E+08	0.18	-3.29	3.68	0.06
E1	GY	1B	AX.89608342	5.89E+08	0.27	-2.70	3.35	0.06
E1	GY	1B	AX.89608342	5.89E+08	0.27	-2.70	3.35	0.06
E1	GY	1B	AX.89531457	6.14E+08	0.20	-3.18	3.71	0.06
E1	GY	1B	AX.89674080	6.54E+08	0.23	-2.70	3.04	0.05
E1	GY	1B	AX.89736403	5.43E+08	0.41	-2.68	3.95	0.07
E1	GY	1B	AX.89736403	5.43E+08	0.41	-2.68	3.95	0.07
E1	GY	1B	AX.89736664	5.90E+08	0.10	-4.12	3.60	0.06
E1	GY	1B	AX.89724852	5.94E+08	0.15	-3.69	4.00	0.06
E1	GY	1B	AX.89532430	4.73E+08	0.14	-3.28	3.06	0.05
E1	GY	1B	AX.89713356	5.72E+08	0.09	-4.20	3.32	0.05
E1	GY	1B	AX.89713356	5.72E+08	0.09	-4.20	3.32	0.05
E1	GY	1B	AX.89713584	6.15E+08	0.20	-3.18	3.71	0.06

E1	GY	1B	AX.89775428	5.71E+08	0.09	-4.17	3.28	0.05
E1	GY	1B	AX.89533661	6.13E+08	0.17	-3.08	3.11	0.05
E1	GY	1B	AX.89533661	6.13E+08	0.17	-3.08	3.11	0.05
E1	GY	1B	AX.89380977	1.78E+07	0.22	-2.82	3.20	0.05
E1	GY	1B	AX.89710848	5.95E+08	0.14	-3.50	3.50	0.05
E1	GY	1B	AX.89657160	5.71E+08	0.09	-4.20	3.32	0.05
E1	GY	1B	AX.89499048	5.43E+08	0.32	-3.09	4.60	0.08
E1	GY	1B	AX.89499048	5.43E+08	0.32	-3.09	4.60	0.08
E1	GY	1B	AX.89524116	5.43E+08	0.42	-2.81	4.32	0.08
E1	GY	1B	AX.89524116	5.43E+08	0.42	-2.81	4.32	0.08
E1	GY	1B	AX.89382181	5.73E+08	0.11	-4.47	4.28	0.07
E1	GY	1B	AX.89594265	5.76E+08	0.09	-4.33	3.64	0.06
E1	GY	1B	AX.89764735	4.30E+08	0.39	2.57	3.60	0.07
E1	GY	1B	AX.89637605	5.73E+08	0.09	-4.93	4.38	0.07
E1	GY	1B	AX.89371586	5.95E+08	0.15	-3.47	3.52	0.05
E1	GY	1B	AX.89642296	5.72E+08	0.11	-3.78	3.32	0.05
E1	GY	1B	AX.89756848	6.14E+08	0.20	-3.19	3.67	0.06
E1	GY	1B	AX.89765557	6.14E+08	0.20	-3.19	3.67	0.06
E1	GY	1B	AX.89500889	6.61E+08	0.15	-3.12	3.01	0.04
E1	GY	1B	AX.89536084	5.46E+08	0.33	-2.57	3.42	0.05
E1	GY	1B	AX.89490329	5.73E+08	0.08	-4.44	3.50	0.05
E1	GY	1B	AX.89326749	5.71E+08	0.09	-4.20	3.32	0.05
E1	GY	1B	AX.89465165	5.49E+08	0.27	-3.07	4.15	0.06
E1	GY	1B	AX.89505604	5.92E+08	0.26	-2.71	3.27	0.05
E1	GY	1B	AX.89655760	6.46E+08	0.07	-4.89	3.58	0.06
E1	GY	1B	AX.89647569	5.91E+08	0.26	-2.71	3.27	0.05
E1	GY	1B	AX.89647569	5.91E+08	0.26	-2.71	3.27	0.05
E1	GY	1B	AX.89361475	6.14E+08	0.20	-3.18	3.71	0.06
E1	GY	1B	AX.89361475	6.14E+08	0.20	-3.18	3.71	0.06
E1	GY	1B	AX.89361588	5.48E+08	0.19	-3.13	3.50	0.05
E1	GY	1B	AX.89680637	5.89E+08	0.27	-2.70	3.35	0.06
E1	GY	1B	AX.89680637	5.89E+08	0.27	-2.70	3.35	0.06
E1	GY	1B	AX.89572193	5.72E+08	0.09	-4.20	3.32	0.05
E1	GY	1B	AX.89714488	5.71E+08	0.08	-4.31	3.33	0.05
E1	GY	1B	AX.89397557	5.94E+08	0.15	-3.69	4.00	0.06
E1	GY	1B	AX.89754580	5.49E+08	0.19	-3.14	3.46	0.05
E1	GY	1B	AX.89754580	5.49E+08	0.19	-3.14	3.46	0.05
E1	GY	1B	AX.89503677	6.29E+08	0.08	-4.40	3.45	0.05
E1	GY	1B	AX.89658398	6.16E+08	0.20	-3.18	3.71	0.06
E1	GY	1B	AX.89658398	6.16E+08	0.20	-3.18	3.71	0.06
E1	GY	1B	AX.89399318	5.95E+08	0.15	-3.72	3.98	0.06
E1	GY	1B	AX.89610191	5.43E+08	0.44	-2.72	4.11	0.07
E1	GY	1B	AX.89610191	5.43E+08	0.44	-2.72	4.11	0.07
E1	GY	1B	AX.89400218	5.49E+08	0.13	-3.58	3.29	0.05
E1	GY	1B	AX.89540478	5.74E+08	0.09	-4.33	3.64	0.06
E1	GPM2	1B	AX.89746393	6.62E+08	0.08	-1141.05	3.35	0.05
E1	GY	1B	AX.89668344	5.71E+08	0.09	-4.20	3.32	0.05
E1	GY	1B	AX.89668344	5.71E+08	0.09	-4.20	3.32	0.05
E1	GPM2	1B	AX.89519011	6.53E+08	0.12	-978.60	3.56	0.06
E1	GY	1B	AX.89660011	6.13E+08	0.14	-3.73	3.91	0.06

E1	GPM2	1B	AX.89446477	5.71E+07	0.34	-642.48	3.30	0.05
E1	GY	1B	AX.89611944	5.43E+08	0.46	-2.86	4.52	0.09
E1	GY	1B	AX.89611944	5.43E+08	0.46	-2.86	4.52	0.09
E1	GY	1B	AX.89694069	6.54E+08	0.14	-3.31	3.11	0.06
E1	GY	1B	AX.89601293	6.14E+08	0.20	-3.19	3.67	0.06
E1	GY	1B	AX.89734677	5.48E+08	0.19	-3.13	3.50	0.05
E1	GPM2	1B	AX.89579570	6.55E+08	0.08	-1073.58	3.02	0.05
E1	GY	1B	AX.89552029	4.30E+08	0.38	2.47	3.37	0.06
E1	GY	1B	AX.89402330	5.93E+08	0.13	-3.89	4.01	0.06
E1	GPM2	1B	AX.89459113	5.63E+08	0.48	-616.75	3.36	0.05
E1	GPM2	1B	AX.89378009	4.84E+08	0.19	807.71	3.47	0.05
E1	GPM2	1B	AX.89586724	6.55E+08	0.08	-1073.58	3.02	0.05
E1	GPM2	1B	AX.89668519	6.52E+08	0.12	-978.60	3.56	0.06
E1	GPM2	1B	AX.89654461	6.54E+08	0.11	-1028.45	3.65	0.06
E1	GPM2	1B	AX.89569221	4.96E+08	0.17	-793.84	3.20	0.06
E1	GY	1B	AX.89735790	5.71E+08	0.09	-4.20	3.32	0.05
E1	GY	1B	AX.89735790	5.71E+08	0.09	-4.20	3.32	0.05
E1	GY	1B	AX.89602399	5.72E+08	0.09	-4.20	3.32	0.05
E1	GY	1B	AX.89674080	6.54E+08	0.23	-2.70	3.04	0.05
E1	GY	1B	AX.89367256	5.91E+08	0.27	-2.80	3.54	0.06
E1	GPM2	1B	AX.89384638	6.52E+08	0.12	-979.71	3.47	0.06
E1	GY	1B	AX.89566210	5.62E+08	0.29	-2.70	3.47	0.05
E1	GY	1B	AX.89411589	5.66E+08	0.16	-3.04	3.00	0.04
E1	GPM2	1B	AX.89546727	6.62E+08	0.08	-1141.05	3.35	0.05
E1	GY	1B	AX.89646690	5.96E+08	0.15	-3.72	3.98	0.06
E1	GY	1B	AX.89646690	5.96E+08	0.15	-3.72	3.98	0.06
E1	GY	1B	AX.89333321	5.50E+08	0.26	-3.07	4.11	0.06
E1	GY	1B	AX.89713215	5.48E+08	0.19	-3.13	3.50	0.05
E1	GPM2	1B	AX.89341022	4.84E+08	0.19	807.71	3.47	0.05
E1	GY	1B	AX.89428761	5.44E+08	0.45	-2.36	3.23	0.05
E1	GPM2	1B	AX.89772593	5.33E+07	0.29	-646.83	3.08	0.05
E1	GPM2	1B	AX.89321187	6.61E+08	0.09	-1080.74	3.46	0.06
E1	GY	1B	AX.89771477	6.15E+08	0.20	-3.18	3.71	0.06
E1	GY	1B	AX.89357479	5.43E+08	0.45	-2.57	3.73	0.06
E1	GY	1B	AX.89632144	5.90E+08	0.25	-2.74	3.27	0.05
E1	GY	1B	AX.89726996	6.15E+08	0.20	-3.18	3.71	0.06
E1	GY	1B	AX.89726996	6.15E+08	0.20	-3.18	3.71	0.06
E1	GY	1B	AX.89605104	5.76E+08	0.09	-4.33	3.64	0.06
E1	GPM2	1B	AX.89774524	4.85E+08	0.19	807.71	3.47	0.05
E1	GY	1B	AX.89690662	5.94E+08	0.15	-3.47	3.52	0.05
E1	GPM2	1B	AX.89756912	4.74E+08	0.14	-942.17	3.70	0.07
E1	GY	1B	AX.89580989	6.46E+08	0.07	-4.89	3.58	0.06
E1	GY	1B	AX.89430295	6.54E+08	0.13	-3.54	3.23	0.06
E1	GY	1B	AX.89546341	5.94E+08	0.15	-3.69	4.00	0.06
E1	GY	1B	AX.89441819	5.89E+08	0.27	-2.70	3.35	0.06
E1	GY	1B	AX.89503576	5.71E+08	0.09	-4.02	3.20	0.05
E1	GY	1B	AX.89728608	5.43E+08	0.41	-2.57	3.68	0.06
E1	GPM2	1B	AX.89430464	5.49E+07	0.16	-849.20	3.39	0.05
E1	GY	1B	AX.89618457	5.43E+08	0.40	-2.46	3.39	0.06
E1	GY	1B	AX.89618457	5.43E+08	0.40	-2.46	3.39	0.06

E1	GY	1B	AX.89407855	5.43E+08	0.44	-2.82	4.39	0.08
E1	GY	1B	AX.89407855	5.43E+08	0.44	-2.82	4.39	0.08
E1	GPM2	1B	AX.89598991	6.61E+08	0.15	-820.92	3.13	0.05
E1	GPM2	1B	AX.89699158	4.85E+08	0.19	807.71	3.47	0.05
E1	GY	1B	AX.89679490	5.73E+08	0.08	-4.44	3.50	0.05
E1	GPM2	1B	AX.89372089	6.62E+08	0.08	-1141.05	3.35	0.05
E1	GY	1B	AX.89645388	5.90E+08	0.25	-2.74	3.27	0.05
E1	GPM2	1B	AX.89655654	6.62E+08	0.08	-1141.05	3.35	0.05
E1	GY	1B	AX.89756650	6.14E+08	0.20	-3.18	3.71	0.06
E1	GY	1B	AX.89465484	5.50E+08	0.26	-3.07	4.11	0.06
E1	GPM2	1B	AX.89422777	6.55E+08	0.08	-1073.58	3.02	0.05
E1	GPM2	1B	AX.89316355	6.52E+08	0.12	-978.60	3.56	0.06
E1	GY	1B	AX.89443701	6.15E+08	0.20	-3.18	3.71	0.06
E1	GY	1B	AX.89749378	6.32E+08	0.08	-4.54	3.63	0.06
E1	GPM2	1B	AX.89332715	6.54E+08	0.12	-978.60	3.56	0.06
E1	GY	1B	AX.89680746	6.14E+08	0.20	-3.18	3.71	0.06
E1	GY	1B	AX.89680746	6.14E+08	0.20	-3.18	3.71	0.06
E1	GPM2	1B	AX.89465453	5.48E+07	0.16	-849.20	3.39	0.05
E1	GPM2	1B	AX.89375527	6.61E+08	0.09	-1080.74	3.46	0.06
E1	GY	1B	AX.89537771	5.48E+08	0.19	-3.13	3.50	0.05
E1	GPM2	1B	AX.89313994	5.63E+07	0.28	-673.00	3.27	0.05
E1	GY	1B	AX.89479897	5.96E+08	0.15	-3.72	3.98	0.06
E1	GPM2	1B	AX.89344904	6.59E+08	0.09	-1080.74	3.46	0.06
E1	GY	1B	AX.89516076	5.50E+08	0.26	-3.07	4.11	0.06
E1	GY	1B	AX.89375029	5.89E+08	0.27	-2.70	3.35	0.06
E1	GY	1B	AX.89680841	5.43E+08	0.25	-3.71	5.55	0.09
E1	GY	1B	AX.89606726	4.45E+07	0.44	-2.44	3.42	0.06
E1	GY	1B	AX.89606726	4.45E+07	0.44	-2.44	3.42	0.06
E1	GPM2	1B	AX.89393822	5.63E+07	0.29	-635.47	3.01	0.05
E1	GY	1B	AX.89364192	6.14E+08	0.20	-3.18	3.71	0.06
E1	GY	1B	AX.89364192	6.14E+08	0.20	-3.18	3.71	0.06
E1	GY	1B	AX.89421818	4.72E+08	0.14	-3.56	3.51	0.06
E1	GY	1B	AX.89622088	5.91E+08	0.25	-2.69	3.20	0.05
E1	GY	1B	AX.89490318	6.14E+08	0.20	-3.26	3.83	0.06
E1	GPM2	1B	AX.89650893	5.46E+07	0.15	-948.76	3.93	0.06
E1	GY	1B	AX.89341296	6.54E+08	0.14	-3.31	3.11	0.06
E1	GPM2	1B	AX.89644951	1.42E+07	0.20	-799.36	3.63	0.06
E1	GY	1B	AX.86166370	6.15E+08	0.20	-3.18	3.71	0.06
E1	GY	1B	AX.89659895	6.14E+08	0.20	-3.19	3.67	0.06
E1	GPM2	1B	AX.89536942	6.55E+08	0.08	-1073.58	3.02	0.05
E1	GPM2	1B	AX.89626512	4.60E+08	0.40	601.27	3.12	0.05
E1	GPM2	1B	AX.89466921	5.58E+07	0.16	-849.20	3.39	0.05
E1	GPM2	1B	AX.89429501	6.54E+08	0.12	-978.60	3.56	0.06
E1	GPM2	1B	AX.89666109	6.62E+08	0.08	-1193.59	3.79	0.06
E1	GPM2	1B	AX.89381763	5.70E+07	0.27	-654.24	3.06	0.05
E1	GPM2	1B	AX.89663520	6.54E+08	0.11	-1028.45	3.65	0.06
E1	GPM2	1B	AX.89500077	4.73E+08	0.14	-837.68	3.10	0.05
E1	GPM2	1B	AX.89666869	6.52E+08	0.12	-978.60	3.56	0.06
E1	GPM2	1B	AX.89460476	6.53E+08	0.11	-1028.45	3.65	0.06
E1	GPM2	1B	AX.89500359	6.53E+08	0.11	-1028.45	3.65	0.06

E1	GY	1B	AX.89619733	6.15E+08	0.20	-3.18	3.71	0.06
E1	GY	1B	AX.89619733	6.15E+08	0.20	-3.18	3.71	0.06
E1	GY	1B	AX.89577198	5.89E+08	0.27	-2.70	3.35	0.06
E1	GY	1B	AX.89585539	6.14E+08	0.20	-3.18	3.71	0.06
E1	GY	1B	AX.89590119	5.63E+08	0.12	-3.53	3.04	0.05
E1	GPM2	1B	AX.89384887	4.87E+08	0.19	807.71	3.47	0.05
E1	GPM2	1B	AX.89424518	4.84E+08	0.19	807.71	3.47	0.05
E1	GY	1B	AX.89755321	6.14E+08	0.20	-3.19	3.67	0.06
E1	GPM2	1B	AX.89316675	6.53E+08	0.12	-978.60	3.56	0.06
E1	GPM2	1B	AX.89356982	6.54E+08	0.13	-1079.82	4.35	0.08
E1	GY	1B	AX.89675132	5.71E+08	0.09	-4.20	3.32	0.05
E1	GY	1B	AX.89580727	5.96E+08	0.15	-3.72	3.98	0.06
E1	GPM2	1B	AX.89721228	6.62E+08	0.08	-1141.05	3.35	0.05
E1	GPM2	1B	AX.89516042	6.53E+08	0.12	-978.60	3.56	0.06
E1	GPM2	1B	AX.89513911	4.84E+08	0.19	807.71	3.47	0.05
E1	GY	1B	AX.89672607	5.93E+08	0.30	-2.66	3.46	0.06
E1	GPM2	1B	AX.89532392	5.55E+07	0.16	-934.78	4.00	0.07
E1	GPM2	1B	AX.89580989	6.46E+08	0.07	-1386.81	4.25	0.07
E1	GY	1B	AX.89635603	5.94E+08	0.15	-3.47	3.52	0.05
E1	GPM2	1B	AX.89624854	6.53E+08	0.12	-978.60	3.56	0.06
E1	GPM2	1B	AX.89430295	6.54E+08	0.13	-1079.82	4.35	0.08
E1	GPM2	1B	AX.89328659	4.73E+08	0.15	-823.32	3.08	0.05
E1	GPM2	1B	AX.89669649	4.92E+08	0.17	-793.84	3.20	0.06
E1	GY	1B	AX.89381273	6.15E+08	0.20	-3.18	3.71	0.06
E1	GY	1B	AX.89566816	5.48E+08	0.19	-3.13	3.50	0.05
E1	GY	1B	AX.89566816	5.48E+08	0.19	-3.13	3.50	0.05
E1	GY	1B	AX.89743657	5.45E+08	0.33	-2.57	3.42	0.05
E1	GY	1B	AX.89743657	5.45E+08	0.33	-2.57	3.42	0.05
E1	GY	1B	AX.89417724	5.94E+08	0.15	-3.47	3.52	0.05
E1	GPM2	1B	AX.89655162	6.54E+08	0.12	-978.60	3.56	0.06
E1	GPM2	1B	AX.89511165	6.62E+08	0.08	-1141.05	3.35	0.05
E1	GPM2	1B	AX.89704754	5.49E+07	0.35	-649.74	3.38	0.05
E1	GY	1B	AX.89686858	6.13E+08	0.14	-3.73	3.91	0.06
E1	GPM2	1B	AX.89597283	6.61E+08	0.09	-1080.74	3.46	0.06
E1	GPM2	1B	AX.89425103	6.53E+08	0.11	-1028.45	3.65	0.06
E1	GY	1B	AX.89637605	5.73E+08	0.09	-4.93	4.38	0.07
E1	GPM2	1B	AX.89629185	6.53E+08	0.12	-978.60	3.56	0.06
E1	GY	1B	AX.89431355	6.14E+08	0.20	-3.19	3.67	0.06
E1	GPM2	1B	AX.89581662	6.52E+08	0.11	-1028.45	3.65	0.06
E1	GPM2	1B	AX.89416093	6.62E+08	0.08	-1141.05	3.35	0.05
E1	GY	1B	AX.89525338	5.73E+08	0.08	-4.58	3.53	0.06
E1	GPM2	1B	AX.89536335	6.52E+08	0.12	-910.13	3.15	0.05
E1	GY	1B	AX.89454201	6.15E+08	0.20	-3.18	3.71	0.06
E1	GY	1B	AX.89383439	6.14E+08	0.20	-3.19	3.67	0.06
E1	GPM2	1B	AX.89428153	5.54E+07	0.15	-894.90	3.63	0.06
E1	GY	1B	AX.89756650	6.14E+08	0.20	-3.18	3.71	0.06
E1	GY	1B	AX.89751221	5.32E+08	0.20	-3.18	3.65	0.06
E1	GY	1B	AX.89432547	5.73E+08	0.08	-4.44	3.50	0.05
E1	GY	1B	AX.89373054	6.15E+08	0.20	-3.14	3.64	0.06
E1	GY	1B	AX.89596159	5.90E+08	0.26	-2.63	3.14	0.05

E1	GPM2	1B	AX.89601532	6.53E+08	0.12	-978.60	3.56	0.06
E1	GY	1B	AX.89765713	6.21E+08	0.19	-3.20	3.64	0.06
E1	GY	1B	AX.89596373	5.62E+08	0.28	-2.83	3.73	0.06
E1	GY	1B	AX.89454941	4.30E+08	0.39	2.57	3.60	0.07
E1	GY	1B	AX.89627039	5.71E+08	0.15	-3.40	3.40	0.05
E1	GPM2	1B	AX.89323974	5.39E+07	0.16	-799.94	3.12	0.05
E1	GY	1B	AX.89462493	5.95E+08	0.15	-3.47	3.52	0.05
E1	GPM2	1B	AX.89468035	6.52E+08	0.12	-978.60	3.56	0.06
E1	GPM2	1B	AX.89633505	5.27E+07	0.49	-594.29	3.15	0.05
E1	GPM2	1B	AX.89655760	6.46E+08	0.07	-1386.81	4.25	0.07
E1	GY	1B	AX.89706711	6.15E+08	0.20	-3.18	3.71	0.06
E1	GPM2	1B	AX.89472332	6.53E+08	0.11	-955.19	3.12	0.05
E1	GPM2	1B	AX.89494116	6.61E+08	0.09	-1080.74	3.46	0.06
E1	GPM2	1B	AX.89611811	5.41E+07	0.16	-849.20	3.39	0.05
E1	GPM2	1B	AX.89643801	6.53E+08	0.11	-1028.45	3.65	0.06
E1	GY	1B	AX.89720333	5.44E+08	0.45	-2.36	3.23	0.05
E1	GPM2	1B	AX.89694069	6.54E+08	0.14	-1059.20	4.54	0.08
E1	GPM2	1B	AX.89583474	5.55E+07	0.15	-839.65	3.25	0.05
E1	GPM2	1B	AX.89527090	6.59E+08	0.09	-1080.74	3.46	0.06
E1	GY	1B	AX.89674825	5.71E+08	0.09	-4.20	3.32	0.05
E1	GY	1B	AX.89674825	5.71E+08	0.09	-4.20	3.32	0.05
E1	GPM2	1B	AX.89426322	6.52E+08	0.11	-1028.45	3.65	0.06
E1	GPM2	1B	AX.89639580	4.84E+08	0.19	807.71	3.47	0.05
E1	GY	1B	AX.89708163	5.48E+08	0.19	-3.13	3.50	0.05
E1	GPM2	1B	AX.89557675	6.52E+08	0.13	-935.39	3.39	0.05
E1	GPM2	1B	AX.89765882	6.46E+08	0.07	-1386.81	4.25	0.07
E1	GY	1B	AX.89625060	5.50E+08	0.13	-3.47	3.13	0.05
E1	GY	1B	AX.89386960	5.73E+08	0.08	-4.44	3.50	0.05
E1	GPM2	1B	AX.89720405	6.54E+08	0.12	-978.60	3.56	0.06
E1	GPM2	1B	AX.89415657	6.32E+08	0.07	-1287.03	3.53	0.06
E1	GPM2	1B	AX.89457977	4.74E+08	0.14	-837.68	3.10	0.05
E1	GPM2	1B	AX.89722366	4.83E+08	0.19	767.15	3.23	0.05
E1	GY	1B	AX.89659701	5.63E+08	0.12	-3.53	3.04	0.05
E1	GY	1B	AX.89759919	5.90E+08	0.27	-2.69	3.30	0.06
E1	GPM2	1B	AX.89560184	6.60E+08	0.09	-1080.74	3.46	0.06
E1	GY	1B	AX.89353327	6.15E+08	0.20	-3.18	3.71	0.06
E1	GY	1B	AX.89685826	5.73E+08	0.10	-3.96	3.24	0.05
E1	GPM2	1B	AX.89717173	6.52E+08	0.12	-1050.54	3.91	0.06
E1	GY	1B	AX.89746518	5.32E+08	0.20	-3.17	3.68	0.06
E1	GY	1B	AX.89746518	5.32E+08	0.20	-3.17	3.68	0.06
E1	GY	1B	AX.89722505	5.71E+08	0.09	-4.20	3.32	0.05
E1	GPM2	1B	AX.89378367	6.53E+08	0.11	-1028.45	3.65	0.06
E1	GY	1B	AX.89553218	5.62E+08	0.28	-2.67	3.38	0.05
E1	GPM2	1B	AX.89731113	5.33E+07	0.16	-849.20	3.39	0.05
E1	GPM2	1B	AX.89321736	6.55E+08	0.08	-1073.58	3.02	0.05
E1	GPM2	1B	AX.89615276	4.84E+08	0.19	807.71	3.47	0.05
E1	GPM2	1B	AX.89410975	6.55E+08	0.08	-1073.58	3.02	0.05
E1	GPM2	1B	AX.89547196	6.53E+08	0.12	-978.60	3.56	0.06
E1	GPM2	1B	AX.89628976	6.59E+08	0.09	-1080.74	3.46	0.06
E1	GPM2	1B	AX.89449919	6.59E+08	0.09	-1080.74	3.46	0.06

E1	GY	1B	AX.89532430	4.73E+08	0.14	-3.28	3.06	0.05
E1	GY	1B	AX.89568217	5.89E+08	0.13	-4.04	4.06	0.07
E1	GY	1B	AX.89605272	6.16E+08	0.20	-3.18	3.71	0.06
E1	GY	1B	AX.89430167	5.71E+08	0.08	-4.16	3.00	0.05
E1	GY	1B	AX.89382181	5.73E+08	0.11	-4.47	4.28	0.07
E1	GY	1B	AX.89760457	5.90E+08	0.25	-2.69	3.20	0.05
E1	GY	1B	AX.89703958	6.15E+08	0.20	-3.18	3.71	0.06
E1	GY	1B	AX.89642296	5.72E+08	0.11	-3.78	3.32	0.05
E1	GY	1B	AX.89679490	5.73E+08	0.08	-4.44	3.50	0.05
E1	GY	1B	AX.89465482	5.43E+08	0.41	-2.68	3.95	0.07
E1	GY	1B	AX.89327316	5.95E+08	0.15	-3.72	3.98	0.06
E1	GY	1B	AX.89361485	6.15E+08	0.20	-3.18	3.71	0.06
E1	GY	1B	AX.89594022	6.14E+08	0.21	-3.21	3.94	0.06
E1	GY	1B	AX.89594022	6.14E+08	0.21	-3.21	3.94	0.06
E1	GY	1B	AX.89683293	5.71E+08	0.09	-4.20	3.32	0.05
E1	GY	1B	AX.89721599	5.94E+08	0.15	-3.72	3.98	0.06
E1	GY	1B	AX.89721858	5.89E+08	0.13	-4.04	4.06	0.07
E1	GY	1B	AX.89647056	5.43E+08	0.46	-2.72	4.15	0.08
E1	GY	1B	AX.89525700	5.91E+08	0.26	-2.63	3.14	0.05
E1	GPM2	1B	AX.89380977	1.78E+07	0.22	-733.12	3.26	0.06
E1	GY	1B	AX.89770837	5.43E+08	0.44	-2.72	4.11	0.07
E1	GPM2	1B	AX.89387732	4.86E+08	0.19	807.71	3.47	0.05
E1	GPM2	1B	AX.89407680	6.61E+08	0.16	-801.79	3.07	0.05
E1	GPM2	1B	AX.89491622	6.61E+08	0.15	-820.92	3.13	0.05
E1	GPM2	1B	AX.89441161	4.86E+08	0.20	762.45	3.34	0.05
E1	GPM2	1B	AX.89451610	6.59E+08	0.09	-1080.74	3.46	0.06
E1	GY	1B	AX.89686897	4.30E+08	0.39	2.58	3.65	0.07
E1	GPM2	1B	AX.89654829	5.66E+08	0.05	-1436.26	3.25	0.06
E1	GY	1B	AX.89566210	5.62E+08	0.29	-2.70	3.47	0.05
E1	GY	1B	AX.89504471	5.95E+08	0.15	-3.69	4.00	0.06
E1	GPM2	1B	AX.89528405	5.89E+08	0.12	-910.17	3.15	0.06
E1	GY	1B	AX.89543383	5.94E+08	0.15	-3.47	3.52	0.05
E1	GPM2	1B	AX.89550567	6.59E+08	0.09	-1080.74	3.46	0.06
E1	GPM2	1B	AX.89470764	6.53E+08	0.11	-1028.45	3.65	0.06
E1	GPM2	1B	AX.89336137	4.85E+08	0.16	803.34	3.08	0.05
E1	GPM2	1B	AX.89493476	5.26E+07	0.49	-639.51	3.57	0.05
E1	GPM2	1B	AX.89512032	6.54E+08	0.16	-810.67	3.13	0.05
E1	GPM2	1B	AX.89601546	4.84E+08	0.27	672.45	3.15	0.06
E1	GPM2	1B	AX.89670448	6.53E+08	0.11	-1028.45	3.65	0.06
E1	GPM2	1B	AX.89665864	6.55E+08	0.08	-1073.58	3.02	0.05
E1	GY	1B	AX.89657160	5.71E+08	0.09	-4.20	3.32	0.05
E1	GY	1B	AX.89463084	6.14E+08	0.20	-3.19	3.67	0.06
E1	GPM2	1B	AX.89494755	6.62E+08	0.05	-1709.59	4.76	0.08
E1	GPM2	1B	AX.89766983	4.86E+08	0.19	855.75	3.90	0.06
E1	GY	1B	AX.89500077	4.73E+08	0.14	-3.52	3.53	0.06
E1	GPM2	1B	AX.89664371	6.52E+08	0.13	-935.39	3.39	0.05
E1	GPM2	1B	AX.89776496	5.49E+07	0.16	-849.20	3.39	0.05
E1	GY	1B	AX.89371586	5.95E+08	0.15	-3.47	3.52	0.05
E1	GY	1B	AX.89503576	5.71E+08	0.09	-4.02	3.20	0.05
E1	GY	1B	AX.89728608	5.43E+08	0.41	-2.57	3.68	0.06

E1	GY	1B	AX.89372056	5.96E+08	0.15	-3.72	3.98	0.06
E1	GPM2	1B	AX.89442346	6.54E+08	0.12	-978.60	3.56	0.06
E1	GY	1B	AX.89645388	5.90E+08	0.25	-2.74	3.27	0.05
E1	GPM2	1B	AX.89341296	6.54E+08	0.14	-1059.20	4.54	0.08
E1	GPM2	1B	AX.89387997	5.53E+07	0.16	-849.20	3.39	0.05
E1	GY	1B	AX.89419135	4.29E+08	0.38	2.49	3.40	0.06
E1	GY	1B	AX.89583603	6.13E+08	0.14	-3.73	3.91	0.06
E1	GPM2	1B	AX.89515980	5.65E+08	0.05	-1436.26	3.25	0.06
E1	GPM2	1B	AX.89584833	5.49E+07	0.16	-849.20	3.39	0.05
E1	GPM2	1B	AX.89640880	6.59E+08	0.09	-1080.74	3.46	0.06
E1	GPM2	1B	AX.89524034	6.46E+08	0.07	-1386.81	4.25	0.07
E1	GPM2	1B	AX.89468290	4.85E+08	0.19	767.15	3.23	0.05
E1	GPM2	1B	AX.89534404	6.54E+08	0.22	-706.48	3.10	0.05
E1	GPM2	1B	AX.89407995	6.55E+08	0.08	-1073.58	3.02	0.05
E1	GPM2	1B	AX.89469915	6.60E+08	0.10	-984.82	3.07	0.05
E1	GPM2	1B	AX.89345423	4.85E+08	0.19	807.71	3.47	0.05
E1	GPM2	1B	AX.89516168	6.59E+08	0.09	-1080.74	3.46	0.06
E1	GY	1B	AX.89549904	5.90E+08	0.26	-2.63	3.13	0.05
E1	GY	1B	AX.89549904	5.90E+08	0.26	-2.63	3.13	0.05
E1	GPM2	1B	AX.89433914	4.85E+08	0.19	767.15	3.23	0.05
E1	GPM2	1B	AX.89426263	5.58E+07	0.16	-849.20	3.39	0.05
E1	GPM2	1B	AX.89337283	5.49E+07	0.16	-823.48	3.28	0.06
E1	GPM2	1B	AX.89679175	4.86E+08	0.19	807.71	3.47	0.05
E1	GPM2	1B	AX.89536040	4.73E+08	0.14	-837.68	3.10	0.05
E1	GPM2	1B	AX.89396197	6.53E+08	0.11	-1028.45	3.65	0.06
E1	GPM2	1B	AX.89356974	5.53E+07	0.15	-887.39	3.58	0.06
E1	GPM2	1B	AX.89335520	6.54E+08	0.08	-1073.58	3.02	0.05
E1	GPM2	1B	AX.89409179	4.73E+08	0.14	-837.68	3.10	0.05
E1	GPM2	1B	AX.89331198	5.64E+08	0.05	-1384.93	3.30	0.06
E1	GY	1B	AX.89436079	6.15E+08	0.17	-3.09	3.14	0.05
E1	GPM2	1B	AX.89712984	6.53E+08	0.12	-978.60	3.56	0.06
E1	GPM2	1B	AX.89713014	6.55E+08	0.08	-1073.58	3.02	0.05
E1	GPM2	1B	AX.89348612	4.83E+08	0.19	767.15	3.23	0.05
E1	GPM2	1B	AX.89683248	6.53E+08	0.12	-978.60	3.56	0.06
E1	GPM2	1B	AX.89500889	6.61E+08	0.15	-851.73	3.33	0.05
E1	GPM2	1B	AX.89616173	6.53E+08	0.12	-954.36	3.31	0.05
E1	GPM2	1B	AX.89565905	6.59E+08	0.09	-1080.74	3.46	0.06
E1	GPM2	1B	AX.89649851	6.53E+08	0.11	-1028.45	3.65	0.06
E1	GY	1B	AX.89596577	5.95E+08	0.15	-3.72	3.98	0.06
E1	GPM2	1B	AX.89658411	4.84E+08	0.19	807.71	3.47	0.05
E1	GPM2	1B	AX.89764633	6.52E+08	0.11	-1028.45	3.65	0.06
E1	GY	1B	AX.89742936	5.89E+08	0.27	-2.70	3.35	0.06
E1	GPM2	1B	AX.89748431	6.53E+08	0.11	-1028.45	3.65	0.06
E1	GPM2	1B	AX.89725397	5.63E+08	0.48	-616.75	3.36	0.05
E1	GPM2	1B	AX.89366249	6.53E+08	0.12	-978.60	3.56	0.06
E1	GPM2	1B	AX.89323536	6.61E+08	0.15	-820.92	3.13	0.05
E1	GY	1B	AX.89381776	4.29E+08	0.39	2.57	3.60	0.07
E1	TKW	1B	AX.89326274	5.49E+08	0.48	0.85	3.13	0.05
E1	GPM2	1B	AX.89431736	5.63E+07	0.29	-635.47	3.01	0.05
E1	GPM2	1B	AX.89689457	5.49E+07	0.22	-701.99	3.03	0.05

E1	GY	1B	AX.89756848	6.14E+08	0.20	-3.19	3.67	0.06
E1	TKW	1B	AX.89475250	5.50E+08	0.47	0.92	3.61	0.06
E1	GPM2	1B	AX.89395053	6.53E+08	0.11	-1028.45	3.65	0.06
E1	GPM2	1B	AX.89607064	6.61E+08	0.15	-820.92	3.13	0.05
E1	GPM2	1B	AX.89395127	6.54E+08	0.12	-1134.04	4.47	0.08
E1	GPM2	1B	AX.89769126	4.72E+08	0.14	-837.68	3.10	0.05
E1	TKW	1B	AX.89551532	5.49E+08	0.48	0.86	3.17	0.05
E1	GPM2	1B	AX.89361355	4.72E+08	0.14	-942.17	3.70	0.07
E1	GPM2	1B	AX.89761483	6.53E+08	0.11	-1028.45	3.65	0.06
E1	GPM2	1B	AX.89749968	6.53E+08	0.12	-978.60	3.56	0.06
E1	GY	1B	AX.89578393	5.71E+08	0.09	-4.20	3.32	0.05
E1	GPM2	1B	AX.89530026	6.52E+08	0.11	-1028.45	3.65	0.06
E1	GPM2	1B	AX.89594959	6.53E+08	0.11	-1028.45	3.65	0.06
E1	GPM2	1B	AX.89484512	6.62E+08	0.08	-1141.05	3.35	0.05
E1	GPM2	1B	AX.89463644	6.53E+08	0.11	-1028.45	3.65	0.06
E1	GPM2	1B	AX.89768752	4.86E+08	0.18	802.88	3.32	0.05
E1	GY	1B	AX.89586686	5.48E+08	0.19	-3.13	3.50	0.05
E1	GPM2	1B	AX.89321912	5.53E+07	0.16	-849.20	3.39	0.05
E1	GY	1B	AX.89584485	4.46E+07	0.47	2.36	3.24	0.05
E1	TKW	1B	AX.89603695	5.49E+08	0.48	0.86	3.17	0.05
E1	GPM2	1B	AX.89338879	6.62E+08	0.08	-1141.05	3.35	0.05
E1	GPM2	1B	AX.89331955	4.53E+07	0.05	-1329.70	3.08	0.05
E1	TKW	1B	AX.89619354	5.51E+08	0.48	0.86	3.17	0.05
E1	TKW	1B	AX.89513504	5.51E+08	0.48	0.86	3.17	0.05
E1	GPM2	1B	AX.89527101	4.86E+08	0.20	815.28	3.64	0.05
E1	GY	1B	AX.89691473	5.96E+08	0.15	-3.72	3.98	0.06
E1	TKW	1B	AX.89681224	6.81E+08	0.20	1.14	3.53	0.06
E1	TKW	1B	AX.89398141	5.49E+08	0.47	0.85	3.12	0.05
E1	TKW	1B	AX.89448417	6.49E+08	0.08	-1.53	3.13	0.05
E1	GPM2	1B	AX.89547484	6.53E+08	0.12	-978.60	3.56	0.06
E1	GPM2	1B	AX.89320488	6.53E+08	0.12	-978.60	3.56	0.06
E1	GPM2	1B	AX.89669607	4.84E+08	0.19	807.71	3.47	0.05
E1	GPM2	1B	AX.89666576	4.84E+08	0.20	779.09	3.37	0.05
E1	GY	1B	AX.89468178	5.89E+08	0.13	-4.04	4.06	0.07
E1	GPM2	1B	AX.89512303	4.85E+08	0.19	807.71	3.47	0.05
E1	GPM2	1B	AX.89639158	6.62E+08	0.08	-1141.05	3.35	0.05
E1	TKW	1B	AX.89725558	5.49E+08	0.48	0.86	3.17	0.05
E1	GY	1B	AX.89600634	5.93E+08	0.13	-3.89	4.01	0.06
E1	GPM2	1B	AX.89706580	5.34E+07	0.16	-799.94	3.12	0.05
E1	TKW	1B	AX.89468317	5.50E+08	0.48	0.86	3.17	0.05
E1	GPM2	1B	AX.89718423	5.64E+08	0.39	623.31	3.28	0.06
E1	GPM2	1B	AX.89684014	4.84E+08	0.19	807.71	3.47	0.05
E1	GPM2	1B	AX.89524297	4.84E+08	0.19	807.71	3.47	0.05
E1	GPM2	1B	AX.89484812	6.55E+08	0.08	-1073.58	3.02	0.05
E1	TKW	1B	AX.89472579	5.50E+08	0.47	0.85	3.12	0.05
E1	TKW	1B	AX.89520572	5.47E+08	0.33	-0.98	3.65	0.06
E1	GY	1B	AX.89589368	5.71E+08	0.15	-3.40	3.40	0.05
E1	GPM2	1B	AX.89577475	5.56E+07	0.16	-849.20	3.39	0.05
E1	GPM2	1B	AX.89695679	6.61E+08	0.09	-1080.74	3.46	0.06
E1	GPM2	1B	AX.89678168	6.62E+08	0.08	-1141.05	3.35	0.05

E1	GPM2	1B	AX.89625377	6.53E+08	0.12	-978.60	3.56	0.06
E1	TKW	1B	AX.89697293	5.47E+08	0.33	-0.89	3.07	0.05
E1	TKW	1B	AX.89672472	5.50E+08	0.48	0.86	3.17	0.05
E1	TKW	1B	AX.89745028	5.50E+08	0.48	0.85	3.13	0.05
E1	TKW	1B	AX.89319566	5.50E+08	0.48	0.86	3.17	0.05
E1	TKW	1B	AX.89342287	6.41E+08	0.13	1.27	3.15	0.05
E1	TKW	1B	AX.89594793	5.49E+08	0.48	0.86	3.17	0.05
E1	TKW	1B	AX.89536084	5.46E+08	0.33	-0.93	3.30	0.05
E1	TKW	1B	AX.89706986	5.49E+08	0.48	0.86	3.17	0.05
E1	TKW	1B	AX.89731954	5.50E+08	0.47	0.90	3.45	0.06
E1	GY	1B	AX.89525700	5.91E+08	0.26	-2.63	3.14	0.05
E1	TKW	1B	AX.89478064	6.49E+08	0.08	-1.53	3.13	0.05
E1	TKW	1B	AX.89701772	6.41E+08	0.13	1.27	3.15	0.05
E1	TKW	1B	AX.89668865	5.43E+08	0.32	-0.99	3.63	0.06
E1	GPM2	1B	AX.89421818	4.72E+08	0.14	-942.17	3.70	0.07
E1	TKW	1B	AX.89419625	5.49E+08	0.31	-0.91	3.05	0.05
E1	TKW	1B	AX.89657961	5.51E+08	0.48	0.86	3.17	0.05
E1	GPM2	1B	AX.89512056	4.83E+08	0.19	767.15	3.23	0.05
E1	GPM2	1B	AX.89679406	6.59E+08	0.09	-1080.74	3.46	0.06
E1	GPM2	1B	AX.89522202	4.87E+08	0.19	807.71	3.47	0.05
E1	GY	1B	AX.89578881	5.45E+08	0.33	-2.57	3.42	0.05
E1	TKW	1B	AX.89511243	5.45E+08	0.33	-0.96	3.47	0.06
E1	TKW	1B	AX.89541507	5.50E+08	0.32	-0.91	3.13	0.05
E1	TKW	1B	AX.89753324	5.49E+08	0.49	0.85	3.12	0.05
E1	GPM2	1B	AX.89679851	5.52E+07	0.16	-849.20	3.39	0.05
E1	GPM2	1B	AX.89318397	6.53E+08	0.11	-1028.45	3.65	0.06
E1	TKW	1B	AX.89577934	5.51E+08	0.48	0.86	3.17	0.05
E1	TKW	1B	AX.89595857	6.80E+08	0.21	1.07	3.28	0.06
E1	TKW	1B	AX.89476140	5.51E+08	0.47	0.85	3.12	0.05
E1	TKW	1B	AX.89743657	5.45E+08	0.33	-0.93	3.30	0.05
E1	GPM2	1B	AX.89594055	5.41E+07	0.16	-849.20	3.39	0.05
E1	GPM2	1B	AX.89361803	5.63E+08	0.48	-616.75	3.36	0.05
E1	TKW	1B	AX.89578881	5.45E+08	0.33	-0.93	3.30	0.05
E1	GPM2	1B	AX.89444867	6.53E+08	0.12	-978.60	3.56	0.06
E1	TKW	1B	AX.89492525	3.83E+08	0.47	0.90	3.43	0.06
E1	TKW	1B	AX.89374992	5.50E+08	0.48	0.86	3.17	0.05
E1	GPM2	1B	AX.89584051	5.55E+07	0.16	-849.20	3.39	0.05
E1	GPM2	1B	AX.89326413	4.85E+08	0.19	807.71	3.47	0.05
E1	GPM2	1B	AX.89566039	5.49E+07	0.16	-849.20	3.39	0.05
E1	GPM2	1B	AX.89760192	6.62E+08	0.07	-1247.19	3.72	0.06
E1	TKW	1B	AX.89388830	5.50E+08	0.48	0.86	3.17	0.05
E1	TKW	1B	AX.89580983	5.51E+08	0.48	0.86	3.17	0.05
E1	TKW	1B	AX.89582073	5.51E+08	0.48	0.86	3.21	0.05
E1	TKW	1B	AX.89347943	5.46E+08	0.33	-0.93	3.30	0.05
E1	TKW	1B	AX.89671741	6.81E+08	0.23	1.01	3.14	0.05
E1	GPM2	1B	AX.89588545	6.59E+08	0.09	-1080.74	3.46	0.06
E1	GPM2	1B	AX.89402478	5.63E+08	0.48	-616.75	3.36	0.05
E1	GY	1B	AX.89568032	6.15E+08	0.20	-3.18	3.71	0.06
E1	GPM2	1B	AX.89602436	6.55E+08	0.08	-1073.58	3.02	0.05
E1	GPM2	1B	AX.89339365	6.62E+08	0.08	-1141.05	3.35	0.05

E1	GPM2	1B	AX.89768630	4.85E+08	0.19	767.15	3.23	0.05
E1	TKW	1B	AX.89517851	6.49E+08	0.08	-1.53	3.13	0.05
E1	TKW	1B	AX.89741485	5.49E+08	0.47	0.85	3.12	0.05
E1	GY	1B	AX.89627315	5.73E+08	0.08	-4.44	3.50	0.05
E1	TKW	1B	AX.89401788	5.49E+08	0.48	0.84	3.08	0.05
E1	TKW	1B	AX.89504746	5.51E+08	0.48	0.86	3.17	0.05
E1	TKW	1B	AX.89705004	5.49E+08	0.48	0.86	3.17	0.05
E1	TKW	1B	AX.89556842	5.50E+08	0.48	0.86	3.17	0.05
E1	GY	1B	AX.89714925	5.71E+08	0.09	-4.20	3.32	0.05
E1	TKW	1B	AX.89434309	5.51E+08	0.48	0.86	3.17	0.05
E1	TKW	1B	AX.89565326	5.49E+08	0.48	0.84	3.08	0.05
E1	TKW	1B	AX.89475531	5.47E+08	0.48	0.86	3.17	0.05
E1	GY	1B	AX.89713584	6.15E+08	0.20	-3.18	3.71	0.06
E1	TKW	1B	AX.89467807	5.46E+08	0.34	-1.02	3.90	0.06
E1	TKW	1B	AX.89442105	5.51E+08	0.48	0.86	3.17	0.05
E1	TKW	1B	AX.89605837	5.48E+08	0.48	0.86	3.17	0.05
E1	TKW	1B	AX.89667596	5.49E+08	0.48	0.86	3.17	0.05
E2	GY	1B	AX.89399318	5.95E+08	0.15	-3.00	3.79	0.05
E2	GY	1B	AX.89399318	5.95E+08	0.15	-3.00	3.79	0.05
E2	GY	1B	AX.89400618	5.96E+08	0.15	-3.00	3.79	0.05
E2	GY	1B	AX.89494116	6.61E+08	0.09	-3.38	3.26	0.04
E2	GY	1B	AX.89494755	6.62E+08	0.05	-6.79	6.93	0.10
E2	GY	1B	AX.89494755	6.62E+08	0.05	-6.79	6.93	0.10
E2	GPM2	1B	AX.89700186	6.65E+08	0.11	-784.37	3.05	0.07
E2	GY	1B	AX.89639158	6.62E+08	0.08	-4.00	3.82	0.05
E2	GPM2	1B	AX.89681224	6.81E+08	0.20	-646.62	3.37	0.05
E2	GY	1B	AX.89507799	6.66E+08	0.07	-4.86	4.86	0.07
E2	GY	1B	AX.89760192	6.62E+08	0.07	-4.34	4.20	0.06
E2	GPM2	1B	AX.89372089	6.62E+08	0.08	-919.40	3.20	0.04
E2	GY	1B	AX.89637779	5.94E+08	0.05	-4.84	3.78	0.05
E2	GY	1B	AX.89368826	5.94E+08	0.05	-4.84	3.78	0.05
E2	GY	1B	AX.89368826	5.94E+08	0.05	-4.84	3.78	0.05
E2	GY	1B	AX.89420250	5.94E+08	0.06	-4.11	3.27	0.04
E2	GY	1B	AX.89420250	5.94E+08	0.06	-4.11	3.27	0.04
E2	GY	1B	AX.89334216	5.76E+08	0.09	-3.23	3.04	0.04
E2	GY	1B	AX.89544768	5.95E+08	0.15	-2.74	3.25	0.04
E2	GY	1B	AX.89544768	5.95E+08	0.15	-2.74	3.25	0.04
E2	TKW	1B	AX.89671741	6.81E+08	0.23	1.15	3.47	0.06
E2	GY	1B	AX.89580727	5.96E+08	0.15	-3.00	3.79	0.05
E2	GY	1B	AX.89690662	5.94E+08	0.15	-2.74	3.25	0.04
E2	GY	1B	AX.89492307	5.75E+08	0.09	-3.23	3.04	0.04
E2	SPM2	1B	AX.89435072	6.70E+08	0.06	25.84	3.36	0.06
E2	GY	1B	AX.89430295	6.54E+08	0.13	-2.91	3.18	0.06
E2	GY	1B	AX.89580989	6.46E+08	0.07	-4.54	4.34	0.06
E2	GY	1B	AX.89580989	6.46E+08	0.07	-4.54	4.34	0.06
E2	GY	1B	AX.89430501	5.94E+08	0.05	-4.84	3.78	0.05
E2	SPM2	1B	AX.89498443	6.82E+08	0.07	26.28	3.87	0.07
E2	GY	1B	AX.89724852	5.94E+08	0.15	-2.98	3.84	0.05
E2	GY	1B	AX.89543383	5.94E+08	0.15	-2.74	3.25	0.04
E2	GY	1B	AX.89543383	5.94E+08	0.15	-2.74	3.25	0.04

E2	GY	1B	AX.89372056	5.96E+08	0.15	-3.00	3.79	0.05
E2	GY	1B	AX.89679406	6.59E+08	0.09	-3.38	3.26	0.04
E2	SPM2	1B	AX.89441693	3.29E+08	0.19	15.43	3.21	0.05
E2	GY	1B	AX.89416093	6.62E+08	0.08	-4.00	3.82	0.05
E2	SPM2	1B	AX.89349200	6.31E+08	0.13	18.94	3.47	0.06
E2	GY	1B	AX.89479897	5.96E+08	0.15	-3.00	3.79	0.05
E2	GY	1B	AX.89727207	5.94E+08	0.15	-2.98	3.84	0.05
E2	GY	1B	AX.89565905	6.59E+08	0.09	-3.38	3.26	0.04
E2	GY	1B	AX.89520284	5.76E+08	0.09	-3.23	3.04	0.04
E2	GY	1B	AX.89375527	6.61E+08	0.09	-3.38	3.26	0.04
E2	GY	1B	AX.89375527	6.61E+08	0.09	-3.38	3.26	0.04
E2	GY	1B	AX.89550567	6.59E+08	0.09	-3.38	3.26	0.04
E2	GY	1B	AX.89550567	6.59E+08	0.09	-3.38	3.26	0.04
E2	GY	1B	AX.89610491	6.62E+08	0.08	-3.87	3.82	0.05
E2	GY	1B	AX.89610491	6.62E+08	0.08	-3.87	3.82	0.05
E2	GY	1B	AX.89655332	6.65E+08	0.10	-3.77	4.16	0.09
E2	GY	1B	AX.89655332	6.65E+08	0.10	-3.77	4.16	0.09
E2	GY	1B	AX.89518107	5.95E+08	0.15	-2.74	3.25	0.04
E2	GY	1B	AX.89518107	5.95E+08	0.15	-2.74	3.25	0.04
E2	GY	1B	AX.89484512	6.62E+08	0.08	-4.00	3.82	0.05
E2	GY	1B	AX.89484512	6.62E+08	0.08	-4.00	3.82	0.05
E2	GY	1B	AX.89660818	6.65E+08	0.13	-3.02	3.42	0.07
E2	GY	1B	AX.89660818	6.65E+08	0.13	-3.02	3.42	0.07
E2	GY	1B	AX.89564830	5.94E+08	0.05	-4.84	3.78	0.05
E2	GY	1B	AX.89564830	5.94E+08	0.05	-4.84	3.78	0.05
E2	GPM2	1B	AX.89765882	6.46E+08	0.07	-1052.91	3.68	0.06
E2	GY	1B	AX.89485285	6.65E+08	0.11	-3.63	4.06	0.09
E2	GY	1B	AX.89485285	6.65E+08	0.11	-3.63	4.06	0.09
E2	GY	1B	AX.89414699	5.95E+08	0.15	-2.98	3.84	0.05
E2	GY	1B	AX.89367182	5.95E+08	0.17	-2.68	3.39	0.04
E2	GY	1B	AX.89367182	5.95E+08	0.17	-2.68	3.39	0.04
E2	GY	1B	AX.89449919	6.59E+08	0.09	-3.38	3.26	0.04
E2	GY	1B	AX.89449919	6.59E+08	0.09	-3.38	3.26	0.04
E2	GY	1B	AX.89598862	6.82E+08	0.08	-3.69	3.35	0.11
E2	GY	1B	AX.89598862	6.82E+08	0.08	-3.69	3.35	0.11
E2	GY	1B	AX.89380858	5.94E+08	0.15	-2.98	3.84	0.05
E2	GY	1B	AX.89380858	5.94E+08	0.15	-2.98	3.84	0.05
E2	GY	1B	AX.89655760	6.46E+08	0.07	-4.54	4.34	0.06
E2	GY	1B	AX.89655760	6.46E+08	0.07	-4.54	4.34	0.06
E2	GY	1B	AX.89666109	6.62E+08	0.08	-3.55	3.26	0.04
E2	GY	1B	AX.89666109	6.62E+08	0.08	-3.55	3.26	0.04
E2	GY	1B	AX.89640880	6.59E+08	0.09	-3.38	3.26	0.04
E2	GY	1B	AX.89640880	6.59E+08	0.09	-3.38	3.26	0.04
E2	GY	1B	AX.89417724	5.94E+08	0.15	-2.74	3.25	0.04
E2	GY	1B	AX.89417724	5.94E+08	0.15	-2.74	3.25	0.04
E2	GY	1B	AX.89628976	6.59E+08	0.09	-3.38	3.26	0.04
E2	GY	1B	AX.89628976	6.59E+08	0.09	-3.38	3.26	0.04
E2	GY	1B	AX.89546341	5.94E+08	0.15	-2.98	3.84	0.05
E2	GY	1B	AX.89546341	5.94E+08	0.15	-2.98	3.84	0.05
E2	GY	1B	AX.89347349	5.94E+08	0.14	-2.75	3.20	0.04

E2	GY	1B	AX.89347349	5.94E+08	0.14	-2.75	3.20	0.04
E2	GY	1B	AX.89635603	5.94E+08	0.15	-2.74	3.25	0.04
E2	GY	1B	AX.89635603	5.94E+08	0.15	-2.74	3.25	0.04
E2	GY	1B	AX.89594265	5.76E+08	0.09	-3.23	3.04	0.04
E2	GY	1B	AX.89594265	5.76E+08	0.09	-3.23	3.04	0.04
E2	GY	1B	AX.89384148	5.76E+08	0.09	-3.23	3.04	0.04
E2	GY	1B	AX.89384148	5.76E+08	0.09	-3.23	3.04	0.04
E2	GY	1B	AX.89527090	6.59E+08	0.09	-3.38	3.26	0.04
E2	GY	1B	AX.89527090	6.59E+08	0.09	-3.38	3.26	0.04
E2	GY	1B	AX.89597283	6.61E+08	0.09	-3.38	3.26	0.04
E2	GY	1B	AX.89597283	6.61E+08	0.09	-3.38	3.26	0.04
E2	GY	1B	AX.89719593	1.44E+07	0.16	-3.02	4.07	0.05
E2	GY	1B	AX.89719593	1.44E+07	0.16	-3.02	4.07	0.05
E2	GY	1B	AX.89644951	1.42E+07	0.20	-2.93	4.49	0.06
E2	GY	1B	AX.89644951	1.42E+07	0.20	-2.93	4.49	0.06
E2	GY	1B	AX.89695679	6.61E+08	0.09	-3.38	3.26	0.04
E2	GY	1B	AX.89317797	5.95E+08	0.14	-2.75	3.20	0.04
E2	GY	1B	AX.89317797	5.95E+08	0.14	-2.75	3.20	0.04
E2	GY	1B	AX.89386642	5.75E+08	0.09	-3.23	3.04	0.04
E2	GY	1B	AX.89386642	5.75E+08	0.09	-3.23	3.04	0.04
E2	TKW	1B	AX.89765155	5.16E+07	0.14	1.48	3.81	0.07
E2	TKW	1B	AX.89773932	5.15E+07	0.19	1.21	3.43	0.06
E2	GY	1B	AX.89655654	6.62E+08	0.08	-4.00	3.82	0.05
E2	GY	1B	AX.89655654	6.62E+08	0.08	-4.00	3.82	0.05
E2	GY	1B	AX.89341296	6.54E+08	0.14	-2.72	3.05	0.06
E2	GY	1B	AX.89341296	6.54E+08	0.14	-2.72	3.05	0.06
E2	GY	1B	AX.89320059	5.95E+08	0.15	-3.00	3.79	0.05
E2	GY	1B	AX.89320059	5.95E+08	0.15	-3.00	3.79	0.05
E2	GY	1B	AX.89710848	5.95E+08	0.14	-2.75	3.20	0.04
E2	GY	1B	AX.89710848	5.95E+08	0.14	-2.75	3.20	0.04
E2	GPM2	1B	AX.89608895	6.81E+08	0.23	-571.77	3.02	0.04
E2	GY	1B	AX.89485001	5.95E+08	0.15	-3.00	3.79	0.05
E2	GY	1B	AX.89485001	5.95E+08	0.15	-3.00	3.79	0.05
E2	GY	1B	AX.89321187	6.61E+08	0.09	-3.38	3.26	0.04
E2	GY	1B	AX.89321187	6.61E+08	0.09	-3.38	3.26	0.04
E2	GY	1B	AX.89414699	5.95E+08	0.15	-2.98	3.84	0.05
E2	GY	1B	AX.89355729	5.94E+08	0.15	-2.74	3.25	0.04
E2	GY	1B	AX.89355729	5.94E+08	0.15	-2.74	3.25	0.04
E2	GY	1B	AX.89392454	5.95E+08	0.14	-2.75	3.20	0.04
E2	GY	1B	AX.89356982	6.54E+08	0.13	-2.91	3.18	0.06
E2	GY	1B	AX.89356982	6.54E+08	0.13	-2.91	3.18	0.06
E2	GY	1B	AX.89323934	6.66E+08	0.10	-3.77	4.16	0.09
E2	GY	1B	AX.89323934	6.66E+08	0.10	-3.77	4.16	0.09
E2	GY	1B	AX.89451610	6.59E+08	0.09	-3.38	3.26	0.04
E2	GY	1B	AX.89462493	5.95E+08	0.15	-2.74	3.25	0.04
E2	GY	1B	AX.89462493	5.95E+08	0.15	-2.74	3.25	0.04
E2	GY	1B	AX.89616546	6.62E+08	0.10	-3.39	3.38	0.04
E2	GY	1B	AX.89605104	5.76E+08	0.09	-3.23	3.04	0.04
E2	GY	1B	AX.89605104	5.76E+08	0.09	-3.23	3.04	0.04
E2	TKW	1B	AX.89673159	5.03E+07	0.18	1.24	3.43	0.06

E2	GY	1B	AX.89395127	6.54E+08	0.12	-3.29	3.71	0.07
E2	GY	1B	AX.89395127	6.54E+08	0.12	-3.29	3.71	0.07
E2	GY	1B	AX.89581957	5.95E+08	0.15	-3.00	3.79	0.05
E2	GY	1B	AX.89581957	5.95E+08	0.15	-3.00	3.79	0.05
E2	GY	1B	AX.89327316	5.95E+08	0.15	-3.00	3.79	0.05
E2	GY	1B	AX.89327316	5.95E+08	0.15	-3.00	3.79	0.05
E2	GY	1B	AX.89397557	5.94E+08	0.15	-2.98	3.84	0.05
E2	GY	1B	AX.89397557	5.94E+08	0.15	-2.98	3.84	0.05
E2	GY	1B	AX.89695679	6.61E+08	0.09	-3.38	3.26	0.04
E2	GY	1B	AX.89339365	6.62E+08	0.08	-4.00	3.82	0.05
E2	GY	1B	AX.89504471	5.95E+08	0.15	-2.98	3.84	0.05
E2	GY	1B	AX.89646690	5.96E+08	0.15	-3.00	3.79	0.05
E2	GY	1B	AX.89646690	5.96E+08	0.15	-3.00	3.79	0.05
E2	GY	1B	AX.89400618	5.96E+08	0.15	-3.00	3.79	0.05
E2	GY	1B	AX.89540478	5.74E+08	0.09	-3.23	3.04	0.04
E2	GY	1B	AX.89540478	5.74E+08	0.09	-3.23	3.04	0.04
E2	TKW	1B	AX.89748603	5.00E+07	0.16	1.23	3.17	0.06
E2	GY	1B	AX.89494116	6.61E+08	0.09	-3.38	3.26	0.04
E2	GY	1B	AX.89435723	6.66E+08	0.10	-3.77	4.16	0.09
E2	TKW	1B	AX.89775611	5.01E+07	0.33	1.00	3.34	0.06
E2	TKW	1B	AX.89620528	5.03E+07	0.17	1.45	4.35	0.08
E2	GPM2	1B	AX.89524034	6.46E+08	0.07	-1052.91	3.68	0.06
E2	GY	1B	AX.89401886	5.95E+08	0.14	-2.75	3.20	0.04
E2	GY	1B	AX.89401886	5.95E+08	0.14	-2.75	3.20	0.04
E2	TKW	1B	AX.89584849	5.38E+07	0.21	1.16	3.36	0.06
E2	GY	1B	AX.89639158	6.62E+08	0.08	-4.00	3.82	0.05
E2	GPM2	1B	AX.89655654	6.62E+08	0.08	-919.40	3.20	0.04
E2	GY	1B	AX.89507799	6.66E+08	0.07	-4.86	4.86	0.07
E2	GY	1B	AX.89760192	6.62E+08	0.07	-4.34	4.20	0.06
E2	TKW	1B	AX.89631333	5.02E+07	0.15	1.29	3.21	0.06
E2	GY	1B	AX.89721228	6.62E+08	0.08	-4.00	3.82	0.05
E2	GY	1B	AX.89700186	6.65E+08	0.11	-3.63	4.06	0.09
E2	GY	1B	AX.89637779	5.94E+08	0.05	-4.84	3.78	0.05
E2	GPM2	1B	AX.89694069	6.54E+08	0.14	-708.90	3.09	0.06
E2	GY	1B	AX.89344904	6.59E+08	0.09	-3.38	3.26	0.04
E2	GY	1B	AX.89344904	6.59E+08	0.09	-3.38	3.26	0.04
E2	GY	1B	AX.89392454	5.95E+08	0.14	-2.75	3.20	0.04
E2	GY	1B	AX.89754639	5.95E+08	0.14	-2.75	3.20	0.04
E2	GPM2	1B	AX.89598862	6.82E+08	0.08	-892.10	3.06	0.10
E2	GY	1B	AX.89357199	5.94E+08	0.15	-2.74	3.25	0.04
E2	GY	1B	AX.89357199	5.94E+08	0.15	-2.74	3.25	0.04
E2	GY	1B	AX.89451610	6.59E+08	0.09	-3.38	3.26	0.04
E2	GY	1B	AX.89334216	5.76E+08	0.09	-3.23	3.04	0.04
E2	GY	1B	AX.89694069	6.54E+08	0.14	-2.72	3.05	0.06
E2	GY	1B	AX.89616546	6.62E+08	0.10	-3.39	3.38	0.04
E2	GY	1B	AX.89334781	5.96E+08	0.15	-3.00	3.79	0.05
E2	GY	1B	AX.89334781	5.96E+08	0.15	-3.00	3.79	0.05
E2	GY	1B	AX.89512032	6.54E+08	0.16	-2.56	3.01	0.05
E2	GY	1B	AX.89693737	5.74E+08	0.09	-3.23	3.04	0.04
E2	GY	1B	AX.89693737	5.74E+08	0.09	-3.23	3.04	0.04

E2	GY	1B	AX.89580727	5.96E+08	0.15	-3.00	3.79	0.05
E2	GY	1B	AX.89516168	6.59E+08	0.09	-3.38	3.26	0.04
E2	GY	1B	AX.89690662	5.94E+08	0.15	-2.74	3.25	0.04
E2	GY	1B	AX.89492307	5.75E+08	0.09	-3.23	3.04	0.04
E2	GPM2	1B	AX.89494755	6.62E+08	0.05	-1605.49	6.05	0.09
E2	GY	1B	AX.89430295	6.54E+08	0.13	-2.91	3.18	0.06
E2	GY	1B	AX.89746393	6.62E+08	0.08	-4.00	3.82	0.05
E2	GY	1B	AX.89746393	6.62E+08	0.08	-4.00	3.82	0.05
E2	GY	1B	AX.89430501	5.94E+08	0.05	-4.84	3.78	0.05
E2	GY	1B	AX.89371586	5.95E+08	0.15	-2.74	3.25	0.04
E2	GY	1B	AX.89371586	5.95E+08	0.15	-2.74	3.25	0.04
E2	GY	1B	AX.89691473	5.96E+08	0.15	-3.00	3.79	0.05
E2	GY	1B	AX.89546727	6.62E+08	0.08	-4.00	3.82	0.05
E2	GY	1B	AX.89546727	6.62E+08	0.08	-4.00	3.82	0.05
E2	GY	1B	AX.89724852	5.94E+08	0.15	-2.98	3.84	0.05
E2	GY	1B	AX.89372056	5.96E+08	0.15	-3.00	3.79	0.05
E2	GY	1B	AX.89679406	6.59E+08	0.09	-3.38	3.26	0.04
E2	GY	1B	AX.89372089	6.62E+08	0.08	-4.00	3.82	0.05
E2	GY	1B	AX.89557668	5.75E+08	0.09	-3.23	3.04	0.04
E2	GY	1B	AX.89557668	5.75E+08	0.09	-3.23	3.04	0.04
E2	GY	1B	AX.89416093	6.62E+08	0.08	-4.00	3.82	0.05
E2	GY	1B	AX.89564115	5.76E+08	0.09	-3.23	3.04	0.04
E2	GY	1B	AX.89655981	5.75E+08	0.09	-3.23	3.04	0.04
E2	GY	1B	AX.89655981	5.75E+08	0.09	-3.23	3.04	0.04
E2	GY	1B	AX.89511165	6.62E+08	0.08	-4.00	3.82	0.05
E2	GY	1B	AX.89511165	6.62E+08	0.08	-4.00	3.82	0.05
E2	TKW	1B	AX.89735628	5.74E+07	0.20	1.17	3.32	0.06
E2	GY	1B	AX.89479897	5.96E+08	0.15	-3.00	3.79	0.05
E2	GY	1B	AX.89727207	5.94E+08	0.15	-2.98	3.84	0.05
E2	GY	1B	AX.89678168	6.62E+08	0.08	-4.00	3.82	0.05
E2	GY	1B	AX.89585146	5.94E+08	0.05	-4.84	3.78	0.05
E2	GY	1B	AX.89338879	6.62E+08	0.08	-4.00	3.82	0.05
E2	GY	1B	AX.89338879	6.62E+08	0.08	-4.00	3.82	0.05
E2	GY	1B	AX.89565905	6.59E+08	0.09	-3.38	3.26	0.04
E2	GY	1B	AX.89339365	6.62E+08	0.08	-4.00	3.82	0.05
E2	GY	1B	AX.89520284	5.76E+08	0.09	-3.23	3.04	0.04
E2	GY	1B	AX.89550273	5.94E+08	0.15	-2.98	3.84	0.05
E2	GY	1B	AX.89550273	5.94E+08	0.15	-2.98	3.84	0.05
E2	GY	1B	AX.89728530	5.96E+08	0.15	-3.00	3.79	0.05
E2	GY	1B	AX.89728530	5.96E+08	0.15	-3.00	3.79	0.05
E2	GY	1B	AX.89744679	6.65E+08	0.13	-3.02	3.42	0.07
E2	TKW	1B	AX.89753065	5.05E+07	0.18	1.22	3.35	0.06
E2	GY	1B	AX.89765882	6.46E+08	0.07	-4.54	4.34	0.06
E2	GY	1B	AX.89765882	6.46E+08	0.07	-4.54	4.34	0.06
E2	GY	1B	AX.89412705	5.95E+08	0.15	-3.00	3.79	0.05
E2	GY	1B	AX.89659812	5.94E+08	0.15	-2.74	3.25	0.04
E2	GY	1B	AX.89659812	5.94E+08	0.15	-2.74	3.25	0.04
E2	GPM2	1B	AX.89746393	6.62E+08	0.08	-919.40	3.20	0.04
E2	GY	1B	AX.89447503	6.62E+08	0.11	-3.22	3.34	0.04
E2	GY	1B	AX.89588545	6.59E+08	0.09	-3.38	3.26	0.04

E2	GY	1B	AX.89588545	6.59E+08	0.09	-3.38	3.26	0.04
E2	GY	1B	AX.89700186	6.65E+08	0.11	-3.63	4.06	0.09
E2	GY	1B	AX.89543657	5.96E+08	0.15	-3.00	3.79	0.05
E2	GY	1B	AX.89543657	5.96E+08	0.15	-3.00	3.79	0.05
E2	TKW	1B	AX.89675560	5.08E+07	0.38	0.98	3.38	0.06
E2	GPS	1B	AX.89499367	1.01E+07	0.47	1.35	3.13	0.04
E2	GPM2	1B	AX.89484512	6.62E+08	0.08	-919.40	3.20	0.04
E2	GY	1B	AX.89754639	5.95E+08	0.14	-2.75	3.20	0.04
E2	GY	1B	AX.89596577	5.95E+08	0.15	-3.00	3.79	0.05
E2	GY	1B	AX.89596577	5.95E+08	0.15	-3.00	3.79	0.05
E2	GY	1B	AX.89694069	6.54E+08	0.14	-2.72	3.05	0.06
E2	GY	1B	AX.89498745	5.76E+08	0.09	-3.23	3.04	0.04
E2	GY	1B	AX.89498745	5.76E+08	0.09	-3.23	3.04	0.04
E2	TKW	1B	AX.89722604	5.02E+07	0.15	1.29	3.28	0.06
E2	TKW	1B	AX.89451891	6.81E+08	0.19	1.19	3.29	0.06
E2	TKW	1B	AX.89674897	5.01E+07	0.34	0.94	3.06	0.05
E2	GY	1B	AX.89372089	6.62E+08	0.08	-4.00	3.82	0.05
E2	GY	1B	AX.89560184	6.60E+08	0.09	-3.38	3.26	0.04
E2	GY	1B	AX.89560184	6.60E+08	0.09	-3.38	3.26	0.04
E2	GPM2	1B	AX.89507799	6.66E+08	0.07	-1019.73	3.44	0.05
E2	GY	1B	AX.89678168	6.62E+08	0.08	-4.00	3.82	0.05
E2	GY	1B	AX.89585146	5.94E+08	0.05	-4.84	3.78	0.05
E2	GPM2	1B	AX.89416093	6.62E+08	0.08	-919.40	3.20	0.04
E2	GY	1B	AX.89504471	5.95E+08	0.15	-2.98	3.84	0.05
E2	GY	1B	AX.89744679	6.65E+08	0.13	-3.02	3.42	0.07
E2	GY	1B	AX.89599305	1.44E+07	0.49	-2.40	4.50	0.06
E2	GY	1B	AX.89599305	1.44E+07	0.49	-2.40	4.50	0.06
E2	GY	1B	AX.89412705	5.95E+08	0.15	-3.00	3.79	0.05
E2	GY	1B	AX.89447503	6.62E+08	0.11	-3.22	3.34	0.04
E2	TKW	1B	AX.89577603	5.01E+07	0.31	1.19	4.32	0.08
E2	GY	1B	AX.89512032	6.54E+08	0.16	-2.56	3.01	0.05
E2	GPM2	1B	AX.89511165	6.62E+08	0.08	-919.40	3.20	0.04
E2	TKW	1B	AX.89585935	5.05E+07	0.38	0.98	3.40	0.06
E2	GPM2	1B	AX.89546727	6.62E+08	0.08	-919.40	3.20	0.04
E2	GY	1B	AX.89608451	5.95E+08	0.15	-2.98	3.84	0.05
E2	TKW	1B	AX.89433211	5.03E+07	0.20	1.16	3.22	0.06
E2	TKW	1B	AX.89393759	5.01E+07	0.31	1.19	4.32	0.08
E2	TKW	1B	AX.89695396	5.10E+07	0.38	0.98	3.38	0.06
E2	GY	1B	AX.89516168	6.59E+08	0.09	-3.38	3.26	0.04
E2	TKW	1B	AX.89583076	4.91E+07	0.35	-0.93	3.01	0.05
E2	GPM2	1B	AX.89338879	6.62E+08	0.08	-919.40	3.20	0.04
E2	TKW	1B	AX.89507320	5.63E+07	0.24	1.13	3.51	0.06
E2	GY	1B	AX.89721599	5.94E+08	0.15	-3.00	3.79	0.05
E2	GY	1B	AX.89721599	5.94E+08	0.15	-3.00	3.79	0.05
E2	GY	1B	AX.89564115	5.76E+08	0.09	-3.23	3.04	0.04
E2	TKW	1B	AX.89409890	5.04E+07	0.18	1.22	3.35	0.06
E2	GY	1B	AX.89524034	6.46E+08	0.07	-4.54	4.34	0.06
E2	GY	1B	AX.89524034	6.46E+08	0.07	-4.54	4.34	0.06
E2	GY	1B	AX.89721228	6.62E+08	0.08	-4.00	3.82	0.05
E2	TKW	1B	AX.89492525	3.83E+08	0.47	0.91	3.11	0.05

E2	GPM2	1B	AX.89323934	6.66E+08	0.10	-824.38	3.18	0.08
E2	GY	1B	AX.89435723	6.66E+08	0.10	-3.77	4.16	0.09
E2	GY	1B	AX.89608451	5.95E+08	0.15	-2.98	3.84	0.05
E2	GY	1B	AX.89589298	5.95E+08	0.15	-3.00	3.79	0.05
E2	GY	1B	AX.89589298	5.95E+08	0.15	-3.00	3.79	0.05
E2	GY	1B	AX.89691473	5.96E+08	0.15	-3.00	3.79	0.05
E2	GPM2	1B	AX.89371246	6.81E+08	0.23	-603.97	3.28	0.05
E2	GPM2	1B	AX.89678168	6.62E+08	0.08	-919.40	3.20	0.04
E2	GY	1B	AX.89486405	5.94E+08	0.15	-3.00	3.79	0.05
E2	GY	1B	AX.89486405	5.94E+08	0.15	-3.00	3.79	0.05
E2	GPM2	1B	AX.89721228	6.62E+08	0.08	-919.40	3.20	0.04
E2	GY	1B	AX.89603516	6.59E+08	0.10	-3.21	3.11	0.04
E2	GY	1B	AX.89494044	5.94E+08	0.05	-4.84	3.78	0.05
E2	GPM2	1B	AX.89356982	6.54E+08	0.12	-734.05	3.04	0.06
E2	GPM2	1B	AX.89430295	6.54E+08	0.12	-734.05	3.04	0.06
E2	TKW	1B	AX.89686029	4.91E+07	0.13	1.33	3.10	0.06
E2	GY	1B	AX.89488197	5.94E+08	0.15	-2.98	3.84	0.05
E2	GY	1B	AX.89665857	5.74E+08	0.09	-3.23	3.04	0.04
E2	GPM2	1B	AX.89639158	6.62E+08	0.08	-919.40	3.20	0.04
E2	TKW	1B	AX.89319753	5.00E+07	0.48	-0.91	3.12	0.06
E2	GY	1B	AX.89692308	5.95E+08	0.15	-3.00	3.79	0.05
E2	GPM2	1B	AX.89402857	6.81E+08	0.33	-613.24	4.06	0.09
E2	TKW	1B	AX.89650808	5.03E+07	0.18	1.24	3.43	0.06
E2	GY	1B	AX.89494044	5.94E+08	0.05	-4.84	3.78	0.05
E2	TKW	1B	AX.89317304	5.09E+07	0.18	1.31	3.73	0.07
E2	GPM2	1B	AX.89395127	6.54E+08	0.11	-827.83	3.52	0.07
E2	GY	1B	AX.89665857	5.74E+08	0.09	-3.23	3.04	0.04
E2	TKW	1B	AX.89496797	6.81E+08	0.20	1.25	3.71	0.07
E2	GPM2	1B	AX.89314667	6.81E+08	0.23	-603.97	3.28	0.05
E2	TKW	1B	AX.89676980	5.02E+07	0.18	1.22	3.35	0.06
E2	TKW	1B	AX.89754637	5.01E+07	0.32	1.02	3.42	0.06
E2	TKW	1B	AX.89430979	5.02E+07	0.31	1.13	4.01	0.07
E2	GPM2	1B	AX.89580989	6.46E+08	0.07	-1052.91	3.68	0.06
E2	GPM2	1B	AX.89339365	6.62E+08	0.08	-919.40	3.20	0.04
E2	TKW	1B	AX.89541344	5.05E+07	0.36	1.08	3.91	0.07
E2	TKW	1B	AX.89718950	3.86E+07	0.21	-1.09	3.00	0.05
E2	TKW	1B	AX.89591367	4.97E+07	0.44	0.90	3.03	0.05
E2	GPM2	1B	AX.89485285	6.65E+08	0.11	-784.37	3.05	0.07
E2	TKW	1B	AX.89552620	5.01E+07	0.15	1.45	3.84	0.07
E2	TKW	1B	AX.89475250	5.50E+08	0.47	0.98	3.54	0.06
E2	GY	1B	AX.89692308	5.95E+08	0.15	-3.00	3.79	0.05
E2	GY	1B	AX.89603516	6.59E+08	0.10	-3.21	3.11	0.04
E2	GPM2	1B	AX.89655760	6.46E+08	0.07	-1052.91	3.68	0.06
E2	TKW	1B	AX.89431803	4.91E+07	0.12	1.43	3.22	0.06
E2	TKW	1B	AX.89681224	6.81E+08	0.20	1.35	4.25	0.08
E2	TKW	1B	AX.89731954	5.50E+08	0.46	0.96	3.43	0.05
E2	TKW	1B	AX.89750424	5.01E+07	0.15	1.29	3.21	0.06
E2	GY	1B	AX.89488197	5.94E+08	0.15	-2.98	3.84	0.05
E2	TKW	1B	AX.89432734	5.02E+07	0.18	1.40	4.20	0.08
E2	TKW	1B	AX.89753324	5.49E+08	0.49	0.90	3.05	0.05

E2	TKW	1B	AX.89773829	5.03E+07	0.18	1.31	3.73	0.07
E2	TKW	1B	AX.89360605	5.31E+07	0.22	1.08	3.05	0.05
E2	GPM2	1B	AX.89655332	6.65E+08	0.10	-824.38	3.18	0.08
E2	GPM2	1B	AX.89671741	6.81E+08	0.23	-670.40	3.94	0.06
E2	GPM2	1B	AX.89517676	6.81E+08	0.23	-632.00	3.55	0.05
E2	GPM2	1B	AX.89760192	6.62E+08	0.08	-960.20	3.28	0.05
E2	GPM2	1B	AX.89435723	6.66E+08	0.10	-824.38	3.18	0.08
E2	TKW	1B	AX.89514229	5.02E+07	0.34	0.94	3.06	0.05
E2	GPM2	1B	AX.89341296	6.54E+08	0.14	-708.90	3.09	0.06
E2	GPM2	1B	AX.89496797	6.81E+08	0.20	-703.81	3.88	0.06
E2	TKW	1B	AX.89487035	5.05E+07	0.18	1.35	4.00	0.07
E3	GPM2	1B	AX.89537486	6.71E+08	0.20	1044.95	3.17	0.04
E3	GPS	1B	AX.89349705	5.50E+07	0.21	-2.37	3.07	0.03
E3	GPS	1B	AX.89370171	5.32E+08	0.08	3.80	3.48	0.10
E3	GPS	1B	AX.89501274	5.32E+08	0.09	3.50	3.18	0.10
E3	GPS	1B	AX.89618894	5.32E+08	0.09	3.55	3.37	0.10
E3	GPS	1B	AX.89680637	5.89E+08	0.17	2.77	3.47	0.09
E3	GPS	1B	AX.89375029	5.89E+08	0.17	2.77	3.47	0.09
E3	GPS	1B	AX.89647569	5.91E+08	0.20	2.40	3.02	0.06
E3	GPS	1B	AX.89755321	6.14E+08	0.32	2.31	3.60	0.04
E3	GPS	1B	AX.89364192	6.14E+08	0.32	2.31	3.59	0.04
E3	GPS	1B	AX.89585539	6.14E+08	0.31	2.29	3.42	0.04
E3	GPS	1B	AX.89624410	6.17E+08	0.14	-3.03	3.34	0.03
E3	GPS	1B	AX.89463909	6.17E+08	0.14	-3.03	3.34	0.03
E3	GPS	1B	AX.89432343	6.19E+08	0.14	-3.03	3.34	0.03
E3	GPS	1B	AX.89705416	6.19E+08	0.32	2.79	4.95	0.10
E3	GPS	1B	AX.89642782	6.20E+08	0.32	2.79	4.95	0.10
E3	GPS	1B	AX.89711608	6.20E+08	0.33	2.78	4.95	0.10
E3	GPS	1B	AX.89680164	6.20E+08	0.32	2.79	4.95	0.10
E3	GPS	1B	AX.89515778	6.20E+08	0.14	-3.03	3.34	0.03
E3	GPS	1B	AX.89469991	6.20E+08	0.14	-2.95	3.13	0.02
E3	GPS	1B	AX.89612667	6.20E+08	0.33	2.72	4.85	0.10
E3	GPS	1B	AX.89679213	6.20E+08	0.32	2.83	5.04	0.10
E3	GPS	1B	AX.89686819	6.20E+08	0.33	2.65	4.64	0.10
E3	GPS	1B	AX.89513568	6.20E+08	0.34	2.65	4.63	0.10
E3	GPS	1B	AX.89634475	6.20E+08	0.33	2.74	4.82	0.10
E3	GPS	1B	AX.89583956	6.20E+08	0.32	2.72	4.72	0.10
E3	GPS	1B	AX.89485937	6.20E+08	0.33	2.72	4.85	0.10
E3	GPS	1B	AX.89422172	6.20E+08	0.32	2.72	4.72	0.10
E3	GPS	1B	AX.89574336	6.20E+08	0.32	2.72	4.72	0.10
E3	GPS	1B	AX.89745715	6.20E+08	0.14	-2.96	3.15	0.02
E3	GPS	1B	AX.89571267	6.21E+08	0.14	-2.96	3.15	0.02
E3	GPS	1B	AX.89377173	6.21E+08	0.33	2.67	4.56	0.10
E3	GPS	1B	AX.89377770	6.21E+08	0.32	2.79	4.95	0.10
E3	GPS	1B	AX.89457493	6.21E+08	0.15	-2.95	3.25	0.03
E3	GPS	1B	AX.89382491	6.21E+08	0.14	-2.96	3.15	0.02
E3	GPS	1B	AX.89496156	6.21E+08	0.16	-2.81	3.12	0.02
E3	GPS	1B	AX.89570740	6.21E+08	0.32	2.83	5.05	0.10
E3	GPS	1B	X.89753980_OT	6.24E+08	0.13	-2.95	3.08	0.02
E3	GPS	1B	X.89458278_OT	6.24E+08	0.33	2.20	3.62	0.08

E3	GPS	1B	AX.89416035	6.21E+08	0.32	2.79	4.95	0.10
E3	GPS	1B	AX.89332288	6.21E+08	0.35	2.76	4.98	0.10
E3	GPS	1B	AX.89409059	6.21E+08	0.35	2.81	5.12	0.10
E3	GPS	1B	AX.89474261	6.21E+08	0.14	-2.96	3.15	0.02
E3	GPS	1B	AX.89550297	6.21E+08	0.35	2.83	5.21	0.11
E3	GPS	1B	AX.89364111	6.21E+08	0.35	2.77	5.02	0.10
E3	GPS	1B	AX.89350105	6.21E+08	0.35	2.75	5.01	0.10
E3	GPS	1B	AX.89395125	6.21E+08	0.36	2.59	4.54	0.10
E3	GPS	1B	AX.89609292	6.22E+08	0.34	2.78	5.05	0.10
E3	GPS	1B	AX.89310700	6.22E+08	0.14	-2.87	3.05	0.03
E3	GPS	1B	AX.89628519	6.22E+08	0.34	2.79	5.11	0.10
E3	GPS	1B	AX.89374853	6.22E+08	0.35	2.83	5.19	0.10
E3	GPS	1B	AX.89757543	6.22E+08	0.34	2.78	5.05	0.10
E3	GPS	1B	AX.89757188	6.22E+08	0.35	2.63	4.63	0.09
E3	GPS	1B	AX.89680192	6.22E+08	0.35	2.71	4.84	0.10
E3	GPS	1B	AX.89569716	6.22E+08	0.36	2.66	4.76	0.10
E3	GPS	1B	AX.89774520	6.22E+08	0.35	2.71	4.84	0.10
E3	GPS	1B	AX.89358300	6.22E+08	0.35	2.71	4.84	0.10
E3	GPS	1B	AX.89763632	6.22E+08	0.14	-2.96	3.15	0.02
E3	GPS	1B	AX.89406155	6.22E+08	0.35	2.71	4.84	0.10
E3	GPS	1B	AX.89642257	6.22E+08	0.34	2.78	5.05	0.10
E3	GPS	1B	AX.89344176	6.22E+08	0.37	2.70	4.94	0.09
E3	GPS	1B	AX.89423711	6.22E+08	0.14	-2.96	3.15	0.02
E3	GPS	1B	AX.89417829	6.22E+08	0.14	-2.96	3.15	0.02
E3	GPS	1B	AX.89576725	6.22E+08	0.35	2.76	4.97	0.10
E3	GPS	1B	AX.89470808	6.22E+08	0.35	2.76	4.97	0.10
E3	GPS	1B	AX.89668518	6.22E+08	0.14	-2.96	3.15	0.02
E3	GPS	1B	AX.89416694	6.22E+08	0.36	2.81	5.16	0.10
E3	GPS	1B	AX.89763480	6.22E+08	0.35	2.76	4.97	0.10
E3	GPS	1B	AX.89543840	6.22E+08	0.34	2.94	5.42	0.12
E3	GPS	1B	AX.89700455	6.22E+08	0.36	2.80	5.15	0.11
E3	GPS	1B	AX.89528764	6.22E+08	0.36	2.79	5.08	0.10
E3	GPS	1B	AX.89490013	6.22E+08	0.35	2.77	4.98	0.10
E3	GPS	1B	AX.89514964	6.22E+08	0.23	2.51	3.61	0.05
E3	GPS	1B	AX.89489416	6.23E+08	0.23	2.51	3.61	0.05
E3	GPS	1B	AX.89322602	6.24E+08	0.33	2.10	3.31	0.07
E3	GPS	1B	AX.89618562	6.24E+08	0.33	2.10	3.31	0.07
E3	GY	1B	AX.89537486	6.71E+08	0.20	3.41	3.04	0.02
E3	SPM2	1B	X.89318798_OT	5.16E+07	0.34	-24.99	4.05	0.09
E3	SPM2	1B	AX.89702058	5.16E+07	0.34	-24.99	4.05	0.09
E3	SPM2	1B	X.89773040_OT	5.19E+07	0.32	-25.66	4.14	0.09
E3	SPM2	1B	X.89481755_OT	5.19E+07	0.34	-24.99	4.05	0.09
E3	SPM2	1B	X.89589121_OT	5.19E+07	0.34	-24.99	4.05	0.09
E3	SPM2	1B	X.89756461_OT	5.19E+07	0.34	-24.76	4.00	0.08
E3	SPM2	1B	X.89667444_OT	5.20E+07	0.34	-24.99	4.05	0.09
E3	SPM2	1B	X.89704317_OT	5.20E+07	0.36	-23.66	3.87	0.08
E3	SPM2	1B	X.89461216_OT	5.22E+07	0.34	-24.99	4.05	0.09
E3	SPM2	1B	X.89691364_OT	5.22E+07	0.33	-24.82	3.98	0.08
E3	SPM2	1B	X.89599251_OT	5.23E+07	0.34	-24.99	4.05	0.09
E3	SPM2	1B	X.89683808_OT	5.23E+07	0.34	-24.99	4.05	0.09

E3	SPM2	1B	X.89364263_OT	5.23E+07	0.34	-24.99	4.05	0.09
E3	SPM2	1B	X.89310818_OT	5.23E+07	0.33	-25.21	4.11	0.09
E3	SPM2	1B	X.89648931_OT	5.23E+07	0.34	-24.99	4.05	0.09
E3	SPM2	1B	AX.89753721	5.26E+07	0.35	-21.01	3.06	0.06
E3	SPM2	1B	AX.89489428	5.34E+07	0.34	-24.76	4.00	0.08
E3	SPM2	1B	X.89446477_OT	5.71E+07	0.32	-22.16	3.14	0.07
E3	SPM2	1B	AX.89761773	5.74E+07	0.37	-24.74	4.15	0.08
E3	SPM2	1B	AX.89582634	5.74E+07	0.29	-20.73	3.06	0.06
E3	SPM2	1B	X.89533360_OT	6.54E+07	0.24	-25.25	3.14	0.07
E3	SPM2	1B	X.89464809_OT	6.55E+07	0.23	-28.30	3.77	0.08
E3	SPM2	1B	X.89645031_OT	6.61E+07	0.22	-25.74	3.18	0.07
E3	SPM2	1B	X.89640230_OT	9.77E+07	0.25	-25.15	3.15	0.07
E3	SPM2	1B	AX.89479802	1.38E+08	0.28	-23.10	3.01	0.06
E3	SPM2	1B	AX.89310005	1.64E+08	0.34	-21.50	3.01	0.06
E3	SPM2	1B	AX.89449853	1.67E+08	0.33	-22.46	3.24	0.06
E3	SPM2	1B	AX.89537822	1.69E+08	0.28	-22.94	3.03	0.07
E3	SPM2	1B	AX.89459844	1.69E+08	0.32	-22.98	3.25	0.07
E3	SPM2	1B	AX.89324778	1.70E+08	0.26	-24.59	3.24	0.07
E3	SPM2	1B	X.89466066_OT	1.94E+08	0.26	-25.03	3.20	0.07
E3	SPM2	1B	AX.89321267	6.60E+08	0.30	-23.74	3.15	0.06
E3	SPM2	1B	X.89450981_OT	3.56E+07	0.24	-26.27	3.40	0.07
E3	SPM2	1B	X.89390904_OT	3.65E+07	0.25	-24.52	3.05	0.07
E3	SPM2	1B	X.89746756_OT	3.65E+07	0.25	-24.52	3.05	0.07
E3	SPM2	1B	X.89377136_OT	3.65E+07	0.25	-24.52	3.05	0.07
E3	SPM2	1B	X.89527145_OT	3.66E+07	0.25	-24.52	3.05	0.07
E3	SPM2	1B	X.89657943_OT	3.67E+07	0.25	-24.52	3.05	0.07
E3	SPM2	1B	X.89309504_OT	3.68E+07	0.25	-24.52	3.05	0.07
E3	SPM2	1B	X.89776118_OT	3.68E+07	0.25	-24.52	3.05	0.07
E3	SPM2	1B	X.89354141_OT	3.68E+07	0.25	-24.52	3.05	0.07
E3	SPM2	1B	X.89348412_OT	3.68E+07	0.25	-24.52	3.05	0.07
E3	SPM2	1B	X.89441583_OT	3.68E+07	0.25	-24.52	3.05	0.07
E3	SPM2	1B	X.89527415_OT	3.68E+07	0.25	-24.52	3.05	0.07
E3	SPM2	1B	X.89459505_OT	3.68E+07	0.25	-24.34	3.03	0.07
E3	SPM2	1B	X.89736482_OT	3.68E+07	0.25	-24.25	3.02	0.06
E3	SPM2	1B	X.89555520_OT	3.70E+07	0.25	-24.34	3.03	0.07
E3	SPM2	1B	X.89665456_OT	3.70E+07	0.25	-25.08	3.21	0.07
E3	SPM2	1B	X.89462065_OT	3.70E+07	0.25	-24.52	3.05	0.07
E3	SPM2	1B	X.89676446_OT	3.70E+07	0.25	-24.52	3.05	0.07
E3	SPM2	1B	X.89427367_OT	3.72E+07	0.25	-24.52	3.05	0.07
E3	SPM2	1B	X.89659027_OT	3.75E+07	0.25	-24.52	3.05	0.07
E3	SPM2	1B	X.89325142_OT	3.76E+07	0.25	-24.52	3.05	0.07
E3	SPM2	1B	X.89454412_OT	3.77E+07	0.25	-24.34	3.03	0.07
E3	SPM2	1B	X.89525939_OT	3.80E+07	0.25	-25.32	3.19	0.07
E3	SPM2	1B	X.89416990_OT	3.81E+07	0.21	-27.89	3.51	0.08
E3	SPM2	1B	X.89473923_OT	4.83E+07	0.24	-25.72	3.16	0.07
E3	SPM2	1B	X.89765155_OT	5.16E+07	0.43	-22.71	3.76	0.07
E3	TKW	1B	X.89507565_OT	1.84E+07	0.15	1.49	3.75	0.08
E3	TKW	1B	X.89404982_OT	2.64E+07	0.26	1.03	3.14	0.06
E3	TKW	1B	X.89486980_OT	1.14E+08	0.15	1.27	3.14	0.07
E3	SPM2	1B	X.89738384_OT	5.14E+06	0.26	-25.70	3.39	0.06

E3	SPM2	1B	X.89580023_OT	6.55E+06	0.32	-22.11	3.14	0.06
E3	SPM2	1B	X.89488881_OT	6.56E+06	0.32	-22.11	3.14	0.06
E3	SPM2	1B	X.89674065_OT	6.56E+06	0.31	-22.12	3.11	0.06
E3	SPM2	1B	X.89348101_OT	6.58E+06	0.31	-22.12	3.11	0.06
E3	SPM2	1B	AX.89688551	8.25E+06	0.26	-25.09	3.30	0.07
E3	SPM2	1B	AX.89323670	8.27E+06	0.28	-23.11	3.13	0.07
E3	SPM2	1B	AX.89546305	8.40E+06	0.29	-23.54	3.30	0.07
E3	SPM2	1B	X.89416766_OT	1.89E+07	0.33	22.03	3.47	0.07
E3	SPM2	1B	X.89600865_OT	2.67E+07	0.26	-24.06	3.01	0.06
E3	SPM2	1B	X.89468663_OT	2.85E+07	0.27	-24.07	3.18	0.06
E3	SPM2	1B	X.89416192_OT	2.85E+07	0.26	-24.89	3.22	0.07
E3	SPM2	1B	X.89508290_OT	2.85E+07	0.27	-24.07	3.18	0.06
E3	SPM2	1B	AX.89426987	2.88E+07	0.26	-23.37	3.04	0.06
E3	SPM2	1B	AX.89671114	2.88E+07	0.28	-23.54	3.12	0.06
E3	SPM2	1B	X.89394980_OT	2.93E+07	0.26	-23.70	3.02	0.06
E3	SPM2	1B	X.89492098_OT	2.93E+07	0.26	-24.56	3.20	0.06
E3	SPM2	1B	X.89375518_OT	2.94E+07	0.26	-23.70	3.02	0.06
E3	SPM2	1B	X.89506722_OT	3.24E+07	0.25	-24.52	3.05	0.07
E3	SPM2	1B	X.89645937_OT	3.24E+07	0.25	-24.52	3.05	0.07
E3	SPM2	1B	X.89681477_OT	3.25E+07	0.25	-24.52	3.05	0.07
E3	SPM2	1B	X.89391511_OT	3.25E+07	0.26	-26.19	3.51	0.08
E3	SPM2	1B	X.89751234_OT	3.25E+07	0.25	-24.52	3.05	0.07
E3	SPM2	1B	X.89465384_OT	3.26E+07	0.25	-25.41	3.30	0.07
E3	SPM2	1B	AX.89680559	3.30E+07	0.25	-24.78	3.15	0.07
E3	SPM2	1B	X.89660883_OT	3.40E+07	0.25	-24.52	3.05	0.07
E3	SPM2	1B	X.89471866_OT	3.42E+07	0.26	-24.09	3.01	0.07
E3	SPM2	1B	X.89391014_OT	3.50E+07	0.25	-24.52	3.05	0.07
E3	SPM2	1B	X.89745162_OT	3.51E+07	0.25	-24.52	3.05	0.07
E3	SPM2	1B	X.89588909_OT	3.51E+07	0.25	-27.34	3.67	0.08
E3	SPM2	1B	X.89744711_OT	3.52E+07	0.25	-24.52	3.05	0.07
E4	GPM2	1B	AX.89610899	6.64E+08	0.13	-731.99	3.83	0.05
E4	GPM2	1B	AX.89690244	6.64E+08	0.13	-731.46	3.99	0.05
E4	GPM2	1B	AX.89654832	6.64E+08	0.14	-670.90	3.50	0.05
E4	GPM2	1B	AX.89764133	6.64E+08	0.12	-718.30	3.62	0.05
E4	GPM2	1B	AX.89496442	6.64E+08	0.14	-670.90	3.50	0.05
E4	GPM2	1B	AX.89522737	6.63E+08	0.13	-693.81	3.48	0.04
E4	GPM2	1B	AX.89544787	6.63E+08	0.13	-693.81	3.48	0.04
E4	GPM2	1B	AX.89571674	6.63E+08	0.14	-657.04	3.36	0.04
E4	GPM2	1B	AX.89684463	6.63E+08	0.15	-750.12	4.55	0.05
E4	GPM2	1B	AX.89536505	6.64E+08	0.14	-670.90	3.50	0.05
E4	GPM2	1B	AX.89503681	6.63E+08	0.13	-693.81	3.48	0.04
E4	GPM2	1B	AX.89388459	6.63E+08	0.13	-693.81	3.48	0.04
E4	GPM2	1B	AX.89499334	6.63E+08	0.13	-693.81	3.48	0.04
E4	GPM2	1B	AX.89478088	6.63E+08	0.13	-731.46	3.99	0.05
E4	GPM2	1B	AX.89366113	6.63E+08	0.14	-657.04	3.36	0.04
E4	GPS	1B	AX.89630061	1.56E+07	0.39	-1.57	3.30	0.02
E4	GPS	1B	AX.89399435	1.64E+07	0.46	-1.63	3.46	0.03
E4	GPS	1B	AX.89739610	1.74E+07	0.45	-1.55	3.34	0.06
E4	GPS	1B	AX.89412325	5.31E+08	0.09	2.71	3.57	0.07
E4	GPS	1B	AX.89643416	5.32E+08	0.10	2.58	3.50	0.07

E4	GPS	1B	AX.89332980	5.32E+08	0.09	2.71	3.57	0.07
E4	GPS	1B	AX.89370171	5.32E+08	0.08	3.04	4.07	0.08
E4	GPS	1B	AX.89501274	5.32E+08	0.09	2.94	3.99	0.08
E4	GPS	1B	AX.89683118	5.32E+08	0.08	3.07	3.93	0.07
E4	GPS	1B	AX.89618894	5.32E+08	0.10	3.04	4.36	0.08
E4	GPS	1B	AX.89733972	6.25E+08	0.11	2.45	3.47	0.07
E4	GY	1B	AX.89575996	6.54E+08	0.28	-1.67	3.16	0.05
E4	GY	1B	X.89638589_OT	6.54E+08	0.22	-1.78	3.11	0.05
E4	GY	1B	AX.89522737	6.63E+08	0.13	-2.52	3.94	0.06
E4	GY	1B	AX.89503681	6.63E+08	0.13	-2.52	3.94	0.06
E4	GY	1B	AX.89388459	6.63E+08	0.13	-2.52	3.94	0.06
E4	GY	1B	AX.89499334	6.63E+08	0.13	-2.52	3.94	0.06
E4	GY	1B	AX.89544787	6.63E+08	0.13	-2.52	3.94	0.06
E4	GY	1B	AX.89571674	6.63E+08	0.14	-2.28	3.52	0.06
E4	GY	1B	AX.89684463	6.63E+08	0.15	-2.65	4.89	0.08
E4	GY	1B	AX.89366113	6.63E+08	0.14	-2.28	3.52	0.06
E4	GY	1B	AX.89478088	6.63E+08	0.14	-2.52	4.12	0.07
E4	GY	1B	AX.89536505	6.64E+08	0.14	-2.26	3.50	0.06
E4	GY	1B	AX.89690244	6.64E+08	0.14	-2.52	4.12	0.07
E4	GY	1B	AX.89496442	6.64E+08	0.14	-2.26	3.50	0.06
E4	GY	1B	AX.89654832	6.64E+08	0.14	-2.26	3.50	0.06
E4	GY	1B	AX.89764133	6.64E+08	0.13	-2.59	4.07	0.07
E4	GY	1B	AX.89610899	6.64E+08	0.13	-2.57	4.11	0.07
E4	GY	1B	AX.89473185	6.68E+08	0.07	-2.79	3.03	0.04
E4	GY	1B	AX.89661321	6.68E+08	0.07	-2.93	3.44	0.05
E4	GY	1B	AX.89405777	6.68E+08	0.07	-2.93	3.44	0.05
E4	GY	1B	AX.89650769	6.68E+08	0.07	-2.79	3.15	0.05
E4	GY	1B	AX.89621403	6.68E+08	0.09	-2.81	3.75	0.05
E4	GY	1B	AX.89462134	6.87E+08	0.11	-2.59	3.53	0.05
E1	GY	1D	AX.89485389	8.10E+06	0.06	-4.98	3.09	0.06
E1	GY	1D	AX.89381613	8.06E+06	0.06	-4.98	3.09	0.06
E1	GY	1D	AX.89496589	8.10E+06	0.06	-4.98	3.09	0.06
E1	GY	1D	AX.89392468	7.90E+06	0.06	-5.19	3.32	0.06
E1	GY	1D	AX.89472889	8.19E+06	0.06	-4.98	3.09	0.06
E1	GY	1D	AX.89496589	8.10E+06	0.06	-4.98	3.09	0.06
E1	GY	1D	AX.89333643	7.92E+06	0.06	-4.74	3.04	0.06
E1	GY	1D	AX.89392468	7.90E+06	0.06	-5.19	3.32	0.06
E1	GY	1D	AX.89590687	7.91E+06	0.06	-4.74	3.04	0.06
E1	GY	1D	AX.89477144	7.94E+06	0.06	-4.74	3.04	0.06
E1	GY	1D	AX.89331550	8.20E+06	0.06	-4.98	3.09	0.06
E1	GY	1D	AX.89472889	8.19E+06	0.06	-4.98	3.09	0.06
E1	GY	1D	AX.89407891	7.95E+06	0.05	-5.45	3.37	0.07
E1	GY	1D	AX.89379975	7.95E+06	0.06	-4.98	3.09	0.06
E1	GY	1D	AX.89379975	7.95E+06	0.06	-4.98	3.09	0.06
E1	GY	1D	AX.89333643	7.92E+06	0.06	-4.74	3.04	0.06
E1	GY	1D	AX.89696945	7.90E+06	0.06	-5.19	3.32	0.06
E1	GY	1D	AX.89696945	7.90E+06	0.06	-5.19	3.32	0.06
E1	GY	1D	AX.89753904	7.92E+06	0.06	-4.74	3.04	0.06
E1	GY	1D	AX.89531175	8.20E+06	0.06	-4.98	3.09	0.06
E1	GY	1D	AX.89417249	8.02E+06	0.06	-4.74	3.04	0.06

E1	GY	1D	AX.89381613	8.06E+06	0.06	-4.98	3.09	0.06
E1	GY	1D	AX.89618537	3.35E+06	0.05	-6.33	4.37	0.07
E1	GY	1D	AX.89407891	7.95E+06	0.05	-5.45	3.37	0.07
E1	GY	1D	AX.89375427	8.10E+06	0.06	-4.98	3.09	0.06
E1	GY	1D	AX.89687966	7.92E+06	0.06	-4.74	3.04	0.06
E1	GY	1D	AX.89531175	8.20E+06	0.06	-4.98	3.09	0.06
E1	GY	1D	AX.89590687	7.91E+06	0.06	-4.74	3.04	0.06
E1	GY	1D	AX.89610470	7.92E+06	0.06	-4.74	3.04	0.06
E1	GY	1D	AX.89494956	7.99E+06	0.05	-5.68	3.61	0.07
E1	GY	1D	AX.89494700	7.92E+06	0.06	-4.74	3.04	0.06
E1	GY	1D	AX.89501415	8.02E+06	0.06	-4.98	3.09	0.06
E1	GY	1D	AX.89641164	8.02E+06	0.06	-4.98	3.09	0.06
E1	GY	1D	AX.89467842	8.04E+06	0.06	-4.98	3.09	0.06
E1	GY	1D	AX.89467842	8.04E+06	0.06	-4.98	3.09	0.06
E1	GY	1D	AX.89676569	8.02E+06	0.06	-4.98	3.09	0.06
E1	GY	1D	AX.89331550	8.20E+06	0.06	-4.98	3.09	0.06
E1	GY	1D	AX.89651440	8.19E+06	0.06	-4.98	3.09	0.06
E1	GY	1D	AX.89428720	7.92E+06	0.06	-4.74	3.04	0.06
E1	GY	1D	AX.89501415	8.02E+06	0.06	-4.98	3.09	0.06
E1	GY	1D	AX.89572294	7.93E+06	0.06	-4.74	3.04	0.06
E1	GY	1D	AX.89620645	7.99E+06	0.06	-4.98	3.09	0.06
E1	GY	1D	AX.89632594	7.95E+06	0.06	-4.98	3.09	0.06
E1	GY	1D	AX.89516494	7.95E+06	0.06	-4.74	3.04	0.06
E1	GY	1D	AX.89485389	8.10E+06	0.06	-4.98	3.09	0.06
E1	GY	1D	AX.89417249	8.02E+06	0.06	-4.74	3.04	0.06
E1	GY	1D	AX.89715211	8.04E+06	0.06	-4.98	3.09	0.06
E1	GY	1D	AX.89489677	7.95E+06	0.06	-4.98	3.09	0.06
E1	GY	1D	AX.89477144	7.94E+06	0.06	-4.74	3.04	0.06
E1	GY	1D	AX.89516494	7.95E+06	0.06	-4.74	3.04	0.06
E1	GY	1D	AX.89690807	8.04E+06	0.06	-4.98	3.09	0.06
E1	GY	1D	AX.89687966	7.92E+06	0.06	-4.74	3.04	0.06
E1	GY	1D	AX.89753904	7.92E+06	0.06	-4.74	3.04	0.06
E1	GY	1D	AX.89697899	8.02E+06	0.06	-4.98	3.09	0.06
E1	GY	1D	AX.89428720	7.92E+06	0.06	-4.74	3.04	0.06
E1	GY	1D	AX.89726393	7.98E+06	0.06	-4.98	3.09	0.06
E1	GY	1D	AX.89572294	7.93E+06	0.06	-4.74	3.04	0.06
E1	GY	1D	AX.89744435	7.92E+06	0.06	-4.74	3.04	0.06
E1	GY	1D	AX.89744435	7.92E+06	0.06	-4.74	3.04	0.06
E1	GY	1D	AX.89683472	7.94E+06	0.06	-4.74	3.04	0.06
E1	GY	1D	AX.89683472	7.94E+06	0.06	-4.74	3.04	0.06
E1	GY	1D	AX.89676569	8.02E+06	0.06	-4.98	3.09	0.06
E1	GY	1D	AX.89651440	8.19E+06	0.06	-4.98	3.09	0.06
E1	GY	1D	AX.89715211	8.04E+06	0.06	-4.98	3.09	0.06
E1	GY	1D	AX.89522651	7.92E+06	0.06	-4.74	3.04	0.06
E1	GY	1D	AX.89522651	7.92E+06	0.06	-4.74	3.04	0.06
E1	GY	1D	AX.89489677	7.95E+06	0.06	-4.98	3.09	0.06
E1	GY	1D	AX.89494700	7.92E+06	0.06	-4.74	3.04	0.06
E1	GY	1D	AX.89641164	8.02E+06	0.06	-4.98	3.09	0.06
E1	GY	1D	AX.89620645	7.99E+06	0.06	-4.98	3.09	0.06
E1	GY	1D	AX.89690807	8.04E+06	0.06	-4.98	3.09	0.06

E1	GPM2	1D	AX.89772918	1.23E+07	0.07	-1182.47	3.24	0.06
E1	GY	1D	AX.89698093	7.95E+06	0.06	-4.98	3.09	0.06
E1	GY	1D	AX.89483393	7.95E+06	0.06	-4.74	3.04	0.06
E1	GY	1D	AX.89610470	7.92E+06	0.06	-4.74	3.04	0.06
E1	GY	1D	AX.89726393	7.98E+06	0.06	-4.98	3.09	0.06
E1	GY	1D	AX.89494956	7.99E+06	0.05	-5.68	3.61	0.07
E1	GY	1D	AX.89632594	7.95E+06	0.06	-4.98	3.09	0.06
E1	GPM2	1D	AX.89471059	1.23E+07	0.07	-1182.47	3.24	0.06
E1	GY	1D	AX.89375427	8.10E+06	0.06	-4.98	3.09	0.06
E1	TKW	1D	AX.89342604	4.51E+08	0.31	-0.95	3.32	0.06
E1	GY	1D	AX.89618537	3.35E+06	0.05	-6.33	4.37	0.07
E1	GY	1D	AX.89698093	7.95E+06	0.06	-4.98	3.09	0.06
E1	GY	1D	AX.89483393	7.95E+06	0.06	-4.74	3.04	0.06
E1	GY	1D	AX.89697899	8.02E+06	0.06	-4.98	3.09	0.06
E2	SPM2	1D	AX.89599278	2.88E+08	0.23	-13.90	3.05	0.06
E2	SPM2	1D	AX.89704089	9.88E+07	0.33	-13.11	3.33	0.06
E2	SPM2	1D	AX.89376391	3.14E+08	0.38	13.13	3.55	0.06
E2	SPM2	1D	AX.89446789	9.30E+07	0.33	-13.11	3.33	0.06
E2	GY	1D	AX.89683472	7.94E+06	0.06	-4.64	3.99	0.08
E2	GY	1D	AX.89364350	1.20E+07	0.07	-4.23	3.98	0.09
E2	SPM2	1D	AX.89438351	2.17E+08	0.33	-12.67	3.18	0.06
E2	SPM2	1D	AX.89377994	4.80E+08	0.48	-11.81	3.11	0.05
E2	SPM2	1D	AX.89314486	2.19E+08	0.40	-11.91	3.04	0.05
E2	SPM2	1D	AX.89659489	8.82E+07	0.24	-13.70	3.08	0.06
E2	SPM2	1D	AX.89333901	2.75E+07	0.26	-15.25	3.86	0.07
E2	GY	1D	AX.89564539	1.21E+07	0.09	-3.83	4.02	0.09
E2	GY	1D	AX.89494956	7.99E+06	0.05	-5.99	5.49	0.11
E2	GY	1D	AX.89494956	7.99E+06	0.05	-5.99	5.49	0.11
E2	GY	1D	AX.89531175	8.20E+06	0.06	-4.85	4.03	0.09
E2	SPM2	1D	AX.89326264	1.25E+07	0.06	-27.84	3.80	0.06
E2	SPM2	1D	AX.89401440	9.30E+07	0.24	-13.70	3.08	0.06
E2	GY	1D	AX.89402917	7.93E+06	0.05	-5.17	3.90	0.08
E2	SPM2	1D	AX.89698237	2.02E+08	0.31	-12.98	3.21	0.06
E2	GY	1D	AX.89651440	8.19E+06	0.06	-4.85	4.03	0.09
E2	GY	1D	AX.89333643	7.92E+06	0.06	-4.64	3.99	0.08
E2	GY	1D	AX.89333643	7.92E+06	0.06	-4.64	3.99	0.08
E2	SPM2	1D	AX.89420601	2.03E+08	0.24	-13.78	3.11	0.06
E2	SPM2	1D	AX.89527033	2.01E+08	0.33	-12.36	3.05	0.05
E2	SPM2	1D	AX.89598945	8.52E+07	0.25	-14.19	3.34	0.06
E2	SPM2	1D	AX.89548480	8.04E+07	0.26	-14.47	3.49	0.07
E2	SPM2	1D	AX.89347242	2.60E+08	0.30	-13.38	3.33	0.06
E2	GY	1D	AX.89632594	7.95E+06	0.06	-4.85	4.03	0.09
E2	SPM2	1D	AX.89609489	5.71E+07	0.26	-14.17	3.37	0.06
E2	GPM2	1D	AX.89632594	7.95E+06	0.06	-1066.96	3.12	0.07
E2	SPM2	1D	AX.89669847	4.79E+08	0.46	-13.36	3.83	0.07
E2	SPM2	1D	AX.89561821	1.77E+08	0.26	-13.48	3.09	0.06
E2	SPM2	1D	AX.89732763	1.65E+08	0.26	-13.48	3.09	0.06
E2	SPM2	1D	AX.89412954	8.82E+07	0.30	-13.80	3.51	0.07
E2	GY	1D	AX.89375427	8.10E+06	0.06	-4.85	4.03	0.09
E2	GY	1D	AX.89375427	8.10E+06	0.06	-4.85	4.03	0.09

E2	GY	1D	AX.89331550	8.20E+06	0.06	-4.85	4.03	0.09
E2	GY	1D	AX.89331550	8.20E+06	0.06	-4.85	4.03	0.09
E2	GY	1D	AX.89516494	7.95E+06	0.06	-4.64	3.99	0.08
E2	GY	1D	AX.89516494	7.95E+06	0.06	-4.64	3.99	0.08
E2	GY	1D	AX.89322053	1.23E+07	0.06	-4.22	3.43	0.07
E2	GY	1D	AX.89709549	1.21E+07	0.10	-3.34	3.31	0.07
E2	GY	1D	AX.89744435	7.92E+06	0.06	-4.64	3.99	0.08
E2	GY	1D	AX.89744435	7.92E+06	0.06	-4.64	3.99	0.08
E2	GY	1D	AX.89590687	7.91E+06	0.06	-4.64	3.99	0.08
E2	GY	1D	AX.89590687	7.91E+06	0.06	-4.64	3.99	0.08
E2	GY	1D	AX.89687966	7.92E+06	0.06	-4.64	3.99	0.08
E2	GY	1D	AX.89687966	7.92E+06	0.06	-4.64	3.99	0.08
E2	GY	1D	AX.89379975	7.95E+06	0.06	-4.85	4.03	0.09
E2	GY	1D	AX.89379975	7.95E+06	0.06	-4.85	4.03	0.09
E2	GY	1D	AX.89489677	7.95E+06	0.06	-4.85	4.03	0.09
E2	GY	1D	AX.89489677	7.95E+06	0.06	-4.85	4.03	0.09
E2	GY	1D	AX.89451469	1.23E+07	0.10	-3.34	3.31	0.07
E2	GY	1D	AX.89451469	1.23E+07	0.10	-3.34	3.31	0.07
E2	GY	1D	AX.89334129	1.23E+07	0.10	-3.34	3.31	0.07
E2	GY	1D	AX.89334129	1.23E+07	0.10	-3.34	3.31	0.07
E2	GY	1D	AX.89715211	8.04E+06	0.06	-4.85	4.03	0.09
E2	GY	1D	AX.89381613	8.06E+06	0.06	-4.85	4.03	0.09
E2	GY	1D	AX.89381613	8.06E+06	0.06	-4.85	4.03	0.09
E2	GPM2	1D	AX.89620645	7.99E+06	0.06	-1066.96	3.12	0.07
E2	GY	1D	AX.89620645	7.99E+06	0.06	-4.85	4.03	0.09
E2	GY	1D	AX.89620645	7.99E+06	0.06	-4.85	4.03	0.09
E2	GY	1D	AX.89456181	1.23E+07	0.10	-3.34	3.31	0.07
E2	GY	1D	AX.89456181	1.23E+07	0.10	-3.34	3.31	0.07
E2	GY	1D	AX.89467842	8.04E+06	0.06	-4.85	4.03	0.09
E2	GY	1D	AX.89467842	8.04E+06	0.06	-4.85	4.03	0.09
E2	GY	1D	AX.89386345	1.23E+07	0.10	-3.34	3.31	0.07
E2	GY	1D	AX.89386345	1.23E+07	0.10	-3.34	3.31	0.07
E2	GY	1D	AX.89364350	1.20E+07	0.07	-4.23	3.98	0.09
E2	GPM2	1D	AX.89641164	8.02E+06	0.06	-1066.96	3.12	0.07
E2	GY	1D	AX.89483393	7.95E+06	0.06	-4.64	3.99	0.08
E2	GY	1D	AX.89483393	7.95E+06	0.06	-4.64	3.99	0.08
E2	GY	1D	AX.89485389	8.10E+06	0.06	-4.85	4.03	0.09
E2	GY	1D	AX.89485389	8.10E+06	0.06	-4.85	4.03	0.09
E2	GY	1D	AX.89760074	1.21E+07	0.11	-4.12	5.10	0.10
E2	GY	1D	AX.89402917	7.93E+06	0.05	-5.17	3.90	0.08
E2	GY	1D	AX.89322053	1.23E+07	0.06	-4.22	3.43	0.07
E2	GY	1D	AX.89709549	1.21E+07	0.10	-3.34	3.31	0.07
E2	GY	1D	AX.89690807	8.04E+06	0.06	-4.85	4.03	0.09
E2	GY	1D	AX.89690807	8.04E+06	0.06	-4.85	4.03	0.09
E2	GPM2	1D	AX.89715211	8.04E+06	0.06	-1066.96	3.12	0.07
E2	GY	1D	AX.89392468	7.90E+06	0.06	-5.37	4.82	0.10
E2	GY	1D	AX.89392468	7.90E+06	0.06	-5.37	4.82	0.10
E2	GY	1D	AX.89428720	7.92E+06	0.06	-4.64	3.99	0.08
E2	GY	1D	AX.89428720	7.92E+06	0.06	-4.64	3.99	0.08
E2	GY	1D	AX.89715211	8.04E+06	0.06	-4.85	4.03	0.09

E2	GY	1D	AX.89472889	8.19E+06	0.06	-4.85	4.03	0.09
E2	GY	1D	AX.89472889	8.19E+06	0.06	-4.85	4.03	0.09
E2	GPS	1D	AX.89598295	7.89E+07	0.27	1.53	3.21	0.05
E2	GY	1D	AX.89683472	7.94E+06	0.06	-4.64	3.99	0.08
E2	GPM2	1D	AX.89472889	8.19E+06	0.06	-1066.96	3.12	0.07
E2	GPM2	1D	AX.89531175	8.20E+06	0.06	-1066.96	3.12	0.07
E2	GY	1D	AX.89564539	1.21E+07	0.09	-3.83	4.02	0.09
E2	GY	1D	AX.89531175	8.20E+06	0.06	-4.85	4.03	0.09
E2	GPM2	1D	AX.89407891	7.95E+06	0.05	-1123.31	3.20	0.07
E2	GY	1D	AX.89651440	8.19E+06	0.06	-4.85	4.03	0.09
E2	GY	1D	AX.89697899	8.02E+06	0.06	-4.85	4.03	0.09
E2	GY	1D	AX.89632594	7.95E+06	0.06	-4.85	4.03	0.09
E2	GY	1D	AX.89407891	7.95E+06	0.05	-5.44	4.62	0.09
E2	GY	1D	AX.89407891	7.95E+06	0.05	-5.44	4.62	0.09
E2	GY	1D	AX.89753904	7.92E+06	0.06	-4.64	3.99	0.08
E2	GY	1D	AX.89762961	1.23E+07	0.10	-3.34	3.31	0.07
E2	GY	1D	AX.89676569	8.02E+06	0.06	-4.85	4.03	0.09
E2	GPM2	1D	AX.89392468	7.90E+06	0.06	-1071.88	3.15	0.07
E2	GY	1D	AX.89610470	7.92E+06	0.06	-4.64	3.99	0.08
E2	GY	1D	AX.89610470	7.92E+06	0.06	-4.64	3.99	0.08
E2	GPS	1D	AX.89333901	2.75E+07	0.26	1.76	3.98	0.06
E2	GY	1D	AX.89496589	8.10E+06	0.06	-4.85	4.03	0.09
E2	GY	1D	AX.89697899	8.02E+06	0.06	-4.85	4.03	0.09
E2	GY	1D	AX.89494700	7.92E+06	0.06	-4.64	3.99	0.08
E2	GY	1D	AX.89494700	7.92E+06	0.06	-4.64	3.99	0.08
E2	GY	1D	AX.89417249	8.02E+06	0.06	-4.64	3.99	0.08
E2	GY	1D	AX.89417249	8.02E+06	0.06	-4.64	3.99	0.08
E2	GY	1D	AX.89641164	8.02E+06	0.06	-4.85	4.03	0.09
E2	GY	1D	AX.89477144	7.94E+06	0.06	-4.64	3.99	0.08
E2	GY	1D	AX.89477144	7.94E+06	0.06	-4.64	3.99	0.08
E2	GY	1D	AX.89570610	1.23E+07	0.09	-3.83	4.02	0.09
E2	GY	1D	AX.89570610	1.23E+07	0.09	-3.83	4.02	0.09
E2	GY	1D	AX.89726393	7.98E+06	0.06	-4.85	4.03	0.09
E2	GY	1D	AX.89726393	7.98E+06	0.06	-4.85	4.03	0.09
E2	GY	1D	AX.89753904	7.92E+06	0.06	-4.64	3.99	0.08
E2	GY	1D	AX.89572294	7.93E+06	0.06	-4.64	3.99	0.08
E2	GY	1D	AX.89572294	7.93E+06	0.06	-4.64	3.99	0.08
E2	GY	1D	AX.89676569	8.02E+06	0.06	-4.85	4.03	0.09
E2	GPM2	1D	AX.89651440	8.19E+06	0.06	-1066.96	3.12	0.07
E2	GY	1D	AX.89641164	8.02E+06	0.06	-4.85	4.03	0.09
E2	GPM2	1D	AX.89726393	7.98E+06	0.06	-1066.96	3.12	0.07
E2	GY	1D	AX.89696945	7.90E+06	0.06	-5.37	4.82	0.10
E2	GY	1D	AX.89696945	7.90E+06	0.06	-5.37	4.82	0.10
E2	GPM2	1D	AX.89676569	8.02E+06	0.06	-1066.96	3.12	0.07
E2	GPM2	1D	AX.89690807	8.04E+06	0.06	-1066.96	3.12	0.07
E2	GY	1D	AX.89698093	7.95E+06	0.06	-4.85	4.03	0.09
E2	GPM2	1D	AX.89760074	1.21E+07	0.11	-844.51	3.46	0.08
E2	GY	1D	AX.89760074	1.21E+07	0.11	-4.12	5.10	0.10
E2	GY	1D	AX.89496589	8.10E+06	0.06	-4.85	4.03	0.09
E2	GY	1D	AX.89522651	7.92E+06	0.06	-4.64	3.99	0.08

E2	GY	1D	AX.89522651	7.92E+06	0.06	-4.64	3.99	0.08
E2	GY	1D	AX.89618537	3.35E+06	0.05	-5.79	5.24	0.07
E2	GY	1D	AX.89501415	8.02E+06	0.06	-4.85	4.03	0.09
E2	GY	1D	AX.89501415	8.02E+06	0.06	-4.85	4.03	0.09
E2	GPS	1D	AX.89736405	4.10E+08	0.31	-1.43	3.05	0.04
E2	GPM2	1D	AX.89696945	7.90E+06	0.06	-1071.88	3.15	0.07
E2	GPM2	1D	AX.89501415	8.02E+06	0.06	-1066.96	3.12	0.07
E2	GPM2	1D	AX.89489677	7.95E+06	0.06	-1066.96	3.12	0.07
E2	GY	1D	AX.89698093	7.95E+06	0.06	-4.85	4.03	0.09
E2	GPM2	1D	AX.89485389	8.10E+06	0.06	-1066.96	3.12	0.07
E2	GPM2	1D	AX.89381613	8.06E+06	0.06	-1066.96	3.12	0.07
E2	GPM2	1D	AX.89379975	7.95E+06	0.06	-1066.96	3.12	0.07
E2	GPM2	1D	AX.89331550	8.20E+06	0.06	-1066.96	3.12	0.07
E2	GY	1D	AX.89618537	3.35E+06	0.05	-5.79	5.24	0.07
E2	GY	1D	AX.89762961	1.23E+07	0.10	-3.34	3.31	0.07
E2	GPM2	1D	AX.89698093	7.95E+06	0.06	-1066.96	3.12	0.07
E2	GPM2	1D	AX.89697899	8.02E+06	0.06	-1066.96	3.12	0.07
E2	GPM2	1D	AX.89496589	8.10E+06	0.06	-1066.96	3.12	0.07
E2	GPM2	1D	AX.89467842	8.04E+06	0.06	-1066.96	3.12	0.07
E2	GPM2	1D	AX.89494956	7.99E+06	0.05	-1126.09	3.22	0.07
E2	GPM2	1D	AX.89375427	8.10E+06	0.06	-1066.96	3.12	0.07
E3	GPM2	1D	AX.89463982	2.09E+07	0.10	1438.85	3.23	0.06
E3	GPM2	1D	AX.89507713	7.31E+07	0.12	1335.59	3.36	0.08
E3	GPM2	1D	AX.89578094	3.66E+08	0.12	1325.83	3.08	0.09
E3	GPS	1D	AX.89364350	1.22E+07	0.39	-2.32	3.93	0.13
E3	GPS	1D	AX.89744650	4.83E+08	0.44	-2.07	3.33	0.07
E3	GY	1D	AX.89684119	7.10E+06	0.37	3.06	3.15	0.11
E3	GY	1D	AX.89463982	2.09E+07	0.10	4.95	3.42	0.04
E3	GY	1D	AX.89507713	7.31E+07	0.12	4.46	3.33	0.09
E3	SPM2	1D	AX.89753708	2.17E+07	0.04	53.65	3.50	0.07
E4	GPM2	1D	AX.89623335	4.77E+08	0.13	-810.64	4.72	0.06
E4	GPS	1D	AX.89318668	4.54E+08	0.14	2.32	3.82	0.07
E4	GY	1D	AX.89606955	2.69E+08	0.08	-3.12	4.01	0.06
E4	GY	1D	AX.89539212	2.70E+08	0.08	-2.75	3.34	0.05
E4	GY	1D	AX.89753687	2.72E+08	0.08	-3.12	4.01	0.06
E4	GY	1D	AX.89613260	2.74E+08	0.08	-3.12	4.01	0.06
E4	GY	1D	AX.89586226	2.74E+08	0.09	-2.76	3.45	0.05
E4	GY	1D	AX.89643148	2.78E+08	0.08	-3.31	4.31	0.06
E4	GY	1D	AX.89529753	2.81E+08	0.08	-3.31	4.31	0.06
E4	GY	1D	AX.89741695	2.83E+08	0.08	-3.31	4.31	0.06
E4	GY	1D	AX.89364341	2.85E+08	0.07	-3.02	3.59	0.05
E4	GY	1D	AX.89444914	2.89E+08	0.07	-3.06	3.64	0.05
E4	GY	1D	AX.89561437	2.93E+08	0.07	-3.41	4.38	0.06
E4	GY	1D	AX.89755578	2.93E+08	0.07	-3.39	4.20	0.06
E4	GY	1D	AX.89411064	2.93E+08	0.08	-3.05	3.78	0.05
E4	GY	1D	AX.89322056	2.94E+08	0.08	-2.88	3.51	0.05
E4	GY	1D	AX.89609066	3.02E+08	0.07	-3.21	3.58	0.06
E4	GY	1D	AX.89623335	4.77E+08	0.13	-2.77	4.82	0.08
E4	GY	1D	X.89577482_OT	7.76E+07	0.07	-3.22	3.77	0.05
E4	GY	1D	AX.89444313	1.73E+08	0.08	-3.19	4.11	0.06

E4	GY	1D	AX.89729040	2.18E+08	0.10	-3.00	4.30	0.07
E4	SPM2	1D	AX.89347517	3.20E+08	0.06	37.74	3.78	0.05
E4	SPM2	1D	AX.89515010	4.34E+08	0.31	17.87	3.11	0.04
E4	SPM2	1D	AX.89722511	4.34E+08	0.31	17.87	3.11	0.04
E4	SPM2	1D	AX.89543956	4.34E+08	0.31	17.87	3.11	0.04
E4	SPM2	1D	AX.89521176	4.34E+08	0.31	17.87	3.11	0.04
E4	SPM2	1D	AX.89475596	4.34E+08	0.31	18.14	3.18	0.04
E4	SPM2	1D	AX.89429238	4.34E+08	0.32	19.04	3.60	0.04
E4	SPM2	1D	AX.89398238	4.34E+08	0.31	17.87	3.11	0.04
E4	SPM2	1D	AX.89347182	4.34E+08	0.41	-16.68	3.29	0.04
E4	SPM2	1D	AX.89482623	4.34E+08	0.31	17.87	3.11	0.04
E4	SPM2	1D	AX.89559638	4.34E+08	0.30	17.93	3.11	0.04
E4	SPM2	1D	AX.89456506	4.34E+08	0.30	18.71	3.37	0.04
E4	TKW	1D	AX.89340324	4.68E+08	0.14	-1.21	3.37	0.05
E1	GY	2A	AX.89343827	8.63E+07	0.07	-4.82	3.53	0.07
E1	GY	2A	AX.89746110	6.12E+08	0.49	-2.30	3.16	0.05
E1	GY	2A	AX.89343827	8.63E+07	0.07	-4.82	3.53	0.07
E1	GY	2A	AX.89517936	7.10E+07	0.32	-2.42	3.06	0.06
E1	GY	2A	AX.86169701	7.76E+08	0.10	-3.68	3.00	0.04
E1	GY	2A	AX.86169701	7.76E+08	0.10	-3.68	3.00	0.04
E1	GY	2A	AX.89534309	6.12E+08	0.49	-2.30	3.16	0.05
E1	GY	2A	AX.89468956	8.39E+07	0.21	-3.01	3.56	0.06
E1	GY	2A	AX.89431398	7.14E+07	0.43	-2.29	3.09	0.06
E1	GY	2A	AX.89534309	6.12E+08	0.49	-2.30	3.16	0.05
E1	GY	2A	AX.89328714	7.69E+08	0.06	-4.93	3.07	0.05
E1	GY	2A	AX.89542461	6.93E+08	0.19	-3.86	5.03	0.08
E1	GY	2A	AX.89318649	6.12E+08	0.50	2.38	3.35	0.05
E1	GY	2A	AX.89757261	7.69E+08	0.21	-3.22	3.95	0.06
E1	GY	2A	AX.89542301	7.59E+08	0.07	-4.32	3.08	0.05
E1	GY	2A	AX.89339520	8.39E+07	0.21	-3.01	3.51	0.06
E1	GY	2A	AX.89501387	8.39E+07	0.21	-3.01	3.56	0.06
E1	GY	2A	AX.89404168	6.23E+07	0.45	-2.26	3.03	0.05
E1	GY	2A	AX.89586153	7.10E+07	0.43	-2.29	3.09	0.06
E1	GY	2A	AX.89457808	7.69E+08	0.20	-3.25	3.95	0.06
E1	GY	2A	AX.89352998	7.11E+07	0.32	-2.42	3.06	0.06
E1	GY	2A	AX.89320118	6.12E+08	0.49	-2.30	3.16	0.05
E1	GY	2A	AX.89378004	8.39E+07	0.21	-3.01	3.56	0.06
E1	GY	2A	AX.89553523	8.63E+07	0.23	-3.06	3.85	0.07
E1	GY	2A	AX.89441861	3.59E+07	0.09	4.03	3.13	0.05
E1	GY	2A	AX.89372440	7.10E+07	0.43	-2.29	3.09	0.06
E1	GY	2A	AX.89353071	8.49E+07	0.23	-2.96	3.60	0.06
E1	GY	2A	AX.89408757	7.11E+07	0.43	-2.29	3.09	0.06
E1	GY	2A	AX.89432407	8.56E+07	0.23	-3.06	3.85	0.07
E1	GY	2A	AX.89369312	8.46E+07	0.23	-2.74	3.16	0.05
E1	GY	2A	AX.89463340	8.63E+07	0.23	-3.06	3.85	0.07
E1	GY	2A	AX.89328714	7.69E+08	0.06	-4.93	3.07	0.05
E1	GY	2A	AX.89508461	8.56E+07	0.07	-4.82	3.53	0.07
E1	GY	2A	AX.89694875	7.11E+07	0.32	-2.42	3.06	0.06
E1	GY	2A	AX.89609674	7.11E+07	0.32	-2.42	3.06	0.06
E1	GY	2A	AX.89420289	7.10E+07	0.32	-2.42	3.06	0.06

E1	GY	2A	AX.89386633	7.12E+07	0.43	-2.29	3.09	0.06
E1	GY	2A	AX.89386633	7.12E+07	0.43	-2.29	3.09	0.06
E1	GY	2A	AX.89673116	7.16E+07	0.32	-2.42	3.06	0.06
E1	GY	2A	AX.89609674	7.11E+07	0.32	-2.42	3.06	0.06
E1	GY	2A	AX.89401600	7.13E+07	0.43	-2.29	3.09	0.06
E1	GY	2A	AX.89757261	7.69E+08	0.21	-3.22	3.95	0.06
E1	GY	2A	AX.89320118	6.12E+08	0.49	-2.30	3.16	0.05
E1	GY	2A	AX.89377760	8.45E+07	0.23	-2.96	3.60	0.06
E1	GY	2A	AX.89414077	8.46E+07	0.07	-5.54	4.26	0.08
E1	GY	2A	AX.89414077	8.46E+07	0.07	-5.54	4.26	0.08
E1	GY	2A	AX.89385952	7.15E+07	0.32	-2.42	3.06	0.06
E1	GY	2A	AX.89508461	8.56E+07	0.07	-4.82	3.53	0.07
E1	GY	2A	AX.89699881	7.14E+07	0.32	-2.42	3.06	0.06
E1	GY	2A	AX.89586153	7.10E+07	0.43	-2.29	3.09	0.06
E1	GY	2A	AX.89602806	8.46E+07	0.23	-2.96	3.60	0.06
E1	GY	2A	AX.89486476	7.59E+08	0.07	-4.32	3.08	0.05
E1	GY	2A	AX.89505306	7.16E+07	0.32	-2.42	3.06	0.06
E1	GY	2A	AX.89517936	7.10E+07	0.32	-2.42	3.06	0.06
E1	GY	2A	AX.89490792	7.14E+07	0.43	-2.29	3.09	0.06
E1	GY	2A	AX.89746110	6.12E+08	0.49	-2.30	3.16	0.05
E1	GY	2A	AX.89564635	7.16E+07	0.43	-2.29	3.09	0.06
E1	GY	2A	AX.89377760	8.45E+07	0.23	-2.96	3.60	0.06
E1	GY	2A	AX.89393559	7.16E+07	0.32	-2.42	3.06	0.06
E1	GY	2A	AX.89731305	7.56E+08	0.25	-2.91	3.71	0.08
E1	GY	2A	AX.89488613	8.56E+07	0.07	-5.54	4.26	0.08
E1	GY	2A	AX.89385952	7.15E+07	0.32	-2.42	3.06	0.06
E1	GY	2A	AX.89358971	8.42E+07	0.21	-3.01	3.56	0.06
E1	GY	2A	AX.89371398	7.10E+07	0.43	-2.29	3.09	0.06
E1	GY	2A	AX.89617723	8.41E+07	0.21	-3.01	3.56	0.06
E1	GY	2A	AX.89382939	8.63E+07	0.07	-4.82	3.53	0.07
E1	GY	2A	AX.89626514	7.61E+08	0.16	3.14	3.20	0.05
E1	GY	2A	AX.89443555	7.56E+08	0.26	-2.76	3.46	0.07
E1	GY	2A	AX.89432407	8.56E+07	0.23	-3.06	3.85	0.07
E1	GY	2A	AX.89369312	8.46E+07	0.23	-2.74	3.16	0.05
E1	GY	2A	AX.89548461	7.16E+07	0.32	-2.42	3.06	0.06
E1	GY	2A	AX.89651445	8.39E+07	0.21	-3.01	3.56	0.06
E1	GY	2A	AX.89694875	7.11E+07	0.32	-2.42	3.06	0.06
E1	GY	2A	AX.89645117	7.13E+07	0.43	-2.29	3.09	0.06
E1	GY	2A	AX.89549784	8.53E+07	0.22	-2.96	3.55	0.06
E1	GY	2A	AX.89549784	8.53E+07	0.22	-2.96	3.55	0.06
E1	GY	2A	AX.89328431	8.39E+07	0.21	-3.01	3.56	0.06
E1	GY	2A	AX.89339520	8.39E+07	0.21	-3.01	3.51	0.06
E1	GY	2A	AX.89434520	8.39E+07	0.21	-3.01	3.56	0.06
E1	GY	2A	AX.89550729	7.14E+07	0.32	-2.42	3.06	0.06
E1	GY	2A	AX.89318649	6.12E+08	0.50	2.38	3.35	0.05
E1	GY	2A	AX.89468956	8.39E+07	0.21	-3.01	3.56	0.06
E1	GY	2A	AX.89563662	6.78E+08	0.47	2.44	3.48	0.05
E1	GY	2A	AX.89563662	6.78E+08	0.47	2.44	3.48	0.05
E1	GY	2A	AX.89741076	7.86E+07	0.29	2.66	3.46	0.06
E1	GY	2A	AX.89741076	7.86E+07	0.29	2.66	3.46	0.06

E1	GY	2A	AX.89490792	7.14E+07	0.43	-2.29	3.09	0.06
E1	GY	2A	AX.89745955	8.46E+07	0.23	-2.96	3.60	0.06
E1	GY	2A	AX.89668676	7.16E+07	0.43	-2.29	3.09	0.06
E1	GY	2A	AX.89401600	7.13E+07	0.43	-2.29	3.09	0.06
E1	GY	2A	AX.89424726	8.50E+07	0.23	-2.96	3.60	0.06
E1	GY	2A	AX.89424726	8.50E+07	0.23	-2.96	3.60	0.06
E1	GY	2A	AX.89731305	7.56E+08	0.25	-2.91	3.71	0.08
E1	GY	2A	AX.89553523	8.63E+07	0.23	-3.06	3.85	0.07
E1	GY	2A	AX.89480595	8.56E+07	0.07	-4.82	3.53	0.07
E1	GY	2A	AX.89390161	7.14E+07	0.43	-2.29	3.09	0.06
E1	GY	2A	AX.89542301	7.59E+08	0.07	-4.32	3.08	0.05
E1	GY	2A	AX.89402644	8.45E+07	0.23	-2.96	3.60	0.06
E1	GY	2A	AX.89343847	8.50E+07	0.06	-4.80	3.13	0.06
E1	GY	2A	AX.89343847	8.50E+07	0.06	-4.80	3.13	0.06
E1	GY	2A	AX.89712759	8.50E+07	0.23	-2.96	3.60	0.06
E1	GY	2A	AX.89311260	7.14E+07	0.43	-2.29	3.09	0.06
E1	GY	2A	AX.89311260	7.14E+07	0.43	-2.29	3.09	0.06
E1	GY	2A	AX.89324170	7.69E+08	0.06	-4.93	3.07	0.05
E1	GY	2A	AX.89628287	8.42E+07	0.06	-6.32	5.04	0.10
E1	GY	2A	AX.89378004	8.39E+07	0.21	-3.01	3.56	0.06
E1	GY	2A	AX.89417730	7.14E+07	0.32	-2.42	3.06	0.06
E1	GY	2A	AX.89417730	7.14E+07	0.32	-2.42	3.06	0.06
E1	GY	2A	AX.89328431	8.39E+07	0.21	-3.01	3.56	0.06
E1	GY	2A	AX.89371398	7.10E+07	0.43	-2.29	3.09	0.06
E1	GY	2A	AX.89617723	8.41E+07	0.21	-3.01	3.56	0.06
E1	GY	2A	AX.89382126	7.10E+07	0.46	-2.29	3.11	0.06
E1	GY	2A	AX.89402644	8.45E+07	0.23	-2.96	3.60	0.06
E1	GY	2A	AX.89617767	7.14E+07	0.33	-2.55	3.38	0.07
E1	GY	2A	AX.89617811	3.59E+07	0.08	4.41	3.35	0.06
E1	GY	2A	AX.89441861	3.59E+07	0.09	4.03	3.13	0.05
E1	GY	2A	AX.89382605	6.12E+08	0.49	-2.30	3.16	0.05
E1	GY	2A	AX.89404168	6.23E+07	0.45	-2.26	3.03	0.05
E1	GY	2A	AX.89372788	7.40E+08	0.14	3.24	3.10	0.04
E1	GY	2A	AX.89372788	7.40E+08	0.14	3.24	3.10	0.04
E1	GY	2A	AX.89348730	8.50E+07	0.23	-2.96	3.60	0.06
E1	GY	2A	AX.89348730	8.50E+07	0.23	-2.96	3.60	0.06
E1	GY	2A	AX.89420289	7.10E+07	0.32	-2.42	3.06	0.06
E1	GY	2A	AX.89645117	7.13E+07	0.43	-2.29	3.09	0.06
E1	GY	2A	AX.89382126	7.10E+07	0.46	-2.29	3.11	0.06
E1	GY	2A	AX.89711069	7.14E+07	0.33	-2.45	3.15	0.06
E1	GY	2A	AX.89457808	7.69E+08	0.20	-3.25	3.95	0.06
E1	GY	2A	AX.89551770	8.39E+07	0.21	-3.01	3.56	0.06
E1	GY	2A	AX.89353071	8.49E+07	0.23	-2.96	3.60	0.06
E1	GY	2A	AX.89552048	8.39E+07	0.21	-3.01	3.56	0.06
E1	GY	2A	AX.89552048	8.39E+07	0.21	-3.01	3.56	0.06
E1	GY	2A	AX.89564635	7.16E+07	0.43	-2.29	3.09	0.06
E1	GY	2A	AX.89320349	8.79E+07	0.25	-2.61	3.08	0.05
E1	GY	2A	AX.89484946	6.78E+08	0.47	2.38	3.34	0.05
E1	GY	2A	AX.89519604	7.14E+07	0.43	-2.29	3.09	0.06
E1	GY	2A	AX.89519604	7.14E+07	0.43	-2.29	3.09	0.06

E1	GY	2A	AX.89425537	8.42E+07	0.21	-3.01	3.56	0.06
E1	GY	2A	AX.89747464	8.45E+07	0.07	-5.54	4.26	0.08
E1	GY	2A	AX.89747464	8.45E+07	0.07	-5.54	4.26	0.08
E1	GY	2A	AX.89699881	7.14E+07	0.32	-2.42	3.06	0.06
E1	GY	2A	AX.89508618	8.56E+07	0.07	-4.82	3.53	0.07
E1	GY	2A	AX.89654157	6.12E+08	0.49	-2.30	3.16	0.05
E1	GY	2A	AX.89461565	8.50E+07	0.23	-2.96	3.60	0.06
E1	GY	2A	AX.89737425	6.97E+08	0.12	-3.56	3.12	0.05
E1	GY	2A	AX.89604444	7.14E+07	0.43	-2.29	3.09	0.06
E1	GY	2A	AX.89393559	7.16E+07	0.32	-2.42	3.06	0.06
E1	GY	2A	AX.89358971	8.42E+07	0.21	-3.01	3.56	0.06
E1	GY	2A	AX.89395191	6.93E+08	0.17	-3.93	4.83	0.08
E1	GY	2A	AX.89431192	7.14E+07	0.32	-2.42	3.06	0.06
E1	GY	2A	AX.89431398	7.14E+07	0.43	-2.29	3.09	0.06
E1	GY	2A	AX.89382939	8.63E+07	0.07	-4.82	3.53	0.07
E1	GY	2A	AX.89372440	7.10E+07	0.43	-2.29	3.09	0.06
E1	GY	2A	AX.89501387	8.39E+07	0.21	-3.01	3.56	0.06
E1	GY	2A	AX.89569793	8.46E+07	0.07	-5.54	4.26	0.08
E1	GY	2A	AX.89443555	7.56E+08	0.26	-2.76	3.46	0.07
E1	GY	2A	AX.89454688	8.46E+07	0.23	-2.96	3.60	0.06
E1	GY	2A	AX.89608550	8.46E+07	0.23	-2.96	3.60	0.06
E1	GY	2A	AX.89455016	7.14E+07	0.43	-2.29	3.09	0.06
E1	GY	2A	AX.89455016	7.14E+07	0.43	-2.29	3.09	0.06
E1	GY	2A	AX.89670276	7.51E+08	0.06	-5.10	3.47	0.07
E1	GY	2A	AX.89768688	8.39E+07	0.21	-3.01	3.56	0.06
E1	GY	2A	AX.89610686	8.42E+07	0.06	-5.37	3.80	0.07
E1	GY	2A	AX.89434520	8.39E+07	0.21	-3.01	3.56	0.06
E1	GY	2A	AX.89434666	8.63E+07	0.23	-3.06	3.85	0.07
E1	GY	2A	AX.89434666	8.63E+07	0.23	-3.06	3.85	0.07
E1	GY	2A	AX.89505306	7.16E+07	0.32	-2.42	3.06	0.06
E1	GY	2A	AX.89721986	8.63E+07	0.07	-4.82	3.53	0.07
E1	GY	2A	AX.89673116	7.16E+07	0.32	-2.42	3.06	0.06
E1	GY	2A	AX.89637021	8.46E+07	0.23	-2.96	3.60	0.06
E1	GY	2A	AX.89495417	8.39E+07	0.21	-3.01	3.56	0.06
E1	GY	2A	AX.89495968	7.69E+08	0.06	-4.93	3.07	0.05
E1	GY	2A	AX.89390161	7.14E+07	0.43	-2.29	3.09	0.06
E1	GY	2A	AX.89486476	7.59E+08	0.07	-4.32	3.08	0.05
E1	GY	2A	AX.89737425	6.97E+08	0.12	-3.56	3.12	0.05
E1	GY	2A	AX.89345178	7.14E+07	0.32	-2.42	3.06	0.06
E1	GY	2A	AX.89775602	7.10E+07	0.32	-2.42	3.06	0.06
E1	GY	2A	AX.89651445	8.39E+07	0.21	-3.01	3.56	0.06
E1	GY	2A	AX.89463340	8.63E+07	0.23	-3.06	3.85	0.07
E1	GPS	2A	AX.89429150	7.47E+08	0.26	1.31	3.27	0.05
E1	GY	2A	AX.89704050	7.69E+08	0.20	-3.25	3.95	0.06
E1	GY	2A	AX.89395191	6.93E+08	0.17	-3.93	4.83	0.08
E1	GPS	2A	AX.89426786	7.79E+08	0.21	1.39	3.20	0.06
E1	GPS	2A	AX.89766512	7.78E+08	0.22	1.35	3.10	0.05
E1	GPS	2A	AX.89531001	7.53E+08	0.28	1.26	3.18	0.05
E1	GPS	2A	AX.89573905	7.55E+08	0.24	1.54	4.11	0.07
E1	GPS	2A	AX.89706460	7.55E+08	0.21	1.53	3.75	0.07

E1	GPS	2A	AX.89771579	7.47E+08	0.21	1.36	3.11	0.05
E1	GPS	2A	AX.89531704	7.78E+08	0.22	1.35	3.10	0.05
E1	GPS	2A	AX.89562993	7.55E+08	0.26	1.39	3.62	0.06
E1	GY	2A	AX.89454688	8.46E+07	0.23	-2.96	3.60	0.06
E1	GPS	2A	AX.89592262	7.53E+08	0.41	1.23	3.56	0.06
E1	GPS	2A	AX.89375933	7.55E+08	0.26	1.39	3.62	0.06
E1	GPS	2A	AX.89347674	7.56E+08	0.26	1.41	3.70	0.06
E1	GY	2A	AX.89409852	7.76E+08	0.10	-3.79	3.04	0.05
E1	GY	2A	AX.89409852	7.76E+08	0.10	-3.79	3.04	0.05
E1	GPS	2A	AX.89405293	7.47E+08	0.17	1.47	3.04	0.05
E1	GPS	2A	AX.89468617	7.80E+08	0.20	1.42	3.20	0.06
E1	GPS	2A	AX.89500632	5.08E+08	0.33	1.17	3.03	0.05
E1	GPS	2A	AX.89583054	7.80E+08	0.21	1.39	3.20	0.06
E1	GPS	2A	AX.89705282	5.11E+08	0.35	1.16	3.06	0.05
E1	GPS	2A	AX.89534141	7.55E+08	0.27	1.49	4.19	0.07
E1	GY	2A	AX.89632373	8.45E+07	0.23	-2.96	3.60	0.06
E1	GPS	2A	AX.89705624	7.59E+08	0.17	1.67	3.77	0.07
E1	GPS	2A	AX.89720303	7.60E+08	0.18	1.45	3.07	0.05
E1	GPS	2A	AX.89413805	4.69E+08	0.33	1.26	3.46	0.06
E1	GY	2A	AX.89777060	8.39E+07	0.21	-3.01	3.56	0.06
E1	GPS	2A	AX.89487768	7.56E+08	0.24	1.42	3.59	0.06
E1	GPS	2A	AX.89538795	7.78E+08	0.22	1.43	3.46	0.06
E1	GPS	2A	AX.89688383	7.49E+08	0.23	1.47	3.74	0.07
E1	GPS	2A	AX.89317553	7.78E+08	0.22	1.43	3.46	0.06
E1	GY	2A	AX.89754767	7.69E+08	0.06	-4.93	3.07	0.05
E1	GY	2A	AX.89550729	7.14E+07	0.32	-2.42	3.06	0.06
E1	GPS	2A	AX.89660004	7.49E+08	0.23	1.49	3.76	0.07
E1	GY	2A	AX.89683882	6.78E+08	0.49	2.35	3.26	0.05
E1	GY	2A	AX.89683882	6.78E+08	0.49	2.35	3.26	0.05
E1	GPS	2A	AX.89468552	7.79E+08	0.20	1.36	3.01	0.05
E1	GY	2A	AX.89551770	8.39E+07	0.21	-3.01	3.56	0.06
E1	GPS	2A	AX.89466785	7.14E+08	0.17	1.51	3.15	0.05
E1	GY	2A	AX.89668676	7.16E+07	0.43	-2.29	3.09	0.06
E1	GY	2A	AX.89603203	7.59E+08	0.07	-4.32	3.08	0.05
E1	GY	2A	AX.89684909	7.13E+07	0.32	-2.42	3.06	0.06
E1	GPS	2A	AX.89475404	5.17E+08	0.33	1.20	3.19	0.06
E1	GPS	2A	AX.89668198	7.47E+08	0.21	1.36	3.11	0.05
E1	GPS	2A	AX.89696276	5.23E+08	0.33	1.25	3.39	0.06
E1	GPS	2A	AX.89752134	7.55E+08	0.26	1.43	3.76	0.07
E1	GPS	2A	AX.89442869	7.55E+08	0.24	1.47	3.77	0.07
E1	GY	2A	AX.89484946	6.78E+08	0.47	2.38	3.34	0.05
E1	GPS	2A	AX.89503320	7.78E+08	0.22	1.35	3.10	0.05
E1	GPS	2A	AX.89622756	7.79E+08	0.20	1.36	3.01	0.05
E1	GPS	2A	AX.89655826	5.11E+08	0.33	1.17	3.03	0.05
E1	GPS	2A	AX.89397285	7.80E+08	0.20	1.42	3.20	0.06
E1	GPS	2A	AX.89555442	4.35E+08	0.35	1.16	3.05	0.05
E1	GPS	2A	AX.89520383	7.55E+08	0.34	1.48	4.65	0.08
E1	GY	2A	AX.89542461	6.93E+08	0.19	-3.86	5.03	0.08
E1	GPS	2A	AX.89685569	7.47E+08	0.20	1.54	3.64	0.06
E1	GPS	2A	AX.89393215	7.56E+08	0.27	1.33	3.42	0.05

E1	GPS	2A	AX.89438787	7.55E+08	0.25	1.37	3.46	0.06
E1	GPS	2A	AX.89457808	7.69E+08	0.20	-1.54	3.75	0.06
E1	GPS	2A	AX.89583185	7.55E+08	0.26	1.39	3.62	0.06
E1	GPS	2A	AX.89737871	7.55E+08	0.24	1.47	3.77	0.07
E1	GY	2A	AX.89700088	8.56E+07	0.23	-3.05	3.80	0.07
E1	GY	2A	AX.89700088	8.56E+07	0.23	-3.05	3.80	0.07
E1	GY	2A	AX.89712759	8.50E+07	0.23	-2.96	3.60	0.06
E1	GY	2A	AX.89450281	8.53E+07	0.22	-3.11	3.88	0.07
E1	GY	2A	AX.89450281	8.53E+07	0.22	-3.11	3.88	0.07
E1	GPS	2A	AX.89771558	5.10E+08	0.32	1.22	3.23	0.06
E1	GPS	2A	AX.89402750	7.79E+08	0.20	1.36	3.01	0.05
E1	GY	2A	AX.89654157	6.12E+08	0.49	-2.30	3.16	0.05
E1	GPS	2A	AX.89526331	7.78E+08	0.22	1.35	3.10	0.05
E1	GPS	2A	AX.89778038	7.79E+08	0.21	1.39	3.20	0.06
E1	GPS	2A	AX.89604366	5.17E+08	0.33	1.20	3.19	0.06
E1	GPS	2A	AX.89561705	7.80E+08	0.20	1.42	3.20	0.06
E1	GPS	2A	AX.89445758	7.79E+08	0.20	1.36	3.01	0.05
E1	GY	2A	AX.89569793	8.46E+07	0.07	-5.54	4.26	0.08
E1	GPS	2A	AX.89750014	5.08E+08	0.32	1.20	3.12	0.05
E1	GPS	2A	AX.89636460	7.55E+08	0.26	1.43	3.76	0.07
E1	GPS	2A	AX.89314076	7.78E+08	0.22	1.35	3.10	0.05
E1	GPS	2A	AX.89522390	7.78E+08	0.22	1.35	3.10	0.05
E1	GY	2A	AX.89543557	8.49E+07	0.23	-2.96	3.60	0.06
E1	GPS	2A	AX.89668955	7.56E+08	0.25	1.40	3.54	0.06
E1	GPS	2A	AX.89637508	7.55E+08	0.27	1.43	3.86	0.07
E1	GPS	2A	AX.89483920	5.18E+08	0.34	1.27	3.53	0.06
E1	GPS	2A	AX.89696493	5.18E+08	0.33	1.20	3.19	0.06
E1	GPS	2A	AX.89527155	7.78E+08	0.22	1.35	3.10	0.05
E1	GY	2A	AX.89429525	8.42E+07	0.07	-5.54	4.26	0.08
E1	GY	2A	AX.89429525	8.42E+07	0.07	-5.54	4.26	0.08
E1	GPS	2A	AX.89441296	7.55E+08	0.26	1.39	3.62	0.06
E1	GPS	2A	AX.89530131	7.55E+08	0.27	1.43	3.86	0.07
E1	GY	2A	AX.89666189	7.10E+07	0.32	-2.42	3.06	0.06
E1	GY	2A	AX.89666189	7.10E+07	0.32	-2.42	3.06	0.06
E1	GPS	2A	AX.89355132	5.15E+08	0.32	1.17	3.01	0.05
E1	GY	2A	AX.89617767	7.14E+07	0.33	-2.55	3.38	0.07
E1	GY	2A	AX.89452906	8.64E+07	0.23	-3.05	3.80	0.07
E1	GY	2A	AX.89313648	3.12E+07	0.10	3.84	3.22	0.05
E1	GY	2A	AX.89654740	1.07E+08	0.13	-3.29	3.04	0.04
E1	GPS	2A	AX.89372948	7.79E+08	0.20	1.38	3.05	0.05
E1	GPS	2A	AX.89414865	7.55E+08	0.24	1.50	3.95	0.07
E1	GPS	2A	AX.89316671	7.47E+08	0.26	1.31	3.27	0.05
E1	GY	2A	AX.89704050	7.69E+08	0.20	-3.25	3.95	0.06
E1	GY	2A	AX.89382605	6.12E+08	0.49	-2.30	3.16	0.05
E1	GPS	2A	AX.89423191	7.55E+08	0.26	1.39	3.62	0.06
E1	GY	2A	AX.89431192	7.14E+07	0.32	-2.42	3.06	0.06
E1	GPS	2A	AX.89609963	4.39E+08	0.34	1.18	3.12	0.05
E1	GPS	2A	AX.89504090	5.23E+08	0.33	1.20	3.19	0.06
E1	GPS	2A	AX.89772957	5.11E+08	0.33	1.17	3.03	0.05
E1	GPS	2A	AX.89747863	7.55E+08	0.27	1.39	3.71	0.06

E1	GY	2A	AX.89336730	7.40E+08	0.09	-4.19	3.34	0.05
E1	GPS	2A	AX.89513724	7.55E+08	0.24	1.47	3.77	0.07
E1	GY	2A	AX.89618874	8.56E+07	0.07	-4.82	3.53	0.07
E1	GPS	2A	AX.89591770	7.56E+08	0.24	1.41	3.58	0.06
E1	GPS	2A	AX.89660639	5.11E+08	0.33	1.17	3.03	0.05
E1	GPS	2A	AX.89646006	7.56E+08	0.26	1.41	3.70	0.06
E1	GPS	2A	AX.89530584	7.56E+08	0.26	1.41	3.70	0.06
E1	GY	2A	AX.89716272	7.14E+07	0.32	-2.42	3.06	0.06
E1	GY	2A	AX.89610686	8.42E+07	0.06	-5.37	3.80	0.07
E1	GY	2A	AX.89711069	7.14E+07	0.33	-2.45	3.15	0.06
E1	GY	2A	AX.89352998	7.11E+07	0.32	-2.42	3.06	0.06
E1	GY	2A	AX.89693178	7.88E+07	0.29	2.48	3.06	0.05
E1	GY	2A	AX.89693178	7.88E+07	0.29	2.48	3.06	0.05
E1	GY	2A	AX.89513541	7.58E+08	0.06	-4.93	3.08	0.05
E1	GY	2A	AX.89513541	7.58E+08	0.06	-4.93	3.08	0.05
E1	GY	2A	AX.89353712	8.63E+07	0.24	-2.84	3.44	0.06
E1	GY	2A	AX.89495219	8.50E+07	0.23	-2.96	3.60	0.06
E1	GPS	2A	AX.89742627	7.54E+08	0.21	1.58	3.99	0.07
E1	GY	2A	AX.89320349	8.79E+07	0.25	-2.61	3.08	0.05
E1	GY	2A	AX.89531056	8.45E+07	0.23	-2.96	3.60	0.06
E1	GY	2A	AX.89531056	8.45E+07	0.23	-2.96	3.60	0.06
E1	GY	2A	AX.89425537	8.42E+07	0.21	-3.01	3.56	0.06
E1	GY	2A	AX.89633157	6.12E+08	0.49	-2.30	3.16	0.05
E1	GY	2A	AX.89633157	6.12E+08	0.49	-2.30	3.16	0.05
E1	GY	2A	AX.89602806	8.46E+07	0.23	-2.96	3.60	0.06
E1	GPS	2A	AX.89697822	7.80E+08	0.20	1.42	3.20	0.06
E1	GY	2A	AX.89728140	6.78E+08	0.49	2.35	3.26	0.05
E1	GPS	2A	AX.89344976	7.56E+08	0.28	1.32	3.49	0.06
E1	GPS	2A	AX.89771272	7.55E+08	0.24	1.42	3.62	0.06
E1	GY	2A	AX.89487119	1.16E+08	0.14	-3.26	3.13	0.05
E1	GY	2A	AX.89487119	1.16E+08	0.14	-3.26	3.13	0.05
E1	GY	2A	AX.89345178	7.14E+07	0.32	-2.42	3.06	0.06
E1	GY	2A	AX.89604444	7.14E+07	0.43	-2.29	3.09	0.06
E1	GPS	2A	AX.89632739	7.47E+08	0.26	1.31	3.27	0.05
E1	GY	2A	AX.89488613	8.56E+07	0.07	-5.54	4.26	0.08
E1	GY	2A	AX.89425114	8.46E+07	0.23	-2.96	3.60	0.06
E1	GY	2A	AX.89425114	8.46E+07	0.23	-2.96	3.60	0.06
E1	GY	2A	AX.89750786	8.46E+07	0.23	-2.96	3.60	0.06
E1	GPS	2A	AX.89322846	7.80E+08	0.20	1.42	3.20	0.06
E1	GY	2A	AX.89743990	6.78E+08	0.47	2.44	3.48	0.05
E1	GY	2A	AX.89743990	6.78E+08	0.47	2.44	3.48	0.05
E1	GY	2A	AX.89617811	3.59E+07	0.08	4.41	3.35	0.06
E1	GY	2A	AX.89463683	7.10E+07	0.32	-2.42	3.06	0.06
E1	GY	2A	AX.89463683	7.10E+07	0.32	-2.42	3.06	0.06
E1	GY	2A	AX.89704676	7.61E+08	0.07	-4.27	3.02	0.05
E1	GY	2A	AX.89704676	7.61E+08	0.07	-4.27	3.02	0.05
E1	GY	2A	AX.89618874	8.56E+07	0.07	-4.82	3.53	0.07
E1	GY	2A	AX.89608550	8.46E+07	0.23	-2.96	3.60	0.06
E1	GY	2A	AX.89690258	7.14E+07	0.32	-2.42	3.06	0.06
E1	GY	2A	AX.89706225	8.42E+07	0.07	-5.54	4.26	0.08

E1	GY	2A	AX.89480595	8.56E+07	0.07	-4.82	3.53	0.07
E1	GY	2A	AX.89364039	1.20E+07	0.32	-2.70	3.68	0.05
E1	GY	2A	AX.89364039	1.20E+07	0.32	-2.70	3.68	0.05
E1	GPM2	2A	AX.89444397	7.79E+08	0.27	776.28	4.11	0.07
E1	GPM2	2A	AX.89342993	6.15E+07	0.21	812.89	3.79	0.07
E1	GPM2	2A	AX.89314076	7.78E+08	0.22	837.88	4.11	0.07
E1	GPM2	2A	AX.89403340	7.79E+08	0.20	860.25	4.06	0.07
E1	GPM2	2A	AX.89317187	7.59E+08	0.20	852.43	4.06	0.07
E1	GY	2A	AX.89702562	7.10E+07	0.32	-2.42	3.06	0.06
E1	GY	2A	AX.89702562	7.10E+07	0.32	-2.42	3.06	0.06
E1	GPM2	2A	AX.89424974	5.84E+07	0.31	755.44	4.18	0.07
E1	GPM2	2A	AX.89456578	7.79E+08	0.20	857.07	3.98	0.07
E1	GY	2A	AX.89495968	7.69E+08	0.06	-4.93	3.07	0.05
E1	GPM2	2A	AX.89404168	6.23E+07	0.45	-706.11	4.21	0.07
E1	GPM2	2A	AX.89490527	7.78E+08	0.25	692.95	3.18	0.06
E1	GPM2	2A	AX.89345178	7.14E+07	0.32	-650.04	3.27	0.06
E1	GPM2	2A	AX.89666189	7.10E+07	0.32	-650.04	3.27	0.06
E1	GPM2	2A	AX.89437001	6.04E+08	0.08	-1224.91	3.97	0.07
E1	GPM2	2A	AX.89328431	8.39E+07	0.21	-709.87	3.05	0.05
E1	GPM2	2A	AX.89673116	7.16E+07	0.32	-650.04	3.27	0.06
E1	GPM2	2A	AX.89377760	8.45E+07	0.23	-710.38	3.17	0.05
E1	GPM2	2A	AX.89443303	7.69E+08	0.17	-833.87	3.48	0.06
E1	GPM2	2A	AX.89377709	7.59E+08	0.20	852.43	4.06	0.07
E1	GPM2	2A	AX.89331385	7.59E+08	0.20	852.43	4.06	0.07
E1	GPM2	2A	AX.89495219	8.50E+07	0.23	-710.38	3.17	0.05
E1	GPM2	2A	AX.89670101	7.88E+07	0.42	-605.90	3.19	0.06
E1	GPM2	2A	AX.89526852	7.61E+08	0.20	888.57	4.30	0.08
E1	GPM2	2A	AX.89451642	7.59E+08	0.20	852.43	4.06	0.07
E1	GPM2	2A	AX.89451573	7.60E+08	0.19	943.55	4.55	0.08
E1	GPM2	2A	AX.89578308	7.60E+08	0.20	852.43	4.06	0.07
E1	GPM2	2A	AX.89575538	7.55E+08	0.26	695.37	3.27	0.06
E1	GPM2	2A	AX.89550729	7.14E+07	0.32	-650.04	3.27	0.06
E1	GPM2	2A	AX.89736779	7.58E+08	0.23	735.98	3.41	0.06
E1	GPM2	2A	AX.89473575	7.80E+08	0.21	948.49	4.97	0.09
E1	GPM2	2A	AX.89747464	8.45E+07	0.07	-1294.07	3.57	0.07
E1	GPM2	2A	AX.89697822	7.80E+08	0.20	927.23	4.64	0.08
E1	GPM2	2A	AX.89646989	7.58E+08	0.22	732.77	3.30	0.06
E1	GY	2A	AX.89677955	8.42E+07	0.20	-3.03	3.45	0.06
E1	GPM2	2A	AX.89530582	7.59E+08	0.20	852.43	4.06	0.07
E1	GY	2A	AX.89775602	7.10E+07	0.32	-2.42	3.06	0.06
E1	GPM2	2A	AX.89594346	1.94E+08	0.48	671.35	3.89	0.07
E1	GPM2	2A	AX.89586153	7.10E+07	0.43	-647.67	3.60	0.07
E1	GPM2	2A	AX.89393559	7.16E+07	0.32	-650.04	3.27	0.06
E1	GPM2	2A	AX.89376021	7.79E+08	0.19	777.68	3.31	0.06
E1	GPM2	2A	AX.89623731	7.80E+08	0.20	905.56	4.51	0.08
E1	GPM2	2A	AX.89537540	7.60E+08	0.20	852.43	4.06	0.07
E1	GPM2	2A	AX.89530584	7.56E+08	0.26	695.68	3.27	0.04
E1	GPM2	2A	AX.89522390	7.78E+08	0.22	837.88	4.11	0.07
E1	GPM2	2A	AX.89553538	6.04E+08	0.09	-1132.54	3.76	0.07
E1	GPM2	2A	AX.89413222	7.80E+08	0.21	948.49	4.97	0.09

E1	GPM2	2A	AX.89577627	7.56E+08	0.27	680.27	3.21	0.05
E1	GY	2A	AX.89442084	7.16E+07	0.32	-2.42	3.06	0.06
E1	GY	2A	AX.89442084	7.16E+07	0.32	-2.42	3.06	0.06
E1	GPS	2A	AX.89539167	7.55E+08	0.27	1.39	3.71	0.06
E1	GPM2	2A	AX.89727692	7.80E+08	0.20	927.23	4.64	0.08
E1	GPS	2A	AX.89380933	5.16E+08	0.34	1.18	3.10	0.05
E1	GPM2	2A	AX.89421305	7.60E+08	0.20	888.57	4.30	0.08
E1	GPM2	2A	AX.89330025	6.04E+08	0.09	-1132.54	3.76	0.07
E1	GPS	2A	AX.89698182	5.08E+08	0.33	1.20	3.16	0.05
E1	GY	2A	AX.89536127	7.69E+08	0.06	-4.93	3.07	0.05
E1	GPS	2A	AX.89750010	7.56E+08	0.26	1.36	3.50	0.06
E1	GPS	2A	AX.89614700	7.78E+08	0.22	1.35	3.10	0.05
E1	GPS	2A	AX.89534203	7.54E+08	0.25	1.67	4.87	0.08
E1	GY	2A	AX.89626514	7.61E+08	0.16	3.14	3.20	0.05
E1	GPS	2A	AX.89689821	7.55E+08	0.24	1.37	3.37	0.06
E1	GPS	2A	AX.89332830	4.50E+08	0.34	1.21	3.26	0.06
E1	GY	2A	AX.89408757	7.11E+07	0.43	-2.29	3.09	0.06
E1	GPS	2A	AX.89390818	7.49E+08	0.24	1.53	4.10	0.07
E1	GPM2	2A	AX.89592705	7.12E+07	0.43	-649.06	3.61	0.07
E1	GPM2	2A	AX.89706225	8.42E+07	0.07	-1294.07	3.57	0.07
E1	GPM2	2A	AX.89444536	7.78E+08	0.22	837.88	4.11	0.07
E1	GPM2	2A	AX.89542461	6.93E+08	0.19	-831.67	3.65	0.06
E1	GPS	2A	AX.89456234	5.11E+08	0.33	1.17	3.03	0.05
E1	GPM2	2A	AX.89580710	7.61E+08	0.20	888.57	4.30	0.08
E1	GPS	2A	AX.89389950	6.89E+08	0.31	-1.36	3.80	0.06
E1	GY	2A	AX.89548461	7.16E+07	0.32	-2.42	3.06	0.06
E1	GPS	2A	AX.89444536	7.78E+08	0.22	1.35	3.10	0.05
E1	GPS	2A	AX.89513185	7.55E+08	0.23	1.41	3.46	0.06
E1	GPS	2A	AX.89486496	7.47E+08	0.21	1.36	3.11	0.05
E1	GPM2	2A	AX.89516661	7.56E+08	0.27	677.79	3.25	0.04
E1	GPM2	2A	AX.89674991	7.78E+08	0.21	748.03	3.29	0.06
E1	GPS	2A	AX.89627848	7.78E+08	0.25	1.42	3.70	0.07
E1	GY	2A	AX.89479590	7.14E+07	0.32	-2.42	3.06	0.06
E1	GPM2	2A	AX.89765361	7.53E+08	0.10	975.50	3.13	0.05
E1	GPS	2A	AX.89473575	7.80E+08	0.21	1.39	3.20	0.06
E1	GPS	2A	AX.89492742	7.80E+08	0.20	1.42	3.20	0.06
E1	GPM2	2A	AX.89770934	6.04E+08	0.09	-1132.54	3.76	0.07
E1	GPS	2A	AX.89447705	5.23E+08	0.33	1.20	3.19	0.06
E1	GY	2A	AX.89632373	8.45E+07	0.23	-2.96	3.60	0.06
E1	GPS	2A	AX.89488337	7.55E+08	0.30	1.33	3.61	0.06
E1	GPM2	2A	AX.89349994	7.61E+08	0.20	888.57	4.30	0.08
E1	GPS	2A	AX.89334194	6.03E+08	0.11	-1.84	3.21	0.05
E1	GPS	2A	AX.89365566	7.55E+08	0.26	1.39	3.62	0.06
E1	GPS	2A	AX.89613544	7.78E+08	0.22	1.35	3.10	0.05
E1	GPM2	2A	AX.89555762	7.11E+07	0.43	-649.06	3.61	0.07
E1	GPS	2A	AX.89373981	7.50E+08	0.44	1.15	3.24	0.06
E1	GPS	2A	AX.89354221	7.56E+08	0.25	1.40	3.54	0.06
E1	GPS	2A	AX.89683066	4.26E+08	0.34	1.21	3.25	0.06
E1	GPM2	2A	AX.89684825	5.96E+07	0.20	761.94	3.34	0.05
E1	GPM2	2A	AX.89668955	7.56E+08	0.25	699.99	3.24	0.05

E1	GPS	2A	AX.89523901	7.78E+08	0.22	1.35	3.10	0.05
E1	GPM2	2A	AX.89610255	7.55E+08	0.27	686.53	3.26	0.05
E1	GPM2	2A	AX.89534203	7.54E+08	0.25	819.14	4.25	0.06
E1	GPS	2A	AX.89670038	5.24E+08	0.33	1.20	3.19	0.06
E1	GPS	2A	AX.89370001	7.47E+08	0.17	1.47	3.04	0.05
E1	GPM2	2A	AX.89762459	7.55E+08	0.27	683.92	3.30	0.05
E1	GPS	2A	AX.89390483	4.73E+08	0.33	1.26	3.46	0.06
E1	GPM2	2A	AX.89530131	7.55E+08	0.27	686.53	3.26	0.05
E1	GPS	2A	AX.89580653	7.55E+08	0.24	1.54	4.11	0.07
E1	GPS	2A	AX.89625773	7.80E+08	0.20	1.42	3.20	0.06
E1	GPM2	2A	AX.89496259	7.78E+08	0.22	870.13	4.45	0.08
E1	GPM2	2A	AX.89589865	5.96E+07	0.18	906.76	4.17	0.07
E1	GY	2A	AX.89754767	7.69E+08	0.06	-4.93	3.07	0.05
E1	GPM2	2A	AX.89542988	1.92E+08	0.48	671.35	3.89	0.07
E1	GPM2	2A	AX.89752134	7.55E+08	0.26	695.37	3.27	0.06
E1	GPM2	2A	AX.89769129	7.78E+08	0.22	837.88	4.11	0.07
E1	GPS	2A	AX.89769129	7.78E+08	0.22	1.35	3.10	0.05
E1	GPS	2A	AX.89492216	4.39E+08	0.34	1.18	3.12	0.05
E1	GPS	2A	AX.89619827	7.56E+08	0.27	1.33	3.42	0.05
E1	GPS	2A	AX.89353495	4.57E+08	0.34	1.37	4.02	0.07
E1	GPS	2A	AX.89769977	5.16E+08	0.33	1.20	3.19	0.06
E1	GPS	2A	AX.89464823	5.10E+08	0.33	1.28	3.56	0.06
E1	GPM2	2A	AX.89737871	7.55E+08	0.24	717.69	3.31	0.06
E1	GPS	2A	AX.89761868	7.56E+08	0.27	1.33	3.42	0.05
E1	GPS	2A	AX.89588999	7.55E+08	0.26	1.33	3.32	0.06
E1	GPS	2A	AX.89722052	7.79E+08	0.20	1.35	3.00	0.05
E1	GPS	2A	AX.89658500	7.55E+08	0.29	1.36	3.74	0.06
E1	GPM2	2A	AX.89451547	6.15E+07	0.22	803.47	3.82	0.07
E1	GPS	2A	AX.89620594	4.68E+08	0.34	1.21	3.26	0.06
E1	GPM2	2A	AX.89397007	7.60E+08	0.20	852.43	4.06	0.07
E1	GPM2	2A	AX.89705764	7.58E+08	0.23	735.98	3.41	0.06
E1	GPM2	2A	AX.89466371	6.04E+08	0.08	-1224.91	3.97	0.07
E1	GPM2	2A	AX.89413417	7.55E+08	0.26	695.37	3.27	0.06
E1	GPM2	2A	AX.89651445	8.39E+07	0.21	-709.87	3.05	0.05
E1	GPS	2A	AX.89332055	7.56E+08	0.25	1.40	3.54	0.06
E1	GPS	2A	AX.89727692	7.80E+08	0.20	1.42	3.20	0.06
E1	GPS	2A	AX.89565907	5.16E+08	0.33	1.20	3.19	0.06
E1	GPS	2A	AX.89623731	7.80E+08	0.20	1.36	3.01	0.05
E1	GPS	2A	AX.89408055	7.55E+08	0.26	1.39	3.62	0.06
E1	GPM2	2A	AX.89690258	7.14E+07	0.32	-650.04	3.27	0.06
E1	GY	2A	AX.89603203	7.59E+08	0.07	-4.32	3.08	0.05
E1	GPS	2A	AX.89709153	5.16E+08	0.33	1.20	3.19	0.06
E1	GPS	2A	AX.89744225	5.24E+08	0.34	1.19	3.16	0.05
E1	GY	2A	AX.89684909	7.13E+07	0.32	-2.42	3.06	0.06
E1	GPM2	2A	AX.89523901	7.78E+08	0.22	837.88	4.11	0.07
E1	GPS	2A	AX.89526254	7.56E+08	0.26	1.38	3.59	0.06
E1	GPS	2A	AX.89614134	5.17E+08	0.33	1.20	3.19	0.06
E1	GPM2	2A	AX.89601983	7.11E+07	0.31	-643.53	3.17	0.06
E1	GPS	2A	AX.89736567	5.07E+08	0.43	1.11	3.04	0.05
E1	GPM2	2A	AX.89492426	7.79E+08	0.20	905.56	4.51	0.08

E1	GPM2	2A	AX.89414865	7.55E+08	0.24	730.37	3.45	0.05
E1	GPM2	2A	AX.89658500	7.55E+08	0.29	669.50	3.30	0.05
E1	GPM2	2A	AX.89468617	7.80E+08	0.20	927.23	4.64	0.08
E1	GPS	2A	AX.89554029	7.78E+08	0.22	1.35	3.10	0.05
E1	GPS	2A	AX.89390240	7.49E+08	0.49	1.14	3.22	0.05
E1	GPS	2A	AX.89514082	7.50E+08	0.23	1.49	3.76	0.07
E1	GPM2	2A	AX.89705653	7.59E+08	0.20	852.43	4.06	0.07
E1	GPM2	2A	AX.89774365	7.53E+08	0.26	717.45	3.45	0.06
E1	GPM2	2A	AX.89431398	7.14E+07	0.43	-647.67	3.60	0.07
E1	GPM2	2A	AX.89586700	7.56E+08	0.27	677.79	3.25	0.04
E1	GPS	2A	AX.89583447	7.47E+08	0.26	1.31	3.27	0.05
E1	GPS	2A	AX.89623304	5.16E+08	0.29	1.29	3.39	0.06
E1	GPS	2A	AX.89774365	7.53E+08	0.26	1.52	4.17	0.07
E1	GPM2	2A	AX.89626514	7.61E+08	0.16	934.46	4.08	0.07
E1	GPM2	2A	AX.89741076	7.86E+07	0.29	692.94	3.50	0.06
E1	GPS	2A	AX.89527911	7.56E+08	0.25	1.40	3.54	0.06
E1	GPS	2A	AX.89496259	7.78E+08	0.22	1.43	3.46	0.06
E1	GPS	2A	AX.89584156	5.10E+08	0.33	1.28	3.56	0.06
E1	GPM2	2A	AX.89361021	7.55E+08	0.18	790.35	3.24	0.06
E1	GPM2	2A	AX.89548461	7.16E+07	0.32	-650.04	3.27	0.06
E1	GPS	2A	AX.89690325	5.13E+08	0.33	1.17	3.03	0.05
E1	GPS	2A	AX.89490527	7.78E+08	0.25	1.37	3.44	0.06
E1	GPM2	2A	AX.89572224	7.59E+08	0.20	852.43	4.06	0.07
E1	GPS	2A	AX.89718253	7.78E+08	0.22	1.35	3.10	0.05
E1	GPS	2A	AX.89657875	5.24E+08	0.33	1.25	3.39	0.06
E1	GPM2	2A	AX.89719826	7.61E+08	0.20	888.57	4.30	0.08
E1	GPM2	2A	AX.89552048	8.39E+07	0.21	-709.87	3.05	0.05
E1	GPM2	2A	AX.89565512	7.79E+08	0.20	828.40	3.75	0.07
E1	GPM2	2A	AX.89637021	8.46E+07	0.23	-710.38	3.17	0.05
E1	GPM2	2A	AX.89527911	7.56E+08	0.25	699.99	3.24	0.05
E1	GY	2A	AX.89508618	8.56E+07	0.07	-4.82	3.53	0.07
E1	GPS	2A	AX.89586376	7.55E+08	0.27	1.49	4.19	0.07
E1	GPS	2A	AX.89451429	7.55E+08	0.24	1.47	3.77	0.07
E1	GPM2	2A	AX.89547326	6.04E+08	0.09	-1132.54	3.76	0.07
E1	GPS	2A	AX.89413222	7.80E+08	0.21	1.39	3.20	0.06
E1	GPM2	2A	AX.89332386	7.59E+08	0.20	852.43	4.06	0.07
E1	GPS	2A	AX.89547112	5.09E+08	0.33	1.17	3.03	0.05
E1	GPS	2A	AX.89400174	7.79E+08	0.21	1.39	3.20	0.06
E1	GPS	2A	AX.89643216	7.55E+08	0.27	1.39	3.71	0.06
E1	GPM2	2A	AX.89434666	8.63E+07	0.23	-714.47	3.24	0.06
E1	GPM2	2A	AX.89386633	7.12E+07	0.43	-647.67	3.60	0.07
E1	GPS	2A	AX.89754385	5.17E+08	0.33	1.20	3.19	0.06
E1	GPS	2A	AX.89340155	7.56E+08	0.25	1.40	3.54	0.06
E1	GPM2	2A	AX.89614700	7.78E+08	0.22	837.88	4.11	0.07
E1	GPM2	2A	AX.89505306	7.16E+07	0.32	-650.04	3.27	0.06
E1	GPM2	2A	AX.89551189	4.17E+08	0.17	774.99	3.07	0.05
E1	GPS	2A	AX.89545729	7.79E+08	0.22	1.53	3.88	0.07
E1	GPM2	2A	AX.89483238	7.59E+08	0.20	899.92	4.33	0.08
E1	GY	2A	AX.89745404	8.42E+07	0.06	-6.32	5.04	0.10
E1	GY	2A	AX.89745404	8.42E+07	0.06	-6.32	5.04	0.10

E1	GPM2	2A	AX.89372948	7.79E+08	0.20	789.02	3.50	0.06
E1	GPS	2A	AX.89620260	5.16E+08	0.33	1.20	3.19	0.06
E1	GPS	2A	AX.89586700	7.56E+08	0.27	1.39	3.71	0.06
E1	GPM2	2A	AX.89378004	8.39E+07	0.21	-709.87	3.05	0.05
E1	GPM2	2A	AX.89451429	7.55E+08	0.24	717.69	3.31	0.06
E1	GPM2	2A	AX.89497975	7.26E+07	0.40	-621.93	3.29	0.06
E1	GPS	2A	AX.89623031	5.11E+08	0.33	1.17	3.03	0.05
E1	GPS	2A	AX.89492426	7.79E+08	0.20	1.36	3.01	0.05
E1	GPS	2A	AX.89762500	5.10E+08	0.33	1.33	3.77	0.07
E1	GPS	2A	AX.89704050	7.69E+08	0.20	-1.54	3.75	0.06
E1	GPM2	2A	AX.89472684	1.85E+08	0.25	819.20	4.30	0.08
E1	GPM2	2A	AX.89459185	7.60E+08	0.20	852.43	4.06	0.07
E1	GPS	2A	AX.89601425	7.56E+08	0.27	1.37	3.55	0.06
E1	GPM2	2A	AX.89531056	8.45E+07	0.23	-710.38	3.17	0.05
E1	GPM2	2A	AX.89422120	7.59E+08	0.20	852.43	4.06	0.07
E1	GY	2A	AX.89543557	8.49E+07	0.23	-2.96	3.60	0.06
E1	GPS	2A	AX.89711226	7.78E+08	0.22	1.43	3.46	0.06
E1	GPS	2A	AX.89554733	7.55E+08	0.27	1.43	3.93	0.07
E1	GPM2	2A	AX.89390161	7.14E+07	0.43	-647.67	3.60	0.07
E1	GPS	2A	AX.89492206	7.47E+08	0.26	1.31	3.27	0.05
E1	GPS	2A	AX.89728983	7.55E+08	0.26	1.43	3.76	0.07
E1	GPS	2A	AX.89622104	7.78E+08	0.22	1.35	3.10	0.05
E1	GPM2	2A	AX.89375310	6.21E+07	0.25	-668.63	3.03	0.05
E1	GPM2	2A	AX.89531704	7.78E+08	0.22	837.88	4.11	0.07
E1	GPM2	2A	AX.89412752	7.59E+08	0.20	852.43	4.06	0.07
E1	GPS	2A	AX.89538983	7.47E+08	0.26	1.31	3.27	0.05
E1	GPS	2A	AX.89311284	4.34E+08	0.34	1.18	3.12	0.05
E1	GPS	2A	AX.89737649	7.55E+08	0.24	1.47	3.77	0.07
E1	GPS	2A	AX.89560842	7.55E+08	0.30	1.31	3.56	0.06
E1	GPS	2A	AX.89603261	4.19E+08	0.37	1.25	3.53	0.06
E1	GPS	2A	AX.89592393	7.56E+08	0.27	1.39	3.71	0.06
E1	GPS	2A	AX.89557781	7.55E+08	0.26	1.43	3.76	0.07
E1	GPM2	2A	AX.89353071	8.49E+07	0.23	-710.38	3.17	0.05
E1	GPS	2A	AX.89444190	7.80E+08	0.20	1.42	3.20	0.06
E1	GPS	2A	AX.89570234	7.55E+08	0.29	1.29	3.35	0.05
E1	GPS	2A	AX.89693872	7.80E+08	0.20	1.42	3.20	0.06
E1	GPM2	2A	AX.89424098	7.70E+08	0.19	-766.41	3.18	0.05
E1	GPM2	2A	AX.89693872	7.80E+08	0.20	927.23	4.64	0.08
E1	GPS	2A	AX.89330185	7.80E+08	0.21	1.39	3.20	0.06
E1	GPS	2A	AX.89425443	7.50E+08	0.23	1.49	3.76	0.07
E1	GPS	2A	AX.89626597	7.78E+08	0.22	1.35	3.10	0.05
E1	GY	2A	AX.89452906	8.64E+07	0.23	-3.05	3.80	0.07
E1	GY	2A	AX.89313648	3.12E+07	0.10	3.84	3.22	0.05
E1	GY	2A	AX.89654740	1.07E+08	0.13	-3.29	3.04	0.04
E1	GPS	2A	AX.89622382	7.55E+08	0.20	1.37	3.08	0.05
E1	GPS	2A	AX.89335578	7.56E+08	0.27	1.37	3.55	0.06
E1	GPS	2A	AX.89577627	7.56E+08	0.27	1.37	3.55	0.06
E1	GPM2	2A	AX.89645117	7.13E+07	0.43	-647.67	3.60	0.07
E1	GPS	2A	AX.89386972	5.24E+08	0.33	1.25	3.39	0.06
E1	GPS	2A	AX.89379833	7.55E+08	0.27	1.33	3.42	0.06

E1	GPS	2A	AX.89762459	7.55E+08	0.27	1.39	3.71	0.06
E1	GPS	2A	AX.89318322	5.10E+08	0.32	1.22	3.23	0.06
E1	GPM2	2A	AX.89646006	7.56E+08	0.26	695.68	3.27	0.04
E1	GPM2	2A	AX.89347674	7.56E+08	0.26	695.68	3.27	0.04
E1	GPM2	2A	AX.89766512	7.78E+08	0.22	837.88	4.11	0.07
E1	GPM2	2A	AX.89712759	8.50E+07	0.23	-710.38	3.17	0.05
E1	GPM2	2A	AX.89778038	7.79E+08	0.21	948.49	4.97	0.09
E1	GY	2A	AX.89336730	7.40E+08	0.09	-4.19	3.34	0.05
E1	GPS	2A	AX.89575538	7.55E+08	0.26	1.43	3.76	0.07
E1	GPM2	2A	AX.89609674	7.11E+07	0.32	-650.04	3.27	0.06
E1	GPS	2A	AX.89644656	7.53E+08	0.29	1.32	3.50	0.06
E1	GPM2	2A	AX.89600036	7.70E+08	0.19	-766.41	3.18	0.05
E1	GPS	2A	AX.89575335	5.15E+08	0.33	1.25	3.39	0.06
E1	GPM2	2A	AX.89638704	7.59E+08	0.21	795.83	3.71	0.07
E1	GPS	2A	AX.89496843	7.78E+08	0.29	1.39	3.85	0.06
E1	GPM2	2A	AX.89625773	7.80E+08	0.20	927.23	4.64	0.08
E1	GPM2	2A	AX.89677955	8.42E+07	0.20	-777.92	3.42	0.06
E1	GPM2	2A	AX.89334194	6.03E+08	0.11	-1200.04	4.64	0.09
E1	GPM2	2A	AX.89332055	7.56E+08	0.25	699.99	3.24	0.05
E1	GPM2	2A	AX.89557781	7.55E+08	0.26	695.37	3.27	0.06
E1	GPM2	2A	AX.89414077	8.46E+07	0.07	-1294.07	3.57	0.07
E1	GY	2A	AX.89776905	8.42E+07	0.21	-3.01	3.51	0.06
E1	GY	2A	AX.89690258	7.14E+07	0.32	-2.42	3.06	0.06
E1	GPM2	2A	AX.89731936	7.59E+08	0.20	852.43	4.06	0.07
E1	GPM2	2A	AX.89617767	7.14E+07	0.33	-687.76	3.63	0.07
E1	GPM2	2A	AX.89452906	8.64E+07	0.23	-702.76	3.11	0.05
E1	GPM2	2A	AX.89740120	7.58E+08	0.23	735.98	3.41	0.06
E1	GPS	2A	AX.89317882	7.55E+08	0.24	1.47	3.77	0.07
E1	GY	2A	AX.89479590	7.14E+07	0.32	-2.42	3.06	0.06
E1	GPM2	2A	AX.89592262	7.53E+08	0.41	695.26	4.02	0.06
E1	GPM2	2A	AX.89549965	6.04E+08	0.08	-1224.91	3.97	0.07
E1	GPM2	2A	AX.89352998	7.11E+07	0.32	-650.04	3.27	0.06
E1	GPM2	2A	AX.89717864	7.59E+08	0.21	841.02	4.02	0.07
E1	GPM2	2A	AX.89733650	7.55E+08	0.27	683.92	3.30	0.05
E1	GPM2	2A	AX.89489684	7.14E+07	0.32	-654.74	3.29	0.06
E1	GPM2	2A	AX.89728983	7.55E+08	0.26	695.37	3.27	0.06
E1	GPM2	2A	AX.89513724	7.55E+08	0.24	717.69	3.31	0.06
E1	GY	2A	AX.89670276	7.51E+08	0.06	-5.10	3.47	0.07
E1	GPM2	2A	AX.89440533	7.55E+08	0.27	680.27	3.21	0.05
E1	GY	2A	AX.89768688	8.39E+07	0.21	-3.01	3.56	0.06
E1	GPM2	2A	AX.89461565	8.50E+07	0.23	-710.38	3.17	0.05
E1	GPM2	2A	AX.89458867	7.59E+08	0.20	852.43	4.06	0.07
E1	GPM2	2A	AX.89665919	7.58E+08	0.17	922.20	4.15	0.07
E1	GPS	2A	AX.89753930	5.10E+08	0.33	1.19	3.13	0.05
E1	GPM2	2A	AX.89604900	6.36E+08	0.33	-630.46	3.13	0.05
E1	GPM2	2A	AX.89514959	6.03E+08	0.09	-1132.54	3.76	0.07
E1	GY	2A	AX.89721986	8.63E+07	0.07	-4.82	3.53	0.07
E1	GPM2	2A	AX.89498193	7.78E+08	0.21	748.03	3.29	0.06
E1	GPM2	2A	AX.89492742	7.80E+08	0.20	927.23	4.64	0.08
E1	GPM2	2A	AX.89561705	7.80E+08	0.20	927.23	4.64	0.08

E1	GPM2	2A	AX.89490792	7.14E+07	0.43	-647.67	3.60	0.07
E1	GY	2A	AX.89688659	8.53E+07	0.23	-3.06	3.85	0.07
E1	GPM2	2A	AX.89636379	7.58E+08	0.24	720.64	3.33	0.06
E1	GY	2A	AX.89637021	8.46E+07	0.23	-2.96	3.60	0.06
E1	GPM2	2A	AX.89502294	7.59E+08	0.21	737.14	3.21	0.06
E1	GY	2A	AX.89353712	8.63E+07	0.24	-2.84	3.44	0.06
E1	GPM2	2A	AX.89434520	8.39E+07	0.21	-709.87	3.05	0.05
E1	GPM2	2A	AX.89519604	7.14E+07	0.43	-647.67	3.60	0.07
E1	GPM2	2A	AX.89554029	7.78E+08	0.22	837.88	4.11	0.07
E1	GPM2	2A	AX.89750010	7.56E+08	0.26	709.09	3.42	0.05
E1	GPM2	2A	AX.89625199	7.78E+08	0.21	748.03	3.29	0.06
E1	GPM2	2A	AX.89700518	7.11E+07	0.43	-649.06	3.61	0.07
E1	GPM2	2A	AX.89503441	7.70E+08	0.19	-766.41	3.18	0.05
E1	GPM2	2A	AX.89495417	8.39E+07	0.21	-709.87	3.05	0.05
E1	GPM2	2A	AX.89330185	7.80E+08	0.21	948.49	4.97	0.09
E1	GPM2	2A	AX.89317882	7.55E+08	0.24	717.69	3.31	0.06
E1	GPM2	2A	AX.89569793	8.46E+07	0.07	-1294.07	3.57	0.07
E1	GPM2	2A	AX.89468956	8.39E+07	0.21	-709.87	3.05	0.05
E1	GPM2	2A	AX.89402644	8.45E+07	0.23	-710.38	3.17	0.05
E1	GPM2	2A	AX.89750786	8.46E+07	0.23	-710.38	3.17	0.05
E1	GPM2	2A	AX.89344976	7.56E+08	0.28	711.95	3.60	0.05
E1	GPM2	2A	AX.89463332	6.40E+07	0.32	673.85	3.48	0.06
E1	GPM2	2A	AX.89543557	8.49E+07	0.23	-710.38	3.17	0.05
E1	GPM2	2A	AX.89462856	7.58E+08	0.23	735.98	3.41	0.06
E1	GPM2	2A	AX.89738181	7.58E+08	0.20	899.92	4.33	0.08
E1	GPM2	2A	AX.89699881	7.14E+07	0.32	-650.04	3.27	0.06
E1	GPM2	2A	AX.89711226	7.78E+08	0.22	870.13	4.45	0.08
E1	GPM2	2A	AX.89542005	6.93E+08	0.19	-831.67	3.65	0.06
E1	GY	2A	AX.89765361	7.53E+08	0.10	4.12	3.63	0.06
E1	GY	2A	AX.89765361	7.53E+08	0.10	4.12	3.63	0.06
E1	GPM2	2A	AX.89636460	7.55E+08	0.26	695.37	3.27	0.06
E1	GPM2	2A	AX.89381894	7.78E+08	0.21	748.03	3.29	0.06
E1	GPM2	2A	AX.89463340	8.63E+07	0.23	-714.47	3.24	0.06
E1	GPM2	2A	AX.89463683	7.10E+07	0.32	-650.04	3.27	0.06
E1	GPM2	2A	AX.89479590	7.14E+07	0.32	-650.04	3.27	0.06
E1	GY	2A	AX.89728140	6.78E+08	0.49	2.35	3.26	0.05
E1	GPM2	2A	AX.89424726	8.50E+07	0.23	-710.38	3.17	0.05
E1	GY	2A	AX.89542005	6.93E+08	0.19	-3.86	5.03	0.08
E1	GY	2A	AX.89461565	8.50E+07	0.23	-2.96	3.60	0.06
E1	GY	2A	AX.89324170	7.69E+08	0.06	-4.93	3.07	0.05
E1	GY	2A	AX.89750786	8.46E+07	0.23	-2.96	3.60	0.06
E1	GY	2A	AX.89706225	8.42E+07	0.07	-5.54	4.26	0.08
E1	GPM2	2A	AX.89453425	6.15E+07	0.21	812.89	3.79	0.07
E1	GPM2	2A	AX.89568492	7.58E+08	0.19	916.62	4.40	0.08
E1	GPM2	2A	AX.89317561	7.14E+07	0.44	-595.68	3.13	0.06
E1	GPM2	2A	AX.89549770	7.59E+08	0.20	852.43	4.06	0.07
E1	GPM2	2A	AX.89460601	7.61E+08	0.23	717.31	3.22	0.06
E1	GPM2	2A	AX.89455016	7.14E+07	0.43	-647.67	3.60	0.07
E1	GPM2	2A	AX.89402750	7.79E+08	0.20	905.56	4.51	0.08
E1	GY	2A	AX.89495417	8.39E+07	0.21	-3.01	3.56	0.06

E1	GPM2	2A	AX.89457808	7.69E+08	0.20	-896.39	4.43	0.07
E1	GPM2	2A	AX.89539167	7.55E+08	0.27	677.79	3.25	0.04
E1	GPM2	2A	AX.89575155	7.58E+08	0.23	735.98	3.41	0.06
E1	GPM2	2A	AX.89601425	7.56E+08	0.27	680.27	3.21	0.05
E1	GPM2	2A	AX.89580653	7.55E+08	0.24	780.68	3.82	0.07
E1	GPM2	2A	AX.89573905	7.55E+08	0.24	780.68	3.82	0.07
E1	GPM2	2A	AX.89340155	7.56E+08	0.25	699.99	3.24	0.05
E1	GPM2	2A	AX.89745404	8.42E+07	0.06	-1455.75	4.12	0.08
E1	GPM2	2A	AX.89348730	8.50E+07	0.23	-710.38	3.17	0.05
E1	GPM2	2A	AX.89468552	7.79E+08	0.20	905.56	4.51	0.08
E1	GPM2	2A	AX.86169701	7.76E+08	0.10	-958.25	3.04	0.05
E1	GPM2	2A	AX.89552978	7.61E+08	0.19	850.14	3.80	0.07
E1	GPM2	2A	AX.89389069	7.58E+08	0.23	735.98	3.41	0.06
E1	GPM2	2A	AX.89313163	6.03E+08	0.09	-1132.54	3.76	0.07
E1	GPM2	2A	AX.89442869	7.55E+08	0.24	717.69	3.31	0.06
E1	GPM2	2A	AX.89702562	7.10E+07	0.32	-650.04	3.27	0.06
E1	GPM2	2A	AX.89711069	7.14E+07	0.33	-670.62	3.47	0.07
E1	GY	2A	AX.89677955	8.42E+07	0.20	-3.03	3.45	0.06
E1	GPM2	2A	AX.89331067	7.76E+08	0.09	-1055.49	3.20	0.05
E1	GPM2	2A	AX.89406193	7.59E+08	0.20	852.43	4.06	0.07
E1	GPM2	2A	AX.89445758	7.79E+08	0.20	905.56	4.51	0.08
E1	GPM2	2A	AX.89372440	7.10E+07	0.43	-647.67	3.60	0.07
E1	GPM2	2A	AX.89602806	8.46E+07	0.23	-710.38	3.17	0.05
E1	GPM2	2A	AX.89755851	7.60E+08	0.17	861.57	3.68	0.06
E1	GPM2	2A	AX.89493190	7.78E+08	0.31	633.90	3.07	0.05
E1	GPM2	2A	AX.89694063	5.84E+07	0.30	748.76	4.06	0.07
E1	GPM2	2A	AX.89631438	7.59E+08	0.20	852.43	4.06	0.07
E1	GPM2	2A	AX.89354221	7.56E+08	0.25	699.99	3.24	0.05
E1	GPM2	2A	AX.89431192	7.14E+07	0.32	-650.04	3.27	0.06
E1	GPM2	2A	AX.89385952	7.15E+07	0.32	-650.04	3.27	0.06
E1	GPM2	2A	AX.89621355	7.59E+08	0.20	852.43	4.06	0.07
E1	GPM2	2A	AX.89549784	8.53E+07	0.22	-698.62	3.05	0.05
E1	GPM2	2A	AX.89517936	7.10E+07	0.32	-650.04	3.27	0.06
E1	GPS	2A	AX.89451660	5.09E+08	0.32	1.22	3.23	0.06
E1	GPS	2A	AX.89756875	4.67E+08	0.33	1.26	3.46	0.06
E1	GPM2	2A	AX.89526331	7.78E+08	0.22	837.88	4.11	0.07
E1	GPM2	2A	AX.89704050	7.69E+08	0.20	-896.39	4.43	0.07
E1	GPM2	2A	AX.89395191	6.93E+08	0.17	-794.91	3.15	0.05
E1	GY	2A	AX.89536127	7.69E+08	0.06	-4.93	3.07	0.05
E1	GPS	2A	AX.89352382	7.47E+08	0.20	1.54	3.64	0.06
E1	GPM2	2A	AX.89425114	8.46E+07	0.23	-710.38	3.17	0.05
E1	GPM2	2A	AX.89583054	7.80E+08	0.21	948.49	4.97	0.09
E1	GPM2	2A	AX.89770581	6.15E+07	0.20	826.82	3.79	0.07
E1	GPM2	2A	AX.89397285	7.80E+08	0.20	927.23	4.64	0.08
E1	GPM2	2A	AX.89569105	7.76E+08	0.08	-1056.70	3.08	0.05
E1	GPS	2A	AX.89732786	7.79E+08	0.21	1.39	3.20	0.06
E1	GPS	2A	AX.89655029	7.55E+08	0.35	1.27	3.59	0.06
E1	GPS	2A	AX.89621466	5.11E+08	0.33	1.17	3.03	0.05
E1	GPM2	2A	AX.89531707	7.58E+08	0.22	732.77	3.30	0.06
E1	GPM2	2A	AX.89610686	8.42E+07	0.06	-1385.30	3.78	0.07

E1	GPS	2A	AX.89757261	7.69E+08	0.21	-1.47	3.50	0.06
E1	GPS	2A	AX.89636229	5.08E+08	0.32	1.22	3.23	0.06
E1	GPM2	2A	AX.89613544	7.78E+08	0.22	837.88	4.11	0.07
E1	GPM2	2A	AX.89491406	7.60E+08	0.20	852.43	4.06	0.07
E1	GPS	2A	AX.89733650	7.55E+08	0.27	1.39	3.71	0.06
E1	GPS	2A	AX.89374034	7.55E+08	0.26	1.39	3.62	0.06
E1	GY	2A	AX.89777060	8.39E+07	0.21	-3.01	3.56	0.06
E1	GPM2	2A	AX.89684909	7.13E+07	0.32	-650.04	3.27	0.06
E1	GPM2	2A	AX.89401600	7.13E+07	0.43	-647.67	3.60	0.07
E1	GPS	2A	AX.89516661	7.56E+08	0.27	1.39	3.71	0.06
E1	GPM2	2A	AX.89449290	6.15E+07	0.22	803.47	3.82	0.07
E1	GPM2	2A	AX.89491754	7.58E+08	0.20	899.92	4.33	0.08
E1	GPS	2A	AX.89461692	5.09E+08	0.33	1.26	3.47	0.06
E1	GPS	2A	AX.89413101	5.23E+08	0.33	1.16	3.01	0.05
E1	GPM2	2A	AX.89473269	6.52E+08	0.05	-1414.64	3.43	0.06
E1	GPS	2A	AX.89614230	7.55E+08	0.21	1.53	3.75	0.07
E1	GPM2	2A	AX.89534141	7.55E+08	0.27	664.85	3.12	0.05
E1	GPS	2A	AX.89440533	7.55E+08	0.27	1.37	3.55	0.06
E1	GPM2	2A	AX.89592393	7.56E+08	0.27	677.79	3.25	0.04
E1	GPM2	2A	AX.89657141	7.60E+08	0.20	824.25	3.83	0.07
E1	GPM2	2A	AX.89510338	7.76E+08	0.08	-1056.70	3.08	0.05
E1	GPM2	2A	AX.89429525	8.42E+07	0.07	-1294.07	3.57	0.07
E1	GPS	2A	AX.89413417	7.55E+08	0.26	1.43	3.76	0.07
E1	GPM2	2A	AX.89408757	7.11E+07	0.43	-647.67	3.60	0.07
E1	GPM2	2A	AX.89526254	7.56E+08	0.26	665.29	3.06	0.05
E1	GPS	2A	AX.89700904	7.53E+08	0.20	1.56	3.77	0.07
E1	GPM2	2A	AX.89487768	7.56E+08	0.24	685.15	3.09	0.05
E1	GPM2	2A	AX.89627848	7.78E+08	0.25	889.26	4.97	0.09
E1	GPS	2A	AX.89503042	5.24E+08	0.33	1.20	3.19	0.06
E1	GPM2	2A	AX.89757261	7.69E+08	0.21	-905.63	4.58	0.08
E1	GPM2	2A	AX.89369914	7.59E+08	0.20	852.43	4.06	0.07
E1	GPS	2A	AX.89452594	7.55E+08	0.26	1.39	3.62	0.06
E1	GPM2	2A	AX.89444190	7.80E+08	0.20	927.23	4.64	0.08
E1	GPM2	2A	AX.89336730	7.40E+08	0.09	-1016.82	3.00	0.05
E1	GPM2	2A	AX.89768688	8.39E+07	0.21	-709.87	3.05	0.05
E1	GPM2	2A	AX.89379457	7.60E+08	0.20	888.57	4.30	0.08
E1	GPS	2A	AX.89464739	7.47E+08	0.26	1.31	3.27	0.05
E1	GPS	2A	AX.89489555	7.53E+08	0.21	1.57	3.92	0.07
E1	GPM2	2A	AX.89736839	6.68E+07	0.17	828.14	3.44	0.06
E1	GPM2	2A	AX.89622104	7.78E+08	0.22	837.88	4.11	0.07
E1	GPM2	2A	AX.89705624	7.59E+08	0.17	902.79	3.92	0.07
E1	GPM2	2A	AX.89552229	7.60E+08	0.20	852.43	4.06	0.07
E1	GPM2	2A	AX.89631377	7.79E+08	0.20	860.25	4.06	0.07
E1	GPM2	2A	AX.89451631	6.23E+07	0.23	754.65	3.52	0.05
E1	GPM2	2A	AX.89371398	7.10E+07	0.43	-647.67	3.60	0.07
E1	GPM2	2A	AX.89475215	6.04E+08	0.09	-1132.54	3.76	0.07
E1	GPM2	2A	AX.89466871	7.60E+08	0.20	888.57	4.30	0.08
E1	GPM2	2A	AX.89320685	7.61E+08	0.20	888.57	4.30	0.08
E1	GPM2	2A	AX.89334859	7.61E+08	0.20	888.57	4.30	0.08
E1	GPM2	2A	AX.89553523	8.63E+07	0.23	-714.47	3.24	0.06

E1	GPS	2A	AX.89693999	7.78E+08	0.22	1.35	3.10	0.05
E1	GPM2	2A	AX.89382126	7.10E+07	0.46	-668.20	3.84	0.07
E1	GY	2A	AX.89628287	8.42E+07	0.06	-6.32	5.04	0.10
E1	GPM2	2A	AX.89554733	7.55E+08	0.27	653.08	3.05	0.05
E1	GPM2	2A	AX.89643216	7.55E+08	0.27	683.92	3.30	0.05
E1	GPS	2A	AX.89509268	5.16E+08	0.33	1.20	3.19	0.06
E1	TKW	2A	AX.89345671	4.28E+07	0.12	-1.50	4.01	0.07
E1	GPM2	2A	AX.89626597	7.78E+08	0.22	837.88	4.11	0.07
E1	TKW	2A	AX.89581383	3.19E+07	0.27	-0.98	3.28	0.06
E1	GPM2	2A	AX.89432407	8.56E+07	0.23	-714.47	3.24	0.06
E1	TKW	2A	AX.89562993	7.55E+08	0.26	-1.06	3.68	0.06
E1	GPM2	2A	AX.89462038	1.93E+08	0.48	671.35	3.89	0.07
E1	TKW	2A	AX.89359914	7.61E+08	0.26	-1.12	4.01	0.07
E1	GPM2	2A	AX.89693999	7.78E+08	0.22	837.88	4.11	0.07
E1	GPM2	2A	AX.89742627	7.54E+08	0.21	838.62	4.00	0.07
E1	GPM2	2A	AX.89502868	7.11E+07	0.32	-654.74	3.29	0.06
E1	GPM2	2A	AX.89503320	7.78E+08	0.22	837.88	4.11	0.07
E1	TKW	2A	AX.89568492	7.58E+08	0.19	-1.06	3.02	0.05
E1	GPM2	2A	AX.89777060	8.39E+07	0.21	-709.87	3.05	0.05
E1	TKW	2A	AX.89365566	7.55E+08	0.26	-1.06	3.68	0.06
E1	GPM2	2A	AX.89322846	7.80E+08	0.20	927.23	4.64	0.08
E1	GPM2	2A	AX.89678558	7.58E+08	0.23	735.98	3.41	0.06
E1	GPS	2A	AX.89552978	7.61E+08	0.19	1.51	3.41	0.06
E1	GPM2	2A	AX.89614230	7.55E+08	0.21	880.45	4.36	0.08
E1	GPS	2A	AX.89624783	7.47E+08	0.21	1.36	3.11	0.05
E1	TKW	2A	AX.89573905	7.55E+08	0.24	-1.10	3.69	0.06
E1	TKW	2A	AX.89689821	7.55E+08	0.24	-1.03	3.36	0.05
E1	GPM2	2A	AX.89617723	8.41E+07	0.21	-709.87	3.05	0.05
E1	TKW	2A	AX.89575538	7.55E+08	0.26	-1.07	3.67	0.06
E1	GPM2	2A	AX.89697473	7.78E+08	0.20	778.95	3.42	0.06
E1	GPM2	2A	AX.89551770	8.39E+07	0.21	-709.87	3.05	0.05
E1	GPM2	2A	AX.89710998	7.59E+08	0.20	852.43	4.06	0.07
E1	TKW	2A	AX.89586614	4.32E+07	0.12	-1.46	3.82	0.06
E1	GPM2	2A	AX.89637508	7.55E+08	0.27	686.53	3.26	0.05
E1	TKW	2A	AX.89442869	7.55E+08	0.24	-0.99	3.10	0.05
E1	GPM2	2A	AX.89358011	7.13E+07	0.43	-649.06	3.61	0.07
E1	GY	2A	AX.89776905	8.42E+07	0.21	-3.01	3.51	0.06
E1	GPM2	2A	AX.89335578	7.56E+08	0.27	680.27	3.21	0.04
E1	TKW	2A	AX.89554733	7.55E+08	0.27	-0.98	3.32	0.06
E1	TKW	2A	AX.89348439	7.57E+08	0.39	0.90	3.31	0.06
E1	GPM2	2A	AX.89343951	7.61E+08	0.20	888.57	4.30	0.08
E1	GPM2	2A	AX.89622756	7.79E+08	0.20	905.56	4.51	0.08
E1	TKW	2A	AX.89577627	7.56E+08	0.27	-0.99	3.30	0.06
E1	GPM2	2A	AX.89700904	7.53E+08	0.20	827.83	3.80	0.07
E1	GPM2	2A	AX.89426786	7.79E+08	0.21	948.49	4.97	0.09
E1	GPM2	2A	AX.89310582	7.59E+08	0.20	852.43	4.06	0.07
E1	GPM2	2A	AX.89668676	7.16E+07	0.43	-647.67	3.60	0.07
E1	TKW	2A	AX.89570234	7.55E+08	0.29	-0.96	3.30	0.06
E1	GPM2	2A	AX.89564635	7.16E+07	0.43	-647.67	3.60	0.07
E1	GY	2A	AX.89716272	7.14E+07	0.32	-2.42	3.06	0.06

E1	GPM2	2A	AX.89488613	8.56E+07	0.07	-1294.07	3.57	0.07
E1	GPM2	2A	AX.89436162	7.59E+08	0.21	747.24	3.33	0.06
E1	GPM2	2A	AX.89417730	7.14E+07	0.32	-650.04	3.27	0.06
E1	TKW	2A	AX.89601729	3.19E+07	0.28	-0.97	3.26	0.06
E1	GPM2	2A	AX.89545729	7.79E+08	0.22	939.60	5.03	0.09
E1	GPM2	2A	AX.89718253	7.78E+08	0.22	837.88	4.11	0.07
E1	TKW	2A	AX.89457700	7.58E+08	0.23	-1.02	3.20	0.05
E1	GPM2	2A	AX.89682243	7.43E+08	0.32	662.57	3.36	0.05
E1	GPM2	2A	AX.89721796	6.03E+08	0.09	-1132.54	3.76	0.07
E1	TKW	2A	AX.89586376	7.55E+08	0.27	-1.02	3.48	0.06
E1	GPM2	2A	AX.89747863	7.55E+08	0.27	683.92	3.30	0.05
E1	GPM2	2A	AX.89716272	7.14E+07	0.32	-650.04	3.27	0.06
E1	GPM2	2A	AX.89311260	7.14E+07	0.43	-647.67	3.60	0.07
E1	TKW	2A	AX.89374034	7.55E+08	0.26	-1.06	3.68	0.06
E1	GPM2	2A	AX.89654143	7.58E+08	0.22	808.93	3.92	0.07
E1	GPM2	2A	AX.89400174	7.79E+08	0.21	948.49	4.97	0.09
E1	GPM2	2A	AX.89424046	7.60E+08	0.20	888.57	4.30	0.08
E1	GY	2A	AX.89688659	8.53E+07	0.23	-3.06	3.85	0.07
E1	GPM2	2A	AX.89632373	8.45E+07	0.23	-710.38	3.17	0.05
E1	GPM2	2A	AX.89702439	7.61E+08	0.20	888.57	4.30	0.08
E1	GPM2	2A	AX.89534013	6.04E+08	0.09	-1034.72	3.09	0.06
E1	GPM2	2A	AX.89335443	6.04E+08	0.09	-1132.54	3.76	0.07
E1	GPM2	2A	AX.89420289	7.10E+07	0.32	-650.04	3.27	0.06
E1	GPM2	2A	AX.89706460	7.55E+08	0.21	880.45	4.36	0.08
E1	GPM2	2A	AX.89425537	8.42E+07	0.21	-709.87	3.05	0.05
E1	GY	2A	AX.89495219	8.50E+07	0.23	-2.96	3.60	0.06
E1	GPM2	2A	AX.89527155	7.78E+08	0.22	837.88	4.11	0.07
E1	GPM2	2A	AX.89591770	7.56E+08	0.24	681.17	3.06	0.04
E1	GPM2	2A	AX.89742073	7.76E+08	0.08	-1056.70	3.08	0.05
E1	GPM2	2A	AX.89775602	7.10E+07	0.32	-650.04	3.27	0.06
E1	TKW	2A	AX.89737871	7.55E+08	0.24	-0.99	3.10	0.05
E1	GPM2	2A	AX.89577287	6.03E+08	0.09	-1095.03	3.41	0.05
E1	GPM2	2A	AX.89685749	7.59E+08	0.20	852.43	4.06	0.07
E1	TKW	2A	AX.89584181	4.28E+07	0.13	-1.41	3.68	0.06
E1	GPM2	2A	AX.89688659	8.53E+07	0.23	-714.47	3.24	0.06
E1	GPM2	2A	AX.89442084	7.16E+07	0.32	-650.04	3.27	0.06
E1	GPM2	2A	AX.89489555	7.53E+08	0.21	814.97	3.81	0.07
E1	GPM2	2A	AX.89608550	8.46E+07	0.23	-710.38	3.17	0.05
E1	TKW	2A	AX.89452594	7.55E+08	0.26	-1.06	3.68	0.06
E1	GPM2	2A	AX.89737649	7.55E+08	0.24	717.69	3.31	0.06
E1	GY	2A	AX.89542005	6.93E+08	0.19	-3.86	5.03	0.08
E1	TKW	2A	AX.89359686	7.17E+08	0.47	0.88	3.35	0.06
E1	TKW	2A	AX.89771272	7.55E+08	0.24	-0.99	3.10	0.05
E1	TKW	2A	AX.89530198	3.18E+07	0.28	-0.93	3.04	0.05
E1	TKW	2A	AX.89408055	7.55E+08	0.26	-1.06	3.68	0.06
E1	TKW	2A	AX.89733650	7.55E+08	0.27	-0.98	3.28	0.06
E1	TKW	2A	AX.89317882	7.55E+08	0.24	-0.99	3.10	0.05
E1	TKW	2A	AX.89630473	7.58E+08	0.12	1.41	3.50	0.06
E1	TKW	2A	AX.89389069	7.58E+08	0.23	-1.02	3.20	0.05
E1	TKW	2A	AX.89530584	7.56E+08	0.26	-1.01	3.35	0.06

E1	TKW	2A	AX.89539167	7.55E+08	0.27	-1.09	3.95	0.07
E1	TKW	2A	AX.89675694	3.18E+07	0.28	-1.01	3.56	0.06
E1	TKW	2A	AX.89627848	7.78E+08	0.25	-0.99	3.21	0.05
E1	TKW	2A	AX.89580653	7.55E+08	0.24	-1.10	3.69	0.06
E1	TKW	2A	AX.89655029	7.55E+08	0.35	-1.03	4.05	0.07
E1	TKW	2A	AX.89752134	7.55E+08	0.26	-1.07	3.67	0.06
E1	TKW	2A	AX.89530131	7.55E+08	0.27	-1.11	4.04	0.07
E1	TKW	2A	AX.89553707	7.60E+08	0.22	-1.03	3.21	0.05
E1	TKW	2A	AX.89658500	7.55E+08	0.29	-1.05	3.87	0.07
E1	TKW	2A	AX.89375933	7.55E+08	0.26	-1.06	3.68	0.06
E1	TKW	2A	AX.89643216	7.55E+08	0.27	-0.98	3.28	0.06
E1	TKW	2A	AX.89344976	7.56E+08	0.28	-1.08	3.97	0.07
E1	TKW	2A	AX.89377018	3.19E+07	0.27	-0.98	3.28	0.06
E1	GY	2A	AX.89745955	8.46E+07	0.23	-2.96	3.60	0.06
E1	GPM2	2A	AX.89694875	7.11E+07	0.32	-650.04	3.27	0.06
E1	GPM2	2A	AX.89586376	7.55E+08	0.27	664.85	3.12	0.05
E1	TKW	2A	AX.89470775	3.19E+07	0.27	-0.94	3.03	0.05
E1	TKW	2A	AX.89370330	3.19E+07	0.27	-0.98	3.28	0.06
E1	GPM2	2A	AX.89538795	7.78E+08	0.22	870.13	4.45	0.08
E1	GPM2	2A	AX.89457700	7.58E+08	0.23	735.98	3.41	0.06
E1	TKW	2A	AX.89637133	7.58E+08	0.50	0.85	3.15	0.06
E1	TKW	2A	AX.89678558	7.58E+08	0.23	-1.02	3.20	0.05
E1	GPM2	2A	AX.89686061	6.03E+08	0.07	-1275.12	3.68	0.06
E1	TKW	2A	AX.89497492	3.25E+07	0.11	-1.63	4.33	0.08
E1	TKW	2A	AX.89761983	3.19E+07	0.27	-0.98	3.28	0.06
E1	TKW	2A	AX.89583185	7.55E+08	0.26	-1.06	3.68	0.06
E1	TKW	2A	AX.89705883	4.28E+07	0.11	-1.41	3.39	0.06
E1	TKW	2A	AX.89747863	7.55E+08	0.27	-0.98	3.28	0.06
E1	TKW	2A	AX.89393215	7.56E+08	0.27	-0.99	3.32	0.06
E1	GPM2	2A	AX.89529168	7.76E+08	0.09	-1055.49	3.20	0.05
E1	GPM2	2A	AX.89553283	1.98E+08	0.47	661.05	3.78	0.07
E1	TKW	2A	AX.89610255	7.55E+08	0.27	-1.11	4.04	0.07
E1	GPM2	2A	AX.89639027	7.59E+08	0.20	852.43	4.06	0.07
E1	TKW	2A	AX.89379200	7.46E+08	0.14	-1.25	3.33	0.06
E1	TKW	2A	AX.89636460	7.55E+08	0.26	-1.07	3.67	0.06
E1	TKW	2A	AX.89488337	7.55E+08	0.30	-1.05	3.89	0.07
E1	GPM2	2A	AX.89358971	8.42E+07	0.21	-709.87	3.05	0.05
E1	GPM2	2A	AX.89700087	6.04E+08	0.09	-1132.54	3.76	0.07
E1	GPM2	2A	AX.89501387	8.39E+07	0.21	-709.87	3.05	0.05
E1	GPM2	2A	AX.89454688	8.46E+07	0.23	-710.38	3.17	0.05
E1	TKW	2A	AX.89761868	7.56E+08	0.27	-0.99	3.32	0.06
E1	TKW	2A	AX.89414865	7.55E+08	0.24	-1.09	3.70	0.06
E1	TKW	2A	AX.89740120	7.58E+08	0.23	-1.02	3.20	0.05
E1	TKW	2A	AX.89586700	7.56E+08	0.27	-1.09	3.95	0.07
E1	GPM2	2A	AX.89628287	8.42E+07	0.06	-1455.75	4.12	0.08
E1	TKW	2A	AX.89610993	7.56E+08	0.16	-1.23	3.50	0.06
E1	GPS	2A	AX.89610255	7.55E+08	0.27	1.43	3.86	0.07
E1	GPM2	2A	AX.89496843	7.78E+08	0.29	822.34	4.71	0.08
E1	TKW	2A	AX.89462856	7.58E+08	0.23	-1.02	3.20	0.05
E1	TKW	2A	AX.89362440	7.57E+08	0.40	0.96	3.72	0.07

E1	TKW	2A	AX.89440533	7.55E+08	0.27	-0.99	3.30	0.06
E1	GPM2	2A	AX.89509125	7.16E+07	0.44	-606.98	3.23	0.06
E1	TKW	2A	AX.89630198	3.19E+07	0.28	-1.01	3.56	0.06
E1	GPM2	2A	AX.89693178	7.88E+07	0.29	687.06	3.44	0.06
E1	TKW	2A	AX.89533629	3.19E+07	0.27	-0.98	3.28	0.06
E1	TKW	2A	AX.89559533	3.19E+07	0.27	-0.98	3.28	0.06
E1	TKW	2A	AX.89651278	4.27E+07	0.13	-1.45	4.00	0.07
E1	TKW	2A	AX.89737649	7.55E+08	0.24	-0.99	3.10	0.05
E1	TKW	2A	AX.89705764	7.58E+08	0.23	-1.02	3.20	0.05
E1	GPM2	2A	AX.89425144	7.59E+08	0.20	852.43	4.06	0.07
E1	GPM2	2A	AX.89732786	7.79E+08	0.21	948.49	4.97	0.09
E1	TKW	2A	AX.89749004	3.19E+07	0.27	-0.98	3.28	0.06
E1	TKW	2A	AX.89557781	7.55E+08	0.26	-1.07	3.67	0.06
E1	TKW	2A	AX.89736779	7.58E+08	0.23	-1.02	3.20	0.05
E1	GPS	2A	AX.89486643	4.86E+08	0.34	1.21	3.26	0.06
E1	GPM2	2A	AX.89381325	7.11E+07	0.32	-654.74	3.29	0.06
E1	GPM2	2A	AX.89701633	7.78E+08	0.21	732.40	3.21	0.06
E1	GPM2	2A	AX.89317553	7.78E+08	0.22	870.13	4.45	0.08
E1	GPM2	2A	AX.89720303	7.60E+08	0.18	1008.92	4.95	0.09
E1	TKW	2A	AX.89728983	7.55E+08	0.26	-1.07	3.67	0.06
E1	TKW	2A	AX.89451429	7.55E+08	0.24	-0.99	3.10	0.05
E1	GPM2	2A	AX.89745955	8.46E+07	0.23	-710.38	3.17	0.05
E1	GPM2	2A	AX.89568284	7.61E+08	0.20	888.57	4.30	0.08
E1	GPS	2A	AX.89751220	7.47E+08	0.21	1.36	3.11	0.05
E1	TKW	2A	AX.89601425	7.56E+08	0.27	-0.99	3.30	0.06
E1	TKW	2A	AX.89460601	7.61E+08	0.23	-1.05	3.33	0.05
E1	GPM2	2A	AX.89627705	8.65E+07	0.20	-730.35	3.07	0.05
E1	GPM2	2A	AX.89700088	8.56E+07	0.23	-702.76	3.11	0.05
E1	TKW	2A	AX.89423191	7.55E+08	0.26	-1.06	3.68	0.06
E1	TKW	2A	AX.89492728	3.19E+07	0.27	-0.98	3.28	0.06
E1	TKW	2A	AX.89430001	3.19E+07	0.28	-1.01	3.51	0.06
E1	TKW	2A	AX.89592393	7.56E+08	0.27	-1.09	3.95	0.07
E1	GPM2	2A	AX.89604444	7.14E+07	0.43	-647.67	3.60	0.07
E1	GPM2	2A	AX.89722052	7.79E+08	0.20	819.58	3.79	0.07
E1	TKW	2A	AX.89619827	7.56E+08	0.27	-0.99	3.32	0.06
E1	TKW	2A	AX.89516661	7.56E+08	0.27	-1.09	3.95	0.07
E1	GPM2	2A	AX.89613041	7.76E+08	0.08	-1056.70	3.08	0.05
E1	TKW	2A	AX.89433132	3.19E+07	0.27	-0.98	3.28	0.06
E1	TKW	2A	AX.89703222	7.55E+08	0.11	-1.46	3.57	0.06
E1	TKW	2A	AX.89637508	7.55E+08	0.27	-1.11	4.04	0.07
E1	TKW	2A	AX.89319120	7.55E+08	0.26	-1.06	3.63	0.06
E1	TKW	2A	AX.89526254	7.56E+08	0.26	-1.00	3.33	0.06
E1	TKW	2A	AX.89347674	7.56E+08	0.26	-1.01	3.35	0.06
E1	TKW	2A	AX.89489720	3.19E+07	0.27	-0.98	3.28	0.06
E1	TKW	2A	AX.89513724	7.55E+08	0.24	-0.99	3.10	0.05
E1	TKW	2A	AX.89438787	7.55E+08	0.25	-0.96	3.05	0.05
E1	TKW	2A	AX.89570704	3.19E+07	0.27	-0.98	3.28	0.06
E1	TKW	2A	AX.89750010	7.56E+08	0.26	-1.00	3.29	0.06
E1	TKW	2A	AX.89588999	7.55E+08	0.26	-0.96	3.06	0.05
E1	TKW	2A	AX.89646006	7.56E+08	0.26	-1.01	3.35	0.06

E1	TKW	2A	AX.89575155	7.58E+08	0.23	-1.02	3.20	0.05
E1	TKW	2A	AX.89569322	7.60E+08	0.24	-0.98	3.06	0.05
E1	TKW	2A	AX.89654143	7.58E+08	0.22	-1.02	3.13	0.05
E1	TKW	2A	AX.89643570	7.60E+08	0.22	-1.03	3.21	0.05
E1	TKW	2A	AX.89502891	4.28E+07	0.13	-1.55	4.34	0.07
E1	TKW	2A	AX.89413417	7.55E+08	0.26	-1.07	3.67	0.06
E1	TKW	2A	AX.89748537	3.25E+07	0.11	-1.63	4.33	0.08
E1	TKW	2A	AX.89746670	3.19E+07	0.27	-0.96	3.19	0.06
E1	TKW	2A	AX.89762459	7.55E+08	0.27	-0.98	3.28	0.06
E1	TKW	2A	AX.89441296	7.55E+08	0.26	-1.06	3.68	0.06
E1	TKW	2A	AX.89534141	7.55E+08	0.27	-1.02	3.48	0.06
E1	TKW	2A	AX.89496843	7.78E+08	0.29	-0.96	3.31	0.06
E1	TKW	2A	AX.89335578	7.56E+08	0.27	-0.99	3.30	0.06
E2	GY	2A	AX.89618874	8.56E+07	0.07	-4.48	4.21	0.09
E2	GY	2A	AX.89597570	8.38E+07	0.09	-3.57	3.56	0.08
E2	GY	2A	AX.89597570	8.38E+07	0.09	-3.57	3.56	0.08
E2	GY	2A	AX.89747464	8.45E+07	0.07	-5.25	5.24	0.12
E2	GY	2A	AX.89747464	8.45E+07	0.07	-5.25	5.24	0.12
E2	SPM2	2A	AX.89765361	7.53E+08	0.10	24.04	4.47	0.08
E2	GY	2A	AX.89737425	6.97E+08	0.12	-3.09	3.34	0.04
E2	GY	2A	AX.89510338	7.76E+08	0.08	-3.49	3.15	0.04
E2	GY	2A	AX.89639258	8.38E+07	0.09	-4.10	4.33	0.10
E2	SPM2	2A	AX.89382803	7.51E+08	0.12	21.83	4.29	0.08
E2	SPM2	2A	AX.89568566	9.98E+07	0.07	23.45	3.34	0.06
E2	GY	2A	AX.89336730	7.40E+08	0.09	-3.78	3.81	0.05
E2	GY	2A	AX.89336730	7.40E+08	0.09	-3.78	3.81	0.05
E2	SPM2	2A	AX.89344555	4.47E+08	0.08	21.81	3.23	0.05
E2	GY	2A	AX.89644181	7.12E+08	0.20	-2.60	3.60	0.06
E2	GY	2A	AX.89644181	7.12E+08	0.20	-2.60	3.60	0.06
E2	SPM2	2A	AX.89653353	7.41E+08	0.11	-19.40	3.19	0.05
E2	GPM2	2A	AX.89770211	8.38E+07	0.08	-954.25	3.23	0.07
E2	GY	2A	AX.89770211	8.38E+07	0.07	-4.32	4.15	0.09
E2	GY	2A	AX.89770211	8.38E+07	0.07	-4.32	4.15	0.09
E2	GY	2A	AX.89365349	8.39E+07	0.09	-3.57	3.56	0.08
E2	GY	2A	AX.89534012	7.62E+08	0.14	-3.13	3.95	0.06
E2	GY	2A	AX.89534012	7.62E+08	0.14	-3.13	3.95	0.06
E2	GY	2A	AX.86169701	7.76E+08	0.10	-3.24	3.26	0.04
E2	GY	2A	AX.86169701	7.76E+08	0.10	-3.24	3.26	0.04
E2	GPS	2A	AX.89608691	7.00E+08	0.43	-1.43	3.43	0.06
E2	GY	2A	AX.89414077	8.46E+07	0.07	-5.25	5.24	0.12
E2	GY	2A	AX.89414077	8.46E+07	0.07	-5.25	5.24	0.12
E2	GPS	2A	AX.89706552	7.12E+08	0.28	-1.90	4.75	0.09
E2	GPS	2A	AX.89593956	7.00E+08	0.43	-1.49	3.69	0.06
E2	GY	2A	AX.89508461	8.56E+07	0.07	-4.48	4.21	0.09
E2	GY	2A	AX.89508461	8.56E+07	0.07	-4.48	4.21	0.09
E2	GY	2A	AX.89343847	8.50E+07	0.06	-5.41	5.17	0.12
E2	GY	2A	AX.89343847	8.50E+07	0.06	-5.41	5.17	0.12
E2	GPM2	2A	AX.89488613	8.56E+07	0.07	-1095.83	3.67	0.09
E2	GY	2A	AX.89344767	8.39E+07	0.09	-3.57	3.56	0.08
E2	GY	2A	AX.89344767	8.39E+07	0.09	-3.57	3.56	0.08

E2	GPS	2A	AX.89728760	6.54E+08	0.43	1.46	3.56	0.06
E2	GY	2A	AX.89382939	8.63E+07	0.07	-4.48	4.21	0.09
E2	GY	2A	AX.89382939	8.63E+07	0.07	-4.48	4.21	0.09
E2	GY	2A	AX.89670276	7.51E+08	0.06	-5.12	4.69	0.10
E2	GY	2A	AX.89670276	7.51E+08	0.06	-5.12	4.69	0.10
E2	TKW	2A	AX.89359914	7.61E+08	0.26	-1.22	4.11	0.07
E2	GY	2A	AX.89365349	8.39E+07	0.09	-3.57	3.56	0.08
E2	GY	2A	AX.89776727	8.39E+07	0.09	-3.57	3.56	0.08
E2	GY	2A	AX.89776727	8.39E+07	0.09	-3.57	3.56	0.08
E2	GY	2A	AX.89321824	8.38E+07	0.09	-3.57	3.56	0.08
E2	GY	2A	AX.89321824	8.38E+07	0.09	-3.57	3.56	0.08
E2	TKW	2A	AX.89689821	7.55E+08	0.24	-1.04	3.01	0.05
E2	GY	2A	AX.89569793	8.46E+07	0.07	-5.25	5.24	0.12
E2	GY	2A	AX.89569793	8.46E+07	0.07	-5.25	5.24	0.12
E2	GY	2A	AX.89628287	8.42E+07	0.06	-5.61	5.52	0.12
E2	GY	2A	AX.89513340	7.03E+08	0.40	-2.03	3.35	0.05
E2	GY	2A	AX.89513340	7.03E+08	0.40	-2.03	3.35	0.05
E2	GY	2A	AX.89765381	7.62E+08	0.14	-2.88	3.46	0.06
E2	GY	2A	AX.89765381	7.62E+08	0.14	-2.88	3.46	0.06
E2	GY	2A	AX.89608084	8.39E+07	0.09	-3.57	3.56	0.08
E2	GY	2A	AX.89608084	8.39E+07	0.09	-3.57	3.56	0.08
E2	GY	2A	AX.89766787	7.03E+08	0.40	-2.03	3.35	0.05
E2	GY	2A	AX.89409852	7.76E+08	0.10	-3.48	3.54	0.04
E2	GY	2A	AX.89409852	7.76E+08	0.10	-3.48	3.54	0.04
E2	GPS	2A	AX.89441954	7.12E+08	0.49	-1.36	3.19	0.05
E2	GY	2A	AX.89480595	8.56E+07	0.07	-4.48	4.21	0.09
E2	GY	2A	AX.89480595	8.56E+07	0.07	-4.48	4.21	0.09
E2	TKW	2A	AX.89414865	7.55E+08	0.24	-1.11	3.36	0.05
E2	TKW	2A	AX.89584181	4.28E+07	0.13	-1.63	4.28	0.08
E2	GY	2A	AX.89618874	8.56E+07	0.07	-4.48	4.21	0.09
E2	GPM2	2A	AX.89745404	8.42E+07	0.06	-1148.92	3.74	0.09
E2	GY	2A	AX.89662622	8.42E+07	0.09	-3.57	3.56	0.08
E2	GY	2A	AX.89367851	7.62E+08	0.17	-2.52	3.02	0.04
E2	GY	2A	AX.89367851	7.62E+08	0.17	-2.52	3.02	0.04
E2	GY	2A	AX.89737425	6.97E+08	0.12	-3.09	3.34	0.04
E2	GY	2A	AX.89510338	7.76E+08	0.08	-3.49	3.15	0.04
E2	GY	2A	AX.89639258	8.38E+07	0.09	-4.10	4.33	0.10
E2	GY	2A	AX.89706225	8.42E+07	0.07	-5.25	5.24	0.12
E2	GY	2A	AX.89706225	8.42E+07	0.07	-5.25	5.24	0.12
E2	TKW	2A	AX.89586614	4.32E+07	0.12	-1.67	4.31	0.08
E2	GPS	2A	AX.89311201	7.15E+08	0.32	-1.43	3.12	0.05
E2	GPS	2A	AX.89331545	7.18E+08	0.48	-1.38	3.26	0.06
E2	GPS	2A	AX.89570522	7.12E+08	0.24	-1.72	3.63	0.07
E2	GPS	2A	AX.89726681	7.00E+08	0.43	-1.41	3.33	0.06
E2	GY	2A	AX.89420517	8.39E+07	0.09	-3.57	3.56	0.08
E2	GY	2A	AX.89742073	7.76E+08	0.08	-3.49	3.15	0.04
E2	GY	2A	AX.89742073	7.76E+08	0.08	-3.49	3.15	0.04
E2	GY	2A	AX.89766787	7.03E+08	0.40	-2.03	3.35	0.05
E2	GPM2	2A	AX.89414077	8.46E+07	0.07	-1095.83	3.67	0.09
E2	GY	2A	AX.89500395	7.63E+08	0.15	-3.08	4.05	0.06

E2	GY	2A	AX.89500395	7.63E+08	0.15	-3.08	4.05	0.06
E2	GPM2	2A	AX.89343847	8.50E+07	0.06	-1080.21	3.36	0.08
E2	GPS	2A	AX.89565323	7.00E+08	0.43	-1.41	3.36	0.06
E2	GY	2A	AX.89508618	8.56E+07	0.07	-4.48	4.21	0.09
E2	TKW	2A	AX.89651278	4.27E+07	0.13	-1.64	4.43	0.08
E2	GY	2A	AX.89610686	8.42E+07	0.06	-5.55	5.42	0.12
E2	GY	2A	AX.89610686	8.42E+07	0.06	-5.55	5.42	0.12
E2	GPS	2A	AX.89598382	7.12E+08	0.29	-1.89	4.76	0.09
E2	TKW	2A	AX.89319120	7.55E+08	0.25	-1.05	3.18	0.05
E2	TKW	2A	AX.89455758	7.61E+08	0.43	-0.93	3.21	0.05
E2	GPS	2A	AX.89652949	7.19E+08	0.31	-1.42	3.01	0.06
E2	GY	2A	AX.89343827	8.63E+07	0.07	-4.48	4.21	0.09
E2	GY	2A	AX.89343827	8.63E+07	0.07	-4.48	4.21	0.09
E2	TKW	2A	AX.89365566	7.55E+08	0.26	-1.05	3.20	0.05
E2	GY	2A	AX.89662622	8.42E+07	0.09	-3.57	3.56	0.08
E2	GPS	2A	AX.89753712	7.19E+08	0.31	-1.42	3.01	0.06
E2	GPM2	2A	AX.89549965	6.04E+08	0.08	-935.71	3.47	0.07
E2	GPS	2A	AX.89326165	7.12E+08	0.28	-1.90	4.75	0.09
E2	GPS	2A	AX.89775314	7.11E+08	0.22	-1.63	3.12	0.06
E2	GPS	2A	AX.89662434	6.40E+08	0.47	1.43	3.45	0.06
E2	GY	2A	AX.89568437	7.62E+08	0.13	-3.12	3.67	0.06
E2	GY	2A	AX.89568437	7.62E+08	0.13	-3.12	3.67	0.06
E2	GY	2A	AX.89605140	8.42E+07	0.09	-4.10	4.33	0.10
E2	GY	2A	AX.89605140	8.42E+07	0.09	-4.10	4.33	0.10
E2	GY	2A	AX.89628287	8.42E+07	0.06	-5.61	5.52	0.12
E2	GY	2A	AX.89429525	8.42E+07	0.07	-5.25	5.24	0.12
E2	GY	2A	AX.89429525	8.42E+07	0.07	-5.25	5.24	0.12
E2	GPS	2A	AX.89380553	7.19E+08	0.46	1.33	3.05	0.06
E2	GY	2A	AX.89488613	8.56E+07	0.07	-5.25	5.24	0.12
E2	GY	2A	AX.89488613	8.56E+07	0.07	-5.25	5.24	0.12
E2	TKW	2A	AX.89345671	4.28E+07	0.12	-1.53	3.72	0.06
E2	TKW	2A	AX.89705883	4.28E+07	0.11	-1.46	3.23	0.05
E2	GPS	2A	AX.89699925	7.00E+08	0.43	-1.43	3.43	0.06
E2	GPS	2A	AX.89453912	7.00E+08	0.43	-1.43	3.43	0.06
E2	GPS	2A	AX.89627604	7.13E+08	0.20	-1.79	3.45	0.05
E2	GPM2	2A	AX.89706225	8.42E+07	0.07	-1095.83	3.67	0.09
E2	GY	2A	AX.89420517	8.39E+07	0.09	-3.57	3.56	0.08
E2	GPS	2A	AX.89655607	7.13E+08	0.13	-2.13	3.43	0.07
E2	TKW	2A	AX.89583185	7.55E+08	0.26	-1.05	3.20	0.05
E2	GY	2A	AX.89745404	8.42E+07	0.06	-5.61	5.52	0.12
E2	GPS	2A	AX.89677861	7.12E+08	0.22	-1.63	3.12	0.06
E2	TKW	2A	AX.89610255	7.55E+08	0.26	-1.13	3.70	0.06
E2	GPS	2A	AX.89487305	7.03E+08	0.50	-1.52	3.88	0.07
E2	TKW	2A	AX.89553707	7.60E+08	0.23	-1.11	3.24	0.05
E2	GY	2A	AX.89652948	8.63E+07	0.07	-4.58	4.11	0.09
E2	GY	2A	AX.89652948	8.63E+07	0.07	-4.58	4.11	0.09
E2	GY	2A	AX.89569105	7.76E+08	0.08	-3.49	3.15	0.04
E2	GY	2A	AX.89569105	7.76E+08	0.08	-3.49	3.15	0.04
E2	TKW	2A	AX.89413417	7.55E+08	0.25	-1.06	3.22	0.05
E2	TKW	2A	AX.89752134	7.55E+08	0.25	-1.06	3.22	0.05

E2	TKW	2A	AX.89460601	7.61E+08	0.23	-1.12	3.34	0.06
E2	GPS	2A	AX.89675494	7.13E+08	0.20	-1.79	3.45	0.05
E2	GY	2A	AX.89721986	8.63E+07	0.07	-4.48	4.21	0.09
E2	GY	2A	AX.89721986	8.63E+07	0.07	-4.48	4.21	0.09
E2	GPS	2A	AX.89597251	7.12E+08	0.22	-1.63	3.12	0.06
E2	TKW	2A	AX.89408055	7.55E+08	0.26	-1.05	3.20	0.05
E2	TKW	2A	AX.89751420	7.60E+08	0.43	-0.94	3.28	0.05
E2	TKW	2A	AX.89637508	7.55E+08	0.26	-1.13	3.70	0.06
E2	GY	2A	AX.89508618	8.56E+07	0.07	-4.48	4.21	0.09
E2	GPS	2A	AX.89491141	7.03E+08	0.50	-1.52	3.88	0.07
E2	TKW	2A	AX.89569322	7.60E+08	0.24	-1.06	3.09	0.05
E2	GY	2A	AX.89745404	8.42E+07	0.06	-5.61	5.52	0.12
E2	GPS	2A	AX.89534207	7.00E+08	0.43	-1.43	3.43	0.06
E2	GY	2A	AX.89642711	8.38E+07	0.09	-3.57	3.56	0.08
E2	GY	2A	AX.89642711	8.38E+07	0.09	-3.57	3.56	0.08
E2	GPM2	2A	AX.89437001	6.04E+08	0.08	-935.71	3.47	0.07
E2	GPM2	2A	AX.89334194	6.03E+08	0.11	-786.50	3.14	0.06
E2	TKW	2A	AX.89452594	7.55E+08	0.26	-1.05	3.20	0.05
E2	GY	2A	AX.89613041	7.76E+08	0.08	-3.49	3.15	0.04
E2	GY	2A	AX.89613041	7.76E+08	0.08	-3.49	3.15	0.04
E2	GPS	2A	AX.89631539	7.00E+08	0.43	-1.41	3.33	0.06
E2	TKW	2A	AX.89423191	7.55E+08	0.26	-1.05	3.20	0.05
E2	GPM2	2A	AX.89639258	8.38E+07	0.09	-927.77	3.51	0.08
E2	GPS	2A	AX.89722531	7.13E+08	0.20	-1.79	3.45	0.05
E2	TKW	2A	AX.89580653	7.55E+08	0.23	-1.12	3.39	0.06
E2	TKW	2A	AX.89575538	7.55E+08	0.25	-1.06	3.22	0.05
E2	GPM2	2A	AX.89628287	8.42E+07	0.06	-1148.92	3.74	0.09
E2	TKW	2A	AX.89678473	7.59E+08	0.33	-0.96	3.07	0.05
E2	GPS	2A	AX.89630984	7.03E+08	0.50	-1.52	3.88	0.07
E2	GPS	2A	AX.89625036	6.53E+08	0.43	1.46	3.56	0.06
E2	GPS	2A	AX.89492668	7.13E+08	0.20	-1.79	3.45	0.05
E2	TKW	2A	AX.89643570	7.60E+08	0.23	-1.11	3.24	0.05
E2	GPS	2A	AX.89557526	7.03E+08	0.50	-1.52	3.88	0.07
E2	GPM2	2A	AX.89569793	8.46E+07	0.07	-1095.83	3.67	0.09
E2	GPM2	2A	AX.89429525	8.42E+07	0.07	-1095.83	3.67	0.09
E2	TKW	2A	AX.89728983	7.55E+08	0.25	-1.06	3.22	0.05
E2	GPS	2A	AX.89710690	7.12E+08	0.19	-1.71	3.16	0.06
E2	TKW	2A	AX.89441296	7.55E+08	0.26	-1.05	3.20	0.05
E2	GPM2	2A	AX.89610686	8.42E+07	0.06	-1222.90	4.18	0.10
E2	GPS	2A	AX.89317775	7.13E+08	0.13	-2.13	3.43	0.07
E2	TKW	2A	AX.89502891	4.28E+07	0.13	-1.54	3.87	0.07
E2	TKW	2A	AX.89530131	7.55E+08	0.26	-1.13	3.70	0.06
E2	TKW	2A	AX.89374034	7.55E+08	0.26	-1.05	3.20	0.05
E2	GPM2	2A	AX.89605140	8.42E+07	0.09	-927.77	3.51	0.08
E2	TKW	2A	AX.89562993	7.55E+08	0.26	-1.05	3.20	0.05
E2	TKW	2A	AX.89573905	7.55E+08	0.23	-1.12	3.39	0.06
E2	GPS	2A	AX.89644181	7.12E+08	0.19	-2.11	4.52	0.09
E2	TKW	2A	AX.89375933	7.55E+08	0.26	-1.05	3.20	0.05
E2	TKW	2A	AX.89630473	7.58E+08	0.11	1.60	3.77	0.07
E2	TKW	2A	AX.89437827	7.11E+08	0.14	1.33	3.16	0.06

E2	GPM2	2A	AX.89747464	8.45E+07	0.07	-1095.83	3.67	0.09
E2	TKW	2A	AX.89636460	7.55E+08	0.25	-1.06	3.22	0.05
E2	GPS	2A	AX.89535340	7.03E+08	0.49	-1.51	3.81	0.06
E2	TKW	2A	AX.89557781	7.55E+08	0.25	-1.06	3.22	0.05
E2	GPM2	2A	AX.89466371	6.04E+08	0.08	-935.71	3.47	0.07
E3	GPM2	2A	AX.89415024	7.70E+08	0.26	985.17	3.07	0.10
E3	GPM2	2A	AX.89711562	7.70E+08	0.29	1004.47	3.47	0.10
E3	GPM2	2A	AX.89478074	7.70E+08	0.27	952.99	3.01	0.10
E3	GPM2	2A	AX.89458713	7.70E+08	0.27	1074.00	3.61	0.11
E3	GPM2	2A	AX.89439261	7.70E+08	0.26	1012.72	3.24	0.10
E3	GPM2	2A	AX.89627764	7.71E+08	0.36	-883.57	3.02	0.07
E3	GPS	2A	AX.89463787	8.23E+07	0.14	3.15	3.60	0.11
E3	GPS	2A	AX.89619058	8.24E+07	0.14	3.15	3.60	0.11
E3	GPS	2A	AX.89688431	8.28E+07	0.17	2.72	3.21	0.09
E3	GPS	2A	AX.89640537	8.92E+07	0.14	2.86	3.09	0.08
E3	GPS	2A	AX.89450367	8.95E+07	0.13	3.05	3.24	0.08
E3	GPS	2A	AX.89559829	7.92E+07	0.26	2.53	3.74	0.08
E3	GPS	2A	AX.89433178	7.92E+07	0.26	2.53	3.74	0.08
E3	GPS	2A	AX.89394956	7.92E+07	0.26	2.53	3.74	0.08
E3	GPS	2A	AX.89312435	7.92E+07	0.26	2.53	3.74	0.08
E3	GPS	2A	AX.89656483	8.05E+07	0.19	2.44	3.01	0.07
E3	GPS	2A	AX.89578301	8.05E+07	0.20	2.49	3.15	0.07
E3	GPS	2A	AX.89356336	8.21E+07	0.13	3.08	3.41	0.10
E3	GPS	2A	X.89624808_OT	8.21E+07	0.14	3.15	3.60	0.11
E3	GPS	2A	X.89717181_OT	8.21E+07	0.14	3.15	3.60	0.11
E3	GPS	2A	X.89492810_OT	8.22E+07	0.14	3.15	3.60	0.11
E3	GPS	2A	X.89545434_OT	8.22E+07	0.14	3.15	3.60	0.11
E3	GPS	2A	X.89694744_OT	8.22E+07	0.14	3.15	3.60	0.11
E3	GPS	2A	X.89603279_OT	8.22E+07	0.14	3.15	3.60	0.11
E3	GPS	2A	AX.89712432	8.22E+07	0.18	2.61	3.12	0.08
E3	GPS	2A	X.89596030_OT	8.22E+07	0.14	3.15	3.60	0.11
E3	GPS	2A	X.89650633_OT	8.22E+07	0.14	3.15	3.60	0.11
E3	GPS	2A	AX.89609600	8.22E+07	0.18	2.61	3.12	0.08
E3	GPS	2A	AX.89396825	8.22E+07	0.14	3.15	3.60	0.11
E3	GPS	2A	AX.89739062	8.22E+07	0.14	3.15	3.60	0.11
E3	GPS	2A	AX.89381836	8.23E+07	0.18	2.61	3.12	0.08
E3	GY	2A	AX.89574661	5.85E+07	0.36	3.12	3.32	0.10
E3	GY	2A	AX.89613371	5.85E+07	0.35	2.94	3.05	0.10
E3	GY	2A	AX.89680495	5.85E+07	0.36	3.14	3.39	0.10
E3	GY	2A	AX.89673524	5.85E+07	0.35	2.94	3.05	0.10
E3	GY	2A	AX.89388636	5.86E+07	0.35	2.94	3.05	0.10
E3	GY	2A	AX.89496743	5.86E+07	0.35	2.94	3.05	0.10
E3	GY	2A	AX.89694611	5.86E+07	0.35	2.94	3.05	0.10
E3	GY	2A	AX.89768257	5.86E+07	0.35	2.94	3.05	0.10
E3	GY	2A	AX.89538948	5.86E+07	0.35	2.94	3.05	0.10
E3	GY	2A	AX.89531893	5.87E+07	0.35	3.18	3.51	0.10
E3	GY	2A	AX.89642236	5.87E+07	0.35	2.94	3.05	0.10
E3	GY	2A	AX.89733897	5.87E+07	0.35	2.94	3.05	0.10
E3	GY	2A	AX.89487115	9.81E+07	0.17	3.85	3.33	0.04
E3	GY	2A	AX.89703640	7.13E+08	0.20	3.44	3.09	0.03

E3	GY	2A	AX.89715805	7.13E+08	0.20	3.44	3.09	0.03
E3	GPS	2A	AX.89723340	3.18E+07	0.10	-3.22	3.02	0.04
E3	GPS	2A	AX.89669753	7.86E+07	0.25	2.60	3.87	0.09
E3	GPS	2A	AX.89388899	7.86E+07	0.25	2.60	3.87	0.09
E3	GPS	2A	AX.89738519	7.86E+07	0.25	2.60	3.87	0.09
E3	GPS	2A	AX.89757105	7.86E+07	0.25	2.60	3.87	0.09
E3	GPS	2A	AX.89426364	7.86E+07	0.24	2.46	3.47	0.08
E3	GPS	2A	AX.89520142	7.87E+07	0.25	2.60	3.87	0.09
E3	GPS	2A	AX.89650961	7.87E+07	0.25	2.60	3.87	0.09
E3	GPS	2A	AX.89539982	7.87E+07	0.25	2.60	3.87	0.09
E3	GPS	2A	AX.89393031	7.88E+07	0.20	2.43	3.03	0.05
E3	GPS	2A	AX.89348583	7.88E+07	0.25	2.60	3.87	0.09
E3	GPS	2A	AX.89351614	7.88E+07	0.25	2.60	3.87	0.09
E3	GPS	2A	AX.89523442	7.88E+07	0.25	2.60	3.87	0.09
E3	GPS	2A	AX.89645760	7.88E+07	0.26	2.68	4.18	0.10
E3	GPS	2A	AX.89519482	7.88E+07	0.26	2.53	3.74	0.08
E3	GPS	2A	AX.89617969	7.88E+07	0.26	2.61	4.04	0.09
E3	GPS	2A	AX.89670101	7.88E+07	0.26	2.61	4.04	0.09
E3	GPS	2A	AX.89529344	7.88E+07	0.25	2.49	3.59	0.08
E3	GPS	2A	AX.89759916	7.88E+07	0.26	2.53	3.74	0.08
E3	GPS	2A	AX.89752332	7.88E+07	0.26	2.53	3.74	0.08
E3	GPS	2A	AX.89762994	7.88E+07	0.26	2.53	3.74	0.08
E3	GPS	2A	AX.89617348	7.88E+07	0.26	2.53	3.74	0.08
E3	GPS	2A	AX.89643234	7.88E+07	0.26	2.53	3.74	0.08
E3	GPS	2A	AX.89520042	7.89E+07	0.26	2.53	3.74	0.08
E3	GPS	2A	AX.89769687	7.89E+07	0.26	2.53	3.74	0.08
E3	GPS	2A	AX.89459735	7.89E+07	0.26	2.61	4.04	0.09
E3	GPS	2A	AX.89778244	7.89E+07	0.26	2.53	3.74	0.08
E3	GPS	2A	AX.89640288	7.90E+07	0.26	2.53	3.74	0.08
E3	GPS	2A	AX.89387048	7.92E+07	0.26	2.53	3.74	0.08
E3	GPS	2A	AX.89608481	7.92E+07	0.26	2.52	3.72	0.09
E3	SPM2	2A	AX.89335172	7.51E+08	0.43	-19.05	3.00	0.07
E3	SPM2	2A	AX.89528587	7.51E+08	0.42	-19.15	3.02	0.07
E3	SPM2	2A	AX.89654831	7.51E+08	0.39	-21.10	3.49	0.06
E3	SPM2	2A	AX.89537145	7.51E+08	0.39	-21.10	3.49	0.06
E3	SPM2	2A	AX.89458713	7.70E+08	0.24	24.74	3.31	0.06
E3	SPM2	2A	AX.89439261	7.70E+08	0.24	23.73	3.04	0.06
E3	TKW	2A	AX.89657156	7.41E+08	0.48	-0.88	3.36	0.01
E3	TKW	2A	AX.89612894	7.41E+08	0.47	-0.88	3.38	0.02
E3	TKW	2A	AX.89476953	7.41E+08	0.49	-0.83	3.03	0.01
E3	TKW	2A	AX.89494740	7.81E+08	0.27	0.96	3.05	0.11
E4	GPM2	2A	AX.89346671	7.37E+08	0.33	504.95	3.06	0.08
E4	GPM2	2A	AX.89383546	7.75E+08	0.32	-493.22	3.22	0.05
E4	GPM2	2A	AX.89563018	7.75E+08	0.32	-493.22	3.22	0.05
E4	GPM2	2A	AX.89694535	7.75E+08	0.32	-493.22	3.22	0.05
E4	GPM2	2A	AX.89527623	7.78E+08	0.07	-833.52	3.09	0.05
E4	GPM2	2A	AX.89734662	6.82E+07	0.32	-493.22	3.22	0.05
E4	GPM2	2A	AX.89313920	7.37E+08	0.33	504.62	3.04	0.08
E4	GPM2	2A	AX.89320627	7.37E+08	0.33	504.62	3.04	0.08
E4	GPS	2A	AX.89591424	5.97E+08	0.38	-1.57	3.22	0.06

E4	GPS	2A	AX.89542280	6.08E+08	0.11	-2.62	3.29	0.08
E4	GPS	2A	AX.89682622	7.04E+08	0.10	2.48	3.31	0.04
E4	GPS	2A	AX.89582787	7.06E+08	0.05	3.65	3.92	0.07
E4	GY	2A	AX.89698177	3.64E+07	0.08	-2.75	3.42	0.05
E4	GY	2A	AX.89593047	6.21E+08	0.12	-2.50	3.34	0.07
E4	GY	2A	AX.89766980	7.56E+08	0.36	-1.52	3.00	0.05
E4	GY	2A	AX.89509202	7.57E+08	0.42	-1.50	3.08	0.05
E4	GY	2A	AX.89755911	7.57E+08	0.41	-1.51	3.09	0.05
E4	GY	2A	AX.89354258	7.75E+08	0.13	-2.14	3.03	0.04
E4	GY	2A	AX.89412806	7.75E+08	0.11	-2.37	3.19	0.04
E4	GY	2A	AX.89320131	7.76E+08	0.11	-2.33	3.18	0.04
E4	GY	2A	AX.89527623	7.78E+08	0.07	-2.94	3.32	0.05
E4	SPM2	2A	AX.89553348	7.01E+08	0.21	21.95	3.50	0.07
E4	SPM2	2A	AX.89448509	7.02E+08	0.18	21.92	3.17	0.07
E4	TKW	2A	AX.89653751	7.03E+08	0.20	-1.23	3.75	0.07
E1	GY	2B	AX.89376677	1.82E+08	0.13	-4.62	5.44	0.10
E1	GY	2B	AX.89381154	7.57E+08	0.06	-5.75	3.97	0.06
E1	GY	2B	AX.89454254	1.01E+08	0.07	-4.61	3.07	0.06
E1	GY	2B	AX.89449676	1.84E+08	0.14	-3.69	3.76	0.07
E1	GY	2B	AX.89404329	9.74E+06	0.27	-2.52	3.01	0.04
E1	GY	2B	AX.89327795	1.39E+08	0.08	-5.46	4.81	0.09
E1	GY	2B	AX.89470805	1.76E+07	0.25	2.63	3.05	0.03
E1	GY	2B	AX.89452576	3.18E+05	0.27	-2.52	3.01	0.04
E1	GY	2B	AX.89470805	1.76E+07	0.25	2.63	3.05	0.03
E1	GY	2B	AX.89551300	7.93E+08	0.35	-2.98	4.54	0.09
E1	GY	2B	AX.89486719	7.85E+08	0.10	-3.90	3.28	0.05
E1	GY	2B	AX.89671402	1.01E+08	0.07	-4.61	3.07	0.06
E1	GY	2B	AX.89676426	1.82E+08	0.13	-4.30	4.67	0.09
E1	GY	2B	AX.89314257	1.43E+08	0.26	-2.60	3.06	0.05
E1	GY	2B	AX.89674559	8.60E+06	0.35	-2.48	3.29	0.05
E1	GY	2B	AX.89486719	7.85E+08	0.10	-3.90	3.28	0.05
E1	GY	2B	AX.89590527	1.33E+08	0.07	-4.72	3.38	0.05
E1	GY	2B	AX.89451061	1.04E+08	0.07	-4.61	3.07	0.06
E1	GY	2B	AX.89327212	1.80E+08	0.20	-3.07	3.45	0.06
E1	GY	2B	AX.89699216	1.82E+08	0.11	-3.88	3.48	0.06
E1	GY	2B	AX.89715595	1.84E+08	0.09	-4.30	3.47	0.06
E1	GY	2B	AX.89395850	1.84E+08	0.14	-3.55	3.60	0.06
E1	GY	2B	AX.89671402	1.01E+08	0.07	-4.61	3.07	0.06
E1	GY	2B	AX.89339086	1.41E+08	0.26	-2.60	3.06	0.05
E1	GY	2B	AX.89407393	1.06E+08	0.08	-5.36	4.87	0.08
E1	GY	2B	AX.89396407	1.04E+08	0.07	-4.61	3.07	0.06
E1	GY	2B	AX.89327212	1.80E+08	0.20	-3.07	3.45	0.06
E1	GY	2B	AX.89379675	1.03E+08	0.07	-4.61	3.07	0.06
E1	GY	2B	AX.89727517	7.57E+08	0.06	-5.01	3.13	0.05
E1	GY	2B	AX.89412544	1.84E+08	0.09	-4.30	3.47	0.06
E1	GY	2B	AX.89412544	1.84E+08	0.09	-4.30	3.47	0.06
E1	GY	2B	AX.89376677	1.82E+08	0.13	-4.62	5.44	0.10
E1	GY	2B	AX.89686951	1.42E+08	0.26	-2.60	3.06	0.05
E1	GY	2B	AX.89359515	5.94E+08	0.33	2.55	3.34	0.06
E1	GY	2B	AX.89490236	4.74E+04	0.27	-2.52	3.01	0.04

E1	GY	2B	AX.89737804	1.79E+08	0.20	-3.07	3.45	0.06
E1	GY	2B	AX.89335901	1.83E+08	0.14	-4.13	4.69	0.09
E1	GY	2B	AX.89450884	5.95E+08	0.31	2.44	3.04	0.05
E1	GY	2B	AX.89460798	1.03E+08	0.07	-4.61	3.07	0.06
E1	GY	2B	AX.89401624	4.53E+05	0.27	-2.52	3.01	0.04
E1	GY	2B	AX.89588628	7.59E+08	0.06	-4.77	3.07	0.05
E1	GY	2B	AX.89760340	7.65E+08	0.12	-3.88	3.59	0.07
E1	GY	2B	AX.89511482	1.83E+08	0.13	-3.70	3.69	0.07
E1	GY	2B	AX.89346022	1.70E+08	0.10	3.80	3.14	0.05
E1	GY	2B	AX.86169218	1.84E+08	0.09	-4.30	3.47	0.06
E1	GY	2B	AX.89459157	1.81E+08	0.12	-3.54	3.15	0.06
E1	GY	2B	AX.89459157	1.81E+08	0.12	-3.54	3.15	0.06
E1	GY	2B	AX.89589315	7.59E+08	0.06	-4.77	3.07	0.05
E1	SPM2	2B	AX.89575791	7.12E+08	0.20	23.05	3.83	0.07
E1	GY	2B	AX.89661546	5.94E+08	0.32	2.42	3.02	0.05
E1	GY	2B	AX.86170531	1.84E+08	0.09	-4.30	3.47	0.06
E1	GY	2B	AX.89727377	5.94E+08	0.33	2.53	3.30	0.06
E1	GY	2B	AX.89496140	5.06E+05	0.27	-2.52	3.01	0.04
E1	GY	2B	AX.89313148	7.85E+08	0.25	-2.76	3.35	0.06
E1	GY	2B	AX.89654194	1.81E+08	0.11	-3.88	3.48	0.06
E1	GY	2B	AX.89591171	1.83E+08	0.14	-3.82	3.99	0.07
E1	GY	2B	AX.89663766	1.43E+08	0.25	-2.76	3.36	0.06
E1	GY	2B	AX.89449676	1.84E+08	0.14	-3.69	3.76	0.07
E1	GY	2B	AX.89686951	1.42E+08	0.26	-2.60	3.06	0.05
E1	GY	2B	AX.89355285	1.83E+08	0.15	-3.99	4.51	0.08
E1	GY	2B	AX.89662478	1.02E+08	0.07	-4.61	3.07	0.06
E1	GY	2B	AX.89662478	1.02E+08	0.07	-4.61	3.07	0.06
E1	GY	2B	AX.89558174	1.81E+08	0.12	-4.25	4.19	0.08
E1	GY	2B	AX.89407393	1.06E+08	0.08	-5.36	4.87	0.08
E1	GY	2B	AX.89541745	1.82E+08	0.14	-3.82	3.99	0.07
E1	GY	2B	AX.89594592	1.80E+08	0.20	-3.07	3.45	0.06
E1	GY	2B	AX.89318072	1.42E+08	0.26	-2.60	3.06	0.05
E1	GY	2B	AX.89318072	1.42E+08	0.26	-2.60	3.06	0.05
E1	GY	2B	AX.89715595	1.84E+08	0.09	-4.30	3.47	0.06
E1	GY	2B	AX.89403225	1.03E+08	0.07	-5.12	3.89	0.07
E1	SPM2	2B	AX.89360641	7.11E+08	0.23	22.08	3.85	0.07
E1	GY	2B	AX.89460798	1.03E+08	0.07	-4.61	3.07	0.06
E1	GY	2B	AX.89673373	7.64E+08	0.12	-3.88	3.59	0.07
E1	GY	2B	AX.89659140	7.93E+08	0.14	-3.75	3.86	0.07
E1	GY	2B	AX.89450525	7.93E+08	0.34	-2.79	4.01	0.08
E1	GY	2B	AX.89761668	5.94E+08	0.32	2.42	3.02	0.05
E1	GY	2B	AX.89470748	6.62E+08	0.06	-5.81	4.32	0.07
E1	GY	2B	AX.89745248	1.82E+08	0.12	-4.25	4.19	0.08
E1	SPM2	2B	AX.89659164	7.12E+08	0.20	23.05	3.83	0.07
E1	GY	2B	AX.89395850	1.84E+08	0.14	-3.55	3.60	0.06
E1	GY	2B	AX.89454254	1.01E+08	0.07	-4.61	3.07	0.06
E1	GY	2B	AX.89676426	1.82E+08	0.13	-4.30	4.67	0.09
E1	GY	2B	AX.89396028	1.44E+08	0.25	-2.59	3.01	0.05
E1	GY	2B	AX.89729590	1.83E+08	0.14	-3.82	3.99	0.07
E1	GY	2B	AX.89590527	1.33E+08	0.07	-4.72	3.38	0.05

E1	GY	2B	AX.89643712	1.44E+08	0.25	-2.59	3.01	0.05
E1	GY	2B	AX.89349377	1.81E+08	0.12	-4.25	4.19	0.08
E1	GY	2B	AX.89439937	7.85E+08	0.22	-2.74	3.09	0.05
E1	GY	2B	AX.89734149	7.57E+08	0.06	-4.77	3.07	0.05
E1	GY	2B	AX.89327702	1.84E+08	0.08	-4.58	3.70	0.07
E1	GY	2B	AX.89444410	1.81E+08	0.12	-3.46	3.03	0.05
E1	GY	2B	AX.89381154	7.57E+08	0.06	-5.75	3.97	0.06
E1	GY	2B	AX.89644546	7.85E+08	0.24	-2.80	3.32	0.06
E1	GY	2B	AX.89424631	7.52E+08	0.30	-2.69	3.51	0.06
E1	GY	2B	AX.89628479	1.85E+08	0.09	-4.30	3.47	0.06
E1	GY	2B	AX.89679164	1.03E+08	0.07	-4.61	3.07	0.06
E1	GY	2B	AX.89385918	1.79E+08	0.20	-3.07	3.45	0.06
E1	GY	2B	AX.89317506	1.03E+08	0.07	-4.61	3.07	0.06
E1	GY	2B	AX.89317506	1.03E+08	0.07	-4.61	3.07	0.06
E1	GY	2B	AX.89727517	7.57E+08	0.06	-5.01	3.13	0.05
E1	GY	2B	AX.89384745	1.83E+08	0.14	-3.82	3.99	0.07
E1	GY	2B	AX.89375220	1.84E+08	0.14	-4.13	4.69	0.09
E1	GY	2B	AX.89720671	1.79E+08	0.20	-3.07	3.45	0.06
E1	GY	2B	AX.89720671	1.79E+08	0.20	-3.07	3.45	0.06
E1	GY	2B	AX.89463795	5.94E+08	0.33	2.53	3.30	0.06
E1	GY	2B	AX.89379675	1.03E+08	0.07	-4.61	3.07	0.06
E1	GY	2B	AX.89400251	7.59E+08	0.06	-4.77	3.07	0.05
E1	GY	2B	AX.89563399	1.84E+08	0.14	-3.82	3.99	0.07
E1	GY	2B	AX.89454067	1.82E+08	0.13	-4.62	5.44	0.10
E1	GY	2B	AX.89326616	1.02E+08	0.07	-4.61	3.07	0.06
E1	GY	2B	AX.89551300	7.93E+08	0.35	-2.98	4.54	0.09
E1	GY	2B	AX.89396028	1.44E+08	0.25	-2.59	3.01	0.05
E1	GY	2B	AX.89635417	1.81E+08	0.12	-4.25	4.19	0.08
E1	GY	2B	AX.89518028	1.81E+08	0.12	-4.25	4.19	0.08
E1	GY	2B	AX.89518028	1.81E+08	0.12	-4.25	4.19	0.08
E1	SPM2	2B	AX.89483671	7.12E+08	0.20	23.05	3.83	0.07
E1	GY	2B	AX.89647344	7.59E+08	0.06	-4.77	3.07	0.05
E1	GY	2B	AX.89465592	4.53E+05	0.27	-2.54	3.04	0.04
E1	GY	2B	AX.89469521	1.83E+08	0.14	-4.13	4.69	0.09
E1	GY	2B	AX.89600434	1.84E+08	0.09	-4.19	3.45	0.06
E1	GY	2B	AX.89396407	1.04E+08	0.07	-4.61	3.07	0.06
E1	GY	2B	AX.89562902	1.84E+08	0.09	-4.30	3.47	0.06
E1	GY	2B	AX.89351791	1.83E+08	0.14	-3.82	3.99	0.07
E1	GY	2B	AX.89643712	1.44E+08	0.25	-2.59	3.01	0.05
E1	GY	2B	AX.86167754	1.28E+08	0.06	-6.36	5.07	0.08
E1	GY	2B	AX.89722560	1.84E+08	0.14	-3.82	3.99	0.07
E1	GY	2B	AX.89436492	7.93E+08	0.35	-2.98	4.54	0.09
E1	GY	2B	AX.89466177	7.95E+08	0.41	-2.34	3.12	0.05
E1	GY	2B	AX.89458271	5.94E+08	0.32	2.55	3.32	0.06
E1	GY	2B	AX.89327702	1.84E+08	0.08	-4.58	3.70	0.07
E1	GY	2B	AX.89384745	1.83E+08	0.14	-3.82	3.99	0.07
E1	GY	2B	AX.89599952	7.93E+08	0.34	-2.90	4.26	0.08
E1	GY	2B	AX.89572385	7.85E+08	0.25	-2.76	3.35	0.06
E1	GY	2B	AX.86169220	1.84E+08	0.09	-4.30	3.47	0.06
E1	GY	2B	AX.89734860	1.81E+08	0.11	-3.88	3.48	0.06

E1	GY	2B	AX.89549450	1.00E+08	0.07	-4.61	3.07	0.06
E1	GY	2B	AX.89538158	9.00E+06	0.27	-2.52	3.01	0.04
E1	GY	2B	AX.86170531	1.84E+08	0.09	-4.30	3.47	0.06
E1	GY	2B	AX.89605804	1.82E+08	0.14	-4.23	4.88	0.09
E1	GY	2B	AX.89674559	8.60E+06	0.35	-2.48	3.29	0.05
E1	GY	2B	AX.89565875	1.03E+08	0.07	-4.61	3.07	0.06
E1	GY	2B	AX.89589720	1.02E+08	0.07	-5.12	3.89	0.07
E1	GY	2B	AX.89456255	4.60E+04	0.27	-2.52	3.01	0.04
E1	GY	2B	AX.89332441	1.83E+08	0.14	-4.13	4.69	0.09
E1	GY	2B	AX.89602560	1.84E+08	0.09	-4.30	3.47	0.06
E1	GY	2B	AX.89602560	1.84E+08	0.09	-4.30	3.47	0.06
E1	GY	2B	AX.89472547	1.42E+08	0.26	-2.60	3.06	0.05
E1	GY	2B	AX.89614104	6.59E+08	0.06	-5.29	3.43	0.05
E1	GY	2B	AX.89760737	7.63E+08	0.24	-2.69	3.15	0.05
E1	GY	2B	AX.89562902	1.84E+08	0.09	-4.30	3.47	0.06
E1	GY	2B	AX.89732643	1.01E+08	0.07	-4.61	3.07	0.06
E1	GY	2B	AX.89732643	1.01E+08	0.07	-4.61	3.07	0.06
E1	GY	2B	AX.89543258	1.47E+08	0.25	-2.63	3.09	0.05
E1	GY	2B	AX.89614451	1.83E+08	0.14	-3.82	3.99	0.07
E1	GY	2B	AX.89534791	1.02E+08	0.07	-5.12	3.89	0.07
E1	GY	2B	AX.89423320	7.52E+08	0.29	-2.81	3.75	0.07
E1	GY	2B	AX.89761668	5.94E+08	0.32	2.42	3.02	0.05
E1	GY	2B	AX.89599952	7.93E+08	0.34	-2.90	4.26	0.08
E1	GY	2B	AX.89713274	7.75E+08	0.08	-5.32	4.81	0.09
E1	GY	2B	AX.89543659	7.85E+08	0.24	-3.19	4.17	0.07
E1	GY	2B	AX.89404329	9.74E+06	0.27	-2.52	3.01	0.04
E1	GY	2B	AX.89600434	1.84E+08	0.09	-4.19	3.45	0.06
E1	GY	2B	AX.89351791	1.83E+08	0.14	-3.82	3.99	0.07
E1	GY	2B	AX.89722560	1.84E+08	0.14	-3.82	3.99	0.07
E1	GY	2B	AX.89556126	7.90E+08	0.27	-2.69	3.36	0.07
E1	GY	2B	AX.89436572	1.82E+08	0.13	-4.30	4.67	0.09
E1	GY	2B	AX.89330831	5.95E+08	0.33	2.53	3.30	0.06
E1	GY	2B	AX.89726427	7.93E+08	0.35	-2.98	4.54	0.09
E1	GY	2B	AX.89673373	7.64E+08	0.12	-3.88	3.59	0.07
E1	GY	2B	AX.89619964	1.78E+08	0.20	-3.07	3.45	0.06
E1	GY	2B	AX.89552651	1.81E+08	0.12	-4.25	4.19	0.08
E1	GY	2B	AX.89370035	9.11E+06	0.23	-3.62	5.15	0.09
E1	GY	2B	AX.89673819	9.13E+06	0.45	-2.37	3.26	0.06
E1	GY	2B	AX.89424631	7.52E+08	0.30	-2.69	3.51	0.06
E1	GY	2B	AX.89523462	1.84E+08	0.13	-3.81	3.77	0.07
E1	GY	2B	AX.89698976	7.57E+08	0.06	-5.01	3.13	0.05
E1	GY	2B	AX.89739171	7.95E+08	0.41	-2.41	3.30	0.05
E1	GY	2B	AX.89657904	1.41E+08	0.26	-2.60	3.06	0.05
E1	GY	2B	AX.89545829	5.94E+08	0.33	2.53	3.30	0.06
E1	GY	2B	AX.89545829	5.94E+08	0.33	2.53	3.30	0.06
E1	GY	2B	AX.89452576	3.18E+05	0.27	-2.52	3.01	0.04
E1	GY	2B	AX.89686633	5.94E+08	0.32	2.55	3.32	0.06
E1	GY	2B	AX.89654194	1.81E+08	0.11	-3.88	3.48	0.06
E1	GY	2B	AX.89332143	1.82E+08	0.13	-4.62	5.44	0.10
E1	GY	2B	AX.89325475	1.81E+08	0.12	-4.25	4.19	0.08

E1	GY	2B	AX.89513011	9.12E+06	0.40	-2.46	3.39	0.07
E1	GY	2B	AX.89325940	1.83E+08	0.14	-3.82	3.99	0.07
E1	GY	2B	AX.89335901	1.83E+08	0.14	-4.13	4.69	0.09
E1	GY	2B	AX.89463795	5.94E+08	0.33	2.53	3.30	0.06
E1	GY	2B	AX.89326280	1.80E+08	0.20	-3.07	3.45	0.06
E1	GY	2B	AX.89525251	7.57E+08	0.06	-4.77	3.07	0.05
E1	GY	2B	AX.89403432	1.79E+07	0.24	2.84	3.40	0.04
E1	GY	2B	AX.89360195	7.59E+08	0.06	-4.77	3.07	0.05
E1	GY	2B	AX.89563399	1.84E+08	0.14	-3.82	3.99	0.07
E1	GY	2B	AX.89603789	1.03E+08	0.07	-4.61	3.07	0.06
E1	GY	2B	AX.89630862	1.84E+08	0.14	-4.13	4.69	0.09
E1	GY	2B	AX.89729590	1.83E+08	0.14	-3.82	3.99	0.07
E1	GY	2B	AX.89664208	6.61E+08	0.06	-5.29	3.43	0.05
E1	GY	2B	AX.89408490	1.83E+08	0.14	-4.29	4.89	0.09
E1	GY	2B	AX.89443676	7.58E+08	0.06	-4.77	3.07	0.05
E1	GY	2B	AX.89713743	1.84E+08	0.14	-3.82	3.99	0.07
E1	GY	2B	AX.89741917	1.44E+08	0.25	-2.59	3.01	0.05
E1	GY	2B	AX.89349377	1.81E+08	0.12	-4.25	4.19	0.08
E1	GY	2B	AX.89436492	7.93E+08	0.35	-2.98	4.54	0.09
E1	GY	2B	AX.89396912	1.02E+08	0.07	-5.12	3.89	0.07
E1	GY	2B	AX.89749678	6.59E+08	0.06	-5.29	3.43	0.05
E1	GY	2B	AX.89494869	5.94E+08	0.33	2.53	3.30	0.06
E1	GY	2B	AX.89494869	5.94E+08	0.33	2.53	3.30	0.06
E1	SPM2	2B	AX.89343204	6.90E+08	0.20	-20.75	3.14	0.06
E1	GY	2B	AX.89771483	7.57E+08	0.06	-4.77	3.07	0.05
E1	GY	2B	AX.89327795	1.39E+08	0.08	-5.46	4.81	0.09
E1	GY	2B	AX.89338392	7.90E+08	0.12	-4.31	4.43	0.08
E1	GY	2B	AX.89644546	7.85E+08	0.24	-2.80	3.32	0.06
E1	GY	2B	AX.89327830	7.59E+08	0.06	-4.77	3.07	0.05
E1	GY	2B	AX.89491567	1.84E+08	0.14	-3.82	3.99	0.07
E1	GY	2B	AX.89451935	1.79E+07	0.24	2.74	3.24	0.04
E1	SPM2	2B	AX.89618976	7.12E+08	0.20	23.05	3.83	0.07
E1	GY	2B	AX.89750183	5.63E+08	0.18	-3.03	3.22	0.05
E1	GY	2B	AX.89410146	1.84E+08	0.14	-4.13	4.69	0.09
E1	GY	2B	AX.89405839	1.81E+08	0.12	-4.25	4.19	0.08
E1	GY	2B	AX.89480571	1.83E+08	0.14	-3.82	3.99	0.07
E1	GY	2B	AX.89463396	1.84E+08	0.12	-4.28	4.38	0.08
E1	GY	2B	AX.89317632	7.64E+08	0.14	-3.30	3.10	0.06
E1	GY	2B	AX.89456255	4.60E+04	0.27	-2.52	3.01	0.04
E1	GY	2B	AX.89375220	1.84E+08	0.14	-4.13	4.69	0.09
E1	SPM2	2B	AX.89648863	7.12E+08	0.20	23.05	3.83	0.07
E1	GY	2B	AX.89562158	6.60E+08	0.06	-5.29	3.43	0.05
E1	GY	2B	AX.89445883	1.83E+08	0.14	-3.82	3.99	0.07
E1	GY	2B	AX.89399328	1.83E+08	0.14	-3.82	3.99	0.07
E1	GY	2B	AX.89539161	1.82E+08	0.13	-4.62	5.44	0.10
E1	GY	2B	AX.89682796	1.84E+08	0.09	-4.72	4.24	0.08
E1	GY	2B	AX.89326280	1.80E+08	0.20	-3.07	3.45	0.06
E1	GY	2B	AX.89400073	7.53E+08	0.29	-2.61	3.29	0.06
E1	GY	2B	AX.89525251	7.57E+08	0.06	-4.77	3.07	0.05
E1	GY	2B	AX.89329130	1.80E+07	0.24	2.84	3.40	0.04

E1	GY	2B	AX.89493461	1.83E+08	0.14	-4.13	4.69	0.09
E1	GY	2B	AX.89658972	1.80E+08	0.20	-3.07	3.45	0.06
E1	GY	2B	AX.89591171	1.83E+08	0.14	-3.82	3.99	0.07
E1	GY	2B	AX.89387913	1.47E+08	0.20	-2.92	3.17	0.05
E1	GY	2B	AX.89447263	5.15E+05	0.27	-2.52	3.01	0.04
E1	GY	2B	AX.89505960	7.46E+08	0.35	-2.35	3.01	0.05
E1	GY	2B	AX.89647582	1.29E+08	0.06	-6.36	5.07	0.08
E1	GY	2B	AX.86167517	7.89E+08	0.09	-4.11	3.20	0.05
E1	GY	2B	AX.86167517	7.89E+08	0.09	-4.11	3.20	0.05
E1	GY	2B	AX.89377106	1.01E+08	0.07	-4.61	3.07	0.06
E1	GY	2B	AX.86167754	1.28E+08	0.06	-6.36	5.07	0.08
E1	GY	2B	AX.89770787	1.43E+08	0.26	-2.73	3.32	0.06
E1	GY	2B	AX.89537259	1.01E+08	0.07	-4.61	3.07	0.06
E1	GY	2B	AX.89470748	6.62E+08	0.06	-5.81	4.32	0.07
E1	GY	2B	AX.89588628	7.59E+08	0.06	-4.77	3.07	0.05
E1	GY	2B	AX.89612574	1.85E+08	0.09	-4.30	3.47	0.06
E1	GY	2B	AX.89589315	7.59E+08	0.06	-4.77	3.07	0.05
E1	GY	2B	AX.89342802	1.82E+08	0.12	-4.25	4.19	0.08
E1	GY	2B	AX.89342802	1.82E+08	0.12	-4.25	4.19	0.08
E1	GY	2B	AX.89699216	1.82E+08	0.11	-3.88	3.48	0.06
E1	GY	2B	AX.89542229	1.02E+08	0.07	-4.61	3.07	0.06
E1	GY	2B	AX.89719172	3.65E+05	0.27	-2.52	3.01	0.04
E1	GY	2B	AX.89472547	1.42E+08	0.26	-2.60	3.06	0.05
E1	GY	2B	AX.89736400	1.82E+08	0.13	-4.30	4.67	0.09
E1	GY	2B	AX.89504410	1.83E+08	0.14	-3.82	3.99	0.07
E1	GY	2B	AX.89332789	1.80E+08	0.20	-3.07	3.45	0.06
E1	GY	2B	AX.89602946	1.02E+08	0.07	-4.61	3.07	0.06
E1	GY	2B	AX.89460581	1.43E+08	0.25	-2.76	3.31	0.06
E1	GY	2B	AX.89731365	1.41E+08	0.26	-2.60	3.06	0.05
E1	SPM2	2B	AX.89523019	7.12E+08	0.21	24.96	4.47	0.08
E1	GY	2B	AX.89493461	1.83E+08	0.14	-4.13	4.69	0.09
E1	GY	2B	AX.86164438	7.85E+08	0.22	-2.74	3.09	0.05
E1	GY	2B	AX.89450525	7.93E+08	0.34	-2.79	4.01	0.08
E1	GY	2B	AX.89581448	1.01E+08	0.07	-5.12	3.89	0.07
E1	GY	2B	AX.89416037	1.83E+08	0.14	-4.13	4.69	0.09
E1	GY	2B	AX.89416037	1.83E+08	0.14	-4.13	4.69	0.09
E1	GY	2B	AX.89688716	7.93E+08	0.17	-3.41	3.81	0.07
E1	GY	2B	AX.89737804	1.79E+08	0.20	-3.07	3.45	0.06
E1	GY	2B	AX.89451061	1.04E+08	0.07	-4.61	3.07	0.06
E1	GY	2B	AX.89770628	7.77E+08	0.49	-2.39	3.34	0.05
E1	GY	2B	AX.89323675	6.59E+08	0.06	-5.29	3.43	0.05
E1	GY	2B	AX.89689244	8.00E+08	0.08	-4.70	3.71	0.06
E1	GY	2B	AX.89474590	1.82E+08	0.12	-4.25	4.19	0.08
E1	GY	2B	AX.89369630	1.46E+08	0.22	-2.73	3.03	0.05
E1	GY	2B	AX.89439937	7.85E+08	0.22	-2.74	3.09	0.05
E1	GY	2B	AX.89616056	5.94E+08	0.32	2.55	3.32	0.06
E1	GY	2B	AX.89734127	7.59E+08	0.05	-6.10	4.09	0.07
E1	GY	2B	AX.89436572	1.82E+08	0.13	-4.30	4.67	0.09
E1	GY	2B	AX.89725266	1.83E+08	0.14	-3.82	3.99	0.07
E1	GY	2B	AX.89487985	1.83E+08	0.14	-3.82	3.99	0.07

E1	GY	2B	AX.89585973	6.15E+08	0.33	2.49	3.22	0.06
E1	GY	2B	AX.86169220	1.84E+08	0.09	-4.30	3.47	0.06
E1	GY	2B	AX.89690045	1.82E+08	0.13	-4.62	5.44	0.10
E1	GY	2B	AX.89593250	1.81E+08	0.12	-4.25	4.19	0.08
E1	GY	2B	AX.89405839	1.81E+08	0.12	-4.25	4.19	0.08
E1	GY	2B	AX.89381365	1.43E+08	0.25	-2.76	3.31	0.06
E1	GY	2B	AX.89381365	1.43E+08	0.25	-2.76	3.31	0.06
E1	GY	2B	AX.89628479	1.85E+08	0.09	-4.30	3.47	0.06
E1	GY	2B	AX.89709245	1.29E+08	0.06	-6.36	5.07	0.08
E1	GY	2B	AX.89575510	1.34E+08	0.06	-6.36	5.07	0.08
E1	GY	2B	AX.89313148	7.85E+08	0.25	-2.76	3.35	0.06
E1	GY	2B	AX.89325475	1.81E+08	0.12	-4.25	4.19	0.08
E1	GY	2B	AX.89666887	1.30E+08	0.07	-6.79	6.05	0.10
E1	GY	2B	AX.89325940	1.83E+08	0.14	-3.82	3.99	0.07
E1	GY	2B	AX.89569931	6.62E+08	0.06	-5.81	4.32	0.07
E1	SPM2	2B	AX.89578062	7.12E+08	0.20	23.05	3.83	0.07
E1	GY	2B	AX.89679164	1.03E+08	0.07	-4.61	3.07	0.06
E1	GY	2B	AX.89359988	1.78E+08	0.20	-3.07	3.45	0.06
E1	GY	2B	AX.89464649	1.84E+08	0.14	-3.55	3.60	0.06
E1	GY	2B	AX.89655408	1.01E+08	0.07	-4.61	3.07	0.06
E1	GY	2B	AX.89765862	6.60E+08	0.06	-5.29	3.43	0.05
E1	GY	2B	AX.89490236	4.74E+04	0.27	-2.52	3.01	0.04
E1	GY	2B	AX.89326616	1.02E+08	0.07	-4.61	3.07	0.06
E1	GY	2B	AX.89692891	1.02E+08	0.07	-4.61	3.07	0.06
E1	GY	2B	AX.89692891	1.02E+08	0.07	-4.61	3.07	0.06
E1	GY	2B	AX.89679762	1.43E+08	0.25	-2.76	3.31	0.06
E1	GY	2B	AX.89408291	1.40E+08	0.13	-3.50	3.18	0.06
E1	GY	2B	AX.89323244	1.83E+08	0.14	-4.13	4.69	0.09
E1	GY	2B	AX.89761980	7.94E+08	0.11	-4.21	3.87	0.07
E1	GY	2B	AX.89408490	1.83E+08	0.14	-4.29	4.89	0.09
E1	GY	2B	AX.89626799	1.82E+08	0.11	-3.88	3.48	0.06
E1	GY	2B	AX.89465592	4.53E+05	0.27	-2.54	3.04	0.04
E1	GY	2B	AX.89526250	1.01E+08	0.07	-4.61	3.07	0.06
E1	GY	2B	AX.89668347	7.64E+08	0.12	-3.88	3.59	0.07
E1	GY	2B	AX.89373025	1.41E+08	0.26	-2.60	3.06	0.05
E1	GY	2B	AX.89373025	1.41E+08	0.26	-2.60	3.06	0.05
E1	GY	2B	AX.89461833	6.59E+08	0.06	-5.29	3.43	0.05
E1	GY	2B	AX.89644113	1.84E+08	0.12	-4.27	4.24	0.08
E1	GY	2B	AX.89596143	1.02E+08	0.07	-4.61	3.07	0.06
E1	GY	2B	AX.89742669	7.57E+08	0.06	-5.01	3.13	0.05
E1	GY	2B	AX.89742669	7.57E+08	0.06	-5.01	3.13	0.05
E1	GY	2B	AX.89440441	1.31E+08	0.06	-5.75	4.24	0.07
E1	GY	2B	AX.89444410	1.81E+08	0.12	-3.46	3.03	0.05
E1	GY	2B	AX.89572385	7.85E+08	0.25	-2.76	3.35	0.06
E1	GY	2B	AX.89694199	1.40E+08	0.24	-2.80	3.35	0.06
E1	GY	2B	AX.89694199	1.40E+08	0.24	-2.80	3.35	0.06
E1	GY	2B	AX.89549378	1.47E+08	0.20	-2.92	3.17	0.05
E1	GY	2B	AX.89549450	1.00E+08	0.07	-4.61	3.07	0.06
E1	GY	2B	AX.89463274	6.59E+08	0.06	-5.29	3.43	0.05
E1	GY	2B	AX.89463274	6.59E+08	0.06	-5.29	3.43	0.05

E1	GY	2B	AX.89385918	1.79E+08	0.20	-3.07	3.45	0.06
E1	GY	2B	AX.89463396	1.84E+08	0.12	-4.28	4.38	0.08
E1	GY	2B	AX.89351218	6.59E+08	0.06	-5.81	4.32	0.07
E1	GY	2B	AX.89317632	7.64E+08	0.14	-3.30	3.10	0.06
E1	GY	2B	AX.89562233	7.64E+08	0.12	-3.88	3.59	0.07
E1	GY	2B	AX.89562233	7.64E+08	0.12	-3.88	3.59	0.07
E1	GY	2B	AX.89445890	1.79E+08	0.20	-2.86	3.10	0.05
E1	GY	2B	AX.89539161	1.82E+08	0.13	-4.62	5.44	0.10
E1	GY	2B	AX.89351692	7.52E+08	0.29	-2.84	3.82	0.07
E1	GY	2B	AX.89411485	4.88E+04	0.27	-2.52	3.01	0.04
E1	GY	2B	AX.89351692	7.52E+08	0.29	-2.84	3.82	0.07
E1	GY	2B	AX.89411485	4.88E+04	0.27	-2.52	3.01	0.04
E1	GY	2B	AX.89528928	3.65E+05	0.27	-2.52	3.01	0.04
E1	GY	2B	AX.89375686	6.61E+08	0.06	-5.29	3.43	0.05
E1	GY	2B	AX.89496808	4.39E+04	0.27	-2.52	3.01	0.04
E1	GY	2B	AX.89329130	1.80E+07	0.24	2.84	3.40	0.04
E1	GY	2B	AX.89687896	1.02E+08	0.07	-4.61	3.07	0.06
E1	GY	2B	AX.89658972	1.80E+08	0.20	-3.07	3.45	0.06
E1	GY	2B	AX.89423116	5.95E+08	0.33	2.53	3.30	0.06
E1	GY	2B	AX.89400251	7.59E+08	0.06	-4.77	3.07	0.05
E1	GY	2B	AX.89423320	7.52E+08	0.29	-2.81	3.75	0.07
E1	GY	2B	AX.89529518	5.94E+08	0.32	2.55	3.32	0.06
E1	GY	2B	AX.89574794	1.02E+08	0.07	-4.61	3.07	0.06
E1	GY	2B	AX.89483240	7.95E+08	0.41	-2.41	3.30	0.05
E1	GY	2B	AX.89635417	1.81E+08	0.12	-4.25	4.19	0.08
E1	GY	2B	AX.89494298	7.60E+08	0.06	-4.77	3.07	0.05
E1	GY	2B	AX.89387913	1.47E+08	0.20	-2.92	3.17	0.05
E1	GY	2B	AX.89650120	1.78E+08	0.20	-3.07	3.45	0.06
E1	GY	2B	AX.89536672	7.85E+08	0.22	-2.74	3.09	0.05
E1	GY	2B	AX.89536672	7.85E+08	0.22	-2.74	3.09	0.05
E1	GY	2B	AX.89413015	6.59E+08	0.06	-5.29	3.43	0.05
E1	GY	2B	AX.89770787	1.43E+08	0.26	-2.73	3.32	0.06
E1	GY	2B	AX.89552327	1.78E+08	0.20	-3.07	3.45	0.06
E1	GY	2B	AX.89341825	1.02E+08	0.07	-5.12	3.89	0.07
E1	GY	2B	AX.89341825	1.02E+08	0.07	-5.12	3.89	0.07
E1	GY	2B	AX.89537014	7.93E+08	0.27	-2.56	3.03	0.05
E1	GY	2B	AX.89458271	5.94E+08	0.32	2.55	3.32	0.06
E1	GY	2B	AX.89717315	1.79E+08	0.20	-2.86	3.10	0.05
E1	GY	2B	AX.89698843	1.83E+08	0.14	-4.13	4.69	0.09
E1	GY	2B	AX.89353728	1.82E+08	0.13	-4.30	4.67	0.09
E1	GY	2B	AX.89703625	6.62E+08	0.06	-5.29	3.43	0.05
E1	GY	2B	AX.89448342	1.01E+08	0.07	-4.61	3.07	0.06
E1	GY	2B	AX.89448342	1.01E+08	0.07	-4.61	3.07	0.06
E1	GY	2B	AX.89603427	1.87E+07	0.35	2.47	3.26	0.04
E1	GY	2B	AX.89515639	5.95E+08	0.32	2.45	3.08	0.05
E1	GY	2B	AX.89507099	1.55E+08	0.19	-3.44	4.14	0.06
E1	GY	2B	AX.89541745	1.82E+08	0.14	-3.82	3.99	0.07
E1	GY	2B	AX.89331795	1.79E+08	0.20	-2.86	3.10	0.05
E1	GY	2B	AX.89561621	7.57E+08	0.06	-5.01	3.13	0.05
E1	GY	2B	AX.89561621	7.57E+08	0.06	-5.01	3.13	0.05

E1	GY	2B	AX.89565875	1.03E+08	0.07	-4.61	3.07	0.06
E1	GY	2B	AX.89332143	1.82E+08	0.13	-4.62	5.44	0.10
E1	GY	2B	AX.89531346	7.82E+08	0.34	-2.60	3.52	0.06
E1	GY	2B	AX.89355285	1.83E+08	0.15	-3.99	4.51	0.08
E1	GY	2B	AX.89506146	1.01E+08	0.07	-4.61	3.07	0.06
E1	GY	2B	AX.89590619	1.81E+08	0.12	-4.25	4.19	0.08
E1	GY	2B	AX.89486060	1.80E+08	0.20	-3.07	3.45	0.06
E1	GY	2B	AX.89765084	1.81E+08	0.12	-4.25	4.19	0.08
E1	GY	2B	AX.89765084	1.81E+08	0.12	-4.25	4.19	0.08
E1	GY	2B	AX.89403225	1.03E+08	0.07	-5.12	3.89	0.07
E1	GY	2B	AX.89680788	7.52E+08	0.29	-2.84	3.82	0.07
E1	GY	2B	AX.89368860	6.59E+08	0.06	-5.29	3.43	0.05
E1	GY	2B	AX.89663766	1.43E+08	0.25	-2.76	3.36	0.06
E1	GY	2B	AX.89581448	1.01E+08	0.07	-5.12	3.89	0.07
E1	GY	2B	AX.89603789	1.03E+08	0.07	-4.61	3.07	0.06
E1	GY	2B	AX.89323244	1.83E+08	0.14	-4.13	4.69	0.09
E1	GY	2B	AX.89450884	5.95E+08	0.31	2.44	3.04	0.05
E1	GY	2B	AX.89775110	1.29E+08	0.06	-6.36	5.07	0.08
E1	GY	2B	AX.89497642	1.82E+08	0.12	-4.25	4.19	0.08
E1	GY	2B	AX.89626847	1.81E+08	0.11	-3.88	3.48	0.06
E1	GY	2B	AX.89626847	1.81E+08	0.11	-3.88	3.48	0.06
E1	GY	2B	AX.89664717	4.57E+04	0.27	-2.52	3.01	0.04
E1	GY	2B	AX.89664717	4.57E+04	0.27	-2.52	3.01	0.04
E1	GY	2B	AX.89556126	7.90E+08	0.27	-2.69	3.36	0.07
E1	GY	2B	AX.89537014	7.93E+08	0.27	-2.56	3.03	0.05
E1	GY	2B	AX.89726427	7.93E+08	0.35	-2.98	4.54	0.09
E1	GY	2B	AX.89719069	7.57E+08	0.06	-4.77	3.07	0.05
E1	GY	2B	AX.89750183	5.63E+08	0.18	-3.03	3.22	0.05
E1	GY	2B	AX.89593250	1.81E+08	0.12	-4.25	4.19	0.08
E1	GY	2B	AX.89393550	7.59E+08	0.06	-4.77	3.07	0.05
E1	GY	2B	AX.89393550	7.59E+08	0.06	-4.77	3.07	0.05
E1	GY	2B	AX.89358906	7.57E+08	0.06	-4.77	3.07	0.05
E1	GY	2B	AX.89358906	7.57E+08	0.06	-4.77	3.07	0.05
E1	GY	2B	AX.89534791	1.02E+08	0.07	-5.12	3.89	0.07
E1	GY	2B	AX.89606237	1.47E+08	0.25	-2.63	3.09	0.05
E1	GY	2B	AX.89535150	1.02E+08	0.07	-5.12	3.89	0.07
E1	GY	2B	AX.89500123	7.85E+08	0.22	-2.74	3.09	0.05
E1	GY	2B	AX.89500123	7.85E+08	0.22	-2.74	3.09	0.05
E1	GY	2B	AX.89359515	5.94E+08	0.33	2.55	3.34	0.06
E1	GY	2B	AX.89569931	6.62E+08	0.06	-5.81	4.32	0.07
E1	GY	2B	AX.89442198	1.02E+08	0.07	-4.61	3.07	0.06
E1	GY	2B	AX.89326350	1.81E+08	0.20	-3.07	3.45	0.06
E1	GY	2B	AX.89326350	1.81E+08	0.20	-3.07	3.45	0.06
E1	GY	2B	AX.89314257	1.43E+08	0.26	-2.60	3.06	0.05
E1	GY	2B	AX.89464649	1.84E+08	0.14	-3.55	3.60	0.06
E1	GY	2B	AX.89500920	1.79E+08	0.20	-2.86	3.10	0.05
E1	GY	2B	AX.89769704	7.58E+08	0.06	-4.77	3.07	0.05
E1	GY	2B	AX.89680788	7.52E+08	0.29	-2.84	3.82	0.07
E1	GY	2B	AX.89561986	1.81E+08	0.11	-3.70	3.10	0.06
E1	GY	2B	AX.89464930	1.42E+08	0.26	-2.60	3.06	0.05

E1	GY	2B	AX.89559315	8.39E+06	0.27	-2.52	3.01	0.04
E1	GY	2B	AX.89443026	5.95E+08	0.33	2.53	3.30	0.06
E1	GY	2B	AX.89570875	1.43E+08	0.25	-2.76	3.31	0.06
E1	GY	2B	AX.89454401	7.84E+08	0.30	-3.42	5.36	0.09
E1	GY	2B	AX.89461833	6.59E+08	0.06	-5.29	3.43	0.05
E1	GY	2B	AX.89608525	1.04E+08	0.07	-5.12	3.89	0.07
E1	GY	2B	AX.89741917	1.44E+08	0.25	-2.59	3.01	0.05
E1	GY	2B	AX.89560360	3.87E+05	0.27	-2.52	3.01	0.04
E1	GY	2B	AX.89742203	1.03E+08	0.07	-4.61	3.07	0.06
E1	GY	2B	AX.89742203	1.03E+08	0.07	-4.61	3.07	0.06
E1	GY	2B	AX.89433133	3.30E+05	0.27	-2.52	3.01	0.04
E1	GY	2B	AX.89619964	1.78E+08	0.20	-3.07	3.45	0.06
E1	GY	2B	AX.89560733	5.95E+08	0.33	2.53	3.30	0.06
E1	GY	2B	AX.89397326	1.79E+08	0.20	-3.07	3.45	0.06
E1	GY	2B	AX.89693837	1.40E+08	0.25	-2.63	3.09	0.05
E1	GY	2B	AX.89726785	6.62E+08	0.06	-5.29	3.43	0.05
E1	GY	2B	AX.89572391	1.82E+08	0.13	-4.30	4.67	0.09
E1	GY	2B	AX.89641042	7.65E+08	0.12	-3.88	3.59	0.07
E1	GY	2B	AX.89564960	1.80E+08	0.20	-3.07	3.45	0.06
E1	GY	2B	AX.89491567	1.84E+08	0.14	-3.82	3.99	0.07
E1	GY	2B	AX.89564994	1.81E+08	0.11	-3.88	3.48	0.06
E1	GY	2B	AX.89549272	1.34E+08	0.06	-6.36	5.07	0.08
E1	GY	2B	AX.89549378	1.47E+08	0.20	-2.92	3.17	0.05
E1	GY	2B	AX.89316984	1.83E+08	0.14	-3.82	3.99	0.07
E1	GY	2B	AX.89538158	9.00E+06	0.27	-2.52	3.01	0.04
E1	GY	2B	AX.89669665	1.82E+08	0.13	-4.30	4.67	0.09
E1	GY	2B	AX.89669665	1.82E+08	0.13	-4.30	4.67	0.09
E1	GY	2B	AX.89681803	7.59E+08	0.06	-5.01	3.13	0.05
E1	GY	2B	AX.89480571	1.83E+08	0.14	-3.82	3.99	0.07
E1	GY	2B	AX.89621449	1.02E+08	0.07	-4.61	3.07	0.06
E1	GY	2B	AX.89363748	1.03E+08	0.07	-4.61	3.07	0.06
E1	GY	2B	AX.89363748	1.03E+08	0.07	-4.61	3.07	0.06
E1	GY	2B	AX.89386320	4.52E+04	0.27	-2.52	3.01	0.04
E1	GY	2B	AX.89434536	1.82E+08	0.13	-4.62	5.44	0.10
E1	GY	2B	AX.89755632	7.93E+08	0.26	-2.79	3.49	0.07
E1	GY	2B	AX.89755743	1.02E+08	0.07	-4.61	3.07	0.06
E1	GY	2B	AX.89631015	1.40E+08	0.24	-2.80	3.35	0.06
E1	GY	2B	AX.89493544	1.00E+08	0.07	-5.12	3.89	0.07
E1	GY	2B	AX.89423116	5.95E+08	0.33	2.53	3.30	0.06
E1	GY	2B	AX.89469345	2.07E+07	0.08	4.32	3.21	0.05
E1	GY	2B	AX.89469521	1.83E+08	0.14	-4.13	4.69	0.09
E1	GY	2B	AX.89647582	1.29E+08	0.06	-6.36	5.07	0.08
E1	GY	2B	AX.89575490	1.81E+08	0.12	-4.25	4.19	0.08
E1	GY	2B	AX.89458240	1.82E+08	0.13	-4.30	4.67	0.09
E1	GY	2B	AX.89710344	7.57E+08	0.07	-5.04	3.58	0.06
E1	GY	2B	AX.89401624	4.53E+05	0.27	-2.52	3.01	0.04
E1	GY	2B	AX.89771483	7.57E+08	0.06	-4.77	3.07	0.05
E1	GY	2B	AX.89552651	1.81E+08	0.12	-4.25	4.19	0.08
E1	GY	2B	AX.89673819	9.13E+06	0.45	-2.37	3.26	0.06
E1	GY	2B	AX.89734860	1.81E+08	0.11	-3.88	3.48	0.06

E1	GY	2B	AX.89507099	1.55E+08	0.19	-3.44	4.14	0.06
E1	GY	2B	AX.89496140	5.06E+05	0.27	-2.52	3.01	0.04
E1	GY	2B	AX.89686633	5.94E+08	0.32	2.55	3.32	0.06
E1	GY	2B	AX.89542229	1.02E+08	0.07	-4.61	3.07	0.06
E1	GY	2B	AX.89637464	7.60E+08	0.06	-4.77	3.07	0.05
E1	GY	2B	AX.89637464	7.60E+08	0.06	-4.77	3.07	0.05
E1	GY	2B	AX.89332441	1.83E+08	0.14	-4.13	4.69	0.09
E1	GY	2B	AX.89577473	7.58E+08	0.06	-4.77	3.07	0.05
E1	GY	2B	AX.89614104	6.59E+08	0.06	-5.29	3.43	0.05
E1	GY	2B	AX.89504410	1.83E+08	0.14	-3.82	3.99	0.07
E1	GY	2B	AX.89486060	1.80E+08	0.20	-3.07	3.45	0.06
E1	GY	2B	AX.89332789	1.80E+08	0.20	-3.07	3.45	0.06
E1	GY	2B	AX.89460581	1.43E+08	0.25	-2.76	3.31	0.06
E1	GY	2B	AX.89543258	1.47E+08	0.25	-2.63	3.09	0.05
E1	GY	2B	AX.89614451	1.83E+08	0.14	-3.82	3.99	0.07
E1	GY	2B	AX.89368225	7.85E+08	0.10	-3.98	3.28	0.05
E1	GY	2B	AX.89403432	1.79E+07	0.24	2.84	3.40	0.04
E1	GY	2B	AX.89403445	7.52E+08	0.27	-2.53	3.00	0.05
E1	GY	2B	AX.89749024	1.82E+08	0.13	-4.30	4.67	0.09
E1	GY	2B	AX.89323590	1.43E+08	0.25	-2.76	3.31	0.06
E1	GY	2B	AX.89323590	1.43E+08	0.25	-2.76	3.31	0.06
E1	GY	2B	AX.89474590	1.82E+08	0.12	-4.25	4.19	0.08
E1	GY	2B	AX.89369934	7.51E+08	0.33	-2.41	3.06	0.05
E1	GY	2B	AX.89552764	7.52E+08	0.29	-2.52	3.10	0.05
E1	GY	2B	AX.89552764	7.52E+08	0.29	-2.52	3.10	0.05
E1	GY	2B	AX.89393166	7.57E+08	0.07	-5.13	3.90	0.06
E1	GY	2B	AX.89416940	7.64E+08	0.12	-3.88	3.59	0.07
E1	GY	2B	AX.89416940	7.64E+08	0.12	-3.88	3.59	0.07
E1	GY	2B	AX.89588768	6.60E+08	0.06	-5.29	3.43	0.05
E1	GY	2B	AX.89370035	9.11E+06	0.23	-3.62	5.15	0.09
E1	GY	2B	AX.89452013	1.82E+08	0.14	-4.28	4.87	0.09
E1	GY	2B	AX.89616747	1.03E+08	0.07	-4.61	3.07	0.06
E1	GY	2B	AX.89523462	1.84E+08	0.13	-3.81	3.77	0.07
E1	GY	2B	AX.89507172	1.79E+08	0.20	-3.07	3.45	0.06
E1	GY	2B	AX.89698976	7.57E+08	0.06	-5.01	3.13	0.05
E1	GY	2B	AX.89739171	7.95E+08	0.41	-2.41	3.30	0.05
E1	GY	2B	AX.89678145	7.95E+08	0.20	-2.87	3.12	0.05
E1	GY	2B	AX.89727377	5.94E+08	0.33	2.53	3.30	0.06
E1	GY	2B	AX.89617368	1.01E+08	0.07	-4.61	3.07	0.06
E1	GY	2B	AX.89617425	3.15E+05	0.28	-2.51	3.02	0.04
E1	GY	2B	AX.89499795	6.62E+08	0.06	-5.29	3.43	0.05
E1	GY	2B	AX.89535150	1.02E+08	0.07	-5.12	3.89	0.07
E1	GY	2B	AX.89682491	1.81E+08	0.12	-3.46	3.03	0.05
E1	GY	2B	AX.89513011	9.12E+06	0.40	-2.46	3.39	0.07
E1	SPM2	2B	AX.89724036	7.09E+08	0.27	19.03	3.28	0.06
E1	GY	2B	AX.89728726	6.62E+08	0.06	-5.29	3.43	0.05
E1	GY	2B	AX.89359988	1.78E+08	0.20	-3.07	3.45	0.06
E1	GPS	2B	AX.89709253	9.30E+07	0.39	1.29	3.72	0.06
E1	GY	2B	AX.89536238	7.90E+08	0.13	-3.99	3.98	0.07
E1	GY	2B	AX.89443026	5.95E+08	0.33	2.53	3.30	0.06

E1	GY	2B	AX.89679762	1.43E+08	0.25	-2.76	3.31	0.06
E1	GY	2B	AX.89630862	1.84E+08	0.14	-4.13	4.69	0.09
E1	GY	2B	AX.89626799	1.82E+08	0.11	-3.88	3.48	0.06
E1	GY	2B	AX.89408614	7.65E+08	0.12	-3.88	3.59	0.07
E1	GY	2B	AX.89443676	7.58E+08	0.06	-4.77	3.07	0.05
E1	GY	2B	AX.89745765	1.02E+08	0.07	-4.61	3.07	0.06
E1	GY	2B	AX.89608525	1.04E+08	0.07	-5.12	3.89	0.07
E1	GY	2B	AX.89373157	1.84E+08	0.14	-4.29	4.89	0.09
E1	GY	2B	AX.89718549	1.00E+08	0.07	-4.61	3.07	0.06
E1	GY	2B	AX.89396912	1.02E+08	0.07	-5.12	3.89	0.07
E1	GPS	2B	AX.89603017	4.79E+06	0.49	1.14	3.18	0.05
E1	GY	2B	AX.89656746	7.93E+08	0.31	-2.98	4.25	0.09
E1	GY	2B	AX.89338392	7.90E+08	0.12	-4.31	4.43	0.08
E1	GY	2B	AX.89327830	7.59E+08	0.06	-4.77	3.07	0.05
E1	GY	2B	AX.89549272	1.34E+08	0.06	-6.36	5.07	0.08
E1	GY	2B	AX.89338416	7.60E+08	0.06	-4.77	3.07	0.05
E1	GY	2B	AX.89410146	1.84E+08	0.14	-4.13	4.69	0.09
E1	GY	2B	AX.89374293	1.02E+08	0.07	-5.12	3.89	0.07
E1	GY	2B	AX.89743242	1.90E+07	0.38	-2.33	3.03	0.04
E1	GY	2B	AX.89708086	1.84E+08	0.14	-3.82	3.99	0.07
E1	GY	2B	AX.89480023	1.81E+08	0.12	-4.25	4.19	0.08
E1	GY	2B	AX.89560172	7.46E+08	0.35	-2.36	3.03	0.05
E1	GY	2B	AX.89681803	7.59E+08	0.06	-5.01	3.13	0.05
E1	GPS	2B	AX.89314636	9.12E+07	0.40	1.13	3.02	0.05
E1	GY	2B	AX.89703355	1.81E+08	0.12	-4.25	4.19	0.08
E1	GPS	2B	AX.89350212	1.12E+07	0.38	-1.23	3.43	0.06
E1	GY	2B	AX.89339086	1.41E+08	0.26	-2.60	3.06	0.05
E1	GY	2B	AX.89730333	1.81E+08	0.12	-3.82	3.48	0.06
E1	GY	2B	AX.89730333	1.81E+08	0.12	-3.82	3.48	0.06
E1	GY	2B	AX.89560602	7.60E+08	0.06	-5.75	3.97	0.06
E1	GY	2B	AX.89641351	3.69E+05	0.27	-2.52	3.01	0.04
E1	GY	2B	AX.89445883	1.83E+08	0.14	-3.82	3.99	0.07
E1	GY	2B	AX.89422340	7.89E+08	0.10	-3.75	3.07	0.05
E1	GY	2B	AX.89434536	1.82E+08	0.13	-4.62	5.44	0.10
E1	GY	2B	AX.89728349	1.84E+08	0.14	-3.82	3.99	0.07
E1	GY	2B	AX.89728349	1.84E+08	0.14	-3.82	3.99	0.07
E1	GY	2B	AX.89621714	1.83E+08	0.14	-4.13	4.69	0.09
E1	GY	2B	AX.89682796	1.84E+08	0.09	-4.72	4.24	0.08
E1	GY	2B	AX.89658674	6.27E+05	0.27	-2.52	3.01	0.04
E1	GY	2B	AX.89658674	6.27E+05	0.27	-2.52	3.01	0.04
E1	GY	2B	AX.89755743	1.02E+08	0.07	-4.61	3.07	0.06
E1	GY	2B	AX.89621767	1.44E+08	0.25	-2.59	3.01	0.05
E1	GY	2B	AX.89621767	1.44E+08	0.25	-2.59	3.01	0.05
E1	GY	2B	AX.89375686	6.61E+08	0.06	-5.29	3.43	0.05
E1	GY	2B	AX.89400073	7.53E+08	0.29	-2.61	3.29	0.06
E1	GY	2B	AX.89659140	7.93E+08	0.14	-3.75	3.86	0.07
E1	GY	2B	AX.89752463	1.14E+07	0.10	3.73	3.05	0.05
E1	GY	2B	AX.86164438	7.85E+08	0.22	-2.74	3.09	0.05
E1	GY	2B	AX.89688062	1.84E+08	0.08	-4.58	3.70	0.07
E1	GY	2B	AX.89483240	7.95E+08	0.41	-2.41	3.30	0.05

E1	GY	2B	AX.89555674	1.80E+08	0.20	-3.07	3.45	0.06
E1	GPS	2B	AX.89612780	9.58E+07	0.40	1.22	3.42	0.06
E1	GY	2B	AX.89483565	6.62E+08	0.06	-5.29	3.43	0.05
E1	GY	2B	AX.89483565	6.62E+08	0.06	-5.29	3.43	0.05
E1	GY	2B	AX.89469711	1.85E+08	0.09	-4.30	3.47	0.06
E1	GY	2B	AX.89447263	5.15E+05	0.27	-2.52	3.01	0.04
E1	GY	2B	AX.89605178	1.03E+08	0.07	-4.61	3.07	0.06
E1	GY	2B	AX.89762335	1.47E+08	0.25	-2.61	3.04	0.05
E1	GY	2B	AX.89762335	1.47E+08	0.25	-2.61	3.04	0.05
E1	GPS	2B	AX.89667403	1.12E+07	0.38	-1.19	3.22	0.05
E1	GY	2B	AX.89413015	6.59E+08	0.06	-5.29	3.43	0.05
E1	GY	2B	AX.89734127	7.59E+08	0.05	-6.10	4.09	0.07
E1	GY	2B	AX.89734149	7.57E+08	0.06	-4.77	3.07	0.05
E1	GY	2B	AX.89717315	1.79E+08	0.20	-2.86	3.10	0.05
E1	GY	2B	AX.89631602	7.93E+08	0.35	-2.98	4.54	0.09
E1	GY	2B	AX.89725266	1.83E+08	0.14	-3.82	3.99	0.07
E1	GY	2B	AX.89774692	1.02E+08	0.07	-4.61	3.07	0.06
E1	GY	2B	AX.89448318	1.02E+08	0.07	-4.61	3.07	0.06
E1	GY	2B	AX.89448318	1.02E+08	0.07	-4.61	3.07	0.06
E1	GY	2B	AX.86169218	1.84E+08	0.09	-4.30	3.47	0.06
E1	GY	2B	AX.89515639	5.95E+08	0.32	2.45	3.08	0.05
E1	GPS	2B	AX.89552494	9.74E+07	0.37	1.22	3.35	0.06
E1	GY	2B	AX.89612967	6.61E+08	0.06	-5.29	3.43	0.05
E1	GY	2B	AX.89612967	6.61E+08	0.06	-5.29	3.43	0.05
E1	GPS	2B	AX.89636806	9.84E+07	0.36	1.25	3.46	0.06
E1	GY	2B	AX.89331795	1.79E+08	0.20	-2.86	3.10	0.05
E1	GY	2B	AX.89524212	1.84E+08	0.14	-3.74	3.94	0.07
E1	GY	2B	AX.89524212	1.84E+08	0.14	-3.74	3.94	0.07
E1	GY	2B	AX.89760340	7.65E+08	0.12	-3.88	3.59	0.07
E1	GY	2B	AX.89598424	1.82E+08	0.14	-3.82	3.99	0.07
E1	GY	2B	AX.89598424	1.82E+08	0.14	-3.82	3.99	0.07
E1	GY	2B	AX.89724351	1.82E+08	0.13	-4.30	4.67	0.09
E1	GY	2B	AX.89736400	1.82E+08	0.13	-4.30	4.67	0.09
E1	GY	2B	AX.89696341	7.59E+08	0.06	-4.77	3.07	0.05
E1	GPS	2B	AX.89559923	9.58E+07	0.40	1.13	3.03	0.05
E1	GY	2B	AX.89760737	7.63E+08	0.24	-2.69	3.15	0.05
E1	GY	2B	AX.89774222	1.42E+08	0.26	-2.60	3.06	0.05
E1	GY	2B	AX.89387058	1.03E+08	0.07	-5.12	3.89	0.07
E1	GY	2B	AX.89387058	1.03E+08	0.07	-5.12	3.89	0.07
E1	GY	2B	AX.89672039	1.02E+08	0.07	-5.12	3.89	0.07
E1	GY	2B	AX.89774529	1.83E+08	0.14	-3.82	3.99	0.07
E1	GY	2B	AX.89591313	6.60E+08	0.07	-4.52	3.14	0.05
E1	GPS	2B	AX.89674753	1.12E+07	0.37	-1.19	3.22	0.05
E1	GPS	2B	AX.89426625	9.22E+07	0.39	1.20	3.33	0.06
E1	GY	2B	AX.89500416	4.81E+04	0.27	-2.52	3.01	0.04
E1	GY	2B	AX.89629939	6.62E+08	0.07	-5.01	3.55	0.06
E1	GY	2B	AX.89689244	8.00E+08	0.08	-4.70	3.71	0.06
E1	GY	2B	AX.89616056	5.94E+08	0.32	2.55	3.32	0.06
E1	GPS	2B	AX.89347241	9.49E+07	0.40	1.22	3.42	0.06
E1	GY	2B	AX.89451802	6.59E+08	0.06	-5.29	3.43	0.05

E1	GY	2B	AX.89585973	6.15E+08	0.33	2.49	3.22	0.06
E1	GY	2B	AX.89452013	1.82E+08	0.14	-4.28	4.87	0.09
E1	GY	2B	AX.89628294	1.81E+08	0.13	-3.82	3.68	0.07
E1	GY	2B	AX.89628294	1.81E+08	0.13	-3.82	3.68	0.07
E1	GY	2B	AX.89523629	1.83E+08	0.14	-3.82	3.99	0.07
E1	GY	2B	AX.89523666	1.02E+08	0.07	-4.61	3.07	0.06
E1	GY	2B	AX.89523666	1.02E+08	0.07	-4.61	3.07	0.06
E1	GY	2B	AX.89709245	1.29E+08	0.06	-6.36	5.07	0.08
E1	GY	2B	AX.89575510	1.34E+08	0.06	-6.36	5.07	0.08
E1	GY	2B	AX.89605804	1.82E+08	0.14	-4.23	4.88	0.09
E1	GY	2B	AX.89418425	7.46E+08	0.40	-2.32	3.09	0.05
E1	GY	2B	AX.89666887	1.30E+08	0.07	-6.79	6.05	0.10
E1	GPS	2B	AX.89527843	9.80E+07	0.37	1.22	3.35	0.06
E1	GPS	2B	AX.89486691	1.13E+07	0.27	-1.32	3.31	0.06
E1	GY	2B	AX.89496808	4.39E+04	0.27	-2.52	3.01	0.04
E1	GY	2B	AX.89632512	1.03E+08	0.07	-4.61	3.07	0.06
E1	GPS	2B	AX.89688762	9.80E+07	0.37	1.22	3.35	0.06
E1	GY	2B	AX.89776966	1.02E+08	0.07	-4.61	3.07	0.06
E1	GY	2B	AX.89655408	1.01E+08	0.07	-4.61	3.07	0.06
E1	GY	2B	AX.89764413	1.83E+08	0.14	-3.82	3.99	0.07
E1	GY	2B	AX.89561986	1.81E+08	0.11	-3.70	3.10	0.06
E1	GY	2B	AX.89559315	8.39E+06	0.27	-2.52	3.01	0.04
E1	GPS	2B	AX.89572059	1.12E+07	0.38	-1.23	3.43	0.06
E1	GY	2B	AX.89699414	1.79E+08	0.20	-2.86	3.10	0.05
E1	GY	2B	AX.89699414	1.79E+08	0.20	-2.86	3.10	0.05
E1	GY	2B	AX.89610028	1.44E+08	0.25	-2.62	3.07	0.05
E1	GY	2B	AX.89668164	1.84E+08	0.09	-4.30	3.47	0.06
E1	GY	2B	AX.89668164	1.84E+08	0.09	-4.30	3.47	0.06
E1	GPS	2B	AX.89407215	9.30E+07	0.39	1.29	3.72	0.06
E1	GY	2B	AX.89644113	1.84E+08	0.12	-4.27	4.24	0.08
E1	GY	2B	AX.89660518	1.00E+08	0.07	-4.61	3.07	0.06
E1	GY	2B	AX.89705968	1.26E+08	0.06	-6.36	5.07	0.08
E1	GY	2B	AX.89665143	1.78E+08	0.20	-3.07	3.45	0.06
E1	GY	2B	AX.89433133	3.30E+05	0.27	-2.52	3.01	0.04
E1	GY	2B	AX.89765905	1.02E+08	0.07	-4.61	3.07	0.06
E1	GY	2B	AX.89765905	1.02E+08	0.07	-4.61	3.07	0.06
E1	GY	2B	AX.89560733	5.95E+08	0.33	2.53	3.30	0.06
E1	GY	2B	AX.89693837	1.40E+08	0.25	-2.63	3.09	0.05
E1	GY	2B	AX.89641042	7.65E+08	0.12	-3.88	3.59	0.07
E1	GPS	2B	AX.86163702	9.37E+07	0.37	1.22	3.35	0.06
E1	GY	2B	AX.89719353	1.54E+08	0.23	-2.77	3.18	0.05
E1	GY	2B	AX.89719353	1.54E+08	0.23	-2.77	3.18	0.05
E1	GY	2B	AX.89338416	7.60E+08	0.06	-4.77	3.07	0.05
E1	GY	2B	AX.89316984	1.83E+08	0.14	-3.82	3.99	0.07
E1	GY	2B	AX.89560172	7.46E+08	0.35	-2.36	3.03	0.05
E1	SPM2	2B	AX.89343410	7.11E+08	0.20	23.05	3.83	0.07
E1	GY	2B	AX.89703355	1.81E+08	0.12	-4.25	4.19	0.08
E1	GY	2B	AX.89351218	6.59E+08	0.06	-5.81	4.32	0.07
E1	GY	2B	AX.89684472	1.81E+08	0.12	-3.87	3.68	0.07
E1	GY	2B	AX.89730024	1.82E+08	0.13	-4.62	5.44	0.10

E1	GY	2B	AX.89730024	1.82E+08	0.13	-4.62	5.44	0.10
E1	GY	2B	AX.89432832	6.31E+05	0.27	-2.52	3.01	0.04
E1	GY	2B	AX.89595331	1.84E+08	0.14	-3.55	3.60	0.06
E1	GY	2B	AX.89595331	1.84E+08	0.14	-3.55	3.60	0.06
E1	GY	2B	AX.89528928	3.65E+05	0.27	-2.52	3.01	0.04
E1	GY	2B	AX.89687896	1.02E+08	0.07	-4.61	3.07	0.06
E1	GY	2B	AX.89664777	1.04E+08	0.07	-4.61	3.07	0.06
E1	GY	2B	AX.89352375	7.95E+08	0.28	-3.06	4.24	0.07
E1	GY	2B	AX.89574794	1.02E+08	0.07	-4.61	3.07	0.06
E1	GPS	2B	AX.89614148	9.05E+07	0.39	1.23	3.42	0.06
E1	GY	2B	AX.89494298	7.60E+08	0.06	-4.77	3.07	0.05
E1	GY	2B	AX.89740553	1.03E+08	0.07	-5.12	3.89	0.07
E1	GY	2B	AX.89740553	1.03E+08	0.07	-5.12	3.89	0.07
E1	GY	2B	AX.89458240	1.82E+08	0.13	-4.30	4.67	0.09
E1	GY	2B	AX.89698843	1.83E+08	0.14	-4.13	4.69	0.09
E1	GY	2B	AX.89599756	1.44E+08	0.25	-2.59	3.01	0.05
E1	GY	2B	AX.89631602	7.93E+08	0.35	-2.98	4.54	0.09
E1	GY	2B	AX.89353728	1.82E+08	0.13	-4.30	4.67	0.09
E1	GY	2B	AX.89771820	7.93E+08	0.35	-2.95	4.44	0.09
E1	GY	2B	AX.89771820	7.93E+08	0.35	-2.95	4.44	0.09
E1	GY	2B	AX.89541694	1.40E+08	0.13	-3.46	3.20	0.06
E1	GY	2B	AX.89603427	1.87E+07	0.35	2.47	3.26	0.04
E1	GPS	2B	AX.89650540	9.18E+07	0.39	1.29	3.72	0.06
E1	GY	2B	AX.89636775	9.13E+06	0.26	-2.71	3.28	0.05
E1	GY	2B	AX.89636775	9.13E+06	0.26	-2.71	3.28	0.05
E1	GY	2B	AX.89558016	1.84E+08	0.14	-3.82	3.99	0.07
E1	GY	2B	AX.89563904	1.40E+08	0.24	-2.80	3.35	0.06
E1	GY	2B	AX.89593173	1.03E+08	0.07	-4.61	3.07	0.06
E1	GY	2B	AX.89593173	1.03E+08	0.07	-4.61	3.07	0.06
E1	GY	2B	AX.89748488	5.06E+05	0.27	-2.72	3.40	0.05
E1	GY	2B	AX.89531346	7.82E+08	0.34	-2.60	3.52	0.06
E1	GY	2B	AX.89717112	3.65E+05	0.27	-2.52	3.01	0.04
E1	GY	2B	AX.89506146	1.01E+08	0.07	-4.61	3.07	0.06
E1	GY	2B	AX.89590619	1.81E+08	0.12	-4.25	4.19	0.08
E1	GY	2B	AX.89625965	1.83E+08	0.14	-4.13	4.69	0.09
E1	GY	2B	AX.89681279	5.95E+08	0.33	2.53	3.30	0.06
E1	GY	2B	AX.89681279	5.95E+08	0.33	2.53	3.30	0.06
E1	GY	2B	AX.89469035	7.60E+08	0.06	-4.77	3.07	0.05
E1	GY	2B	AX.89713274	7.75E+08	0.08	-5.32	4.81	0.09
E1	GY	2B	AX.89676429	5.94E+08	0.32	2.42	3.02	0.05
E1	GY	2B	AX.89676429	5.94E+08	0.32	2.42	3.02	0.05
E1	GY	2B	AX.89540932	6.62E+08	0.06	-5.29	3.43	0.05
E1	GY	2B	AX.89718685	1.82E+08	0.13	-4.62	5.44	0.10
E1	GY	2B	AX.89677120	1.79E+08	0.20	-2.85	3.14	0.05
E1	GY	2B	AX.89677120	1.79E+08	0.20	-2.85	3.14	0.05
E1	GY	2B	AX.89652950	5.94E+08	0.32	2.42	3.02	0.05
E1	GY	2B	AX.89556519	1.83E+08	0.14	-3.82	3.99	0.07
E1	GY	2B	AX.89719069	7.57E+08	0.06	-4.77	3.07	0.05
E1	GY	2B	AX.89346022	1.70E+08	0.10	3.80	3.14	0.05
E1	GY	2B	AX.89451935	1.79E+07	0.24	2.74	3.24	0.04

E1	GY	2B	AX.89690045	1.82E+08	0.13	-4.62	5.44	0.10
E1	GPS	2B	AX.89546593	9.59E+07	0.40	1.24	3.51	0.06
E1	GY	2B	AX.89678145	7.95E+08	0.20	-2.87	3.12	0.05
E1	GY	2B	AX.89605899	1.81E+08	0.13	-4.55	5.29	0.10
E1	GY	2B	AX.89606237	1.47E+08	0.25	-2.63	3.09	0.05
E1	GY	2B	AX.89359231	4.67E+04	0.27	-2.52	3.01	0.04
E1	GY	2B	AX.89359231	4.67E+04	0.27	-2.52	3.01	0.04
E1	GY	2B	AX.89594592	1.80E+08	0.20	-3.07	3.45	0.06
E1	GY	2B	AX.89721075	1.03E+08	0.07	-5.12	3.89	0.07
E1	GY	2B	AX.89721075	1.03E+08	0.07	-5.12	3.89	0.07
E1	GY	2B	AX.89629911	5.25E+05	0.27	-2.52	3.01	0.04
E1	GY	2B	AX.89752453	7.64E+08	0.12	-3.88	3.59	0.07
E1	GY	2B	AX.89360195	7.59E+08	0.06	-4.77	3.07	0.05
E1	GY	2B	AX.89500920	1.79E+08	0.20	-2.86	3.10	0.05
E1	GY	2B	AX.89769704	7.58E+08	0.06	-4.77	3.07	0.05
E1	GY	2B	AX.89454067	1.82E+08	0.13	-4.62	5.44	0.10
E1	GY	2B	AX.89464930	1.42E+08	0.26	-2.60	3.06	0.05
E1	GY	2B	AX.89729189	1.83E+08	0.14	-4.13	4.69	0.09
E1	GY	2B	AX.89570875	1.43E+08	0.25	-2.76	3.31	0.06
E1	GY	2B	AX.89610028	1.44E+08	0.25	-2.62	3.07	0.05
E1	GY	2B	AX.89770264	1.83E+08	0.14	-4.13	4.69	0.09
E1	GY	2B	AX.89664208	6.61E+08	0.06	-5.29	3.43	0.05
E1	GY	2B	AX.89454401	7.84E+08	0.30	-3.42	5.36	0.09
E1	GY	2B	AX.89408614	7.65E+08	0.12	-3.88	3.59	0.07
E1	GY	2B	AX.89745765	1.02E+08	0.07	-4.61	3.07	0.06
E1	GY	2B	AX.89466177	7.95E+08	0.41	-2.34	3.12	0.05
E1	GY	2B	AX.89560360	3.87E+05	0.27	-2.52	3.01	0.04
E1	GY	2B	AX.89440441	1.31E+08	0.06	-5.75	4.24	0.07
E1	GY	2B	AX.89397326	1.79E+08	0.20	-3.07	3.45	0.06
E1	GY	2B	AX.89568935	5.94E+08	0.32	2.55	3.32	0.06
E1	GY	2B	AX.89538603	4.69E+04	0.27	-2.52	3.01	0.04
E1	GY	2B	AX.89621449	1.02E+08	0.07	-4.61	3.07	0.06
E1	GY	2B	AX.89731814	7.53E+08	0.29	-2.52	3.10	0.05
E1	GY	2B	AX.89731814	7.53E+08	0.29	-2.52	3.10	0.05
E1	GY	2B	AX.89641351	3.69E+05	0.27	-2.52	3.01	0.04
E1	GY	2B	AX.89562158	6.60E+08	0.06	-5.29	3.43	0.05
E1	GY	2B	AX.89399328	1.83E+08	0.14	-3.82	3.99	0.07
E1	GY	2B	AX.89476521	1.82E+08	0.12	-4.25	4.19	0.08
E1	GY	2B	AX.89476521	1.82E+08	0.12	-4.25	4.19	0.08
E1	GY	2B	AX.89755632	7.93E+08	0.26	-2.79	3.49	0.07
E1	GY	2B	AX.89562493	1.02E+08	0.07	-4.61	3.07	0.06
E1	GY	2B	AX.89562493	1.02E+08	0.07	-4.61	3.07	0.06
E1	GY	2B	AX.89631015	1.40E+08	0.24	-2.80	3.35	0.06
E1	GY	2B	AX.89457015	1.81E+08	0.11	-3.88	3.48	0.06
E1	GY	2B	AX.89493544	1.00E+08	0.07	-5.12	3.89	0.07
E1	GY	2B	AX.89618714	1.03E+08	0.07	-4.61	3.07	0.06
E1	GY	2B	AX.89695960	1.01E+08	0.07	-4.61	3.07	0.06
E1	GY	2B	AX.89695960	1.01E+08	0.07	-4.61	3.07	0.06
E1	GPM2	2B	AX.89409101	7.85E+08	0.14	-827.86	3.08	0.05
E1	GY	2B	AX.89469711	1.85E+08	0.09	-4.30	3.47	0.06

E1	GY	2B	AX.89505960	7.46E+08	0.35	-2.35	3.01	0.05
E1	GY	2B	AX.89330831	5.95E+08	0.33	2.53	3.30	0.06
E1	GY	2B	AX.89710344	7.57E+08	0.07	-5.04	3.58	0.06
E1	GPM2	2B	AX.89456255	4.60E+04	0.27	-659.01	3.14	0.05
E1	GPM2	2B	AX.89629911	5.25E+05	0.27	-659.01	3.14	0.05
E1	GPM2	2B	AX.89682796	1.84E+08	0.09	-990.87	3.02	0.05
E1	GY	2B	AX.89612574	1.85E+08	0.09	-4.30	3.47	0.06
E1	GPM2	2B	AX.89659140	7.93E+08	0.14	-889.67	3.40	0.06
E1	GY	2B	AX.89589720	1.02E+08	0.07	-5.12	3.89	0.07
E1	GY	2B	AX.89724272	6.62E+08	0.06	-5.81	4.32	0.07
E1	GY	2B	AX.89724272	6.62E+08	0.06	-5.81	4.32	0.07
E1	GY	2B	AX.89686938	4.55E+04	0.27	-2.52	3.01	0.04
E1	GY	2B	AX.89577473	7.58E+08	0.06	-4.77	3.07	0.05
E1	GPM2	2B	AX.89538158	9.00E+06	0.27	-659.01	3.14	0.05
E1	GY	2B	AX.89602946	1.02E+08	0.07	-4.61	3.07	0.06
E1	GY	2B	AX.89368225	7.85E+08	0.10	-3.98	3.28	0.05
E1	SPM2	2B	AX.89398225	1.78E+07	0.38	16.89	3.10	0.04
E1	GY	2B	AX.89543659	7.85E+08	0.24	-3.19	4.17	0.07
E1	GY	2B	AX.89697559	1.84E+08	0.09	-4.30	3.47	0.06
E1	GPM2	2B	AX.89617425	3.15E+05	0.28	-675.72	3.30	0.05
E1	GY	2B	AX.89571192	1.02E+08	0.07	-4.61	3.07	0.06
E1	GY	2B	AX.89571192	1.02E+08	0.07	-4.61	3.07	0.06
E1	GY	2B	AX.89770628	7.77E+08	0.49	-2.39	3.34	0.05
E1	GY	2B	AX.89500416	4.81E+04	0.27	-2.52	3.01	0.04
E1	SPM2	2B	AX.89477745	4.32E+08	0.05	39.99	3.49	0.06
E1	GY	2B	AX.89369630	1.46E+08	0.22	-2.73	3.03	0.05
E1	GY	2B	AX.89677005	1.40E+08	0.24	-2.80	3.35	0.06
E1	GY	2B	AX.89652950	5.94E+08	0.32	2.42	3.02	0.05
E1	GY	2B	AX.89511482	1.83E+08	0.13	-3.70	3.69	0.07
E1	GY	2B	AX.89616747	1.03E+08	0.07	-4.61	3.07	0.06
E1	GY	2B	AX.89507172	1.79E+08	0.20	-3.07	3.45	0.06
E1	GY	2B	AX.89657904	1.41E+08	0.26	-2.60	3.06	0.05
E1	GY	2B	AX.89617368	1.01E+08	0.07	-4.61	3.07	0.06
E1	GY	2B	AX.89617425	3.15E+05	0.28	-2.51	3.02	0.04
E1	GY	2B	AX.89499795	6.62E+08	0.06	-5.29	3.43	0.05
E1	GY	2B	AX.89776966	1.02E+08	0.07	-4.61	3.07	0.06
E1	GY	2B	AX.89547217	1.81E+08	0.11	-3.88	3.48	0.06
E1	GY	2B	AX.89547217	1.81E+08	0.11	-3.88	3.48	0.06
E1	GY	2B	AX.89595787	1.84E+08	0.14	-3.82	3.99	0.07
E1	GY	2B	AX.89595787	1.84E+08	0.14	-3.82	3.99	0.07
E1	GPS	2B	AX.89634274	9.25E+07	0.40	1.30	3.81	0.06
E1	GY	2B	AX.89713743	1.84E+08	0.14	-3.82	3.99	0.07
E1	GY	2B	AX.89373157	1.84E+08	0.14	-4.29	4.89	0.09
E1	GY	2B	AX.89718549	1.00E+08	0.07	-4.61	3.07	0.06
E1	GY	2B	AX.89749678	6.59E+08	0.06	-5.29	3.43	0.05
E1	GY	2B	AX.89660518	1.00E+08	0.07	-4.61	3.07	0.06
E1	GPM2	2B	AX.89674559	8.60E+06	0.35	-616.91	3.15	0.05
E1	GY	2B	AX.89656746	7.93E+08	0.31	-2.98	4.25	0.09
E1	GPS	2B	AX.89753034	1.12E+07	0.38	-1.19	3.22	0.05
E1	GY	2B	AX.89374293	1.02E+08	0.07	-5.12	3.89	0.07

E1	GY	2B	AX.89583382	1.81E+08	0.12	-4.25	4.19	0.08
E1	GY	2B	AX.89480023	1.81E+08	0.12	-4.25	4.19	0.08
E1	GPM2	2B	AX.89486691	1.13E+07	0.27	-724.00	3.65	0.06
E1	GPS	2B	AX.86163701	9.37E+07	0.37	1.22	3.35	0.06
E1	GY	2B	AX.89560602	7.60E+08	0.06	-5.75	3.97	0.06
E1	GY	2B	AX.89445890	1.79E+08	0.20	-2.86	3.10	0.05
E1	GY	2B	AX.89422340	7.89E+08	0.10	-3.75	3.07	0.05
E1	GY	2B	AX.89621714	1.83E+08	0.14	-4.13	4.69	0.09
E1	GY	2B	AX.89752463	1.14E+07	0.10	3.73	3.05	0.05
E1	GY	2B	AX.89688062	1.84E+08	0.08	-4.58	3.70	0.07
E1	GPS	2B	AX.89601276	9.76E+07	0.37	1.22	3.35	0.06
E1	GPS	2B	AX.89561906	9.84E+07	0.36	1.21	3.27	0.06
E1	GY	2B	AX.89555674	1.80E+08	0.20	-3.07	3.45	0.06
E1	GY	2B	AX.89605178	1.03E+08	0.07	-4.61	3.07	0.06
E1	GY	2B	AX.89510083	1.85E+08	0.09	-4.30	3.47	0.06
E1	GY	2B	AX.89510083	1.85E+08	0.09	-4.30	3.47	0.06
E1	GY	2B	AX.89377106	1.01E+08	0.07	-4.61	3.07	0.06
E1	GPS	2B	AX.89474800	9.58E+07	0.40	1.22	3.42	0.06
E1	GY	2B	AX.89774692	1.02E+08	0.07	-4.61	3.07	0.06
E1	GPM2	2B	AX.89748488	5.06E+05	0.27	-684.76	3.32	0.05
E1	GPS	2B	AX.86162637	9.49E+07	0.37	1.22	3.35	0.06
E1	GPM2	2B	AX.89463396	1.84E+08	0.12	-893.74	3.09	0.05
E1	GY	2B	AX.89661546	5.94E+08	0.32	2.42	3.02	0.05
E1	GPS	2B	AX.89699089	9.84E+07	0.37	1.16	3.05	0.05
E1	GY	2B	AX.89719172	3.65E+05	0.27	-2.52	3.01	0.04
E1	GY	2B	AX.89724351	1.82E+08	0.13	-4.30	4.67	0.09
E1	GPS	2B	AX.86169040	9.30E+07	0.38	1.36	4.04	0.07
E1	GPM2	2B	AX.89760312	7.72E+08	0.19	848.40	3.89	0.07
E1	GY	2B	AX.89774222	1.42E+08	0.26	-2.60	3.06	0.05
E1	GY	2B	AX.89672039	1.02E+08	0.07	-5.12	3.89	0.07
E1	GPM2	2B	AX.89599657	1.02E+07	0.24	-682.38	3.10	0.05
E1	GY	2B	AX.89774529	1.83E+08	0.14	-3.82	3.99	0.07
E1	GY	2B	AX.89591313	6.60E+08	0.07	-4.52	3.14	0.05
E1	GPM2	2B	AX.89559315	8.39E+06	0.27	-659.01	3.14	0.05
E1	GPS	2B	AX.89353462	1.12E+07	0.38	-1.19	3.22	0.05
E1	GY	2B	AX.89761980	7.94E+08	0.11	-4.21	3.87	0.07
E1	GY	2B	AX.89688716	7.93E+08	0.17	-3.41	3.81	0.07
E1	GY	2B	AX.89775110	1.29E+08	0.06	-6.36	5.07	0.08
E1	GY	2B	AX.89737929	1.01E+08	0.07	-4.61	3.07	0.06
E1	GY	2B	AX.89737929	1.01E+08	0.07	-4.61	3.07	0.06
E1	GY	2B	AX.89629939	6.62E+08	0.07	-5.01	3.55	0.06
E1	GPS	2B	AX.89736015	9.58E+07	0.40	1.15	3.09	0.05
E1	GY	2B	AX.89487985	1.83E+08	0.14	-3.82	3.99	0.07
E1	GY	2B	AX.89451802	6.59E+08	0.06	-5.29	3.43	0.05
E1	GPM2	2B	AX.89666887	1.30E+08	0.07	-1166.73	3.02	0.05
E1	GPM2	2B	AX.89755632	7.93E+08	0.26	-665.04	3.10	0.06
E1	GPM2	2B	AX.89496140	5.06E+05	0.27	-659.01	3.14	0.05
E1	GY	2B	AX.89523629	1.83E+08	0.14	-3.82	3.99	0.07
E1	GPS	2B	AX.89506182	1.03E+08	0.44	-1.18	3.29	0.06
E1	GPS	2B	AX.89697344	9.58E+07	0.40	1.13	3.03	0.05

E1	GPS	2B	AX.89454401	7.84E+08	0.30	-1.24	3.14	0.05
E1	GY	2B	AX.89418425	7.46E+08	0.40	-2.32	3.09	0.05
E1	GY	2B	AX.89558174	1.81E+08	0.12	-4.25	4.19	0.08
E1	GPM2	2B	AX.89664717	4.57E+04	0.27	-659.01	3.14	0.05
E1	GY	2B	AX.89627218	7.64E+08	0.12	-3.88	3.59	0.07
E1	GY	2B	AX.89632512	1.03E+08	0.07	-4.61	3.07	0.06
E1	GPS	2B	AX.89420920	9.23E+07	0.39	1.28	3.68	0.06
E1	GY	2B	AX.89764413	1.83E+08	0.14	-3.82	3.99	0.07
E1	GY	2B	AX.89408291	1.40E+08	0.13	-3.50	3.18	0.06
E1	GPM2	2B	AX.89465592	4.53E+05	0.27	-650.18	3.04	0.05
E1	GY	2B	AX.89526250	1.01E+08	0.07	-4.61	3.07	0.06
E1	GY	2B	AX.89668347	7.64E+08	0.12	-3.88	3.59	0.07
E1	GY	2B	AX.89705968	1.26E+08	0.06	-6.36	5.07	0.08
E1	GY	2B	AX.89665143	1.78E+08	0.20	-3.07	3.45	0.06
E1	GY	2B	AX.89596143	1.02E+08	0.07	-4.61	3.07	0.06
E1	GY	2B	AX.89564960	1.80E+08	0.20	-3.07	3.45	0.06
E1	GPM2	2B	AX.89770628	7.77E+08	0.49	-606.90	3.31	0.05
E1	GPM2	2B	AX.89717112	3.65E+05	0.27	-659.01	3.14	0.05
E1	GY	2B	AX.89676847	1.54E+08	0.23	-2.77	3.18	0.05
E1	GY	2B	AX.89676847	1.54E+08	0.23	-2.77	3.18	0.05
E1	GY	2B	AX.89645229	7.93E+08	0.35	-2.98	4.54	0.09
E1	GY	2B	AX.89645229	7.93E+08	0.35	-2.98	4.54	0.09
E1	GY	2B	AX.89432832	6.31E+05	0.27	-2.52	3.01	0.04
E1	GY	2B	AX.89457015	1.81E+08	0.11	-3.88	3.48	0.06
E1	GY	2B	AX.89664777	1.04E+08	0.07	-4.61	3.07	0.06
E1	GY	2B	AX.89352375	7.95E+08	0.28	-3.06	4.24	0.07
E1	GY	2B	AX.89529518	5.94E+08	0.32	2.55	3.32	0.06
E1	GPM2	2B	AX.89390226	1.02E+07	0.24	-682.38	3.10	0.05
E1	GY	2B	AX.89650120	1.78E+08	0.20	-3.07	3.45	0.06
E1	GPM2	2B	AX.89686938	4.55E+04	0.27	-659.01	3.14	0.05
E1	GY	2B	AX.89552327	1.78E+08	0.20	-3.07	3.45	0.06
E1	GY	2B	AX.89599756	1.44E+08	0.25	-2.59	3.01	0.05
E1	GY	2B	AX.89750857	1.81E+08	0.11	-3.88	3.48	0.06
E1	GY	2B	AX.89703625	6.62E+08	0.06	-5.29	3.43	0.05
E1	GY	2B	AX.89678102	1.03E+08	0.07	-4.61	3.07	0.06
E1	GY	2B	AX.89678102	1.03E+08	0.07	-4.61	3.07	0.06
E1	GY	2B	AX.89541694	1.40E+08	0.13	-3.46	3.20	0.06
E1	GPM2	2B	AX.89727516	1.45E+07	0.39	613.29	3.23	0.06
E1	GY	2B	AX.89538197	1.80E+08	0.20	-2.85	3.04	0.05
E1	GY	2B	AX.89538197	1.80E+08	0.20	-2.85	3.04	0.05
E1	GPM2	2B	AX.89556126	7.90E+08	0.27	-658.99	3.14	0.06
E1	GY	2B	AX.89563904	1.40E+08	0.24	-2.80	3.35	0.06
E1	GPM2	2B	AX.89432832	6.31E+05	0.27	-659.01	3.14	0.05
E1	GY	2B	AX.89748488	5.06E+05	0.27	-2.72	3.40	0.05
E1	GY	2B	AX.89717112	3.65E+05	0.27	-2.52	3.01	0.04
E1	GPM2	2B	AX.89404329	9.74E+06	0.27	-659.01	3.14	0.05
E1	GPM2	2B	AX.89743242	1.90E+07	0.38	-825.47	5.36	0.09
E1	GY	2B	AX.89673041	6.59E+08	0.06	-5.29	3.43	0.05
E1	GY	2B	AX.89673041	6.59E+08	0.06	-5.29	3.43	0.05
E1	GY	2B	AX.89625965	1.83E+08	0.14	-4.13	4.69	0.09

E1	GY	2B	AX.89469035	7.60E+08	0.06	-4.77	3.07	0.05
E1	GY	2B	AX.89749024	1.82E+08	0.13	-4.30	4.67	0.09
E1	GY	2B	AX.89540932	6.62E+08	0.06	-5.29	3.43	0.05
E1	GY	2B	AX.89323675	6.59E+08	0.06	-5.29	3.43	0.05
E1	GY	2B	AX.89556519	1.83E+08	0.14	-3.82	3.99	0.07
E1	GY	2B	AX.89564994	1.81E+08	0.11	-3.88	3.48	0.06
E1	GY	2B	AX.89605899	1.81E+08	0.13	-4.55	5.29	0.10
E1	GY	2B	AX.89359145	9.58E+07	0.06	-5.39	3.78	0.06
E1	GY	2B	AX.89359145	9.58E+07	0.06	-5.39	3.78	0.06
E1	GY	2B	AX.89629911	5.25E+05	0.27	-2.52	3.01	0.04
E1	GY	2B	AX.89752453	7.64E+08	0.12	-3.88	3.59	0.07
E1	GY	2B	AX.89536238	7.90E+08	0.13	-3.99	3.98	0.07
E1	GY	2B	AX.89729189	1.83E+08	0.14	-4.13	4.69	0.09
E1	GY	2B	AX.89770264	1.83E+08	0.14	-4.13	4.69	0.09
E1	GY	2B	AX.89572391	1.82E+08	0.13	-4.30	4.67	0.09
E1	GY	2B	AX.89568935	5.94E+08	0.32	2.55	3.32	0.06
E1	GY	2B	AX.89538603	4.69E+04	0.27	-2.52	3.01	0.04
E1	GY	2B	AX.89745248	1.82E+08	0.12	-4.25	4.19	0.08
E1	GY	2B	AX.89469345	2.07E+07	0.08	4.32	3.21	0.05
E1	GY	2B	AX.89647344	7.59E+08	0.06	-4.77	3.07	0.05
E1	GPM2	2B	AX.89656746	7.93E+08	0.31	-625.94	3.04	0.06
E1	GY	2B	AX.89575490	1.81E+08	0.12	-4.25	4.19	0.08
E1	GPM2	2B	AX.89496808	4.39E+04	0.27	-659.01	3.14	0.05
E1	GPM2	2B	AX.89528928	3.65E+05	0.27	-659.01	3.14	0.05
E1	GY	2B	AX.89686938	4.55E+04	0.27	-2.52	3.01	0.04
E1	GY	2B	AX.89403445	7.52E+08	0.27	-2.53	3.00	0.05
E1	GY	2B	AX.89368860	6.59E+08	0.06	-5.29	3.43	0.05
E1	GY	2B	AX.89697559	1.84E+08	0.09	-4.30	3.47	0.06
E1	GY	2B	AX.89677005	1.40E+08	0.24	-2.80	3.35	0.06
E1	GY	2B	AX.89369934	7.51E+08	0.33	-2.41	3.06	0.05
E1	GPM2	2B	AX.89761980	7.94E+08	0.11	-957.08	3.17	0.06
E1	GPM2	2B	AX.89433133	3.30E+05	0.27	-659.01	3.14	0.05
E1	GY	2B	AX.89682491	1.81E+08	0.12	-3.46	3.03	0.05
E1	GY	2B	AX.89442198	1.02E+08	0.07	-4.61	3.07	0.06
E1	GY	2B	AX.89728726	6.62E+08	0.06	-5.29	3.43	0.05
E1	GY	2B	AX.89765862	6.60E+08	0.06	-5.29	3.43	0.05
E1	GPS	2B	AX.89634319	1.12E+07	0.37	-1.15	3.01	0.05
E1	GPS	2B	AX.89539151	9.84E+07	0.37	1.16	3.05	0.05
E1	GPM2	2B	AX.89538603	4.69E+04	0.27	-659.01	3.14	0.05
E1	GPM2	2B	AX.89500416	4.81E+04	0.27	-659.01	3.14	0.05
E1	GY	2B	AX.89743242	1.90E+07	0.38	-2.33	3.03	0.04
E1	GPM2	2B	AX.89560360	3.87E+05	0.27	-659.01	3.14	0.05
E1	GY	2B	AX.89583382	1.81E+08	0.12	-4.25	4.19	0.08
E1	GY	2B	AX.89731365	1.41E+08	0.26	-2.60	3.06	0.05
E1	GPM2	2B	AX.89536238	7.90E+08	0.13	-988.78	3.78	0.07
E1	GPM2	2B	AX.89452576	3.18E+05	0.27	-659.01	3.14	0.05
E1	GPS	2B	AX.89409381	9.59E+07	0.40	1.13	3.03	0.05
E1	GPS	2B	AX.89739904	9.72E+07	0.37	1.22	3.35	0.06
E1	GPM2	2B	AX.89411485	4.88E+04	0.27	-659.01	3.14	0.05
E1	GPS	2B	AX.89679990	9.59E+07	0.40	1.22	3.42	0.06

E1	GPM2	2B	AX.89733954	1.02E+07	0.23	-690.62	3.09	0.05
E1	GPM2	2B	AX.89658674	6.27E+05	0.27	-659.01	3.14	0.05
E1	TKW	2B	AX.89386261	5.18E+08	0.09	-1.54	3.42	0.06
E1	TKW	2B	AX.89388627	2.45E+07	0.45	0.95	3.77	0.06
E1	GPS	2B	AX.89493965	9.59E+07	0.40	1.13	3.03	0.05
E1	GPS	2B	AX.89547662	4.75E+06	0.45	-1.18	3.31	0.06
E1	TKW	2B	AX.89401751	7.44E+08	0.37	0.90	3.24	0.06
E1	GPM2	2B	AX.89401624	4.53E+05	0.27	-659.01	3.14	0.05
E1	GPM2	2B	AX.89359231	4.67E+04	0.27	-659.01	3.14	0.05
E1	GPM2	2B	AX.89641351	3.69E+05	0.27	-659.01	3.14	0.05
E1	GPM2	2B	AX.89338392	7.90E+08	0.12	-1036.92	3.99	0.07
E1	TKW	2B	AX.89677248	7.08E+08	0.37	0.93	3.47	0.06
E1	GY	2B	AX.89627218	7.64E+08	0.12	-3.88	3.59	0.07
E1	GPM2	2B	AX.89386320	4.52E+04	0.27	-659.01	3.14	0.05
E1	TKW	2B	AX.89688417	7.43E+08	0.36	0.88	3.09	0.05
E1	TKW	2B	AX.89450083	6.91E+08	0.28	0.99	3.42	0.06
E1	TKW	2B	AX.89452566	7.68E+08	0.40	-0.85	3.07	0.05
E1	GY	2B	AX.89726785	6.62E+08	0.06	-5.29	3.43	0.05
E1	TKW	2B	AX.89661678	1.90E+07	0.36	0.87	3.08	0.05
E1	TKW	2B	AX.89768058	1.90E+07	0.35	0.89	3.17	0.05
E1	GY	2B	AX.89579357	1.83E+08	0.14	-3.82	3.99	0.07
E1	TKW	2B	AX.89534242	7.42E+08	0.18	-1.12	3.21	0.05
E1	TKW	2B	AX.89522387	7.18E+08	0.28	0.96	3.20	0.06
E1	TKW	2B	AX.89639712	7.65E+08	0.24	-1.06	3.52	0.06
E1	GY	2B	AX.89684472	1.81E+08	0.12	-3.87	3.68	0.07
E1	TKW	2B	AX.89771525	6.94E+08	0.37	0.89	3.18	0.05
E1	TKW	2B	AX.89400493	7.19E+08	0.29	0.97	3.35	0.06
E1	TKW	2B	AX.89462410	7.38E+08	0.37	0.86	3.03	0.05
E1	GY	2B	AX.89750857	1.81E+08	0.11	-3.88	3.48	0.06
E1	TKW	2B	AX.89556952	2.42E+07	0.46	0.97	3.88	0.07
E1	TKW	2B	AX.89661546	5.94E+08	0.32	0.96	3.44	0.06
E1	GPM2	2B	AX.89447263	5.15E+05	0.27	-659.01	3.14	0.05
E1	TKW	2B	AX.89573057	7.44E+08	0.36	0.87	3.07	0.05
E1	TKW	2B	AX.89383653	6.02E+08	0.38	1.00	3.93	0.07
E1	GPM2	2B	AX.89719172	3.65E+05	0.27	-659.01	3.14	0.05
E1	GY	2B	AX.89558016	1.84E+08	0.14	-3.82	3.99	0.07
E1	TKW	2B	AX.89652950	5.94E+08	0.32	0.96	3.44	0.06
E1	TKW	2B	AX.89593167	7.44E+08	0.36	0.90	3.21	0.06
E1	TKW	2B	AX.89491975	7.15E+08	0.07	-1.80	3.38	0.06
E1	TKW	2B	AX.89429731	7.44E+08	0.37	0.90	3.24	0.06
E1	TKW	2B	AX.89633106	7.89E+08	0.10	-1.41	3.05	0.05
E1	TKW	2B	AX.89531301	7.38E+08	0.36	0.90	3.21	0.06
E1	TKW	2B	AX.89675483	2.82E+08	0.21	-1.07	3.33	0.06
E1	TKW	2B	AX.89533376	7.65E+08	0.37	-0.86	3.01	0.05
E1	GY	2B	AX.89718685	1.82E+08	0.13	-4.62	5.44	0.10
E1	TKW	2B	AX.89602749	7.64E+08	0.24	-1.06	3.52	0.06
E1	GY	2B	AX.89588768	6.60E+08	0.06	-5.29	3.43	0.05
E1	TKW	2B	AX.89616056	5.94E+08	0.32	0.96	3.44	0.06
E1	TKW	2B	AX.89732849	7.16E+08	0.28	0.95	3.20	0.06
E1	TKW	2B	AX.89751944	7.57E+08	0.35	0.92	3.34	0.06

E1	TKW	2B	AX.89679046	7.44E+08	0.37	0.90	3.24	0.06
E1	TKW	2B	AX.89418966	7.98E+08	0.05	-2.00	3.34	0.06
E1	TKW	2B	AX.89696810	7.09E+08	0.40	1.01	4.10	0.07
E1	TKW	2B	AX.89435065	6.74E+08	0.26	0.95	3.03	0.05
E1	TKW	2B	AX.89761668	5.94E+08	0.32	0.96	3.44	0.06
E1	TKW	2B	AX.89615317	7.65E+08	0.37	-0.86	3.01	0.05
E1	TKW	2B	AX.89624401	7.89E+08	0.07	-2.11	4.45	0.08
E1	TKW	2B	AX.89553116	7.37E+08	0.36	0.90	3.21	0.06
E1	TKW	2B	AX.89406449	2.82E+08	0.22	-1.11	3.61	0.07
E1	TKW	2B	AX.89328414	7.64E+08	0.24	-1.06	3.52	0.06
E1	TKW	2B	AX.89554098	7.10E+08	0.40	0.97	3.78	0.07
E1	TKW	2B	AX.89532521	7.65E+08	0.24	-1.06	3.52	0.06
E1	TKW	2B	AX.89391731	6.01E+08	0.35	0.88	3.12	0.05
E1	TKW	2B	AX.89676427	6.94E+08	0.27	0.93	3.03	0.05
E1	TKW	2B	AX.89345738	7.45E+08	0.50	0.89	3.37	0.06
E1	GY	2B	AX.89537259	1.01E+08	0.07	-4.61	3.07	0.06
E1	TKW	2B	AX.89417163	7.68E+08	0.40	-0.85	3.07	0.05
E1	TKW	2B	AX.89613561	7.46E+08	0.49	0.91	3.53	0.06
E1	TKW	2B	AX.89418162	7.19E+08	0.29	0.97	3.35	0.06
E1	TKW	2B	AX.89590942	7.43E+08	0.36	0.90	3.21	0.06
E1	TKW	2B	AX.89340885	5.94E+08	0.32	0.96	3.44	0.06
E1	TKW	2B	AX.89679759	7.98E+08	0.06	-1.80	3.20	0.05
E1	TKW	2B	AX.89753037	7.10E+08	0.40	0.88	3.20	0.06
E1	TKW	2B	AX.89762444	7.03E+08	0.38	0.94	3.51	0.06
E1	TKW	2B	AX.89515639	5.95E+08	0.32	1.01	3.76	0.07
E1	TKW	2B	AX.89723481	5.94E+08	0.33	0.96	3.44	0.06
E1	TKW	2B	AX.89657622	7.19E+08	0.28	0.96	3.20	0.06
E1	GY	2B	AX.89618714	1.03E+08	0.07	-4.61	3.07	0.06
E1	TKW	2B	AX.89753690	7.44E+08	0.37	0.88	3.12	0.05
E1	TKW	2B	AX.89329425	6.73E+08	0.33	0.99	3.68	0.07
E1	TKW	2B	AX.89563683	1.89E+07	0.36	0.92	3.37	0.06
E1	TKW	2B	AX.89619779	7.15E+08	0.07	-1.80	3.38	0.06
E1	TKW	2B	AX.89345607	7.65E+08	0.24	-1.06	3.52	0.06
E1	TKW	2B	AX.89356319	7.46E+08	0.49	0.91	3.53	0.06
E1	TKW	2B	AX.89338402	7.19E+08	0.30	0.91	3.05	0.05
E1	TKW	2B	AX.89556917	7.65E+08	0.24	-1.06	3.52	0.06
E1	TKW	2B	AX.89631447	7.18E+08	0.29	0.97	3.35	0.06
E1	TKW	2B	AX.89403372	7.38E+08	0.36	0.90	3.21	0.06
E1	TKW	2B	AX.89335723	7.18E+08	0.29	0.97	3.35	0.06
E1	TKW	2B	AX.89745821	7.44E+08	0.37	0.90	3.24	0.06
E1	TKW	2B	AX.89608836	7.89E+08	0.07	-2.11	4.45	0.08
E1	GY	2B	AX.89696341	7.59E+08	0.06	-4.77	3.07	0.05
E1	TKW	2B	AX.89515768	7.19E+08	0.28	0.95	3.20	0.06
E1	TKW	2B	AX.89475785	2.53E+07	0.41	0.86	3.11	0.05
E1	TKW	2B	AX.89414376	2.42E+07	0.45	0.97	3.93	0.07
E1	TKW	2B	AX.89338197	7.65E+08	0.24	-1.06	3.52	0.06
E1	TKW	2B	AX.89594259	6.94E+08	0.28	0.94	3.07	0.05
E1	TKW	2B	AX.89634429	7.41E+08	0.39	0.88	3.17	0.05
E1	TKW	2B	AX.89322589	7.14E+08	0.28	0.94	3.11	0.05
E1	TKW	2B	AX.89391193	7.34E+08	0.36	0.90	3.21	0.06

E1	GY	2B	AX.89497642	1.82E+08	0.12	-4.25	4.19	0.08
E1	TKW	2B	AX.89385040	7.44E+08	0.37	0.90	3.24	0.06
E1	TKW	2B	AX.89669637	7.64E+08	0.24	-1.06	3.52	0.06
E1	TKW	2B	AX.89376641	1.89E+07	0.36	0.89	3.18	0.06
E1	TKW	2B	AX.89772344	7.68E+08	0.40	-0.85	3.07	0.05
E1	TKW	2B	AX.89340151	7.43E+08	0.38	0.86	3.06	0.05
E1	TKW	2B	AX.89769642	6.74E+08	0.33	0.99	3.68	0.07
E1	TKW	2B	AX.89359515	5.94E+08	0.33	0.91	3.15	0.05
E1	TKW	2B	AX.89380713	7.38E+08	0.36	0.90	3.21	0.06
E1	TKW	2B	AX.89707286	6.91E+08	0.27	0.96	3.14	0.05
E1	TKW	2B	AX.89682070	7.06E+08	0.41	0.87	3.18	0.05
E1	TKW	2B	AX.89724284	7.08E+08	0.36	0.92	3.34	0.06
E1	TKW	2B	AX.89726836	2.82E+08	0.21	-1.09	3.40	0.06
E1	GPM2	2B	AX.89490236	4.74E+04	0.27	-659.01	3.14	0.05
E1	TKW	2B	AX.89362541	7.97E+08	0.07	-2.01	4.34	0.08
E1	TKW	2B	AX.89637526	1.90E+07	0.35	0.89	3.17	0.05
E1	TKW	2B	AX.89749179	2.42E+07	0.46	0.97	3.88	0.07
E1	TKW	2B	AX.89735631	6.02E+08	0.38	1.00	3.93	0.07
E1	TKW	2B	AX.89548333	7.09E+08	0.45	0.92	3.55	0.06
E1	TKW	2B	AX.89463337	7.06E+08	0.41	0.87	3.18	0.05
E1	TKW	2B	AX.89686435	7.65E+08	0.23	-1.07	3.46	0.06
E1	TKW	2B	AX.89549868	7.10E+08	0.40	0.88	3.22	0.06
E1	GY	2B	AX.89708086	1.84E+08	0.14	-3.82	3.99	0.07
E1	TKW	2B	AX.89624820	7.64E+08	0.24	-1.08	3.59	0.06
E1	TKW	2B	AX.89727986	7.97E+08	0.07	-2.01	4.34	0.08
E1	TKW	2B	AX.89430323	7.68E+08	0.40	-0.85	3.07	0.05
E1	TKW	2B	AX.89652699	7.65E+08	0.24	-1.06	3.52	0.06
E1	TKW	2B	AX.89757336	7.42E+08	0.21	-1.04	3.18	0.05
E1	TKW	2B	AX.89502034	6.96E+08	0.28	0.93	3.02	0.05
E1	TKW	2B	AX.89533402	5.21E+08	0.09	-1.67	3.93	0.07
E1	TKW	2B	AX.89550733	7.47E+08	0.07	-1.90	3.92	0.07
E1	GPM2	2B	AX.89454401	7.84E+08	0.30	-757.68	4.16	0.07
E1	GPM2	2B	AX.89504442	7.73E+08	0.20	762.71	3.39	0.06
E1	TKW	2B	AX.89395504	6.34E+08	0.28	0.97	3.25	0.06
E1	TKW	2B	AX.89540537	2.42E+07	0.46	0.97	3.88	0.07
E1	TKW	2B	AX.89316432	7.37E+08	0.36	0.90	3.21	0.06
E1	TKW	2B	AX.89500925	7.80E+08	0.09	-1.58	3.59	0.06
E1	TKW	2B	AX.89560674	6.73E+08	0.32	0.94	3.32	0.06
E1	TKW	2B	AX.89403850	7.11E+08	0.40	0.97	3.78	0.07
E1	TKW	2B	AX.89644872	7.38E+08	0.37	0.86	3.01	0.05
E1	TKW	2B	AX.89450884	5.95E+08	0.31	0.96	3.38	0.06
E1	TKW	2B	AX.89624118	2.45E+07	0.45	0.94	3.71	0.06
E1	TKW	2B	AX.89550482	7.98E+08	0.06	-1.95	3.67	0.06
E1	TKW	2B	AX.89568935	5.94E+08	0.32	0.96	3.44	0.06
E1	TKW	2B	AX.89398799	6.08E+08	0.36	1.06	4.28	0.07
E1	TKW	2B	AX.89458271	5.94E+08	0.32	0.96	3.44	0.06
E1	TKW	2B	AX.89489593	7.65E+08	0.36	-0.90	3.23	0.05
E1	GY	2B	AX.89579357	1.83E+08	0.14	-3.82	3.99	0.07
E1	GY	2B	AX.89386320	4.52E+04	0.27	-2.52	3.01	0.04
E1	TKW	2B	AX.89507634	7.89E+08	0.07	-2.11	4.45	0.08

E1	TKW	2B	AX.89682088	6.94E+08	0.37	0.89	3.18	0.05
E1	TKW	2B	AX.89322798	7.65E+08	0.24	-1.06	3.52	0.06
E1	TKW	2B	AX.89626720	2.49E+07	0.33	0.92	3.26	0.05
E1	TKW	2B	AX.89710581	7.64E+08	0.24	-1.06	3.52	0.06
E1	TKW	2B	AX.89351738	6.74E+08	0.33	0.99	3.68	0.07
E1	TKW	2B	AX.89503580	7.43E+08	0.36	0.90	3.21	0.06
E1	TKW	2B	AX.89686633	5.94E+08	0.32	0.96	3.44	0.06
E1	TKW	2B	AX.89745453	7.65E+08	0.36	-0.90	3.23	0.05
E1	TKW	2B	AX.89427256	7.06E+08	0.41	0.87	3.18	0.05
E1	TKW	2B	AX.89585973	6.15E+08	0.33	0.97	3.52	0.06
E1	TKW	2B	AX.89399434	6.97E+08	0.42	0.89	3.32	0.06
E1	TKW	2B	AX.89361908	1.90E+07	0.36	0.87	3.08	0.05
E1	TKW	2B	AX.89417089	7.37E+08	0.37	0.88	3.13	0.05
E1	GY	2B	AX.89393166	7.57E+08	0.07	-5.13	3.90	0.06
E1	TKW	2B	AX.89728064	9.03E+07	0.09	1.45	3.10	0.05
E1	TKW	2B	AX.89696530	3.83E+07	0.15	1.31	3.79	0.05
E1	TKW	2B	AX.89333824	6.94E+08	0.37	0.92	3.38	0.06
E1	TKW	2B	AX.89501127	3.70E+07	0.13	1.28	3.11	0.05
E1	TKW	2B	AX.89498540	2.82E+08	0.22	-1.11	3.61	0.07
E1	TKW	2B	AX.89640514	2.45E+07	0.45	0.94	3.71	0.06
E1	TKW	2B	AX.89673994	1.89E+07	0.36	0.89	3.18	0.06
E1	TKW	2B	AX.89676429	5.94E+08	0.32	0.96	3.44	0.06
E1	TKW	2B	AX.89334693	7.64E+08	0.24	-1.06	3.52	0.06
E1	TKW	2B	AX.89748080	7.19E+08	0.28	0.95	3.20	0.06
E1	TKW	2B	AX.89586311	7.65E+08	0.24	-1.06	3.52	0.06
E1	TKW	2B	AX.89727367	1.89E+07	0.36	0.89	3.18	0.06
E1	TKW	2B	AX.89566096	7.88E+08	0.06	-1.87	3.41	0.06
E1	TKW	2B	AX.89768931	7.65E+08	0.24	-1.06	3.52	0.06
E1	TKW	2B	AX.89333155	7.38E+08	0.34	0.90	3.15	0.05
E1	TKW	2B	AX.89529518	5.94E+08	0.32	0.96	3.44	0.06
E1	TKW	2B	AX.89476254	7.16E+08	0.28	0.96	3.20	0.06
E1	TKW	2B	AX.89597215	1.89E+07	0.37	0.91	3.31	0.06
E1	TKW	2B	AX.89362204	7.65E+08	0.24	-1.06	3.52	0.06
E1	TKW	2B	AX.89588122	7.65E+08	0.36	-0.90	3.23	0.05
E1	TKW	2B	AX.89608778	2.43E+07	0.45	0.97	3.93	0.07
E1	TKW	2B	AX.89720857	2.45E+07	0.43	0.84	3.05	0.05
E1	TKW	2B	AX.89317686	7.18E+08	0.28	0.96	3.20	0.06
E1	TKW	2B	AX.89430844	5.94E+08	0.33	0.96	3.44	0.06
E1	TKW	2B	AX.89315115	2.42E+07	0.46	0.97	3.88	0.07
E1	TKW	2B	AX.89428712	2.43E+07	0.45	1.00	4.15	0.07
E2	GY	2B	AX.89539161	1.82E+08	0.13	-4.02	5.87	0.09
E2	SPM2	2B	AX.89521803	1.21E+07	0.12	18.12	3.12	0.05
E2	GY	2B	AX.89573578	5.93E+08	0.06	-4.54	3.80	0.07
E2	GY	2B	AX.89573578	5.93E+08	0.06	-4.54	3.80	0.07
E2	SPM2	2B	AX.89756971	1.69E+07	0.49	-13.08	3.71	0.06
E2	GY	2B	AX.89682796	1.84E+08	0.09	-3.41	3.22	0.05
E2	SPM2	2B	AX.89754101	7.48E+08	0.05	28.05	3.37	0.06
E2	GY	2B	AX.89351791	1.83E+08	0.14	-3.11	3.81	0.06
E2	SPM2	2B	AX.89658601	1.89E+07	0.20	15.02	3.21	0.05
E2	GY	2B	AX.89634458	1.79E+08	0.19	-2.80	4.02	0.06

E2	GY	2B	AX.89696744	1.79E+08	0.19	-2.80	4.02	0.06
E2	GY	2B	AX.89352110	1.29E+08	0.25	-2.20	3.12	0.05
E2	GY	2B	AX.89352110	1.29E+08	0.25	-2.20	3.12	0.05
E2	SPM2	2B	AX.89519465	7.86E+08	0.05	28.90	3.55	0.06
E2	GY	2B	AX.89400251	7.59E+08	0.06	-3.99	3.11	0.04
E2	SPM2	2B	AX.89540507	7.48E+08	0.05	28.05	3.37	0.06
E2	GY	2B	AX.89745228	1.22E+08	0.25	-2.18	3.04	0.05
E2	GY	2B	AX.89570875	1.43E+08	0.25	-2.18	3.05	0.05
E2	GY	2B	AX.89570875	1.43E+08	0.25	-2.18	3.05	0.05
E2	SPM2	2B	AX.86169748	1.82E+07	0.48	-12.26	3.32	0.05
E2	SPM2	2B	AX.89483069	1.82E+07	0.48	-12.60	3.48	0.05
E2	GY	2B	AX.89469521	1.83E+08	0.14	-3.55	4.92	0.08
E2	SPM2	2B	AX.89566909	1.76E+07	0.38	12.30	3.18	0.05
E2	GY	2B	AX.89575499	6.63E+08	0.06	-4.94	4.16	0.06
E2	GY	2B	AX.89575499	6.63E+08	0.06	-4.94	4.16	0.06
E2	GY	2B	AX.89330672	6.60E+08	0.07	-4.01	3.30	0.04
E2	GY	2B	AX.89436572	1.82E+08	0.13	-3.57	4.64	0.07
E2	SPM2	2B	AX.89445580	1.89E+07	0.20	15.02	3.21	0.05
E2	GY	2B	AX.89575757	1.80E+08	0.19	-2.80	4.02	0.06
E2	GY	2B	AX.89331026	1.53E+08	0.06	-4.16	3.10	0.05
E2	SPM2	2B	AX.89454978	6.56E+08	0.10	-20.40	3.25	0.05
E2	GY	2B	AX.89470748	6.62E+08	0.06	-5.79	5.90	0.09
E2	GY	2B	AX.89541454	1.79E+08	0.20	-2.79	4.07	0.07
E2	GY	2B	AX.89541454	1.79E+08	0.20	-2.79	4.07	0.07
E2	SPM2	2B	AX.89762454	1.29E+07	0.14	17.53	3.27	0.06
E2	GY	2B	AX.89564994	1.81E+08	0.11	-3.62	4.24	0.07
E2	GY	2B	AX.89541694	1.40E+08	0.13	-3.15	3.65	0.06
E2	SPM2	2B	AX.89680341	7.47E+08	0.05	28.05	3.37	0.06
E2	GY	2B	AX.89402018	1.76E+08	0.18	-3.40	5.31	0.09
E2	GY	2B	AX.89507024	6.62E+08	0.06	-4.41	3.40	0.04
E2	GY	2B	AX.89507172	1.79E+08	0.20	-3.16	5.03	0.08
E2	SPM2	2B	AX.89678258	1.89E+07	0.20	15.02	3.21	0.05
E2	GY	2B	AX.89612967	6.61E+08	0.06	-5.03	4.29	0.06
E2	GY	2B	AX.89437310	5.64E+08	0.11	-3.20	3.30	0.04
E2	SPM2	2B	AX.89608908	1.82E+07	0.48	-12.60	3.48	0.05
E2	GY	2B	AX.89331795	1.79E+08	0.20	-2.93	4.48	0.07
E2	SPM2	2B	AX.89766607	7.48E+08	0.05	28.05	3.37	0.06
E2	SPM2	2B	AX.89494773	1.89E+07	0.20	15.02	3.21	0.05
E2	GY	2B	AX.89332143	1.82E+08	0.13	-4.02	5.87	0.09
E2	GY	2B	AX.89332143	1.82E+08	0.13	-4.02	5.87	0.09
E2	GY	2B	AX.89650120	1.78E+08	0.20	-3.16	5.03	0.08
E2	GY	2B	AX.89650120	1.78E+08	0.20	-3.16	5.03	0.08
E2	GY	2B	AX.89542438	6.64E+08	0.05	-4.64	3.48	0.05
E2	GY	2B	AX.89472568	6.64E+08	0.05	-4.64	3.48	0.05
E2	SPM2	2B	AX.89471633	1.76E+07	0.38	12.30	3.18	0.05
E2	GY	2B	AX.89590527	1.33E+08	0.07	-4.06	3.45	0.04
E2	GY	2B	AX.89489589	5.86E+08	0.06	-4.54	3.80	0.07
E2	GY	2B	AX.89768980	1.22E+08	0.25	-2.20	3.12	0.05
E2	GY	2B	AX.89403225	1.03E+08	0.07	-4.38	4.09	0.08
E2	GY	2B	AX.89403225	1.03E+08	0.07	-4.38	4.09	0.08

E2	GY	2B	AX.89460927	1.79E+08	0.19	-2.80	4.02	0.06
E2	SPM2	2B	AX.89666169	1.59E+08	0.07	25.80	3.54	0.06
E2	GY	2B	AX.89500923	1.76E+08	0.17	-3.28	4.79	0.08
E2	SPM2	2B	AX.89619909	1.84E+07	0.45	11.71	3.04	0.04
E2	SPM2	2B	AX.89404964	7.49E+08	0.17	15.97	3.17	0.05
E2	SPM2	2B	AX.89705729	1.29E+07	0.30	13.06	3.20	0.05
E2	GY	2B	AX.89713278	5.93E+08	0.06	-4.54	3.80	0.07
E2	GY	2B	AX.89679762	1.43E+08	0.25	-2.18	3.05	0.05
E2	GY	2B	AX.89555674	1.80E+08	0.20	-3.16	5.03	0.08
E2	GY	2B	AX.89416037	1.83E+08	0.14	-3.55	4.92	0.08
E2	SPM2	2B	AX.89555283	7.48E+08	0.05	28.05	3.37	0.06
E2	GY	2B	AX.89369437	1.22E+08	0.25	-2.32	3.39	0.05
E2	GY	2B	AX.89369437	1.22E+08	0.25	-2.32	3.39	0.05
E2	SPM2	2B	AX.89580678	1.71E+07	0.40	12.08	3.12	0.05
E2	SPM2	2B	AX.89446539	1.74E+07	0.40	12.08	3.12	0.05
E2	GY	2B	AX.89722560	1.84E+08	0.14	-3.11	3.81	0.06
E2	GY	2B	AX.89357145	1.80E+08	0.19	-2.80	4.02	0.06
E2	GY	2B	AX.89552327	1.78E+08	0.20	-3.16	5.03	0.08
E2	GY	2B	AX.89677120	1.79E+08	0.20	-2.93	4.55	0.07
E2	SPM2	2B	AX.89611282	7.48E+08	0.05	28.05	3.37	0.06
E2	GY	2B	AX.89511482	1.83E+08	0.13	-3.18	3.87	0.06
E2	GY	2B	AX.89511482	1.83E+08	0.13	-3.18	3.87	0.06
E2	SPM2	2B	AX.89722263	1.90E+07	0.20	15.02	3.21	0.05
E2	SPM2	2B	AX.89449821	1.71E+07	0.40	12.08	3.12	0.05
E2	GY	2B	AX.89452013	1.82E+08	0.14	-3.66	5.11	0.08
E2	GY	2B	AX.89511784	1.19E+08	0.24	-2.19	3.01	0.05
E2	GY	2B	AX.89545552	6.63E+08	0.05	-4.88	3.79	0.05
E2	GY	2B	AX.89545552	6.63E+08	0.05	-4.88	3.79	0.05
E2	GY	2B	AX.89754603	1.22E+08	0.25	-2.18	3.04	0.05
E2	SPM2	2B	AX.89448941	1.71E+07	0.39	12.72	3.39	0.05
E2	GY	2B	AX.89690569	1.78E+08	0.18	-3.40	5.31	0.09
E2	SPM2	2B	AX.89355595	1.82E+07	0.48	-12.60	3.48	0.05
E2	GY	2B	AX.89727447	1.76E+08	0.17	-3.02	4.23	0.07
E2	GY	2B	AX.89441347	1.79E+08	0.19	-2.80	4.02	0.06
E2	SPM2	2B	AX.89395502	1.89E+07	0.20	15.02	3.21	0.05
E2	GY	2B	AX.89406830	1.22E+08	0.25	-2.18	3.04	0.05
E2	GY	2B	AX.89617915	1.47E+08	0.16	-2.83	3.49	0.05
E2	SPM2	2B	AX.89438400	1.71E+07	0.39	13.28	3.65	0.05
E2	GY	2B	AX.89736400	1.82E+08	0.13	-3.57	4.64	0.07
E2	SPM2	2B	AX.89532288	7.86E+08	0.05	28.90	3.55	0.06
E2	GY	2B	AX.89407434	7.95E+08	0.08	-3.54	3.10	0.04
E2	GY	2B	AX.89407434	7.95E+08	0.08	-3.54	3.10	0.04
E2	GY	2B	AX.89692003	1.76E+08	0.17	-3.02	4.23	0.07
E2	GY	2B	AX.89336171	5.94E+08	0.06	-4.54	3.80	0.07
E2	SPM2	2B	AX.89574048	7.86E+08	0.05	28.90	3.55	0.06
E2	SPM2	2B	AX.89702619	7.47E+08	0.05	28.05	3.37	0.06
E2	SPM2	2B	AX.89384802	1.76E+07	0.38	12.30	3.18	0.05
E2	SPM2	2B	AX.89338321	7.48E+08	0.05	28.05	3.37	0.06
E2	GY	2B	AX.89408291	1.40E+08	0.13	-3.14	3.57	0.06
E2	SPM2	2B	AX.89625996	1.76E+07	0.38	12.30	3.18	0.05

E2	GY	2B	AX.89619321	1.80E+08	0.19	-2.80	4.02	0.06
E2	GY	2B	AX.89443676	7.58E+08	0.06	-3.99	3.11	0.04
E2	SPM2	2B	AX.89368784	6.23E+08	0.23	14.06	3.11	0.05
E2	GY	2B	AX.89583382	1.81E+08	0.12	-4.12	5.49	0.09
E2	SPM2	2B	AX.89612878	8.01E+08	0.05	26.76	3.11	0.05
E2	GY	2B	AX.89561740	6.64E+08	0.06	-5.47	5.01	0.07
E2	SPM2	2B	AX.89694614	1.89E+07	0.20	14.50	3.07	0.05
E2	GY	2B	AX.89718685	1.82E+08	0.13	-4.02	5.87	0.09
E2	GY	2B	AX.89710344	7.57E+08	0.07	-4.00	3.30	0.04
E2	GY	2B	AX.89754232	1.30E+08	0.25	-2.20	3.12	0.05
E2	GY	2B	AX.89549272	1.34E+08	0.06	-4.89	4.26	0.05
E2	GY	2B	AX.89549272	1.34E+08	0.06	-4.89	4.26	0.05
E2	GY	2B	AX.89410146	1.84E+08	0.14	-3.55	4.92	0.08
E2	GY	2B	AX.89374293	1.02E+08	0.07	-4.38	4.09	0.08
E2	GY	2B	AX.89374293	1.02E+08	0.07	-4.38	4.09	0.08
E2	GY	2B	AX.89561014	5.93E+08	0.06	-4.54	3.80	0.07
E2	GY	2B	AX.89561014	5.93E+08	0.06	-4.54	3.80	0.07
E2	GY	2B	AX.89410514	4.13E+08	0.06	-4.75	4.16	0.06
E2	GY	2B	AX.89410514	4.13E+08	0.06	-4.75	4.16	0.06
E2	GY	2B	AX.89750857	1.81E+08	0.11	-3.62	4.24	0.07
E2	GY	2B	AX.89750857	1.81E+08	0.11	-3.62	4.24	0.07
E2	GY	2B	AX.89654194	1.81E+08	0.11	-3.62	4.24	0.07
E2	SPM2	2B	AX.89662480	1.71E+07	0.38	12.96	3.48	0.05
E2	SPM2	2B	AX.89410576	1.89E+07	0.20	15.02	3.21	0.05
E2	GY	2B	AX.89445883	1.83E+08	0.14	-3.11	3.81	0.06
E2	SPM2	2B	AX.89494078	1.74E+07	0.40	12.08	3.12	0.05
E2	GY	2B	AX.89434536	1.82E+08	0.13	-4.02	5.87	0.09
E2	GY	2B	AX.89434536	1.82E+08	0.13	-4.02	5.87	0.09
E2	GY	2B	AX.89517124	1.48E+08	0.16	-2.85	3.58	0.05
E2	GY	2B	AX.89517124	1.48E+08	0.16	-2.85	3.58	0.05
E2	GPS	2B	AX.89465814	6.60E+08	0.16	-1.79	3.03	0.04
E2	GY	2B	AX.89375686	6.61E+08	0.06	-5.03	4.29	0.06
E2	GY	2B	AX.89375686	6.61E+08	0.06	-5.03	4.29	0.06
E2	GY	2B	AX.89469035	7.60E+08	0.06	-3.99	3.11	0.04
E2	GY	2B	AX.89469035	7.60E+08	0.06	-3.99	3.11	0.04
E2	GY	2B	AX.89729189	1.83E+08	0.14	-3.55	4.92	0.08
E2	GY	2B	AX.89729189	1.83E+08	0.14	-3.55	4.92	0.08
E2	GY	2B	AX.89376677	1.82E+08	0.13	-4.02	5.87	0.09
E2	GY	2B	AX.89376677	1.82E+08	0.13	-4.02	5.87	0.09
E2	GY	2B	AX.89551447	1.75E+08	0.18	-3.40	5.31	0.09
E2	GY	2B	AX.89551447	1.75E+08	0.18	-3.40	5.31	0.09
E2	GY	2B	AX.89647344	7.59E+08	0.06	-3.99	3.11	0.04
E2	GY	2B	AX.89647344	7.59E+08	0.06	-3.99	3.11	0.04
E2	GY	2B	AX.89483565	6.62E+08	0.06	-5.03	4.29	0.06
E2	GY	2B	AX.89483565	6.62E+08	0.06	-5.03	4.29	0.06
E2	GY	2B	AX.89413015	6.59E+08	0.06	-5.03	4.29	0.06
E2	GY	2B	AX.89413015	6.59E+08	0.06	-5.03	4.29	0.06
E2	GY	2B	AX.86167754	1.28E+08	0.06	-4.89	4.26	0.05
E2	GY	2B	AX.86167754	1.28E+08	0.06	-4.89	4.26	0.05
E2	GY	2B	AX.89673041	6.59E+08	0.06	-5.03	4.29	0.06

E2	GY	2B	AX.89673041	6.59E+08	0.06	-5.03	4.29	0.06
E2	GY	2B	AX.89730024	1.82E+08	0.13	-4.02	5.87	0.09
E2	GY	2B	AX.89730024	1.82E+08	0.13	-4.02	5.87	0.09
E2	GY	2B	AX.89458240	1.82E+08	0.13	-3.57	4.64	0.07
E2	GY	2B	AX.89341825	1.02E+08	0.07	-4.38	4.09	0.08
E2	GY	2B	AX.89341825	1.02E+08	0.07	-4.38	4.09	0.08
E2	GY	2B	AX.89734127	7.59E+08	0.05	-5.13	4.22	0.05
E2	GY	2B	AX.89734127	7.59E+08	0.05	-5.13	4.22	0.05
E2	GY	2B	AX.89734149	7.57E+08	0.06	-3.99	3.11	0.04
E2	GY	2B	AX.89734149	7.57E+08	0.06	-3.99	3.11	0.04
E2	GY	2B	AX.89518723	1.80E+08	0.19	-2.80	4.02	0.06
E2	GY	2B	AX.89518723	1.80E+08	0.19	-2.80	4.02	0.06
E2	GY	2B	AX.89342210	6.64E+08	0.05	-4.64	3.48	0.05
E2	GY	2B	AX.89470748	6.62E+08	0.06	-5.79	5.90	0.09
E2	GY	2B	AX.89588628	7.59E+08	0.06	-3.99	3.11	0.04
E2	GY	2B	AX.89588628	7.59E+08	0.06	-3.99	3.11	0.04
E2	GY	2B	AX.89564960	1.80E+08	0.20	-3.16	5.03	0.08
E2	GY	2B	AX.89564960	1.80E+08	0.20	-3.16	5.03	0.08
E2	GY	2B	AX.89771820	7.93E+08	0.35	-2.09	3.34	0.07
E2	GY	2B	AX.89771820	7.93E+08	0.35	-2.09	3.34	0.07
E2	GY	2B	AX.89459151	1.31E+08	0.25	-2.18	3.04	0.05
E2	GY	2B	AX.89459151	1.31E+08	0.25	-2.18	3.04	0.05
E2	GY	2B	AX.89377960	7.75E+08	0.11	-3.14	3.16	0.06
E2	GY	2B	AX.89377960	7.75E+08	0.11	-3.14	3.16	0.06
E2	GY	2B	AX.89342772	1.23E+08	0.25	-2.20	3.12	0.05
E2	GY	2B	AX.89342772	1.23E+08	0.25	-2.20	3.12	0.05
E2	GY	2B	AX.89342802	1.82E+08	0.12	-4.12	5.49	0.09
E2	GY	2B	AX.89342802	1.82E+08	0.12	-4.12	5.49	0.09
E2	GY	2B	AX.86184320	1.77E+08	0.18	-3.40	5.31	0.09
E2	GY	2B	AX.86184320	1.77E+08	0.18	-3.40	5.31	0.09
E2	GY	2B	AX.89459648	1.75E+08	0.18	-3.40	5.31	0.09
E2	GY	2B	AX.89674559	8.60E+06	0.35	-2.19	3.57	0.04
E2	GY	2B	AX.89589720	1.02E+08	0.07	-4.38	4.09	0.08
E2	GY	2B	AX.89589720	1.02E+08	0.07	-4.38	4.09	0.08
E2	GY	2B	AX.89449337	1.80E+08	0.20	-2.59	3.61	0.06
E2	GY	2B	AX.89449337	1.80E+08	0.20	-2.59	3.61	0.06
E2	GY	2B	AX.89343431	1.75E+08	0.18	-3.40	5.31	0.09
E2	GY	2B	AX.89343431	1.75E+08	0.18	-3.40	5.31	0.09
E2	GPM2	2B	AX.89385328	9.84E+07	0.08	-1036.75	3.92	0.07
E2	GY	2B	AX.89460256	1.76E+08	0.17	-3.02	4.23	0.07
E2	GY	2B	AX.89590527	1.33E+08	0.07	-4.06	3.45	0.04
E2	GY	2B	AX.89486060	1.80E+08	0.20	-3.16	5.03	0.08
E2	GY	2B	AX.89486060	1.80E+08	0.20	-3.16	5.03	0.08
E2	GY	2B	AX.89736861	6.64E+08	0.05	-4.64	3.48	0.05
E2	GY	2B	AX.89736861	6.64E+08	0.05	-4.64	3.48	0.05
E2	GY	2B	AX.89727517	7.57E+08	0.06	-4.29	3.32	0.04
E2	GY	2B	AX.89727517	7.57E+08	0.06	-4.29	3.32	0.04
E2	GY	2B	AX.89461227	1.22E+08	0.25	-2.18	3.04	0.05
E2	GY	2B	AX.89391373	1.78E+08	0.19	-2.80	4.02	0.06
E2	GY	2B	AX.89730333	1.81E+08	0.12	-3.57	4.28	0.07

E2	GY	2B	AX.89730333	1.81E+08	0.12	-3.57	4.28	0.07
E2	GY	2B	AX.89691900	1.80E+08	0.20	-2.59	3.61	0.06
E2	GY	2B	AX.89691900	1.80E+08	0.20	-2.59	3.61	0.06
E2	GY	2B	AX.89555674	1.80E+08	0.20	-3.16	5.03	0.08
E2	TKW	2B	AX.89501127	3.70E+07	0.13	1.54	3.77	0.07
E2	GY	2B	AX.89416037	1.83E+08	0.14	-3.55	4.92	0.08
E2	GY	2B	AX.89544169	1.22E+08	0.25	-2.18	3.04	0.05
E2	GY	2B	AX.89544169	1.22E+08	0.25	-2.18	3.04	0.05
E2	GY	2B	AX.89369551	1.81E+08	0.24	-2.59	4.06	0.06
E2	GY	2B	AX.89369551	1.81E+08	0.24	-2.59	4.06	0.06
E2	GY	2B	AX.89474590	1.82E+08	0.12	-4.12	5.49	0.09
E2	GY	2B	AX.89474590	1.82E+08	0.12	-4.12	5.49	0.09
E2	GY	2B	AX.89736105	1.48E+08	0.17	-2.87	3.72	0.06
E2	GY	2B	AX.89736105	1.48E+08	0.17	-2.87	3.72	0.06
E2	GY	2B	AX.89665143	1.78E+08	0.20	-3.16	5.03	0.08
E2	GY	2B	AX.89665143	1.78E+08	0.20	-3.16	5.03	0.08
E2	GY	2B	AX.89771483	7.57E+08	0.06	-3.99	3.11	0.04
E2	GY	2B	AX.89771483	7.57E+08	0.06	-3.99	3.11	0.04
E2	GY	2B	AX.89552651	1.81E+08	0.12	-4.12	5.49	0.09
E2	GY	2B	AX.89552651	1.81E+08	0.12	-4.12	5.49	0.09
E2	GY	2B	AX.89487985	1.83E+08	0.14	-3.11	3.81	0.06
E2	GY	2B	AX.89487985	1.83E+08	0.14	-3.11	3.81	0.06
E2	GY	2B	AX.89451802	6.59E+08	0.06	-5.03	4.29	0.06
E2	GY	2B	AX.89451802	6.59E+08	0.06	-5.03	4.29	0.06
E2	GY	2B	AX.89705102	1.58E+08	0.08	-3.47	3.09	0.05
E2	GY	2B	AX.89705102	1.58E+08	0.08	-3.47	3.09	0.05
E2	GY	2B	AX.89381154	7.57E+08	0.06	-4.75	3.93	0.05
E2	GY	2B	AX.89381154	7.57E+08	0.06	-4.75	3.93	0.05
E2	GY	2B	AX.89312467	6.60E+08	0.07	-4.01	3.30	0.04
E2	GY	2B	AX.89312467	6.60E+08	0.07	-4.01	3.30	0.04
E2	GY	2B	AX.89593197	9.71E+06	0.22	-2.52	3.55	0.05
E2	GY	2B	AX.89593197	9.71E+06	0.22	-2.52	3.55	0.05
E2	GY	2B	AX.89690045	1.82E+08	0.13	-4.02	5.87	0.09
E2	GY	2B	AX.89690045	1.82E+08	0.13	-4.02	5.87	0.09
E2	GY	2B	AX.89628294	1.81E+08	0.13	-3.66	4.75	0.08
E2	GY	2B	AX.89628294	1.81E+08	0.13	-3.66	4.75	0.08
E2	GY	2B	AX.89523462	1.84E+08	0.13	-3.16	3.75	0.06
E2	GY	2B	AX.89523462	1.84E+08	0.13	-3.16	3.75	0.06
E2	GY	2B	AX.89452163	1.80E+08	0.19	-2.80	4.02	0.06
E2	GY	2B	AX.89452163	1.80E+08	0.19	-2.80	4.02	0.06
E2	GY	2B	AX.89381365	1.43E+08	0.25	-2.18	3.05	0.05
E2	GY	2B	AX.89381365	1.43E+08	0.25	-2.18	3.05	0.05
E2	GY	2B	AX.89749678	6.59E+08	0.06	-5.03	4.29	0.06
E2	GY	2B	AX.89749678	6.59E+08	0.06	-5.03	4.29	0.06
E2	GY	2B	AX.89703355	1.81E+08	0.12	-4.12	5.49	0.09
E2	GY	2B	AX.89703355	1.81E+08	0.12	-4.12	5.49	0.09
E2	GY	2B	AX.89534791	1.02E+08	0.07	-4.38	4.09	0.08
E2	GY	2B	AX.89593986	1.53E+08	0.06	-4.16	3.10	0.05
E2	GY	2B	AX.89593986	1.53E+08	0.06	-4.16	3.10	0.05
E2	GY	2B	AX.89417809	1.31E+08	0.25	-2.18	3.04	0.05

E2	GY	2B	AX.89417809	1.31E+08	0.25	-2.18	3.04	0.05
E2	GY	2B	AX.89764413	1.83E+08	0.14	-3.11	3.81	0.06
E2	GY	2B	AX.89764413	1.83E+08	0.14	-3.11	3.81	0.06
E2	GY	2B	AX.89524212	1.84E+08	0.14	-2.99	3.66	0.06
E2	GY	2B	AX.89524212	1.84E+08	0.14	-2.99	3.66	0.06
E2	GY	2B	AX.89703625	6.62E+08	0.06	-5.03	4.29	0.06
E2	GY	2B	AX.89666887	1.30E+08	0.07	-5.62	5.82	0.08
E2	GY	2B	AX.89666887	1.30E+08	0.07	-5.62	5.82	0.08
E2	GY	2B	AX.89558174	1.81E+08	0.12	-4.12	5.49	0.09
E2	GY	2B	AX.89558174	1.81E+08	0.12	-4.12	5.49	0.09
E2	GY	2B	AX.89736400	1.82E+08	0.13	-3.57	4.64	0.07
E2	GY	2B	AX.89325940	1.83E+08	0.14	-3.11	3.81	0.06
E2	GY	2B	AX.89594592	1.80E+08	0.20	-3.16	5.03	0.08
E2	GY	2B	AX.89594592	1.80E+08	0.20	-3.16	5.03	0.08
E2	GY	2B	AX.89418966	7.98E+08	0.05	-4.30	3.09	0.04
E2	GY	2B	AX.89418966	7.98E+08	0.05	-4.30	3.09	0.04
E2	GY	2B	AX.89525241	6.92E+08	0.08	-3.70	3.31	0.06
E2	GY	2B	AX.89525241	6.92E+08	0.08	-3.70	3.31	0.06
E2	GY	2B	AX.89525251	7.57E+08	0.06	-3.99	3.11	0.04
E2	GY	2B	AX.89525251	7.57E+08	0.06	-3.99	3.11	0.04
E2	GY	2B	AX.89752546	1.80E+08	0.19	-2.80	4.02	0.06
E2	GY	2B	AX.89752546	1.80E+08	0.19	-2.80	4.02	0.06
E2	GPS	2B	AX.89343474	6.60E+08	0.16	-1.79	3.03	0.04
E2	GY	2B	AX.89454067	1.82E+08	0.13	-4.02	5.87	0.09
E2	GY	2B	AX.89454067	1.82E+08	0.13	-4.02	5.87	0.09
E2	GY	2B	AX.89348028	1.30E+08	0.25	-2.16	3.02	0.05
E2	GY	2B	AX.89348028	1.30E+08	0.25	-2.16	3.02	0.05
E2	GY	2B	AX.89699414	1.79E+08	0.20	-2.93	4.48	0.07
E2	GY	2B	AX.89699414	1.79E+08	0.20	-2.93	4.48	0.07
E2	GY	2B	AX.89595331	1.84E+08	0.14	-2.71	3.08	0.04
E2	TKW	2B	AX.89688417	7.43E+08	0.36	0.99	3.33	0.06
E2	GY	2B	AX.89395850	1.84E+08	0.14	-2.71	3.08	0.04
E2	TKW	2B	AX.89713335	1.55E+08	0.20	-1.24	3.53	0.06
E2	GY	2B	AX.89419963	6.60E+08	0.06	-4.09	3.20	0.04
E2	GY	2B	AX.89419963	6.60E+08	0.06	-4.09	3.20	0.04
E2	GY	2B	AX.89408490	1.83E+08	0.14	-3.56	4.86	0.08
E2	GY	2B	AX.89372938	1.22E+08	0.25	-2.18	3.04	0.05
E2	GY	2B	AX.89372938	1.22E+08	0.25	-2.18	3.04	0.05
E2	GY	2B	AX.89561740	6.64E+08	0.06	-5.47	5.01	0.07
E2	GY	2B	AX.89537067	1.22E+08	0.25	-2.18	3.04	0.05
E2	GY	2B	AX.89560602	7.60E+08	0.06	-4.75	3.93	0.05
E2	GY	2B	AX.89560602	7.60E+08	0.06	-4.75	3.93	0.05
E2	GY	2B	AX.89455015	1.21E+08	0.25	-2.18	3.04	0.05
E2	GY	2B	AX.89455015	1.21E+08	0.25	-2.18	3.04	0.05
E2	GY	2B	AX.89316103	1.75E+08	0.18	-3.40	5.31	0.09
E2	GY	2B	AX.89316103	1.75E+08	0.18	-3.40	5.31	0.09
E2	GY	2B	AX.89384745	1.83E+08	0.14	-3.11	3.81	0.06
E2	GY	2B	AX.89384745	1.83E+08	0.14	-3.11	3.81	0.06
E2	GY	2B	AX.89669019	6.64E+08	0.06	-4.62	3.95	0.05
E2	GY	2B	AX.89669019	6.64E+08	0.06	-4.62	3.95	0.05

E2	GY	2B	AX.89556519	1.83E+08	0.14	-3.11	3.81	0.06
E2	GY	2B	AX.89556519	1.83E+08	0.14	-3.11	3.81	0.06
E2	GY	2B	AX.89632158	1.53E+08	0.06	-4.16	3.10	0.05
E2	GY	2B	AX.89632158	1.53E+08	0.06	-4.16	3.10	0.05
E2	GY	2B	AX.89455556	1.80E+08	0.19	-2.80	4.02	0.06
E2	GY	2B	AX.89455556	1.80E+08	0.19	-2.80	4.02	0.06
E2	GY	2B	AX.89527353	1.81E+08	0.20	-2.71	3.96	0.06
E2	GY	2B	AX.89527353	1.81E+08	0.20	-2.71	3.96	0.06
E2	GY	2B	AX.89316984	1.83E+08	0.14	-3.11	3.81	0.06
E2	GY	2B	AX.89316984	1.83E+08	0.14	-3.11	3.81	0.06
E2	GY	2B	AX.89374622	1.78E+08	0.19	-2.80	4.02	0.06
E2	GY	2B	AX.89374622	1.78E+08	0.19	-2.80	4.02	0.06
E2	GY	2B	AX.89385918	1.79E+08	0.20	-3.16	5.03	0.08
E2	GY	2B	AX.89385918	1.79E+08	0.20	-3.16	5.03	0.08
E2	GY	2B	AX.89455994	1.28E+08	0.25	-2.20	3.12	0.05
E2	GY	2B	AX.89455994	1.28E+08	0.25	-2.20	3.12	0.05
E2	GY	2B	AX.89351218	6.59E+08	0.06	-5.79	5.90	0.09
E2	GY	2B	AX.89351218	6.59E+08	0.06	-5.79	5.90	0.09
E2	TKW	2B	AX.89390936	7.40E+08	0.37	0.96	3.20	0.06
E2	GY	2B	AX.89562032	6.59E+08	0.07	-4.01	3.30	0.04
E2	GY	2B	AX.89562032	6.59E+08	0.07	-4.01	3.30	0.04
E2	GY	2B	AX.89562158	6.60E+08	0.06	-5.03	4.29	0.06
E2	GY	2B	AX.89562158	6.60E+08	0.06	-5.03	4.29	0.06
E2	GY	2B	AX.89598424	1.82E+08	0.14	-3.11	3.81	0.06
E2	GY	2B	AX.89598424	1.82E+08	0.14	-3.11	3.81	0.06
E2	GY	2B	AX.89386261	5.18E+08	0.09	-3.52	3.51	0.05
E2	GY	2B	AX.89732531	6.60E+08	0.07	-4.01	3.30	0.04
E2	GY	2B	AX.89732531	6.60E+08	0.07	-4.01	3.30	0.04
E2	GY	2B	AX.89535945	1.80E+08	0.19	-2.80	4.02	0.06
E2	GY	2B	AX.89535945	1.80E+08	0.19	-2.80	4.02	0.06
E2	GY	2B	AX.89493461	1.83E+08	0.14	-3.55	4.92	0.08
E2	GY	2B	AX.89493461	1.83E+08	0.14	-3.55	4.92	0.08
E2	GY	2B	AX.89493544	1.00E+08	0.07	-4.38	4.09	0.08
E2	GY	2B	AX.89493544	1.00E+08	0.07	-4.38	4.09	0.08
E2	GY	2B	AX.89749264	1.79E+08	0.19	-2.80	4.02	0.06
E2	GY	2B	AX.89749264	1.79E+08	0.19	-2.80	4.02	0.06
E2	GY	2B	AX.89387058	1.03E+08	0.07	-4.38	4.09	0.08
E2	GY	2B	AX.89387058	1.03E+08	0.07	-4.38	4.09	0.08
E2	GY	2B	AX.89745248	1.82E+08	0.12	-4.12	5.49	0.09
E2	GY	2B	AX.89745248	1.82E+08	0.12	-4.12	5.49	0.09
E2	GY	2B	AX.89623870	5.89E+08	0.06	-4.54	3.80	0.07
E2	GY	2B	AX.89623870	5.89E+08	0.06	-4.54	3.80	0.07
E2	GY	2B	AX.89319203	6.59E+08	0.06	-4.20	3.34	0.04
E2	GY	2B	AX.89319203	6.59E+08	0.06	-4.20	3.34	0.04
E2	GY	2B	AX.89529850	1.78E+08	0.19	-2.80	4.02	0.06
E2	GY	2B	AX.89529850	1.78E+08	0.19	-2.80	4.02	0.06
E2	GY	2B	AX.89387913	1.47E+08	0.20	-2.46	3.18	0.06
E2	GY	2B	AX.89387913	1.47E+08	0.20	-2.46	3.18	0.06
E2	GY	2B	AX.89388066	1.77E+08	0.17	-3.02	4.23	0.07
E2	GY	2B	AX.89388066	1.77E+08	0.17	-3.02	4.23	0.07

E2	GY	2B	AX.89319660	1.52E+08	0.06	-4.16	3.10	0.05
E2	GY	2B	AX.89319660	1.52E+08	0.06	-4.16	3.10	0.05
E2	GY	2B	AX.89432728	1.81E+08	0.26	-2.46	3.87	0.06
E2	GY	2B	AX.89432728	1.81E+08	0.26	-2.46	3.87	0.06
E2	GY	2B	AX.89319725	6.64E+08	0.05	-4.64	3.48	0.05
E2	GY	2B	AX.89319725	6.64E+08	0.05	-4.64	3.48	0.05
E2	GY	2B	AX.89458240	1.82E+08	0.13	-3.57	4.64	0.07
E2	GY	2B	AX.89672039	1.02E+08	0.07	-4.38	4.09	0.08
E2	GY	2B	AX.89672039	1.02E+08	0.07	-4.38	4.09	0.08
E2	GY	2B	AX.89753493	1.81E+08	0.24	-2.28	3.21	0.05
E2	GY	2B	AX.89753493	1.81E+08	0.24	-2.28	3.21	0.05
E2	GY	2B	AX.89484326	1.78E+08	0.19	-2.80	4.02	0.06
E2	GY	2B	AX.89484326	1.78E+08	0.19	-2.80	4.02	0.06
E2	GY	2B	AX.89746318	5.76E+08	0.05	-5.83	4.80	0.10
E2	GY	2B	AX.89746318	5.76E+08	0.05	-5.83	4.80	0.10
E2	GY	2B	AX.89388755	1.23E+08	0.25	-2.20	3.12	0.05
E2	GY	2B	AX.89388755	1.23E+08	0.25	-2.20	3.12	0.05
E2	GY	2B	AX.89342210	6.64E+08	0.05	-4.64	3.48	0.05
E2	GY	2B	AX.89458714	1.76E+08	0.17	-3.02	4.23	0.07
E2	GY	2B	AX.89458714	1.76E+08	0.17	-3.02	4.23	0.07
E2	GY	2B	AX.89353728	1.82E+08	0.13	-3.57	4.64	0.07
E2	GY	2B	AX.89576162	5.94E+08	0.06	-4.54	3.80	0.07
E2	GY	2B	AX.89576162	5.94E+08	0.06	-4.54	3.80	0.07
E2	GY	2B	AX.89605899	1.81E+08	0.13	-4.14	6.29	0.11
E2	GY	2B	AX.89605899	1.81E+08	0.13	-4.14	6.29	0.11
E2	GY	2B	AX.89354314	1.80E+08	0.20	-2.81	4.09	0.07
E2	GY	2B	AX.89354314	1.80E+08	0.20	-2.81	4.09	0.07
E2	GY	2B	AX.89459648	1.75E+08	0.18	-3.40	5.31	0.09
E2	GY	2B	AX.89674559	8.60E+06	0.35	-2.19	3.57	0.04
E2	GY	2B	AX.89624886	4.95E+08	0.11	-3.32	3.50	0.04
E2	GY	2B	AX.89624886	4.95E+08	0.11	-3.32	3.50	0.04
E2	GY	2B	AX.89343257	1.79E+08	0.20	-2.59	3.61	0.06
E2	GY	2B	AX.89343257	1.79E+08	0.20	-2.59	3.61	0.06
E2	GY	2B	AX.89637464	7.60E+08	0.06	-3.99	3.11	0.04
E2	GY	2B	AX.89637464	7.60E+08	0.06	-3.99	3.11	0.04
E2	GY	2B	AX.89496480	1.75E+08	0.18	-3.40	5.31	0.09
E2	GY	2B	AX.89496480	1.75E+08	0.18	-3.40	5.31	0.09
E2	GY	2B	AX.89460256	1.76E+08	0.17	-3.02	4.23	0.07
E2	GY	2B	AX.89355285	1.83E+08	0.15	-3.18	4.16	0.06
E2	GY	2B	AX.89355285	1.83E+08	0.15	-3.18	4.16	0.06
E2	GY	2B	AX.89332441	1.83E+08	0.14	-3.55	4.92	0.08
E2	GY	2B	AX.89577473	7.58E+08	0.06	-3.99	3.11	0.04
E2	GY	2B	AX.89577473	7.58E+08	0.06	-3.99	3.11	0.04
E2	GY	2B	AX.89590619	1.81E+08	0.12	-4.12	5.49	0.09
E2	GY	2B	AX.89590619	1.81E+08	0.12	-4.12	5.49	0.09
E2	GY	2B	AX.89460581	1.43E+08	0.25	-2.18	3.05	0.05
E2	GY	2B	AX.89460581	1.43E+08	0.25	-2.18	3.05	0.05
E2	GY	2B	AX.89332789	1.80E+08	0.20	-3.16	5.03	0.08
E2	GY	2B	AX.89625965	1.83E+08	0.14	-3.55	4.92	0.08
E2	GY	2B	AX.89625965	1.83E+08	0.14	-3.55	4.92	0.08

E2	GY	2B	AX.89521003	9.66E+05	0.07	-5.59	6.51	0.10
E2	GY	2B	AX.89521003	9.66E+05	0.07	-5.59	6.51	0.10
E2	GY	2B	AX.89486570	1.79E+08	0.19	-2.80	4.02	0.06
E2	GY	2B	AX.89486570	1.79E+08	0.19	-2.80	4.02	0.06
E2	GY	2B	AX.89591121	1.76E+08	0.17	-3.07	4.29	0.07
E2	GY	2B	AX.89591121	1.76E+08	0.17	-3.07	4.29	0.07
E2	GY	2B	AX.89663318	7.94E+08	0.09	-3.65	3.55	0.05
E2	GY	2B	AX.89663318	7.94E+08	0.09	-3.65	3.55	0.05
E2	GY	2B	AX.89774529	1.83E+08	0.14	-3.11	3.81	0.06
E2	GY	2B	AX.89774529	1.83E+08	0.14	-3.11	3.81	0.06
E2	GY	2B	AX.89461227	1.22E+08	0.25	-2.18	3.04	0.05
E2	GY	2B	AX.89391373	1.78E+08	0.19	-2.80	4.02	0.06
E2	GY	2B	AX.89603443	6.64E+08	0.05	-4.74	3.62	0.05
E2	GY	2B	AX.89603443	6.64E+08	0.05	-4.74	3.62	0.05
E2	GY	2B	AX.89615270	1.76E+08	0.17	-3.02	4.23	0.07
E2	GY	2B	AX.89615270	1.76E+08	0.17	-3.02	4.23	0.07
E2	GY	2B	AX.89323244	1.83E+08	0.14	-3.55	4.92	0.08
E2	GY	2B	AX.89323244	1.83E+08	0.14	-3.55	4.92	0.08
E2	GY	2B	AX.89749024	1.82E+08	0.13	-3.57	4.64	0.07
E2	GY	2B	AX.89749024	1.82E+08	0.13	-3.57	4.64	0.07
E2	GY	2B	AX.89497642	1.82E+08	0.12	-4.12	5.49	0.09
E2	GY	2B	AX.89497642	1.82E+08	0.12	-4.12	5.49	0.09
E2	GY	2B	AX.89323590	1.43E+08	0.25	-2.18	3.05	0.05
E2	GY	2B	AX.89323590	1.43E+08	0.25	-2.18	3.05	0.05
E2	GY	2B	AX.89647581	1.76E+08	0.17	-3.02	4.23	0.07
E2	GY	2B	AX.89647581	1.76E+08	0.17	-3.02	4.23	0.07
E2	GY	2B	AX.89461833	6.59E+08	0.06	-5.03	4.29	0.06
E2	GY	2B	AX.89461833	6.59E+08	0.06	-5.03	4.29	0.06
E2	GY	2B	AX.89323675	6.59E+08	0.06	-5.03	4.29	0.06
E2	GY	2B	AX.89323675	6.59E+08	0.06	-5.03	4.29	0.06
E2	GY	2B	AX.89323891	1.22E+08	0.25	-2.18	3.04	0.05
E2	GY	2B	AX.89323891	1.22E+08	0.25	-2.18	3.04	0.05
E2	GY	2B	AX.89323978	1.76E+08	0.17	-3.02	4.23	0.07
E2	GY	2B	AX.89323978	1.76E+08	0.17	-3.02	4.23	0.07
E2	GY	2B	AX.89393166	7.57E+08	0.07	-4.66	4.58	0.06
E2	GY	2B	AX.89393166	7.57E+08	0.07	-4.66	4.58	0.06
E2	GY	2B	AX.89324249	1.77E+08	0.18	-3.40	5.31	0.09
E2	GY	2B	AX.89324249	1.77E+08	0.18	-3.40	5.31	0.09
E2	GY	2B	AX.89511784	1.19E+08	0.24	-2.19	3.01	0.05
E2	GY	2B	AX.89393550	7.59E+08	0.06	-3.99	3.11	0.04
E2	GY	2B	AX.89393550	7.59E+08	0.06	-3.99	3.11	0.04
E2	GY	2B	AX.89523629	1.83E+08	0.14	-3.11	3.81	0.06
E2	GY	2B	AX.89523629	1.83E+08	0.14	-3.11	3.81	0.06
E2	GY	2B	AX.89690400	1.78E+08	0.19	-2.80	4.02	0.06
E2	GY	2B	AX.89690400	1.78E+08	0.19	-2.80	4.02	0.06
E2	GY	2B	AX.89534363	1.80E+08	0.19	-2.80	4.02	0.06
E2	GY	2B	AX.89534363	1.80E+08	0.19	-2.80	4.02	0.06
E2	GY	2B	AX.89463274	6.59E+08	0.06	-5.03	4.29	0.06
E2	GY	2B	AX.89463274	6.59E+08	0.06	-5.03	4.29	0.06
E2	GY	2B	AX.89605804	1.82E+08	0.14	-3.62	5.15	0.08

E2	GY	2B	AX.89605804	1.82E+08	0.14	-3.62	5.15	0.08
E2	GY	2B	AX.89605884	1.78E+08	0.22	-2.80	4.43	0.07
E2	GY	2B	AX.89605884	1.78E+08	0.22	-2.80	4.43	0.07
E2	GY	2B	AX.89358906	7.57E+08	0.06	-3.99	3.11	0.04
E2	GY	2B	AX.89358906	7.57E+08	0.06	-3.99	3.11	0.04
E2	TKW	2B	AX.89529518	5.94E+08	0.32	0.97	3.09	0.06
E2	GY	2B	AX.89534791	1.02E+08	0.07	-4.38	4.09	0.08
E2	GY	2B	AX.89617519	6.63E+08	0.06	-4.94	4.16	0.06
E2	GY	2B	AX.89739652	5.73E+08	0.06	-5.00	4.26	0.09
E2	GY	2B	AX.89739652	5.73E+08	0.06	-5.00	4.26	0.09
E2	GY	2B	AX.89463396	1.84E+08	0.12	-3.02	3.16	0.05
E2	GY	2B	AX.89463396	1.84E+08	0.12	-3.02	3.16	0.05
E2	GY	2B	AX.89499795	6.62E+08	0.06	-5.03	4.29	0.06
E2	GY	2B	AX.89499795	6.62E+08	0.06	-5.03	4.29	0.06
E2	GY	2B	AX.89325475	1.81E+08	0.12	-4.12	5.49	0.09
E2	GY	2B	AX.89325475	1.81E+08	0.12	-4.12	5.49	0.09
E2	GY	2B	AX.89703625	6.62E+08	0.06	-5.03	4.29	0.06
E2	GY	2B	AX.89535150	1.02E+08	0.07	-4.38	4.09	0.08
E2	GY	2B	AX.89535150	1.02E+08	0.07	-4.38	4.09	0.08
E2	GY	2B	AX.89617915	1.47E+08	0.16	-2.83	3.49	0.05
E2	GY	2B	AX.89558016	1.84E+08	0.14	-3.11	3.81	0.06
E2	GY	2B	AX.89558016	1.84E+08	0.14	-3.11	3.81	0.06
E2	GY	2B	AX.89335901	1.83E+08	0.14	-3.55	4.92	0.08
E2	GY	2B	AX.89325940	1.83E+08	0.14	-3.11	3.81	0.06
E2	GY	2B	AX.89629939	6.62E+08	0.07	-4.97	4.77	0.07
E2	GY	2B	AX.89629939	6.62E+08	0.07	-4.97	4.77	0.07
E2	GY	2B	AX.89326280	1.80E+08	0.20	-3.16	5.03	0.08
E2	GY	2B	AX.89326280	1.80E+08	0.20	-3.16	5.03	0.08
E2	GY	2B	AX.89326350	1.81E+08	0.20	-3.16	5.03	0.08
E2	GY	2B	AX.89326350	1.81E+08	0.20	-3.16	5.03	0.08
E2	GY	2B	AX.89360195	7.59E+08	0.06	-3.99	3.11	0.04
E2	GY	2B	AX.89360195	7.59E+08	0.06	-3.99	3.11	0.04
E2	GY	2B	AX.89464649	1.84E+08	0.14	-2.71	3.08	0.04
E2	GY	2B	AX.89464649	1.84E+08	0.14	-2.71	3.08	0.04
E2	GY	2B	AX.89769704	7.58E+08	0.06	-3.99	3.11	0.04
E2	GY	2B	AX.89769704	7.58E+08	0.06	-3.99	3.11	0.04
E2	GY	2B	AX.89725266	1.83E+08	0.14	-3.11	3.81	0.06
E2	GY	2B	AX.89725266	1.83E+08	0.14	-3.11	3.81	0.06
E2	GY	2B	AX.89595331	1.84E+08	0.14	-2.71	3.08	0.04
E2	GY	2B	AX.89395850	1.84E+08	0.14	-2.71	3.08	0.04
E2	GY	2B	AX.89432025	1.22E+08	0.25	-2.18	3.04	0.05
E2	GY	2B	AX.89432025	1.22E+08	0.25	-2.18	3.04	0.05
E2	GY	2B	AX.89408490	1.83E+08	0.14	-3.56	4.86	0.08
E2	GY	2B	AX.89396452	5.72E+08	0.09	-4.00	4.18	0.08
E2	GY	2B	AX.89396452	5.72E+08	0.09	-4.00	4.18	0.08
E2	GY	2B	AX.89327212	1.80E+08	0.20	-3.16	5.03	0.08
E2	GY	2B	AX.89327212	1.80E+08	0.20	-3.16	5.03	0.08
E2	GY	2B	AX.89537067	1.22E+08	0.25	-2.18	3.04	0.05
E2	TKW	2B	AX.89519502	6.75E+08	0.27	1.02	3.03	0.05
E2	GY	2B	AX.89397326	1.79E+08	0.20	-3.16	5.03	0.08

E2	GY	2B	AX.89397326	1.79E+08	0.20	-3.16	5.03	0.08
E2	GY	2B	AX.89327794	1.78E+08	0.19	-2.80	4.02	0.06
E2	GY	2B	AX.89327794	1.78E+08	0.19	-2.80	4.02	0.06
E2	GY	2B	AX.89327795	1.39E+08	0.08	-4.12	3.97	0.07
E2	GY	2B	AX.89327795	1.39E+08	0.08	-4.12	3.97	0.07
E2	GY	2B	AX.89327830	7.59E+08	0.06	-3.99	3.11	0.04
E2	GY	2B	AX.89327830	7.59E+08	0.06	-3.99	3.11	0.04
E2	GY	2B	AX.89731091	1.75E+08	0.18	-3.40	5.31	0.09
E2	GY	2B	AX.89731091	1.75E+08	0.18	-3.40	5.31	0.09
E2	GY	2B	AX.89549378	1.47E+08	0.20	-2.46	3.18	0.06
E2	GY	2B	AX.89549378	1.47E+08	0.20	-2.46	3.18	0.06
E2	GY	2B	AX.89362279	5.72E+08	0.16	-2.65	3.23	0.06
E2	GY	2B	AX.89362279	5.72E+08	0.16	-2.65	3.23	0.06
E2	GY	2B	AX.89475557	6.59E+08	0.07	-4.01	3.30	0.04
E2	GY	2B	AX.89475557	6.59E+08	0.07	-4.01	3.30	0.04
E2	GY	2B	AX.89681647	1.48E+08	0.15	-2.97	3.61	0.05
E2	GY	2B	AX.89681647	1.48E+08	0.15	-2.97	3.61	0.05
E2	GY	2B	AX.89561621	7.57E+08	0.06	-4.29	3.32	0.04
E2	GY	2B	AX.89561621	7.57E+08	0.06	-4.29	3.32	0.04
E2	GY	2B	AX.89645080	1.23E+08	0.25	-2.20	3.12	0.05
E2	GY	2B	AX.89480571	1.83E+08	0.14	-3.11	3.81	0.06
E2	GY	2B	AX.89480571	1.83E+08	0.14	-3.11	3.81	0.06
E2	GY	2B	AX.89398806	1.81E+08	0.20	-2.71	3.96	0.06
E2	GY	2B	AX.89398806	1.81E+08	0.20	-2.71	3.96	0.06
E2	GY	2B	AX.89445883	1.83E+08	0.14	-3.11	3.81	0.06
E2	GY	2B	AX.89399328	1.83E+08	0.14	-3.11	3.81	0.06
E2	GY	2B	AX.89399328	1.83E+08	0.14	-3.11	3.81	0.06
E2	GY	2B	AX.89682491	1.81E+08	0.12	-3.18	3.62	0.06
E2	GY	2B	AX.89682491	1.81E+08	0.12	-3.18	3.62	0.06
E2	GY	2B	AX.89539161	1.82E+08	0.13	-4.02	5.87	0.09
E2	GY	2B	AX.89386261	5.18E+08	0.09	-3.52	3.51	0.05
E2	GY	2B	AX.89682796	1.84E+08	0.09	-3.41	3.22	0.05
E2	GY	2B	AX.89765084	1.81E+08	0.12	-4.12	5.49	0.09
E2	GY	2B	AX.89363904	6.64E+08	0.06	-5.47	5.01	0.07
E2	GY	2B	AX.89363904	6.64E+08	0.06	-5.47	5.01	0.07
E2	GY	2B	AX.89351791	1.83E+08	0.14	-3.11	3.81	0.06
E2	GY	2B	AX.89634458	1.79E+08	0.19	-2.80	4.02	0.06
E2	GY	2B	AX.89696744	1.79E+08	0.19	-2.80	4.02	0.06
E2	GY	2B	AX.89400251	7.59E+08	0.06	-3.99	3.11	0.04
E2	GY	2B	AX.89745228	1.22E+08	0.25	-2.18	3.04	0.05
E2	GY	2B	AX.89518028	1.81E+08	0.12	-4.12	5.49	0.09
E2	GY	2B	AX.89709245	1.29E+08	0.06	-4.89	4.26	0.05
E2	GY	2B	AX.89709245	1.29E+08	0.06	-4.89	4.26	0.05
E2	GY	2B	AX.89469521	1.83E+08	0.14	-3.55	4.92	0.08
E2	GY	2B	AX.89575510	1.34E+08	0.06	-4.89	4.26	0.05
E2	GY	2B	AX.89575510	1.34E+08	0.06	-4.89	4.26	0.05
E2	GY	2B	AX.89330672	6.60E+08	0.07	-4.01	3.30	0.04
E2	GY	2B	AX.89365441	1.79E+08	0.20	-2.72	3.87	0.06
E2	GY	2B	AX.89365441	1.79E+08	0.20	-2.72	3.87	0.06
E2	GY	2B	AX.89436572	1.82E+08	0.13	-3.57	4.64	0.07

E2	GY	2B	AX.89623578	1.79E+08	0.20	-2.58	3.53	0.05
E2	GY	2B	AX.89575757	1.80E+08	0.19	-2.80	4.02	0.06
E2	GY	2B	AX.89424214	1.79E+08	0.19	-2.80	4.02	0.06
E2	GY	2B	AX.89331026	1.53E+08	0.06	-4.16	3.10	0.05
E2	GY	2B	AX.89353728	1.82E+08	0.13	-3.57	4.64	0.07
E2	GY	2B	AX.89444410	1.81E+08	0.12	-3.18	3.62	0.06
E2	GY	2B	AX.89719069	7.57E+08	0.06	-3.99	3.11	0.04
E2	GY	2B	AX.89564994	1.81E+08	0.11	-3.62	4.24	0.07
E2	GY	2B	AX.89541694	1.40E+08	0.13	-3.15	3.65	0.06
E2	GY	2B	AX.89402018	1.76E+08	0.18	-3.40	5.31	0.09
E2	GY	2B	AX.89734860	1.81E+08	0.11	-3.62	4.24	0.07
E2	GY	2B	AX.89507024	6.62E+08	0.06	-4.41	3.40	0.04
E2	GY	2B	AX.89437088	1.22E+08	0.25	-2.20	3.12	0.05
E2	GY	2B	AX.89698843	1.83E+08	0.14	-3.55	4.92	0.08
E2	GY	2B	AX.89541745	1.82E+08	0.14	-3.11	3.81	0.06
E2	GY	2B	AX.89541745	1.82E+08	0.14	-3.11	3.81	0.06
E2	GY	2B	AX.89507172	1.79E+08	0.20	-3.16	5.03	0.08
E2	GY	2B	AX.89612967	6.61E+08	0.06	-5.03	4.29	0.06
E2	GY	2B	AX.89437310	5.64E+08	0.11	-3.20	3.30	0.04
E2	GY	2B	AX.89331795	1.79E+08	0.20	-2.93	4.48	0.07
E2	GY	2B	AX.89496154	1.79E+08	0.19	-2.80	4.02	0.06
E2	GY	2B	AX.89496154	1.79E+08	0.19	-2.80	4.02	0.06
E2	GY	2B	AX.89542438	6.64E+08	0.05	-4.64	3.48	0.05
E2	GY	2B	AX.89332441	1.83E+08	0.14	-3.55	4.92	0.08
E2	GY	2B	AX.89472568	6.64E+08	0.05	-4.64	3.48	0.05
E2	GY	2B	AX.89686171	1.75E+08	0.17	-3.02	4.23	0.07
E2	GY	2B	AX.89569931	6.62E+08	0.06	-5.79	5.90	0.09
E2	GY	2B	AX.89489589	5.86E+08	0.06	-4.54	3.80	0.07
E2	GY	2B	AX.89768980	1.22E+08	0.25	-2.20	3.12	0.05
E2	GY	2B	AX.89332789	1.80E+08	0.20	-3.16	5.03	0.08
E2	GY	2B	AX.89724642	1.22E+08	0.25	-2.18	3.04	0.05
E2	GY	2B	AX.89724642	1.22E+08	0.25	-2.18	3.04	0.05
E2	GY	2B	AX.89460927	1.79E+08	0.19	-2.80	4.02	0.06
E2	GY	2B	AX.89500923	1.76E+08	0.17	-3.28	4.79	0.08
E2	GY	2B	AX.89368860	6.59E+08	0.06	-5.03	4.29	0.06
E2	GY	2B	AX.89368860	6.59E+08	0.06	-5.03	4.29	0.06
E2	GY	2B	AX.89713278	5.93E+08	0.06	-4.54	3.80	0.07
E2	GY	2B	AX.89679762	1.43E+08	0.25	-2.18	3.05	0.05
E2	TKW	2B	AX.89676429	5.94E+08	0.32	0.99	3.16	0.06
E2	GY	2B	AX.89722560	1.84E+08	0.14	-3.11	3.81	0.06
E2	GY	2B	AX.89357145	1.80E+08	0.19	-2.80	4.02	0.06
E2	GY	2B	AX.89552327	1.78E+08	0.20	-3.16	5.03	0.08
E2	GY	2B	AX.89579357	1.83E+08	0.14	-3.11	3.81	0.06
E2	GY	2B	AX.89579357	1.83E+08	0.14	-3.11	3.81	0.06
E2	GY	2B	AX.89677120	1.79E+08	0.20	-2.93	4.55	0.07
E2	GY	2B	AX.89563399	1.84E+08	0.14	-3.11	3.81	0.06
E2	GY	2B	AX.89440441	1.31E+08	0.06	-4.19	3.31	0.04
E2	GY	2B	AX.89440441	1.31E+08	0.06	-4.19	3.31	0.04
E2	GY	2B	AX.89742669	7.57E+08	0.06	-4.29	3.32	0.04
E2	GY	2B	AX.89689770	1.80E+08	0.20	-2.81	4.09	0.07

E2	GY	2B	AX.89689770	1.80E+08	0.20	-2.81	4.09	0.07
E2	GY	2B	AX.89604923	1.78E+08	0.19	-2.80	4.02	0.06
E2	GY	2B	AX.89742961	6.60E+08	0.07	-4.01	3.30	0.04
E2	GY	2B	AX.89452013	1.82E+08	0.14	-3.66	5.11	0.08
E2	GY	2B	AX.89702737	1.78E+08	0.19	-2.80	4.02	0.06
E2	GY	2B	AX.89754603	1.22E+08	0.25	-2.18	3.04	0.05
E2	TKW	2B	AX.89590942	7.43E+08	0.37	1.01	3.43	0.06
E2	GY	2B	AX.89690569	1.78E+08	0.18	-3.40	5.31	0.09
E2	GY	2B	AX.89727447	1.76E+08	0.17	-3.02	4.23	0.07
E2	GY	2B	AX.89699216	1.82E+08	0.11	-3.62	4.24	0.07
E2	GY	2B	AX.89441347	1.79E+08	0.19	-2.80	4.02	0.06
E2	GY	2B	AX.89617519	6.63E+08	0.06	-4.94	4.16	0.06
E2	GY	2B	AX.89666461	6.80E+08	0.06	-4.06	3.17	0.04
E2	GY	2B	AX.89666461	6.80E+08	0.06	-4.06	3.17	0.04
E2	GY	2B	AX.89406830	1.22E+08	0.25	-2.18	3.04	0.05
E2	TKW	2B	AX.89753690	7.44E+08	0.37	0.99	3.38	0.06
E2	GY	2B	AX.89724351	1.82E+08	0.13	-3.57	4.64	0.07
E2	GY	2B	AX.89335901	1.83E+08	0.14	-3.55	4.92	0.08
E2	GY	2B	AX.89407393	1.06E+08	0.08	-3.56	3.29	0.05
E2	GY	2B	AX.89407393	1.06E+08	0.08	-3.56	3.29	0.05
E2	GY	2B	AX.89692003	1.76E+08	0.17	-3.02	4.23	0.07
E2	GY	2B	AX.89336171	5.94E+08	0.06	-4.54	3.80	0.07
E2	GY	2B	AX.89445890	1.79E+08	0.20	-2.93	4.48	0.07
E2	GY	2B	AX.89431286	6.35E+08	0.06	-4.46	3.70	0.05
E2	GY	2B	AX.89431286	6.35E+08	0.06	-4.46	3.70	0.05
E2	TKW	2B	AX.89531301	7.38E+08	0.37	1.01	3.43	0.06
E2	TKW	2B	AX.89625657	7.26E+08	0.40	1.00	3.48	0.06
E2	GY	2B	AX.89713274	7.75E+08	0.08	-4.63	5.04	0.09
E2	GY	2B	AX.89713274	7.75E+08	0.08	-4.63	5.04	0.09
E2	GY	2B	AX.89408291	1.40E+08	0.13	-3.14	3.57	0.06
E2	GY	2B	AX.89630862	1.84E+08	0.14	-3.55	4.92	0.08
E2	GY	2B	AX.89770264	1.83E+08	0.14	-3.55	4.92	0.08
E2	GY	2B	AX.89419977	1.79E+08	0.20	-2.58	3.53	0.05
E2	GY	2B	AX.89619321	1.80E+08	0.19	-2.80	4.02	0.06
E2	GY	2B	AX.89443676	7.58E+08	0.06	-3.99	3.11	0.04
E2	GY	2B	AX.89762396	1.75E+08	0.18	-3.40	5.31	0.09
E2	GY	2B	AX.89762396	1.75E+08	0.18	-3.40	5.31	0.09
E2	GY	2B	AX.89348759	1.78E+08	0.20	-2.58	3.53	0.05
E2	GY	2B	AX.89583382	1.81E+08	0.12	-4.12	5.49	0.09
E2	GY	2B	AX.89373157	1.84E+08	0.14	-3.56	4.86	0.08
E2	GY	2B	AX.89373157	1.84E+08	0.14	-3.56	4.86	0.08
E2	GY	2B	AX.89718685	1.82E+08	0.13	-4.02	5.87	0.09
E2	GY	2B	AX.89710344	7.57E+08	0.07	-4.00	3.30	0.04
E2	GY	2B	AX.89754232	1.30E+08	0.25	-2.20	3.12	0.05
E2	GY	2B	AX.89491567	1.84E+08	0.14	-3.11	3.81	0.06
E2	GY	2B	AX.89338416	7.60E+08	0.06	-3.99	3.11	0.04
E2	GY	2B	AX.89338416	7.60E+08	0.06	-3.99	3.11	0.04
E2	GY	2B	AX.89410146	1.84E+08	0.14	-3.55	4.92	0.08
E2	GY	2B	AX.89664208	6.61E+08	0.06	-5.03	4.29	0.06
E2	GY	2B	AX.89480023	1.81E+08	0.12	-4.12	5.49	0.09

E2	GY	2B	AX.89480023	1.81E+08	0.12	-4.12	5.49	0.09
E2	GY	2B	AX.89483881	1.75E+08	0.18	-3.40	5.31	0.09
E2	GY	2B	AX.89645080	1.23E+08	0.25	-2.20	3.12	0.05
E2	GY	2B	AX.89654194	1.81E+08	0.11	-3.62	4.24	0.07
E2	GPM2	2B	AX.89699129	1.09E+07	0.06	1118.53	3.64	0.07
E2	GY	2B	AX.89375220	1.84E+08	0.14	-3.55	4.92	0.08
E2	GY	2B	AX.89375220	1.84E+08	0.14	-3.55	4.92	0.08
E2	GY	2B	AX.89516909	6.64E+08	0.05	-4.64	3.48	0.05
E2	GY	2B	AX.89516909	6.64E+08	0.05	-4.64	3.48	0.05
E2	GY	2B	AX.89728349	1.84E+08	0.14	-3.11	3.81	0.06
E2	GY	2B	AX.89728349	1.84E+08	0.14	-3.11	3.81	0.06
E2	GY	2B	AX.89539598	1.80E+08	0.19	-2.80	4.02	0.06
E2	GY	2B	AX.89614451	1.83E+08	0.14	-3.11	3.81	0.06
E2	GY	2B	AX.89614451	1.83E+08	0.14	-3.11	3.81	0.06
E2	TKW	2B	AX.89375549	7.39E+08	0.37	0.96	3.20	0.06
E2	GY	2B	AX.89504410	1.83E+08	0.14	-3.11	3.81	0.06
E2	GY	2B	AX.89635417	1.81E+08	0.12	-4.12	5.49	0.09
E2	GY	2B	AX.89547594	1.75E+08	0.17	-3.02	4.23	0.07
E2	GY	2B	AX.89547594	1.75E+08	0.17	-3.02	4.23	0.07
E2	GY	2B	AX.89518028	1.81E+08	0.12	-4.12	5.49	0.09
E2	GY	2B	AX.89376827	5.72E+08	0.16	-2.65	3.23	0.06
E2	GY	2B	AX.89376827	5.72E+08	0.16	-2.65	3.23	0.06
E2	GY	2B	AX.89713743	1.84E+08	0.14	-3.11	3.81	0.06
E2	GY	2B	AX.89647582	1.29E+08	0.06	-4.89	4.26	0.05
E2	GY	2B	AX.89647582	1.29E+08	0.06	-4.89	4.26	0.05
E2	GY	2B	AX.89588768	6.60E+08	0.06	-5.03	4.29	0.06
E2	GY	2B	AX.89588768	6.60E+08	0.06	-5.03	4.29	0.06
E2	GY	2B	AX.89444410	1.81E+08	0.12	-3.18	3.62	0.06
E2	TKW	2B	AX.89573057	7.44E+08	0.36	0.98	3.27	0.06
E2	GY	2B	AX.89459157	1.81E+08	0.12	-3.65	4.60	0.08
E2	GY	2B	AX.89698843	1.83E+08	0.14	-3.55	4.92	0.08
E2	GY	2B	AX.89589315	7.59E+08	0.06	-3.99	3.11	0.04
E2	GY	2B	AX.89589315	7.59E+08	0.06	-3.99	3.11	0.04
E2	GY	2B	AX.89669665	1.82E+08	0.13	-3.57	4.64	0.07
E2	GY	2B	AX.89669665	1.82E+08	0.13	-3.57	4.64	0.07
E2	GY	2B	AX.89684472	1.81E+08	0.12	-3.71	4.72	0.08
E2	GY	2B	AX.89684472	1.81E+08	0.12	-3.71	4.72	0.08
E2	GY	2B	AX.89354288	1.48E+08	0.16	-2.83	3.49	0.05
E2	GY	2B	AX.89354288	1.48E+08	0.16	-2.83	3.49	0.05
E2	GY	2B	AX.89575490	1.81E+08	0.12	-4.12	5.49	0.09
E2	TKW	2B	AX.89326712	7.44E+08	0.38	1.01	3.48	0.06
E2	GY	2B	AX.89760140	1.78E+08	0.19	-2.80	4.02	0.06
E2	GY	2B	AX.89760140	1.78E+08	0.19	-2.80	4.02	0.06
E2	GPM2	2B	AX.89470233	7.74E+08	0.07	-1036.64	3.37	0.06
E2	GY	2B	AX.89686171	1.75E+08	0.17	-3.02	4.23	0.07
E2	GY	2B	AX.89569931	6.62E+08	0.06	-5.79	5.90	0.09
E2	GY	2B	AX.89621714	1.83E+08	0.14	-3.55	4.92	0.08
E2	GY	2B	AX.89621714	1.83E+08	0.14	-3.55	4.92	0.08
E2	GY	2B	AX.89554716	1.76E+08	0.18	-2.77	3.78	0.06
E2	GY	2B	AX.89554716	1.76E+08	0.18	-2.77	3.78	0.06

E2	GY	2B	AX.89626799	1.82E+08	0.11	-3.62	4.24	0.07
E2	GY	2B	AX.89678239	1.81E+08	0.20	-2.71	3.96	0.06
E2	GY	2B	AX.89678239	1.81E+08	0.20	-2.71	3.96	0.06
E2	GY	2B	AX.89578198	7.12E+08	0.06	-4.45	3.52	0.05
E2	GY	2B	AX.89591313	6.60E+08	0.07	-4.73	4.67	0.07
E2	GY	2B	AX.89591313	6.60E+08	0.07	-4.73	4.67	0.07
E2	GY	2B	AX.89638370	1.81E+08	0.24	-2.52	3.81	0.05
E2	GY	2B	AX.89663766	1.43E+08	0.25	-2.20	3.13	0.05
E2	GY	2B	AX.89663766	1.43E+08	0.25	-2.20	3.13	0.05
E2	GY	2B	AX.89676426	1.82E+08	0.13	-3.57	4.64	0.07
E2	GY	2B	AX.89676426	1.82E+08	0.13	-3.57	4.64	0.07
E2	GY	2B	AX.89775125	7.95E+08	0.36	-2.01	3.12	0.05
E2	GY	2B	AX.89737804	1.79E+08	0.20	-3.16	5.03	0.08
E2	GY	2B	AX.89737804	1.79E+08	0.20	-3.16	5.03	0.08
E2	GY	2B	AX.89626847	1.81E+08	0.11	-3.62	4.24	0.07
E2	GY	2B	AX.89626847	1.81E+08	0.11	-3.62	4.24	0.07
E2	GY	2B	AX.89742669	7.57E+08	0.06	-4.29	3.32	0.04
E2	GY	2B	AX.89388929	1.79E+08	0.19	-2.80	4.02	0.06
E2	GY	2B	AX.89388929	1.79E+08	0.19	-2.80	4.02	0.06
E2	GY	2B	AX.89640311	1.81E+08	0.19	-2.80	4.02	0.06
E2	GY	2B	AX.89742961	6.60E+08	0.07	-4.01	3.30	0.04
E2	GY	2B	AX.89702737	1.78E+08	0.19	-2.80	4.02	0.06
E2	GY	2B	AX.89561095	1.79E+08	0.19	-2.80	4.02	0.06
E2	GY	2B	AX.89346764	1.79E+08	0.19	-2.80	4.02	0.06
E2	GY	2B	AX.89346764	1.79E+08	0.19	-2.80	4.02	0.06
E2	GY	2B	AX.89581448	1.01E+08	0.07	-4.38	4.09	0.08
E2	GY	2B	AX.89581448	1.01E+08	0.07	-4.38	4.09	0.08
E2	TKW	2B	AX.89458271	5.94E+08	0.32	0.97	3.09	0.06
E2	GY	2B	AX.89489608	6.60E+08	0.07	-4.07	3.79	0.05
E2	GY	2B	AX.89489608	6.60E+08	0.07	-4.07	3.79	0.05
E2	GY	2B	AX.89445890	1.79E+08	0.20	-2.93	4.48	0.07
E2	GY	2B	AX.89359988	1.78E+08	0.20	-3.16	5.03	0.08
E2	GY	2B	AX.89359988	1.78E+08	0.20	-3.16	5.03	0.08
E2	GY	2B	AX.89740553	1.03E+08	0.07	-4.38	4.09	0.08
E2	GY	2B	AX.89740553	1.03E+08	0.07	-4.38	4.09	0.08
E2	GY	2B	AX.89500920	1.79E+08	0.20	-2.93	4.48	0.07
E2	GY	2B	AX.89500920	1.79E+08	0.20	-2.93	4.48	0.07
E2	GY	2B	AX.89547217	1.81E+08	0.11	-3.62	4.24	0.07
E2	GY	2B	AX.89547217	1.81E+08	0.11	-3.62	4.24	0.07
E2	GY	2B	AX.89595787	1.84E+08	0.14	-3.11	3.81	0.06
E2	GY	2B	AX.89595787	1.84E+08	0.14	-3.11	3.81	0.06
E2	GY	2B	AX.89770264	1.83E+08	0.14	-3.55	4.92	0.08
E2	GY	2B	AX.89348759	1.78E+08	0.20	-2.58	3.53	0.05
E2	GY	2B	AX.89349377	1.81E+08	0.12	-4.12	5.49	0.09
E2	GY	2B	AX.89349377	1.81E+08	0.12	-4.12	5.49	0.09
E2	GY	2B	AX.89644113	1.84E+08	0.12	-3.52	4.09	0.06
E2	GY	2B	AX.89619964	1.78E+08	0.20	-3.16	5.03	0.08
E2	GY	2B	AX.89619964	1.78E+08	0.20	-3.16	5.03	0.08
E2	TKW	2B	AX.89495903	7.34E+08	0.46	0.99	3.56	0.07
E2	GY	2B	AX.89572391	1.82E+08	0.13	-3.57	4.64	0.07

E2	GY	2B	AX.89669239	6.52E+08	0.05	-4.45	3.22	0.04
E2	GY	2B	AX.89669239	6.52E+08	0.05	-4.45	3.22	0.04
E2	GY	2B	AX.89491567	1.84E+08	0.14	-3.11	3.81	0.06
E2	GY	2B	AX.89350199	1.80E+08	0.19	-2.80	4.02	0.06
E2	GY	2B	AX.89350199	1.80E+08	0.19	-2.80	4.02	0.06
E2	GY	2B	AX.89561095	1.79E+08	0.19	-2.80	4.02	0.06
E2	GY	2B	AX.89698976	7.57E+08	0.06	-4.29	3.32	0.04
E2	GY	2B	AX.89534554	6.63E+08	0.05	-5.90	5.31	0.08
E2	GY	2B	AX.89534554	6.63E+08	0.05	-5.90	5.31	0.08
E2	GY	2B	AX.89743777	1.79E+08	0.19	-2.80	4.02	0.06
E2	GY	2B	AX.89743777	1.79E+08	0.19	-2.80	4.02	0.06
E2	TKW	2B	AX.89430844	5.94E+08	0.33	0.96	3.04	0.05
E2	GY	2B	AX.89726785	6.62E+08	0.06	-5.03	4.29	0.06
E2	GY	2B	AX.89726785	6.62E+08	0.06	-5.03	4.29	0.06
E2	GY	2B	AX.89496528	1.54E+08	0.06	-4.16	3.10	0.05
E2	GY	2B	AX.89496528	1.54E+08	0.06	-4.16	3.10	0.05
E2	GY	2B	AX.89728726	6.62E+08	0.06	-5.03	4.29	0.06
E2	GY	2B	AX.89728726	6.62E+08	0.06	-5.03	4.29	0.06
E2	GY	2B	AX.89457015	1.81E+08	0.11	-3.62	4.24	0.07
E2	GY	2B	AX.89457015	1.81E+08	0.11	-3.62	4.24	0.07
E2	GY	2B	AX.89538197	1.80E+08	0.20	-2.91	4.35	0.07
E2	GY	2B	AX.89539598	1.80E+08	0.19	-2.80	4.02	0.06
E2	GY	2B	AX.89717315	1.79E+08	0.20	-2.93	4.48	0.07
E2	TKW	2B	AX.89399434	6.97E+08	0.42	0.95	3.22	0.06
E2	TKW	2B	AX.89380713	7.38E+08	0.37	1.01	3.43	0.06
E2	GY	2B	AX.89504410	1.83E+08	0.14	-3.11	3.81	0.06
E2	GY	2B	AX.89635417	1.81E+08	0.12	-4.12	5.49	0.09
E2	GY	2B	AX.89581629	1.76E+08	0.18	-3.40	5.31	0.09
E2	GY	2B	AX.89581629	1.76E+08	0.18	-3.40	5.31	0.09
E2	GY	2B	AX.89713743	1.84E+08	0.14	-3.11	3.81	0.06
E2	GY	2B	AX.89634349	1.79E+08	0.20	-2.58	3.53	0.05
E2	GY	2B	AX.89634349	1.79E+08	0.20	-2.58	3.53	0.05
E2	GY	2B	AX.89724272	6.62E+08	0.06	-5.79	5.90	0.09
E2	GY	2B	AX.89724272	6.62E+08	0.06	-5.79	5.90	0.09
E2	GY	2B	AX.89424214	1.79E+08	0.19	-2.80	4.02	0.06
E2	GY	2B	AX.89705968	1.26E+08	0.06	-4.89	4.26	0.05
E2	GY	2B	AX.89618666	1.52E+08	0.06	-4.16	3.10	0.05
E2	GY	2B	AX.89459157	1.81E+08	0.12	-3.65	4.60	0.08
E2	GY	2B	AX.89437088	1.22E+08	0.25	-2.20	3.12	0.05
E2	GY	2B	AX.89593250	1.81E+08	0.12	-4.12	5.49	0.09
E2	GY	2B	AX.89405839	1.81E+08	0.12	-4.12	5.49	0.09
E2	TKW	2B	AX.89403372	7.38E+08	0.37	1.01	3.43	0.06
E2	GY	2B	AX.89575490	1.81E+08	0.12	-4.12	5.49	0.09
E2	GY	2B	AX.89706972	6.63E+08	0.06	-4.94	4.16	0.06
E2	GY	2B	AX.89706972	6.63E+08	0.06	-4.94	4.16	0.06
E2	TKW	2B	AX.89440443	7.40E+08	0.37	0.96	3.20	0.06
E2	GY	2B	AX.89425790	1.81E+08	0.24	-2.59	4.06	0.06
E2	GY	2B	AX.89425790	1.81E+08	0.24	-2.59	4.06	0.06
E2	GY	2B	AX.89591171	1.83E+08	0.14	-3.11	3.81	0.06
E2	GY	2B	AX.89591171	1.83E+08	0.14	-3.11	3.81	0.06

E2	TKW	2B	AX.89391193	7.34E+08	0.37	1.01	3.43	0.06
E2	TKW	2B	AX.89349703	7.34E+08	0.46	0.99	3.56	0.07
E2	GY	2B	AX.89438175	5.82E+08	0.06	-4.94	4.12	0.08
E2	GY	2B	AX.89438175	5.82E+08	0.06	-4.94	4.12	0.08
E2	TKW	2B	AX.89593167	7.44E+08	0.37	1.01	3.43	0.06
E2	TKW	2B	AX.89568935	5.94E+08	0.32	0.97	3.09	0.06
E2	GY	2B	AX.89728253	1.79E+08	0.19	-2.80	4.02	0.06
E2	GY	2B	AX.89728253	1.79E+08	0.19	-2.80	4.02	0.06
E2	GY	2B	AX.89658972	1.80E+08	0.20	-3.16	5.03	0.08
E2	GY	2B	AX.89626799	1.82E+08	0.11	-3.62	4.24	0.07
E2	GY	2B	AX.89578198	7.12E+08	0.06	-4.45	3.52	0.05
E2	TKW	2B	AX.89382407	7.34E+08	0.46	0.99	3.56	0.07
E2	GY	2B	AX.89638370	1.81E+08	0.24	-2.52	3.81	0.05
E2	GY	2B	AX.89626420	6.35E+08	0.06	-4.99	4.23	0.05
E2	GY	2B	AX.89775125	7.95E+08	0.36	-2.01	3.12	0.05
E2	GY	2B	AX.89567954	1.81E+08	0.19	-2.80	4.02	0.06
E2	GY	2B	AX.89567954	1.81E+08	0.19	-2.80	4.02	0.06
E2	TKW	2B	AX.89316432	7.37E+08	0.37	1.01	3.43	0.06
E2	TKW	2B	AX.89417089	7.37E+08	0.38	0.94	3.09	0.06
E2	GY	2B	AX.89640311	1.81E+08	0.19	-2.80	4.02	0.06
E2	GY	2B	AX.89604923	1.78E+08	0.19	-2.80	4.02	0.06
E2	GY	2B	AX.89653407	1.76E+08	0.17	-3.02	4.23	0.07
E2	GY	2B	AX.89653407	1.76E+08	0.17	-3.02	4.23	0.07
E2	GY	2B	AX.89593250	1.81E+08	0.12	-4.12	5.49	0.09
E2	GY	2B	AX.89722172	1.75E+08	0.18	-3.40	5.31	0.09
E2	GY	2B	AX.89616947	1.30E+08	0.25	-2.20	3.12	0.05
E2	GY	2B	AX.89616947	1.30E+08	0.25	-2.20	3.12	0.05
E2	GY	2B	AX.89405839	1.81E+08	0.12	-4.12	5.49	0.09
E2	GY	2B	AX.89698976	7.57E+08	0.06	-4.29	3.32	0.04
E2	GY	2B	AX.89699216	1.82E+08	0.11	-3.62	4.24	0.07
E2	TKW	2B	AX.89661546	5.94E+08	0.32	0.99	3.16	0.06
E2	TKW	2B	AX.89344400	7.39E+08	0.37	0.96	3.20	0.06
E2	TKW	2B	AX.89652950	5.94E+08	0.32	0.99	3.16	0.06
E2	GY	2B	AX.89724351	1.82E+08	0.13	-3.57	4.64	0.07
E2	TKW	2B	AX.89723481	5.94E+08	0.33	0.96	3.04	0.05
E2	TKW	2B	AX.89398799	6.08E+08	0.37	1.11	4.08	0.08
E2	TKW	2B	AX.89352050	7.41E+08	0.37	0.96	3.20	0.06
E2	GY	2B	AX.89630862	1.84E+08	0.14	-3.55	4.92	0.08
E2	GY	2B	AX.89419977	1.79E+08	0.20	-2.58	3.53	0.05
E2	TKW	2B	AX.89725518	7.39E+08	0.37	0.96	3.20	0.06
E2	GY	2B	AX.89540932	6.62E+08	0.06	-5.03	4.29	0.06
E2	GY	2B	AX.89396912	1.02E+08	0.07	-4.38	4.09	0.08
E2	GY	2B	AX.89396912	1.02E+08	0.07	-4.38	4.09	0.08
E2	GY	2B	AX.89572391	1.82E+08	0.13	-3.57	4.64	0.07
E2	TKW	2B	AX.89735631	6.02E+08	0.38	1.07	3.87	0.07
E2	TKW	2B	AX.89515639	5.95E+08	0.32	1.02	3.33	0.06
E2	TKW	2B	AX.89761668	5.94E+08	0.32	0.99	3.16	0.06
E2	TKW	2B	AX.89497472	7.44E+08	0.37	0.97	3.24	0.06
E2	GY	2B	AX.89765084	1.81E+08	0.12	-4.12	5.49	0.09
E2	GY	2B	AX.89538197	1.80E+08	0.20	-2.91	4.35	0.07

E2	GY	2B	AX.89577826	1.76E+08	0.17	-3.02	4.23	0.07
E2	GPS	2B	AX.89409931	6.01E+08	0.46	1.37	3.22	0.05
E2	TKW	2B	AX.89572815	7.33E+08	0.18	1.17	3.02	0.05
E2	GY	2B	AX.89540301	5.76E+08	0.05	-5.83	4.80	0.10
E2	TKW	2B	AX.89563683	1.89E+07	0.37	0.96	3.15	0.06
E2	GY	2B	AX.89623578	1.79E+08	0.20	-2.58	3.53	0.05
E2	GY	2B	AX.89563399	1.84E+08	0.14	-3.11	3.81	0.06
E2	GY	2B	AX.89705968	1.26E+08	0.06	-4.89	4.26	0.05
E2	TKW	2B	AX.89351738	6.74E+08	0.34	1.00	3.28	0.06
E2	GY	2B	AX.89618666	1.52E+08	0.06	-4.16	3.10	0.05
E2	GY	2B	AX.89719069	7.57E+08	0.06	-3.99	3.11	0.04
E2	TKW	2B	AX.89686633	5.94E+08	0.32	0.97	3.09	0.06
E2	GY	2B	AX.89541690	1.78E+08	0.19	-2.80	4.02	0.06
E2	GY	2B	AX.89541690	1.78E+08	0.19	-2.80	4.02	0.06
E2	GY	2B	AX.89734860	1.81E+08	0.11	-3.62	4.24	0.07
E2	GY	2B	AX.89664208	6.61E+08	0.06	-5.03	4.29	0.06
E2	TKW	2B	AX.89679254	7.33E+08	0.47	0.92	3.14	0.06
E2	TKW	2B	AX.89372916	7.41E+08	0.37	0.96	3.20	0.06
E2	GY	2B	AX.89449676	1.84E+08	0.14	-2.71	3.01	0.04
E2	GY	2B	AX.89661751	6.36E+08	0.06	-5.72	5.80	0.08
E2	GY	2B	AX.89696261	5.94E+08	0.06	-4.54	3.80	0.07
E2	TKW	2B	AX.89553116	7.37E+08	0.37	1.01	3.43	0.06
E2	GY	2B	AX.89658972	1.80E+08	0.20	-3.16	5.03	0.08
E2	GY	2B	AX.89775110	1.29E+08	0.06	-4.89	4.26	0.05
E2	GPM2	2B	AX.89521003	9.66E+05	0.08	-1094.80	4.07	0.07
E2	GY	2B	AX.89626420	6.35E+08	0.06	-4.99	4.23	0.05
E2	GY	2B	AX.89729590	1.83E+08	0.14	-3.11	3.81	0.06
E2	TKW	2B	AX.89739809	7.40E+08	0.37	0.96	3.20	0.06
E2	GY	2B	AX.89722172	1.75E+08	0.18	-3.40	5.31	0.09
E2	TKW	2B	AX.89383653	6.02E+08	0.38	1.07	3.87	0.07
E2	GY	2B	AX.89511866	1.29E+08	0.25	-2.20	3.12	0.05
E2	GY	2B	AX.89609412	1.81E+08	0.24	-2.59	4.06	0.06
E2	GY	2B	AX.89476521	1.82E+08	0.12	-4.12	5.49	0.09
E2	GY	2B	AX.89476521	1.82E+08	0.12	-4.12	5.49	0.09
E2	GY	2B	AX.89637821	1.22E+08	0.25	-2.20	3.12	0.05
E2	GY	2B	AX.89637821	1.22E+08	0.25	-2.20	3.12	0.05
E2	GY	2B	AX.89729590	1.83E+08	0.14	-3.11	3.81	0.06
E2	TKW	2B	AX.89610946	7.44E+08	0.39	0.98	3.37	0.06
E2	GY	2B	AX.89708086	1.84E+08	0.14	-3.11	3.81	0.06
E2	GY	2B	AX.89540932	6.62E+08	0.06	-5.03	4.29	0.06
E2	GY	2B	AX.89644113	1.84E+08	0.12	-3.52	4.09	0.06
E2	GY	2B	AX.89765862	6.60E+08	0.06	-5.03	4.29	0.06
E2	GY	2B	AX.89720671	1.79E+08	0.20	-3.16	5.03	0.08
E2	GY	2B	AX.89696341	7.59E+08	0.06	-3.99	3.11	0.04
E2	GY	2B	AX.89511866	1.29E+08	0.25	-2.20	3.12	0.05
E2	GY	2B	AX.89483881	1.75E+08	0.18	-3.40	5.31	0.09
E2	GY	2B	AX.89765752	1.78E+08	0.19	-2.80	4.02	0.06
E2	GY	2B	AX.89608525	1.04E+08	0.07	-4.38	4.09	0.08
E2	TKW	2B	AX.89438967	7.42E+08	0.38	0.93	3.06	0.05
E2	TKW	2B	AX.89616056	5.94E+08	0.32	0.97	3.09	0.06

E2	GY	2B	AX.89696261	5.94E+08	0.06	-4.54	3.80	0.07
E2	GY	2B	AX.89614104	6.59E+08	0.06	-5.03	4.29	0.06
E2	GY	2B	AX.89494298	7.60E+08	0.06	-3.99	3.11	0.04
E2	GY	2B	AX.89494298	7.60E+08	0.06	-3.99	3.11	0.04
E2	GY	2B	AX.89540301	5.76E+08	0.05	-5.83	4.80	0.10
E2	TKW	2B	AX.89510700	7.39E+08	0.37	0.96	3.20	0.06
E2	GY	2B	AX.89696341	7.59E+08	0.06	-3.99	3.11	0.04
E2	TKW	2B	AX.89634429	7.41E+08	0.39	0.92	3.03	0.05
E2	TKW	2B	AX.89769642	6.74E+08	0.34	1.00	3.28	0.06
E2	TKW	2B	AX.89462410	7.38E+08	0.38	1.01	3.47	0.06
E2	TKW	2B	AX.89451766	7.26E+08	0.40	1.02	3.65	0.07
E2	GY	2B	AX.89721075	1.03E+08	0.07	-4.38	4.09	0.08
E2	TKW	2B	AX.89406404	7.34E+08	0.46	1.01	3.63	0.07
E2	GY	2B	AX.89681803	7.59E+08	0.06	-4.29	3.32	0.04
E2	TKW	2B	AX.89470461	7.39E+08	0.37	0.96	3.20	0.06
E2	GY	2B	AX.89775110	1.29E+08	0.06	-4.89	4.26	0.05
E2	GY	2B	AX.89614104	6.59E+08	0.06	-5.03	4.29	0.06
E2	GPS	2B	AX.89374881	6.60E+08	0.17	-1.78	3.14	0.05
E2	TKW	2B	AX.89418966	7.98E+08	0.05	-2.05	3.06	0.05
E2	GY	2B	AX.89577826	1.76E+08	0.17	-3.02	4.23	0.07
E2	TKW	2B	AX.89340885	5.94E+08	0.32	0.98	3.10	0.06
E2	GY	2B	AX.89765862	6.60E+08	0.06	-5.03	4.29	0.06
E2	GY	2B	AX.89609412	1.81E+08	0.24	-2.59	4.06	0.06
E2	GY	2B	AX.89681803	7.59E+08	0.06	-4.29	3.32	0.04
E2	TKW	2B	AX.89333155	7.38E+08	0.35	1.08	3.78	0.07
E2	TKW	2B	AX.89607622	7.26E+08	0.40	1.01	3.54	0.06
E2	TKW	2B	AX.89696530	3.83E+07	0.15	1.57	4.52	0.07
E2	GY	2B	AX.89608525	1.04E+08	0.07	-4.38	4.09	0.08
E2	GY	2B	AX.89449676	1.84E+08	0.14	-2.71	3.01	0.04
E2	TKW	2B	AX.89630814	7.26E+08	0.40	0.93	3.10	0.06
E2	GY	2B	AX.89717315	1.79E+08	0.20	-2.93	4.48	0.07
E2	TKW	2B	AX.89560674	6.73E+08	0.32	0.97	3.08	0.05
E2	GY	2B	AX.89720671	1.79E+08	0.20	-3.16	5.03	0.08
E2	TKW	2B	AX.89329425	6.73E+08	0.34	1.00	3.28	0.06
E2	TKW	2B	AX.89534242	7.42E+08	0.18	-1.16	3.01	0.05
E2	GY	2B	AX.89661751	6.36E+08	0.06	-5.72	5.80	0.08
E2	GY	2B	AX.89765752	1.78E+08	0.19	-2.80	4.02	0.06
E2	GY	2B	AX.89721075	1.03E+08	0.07	-4.38	4.09	0.08
E2	TKW	2B	AX.89453246	7.41E+08	0.38	0.95	3.14	0.06
E2	TKW	2B	AX.89503580	7.43E+08	0.37	1.01	3.43	0.06
E2	GY	2B	AX.89673819	9.13E+06	0.45	-1.98	3.16	0.07
E2	GY	2B	AX.89673819	9.13E+06	0.45	-1.98	3.16	0.07
E2	TKW	2B	AX.89644872	7.38E+08	0.37	1.02	3.53	0.06
E2	GY	2B	AX.89708086	1.84E+08	0.14	-3.11	3.81	0.06
E2	TKW	2B	AX.89585973	6.15E+08	0.33	0.96	3.03	0.05
E2	GPS	2B	AX.89774753	6.60E+08	0.16	-1.81	3.04	0.05
E3	GPM2	2B	AX.89501127	3.70E+07	0.34	964.84	3.47	0.08
E3	GPM2	2B	AX.89557087	4.74E+07	0.21	-1062.76	3.27	0.08
E3	GPM2	2B	AX.89743740	6.86E+07	0.10	1621.42	3.79	0.05
E3	GPM2	2B	AX.89769577	6.86E+07	0.10	1621.42	3.79	0.05

E3	GPM2	2B	AX.89370705	6.86E+07	0.08	2009.46	4.65	0.05
E3	GPM2	2B	AX.89503857	6.88E+07	0.10	1591.03	3.78	0.05
E3	GPS	2B	AX.89397447	4.93E+07	0.14	-2.74	3.04	0.03
E3	GPS	2B	AX.89718064	6.38E+07	0.42	1.99	3.17	0.07
E3	GPS	2B	AX.89673606	7.46E+08	0.38	2.15	3.50	0.08
E3	GPS	2B	AX.89572765	7.46E+08	0.45	1.99	3.19	0.08
E3	GPS	2B	AX.89634826	7.46E+08	0.40	2.19	3.61	0.09
E3	GPS	2B	AX.89601989	7.46E+08	0.40	2.01	3.18	0.09
E3	GPS	2B	AX.89382399	7.46E+08	0.40	2.01	3.18	0.09
E3	GY	2B	AX.89501127	3.70E+07	0.34	3.77	4.56	0.11
E3	GY	2B	AX.89560158	6.82E+07	0.10	4.78	3.15	0.02
E3	GY	2B	AX.89743740	6.86E+07	0.10	5.63	4.06	0.03
E3	GY	2B	AX.89769577	6.86E+07	0.10	5.63	4.06	0.03
E3	GY	2B	AX.89370705	6.86E+07	0.08	6.32	4.17	0.04
E3	GY	2B	AX.89503857	6.88E+07	0.10	5.58	4.12	0.03
E3	GY	2B	AX.89550407	1.54E+08	0.40	2.86	3.15	0.01
E3	GY	2B	AX.89500028	1.54E+08	0.39	2.95	3.33	0.02
E3	GY	2B	AX.89605601	1.54E+08	0.39	2.95	3.33	0.02
E3	SPM2	2B	AX.89400127	7.07E+08	0.18	25.48	3.26	0.04
E3	SPM2	2B	AX.89715665	7.07E+08	0.19	24.14	3.14	0.04
E3	SPM2	2B	AX.89577125	7.07E+08	0.17	27.75	3.75	0.05
E3	SPM2	2B	X.89424732_OT	7.08E+08	0.18	24.63	3.19	0.04
E3	SPM2	2B	AX.89485058	7.08E+08	0.13	29.10	3.21	0.04
E3	SPM2	2B	AX.89630235	7.08E+08	0.13	29.10	3.21	0.04
E3	TKW	2B	AX.89597425	2.54E+08	0.33	1.01	3.28	0.11
E3	TKW	2B	AX.89756391	2.92E+08	0.22	-1.08	3.50	0.05
E3	TKW	2B	AX.89737535	7.49E+08	0.09	1.63	4.06	0.10
E3	TKW	2B	AX.89313663	7.49E+08	0.13	1.21	3.17	0.08
E3	TKW	2B	AX.89324477	7.50E+08	0.14	1.31	3.71	0.08
E3	TKW	2B	AX.89696490	7.50E+08	0.14	1.28	3.53	0.08
E3	TKW	2B	AX.89717881	7.52E+08	0.17	1.08	3.21	0.06
E3	TKW	2B	X.89532801_OT	7.94E+08	0.43	0.90	3.69	0.05
E3	SPM2	2B	AX.89733515	7.33E+08	0.05	44.37	3.23	0.07
E3	SPM2	2B	AX.89641106	7.33E+08	0.04	69.95	5.58	0.12
E3	SPM2	2B	AX.89329299	7.33E+08	0.05	55.78	4.47	0.10
E3	SPM2	2B	AX.89351333	7.34E+08	0.04	64.11	5.20	0.11
E3	SPM2	2B	AX.89637930	7.34E+08	0.05	57.33	4.97	0.11
E3	SPM2	2B	AX.89521802	7.34E+08	0.05	65.91	5.94	0.12
E3	SPM2	2B	AX.89462422	7.34E+08	0.04	66.21	5.57	0.12
E3	SPM2	2B	AX.89708076	7.34E+08	0.06	52.14	4.65	0.10
E3	SPM2	2B	AX.89695677	7.71E+08	0.13	29.10	3.21	0.04
E3	SPM2	2B	AX.89439095	7.33E+08	0.05	49.36	3.84	0.09
E3	SPM2	2B	AX.89656537	7.33E+08	0.05	49.36	3.84	0.09
E3	SPM2	2B	AX.89513784	7.33E+08	0.05	49.36	3.84	0.09
E3	SPM2	2B	AX.89432828	7.33E+08	0.04	64.11	5.20	0.11
E3	SPM2	2B	AX.89556473	7.29E+08	0.05	44.69	3.06	0.07
E3	SPM2	2B	AX.89384448	7.29E+08	0.04	48.11	3.23	0.08
E3	SPM2	2B	AX.89401062	7.33E+08	0.04	54.84	3.63	0.07
E3	SPM2	2B	AX.89314901	7.33E+08	0.04	69.95	5.58	0.12
E3	SPM2	2B	AX.89331823	7.33E+08	0.04	64.11	5.20	0.11

E3	SPM2	2B	AX.89497538	7.33E+08	0.05	64.77	5.75	0.13
E4	GPM2	2B	AX.89478758	4.99E+06	0.29	-475.86	3.22	0.03
E4	GPM2	2B	AX.89557087	4.74E+07	0.21	-580.11	3.56	0.07
E4	GPM2	2B	AX.89414821	7.46E+08	0.48	-457.04	3.50	0.04
E4	GPM2	2B	X.89454275_OT	1.59E+08	0.14	-628.66	3.29	0.06
E4	GPM2	2B	AX.89694845	1.59E+08	0.14	-642.90	3.35	0.06
E4	GPM2	2B	AX.89771289	5.83E+08	0.07	-879.23	3.17	0.05
E4	GPM2	2B	AX.89356824	5.84E+08	0.07	-879.23	3.17	0.05
E4	GPM2	2B	AX.89636518	5.84E+08	0.07	-879.23	3.17	0.05
E4	GPM2	2B	AX.89727255	7.46E+08	0.27	498.16	3.23	0.04
E4	GPM2	2B	AX.89656453	5.83E+08	0.07	-879.23	3.17	0.05
E4	GPM2	2B	AX.89437010	5.83E+08	0.07	-879.23	3.17	0.05
E4	GPS	2B	AX.89748791	5.74E+07	0.46	1.50	3.13	0.08
E4	GPS	2B	AX.89718064	6.38E+07	0.38	1.48	3.04	0.08
E4	GPS	2B	AX.89392286	2.62E+07	0.11	2.42	3.41	0.03
E4	GPS	2B	AX.89597712	2.63E+07	0.11	2.32	3.24	0.03
E4	GPS	2B	AX.89449728	2.01E+08	0.12	2.36	3.27	0.04
E4	GPS	2B	AX.89673482	5.43E+08	0.32	-1.66	3.48	0.06
E4	GPS	2B	AX.89537726	6.79E+08	0.29	1.66	3.37	0.05
E4	GPS	2B	AX.89665560	7.67E+08	0.46	1.63	3.56	0.09
E4	GY	2B	X.89454275_OT	1.59E+08	0.15	-2.37	3.99	0.06
E4	GY	2B	AX.89694845	1.59E+08	0.14	-2.32	3.78	0.05
E4	GY	2B	X.89394396_OT	2.91E+08	0.08	-3.19	4.21	0.06
E4	GY	2B	X.89531585_OT	2.91E+08	0.08	-2.90	3.66	0.05
E4	GY	2B	X.89442099_OT	2.91E+08	0.08	-3.28	4.24	0.06
E4	GY	2B	X.89532001_OT	2.91E+08	0.08	-2.90	3.66	0.05
E4	GY	2B	X.89513538_OT	2.93E+08	0.08	-3.19	4.21	0.06
E4	GY	2B	X.89729857_OT	2.93E+08	0.08	-3.07	3.82	0.05
E4	GY	2B	X.89394632_OT	2.94E+08	0.08	-3.19	4.21	0.06
E4	GY	2B	AX.89729008	2.96E+08	0.06	-2.95	3.13	0.05
E4	GY	2B	AX.89450946	2.96E+08	0.10	-2.59	3.33	0.05
E4	GY	2B	X.89327061_OT	2.97E+08	0.08	-3.34	4.46	0.06
E4	GY	2B	X.89489380_OT	2.97E+08	0.08	-3.19	4.21	0.06
E4	GY	2B	X.89406077_OT	2.97E+08	0.08	-3.19	4.21	0.06
E4	GY	2B	X.89619907_OT	2.98E+08	0.08	-3.19	4.21	0.06
E4	GY	2B	AX.89459997	3.54E+08	0.09	-2.86	3.71	0.04
E4	GY	2B	AX.89566564	3.59E+08	0.08	-3.28	4.25	0.05
E4	GY	2B	AX.89484994	4.06E+08	0.07	-3.77	5.13	0.07
E4	GY	2B	AX.89656453	5.83E+08	0.07	-3.03	3.34	0.05
E4	GY	2B	AX.89437010	5.83E+08	0.07	-3.03	3.34	0.05
E4	GY	2B	AX.89771289	5.83E+08	0.07	-3.03	3.34	0.05
E4	GY	2B	AX.89356824	5.84E+08	0.07	-3.03	3.34	0.05
E4	GY	2B	AX.89636518	5.84E+08	0.07	-3.03	3.34	0.05
E4	GY	2B	AX.89352390	5.84E+08	0.10	-2.45	3.11	0.05
E4	GY	2B	AX.89592898	5.84E+08	0.10	-2.45	3.11	0.05
E4	GY	2B	AX.89363506	5.84E+08	0.07	-2.89	3.23	0.05
E4	GY	2B	AX.89517302	6.01E+08	0.09	-2.69	3.21	0.05
E4	GY	2B	AX.89533752	6.01E+08	0.08	-2.80	3.27	0.05
E4	GY	2B	AX.89641491	6.01E+08	0.08	-3.08	3.94	0.06
E4	GY	2B	AX.89522387	7.18E+08	0.22	-1.89	3.41	0.05

E4	GY	2B	AX.89727255	7.46E+08	0.27	1.66	3.11	0.05
E4	GY	2B	AX.89316979	7.65E+08	0.06	-3.02	3.17	0.04
E4	GY	2B	AX.89413543	3.39E+07	0.37	-1.52	3.15	0.04
E4	GY	2B	AX.89418752	1.46E+08	0.13	-2.39	3.81	0.05
E4	GY	2B	AX.89341322	1.47E+08	0.16	-1.99	3.16	0.04
E4	GY	2B	AX.89762335	1.47E+08	0.08	-3.08	4.04	0.05
E4	SPM2	2B	AX.89613304	1.66E+08	0.49	15.42	3.02	0.05
E4	SPM2	2B	X.89612547_OT	2.44E+08	0.10	26.76	3.10	0.05
E4	SPM2	2B	AX.89489368	4.91E+08	0.09	28.24	3.06	0.04
E4	SPM2	2B	AX.89396236	5.32E+08	0.08	30.90	3.32	0.04
E4	SPM2	2B	AX.89340804	5.32E+08	0.08	30.90	3.32	0.04
E4	SPM2	2B	AX.89503205	5.32E+08	0.08	30.90	3.32	0.04
E4	SPM2	2B	AX.89653530	5.32E+08	0.08	30.90	3.32	0.04
E4	SPM2	2B	AX.89761213	5.32E+08	0.08	30.90	3.32	0.04
E4	SPM2	2B	AX.89718643	5.33E+08	0.08	30.90	3.32	0.04
E4	SPM2	2B	AX.89541750	5.34E+08	0.08	30.90	3.32	0.04
E4	SPM2	2B	AX.89734593	5.34E+08	0.08	30.90	3.32	0.04
E4	SPM2	2B	AX.89660308	5.35E+08	0.07	30.15	3.08	0.04
E4	SPM2	2B	AX.89543079	5.35E+08	0.08	30.90	3.32	0.04
E4	SPM2	2B	AX.89543564	5.37E+08	0.08	30.90	3.32	0.04
E4	SPM2	2B	AX.89350734	5.37E+08	0.08	30.90	3.32	0.04
E4	SPM2	2B	AX.89615959	5.37E+08	0.08	30.90	3.32	0.04
E4	SPM2	2B	AX.89777658	5.37E+08	0.08	30.90	3.32	0.04
E4	SPM2	2B	AX.89691553	5.37E+08	0.08	30.90	3.32	0.04
E4	SPM2	2B	AX.89591952	5.38E+08	0.08	30.90	3.32	0.04
E4	SPM2	2B	AX.89411246	5.38E+08	0.07	30.15	3.08	0.04
E4	SPM2	2B	AX.89673482	5.43E+08	0.32	21.52	4.73	0.08
E4	SPM2	2B	AX.89321845	7.53E+08	0.12	24.49	3.25	0.05
E1	SPM2	2D	AX.89348011	5.65E+08	0.47	-19.15	4.03	0.07
E1	GY	2D	AX.89380230	6.20E+08	0.06	-4.77	3.08	0.05
E1	GY	2D	AX.89382235	3.50E+07	0.49	-2.65	4.00	0.08
E1	GY	2D	AX.89496787	5.01E+08	0.13	-4.13	4.24	0.08
E1	GY	2D	AX.89489393	5.42E+08	0.16	-3.42	3.62	0.06
E1	GY	2D	AX.89496787	5.01E+08	0.13	-4.13	4.24	0.08
E1	GY	2D	AX.89519128	4.15E+08	0.46	2.36	3.27	0.05
E1	GY	2D	AX.89380230	6.20E+08	0.06	-4.77	3.08	0.05
E1	GY	2D	AX.89382235	3.50E+07	0.49	-2.65	4.00	0.08
E1	GY	2D	AX.89519320	6.21E+08	0.06	-4.77	3.08	0.05
E1	GY	2D	AX.89646023	3.40E+07	0.28	-3.14	4.50	0.07
E1	GY	2D	AX.89491550	6.11E+08	0.31	-2.54	3.26	0.05
E1	GY	2D	AX.89489393	5.42E+08	0.16	-3.42	3.62	0.06
E1	GY	2D	AX.89491550	6.11E+08	0.31	-2.54	3.26	0.05
E1	GY	2D	AX.89519128	4.15E+08	0.46	2.36	3.27	0.05
E1	GY	2D	AX.89415495	6.21E+08	0.06	-4.77	3.08	0.05
E1	GY	2D	AX.89415495	6.21E+08	0.06	-4.77	3.08	0.05
E1	GY	2D	AX.89519320	6.21E+08	0.06	-4.77	3.08	0.05
E1	GPM2	2D	AX.89419786	1.65E+08	0.37	677.37	3.71	0.07
E1	GY	2D	AX.89646023	3.40E+07	0.28	-3.14	4.50	0.07
E1	GPM2	2D	AX.89496787	5.01E+08	0.13	-874.12	3.02	0.05
E1	GPM2	2D	AX.89519128	4.15E+08	0.46	627.72	3.44	0.06

E1	GPM2	2D	AX.89673053	8.21E+07	0.26	-682.59	3.17	0.05
E1	TKW	2D	AX.89350526	7.74E+07	0.33	0.98	3.64	0.06
E1	TKW	2D	AX.89433606	7.74E+07	0.33	0.98	3.64	0.06
E1	TKW	2D	AX.89584589	5.79E+08	0.34	0.92	3.27	0.06
E1	TKW	2D	AX.89533726	5.71E+08	0.36	1.04	4.14	0.07
E1	TKW	2D	AX.89357550	7.74E+07	0.35	0.93	3.36	0.06
E1	TKW	2D	AX.89556429	7.70E+07	0.36	0.90	3.27	0.06
E1	TKW	2D	AX.89776110	7.73E+07	0.35	0.93	3.36	0.06
E1	TKW	2D	AX.89612783	6.09E+08	0.43	0.85	3.08	0.05
E1	TKW	2D	AX.89455622	7.74E+07	0.33	0.98	3.64	0.06
E1	TKW	2D	AX.89431879	7.73E+07	0.35	0.93	3.41	0.06
E1	TKW	2D	AX.89766573	7.71E+07	0.36	0.90	3.27	0.06
E1	TKW	2D	AX.89596235	7.72E+07	0.35	0.93	3.36	0.06
E1	TKW	2D	AX.89673053	8.21E+07	0.26	1.11	3.96	0.07
E1	TKW	2D	AX.89329392	7.70E+07	0.36	0.90	3.27	0.06
E1	TKW	2D	AX.89533458	7.74E+07	0.34	1.00	3.76	0.07
E1	TKW	2D	AX.89557747	7.71E+07	0.36	0.90	3.27	0.06
E1	TKW	2D	AX.89566420	7.70E+07	0.36	0.90	3.27	0.06
E1	TKW	2D	AX.89663377	7.74E+07	0.33	1.05	4.07	0.07
E1	TKW	2D	AX.89356326	7.74E+07	0.33	1.05	4.07	0.07
E1	TKW	2D	AX.89738253	7.74E+07	0.33	1.05	4.07	0.07
E1	TKW	2D	AX.89466378	7.71E+07	0.35	0.93	3.36	0.06
E1	TKW	2D	AX.89684462	5.94E+08	0.39	0.90	3.31	0.06
E1	TKW	2D	AX.89684771	7.72E+07	0.35	0.93	3.36	0.06
E1	TKW	2D	AX.89596220	7.70E+07	0.36	0.90	3.27	0.06
E1	TKW	2D	AX.89671873	7.71E+07	0.36	0.90	3.27	0.06
E1	TKW	2D	AX.89506063	7.74E+07	0.33	1.05	4.07	0.07
E1	TKW	2D	AX.89580589	5.84E+08	0.37	0.94	3.51	0.06
E1	TKW	2D	AX.89574114	7.74E+07	0.33	0.98	3.64	0.06
E1	TKW	2D	AX.89614392	7.74E+07	0.33	0.88	3.02	0.05
E1	TKW	2D	AX.89457560	8.18E+07	0.46	-0.86	3.21	0.06
E1	TKW	2D	AX.89722081	7.66E+07	0.35	0.91	3.22	0.06
E1	TKW	2D	AX.89564320	7.74E+07	0.33	0.98	3.64	0.06
E1	TKW	2D	AX.89703856	7.70E+07	0.36	0.90	3.27	0.06
E1	TKW	2D	AX.89584377	7.74E+07	0.33	0.95	3.44	0.06
E1	TKW	2D	AX.89453418	7.73E+07	0.35	0.93	3.36	0.06
E1	TKW	2D	AX.89327593	7.74E+07	0.35	0.93	3.36	0.06
E1	TKW	2D	AX.89515222	7.74E+07	0.33	0.98	3.64	0.06
E1	TKW	2D	AX.89447235	7.74E+07	0.34	1.00	3.76	0.07
E1	TKW	2D	AX.89700060	8.19E+07	0.24	1.17	4.14	0.07
E1	TKW	2D	AX.89359112	7.70E+07	0.36	0.90	3.27	0.06
E1	TKW	2D	AX.89582111	7.71E+07	0.36	0.90	3.27	0.06
E1	TKW	2D	AX.89623305	7.83E+07	0.24	1.00	3.12	0.05
E1	TKW	2D	AX.89733080	7.74E+07	0.33	0.98	3.64	0.06
E1	TKW	2D	AX.89510886	6.09E+08	0.34	0.88	3.03	0.05
E1	TKW	2D	AX.89644060	7.69E+07	0.36	0.90	3.27	0.06
E1	TKW	2D	AX.89493758	7.73E+07	0.35	0.93	3.36	0.06
E1	TKW	2D	AX.89489393	5.42E+08	0.16	-1.20	3.30	0.05
E1	TKW	2D	AX.89464875	7.69E+07	0.36	0.90	3.27	0.06
E1	TKW	2D	AX.89775034	7.73E+07	0.34	0.94	3.42	0.06

E1	TKW	2D	AX.89724371	5.86E+08	0.38	0.94	3.56	0.06
E2	SPM2	2D	AX.89438279	7.73E+07	0.20	15.33	3.27	0.06
E2	SPM2	2D	AX.89518048	6.00E+08	0.19	15.83	3.40	0.06
E2	SPM2	2D	AX.89402432	1.83E+08	0.07	25.80	3.54	0.06
E2	GY	2D	AX.89415495	6.21E+08	0.06	-3.99	3.12	0.04
E2	GY	2D	AX.89415495	6.21E+08	0.06	-3.99	3.12	0.04
E2	GY	2D	AX.89380230	6.20E+08	0.06	-3.99	3.12	0.04
E2	GY	2D	AX.89380230	6.20E+08	0.06	-3.99	3.12	0.04
E2	GY	2D	AX.89452098	5.22E+08	0.27	-2.26	3.33	0.05
E2	GY	2D	AX.89452098	5.22E+08	0.27	-2.26	3.33	0.05
E2	GPS	2D	AX.89671885	8.11E+07	0.22	1.63	3.11	0.05
E2	GY	2D	AX.89496787	5.01E+08	0.13	-4.10	5.79	0.10
E2	TKW	2D	AX.89646023	3.40E+07	0.29	-1.13	3.78	0.07
E2	GY	2D	AX.89519320	6.21E+08	0.06	-3.99	3.12	0.04
E2	GPM2	2D	AX.89596012	9.88E+06	0.20	678.57	3.69	0.03
E2	GPS	2D	AX.89596012	9.88E+06	0.20	2.11	4.60	0.06
E2	GY	2D	AX.89519320	6.21E+08	0.06	-3.99	3.12	0.04
E2	GY	2D	AX.89496787	5.01E+08	0.13	-4.10	5.79	0.10
E2	TKW	2D	AX.89371003	3.71E+07	0.32	1.00	3.21	0.06
E2	TKW	2D	AX.89612783	6.09E+08	0.43	0.90	3.00	0.06
E2	TKW	2D	AX.89458193	6.09E+08	0.38	0.98	3.32	0.06
E2	GPM2	2D	AX.89496787	5.01E+08	0.13	-756.26	3.26	0.06
E2	TKW	2D	AX.89382235	3.50E+07	0.49	-1.09	4.15	0.08
E2	TKW	2D	AX.89729447	6.40E+08	0.50	-0.92	3.17	0.06
E2	TKW	2D	AX.89491348	3.23E+07	0.38	-1.00	3.50	0.07
E3	TKW	2D	AX.89525510	2.09E+07	0.42	1.16	3.81	0.21
E3	TKW	2D	AX.89366600	2.09E+07	0.42	1.20	4.02	0.22
E3	TKW	2D	AX.89506509	2.09E+07	0.42	1.17	3.89	0.22
E3	TKW	2D	AX.89466007	2.09E+07	0.42	1.20	4.02	0.22
E3	TKW	2D	AX.89332793	2.09E+07	0.42	1.18	3.94	0.22
E3	TKW	2D	AX.89693001	2.09E+07	0.42	1.18	3.96	0.22
E3	TKW	2D	AX.89389022	2.09E+07	0.42	1.15	3.76	0.21
E3	TKW	2D	AX.89736189	2.09E+07	0.42	1.16	3.81	0.21
E3	TKW	2D	AX.89390334	2.09E+07	0.42	1.16	3.81	0.21
E3	TKW	2D	AX.89547695	2.09E+07	0.42	1.16	3.81	0.21
E3	TKW	2D	AX.89410391	2.09E+07	0.42	1.16	3.81	0.21
E3	TKW	2D	AX.89573403	2.09E+07	0.42	1.16	3.81	0.21
E3	TKW	2D	AX.89685659	2.09E+07	0.43	1.13	3.60	0.21
E3	TKW	2D	AX.89524929	2.10E+07	0.42	1.15	3.76	0.21
E3	TKW	2D	AX.89320828	2.10E+07	0.42	1.15	3.76	0.21
E3	TKW	2D	AX.89630175	2.10E+07	0.41	1.02	3.04	0.20
E3	TKW	2D	AX.89571653	2.10E+07	0.41	1.02	3.04	0.20
E3	TKW	2D	AX.89450500	2.10E+07	0.41	1.02	3.04	0.20
E3	TKW	2D	AX.89769026	2.10E+07	0.42	1.10	3.47	0.21
E3	TKW	2D	AX.89536557	2.10E+07	0.42	1.10	3.47	0.21
E3	TKW	2D	AX.89347123	2.10E+07	0.41	1.02	3.04	0.20
E3	TKW	2D	AX.89552766	2.10E+07	0.42	1.09	3.43	0.20
E3	TKW	2D	AX.89436775	2.13E+07	0.43	1.13	3.60	0.21
E3	TKW	2D	AX.89414365	2.15E+07	0.42	1.15	3.76	0.21
E3	TKW	2D	AX.89445253	2.15E+07	0.42	1.15	3.76	0.21

E3	TKW	2D	AX.89680110	2.15E+07	0.42	1.15	3.76	0.21
E3	TKW	2D	AX.89735804	2.15E+07	0.42	1.15	3.76	0.21
E3	TKW	2D	AX.89399410	2.15E+07	0.42	1.15	3.76	0.21
E3	TKW	2D	AX.89589257	2.15E+07	0.42	1.15	3.76	0.21
E3	TKW	2D	AX.89428087	2.15E+07	0.42	1.16	3.80	0.21
E3	TKW	2D	AX.89621281	2.15E+07	0.41	1.06	3.30	0.20
E3	TKW	2D	AX.89367033	2.16E+07	0.42	1.15	3.76	0.21
E3	TKW	2D	AX.89477763	2.16E+07	0.42	1.15	3.76	0.21
E3	TKW	2D	AX.89440072	2.16E+07	0.42	1.18	3.96	0.22
E3	TKW	2D	AX.89687536	2.16E+07	0.42	1.20	4.07	0.22
E3	TKW	2D	AX.89563629	2.16E+07	0.42	1.15	3.76	0.21
E3	TKW	2D	AX.89390584	2.16E+07	0.42	1.18	3.96	0.22
E3	TKW	2D	AX.89419142	2.16E+07	0.42	1.12	3.63	0.21
E3	TKW	2D	AX.89430147	2.16E+07	0.42	1.15	3.76	0.21
E3	TKW	2D	AX.89728485	2.16E+07	0.42	1.15	3.76	0.21
E3	TKW	2D	AX.89387802	2.16E+07	0.42	1.18	3.96	0.22
E3	TKW	2D	AX.89410112	2.16E+07	0.42	1.18	3.96	0.22
E3	TKW	2D	AX.89488513	2.16E+07	0.42	1.18	3.96	0.22
E3	TKW	2D	AX.89627631	2.16E+07	0.42	1.15	3.76	0.21
E3	TKW	2D	AX.89492844	2.16E+07	0.42	1.15	3.76	0.21
E3	TKW	2D	AX.89666819	2.16E+07	0.43	1.22	4.22	0.21
E3	TKW	2D	AX.89355761	2.16E+07	0.42	1.15	3.76	0.21
E3	TKW	2D	AX.89550995	2.16E+07	0.42	1.13	3.68	0.20
E3	TKW	2D	AX.89675693	2.16E+07	0.42	1.15	3.76	0.21
E3	TKW	2D	AX.89443581	2.16E+07	0.42	1.15	3.76	0.21
E3	TKW	2D	AX.89706536	2.16E+07	0.42	1.15	3.76	0.21
E3	TKW	2D	AX.89456760	2.16E+07	0.42	1.15	3.76	0.21
E3	TKW	2D	AX.89777059	2.16E+07	0.42	1.15	3.76	0.21
E3	TKW	2D	AX.89667231	2.16E+07	0.42	1.15	3.76	0.21
E3	TKW	2D	AX.89429599	2.16E+07	0.42	1.15	3.76	0.21
E3	TKW	2D	AX.89637527	2.16E+07	0.42	1.15	3.76	0.21
E3	TKW	2D	AX.89608814	2.16E+07	0.43	1.22	4.22	0.21
E3	TKW	2D	AX.89680314	2.17E+07	0.42	1.15	3.76	0.21
E3	TKW	2D	AX.89644595	2.18E+07	0.42	1.15	3.76	0.21
E3	TKW	2D	AX.89380548	2.18E+07	0.42	1.15	3.76	0.21
E3	TKW	2D	AX.89418577	2.18E+07	0.42	1.20	4.02	0.22
E3	TKW	2D	AX.89317178	2.18E+07	0.42	1.16	3.81	0.21
E3	TKW	2D	AX.89411657	2.18E+07	0.42	1.24	4.35	0.23
E3	TKW	2D	AX.89473251	2.18E+07	0.42	1.20	4.02	0.22
E3	TKW	2D	AX.89462605	2.19E+07	0.42	1.20	4.02	0.22
E3	TKW	2D	AX.89731153	2.19E+07	0.42	1.20	4.02	0.22
E3	TKW	2D	AX.89729498	2.19E+07	0.42	1.15	3.76	0.21
E3	TKW	2D	AX.89332538	2.19E+07	0.42	1.15	3.76	0.21
E3	TKW	2D	AX.89682149	2.19E+07	0.42	1.15	3.76	0.21
E3	TKW	2D	AX.89542539	2.19E+07	0.42	1.20	4.02	0.22
E3	TKW	2D	AX.89501839	2.19E+07	0.42	1.20	4.02	0.22
E3	TKW	2D	AX.89392697	2.19E+07	0.42	1.20	4.02	0.22
E3	TKW	2D	AX.89516861	2.19E+07	0.42	1.20	4.02	0.22
E3	TKW	2D	AX.89456220	2.19E+07	0.42	1.20	4.02	0.22
E3	TKW	2D	AX.89464478	2.19E+07	0.42	1.15	3.76	0.21

E3	TKW	2D	AX.89382211	2.19E+07	0.42	1.15	3.76	0.21
E3	TKW	2D	AX.89406122	2.19E+07	0.42	1.16	3.81	0.21
E3	TKW	2D	AX.89771438	2.21E+07	0.42	1.15	3.76	0.21
E3	TKW	2D	AX.89616561	2.21E+07	0.42	1.15	3.76	0.21
E3	TKW	2D	AX.89725607	2.21E+07	0.42	1.15	3.76	0.21
E3	TKW	2D	AX.89625569	2.21E+07	0.42	1.16	3.81	0.21
E3	TKW	2D	AX.89721153	2.21E+07	0.42	1.15	3.76	0.21
E3	TKW	2D	AX.89531234	2.21E+07	0.42	1.20	4.02	0.22
E3	TKW	2D	AX.89716105	2.21E+07	0.42	1.15	3.76	0.21
E3	TKW	2D	AX.89679130	2.21E+07	0.42	1.15	3.76	0.21
E3	TKW	2D	AX.89554568	2.22E+07	0.42	1.03	3.15	0.19
E3	TKW	2D	AX.89613806	2.22E+07	0.42	1.03	3.15	0.19
E3	TKW	2D	AX.89675543	2.22E+07	0.42	1.04	3.20	0.19
E3	TKW	2D	AX.89602686	2.22E+07	0.42	1.03	3.15	0.19
E3	TKW	2D	AX.89388871	2.22E+07	0.41	1.10	3.37	0.20
E3	TKW	2D	AX.89503845	2.22E+07	0.41	1.10	3.37	0.20
E3	TKW	2D	AX.89576820	2.22E+07	0.41	1.08	3.21	0.20
E3	TKW	2D	AX.89473142	2.22E+07	0.41	1.08	3.21	0.20
E3	TKW	2D	AX.89546398	2.22E+07	0.41	1.10	3.37	0.20
E3	TKW	2D	AX.89577557	2.22E+07	0.40	1.15	3.63	0.21
E3	TKW	2D	AX.89414549	2.22E+07	0.40	1.15	3.62	0.21
E3	TKW	2D	AX.89712500	2.22E+07	0.35	1.34	4.00	0.24
E3	TKW	2D	AX.89555008	2.22E+07	0.35	1.41	4.34	0.26
E3	TKW	2D	AX.89564577	2.22E+07	0.36	1.32	3.82	0.24
E3	TKW	2D	AX.89776372	2.23E+07	0.35	1.41	4.34	0.26
E3	TKW	2D	AX.89481582	2.23E+07	0.35	1.36	4.07	0.25
E3	TKW	2D	AX.89322073	2.23E+07	0.36	1.32	3.82	0.24
E3	TKW	2D	AX.89477710	2.23E+07	0.35	1.34	4.00	0.24
E3	TKW	2D	AX.89677081	2.23E+07	0.36	1.40	4.38	0.24
E3	TKW	2D	AX.89755571	2.23E+07	0.35	1.16	3.32	0.22
E3	TKW	2D	AX.89693607	2.23E+07	0.35	1.20	3.31	0.22
E3	TKW	2D	AX.89354646	2.23E+07	0.35	1.34	4.00	0.24
E3	TKW	2D	AX.89544290	2.23E+07	0.35	1.41	4.34	0.26
E3	TKW	2D	AX.89467079	2.23E+07	0.35	1.34	4.00	0.24
E3	TKW	2D	AX.89341306	2.23E+07	0.35	1.34	4.00	0.24
E3	TKW	2D	AX.89491974	2.23E+07	0.35	1.34	4.00	0.24
E3	TKW	2D	AX.89401879	2.23E+07	0.35	1.34	4.00	0.24
E3	TKW	2D	AX.89345649	2.23E+07	0.35	1.39	4.25	0.25
E3	TKW	2D	AX.89724416	2.23E+07	0.35	1.39	4.25	0.25
E3	TKW	2D	AX.89520361	2.24E+07	0.36	1.32	3.82	0.24
E3	TKW	2D	AX.89375022	2.24E+07	0.35	1.39	4.25	0.25
E3	TKW	2D	AX.89743259	2.24E+07	0.35	1.34	4.00	0.24
E3	TKW	2D	AX.89679854	2.24E+07	0.35	1.39	4.25	0.25
E3	TKW	2D	AX.89617363	2.24E+07	0.35	1.39	4.25	0.25
E3	TKW	2D	AX.89677114	2.24E+07	0.35	1.34	4.00	0.24
E3	TKW	2D	AX.89687870	2.24E+07	0.35	1.31	3.82	0.24
E3	TKW	2D	AX.89411109	2.24E+07	0.35	1.34	4.00	0.24
E3	TKW	2D	AX.89478232	2.24E+07	0.35	1.36	4.16	0.25
E3	TKW	2D	AX.89555850	2.24E+07	0.35	1.32	3.84	0.24
E3	TKW	2D	AX.89588976	2.24E+07	0.36	1.32	3.82	0.24

E3	TKW	2D	AX.89403811	2.24E+07	0.35	1.34	4.00	0.24
E3	TKW	2D	AX.89752376	2.24E+07	0.36	1.32	3.82	0.24
E3	TKW	2D	AX.89745031	2.24E+07	0.35	1.48	4.92	0.26
E3	TKW	2D	AX.89704562	2.24E+07	0.36	1.32	3.82	0.24
E3	TKW	2D	AX.89366236	2.24E+07	0.35	1.37	4.16	0.25
E3	TKW	2D	AX.89674338	2.24E+07	0.35	1.34	4.00	0.24
E3	TKW	2D	AX.89607327	2.24E+07	0.35	1.34	4.00	0.24
E3	TKW	2D	AX.89648370	2.24E+07	0.34	1.35	4.06	0.25
E3	TKW	2D	AX.89754570	2.24E+07	0.36	1.40	4.33	0.25
E3	TKW	2D	AX.89557543	2.24E+07	0.35	1.34	4.00	0.24
E3	TKW	2D	AX.89596595	2.24E+07	0.35	1.34	4.00	0.24
E3	TKW	2D	AX.89687747	2.24E+07	0.35	1.34	4.00	0.24
E3	TKW	2D	AX.89345066	2.24E+07	0.35	1.34	4.00	0.24
E3	TKW	2D	AX.89354356	2.24E+07	0.35	1.34	4.00	0.24
E3	TKW	2D	AX.89414005	2.24E+07	0.40	1.03	3.04	0.19
E3	TKW	2D	AX.89753790	2.25E+07	0.38	1.13	3.32	0.23
E3	TKW	2D	AX.89476445	2.25E+07	0.36	1.19	3.40	0.23
E3	TKW	2D	AX.89721051	2.25E+07	0.36	1.19	3.37	0.23
E3	TKW	2D	AX.89344037	2.25E+07	0.36	1.24	3.60	0.23
E3	TKW	2D	AX.89314900	2.25E+07	0.36	1.35	4.27	0.24
E3	TKW	2D	AX.89578973	2.32E+07	0.35	1.23	3.55	0.23
E3	TKW	2D	AX.89393905	2.35E+07	0.31	1.25	3.29	0.24
E3	TKW	2D	AX.89549863	2.49E+07	0.32	1.12	3.13	0.21
E3	TKW	2D	AX.89429345	2.63E+07	0.35	1.45	5.61	0.23
E3	TKW	2D	AX.89728114	2.63E+07	0.37	1.42	5.39	0.22
E3	TKW	2D	AX.89693146	2.73E+07	0.35	1.33	4.72	0.21
E3	GPM2	2D	AX.89666819	2.16E+07	0.43	-1025.44	3.07	0.12
E3	GPM2	2D	AX.89608814	2.16E+07	0.43	-1025.44	3.07	0.12
E3	GPM2	2D	AX.89487867	3.42E+07	0.22	1159.05	3.99	0.07
E3	GPM2	2D	AX.89558081	3.47E+07	0.41	-1075.90	3.21	0.13
E3	GPM2	2D	AX.89382235	3.50E+07	0.46	1011.73	3.52	0.14
E3	GPM2	2D	AX.89593262	5.12E+07	0.13	1394.89	3.55	0.11
E3	GPM2	2D	AX.89635453	5.49E+07	0.26	943.06	3.01	0.10
E3	GPM2	2D	AX.89384993	7.94E+07	0.27	940.53	3.02	0.07
E3	GPM2	2D	AX.89669389	7.94E+07	0.27	940.53	3.02	0.07
E3	GPM2	2D	AX.89584610	7.95E+07	0.27	940.53	3.02	0.07
E3	GPM2	2D	AX.89737163	7.95E+07	0.27	940.53	3.02	0.07
E3	GPM2	2D	X.89494294_OT	6.38E+08	0.28	-934.60	3.03	0.02
E3	TKW	2D	X.89629101_OT	2.06E+07	0.36	1.42	4.56	0.25
E3	TKW	2D	AX.89436271	2.07E+07	0.36	1.26	3.76	0.24
E3	TKW	2D	AX.89710846	2.07E+07	0.44	1.06	3.39	0.20
E3	TKW	2D	AX.89538003	2.07E+07	0.44	1.06	3.39	0.20
E3	TKW	2D	AX.89518563	2.07E+07	0.44	1.06	3.39	0.20
E3	TKW	2D	AX.89351496	2.07E+07	0.44	1.06	3.39	0.20
E3	TKW	2D	AX.89480356	2.07E+07	0.44	1.06	3.39	0.20
E3	TKW	2D	AX.89685580	2.07E+07	0.43	1.05	3.38	0.19
E3	TKW	2D	AX.89418892	2.08E+07	0.43	1.17	4.00	0.21
E3	TKW	2D	AX.89320470	2.08E+07	0.42	1.15	3.76	0.21
E3	TKW	2D	AX.89768949	2.08E+07	0.42	1.15	3.76	0.21
E3	TKW	2D	AX.89366683	2.08E+07	0.41	1.04	3.16	0.20

E3	TKW	2D	AX.89382191	2.08E+07	0.42	1.15	3.76	0.21
E3	TKW	2D	AX.89702393	2.08E+07	0.42	1.15	3.76	0.21
E3	TKW	2D	AX.89339112	2.08E+07	0.42	1.12	3.63	0.20
E3	TKW	2D	AX.89682313	2.08E+07	0.42	1.15	3.76	0.21
E3	TKW	2D	AX.89331719	2.08E+07	0.44	1.00	3.11	0.19
E3	TKW	2D	AX.89435345	2.09E+07	0.42	1.18	3.96	0.22
E3	TKW	2D	AX.89700899	2.09E+07	0.42	1.15	3.76	0.21
E3	TKW	2D	AX.89386215	2.09E+07	0.42	1.18	3.96	0.22
E3	TKW	2D	AX.89330774	2.09E+07	0.42	1.14	3.70	0.21
E3	TKW	2D	AX.89469855	2.09E+07	0.42	1.15	3.76	0.21
E3	TKW	2D	AX.89394485	2.09E+07	0.43	1.16	3.72	0.22
E3	TKW	2D	AX.89709101	2.09E+07	0.42	1.15	3.76	0.21
E3	TKW	2D	AX.89552991	2.09E+07	0.42	1.15	3.76	0.21
E3	TKW	2D	AX.89422546	2.09E+07	0.42	1.15	3.76	0.21
E3	TKW	2D	AX.89429864	2.09E+07	0.42	1.15	3.76	0.21
E3	TKW	2D	AX.89331141	2.09E+07	0.42	1.15	3.76	0.21
E3	TKW	2D	AX.89398165	2.09E+07	0.42	1.15	3.76	0.21
E3	TKW	2D	AX.89581719	2.09E+07	0.41	1.11	3.69	0.21
E3	TKW	2D	AX.89449164	2.09E+07	0.42	1.15	3.76	0.21
E3	TKW	2D	AX.89353697	2.09E+07	0.42	1.15	3.76	0.21
E3	TKW	2D	AX.89617233	2.96E+07	0.34	1.31	4.15	0.22
E3	TKW	2D	AX.89487867	3.42E+07	0.22	-1.08	3.64	0.08
E3	TKW	2D	AX.89558081	3.47E+07	0.41	1.20	3.99	0.20
E3	GY	2D	AX.89593262	5.12E+07	0.13	4.74	3.67	0.10
E3	SPM2	2D	AX.89635110	6.00E+08	0.06	41.30	3.16	0.08
E3	SPM2	2D	AX.89478998	6.08E+08	0.10	-33.20	3.18	0.02
E3	SPM2	2D	AX.89564769	6.08E+08	0.10	-33.20	3.18	0.02
E3	SPM2	2D	AX.89404429	6.08E+08	0.10	-35.66	3.46	0.03
E4	GPM2	2D	AX.89558081	3.47E+07	0.41	-625.72	4.19	0.11
E4	GPM2	2D	AX.89382235	3.50E+07	0.46	515.02	3.51	0.10
E4	GPS	2D	AX.89545944	1.83E+07	0.15	2.20	3.37	0.05
E4	TKW	2D	AX.89367033	2.16E+07	0.42	1.06	3.39	0.17
E4	TKW	2D	AX.89477763	2.16E+07	0.42	1.06	3.39	0.17
E4	TKW	2D	AX.89440072	2.16E+07	0.42	1.08	3.50	0.18
E4	TKW	2D	AX.89687536	2.16E+07	0.42	1.10	3.62	0.18
E4	TKW	2D	AX.89563629	2.16E+07	0.42	1.06	3.39	0.17
E4	TKW	2D	AX.89390584	2.16E+07	0.42	1.08	3.50	0.18
E4	TKW	2D	AX.89419142	2.16E+07	0.42	1.08	3.51	0.17
E4	TKW	2D	AX.89430147	2.16E+07	0.42	1.06	3.39	0.17
E4	TKW	2D	AX.89728485	2.16E+07	0.42	1.06	3.39	0.17
E4	TKW	2D	AX.89387802	2.16E+07	0.42	1.08	3.50	0.18
E4	TKW	2D	AX.89410112	2.16E+07	0.42	1.08	3.50	0.18
E4	TKW	2D	AX.89488513	2.16E+07	0.42	1.08	3.50	0.18
E4	TKW	2D	AX.89627631	2.16E+07	0.42	1.06	3.39	0.17
E4	TKW	2D	AX.89492844	2.16E+07	0.42	1.06	3.39	0.17
E4	TKW	2D	AX.89666819	2.16E+07	0.43	1.01	3.15	0.17
E4	TKW	2D	AX.89355761	2.16E+07	0.42	1.06	3.39	0.17
E4	TKW	2D	AX.89550995	2.16E+07	0.42	1.01	3.14	0.16
E4	TKW	2D	AX.89675693	2.16E+07	0.42	1.06	3.39	0.17
E4	TKW	2D	AX.89443581	2.16E+07	0.42	1.06	3.39	0.17

E4	TKW	2D	AX.89706536	2.16E+07	0.42	1.06	3.39	0.17
E4	TKW	2D	AX.89317945	1.05E+07	0.22	-1.03	3.26	0.07
E4	TKW	2D	X.89491138_OT	1.07E+07	0.22	-1.03	3.26	0.07
E4	TKW	2D	X.89629101_OT	2.06E+07	0.36	1.20	3.54	0.20
E4	TKW	2D	AX.89621969	2.07E+07	0.34	-0.91	3.07	0.12
E4	TKW	2D	AX.89606458	2.07E+07	0.40	-0.93	3.08	0.12
E4	TKW	2D	AX.89491215	2.07E+07	0.40	-0.93	3.08	0.12
E4	TKW	2D	AX.89418892	2.08E+07	0.43	1.00	3.12	0.17
E4	TKW	2D	AX.89448778	2.08E+07	0.50	1.06	3.92	0.16
E4	TKW	2D	AX.89320470	2.08E+07	0.42	1.06	3.39	0.17
E4	TKW	2D	AX.89768949	2.08E+07	0.42	1.06	3.39	0.17
E4	TKW	2D	AX.89382191	2.08E+07	0.42	1.06	3.39	0.17
E4	TKW	2D	AX.89702393	2.08E+07	0.42	1.06	3.39	0.17
E4	TKW	2D	AX.89339112	2.08E+07	0.42	1.03	3.21	0.17
E4	TKW	2D	AX.89682313	2.08E+07	0.42	1.06	3.39	0.17
E4	TKW	2D	AX.89435345	2.09E+07	0.42	1.08	3.50	0.18
E4	TKW	2D	AX.89700899	2.09E+07	0.42	1.06	3.39	0.17
E4	TKW	2D	AX.89386215	2.09E+07	0.42	1.08	3.50	0.18
E4	TKW	2D	AX.89330774	2.09E+07	0.42	1.06	3.34	0.17
E4	TKW	2D	AX.89469855	2.09E+07	0.42	1.06	3.39	0.17
E4	TKW	2D	AX.89394485	2.09E+07	0.43	1.08	3.39	0.18
E4	TKW	2D	AX.89709101	2.09E+07	0.42	1.06	3.39	0.17
E4	TKW	2D	AX.89552991	2.09E+07	0.42	1.06	3.39	0.17
E4	TKW	2D	AX.89422546	2.09E+07	0.42	1.06	3.39	0.17
E4	TKW	2D	AX.89429864	2.09E+07	0.42	1.06	3.39	0.17
E4	TKW	2D	AX.89331141	2.09E+07	0.42	1.06	3.39	0.17
E4	TKW	2D	AX.89398165	2.09E+07	0.42	1.06	3.39	0.17
E4	TKW	2D	AX.89581719	2.09E+07	0.41	1.01	3.23	0.17
E4	TKW	2D	AX.89449164	2.09E+07	0.42	1.06	3.39	0.17
E4	TKW	2D	AX.89353697	2.09E+07	0.42	1.06	3.39	0.17
E4	TKW	2D	AX.89506509	2.09E+07	0.42	1.01	3.09	0.17
E4	TKW	2D	AX.89693001	2.09E+07	0.42	1.08	3.50	0.18
E4	TKW	2D	AX.89389022	2.09E+07	0.42	1.06	3.39	0.17
E4	TKW	2D	AX.89685659	2.09E+07	0.43	1.06	3.32	0.17
E4	TKW	2D	AX.89524929	2.10E+07	0.42	1.06	3.39	0.17
E4	TKW	2D	AX.89320828	2.10E+07	0.42	1.06	3.39	0.17
E4	TKW	2D	AX.89552766	2.10E+07	0.42	0.99	3.04	0.16
E4	TKW	2D	AX.89436775	2.13E+07	0.43	1.06	3.32	0.17
E4	TKW	2D	AX.89414365	2.15E+07	0.42	1.06	3.39	0.17
E4	TKW	2D	AX.89445253	2.15E+07	0.42	1.06	3.39	0.17
E4	TKW	2D	AX.89680110	2.15E+07	0.42	1.06	3.39	0.17
E4	TKW	2D	AX.89735804	2.15E+07	0.42	1.06	3.39	0.17
E4	TKW	2D	AX.89399410	2.15E+07	0.42	1.06	3.39	0.17
E4	TKW	2D	AX.89589257	2.15E+07	0.42	1.06	3.39	0.17
E4	TKW	2D	AX.89428087	2.15E+07	0.42	1.01	3.11	0.17
E4	TKW	2D	AX.89679130	2.21E+07	0.42	1.06	3.39	0.17
E4	TKW	2D	AX.89388871	2.22E+07	0.41	1.04	3.19	0.16
E4	TKW	2D	AX.89503845	2.22E+07	0.41	1.04	3.19	0.16
E4	TKW	2D	AX.89576820	2.22E+07	0.41	1.04	3.11	0.16
E4	TKW	2D	AX.89473142	2.22E+07	0.41	1.04	3.11	0.16

E4	TKW	2D	AX.89546398	2.22E+07	0.41	1.04	3.19	0.16
E4	TKW	2D	AX.89712500	2.22E+07	0.35	1.25	3.67	0.20
E4	TKW	2D	AX.89555008	2.22E+07	0.35	1.17	3.22	0.20
E4	TKW	2D	AX.89564577	2.22E+07	0.36	1.25	3.60	0.20
E4	TKW	2D	AX.89776372	2.23E+07	0.35	1.17	3.22	0.20
E4	TKW	2D	AX.89481582	2.23E+07	0.35	1.18	3.31	0.20
E4	TKW	2D	AX.89322073	2.23E+07	0.36	1.25	3.60	0.20
E4	TKW	2D	AX.89477710	2.23E+07	0.35	1.25	3.67	0.20
E4	TKW	2D	AX.89677081	2.23E+07	0.36	1.31	4.04	0.20
E4	TKW	2D	AX.89755571	2.23E+07	0.35	1.15	3.36	0.18
E4	TKW	2D	AX.89693607	2.23E+07	0.35	1.11	3.01	0.18
E4	TKW	2D	AX.89354646	2.23E+07	0.35	1.25	3.67	0.20
E4	TKW	2D	AX.89544290	2.23E+07	0.35	1.17	3.22	0.20
E4	TKW	2D	AX.89467079	2.23E+07	0.35	1.25	3.67	0.20
E4	TKW	2D	AX.89341306	2.23E+07	0.35	1.25	3.67	0.20
E4	TKW	2D	AX.89456760	2.16E+07	0.42	1.06	3.39	0.17
E4	TKW	2D	AX.89777059	2.16E+07	0.42	1.06	3.39	0.17
E4	TKW	2D	AX.89667231	2.16E+07	0.42	1.06	3.39	0.17
E4	TKW	2D	AX.89429599	2.16E+07	0.42	1.06	3.39	0.17
E4	TKW	2D	AX.89637527	2.16E+07	0.42	1.06	3.39	0.17
E4	TKW	2D	AX.89608814	2.16E+07	0.43	1.01	3.15	0.17
E4	TKW	2D	AX.89680314	2.17E+07	0.42	1.06	3.39	0.17
E4	TKW	2D	AX.89644595	2.18E+07	0.42	1.06	3.39	0.17
E4	TKW	2D	AX.89380548	2.18E+07	0.42	1.06	3.39	0.17
E4	TKW	2D	AX.89411657	2.18E+07	0.42	0.99	3.03	0.17
E4	TKW	2D	AX.89729498	2.19E+07	0.42	1.06	3.39	0.17
E4	TKW	2D	AX.89332538	2.19E+07	0.42	1.06	3.39	0.17
E4	TKW	2D	AX.89682149	2.19E+07	0.42	1.06	3.39	0.17
E4	TKW	2D	AX.89464478	2.19E+07	0.42	1.06	3.39	0.17
E4	TKW	2D	AX.89382211	2.19E+07	0.42	1.06	3.39	0.17
E4	TKW	2D	AX.89771438	2.21E+07	0.42	1.06	3.39	0.17
E4	TKW	2D	AX.89616561	2.21E+07	0.42	1.06	3.39	0.17
E4	TKW	2D	AX.89725607	2.21E+07	0.42	1.06	3.39	0.17
E4	TKW	2D	AX.89721153	2.21E+07	0.42	1.06	3.39	0.17
E4	TKW	2D	AX.89716105	2.21E+07	0.42	1.06	3.39	0.17
E4	TKW	2D	AX.89674338	2.24E+07	0.35	1.25	3.67	0.20
E4	TKW	2D	AX.89607327	2.24E+07	0.35	1.25	3.67	0.20
E4	TKW	2D	AX.89648370	2.24E+07	0.34	1.32	3.99	0.21
E4	TKW	2D	AX.89754570	2.24E+07	0.36	1.24	3.65	0.20
E4	TKW	2D	AX.89557543	2.24E+07	0.35	1.25	3.67	0.20
E4	TKW	2D	AX.89596595	2.24E+07	0.35	1.25	3.67	0.20
E4	TKW	2D	AX.89687747	2.24E+07	0.35	1.25	3.67	0.20
E4	TKW	2D	AX.89345066	2.24E+07	0.35	1.25	3.67	0.20
E4	TKW	2D	AX.89354356	2.24E+07	0.35	1.25	3.67	0.20
E4	TKW	2D	AX.89476445	2.25E+07	0.36	1.10	3.05	0.18
E4	TKW	2D	AX.89344037	2.25E+07	0.36	1.15	3.23	0.19
E4	TKW	2D	AX.89314900	2.25E+07	0.36	1.15	3.31	0.19
E4	TKW	2D	AX.89578973	2.32E+07	0.35	1.24	3.70	0.20
E4	TKW	2D	AX.89393905	2.35E+07	0.31	1.24	3.36	0.19
E4	TKW	2D	AX.89549863	2.49E+07	0.32	1.18	3.49	0.18

E4	TKW	2D	AX.89429345	2.63E+07	0.35	1.23	4.33	0.19
E4	TKW	2D	AX.89728114	2.63E+07	0.37	1.40	5.38	0.20
E4	TKW	2D	AX.89693146	2.73E+07	0.35	1.44	5.58	0.21
E4	TKW	2D	AX.89647365	2.79E+07	0.20	-1.20	3.92	0.11
E4	TKW	2D	AX.89408510	2.79E+07	0.20	-1.18	3.88	0.11
E4	TKW	2D	AX.89491974	2.23E+07	0.35	1.25	3.67	0.20
E4	TKW	2D	AX.89401879	2.23E+07	0.35	1.25	3.67	0.20
E4	TKW	2D	AX.89345649	2.23E+07	0.35	1.28	3.81	0.21
E4	TKW	2D	AX.89724416	2.23E+07	0.35	1.28	3.81	0.21
E4	TKW	2D	AX.89520361	2.24E+07	0.36	1.25	3.60	0.20
E4	TKW	2D	AX.89375022	2.24E+07	0.35	1.28	3.81	0.21
E4	TKW	2D	AX.89743259	2.24E+07	0.35	1.25	3.67	0.20
E4	TKW	2D	AX.89679854	2.24E+07	0.35	1.28	3.81	0.21
E4	TKW	2D	AX.89617363	2.24E+07	0.35	1.28	3.81	0.21
E4	TKW	2D	AX.89677114	2.24E+07	0.35	1.25	3.67	0.20
E4	TKW	2D	AX.89687870	2.24E+07	0.35	1.27	3.75	0.20
E4	TKW	2D	AX.89411109	2.24E+07	0.35	1.25	3.67	0.20
E4	TKW	2D	AX.89478232	2.24E+07	0.35	1.21	3.51	0.20
E4	TKW	2D	AX.89555850	2.24E+07	0.35	1.24	3.57	0.20
E4	TKW	2D	AX.89588976	2.24E+07	0.36	1.25	3.60	0.20
E4	TKW	2D	AX.89403811	2.24E+07	0.35	1.25	3.67	0.20
E4	TKW	2D	AX.89752376	2.24E+07	0.36	1.25	3.60	0.20
E4	TKW	2D	AX.89745031	2.24E+07	0.35	1.37	4.42	0.21
E4	TKW	2D	AX.89704562	2.24E+07	0.36	1.25	3.60	0.20
E4	TKW	2D	AX.89366236	2.24E+07	0.35	1.28	3.77	0.21
E4	TKW	2D	AX.89669389	7.94E+07	0.27	-1.10	4.15	0.09
E4	TKW	2D	AX.89584610	7.95E+07	0.27	-1.10	4.15	0.09
E4	TKW	2D	AX.89737163	7.95E+07	0.27	-1.10	4.15	0.09
E4	TKW	2D	AX.89654955	7.95E+07	0.27	-1.10	4.20	0.09
E4	TKW	2D	AX.89313540	7.98E+07	0.27	-1.13	4.39	0.09
E4	TKW	2D	AX.89338432	7.98E+07	0.28	-1.05	3.91	0.09
E4	TKW	2D	AX.89441180	2.80E+07	0.29	1.23	3.85	0.17
E4	TKW	2D	AX.89617233	2.96E+07	0.34	1.65	6.42	0.24
E4	TKW	2D	AX.89558081	3.47E+07	0.41	1.85	8.97	0.27
E4	TKW	2D	AX.89382235	3.50E+07	0.46	-1.30	5.72	0.15
E4	TKW	2D	AX.89371003	3.71E+07	0.20	1.42	4.63	0.07
E4	TKW	2D	AX.89384993	7.94E+07	0.27	-1.10	4.15	0.09
E1	GY	3A	AX.89517433	5.57E+07	0.37	-2.50	3.45	0.06
E1	GY	3A	AX.89596917	5.62E+07	0.38	-2.49	3.45	0.06
E1	GY	3A	AX.89410914	5.70E+07	0.38	-2.49	3.45	0.06
E1	GY	3A	AX.86166408	7.19E+08	0.46	-2.29	3.12	0.04
E1	GY	3A	AX.89351757	7.11E+08	0.20	2.83	3.12	0.05
E1	GY	3A	AX.89410914	5.70E+07	0.38	-2.49	3.45	0.06
E1	SPM2	3A	AX.89449366	2.36E+07	0.12	28.09	3.73	0.07
E1	GY	3A	AX.89344030	5.71E+07	0.37	-2.77	4.08	0.07
E1	GY	3A	AX.89413279	7.19E+08	0.46	-2.29	3.12	0.04
E1	GY	3A	AX.89529890	6.93E+07	0.33	-2.43	3.14	0.05
E1	GY	3A	AX.89436052	5.70E+07	0.38	-2.49	3.45	0.06
E1	GY	3A	AX.89529890	6.93E+07	0.33	-2.43	3.14	0.05
E1	GY	3A	AX.89471245	1.02E+07	0.32	2.60	3.47	0.06

E1	GY	3A	AX.89416077	5.70E+07	0.38	-2.49	3.45	0.06
E1	GY	3A	AX.89587804	7.11E+08	0.20	2.83	3.12	0.05
E1	GY	3A	AX.86166408	7.19E+08	0.46	-2.29	3.12	0.04
E1	GY	3A	AX.89436052	5.70E+07	0.38	-2.49	3.45	0.06
E1	GY	3A	AX.89563886	5.17E+08	0.05	-6.25	3.98	0.08
E1	GY	3A	AX.89313133	5.67E+07	0.38	-2.49	3.45	0.06
E1	GY	3A	AX.89351757	7.11E+08	0.20	2.83	3.12	0.05
E1	GY	3A	AX.89386202	7.11E+08	0.26	2.68	3.25	0.06
E1	GY	3A	AX.89468914	5.68E+07	0.37	-2.50	3.45	0.06
E1	GY	3A	AX.89373966	5.64E+07	0.37	-2.50	3.45	0.06
E1	GY	3A	AX.89414890	5.67E+07	0.39	-2.39	3.24	0.05
E1	GY	3A	AX.89337319	7.11E+08	0.06	-6.47	5.27	0.09
E1	GY	3A	AX.89471245	1.02E+07	0.32	2.60	3.47	0.06
E1	GY	3A	AX.89775578	5.61E+07	0.38	-2.49	3.45	0.06
E1	SPM2	3A	AX.89519149	2.36E+07	0.12	28.09	3.73	0.07
E1	GY	3A	AX.89735763	5.67E+07	0.37	-2.50	3.45	0.06
E1	GY	3A	AX.89312238	1.35E+08	0.09	-4.28	3.47	0.05
E1	GY	3A	AX.89450737	5.70E+07	0.37	-2.50	3.45	0.06
E1	GY	3A	AX.89389379	7.11E+08	0.06	-4.99	3.35	0.05
E1	GY	3A	AX.89389379	7.11E+08	0.06	-4.99	3.35	0.05
E1	SPM2	3A	AX.89391363	2.36E+07	0.12	28.09	3.73	0.07
E1	GY	3A	AX.89411579	6.38E+08	0.41	2.28	3.04	0.05
E1	GY	3A	AX.89517433	5.57E+07	0.37	-2.50	3.45	0.06
E1	GY	3A	AX.89411579	6.38E+08	0.41	2.28	3.04	0.05
E1	SPM2	3A	AX.89599951	2.36E+07	0.12	28.09	3.73	0.07
E1	GY	3A	AX.89599243	7.11E+08	0.07	-5.26	4.12	0.07
E1	GY	3A	AX.89427924	5.61E+07	0.37	-2.50	3.45	0.06
E1	SPM2	3A	AX.89768349	2.36E+07	0.12	28.09	3.73	0.07
E1	GY	3A	AX.89337319	7.11E+08	0.06	-6.47	5.27	0.09
E1	GY	3A	AX.89404755	7.11E+08	0.24	3.52	5.00	0.09
E1	SPM2	3A	AX.89573701	2.32E+07	0.09	34.31	4.31	0.08
E1	GY	3A	AX.89329876	1.18E+07	0.32	2.41	3.04	0.05
E1	GY	3A	AX.89673565	2.17E+05	0.34	2.44	3.19	0.06
E1	GY	3A	AX.89373966	5.64E+07	0.37	-2.50	3.45	0.06
E1	SPM2	3A	AX.89538706	2.35E+07	0.09	31.32	3.68	0.07
E1	GY	3A	AX.89414890	5.67E+07	0.39	-2.39	3.24	0.05
E1	GY	3A	AX.89405834	6.34E+08	0.47	-2.47	3.56	0.06
E1	GY	3A	AX.89684384	5.70E+07	0.38	-2.49	3.45	0.06
E1	GY	3A	AX.89463175	5.61E+07	0.38	-2.49	3.45	0.06
E1	GY	3A	AX.89437855	5.68E+07	0.38	-2.61	3.72	0.06
E1	GY	3A	AX.89512498	5.68E+07	0.37	-2.50	3.45	0.06
E1	GY	3A	AX.89744040	5.16E+08	0.05	-5.81	3.79	0.07
E1	SPM2	3A	AX.89340976	2.36E+07	0.12	28.09	3.73	0.07
E1	GY	3A	AX.89476226	5.70E+07	0.38	-2.49	3.45	0.06
E1	GY	3A	AX.89740070	7.12E+08	0.20	2.83	3.12	0.05
E1	GY	3A	AX.89463175	5.61E+07	0.38	-2.49	3.45	0.06
E1	GY	3A	AX.89437855	5.68E+07	0.38	-2.61	3.72	0.06
E1	GY	3A	AX.89322989	5.44E+08	0.05	-5.81	3.79	0.07
E1	GY	3A	AX.89450737	5.70E+07	0.37	-2.50	3.45	0.06
E1	GY	3A	AX.89422432	1.15E+07	0.31	2.54	3.26	0.06

E1	GY	3A	AX.89348668	6.40E+08	0.13	-3.61	3.48	0.05
E1	GY	3A	AX.89344030	5.71E+07	0.37	-2.77	4.08	0.07
E1	GY	3A	AX.89312843	5.67E+07	0.38	-2.49	3.45	0.06
E1	GY	3A	AX.89396715	6.59E+08	0.29	2.60	3.30	0.06
E1	GY	3A	AX.89461923	5.61E+07	0.38	-2.52	3.50	0.06
E1	GY	3A	AX.89345333	5.62E+07	0.37	-2.50	3.45	0.06
E1	GY	3A	AX.89392516	2.27E+05	0.50	2.51	3.67	0.06
E1	SPM2	3A	AX.89584828	2.36E+07	0.12	28.09	3.73	0.07
E1	GY	3A	AX.89742601	6.59E+08	0.21	3.27	4.13	0.07
E1	SPM2	3A	AX.89635508	2.34E+07	0.09	31.32	3.68	0.07
E1	GY	3A	AX.89318922	5.67E+07	0.37	-2.50	3.45	0.06
E1	GY	3A	AX.89320189	5.61E+07	0.38	-2.49	3.45	0.06
E1	GY	3A	AX.89669221	7.11E+08	0.20	2.83	3.12	0.05
E1	GY	3A	AX.89415949	7.11E+08	0.07	-6.05	4.97	0.08
E1	SPM2	3A	AX.89753864	2.34E+07	0.09	31.32	3.68	0.07
E1	GY	3A	AX.89475487	5.67E+07	0.37	-2.50	3.45	0.06
E1	GY	3A	AX.89410438	2.25E+05	0.50	-2.33	3.24	0.04
E1	SPM2	3A	AX.89587445	2.31E+07	0.10	30.49	3.65	0.07
E1	GY	3A	AX.89320950	6.59E+08	0.21	3.18	3.86	0.07
E1	GY	3A	AX.89658110	5.70E+07	0.38	-2.49	3.45	0.06
E1	GY	3A	AX.89312238	1.35E+08	0.09	-4.28	3.47	0.05
E1	GY	3A	AX.89335688	7.11E+08	0.07	-5.26	4.12	0.07
E1	GY	3A	AX.89386202	7.11E+08	0.26	2.68	3.25	0.06
E1	GY	3A	AX.89398270	5.67E+07	0.37	-2.50	3.45	0.06
E1	GY	3A	AX.89540978	5.44E+08	0.05	-6.25	3.98	0.08
E1	SPM2	3A	AX.89516485	6.86E+08	0.27	21.85	4.12	0.08
E1	GY	3A	AX.89322500	5.62E+07	0.38	-2.49	3.45	0.06
E1	GY	3A	AX.89336419	7.12E+08	0.20	2.83	3.12	0.05
E1	GY	3A	AX.89446558	7.11E+08	0.07	-6.05	4.97	0.08
E1	GY	3A	AX.89599243	7.11E+08	0.07	-5.26	4.12	0.07
E1	GY	3A	AX.89329876	1.18E+07	0.32	2.41	3.04	0.05
E1	GY	3A	AX.89630559	2.13E+05	0.35	2.38	3.10	0.05
E1	GY	3A	AX.89465002	2.08E+05	0.35	2.40	3.14	0.06
E1	GY	3A	AX.89521839	7.11E+08	0.25	2.58	3.02	0.05
E1	GY	3A	AX.89412798	7.11E+08	0.20	2.83	3.12	0.05
E1	SPM2	3A	AX.89679071	2.36E+07	0.12	28.09	3.73	0.07
E1	GY	3A	AX.89631332	6.60E+08	0.24	2.83	3.41	0.06
E1	GY	3A	AX.89413279	7.19E+08	0.46	-2.29	3.12	0.04
E1	SPM2	3A	AX.89669278	7.26E+08	0.23	-19.66	3.15	0.05
E1	GY	3A	AX.89596917	5.62E+07	0.38	-2.49	3.45	0.06
E1	GY	3A	AX.89564968	6.59E+08	0.29	2.50	3.09	0.05
E1	GY	3A	AX.89424557	5.71E+07	0.38	-2.49	3.45	0.06
E1	GY	3A	AX.89471074	7.19E+08	0.46	-2.29	3.12	0.04
E1	GY	3A	AX.89402066	7.19E+08	0.22	-3.89	5.69	0.10
E1	GY	3A	AX.89338545	5.71E+07	0.38	-2.49	3.45	0.06
E1	SPM2	3A	AX.89394999	2.34E+07	0.09	30.57	3.54	0.06
E1	GY	3A	AX.89402456	7.11E+08	0.24	3.52	5.00	0.09
E1	GY	3A	AX.89358928	5.71E+07	0.38	-2.49	3.45	0.06
E1	GY	3A	AX.89613894	7.11E+08	0.07	-6.16	5.13	0.08
E1	GY	3A	AX.89422432	1.15E+07	0.31	2.54	3.26	0.06

E1	GY	3A	AX.89577654	6.59E+08	0.29	2.60	3.30	0.06
E1	GY	3A	AX.89322500	5.62E+07	0.38	-2.49	3.45	0.06
E1	GY	3A	AX.89412117	5.16E+08	0.05	-5.81	3.79	0.07
E1	GY	3A	AX.89431563	7.12E+08	0.20	2.83	3.12	0.05
E1	GY	3A	AX.89684173	7.12E+08	0.20	2.83	3.12	0.05
E1	GY	3A	AX.89412798	7.11E+08	0.20	2.83	3.12	0.05
E1	GY	3A	AX.89587706	1.78E+05	0.35	-2.46	3.29	0.05
E1	GY	3A	AX.89591989	7.12E+08	0.20	2.83	3.12	0.05
E1	GY	3A	AX.89404755	7.11E+08	0.24	3.52	5.00	0.09
E1	SPM2	3A	AX.89312604	2.34E+07	0.10	30.49	3.65	0.07
E1	GY	3A	AX.89334033	5.71E+07	0.38	-2.49	3.45	0.06
E1	GY	3A	AX.89405051	6.59E+08	0.29	2.60	3.30	0.06
E1	GY	3A	AX.89405051	6.59E+08	0.29	2.60	3.30	0.06
E1	GY	3A	AX.89530250	5.67E+07	0.37	-2.50	3.45	0.06
E1	GY	3A	AX.89673565	2.17E+05	0.34	2.44	3.19	0.06
E1	GY	3A	AX.89334290	1.15E+07	0.23	3.00	3.73	0.07
E1	GY	3A	AX.89320189	5.61E+07	0.38	-2.49	3.45	0.06
E1	GY	3A	AX.89338545	5.71E+07	0.38	-2.49	3.45	0.06
E1	GY	3A	AX.89727293	6.93E+07	0.33	-2.43	3.14	0.05
E1	GY	3A	AX.89538282	5.64E+07	0.38	-2.49	3.45	0.06
E1	GY	3A	AX.89531199	5.57E+07	0.37	-2.50	3.45	0.06
E1	GY	3A	AX.89534837	5.70E+07	0.38	-2.49	3.45	0.06
E1	GY	3A	AX.89355033	7.11E+08	0.08	-4.92	4.05	0.06
E1	GY	3A	AX.89740070	7.12E+08	0.20	2.83	3.12	0.05
E1	GY	3A	AX.89617804	6.93E+07	0.33	-2.43	3.14	0.05
E1	GY	3A	AX.89607034	5.64E+07	0.37	-2.50	3.45	0.06
E1	GY	3A	AX.89313868	5.67E+07	0.39	-2.51	3.51	0.06
E1	GY	3A	AX.89407423	5.38E+07	0.07	-4.65	3.14	0.06
E1	GY	3A	AX.89336419	7.12E+08	0.20	2.83	3.12	0.05
E1	GY	3A	AX.89539719	5.67E+07	0.37	-2.50	3.45	0.06
E1	GY	3A	AX.89509264	5.16E+08	0.05	-5.81	3.79	0.07
E1	SPM2	3A	AX.89775349	2.35E+07	0.09	31.32	3.68	0.07
E1	GY	3A	AX.89336503	2.11E+05	0.35	2.40	3.14	0.06
E1	GY	3A	AX.89675847	6.59E+08	0.28	2.73	3.52	0.06
E1	GY	3A	AX.89543452	1.14E+07	0.30	2.96	4.23	0.08
E1	GY	3A	AX.89700698	2.08E+05	0.35	2.40	3.14	0.06
E1	GY	3A	AX.89477720	5.71E+07	0.38	-2.49	3.45	0.06
E1	GY	3A	AX.89443263	7.12E+08	0.20	2.83	3.12	0.05
E1	GY	3A	AX.89443263	7.12E+08	0.20	2.83	3.12	0.05
E1	GY	3A	AX.89701109	6.59E+08	0.29	2.60	3.30	0.06
E1	GY	3A	AX.89348668	6.40E+08	0.13	-3.61	3.48	0.05
E1	GY	3A	AX.89372891	6.93E+07	0.33	-2.43	3.14	0.05
E1	GY	3A	AX.89326987	5.64E+07	0.38	-2.49	3.45	0.06
E1	GY	3A	AX.89360803	5.62E+07	0.38	-2.49	3.45	0.06
E1	GY	3A	AX.89401377	5.67E+07	0.37	-2.50	3.45	0.06
E1	GY	3A	AX.89604070	5.70E+07	0.38	-2.49	3.45	0.06
E1	GY	3A	AX.89432808	5.58E+07	0.37	-2.50	3.45	0.06
E1	GY	3A	AX.89526633	5.17E+08	0.05	-6.25	3.98	0.08
E1	GY	3A	AX.89579168	8.74E+06	0.39	2.50	3.47	0.06
E1	GY	3A	AX.89502635	5.71E+07	0.39	-2.51	3.51	0.06

E1	GY	3A	AX.89502639	5.68E+07	0.37	-2.50	3.45	0.06
E1	SPM2	3A	AX.89468181	2.35E+07	0.09	31.32	3.68	0.07
E1	GY	3A	AX.89549204	5.64E+07	0.38	-2.49	3.45	0.06
E1	GY	3A	AX.89374417	7.11E+08	0.24	3.52	5.00	0.09
E1	GY	3A	AX.89467246	5.70E+07	0.38	-2.49	3.45	0.06
E1	GY	3A	AX.89530750	7.11E+08	0.06	-6.47	5.27	0.09
E1	GY	3A	AX.89362464	6.59E+08	0.28	2.51	3.06	0.05
E1	GY	3A	AX.89398270	5.67E+07	0.37	-2.50	3.45	0.06
E1	GY	3A	AX.89410438	2.25E+05	0.50	-2.33	3.24	0.04
E1	GY	3A	AX.89538282	5.64E+07	0.38	-2.49	3.45	0.06
E1	GY	3A	AX.89695116	5.24E+08	0.05	-6.25	3.98	0.08
E1	GY	3A	AX.89695116	5.24E+08	0.05	-6.25	3.98	0.08
E1	GY	3A	AX.89371144	7.11E+08	0.06	-6.47	5.27	0.09
E1	GY	3A	AX.89720264	7.11E+08	0.08	-4.91	4.04	0.06
E1	GY	3A	AX.89531199	5.57E+07	0.37	-2.50	3.45	0.06
E1	GY	3A	AX.89363017	7.11E+08	0.06	-5.26	3.66	0.06
E1	GY	3A	AX.89538943	7.11E+08	0.07	-5.90	5.31	0.09
E1	GY	3A	AX.89633282	1.31E+06	0.36	-2.35	3.04	0.04
E1	GY	3A	AX.89463636	7.11E+08	0.07	-6.05	4.97	0.08
E1	GY	3A	AX.89386200	2.27E+05	0.49	2.31	3.18	0.05
E1	SPM2	3A	AX.89606521	2.34E+07	0.09	31.02	3.62	0.06
E1	SPM2	3A	AX.89414162	2.34E+07	0.10	30.49	3.65	0.07
E1	GY	3A	AX.89577654	6.59E+08	0.29	2.60	3.30	0.06
E1	GY	3A	AX.89411704	7.04E+08	0.30	2.50	3.12	0.04
E1	SPM2	3A	AX.89344128	2.36E+07	0.13	26.36	3.44	0.06
E1	GY	3A	AX.89465002	2.08E+05	0.35	2.40	3.14	0.06
E1	GY	3A	AX.89412589	5.62E+07	0.38	-2.49	3.45	0.06
E1	GY	3A	AX.89412589	5.62E+07	0.38	-2.49	3.45	0.06
E1	GY	3A	AX.89521839	7.11E+08	0.25	2.58	3.02	0.05
E1	GY	3A	AX.89563886	5.17E+08	0.05	-6.25	3.98	0.08
E1	GY	3A	AX.89587706	1.78E+05	0.35	-2.46	3.29	0.05
E1	GY	3A	AX.89587804	7.11E+08	0.20	2.83	3.12	0.05
E1	GY	3A	AX.89684384	5.70E+07	0.38	-2.49	3.45	0.06
E1	GY	3A	AX.89635924	5.61E+07	0.37	-2.50	3.45	0.06
E1	GY	3A	AX.89635924	5.61E+07	0.37	-2.50	3.45	0.06
E1	GY	3A	AX.89444324	7.12E+08	0.21	2.98	3.45	0.06
E1	SPM2	3A	AX.89656077	2.35E+07	0.09	31.32	3.68	0.07
E1	GY	3A	AX.89402066	7.19E+08	0.22	-3.89	5.69	0.10
E1	GY	3A	AX.89471595	7.11E+08	0.07	-6.05	4.97	0.08
E1	GY	3A	AX.89402335	7.11E+08	0.24	3.52	5.00	0.09
E1	GY	3A	AX.89735763	5.67E+07	0.37	-2.50	3.45	0.06
E1	GY	3A	AX.89378945	5.68E+07	0.37	-2.50	3.45	0.06
E1	GY	3A	AX.89378945	5.68E+07	0.37	-2.50	3.45	0.06
E1	SPM2	3A	AX.89442115	2.36E+07	0.12	28.09	3.73	0.07
E1	GY	3A	AX.89379315	2.06E+05	0.35	2.40	3.14	0.06
E1	GY	3A	AX.89446558	7.11E+08	0.07	-6.05	4.97	0.08
E1	GY	3A	AX.89675847	6.59E+08	0.28	2.73	3.52	0.06
E1	GY	3A	AX.89517819	6.93E+07	0.33	-2.43	3.14	0.05
E1	GY	3A	AX.89322989	5.44E+08	0.05	-5.81	3.79	0.07
E1	GY	3A	AX.89567118	5.67E+07	0.37	-2.50	3.45	0.06

E1	SPM2	3A	AX.89468470	2.34E+07	0.09	31.32	3.68	0.07
E1	GY	3A	AX.89427948	5.67E+07	0.37	-2.50	3.45	0.06
E1	GY	3A	AX.89701109	6.59E+08	0.29	2.60	3.30	0.06
E1	GY	3A	AX.89775222	5.61E+07	0.37	-2.50	3.45	0.06
E1	GY	3A	AX.89416353	7.11E+08	0.24	3.52	5.00	0.09
E1	GY	3A	AX.89762555	7.19E+08	0.46	-2.29	3.12	0.04
E1	GY	3A	AX.89345333	5.62E+07	0.37	-2.50	3.45	0.06
E1	GY	3A	AX.89775578	5.61E+07	0.38	-2.49	3.45	0.06
E1	GY	3A	AX.89369804	5.57E+07	0.37	-2.50	3.45	0.06
E1	SPM2	3A	AX.89499565	7.10E+08	0.23	-19.30	3.06	0.05
E1	GY	3A	AX.89334290	1.15E+07	0.23	3.00	3.73	0.07
E1	GY	3A	AX.89616535	7.11E+08	0.06	-6.47	5.27	0.09
E1	GY	3A	AX.89513989	7.12E+08	0.21	2.91	3.32	0.06
E1	GY	3A	AX.89727293	6.93E+07	0.33	-2.43	3.14	0.05
E1	GY	3A	AX.89312843	5.67E+07	0.38	-2.49	3.45	0.06
E1	GY	3A	AX.89534837	5.70E+07	0.38	-2.49	3.45	0.06
E1	GY	3A	AX.89727701	6.25E+08	0.42	2.32	3.15	0.06
E1	GY	3A	AX.89313868	5.67E+07	0.39	-2.51	3.51	0.06
E1	GY	3A	AX.89603026	5.57E+08	0.05	-5.95	3.66	0.07
E1	GY	3A	AX.89546950	5.68E+07	0.37	-2.50	3.45	0.06
E1	GY	3A	AX.89372086	5.57E+07	0.37	-2.50	3.45	0.06
E1	SPM2	3A	AX.89347138	2.36E+07	0.12	29.78	4.01	0.07
E1	GY	3A	AX.89356377	5.62E+07	0.37	-2.50	3.45	0.06
E1	GY	3A	AX.89477720	5.71E+07	0.38	-2.49	3.45	0.06
E1	SPM2	3A	AX.89745749	2.36E+07	0.12	28.09	3.73	0.07
E1	GY	3A	AX.89595635	5.62E+07	0.37	-2.50	3.45	0.06
E1	GY	3A	AX.89753188	5.61E+07	0.37	-2.50	3.45	0.06
E1	GY	3A	AX.89514349	5.70E+07	0.38	-2.49	3.45	0.06
E1	GY	3A	AX.89631332	6.60E+08	0.24	2.83	3.41	0.06
E1	GY	3A	AX.89742109	5.67E+07	0.37	-2.50	3.45	0.06
E1	GY	3A	AX.89502639	5.68E+07	0.37	-2.50	3.45	0.06
E1	GY	3A	AX.89644393	6.95E+07	0.33	-2.43	3.14	0.05
E1	GY	3A	AX.89669221	7.11E+08	0.20	2.83	3.12	0.05
E1	GY	3A	AX.89549204	5.64E+07	0.38	-2.49	3.45	0.06
E1	GY	3A	AX.89409977	9.90E+04	0.40	-2.96	4.73	0.07
E1	GY	3A	AX.89316834	5.67E+07	0.37	-2.50	3.45	0.06
E1	GY	3A	AX.89374417	7.11E+08	0.24	3.52	5.00	0.09
E1	GY	3A	AX.89421469	5.64E+07	0.38	-2.49	3.45	0.06
E1	SPM2	3A	AX.89506554	2.36E+07	0.12	28.09	3.73	0.07
E1	GY	3A	AX.89467391	7.19E+08	0.46	-2.29	3.12	0.04
E1	GY	3A	AX.89573099	5.67E+07	0.37	-2.50	3.45	0.06
E1	GY	3A	AX.89749604	5.64E+07	0.37	-2.50	3.45	0.06
E1	GY	3A	AX.89537064	6.93E+07	0.33	-2.43	3.14	0.05
E1	GY	3A	AX.89768286	7.11E+08	0.06	-6.47	5.27	0.09
E1	GY	3A	AX.89538943	7.11E+08	0.07	-5.90	5.31	0.09
E1	GY	3A	AX.89744040	5.16E+08	0.05	-5.81	3.79	0.07
E1	GY	3A	AX.89375233	2.28E+05	0.35	2.40	3.14	0.06
E1	GY	3A	AX.89386200	2.27E+05	0.49	2.31	3.18	0.05
E1	GY	3A	AX.89550109	5.70E+07	0.38	-2.49	3.45	0.06
E1	GY	3A	AX.86161945	7.19E+08	0.46	-2.29	3.12	0.04

E1	GY	3A	AX.89411438	5.70E+07	0.37	-2.50	3.45	0.06
E1	GY	3A	AX.89411438	5.70E+07	0.37	-2.50	3.45	0.06
E1	GY	3A	AX.89645968	5.71E+07	0.38	-2.49	3.45	0.06
E1	GY	3A	AX.89528809	7.11E+08	0.08	-4.91	4.04	0.06
E1	GY	3A	AX.89634345	7.11E+08	0.06	-5.83	4.10	0.07
E1	GY	3A	AX.89496066	6.93E+07	0.33	-2.43	3.14	0.05
E1	GY	3A	AX.89340321	1.15E+07	0.32	2.41	3.04	0.05
E1	GY	3A	AX.89340321	1.15E+07	0.32	2.41	3.04	0.05
E1	GY	3A	AX.89468914	5.68E+07	0.37	-2.50	3.45	0.06
E1	GY	3A	AX.89646447	7.11E+08	0.06	-6.47	5.27	0.09
E1	GY	3A	AX.89726966	5.67E+07	0.37	-2.50	3.45	0.06
E1	GY	3A	AX.89726966	5.67E+07	0.37	-2.50	3.45	0.06
E1	GY	3A	AX.89318922	5.67E+07	0.37	-2.50	3.45	0.06
E1	GY	3A	AX.89637550	1.60E+05	0.50	-2.30	3.17	0.04
E1	GY	3A	AX.89672273	5.61E+07	0.37	-2.50	3.45	0.06
E1	GY	3A	AX.89505805	6.60E+08	0.24	2.83	3.41	0.06
E1	GY	3A	AX.89529884	5.62E+07	0.37	-2.50	3.45	0.06
E1	GY	3A	AX.89554883	5.67E+07	0.38	-2.52	3.50	0.06
E1	GY	3A	AX.89484211	5.70E+07	0.38	-2.49	3.45	0.06
E1	GY	3A	AX.89484211	5.70E+07	0.38	-2.49	3.45	0.06
E1	GY	3A	AX.89484890	1.16E+07	0.32	2.41	3.04	0.05
E1	GY	3A	AX.89402335	7.11E+08	0.24	3.52	5.00	0.09
E1	GY	3A	AX.89585123	5.16E+08	0.05	-5.81	3.79	0.07
E1	GY	3A	AX.89320950	6.59E+08	0.21	3.18	3.86	0.07
E1	GY	3A	AX.89402456	7.11E+08	0.24	3.52	5.00	0.09
E1	GY	3A	AX.89699257	5.67E+07	0.37	-2.47	3.34	0.05
E1	GY	3A	AX.89777576	6.25E+08	0.24	2.67	3.10	0.04
E1	GY	3A	AX.89637185	5.62E+07	0.36	-2.50	3.41	0.06
E1	GY	3A	AX.89637185	5.62E+07	0.36	-2.50	3.41	0.06
E1	GY	3A	AX.89355033	7.11E+08	0.08	-4.92	4.05	0.06
E1	GY	3A	AX.89654923	6.93E+07	0.33	-2.43	3.14	0.05
E1	GY	3A	AX.89748055	5.64E+07	0.37	-2.50	3.45	0.06
E1	GY	3A	AX.89748055	5.64E+07	0.37	-2.50	3.45	0.06
E1	GY	3A	AX.89654132	6.60E+08	0.24	2.83	3.41	0.06
E1	GY	3A	AX.89570766	5.67E+07	0.37	-2.50	3.45	0.06
E1	GY	3A	AX.89570766	5.67E+07	0.37	-2.50	3.45	0.06
E1	GY	3A	AX.89427924	5.61E+07	0.37	-2.50	3.45	0.06
E1	GY	3A	AX.89684173	7.12E+08	0.20	2.83	3.12	0.05
E1	GY	3A	AX.89591634	2.13E+05	0.35	2.40	3.14	0.06
E1	GY	3A	AX.89733840	2.07E+05	0.35	2.40	3.14	0.06
E1	GY	3A	AX.89461923	5.61E+07	0.38	-2.52	3.50	0.06
E1	GY	3A	AX.89416520	5.70E+07	0.38	-2.49	3.45	0.06
E1	GY	3A	AX.89416520	5.70E+07	0.38	-2.49	3.45	0.06
E1	GY	3A	AX.89357150	7.09E+08	0.07	-4.53	3.01	0.05
E1	SPM2	3A	AX.89419170	2.34E+07	0.10	30.49	3.65	0.07
E1	SPM2	3A	AX.89311041	2.34E+07	0.10	30.49	3.65	0.07
E1	GY	3A	AX.89763365	6.59E+08	0.29	2.60	3.30	0.06
E1	GY	3A	AX.89405834	6.34E+08	0.47	-2.47	3.56	0.06
E1	GY	3A	AX.89690421	7.14E+08	0.38	2.30	3.00	0.05
E1	GY	3A	AX.89325079	7.11E+08	0.07	-5.78	4.86	0.08

E1	GY	3A	AX.89716020	6.12E+08	0.23	-2.88	3.47	0.06
E1	GY	3A	AX.89358928	5.71E+07	0.38	-2.49	3.45	0.06
E1	GY	3A	AX.89417960	5.69E+07	0.37	-2.50	3.45	0.06
E1	GY	3A	AX.89417960	5.69E+07	0.37	-2.50	3.45	0.06
E1	GY	3A	AX.89740047	6.59E+08	0.28	2.51	3.06	0.05
E1	GY	3A	AX.89464016	5.64E+07	0.37	-2.50	3.45	0.06
E1	GY	3A	AX.89395219	5.67E+07	0.38	-2.40	3.22	0.05
E1	GY	3A	AX.89603026	5.57E+08	0.05	-5.95	3.66	0.07
E1	GY	3A	AX.89336503	2.11E+05	0.35	2.40	3.14	0.06
E1	GY	3A	AX.89431563	7.12E+08	0.20	2.83	3.12	0.05
E1	GY	3A	AX.89766111	5.68E+07	0.38	-2.49	3.45	0.06
E1	GY	3A	AX.89630559	2.13E+05	0.35	2.38	3.10	0.05
E1	GY	3A	AX.89595635	5.62E+07	0.37	-2.50	3.45	0.06
E1	GY	3A	AX.89326987	5.64E+07	0.38	-2.49	3.45	0.06
E1	GY	3A	AX.89360803	5.62E+07	0.38	-2.49	3.45	0.06
E1	GY	3A	AX.89541040	5.16E+08	0.05	-5.81	3.79	0.07
E1	GY	3A	AX.89541040	5.16E+08	0.05	-5.81	3.79	0.07
E1	GY	3A	AX.89579168	8.74E+06	0.39	2.50	3.47	0.06
E1	GY	3A	AX.89619765	6.59E+08	0.21	3.18	3.86	0.07
E1	GY	3A	AX.89619765	6.59E+08	0.21	3.18	3.86	0.07
E1	GY	3A	AX.89644393	6.95E+07	0.33	-2.43	3.14	0.05
E1	GY	3A	AX.89706416	6.95E+07	0.33	-2.43	3.14	0.05
E1	GY	3A	AX.89706416	6.95E+07	0.33	-2.43	3.14	0.05
E1	GY	3A	AX.89421469	5.64E+07	0.38	-2.49	3.45	0.06
E1	GY	3A	AX.89467246	5.70E+07	0.38	-2.49	3.45	0.06
E1	GY	3A	AX.89755194	7.12E+08	0.20	2.83	3.12	0.05
E1	GY	3A	AX.89633282	1.31E+06	0.36	-2.35	3.04	0.04
E1	GY	3A	AX.89375233	2.28E+05	0.35	2.40	3.14	0.06
E1	GY	3A	AX.89617932	5.16E+08	0.05	-5.81	3.79	0.07
E1	GY	3A	AX.89528765	5.67E+07	0.37	-2.50	3.45	0.06
E1	GY	3A	AX.89528765	5.67E+07	0.37	-2.50	3.45	0.06
E1	GY	3A	AX.89399628	2.16E+05	0.35	2.40	3.14	0.06
E1	GY	3A	AX.89399628	2.16E+05	0.35	2.40	3.14	0.06
E1	GY	3A	AX.89732524	5.67E+07	0.38	-2.52	3.50	0.06
E1	GY	3A	AX.89411704	7.04E+08	0.30	2.50	3.12	0.04
E1	GY	3A	AX.89412117	5.16E+08	0.05	-5.81	3.79	0.07
E1	SPM2	3A	AX.89579477	9.57E+06	0.26	-19.18	3.20	0.05
E1	GY	3A	AX.89647537	7.11E+08	0.07	-5.96	4.84	0.08
E1	GY	3A	AX.89660075	9.67E+04	0.35	2.40	3.14	0.06
E1	GY	3A	AX.89401377	5.67E+07	0.37	-2.50	3.45	0.06
E1	GY	3A	AX.89697914	5.71E+07	0.38	-2.49	3.45	0.06
E1	GY	3A	AX.89470027	5.61E+07	0.37	-2.50	3.45	0.06
E1	GY	3A	AX.89470027	5.61E+07	0.37	-2.50	3.45	0.06
E1	GY	3A	AX.89635943	1.44E+08	0.09	-4.47	3.89	0.06
E1	GY	3A	AX.89635943	1.44E+08	0.09	-4.47	3.89	0.06
E1	GY	3A	AX.89579477	9.57E+06	0.26	-2.62	3.13	0.04
E1	GY	3A	AX.89698074	6.93E+07	0.33	-2.43	3.14	0.05
E1	GY	3A	AX.89471595	7.11E+08	0.07	-6.05	4.97	0.08
E1	GY	3A	AX.89731479	7.11E+08	0.06	-6.47	5.27	0.09
E1	SPM2	3A	AX.89587477	2.35E+07	0.09	33.71	4.02	0.07

E1	GY	3A	AX.89637093	5.70E+07	0.38	-2.49	3.45	0.06
E1	GY	3A	AX.89637093	5.70E+07	0.38	-2.49	3.45	0.06
E1	GY	3A	AX.89549948	5.70E+07	0.38	-2.49	3.45	0.06
E1	GY	3A	AX.89736209	5.61E+07	0.37	-2.50	3.45	0.06
E1	GY	3A	AX.89613894	7.11E+08	0.07	-6.16	5.13	0.08
E1	GY	3A	AX.89508580	5.61E+07	0.37	-2.50	3.45	0.06
E1	SPM2	3A	AX.89643343	7.10E+08	0.23	-19.30	3.06	0.05
E1	GY	3A	AX.89725109	7.11E+08	0.06	-6.47	5.27	0.09
E1	GY	3A	AX.89543452	1.14E+07	0.30	2.96	4.23	0.08
E1	GY	3A	AX.89683629	6.59E+08	0.28	2.73	3.52	0.06
E1	GY	3A	AX.89700698	2.08E+05	0.35	2.40	3.14	0.06
E1	GY	3A	AX.89356377	5.62E+07	0.37	-2.50	3.45	0.06
E1	GY	3A	AX.89591634	2.13E+05	0.35	2.40	3.14	0.06
E1	GY	3A	AX.89427948	5.67E+07	0.37	-2.50	3.45	0.06
E1	GY	3A	AX.89775222	5.61E+07	0.37	-2.50	3.45	0.06
E1	GY	3A	AX.89591989	7.12E+08	0.20	2.83	3.12	0.05
E1	GY	3A	AX.89417627	7.12E+08	0.20	2.83	3.12	0.05
E1	GY	3A	AX.89676793	5.70E+07	0.38	-2.49	3.45	0.06
E1	GY	3A	AX.89334033	5.71E+07	0.38	-2.49	3.45	0.06
E1	GY	3A	AX.89616067	5.68E+07	0.37	-2.50	3.45	0.06
E1	GY	3A	AX.89369804	5.57E+07	0.37	-2.50	3.45	0.06
E1	GY	3A	AX.89552884	7.11E+08	0.11	-5.49	6.26	0.10
E1	GY	3A	AX.89371144	7.11E+08	0.06	-6.47	5.27	0.09
E1	GY	3A	AX.89678328	5.62E+07	0.38	-2.49	3.45	0.06
E1	GY	3A	AX.89678328	5.62E+07	0.38	-2.49	3.45	0.06
E1	GY	3A	AX.89512498	5.68E+07	0.37	-2.50	3.45	0.06
E1	SPM2	3A	AX.89333265	2.36E+07	0.12	28.09	3.73	0.07
E1	GY	3A	AX.89727701	6.25E+08	0.42	2.32	3.15	0.06
E1	GY	3A	AX.89577247	5.67E+07	0.37	-2.50	3.45	0.06
E1	GY	3A	AX.89463636	7.11E+08	0.07	-6.05	4.97	0.08
E1	GY	3A	AX.89335688	7.11E+08	0.07	-5.26	4.12	0.07
E1	GY	3A	AX.89476226	5.70E+07	0.38	-2.49	3.45	0.06
E1	GY	3A	AX.89371520	5.70E+07	0.38	-2.49	3.45	0.06
E1	GY	3A	AX.89485992	5.64E+07	0.37	-2.50	3.45	0.06
E1	GY	3A	AX.89407423	5.38E+07	0.07	-4.65	3.14	0.06
E1	GY	3A	AX.89629820	5.28E+08	0.05	-6.25	3.98	0.08
E1	GY	3A	AX.89326089	5.67E+07	0.37	-2.50	3.45	0.06
E1	GY	3A	AX.89464016	5.64E+07	0.37	-2.50	3.45	0.06
E1	GY	3A	AX.89705622	5.64E+07	0.37	-2.50	3.45	0.06
E1	GY	3A	AX.89464249	4.29E+05	0.35	-2.38	3.10	0.04
E1	GY	3A	AX.89539719	5.67E+07	0.37	-2.50	3.45	0.06
E1	GY	3A	AX.89314273	5.17E+08	0.05	-5.27	3.21	0.06
E1	GY	3A	AX.89372086	5.57E+07	0.37	-2.50	3.45	0.06
E1	GY	3A	AX.89626555	6.59E+08	0.28	2.73	3.52	0.06
E1	GY	3A	AX.89559867	5.67E+07	0.38	-2.49	3.45	0.06
E1	GY	3A	AX.89559867	5.67E+07	0.38	-2.49	3.45	0.06
E1	GY	3A	AX.89526633	5.17E+08	0.05	-6.25	3.98	0.08
E1	SPM2	3A	AX.89379585	2.34E+07	0.10	30.49	3.65	0.07
E1	GY	3A	AX.89397074	5.61E+07	0.38	-2.49	3.45	0.06
E1	GY	3A	AX.89397074	5.61E+07	0.38	-2.49	3.45	0.06

E1	GY	3A	AX.89502635	5.71E+07	0.39	-2.51	3.51	0.06
E1	GY	3A	AX.89444324	7.12E+08	0.21	2.98	3.45	0.06
E1	GY	3A	AX.89445155	5.67E+07	0.37	-2.50	3.45	0.06
E1	GY	3A	AX.89362464	6.59E+08	0.28	2.51	3.06	0.05
E1	GY	3A	AX.89467391	7.19E+08	0.46	-2.29	3.12	0.04
E1	GY	3A	AX.89745936	7.11E+08	0.20	2.83	3.12	0.05
E1	GY	3A	AX.89720264	7.11E+08	0.08	-4.91	4.04	0.06
E1	GY	3A	AX.89537064	6.93E+07	0.33	-2.43	3.14	0.05
E1	GY	3A	AX.89768286	7.11E+08	0.06	-6.47	5.27	0.09
E1	GY	3A	AX.89363017	7.11E+08	0.06	-5.26	3.66	0.06
E1	GY	3A	AX.89573360	7.11E+08	0.20	2.83	3.12	0.05
E1	GY	3A	AX.89573360	7.11E+08	0.20	2.83	3.12	0.05
E1	GY	3A	AX.89445873	5.57E+08	0.05	-5.95	3.66	0.07
E1	GY	3A	AX.89445873	5.57E+08	0.05	-5.95	3.66	0.07
E1	GY	3A	AX.86161945	7.19E+08	0.46	-2.29	3.12	0.04
E1	GY	3A	AX.89649354	7.11E+08	0.06	-6.47	5.27	0.09
E1	GY	3A	AX.89649354	7.11E+08	0.06	-6.47	5.27	0.09
E1	GY	3A	AX.89504535	6.25E+08	0.48	-2.42	3.44	0.05
E1	GY	3A	AX.89602017	5.61E+07	0.38	-2.49	3.45	0.06
E1	GY	3A	AX.89517819	6.93E+07	0.33	-2.43	3.14	0.05
E1	GY	3A	AX.89672273	5.61E+07	0.37	-2.50	3.45	0.06
E1	GY	3A	AX.89697914	5.71E+07	0.38	-2.49	3.45	0.06
E1	GY	3A	AX.89734012	5.64E+08	0.06	-5.29	3.46	0.07
E1	GY	3A	AX.89554883	5.67E+07	0.38	-2.52	3.50	0.06
E1	GY	3A	AX.89579477	9.57E+06	0.26	-2.62	3.13	0.04
E1	GY	3A	AX.89530250	5.67E+07	0.37	-2.50	3.45	0.06
E1	GY	3A	AX.89742601	6.59E+08	0.21	3.27	4.13	0.07
E1	GY	3A	AX.89556622	6.59E+08	0.28	2.52	3.10	0.05
E1	GY	3A	AX.89723196	1.14E+07	0.32	2.43	3.06	0.05
E1	GY	3A	AX.89418431	5.65E+08	0.06	-5.29	3.46	0.07
E1	GY	3A	AX.89484890	1.16E+07	0.32	2.41	3.04	0.05
E1	GY	3A	AX.89541934	1.14E+07	0.44	2.25	3.00	0.05
E1	GY	3A	AX.89699862	1.18E+07	0.43	2.57	3.77	0.07
E1	GY	3A	AX.89699862	1.18E+07	0.43	2.57	3.77	0.07
E1	GY	3A	AX.89379315	2.06E+05	0.35	2.40	3.14	0.06
E1	GY	3A	AX.89543199	5.06E+08	0.06	-4.93	3.08	0.06
E1	GY	3A	AX.89566650	7.11E+08	0.26	2.68	3.25	0.06
E1	GY	3A	AX.89566650	7.11E+08	0.26	2.68	3.25	0.06
E1	GY	3A	AX.89509264	5.16E+08	0.05	-5.81	3.79	0.07
E1	GY	3A	AX.89654132	6.60E+08	0.24	2.83	3.41	0.06
E1	GY	3A	AX.89450721	6.34E+08	0.41	2.41	3.34	0.06
E1	GY	3A	AX.89486754	2.25E+05	0.49	-2.40	3.41	0.05
E1	GY	3A	AX.89486754	2.25E+05	0.49	-2.40	3.41	0.05
E1	GY	3A	AX.89450928	7.95E+06	0.40	-2.36	3.18	0.04
E1	GY	3A	AX.89450928	7.95E+06	0.40	-2.36	3.18	0.04
E1	GY	3A	AX.89416077	5.70E+07	0.38	-2.49	3.45	0.06
E1	GY	3A	AX.89762555	7.19E+08	0.46	-2.29	3.12	0.04
E1	GY	3A	AX.89369723	6.93E+07	0.33	-2.43	3.14	0.05
E1	GPS	3A	AX.89445039	7.11E+08	0.06	-2.38	3.13	0.05
E1	GY	3A	AX.89513989	7.12E+08	0.21	2.91	3.32	0.06

E1	GY	3A	AX.89556938	6.59E+08	0.29	2.60	3.30	0.06
E1	GY	3A	AX.89475487	5.67E+07	0.37	-2.50	3.45	0.06
E1	GY	3A	AX.89452352	1.25E+07	0.33	-2.41	3.11	0.04
E1	GY	3A	AX.89452352	1.25E+07	0.33	-2.41	3.11	0.04
E1	GY	3A	AX.89313133	5.67E+07	0.38	-2.49	3.45	0.06
E1	GY	3A	AX.89756161	5.62E+07	0.38	-2.49	3.45	0.06
E1	GY	3A	AX.89489326	7.11E+08	0.07	-4.69	3.19	0.05
E1	GY	3A	AX.89680239	6.25E+08	0.47	-2.27	3.07	0.05
E1	GY	3A	AX.89740185	5.57E+07	0.38	-2.49	3.45	0.06
E1	GY	3A	AX.89740185	5.57E+07	0.38	-2.49	3.45	0.06
E1	GY	3A	AX.89629820	5.28E+08	0.05	-6.25	3.98	0.08
E1	GY	3A	AX.89347777	5.68E+07	0.37	-2.50	3.45	0.06
E1	GY	3A	AX.89347777	5.68E+07	0.37	-2.50	3.45	0.06
E1	GY	3A	AX.89464249	4.29E+05	0.35	-2.38	3.10	0.04
E1	GPS	3A	AX.89616535	7.11E+08	0.06	-2.38	3.13	0.05
E1	GY	3A	AX.89314273	5.17E+08	0.05	-5.27	3.21	0.06
E1	SPM2	3A	AX.89369015	2.35E+07	0.09	31.32	3.68	0.07
E1	GPS	3A	AX.89463636	7.11E+08	0.07	-2.32	3.18	0.05
E1	GY	3A	AX.89536450	5.68E+07	0.38	-2.49	3.45	0.06
E1	GY	3A	AX.89372891	6.93E+07	0.33	-2.43	3.14	0.05
E1	GY	3A	AX.89762173	5.71E+07	0.38	-2.49	3.45	0.06
E1	GY	3A	AX.89755885	5.64E+07	0.38	-2.49	3.45	0.06
E1	GY	3A	AX.89742109	5.67E+07	0.37	-2.50	3.45	0.06
E1	GY	3A	AX.89765606	7.11E+08	0.24	3.52	5.00	0.09
E1	GY	3A	AX.89409977	9.90E+04	0.40	-2.96	4.73	0.07
E1	GY	3A	AX.89706491	6.59E+08	0.28	2.73	3.52	0.06
E1	GY	3A	AX.89706491	6.59E+08	0.28	2.73	3.52	0.06
E1	GY	3A	AX.89316834	5.67E+07	0.37	-2.50	3.45	0.06
E1	GY	3A	AX.89445039	7.11E+08	0.06	-6.47	5.27	0.09
E1	GY	3A	AX.89666899	6.59E+08	0.21	3.27	4.13	0.07
E1	GY	3A	AX.89666899	6.59E+08	0.21	3.27	4.13	0.07
E1	GY	3A	AX.89749604	5.64E+07	0.37	-2.50	3.45	0.06
E1	GY	3A	AX.89579551	7.11E+08	0.09	-4.64	4.16	0.07
E1	GY	3A	AX.89579551	7.11E+08	0.09	-4.64	4.16	0.07
E1	GY	3A	AX.89528629	6.25E+08	0.48	-2.52	3.70	0.06
E1	GY	3A	AX.89528629	6.25E+08	0.48	-2.52	3.70	0.06
E1	GY	3A	AX.89528809	7.11E+08	0.08	-4.91	4.04	0.06
E1	GY	3A	AX.89496066	6.93E+07	0.33	-2.43	3.14	0.05
E1	GY	3A	AX.89646447	7.11E+08	0.06	-6.47	5.27	0.09
E1	GY	3A	AX.89723050	5.61E+07	0.38	-2.49	3.45	0.06
E1	GY	3A	AX.89701379	5.20E+08	0.05	-7.82	5.92	0.11
E1	GY	3A	AX.89748618	5.67E+07	0.37	-2.50	3.45	0.06
E1	GY	3A	AX.89748618	5.67E+07	0.37	-2.50	3.45	0.06
E1	GY	3A	AX.89423759	5.62E+07	0.38	-2.52	3.50	0.06
E1	GY	3A	AX.89505805	6.60E+08	0.24	2.83	3.41	0.06
E1	GY	3A	AX.89707395	1.25E+07	0.33	-2.41	3.11	0.04
E1	GY	3A	AX.89707395	1.25E+07	0.33	-2.41	3.11	0.04
E1	GY	3A	AX.89753942	7.12E+08	0.20	2.83	3.12	0.05
E1	GY	3A	AX.89413621	5.67E+07	0.37	-2.50	3.45	0.06
E1	GY	3A	AX.89413621	5.67E+07	0.37	-2.50	3.45	0.06

E1	GY	3A	AX.89564968	6.59E+08	0.29	2.50	3.09	0.05
E1	GY	3A	AX.89353961	5.61E+07	0.38	-2.49	3.45	0.06
E1	GY	3A	AX.89424557	5.71E+07	0.38	-2.49	3.45	0.06
E1	GY	3A	AX.89581641	5.68E+07	0.37	-2.50	3.45	0.06
E1	GY	3A	AX.89777576	6.25E+08	0.24	2.67	3.10	0.04
E1	GY	3A	AX.89650770	5.68E+07	0.38	-2.49	3.45	0.06
E1	GY	3A	AX.89711077	7.11E+08	0.24	3.57	5.12	0.09
E1	GY	3A	AX.89725109	7.11E+08	0.06	-6.47	5.27	0.09
E1	GY	3A	AX.89415949	7.11E+08	0.07	-6.05	4.97	0.08
E1	GY	3A	AX.89461796	5.57E+08	0.05	-5.95	3.66	0.07
E1	GY	3A	AX.89461796	5.57E+08	0.05	-5.95	3.66	0.07
E1	GY	3A	AX.89325079	7.11E+08	0.07	-5.78	4.86	0.08
E1	GY	3A	AX.89716020	6.12E+08	0.23	-2.88	3.47	0.06
E1	GY	3A	AX.89359371	5.61E+07	0.38	-2.49	3.45	0.06
E1	GY	3A	AX.89359371	5.61E+07	0.38	-2.49	3.45	0.06
E1	GY	3A	AX.89740047	6.59E+08	0.28	2.51	3.06	0.05
E1	GY	3A	AX.89607034	5.64E+07	0.37	-2.50	3.45	0.06
E1	GY	3A	AX.89655544	5.70E+07	0.38	-2.49	3.45	0.06
E1	GY	3A	AX.89655544	5.70E+07	0.38	-2.49	3.45	0.06
E1	GY	3A	AX.89360487	5.67E+07	0.37	-2.50	3.45	0.06
E1	GY	3A	AX.89741338	7.11E+08	0.07	-4.69	3.19	0.05
E1	GY	3A	AX.89753115	2.21E+05	0.49	-2.40	3.41	0.05
E1	GY	3A	AX.89536603	5.70E+07	0.38	-2.49	3.45	0.06
E1	GY	3A	AX.89396715	6.59E+08	0.29	2.60	3.30	0.06
E1	GY	3A	AX.89722953	1.16E+07	0.21	2.74	3.00	0.05
E1	GY	3A	AX.89719431	5.70E+07	0.38	-2.49	3.45	0.06
E1	GY	3A	AX.89684368	7.12E+08	0.20	2.83	3.12	0.05
E1	GY	3A	AX.89658110	5.70E+07	0.38	-2.49	3.45	0.06
E1	GY	3A	AX.89363091	5.61E+07	0.38	-2.49	3.45	0.06
E1	GY	3A	AX.89363091	5.61E+07	0.38	-2.49	3.45	0.06
E1	GY	3A	AX.89617932	5.16E+08	0.05	-5.81	3.79	0.07
E1	GY	3A	AX.89732198	7.11E+08	0.07	-5.26	4.12	0.07
E1	GY	3A	AX.89732198	7.11E+08	0.07	-5.26	4.12	0.07
E1	GY	3A	AX.89626419	5.71E+07	0.42	-2.55	3.69	0.06
E1	GY	3A	AX.89500862	5.71E+07	0.37	-2.63	3.75	0.06
E1	GY	3A	AX.89500862	5.71E+07	0.37	-2.63	3.75	0.06
E1	GY	3A	AX.89647537	7.11E+08	0.07	-5.96	4.84	0.08
E1	GPM2	3A	AX.89395219	5.67E+07	0.38	-632.10	3.36	0.06
E1	GPM2	3A	AX.89729691	7.43E+06	0.49	-615.41	3.38	0.05
E1	GPM2	3A	AX.89628511	6.90E+06	0.37	-610.03	3.13	0.05
E1	GPM2	3A	AX.89732524	5.67E+07	0.38	-609.23	3.15	0.05
E1	GPM2	3A	AX.89559867	5.67E+07	0.38	-639.74	3.44	0.06
E1	GPM2	3A	AX.89334290	1.15E+07	0.23	742.96	3.50	0.06
E1	GPM2	3A	AX.89603026	5.57E+08	0.05	-1485.41	3.48	0.06
E1	GPM2	3A	AX.89354663	9.68E+04	0.35	-710.73	4.00	0.06
E1	GY	3A	AX.89653072	5.70E+07	0.37	-2.50	3.45	0.06
E1	GPM2	3A	AX.89546950	5.68E+07	0.37	-654.03	3.55	0.06
E1	GPM2	3A	AX.89450866	1.18E+07	0.33	652.45	3.35	0.06
E1	GPM2	3A	AX.89411704	7.04E+08	0.30	679.84	3.44	0.05
E1	GY	3A	AX.89471074	7.19E+08	0.46	-2.29	3.12	0.04

E1	GPM2	3A	AX.89529884	5.62E+07	0.37	-654.03	3.55	0.06
E1	GPM2	3A	AX.89672273	5.61E+07	0.37	-654.03	3.55	0.06
E1	GY	3A	AX.89530750	7.11E+08	0.06	-6.47	5.27	0.09
E1	GY	3A	AX.89541934	1.14E+07	0.44	2.25	3.00	0.05
E1	GY	3A	AX.89731479	7.11E+08	0.06	-6.47	5.27	0.09
E1	GPM2	3A	AX.89427948	5.67E+07	0.37	-654.03	3.55	0.06
E1	GPM2	3A	AX.89637185	5.62E+07	0.36	-682.24	3.78	0.06
E1	GY	3A	AX.89577141	6.40E+08	0.05	-5.34	3.29	0.05
E1	GY	3A	AX.89577141	6.40E+08	0.05	-5.34	3.29	0.05
E1	GPM2	3A	AX.89413621	5.67E+07	0.37	-654.03	3.55	0.06
E1	GY	3A	AX.89736209	5.61E+07	0.37	-2.50	3.45	0.06
E1	GPM2	3A	AX.89508580	5.61E+07	0.37	-654.03	3.55	0.06
E1	GY	3A	AX.89532206	5.62E+07	0.38	-2.49	3.45	0.06
E1	GY	3A	AX.89532206	5.62E+07	0.38	-2.49	3.45	0.06
E1	GPM2	3A	AX.89497516	5.70E+07	0.38	-639.74	3.44	0.06
E1	GY	3A	AX.89439144	1.10E+07	0.32	2.41	3.04	0.05
E1	GY	3A	AX.89439144	1.10E+07	0.32	2.41	3.04	0.05
E1	GY	3A	AX.89497516	5.70E+07	0.38	-2.49	3.45	0.06
E1	GPM2	3A	AX.89765950	7.17E+06	0.34	708.02	3.92	0.07
E1	GPM2	3A	AX.89458621	5.67E+07	0.37	-654.03	3.55	0.06
E1	GY	3A	AX.89417627	7.12E+08	0.20	2.83	3.12	0.05
E1	GPM2	3A	AX.89373966	5.64E+07	0.37	-654.03	3.55	0.06
E1	GY	3A	AX.89676793	5.70E+07	0.38	-2.49	3.45	0.06
E1	GY	3A	AX.89616067	5.68E+07	0.37	-2.50	3.45	0.06
E1	GPM2	3A	AX.89502635	5.71E+07	0.39	-595.67	3.06	0.05
E1	GPM2	3A	AX.89412589	5.62E+07	0.38	-639.74	3.44	0.06
E1	GY	3A	AX.89552884	7.11E+08	0.11	-5.49	6.26	0.10
E1	GPM2	3A	AX.89753115	2.21E+05	0.49	-657.87	3.80	0.06
E1	GY	3A	AX.89763365	6.59E+08	0.29	2.60	3.30	0.06
E1	GPM2	3A	AX.89642626	7.09E+08	0.10	-1032.15	3.48	0.06
E1	GPM2	3A	AX.89357150	7.09E+08	0.07	-1191.77	3.13	0.05
E1	GPM2	3A	AX.89616067	5.68E+07	0.37	-654.03	3.55	0.06
E1	GPM2	3A	AX.89391530	1.60E+06	0.33	-685.54	3.64	0.06
E1	GY	3A	AX.89690421	7.14E+08	0.38	2.30	3.00	0.05
E1	GPM2	3A	AX.89392516	2.27E+05	0.50	665.26	3.87	0.06
E1	GY	3A	AX.89724076	7.19E+08	0.46	-2.29	3.12	0.04
E1	GY	3A	AX.89724076	7.19E+08	0.46	-2.29	3.12	0.04
E1	GY	3A	AX.89406798	2.06E+05	0.35	2.40	3.14	0.06
E1	GY	3A	AX.89406798	2.06E+05	0.35	2.40	3.14	0.06
E1	GY	3A	AX.89577247	5.67E+07	0.37	-2.50	3.45	0.06
E1	GPM2	3A	AX.89468093	1.18E+07	0.29	642.67	3.10	0.05
E1	GPM2	3A	AX.89660760	1.52E+06	0.34	-624.90	3.18	0.05
E1	GPM2	3A	AX.89484211	5.70E+07	0.38	-639.74	3.44	0.06
E1	GPM2	3A	AX.89318922	5.67E+07	0.37	-654.03	3.55	0.06
E1	GPM2	3A	AX.89623534	1.39E+06	0.36	-663.27	3.58	0.05
E1	GY	3A	AX.89326089	5.67E+07	0.37	-2.50	3.45	0.06
E1	GY	3A	AX.89705622	5.64E+07	0.37	-2.50	3.45	0.06
E1	GY	3A	AX.89546950	5.68E+07	0.37	-2.50	3.45	0.06
E1	GPM2	3A	AX.89655417	6.26E+06	0.41	-594.56	3.10	0.05
E1	GY	3A	AX.89626555	6.59E+08	0.28	2.73	3.52	0.06

E1	GY	3A	AX.89753188	5.61E+07	0.37	-2.50	3.45	0.06
E1	GY	3A	AX.89514349	5.70E+07	0.38	-2.49	3.45	0.06
E1	GY	3A	AX.89604070	5.70E+07	0.38	-2.49	3.45	0.06
E1	GY	3A	AX.89432808	5.58E+07	0.37	-2.50	3.45	0.06
E1	GPM2	3A	AX.89553150	4.22E+05	0.37	-617.82	3.22	0.05
E1	GPM2	3A	AX.89531199	5.57E+07	0.37	-654.03	3.55	0.06
E1	GPM2	3A	AX.89551243	6.23E+08	0.23	705.86	3.21	0.05
E1	GY	3A	AX.89564685	5.17E+08	0.05	-6.25	3.98	0.08
E1	GPM2	3A	AX.89359371	5.61E+07	0.38	-639.74	3.44	0.06
E1	GY	3A	AX.89445155	5.67E+07	0.37	-2.50	3.45	0.06
E1	GPS	3A	AX.89552884	7.11E+08	0.11	-1.98	3.58	0.06
E1	GY	3A	AX.89745936	7.11E+08	0.20	2.83	3.12	0.05
E1	GPS	3A	AX.89446558	7.11E+08	0.07	-2.32	3.18	0.05
E1	GY	3A	AX.89631354	5.16E+08	0.05	-5.81	3.79	0.07
E1	GY	3A	AX.89631354	5.16E+08	0.05	-5.81	3.79	0.07
E1	GPM2	3A	AX.89389285	1.18E+07	0.33	652.45	3.35	0.06
E1	GPM2	3A	AX.89461923	5.61E+07	0.38	-609.23	3.15	0.05
E1	GPM2	3A	AX.89652632	1.04E+07	0.23	686.44	3.02	0.05
E1	GPS	3A	AX.89471595	7.11E+08	0.07	-2.32	3.18	0.05
E1	GPS	3A	AX.89530750	7.11E+08	0.06	-2.38	3.13	0.05
E1	GPS	3A	AX.89649354	7.11E+08	0.06	-2.38	3.13	0.05
E1	GPM2	3A	AX.89424557	5.71E+07	0.38	-639.74	3.44	0.06
E1	GY	3A	AX.89550109	5.70E+07	0.38	-2.49	3.45	0.06
E1	GPM2	3A	AX.89382764	1.38E+06	0.36	-663.27	3.58	0.05
E1	GY	3A	AX.89645968	5.71E+07	0.38	-2.49	3.45	0.06
E1	GY	3A	AX.89634345	7.11E+08	0.06	-5.83	4.10	0.07
E1	GY	3A	AX.89732524	5.67E+07	0.38	-2.52	3.50	0.06
E1	GPM2	3A	AX.89607034	5.64E+07	0.37	-654.03	3.55	0.06
E1	GY	3A	AX.89563227	7.10E+08	0.07	-4.87	3.40	0.06
E1	GY	3A	AX.89563227	7.10E+08	0.07	-4.87	3.40	0.06
E1	GY	3A	AX.89602017	5.61E+07	0.38	-2.49	3.45	0.06
E1	GPM2	3A	AX.89655544	5.70E+07	0.38	-639.74	3.44	0.06
E1	GPM2	3A	AX.89543993	6.23E+08	0.23	705.86	3.21	0.05
E1	GY	3A	AX.89423759	5.62E+07	0.38	-2.52	3.50	0.06
E1	GY	3A	AX.89660075	9.67E+04	0.35	2.40	3.14	0.06
E1	GY	3A	AX.89734012	5.64E+08	0.06	-5.29	3.46	0.07
E1	GPM2	3A	AX.89398270	5.67E+07	0.37	-654.03	3.55	0.06
E1	GPM2	3A	AX.89499517	5.70E+07	0.38	-639.74	3.44	0.06
E1	GPM2	3A	AX.89313133	5.67E+07	0.38	-639.74	3.44	0.06
E1	GPM2	3A	AX.89569458	7.26E+06	0.47	624.14	3.46	0.05
E1	GPS	3A	AX.89646447	7.11E+08	0.06	-2.38	3.13	0.05
E1	GY	3A	AX.89556622	6.59E+08	0.28	2.52	3.10	0.05
E1	GY	3A	AX.89418431	5.65E+08	0.06	-5.29	3.46	0.07
E1	GPM2	3A	AX.89322500	5.62E+07	0.38	-639.74	3.44	0.06
E1	GPM2	3A	AX.89415372	7.22E+06	0.39	605.86	3.16	0.04
E1	GPM2	3A	AX.89539719	5.67E+07	0.37	-654.03	3.55	0.06
E1	GPS	3A	AX.89725109	7.11E+08	0.06	-2.38	3.13	0.05
E1	GPM2	3A	AX.89684384	5.70E+07	0.38	-639.74	3.44	0.06
E1	GPM2	3A	AX.89604070	5.70E+07	0.38	-639.74	3.44	0.06
E1	GPM2	3A	AX.89676793	5.70E+07	0.38	-639.74	3.44	0.06

E1	GPM2	3A	AX.89409977	9.90E+04	0.40	-838.55	5.64	0.09
E1	GPM2	3A	AX.89338545	5.71E+07	0.38	-639.74	3.44	0.06
E1	GPM2	3A	AX.89346739	2.07E+05	0.34	-653.40	3.44	0.05
E1	GPM2	3A	AX.89645252	1.18E+07	0.30	636.43	3.08	0.05
E1	GPS	3A	AX.89371144	7.11E+08	0.06	-2.38	3.13	0.05
E1	GPM2	3A	AX.89378945	5.68E+07	0.37	-654.03	3.55	0.06
E1	GPM2	3A	AX.89416077	5.70E+07	0.38	-639.74	3.44	0.06
E1	GY	3A	AX.89683629	6.59E+08	0.28	2.73	3.52	0.06
E1	GPM2	3A	AX.89372086	5.57E+07	0.37	-654.03	3.55	0.06
E1	GY	3A	AX.89567118	5.67E+07	0.37	-2.50	3.45	0.06
E1	GY	3A	AX.89450721	6.34E+08	0.41	2.41	3.34	0.06
E1	GPM2	3A	AX.89536450	5.68E+07	0.38	-639.74	3.44	0.06
E1	GPM2	3A	AX.89490667	5.71E+07	0.39	-647.25	3.53	0.06
E1	GY	3A	AX.89733840	2.07E+05	0.35	2.40	3.14	0.06
E1	GY	3A	AX.89416353	7.11E+08	0.24	3.52	5.00	0.09
E1	GPM2	3A	AX.89530564	7.25E+06	0.41	609.68	3.25	0.05
E1	GPM2	3A	AX.89320189	5.61E+07	0.38	-639.74	3.44	0.06
E1	GPM2	3A	AX.89475487	5.67E+07	0.37	-654.03	3.55	0.06
E1	GPM2	3A	AX.89516008	6.90E+06	0.37	-610.03	3.13	0.05
E1	GPM2	3A	AX.89658110	5.70E+07	0.38	-639.74	3.44	0.06
E1	GPM2	3A	AX.89697914	5.71E+07	0.38	-639.74	3.44	0.06
E1	GY	3A	AX.89556938	6.59E+08	0.29	2.60	3.30	0.06
E1	GPM2	3A	AX.89755885	5.64E+07	0.38	-639.74	3.44	0.06
E1	GPM2	3A	AX.89554883	5.67E+07	0.38	-609.23	3.15	0.05
E1	GPM2	3A	AX.89408108	7.26E+06	0.45	670.93	3.90	0.06
E1	GY	3A	AX.89499517	5.70E+07	0.38	-2.49	3.45	0.06
E1	GY	3A	AX.89499517	5.70E+07	0.38	-2.49	3.45	0.06
E1	GPM2	3A	AX.89547815	1.89E+06	0.49	601.21	3.25	0.05
E1	GPS	3A	AX.89731479	7.11E+08	0.06	-2.38	3.13	0.05
E1	GPM2	3A	AX.89345333	5.62E+07	0.37	-654.03	3.55	0.06
E1	GPM2	3A	AX.89416520	5.70E+07	0.38	-639.74	3.44	0.06
E1	GPS	3A	AX.89652632	1.04E+07	0.23	1.33	3.07	0.05
E1	GY	3A	AX.89489326	7.11E+08	0.07	-4.69	3.19	0.05
E1	GPM2	3A	AX.89549204	5.64E+07	0.38	-639.74	3.44	0.06
E1	GPS	3A	AX.89716020	6.12E+08	0.23	-1.36	3.21	0.06
E1	GY	3A	AX.89680239	6.25E+08	0.47	-2.27	3.07	0.05
E1	GPM2	3A	AX.89530250	5.67E+07	0.37	-654.03	3.55	0.06
E1	GPM2	3A	AX.89626419	5.71E+07	0.42	-586.23	3.05	0.05
E1	GY	3A	AX.89766111	5.68E+07	0.38	-2.49	3.45	0.06
E1	GY	3A	AX.89559269	5.61E+07	0.39	-2.51	3.51	0.06
E1	GPM2	3A	AX.89437855	5.68E+07	0.38	-671.04	3.72	0.06
E1	GY	3A	AX.89741338	7.11E+08	0.07	-4.69	3.19	0.05
E1	GPM2	3A	AX.89468914	5.68E+07	0.37	-654.03	3.55	0.06
E1	GPM2	3A	AX.89363091	5.61E+07	0.38	-639.74	3.44	0.06
E1	GPM2	3A	AX.89421469	5.64E+07	0.38	-639.74	3.44	0.06
E1	GY	3A	AX.89765606	7.11E+08	0.24	3.52	5.00	0.09
E1	GPM2	3A	AX.89632417	1.14E+07	0.31	628.31	3.08	0.05
E1	GY	3A	AX.89597151	6.60E+08	0.24	2.83	3.41	0.06
E1	GY	3A	AX.89445039	7.11E+08	0.06	-6.47	5.27	0.09
E1	GPM2	3A	AX.89500862	5.71E+07	0.37	-666.93	3.67	0.06

E1	GPM2	3A	AX.89628180	1.92E+06	0.36	621.11	3.22	0.04
E1	GPM2	3A	AX.89486754	2.25E+05	0.49	-657.87	3.80	0.06
E1	GY	3A	AX.89598815	5.67E+07	0.37	-2.50	3.45	0.06
E1	GY	3A	AX.89626419	5.71E+07	0.42	-2.55	3.69	0.06
E1	GPM2	3A	AX.89580969	6.23E+08	0.23	705.86	3.21	0.05
E1	GPM2	3A	AX.89463578	3.72E+05	0.35	-690.21	3.80	0.06
E1	GPM2	3A	AX.89461667	7.27E+06	0.42	619.30	3.35	0.05
E1	GPM2	3A	AX.89645968	5.71E+07	0.38	-639.74	3.44	0.06
E1	GY	3A	AX.89723050	5.61E+07	0.38	-2.49	3.45	0.06
E1	GY	3A	AX.89637550	1.60E+05	0.50	-2.30	3.17	0.04
E1	GPM2	3A	AX.89577247	5.67E+07	0.37	-654.03	3.55	0.06
E1	GPM2	3A	AX.89753188	5.61E+07	0.37	-654.03	3.55	0.06
E1	GY	3A	AX.89490667	5.71E+07	0.39	-2.46	3.38	0.05
E1	GPM2	3A	AX.89358928	5.71E+07	0.38	-639.74	3.44	0.06
E1	GY	3A	AX.89529884	5.62E+07	0.37	-2.50	3.45	0.06
E1	GPM2	3A	AX.89421502	1.52E+06	0.33	-699.95	3.80	0.06
E1	GY	3A	AX.89600503	5.68E+07	0.38	-2.49	3.45	0.06
E1	GY	3A	AX.89600503	5.68E+07	0.38	-2.49	3.45	0.06
E1	GPM2	3A	AX.89467246	5.70E+07	0.38	-639.74	3.44	0.06
E1	GPM2	3A	AX.89347777	5.68E+07	0.37	-654.03	3.55	0.06
E1	GY	3A	AX.89698074	6.93E+07	0.33	-2.43	3.14	0.05
E1	GY	3A	AX.89458621	5.67E+07	0.37	-2.50	3.45	0.06
E1	GY	3A	AX.89753942	7.12E+08	0.20	2.83	3.12	0.05
E1	GPM2	3A	AX.89726966	5.67E+07	0.37	-654.03	3.55	0.06
E1	GY	3A	AX.89353961	5.61E+07	0.38	-2.49	3.45	0.06
E1	GPM2	3A	AX.89756161	5.62E+07	0.38	-639.74	3.44	0.06
E1	GPM2	3A	AX.89637093	5.70E+07	0.38	-639.74	3.44	0.06
E1	GPM2	3A	AX.89595635	5.62E+07	0.37	-654.03	3.55	0.06
E1	GY	3A	AX.89585123	5.16E+08	0.05	-5.81	3.79	0.07
E1	GY	3A	AX.89581641	5.68E+07	0.37	-2.50	3.45	0.06
E1	GY	3A	AX.89699257	5.67E+07	0.37	-2.47	3.34	0.05
E1	GPM2	3A	AX.89748055	5.64E+07	0.37	-654.03	3.55	0.06
E1	GPM2	3A	AX.89748618	5.67E+07	0.37	-654.03	3.55	0.06
E1	GY	3A	AX.89654923	6.93E+07	0.33	-2.43	3.14	0.05
E1	GPM2	3A	AX.89719431	5.70E+07	0.38	-639.74	3.44	0.06
E1	GPM2	3A	AX.89550109	5.70E+07	0.38	-639.74	3.44	0.06
E1	GY	3A	AX.89650770	5.68E+07	0.38	-2.49	3.45	0.06
E1	GPM2	3A	AX.89313868	5.67E+07	0.39	-595.67	3.06	0.05
E1	GPM2	3A	AX.89573099	5.67E+07	0.37	-654.03	3.55	0.06
E1	GY	3A	AX.89711077	7.11E+08	0.24	3.57	5.12	0.09
E1	GY	3A	AX.89392516	2.27E+05	0.50	2.51	3.67	0.06
E1	GY	3A	AX.89395219	5.67E+07	0.38	-2.40	3.22	0.05
E1	GY	3A	AX.89360487	5.67E+07	0.37	-2.50	3.45	0.06
E1	GY	3A	AX.89753115	2.21E+05	0.49	-2.40	3.41	0.05
E1	GY	3A	AX.89536603	5.70E+07	0.38	-2.49	3.45	0.06
E1	GPM2	3A	AX.89716020	6.12E+08	0.23	-774.05	3.76	0.06
E1	GY	3A	AX.89722953	1.16E+07	0.21	2.74	3.00	0.05
E1	GY	3A	AX.89719431	5.70E+07	0.38	-2.49	3.45	0.06
E1	GY	3A	AX.89632417	1.14E+07	0.31	2.73	3.71	0.07
E1	GY	3A	AX.89632417	1.14E+07	0.31	2.73	3.71	0.07

E1	GY	3A	AX.89755194	7.12E+08	0.20	2.83	3.12	0.05
E1	GY	3A	AX.89540978	5.44E+08	0.05	-6.25	3.98	0.08
E1	GPM2	3A	AX.89410438	2.25E+05	0.50	-580.45	3.06	0.04
E1	GPM2	3A	AX.89563517	7.20E+06	0.45	670.93	3.90	0.06
E1	GPM2	3A	AX.89452532	9.90E+04	0.35	-664.54	3.57	0.05
E1	GY	3A	AX.89653072	5.70E+07	0.37	-2.50	3.45	0.06
E1	GPM2	3A	AX.89476226	5.70E+07	0.38	-639.74	3.44	0.06
E1	GY	3A	AX.89723196	1.14E+07	0.32	2.43	3.06	0.05
E1	GPM2	3A	AX.89353961	5.61E+07	0.38	-639.74	3.44	0.06
E1	GPM2	3A	AX.89407423	5.38E+07	0.07	-1176.98	3.07	0.05
E1	GPM2	3A	AX.89463175	5.61E+07	0.38	-639.74	3.44	0.06
E1	GPM2	3A	AX.89467487	7.31E+06	0.42	619.30	3.35	0.05
E1	GY	3A	AX.89549948	5.70E+07	0.38	-2.49	3.45	0.06
E1	GPM2	3A	AX.89759598	1.18E+07	0.33	652.45	3.35	0.06
E1	GY	3A	AX.89508580	5.61E+07	0.37	-2.50	3.45	0.06
E1	GPM2	3A	AX.89371520	5.70E+07	0.38	-639.74	3.44	0.06
E1	GPM2	3A	AX.89326987	5.64E+07	0.38	-639.74	3.44	0.06
E1	GY	3A	AX.89497516	5.70E+07	0.38	-2.49	3.45	0.06
E1	GPM2	3A	AX.89477720	5.71E+07	0.38	-639.74	3.44	0.06
E1	GPM2	3A	AX.89670742	7.09E+08	0.10	-1032.15	3.48	0.06
E1	GPM2	3A	AX.89587706	1.78E+05	0.35	-726.07	4.17	0.07
E1	GY	3A	AX.89357150	7.09E+08	0.07	-4.53	3.01	0.05
E1	GPM2	3A	AX.89445155	5.67E+07	0.37	-654.03	3.55	0.06
E1	GPM2	3A	AX.89471055	1.29E+06	0.34	-653.40	3.44	0.05
E1	GPM2	3A	AX.89356377	5.62E+07	0.37	-654.03	3.55	0.06
E1	GPM2	3A	AX.89639547	1.18E+07	0.33	652.45	3.35	0.06
E1	GPM2	3A	AX.89667791	4.17E+05	0.34	-700.37	3.85	0.06
E1	GPM2	3A	AX.89417960	5.69E+07	0.37	-654.03	3.55	0.06
E1	GY	3A	AX.89617804	6.93E+07	0.33	-2.43	3.14	0.05
E1	GY	3A	AX.89371520	5.70E+07	0.38	-2.49	3.45	0.06
E1	GY	3A	AX.89485992	5.64E+07	0.37	-2.50	3.45	0.06
E1	SPM2	3A	AX.89420905	2.36E+07	0.13	29.06	4.29	0.08
E1	GPM2	3A	AX.89736209	5.61E+07	0.37	-654.03	3.55	0.06
E1	GPM2	3A	AX.89699257	5.67E+07	0.37	-666.58	3.65	0.06
E1	GPM2	3A	AX.89483235	7.09E+06	0.37	-610.03	3.13	0.05
E1	GPM2	3A	AX.89600503	5.68E+07	0.38	-639.74	3.44	0.06
E1	GPM2	3A	AX.89498068	7.28E+06	0.37	-600.16	3.06	0.04
E1	GPM2	3A	AX.89397074	5.61E+07	0.38	-639.74	3.44	0.06
E1	GPM2	3A	AX.89414130	7.09E+06	0.37	-610.03	3.13	0.05
E1	GPS	3A	AX.89337319	7.11E+08	0.06	-2.38	3.13	0.05
E1	GPM2	3A	AX.89602017	5.61E+07	0.38	-639.74	3.44	0.06
E1	GY	3A	AX.89564685	5.17E+08	0.05	-6.25	3.98	0.08
E1	GPM2	3A	AX.89567118	5.67E+07	0.37	-654.03	3.55	0.06
E1	GPM2	3A	AX.89344030	5.71E+07	0.37	-741.73	4.39	0.08
E1	GY	3A	AX.89636418	5.64E+07	0.37	-2.50	3.45	0.06
E1	GPM2	3A	AX.89650770	5.68E+07	0.38	-639.74	3.44	0.06
E1	GPM2	3A	AX.89532206	5.62E+07	0.38	-639.74	3.44	0.06
E1	GPM2	3A	AX.89517433	5.57E+07	0.37	-654.03	3.55	0.06
E1	GPM2	3A	AX.89640393	4.31E+05	0.34	-700.37	3.85	0.06
E1	GPM2	3A	AX.89423759	5.62E+07	0.38	-609.23	3.15	0.05

E1	GY	3A	AX.89573099	5.67E+07	0.37	-2.50	3.45	0.06
E1	GPM2	3A	AX.89409258	7.09E+06	0.37	-610.03	3.13	0.05
E1	GPM2	3A	AX.89502639	5.68E+07	0.37	-654.03	3.55	0.06
E1	GPM2	3A	AX.89445873	5.57E+08	0.05	-1485.41	3.48	0.06
E1	GPM2	3A	AX.89620261	7.22E+06	0.43	656.66	3.72	0.05
E1	GPM2	3A	AX.89370524	7.21E+06	0.42	619.30	3.35	0.05
E1	GPM2	3A	AX.89743861	7.26E+06	0.45	670.93	3.90	0.06
E1	GY	3A	AX.89504535	6.25E+08	0.48	-2.42	3.44	0.05
E1	GPS	3A	AX.89768286	7.11E+08	0.06	-2.38	3.13	0.05
E1	GPM2	3A	AX.89543452	1.14E+07	0.30	688.88	3.55	0.06
E1	GPM2	3A	AX.89450737	5.70E+07	0.37	-654.03	3.55	0.06
E1	GPM2	3A	AX.89427924	5.61E+07	0.37	-654.03	3.55	0.06
E1	GPM2	3A	AX.89705622	5.64E+07	0.37	-654.03	3.55	0.06
E1	GPM2	3A	AX.89360803	5.62E+07	0.38	-639.74	3.44	0.06
E1	GPM2	3A	AX.89690421	7.14E+08	0.38	596.12	3.04	0.05
E1	GPM2	3A	AX.89701379	5.20E+08	0.05	-1606.01	3.98	0.07
E1	GPM2	3A	AX.89653072	5.70E+07	0.37	-654.03	3.55	0.06
E1	GPM2	3A	AX.89402066	7.19E+08	0.22	-777.44	3.65	0.06
E1	GPM2	3A	AX.89636653	1.18E+07	0.33	652.45	3.35	0.06
E1	GPM2	3A	AX.89471245	1.02E+07	0.32	637.42	3.20	0.06
E1	GPM2	3A	AX.89549519	7.26E+06	0.42	619.30	3.35	0.05
E1	GY	3A	AX.89543199	5.06E+08	0.06	-4.93	3.08	0.06
E1	GPM2	3A	AX.89598815	5.67E+07	0.37	-654.03	3.55	0.06
E1	GPM2	3A	AX.89326089	5.67E+07	0.37	-654.03	3.55	0.06
E1	GPM2	3A	AX.89464016	5.64E+07	0.37	-654.03	3.55	0.06
E1	GPS	3A	AX.89415949	7.11E+08	0.07	-2.32	3.18	0.05
E1	GPM2	3A	AX.89536603	5.70E+07	0.38	-639.74	3.44	0.06
E1	GPM2	3A	AX.89468962	7.21E+06	0.45	670.93	3.90	0.06
E1	GPM2	3A	AX.89360487	5.67E+07	0.37	-654.03	3.55	0.06
E1	GPM2	3A	AX.89749604	5.64E+07	0.37	-654.03	3.55	0.06
E1	GPM2	3A	AX.89414288	7.28E+06	0.37	-600.16	3.06	0.04
E1	GPM2	3A	AX.89485992	5.64E+07	0.37	-654.03	3.55	0.06
E1	GPM2	3A	AX.89740185	5.57E+07	0.38	-639.74	3.44	0.06
E1	GPM2	3A	AX.89410914	5.70E+07	0.38	-639.74	3.44	0.06
E1	GPM2	3A	AX.89740631	7.26E+06	0.42	619.30	3.35	0.05
E1	GPM2	3A	AX.89678328	5.62E+07	0.38	-639.74	3.44	0.06
E1	TKW	3A	AX.89515637	5.00E+08	0.13	-1.59	4.54	0.08
E1	GPM2	3A	AX.89775222	5.61E+07	0.37	-654.03	3.55	0.06
E1	GPM2	3A	AX.89462305	1.18E+07	0.29	642.67	3.10	0.05
E1	TKW	3A	AX.89731837	4.79E+08	0.34	-0.89	3.12	0.06
E1	TKW	3A	AX.89476722	4.80E+08	0.41	0.85	3.08	0.05
E1	GPM2	3A	AX.89636418	5.64E+07	0.37	-654.03	3.55	0.06
E1	GPM2	3A	AX.89596917	5.62E+07	0.38	-639.74	3.44	0.06
E1	TKW	3A	AX.89596098	7.44E+08	0.35	-0.96	3.55	0.06
E1	TKW	3A	AX.89730621	4.80E+08	0.41	0.85	3.07	0.05
E1	TKW	3A	AX.89710837	5.32E+08	0.17	-1.22	3.54	0.06
E1	TKW	3A	AX.89385976	5.32E+08	0.16	-1.28	3.77	0.07
E1	GPM2	3A	AX.89549948	5.70E+07	0.38	-639.74	3.44	0.06
E1	TKW	3A	AX.89552376	4.86E+08	0.16	-1.24	3.48	0.06
E1	TKW	3A	AX.89612704	4.80E+08	0.40	0.86	3.12	0.05

E1	TKW	3A	AX.89335343	5.65E+08	0.15	-1.15	3.03	0.05
E1	GY	3A	AX.89559269	5.61E+07	0.39	-2.51	3.51	0.06
E1	TKW	3A	AX.89539595	4.79E+08	0.34	-0.89	3.12	0.06
E1	GY	3A	AX.89536450	5.68E+07	0.38	-2.49	3.45	0.06
E1	TKW	3A	AX.89395544	4.86E+08	0.16	-1.24	3.48	0.06
E1	TKW	3A	AX.89771728	7.44E+08	0.26	-0.97	3.15	0.05
E1	GY	3A	AX.89762173	5.71E+07	0.38	-2.49	3.45	0.06
E1	TKW	3A	AX.89319918	5.34E+08	0.13	-1.48	4.13	0.07
E1	GPM2	3A	AX.89444118	4.17E+05	0.50	-606.80	3.30	0.05
E1	GY	3A	AX.89755885	5.64E+07	0.38	-2.49	3.45	0.06
E1	TKW	3A	AX.89767042	5.32E+08	0.16	-1.28	3.77	0.07
E1	TKW	3A	AX.89702309	4.80E+08	0.22	-1.39	5.38	0.10
E1	TKW	3A	AX.89595384	5.32E+08	0.17	-1.17	3.29	0.06
E1	GPM2	3A	AX.89633282	1.31E+06	0.36	-673.59	3.68	0.06
E1	TKW	3A	AX.89375669	4.80E+08	0.24	-1.31	5.07	0.09
E1	GY	3A	AX.89597151	6.60E+08	0.24	2.83	3.41	0.06
E1	TKW	3A	AX.89377201	5.34E+08	0.17	-1.26	3.83	0.07
E1	TKW	3A	AX.89664544	7.44E+08	0.37	-0.96	3.66	0.07
E1	GPM2	3A	AX.89514349	5.70E+07	0.38	-639.74	3.44	0.06
E1	TKW	3A	AX.89720844	4.94E+08	0.14	-1.28	3.44	0.06
E1	GY	3A	AX.89684368	7.12E+08	0.20	2.83	3.12	0.05
E1	TKW	3A	AX.89310434	5.32E+08	0.16	-1.28	3.77	0.07
E1	TKW	3A	AX.89777932	5.34E+08	0.13	-1.43	3.99	0.07
E1	TKW	3A	AX.89775562	5.32E+08	0.14	-1.21	3.07	0.05
E1	GPM2	3A	AX.89618886	6.23E+08	0.23	705.86	3.21	0.05
E1	TKW	3A	AX.89311054	4.80E+08	0.22	-1.39	5.38	0.10
E1	TKW	3A	AX.89492626	7.44E+08	0.37	-0.88	3.14	0.06
E1	TKW	3A	AX.89511542	5.32E+08	0.14	-1.21	3.07	0.05
E1	TKW	3A	AX.89497520	7.44E+08	0.35	-0.96	3.55	0.06
E1	TKW	3A	AX.89426956	5.32E+08	0.14	-1.21	3.07	0.05
E1	GPM2	3A	AX.89538943	7.11E+08	0.07	-1224.61	3.65	0.06
E1	TKW	3A	AX.89433968	4.86E+08	0.16	-1.24	3.48	0.06
E1	TKW	3A	AX.89346113	4.80E+08	0.22	-1.39	5.38	0.10
E1	GY	3A	AX.89598815	5.67E+07	0.37	-2.50	3.45	0.06
E1	TKW	3A	AX.89595925	4.86E+08	0.16	-1.24	3.48	0.06
E1	TKW	3A	AX.89467371	9.85E+06	0.26	0.96	3.07	0.05
E1	TKW	3A	AX.89609675	5.34E+08	0.13	-1.44	3.81	0.07
E1	TKW	3A	AX.89669017	5.34E+08	0.17	-1.26	3.83	0.07
E1	GPM2	3A	AX.89657684	7.22E+06	0.45	632.74	3.51	0.05
E1	TKW	3A	AX.89609294	5.32E+08	0.14	-1.21	3.07	0.05
E1	GPM2	3A	AX.89543954	5.70E+07	0.38	-610.85	3.18	0.05
E1	GY	3A	AX.89701379	5.20E+08	0.05	-7.82	5.92	0.11
E1	GPM2	3A	AX.89579477	9.57E+06	0.26	-678.06	3.16	0.05
E1	TKW	3A	AX.89525277	5.32E+08	0.17	-1.22	3.54	0.06
E1	TKW	3A	AX.89367927	5.20E+08	0.13	-1.26	3.07	0.05
E1	TKW	3A	AX.89634502	4.86E+08	0.16	-1.24	3.48	0.06
E1	GY	3A	AX.89490667	5.71E+07	0.39	-2.46	3.38	0.05
E1	GPM2	3A	AX.89766111	5.68E+07	0.38	-639.74	3.44	0.06
E1	TKW	3A	AX.89439213	5.14E+08	0.22	-1.10	3.50	0.06
E1	TKW	3A	AX.89542835	1.88E+06	0.25	0.97	3.09	0.05

E1	TKW	3A	AX.89447799	5.34E+08	0.17	-1.26	3.83	0.07
E1	GPM2	3A	AX.89762173	5.71E+07	0.38	-639.74	3.44	0.06
E1	GY	3A	AX.89458621	5.67E+07	0.37	-2.50	3.45	0.06
E1	TKW	3A	AX.89702901	5.34E+08	0.17	-1.26	3.83	0.07
E1	TKW	3A	AX.89658848	4.80E+08	0.25	-1.23	4.67	0.08
E1	TKW	3A	AX.89405100	4.86E+08	0.16	-1.24	3.48	0.06
E1	GPM2	3A	AX.89724310	1.17E+07	0.33	652.45	3.35	0.06
E1	GPM2	3A	AX.89470027	5.61E+07	0.37	-654.03	3.55	0.06
E1	GPM2	3A	AX.89370767	1.52E+06	0.34	-626.33	3.18	0.05
E1	TKW	3A	AX.89768808	5.34E+08	0.17	-1.26	3.83	0.07
E1	TKW	3A	AX.89643406	5.22E+08	0.13	-1.26	3.07	0.05
E1	TKW	3A	AX.89528884	4.88E+08	0.14	-1.23	3.15	0.05
E1	TKW	3A	AX.89737513	5.34E+08	0.15	-1.30	3.64	0.06
E1	TKW	3A	AX.89726992	5.32E+08	0.17	-1.22	3.54	0.06
E1	TKW	3A	AX.89440804	4.80E+08	0.24	-1.31	5.07	0.09
E1	TKW	3A	AX.89441173	4.79E+08	0.34	-0.89	3.12	0.06
E1	TKW	3A	AX.89396866	5.32E+08	0.15	-1.36	4.02	0.07
E1	GPM2	3A	AX.89777576	6.25E+08	0.24	694.77	3.16	0.05
E1	GPM2	3A	AX.89528765	5.67E+07	0.37	-654.03	3.55	0.06
E1	GPM2	3A	AX.89371568	1.03E+06	0.34	-653.40	3.44	0.05
E1	TKW	3A	AX.89665785	4.80E+08	0.24	-1.27	4.75	0.08
E1	GPM2	3A	AX.89411438	5.70E+07	0.37	-654.03	3.55	0.06
E1	GPM2	3A	AX.89352203	4.14E+05	0.35	-657.03	3.51	0.05
E1	TKW	3A	AX.89328327	5.20E+08	0.13	-1.26	3.07	0.05
E1	GPM2	3A	AX.89581641	5.68E+07	0.37	-654.03	3.55	0.06
E1	GPM2	3A	AX.89637550	1.60E+05	0.50	-619.47	3.42	0.05
E1	TKW	3A	AX.89622363	5.34E+08	0.17	-1.26	3.83	0.07
E1	TKW	3A	AX.89333489	4.86E+08	0.13	-1.31	3.42	0.06
E1	TKW	3A	AX.89680560	5.34E+08	0.14	-1.20	3.02	0.05
E1	TKW	3A	AX.89493328	5.34E+08	0.17	-1.26	3.83	0.07
E1	TKW	3A	AX.89673642	5.32E+08	0.14	-1.21	3.07	0.05
E1	TKW	3A	AX.89454633	5.32E+08	0.13	-1.28	3.30	0.06
E1	TKW	3A	AX.89611849	4.80E+08	0.22	-1.39	5.38	0.10
E1	TKW	3A	AX.89416670	5.20E+08	0.13	-1.26	3.07	0.05
E1	TKW	3A	AX.89757174	5.32E+08	0.16	-1.28	3.77	0.07
E1	TKW	3A	AX.89757523	7.44E+08	0.27	-0.94	3.05	0.05
E1	TKW	3A	AX.89375431	4.80E+08	0.35	-0.92	3.32	0.06
E1	TKW	3A	AX.89636742	5.32E+08	0.16	-1.28	3.77	0.07
E1	TKW	3A	AX.89471498	7.24E+08	0.22	-1.10	3.52	0.06
E1	GY	3A	AX.89616535	7.11E+08	0.06	-6.47	5.27	0.09
E1	TKW	3A	AX.89380817	4.87E+08	0.17	-1.15	3.21	0.05
E1	TKW	3A	AX.89608704	4.79E+08	0.34	-0.89	3.12	0.06
E1	TKW	3A	AX.89394934	5.32E+08	0.16	-1.18	3.25	0.06
E1	TKW	3A	AX.89394935	4.86E+08	0.16	-1.24	3.48	0.06
E1	TKW	3A	AX.89614123	4.86E+08	0.15	-1.19	3.19	0.05
E1	TKW	3A	AX.89513582	5.02E+08	0.15	-1.35	3.87	0.07
E1	TKW	3A	AX.89567282	4.79E+08	0.27	-0.94	3.05	0.05
E1	TKW	3A	AX.89729748	5.32E+08	0.17	-1.22	3.54	0.06
E1	TKW	3A	AX.89575384	7.44E+08	0.26	-1.06	3.64	0.06
E1	TKW	3A	AX.89762599	5.17E+08	0.17	-1.12	3.07	0.05

E1	TKW	3A	AX.89474714	4.79E+08	0.34	-0.89	3.12	0.06
E1	TKW	3A	AX.89507009	5.32E+08	0.17	-1.22	3.54	0.06
E1	TKW	3A	AX.89389553	4.87E+08	0.17	-1.22	3.51	0.06
E1	TKW	3A	AX.89747368	4.79E+08	0.34	-0.89	3.12	0.06
E1	TKW	3A	AX.89445760	5.32E+08	0.16	-1.28	3.77	0.07
E1	TKW	3A	AX.89756173	5.32E+08	0.16	-1.28	3.77	0.07
E1	TKW	3A	AX.89461601	5.32E+08	0.13	-1.24	3.04	0.05
E1	TKW	3A	AX.89676298	5.32E+08	0.14	-1.21	3.07	0.05
E1	TKW	3A	AX.89477758	5.32E+08	0.17	-1.22	3.54	0.06
E1	TKW	3A	AX.89315109	5.34E+08	0.17	-1.26	3.83	0.07
E1	TKW	3A	AX.89718581	4.80E+08	0.40	0.86	3.12	0.05
E1	TKW	3A	AX.89644673	5.34E+08	0.17	-1.26	3.83	0.07
E1	TKW	3A	AX.89750696	5.34E+08	0.17	-1.35	4.28	0.08
E1	TKW	3A	AX.89325426	4.80E+08	0.24	-1.31	5.07	0.09
E1	TKW	3A	AX.89513937	4.86E+08	0.16	-1.24	3.48	0.06
E1	TKW	3A	AX.89686928	4.79E+08	0.34	-0.89	3.12	0.06
E1	TKW	3A	AX.89444920	7.44E+08	0.27	-0.95	3.07	0.05
E1	TKW	3A	AX.89374451	5.32E+08	0.17	-1.22	3.54	0.06
E1	TKW	3A	AX.89584807	5.20E+08	0.13	-1.26	3.07	0.05
E1	TKW	3A	AX.89650073	4.87E+08	0.13	-1.25	3.18	0.05
E1	TKW	3A	AX.89382187	5.34E+08	0.17	-1.26	3.83	0.07
E1	TKW	3A	AX.89691225	4.86E+08	0.16	-1.24	3.48	0.06
E1	TKW	3A	AX.89610052	2.23E+08	0.26	0.96	3.06	0.05
E1	TKW	3A	AX.89390721	4.88E+08	0.16	-1.17	3.23	0.05
E1	TKW	3A	AX.89716790	5.34E+08	0.15	-1.30	3.64	0.06
E1	TKW	3A	AX.89610689	5.32E+08	0.17	-1.22	3.54	0.06
E1	TKW	3A	AX.89509711	5.32E+08	0.17	-1.22	3.54	0.06
E1	TKW	3A	AX.89603382	4.88E+08	0.14	-1.23	3.15	0.05
E1	TKW	3A	AX.89623712	5.32E+08	0.13	-1.28	3.30	0.06
E1	GPM2	3A	AX.89606467	7.27E+06	0.37	-600.16	3.06	0.04
E1	TKW	3A	AX.89376790	5.00E+08	0.13	-1.42	3.82	0.06
E1	TKW	3A	AX.89709765	4.80E+08	0.34	-0.89	3.12	0.06
E1	TKW	3A	AX.89643606	5.32E+08	0.10	-1.43	3.14	0.05
E1	TKW	3A	AX.89607276	5.32E+08	0.17	-1.13	3.14	0.05
E1	TKW	3A	AX.89668982	5.32E+08	0.17	-1.35	4.29	0.08
E1	TKW	3A	AX.89401719	7.24E+08	0.22	-1.10	3.52	0.06
E1	TKW	3A	AX.89455172	5.32E+08	0.16	-1.28	3.77	0.07
E1	GPM2	3A	AX.89448108	1.18E+07	0.33	652.45	3.35	0.06
E1	TKW	3A	AX.89362246	4.59E+08	0.39	0.91	3.41	0.06
E1	TKW	3A	AX.89727232	5.34E+08	0.17	-1.26	3.83	0.07
E1	TKW	3A	AX.89739009	4.79E+08	0.40	0.86	3.12	0.05
E1	TKW	3A	AX.89347824	4.87E+08	0.14	-1.23	3.15	0.05
E1	TKW	3A	AX.89326650	5.27E+08	0.17	-1.21	3.48	0.06
E1	GPM2	3A	AX.89401377	5.67E+07	0.37	-654.03	3.55	0.06
E1	TKW	3A	AX.89360413	4.79E+08	0.40	0.86	3.12	0.05
E1	TKW	3A	AX.89512513	7.44E+08	0.26	-0.97	3.15	0.05
E1	TKW	3A	AX.89659664	5.34E+08	0.17	-1.26	3.83	0.07
E1	TKW	3A	AX.89413743	4.91E+08	0.13	-1.26	3.12	0.05
E1	TKW	3A	AX.89523321	5.32E+08	0.14	-1.21	3.07	0.05
E1	TKW	3A	AX.89405913	4.80E+08	0.44	0.87	3.26	0.06

E1	TKW	3A	AX.89445609	4.86E+08	0.16	-1.24	3.48	0.06
E1	TKW	3A	AX.89422142	7.24E+08	0.22	-1.10	3.52	0.06
E1	TKW	3A	AX.89319278	7.44E+08	0.26	-0.97	3.15	0.05
E1	GPM2	3A	AX.89451205	7.09E+08	0.10	-1032.15	3.48	0.06
E1	TKW	3A	AX.89486717	5.34E+08	0.18	-1.24	3.77	0.07
E1	TKW	3A	AX.89777457	5.15E+08	0.23	-0.99	3.07	0.05
E1	TKW	3A	AX.89766135	5.32E+08	0.16	-1.28	3.77	0.07
E1	TKW	3A	AX.89332330	5.32E+08	0.14	-1.21	3.07	0.05
E1	GPM2	3A	AX.89648063	7.26E+06	0.45	670.93	3.90	0.06
E1	TKW	3A	AX.89749784	4.87E+08	0.14	-1.34	3.72	0.06
E1	GY	3A	AX.89369723	6.93E+07	0.33	-2.43	3.14	0.05
E1	TKW	3A	AX.89557687	5.32E+08	0.16	-1.28	3.77	0.07
E1	TKW	3A	AX.89629822	4.44E+08	0.38	-0.87	3.13	0.05
E1	TKW	3A	AX.89755097	4.79E+08	0.34	-0.89	3.12	0.06
E1	TKW	3A	AX.89699436	5.34E+08	0.16	-1.19	3.32	0.06
E1	TKW	3A	AX.89707628	7.44E+08	0.27	-0.94	3.05	0.05
E1	TKW	3A	AX.89436418	4.80E+08	0.41	0.85	3.07	0.05
E1	TKW	3A	AX.89654631	4.87E+08	0.14	-1.23	3.15	0.05
E1	TKW	3A	AX.89619570	7.44E+08	0.38	-0.92	3.38	0.06
E1	GPM2	3A	AX.89363577	4.16E+05	0.35	-690.21	3.80	0.06
E1	TKW	3A	AX.89726186	4.80E+08	0.35	-0.93	3.40	0.06
E1	TKW	3A	AX.89343172	7.79E+07	0.29	-0.97	3.31	0.06
E1	TKW	3A	AX.89455128	4.75E+08	0.38	1.01	4.00	0.07
E1	TKW	3A	AX.89401344	4.87E+08	0.16	-1.17	3.23	0.05
E1	TKW	3A	AX.89508575	4.44E+08	0.25	-1.15	4.10	0.07
E1	TKW	3A	AX.89748517	5.34E+08	0.13	-1.48	4.13	0.07
E1	TKW	3A	AX.89463389	5.32E+08	0.14	-1.21	3.07	0.05
E1	TKW	3A	AX.89464242	5.32E+08	0.17	-1.22	3.54	0.06
E1	TKW	3A	AX.89573974	5.34E+08	0.17	-1.26	3.83	0.07
E1	TKW	3A	AX.89431838	7.24E+08	0.22	-1.10	3.52	0.06
E1	TKW	3A	AX.89519239	5.12E+08	0.10	-1.43	3.14	0.05
E1	TKW	3A	AX.89527266	4.87E+08	0.16	-1.17	3.23	0.05
E1	TKW	3A	AX.89341415	5.21E+08	0.13	-1.26	3.07	0.05
E1	TKW	3A	AX.89325014	4.79E+08	0.34	-0.89	3.12	0.06
E1	TKW	3A	AX.89402327	5.20E+08	0.13	-1.26	3.07	0.05
E1	TKW	3A	AX.89433907	4.80E+08	0.22	-1.39	5.38	0.10
E1	GY	3A	AX.89636418	5.64E+07	0.37	-2.50	3.45	0.06
E1	TKW	3A	AX.89752385	4.86E+08	0.16	-1.24	3.48	0.06
E1	TKW	3A	AX.89445636	2.81E+08	0.25	0.96	3.01	0.05
E1	TKW	3A	AX.89756096	5.32E+08	0.16	-1.28	3.77	0.07
E1	GPM2	3A	AX.89563227	7.10E+08	0.07	-1340.44	3.83	0.07
E1	TKW	3A	AX.89710971	4.86E+08	0.16	-1.24	3.48	0.06
E1	GPM2	3A	AX.89570766	5.67E+07	0.37	-654.03	3.55	0.06
E1	TKW	3A	AX.89391694	5.32E+08	0.17	-1.22	3.54	0.06
E1	TKW	3A	AX.89715165	7.44E+08	0.27	-0.94	3.05	0.05
E1	TKW	3A	AX.89337779	7.44E+08	0.35	-1.00	3.81	0.07
E1	GPM2	3A	AX.89735763	5.67E+07	0.37	-654.03	3.55	0.06
E1	TKW	3A	AX.89344911	4.80E+08	0.22	-1.39	5.38	0.10
E1	TKW	3A	AX.89436116	5.11E+08	0.22	-1.04	3.20	0.05
E1	TKW	3A	AX.89566122	5.32E+08	0.16	-1.28	3.77	0.07

E1	GPM2	3A	AX.89464249	4.29E+05	0.35	-709.04	4.00	0.06
E1	GPM2	3A	AX.89559269	5.61E+07	0.39	-595.67	3.06	0.05
E1	GPM2	3A	AX.89723050	5.61E+07	0.38	-639.74	3.44	0.06
E1	GPM2	3A	AX.89316834	5.67E+07	0.37	-654.03	3.55	0.06
E1	GPM2	3A	AX.89334033	5.71E+07	0.38	-639.74	3.44	0.06
E1	GPM2	3A	AX.89538282	5.64E+07	0.38	-639.74	3.44	0.06
E1	GPM2	3A	AX.89635924	5.61E+07	0.37	-654.03	3.55	0.06
E1	GPM2	3A	AX.89654753	1.18E+07	0.29	642.67	3.10	0.05
E1	TKW	3A	AX.89374984	7.44E+08	0.26	-1.03	3.52	0.06
E1	GY	3A	AX.89756161	5.62E+07	0.38	-2.49	3.45	0.06
E1	GPM2	3A	AX.89432808	5.58E+07	0.37	-654.03	3.55	0.06
E1	TKW	3A	AX.89622080	5.32E+08	0.17	-1.22	3.54	0.06
E1	TKW	3A	AX.89362477	4.86E+08	0.16	-1.24	3.48	0.06
E1	TKW	3A	AX.89354916	4.87E+08	0.17	-1.22	3.51	0.06
E1	TKW	3A	AX.89723710	5.32E+08	0.16	-1.28	3.77	0.07
E1	TKW	3A	AX.89608966	5.34E+08	0.17	-1.26	3.83	0.07
E1	TKW	3A	AX.89364280	4.80E+08	0.22	-1.39	5.38	0.10
E1	TKW	3A	AX.89733534	4.80E+08	0.22	-1.39	5.38	0.10
E1	GPM2	3A	AX.89512498	5.68E+07	0.37	-654.03	3.55	0.06
E1	TKW	3A	AX.89323186	4.80E+08	0.40	0.86	3.12	0.05
E1	TKW	3A	AX.89521685	5.32E+08	0.17	-1.22	3.54	0.06
E1	TKW	3A	AX.89613546	4.09E+08	0.26	0.96	3.06	0.05
E1	TKW	3A	AX.89382610	7.44E+08	0.38	-0.92	3.38	0.06
E1	TKW	3A	AX.89697964	5.32E+08	0.10	-1.43	3.14	0.05
E1	GPM2	3A	AX.89735030	1.31E+06	0.35	-668.20	3.61	0.05
E1	TKW	3A	AX.89537624	4.79E+08	0.34	-0.89	3.12	0.06
E1	TKW	3A	AX.89746328	5.34E+08	0.15	-1.30	3.64	0.06
E1	TKW	3A	AX.89674401	4.80E+08	0.41	0.85	3.07	0.05
E1	TKW	3A	AX.89556300	5.34E+08	0.13	-1.48	4.13	0.07
E1	GPM2	3A	AX.89461796	5.57E+08	0.05	-1485.41	3.48	0.06
E1	TKW	3A	AX.89494558	7.44E+08	0.36	-0.96	3.60	0.06
E1	TKW	3A	AX.89543058	7.24E+08	0.22	-1.10	3.52	0.06
E1	TKW	3A	AX.89319910	5.22E+08	0.13	-1.26	3.07	0.05
E1	TKW	3A	AX.89751607	4.79E+08	0.34	-0.94	3.44	0.06
E1	TKW	3A	AX.89561000	5.32E+08	0.16	-1.28	3.77	0.07
E1	GPM2	3A	AX.89775578	5.61E+07	0.38	-639.74	3.44	0.06
E1	TKW	3A	AX.89452513	5.17E+08	0.13	-1.26	3.07	0.05
E1	GPM2	3A	AX.89369804	5.57E+07	0.37	-654.03	3.55	0.06
E1	TKW	3A	AX.89765605	5.34E+08	0.17	-1.26	3.83	0.07
E1	TKW	3A	AX.89648650	5.32E+08	0.15	-1.19	3.18	0.05
E1	TKW	3A	AX.89533025	5.32E+08	0.16	-1.28	3.77	0.07
E1	TKW	3A	AX.89749568	7.24E+08	0.22	-1.10	3.52	0.06
E1	GPM2	3A	AX.89742109	5.67E+07	0.37	-654.03	3.55	0.06
E1	GPM2	3A	AX.89764338	1.27E+06	0.34	-653.40	3.44	0.05
E1	TKW	3A	AX.89322549	4.80E+08	0.34	-0.89	3.12	0.06
E1	GPM2	3A	AX.89534837	5.70E+07	0.38	-639.74	3.44	0.06
E1	TKW	3A	AX.89590832	4.57E+08	0.42	0.86	3.14	0.05
E1	TKW	3A	AX.89371956	5.32E+08	0.16	-1.23	3.47	0.06
E1	TKW	3A	AX.89460722	5.32E+08	0.17	-1.22	3.54	0.06
E1	TKW	3A	AX.89498138	4.32E+08	0.22	-1.04	3.26	0.06

E1	GPM2	3A	AX.89311273	1.42E+05	0.34	-653.40	3.44	0.05
E1	TKW	3A	AX.89313940	5.32E+08	0.17	-1.22	3.54	0.06
E1	TKW	3A	AX.89746144	5.15E+08	0.23	-0.99	3.07	0.05
E1	TKW	3A	AX.89469040	7.44E+08	0.36	-0.93	3.39	0.06
E1	TKW	3A	AX.89401394	5.34E+08	0.17	-1.26	3.83	0.07
E1	TKW	3A	AX.89441206	4.80E+08	0.24	-1.31	5.07	0.09
E1	TKW	3A	AX.89489359	5.32E+08	0.15	-1.18	3.07	0.05
E1	TKW	3A	AX.89311196	7.44E+08	0.27	-0.94	3.05	0.05
E1	TKW	3A	AX.89420035	5.37E+08	0.13	-1.25	3.17	0.05
E1	TKW	3A	AX.89500738	4.79E+08	0.34	-0.89	3.12	0.06
E1	TKW	3A	AX.89437419	5.34E+08	0.12	-1.39	3.39	0.06
E1	TKW	3A	AX.89748002	5.32E+08	0.16	-1.28	3.77	0.07
E1	TKW	3A	AX.89755805	7.21E+06	0.40	0.86	3.11	0.05
E1	TKW	3A	AX.89352479	4.80E+08	0.41	0.87	3.19	0.06
E1	TKW	3A	AX.89383923	5.17E+08	0.13	-1.26	3.07	0.05
E1	TKW	3A	AX.89766488	4.79E+08	0.34	-0.89	3.12	0.06
E1	TKW	3A	AX.89470090	4.70E+08	0.19	-1.10	3.18	0.05
E1	TKW	3A	AX.89619996	4.79E+08	0.34	-0.89	3.12	0.06
E1	TKW	3A	AX.89342059	5.32E+08	0.17	-1.22	3.54	0.06
E1	TKW	3A	AX.89347510	5.34E+08	0.13	-1.33	3.35	0.06
E1	TKW	3A	AX.89655083	2.12E+08	0.26	0.96	3.06	0.05
E1	TKW	3A	AX.89600269	5.32E+08	0.14	-1.21	3.07	0.05
E1	TKW	3A	AX.89632422	7.44E+08	0.26	-0.97	3.15	0.05
E1	TKW	3A	AX.89674577	5.32E+08	0.16	-1.23	3.45	0.06
E1	TKW	3A	AX.89724486	5.32E+08	0.17	-1.22	3.54	0.06
E1	TKW	3A	AX.89643254	5.34E+08	0.17	-1.26	3.83	0.07
E1	TKW	3A	AX.89519171	4.87E+08	0.16	-1.17	3.23	0.05
E1	TKW	3A	AX.89385871	4.86E+08	0.15	-1.19	3.19	0.05
E1	TKW	3A	AX.89380739	4.80E+08	0.22	-1.39	5.38	0.10
E1	TKW	3A	AX.89661792	7.24E+08	0.22	-1.05	3.27	0.05
E1	TKW	3A	AX.89686826	5.34E+08	0.13	-1.58	4.65	0.08
E1	TKW	3A	AX.89463665	5.32E+08	0.16	-1.28	3.77	0.07
E1	TKW	3A	AX.89729990	4.79E+08	0.34	-0.89	3.12	0.06
E1	TKW	3A	AX.89354980	4.79E+08	0.34	-0.89	3.12	0.06
E1	GPM2	3A	AX.89436052	5.70E+07	0.38	-639.74	3.44	0.06
E1	TKW	3A	AX.89724119	4.86E+08	0.16	-1.24	3.48	0.06
E1	TKW	3A	AX.89580115	4.50E+08	0.24	-1.11	3.81	0.07
E1	TKW	3A	AX.89371414	4.36E+08	0.23	-1.09	3.53	0.06
E1	TKW	3A	AX.89501453	5.32E+08	0.14	-1.21	3.07	0.05
E1	TKW	3A	AX.89440050	5.32E+08	0.16	-1.34	4.00	0.07
E1	GPM2	3A	AX.89312843	5.67E+07	0.38	-639.74	3.44	0.06
E1	TKW	3A	AX.89696268	4.87E+08	0.16	-1.17	3.23	0.05
E1	TKW	3A	AX.89771993	5.34E+08	0.14	-1.47	4.41	0.08
E1	TKW	3A	AX.89350488	5.62E+08	0.17	-1.31	3.98	0.07
E1	TKW	3A	AX.89572200	4.80E+08	0.24	-1.37	5.52	0.10
E1	TKW	3A	AX.89474315	4.45E+08	0.38	-0.87	3.13	0.05
E1	TKW	3A	AX.89568617	5.18E+08	0.13	-1.26	3.07	0.05
E1	TKW	3A	AX.89668013	5.20E+08	0.13	-1.26	3.07	0.05
E1	TKW	3A	AX.89510213	5.32E+08	0.14	-1.21	3.07	0.05
E1	TKW	3A	AX.89523370	5.32E+08	0.17	-1.22	3.54	0.06

E1	TKW	3A	AX.89768859	4.87E+08	0.16	-1.24	3.48	0.06
E1	TKW	3A	AX.89705426	5.34E+08	0.17	-1.26	3.83	0.07
E1	TKW	3A	AX.89607450	4.79E+08	0.40	0.86	3.12	0.05
E2	GY	3A	AX.89445873	5.57E+08	0.05	-5.57	4.47	0.09
E2	GY	3A	AX.89445873	5.57E+08	0.05	-5.57	4.47	0.09
E2	GY	3A	AX.89411739	5.60E+08	0.07	-4.26	3.69	0.07
E2	GY	3A	AX.89329184	5.30E+08	0.07	-4.26	3.69	0.07
E2	GY	3A	AX.89329184	5.30E+08	0.07	-4.26	3.69	0.07
E2	SPM2	3A	AX.89642254	7.10E+08	0.20	-15.60	3.42	0.05
E2	GY	3A	AX.89329561	5.61E+08	0.07	-4.26	3.69	0.07
E2	SPM2	3A	AX.89652812	7.10E+08	0.22	-13.99	3.04	0.05
E2	SPM2	3A	AX.89506953	7.10E+08	0.22	-13.99	3.04	0.05
E2	GY	3A	AX.89505764	5.30E+08	0.07	-4.26	3.69	0.07
E2	GY	3A	AX.89435911	5.59E+08	0.07	-4.26	3.69	0.07
E2	GY	3A	AX.89588092	5.57E+08	0.07	-4.26	3.69	0.07
E2	SPM2	3A	AX.89498752	7.10E+08	0.22	-13.99	3.04	0.05
E2	GY	3A	AX.89611948	5.33E+08	0.07	-4.04	3.76	0.08
E2	SPM2	3A	AX.89541316	7.09E+08	0.22	-13.99	3.04	0.05
E2	SPM2	3A	AX.89724076	7.19E+08	0.46	-13.57	3.93	0.06
E2	SPM2	3A	AX.89451569	7.10E+08	0.22	-13.99	3.04	0.05
E2	SPM2	3A	AX.89438257	7.09E+08	0.22	-13.99	3.04	0.05
E2	SPM2	3A	AX.89518967	7.19E+08	0.49	11.57	3.01	0.04
E2	GY	3A	AX.89437126	5.58E+08	0.07	-4.26	3.69	0.07
E2	SPM2	3A	AX.89496561	7.10E+08	0.22	-13.99	3.04	0.05
E2	SPM2	3A	AX.89449835	7.09E+08	0.22	-13.99	3.04	0.05
E2	GY	3A	AX.89723908	5.59E+08	0.07	-4.26	3.69	0.07
E2	GY	3A	AX.89723908	5.59E+08	0.07	-4.26	3.69	0.07
E2	GY	3A	AX.89366811	5.61E+08	0.07	-4.26	3.69	0.07
E2	GY	3A	AX.89366811	5.61E+08	0.07	-4.26	3.69	0.07
E2	SPM2	3A	AX.89467391	7.19E+08	0.46	-13.57	3.93	0.06
E2	GY	3A	AX.89613280	5.59E+08	0.07	-4.26	3.69	0.07
E2	GY	3A	AX.89496237	5.65E+08	0.07	-4.26	3.69	0.07
E2	GY	3A	AX.89496237	5.65E+08	0.07	-4.26	3.69	0.07
E2	SPM2	3A	AX.89679882	7.10E+08	0.22	-13.99	3.04	0.05
E2	SPM2	3A	AX.89551880	7.10E+08	0.22	-13.99	3.04	0.05
E2	GY	3A	AX.89748200	5.66E+08	0.07	-4.26	3.69	0.07
E2	SPM2	3A	AX.89413336	7.09E+08	0.22	-13.99	3.04	0.05
E2	GY	3A	AX.89626071	5.57E+08	0.06	-4.59	3.95	0.08
E2	GY	3A	AX.89460831	5.29E+08	0.07	-4.26	3.69	0.07
E2	GY	3A	AX.89554912	5.66E+08	0.07	-4.26	3.69	0.07
E2	GY	3A	AX.89473126	5.62E+08	0.07	-4.26	3.69	0.07
E2	GY	3A	AX.89473126	5.62E+08	0.07	-4.26	3.69	0.07
E2	GY	3A	AX.89497356	5.60E+08	0.07	-4.26	3.69	0.07
E2	SPM2	3A	AX.89348508	7.09E+08	0.22	-13.99	3.04	0.05
E2	SPM2	3A	AX.89468424	7.10E+08	0.22	-13.99	3.04	0.05
E2	SPM2	3A	AX.86161945	7.19E+08	0.46	-13.57	3.93	0.06
E2	SPM2	3A	AX.89322147	7.09E+08	0.22	-13.99	3.04	0.05
E2	GY	3A	AX.89567808	5.58E+08	0.07	-4.26	3.69	0.07
E2	SPM2	3A	AX.89349777	7.09E+08	0.22	-13.99	3.04	0.05
E2	GY	3A	AX.89540978	5.44E+08	0.05	-5.07	3.72	0.07

E2	GY	3A	AX.89689217	5.33E+08	0.07	-4.26	3.69	0.07
E2	GY	3A	AX.89689217	5.33E+08	0.07	-4.26	3.69	0.07
E2	SPM2	3A	AX.89423665	7.10E+08	0.22	-13.99	3.04	0.05
E2	SPM2	3A	AX.89426872	7.09E+08	0.22	-13.99	3.04	0.05
E2	SPM2	3A	AX.89571934	7.09E+08	0.22	-13.99	3.04	0.05
E2	GY	3A	AX.89487853	5.57E+08	0.07	-4.26	3.69	0.07
E2	SPM2	3A	AX.86166408	7.19E+08	0.46	-13.57	3.93	0.06
E2	SPM2	3A	AX.89726913	7.19E+08	0.48	-11.87	3.13	0.05
E2	GY	3A	AX.89545138	5.57E+08	0.07	-4.26	3.69	0.07
E2	SPM2	3A	AX.89474225	7.10E+08	0.22	-13.99	3.04	0.05
E2	GY	3A	AX.89405683	5.60E+08	0.07	-4.26	3.69	0.07
E2	GY	3A	AX.89358066	5.33E+08	0.07	-4.04	3.76	0.08
E2	GY	3A	AX.89334785	5.64E+08	0.07	-4.26	3.69	0.07
E2	TKW	3A	AX.89414009	7.44E+08	0.30	-1.05	3.37	0.06
E2	SPM2	3A	AX.89651667	7.10E+08	0.21	-14.58	3.18	0.05
E2	SPM2	3A	AX.89379903	7.09E+08	0.22	-13.99	3.04	0.05
E2	SPM2	3A	AX.89690507	7.10E+08	0.22	-13.99	3.04	0.05
E2	SPM2	3A	AX.89499565	7.10E+08	0.23	-13.71	3.01	0.04
E2	GY	3A	AX.89407423	5.38E+07	0.07	-4.67	4.21	0.08
E2	SPM2	3A	AX.89470130	7.09E+08	0.22	-13.99	3.04	0.05
E2	SPM2	3A	AX.89659165	1.95E+07	0.19	19.73	4.92	0.09
E2	SPM2	3A	AX.89643343	7.10E+08	0.23	-13.71	3.01	0.04
E2	SPM2	3A	AX.89323561	7.10E+08	0.22	-13.99	3.04	0.05
E2	GY	3A	AX.89477075	5.57E+08	0.08	-3.80	3.51	0.07
E2	SPM2	3A	AX.89382217	7.10E+08	0.22	-13.99	3.04	0.05
E2	GY	3A	AX.89606047	5.30E+08	0.07	-4.26	3.69	0.07
E2	GY	3A	AX.89675998	5.30E+08	0.06	-4.70	4.13	0.08
E2	GY	3A	AX.89408054	5.62E+08	0.07	-4.26	3.69	0.07
E2	GY	3A	AX.89618721	5.57E+08	0.07	-4.26	3.69	0.07
E2	GY	3A	AX.89618721	5.57E+08	0.07	-4.26	3.69	0.07
E2	GY	3A	AX.89395845	5.57E+08	0.07	-4.26	3.69	0.07
E2	GY	3A	AX.89395845	5.57E+08	0.07	-4.26	3.69	0.07
E2	SPM2	3A	AX.89641664	7.10E+08	0.22	-13.99	3.04	0.05
E2	SPM2	3A	AX.89471074	7.19E+08	0.46	-13.57	3.93	0.06
E2	GY	3A	AX.89337100	5.65E+08	0.07	-4.26	3.69	0.07
E2	SPM2	3A	AX.89609794	7.09E+08	0.22	-13.99	3.04	0.05
E2	GY	3A	AX.89408696	5.64E+08	0.07	-4.26	3.69	0.07
E2	GY	3A	AX.89408696	5.64E+08	0.07	-4.26	3.69	0.07
E2	GPM2	3A	AX.89453948	7.49E+08	0.08	-855.62	3.04	0.04
E2	GY	3A	AX.89734012	5.64E+08	0.06	-5.01	4.31	0.08
E2	GY	3A	AX.89734012	5.64E+08	0.06	-5.01	4.31	0.08
E2	GY	3A	AX.89373419	5.60E+08	0.07	-4.26	3.69	0.07
E2	GY	3A	AX.89373419	5.60E+08	0.07	-4.26	3.69	0.07
E2	SPM2	3A	AX.89699770	7.10E+08	0.22	-13.99	3.04	0.05
E2	SPM2	3A	AX.89413279	7.19E+08	0.46	-13.57	3.93	0.06
E2	SPM2	3A	AX.89658539	7.10E+08	0.22	-13.99	3.04	0.05
E2	GY	3A	AX.89397281	5.30E+08	0.07	-4.26	3.69	0.07
E2	GY	3A	AX.89397281	5.30E+08	0.07	-4.26	3.69	0.07
E2	GY	3A	AX.89338270	5.34E+08	0.07	-4.04	3.76	0.08
E2	SPM2	3A	AX.89773608	7.09E+08	0.22	-13.99	3.04	0.05

E2	GY	3A	AX.89681123	5.62E+08	0.07	-4.26	3.69	0.07
E2	SPM2	3A	AX.89324207	7.10E+08	0.22	-13.99	3.04	0.05
E2	GY	3A	AX.89410241	5.01E+08	0.06	-4.16	3.31	0.07
E2	SPM2	3A	AX.89643823	7.10E+08	0.22	-13.99	3.04	0.05
E2	SPM2	3A	AX.89621689	7.10E+08	0.22	-13.99	3.04	0.05
E2	SPM2	3A	AX.89670211	7.09E+08	0.22	-13.99	3.04	0.05
E2	SPM2	3A	AX.89550259	7.09E+08	0.22	-13.99	3.04	0.05
E2	SPM2	3A	AX.89423989	5.72E+08	0.22	15.12	3.43	0.06
E2	GY	3A	AX.89626214	5.58E+08	0.07	-4.26	3.69	0.07
E2	SPM2	3A	AX.89770711	7.10E+08	0.22	-13.99	3.04	0.05
E2	SPM2	3A	AX.89762555	7.19E+08	0.46	-13.57	3.93	0.06
E2	GY	3A	AX.89658263	5.56E+08	0.10	-3.36	3.49	0.07
E2	GY	3A	AX.89658263	5.56E+08	0.10	-3.36	3.49	0.07
E2	SPM2	3A	AX.89632316	7.09E+08	0.22	-13.99	3.04	0.05
E2	GY	3A	AX.89695735	5.56E+08	0.10	-3.36	3.49	0.07
E2	GY	3A	AX.89411123	5.59E+08	0.07	-4.26	3.69	0.07
E2	SPM2	3A	AX.89657455	7.09E+08	0.22	-13.99	3.04	0.05
E2	GY	3A	AX.89542448	5.30E+08	0.07	-4.26	3.69	0.07
E2	GY	3A	AX.89598565	5.64E+08	0.07	-4.15	3.71	0.07
E2	GY	3A	AX.89573549	5.30E+08	0.07	-4.26	3.69	0.07
E2	GY	3A	AX.89573549	5.30E+08	0.07	-4.26	3.69	0.07
E2	GY	3A	AX.89658666	5.63E+08	0.07	-4.26	3.69	0.07
E2	GY	3A	AX.89658666	5.63E+08	0.07	-4.26	3.69	0.07
E2	GY	3A	AX.89411739	5.60E+08	0.07	-4.26	3.69	0.07
E2	GY	3A	AX.89468877	5.31E+08	0.07	-4.26	3.69	0.07
E2	GY	3A	AX.89468877	5.31E+08	0.07	-4.26	3.69	0.07
E2	GY	3A	AX.89412117	5.16E+08	0.05	-4.80	3.69	0.08
E2	GY	3A	AX.89412117	5.16E+08	0.05	-4.80	3.69	0.08
E2	GY	3A	AX.89468967	5.30E+08	0.07	-4.26	3.69	0.07
E2	GY	3A	AX.89468967	5.30E+08	0.07	-4.26	3.69	0.07
E2	GY	3A	AX.89412292	5.57E+08	0.07	-4.26	3.69	0.07
E2	GY	3A	AX.89412292	5.57E+08	0.07	-4.26	3.69	0.07
E2	GY	3A	AX.89745272	5.57E+08	0.07	-4.42	3.94	0.08
E2	GY	3A	AX.89745272	5.57E+08	0.07	-4.42	3.94	0.08
E2	GY	3A	AX.89505764	5.30E+08	0.07	-4.26	3.69	0.07
E2	GY	3A	AX.89673110	5.63E+08	0.07	-4.26	3.69	0.07
E2	GY	3A	AX.89673110	5.63E+08	0.07	-4.26	3.69	0.07
E2	GY	3A	AX.89660420	5.66E+08	0.07	-4.26	3.69	0.07
E2	GY	3A	AX.89660420	5.66E+08	0.07	-4.26	3.69	0.07
E2	GY	3A	AX.89401576	5.57E+08	0.06	-4.59	3.95	0.08
E2	GY	3A	AX.89401576	5.57E+08	0.06	-4.59	3.95	0.08
E2	GY	3A	AX.89722884	5.64E+08	0.07	-4.15	3.71	0.07
E2	GY	3A	AX.89722884	5.64E+08	0.07	-4.15	3.71	0.07
E2	GY	3A	AX.89673581	5.33E+08	0.07	-4.04	3.76	0.08
E2	GY	3A	AX.89601402	5.65E+08	0.07	-4.26	3.69	0.07
E2	GY	3A	AX.89601402	5.65E+08	0.07	-4.26	3.69	0.07
E2	GY	3A	AX.89601456	5.32E+08	0.07	-4.26	3.69	0.07
E2	GY	3A	AX.89726743	5.29E+08	0.07	-4.26	3.69	0.07
E2	GPS	3A	AX.89319922	6.13E+08	0.10	-2.36	3.51	0.07
E2	GY	3A	AX.89449189	5.64E+08	0.07	-4.26	3.69	0.07

E2	GY	3A	AX.89449189	5.64E+08	0.07	-4.26	3.69	0.07
E2	GY	3A	AX.89553815	4.98E+08	0.06	-4.16	3.31	0.07
E2	GY	3A	AX.89553815	4.98E+08	0.06	-4.16	3.31	0.07
E2	GPM2	3A	AX.89502795	7.49E+08	0.08	-855.62	3.04	0.04
E2	GY	3A	AX.89734924	5.64E+08	0.07	-4.26	3.69	0.07
E2	GY	3A	AX.89734924	5.64E+08	0.07	-4.26	3.69	0.07
E2	GY	3A	AX.89460523	5.60E+08	0.07	-4.26	3.69	0.07
E2	GY	3A	AX.89590750	5.63E+08	0.07	-4.15	3.71	0.07
E2	GY	3A	AX.89590750	5.63E+08	0.07	-4.15	3.71	0.07
E2	GY	3A	AX.89520771	5.30E+08	0.07	-4.26	3.69	0.07
E2	GY	3A	AX.89520771	5.30E+08	0.07	-4.26	3.69	0.07
E2	GY	3A	AX.89543190	5.66E+08	0.07	-4.26	3.69	0.07
E2	GY	3A	AX.89543190	5.66E+08	0.07	-4.26	3.69	0.07
E2	GY	3A	AX.89543199	5.06E+08	0.06	-4.37	3.37	0.07
E2	GY	3A	AX.89543199	5.06E+08	0.06	-4.37	3.37	0.07
E2	GY	3A	AX.89310676	5.33E+08	0.07	-4.04	3.76	0.08
E2	GY	3A	AX.89310676	5.33E+08	0.07	-4.04	3.76	0.08
E2	GY	3A	AX.89509103	5.59E+08	0.07	-4.08	3.82	0.07
E2	GY	3A	AX.89509103	5.59E+08	0.07	-4.08	3.82	0.07
E2	GY	3A	AX.89626071	5.57E+08	0.06	-4.59	3.95	0.08
E2	GY	3A	AX.89774928	5.02E+08	0.07	-3.92	3.37	0.07
E2	GY	3A	AX.89774928	5.02E+08	0.07	-3.92	3.37	0.07
E2	GY	3A	AX.89521612	5.59E+08	0.07	-4.26	3.69	0.07
E2	GY	3A	AX.89521612	5.59E+08	0.07	-4.26	3.69	0.07
E2	GY	3A	AX.89679779	5.57E+08	0.07	-4.03	3.71	0.07
E2	GY	3A	AX.89679779	5.57E+08	0.07	-4.03	3.71	0.07
E2	GPS	3A	AX.89718821	6.12E+08	0.09	-2.59	3.81	0.08
E2	GY	3A	AX.89311399	5.57E+08	0.06	-4.59	3.95	0.08
E2	GY	3A	AX.89311399	5.57E+08	0.06	-4.59	3.95	0.08
E2	GY	3A	AX.89725821	5.30E+08	0.07	-4.26	3.69	0.07
E2	GY	3A	AX.89725821	5.30E+08	0.07	-4.26	3.69	0.07
E2	GY	3A	AX.89664608	5.64E+08	0.07	-4.15	3.71	0.07
E2	GY	3A	AX.89664608	5.64E+08	0.07	-4.15	3.71	0.07
E2	GY	3A	AX.89345293	5.30E+08	0.07	-4.26	3.69	0.07
E2	GY	3A	AX.89345293	5.30E+08	0.07	-4.26	3.69	0.07
E2	GY	3A	AX.89522243	5.64E+08	0.07	-4.26	3.69	0.07
E2	GY	3A	AX.89522243	5.64E+08	0.07	-4.26	3.69	0.07
E2	GY	3A	AX.89345380	5.30E+08	0.07	-4.26	3.69	0.07
E2	GY	3A	AX.89345380	5.30E+08	0.07	-4.26	3.69	0.07
E2	GY	3A	AX.89345419	5.56E+08	0.10	-3.52	3.64	0.08
E2	GY	3A	AX.89345419	5.56E+08	0.10	-3.52	3.64	0.08
E2	GY	3A	AX.89440110	5.60E+08	0.07	-4.26	3.69	0.07
E2	GY	3A	AX.89440110	5.60E+08	0.07	-4.26	3.69	0.07
E2	GY	3A	AX.89660590	5.67E+08	0.07	-4.26	3.69	0.07
E2	GY	3A	AX.89660590	5.67E+08	0.07	-4.26	3.69	0.07
E2	GY	3A	AX.89556344	5.67E+08	0.07	-4.26	3.69	0.07
E2	GY	3A	AX.89556344	5.67E+08	0.07	-4.26	3.69	0.07
E2	GY	3A	AX.89568518	5.64E+08	0.08	-3.74	3.43	0.07
E2	GY	3A	AX.89568518	5.64E+08	0.08	-3.74	3.43	0.07
E2	GY	3A	AX.89462670	5.60E+08	0.07	-4.26	3.69	0.07

E2	GY	3A	AX.89462670	5.60E+08	0.07	-4.26	3.69	0.07
E2	GY	3A	AX.89393527	5.30E+08	0.07	-4.26	3.69	0.07
E2	GY	3A	AX.89452058	5.59E+08	0.07	-4.15	3.71	0.07
E2	GY	3A	AX.89452058	5.59E+08	0.07	-4.15	3.71	0.07
E2	GPM2	3A	AX.89397235	7.49E+08	0.08	-880.17	3.04	0.04
E2	TKW	3A	AX.89768808	5.34E+08	0.17	-1.20	3.02	0.05
E2	TKW	3A	AX.89382610	7.44E+08	0.38	-1.11	4.11	0.08
E2	GY	3A	AX.89463371	5.61E+08	0.07	-4.26	3.69	0.07
E2	GY	3A	AX.89394430	5.56E+08	0.10	-3.52	3.64	0.08
E2	GY	3A	AX.89313638	5.62E+08	0.07	-4.15	3.71	0.07
E2	GY	3A	AX.89313638	5.62E+08	0.07	-4.15	3.71	0.07
E2	GY	3A	AX.89607044	5.64E+08	0.07	-4.26	3.69	0.07
E2	GY	3A	AX.89607044	5.64E+08	0.07	-4.26	3.69	0.07
E2	GY	3A	AX.89720963	5.63E+08	0.07	-4.26	3.69	0.07
E2	GY	3A	AX.89720963	5.63E+08	0.07	-4.26	3.69	0.07
E2	GY	3A	AX.89593477	5.30E+08	0.07	-4.26	3.69	0.07
E2	GY	3A	AX.89593477	5.30E+08	0.07	-4.26	3.69	0.07
E2	GY	3A	AX.89594762	5.59E+08	0.07	-4.26	3.69	0.07
E2	GY	3A	AX.89594762	5.59E+08	0.07	-4.26	3.69	0.07
E2	GY	3A	AX.89765446	5.59E+08	0.07	-4.26	3.69	0.07
E2	GY	3A	AX.89765446	5.59E+08	0.07	-4.26	3.69	0.07
E2	GY	3A	AX.89630315	5.61E+08	0.07	-4.26	3.69	0.07
E2	GY	3A	AX.89630315	5.61E+08	0.07	-4.26	3.69	0.07
E2	GY	3A	AX.89314273	5.17E+08	0.05	-4.70	3.51	0.07
E2	GY	3A	AX.89314273	5.17E+08	0.05	-4.70	3.51	0.07
E2	GY	3A	AX.89607530	5.57E+08	0.06	-4.59	3.95	0.08
E2	GY	3A	AX.89630400	4.98E+08	0.06	-4.16	3.31	0.07
E2	GY	3A	AX.89630400	4.98E+08	0.06	-4.16	3.31	0.07
E2	GPM2	3A	AX.89529388	7.49E+08	0.08	-855.62	3.04	0.04
E2	GY	3A	AX.89454165	5.61E+08	0.07	-4.55	4.13	0.08
E2	GY	3A	AX.89454165	5.61E+08	0.07	-4.55	4.13	0.08
E2	GY	3A	AX.89727512	5.68E+08	0.10	-3.52	3.64	0.08
E2	GY	3A	AX.89727512	5.68E+08	0.10	-3.52	3.64	0.08
E2	GY	3A	AX.89383700	5.60E+08	0.07	-4.26	3.69	0.07
E2	GY	3A	AX.89383700	5.60E+08	0.07	-4.26	3.69	0.07
E2	GY	3A	AX.89383890	5.61E+08	0.07	-4.26	3.69	0.07
E2	GY	3A	AX.89383890	5.61E+08	0.07	-4.26	3.69	0.07
E2	GY	3A	AX.89668618	5.57E+08	0.07	-4.15	3.71	0.07
E2	GY	3A	AX.89668618	5.57E+08	0.07	-4.15	3.71	0.07
E2	GY	3A	AX.89526633	5.17E+08	0.05	-5.07	3.72	0.07
E2	GY	3A	AX.89526633	5.17E+08	0.05	-5.07	3.72	0.07
E2	GY	3A	AX.89384580	5.62E+08	0.07	-4.26	3.69	0.07
E2	GY	3A	AX.89384580	5.62E+08	0.07	-4.26	3.69	0.07
E2	GY	3A	AX.89349395	5.59E+08	0.07	-4.15	3.71	0.07
E2	GY	3A	AX.89349395	5.59E+08	0.07	-4.15	3.71	0.07
E2	GY	3A	AX.89526840	5.61E+08	0.07	-4.26	3.69	0.07
E2	GY	3A	AX.89526840	5.61E+08	0.07	-4.26	3.69	0.07
E2	GY	3A	AX.89742239	5.29E+08	0.07	-4.26	3.69	0.07
E2	GY	3A	AX.89527187	5.33E+08	0.07	-4.04	3.76	0.08
E2	GY	3A	AX.89527187	5.33E+08	0.07	-4.04	3.76	0.08

E2	GY	3A	AX.89385367	5.64E+08	0.07	-4.26	3.69	0.07
E2	GY	3A	AX.89385367	5.64E+08	0.07	-4.26	3.69	0.07
E2	GY	3A	AX.89385489	5.65E+08	0.07	-4.26	3.69	0.07
E2	GY	3A	AX.89385489	5.65E+08	0.07	-4.26	3.69	0.07
E2	GY	3A	AX.89375055	5.30E+08	0.07	-4.26	3.69	0.07
E2	GY	3A	AX.89375055	5.30E+08	0.07	-4.26	3.69	0.07
E2	GY	3A	AX.89516559	5.60E+08	0.07	-4.26	3.69	0.07
E2	GY	3A	AX.89516559	5.60E+08	0.07	-4.26	3.69	0.07
E2	GY	3A	AX.89744040	5.16E+08	0.05	-4.80	3.69	0.08
E2	GY	3A	AX.89744040	5.16E+08	0.05	-4.80	3.69	0.08
E2	GY	3A	AX.89682331	5.59E+08	0.07	-4.26	3.69	0.07
E2	GY	3A	AX.89438779	5.57E+08	0.07	-4.26	3.69	0.07
E2	GY	3A	AX.89438779	5.57E+08	0.07	-4.26	3.69	0.07
E2	GY	3A	AX.89386252	5.57E+08	0.06	-4.59	3.95	0.08
E2	GY	3A	AX.89386252	5.57E+08	0.06	-4.59	3.95	0.08
E2	GY	3A	AX.89598565	5.64E+08	0.07	-4.15	3.71	0.07
E2	GY	3A	AX.89481801	5.59E+08	0.07	-4.26	3.69	0.07
E2	GY	3A	AX.89481801	5.59E+08	0.07	-4.26	3.69	0.07
E2	GY	3A	AX.89716645	5.30E+08	0.07	-4.26	3.69	0.07
E2	GY	3A	AX.89716645	5.30E+08	0.07	-4.26	3.69	0.07
E2	GY	3A	AX.89562813	5.63E+08	0.07	-4.26	3.69	0.07
E2	GY	3A	AX.89562813	5.63E+08	0.07	-4.26	3.69	0.07
E2	GY	3A	AX.89597375	5.30E+08	0.07	-4.26	3.69	0.07
E2	GY	3A	AX.89704455	5.33E+08	0.08	-3.96	3.79	0.07
E2	GY	3A	AX.89704455	5.33E+08	0.08	-3.96	3.79	0.07
E2	GY	3A	AX.89599695	5.30E+08	0.07	-4.26	3.69	0.07
E2	GY	3A	AX.89599695	5.30E+08	0.07	-4.26	3.69	0.07
E2	GY	3A	AX.89750188	5.33E+08	0.07	-4.04	3.76	0.08
E2	GY	3A	AX.89750188	5.33E+08	0.07	-4.04	3.76	0.08
E2	GY	3A	AX.89387248	5.59E+08	0.07	-4.26	3.69	0.07
E2	GY	3A	AX.89387248	5.59E+08	0.07	-4.26	3.69	0.07
E2	GY	3A	AX.89423623	5.61E+08	0.07	-4.26	3.69	0.07
E2	GY	3A	AX.89423623	5.61E+08	0.07	-4.26	3.69	0.07
E2	GY	3A	AX.89457642	5.57E+08	0.06	-4.59	3.95	0.08
E2	GY	3A	AX.89457642	5.57E+08	0.06	-4.59	3.95	0.08
E2	GY	3A	AX.89756665	5.31E+08	0.07	-4.26	3.69	0.07
E2	GY	3A	AX.89563886	5.17E+08	0.05	-5.07	3.72	0.07
E2	GY	3A	AX.89563886	5.17E+08	0.05	-5.07	3.72	0.07
E2	GY	3A	AX.89635567	5.32E+08	0.07	-4.26	3.69	0.07
E2	GY	3A	AX.89635567	5.32E+08	0.07	-4.26	3.69	0.07
E2	GY	3A	AX.89720247	5.64E+08	0.07	-4.26	3.69	0.07
E2	GY	3A	AX.89720247	5.64E+08	0.07	-4.26	3.69	0.07
E2	GPM2	3A	AX.89636747	5.56E+08	0.10	-811.26	3.06	0.06
E2	GY	3A	AX.89353322	5.64E+08	0.07	-4.26	3.69	0.07
E2	GY	3A	AX.89353322	5.64E+08	0.07	-4.26	3.69	0.07
E2	GY	3A	AX.89319693	5.59E+08	0.07	-4.26	3.69	0.07
E2	GY	3A	AX.89319693	5.59E+08	0.07	-4.26	3.69	0.07
E2	GY	3A	AX.89757136	5.30E+08	0.07	-4.26	3.69	0.07
E2	GY	3A	AX.89757136	5.30E+08	0.07	-4.26	3.69	0.07
E2	GY	3A	AX.89564453	5.30E+08	0.07	-4.26	3.69	0.07

E2	GY	3A	AX.89564453	5.30E+08	0.07	-4.26	3.69	0.07
E2	GY	3A	AX.89612125	5.58E+08	0.07	-4.15	3.71	0.07
E2	GY	3A	AX.89518939	5.57E+08	0.07	-4.03	3.71	0.07
E2	GY	3A	AX.89518939	5.57E+08	0.07	-4.03	3.71	0.07
E2	GY	3A	AX.89673581	5.33E+08	0.07	-4.04	3.76	0.08
E2	GY	3A	AX.89655976	5.65E+08	0.07	-4.26	3.69	0.07
E2	GY	3A	AX.89655976	5.65E+08	0.07	-4.26	3.69	0.07
E2	GY	3A	AX.89601255	5.59E+08	0.07	-4.26	3.69	0.07
E2	GY	3A	AX.89601255	5.59E+08	0.07	-4.26	3.69	0.07
E2	GY	3A	AX.89458977	5.62E+08	0.07	-4.26	3.69	0.07
E2	GY	3A	AX.89458977	5.62E+08	0.07	-4.26	3.69	0.07
E2	GY	3A	AX.89734810	5.33E+08	0.07	-4.04	3.76	0.08
E2	GY	3A	AX.89601456	5.32E+08	0.07	-4.26	3.69	0.07
E2	GY	3A	AX.89754538	5.61E+08	0.07	-4.26	3.69	0.07
E2	GY	3A	AX.89754538	5.61E+08	0.07	-4.26	3.69	0.07
E2	GY	3A	AX.89726743	5.29E+08	0.07	-4.26	3.69	0.07
E2	GY	3A	AX.89496168	5.64E+08	0.07	-4.26	3.69	0.07
E2	GY	3A	AX.89496168	5.64E+08	0.07	-4.26	3.69	0.07
E2	GY	3A	AX.89321584	5.30E+08	0.07	-4.26	3.69	0.07
E2	GY	3A	AX.89321584	5.30E+08	0.07	-4.26	3.69	0.07
E2	GY	3A	AX.89460048	5.30E+08	0.07	-4.26	3.69	0.07
E2	GY	3A	AX.89460048	5.30E+08	0.07	-4.26	3.69	0.07
E2	GY	3A	AX.89496396	5.57E+08	0.06	-4.93	4.17	0.08
E2	GY	3A	AX.89496396	5.57E+08	0.06	-4.93	4.17	0.08
E2	GY	3A	AX.89460523	5.60E+08	0.07	-4.26	3.69	0.07
E2	GY	3A	AX.89670992	5.64E+08	0.07	-4.26	3.69	0.07
E2	GY	3A	AX.89670992	5.64E+08	0.07	-4.26	3.69	0.07
E2	GY	3A	AX.89344238	5.63E+08	0.07	-4.26	3.69	0.07
E2	GY	3A	AX.89344238	5.63E+08	0.07	-4.26	3.69	0.07
E2	GY	3A	AX.89733627	5.59E+08	0.07	-4.26	3.69	0.07
E2	GY	3A	AX.89733627	5.59E+08	0.07	-4.26	3.69	0.07
E2	GPM2	3A	AX.89331029	7.50E+08	0.08	-880.17	3.04	0.04
E2	GY	3A	AX.89703808	5.63E+08	0.07	-4.26	3.69	0.07
E2	GY	3A	AX.89703808	5.63E+08	0.07	-4.26	3.69	0.07
E2	GY	3A	AX.89567012	5.58E+08	0.07	-4.26	3.69	0.07
E2	GY	3A	AX.89567012	5.58E+08	0.07	-4.26	3.69	0.07
E2	GY	3A	AX.89322989	5.44E+08	0.05	-4.80	3.69	0.08
E2	GY	3A	AX.89322989	5.44E+08	0.05	-4.80	3.69	0.08
E2	GY	3A	AX.89676497	5.59E+08	0.07	-4.26	3.69	0.07
E2	GY	3A	AX.89676497	5.59E+08	0.07	-4.26	3.69	0.07
E2	GY	3A	AX.89567808	5.58E+08	0.07	-4.26	3.69	0.07
E2	GY	3A	AX.89497689	7.37E+08	0.18	-2.47	3.08	0.05
E2	GY	3A	AX.89497689	7.37E+08	0.18	-2.47	3.08	0.05
E2	GY	3A	AX.89713866	5.62E+08	0.07	-4.26	3.69	0.07
E2	GY	3A	AX.89713866	5.62E+08	0.07	-4.26	3.69	0.07
E2	GY	3A	AX.89718518	5.63E+08	0.06	-4.52	3.87	0.08
E2	GY	3A	AX.89718518	5.63E+08	0.06	-4.52	3.87	0.08
E2	GY	3A	AX.89677070	5.60E+08	0.07	-4.26	3.69	0.07
E2	GY	3A	AX.89677070	5.60E+08	0.07	-4.26	3.69	0.07
E2	GY	3A	AX.89357905	7.37E+08	0.18	-2.47	3.08	0.05

E2	GY	3A	AX.89357905	7.37E+08	0.18	-2.47	3.08	0.05
E2	GY	3A	AX.89677780	5.30E+08	0.07	-4.26	3.69	0.07
E2	GY	3A	AX.89677780	5.30E+08	0.07	-4.26	3.69	0.07
E2	GY	3A	AX.89393527	5.30E+08	0.07	-4.26	3.69	0.07
E2	GY	3A	AX.89715587	5.57E+08	0.07	-4.26	3.69	0.07
E2	GY	3A	AX.89715587	5.57E+08	0.07	-4.26	3.69	0.07
E2	GY	3A	AX.89393792	5.31E+08	0.07	-4.26	3.69	0.07
E2	GY	3A	AX.89393792	5.31E+08	0.07	-4.26	3.69	0.07
E2	GY	3A	AX.89358655	5.57E+08	0.07	-4.26	3.69	0.07
E2	GY	3A	AX.89358655	5.57E+08	0.07	-4.26	3.69	0.07
E2	GY	3A	AX.89557585	5.34E+08	0.07	-4.04	3.76	0.08
E2	GY	3A	AX.89557585	5.34E+08	0.07	-4.04	3.76	0.08
E2	GY	3A	AX.89463371	5.61E+08	0.07	-4.26	3.69	0.07
E2	GY	3A	AX.89394430	5.56E+08	0.10	-3.52	3.64	0.08
E2	GY	3A	AX.89573222	5.33E+08	0.07	-4.04	3.76	0.08
E2	GY	3A	AX.89573222	5.33E+08	0.07	-4.04	3.76	0.08
E2	GY	3A	AX.89629072	5.57E+08	0.07	-4.26	3.69	0.07
E2	GY	3A	AX.89629072	5.57E+08	0.07	-4.26	3.69	0.07
E2	GY	3A	AX.89768652	5.63E+08	0.07	-4.26	3.69	0.07
E2	GY	3A	AX.89768652	5.63E+08	0.07	-4.26	3.69	0.07
E2	GY	3A	AX.89777808	5.68E+08	0.07	-4.26	3.69	0.07
E2	GY	3A	AX.89777808	5.68E+08	0.07	-4.26	3.69	0.07
E2	GY	3A	AX.89535483	5.65E+08	0.07	-4.26	3.69	0.07
E2	GY	3A	AX.89535483	5.65E+08	0.07	-4.26	3.69	0.07
E2	GY	3A	AX.89594743	5.56E+08	0.10	-3.52	3.64	0.08
E2	GY	3A	AX.89594743	5.56E+08	0.10	-3.52	3.64	0.08
E2	GY	3A	AX.89395329	5.66E+08	0.07	-4.26	3.69	0.07
E2	GY	3A	AX.89395329	5.66E+08	0.07	-4.26	3.69	0.07
E2	GY	3A	AX.89431307	5.57E+08	0.06	-4.59	3.95	0.08
E2	GY	3A	AX.89431307	5.57E+08	0.06	-4.59	3.95	0.08
E2	GY	3A	AX.89679311	5.33E+08	0.07	-4.04	3.76	0.08
E2	GY	3A	AX.89679311	5.33E+08	0.07	-4.04	3.76	0.08
E2	GY	3A	AX.89607530	5.57E+08	0.06	-4.59	3.95	0.08
E2	GY	3A	AX.89326532	5.65E+08	0.07	-4.26	3.69	0.07
E2	GY	3A	AX.89326532	5.65E+08	0.07	-4.26	3.69	0.07
E2	GY	3A	AX.89464981	5.30E+08	0.07	-4.26	3.69	0.07
E2	GY	3A	AX.89464981	5.30E+08	0.07	-4.26	3.69	0.07
E2	GY	3A	AX.89326782	5.02E+08	0.07	-3.78	3.01	0.06
E2	GY	3A	AX.89326782	5.02E+08	0.07	-3.78	3.01	0.06
E2	GY	3A	AX.89638545	4.97E+08	0.06	-4.16	3.31	0.07
E2	GY	3A	AX.89360632	5.30E+08	0.07	-4.26	3.69	0.07
E2	GY	3A	AX.89360632	5.30E+08	0.07	-4.26	3.69	0.07
E2	GY	3A	AX.89326956	5.68E+08	0.10	-3.52	3.64	0.08
E2	GY	3A	AX.89326956	5.68E+08	0.10	-3.52	3.64	0.08
E2	GY	3A	AX.89536540	5.31E+08	0.07	-4.26	3.69	0.07
E2	GY	3A	AX.89536540	5.31E+08	0.07	-4.26	3.69	0.07
E2	GY	3A	AX.89327064	5.56E+08	0.06	-4.59	3.95	0.08
E2	GY	3A	AX.89327064	5.56E+08	0.06	-4.59	3.95	0.08
E2	GY	3A	AX.89327256	5.30E+08	0.07	-4.26	3.69	0.07
E2	GY	3A	AX.89327256	5.30E+08	0.07	-4.26	3.69	0.07

E2	GY	3A	AX.89327279	5.58E+08	0.07	-4.26	3.69	0.07
E2	GY	3A	AX.89327279	5.58E+08	0.07	-4.26	3.69	0.07
E2	GY	3A	AX.89762706	5.30E+08	0.07	-4.26	3.69	0.07
E2	GY	3A	AX.89762706	5.30E+08	0.07	-4.26	3.69	0.07
E2	GY	3A	AX.89742239	5.29E+08	0.07	-4.26	3.69	0.07
E2	GY	3A	AX.89398105	5.61E+08	0.07	-4.26	3.69	0.07
E2	GY	3A	AX.89398105	5.61E+08	0.07	-4.26	3.69	0.07
E2	GY	3A	AX.89433768	5.62E+08	0.07	-4.26	3.69	0.07
E2	GY	3A	AX.89573025	5.63E+08	0.07	-4.26	3.69	0.07
E2	GY	3A	AX.89573025	5.63E+08	0.07	-4.26	3.69	0.07
E2	GY	3A	AX.89569139	5.30E+08	0.07	-4.26	3.69	0.07
E2	GY	3A	AX.89569139	5.30E+08	0.07	-4.26	3.69	0.07
E2	GY	3A	AX.89695116	5.24E+08	0.05	-5.07	3.72	0.07
E2	GY	3A	AX.89467726	5.31E+08	0.07	-3.98	3.47	0.07
E2	GY	3A	AX.89467726	5.31E+08	0.07	-3.98	3.47	0.07
E2	GY	3A	AX.89528383	5.63E+08	0.07	-4.26	3.69	0.07
E2	GY	3A	AX.89411123	5.59E+08	0.07	-4.26	3.69	0.07
E2	GY	3A	AX.89682331	5.59E+08	0.07	-4.26	3.69	0.07
E2	GY	3A	AX.89707852	5.59E+08	0.07	-4.26	3.69	0.07
E2	GY	3A	AX.89573566	5.58E+08	0.07	-4.26	3.69	0.07
E2	GY	3A	AX.89573566	5.58E+08	0.07	-4.26	3.69	0.07
E2	GY	3A	AX.89597375	5.30E+08	0.07	-4.26	3.69	0.07
E2	TKW	3A	AX.89766135	5.32E+08	0.16	-1.25	3.11	0.05
E2	GY	3A	AX.89493587	5.57E+08	0.06	-4.59	3.95	0.08
E2	GY	3A	AX.89329561	5.61E+08	0.07	-4.26	3.69	0.07
E2	GY	3A	AX.89423647	5.56E+08	0.10	-3.52	3.64	0.08
E2	GY	3A	AX.89423647	5.56E+08	0.10	-3.52	3.64	0.08
E2	GY	3A	AX.89435715	5.30E+08	0.07	-4.26	3.69	0.07
E2	GY	3A	AX.89756665	5.31E+08	0.07	-4.26	3.69	0.07
E2	GY	3A	AX.89435911	5.59E+08	0.07	-4.26	3.69	0.07
E2	GY	3A	AX.89469542	4.98E+08	0.06	-4.16	3.31	0.07
E2	GY	3A	AX.89619325	5.29E+08	0.07	-4.26	3.69	0.07
E2	GY	3A	AX.89619325	5.29E+08	0.07	-4.26	3.69	0.07
E2	GY	3A	AX.89506121	5.66E+08	0.07	-4.26	3.69	0.07
E2	GPM2	3A	AX.89318515	7.49E+08	0.08	-855.62	3.04	0.04
E2	GY	3A	AX.89588092	5.57E+08	0.07	-4.26	3.69	0.07
E2	GY	3A	AX.89726130	5.63E+08	0.07	-4.26	3.69	0.07
E2	GY	3A	AX.89365214	5.31E+08	0.07	-4.26	3.69	0.07
E2	GY	3A	AX.89361362	5.31E+08	0.06	-4.59	3.95	0.08
E2	GY	3A	AX.89611948	5.33E+08	0.07	-4.04	3.76	0.08
E2	GY	3A	AX.89612125	5.58E+08	0.07	-4.15	3.71	0.07
E2	GY	3A	AX.89698099	5.30E+08	0.07	-4.26	3.69	0.07
E2	GY	3A	AX.89601022	5.66E+08	0.06	-4.59	3.95	0.08
E2	GY	3A	AX.89601022	5.66E+08	0.06	-4.59	3.95	0.08
E2	GPM2	3A	AX.89577150	7.50E+08	0.08	-880.17	3.04	0.04
E2	GY	3A	AX.89402066	7.19E+08	0.22	-2.33	3.14	0.05
E2	GY	3A	AX.89402066	7.19E+08	0.22	-2.33	3.14	0.05
E2	TKW	3A	AX.89463665	5.32E+08	0.16	-1.25	3.11	0.05
E2	GY	3A	AX.89437126	5.58E+08	0.07	-4.26	3.69	0.07
E2	GY	3A	AX.89589319	5.59E+08	0.07	-4.26	3.69	0.07

E2	GY	3A	AX.89711026	5.33E+08	0.07	-4.04	3.76	0.08
E2	GY	3A	AX.89711026	5.33E+08	0.07	-4.04	3.76	0.08
E2	GY	3A	AX.89636747	5.56E+08	0.10	-3.52	3.64	0.08
E2	GY	3A	AX.89636747	5.56E+08	0.10	-3.52	3.64	0.08
E2	GY	3A	AX.89647632	5.57E+08	0.07	-4.26	3.69	0.07
E2	GY	3A	AX.89511769	5.59E+08	0.07	-4.26	3.69	0.07
E2	GY	3A	AX.89471709	5.56E+08	0.06	-4.59	3.95	0.08
E2	GY	3A	AX.89471709	5.56E+08	0.06	-4.59	3.95	0.08
E2	GY	3A	AX.89613280	5.59E+08	0.07	-4.26	3.69	0.07
E2	GPM2	3A	AX.89345419	5.56E+08	0.10	-811.26	3.06	0.06
E2	GY	3A	AX.89728466	4.99E+08	0.06	-4.16	3.31	0.07
E2	GY	3A	AX.89720378	5.65E+08	0.07	-4.26	3.69	0.07
E2	GY	3A	AX.89720378	5.65E+08	0.07	-4.26	3.69	0.07
E2	GY	3A	AX.89343454	5.62E+08	0.07	-4.45	4.18	0.08
E2	GY	3A	AX.89343454	5.62E+08	0.07	-4.45	4.18	0.08
E2	GY	3A	AX.89674842	5.60E+08	0.07	-4.26	3.69	0.07
E2	GY	3A	AX.89613713	5.57E+08	0.06	-4.59	3.95	0.08
E2	GY	3A	AX.89554083	5.64E+08	0.07	-4.26	3.69	0.07
E2	TKW	3A	AX.89557687	5.32E+08	0.16	-1.25	3.11	0.05
E2	GY	3A	AX.89414910	5.57E+08	0.06	-4.59	3.95	0.08
E2	GY	3A	AX.89414910	5.57E+08	0.06	-4.59	3.95	0.08
E2	GY	3A	AX.89748200	5.66E+08	0.07	-4.26	3.69	0.07
E2	GY	3A	AX.89712503	5.58E+08	0.07	-4.26	3.69	0.07
E2	GY	3A	AX.89460831	5.29E+08	0.07	-4.26	3.69	0.07
E2	GY	3A	AX.89427057	5.63E+08	0.07	-4.26	3.69	0.07
E2	GY	3A	AX.89590996	5.31E+08	0.07	-4.26	3.69	0.07
E2	GY	3A	AX.89554912	5.66E+08	0.07	-4.26	3.69	0.07
E2	TKW	3A	AX.89644673	5.34E+08	0.17	-1.20	3.02	0.05
E2	GY	3A	AX.89614818	5.59E+08	0.07	-4.26	3.69	0.07
E2	GY	3A	AX.89497356	5.60E+08	0.07	-4.26	3.69	0.07
E2	GY	3A	AX.89638327	5.66E+08	0.07	-4.26	3.69	0.07
E2	GY	3A	AX.89638327	5.66E+08	0.07	-4.26	3.69	0.07
E2	GY	3A	AX.89418431	5.65E+08	0.06	-5.01	4.31	0.08
E2	GY	3A	AX.89626731	5.33E+08	0.07	-4.04	3.76	0.08
E2	GY	3A	AX.89688752	5.56E+08	0.06	-4.59	3.95	0.08
E2	GY	3A	AX.89701379	5.20E+08	0.05	-6.52	5.90	0.12
E2	GY	3A	AX.89540978	5.44E+08	0.05	-5.07	3.72	0.07
E2	GY	3A	AX.89583436	5.57E+08	0.06	-4.59	3.95	0.08
E2	GY	3A	AX.89497931	5.57E+08	0.07	-4.26	3.69	0.07
E2	GY	3A	AX.89497931	5.57E+08	0.07	-4.26	3.69	0.07
E2	GY	3A	AX.89541040	5.16E+08	0.05	-4.80	3.69	0.08
E2	GY	3A	AX.89558874	5.66E+08	0.06	-4.59	3.95	0.08
E2	GY	3A	AX.89487853	5.57E+08	0.07	-4.26	3.69	0.07
E2	GY	3A	AX.89673529	5.66E+08	0.07	-4.26	3.69	0.07
E2	GY	3A	AX.89673529	5.66E+08	0.07	-4.26	3.69	0.07
E2	GY	3A	AX.89665504	5.64E+08	0.07	-4.26	3.69	0.07
E2	GY	3A	AX.89665504	5.64E+08	0.07	-4.26	3.69	0.07
E2	GY	3A	AX.89545138	5.57E+08	0.07	-4.26	3.69	0.07
E2	GY	3A	AX.89556791	5.64E+08	0.06	-4.52	3.87	0.08
E2	GY	3A	AX.89556791	5.64E+08	0.06	-4.52	3.87	0.08

E2	GPM2	3A	AX.89353440	7.50E+08	0.08	-880.17	3.04	0.04
E2	GY	3A	AX.89702635	5.33E+08	0.07	-4.04	3.76	0.08
E2	GY	3A	AX.89405683	5.60E+08	0.07	-4.26	3.69	0.07
E2	GY	3A	AX.89530615	5.64E+08	0.07	-4.26	3.69	0.07
E2	GY	3A	AX.89530615	5.64E+08	0.07	-4.26	3.69	0.07
E2	GY	3A	AX.89358066	5.33E+08	0.07	-4.04	3.76	0.08
E2	GY	3A	AX.89370365	5.29E+08	0.07	-4.26	3.69	0.07
E2	GY	3A	AX.89440859	5.33E+08	0.07	-4.04	3.76	0.08
E2	GY	3A	AX.89440859	5.33E+08	0.07	-4.04	3.76	0.08
E2	GY	3A	AX.89334785	5.64E+08	0.07	-4.26	3.69	0.07
E2	GY	3A	AX.89585123	5.16E+08	0.05	-4.80	3.69	0.08
E2	GY	3A	AX.89406714	5.33E+08	0.07	-4.04	3.76	0.08
E2	GY	3A	AX.89406714	5.33E+08	0.07	-4.04	3.76	0.08
E2	TKW	3A	AX.89497520	7.44E+08	0.35	-0.99	3.30	0.06
E2	GY	3A	AX.89407423	5.38E+07	0.07	-4.67	4.21	0.08
E2	GY	3A	AX.89603026	5.57E+08	0.05	-5.57	4.47	0.09
E2	GY	3A	AX.89603026	5.57E+08	0.05	-5.57	4.47	0.09
E2	TKW	3A	AX.89690684	7.44E+08	0.30	-1.01	3.17	0.05
E2	GY	3A	AX.89477075	5.57E+08	0.08	-3.80	3.51	0.07
E2	GPS	3A	AX.89504837	6.13E+08	0.10	-2.36	3.51	0.07
E2	GY	3A	AX.89606047	5.30E+08	0.07	-4.26	3.69	0.07
E2	GY	3A	AX.89675998	5.30E+08	0.06	-4.70	4.13	0.08
E2	GY	3A	AX.89408054	5.62E+08	0.07	-4.26	3.69	0.07
E2	GY	3A	AX.89562144	5.57E+08	0.06	-4.59	3.95	0.08
E2	GY	3A	AX.89562144	5.57E+08	0.06	-4.59	3.95	0.08
E2	GY	3A	AX.89477864	5.66E+08	0.07	-4.26	3.69	0.07
E2	GY	3A	AX.89477864	5.66E+08	0.07	-4.26	3.69	0.07
E2	GY	3A	AX.89337100	5.65E+08	0.07	-4.26	3.69	0.07
E2	GY	3A	AX.89701396	5.59E+08	0.07	-4.26	3.69	0.07
E2	GY	3A	AX.89701396	5.59E+08	0.07	-4.26	3.69	0.07
E2	GPM2	3A	AX.89573484	7.50E+08	0.08	-880.17	3.04	0.04
E2	GPM2	3A	AX.89501429	7.49E+08	0.08	-880.17	3.04	0.04
E2	GY	3A	AX.89361438	5.57E+08	0.06	-4.59	3.95	0.08
E2	GY	3A	AX.89361438	5.57E+08	0.06	-4.59	3.95	0.08
E2	GY	3A	AX.89778275	5.58E+08	0.07	-4.26	3.69	0.07
E2	GY	3A	AX.89338270	5.34E+08	0.07	-4.04	3.76	0.08
E2	GPM2	3A	AX.89445873	5.57E+08	0.05	-1346.35	4.07	0.08
E2	GY	3A	AX.89681123	5.62E+08	0.07	-4.26	3.69	0.07
E2	GPM2	3A	AX.89356571	7.49E+08	0.08	-855.62	3.04	0.04
E2	GY	3A	AX.89702492	5.56E+08	0.10	-3.36	3.49	0.07
E2	GY	3A	AX.89702492	5.56E+08	0.10	-3.36	3.49	0.07
E2	GY	3A	AX.89410241	5.01E+08	0.06	-4.16	3.31	0.07
E2	GY	3A	AX.89433768	5.62E+08	0.07	-4.26	3.69	0.07
E2	GY	3A	AX.89695116	5.24E+08	0.05	-5.07	3.72	0.07
E2	GPM2	3A	AX.89540925	7.48E+08	0.08	-880.17	3.04	0.04
E2	GY	3A	AX.89561819	5.65E+08	0.07	-4.26	3.69	0.07
E2	GY	3A	AX.89626214	5.58E+08	0.07	-4.26	3.69	0.07
E2	GY	3A	AX.89375166	5.57E+08	0.06	-5.33	4.82	0.10
E2	GY	3A	AX.89751312	5.57E+08	0.08	-3.80	3.51	0.07
E2	GY	3A	AX.89695735	5.56E+08	0.10	-3.36	3.49	0.07

E2	GY	3A	AX.89542448	5.30E+08	0.07	-4.26	3.69	0.07
E2	GY	3A	AX.89654794	5.64E+08	0.07	-4.26	3.69	0.07
E2	GY	3A	AX.89567996	5.33E+08	0.07	-4.04	3.76	0.08
E2	GY	3A	AX.89468906	5.66E+08	0.07	-4.26	3.69	0.07
E2	GY	3A	AX.89468906	5.66E+08	0.07	-4.26	3.69	0.07
E2	GY	3A	AX.89483106	5.63E+08	0.07	-4.26	3.69	0.07
E2	GY	3A	AX.89483106	5.63E+08	0.07	-4.26	3.69	0.07
E2	GY	3A	AX.89629820	5.28E+08	0.05	-5.07	3.72	0.07
E2	GY	3A	AX.89629820	5.28E+08	0.05	-5.07	3.72	0.07
E2	GY	3A	AX.89412712	5.58E+08	0.07	-4.26	3.69	0.07
E2	GY	3A	AX.89412712	5.58E+08	0.07	-4.26	3.69	0.07
E2	GY	3A	AX.89469542	4.98E+08	0.06	-4.16	3.31	0.07
E2	GPS	3A	AX.89324539	6.12E+08	0.09	-2.59	3.81	0.08
E2	GY	3A	AX.89701379	5.20E+08	0.05	-6.52	5.90	0.12
E2	GY	3A	AX.89361362	5.31E+08	0.06	-4.59	3.95	0.08
E2	GY	3A	AX.89770986	5.66E+08	0.07	-4.26	3.69	0.07
E2	GY	3A	AX.89770986	5.66E+08	0.07	-4.26	3.69	0.07
E2	GPM2	3A	AX.89312937	7.48E+08	0.08	-880.17	3.04	0.04
E2	GY	3A	AX.89460171	5.58E+08	0.07	-4.26	3.69	0.07
E2	GY	3A	AX.89655270	5.65E+08	0.07	-4.26	3.69	0.07
E2	GY	3A	AX.89587846	5.67E+08	0.07	-4.26	3.69	0.07
E2	GY	3A	AX.89587846	5.67E+08	0.07	-4.26	3.69	0.07
E2	GY	3A	AX.89707382	5.17E+08	0.05	-4.53	3.32	0.07
E2	GY	3A	AX.89773033	5.62E+08	0.07	-4.26	3.69	0.07
E2	GY	3A	AX.89511769	5.59E+08	0.07	-4.26	3.69	0.07
E2	GY	3A	AX.89437577	5.56E+08	0.06	-4.89	4.16	0.08
E2	GY	3A	AX.89437577	5.56E+08	0.06	-4.89	4.16	0.08
E2	GY	3A	AX.89366707	5.33E+08	0.07	-4.04	3.76	0.08
E2	GY	3A	AX.89366707	5.33E+08	0.07	-4.04	3.76	0.08
E2	GY	3A	AX.89507644	5.57E+08	0.07	-4.26	3.69	0.07
E2	GY	3A	AX.89507644	5.57E+08	0.07	-4.26	3.69	0.07
E2	GY	3A	AX.89728466	4.99E+08	0.06	-4.16	3.31	0.07
E2	GY	3A	AX.89542323	5.66E+08	0.07	-4.26	3.69	0.07
E2	GY	3A	AX.89674842	5.60E+08	0.07	-4.26	3.69	0.07
E2	GY	3A	AX.89426061	5.32E+08	0.06	-4.52	3.87	0.08
E2	GY	3A	AX.89426061	5.32E+08	0.06	-4.52	3.87	0.08
E2	GY	3A	AX.89390420	5.60E+08	0.07	-4.26	3.69	0.07
E2	GY	3A	AX.89426725	5.56E+08	0.06	-4.59	3.95	0.08
E2	GPS	3A	AX.89580701	6.13E+08	0.10	-2.36	3.51	0.07
E2	GY	3A	AX.89760649	5.30E+08	0.07	-4.15	3.71	0.07
E2	GPS	3A	AX.89659165	1.95E+07	0.18	-1.74	3.12	0.05
E2	GPS	3A	AX.89391411	6.13E+08	0.10	-2.36	3.51	0.07
E2	GY	3A	AX.89590996	5.31E+08	0.07	-4.26	3.69	0.07
E2	GY	3A	AX.89509264	5.16E+08	0.05	-4.80	3.69	0.08
E2	GY	3A	AX.89509264	5.16E+08	0.05	-4.80	3.69	0.08
E2	GY	3A	AX.89614818	5.59E+08	0.07	-4.26	3.69	0.07
E2	GY	3A	AX.89418431	5.65E+08	0.06	-5.01	4.31	0.08
E2	GY	3A	AX.89626731	5.33E+08	0.07	-4.04	3.76	0.08
E2	GPM2	3A	AX.89380387	7.49E+08	0.08	-855.62	3.04	0.04
E2	TKW	3A	AX.89477257	7.44E+08	0.30	-1.01	3.17	0.05

E2	GY	3A	AX.89345044	5.30E+08	0.06	-4.59	3.95	0.08
E2	GY	3A	AX.89345044	5.30E+08	0.06	-4.59	3.95	0.08
E2	GY	3A	AX.89345164	5.31E+08	0.07	-4.26	3.69	0.07
E2	GY	3A	AX.89345164	5.31E+08	0.07	-4.26	3.69	0.07
E2	GY	3A	AX.89558874	5.66E+08	0.06	-4.59	3.95	0.08
E2	GY	3A	AX.89604406	5.31E+08	0.07	-4.26	3.69	0.07
E2	TKW	3A	AX.89702309	4.80E+08	0.22	-1.20	3.58	0.06
E2	TKW	3A	AX.89767042	5.32E+08	0.16	-1.25	3.11	0.05
E2	GY	3A	AX.89714454	5.31E+08	0.07	-4.26	3.69	0.07
E2	GY	3A	AX.89714454	5.31E+08	0.07	-4.26	3.69	0.07
E2	GY	3A	AX.89345856	5.29E+08	0.07	-4.26	3.69	0.07
E2	GY	3A	AX.89345856	5.29E+08	0.07	-4.26	3.69	0.07
E2	GY	3A	AX.89393225	5.33E+08	0.07	-4.04	3.76	0.08
E2	GPM2	3A	AX.89508524	7.48E+08	0.08	-880.17	3.04	0.04
E2	GY	3A	AX.89498962	2.02E+07	0.05	-5.09	3.77	0.08
E2	GY	3A	AX.89690071	5.66E+08	0.07	-4.26	3.69	0.07
E2	GY	3A	AX.89686328	5.29E+08	0.07	-4.26	3.69	0.07
E2	GY	3A	AX.89358865	5.29E+08	0.07	-4.26	3.69	0.07
E2	GY	3A	AX.89358865	5.29E+08	0.07	-4.26	3.69	0.07
E2	GY	3A	AX.89499718	5.31E+08	0.07	-4.26	3.69	0.07
E2	GY	3A	AX.89499718	5.31E+08	0.07	-4.26	3.69	0.07
E2	GY	3A	AX.89662284	5.63E+08	0.05	-4.80	3.45	0.07
E2	GY	3A	AX.89662284	5.63E+08	0.05	-4.80	3.45	0.07
E2	GY	3A	AX.89581530	5.61E+08	0.07	-4.26	3.69	0.07
E2	GY	3A	AX.89581530	5.61E+08	0.07	-4.26	3.69	0.07
E2	GPM2	3A	AX.89719342	7.50E+08	0.08	-880.17	3.04	0.04
E2	GY	3A	AX.89679330	5.63E+08	0.07	-4.15	3.71	0.07
E2	GY	3A	AX.89679330	5.63E+08	0.07	-4.15	3.71	0.07
E2	GPS	3A	AX.89717399	6.13E+08	0.10	-2.36	3.51	0.07
E2	GPM2	3A	AX.89311476	7.49E+08	0.08	-880.17	3.04	0.04
E2	GY	3A	AX.89700017	5.58E+08	0.06	-4.59	3.95	0.08
E2	GY	3A	AX.89700017	5.58E+08	0.06	-4.59	3.95	0.08
E2	GY	3A	AX.89704338	5.57E+08	0.07	-4.26	3.69	0.07
E2	GY	3A	AX.89704338	5.57E+08	0.07	-4.26	3.69	0.07
E2	TKW	3A	AX.89632174	5.50E+08	0.05	-2.34	3.54	0.06
E2	GY	3A	AX.89748881	5.29E+08	0.07	-4.26	3.69	0.07
E2	GY	3A	AX.89748881	5.29E+08	0.07	-4.26	3.69	0.07
E2	GY	3A	AX.89383778	7.36E+08	0.18	-2.46	3.07	0.05
E2	GY	3A	AX.89383778	7.36E+08	0.18	-2.46	3.07	0.05
E2	GY	3A	AX.89680429	5.63E+08	0.07	-4.26	3.69	0.07
E2	GY	3A	AX.89680429	5.63E+08	0.07	-4.26	3.69	0.07
E2	GY	3A	AX.89705720	6.96E+08	0.09	-3.35	3.07	0.04
E2	GY	3A	AX.89705720	6.96E+08	0.09	-3.35	3.07	0.04
E2	GY	3A	AX.89631354	5.16E+08	0.05	-4.80	3.69	0.08
E2	GY	3A	AX.89631354	5.16E+08	0.05	-4.80	3.69	0.08
E2	GY	3A	AX.89778275	5.58E+08	0.07	-4.26	3.69	0.07
E2	TKW	3A	AX.89596098	7.44E+08	0.35	-0.99	3.30	0.06
E2	TKW	3A	AX.89440804	4.80E+08	0.24	-1.22	3.89	0.07
E2	GY	3A	AX.89637285	5.68E+08	0.07	-4.26	3.69	0.07
E2	GY	3A	AX.89637285	5.68E+08	0.07	-4.26	3.69	0.07

E2	TKW	3A	AX.89571342	7.44E+08	0.30	-1.05	3.37	0.06
E2	GY	3A	AX.89576296	5.65E+08	0.07	-4.26	3.69	0.07
E2	GY	3A	AX.89576296	5.65E+08	0.07	-4.26	3.69	0.07
E2	GY	3A	AX.89561819	5.65E+08	0.07	-4.26	3.69	0.07
E2	GY	3A	AX.89354763	5.57E+08	0.07	-4.15	3.71	0.07
E2	GY	3A	AX.89688097	5.57E+08	0.06	-4.59	3.95	0.08
E2	GY	3A	AX.89688097	5.57E+08	0.06	-4.59	3.95	0.08
E2	GPM2	3A	AX.89693565	7.49E+08	0.08	-855.62	3.04	0.04
E2	GY	3A	AX.89654794	5.64E+08	0.07	-4.26	3.69	0.07
E2	GY	3A	AX.89707852	5.59E+08	0.07	-4.26	3.69	0.07
E2	TKW	3A	AX.89608966	5.34E+08	0.17	-1.20	3.02	0.05
E2	GY	3A	AX.89567996	5.33E+08	0.07	-4.04	3.76	0.08
E2	GY	3A	AX.89529194	5.30E+08	0.07	-4.26	3.69	0.07
E2	GPM2	3A	AX.89555917	7.50E+08	0.08	-880.17	3.04	0.04
E2	GY	3A	AX.89646606	5.57E+08	0.07	-4.26	3.69	0.07
E2	GY	3A	AX.89646606	5.57E+08	0.07	-4.26	3.69	0.07
E2	TKW	3A	AX.89382187	5.34E+08	0.17	-1.20	3.02	0.05
E2	TKW	3A	AX.89493328	5.34E+08	0.17	-1.20	3.02	0.05
E2	GPM2	3A	AX.89326956	5.68E+08	0.10	-811.26	3.06	0.06
E2	GY	3A	AX.89506121	5.66E+08	0.07	-4.26	3.69	0.07
E2	GY	3A	AX.89726130	5.63E+08	0.07	-4.26	3.69	0.07
E2	GPM2	3A	AX.89342351	7.50E+08	0.08	-880.17	3.04	0.04
E2	GY	3A	AX.89698099	5.30E+08	0.07	-4.26	3.69	0.07
E2	GY	3A	AX.89460171	5.58E+08	0.07	-4.26	3.69	0.07
E2	GY	3A	AX.89655270	5.65E+08	0.07	-4.26	3.69	0.07
E2	GPM2	3A	AX.89464588	7.47E+08	0.08	-880.17	3.04	0.04
E2	GY	3A	AX.89589319	5.59E+08	0.07	-4.26	3.69	0.07
E2	GY	3A	AX.89448842	5.01E+08	0.05	-4.65	3.24	0.07
E2	GY	3A	AX.89448842	5.01E+08	0.05	-4.65	3.24	0.07
E2	GY	3A	AX.89707382	5.17E+08	0.05	-4.53	3.32	0.07
E2	GY	3A	AX.89647632	5.57E+08	0.07	-4.26	3.69	0.07
E2	GY	3A	AX.89773033	5.62E+08	0.07	-4.26	3.69	0.07
E2	GY	3A	AX.89723994	5.33E+08	0.07	-4.04	3.76	0.08
E2	GY	3A	AX.89723994	5.33E+08	0.07	-4.04	3.76	0.08
E2	GY	3A	AX.89354763	5.57E+08	0.07	-4.15	3.71	0.07
E2	GPM2	3A	AX.89572110	7.47E+08	0.08	-880.17	3.04	0.04
E2	TKW	3A	AX.89664544	7.44E+08	0.37	-0.95	3.09	0.06
E2	GY	3A	AX.89390420	5.60E+08	0.07	-4.26	3.69	0.07
E2	GY	3A	AX.89554083	5.64E+08	0.07	-4.26	3.69	0.07
E2	TKW	3A	AX.89580115	4.50E+08	0.24	-1.15	3.48	0.06
E2	GPM2	3A	AX.89727512	5.68E+08	0.10	-811.26	3.06	0.06
E2	GY	3A	AX.89426725	5.56E+08	0.06	-4.59	3.95	0.08
E2	GY	3A	AX.89760649	5.30E+08	0.07	-4.15	3.71	0.07
E2	GPM2	3A	AX.89493850	7.50E+08	0.08	-880.17	3.04	0.04
E2	GY	3A	AX.89415611	5.65E+08	0.06	-4.38	3.63	0.07
E2	GY	3A	AX.89415611	5.65E+08	0.06	-4.38	3.63	0.07
E2	TKW	3A	AX.89374984	7.44E+08	0.26	-1.03	3.07	0.05
E2	GY	3A	AX.89578504	5.57E+08	0.07	-4.03	3.71	0.07
E2	GY	3A	AX.89688752	5.56E+08	0.06	-4.59	3.95	0.08
E2	GY	3A	AX.89461796	5.57E+08	0.05	-5.57	4.47	0.09

E2	GY	3A	AX.89461796	5.57E+08	0.05	-5.57	4.47	0.09
E2	GY	3A	AX.89514441	5.56E+08	0.10	-3.36	3.49	0.07
E2	TKW	3A	AX.89555948	7.44E+08	0.30	-1.05	3.37	0.06
E2	GY	3A	AX.89541040	5.16E+08	0.05	-4.80	3.69	0.08
E2	GPM2	3A	AX.89601433	7.49E+08	0.08	-855.62	3.04	0.04
E2	GY	3A	AX.89604406	5.31E+08	0.07	-4.26	3.69	0.07
E2	GY	3A	AX.89623669	5.60E+08	0.07	-4.26	3.69	0.07
E2	GY	3A	AX.89564685	5.17E+08	0.05	-5.07	3.72	0.07
E2	GY	3A	AX.89564685	5.17E+08	0.05	-5.07	3.72	0.07
E2	GPM2	3A	AX.89516976	7.48E+08	0.08	-880.17	3.04	0.04
E2	GY	3A	AX.89498962	2.02E+07	0.05	-5.09	3.77	0.08
E2	GY	3A	AX.89362303	5.56E+08	0.10	-3.36	3.49	0.07
E2	GY	3A	AX.89686328	5.29E+08	0.07	-4.26	3.69	0.07
E2	GPS	3A	AX.89623673	6.12E+08	0.09	-2.59	3.81	0.08
E2	GPM2	3A	AX.89701379	5.20E+08	0.05	-1218.05	3.42	0.07
E2	GY	3A	AX.89628912	5.65E+08	0.07	-4.26	3.69	0.07
E2	GY	3A	AX.89628912	5.65E+08	0.07	-4.26	3.69	0.07
E2	TKW	3A	AX.89575384	7.44E+08	0.26	-1.06	3.21	0.05
E2	GY	3A	AX.89707474	5.59E+08	0.07	-4.26	3.69	0.07
E2	GY	3A	AX.89499912	5.64E+08	0.07	-4.26	3.69	0.07
E2	GY	3A	AX.89499912	5.64E+08	0.07	-4.26	3.69	0.07
E2	GY	3A	AX.89617932	5.16E+08	0.05	-4.80	3.69	0.08
E2	GY	3A	AX.89679150	5.02E+08	0.07	-3.92	3.37	0.07
E2	GY	3A	AX.89679150	5.02E+08	0.07	-3.92	3.37	0.07
E2	TKW	3A	AX.89445760	5.32E+08	0.16	-1.25	3.11	0.05
E2	TKW	3A	AX.89346113	4.80E+08	0.22	-1.20	3.58	0.06
E2	GPM2	3A	AX.89427953	7.51E+08	0.08	-880.17	3.04	0.04
E2	TKW	3A	AX.89756173	5.32E+08	0.16	-1.25	3.11	0.05
E2	GY	3A	AX.89465602	5.60E+08	0.07	-4.26	3.69	0.07
E2	GPM2	3A	AX.89486709	7.47E+08	0.08	-880.17	3.04	0.04
E2	TKW	3A	AX.89337779	7.44E+08	0.35	-1.03	3.52	0.07
E2	GPM2	3A	AX.89723572	7.51E+08	0.08	-880.17	3.04	0.04
E2	GY	3A	AX.89681095	5.66E+08	0.07	-4.26	3.69	0.07
E2	GY	3A	AX.89681095	5.66E+08	0.07	-4.26	3.69	0.07
E2	GY	3A	AX.89397410	5.67E+08	0.07	-4.26	3.69	0.07
E2	GY	3A	AX.89397410	5.67E+08	0.07	-4.26	3.69	0.07
E2	GY	3A	AX.89592870	5.31E+08	0.07	-4.26	3.69	0.07
E2	GY	3A	AX.89560834	5.58E+08	0.07	-4.26	3.69	0.07
E2	GY	3A	AX.89560834	5.58E+08	0.07	-4.26	3.69	0.07
E2	TKW	3A	AX.89325426	4.80E+08	0.24	-1.22	3.89	0.07
E2	GY	3A	AX.89731030	5.65E+08	0.07	-4.26	3.69	0.07
E2	TKW	3A	AX.89310253	5.05E+08	0.30	1.04	3.31	0.06
E2	TKW	3A	AX.89566122	5.32E+08	0.16	-1.25	3.11	0.05
E2	GY	3A	AX.89362303	5.56E+08	0.10	-3.36	3.49	0.07
E2	GY	3A	AX.89370891	5.60E+08	0.07	-4.26	3.69	0.07
E2	GY	3A	AX.89370891	5.60E+08	0.07	-4.26	3.69	0.07
E2	GY	3A	AX.89375166	5.57E+08	0.06	-5.33	4.82	0.10
E2	GY	3A	AX.89542323	5.66E+08	0.07	-4.26	3.69	0.07
E2	GY	3A	AX.89707474	5.59E+08	0.07	-4.26	3.69	0.07
E2	GY	3A	AX.89528383	5.63E+08	0.07	-4.26	3.69	0.07

E2	GY	3A	AX.89751312	5.57E+08	0.08	-3.80	3.51	0.07
E2	GPM2	3A	AX.89423647	5.56E+08	0.10	-811.26	3.06	0.06
E2	GPM2	3A	AX.89568027	7.47E+08	0.08	-880.17	3.04	0.04
E2	GY	3A	AX.89707136	5.31E+08	0.07	-4.26	3.69	0.07
E2	GY	3A	AX.89483342	5.33E+08	0.07	-4.04	3.76	0.08
E2	GY	3A	AX.89529194	5.30E+08	0.07	-4.26	3.69	0.07
E2	TKW	3A	AX.89492626	7.44E+08	0.37	-1.06	3.80	0.07
E2	GY	3A	AX.89659061	5.32E+08	0.07	-4.26	3.69	0.07
E2	GY	3A	AX.89659061	5.32E+08	0.07	-4.26	3.69	0.07
E2	GY	3A	AX.89493587	5.57E+08	0.06	-4.59	3.95	0.08
E2	TKW	3A	AX.89748002	5.32E+08	0.16	-1.25	3.11	0.05
E2	GPM2	3A	AX.89364941	7.50E+08	0.08	-880.17	3.04	0.04
E2	TKW	3A	AX.89708628	7.44E+08	0.30	-1.01	3.17	0.05
E2	GY	3A	AX.89435715	5.30E+08	0.07	-4.26	3.69	0.07
E2	GY	3A	AX.89722203	5.64E+08	0.07	-4.26	3.69	0.07
E2	GPS	3A	AX.89402066	7.19E+08	0.22	-1.63	3.16	0.06
E2	GY	3A	AX.89365214	5.31E+08	0.07	-4.26	3.69	0.07
E2	GY	3A	AX.89623669	5.60E+08	0.07	-4.26	3.69	0.07
E2	GPM2	3A	AX.89633109	7.48E+08	0.08	-880.17	3.04	0.04
E2	GPM2	3A	AX.89500043	7.50E+08	0.08	-880.17	3.04	0.04
E2	GY	3A	AX.89592870	5.31E+08	0.07	-4.26	3.69	0.07
E2	GY	3A	AX.89734810	5.33E+08	0.07	-4.04	3.76	0.08
E2	GY	3A	AX.89370365	5.29E+08	0.07	-4.26	3.69	0.07
E2	GY	3A	AX.89437309	5.59E+08	0.07	-4.26	3.69	0.07
E2	GY	3A	AX.89437309	5.59E+08	0.07	-4.26	3.69	0.07
E2	GY	3A	AX.89585123	5.16E+08	0.05	-4.80	3.69	0.08
E2	GY	3A	AX.89613713	5.57E+08	0.06	-4.59	3.95	0.08
E2	GY	3A	AX.89460227	7.36E+08	0.18	-2.47	3.08	0.05
E2	GY	3A	AX.89460227	7.36E+08	0.18	-2.47	3.08	0.05
E2	GY	3A	AX.89675504	5.63E+08	0.07	-4.26	3.69	0.07
E2	GY	3A	AX.89712503	5.58E+08	0.07	-4.26	3.69	0.07
E2	GPM2	3A	AX.89738862	7.50E+08	0.08	-880.17	3.04	0.04
E2	GY	3A	AX.89427057	5.63E+08	0.07	-4.26	3.69	0.07
E2	GY	3A	AX.89614630	5.33E+08	0.07	-4.04	3.76	0.08
E2	GY	3A	AX.89614630	5.33E+08	0.07	-4.04	3.76	0.08
E2	TKW	3A	AX.89756096	5.32E+08	0.16	-1.25	3.11	0.05
E2	GPM2	3A	AX.89752607	7.49E+08	0.08	-880.17	3.04	0.04
E2	GY	3A	AX.89578504	5.57E+08	0.07	-4.03	3.71	0.07
E2	GY	3A	AX.89551367	5.33E+08	0.07	-4.04	3.76	0.08
E2	GY	3A	AX.89551367	5.33E+08	0.07	-4.04	3.76	0.08
E2	TKW	3A	AX.89748517	5.34E+08	0.13	-1.36	3.06	0.05
E2	GY	3A	AX.89465602	5.60E+08	0.07	-4.26	3.69	0.07
E2	GY	3A	AX.89766254	5.30E+08	0.07	-4.26	3.69	0.07
E2	GY	3A	AX.89766254	5.30E+08	0.07	-4.26	3.69	0.07
E2	GY	3A	AX.89514441	5.56E+08	0.10	-3.36	3.49	0.07
E2	GY	3A	AX.89583436	5.57E+08	0.06	-4.59	3.95	0.08
E2	TKW	3A	AX.89611849	4.80E+08	0.22	-1.20	3.58	0.06
E2	TKW	3A	AX.89440050	5.32E+08	0.15	-1.32	3.33	0.06
E2	GY	3A	AX.89487737	5.57E+08	0.07	-4.26	3.69	0.07
E2	GY	3A	AX.89487737	5.57E+08	0.07	-4.26	3.69	0.07

E2	GPM2	3A	AX.89624691	7.48E+08	0.08	-880.17	3.04	0.04
E2	TKW	3A	AX.89705426	5.34E+08	0.17	-1.20	3.02	0.05
E2	GY	3A	AX.89393225	5.33E+08	0.07	-4.04	3.76	0.08
E2	GPM2	3A	AX.89594743	5.56E+08	0.10	-811.26	3.06	0.06
E2	GY	3A	AX.89702635	5.33E+08	0.07	-4.04	3.76	0.08
E2	GPM2	3A	AX.89519250	7.50E+08	0.08	-880.17	3.04	0.04
E2	GPM2	3A	AX.89342565	7.47E+08	0.08	-880.17	3.04	0.04
E2	TKW	3A	AX.89561000	5.32E+08	0.16	-1.25	3.11	0.05
E2	GPM2	3A	AX.89652707	7.50E+08	0.08	-880.17	3.04	0.04
E2	GY	3A	AX.89678620	5.65E+08	0.07	-4.26	3.69	0.07
E2	TKW	3A	AX.89723710	5.32E+08	0.16	-1.25	3.11	0.05
E2	GY	3A	AX.89617932	5.16E+08	0.05	-4.80	3.69	0.08
E2	TKW	3A	AX.89315109	5.34E+08	0.17	-1.20	3.02	0.05
E2	GPM2	3A	AX.89683799	7.49E+08	0.08	-880.17	3.04	0.04
E2	TKW	3A	AX.89508575	4.44E+08	0.24	-1.17	3.64	0.06
E2	TKW	3A	AX.89636742	5.32E+08	0.16	-1.25	3.11	0.05
E2	GY	3A	AX.89490471	5.59E+08	0.07	-4.26	3.69	0.07
E2	GY	3A	AX.89638545	4.97E+08	0.06	-4.16	3.31	0.07
E2	TKW	3A	AX.89733534	4.80E+08	0.22	-1.20	3.58	0.06
E2	TKW	3A	AX.89757174	5.32E+08	0.16	-1.25	3.11	0.05
E2	TKW	3A	AX.89319918	5.34E+08	0.13	-1.36	3.06	0.05
E2	TKW	3A	AX.89622363	5.34E+08	0.17	-1.20	3.02	0.05
E2	GPM2	3A	AX.89625333	7.47E+08	0.08	-880.17	3.04	0.04
E2	GY	3A	AX.89677167	5.57E+08	0.07	-4.42	3.94	0.08
E2	GY	3A	AX.89685274	5.30E+08	0.07	-4.26	3.69	0.07
E2	GY	3A	AX.89742858	5.29E+08	0.07	-4.26	3.69	0.07
E2	GY	3A	AX.89742858	5.29E+08	0.07	-4.26	3.69	0.07
E2	TKW	3A	AX.89469040	7.44E+08	0.36	-1.00	3.35	0.06
E2	GY	3A	AX.89731030	5.65E+08	0.07	-4.26	3.69	0.07
E2	GPM2	3A	AX.89563396	7.49E+08	0.08	-855.62	3.04	0.04
E2	GY	3A	AX.89707136	5.31E+08	0.07	-4.26	3.69	0.07
E2	TKW	3A	AX.89750696	5.34E+08	0.17	-1.23	3.17	0.05
E2	TKW	3A	AX.89310434	5.32E+08	0.16	-1.25	3.11	0.05
E2	GPM2	3A	AX.89549417	7.47E+08	0.08	-880.17	3.04	0.04
E2	GPM2	3A	AX.89424933	7.48E+08	0.08	-880.17	3.04	0.04
E2	TKW	3A	AX.89455172	5.32E+08	0.16	-1.25	3.11	0.05
E2	GY	3A	AX.89750593	5.33E+08	0.07	-4.26	3.69	0.07
E2	TKW	3A	AX.89401394	5.34E+08	0.17	-1.20	3.02	0.05
E2	TKW	3A	AX.89364280	4.80E+08	0.22	-1.20	3.58	0.06
E2	GY	3A	AX.89483342	5.33E+08	0.07	-4.04	3.76	0.08
E2	GPM2	3A	AX.89341042	7.47E+08	0.08	-880.17	3.04	0.04
E2	GY	3A	AX.89699679	5.62E+08	0.07	-4.15	3.71	0.07
E2	GY	3A	AX.89699679	5.62E+08	0.07	-4.15	3.71	0.07
E2	TKW	3A	AX.89385976	5.32E+08	0.16	-1.25	3.11	0.05
E2	GY	3A	AX.89722203	5.64E+08	0.07	-4.26	3.69	0.07
E2	TKW	3A	AX.89659664	5.34E+08	0.17	-1.20	3.02	0.05
E2	TKW	3A	AX.89587656	7.44E+08	0.30	-1.01	3.17	0.05
E2	TKW	3A	AX.89777932	5.34E+08	0.13	-1.33	3.03	0.05
E2	GPM2	3A	AX.89394430	5.56E+08	0.10	-811.26	3.06	0.06
E2	TKW	3A	AX.89572200	4.80E+08	0.24	-1.25	4.04	0.07

E2	TKW	3A	AX.89441206	4.80E+08	0.24	-1.22	3.89	0.07
E2	TKW	3A	AX.89683357	7.44E+08	0.30	-1.05	3.37	0.06
E2	GY	3A	AX.89690071	5.66E+08	0.07	-4.26	3.69	0.07
E2	TKW	3A	AX.89573974	5.34E+08	0.17	-1.20	3.02	0.05
E2	GPM2	3A	AX.89498962	2.02E+07	0.05	-1390.70	4.24	0.10
E2	TKW	3A	AX.89311054	4.80E+08	0.22	-1.20	3.58	0.06
E2	TKW	3A	AX.89447799	5.34E+08	0.17	-1.20	3.02	0.05
E2	TKW	3A	AX.89643254	5.34E+08	0.17	-1.20	3.02	0.05
E2	TKW	3A	AX.89702901	5.34E+08	0.17	-1.20	3.02	0.05
E2	GY	3A	AX.89675504	5.63E+08	0.07	-4.26	3.69	0.07
E2	TKW	3A	AX.89350488	5.62E+08	0.16	-1.21	3.02	0.05
E2	GPS	3A	AX.89766237	6.12E+08	0.09	-2.59	3.81	0.08
E2	TKW	3A	AX.89619570	7.44E+08	0.38	-1.11	4.11	0.08
E2	GY	3A	AX.89490471	5.59E+08	0.07	-4.26	3.69	0.07
E2	TKW	3A	AX.89765605	5.34E+08	0.17	-1.20	3.02	0.05
E2	GPM2	3A	AX.89311459	7.48E+08	0.08	-880.17	3.04	0.04
E2	TKW	3A	AX.89665785	4.80E+08	0.23	-1.19	3.68	0.06
E2	GY	3A	AX.89778201	5.30E+08	0.07	-4.26	3.69	0.07
E2	GY	3A	AX.89778201	5.30E+08	0.07	-4.26	3.69	0.07
E2	GY	3A	AX.89677167	5.57E+08	0.07	-4.42	3.94	0.08
E2	TKW	3A	AX.89658848	4.80E+08	0.25	-1.06	3.13	0.05
E2	TKW	3A	AX.89433907	4.80E+08	0.22	-1.20	3.58	0.06
E2	GPM2	3A	AX.89628619	7.49E+08	0.08	-855.62	3.04	0.04
E2	TKW	3A	AX.89344911	4.80E+08	0.22	-1.20	3.58	0.06
E2	TKW	3A	AX.89668982	5.32E+08	0.17	-1.24	3.18	0.05
E2	GY	3A	AX.89774966	5.33E+08	0.07	-4.04	3.76	0.08
E2	TKW	3A	AX.89686826	5.34E+08	0.13	-1.42	3.28	0.05
E2	TKW	3A	AX.89375669	4.80E+08	0.24	-1.22	3.89	0.07
E2	TKW	3A	AX.89443841	7.44E+08	0.30	-1.05	3.37	0.06
E2	TKW	3A	AX.89380739	4.80E+08	0.22	-1.20	3.58	0.06
E2	GY	3A	AX.89678620	5.65E+08	0.07	-4.26	3.69	0.07
E2	TKW	3A	AX.89377201	5.34E+08	0.17	-1.20	3.02	0.05
E2	TKW	3A	AX.89669017	5.34E+08	0.17	-1.20	3.02	0.05
E2	GPM2	3A	AX.89617137	7.49E+08	0.08	-855.62	3.04	0.04
E2	TKW	3A	AX.89727232	5.34E+08	0.17	-1.20	3.02	0.05
E2	GY	3A	AX.89750593	5.33E+08	0.07	-4.26	3.69	0.07
E2	GPM2	3A	AX.89603026	5.57E+08	0.05	-1346.35	4.07	0.08
E2	GPM2	3A	AX.89583574	7.49E+08	0.08	-880.17	3.04	0.04
E2	GPM2	3A	AX.89766704	7.50E+08	0.08	-880.17	3.04	0.04
E2	TKW	3A	AX.89556300	5.34E+08	0.13	-1.36	3.06	0.05
E2	GY	3A	AX.89685274	5.30E+08	0.07	-4.26	3.69	0.07
E2	GY	3A	AX.89774966	5.33E+08	0.07	-4.04	3.76	0.08
E2	GPM2	3A	AX.89435419	7.50E+08	0.08	-880.17	3.04	0.04
E2	TKW	3A	AX.89494558	7.44E+08	0.36	-0.95	3.07	0.06
E2	TKW	3A	AX.89533025	5.32E+08	0.16	-1.25	3.11	0.05
E2	GPM2	3A	AX.89461796	5.57E+08	0.05	-1346.35	4.07	0.08
E3	GPM2	3A	AX.89529363	1.92E+06	0.38	902.95	3.24	0.01
E3	GPM2	3A	AX.89550801	1.92E+06	0.41	851.23	3.03	0.00
E3	GPM2	3A	AX.89325461	1.93E+06	0.38	914.91	3.25	0.00
E3	GPM2	3A	AX.89715408	6.96E+08	0.28	942.41	3.06	0.02

E3	GPM2	3A	AX.89418322	6.96E+08	0.28	958.95	3.16	0.03
E3	GPM2	3A	AX.89569615	6.96E+08	0.29	941.33	3.10	0.03
E3	GPM2	3A	AX.89374497	7.38E+08	0.32	942.13	3.14	0.11
E3	GPS	3A	AX.89605667	6.03E+08	0.07	-3.78	3.01	0.04
E3	GY	3A	AX.89622070	6.11E+08	0.09	5.09	3.37	0.06
E3	GY	3A	AX.89638485	6.16E+08	0.10	4.75	3.25	0.06
E3	GY	3A	AX.89525214	6.29E+08	0.27	3.25	3.21	0.03
E3	GY	3A	AX.89647039	6.96E+08	0.28	3.32	3.22	0.03
E3	GY	3A	AX.89717934	6.96E+08	0.28	3.24	3.17	0.03
E3	GY	3A	AX.89755163	6.96E+08	0.27	3.37	3.41	0.03
E3	GY	3A	AX.89551584	6.96E+08	0.34	3.08	3.05	0.03
E3	GY	3A	AX.89428758	6.96E+08	0.34	3.08	3.05	0.03
E3	GY	3A	AX.89324452	6.96E+08	0.26	3.23	3.12	0.03
E3	GY	3A	AX.89379464	6.96E+08	0.29	3.36	3.43	0.04
E3	GY	3A	AX.89739351	6.96E+08	0.27	3.37	3.38	0.03
E3	GY	3A	AX.89619067	6.96E+08	0.27	3.37	3.38	0.03
E3	GY	3A	AX.89723205	6.96E+08	0.34	3.08	3.05	0.03
E3	GY	3A	AX.89684284	6.96E+08	0.28	3.52	3.69	0.03
E3	GY	3A	AX.89715408	6.96E+08	0.28	3.53	3.74	0.03
E3	GY	3A	AX.89353034	6.96E+08	0.28	3.44	3.58	0.03
E3	GY	3A	AX.89351723	6.96E+08	0.27	3.29	3.27	0.03
E3	GY	3A	AX.89365774	6.96E+08	0.28	3.25	3.33	0.03
E3	GY	3A	AX.89418322	6.96E+08	0.28	3.18	3.12	0.02
E3	GY	3A	AX.89569615	6.96E+08	0.29	3.09	3.01	0.02
E3	GY	3A	AX.89557559	6.97E+08	0.08	5.35	3.16	0.02
E3	GY	3A	AX.89552270	6.97E+08	0.08	5.28	3.03	0.02
E3	SPM2	3A	AX.89776298	4.67E+07	0.20	28.07	3.86	0.07
E3	SPM2	3A	AX.89456601	4.67E+07	0.19	24.92	3.09	0.07
E3	TKW	3A	AX.89506498	1.50E+06	0.46	1.01	3.86	0.01
E3	TKW	3A	AX.89516762	1.62E+06	0.44	1.04	3.98	0.01
E3	TKW	3A	AX.89334786	1.77E+06	0.47	1.07	4.25	0.01
E3	TKW	3A	AX.89685817	1.77E+06	0.43	0.96	3.47	0.01
E3	TKW	3A	AX.89405859	1.77E+06	0.46	1.10	4.44	0.01
E3	TKW	3A	AX.89680505	1.77E+06	0.45	0.96	3.51	0.01
E3	TKW	3A	AX.89767528	1.78E+06	0.46	1.04	3.99	0.01
E3	TKW	3A	AX.89504786	1.79E+06	0.44	1.04	4.04	0.01
E3	TKW	3A	AX.89656802	1.79E+06	0.43	1.01	3.86	0.01
E3	TKW	3A	AX.89569283	1.80E+06	0.44	1.07	4.20	0.01
E3	TKW	3A	AX.89321511	1.62E+06	0.44	1.03	3.98	0.01
E3	TKW	3A	AX.89434751	1.72E+06	0.44	1.07	4.20	0.01
E3	TKW	3A	AX.89700949	1.72E+06	0.44	1.07	4.20	0.01
E3	TKW	3A	AX.89549616	1.73E+06	0.44	1.07	4.20	0.01
E3	TKW	3A	AX.89462585	1.77E+06	0.44	1.07	4.20	0.01
E3	TKW	3A	AX.89705177	6.54E+08	0.22	1.02	3.12	0.04
E3	TKW	3A	AX.89501481	6.54E+08	0.22	1.02	3.12	0.04
E3	TKW	3A	AX.89360854	1.80E+06	0.44	1.07	4.20	0.01
E3	TKW	3A	AX.89418185	1.82E+06	0.35	0.94	3.45	0.01
E3	TKW	3A	AX.89558129	1.89E+06	0.44	1.10	4.37	0.01
E3	TKW	3A	AX.89772408	1.89E+06	0.44	1.08	4.30	0.01
E3	TKW	3A	AX.89621737	1.90E+06	0.45	1.10	4.42	0.01

E3	TKW	3A	AX.89529363	1.92E+06	0.38	-1.23	5.62	0.02
E3	TKW	3A	AX.89550801	1.92E+06	0.41	-1.03	4.25	0.01
E3	TKW	3A	AX.89685524	1.93E+06	0.49	1.08	4.24	0.01
E3	TKW	3A	AX.89325461	1.93E+06	0.38	-1.18	5.12	0.01
E3	TKW	3A	AX.89649744	1.93E+06	0.48	1.10	4.36	0.01
E3	TKW	3A	AX.89601427	6.45E+06	0.28	0.94	3.04	0.00
E3	TKW	3A	AX.89769205	6.90E+06	0.27	0.96	3.01	0.00
E3	TKW	3A	AX.89341270	6.90E+06	0.27	0.96	3.01	0.00
E3	TKW	3A	X.89626769_OT	1.25E+07	0.15	-1.20	3.37	0.04
E3	TKW	3A	AX.89471146	1.25E+07	0.13	-1.23	3.23	0.04
E3	TKW	3A	AX.89769379	6.54E+08	0.21	1.00	3.04	0.03
E3	TKW	3A	AX.89724432	6.54E+08	0.20	1.05	3.22	0.03
E3	TKW	3A	AX.89382845	6.54E+08	0.21	1.00	3.03	0.03
E3	TKW	3A	X.89769143_OT	4.41E+05	0.26	0.97	3.32	0.00
E3	TKW	3A	X.89668298_OT	5.41E+05	0.34	0.95	3.49	0.00
E3	TKW	3A	X.89714989_OT	6.80E+05	0.27	0.96	3.10	0.00
E4	GPS	3A	AX.89718553	1.57E+07	0.30	1.63	3.16	0.06
E4	GPS	3A	AX.89469818	1.57E+07	0.30	1.66	3.28	0.06
E4	GPS	3A	AX.89323020	2.25E+07	0.38	-1.51	3.08	0.07
E4	GPS	3A	AX.89717553	6.42E+08	0.20	2.02	3.41	0.08
E4	GPS	3A	AX.89717642	6.42E+08	0.20	2.02	3.41	0.08
E4	GPS	3A	AX.89369930	6.42E+08	0.20	2.02	3.41	0.08
E4	GPM2	3A	AX.89506498	1.50E+06	0.46	-464.48	3.10	0.01
E4	GPM2	3A	AX.89357239	1.52E+06	0.38	-442.70	3.03	0.01
E4	GPM2	3A	AX.89529363	1.92E+06	0.38	476.28	3.32	0.01
E4	GPM2	3A	AX.89733345	2.45E+07	0.07	856.00	3.39	0.06
E4	GPM2	3A	AX.89744434	1.34E+08	0.06	921.43	3.02	0.02
E4	GPM2	3A	AX.89679638	1.34E+08	0.06	921.43	3.02	0.02
E4	GPM2	3A	AX.89459928	1.35E+08	0.06	921.43	3.02	0.02
E4	GPS	3A	AX.89678665	2.25E+07	0.38	-1.51	3.08	0.07
E4	GPS	3A	AX.89457595	2.25E+07	0.39	-1.57	3.33	0.07
E4	GPS	3A	AX.89742220	2.25E+07	0.35	-1.56	3.17	0.07
E4	GPM2	3A	AX.89598114	6.58E+08	0.08	-895.58	3.95	0.06
E4	GPM2	3A	AX.89639531	6.58E+08	0.07	-875.88	3.26	0.05
E4	GPM2	3A	AX.89747164	1.36E+08	0.06	921.43	3.02	0.02
E4	GPM2	3A	AX.89434162	1.36E+08	0.06	921.43	3.02	0.02
E4	GPM2	3A	AX.89771465	1.36E+08	0.06	921.43	3.02	0.02
E4	GPM2	3A	AX.89717877	1.36E+08	0.06	921.43	3.02	0.02
E4	GPM2	3A	AX.89366281	1.36E+08	0.06	921.43	3.02	0.02
E4	GPM2	3A	AX.89743592	1.36E+08	0.06	891.96	3.00	0.02
E4	GPM2	3A	AX.89748917	1.36E+08	0.06	921.43	3.02	0.02
E4	GPM2	3A	AX.89512318	1.36E+08	0.06	891.96	3.00	0.02
E4	GPM2	3A	AX.89646454	1.38E+08	0.06	921.43	3.02	0.02
E4	GPM2	3A	AX.89317972	1.38E+08	0.06	921.43	3.02	0.02
E4	GPM2	3A	AX.89509387	1.40E+08	0.05	981.94	3.18	0.03
E4	GPM2	3A	AX.89672074	1.40E+08	0.06	891.96	3.00	0.02
E4	GPM2	3A	AX.89584929	1.41E+08	0.05	981.94	3.18	0.03
E4	GPM2	3A	AX.89720290	1.42E+08	0.05	981.94	3.18	0.03
E4	GPM2	3A	AX.89440007	1.42E+08	0.05	981.94	3.18	0.03
E4	GPM2	3A	AX.89734062	1.43E+08	0.06	921.43	3.02	0.02

E4	GPM2	3A	X.89693520_OT	6.57E+08	0.07	-898.80	3.52	0.06
E4	GPM2	3A	AX.89580465	6.58E+08	0.10	-818.80	4.24	0.06
E4	GPM2	3A	AX.89597120	6.58E+08	0.11	-826.70	4.41	0.07
E4	GPM2	3A	AX.89412937	6.58E+08	0.07	-919.26	3.67	0.06
E4	GPM2	3A	AX.89573930	6.58E+08	0.08	-816.44	3.59	0.06
E4	GY	3A	AX.89706784	6.93E+07	0.10	2.44	3.04	0.04
E4	GY	3A	AX.89599548	6.93E+07	0.10	2.44	3.04	0.04
E4	GY	3A	AX.89375712	7.23E+07	0.09	2.91	3.96	0.06
E4	GY	3A	AX.89498050	6.54E+07	0.08	2.75	3.31	0.05
E4	GY	3A	AX.89687145	6.62E+07	0.09	2.47	3.00	0.04
E4	GY	3A	AX.89451561	6.73E+07	0.09	2.91	3.77	0.05
E4	GY	3A	AX.89382999	6.74E+07	0.08	2.80	3.42	0.05
E4	GY	3A	AX.89744495	6.74E+07	0.09	2.53	3.05	0.04
E4	GY	3A	AX.89426500	7.23E+07	0.09	2.91	3.96	0.06
E4	GY	3A	AX.89669526	7.35E+07	0.09	2.70	3.34	0.05
E4	GY	3A	AX.89317931	7.40E+07	0.09	2.70	3.34	0.05
E4	GY	3A	X.89430743_OT	7.46E+07	0.16	-2.10	3.40	0.05
E4	GY	3A	AX.89747308	6.93E+07	0.10	2.44	3.04	0.04
E4	GY	3A	AX.89380711	6.95E+07	0.10	2.44	3.04	0.04
E4	GY	3A	AX.89742909	6.95E+07	0.10	2.44	3.04	0.04
E4	GY	3A	AX.89325624	6.95E+07	0.10	2.44	3.04	0.04
E4	GY	3A	AX.89441160	6.96E+07	0.09	2.74	3.50	0.05
E4	GY	3A	AX.89406939	7.07E+07	0.08	2.96	3.78	0.05
E4	GY	3A	AX.89678198	7.18E+07	0.09	2.76	3.53	0.05
E4	GY	3A	AX.89464147	7.18E+07	0.09	2.91	3.96	0.06
E4	SPM2	3A	AX.89315209	1.24E+07	0.28	17.86	3.09	0.05
E4	SPM2	3A	AX.89718553	1.57E+07	0.30	-19.41	3.62	0.07
E4	SPM2	3A	X.89652010_OT	9.47E+06	0.16	22.27	3.37	0.04
E4	SPM2	3A	AX.89365659	1.17E+07	0.47	16.80	3.17	0.04
E4	SPM2	3A	AX.89450866	1.18E+07	0.47	17.16	3.28	0.04
E4	SPM2	3A	AX.89469818	1.57E+07	0.30	-18.98	3.49	0.06
E4	SPM2	3A	X.89692579_OT	7.11E+08	0.08	29.12	3.31	0.05
E4	SPM2	3A	X.89396933_OT	7.11E+08	0.09	28.03	3.13	0.07
E4	SPM2	3A	X.89440178_OT	7.11E+08	0.09	28.03	3.13	0.07
E4	SPM2	3A	X.89433732_OT	7.11E+08	0.09	28.03	3.13	0.07
E4	SPM2	3A	AX.89424818	7.26E+08	0.16	-23.20	3.45	0.06
E4	GY	3A	AX.89580465	6.58E+08	0.11	-2.48	3.58	0.05
E4	GY	3A	AX.89597120	6.58E+08	0.11	-2.57	3.90	0.06
E4	GY	3A	AX.89573930	6.58E+08	0.09	-2.50	3.12	0.05
E4	GY	3A	AX.89598114	6.58E+08	0.08	-2.68	3.32	0.05
E4	TKW	3A	AX.89529363	1.92E+06	0.38	-0.90	3.39	0.01
E4	TKW	3A	AX.89410293	6.33E+08	0.26	-0.96	3.24	0.00
E4	TKW	3A	AX.89444445	6.33E+08	0.26	-0.96	3.24	0.00
E4	TKW	3A	AX.89488313	6.33E+08	0.26	-0.96	3.24	0.00
E4	TKW	3A	AX.89486391	6.33E+08	0.26	-0.96	3.24	0.00
E4	TKW	3A	AX.89657477	6.33E+08	0.26	-0.96	3.24	0.00
E4	TKW	3A	AX.89770721	6.33E+08	0.27	-0.94	3.13	0.00
E4	TKW	3A	AX.89324391	6.33E+08	0.26	-0.96	3.24	0.00
E4	TKW	3A	AX.89361167	6.33E+08	0.26	-0.96	3.24	0.00
E4	TKW	3A	AX.89381089	6.33E+08	0.26	-0.96	3.27	0.00

E4	TKW	3A	AX.89569699	6.33E+08	0.26	-0.97	3.34	0.00
E4	TKW	3A	AX.89660874	6.34E+08	0.26	-0.93	3.10	0.00
E4	TKW	3A	AX.89535418	6.34E+08	0.27	-0.96	3.34	0.00
E4	TKW	3A	AX.89329608	6.34E+08	0.26	-0.97	3.34	0.00
E4	TKW	3A	AX.89597484	6.34E+08	0.26	-0.97	3.34	0.00
E4	TKW	3A	AX.89762611	6.34E+08	0.26	-0.97	3.34	0.00
E4	TKW	3A	AX.89556790	6.34E+08	0.26	-0.97	3.34	0.00
E4	TKW	3A	AX.89696155	6.34E+08	0.26	-0.97	3.34	0.00
E4	TKW	3A	AX.89697826	6.35E+08	0.26	-0.97	3.34	0.00
E1	GY	3B	AX.89585986	7.83E+08	0.15	-4.14	4.81	0.08
E1	GY	3B	AX.89675055	2.27E+07	0.20	-3.24	3.79	0.06
E1	SPM2	3B	AX.89403382	8.00E+08	0.40	-20.13	4.23	0.08
E1	GY	3B	AX.89676377	2.36E+08	0.08	4.32	3.20	0.05
E1	SPM2	3B	AX.89644519	8.00E+08	0.44	-18.45	3.73	0.06
E1	GY	3B	AX.89335703	1.03E+07	0.30	-2.72	3.57	0.06
E1	GY	3B	AX.89318080	1.71E+07	0.42	2.46	3.42	0.06
E1	GY	3B	AX.89376995	1.21E+08	0.09	3.91	3.06	0.05
E1	GY	3B	AX.89376995	1.21E+08	0.09	3.91	3.06	0.05
E1	SPM2	3B	AX.89480220	8.00E+08	0.40	-20.13	4.23	0.08
E1	GY	3B	AX.89386142	1.61E+07	0.26	2.63	3.11	0.04
E1	GY	3B	AX.89386142	1.61E+07	0.26	2.63	3.11	0.04
E1	GY	3B	AX.89559462	7.06E+07	0.09	3.90	3.05	0.05
E1	GY	3B	AX.89524737	4.48E+08	0.11	3.81	3.37	0.06
E1	SPM2	3B	AX.89552994	8.00E+08	0.44	-18.45	3.73	0.06
E1	GY	3B	AX.89559462	7.06E+07	0.09	3.90	3.05	0.05
E1	GY	3B	AX.89449959	2.25E+08	0.08	4.32	3.20	0.05
E1	GY	3B	AX.89376983	2.03E+08	0.08	4.12	3.08	0.05
E1	SPM2	3B	AX.89634380	8.00E+08	0.40	-20.13	4.23	0.08
E1	GY	3B	AX.89764741	2.15E+08	0.07	4.99	3.71	0.06
E1	GY	3B	AX.89376983	2.03E+08	0.08	4.12	3.08	0.05
E1	SPM2	3B	AX.89715464	7.40E+08	0.40	-17.71	3.40	0.06
E1	SPM2	3B	AX.86168525	8.00E+08	0.40	-20.13	4.23	0.08
E1	SPM2	3B	AX.89767393	8.03E+08	0.39	-21.32	4.64	0.08
E1	SPM2	3B	AX.89647841	7.99E+08	0.44	-18.48	3.73	0.06
E1	GY	3B	AX.89396277	1.03E+07	0.29	-2.74	3.55	0.06
E1	GY	3B	AX.89524737	4.48E+08	0.11	3.81	3.37	0.06
E1	GY	3B	AX.89597884	1.61E+07	0.34	2.63	3.59	0.05
E1	GY	3B	AX.89386944	2.03E+08	0.07	4.53	3.31	0.06
E1	SPM2	3B	AX.89531838	7.99E+08	0.44	-18.45	3.73	0.06
E1	SPM2	3B	AX.89422216	8.01E+08	0.41	-20.67	4.47	0.08
E1	GY	3B	AX.89450012	7.81E+08	0.49	3.03	5.04	0.10
E1	SPM2	3B	AX.89717951	8.04E+08	0.35	-20.79	4.28	0.08
E1	GY	3B	AX.89450012	7.81E+08	0.49	3.03	5.04	0.10
E1	GY	3B	AX.89731729	1.59E+07	0.33	2.95	4.32	0.07
E1	GY	3B	AX.89669218	2.05E+07	0.26	-2.68	3.20	0.05
E1	GY	3B	AX.89600688	2.04E+08	0.07	4.53	3.31	0.06
E1	SPM2	3B	AX.89439005	7.99E+08	0.44	-18.48	3.73	0.06
E1	SPM2	3B	AX.89388184	8.01E+08	0.43	-19.35	4.02	0.07
E1	SPM2	3B	AX.89725141	8.03E+08	0.39	-21.00	4.53	0.08
E1	SPM2	3B	AX.89468697	7.99E+08	0.45	-19.21	4.00	0.07

E1	GY	3B	AX.89465739	7.82E+08	0.37	-2.74	3.98	0.07
E1	GY	3B	AX.89312853	3.06E+08	0.09	4.00	3.06	0.05
E1	SPM2	3B	AX.89736860	8.03E+08	0.39	-21.00	4.53	0.08
E1	SPM2	3B	AX.89735403	7.99E+08	0.44	-18.48	3.73	0.06
E1	SPM2	3B	AX.89488780	8.04E+08	0.43	-19.78	4.18	0.07
E1	GY	3B	AX.89475327	1.06E+07	0.46	2.37	3.28	0.05
E1	SPM2	3B	AX.89390128	8.00E+08	0.40	-20.13	4.23	0.08
E1	SPM2	3B	AX.89741308	7.99E+08	0.44	-18.48	3.73	0.06
E1	GY	3B	AX.89593712	7.81E+08	0.46	3.18	5.46	0.11
E1	GY	3B	AX.89460074	5.56E+08	0.23	-2.73	3.10	0.05
E1	GY	3B	AX.89735575	7.93E+07	0.09	3.90	3.05	0.05
E1	SPM2	3B	AX.89644568	7.99E+08	0.40	-20.13	4.23	0.08
E1	SPM2	3B	AX.89661483	8.04E+08	0.38	-21.19	4.57	0.08
E1	SPM2	3B	AX.89775761	8.00E+08	0.40	-20.13	4.23	0.08
E1	SPM2	3B	AX.89692155	8.03E+08	0.39	-21.00	4.53	0.08
E1	SPM2	3B	AX.89605900	7.99E+08	0.40	-20.13	4.23	0.08
E1	GY	3B	AX.89318080	1.71E+07	0.42	2.46	3.42	0.06
E1	GY	3B	AX.89447307	9.06E+06	0.36	2.89	4.34	0.07
E1	SPM2	3B	AX.89549816	8.01E+08	0.45	-18.02	3.59	0.06
E1	SPM2	3B	AX.89775805	8.03E+08	0.39	-21.08	4.55	0.08
E1	SPM2	3B	AX.89331809	4.04E+07	0.31	17.79	3.08	0.05
E1	SPM2	3B	AX.89421080	7.99E+08	0.39	-20.20	4.24	0.08
E1	SPM2	3B	AX.89427569	8.01E+08	0.40	-20.13	4.23	0.08
E1	SPM2	3B	AX.89681718	8.03E+08	0.39	-21.00	4.53	0.08
E1	SPM2	3B	AX.89501826	8.03E+08	0.39	-21.00	4.53	0.08
E1	SPM2	3B	AX.89442641	8.03E+08	0.43	-19.35	4.02	0.07
E1	SPM2	3B	AX.89332425	8.03E+08	0.43	-19.35	4.02	0.07
E1	GY	3B	AX.89520965	7.84E+08	0.46	-2.75	4.21	0.07
E1	SPM2	3B	AX.89573638	7.99E+08	0.40	-20.13	4.23	0.08
E1	SPM2	3B	AX.86167776	8.03E+08	0.43	-20.11	4.31	0.08
E1	GY	3B	AX.89333525	5.45E+08	0.27	-2.61	3.13	0.06
E1	GY	3B	AX.89459497	2.33E+07	0.21	-3.19	3.85	0.06
E1	GY	3B	AX.89447307	9.06E+06	0.36	2.89	4.34	0.07
E1	SPM2	3B	AX.89532013	8.00E+08	0.39	-20.20	4.24	0.08
E1	GY	3B	AX.89520965	7.84E+08	0.46	-2.75	4.21	0.07
E1	SPM2	3B	AX.89539699	2.29E+07	0.20	22.78	3.62	0.06
E1	GY	3B	AX.89316012	7.82E+08	0.38	-2.70	3.91	0.07
E1	GY	3B	AX.89377431	7.83E+08	0.08	-4.26	3.26	0.05
E1	SPM2	3B	AX.86168523	8.00E+08	0.44	-18.48	3.73	0.06
E1	GY	3B	AX.86168705	1.52E+07	0.33	2.70	3.72	0.06
E1	SPM2	3B	AX.89419189	8.04E+08	0.35	-21.53	4.57	0.08
E1	GY	3B	AX.89383104	7.82E+08	0.48	2.46	3.49	0.07
E1	GY	3B	AX.89412494	5.51E+08	0.09	-5.01	4.52	0.07
E1	GY	3B	AX.89634987	5.87E+08	0.30	-2.69	3.54	0.07
E1	SPM2	3B	AX.89504457	7.99E+08	0.40	-20.13	4.23	0.08
E1	GY	3B	AX.89483101	2.34E+07	0.21	-3.19	3.85	0.06
E1	GY	3B	AX.89380994	7.18E+07	0.09	3.90	3.05	0.05
E1	SPM2	3B	AX.89512704	8.00E+08	0.39	-20.20	4.24	0.08
E1	SPM2	3B	AX.89381297	8.00E+08	0.44	-18.45	3.73	0.06
E1	GY	3B	AX.89312632	4.05E+07	0.10	3.95	3.35	0.06

E1	SPM2	3B	AX.89519904	8.00E+08	0.40	-20.13	4.23	0.08
E1	SPM2	3B	AX.89563480	8.00E+08	0.44	-18.45	3.73	0.06
E1	GY	3B	AX.89317109	7.01E+07	0.09	3.90	3.05	0.05
E1	GY	3B	AX.89497934	4.48E+08	0.12	3.75	3.37	0.06
E1	SPM2	3B	AX.89749034	4.02E+07	0.31	17.79	3.08	0.05
E1	SPM2	3B	AX.89521658	8.04E+08	0.38	-19.61	3.99	0.07
E1	SPM2	3B	AX.89436691	8.03E+08	0.39	-21.32	4.64	0.08
E1	GY	3B	AX.89313026	4.47E+08	0.12	3.52	3.11	0.05
E1	GY	3B	AX.89561688	4.47E+08	0.12	3.52	3.11	0.05
E1	GY	3B	AX.89388524	1.03E+07	0.30	-2.72	3.57	0.06
E1	SPM2	3B	AX.86167774	8.03E+08	0.42	-19.39	4.02	0.07
E1	SPM2	3B	AX.89513284	7.99E+08	0.40	-19.41	3.98	0.07
E1	SPM2	3B	AX.89562717	8.01E+08	0.40	-20.88	4.52	0.08
E1	SPM2	3B	AX.89341812	2.25E+07	0.48	17.72	3.50	0.07
E1	SPM2	3B	AX.89734728	8.01E+08	0.40	-20.13	4.23	0.08
E1	GY	3B	AX.89760382	1.25E+08	0.10	4.02	3.34	0.06
E1	SPM2	3B	AX.89392017	4.03E+07	0.31	17.79	3.08	0.05
E1	SPM2	3B	AX.89453189	7.99E+08	0.40	-20.13	4.23	0.08
E1	GY	3B	AX.89764741	2.15E+08	0.07	4.99	3.71	0.06
E1	GY	3B	AX.89500234	4.52E+08	0.12	3.56	3.08	0.05
E1	GY	3B	AX.89500234	4.52E+08	0.12	3.56	3.08	0.05
E1	SPM2	3B	AX.89622338	8.01E+08	0.44	-19.24	4.01	0.07
E1	SPM2	3B	AX.89651127	8.00E+08	0.44	-18.48	3.73	0.06
E1	SPM2	3B	AX.89365302	8.01E+08	0.43	-19.40	4.04	0.07
E1	GY	3B	AX.89449959	2.25E+08	0.08	4.32	3.20	0.05
E1	GY	3B	AX.89593554	7.01E+07	0.09	3.90	3.05	0.05
E1	GY	3B	AX.89371943	4.99E+08	0.43	-2.38	3.26	0.05
E1	GY	3B	AX.89554686	4.52E+08	0.12	3.56	3.08	0.05
E1	SPM2	3B	AX.89338600	7.99E+08	0.39	-20.20	4.24	0.08
E1	SPM2	3B	AX.89543388	8.04E+08	0.38	-21.19	4.57	0.08
E1	SPM2	3B	AX.89700247	8.04E+08	0.38	-21.19	4.57	0.08
E1	GY	3B	AX.89314692	2.46E+07	0.28	-2.64	3.28	0.05
E1	SPM2	3B	AX.89451096	7.99E+08	0.40	-20.13	4.23	0.08
E1	GY	3B	AX.89348709	4.08E+07	0.11	3.64	3.00	0.05
E1	GY	3B	AX.89701327	7.82E+08	0.36	-2.58	3.55	0.06
E1	GY	3B	AX.89775197	7.83E+08	0.48	2.46	3.49	0.07
E1	SPM2	3B	AX.89586004	8.04E+08	0.38	-20.34	4.25	0.08
E1	SPM2	3B	AX.89643156	5.81E+08	0.44	-18.45	3.73	0.06
E1	GY	3B	AX.89311864	4.49E+08	0.12	3.52	3.11	0.05
E1	SPM2	3B	AX.89503564	8.03E+08	0.39	-21.08	4.55	0.08
E1	SPM2	3B	AX.89404122	8.03E+08	0.39	-21.00	4.53	0.08
E1	SPM2	3B	AX.89456160	8.00E+08	0.40	-20.84	4.49	0.08
E1	SPM2	3B	AX.89583092	8.03E+08	0.43	-19.35	4.02	0.07
E1	GY	3B	AX.89669218	2.05E+07	0.26	-2.68	3.20	0.05
E1	GY	3B	AX.89366224	1.10E+07	0.43	-2.35	3.18	0.05
E1	GY	3B	AX.89312632	4.05E+07	0.10	3.95	3.35	0.06
E1	SPM2	3B	AX.89498579	8.01E+08	0.44	-18.45	3.73	0.06
E1	GY	3B	AX.89328273	7.81E+08	0.46	3.18	5.46	0.11
E1	GY	3B	AX.89485267	2.68E+08	0.10	3.81	3.15	0.05
E1	GY	3B	AX.89394006	2.40E+08	0.08	4.17	3.14	0.05

E1	GY	3B	AX.89394006	2.40E+08	0.08	4.17	3.14	0.05
E1	GY	3B	AX.89597884	1.61E+07	0.34	2.63	3.59	0.05
E1	GY	3B	AX.89398767	7.04E+07	0.09	3.90	3.05	0.05
E1	SPM2	3B	AX.89579562	8.00E+08	0.40	-20.13	4.23	0.08
E1	GY	3B	AX.89707408	4.98E+08	0.44	-2.27	3.03	0.05
E1	GY	3B	AX.89528591	4.09E+07	0.11	3.64	3.00	0.05
E1	GY	3B	AX.89562240	7.08E+07	0.09	3.90	3.05	0.05
E1	SPM2	3B	AX.89620763	8.03E+08	0.39	-21.00	4.53	0.08
E1	GY	3B	AX.89732442	7.83E+08	0.14	-3.84	4.01	0.06
E1	GY	3B	AX.89630147	7.06E+07	0.09	3.90	3.05	0.05
E1	SPM2	3B	AX.89423755	8.04E+08	0.39	-20.16	4.20	0.07
E1	SPM2	3B	AX.89579114	8.04E+08	0.35	-20.79	4.28	0.08
E1	SPM2	3B	AX.89695576	8.00E+08	0.45	-19.22	4.02	0.07
E1	GY	3B	AX.89329336	7.03E+07	0.09	3.90	3.05	0.05
E1	GY	3B	AX.89329336	7.03E+07	0.09	3.90	3.05	0.05
E1	SPM2	3B	AX.89534288	8.04E+08	0.39	-20.16	4.20	0.07
E1	SPM2	3B	AX.89617569	8.00E+08	0.40	-20.13	4.23	0.08
E1	GY	3B	AX.89383104	7.82E+08	0.48	2.46	3.49	0.07
E1	SPM2	3B	AX.89600175	4.02E+07	0.31	17.79	3.08	0.05
E1	SPM2	3B	AX.89473605	8.03E+08	0.43	-19.35	4.02	0.07
E1	GY	3B	AX.89391940	3.76E+08	0.11	3.81	3.25	0.06
E1	SPM2	3B	AX.89385020	7.99E+08	0.40	-20.13	4.23	0.08
E1	SPM2	3B	AX.89492813	7.99E+08	0.44	-18.45	3.73	0.06
E1	SPM2	3B	AX.89340548	4.04E+07	0.31	17.79	3.08	0.05
E1	SPM2	3B	AX.89361784	4.03E+07	0.31	17.79	3.08	0.05
E1	GY	3B	AX.89330691	2.33E+08	0.08	4.32	3.20	0.05
E1	SPM2	3B	AX.89659920	8.03E+08	0.39	-21.00	4.53	0.08
E1	SPM2	3B	AX.89552911	7.40E+08	0.40	-18.63	3.72	0.07
E1	GY	3B	AX.89388524	1.03E+07	0.30	-2.72	3.57	0.06
E1	SPM2	3B	AX.89649431	4.05E+07	0.27	18.45	3.11	0.05
E1	SPM2	3B	AX.89547794	8.04E+08	0.35	-21.64	4.61	0.08
E1	GY	3B	AX.89560707	7.01E+07	0.09	3.90	3.05	0.05
E1	GY	3B	AX.89560707	7.01E+07	0.09	3.90	3.05	0.05
E1	SPM2	3B	AX.89529832	8.04E+08	0.38	-20.12	4.17	0.07
E1	GY	3B	AX.89708796	4.48E+08	0.12	3.75	3.37	0.06
E1	GY	3B	AX.89331170	7.82E+08	0.37	-2.92	4.44	0.08
E1	GY	3B	AX.89475327	1.06E+07	0.46	2.37	3.28	0.05
E1	SPM2	3B	AX.89700345	7.99E+08	0.46	-18.00	3.59	0.06
E1	SPM2	3B	AX.89708631	8.00E+08	0.44	-18.45	3.73	0.06
E1	SPM2	3B	AX.89458984	8.00E+08	0.45	-18.63	3.80	0.07
E1	SPM2	3B	AX.89337006	8.03E+08	0.39	-21.00	4.53	0.08
E1	GY	3B	AX.89674421	7.82E+08	0.46	3.05	5.07	0.10
E1	GY	3B	AX.89459497	2.33E+07	0.21	-3.19	3.85	0.06
E1	SPM2	3B	AX.89662577	8.04E+08	0.35	-20.79	4.28	0.08
E1	GY	3B	AX.89455859	2.29E+08	0.08	4.32	3.20	0.05
E1	GY	3B	AX.89456017	4.08E+07	0.11	3.64	3.00	0.05
E1	GY	3B	AX.89402447	5.02E+08	0.39	2.33	3.04	0.06
E1	GY	3B	AX.89658098	6.85E+07	0.09	3.90	3.05	0.05
E1	SPM2	3B	AX.89504913	8.03E+08	0.40	-21.68	4.81	0.09
E1	SPM2	3B	AX.89345601	7.99E+08	0.42	-18.83	3.83	0.07

E1	SPM2	3B	AX.89345504	8.01E+08	0.44	-18.45	3.73	0.06
E1	GY	3B	AX.89460074	5.56E+08	0.23	-2.73	3.10	0.05
E1	SPM2	3B	AX.89475210	8.03E+08	0.39	-21.08	4.55	0.08
E1	GY	3B	AX.89760382	1.25E+08	0.10	4.02	3.34	0.06
E1	GY	3B	AX.89528591	4.09E+07	0.11	3.64	3.00	0.05
E1	GY	3B	AX.89566060	7.18E+06	0.46	-2.30	3.11	0.05
E1	SPM2	3B	AX.89625599	7.99E+08	0.40	-20.13	4.23	0.08
E1	GY	3B	AX.89675055	2.27E+07	0.20	-3.24	3.79	0.06
E1	GY	3B	AX.89682581	7.05E+07	0.09	3.90	3.05	0.05
E1	SPM2	3B	AX.89362506	8.02E+08	0.37	-23.86	5.59	0.10
E1	SPM2	3B	AX.89703708	8.03E+08	0.39	-21.00	4.53	0.08
E1	GY	3B	AX.89460947	2.29E+08	0.08	4.32	3.20	0.05
E1	GY	3B	AX.89578279	1.91E+08	0.09	4.01	3.07	0.05
E1	GY	3B	AX.89676377	2.36E+08	0.08	4.32	3.20	0.05
E1	SPM2	3B	AX.89596222	7.99E+08	0.44	-18.48	3.73	0.06
E1	SPM2	3B	AX.89495516	8.03E+08	0.39	-21.32	4.64	0.08
E1	GY	3B	AX.89391940	3.76E+08	0.11	3.81	3.25	0.06
E1	GY	3B	AX.89383562	6.50E+08	0.42	-2.64	3.89	0.08
E1	SPM2	3B	AX.89518649	8.00E+08	0.45	-19.21	4.00	0.07
E1	GY	3B	AX.89353028	1.23E+08	0.09	3.91	3.06	0.05
E1	GY	3B	AX.89311427	4.98E+08	0.43	-2.34	3.17	0.05
E1	SPM2	3B	AX.89532604	8.01E+08	0.40	-20.13	4.23	0.08
E1	GY	3B	AX.89600688	2.04E+08	0.07	4.53	3.31	0.06
E1	GY	3B	AX.89357134	2.28E+08	0.07	4.74	3.57	0.06
E1	GY	3B	AX.89323856	1.02E+07	0.48	2.33	3.18	0.05
E1	GY	3B	AX.89311864	4.49E+08	0.12	3.52	3.11	0.05
E1	GY	3B	AX.89564518	7.03E+07	0.09	3.90	3.05	0.05
E1	GY	3B	AX.89564495	7.06E+07	0.09	3.90	3.05	0.05
E1	GY	3B	AX.86168705	1.52E+07	0.33	2.70	3.72	0.06
E1	SPM2	3B	AX.89403940	8.03E+08	0.43	-19.35	4.02	0.07
E1	SPM2	3B	AX.89674879	8.00E+08	0.44	-18.45	3.73	0.06
E1	SPM2	3B	AX.89461841	8.04E+08	0.38	-21.19	4.57	0.08
E1	SPM2	3B	AX.89620465	8.04E+08	0.35	-20.79	4.28	0.08
E1	SPM2	3B	AX.89694777	8.03E+08	0.42	-19.39	4.02	0.07
E1	GY	3B	AX.89719543	1.02E+07	0.29	-2.74	3.55	0.06
E1	GY	3B	AX.89402100	2.03E+08	0.08	4.12	3.08	0.05
E1	GY	3B	AX.89467345	2.40E+08	0.08	4.32	3.20	0.05
E1	GY	3B	AX.86166684	1.25E+08	0.09	3.91	3.06	0.05
E1	GY	3B	AX.89406016	1.73E+07	0.24	2.66	3.07	0.05
E1	SPM2	3B	AX.89735956	7.99E+08	0.44	-18.48	3.73	0.06
E1	SPM2	3B	AX.89695993	8.01E+08	0.44	-18.45	3.73	0.06
E1	GY	3B	AX.89325062	3.70E+08	0.11	3.81	3.26	0.06
E1	SPM2	3B	AX.89492044	8.00E+08	0.44	-18.48	3.73	0.06
E1	GY	3B	AX.89593889	7.00E+07	0.09	4.10	3.31	0.06
E1	GY	3B	AX.89549698	7.82E+08	0.37	-2.74	3.98	0.07
E1	SPM2	3B	AX.89775729	8.00E+08	0.44	-18.45	3.73	0.06
E1	SPM2	3B	AX.89326322	8.04E+08	0.39	-21.00	4.53	0.08
E1	GY	3B	AX.89394258	7.03E+07	0.09	3.90	3.05	0.05
E1	SPM2	3B	AX.89364611	7.99E+08	0.39	-20.20	4.24	0.08
E1	SPM2	3B	AX.89672041	8.03E+08	0.46	-19.40	4.09	0.08

E1	SPM2	3B	AX.89313775	8.01E+08	0.43	-19.35	4.02	0.07
E1	SPM2	3B	AX.89387991	8.03E+08	0.43	-19.35	4.02	0.07
E1	GY	3B	AX.89485953	7.82E+08	0.36	-2.58	3.55	0.06
E1	GY	3B	AX.89569831	7.82E+08	0.37	-2.75	3.98	0.07
E1	SPM2	3B	AX.89544767	7.99E+08	0.40	-20.13	4.23	0.08
E1	GY	3B	AX.89630147	7.06E+07	0.09	3.90	3.05	0.05
E1	SPM2	3B	AX.89374438	8.04E+08	0.39	-21.00	4.53	0.08
E1	GY	3B	AX.89732865	2.40E+08	0.08	4.17	3.14	0.05
E1	SPM2	3B	AX.89386915	8.00E+08	0.44	-18.45	3.73	0.06
E1	GY	3B	AX.89578279	1.91E+08	0.09	4.01	3.07	0.05
E1	SPM2	3B	AX.89340823	7.99E+08	0.39	-20.20	4.24	0.08
E1	GY	3B	AX.89454148	4.48E+08	0.12	3.52	3.11	0.05
E1	GY	3B	AX.89692867	7.82E+08	0.37	-2.74	3.98	0.07
E1	SPM2	3B	AX.89344886	8.03E+08	0.39	-21.00	4.53	0.08
E1	GY	3B	AX.89567643	5.56E+08	0.23	-2.73	3.10	0.05
E1	GY	3B	AX.89643547	2.37E+08	0.08	4.32	3.20	0.05
E1	GY	3B	AX.89348758	9.06E+06	0.36	2.89	4.34	0.07
E1	GY	3B	AX.89478502	1.03E+07	0.30	-2.75	3.66	0.06
E1	SPM2	3B	AX.89723055	8.01E+08	0.43	-20.15	4.32	0.08
E1	GY	3B	AX.89327655	7.03E+07	0.09	3.90	3.05	0.05
E1	GY	3B	AX.89575875	2.44E+07	0.23	-2.87	3.39	0.05
E1	SPM2	3B	AX.89609788	8.01E+08	0.40	-22.97	5.35	0.10
E1	GY	3B	AX.89373893	7.83E+08	0.48	2.46	3.49	0.07
E1	GY	3B	AX.89324266	7.59E+08	0.26	-2.72	3.30	0.06
E1	GY	3B	AX.89366224	1.10E+07	0.43	-2.35	3.18	0.05
E1	GY	3B	AX.89515680	7.03E+07	0.09	3.90	3.05	0.05
E1	GY	3B	AX.89467345	2.40E+08	0.08	4.32	3.20	0.05
E1	GY	3B	AX.89561191	4.47E+08	0.12	3.52	3.11	0.05
E1	GY	3B	AX.89448959	7.03E+07	0.09	3.90	3.05	0.05
E1	GY	3B	AX.89456017	4.08E+07	0.11	3.64	3.00	0.05
E1	GY	3B	AX.89731729	1.59E+07	0.33	2.95	4.32	0.07
E1	SPM2	3B	AX.89400782	7.99E+08	0.40	-20.13	4.23	0.08
E1	GY	3B	AX.89658098	6.85E+07	0.09	3.90	3.05	0.05
E1	GY	3B	AX.89411035	7.01E+07	0.09	3.90	3.05	0.05
E1	SPM2	3B	AX.89527522	7.99E+08	0.44	-18.45	3.73	0.06
E1	GY	3B	AX.89339369	2.33E+08	0.08	4.32	3.20	0.05
E1	SPM2	3B	AX.89672915	8.04E+08	0.39	-20.89	4.49	0.08
E1	SPM2	3B	AX.89763367	8.00E+08	0.40	-20.89	4.54	0.08
E1	SPM2	3B	AX.89491560	7.99E+08	0.44	-18.48	3.73	0.06
E1	GY	3B	AX.89625540	7.82E+08	0.37	-2.75	3.98	0.07
E1	GY	3B	AX.89371805	2.06E+08	0.07	4.53	3.31	0.06
E1	SPM2	3B	AX.89637096	8.04E+08	0.40	-19.31	3.94	0.07
E1	SPM2	3B	AX.89393570	8.01E+08	0.40	-22.01	4.98	0.09
E1	SPM2	3B	AX.89580278	8.00E+08	0.43	-17.70	3.46	0.06
E1	GY	3B	AX.89732865	2.40E+08	0.08	4.17	3.14	0.05
E1	GY	3B	AX.89387027	4.48E+08	0.11	3.90	3.50	0.06
E1	SPM2	3B	AX.89731763	8.03E+08	0.39	-21.00	4.53	0.08
E1	GY	3B	AX.89610917	2.31E+07	0.21	-3.19	3.85	0.06
E1	GY	3B	AX.89517853	1.56E+08	0.08	4.24	3.10	0.05
E1	GY	3B	AX.89517853	1.56E+08	0.08	4.24	3.10	0.05

E1	GY	3B	AX.89364758	7.47E+07	0.09	3.90	3.05	0.05
E1	GY	3B	AX.89587214	5.79E+08	0.19	-2.96	3.13	0.05
E1	GY	3B	AX.89329922	7.01E+07	0.09	3.90	3.05	0.05
E1	SPM2	3B	AX.89357763	8.01E+08	0.38	-22.76	5.19	0.09
E1	SPM2	3B	AX.89737012	8.03E+08	0.38	-21.19	4.57	0.08
E1	SPM2	3B	AX.89411012	8.03E+08	0.39	-21.00	4.53	0.08
E1	GY	3B	AX.89396277	1.03E+07	0.29	-2.74	3.55	0.06
E1	GY	3B	AX.89353028	1.23E+08	0.09	3.91	3.06	0.05
E1	SPM2	3B	AX.89618460	8.00E+08	0.44	-18.48	3.73	0.06
E1	GY	3B	AX.89551857	7.04E+07	0.09	3.90	3.05	0.05
E1	SPM2	3B	AX.89314874	8.03E+08	0.40	-21.68	4.81	0.09
E1	SPM2	3B	AX.89444716	8.00E+08	0.44	-18.45	3.73	0.06
E1	SPM2	3B	AX.89416863	7.99E+08	0.39	-20.20	4.24	0.08
E1	GY	3B	AX.89483927	7.79E+07	0.09	3.90	3.05	0.05
E1	SPM2	3B	AX.89408600	8.00E+08	0.39	-20.39	4.29	0.08
E1	GY	3B	AX.89330691	2.33E+08	0.08	4.32	3.20	0.05
E1	GY	3B	AX.89479047	2.26E+08	0.08	4.32	3.20	0.05
E1	GY	3B	AX.89660595	2.37E+07	0.21	-3.19	3.85	0.06
E1	SPM2	3B	AX.89649950	8.04E+08	0.38	-21.19	4.57	0.08
E1	GY	3B	AX.89409713	2.32E+07	0.21	-3.19	3.85	0.06
E1	GY	3B	AX.89660595	2.37E+07	0.21	-3.19	3.85	0.06
E1	GY	3B	AX.89466525	7.83E+08	0.08	-4.26	3.26	0.05
E1	GY	3B	AX.89644459	1.90E+08	0.07	4.99	3.71	0.06
E1	GY	3B	AX.89506895	1.38E+07	0.22	-2.80	3.20	0.05
E1	SPM2	3B	AX.89773679	8.02E+08	0.43	-20.11	4.31	0.08
E1	GY	3B	AX.89448477	2.33E+08	0.08	4.32	3.20	0.05
E1	GY	3B	AX.89743164	7.82E+08	0.37	-2.75	3.98	0.07
E1	GY	3B	AX.89437067	4.48E+08	0.12	3.52	3.11	0.05
E1	GY	3B	AX.89437067	4.48E+08	0.12	3.52	3.11	0.05
E1	SPM2	3B	AX.89364800	8.01E+08	0.44	-18.45	3.73	0.06
E1	GY	3B	AX.89715248	8.26E+08	0.06	-4.98	3.30	0.05
E1	GY	3B	AX.89366640	2.51E+07	0.23	-2.99	3.66	0.06
E1	GY	3B	AX.89366640	2.51E+07	0.23	-2.99	3.66	0.06
E1	GY	3B	AX.89485267	2.68E+08	0.10	3.81	3.15	0.05
E1	GY	3B	AX.89589610	4.48E+08	0.12	3.52	3.11	0.05
E1	SPM2	3B	AX.89777460	8.01E+08	0.44	-18.45	3.73	0.06
E1	GY	3B	AX.89650451	5.79E+08	0.19	-2.96	3.13	0.05
E1	GY	3B	AX.89650451	5.79E+08	0.19	-2.96	3.13	0.05
E1	SPM2	3B	AX.89393873	7.99E+08	0.44	-18.45	3.73	0.06
E1	SPM2	3B	AX.89505485	8.03E+08	0.40	-21.68	4.81	0.09
E1	SPM2	3B	AX.89523885	8.01E+08	0.44	-18.48	3.73	0.06
E1	SPM2	3B	AX.89486736	8.01E+08	0.43	-19.35	4.02	0.07
E1	GY	3B	AX.89460947	2.29E+08	0.08	4.32	3.20	0.05
E1	SPM2	3B	AX.89721513	7.99E+08	0.39	-20.20	4.24	0.08
E1	GY	3B	AX.89761468	5.79E+08	0.19	-2.96	3.13	0.05
E1	SPM2	3B	AX.89358912	8.03E+08	0.39	-21.00	4.53	0.08
E1	GY	3B	AX.89540019	2.37E+07	0.21	-3.19	3.85	0.06
E1	SPM2	3B	AX.89339775	8.00E+08	0.39	-19.80	4.08	0.07
E1	GY	3B	AX.89473429	4.08E+07	0.11	3.64	3.00	0.05
E1	SPM2	3B	AX.89479409	7.99E+08	0.40	-20.13	4.23	0.08

E1	GY	3B	AX.89450810	1.60E+07	0.33	2.95	4.32	0.07
E1	GY	3B	AX.89611369	7.08E+07	0.10	4.01	3.31	0.06
E1	SPM2	3B	AX.89730128	8.03E+08	0.44	-17.51	3.40	0.06
E1	SPM2	3B	AX.89656217	7.99E+08	0.40	-20.13	4.23	0.08
E1	GY	3B	AX.89615340	2.03E+08	0.08	4.12	3.08	0.05
E1	GY	3B	AX.89404563	9.10E+06	0.38	2.68	3.87	0.06
E1	SPM2	3B	AX.89323935	7.99E+08	0.39	-20.20	4.24	0.08
E1	GY	3B	AX.89616116	7.83E+08	0.48	2.39	3.34	0.06
E1	GY	3B	AX.89616116	7.83E+08	0.48	2.39	3.34	0.06
E1	GY	3B	AX.89544604	2.30E+07	0.21	-3.19	3.85	0.06
E1	SPM2	3B	AX.89372370	8.03E+08	0.39	-21.00	4.53	0.08
E1	GY	3B	AX.89312150	2.26E+08	0.08	4.32	3.20	0.05
E1	GY	3B	AX.89312150	2.26E+08	0.08	4.32	3.20	0.05
E1	GY	3B	AX.89575875	2.44E+07	0.23	-2.87	3.39	0.05
E1	GY	3B	AX.89714524	7.82E+08	0.37	-2.74	3.98	0.07
E1	SPM2	3B	AX.89353869	8.03E+08	0.45	-17.94	3.55	0.06
E1	SPM2	3B	AX.86168521	8.00E+08	0.40	-20.88	4.52	0.08
E1	GY	3B	AX.89381129	2.40E+08	0.08	4.17	3.14	0.05
E1	SPM2	3B	AX.89359679	8.04E+08	0.38	-21.19	4.57	0.08
E1	SPM2	3B	AX.89365459	8.02E+08	0.43	-19.35	4.02	0.07
E1	GY	3B	AX.89358096	7.82E+08	0.47	2.84	4.48	0.09
E1	GY	3B	AX.89511806	2.45E+07	0.27	-2.69	3.36	0.05
E1	GY	3B	AX.89511806	2.45E+07	0.27	-2.69	3.36	0.05
E1	GY	3B	AX.89523771	7.82E+08	0.47	2.99	4.92	0.09
E1	GY	3B	AX.89593554	7.01E+07	0.09	3.90	3.05	0.05
E1	GY	3B	AX.89497828	1.61E+07	0.34	2.63	3.59	0.05
E1	GY	3B	AX.89312853	3.06E+08	0.09	4.00	3.06	0.05
E1	GY	3B	AX.89640877	9.11E+06	0.39	2.36	3.12	0.05
E1	SPM2	3B	AX.89463985	8.00E+08	0.40	-20.13	4.23	0.08
E1	GY	3B	AX.89313026	4.47E+08	0.12	3.52	3.11	0.05
E1	SPM2	3B	AX.89406125	8.03E+08	0.39	-21.00	4.53	0.08
E1	GY	3B	AX.89313251	2.27E+08	0.07	4.74	3.57	0.06
E1	GY	3B	AX.89568398	4.08E+07	0.11	3.64	3.00	0.05
E1	GY	3B	AX.89512828	7.83E+08	0.15	-4.14	4.81	0.08
E1	GY	3B	AX.89524440	9.06E+06	0.36	2.89	4.34	0.07
E1	GY	3B	AX.89476611	7.05E+07	0.09	3.90	3.05	0.05
E1	GY	3B	AX.89676562	1.27E+08	0.09	3.91	3.06	0.05
E1	SPM2	3B	AX.89437166	8.00E+08	0.44	-18.45	3.73	0.06
E1	GY	3B	AX.89489735	7.82E+08	0.37	-2.75	3.98	0.07
E1	GY	3B	AX.89546982	7.82E+08	0.37	-2.54	3.48	0.06
E1	GY	3B	AX.89642745	7.01E+07	0.09	3.90	3.05	0.05
E1	GY	3B	AX.89515390	7.03E+07	0.09	3.90	3.05	0.05
E1	GY	3B	AX.89356046	7.82E+08	0.36	-2.58	3.55	0.06
E1	SPM2	3B	AX.89433905	8.04E+08	0.45	-18.04	3.59	0.06
E1	GY	3B	AX.89348080	7.82E+08	0.38	-2.55	3.53	0.06
E1	GY	3B	AX.89443091	7.04E+07	0.09	3.90	3.05	0.05
E1	GY	3B	AX.89314692	2.46E+07	0.28	-2.64	3.28	0.05
E1	GY	3B	AX.89443091	7.04E+07	0.09	3.90	3.05	0.05
E1	GY	3B	AX.89525850	7.82E+08	0.46	3.18	5.46	0.11
E1	GY	3B	AX.89443514	2.37E+07	0.21	-3.19	3.85	0.06

E1	GY	3B	AX.89323856	1.02E+07	0.48	2.33	3.18	0.05
E1	GY	3B	AX.89718547	1.30E+08	0.09	3.91	3.06	0.05
E1	GY	3B	AX.89316012	7.82E+08	0.38	-2.70	3.91	0.07
E1	GY	3B	AX.89432992	7.82E+08	0.47	2.99	4.92	0.09
E1	SPM2	3B	AX.89703019	8.04E+08	0.39	-21.00	4.53	0.08
E1	SPM2	3B	AX.89599709	7.99E+08	0.39	-20.20	4.24	0.08
E1	SPM2	3B	AX.89754549	8.04E+08	0.39	-21.00	4.53	0.08
E1	SPM2	3B	AX.89501443	8.04E+08	0.39	-21.00	4.53	0.08
E1	GY	3B	AX.89455587	2.29E+08	0.08	4.32	3.20	0.05
E1	GY	3B	AX.89455587	2.29E+08	0.08	4.32	3.20	0.05
E1	SPM2	3B	AX.89528748	8.03E+08	0.40	-18.16	3.53	0.06
E1	GY	3B	AX.89317109	7.01E+07	0.09	3.90	3.05	0.05
E1	GY	3B	AX.89350546	7.84E+08	0.44	-2.43	3.39	0.05
E1	GY	3B	AX.89597701	4.09E+07	0.11	3.64	3.00	0.05
E1	GY	3B	AX.89597701	4.09E+07	0.11	3.64	3.00	0.05
E1	GY	3B	AX.89706937	7.82E+08	0.37	-2.73	3.95	0.07
E1	GY	3B	AX.89597766	7.84E+08	0.46	-2.67	4.02	0.07
E1	GY	3B	AX.89350796	5.79E+08	0.19	-2.96	3.13	0.05
E1	GY	3B	AX.89492320	2.82E+07	0.24	2.63	3.01	0.04
E1	GY	3B	AX.89697322	7.03E+07	0.09	3.90	3.05	0.05
E1	GY	3B	AX.89682271	4.95E+08	0.12	3.47	3.04	0.05
E1	GY	3B	AX.89682271	4.95E+08	0.12	3.47	3.04	0.05
E1	GY	3B	AX.89598242	7.03E+07	0.09	3.90	3.05	0.05
E1	GY	3B	AX.89598242	7.03E+07	0.09	3.90	3.05	0.05
E1	SPM2	3B	AX.89743728	8.03E+08	0.39	-21.00	4.53	0.08
E1	GY	3B	AX.89504416	2.28E+08	0.08	4.32	3.20	0.05
E1	GY	3B	AX.89446220	7.82E+08	0.47	2.99	4.92	0.09
E1	SPM2	3B	AX.89768780	8.04E+08	0.43	-18.64	3.76	0.07
E1	GY	3B	AX.89732442	7.83E+08	0.14	-3.84	4.01	0.06
E1	SPM2	3B	AX.89666371	8.01E+08	0.42	-19.39	4.02	0.07
E1	GY	3B	AX.89712587	1.38E+07	0.24	-3.00	3.73	0.06
E1	GY	3B	AX.89386944	2.03E+08	0.07	4.53	3.31	0.06
E1	SPM2	3B	AX.89454240	8.03E+08	0.40	-21.68	4.81	0.09
E1	GY	3B	AX.89712787	7.82E+08	0.37	-2.75	3.98	0.07
E1	GY	3B	AX.89483101	2.34E+07	0.21	-3.19	3.85	0.06
E1	GY	3B	AX.89587214	5.79E+08	0.19	-2.96	3.13	0.05
E1	GY	3B	AX.89329922	7.01E+07	0.09	3.90	3.05	0.05
E1	GY	3B	AX.89336987	7.82E+08	0.37	-2.74	3.98	0.07
E1	GY	3B	AX.89733538	1.58E+07	0.33	2.79	3.89	0.06
E1	GY	3B	AX.89457880	1.25E+08	0.09	3.91	3.06	0.05
E1	GY	3B	AX.89587994	1.56E+08	0.08	4.24	3.10	0.05
E1	GY	3B	AX.86166684	1.25E+08	0.09	3.91	3.06	0.05
E1	GY	3B	AX.89709938	4.09E+07	0.11	3.64	3.00	0.05
E1	GY	3B	AX.89537035	1.91E+08	0.09	4.01	3.07	0.05
E1	GY	3B	AX.89506367	5.79E+08	0.19	-2.96	3.13	0.05
E1	SPM2	3B	AX.89315788	8.03E+08	0.39	-21.00	4.53	0.08
E1	GY	3B	AX.89377431	7.83E+08	0.08	-4.26	3.26	0.05
E1	GY	3B	AX.89648362	7.82E+08	0.45	3.09	5.17	0.10
E1	SPM2	3B	AX.89761672	8.00E+08	0.44	-18.45	3.73	0.06
E1	GY	3B	AX.89331170	7.82E+08	0.37	-2.92	4.44	0.08

E1	GY	3B	AX.89746446	1.21E+08	0.09	3.91	3.06	0.05
E1	GY	3B	AX.89746446	1.21E+08	0.09	3.91	3.06	0.05
E1	GY	3B	AX.89448477	2.33E+08	0.08	4.32	3.20	0.05
E1	GY	3B	AX.89772132	5.01E+08	0.49	-2.28	3.08	0.06
E1	GY	3B	AX.89674141	4.45E+08	0.12	3.59	3.13	0.05
E1	GY	3B	AX.89320718	7.82E+08	0.36	-2.58	3.54	0.06
E1	GY	3B	AX.89613009	2.28E+07	0.35	-2.94	4.39	0.08
E1	GY	3B	AX.89613009	2.28E+07	0.35	-2.94	4.39	0.08
E1	GY	3B	AX.89674254	1.95E+08	0.07	4.99	3.71	0.06
E1	GY	3B	AX.89674254	1.95E+08	0.07	4.99	3.71	0.06
E1	GY	3B	AX.89448959	7.03E+07	0.09	3.90	3.05	0.05
E1	GY	3B	AX.89589610	4.48E+08	0.12	3.52	3.11	0.05
E1	SPM2	3B	AX.89766559	8.01E+08	0.44	-18.45	3.73	0.06
E1	GY	3B	AX.89425717	8.11E+08	0.17	3.25	3.43	0.06
E1	GY	3B	AX.89613532	2.29E+08	0.07	4.74	3.57	0.06
E1	GY	3B	AX.89718794	1.07E+07	0.43	-2.29	3.06	0.05
E1	GY	3B	AX.89747835	7.03E+07	0.09	3.90	3.05	0.05
E1	GY	3B	AX.89438018	2.36E+08	0.08	4.32	3.20	0.05
E1	GY	3B	AX.89438018	2.36E+08	0.08	4.32	3.20	0.05
E1	GY	3B	AX.89449822	1.03E+07	0.42	-2.42	3.34	0.05
E1	GY	3B	AX.89449822	1.03E+07	0.42	-2.42	3.34	0.05
E1	GY	3B	AX.89744326	4.06E+07	0.11	3.64	3.00	0.05
E1	GY	3B	AX.89355686	1.21E+08	0.09	3.91	3.06	0.05
E1	GY	3B	AX.89603104	1.03E+07	0.44	2.47	3.48	0.05
E1	GY	3B	AX.89415541	7.82E+08	0.46	3.05	5.07	0.10
E1	GY	3B	AX.89356046	7.82E+08	0.36	-2.58	3.55	0.06
E1	GY	3B	AX.89344711	7.05E+07	0.09	3.90	3.05	0.05
E1	GY	3B	AX.89368821	5.45E+08	0.26	-2.68	3.25	0.07
E1	GY	3B	AX.89368821	5.45E+08	0.26	-2.68	3.25	0.07
E1	GY	3B	AX.89450660	2.37E+07	0.21	-3.19	3.85	0.06
E1	GY	3B	AX.89450660	2.37E+07	0.21	-3.19	3.85	0.06
E1	SPM2	3B	AX.89597132	8.00E+08	0.44	-18.45	3.73	0.06
E1	GY	3B	AX.89643290	7.03E+07	0.09	3.90	3.05	0.05
E1	GY	3B	AX.89701327	7.82E+08	0.36	-2.58	3.55	0.06
E1	GY	3B	AX.89311427	4.98E+08	0.43	-2.34	3.17	0.05
E1	GY	3B	AX.89532812	1.25E+08	0.09	3.91	3.06	0.05
E1	GY	3B	AX.89615340	2.03E+08	0.08	4.12	3.08	0.05
E1	SPM2	3B	AX.89352432	8.04E+08	0.38	-21.19	4.57	0.08
E1	GY	3B	AX.89749476	4.10E+07	0.11	3.64	3.00	0.05
E1	GY	3B	AX.89497934	4.48E+08	0.12	3.75	3.37	0.06
E1	GY	3B	AX.89639311	4.98E+08	0.43	-2.43	3.37	0.06
E1	GY	3B	AX.89702348	7.82E+08	0.45	3.09	5.17	0.10
E1	GY	3B	AX.89714524	7.82E+08	0.37	-2.74	3.98	0.07
E1	GY	3B	AX.89380994	7.18E+07	0.09	3.90	3.05	0.05
E1	GY	3B	AX.89324194	1.03E+07	0.47	-2.62	3.90	0.06
E1	SPM2	3B	AX.89720786	8.01E+08	0.43	-20.81	4.58	0.08
E1	GY	3B	AX.89674141	4.45E+08	0.12	3.59	3.13	0.05
E1	GY	3B	AX.89346209	2.29E+08	0.08	4.32	3.20	0.05
E1	GY	3B	AX.89717464	5.79E+08	0.19	-2.96	3.13	0.05
E1	GY	3B	AX.89593712	7.81E+08	0.46	3.18	5.46	0.11

E1	GY	3B	AX.89542020	3.24E+07	0.46	2.52	3.64	0.06
E1	GY	3B	AX.89763741	2.25E+08	0.08	4.32	3.20	0.05
E1	GY	3B	AX.89763741	2.25E+08	0.08	4.32	3.20	0.05
E1	GY	3B	AX.89393889	7.82E+08	0.46	3.18	5.46	0.11
E1	GY	3B	AX.89641049	3.03E+07	0.17	3.55	4.06	0.07
E1	GY	3B	AX.89593889	7.00E+07	0.09	4.10	3.31	0.06
E1	GY	3B	AX.89394258	7.03E+07	0.09	3.90	3.05	0.05
E1	GY	3B	AX.89716122	7.01E+08	0.05	-5.86	3.52	0.07
E1	GY	3B	AX.89617989	4.08E+07	0.11	3.64	3.00	0.05
E1	GY	3B	AX.89617989	4.08E+07	0.11	3.64	3.00	0.05
E1	GY	3B	AX.89476611	7.05E+07	0.09	3.90	3.05	0.05
E1	GY	3B	AX.89642350	7.85E+08	0.15	-3.42	3.52	0.05
E1	GY	3B	AX.89642350	7.85E+08	0.15	-3.42	3.52	0.05
E1	GY	3B	AX.89431154	1.07E+07	0.43	-2.29	3.06	0.05
E1	SPM2	3B	AX.89583201	8.01E+08	0.43	-18.04	3.57	0.06
E1	SPM2	3B	AX.89449178	8.01E+08	0.40	-20.13	4.23	0.08
E1	GY	3B	AX.89704524	9.78E+06	0.40	-2.60	3.72	0.05
E1	SPM2	3B	AX.89390144	8.00E+08	0.44	-18.45	3.73	0.06
E1	GY	3B	AX.89454148	4.48E+08	0.12	3.52	3.11	0.05
E1	GY	3B	AX.89383562	6.50E+08	0.42	-2.64	3.89	0.08
E1	GY	3B	AX.89348709	4.08E+07	0.11	3.64	3.00	0.05
E1	GY	3B	AX.89396346	5.79E+08	0.19	-2.96	3.13	0.05
E1	GY	3B	AX.89396346	5.79E+08	0.19	-2.96	3.13	0.05
E1	GY	3B	AX.89501558	1.25E+08	0.09	3.91	3.06	0.05
E1	GY	3B	AX.89643547	2.37E+08	0.08	4.32	3.20	0.05
E1	GY	3B	AX.89348758	9.06E+06	0.36	2.89	4.34	0.07
E1	SPM2	3B	AX.89641049	3.03E+07	0.17	24.53	3.79	0.07
E1	GY	3B	AX.89396850	7.82E+08	0.37	-2.62	3.70	0.06
E1	GY	3B	AX.89396850	7.82E+08	0.37	-2.62	3.70	0.06
E1	GY	3B	AX.89466138	4.48E+08	0.12	3.75	3.37	0.06
E1	GY	3B	AX.89466138	4.48E+08	0.12	3.75	3.37	0.06
E1	GY	3B	AX.89466525	7.83E+08	0.08	-4.26	3.26	0.05
E1	SPM2	3B	AX.89714810	8.04E+08	0.38	-21.19	4.57	0.08
E1	GY	3B	AX.89661779	2.33E+08	0.08	4.32	3.20	0.05
E1	GY	3B	AX.89350546	7.84E+08	0.44	-2.43	3.39	0.05
E1	GY	3B	AX.89328273	7.81E+08	0.46	3.18	5.46	0.11
E1	GY	3B	AX.89609467	7.81E+08	0.36	-2.74	3.95	0.07
E1	GY	3B	AX.89706937	7.82E+08	0.37	-2.73	3.95	0.07
E1	GY	3B	AX.89492320	2.82E+07	0.24	2.63	3.01	0.04
E1	GY	3B	AX.89666287	2.27E+07	0.20	-3.16	3.67	0.06
E1	SPM2	3B	AX.89676518	8.04E+08	0.38	-21.39	4.62	0.08
E1	GY	3B	AX.89433995	2.69E+07	0.31	2.56	3.27	0.05
E1	GY	3B	AX.89411035	7.01E+07	0.09	3.90	3.05	0.05
E1	GY	3B	AX.89339369	2.33E+08	0.08	4.32	3.20	0.05
E1	GY	3B	AX.89456542	7.01E+07	0.09	3.90	3.05	0.05
E1	SPM2	3B	AX.89743587	8.01E+08	0.44	-18.45	3.73	0.06
E1	GY	3B	AX.89708070	1.73E+07	0.25	2.84	3.47	0.06
E1	GY	3B	AX.89482460	7.82E+08	0.37	-2.75	3.98	0.07
E1	GY	3B	AX.89610535	7.95E+07	0.09	3.90	3.05	0.05
E1	GY	3B	AX.89708291	7.82E+08	0.46	3.18	5.46	0.11

E1	SPM2	3B	AX.89686025	8.03E+08	0.43	-19.35	4.02	0.07
E1	SPM2	3B	AX.89768378	8.03E+08	0.47	-18.89	3.92	0.07
E1	GY	3B	AX.89551077	5.79E+08	0.19	-2.96	3.13	0.05
E1	GY	3B	AX.89457880	1.25E+08	0.09	3.91	3.06	0.05
E1	GY	3B	AX.89457913	2.28E+08	0.08	4.32	3.20	0.05
E1	GY	3B	AX.89709731	1.52E+07	0.33	2.70	3.72	0.06
E1	GY	3B	AX.89709731	1.52E+07	0.33	2.70	3.72	0.06
E1	GY	3B	AX.89749484	4.47E+08	0.12	3.52	3.11	0.05
E1	GY	3B	AX.89749484	4.47E+08	0.12	3.52	3.11	0.05
E1	GY	3B	AX.89501988	1.06E+07	0.46	2.37	3.28	0.05
E1	GY	3B	AX.89501988	1.06E+07	0.46	2.37	3.28	0.05
E1	SPM2	3B	AX.89447359	8.03E+08	0.43	-20.04	4.27	0.08
E1	SPM2	3B	AX.89721250	8.00E+08	0.40	-20.13	4.23	0.08
E1	GY	3B	AX.89564518	7.03E+07	0.09	3.90	3.05	0.05
E1	SPM2	3B	AX.89576984	4.04E+07	0.31	17.79	3.08	0.05
E1	GY	3B	AX.89710866	1.02E+07	0.29	-2.74	3.55	0.06
E1	SPM2	3B	AX.89472809	4.05E+07	0.31	17.79	3.08	0.05
E1	GY	3B	AX.89459177	5.02E+08	0.48	-2.26	3.04	0.06
E1	GY	3B	AX.89772132	5.01E+08	0.49	-2.28	3.08	0.06
E1	GY	3B	AX.89719543	1.02E+07	0.29	-2.74	3.55	0.06
E1	GY	3B	AX.89402100	2.03E+08	0.08	4.12	3.08	0.05
E1	SPM2	3B	AX.89383290	7.99E+08	0.40	-20.13	4.23	0.08
E1	GY	3B	AX.89686563	1.90E+08	0.09	4.01	3.07	0.05
E1	GY	3B	AX.89549698	7.82E+08	0.37	-2.74	3.98	0.07
E1	SPM2	3B	AX.89677281	8.00E+08	0.40	-20.13	4.23	0.08
E1	GY	3B	AX.89332256	2.36E+07	0.21	-2.95	3.41	0.05
E1	GY	3B	AX.89390294	2.45E+07	0.28	-2.64	3.28	0.05
E1	GY	3B	AX.89343746	4.09E+07	0.11	3.64	3.00	0.05
E1	GY	3B	AX.89485953	7.82E+08	0.36	-2.58	3.55	0.06
E1	GY	3B	AX.89625540	7.82E+08	0.37	-2.75	3.98	0.07
E1	GY	3B	AX.89554686	4.52E+08	0.12	3.56	3.08	0.05
E1	SPM2	3B	AX.89398301	8.00E+08	0.39	-20.20	4.24	0.08
E1	SPM2	3B	AX.89668740	8.04E+08	0.42	-18.87	3.84	0.07
E1	GY	3B	AX.89737117	7.03E+07	0.09	3.90	3.05	0.05
E1	GY	3B	AX.89473429	4.08E+07	0.11	3.64	3.00	0.05
E1	GY	3B	AX.89737415	7.05E+07	0.09	3.90	3.05	0.05
E1	GY	3B	AX.89737415	7.05E+07	0.09	3.90	3.05	0.05
E1	SPM2	3B	AX.89424787	7.99E+08	0.40	-20.13	4.23	0.08
E1	GY	3B	AX.89672215	7.83E+08	0.48	2.46	3.49	0.07
E1	GY	3B	AX.89333525	5.45E+08	0.27	-2.61	3.13	0.06
E1	GY	3B	AX.89578679	7.83E+08	0.46	-2.67	4.02	0.07
E1	SPM2	3B	AX.89460703	8.03E+08	0.39	-21.00	4.53	0.08
E1	GY	3B	AX.89497601	7.14E+07	0.10	3.97	3.26	0.06
E1	GY	3B	AX.89497601	7.14E+07	0.10	3.97	3.26	0.06
E1	GY	3B	AX.89567643	5.56E+08	0.23	-2.73	3.10	0.05
E1	GY	3B	AX.89497659	7.82E+08	0.37	-2.74	3.98	0.07
E1	GY	3B	AX.89404398	1.03E+07	0.48	2.51	3.62	0.06
E1	GY	3B	AX.89404398	1.03E+07	0.48	2.51	3.62	0.06
E1	GY	3B	AX.89748250	7.03E+07	0.09	4.52	3.93	0.07
E1	GY	3B	AX.89626033	2.27E+08	0.08	4.32	3.20	0.05

E1	GY	3B	AX.89579270	7.04E+07	0.09	3.90	3.05	0.05
E1	GY	3B	AX.89762790	2.29E+08	0.07	4.74	3.57	0.06
E1	GY	3B	AX.89762790	2.29E+08	0.07	4.74	3.57	0.06
E1	GY	3B	AX.89702348	7.82E+08	0.45	3.09	5.17	0.10
E1	GY	3B	AX.89475144	4.45E+08	0.12	3.52	3.11	0.05
E1	SPM2	3B	AX.89567903	4.05E+07	0.31	17.79	3.08	0.05
E1	SPM2	3B	AX.89325676	8.01E+08	0.39	-20.64	4.40	0.08
E1	GY	3B	AX.89462944	2.28E+08	0.08	4.32	3.20	0.05
E1	SPM2	3B	AX.89772928	8.00E+08	0.40	-20.13	4.23	0.08
E1	SPM2	3B	AX.89471918	8.00E+08	0.40	-20.13	4.23	0.08
E1	SPM2	3B	AX.89522839	8.00E+08	0.44	-18.48	3.73	0.06
E1	GY	3B	AX.89406016	1.73E+07	0.24	2.66	3.07	0.05
E1	GY	3B	AX.89703117	4.48E+08	0.12	3.75	3.37	0.06
E1	GY	3B	AX.89325062	3.70E+08	0.11	3.81	3.26	0.06
E1	GY	3B	AX.89735675	7.82E+08	0.37	-2.74	3.98	0.07
E1	GY	3B	AX.89371203	4.05E+07	0.11	3.64	3.00	0.05
E1	GY	3B	AX.89371203	4.05E+07	0.11	3.64	3.00	0.05
E1	GY	3B	AX.89606724	8.01E+05	0.07	-4.61	3.06	0.05
E1	GY	3B	AX.89716233	7.03E+07	0.09	3.90	3.05	0.05
E1	GY	3B	AX.89359431	4.09E+07	0.11	3.64	3.00	0.05
E1	GY	3B	AX.89569831	7.82E+08	0.37	-2.75	3.98	0.07
E1	SPM2	3B	AX.89503767	8.01E+08	0.40	-20.31	4.30	0.08
E1	GY	3B	AX.89371805	2.06E+08	0.07	4.53	3.31	0.06
E1	GY	3B	AX.89647652	2.04E+08	0.07	4.53	3.31	0.06
E1	GY	3B	AX.89647652	2.04E+08	0.07	4.53	3.31	0.06
E1	GY	3B	AX.89371943	4.99E+08	0.43	-2.38	3.26	0.05
E1	GY	3B	AX.89642745	7.01E+07	0.09	3.90	3.05	0.05
E1	SPM2	3B	AX.89536448	7.99E+08	0.40	-20.95	4.53	0.08
E1	GY	3B	AX.89692867	7.82E+08	0.37	-2.74	3.98	0.07
E1	GY	3B	AX.89336987	7.82E+08	0.37	-2.74	3.98	0.07
E1	SPM2	3B	AX.89310850	8.01E+08	0.42	-19.07	3.90	0.07
E1	GY	3B	AX.89478502	1.03E+07	0.30	-2.75	3.66	0.06
E1	GY	3B	AX.89465739	7.82E+08	0.37	-2.74	3.98	0.07
E1	SPM2	3B	AX.89608889	4.05E+07	0.31	17.79	3.08	0.05
E1	SPM2	3B	AX.89642903	8.00E+08	0.40	-20.13	4.23	0.08
E1	GY	3B	AX.89656498	1.25E+08	0.09	3.91	3.06	0.05
E1	GY	3B	AX.89656498	1.25E+08	0.09	3.91	3.06	0.05
E1	SPM2	3B	AX.89422160	4.05E+07	0.31	17.79	3.08	0.05
E1	SPM2	3B	AX.89586500	8.01E+08	0.40	-20.13	4.23	0.08
E1	GY	3B	AX.89515680	7.03E+07	0.09	3.90	3.05	0.05
E1	GY	3B	AX.89433585	1.27E+08	0.09	3.91	3.06	0.05
E1	GY	3B	AX.89621185	7.82E+08	0.37	-2.74	3.98	0.07
E1	GY	3B	AX.89350796	5.79E+08	0.19	-2.96	3.13	0.05
E1	GY	3B	AX.89398767	7.04E+07	0.09	3.90	3.05	0.05
E1	GY	3B	AX.89317630	2.36E+08	0.08	4.32	3.20	0.05
E1	SPM2	3B	AX.89395005	8.00E+08	0.40	-20.13	4.23	0.08
E1	SPM2	3B	AX.89601515	8.01E+08	0.44	-18.45	3.73	0.06
E1	GY	3B	AX.89411094	7.72E+07	0.09	3.90	3.05	0.05
E1	GY	3B	AX.89411094	7.72E+07	0.09	3.90	3.05	0.05
E1	GY	3B	AX.89585986	7.83E+08	0.15	-4.14	4.81	0.08

E1	SPM2	3B	AX.89668153	8.01E+08	0.40	-20.13	4.23	0.08
E1	SPM2	3B	AX.89526703	8.01E+08	0.39	-21.87	4.87	0.09
E1	GY	3B	AX.89551077	5.79E+08	0.19	-2.96	3.13	0.05
E1	GY	3B	AX.89540019	2.37E+07	0.21	-3.19	3.85	0.06
E1	GY	3B	AX.89412494	5.51E+08	0.09	-5.01	4.52	0.07
E1	GY	3B	AX.89364758	7.47E+07	0.09	3.90	3.05	0.05
E1	GY	3B	AX.89447089	8.10E+08	0.18	3.23	3.58	0.06
E1	GY	3B	AX.89611369	7.08E+07	0.10	4.01	3.31	0.06
E1	GY	3B	AX.89587994	1.56E+08	0.08	4.24	3.10	0.05
E1	SPM2	3B	AX.89771913	8.00E+08	0.39	-20.20	4.24	0.08
E1	GY	3B	AX.89483927	7.79E+07	0.09	3.90	3.05	0.05
E1	GY	3B	AX.89478738	2.36E+08	0.08	4.32	3.20	0.05
E1	GY	3B	AX.89612255	5.79E+08	0.19	-2.96	3.13	0.05
E1	GY	3B	AX.89612255	5.79E+08	0.19	-2.96	3.13	0.05
E1	GY	3B	AX.89644459	1.90E+08	0.07	4.99	3.71	0.06
E1	GY	3B	AX.89710866	1.02E+07	0.29	-2.74	3.55	0.06
E1	GY	3B	AX.89506895	1.38E+07	0.22	-2.80	3.20	0.05
E1	GY	3B	AX.89592950	1.91E+08	0.09	4.01	3.07	0.05
E1	GY	3B	AX.89701197	1.25E+08	0.09	3.91	3.06	0.05
E1	GY	3B	AX.89715248	8.26E+08	0.06	-4.98	3.30	0.05
E1	GY	3B	AX.89593435	1.73E+08	0.10	3.97	3.38	0.06
E1	GY	3B	AX.89593435	1.73E+08	0.10	3.97	3.38	0.06
E1	GY	3B	AX.89726171	7.82E+08	0.46	3.18	5.46	0.11
E1	GY	3B	AX.89726171	7.82E+08	0.46	3.18	5.46	0.11
E1	GY	3B	AX.89455859	2.29E+08	0.08	4.32	3.20	0.05
E1	GY	3B	AX.89402447	5.02E+08	0.39	2.33	3.04	0.06
E1	GY	3B	AX.89332256	2.36E+07	0.21	-2.95	3.41	0.05
E1	GY	3B	AX.89724263	7.82E+08	0.37	-2.74	3.98	0.07
E1	GY	3B	AX.89637578	1.70E+08	0.09	4.18	3.30	0.06
E1	GY	3B	AX.89637578	1.70E+08	0.09	4.18	3.30	0.06
E1	GY	3B	AX.89415541	7.82E+08	0.46	3.05	5.07	0.10
E1	GY	3B	AX.89344711	7.05E+07	0.09	3.90	3.05	0.05
E1	GY	3B	AX.89775197	7.83E+08	0.48	2.46	3.49	0.07
E1	GY	3B	AX.89638988	1.25E+08	0.09	3.91	3.06	0.05
E1	GY	3B	AX.89638988	1.25E+08	0.09	3.91	3.06	0.05
E1	GY	3B	AX.89532812	1.25E+08	0.09	3.91	3.06	0.05
E1	GY	3B	AX.89404563	9.10E+06	0.38	2.68	3.87	0.06
E1	GY	3B	AX.89507261	7.04E+07	0.10	3.97	3.26	0.06
E1	GY	3B	AX.89649582	2.27E+08	0.07	4.74	3.57	0.06
E1	GY	3B	AX.89451599	1.73E+07	0.43	3.10	5.18	0.09
E1	GY	3B	AX.89451599	1.73E+07	0.43	3.10	5.18	0.09
E1	GY	3B	AX.89623656	4.47E+08	0.12	3.52	3.11	0.05
E1	GY	3B	AX.89726612	1.91E+08	0.09	4.01	3.07	0.05
E1	GY	3B	AX.89640219	2.40E+08	0.08	4.17	3.14	0.05
E1	GY	3B	AX.89523771	7.82E+08	0.47	2.99	4.92	0.09
E1	GY	3B	AX.89536978	2.40E+08	0.08	4.17	3.14	0.05
E1	GY	3B	AX.89536978	2.40E+08	0.08	4.17	3.14	0.05
E1	GY	3B	AX.89641049	3.03E+07	0.17	3.55	4.06	0.07
E1	GY	3B	AX.89750860	4.47E+08	0.12	3.52	3.11	0.05
E1	GY	3B	AX.89313278	1.88E+08	0.09	3.91	3.06	0.05

E1	GY	3B	AX.89420916	8.11E+08	0.17	3.25	3.43	0.06
E1	GY	3B	AX.89568398	4.08E+07	0.11	3.64	3.00	0.05
E1	GY	3B	AX.89716233	7.03E+07	0.09	3.90	3.05	0.05
E1	GY	3B	AX.89569738	8.10E+08	0.17	3.12	3.26	0.06
E1	GY	3B	AX.89335703	1.03E+07	0.30	-2.72	3.57	0.06
E1	GY	3B	AX.89524440	9.06E+06	0.36	2.89	4.34	0.07
E1	GY	3B	AX.89629664	5.32E+08	0.09	-5.81	5.88	0.10
E1	GY	3B	AX.89629664	5.32E+08	0.09	-5.81	5.88	0.10
E1	GY	3B	AX.89489735	7.82E+08	0.37	-2.75	3.98	0.07
E1	GY	3B	AX.89704353	3.78E+08	0.14	3.21	3.02	0.05
E1	GY	3B	AX.89704524	9.78E+06	0.40	-2.60	3.72	0.05
E1	GY	3B	AX.89348080	7.82E+08	0.38	-2.55	3.53	0.06
E1	GY	3B	AX.89525850	7.82E+08	0.46	3.18	5.46	0.11
E1	GY	3B	AX.89443514	2.37E+07	0.21	-3.19	3.85	0.06
E1	GY	3B	AX.89681951	4.99E+08	0.44	-2.27	3.03	0.05
E1	GPS	3B	AX.89519027	6.24E+06	0.20	1.50	3.54	0.06
E1	GY	3B	AX.89547637	9.41E+06	0.40	-2.49	3.46	0.05
E1	SPM2	3B	AX.89731037	8.00E+08	0.44	-18.45	3.73	0.06
E1	GY	3B	AX.89708451	7.83E+08	0.46	-2.67	4.02	0.07
E1	GY	3B	AX.89512476	2.33E+08	0.08	4.32	3.20	0.05
E1	GY	3B	AX.89564495	7.06E+07	0.09	3.90	3.05	0.05
E1	GY	3B	AX.89409713	2.32E+07	0.21	-3.19	3.85	0.06
E1	GY	3B	AX.89676084	5.56E+08	0.23	-2.73	3.10	0.05
E1	GY	3B	AX.89676084	5.56E+08	0.23	-2.73	3.10	0.05
E1	GY	3B	AX.89385111	8.11E+08	0.15	3.17	3.09	0.05
E1	SPM2	3B	AX.89557510	4.04E+07	0.31	17.79	3.08	0.05
E1	GY	3B	AX.89467096	7.82E+08	0.46	3.18	5.46	0.11
E1	GY	3B	AX.89467096	7.82E+08	0.46	3.18	5.46	0.11
E1	GY	3B	AX.89350426	1.27E+08	0.09	3.91	3.06	0.05
E1	GY	3B	AX.89350426	1.27E+08	0.09	3.91	3.06	0.05
E1	GY	3B	AX.89640549	7.08E+07	0.09	3.90	3.05	0.05
E1	GY	3B	AX.89640549	7.08E+07	0.09	3.90	3.05	0.05
E1	GY	3B	AX.89661779	2.33E+08	0.08	4.32	3.20	0.05
E1	GY	3B	AX.89350608	4.47E+08	0.12	3.52	3.11	0.05
E1	GY	3B	AX.89350608	4.47E+08	0.12	3.52	3.11	0.05
E1	GY	3B	AX.89621185	7.82E+08	0.37	-2.74	3.98	0.07
E1	GY	3B	AX.89697322	7.03E+07	0.09	3.90	3.05	0.05
E1	GY	3B	AX.89707408	4.98E+08	0.44	-2.27	3.03	0.05
E1	GY	3B	AX.89670424	2.36E+08	0.08	4.32	3.20	0.05
E1	GY	3B	AX.89670424	2.36E+08	0.08	4.32	3.20	0.05
E1	GY	3B	AX.89562240	7.08E+07	0.09	3.90	3.05	0.05
E1	GY	3B	AX.89550050	1.26E+08	0.09	3.91	3.06	0.05
E1	GY	3B	AX.89550050	1.26E+08	0.09	3.91	3.06	0.05
E1	GY	3B	AX.89446220	7.82E+08	0.47	2.99	4.92	0.09
E1	GY	3B	AX.89610535	7.95E+07	0.09	3.90	3.05	0.05
E1	GY	3B	AX.89387027	4.48E+08	0.11	3.90	3.50	0.06
E1	GY	3B	AX.89634987	5.87E+08	0.30	-2.69	3.54	0.07
E1	GY	3B	AX.89712787	7.82E+08	0.37	-2.75	3.98	0.07
E1	GY	3B	AX.89733538	1.58E+07	0.33	2.79	3.89	0.06
E1	GY	3B	AX.89457913	2.28E+08	0.08	4.32	3.20	0.05

E1	GY	3B	AX.89572586	2.36E+08	0.08	4.32	3.20	0.05
E1	GY	3B	AX.89549485	2.33E+07	0.21	-3.19	3.85	0.06
E1	GY	3B	AX.89458065	2.69E+07	0.31	2.56	3.27	0.05
E1	GY	3B	AX.89478738	2.36E+08	0.08	4.32	3.20	0.05
E1	GY	3B	AX.89567015	4.10E+07	0.11	3.64	3.00	0.05
E1	GY	3B	AX.89537035	1.91E+08	0.09	4.01	3.07	0.05
E1	GY	3B	AX.89506367	5.79E+08	0.19	-2.96	3.13	0.05
E1	GY	3B	AX.89616953	2.33E+07	0.21	-3.19	3.85	0.06
E1	GY	3B	AX.89616953	2.33E+07	0.21	-3.19	3.85	0.06
E1	GY	3B	AX.89459177	5.02E+08	0.48	-2.26	3.04	0.06
E1	GY	3B	AX.89702448	1.61E+07	0.34	2.63	3.59	0.05
E1	GY	3B	AX.89702448	1.61E+07	0.34	2.63	3.59	0.05
E1	GY	3B	AX.89731446	4.47E+08	0.12	3.52	3.11	0.05
E1	GY	3B	AX.89593642	7.15E+07	0.09	3.90	3.05	0.05
E1	GY	3B	AX.89674421	7.82E+08	0.46	3.05	5.07	0.10
E1	GY	3B	AX.89735575	7.93E+07	0.09	3.90	3.05	0.05
E1	GY	3B	AX.89613532	2.29E+08	0.07	4.74	3.57	0.06
E1	GY	3B	AX.89743633	6.89E+07	0.09	3.90	3.05	0.05
E1	GY	3B	AX.89390294	2.45E+07	0.28	-2.64	3.28	0.05
E1	GY	3B	AX.89648935	5.45E+05	0.06	-5.13	3.26	0.05
E1	GY	3B	AX.89343746	4.09E+07	0.11	3.64	3.00	0.05
E1	GY	3B	AX.89682581	7.05E+07	0.09	3.90	3.05	0.05
E1	GY	3B	AX.89355686	1.21E+08	0.09	3.91	3.06	0.05
E1	GY	3B	AX.89603104	1.03E+07	0.44	2.47	3.48	0.05
E1	GY	3B	AX.89626166	7.82E+08	0.37	-2.74	3.98	0.07
E1	GY	3B	AX.89626166	7.82E+08	0.37	-2.74	3.98	0.07
E1	GY	3B	AX.89776937	2.37E+07	0.21	-3.19	3.85	0.06
E1	GY	3B	AX.89587259	7.82E+08	0.48	2.46	3.49	0.07
E1	GY	3B	AX.89587259	7.82E+08	0.48	2.46	3.49	0.07
E1	GY	3B	AX.89643290	7.03E+07	0.09	3.90	3.05	0.05
E1	GY	3B	AX.89497659	7.82E+08	0.37	-2.74	3.98	0.07
E1	GY	3B	AX.89749476	4.10E+07	0.11	3.64	3.00	0.05
E1	GY	3B	AX.89639311	4.98E+08	0.43	-2.43	3.37	0.06
E1	GY	3B	AX.89623656	4.47E+08	0.12	3.52	3.11	0.05
E1	GY	3B	AX.89428910	2.50E+08	0.07	4.99	3.71	0.06
E1	GY	3B	AX.89428910	2.50E+08	0.07	4.99	3.71	0.06
E1	GY	3B	AX.89357554	1.31E+08	0.09	3.91	3.06	0.05
E1	GY	3B	AX.89324266	7.59E+08	0.26	-2.72	3.30	0.06
E1	GY	3B	AX.89358096	7.82E+08	0.47	2.84	4.48	0.09
E1	GY	3B	AX.89640219	2.40E+08	0.08	4.17	3.14	0.05
E1	GY	3B	AX.89346209	2.29E+08	0.08	4.32	3.20	0.05
E1	GY	3B	AX.89542020	3.24E+07	0.46	2.52	3.64	0.06
E1	GY	3B	AX.89393889	7.82E+08	0.46	3.18	5.46	0.11
E1	GY	3B	AX.89325193	5.79E+08	0.19	-3.11	3.46	0.05
E1	GY	3B	AX.89325193	5.79E+08	0.19	-3.11	3.46	0.05
E1	GY	3B	AX.89569738	8.10E+08	0.17	3.12	3.26	0.06
E1	GY	3B	AX.89359431	4.09E+07	0.11	3.64	3.00	0.05
E1	GY	3B	AX.89501558	1.25E+08	0.09	3.91	3.06	0.05
E1	GY	3B	AX.89619334	2.03E+08	0.08	4.12	3.08	0.05
E1	GY	3B	AX.89619334	2.03E+08	0.08	4.12	3.08	0.05

E1	GY	3B	AX.89656360	4.48E+08	0.12	3.52	3.11	0.05
E1	GY	3B	AX.89506820	7.83E+08	0.42	2.79	4.27	0.08
E1	GY	3B	AX.89385111	8.11E+08	0.15	3.17	3.09	0.05
E1	GY	3B	AX.89743164	7.82E+08	0.37	-2.75	3.98	0.07
E1	GY	3B	AX.89374324	7.74E+08	0.31	2.49	3.12	0.05
E1	GY	3B	AX.89374324	7.74E+08	0.31	2.49	3.12	0.05
E1	GY	3B	AX.89561191	4.47E+08	0.12	3.52	3.11	0.05
E1	GY	3B	AX.89666287	2.27E+07	0.20	-3.16	3.67	0.06
E1	GY	3B	AX.89433995	2.69E+07	0.31	2.56	3.27	0.05
E1	SPM2	3B	AX.89729061	8.00E+08	0.40	-20.13	4.23	0.08
E1	GY	3B	AX.89708070	1.73E+07	0.25	2.84	3.47	0.06
E1	SPM2	3B	AX.89517744	8.03E+08	0.40	-22.22	5.04	0.09
E1	GPM2	3B	AX.89568494	7.84E+08	0.40	-630.62	3.40	0.06
E1	SPM2	3B	AX.89376242	8.00E+08	0.44	-18.45	3.73	0.06
E1	GPM2	3B	AX.89324266	7.59E+08	0.26	-836.65	4.54	0.08
E1	GPM2	3B	AX.89527523	7.79E+08	0.27	-702.25	3.45	0.06
E1	SPM2	3B	AX.89491936	8.01E+08	0.40	-20.13	4.23	0.08
E1	GPM2	3B	AX.89731729	1.59E+07	0.33	612.32	3.02	0.05
E1	GY	3B	AX.89648362	7.82E+08	0.45	3.09	5.17	0.10
E1	GPM2	3B	AX.89343079	7.80E+08	0.32	-647.99	3.26	0.05
E1	GPM2	3B	AX.89642355	7.79E+08	0.29	-681.91	3.40	0.06
E1	GPM2	3B	AX.86168777	7.57E+08	0.31	-633.53	3.09	0.06
E1	GPM2	3B	AX.89347050	7.79E+08	0.29	-681.91	3.40	0.06
E1	GY	3B	AX.89686563	1.90E+08	0.09	4.01	3.07	0.05
E1	GPM2	3B	AX.89478502	1.03E+07	0.30	-691.22	3.56	0.06
E1	GPM2	3B	AX.89353573	7.79E+08	0.29	-681.91	3.40	0.06
E1	GPM2	3B	AX.89346210	9.09E+06	0.23	688.62	3.03	0.05
E1	GY	3B	AX.89566060	7.18E+06	0.46	-2.30	3.11	0.05
E1	GPM2	3B	AX.89710866	1.02E+07	0.29	-672.83	3.32	0.06
E1	GPM2	3B	AX.89524440	9.06E+06	0.36	875.77	5.86	0.10
E1	GPM2	3B	AX.89444077	7.79E+08	0.28	-662.71	3.21	0.05
E1	GY	3B	AX.89662734	2.42E+08	0.07	4.53	3.31	0.06
E1	GY	3B	AX.89662734	2.42E+08	0.07	4.53	3.31	0.06
E1	GPM2	3B	AX.89667159	7.81E+08	0.32	-647.99	3.26	0.05
E1	GY	3B	AX.89761468	5.79E+08	0.19	-2.96	3.13	0.05
E1	GY	3B	AX.89412401	2.32E+07	0.21	-3.19	3.85	0.06
E1	GPM2	3B	AX.89693634	7.84E+08	0.40	-630.62	3.40	0.06
E1	GY	3B	AX.89737117	7.03E+07	0.09	3.90	3.05	0.05
E1	GPM2	3B	AX.89585449	7.78E+08	0.28	-652.49	3.13	0.05
E1	GY	3B	AX.89578679	7.83E+08	0.46	-2.67	4.02	0.07
E1	GY	3B	AX.89748250	7.03E+07	0.09	4.52	3.93	0.07
E1	GPM2	3B	AX.89348758	9.06E+06	0.36	875.77	5.86	0.10
E1	GY	3B	AX.89626033	2.27E+08	0.08	4.32	3.20	0.05
E1	GPM2	3B	AX.89658901	7.78E+08	0.28	-665.44	3.20	0.05
E1	GY	3B	AX.89475144	4.45E+08	0.12	3.52	3.11	0.05
E1	GPM2	3B	AX.89682320	7.83E+08	0.40	-630.62	3.40	0.06
E1	GY	3B	AX.89357554	1.31E+08	0.09	3.91	3.06	0.05
E1	GPM2	3B	AX.89431154	1.07E+07	0.43	-606.21	3.24	0.05
E1	GPM2	3B	AX.89770084	7.79E+08	0.29	-681.91	3.40	0.06
E1	GY	3B	AX.89640877	9.11E+06	0.39	2.36	3.12	0.05

E1	GY	3B	AX.89716122	7.01E+08	0.05	-5.86	3.52	0.07
E1	GY	3B	AX.89606724	8.01E+05	0.07	-4.61	3.06	0.05
E1	GY	3B	AX.89512828	7.83E+08	0.15	-4.14	4.81	0.08
E1	GPM2	3B	AX.89688746	7.84E+08	0.40	-630.62	3.40	0.06
E1	GPM2	3B	AX.89557750	7.79E+08	0.28	-662.71	3.21	0.05
E1	GY	3B	AX.89647639	4.08E+07	0.11	3.64	3.00	0.05
E1	GPM2	3B	AX.89686365	7.80E+08	0.29	-641.62	3.06	0.05
E1	GY	3B	AX.89509695	4.48E+08	0.12	3.52	3.11	0.05
E1	GPM2	3B	AX.89450810	1.60E+07	0.33	612.32	3.02	0.05
E1	GPM2	3B	AX.89473642	7.81E+08	0.32	-647.99	3.26	0.05
E1	GPM2	3B	AX.89366224	1.10E+07	0.43	-581.72	3.01	0.05
E1	SPM2	3B	AX.89509875	7.99E+08	0.39	-20.20	4.24	0.08
E1	GY	3B	AX.89518919	4.47E+08	0.12	3.52	3.11	0.05
E1	GY	3B	AX.89518919	4.47E+08	0.12	3.52	3.11	0.05
E1	GY	3B	AX.89628047	2.03E+08	0.07	4.99	3.71	0.06
E1	GY	3B	AX.89628047	2.03E+08	0.07	4.99	3.71	0.06
E1	SPM2	3B	AX.89579245	8.01E+08	0.44	-18.15	3.62	0.06
E1	GPS	3B	AX.89328945	7.64E+08	0.12	1.79	3.37	0.06
E1	GPM2	3B	AX.86164426	8.53E+06	0.22	706.05	3.08	0.05
E1	GY	3B	AX.89597766	7.84E+08	0.46	-2.67	4.02	0.07
E1	GPM2	3B	AX.89312665	7.79E+08	0.28	-662.71	3.21	0.05
E1	GY	3B	AX.89561688	4.47E+08	0.12	3.52	3.11	0.05
E1	GPM2	3B	AX.89331199	7.81E+08	0.32	-647.99	3.26	0.05
E1	GPM2	3B	AX.89519170	7.81E+08	0.32	-643.94	3.24	0.05
E1	GPM2	3B	AX.89669995	7.80E+08	0.32	-647.99	3.26	0.05
E1	GPM2	3B	AX.89386142	1.61E+07	0.26	682.10	3.19	0.05
E1	GY	3B	AX.89712587	1.38E+07	0.24	-3.00	3.73	0.06
E1	GPM2	3B	AX.89344914	7.99E+08	0.28	-685.93	3.40	0.05
E1	GY	3B	AX.89376090	4.72E+07	0.17	-3.02	3.07	0.05
E1	GY	3B	AX.89376090	4.72E+07	0.17	-3.02	3.07	0.05
E1	GPM2	3B	AX.89712587	1.38E+07	0.24	-690.57	3.12	0.05
E1	GY	3B	AX.89610917	2.31E+07	0.21	-3.19	3.85	0.06
E1	GPM2	3B	AX.89364321	7.79E+08	0.29	-681.91	3.40	0.06
E1	GPS	3B	AX.89478502	1.03E+07	0.30	-1.22	3.16	0.05
E1	GY	3B	AX.89447089	8.10E+08	0.18	3.23	3.58	0.06
E1	GY	3B	AX.89551857	7.04E+07	0.09	3.90	3.05	0.05
E1	GY	3B	AX.89549485	2.33E+07	0.21	-3.19	3.85	0.06
E1	GPM2	3B	AX.89350546	7.84E+08	0.44	-688.14	4.05	0.07
E1	GPM2	3B	AX.89569738	8.10E+08	0.17	780.57	3.13	0.05
E1	GY	3B	AX.89673700	6.89E+07	0.10	3.97	3.26	0.06
E1	GY	3B	AX.89592950	1.91E+08	0.09	4.01	3.07	0.05
E1	GPM2	3B	AX.89597766	7.84E+08	0.46	-721.10	4.41	0.07
E1	GPM2	3B	AX.89573300	7.79E+08	0.28	-662.71	3.21	0.05
E1	GPM2	3B	AX.89611713	7.80E+08	0.28	-673.62	3.30	0.06
E1	GPM2	3B	AX.89324194	1.03E+07	0.47	-592.02	3.15	0.05
E1	GY	3B	AX.89438012	7.83E+08	0.15	-4.03	4.57	0.07
E1	GY	3B	AX.89438012	7.83E+08	0.15	-4.03	4.57	0.07
E1	GPM2	3B	AX.89442266	7.84E+08	0.40	-630.62	3.40	0.06
E1	GY	3B	AX.89776937	2.37E+07	0.21	-3.19	3.85	0.06
E1	GPM2	3B	AX.89520965	7.84E+08	0.46	-701.25	4.20	0.07

E1	GY	3B	AX.89450810	1.60E+07	0.33	2.95	4.32	0.07
E1	GPM2	3B	AX.89319461	7.78E+08	0.31	-648.49	3.22	0.05
E1	GY	3B	AX.89652048	7.05E+07	0.09	3.90	3.05	0.05
E1	GPM2	3B	AX.89451599	1.73E+07	0.43	678.36	3.93	0.06
E1	GY	3B	AX.89507261	7.04E+07	0.10	3.97	3.26	0.06
E1	GY	3B	AX.89649582	2.27E+08	0.07	4.74	3.57	0.06
E1	GPM2	3B	AX.89530695	7.80E+08	0.29	-641.62	3.06	0.05
E1	GY	3B	AX.89579270	7.04E+07	0.09	3.90	3.05	0.05
E1	GY	3B	AX.89544604	2.30E+07	0.21	-3.19	3.85	0.06
E1	GPM2	3B	AX.89404563	9.10E+06	0.38	819.97	5.30	0.09
E1	GPM2	3B	AX.89675810	7.78E+08	0.28	-652.49	3.13	0.05
E1	GY	3B	AX.89717464	5.79E+08	0.19	-2.96	3.13	0.05
E1	GY	3B	AX.89497828	1.61E+07	0.34	2.63	3.59	0.05
E1	GY	3B	AX.89750860	4.47E+08	0.12	3.52	3.11	0.05
E1	GY	3B	AX.89313251	2.27E+08	0.07	4.74	3.57	0.06
E1	GY	3B	AX.89313278	1.88E+08	0.09	3.91	3.06	0.05
E1	GY	3B	AX.89420916	8.11E+08	0.17	3.25	3.43	0.06
E1	GY	3B	AX.89676562	1.27E+08	0.09	3.91	3.06	0.05
E1	GPM2	3B	AX.89469152	7.80E+08	0.29	-681.91	3.40	0.06
E1	GY	3B	AX.89546982	7.82E+08	0.37	-2.54	3.48	0.06
E1	GPM2	3B	AX.89678975	7.79E+08	0.28	-736.60	3.85	0.07
E1	GPM2	3B	AX.89482090	7.80E+08	0.32	-647.99	3.26	0.05
E1	GY	3B	AX.89515390	7.03E+07	0.09	3.90	3.05	0.05
E1	GPS	3B	AX.89404751	1.03E+07	0.34	-1.16	3.06	0.05
E1	GY	3B	AX.89477823	1.26E+08	0.09	3.95	3.11	0.05
E1	GY	3B	AX.89477823	1.26E+08	0.09	3.95	3.11	0.05
E1	GY	3B	AX.89681951	4.99E+08	0.44	-2.27	3.03	0.05
E1	GY	3B	AX.89547637	9.41E+06	0.40	-2.49	3.46	0.05
E1	GPM2	3B	AX.89444477	7.80E+08	0.32	-647.99	3.26	0.05
E1	GY	3B	AX.89718547	1.30E+08	0.09	3.91	3.06	0.05
E1	GY	3B	AX.89708451	7.83E+08	0.46	-2.67	4.02	0.07
E1	GPM2	3B	AX.89715478	7.79E+08	0.29	-681.91	3.40	0.06
E1	GY	3B	AX.89512476	2.33E+08	0.08	4.32	3.20	0.05
E1	GY	3B	AX.89373893	7.83E+08	0.48	2.46	3.49	0.07
E1	GPM2	3B	AX.89509274	9.09E+06	0.49	-582.80	3.08	0.04
E1	GPM2	3B	AX.89425717	8.11E+08	0.17	821.00	3.35	0.06
E1	GPM2	3B	AX.89403052	7.79E+08	0.28	-662.71	3.21	0.05
E1	GPM2	3B	AX.89447307	9.06E+06	0.36	875.77	5.86	0.10
E1	GY	3B	AX.89317630	2.36E+08	0.08	4.32	3.20	0.05
E1	GPM2	3B	AX.89612830	7.79E+08	0.28	-662.71	3.21	0.05
E1	GPM2	3B	AX.89417381	7.94E+08	0.28	-662.71	3.21	0.05
E1	GPM2	3B	AX.89529960	9.05E+06	0.50	-618.81	3.41	0.05
E1	GY	3B	AX.89504416	2.28E+08	0.08	4.32	3.20	0.05
E1	GY	3B	AX.89708291	7.82E+08	0.46	3.18	5.46	0.11
E1	GPM2	3B	AX.89511158	7.79E+08	0.28	-662.71	3.21	0.05
E1	GY	3B	AX.89622367	6.19E+08	0.27	2.60	3.10	0.05
E1	GY	3B	AX.89622367	6.19E+08	0.27	2.60	3.10	0.05
E1	GPM2	3B	AX.89396277	1.03E+07	0.29	-672.83	3.32	0.06
E1	GY	3B	AX.89352945	7.82E+08	0.36	-2.47	3.30	0.06
E1	GY	3B	AX.89572586	2.36E+08	0.08	4.32	3.20	0.05

E1	GPM2	3B	AX.89671073	8.69E+06	0.23	688.62	3.03	0.05
E1	GY	3B	AX.89458065	2.69E+07	0.31	2.56	3.27	0.05
E1	GY	3B	AX.89695720	4.39E+07	0.10	-4.84	4.59	0.09
E1	GY	3B	AX.89695720	4.39E+07	0.10	-4.84	4.59	0.09
E1	GY	3B	AX.89709938	4.09E+07	0.11	3.64	3.00	0.05
E1	GPM2	3B	AX.89537790	3.23E+07	0.20	759.73	3.35	0.05
E1	GY	3B	AX.89567015	4.10E+07	0.11	3.64	3.00	0.05
E1	GPM2	3B	AX.89535981	7.80E+08	0.32	-647.99	3.26	0.05
E1	GY	3B	AX.89598717	4.47E+08	0.12	3.52	3.11	0.05
E1	GY	3B	AX.89598717	4.47E+08	0.12	3.52	3.11	0.05
E1	GPM2	3B	AX.89702431	8.88E+06	0.48	-579.68	3.05	0.04
E1	GY	3B	AX.89320718	7.82E+08	0.36	-2.58	3.54	0.06
E1	GY	3B	AX.89731446	4.47E+08	0.12	3.52	3.11	0.05
E1	GPM2	3B	AX.89708451	7.83E+08	0.46	-721.10	4.41	0.07
E1	GY	3B	AX.89425717	8.11E+08	0.17	3.25	3.43	0.06
E1	GPM2	3B	AX.89585986	7.83E+08	0.15	-839.22	3.21	0.05
E1	GY	3B	AX.89747835	7.03E+07	0.09	3.90	3.05	0.05
E1	GY	3B	AX.89648935	5.45E+05	0.06	-5.13	3.26	0.05
E1	GPM2	3B	AX.89397930	7.79E+08	0.29	-681.91	3.40	0.06
E1	GY	3B	AX.89672215	7.83E+08	0.48	2.46	3.49	0.07
E1	GY	3B	AX.89587730	2.36E+07	0.28	-2.64	3.28	0.05
E1	GY	3B	AX.89587730	2.36E+07	0.28	-2.64	3.28	0.05
E1	GY	3B	AX.89324194	1.03E+07	0.47	-2.62	3.90	0.06
E1	GY	3B	AX.89462944	2.28E+08	0.08	4.32	3.20	0.05
E1	GY	3B	AX.89703117	4.48E+08	0.12	3.75	3.37	0.06
E1	GY	3B	AX.89735675	7.82E+08	0.37	-2.74	3.98	0.07
E1	GY	3B	AX.89431154	1.07E+07	0.43	-2.29	3.06	0.05
E1	GY	3B	AX.89656360	4.48E+08	0.12	3.52	3.11	0.05
E1	GY	3B	AX.89506820	7.83E+08	0.42	2.79	4.27	0.08
E1	GY	3B	AX.89433585	1.27E+08	0.09	3.91	3.06	0.05
E1	GY	3B	AX.89609467	7.81E+08	0.36	-2.74	3.95	0.07
E1	GY	3B	AX.89456542	7.01E+07	0.09	3.90	3.05	0.05
E1	GPM2	3B	AX.89445456	7.79E+08	0.28	-736.60	3.85	0.07
E1	GPM2	3B	AX.89346962	1.73E+07	0.28	678.34	3.31	0.06
E1	GPM2	3B	AX.89406016	1.73E+07	0.24	678.83	3.06	0.05
E1	GY	3B	AX.89479047	2.26E+08	0.08	4.32	3.20	0.05
E1	GPM2	3B	AX.89723221	7.79E+08	0.28	-662.71	3.21	0.05
E1	GPM2	3B	AX.89613009	2.28E+07	0.35	-621.38	3.16	0.05
E1	GPM2	3B	AX.89310735	7.84E+08	0.40	-630.62	3.40	0.06
E1	GPM2	3B	AX.89570689	7.80E+08	0.29	-641.62	3.06	0.05
E1	GPM2	3B	AX.89447089	8.10E+08	0.18	769.74	3.17	0.05
E1	GY	3B	AX.89412401	2.32E+07	0.21	-3.19	3.85	0.06
E1	GPM2	3B	AX.89552393	7.81E+08	0.32	-647.99	3.26	0.05
E1	GPM2	3B	AX.89388524	1.03E+07	0.30	-670.63	3.36	0.06
E1	GPM2	3B	AX.89578679	7.83E+08	0.46	-721.10	4.41	0.07
E1	GPM2	3B	AX.89640753	7.81E+08	0.32	-647.99	3.26	0.05
E1	SPM2	3B	AX.89655431	8.00E+08	0.40	-20.13	4.23	0.08
E1	GPM2	3B	AX.89612085	7.80E+08	0.32	-647.99	3.26	0.05
E1	GPM2	3B	AX.86184460	7.78E+08	0.29	-681.91	3.40	0.06
E1	GPM2	3B	AX.89334115	7.79E+08	0.28	-662.71	3.21	0.05

E1	GPM2	3B	AX.89621691	1.07E+07	0.43	-584.98	3.04	0.05
E1	GPM2	3B	AX.89350549	7.79E+08	0.28	-662.71	3.21	0.05
E1	GPM2	3B	AX.89640877	9.11E+06	0.39	767.69	4.73	0.08
E1	GY	3B	AX.89432992	7.82E+08	0.47	2.99	4.92	0.09
E1	GPM2	3B	AX.89732442	7.83E+08	0.14	-849.51	3.13	0.05
E1	GPM2	3B	AX.89397659	7.80E+08	0.29	-641.62	3.06	0.05
E1	GPM2	3B	AX.89632308	8.88E+06	0.22	751.75	3.48	0.06
E1	GPM2	3B	AX.89775990	7.79E+08	0.29	-681.91	3.40	0.06
E1	GPM2	3B	AX.89628117	7.80E+08	0.32	-647.99	3.26	0.05
E1	GPS	3B	AX.89684282	6.24E+06	0.20	1.49	3.55	0.06
E1	GPM2	3B	AX.89652742	7.78E+08	0.28	-662.71	3.21	0.05
E1	GPM2	3B	AX.89473109	7.84E+08	0.40	-630.62	3.40	0.06
E1	GPM2	3B	AX.89426226	7.81E+08	0.32	-647.99	3.26	0.05
E1	GPM2	3B	AX.89486900	7.81E+08	0.32	-644.25	3.25	0.05
E1	GPM2	3B	AX.89335703	1.03E+07	0.30	-670.63	3.36	0.06
E1	GY	3B	AX.89673700	6.89E+07	0.10	3.97	3.26	0.06
E1	GPM2	3B	AX.89562306	7.78E+08	0.28	-662.71	3.21	0.05
E1	GY	3B	AX.89701197	1.25E+08	0.09	3.91	3.06	0.05
E1	GY	3B	AX.89724263	7.82E+08	0.37	-2.74	3.98	0.07
E1	GPM2	3B	AX.89363834	7.78E+08	0.28	-736.60	3.85	0.07
E1	GPM2	3B	AX.89419440	7.78E+08	0.28	-652.49	3.13	0.05
E1	GY	3B	AX.89652048	7.05E+07	0.09	3.90	3.05	0.05
E1	GPM2	3B	AX.89492440	7.79E+08	0.28	-662.71	3.21	0.05
E1	GPM2	3B	AX.89438012	7.83E+08	0.15	-841.49	3.22	0.05
E1	GY	3B	AX.89381129	2.40E+08	0.08	4.17	3.14	0.05
E1	GPM2	3B	AX.89684935	7.79E+08	0.28	-662.71	3.21	0.05
E1	GPM2	3B	AX.89718794	1.07E+07	0.43	-606.21	3.24	0.05
E1	GPM2	3B	AX.89488281	7.84E+08	0.40	-630.62	3.40	0.06
E1	TKW	3B	AX.89576618	7.81E+08	0.22	-1.23	4.29	0.08
E1	GY	3B	AX.89704353	3.78E+08	0.14	3.21	3.02	0.05
E1	GPM2	3B	AX.89591832	7.60E+08	0.28	-736.60	3.85	0.07
E1	GPM2	3B	AX.89717332	7.79E+08	0.28	-662.71	3.21	0.05
E1	GPM2	3B	AX.89749397	7.80E+08	0.30	-634.05	3.05	0.05
E1	GPM2	3B	AX.89516673	7.78E+08	0.28	-665.44	3.20	0.05
E1	GPM2	3B	AX.89708070	1.73E+07	0.25	736.90	3.56	0.06
E1	GPM2	3B	AX.89695048	7.79E+08	0.29	-681.91	3.40	0.06
E1	GPM2	3B	AX.89402274	7.79E+08	0.29	-681.91	3.40	0.06
E1	GPM2	3B	AX.89771587	7.79E+08	0.29	-681.91	3.40	0.06
E1	GY	3B	AX.89718794	1.07E+07	0.43	-2.29	3.06	0.05
E1	GPM2	3B	AX.89730557	7.56E+08	0.23	-680.20	3.00	0.06
E1	GPM2	3B	AX.89512828	7.83E+08	0.15	-839.22	3.21	0.05
E1	GY	3B	AX.89352945	7.82E+08	0.36	-2.47	3.30	0.06
E1	GPM2	3B	AX.89719543	1.02E+07	0.29	-672.83	3.32	0.06
E1	TKW	3B	AX.89500032	5.89E+06	0.16	1.21	3.34	0.06
E1	TKW	3B	AX.89436601	6.67E+07	0.10	-1.52	3.47	0.06
E1	TKW	3B	AX.89520178	6.68E+07	0.10	-1.54	3.69	0.06
E1	GPM2	3B	AX.89406443	7.81E+08	0.32	-647.99	3.26	0.05
E1	GY	3B	AX.89593642	7.15E+07	0.09	3.90	3.05	0.05
E1	TKW	3B	AX.89316012	7.82E+08	0.38	-0.92	3.42	0.06
E1	GPM2	3B	AX.89725034	7.81E+08	0.31	-710.54	3.76	0.06

E1	GPM2	3B	AX.89698718	7.94E+08	0.26	-681.50	3.22	0.05
E1	GY	3B	AX.89743633	6.89E+07	0.09	3.90	3.05	0.05
E1	GPM2	3B	AX.89488878	7.79E+08	0.28	-662.71	3.21	0.05
E1	GY	3B	AX.89744326	4.06E+07	0.11	3.64	3.00	0.05
E1	TKW	3B	AX.89621185	7.82E+08	0.37	-0.88	3.17	0.05
E1	TKW	3B	AX.89546982	7.82E+08	0.37	-0.86	3.00	0.05
E1	TKW	3B	AX.89714524	7.82E+08	0.37	-0.88	3.17	0.05
E1	TKW	3B	AX.89352945	7.82E+08	0.36	-0.91	3.29	0.06
E1	GY	3B	AX.89357134	2.28E+08	0.07	4.74	3.57	0.06
E1	TKW	3B	AX.89667416	5.89E+06	0.19	1.20	3.75	0.07
E1	GY	3B	AX.89327655	7.03E+07	0.09	3.90	3.05	0.05
E1	GPM2	3B	AX.89724483	8.53E+06	0.22	706.05	3.08	0.05
E1	TKW	3B	AX.89582655	5.95E+06	0.16	1.21	3.34	0.06
E1	TKW	3B	AX.89549698	7.82E+08	0.37	-0.88	3.17	0.05
E1	TKW	3B	AX.89625540	7.82E+08	0.37	-0.88	3.12	0.05
E1	GY	3B	AX.89708796	4.48E+08	0.12	3.75	3.37	0.06
E1	TKW	3B	AX.89352330	5.59E+06	0.16	1.16	3.19	0.05
E1	SPM2	3B	AX.89627455	7.99E+08	0.41	-18.79	3.78	0.07
E1	TKW	3B	AX.89482460	7.82E+08	0.37	-0.88	3.12	0.05
E1	TKW	3B	AX.89650372	5.95E+06	0.16	1.21	3.34	0.06
E1	TKW	3B	AX.89609467	7.81E+08	0.36	-0.88	3.13	0.05
E1	TKW	3B	AX.89626166	7.82E+08	0.37	-0.88	3.17	0.05
E1	TKW	3B	AX.89603356	7.83E+08	0.21	-1.44	5.48	0.10
E1	TKW	3B	AX.89431787	7.81E+08	0.22	-1.23	4.29	0.08
E1	TKW	3B	AX.89743164	7.82E+08	0.37	-0.88	3.12	0.05
E1	GY	3B	AX.89726612	1.91E+08	0.09	4.01	3.07	0.05
E1	TKW	3B	AX.89415162	8.22E+08	0.40	-0.96	3.76	0.07
E1	TKW	3B	AX.89336987	7.82E+08	0.37	-0.88	3.17	0.05
E1	TKW	3B	AX.89689905	7.81E+08	0.23	-1.21	4.30	0.08
E1	TKW	3B	AX.89497659	7.82E+08	0.37	-0.88	3.17	0.05
E1	TKW	3B	AX.89396850	7.82E+08	0.37	-0.92	3.37	0.06
E1	GY	3B	AX.89647639	4.08E+07	0.11	3.64	3.00	0.05
E1	GY	3B	AX.89509695	4.48E+08	0.12	3.52	3.11	0.05
E1	TKW	3B	AX.89489735	7.82E+08	0.37	-0.88	3.12	0.05
E1	TKW	3B	AX.89677381	5.89E+06	0.17	1.18	3.32	0.06
E1	TKW	3B	AX.89764435	7.21E+08	0.13	-1.32	3.29	0.06
E1	GPM2	3B	AX.89526313	1.06E+07	0.43	-584.98	3.04	0.05
E1	TKW	3B	AX.89712787	7.82E+08	0.37	-0.88	3.12	0.05
E1	GY	3B	AX.89482460	7.82E+08	0.37	-2.75	3.98	0.07
E1	TKW	3B	AX.89752358	5.67E+06	0.16	1.20	3.35	0.06
E1	GPM2	3B	AX.89437605	7.80E+08	0.27	-697.12	3.44	0.06
E1	TKW	3B	AX.89623881	7.81E+08	0.22	-1.23	4.29	0.08
E1	TKW	3B	AX.89569831	7.82E+08	0.37	-0.88	3.12	0.05
E1	TKW	3B	AX.86162701	6.67E+07	0.10	-1.52	3.47	0.06
E1	TKW	3B	AX.89535127	5.95E+06	0.19	1.20	3.75	0.07
E1	GPM2	3B	AX.89420916	8.11E+08	0.17	821.00	3.35	0.06
E1	GPM2	3B	AX.89471698	7.80E+08	0.32	-647.99	3.26	0.05
E1	TKW	3B	AX.89706937	7.82E+08	0.37	-0.89	3.18	0.05
E1	TKW	3B	AX.89402216	6.67E+07	0.10	-1.52	3.47	0.06
E1	TKW	3B	AX.89385248	5.95E+06	0.16	1.21	3.34	0.06

E1	TKW	3B	AX.89735675	7.82E+08	0.37	-0.88	3.17	0.05
E1	TKW	3B	AX.89617963	6.68E+07	0.10	-1.52	3.47	0.06
E1	TKW	3B	AX.89692867	7.82E+08	0.37	-0.88	3.17	0.05
E1	TKW	3B	AX.89724263	7.82E+08	0.37	-0.88	3.17	0.05
E1	TKW	3B	AX.89465739	7.82E+08	0.37	-0.88	3.17	0.05
E1	TKW	3B	AX.86169570	6.52E+07	0.11	-1.65	4.46	0.08
E2	SPM2	3B	AX.89616723	8.04E+08	0.30	12.87	3.12	0.05
E2	SPM2	3B	AX.89733548	7.88E+08	0.39	-13.33	3.67	0.07
E2	SPM2	3B	AX.86170407	7.57E+08	0.30	-13.07	3.20	0.06
E2	SPM2	3B	AX.89485134	2.46E+07	0.19	19.73	4.91	0.09
E2	GY	3B	AX.89708404	7.49E+08	0.11	-3.25	3.38	0.04
E2	GY	3B	AX.89412494	5.51E+08	0.09	-3.55	3.38	0.04
E2	GY	3B	AX.89412494	5.51E+08	0.09	-3.55	3.38	0.04
E2	SPM2	3B	AX.89513892	7.88E+08	0.39	-13.33	3.67	0.07
E2	SPM2	3B	AX.89422036	2.44E+07	0.09	19.96	3.02	0.05
E2	SPM2	3B	AX.89617988	7.88E+08	0.38	-12.28	3.15	0.06
E2	SPM2	3B	AX.89723222	2.99E+07	0.22	18.90	5.06	0.09
E2	GY	3B	AX.89647962	4.98E+08	0.41	-2.18	3.76	0.07
E2	GY	3B	AX.89647962	4.98E+08	0.41	-2.18	3.76	0.07
E2	SPM2	3B	AX.89549213	7.88E+08	0.39	-13.33	3.67	0.07
E2	SPM2	3B	AX.89414924	7.88E+08	0.38	-12.28	3.15	0.06
E2	GY	3B	AX.89459177	5.02E+08	0.48	-1.90	3.06	0.06
E2	SPM2	3B	AX.89665225	7.88E+08	0.39	-13.33	3.67	0.07
E2	GY	3B	AX.89366640	2.51E+07	0.23	-2.45	3.61	0.06
E2	SPM2	3B	AX.89544549	4.70E+08	0.09	22.46	3.55	0.06
E2	SPM2	3B	AX.89409920	2.45E+07	0.23	18.32	4.92	0.09
E2	TKW	3B	AX.89746044	7.24E+08	0.35	-0.95	3.07	0.06
E2	SPM2	3B	AX.89334546	7.88E+08	0.39	-13.01	3.50	0.06
E2	SPM2	3B	AX.89660660	7.71E+08	0.22	-14.76	3.33	0.06
E2	SPM2	3B	AX.89436721	7.88E+08	0.38	-12.28	3.15	0.06
E2	SPM2	3B	AX.89347051	7.88E+08	0.37	12.24	3.11	0.05
E2	SPM2	3B	AX.89599926	7.88E+08	0.37	12.24	3.11	0.05
E2	SPM2	3B	AX.89507462	7.88E+08	0.39	-13.33	3.67	0.07
E2	SPM2	3B	AX.89366611	7.88E+08	0.37	12.24	3.11	0.05
E2	SPM2	3B	AX.89315807	7.88E+08	0.39	-13.25	3.63	0.07
E2	SPM2	3B	AX.89351074	2.47E+07	0.19	19.73	4.91	0.09
E2	SPM2	3B	AX.89677428	7.88E+08	0.37	12.24	3.11	0.05
E2	SPM2	3B	AX.89387995	4.71E+08	0.07	24.16	3.34	0.06
E2	GY	3B	AX.89714336	7.47E+08	0.11	-3.25	3.38	0.04
E2	SPM2	3B	AX.89628799	2.46E+07	0.19	19.73	4.91	0.09
E2	SPM2	3B	AX.89485019	8.04E+08	0.26	13.49	3.13	0.05
E2	SPM2	3B	AX.89624504	5.02E+07	0.05	26.43	3.04	0.05
E2	SPM2	3B	AX.89641049	3.03E+07	0.17	20.48	4.97	0.09
E2	SPM2	3B	AX.89729494	7.88E+08	0.39	-13.33	3.67	0.07
E2	GY	3B	AX.89442003	2.50E+07	0.11	-4.32	5.81	0.08
E2	SPM2	3B	AX.89416738	7.88E+08	0.40	-12.92	3.49	0.06
E2	SPM2	3B	AX.89334477	2.90E+08	0.06	30.20	4.38	0.08
E2	GY	3B	AX.89371943	4.99E+08	0.43	-1.92	3.05	0.05
E2	SPM2	3B	AX.89485976	7.88E+08	0.39	-13.01	3.50	0.06
E2	SPM2	3B	AX.89574706	7.88E+08	0.38	-12.28	3.15	0.06

E2	SPM2	3B	AX.89648148	2.46E+07	0.20	15.29	3.30	0.06
E2	SPM2	3B	AX.89354127	7.88E+08	0.39	-13.33	3.67	0.07
E2	SPM2	3B	AX.89392777	7.88E+08	0.22	-15.93	3.80	0.07
E2	SPM2	3B	AX.89453789	7.88E+08	0.36	12.28	3.11	0.05
E2	SPM2	3B	AX.89385397	8.19E+08	0.07	24.05	3.14	0.05
E2	SPM2	3B	AX.89386935	7.69E+08	0.48	12.76	3.55	0.06
E2	SPM2	3B	AX.89632021	7.71E+08	0.49	11.61	3.02	0.05
E2	SPM2	3B	AX.89595574	7.88E+08	0.40	-12.57	3.33	0.06
E2	SPM2	3B	AX.89458103	7.88E+08	0.40	-13.83	3.93	0.07
E2	GY	3B	AX.89683325	2.49E+07	0.11	-4.40	5.80	0.08
E2	GY	3B	AX.89683325	2.49E+07	0.11	-4.40	5.80	0.08
E2	GY	3B	AX.89559935	2.49E+07	0.09	-3.79	4.00	0.05
E2	GY	3B	AX.89559935	2.49E+07	0.09	-3.79	4.00	0.05
E2	GY	3B	AX.89662130	2.51E+07	0.11	-4.40	5.80	0.08
E2	GY	3B	AX.89662130	2.51E+07	0.11	-4.40	5.80	0.08
E2	GY	3B	AX.89415429	7.49E+08	0.11	-3.25	3.38	0.04
E2	GY	3B	AX.89311113	7.49E+08	0.11	-3.25	3.38	0.04
E2	GY	3B	AX.89311113	7.49E+08	0.11	-3.25	3.38	0.04
E2	GY	3B	AX.89510036	7.48E+08	0.11	-3.25	3.38	0.04
E2	GY	3B	AX.89510036	7.48E+08	0.11	-3.25	3.38	0.04
E2	GY	3B	AX.89311427	4.98E+08	0.43	-1.97	3.21	0.05
E2	GY	3B	AX.89311427	4.98E+08	0.43	-1.97	3.21	0.05
E2	GY	3B	AX.89627057	2.49E+07	0.15	-2.63	3.09	0.06
E2	GY	3B	AX.89627057	2.49E+07	0.15	-2.63	3.09	0.06
E2	GY	3B	AX.89357555	2.51E+07	0.13	-3.26	3.93	0.05
E2	GY	3B	AX.89451868	7.50E+08	0.06	-4.89	4.04	0.05
E2	GY	3B	AX.89451868	7.50E+08	0.06	-4.89	4.04	0.05
E2	GY	3B	AX.89500820	2.51E+07	0.10	-3.71	4.13	0.06
E2	GY	3B	AX.89500820	2.51E+07	0.10	-3.71	4.13	0.06
E2	GY	3B	AX.89419406	2.49E+07	0.11	-4.40	5.80	0.08
E2	GY	3B	AX.89419406	2.49E+07	0.11	-4.40	5.80	0.08
E2	GY	3B	AX.89372430	2.51E+07	0.11	-4.40	5.80	0.08
E2	GY	3B	AX.89372430	2.51E+07	0.11	-4.40	5.80	0.08
E2	GY	3B	AX.89361709	2.49E+07	0.25	-2.28	3.24	0.04
E2	GY	3B	AX.89455482	4.97E+08	0.42	-1.93	3.05	0.05
E2	GY	3B	AX.89455482	4.97E+08	0.42	-1.93	3.05	0.05
E2	GY	3B	AX.89339028	2.51E+07	0.11	-4.40	5.80	0.08
E2	GY	3B	AX.89339028	2.51E+07	0.11	-4.40	5.80	0.08
E2	GY	3B	AX.89773338	7.47E+08	0.11	-3.25	3.38	0.04
E2	GY	3B	AX.89773338	7.47E+08	0.11	-3.25	3.38	0.04
E2	GPM2	3B	AX.89776719	4.32E+06	0.17	-641.04	3.04	0.05
E2	GY	3B	AX.89577476	7.47E+08	0.11	-3.25	3.38	0.04
E2	GY	3B	AX.89415429	7.49E+08	0.11	-3.25	3.38	0.04
E2	GY	3B	AX.89716122	7.01E+08	0.05	-6.56	5.96	0.12
E2	GY	3B	AX.89716122	7.01E+08	0.05	-6.56	5.96	0.12
E2	GY	3B	AX.89357555	2.51E+07	0.13	-3.26	3.93	0.05
E2	GY	3B	AX.89512828	7.83E+08	0.15	-2.86	3.42	0.05
E2	GY	3B	AX.89442003	2.50E+07	0.11	-4.32	5.81	0.08
E2	GY	3B	AX.89327129	3.45E+08	0.26	-2.39	3.65	0.06
E2	GY	3B	AX.89327129	3.45E+08	0.26	-2.39	3.65	0.06

E2	GY	3B	AX.89502384	7.49E+08	0.10	-3.37	3.48	0.04
E2	GY	3B	AX.89502384	7.49E+08	0.10	-3.37	3.48	0.04
E2	GY	3B	AX.89361709	2.49E+07	0.25	-2.28	3.24	0.04
E2	TKW	3B	AX.89729166	7.22E+08	0.38	-0.95	3.16	0.06
E2	GY	3B	AX.89629664	5.32E+08	0.09	-4.23	4.63	0.06
E2	GY	3B	AX.89434921	7.48E+08	0.11	-3.25	3.38	0.04
E2	GY	3B	AX.89708404	7.49E+08	0.11	-3.25	3.38	0.04
E2	GY	3B	AX.89469689	4.97E+08	0.42	-1.93	3.05	0.05
E2	GY	3B	AX.89469689	4.97E+08	0.42	-1.93	3.05	0.05
E2	GY	3B	AX.89564444	7.49E+08	0.11	-3.25	3.38	0.04
E2	GY	3B	AX.89564444	7.49E+08	0.11	-3.25	3.38	0.04
E2	GY	3B	AX.89459177	5.02E+08	0.48	-1.90	3.06	0.06
E2	GY	3B	AX.89366640	2.51E+07	0.23	-2.45	3.61	0.06
E2	GPM2	3B	AX.89643610	8.19E+08	0.25	565.18	3.09	0.04
E2	GY	3B	AX.89438012	7.83E+08	0.15	-2.98	3.69	0.05
E2	GY	3B	AX.89438012	7.83E+08	0.15	-2.98	3.69	0.05
E2	GY	3B	AX.89577476	7.47E+08	0.11	-3.25	3.38	0.04
E2	GY	3B	AX.89714336	7.47E+08	0.11	-3.25	3.38	0.04
E2	GY	3B	AX.89776584	7.49E+08	0.11	-3.25	3.38	0.04
E2	GY	3B	AX.89776584	7.49E+08	0.11	-3.25	3.38	0.04
E2	GY	3B	AX.89639311	4.98E+08	0.43	-2.01	3.31	0.05
E2	GY	3B	AX.89512828	7.83E+08	0.15	-2.86	3.42	0.05
E2	TKW	3B	AX.89740357	7.24E+08	0.35	-0.95	3.07	0.06
E2	GY	3B	AX.89753272	7.48E+08	0.11	-3.25	3.38	0.04
E2	GY	3B	AX.89371943	4.99E+08	0.43	-1.92	3.05	0.05
E2	TKW	3B	AX.89317067	7.23E+08	0.35	-0.95	3.07	0.06
E2	GY	3B	AX.89482510	7.48E+08	0.11	-3.25	3.38	0.04
E2	GY	3B	AX.89482510	7.48E+08	0.11	-3.25	3.38	0.04
E2	GY	3B	AX.89443876	7.62E+08	0.07	-4.04	3.56	0.04
E2	GPS	3B	AX.89660660	7.71E+08	0.23	1.83	3.94	0.06
E2	GY	3B	AX.89621430	2.49E+07	0.11	-4.40	5.80	0.08
E2	GY	3B	AX.89621430	2.49E+07	0.11	-4.40	5.80	0.08
E2	GY	3B	AX.89645874	7.50E+08	0.10	-3.29	3.32	0.04
E2	GY	3B	AX.89629664	5.32E+08	0.09	-4.23	4.63	0.06
E2	GY	3B	AX.89696399	7.50E+08	0.11	-3.25	3.38	0.04
E2	GY	3B	AX.89696399	7.50E+08	0.11	-3.25	3.38	0.04
E2	GY	3B	AX.89414707	7.50E+08	0.09	-3.29	3.11	0.04
E2	GY	3B	AX.89414707	7.50E+08	0.09	-3.29	3.11	0.04
E2	GY	3B	AX.89565303	7.48E+08	0.11	-3.25	3.38	0.04
E2	GY	3B	AX.89483525	7.47E+08	0.11	-3.25	3.38	0.04
E2	GY	3B	AX.89483525	7.47E+08	0.11	-3.25	3.38	0.04
E2	TKW	3B	AX.89603356	7.83E+08	0.21	-1.23	3.60	0.07
E2	GY	3B	AX.89639311	4.98E+08	0.43	-2.01	3.31	0.05
E2	TKW	3B	AX.89645549	7.21E+08	0.38	-0.93	3.04	0.06
E2	GY	3B	AX.89662516	7.50E+08	0.11	-3.25	3.38	0.04
E2	GY	3B	AX.89662516	7.50E+08	0.11	-3.25	3.38	0.04
E2	GY	3B	AX.89645874	7.50E+08	0.10	-3.29	3.32	0.04
E2	GY	3B	AX.89434921	7.48E+08	0.11	-3.25	3.38	0.04
E2	TKW	3B	AX.89481743	7.24E+08	0.35	-0.95	3.07	0.06
E2	GY	3B	AX.89729251	7.50E+08	0.11	-3.25	3.38	0.04

E2	GY	3B	AX.89601168	7.48E+08	0.11	-3.25	3.38	0.04
E2	GY	3B	AX.89565303	7.48E+08	0.11	-3.25	3.38	0.04
E2	GPS	3B	AX.89485134	2.46E+07	0.18	-1.74	3.15	0.05
E2	TKW	3B	AX.89356626	7.24E+08	0.35	-0.95	3.07	0.06
E2	GY	3B	AX.89443876	7.62E+08	0.07	-4.04	3.56	0.04
E2	GY	3B	AX.89702791	7.48E+08	0.11	-3.25	3.38	0.04
E2	TKW	3B	AX.89462566	7.21E+08	0.17	-1.31	3.49	0.06
E2	TKW	3B	AX.89704746	7.21E+08	0.36	-0.94	3.02	0.06
E2	GY	3B	AX.89585986	7.83E+08	0.15	-2.86	3.42	0.05
E2	TKW	3B	AX.89575999	7.21E+08	0.38	-0.93	3.04	0.06
E2	GPS	3B	AX.89628799	2.46E+07	0.18	-1.74	3.15	0.05
E2	TKW	3B	AX.89520460	7.23E+08	0.34	-0.99	3.23	0.06
E2	GY	3B	AX.89601168	7.48E+08	0.11	-3.25	3.38	0.04
E2	TKW	3B	AX.89353779	7.24E+08	0.35	-0.95	3.07	0.06
E2	GY	3B	AX.89643174	7.50E+08	0.11	-3.25	3.38	0.04
E2	GY	3B	AX.89643174	7.50E+08	0.11	-3.25	3.38	0.04
E2	TKW	3B	AX.89359805	7.24E+08	0.36	-0.94	3.01	0.06
E2	TKW	3B	AX.89651138	7.24E+08	0.35	-0.95	3.07	0.06
E2	TKW	3B	AX.89635474	7.24E+08	0.35	-0.95	3.07	0.06
E2	TKW	3B	AX.89702899	7.21E+08	0.38	-0.93	3.04	0.06
E2	GY	3B	AX.89585986	7.83E+08	0.15	-2.86	3.42	0.05
E2	GY	3B	AX.89558408	7.50E+08	0.11	-3.25	3.38	0.04
E2	GY	3B	AX.89753272	7.48E+08	0.11	-3.25	3.38	0.04
E2	GY	3B	AX.89729251	7.50E+08	0.11	-3.25	3.38	0.04
E2	TKW	3B	AX.89313612	6.24E+07	0.28	1.02	3.07	0.05
E2	GY	3B	AX.89558408	7.50E+08	0.11	-3.25	3.38	0.04
E2	TKW	3B	AX.89726557	7.24E+08	0.35	-0.95	3.07	0.06
E2	GPM2	3B	AX.89716122	7.01E+08	0.05	-1337.53	4.01	0.08
E2	TKW	3B	AX.89683955	7.24E+08	0.35	-0.95	3.07	0.06
E2	GPM2	3B	AX.89742555	4.74E+06	0.17	-641.04	3.04	0.05
E2	GY	3B	AX.89702791	7.48E+08	0.11	-3.25	3.38	0.04
E2	TKW	3B	AX.89548163	7.21E+08	0.38	-0.93	3.04	0.06
E2	TKW	3B	AX.89433207	7.21E+08	0.38	-0.93	3.04	0.06
E2	TKW	3B	AX.89723273	6.34E+07	0.27	1.03	3.08	0.05
E2	TKW	3B	AX.89686982	8.20E+08	0.08	-1.68	3.07	0.05
E2	TKW	3B	AX.89754895	7.24E+08	0.35	-0.95	3.07	0.06
E2	TKW	3B	AX.89602809	8.19E+08	0.08	-1.68	3.07	0.05
E2	TKW	3B	AX.89330964	8.19E+08	0.08	-1.68	3.07	0.05
E2	TKW	3B	AX.89694757	7.24E+08	0.35	-0.95	3.07	0.06
E2	GPS	3B	AX.89351074	2.47E+07	0.18	-1.74	3.15	0.05
E3	GPM2	3B	AX.89361709	2.49E+07	0.38	945.15	3.24	0.10
E3	GPM2	3B	AX.89450369	2.52E+07	0.42	855.49	3.30	0.09
E3	GPM2	3B	AX.89721932	2.52E+07	0.42	868.17	3.27	0.10
E3	GPM2	3B	AX.89500326	2.52E+07	0.25	1260.46	4.72	0.14
E3	GPM2	3B	AX.89398704	2.52E+07	0.36	944.86	3.72	0.12
E3	GPM2	3B	AX.89340298	2.52E+07	0.30	987.19	3.58	0.12
E3	GPM2	3B	AX.89332002	2.53E+07	0.30	987.19	3.58	0.12
E3	GPM2	3B	AX.89746790	2.53E+07	0.30	987.19	3.58	0.12
E3	GPM2	3B	AX.89745105	2.66E+07	0.36	918.83	3.35	0.10
E3	GPM2	3B	AX.89638378	2.89E+07	0.39	925.88	3.44	0.10

E3	GPM2	3B	AX.89564222	7.62E+08	0.45	-896.75	3.42	0.11
E3	GPM2	3B	AX.89703941	7.62E+08	0.45	-896.75	3.42	0.11
E3	GPM2	3B	AX.89328013	7.62E+08	0.49	-844.85	3.23	0.07
E3	GPM2	3B	AX.89518796	7.62E+08	0.45	-882.96	3.34	0.11
E3	GPM2	3B	AX.89468143	7.62E+08	0.45	-874.71	3.26	0.10
E3	GPM2	3B	AX.89456880	7.62E+08	0.45	-882.96	3.34	0.11
E3	GPM2	3B	AX.89470996	7.62E+08	0.45	-882.96	3.34	0.11
E3	GPM2	3B	AX.89651315	7.62E+08	0.45	-882.96	3.34	0.11
E3	GPM2	3B	AX.89398698	7.62E+08	0.45	-874.71	3.26	0.10
E3	GPM2	3B	AX.89727081	7.62E+08	0.45	-881.02	3.29	0.10
E3	GPM2	3B	AX.89378355	7.62E+08	0.45	-874.71	3.26	0.10
E3	GPM2	3B	AX.89471736	7.62E+08	0.30	-974.59	3.39	0.12
E3	GPS	3B	AX.89669180	1.93E+07	0.35	2.25	3.50	0.11
E3	GPS	3B	AX.89748259	7.61E+08	0.52	1.91	3.02	0.06
E3	GPS	3B	AX.89325043	7.61E+08	0.52	1.91	3.02	0.06
E3	GPS	3B	AX.89481321	7.61E+08	0.39	2.00	3.17	0.09
E3	GPS	3B	AX.89403171	7.61E+08	0.52	1.96	3.19	0.06
E3	GPS	3B	AX.89566715	7.61E+08	0.39	2.00	3.17	0.09
E3	GPS	3B	AX.89712629	7.61E+08	0.42	1.92	3.02	0.08
E3	GPS	3B	AX.89595359	7.61E+08	0.46	-2.01	3.30	0.05
E3	GPS	3B	AX.89362933	7.61E+08	0.46	-2.01	3.30	0.05
E3	GPS	3B	AX.89725883	7.61E+08	0.47	-2.02	3.35	0.05
E3	GPS	3B	AX.89633568	7.61E+08	0.46	-1.98	3.20	0.05
E3	GPS	3B	AX.89414048	7.61E+08	0.46	-2.01	3.30	0.05
E3	GPS	3B	AX.89313664	7.61E+08	0.46	-2.01	3.30	0.05
E3	GPS	3B	AX.89325522	7.61E+08	0.46	-1.95	3.13	0.05
E3	GPS	3B	AX.89475072	7.61E+08	0.46	-1.95	3.13	0.05
E3	GPS	3B	AX.89634496	7.61E+08	0.45	-1.96	3.15	0.05
E3	GPS	3B	AX.89754938	7.61E+08	0.46	-1.95	3.13	0.05
E3	GPS	3B	AX.89514120	7.61E+08	0.46	-1.98	3.25	0.05
E3	GPS	3B	AX.89469013	7.61E+08	0.45	-1.93	3.08	0.05
E3	GPS	3B	AX.89763353	7.61E+08	0.46	-2.01	3.30	0.05
E3	GPS	3B	AX.89500846	7.61E+08	0.45	-1.96	3.15	0.05
E3	GPS	3B	AX.89513252	7.63E+08	0.30	-2.11	3.11	0.03
E3	GPS	3B	AX.89530132	7.63E+08	0.31	-2.07	3.05	0.03
E3	GPS	3B	AX.89591847	8.17E+08	0.23	2.65	3.80	0.09
E3	GY	3B	AX.89732128	2.52E+07	0.31	-3.60	3.88	0.04
E3	GY	3B	AX.89340298	2.52E+07	0.30	3.92	4.80	0.12
E3	GY	3B	AX.89332002	2.53E+07	0.30	3.92	4.80	0.12
E3	GY	3B	AX.89746790	2.53E+07	0.30	3.92	4.80	0.12
E3	GY	3B	AX.89476808	2.53E+07	0.30	-3.43	3.58	0.04
E3	GY	3B	AX.89638852	2.54E+07	0.32	-3.19	3.19	0.03
E3	GY	3B	AX.89589895	2.66E+07	0.27	3.36	3.16	0.10
E3	GY	3B	AX.89446080	2.66E+07	0.26	3.47	3.32	0.10
E3	GY	3B	AX.89571512	2.66E+07	0.26	4.06	4.24	0.12
E3	GY	3B	AX.89564222	7.62E+08	0.45	-2.92	3.24	0.07
E3	GY	3B	AX.89703941	7.62E+08	0.45	-2.92	3.24	0.07
E3	GY	3B	AX.89518796	7.62E+08	0.45	-2.85	3.13	0.07
E3	GY	3B	AX.89468143	7.62E+08	0.45	-2.80	3.01	0.06
E3	GY	3B	AX.89349219	7.62E+08	0.40	2.76	3.07	0.03

E3	GY	3B	AX.89456880	7.62E+08	0.45	-2.85	3.13	0.07
E3	GY	3B	AX.89470996	7.62E+08	0.45	-2.85	3.13	0.07
E3	GY	3B	AX.89651315	7.62E+08	0.45	-2.85	3.13	0.07
E3	GY	3B	AX.89398698	7.62E+08	0.45	-2.80	3.01	0.06
E3	GY	3B	AX.89378355	7.62E+08	0.45	-2.80	3.01	0.06
E3	GY	3B	X.89462720_OT	1.87E+07	0.15	4.08	3.11	0.09
E3	GY	3B	AX.89361709	2.49E+07	0.38	3.12	3.16	0.08
E3	GY	3B	X.89357555_OT	2.51E+07	0.32	3.17	3.17	0.10
E3	GY	3B	AX.89312638	2.52E+07	0.31	-3.60	3.88	0.04
E3	GY	3B	AX.89450369	2.52E+07	0.42	3.47	4.57	0.08
E3	GY	3B	AX.89717165	2.52E+07	0.43	3.24	3.95	0.09
E3	GY	3B	AX.89721932	2.52E+07	0.42	3.38	4.23	0.09
E3	GY	3B	AX.89500326	2.52E+07	0.25	3.89	4.09	0.09
E3	GY	3B	AX.89378918	2.52E+07	0.31	-3.10	3.02	0.03
E3	GY	3B	AX.89398704	2.52E+07	0.36	3.39	4.16	0.10
E3	GY	3B	AX.89599748	2.52E+07	0.30	3.55	4.05	0.10
E3	TKW	3B	X.89583616_OT	2.20E+07	0.17	-1.11	3.00	0.04
E3	TKW	3B	X.89603855_OT	2.20E+07	0.16	-1.18	3.29	0.05
E3	SPM2	3B	AX.89312296	7.85E+08	0.07	40.99	3.61	0.08
E3	SPM2	3B	AX.89495829	7.86E+08	0.08	39.79	3.90	0.07
E4	GPM2	3B	AX.89375902	1.60E+06	0.06	-957.29	3.11	0.06
E4	GPM2	3B	AX.89414398	2.32E+06	0.25	-524.12	3.27	0.03
E4	GPM2	3B	AX.89343438	2.05E+07	0.43	436.98	3.08	0.03
E4	GPM2	3B	AX.89551197	2.05E+07	0.42	494.56	3.80	0.03
E4	GPM2	3B	AX.89346438	2.07E+07	0.42	-553.96	4.27	0.06
E4	GPM2	3B	AX.89511603	2.07E+07	0.48	444.96	3.10	0.04
E4	GPM2	3B	AX.89349060	3.98E+07	0.06	-1017.23	3.21	0.05
E4	GPM2	3B	X.89514602_OT	5.18E+07	0.09	-809.79	3.67	0.05
E4	GPM2	3B	X.89330274_OT	5.18E+07	0.10	-822.20	3.97	0.05
E4	GPM2	3B	X.89583868_OT	5.19E+07	0.10	-811.51	3.90	0.05
E4	GPM2	3B	AX.89418290	5.19E+07	0.10	-743.74	3.46	0.05
E4	GPM2	3B	X.89326997_OT	5.19E+07	0.09	-870.17	4.28	0.06
E4	GPM2	3B	X.89417732_OT	5.19E+07	0.10	-822.20	3.97	0.05
E4	GPM2	3B	X.89765198_OT	5.20E+07	0.08	-799.03	3.25	0.04
E4	GPM2	3B	AX.89316463	5.28E+07	0.09	-852.02	4.17	0.06
E4	GPM2	3B	AX.89742018	6.28E+08	0.14	-627.69	3.06	0.04
E4	GPM2	3B	AX.89767504	6.29E+08	0.14	-627.69	3.06	0.04
E4	GPM2	3B	X.89364843_OT	5.19E+07	0.10	-725.51	3.26	0.04
E4	GPM2	3B	AX.89440384	6.29E+08	0.15	-645.08	3.25	0.04
E4	GPM2	3B	AX.89747109	7.32E+08	0.42	468.89	3.38	0.03
E4	GPS	3B	AX.89619803	2.37E+07	0.17	2.46	4.43	0.10
E4	GPS	3B	AX.89545348	2.37E+07	0.12	2.90	4.59	0.10
E4	GPS	3B	AX.89742676	6.65E+08	0.22	1.85	3.05	0.07
E4	GY	3B	AX.89551197	2.05E+07	0.42	1.48	3.08	0.03
E4	GY	3B	AX.89346438	2.07E+07	0.41	-2.00	4.93	0.07
E4	GY	3B	AX.89511603	2.07E+07	0.48	1.55	3.33	0.05
E4	GY	3B	AX.89349060	3.98E+07	0.06	-3.30	3.20	0.05
E4	GY	3B	AX.89417490	4.51E+07	0.45	1.52	3.05	0.03
E4	SPM2	3B	AX.89328925	7.31E+08	0.44	16.94	3.28	0.03
E4	TKW	3B	AX.89500326	2.52E+07	0.25	-1.02	3.39	0.10

E4	TKW	3B	AX.89591847	8.17E+08	0.23	-1.05	3.58	0.04
E1	GY	3D	AX.89402594	1.76E+07	0.18	3.40	3.86	0.07
E1	SPM2	3D	AX.89369772	1.85E+07	0.19	23.16	3.69	0.07
E1	GY	3D	AX.89489985	5.90E+08	0.47	2.37	3.25	0.06
E1	SPM2	3D	AX.89355409	1.88E+07	0.19	23.95	3.85	0.07
E1	GY	3D	AX.89453199	5.95E+08	0.07	-5.19	3.92	0.06
E1	SPM2	3D	AX.89381301	1.88E+07	0.23	19.32	3.06	0.05
E1	GY	3D	AX.89760480	1.65E+08	0.36	2.39	3.06	0.06
E1	SPM2	3D	AX.89668035	5.97E+08	0.31	-21.77	4.43	0.08
E1	GY	3D	AX.89489985	5.90E+08	0.47	2.37	3.25	0.06
E1	SPM2	3D	AX.89402594	1.76E+07	0.18	22.87	3.50	0.06
E1	GY	3D	AX.89760480	1.65E+08	0.36	2.39	3.06	0.06
E1	GY	3D	AX.89453199	5.95E+08	0.07	-5.19	3.92	0.06
E1	SPM2	3D	AX.89443707	1.85E+07	0.19	23.95	3.85	0.07
E1	GY	3D	AX.89355409	1.88E+07	0.19	3.47	4.07	0.07
E1	GY	3D	AX.89355409	1.88E+07	0.19	3.47	4.07	0.07
E1	SPM2	3D	AX.89610452	1.86E+07	0.19	23.95	3.85	0.07
E1	GY	3D	AX.89433577	1.84E+07	0.18	3.40	3.86	0.07
E1	GY	3D	AX.89354458	5.99E+08	0.20	-2.92	3.21	0.05
E1	GY	3D	AX.89610452	1.86E+07	0.19	3.47	4.07	0.07
E1	SPM2	3D	AX.89433577	1.84E+07	0.18	22.87	3.50	0.06
E1	SPM2	3D	AX.89602134	1.86E+07	0.19	23.95	3.85	0.07
E1	GY	3D	AX.89404185	1.88E+07	0.19	3.48	4.15	0.08
E1	SPM2	3D	AX.89345657	1.85E+07	0.19	23.95	3.85	0.07
E1	SPM2	3D	AX.89441611	1.79E+07	0.18	22.87	3.50	0.06
E1	GY	3D	AX.89602134	1.86E+07	0.19	3.47	4.07	0.07
E1	GY	3D	AX.89621615	1.93E+07	0.13	3.42	3.00	0.05
E1	GY	3D	AX.89610452	1.86E+07	0.19	3.47	4.07	0.07
E1	SPM2	3D	AX.89529257	4.06E+08	0.40	17.59	3.36	0.06
E1	GY	3D	AX.89461788	4.17E+06	0.49	-2.30	3.09	0.05
E1	SPM2	3D	AX.89717369	1.72E+07	0.18	21.14	3.06	0.05
E1	GY	3D	AX.89345657	1.85E+07	0.19	3.47	4.07	0.07
E1	SPM2	3D	AX.89505227	1.75E+07	0.18	22.87	3.50	0.06
E1	GY	3D	AX.89312909	6.45E+07	0.30	-2.80	3.71	0.06
E1	GY	3D	AX.89354458	5.99E+08	0.20	-2.92	3.21	0.05
E1	GY	3D	AX.89402594	1.76E+07	0.18	3.40	3.86	0.07
E1	GY	3D	AX.89369772	1.85E+07	0.19	3.31	3.82	0.07
E1	SPM2	3D	AX.89404185	1.88E+07	0.19	23.98	3.92	0.07
E1	GY	3D	AX.89312909	6.45E+07	0.30	-2.80	3.71	0.06
E1	SPM2	3D	AX.89689278	1.76E+07	0.19	21.31	3.14	0.05
E1	GY	3D	AX.89767270	1.17E+07	0.43	2.29	3.02	0.05
E1	GY	3D	AX.89621615	1.93E+07	0.13	3.42	3.00	0.05
E1	GY	3D	AX.89689278	1.76E+07	0.19	3.42	3.97	0.07
E1	GY	3D	AX.89404185	1.88E+07	0.19	3.48	4.15	0.08
E1	GY	3D	AX.89441611	1.79E+07	0.18	3.40	3.86	0.07
E1	GY	3D	AX.89441611	1.79E+07	0.18	3.40	3.86	0.07
E1	GY	3D	AX.89443707	1.85E+07	0.19	3.47	4.07	0.07
E1	GY	3D	AX.89695674	4.80E+08	0.33	-2.41	3.03	0.05
E1	GY	3D	AX.89602134	1.86E+07	0.19	3.47	4.07	0.07
E1	GY	3D	AX.89505227	1.75E+07	0.18	3.40	3.86	0.07

E1	GY	3D	AX.89505227	1.75E+07	0.18	3.40	3.86	0.07
E1	GY	3D	AX.89712987	1.11E+07	0.40	2.32	3.03	0.04
E1	GY	3D	AX.89433577	1.84E+07	0.18	3.40	3.86	0.07
E1	GY	3D	AX.89689278	1.76E+07	0.19	3.42	3.97	0.07
E1	GPM2	3D	AX.89370178	2.06E+07	0.13	879.03	3.13	0.05
E1	GY	3D	AX.89461788	4.17E+06	0.49	-2.30	3.09	0.05
E1	GPM2	3D	AX.89656257	2.03E+07	0.13	879.03	3.13	0.05
E1	GY	3D	AX.89369772	1.85E+07	0.19	3.31	3.82	0.07
E1	GY	3D	AX.89345657	1.85E+07	0.19	3.47	4.07	0.07
E1	GPM2	3D	AX.89529257	4.06E+08	0.40	618.51	3.25	0.05
E1	GPM2	3D	AX.89461788	4.17E+06	0.49	-607.17	3.27	0.05
E1	GPM2	3D	AX.89467529	2.27E+07	0.13	879.03	3.13	0.05
E1	GY	3D	AX.89767270	1.17E+07	0.43	2.29	3.02	0.05
E1	GPM2	3D	AX.89417870	2.03E+07	0.13	879.03	3.13	0.05
E1	GPM2	3D	AX.89548359	2.29E+07	0.13	879.03	3.13	0.05
E1	GPM2	3D	AX.89760480	1.65E+08	0.36	631.23	3.25	0.06
E1	GPM2	3D	AX.89754856	5.67E+08	0.33	630.99	3.16	0.06
E1	GPM2	3D	AX.89344287	4.55E+07	0.47	-604.96	3.23	0.06
E1	GPM2	3D	AX.89703410	2.22E+07	0.13	879.03	3.13	0.05
E1	GPM2	3D	AX.89610832	6.43E+07	0.27	-705.77	3.42	0.06
E1	GPM2	3D	AX.89312909	6.45E+07	0.30	-850.88	5.04	0.09
E1	GY	3D	AX.89712987	1.11E+07	0.40	2.32	3.03	0.04
E1	GY	3D	AX.89443707	1.85E+07	0.19	3.47	4.07	0.07
E1	GPM2	3D	AX.89520717	2.27E+07	0.13	879.03	3.13	0.05
E1	GPM2	3D	AX.89336389	6.75E+07	0.16	-941.61	4.13	0.07
E1	GPM2	3D	AX.89572280	2.27E+07	0.13	879.03	3.13	0.05
E1	GY	3D	AX.89695674	4.80E+08	0.33	-2.41	3.03	0.05
E1	TKW	3D	AX.89600888	4.49E+06	0.25	0.96	3.01	0.05
E1	TKW	3D	AX.89315294	6.06E+06	0.22	1.20	4.15	0.07
E1	TKW	3D	AX.89515175	5.92E+08	0.47	-0.93	3.66	0.07
E1	TKW	3D	AX.89600728	4.47E+06	0.25	1.01	3.26	0.06
E1	TKW	3D	AX.89422652	5.57E+05	0.32	1.12	4.51	0.08
E1	TKW	3D	AX.89447134	4.50E+06	0.23	1.04	3.27	0.06
E1	TKW	3D	AX.89457308	4.18E+06	0.27	0.94	3.04	0.05
E1	TKW	3D	AX.89607970	3.98E+07	0.17	1.20	3.49	0.06
E1	TKW	3D	AX.89771060	4.08E+06	0.41	0.86	3.14	0.05
E1	TKW	3D	AX.89605534	3.94E+07	0.24	1.05	3.45	0.06
E1	TKW	3D	AX.89338618	4.06E+06	0.41	0.95	3.72	0.07
E1	GPM2	3D	AX.89775875	6.66E+07	0.15	-948.98	3.93	0.07
E1	TKW	3D	AX.89518432	3.95E+05	0.34	0.90	3.18	0.06
E1	TKW	3D	AX.89520380	4.04E+06	0.40	0.85	3.04	0.05
E1	GPM2	3D	AX.89394916	2.06E+07	0.13	879.03	3.13	0.05
E1	TKW	3D	AX.89414234	4.47E+06	0.33	1.16	4.78	0.09
E1	TKW	3D	AX.89431512	4.08E+06	0.49	0.84	3.08	0.05
E1	TKW	3D	AX.89460086	5.92E+08	0.48	-0.91	3.54	0.06
E1	TKW	3D	AX.89481773	4.06E+06	0.27	0.96	3.18	0.05
E1	GPM2	3D	AX.89519943	2.06E+07	0.13	879.03	3.13	0.05
E1	TKW	3D	AX.89624233	4.08E+06	0.28	0.92	3.03	0.05
E1	TKW	3D	AX.89749986	4.06E+06	0.20	1.14	3.59	0.06
E1	TKW	3D	AX.89366027	4.06E+06	0.21	1.10	3.47	0.06

E1	TKW	3D	AX.89353117	4.11E+06	0.47	0.89	3.36	0.06
E1	TKW	3D	AX.89363224	4.10E+07	0.21	1.08	3.31	0.06
E1	TKW	3D	AX.89318435	4.18E+06	0.18	1.10	3.09	0.05
E1	TKW	3D	AX.89622600	5.93E+05	0.39	1.00	3.96	0.07
E1	TKW	3D	AX.89312605	5.59E+05	0.27	0.98	3.23	0.06
E1	TKW	3D	AX.89598076	5.26E+05	0.27	1.00	3.33	0.06
E1	TKW	3D	AX.89398494	4.20E+06	0.27	0.97	3.19	0.05
E1	TKW	3D	AX.89570474	2.35E+06	0.16	1.16	3.18	0.05
E1	TKW	3D	AX.89770522	4.29E+06	0.27	0.95	3.11	0.05
E1	TKW	3D	AX.89669031	4.18E+06	0.27	0.95	3.10	0.05
E1	TKW	3D	AX.89366271	3.68E+05	0.18	1.20	3.64	0.06
E2	SPM2	3D	AX.89389704	1.74E+07	0.19	19.74	4.93	0.09
E2	SPM2	3D	AX.89410415	1.72E+07	0.19	19.74	4.93	0.09
E2	SPM2	3D	AX.89476748	1.76E+07	0.22	19.39	5.39	0.10
E2	SPM2	3D	AX.89453646	1.71E+07	0.19	19.74	4.93	0.09
E2	SPM2	3D	AX.89531997	1.74E+07	0.23	18.32	4.94	0.09
E2	SPM2	3D	AX.89711565	1.71E+07	0.09	19.96	3.03	0.05
E2	SPM2	3D	AX.89717369	1.72E+07	0.18	20.00	4.96	0.09
E2	SPM2	3D	AX.89708280	1.71E+07	0.23	18.32	4.94	0.09
E2	SPM2	3D	AX.89567994	6.04E+08	0.07	23.01	3.24	0.05
E2	SPM2	3D	AX.89493070	1.72E+07	0.23	18.32	4.94	0.09
E2	SPM2	3D	AX.89433577	1.84E+07	0.18	21.06	5.44	0.10
E2	SPM2	3D	AX.89322424	1.71E+07	0.19	19.74	4.93	0.09
E2	SPM2	3D	AX.89615509	1.77E+07	0.22	19.39	5.39	0.10
E2	SPM2	3D	AX.89443707	1.85E+07	0.19	20.31	5.19	0.09
E2	SPM2	3D	AX.89507597	1.71E+07	0.23	18.32	4.94	0.09
E2	SPM2	3D	AX.89309755	1.72E+07	0.23	18.32	4.94	0.09
E2	SPM2	3D	AX.89610452	1.86E+07	0.19	20.31	5.19	0.09
E2	SPM2	3D	AX.89694304	1.72E+07	0.23	18.32	4.94	0.09
E2	SPM2	3D	AX.89335774	1.87E+07	0.22	19.67	5.46	0.10
E2	SPM2	3D	AX.89404185	1.88E+07	0.19	19.15	4.75	0.08
E2	SPM2	3D	AX.89602134	1.86E+07	0.19	20.31	5.19	0.09
E2	SPM2	3D	AX.89402594	1.76E+07	0.18	21.06	5.44	0.10
E2	SPM2	3D	AX.89687725	1.84E+07	0.23	18.82	5.18	0.09
E2	SPM2	3D	AX.89320829	1.74E+07	0.23	18.32	4.94	0.09
E2	SPM2	3D	AX.89505227	1.75E+07	0.18	21.06	5.44	0.10
E2	SPM2	3D	AX.89660803	1.72E+07	0.23	18.32	4.94	0.09
E2	SPM2	3D	AX.89517416	1.71E+07	0.23	18.32	4.94	0.09
E2	SPM2	3D	AX.89544588	1.74E+07	0.23	18.32	4.94	0.09
E2	SPM2	3D	AX.89405175	1.74E+07	0.23	18.32	4.94	0.09
E2	SPM2	3D	AX.89573416	1.71E+07	0.23	18.32	4.94	0.09
E2	SPM2	3D	AX.89763668	1.86E+07	0.23	18.82	5.18	0.09
E2	SPM2	3D	AX.89619764	1.71E+07	0.21	20.17	5.63	0.10
E2	SPM2	3D	AX.89544986	1.73E+07	0.19	19.74	4.93	0.09
E2	SPM2	3D	AX.89616014	1.72E+07	0.23	18.32	4.94	0.09
E2	SPM2	3D	AX.89733349	1.72E+07	0.23	18.32	4.94	0.09
E2	SPM2	3D	AX.89432654	1.72E+07	0.23	18.32	4.94	0.09
E2	SPM2	3D	AX.89533054	1.73E+07	0.23	18.32	4.94	0.09
E2	SPM2	3D	AX.89693910	1.73E+07	0.22	18.45	4.93	0.09
E2	SPM2	3D	AX.89345657	1.85E+07	0.19	20.31	5.19	0.09

E2	SPM2	3D	AX.89370256	1.84E+07	0.22	19.39	5.39	0.10
E2	SPM2	3D	AX.89661787	1.88E+07	0.23	18.82	5.18	0.09
E2	SPM2	3D	AX.89520276	1.71E+07	0.23	18.32	4.94	0.09
E2	SPM2	3D	AX.89403471	1.72E+07	0.19	19.74	4.93	0.09
E2	SPM2	3D	AX.89683342	1.71E+07	0.09	19.96	3.03	0.05
E2	SPM2	3D	AX.89323609	1.72E+07	0.20	19.30	4.90	0.09
E2	SPM2	3D	AX.89685514	1.88E+07	0.22	19.39	5.39	0.10
E2	SPM2	3D	AX.89435498	1.72E+07	0.23	18.32	4.94	0.09
E2	SPM2	3D	AX.89407278	1.74E+07	0.23	18.32	4.94	0.09
E2	SPM2	3D	AX.89490994	1.72E+07	0.19	19.74	4.93	0.09
E2	SPM2	3D	AX.89541216	6.03E+08	0.10	19.23	3.05	0.05
E2	SPM2	3D	AX.89665951	1.71E+07	0.23	18.32	4.94	0.09
E2	SPM2	3D	AX.89369772	1.85E+07	0.19	19.66	4.98	0.09
E2	SPM2	3D	AX.89538960	1.73E+07	0.19	19.74	4.93	0.09
E2	SPM2	3D	AX.89370258	1.74E+07	0.23	18.32	4.94	0.09
E2	SPM2	3D	AX.89591091	1.71E+07	0.23	18.32	4.94	0.09
E2	SPM2	3D	AX.89438456	1.74E+07	0.23	18.32	4.94	0.09
E2	SPM2	3D	AX.89503629	1.71E+07	0.09	21.45	3.29	0.06
E2	SPM2	3D	AX.89661843	1.72E+07	0.19	19.74	4.93	0.09
E2	SPM2	3D	AX.89414746	1.70E+07	0.10	21.51	3.57	0.06
E2	SPM2	3D	AX.89381301	1.88E+07	0.23	17.88	4.79	0.09
E2	SPM2	3D	AX.89317317	1.71E+07	0.23	18.32	4.94	0.09
E2	SPM2	3D	AX.89773365	1.71E+07	0.23	18.32	4.94	0.09
E2	SPM2	3D	AX.89603618	1.74E+07	0.23	18.32	4.94	0.09
E2	SPM2	3D	AX.89428216	1.71E+07	0.23	18.32	4.94	0.09
E2	SPM2	3D	AX.89595636	1.85E+07	0.23	18.82	5.18	0.09
E2	SPM2	3D	AX.89441611	1.79E+07	0.18	21.06	5.44	0.10
E2	SPM2	3D	AX.89768223	1.71E+07	0.19	19.74	4.93	0.09
E2	SPM2	3D	AX.89626829	1.73E+07	0.18	19.95	4.94	0.09
E2	SPM2	3D	AX.89756706	1.71E+07	0.09	19.96	3.03	0.05
E2	SPM2	3D	AX.89355409	1.88E+07	0.19	20.31	5.19	0.09
E2	SPM2	3D	AX.89689278	1.76E+07	0.19	20.11	5.10	0.09
E2	SPM2	3D	AX.89480281	1.76E+07	0.22	19.39	5.39	0.10
E2	SPM2	3D	AX.89745957	1.73E+07	0.19	19.74	4.93	0.09
E2	GPS	3D	AX.89661843	1.72E+07	0.18	-1.74	3.13	0.05
E2	GPS	3D	AX.89389704	1.74E+07	0.18	-1.74	3.13	0.05
E2	GPS	3D	AX.89490994	1.72E+07	0.18	-1.74	3.13	0.05
E2	GPS	3D	AX.89512995	6.13E+08	0.46	1.41	3.39	0.06
E2	GPM2	3D	AX.89618585	4.07E+06	0.28	-531.93	3.02	0.05
E2	GPS	3D	AX.89544986	1.73E+07	0.18	-1.74	3.13	0.05
E2	GPS	3D	AX.89453646	1.71E+07	0.18	-1.74	3.13	0.05
E2	GPS	3D	AX.89410415	1.72E+07	0.18	-1.74	3.13	0.05
E2	GPS	3D	AX.89717369	1.72E+07	0.18	-1.76	3.14	0.05
E2	GPS	3D	AX.89745957	1.73E+07	0.18	-1.74	3.13	0.05
E2	GPS	3D	AX.89768223	1.71E+07	0.18	-1.74	3.13	0.05
E2	GPS	3D	AX.89626829	1.73E+07	0.18	-1.80	3.27	0.05
E2	TKW	3D	AX.89460086	5.92E+08	0.48	-1.02	3.53	0.07
E2	GPS	3D	AX.89538960	1.73E+07	0.18	-1.74	3.13	0.05
E2	TKW	3D	AX.89515175	5.92E+08	0.47	-1.02	3.51	0.07
E2	GPS	3D	AX.89322424	1.71E+07	0.18	-1.74	3.13	0.05

E2	GPS	3D	AX.89403471	1.72E+07	0.18	-1.74	3.13	0.05
E2	GPM2	3D	AX.89624233	4.08E+06	0.28	-551.62	3.22	0.05
E3	TKW	3D	AX.89442683	2.45E+06	0.45	1.13	3.64	0.01
E3	TKW	3D	AX.89362642	7.22E+06	0.16	-1.29	3.16	0.04
E3	TKW	3D	AX.89617227	2.34E+07	0.06	1.96	4.13	0.09
E3	TKW	3D	AX.89716925	2.42E+07	0.06	1.86	3.43	0.07
E3	TKW	3D	AX.89747401	2.45E+07	0.06	1.99	3.99	0.07
E3	TKW	3D	AX.89478192	2.47E+07	0.06	1.99	3.99	0.07
E3	TKW	3D	AX.89343882	2.62E+07	0.07	1.90	4.09	0.08
E3	GY	3D	AX.89736351	1.83E+07	0.09	-4.86	3.23	0.01
E4	GPM2	3D	AX.89617227	2.34E+07	0.06	-892.56	3.17	0.06
E4	GPM2	3D	AX.89716925	2.42E+07	0.06	-953.35	3.24	0.06
E4	GPM2	3D	AX.89747401	2.45E+07	0.06	-898.99	3.05	0.05
E4	GPM2	3D	AX.89478192	2.47E+07	0.06	-898.99	3.05	0.05
E4	GPM2	3D	AX.89343882	2.62E+07	0.07	-946.14	3.66	0.06
E4	GPM2	3D	AX.89543114	3.99E+07	0.10	-728.55	3.28	0.07
E4	GPM2	3D	AX.89640561	6.03E+08	0.20	-554.16	3.08	0.07
E4	GPM2	3D	AX.89501088	6.04E+08	0.20	-566.13	3.17	0.07
E4	GPM2	3D	AX.89645710	6.04E+08	0.20	-540.16	3.02	0.06
E4	GPM2	3D	AX.89329033	6.04E+08	0.20	-543.57	3.05	0.07
E4	GPM2	3D	AX.89363126	6.04E+08	0.20	-541.51	3.01	0.06
E4	SPM2	3D	AX.89487272	6.20E+06	0.24	-19.52	3.15	0.05
E4	SPM2	3D	AX.89408613	2.18E+07	0.20	21.17	3.41	0.07
E4	SPM2	3D	X.89561280_OT	5.62E+08	0.09	28.86	3.40	0.05
E4	TKW	3D	AX.89343882	2.62E+07	0.07	1.66	3.30	0.06
E1	GY	4A	AX.89537167	7.39E+08	0.13	-3.32	3.06	0.05
E1	GY	4A	AX.86169711	7.41E+08	0.33	-2.72	3.73	0.05
E1	GY	4A	AX.89406498	6.61E+08	0.10	-3.77	3.10	0.05
E1	GY	4A	AX.89313054	7.23E+08	0.12	-3.61	3.26	0.05
E1	GY	4A	AX.89744180	7.19E+08	0.22	-2.75	3.11	0.05
E1	GY	4A	AX.89314231	7.39E+08	0.16	-3.52	3.79	0.06
E1	GY	4A	AX.89562467	4.09E+07	0.11	-3.73	3.15	0.05
E1	GY	4A	AX.89689789	7.25E+08	0.21	-2.77	3.07	0.04
E1	SPM2	4A	AX.89699897	6.17E+08	0.22	20.43	3.29	0.06
E1	GY	4A	AX.89483630	7.41E+08	0.36	-3.26	5.34	0.08
E1	GY	4A	AX.86163267	7.06E+08	0.16	-3.16	3.15	0.06
E1	GY	4A	AX.86163265	7.06E+08	0.16	-3.16	3.15	0.06
E1	GY	4A	AX.89598281	7.31E+08	0.17	-4.31	5.77	0.09
E1	GY	4A	AX.89586557	7.43E+08	0.20	-3.26	3.96	0.06
E1	GY	4A	AX.89392070	7.10E+08	0.15	-3.31	3.26	0.06
E1	GY	4A	AX.89522316	7.20E+08	0.22	-2.85	3.31	0.06
E1	GY	4A	AX.89618425	7.24E+08	0.12	-3.61	3.26	0.05
E1	GY	4A	AX.89385932	7.27E+08	0.13	-3.56	3.28	0.05
E1	GY	4A	AX.89332213	7.41E+08	0.35	-3.24	5.28	0.08
E1	GY	4A	AX.89346010	7.32E+08	0.26	-3.45	5.05	0.08
E1	GY	4A	AX.89429350	6.61E+08	0.09	-4.03	3.11	0.05
E1	GY	4A	AX.89351503	7.26E+08	0.18	-3.14	3.36	0.05
E1	GY	4A	AX.89640857	7.24E+08	0.12	-3.61	3.26	0.05
E1	GY	4A	AX.89446861	7.33E+08	0.11	-4.31	4.18	0.07
E1	GY	4A	AX.89623044	7.31E+08	0.20	-4.25	6.23	0.10

E1	GY	4A	AX.89346242	7.26E+08	0.18	-3.19	3.51	0.05
E1	GY	4A	AX.86160988	7.42E+08	0.14	-3.81	4.07	0.06
E1	GY	4A	AX.89746072	4.09E+07	0.11	-3.73	3.15	0.05
E1	GY	4A	AX.89313054	7.23E+08	0.12	-3.61	3.26	0.05
E1	SPM2	4A	AX.89366542	6.17E+08	0.16	23.05	3.23	0.06
E1	GY	4A	AX.89537167	7.39E+08	0.13	-3.32	3.06	0.05
E1	SPM2	4A	AX.89380497	6.15E+08	0.35	19.07	3.68	0.07
E1	GY	4A	AX.89339840	7.33E+08	0.22	-3.03	3.67	0.06
E1	GY	4A	AX.89412766	7.37E+08	0.14	-3.43	3.40	0.05
E1	GY	4A	AX.89520254	7.26E+08	0.21	-3.01	3.55	0.05
E1	GY	4A	AX.89353399	7.26E+08	0.20	-2.95	3.27	0.05
E1	GY	4A	AX.89469231	7.30E+08	0.20	-2.88	3.19	0.05
E1	GY	4A	AX.89342370	7.28E+08	0.17	-3.95	4.93	0.08
E1	GY	4A	AX.89423577	7.26E+08	0.13	-3.37	3.15	0.05
E1	SPM2	4A	AX.89623244	6.15E+08	0.35	19.07	3.68	0.07
E1	GY	4A	AX.89670415	7.22E+08	0.17	-3.14	3.25	0.06
E1	GY	4A	AX.89768703	7.40E+08	0.27	-3.09	4.26	0.07
E1	GY	4A	AX.89447138	7.20E+08	0.17	-3.14	3.25	0.06
E1	GY	4A	AX.89367479	7.34E+08	0.22	-2.75	3.12	0.05
E1	GY	4A	AX.89334906	7.30E+08	0.23	-3.46	4.76	0.07
E1	GY	4A	AX.89385882	7.29E+08	0.22	-3.44	4.58	0.07
E1	GY	4A	AX.89310768	7.28E+08	0.21	-3.53	4.68	0.07
E1	GY	4A	AX.89385932	7.27E+08	0.13	-3.56	3.28	0.05
E1	GY	4A	AX.89446643	7.40E+08	0.36	-3.26	5.34	0.08
E1	GY	4A	AX.89322993	7.39E+08	0.15	-3.46	3.52	0.05
E1	GY	4A	AX.89412618	7.34E+08	0.14	-3.62	3.72	0.06
E1	GY	4A	AX.89615154	7.40E+08	0.25	-3.16	4.20	0.06
E1	GY	4A	AX.89322015	7.39E+08	0.11	-4.03	3.60	0.06
E1	GY	4A	AX.89392053	7.22E+08	0.22	-2.94	3.44	0.06
E1	GY	4A	AX.89392070	7.10E+08	0.15	-3.31	3.26	0.06
E1	GY	4A	AX.89446059	7.40E+08	0.16	-3.59	4.00	0.06
E1	GY	4A	AX.89700054	7.25E+08	0.21	-2.77	3.07	0.04
E1	GY	4A	AX.89671253	7.22E+08	0.16	-3.31	3.48	0.06
E1	GY	4A	AX.89432469	7.31E+08	0.20	-3.97	5.60	0.09
E1	GY	4A	AX.89483630	7.41E+08	0.36	-3.26	5.34	0.08
E1	GY	4A	AX.89640857	7.24E+08	0.12	-3.61	3.26	0.05
E1	GY	4A	AX.89353399	7.26E+08	0.20	-2.95	3.27	0.05
E1	GY	4A	AX.89310801	7.27E+08	0.13	-3.56	3.28	0.05
E1	GY	4A	AX.89560573	7.26E+08	0.21	-3.47	4.48	0.07
E1	GY	4A	AX.89314783	7.24E+08	0.21	-2.78	3.06	0.04
E1	GY	4A	AX.86160988	7.42E+08	0.14	-3.81	4.07	0.06
E1	GY	4A	AX.89310426	7.41E+08	0.35	-2.98	4.55	0.07
E1	GY	4A	AX.89746890	7.25E+08	0.07	-5.25	4.29	0.07
E1	GY	4A	AX.89746890	7.25E+08	0.07	-5.25	4.29	0.07
E1	GY	4A	AX.89334906	7.30E+08	0.23	-3.46	4.76	0.07
E1	GY	4A	AX.89346242	7.26E+08	0.18	-3.19	3.51	0.05
E1	GY	4A	AX.89609557	7.26E+08	0.21	-3.35	4.20	0.06
E1	GY	4A	AX.89398534	7.26E+08	0.09	-4.18	3.45	0.05
E1	GY	4A	AX.89414590	7.30E+08	0.17	-3.31	3.56	0.05
E1	GY	4A	AX.89520266	7.26E+08	0.20	-2.95	3.27	0.05

E1	GY	4A	AX.89356388	7.34E+08	0.20	-3.32	4.01	0.06
E1	GY	4A	AX.89629181	7.39E+08	0.16	-3.67	4.16	0.07
E1	SPM2	4A	AX.89606547	2.16E+07	0.35	-17.67	3.25	0.06
E1	GY	4A	AX.89447138	7.20E+08	0.17	-3.14	3.25	0.06
E1	GY	4A	AX.89310426	7.41E+08	0.35	-2.98	4.55	0.07
E1	GY	4A	AX.89602929	7.27E+08	0.20	-2.86	3.06	0.04
E1	GY	4A	AX.89637772	7.21E+08	0.17	-3.14	3.25	0.06
E1	GY	4A	AX.89360038	7.33E+08	0.22	-3.03	3.67	0.06
E1	GY	4A	AX.89586557	7.43E+08	0.20	-3.26	3.96	0.06
E1	GY	4A	AX.89618425	7.24E+08	0.12	-3.61	3.26	0.05
E1	GY	4A	AX.89476973	7.30E+08	0.20	-3.02	3.42	0.05
E1	GY	4A	AX.89459794	7.40E+08	0.14	-3.28	3.16	0.05
E1	GY	4A	AX.89482915	7.04E+08	0.16	-3.16	3.15	0.06
E1	GY	4A	AX.89450482	4.11E+07	0.11	-3.73	3.15	0.05
E1	GY	4A	AX.89338248	7.40E+08	0.31	-3.26	5.00	0.08
E1	GY	4A	AX.89376551	7.22E+08	0.22	-2.77	3.16	0.05
E1	GY	4A	AX.89314783	7.24E+08	0.21	-2.78	3.06	0.04
E1	GY	4A	AX.89369162	7.26E+08	0.20	-2.95	3.27	0.05
E1	GY	4A	AX.89392053	7.22E+08	0.22	-2.94	3.44	0.06
E1	GY	4A	AX.89383862	7.22E+08	0.17	-3.02	3.04	0.05
E1	GY	4A	AX.86163265	7.06E+08	0.16	-3.16	3.15	0.06
E1	GY	4A	AX.89465835	7.41E+08	0.35	-3.11	4.91	0.08
E1	GY	4A	AX.89327236	7.20E+08	0.18	-2.96	3.09	0.05
E1	GY	4A	AX.89396876	7.13E+08	0.20	-3.47	4.28	0.08
E1	GY	4A	AX.89327430	7.26E+08	0.28	-3.45	5.27	0.08
E1	GY	4A	AX.89699271	7.22E+08	0.22	-2.75	3.11	0.05
E1	GY	4A	AX.89377454	7.19E+08	0.13	-3.52	3.39	0.06
E1	GY	4A	AX.89706134	7.42E+08	0.36	-3.26	5.34	0.08
E1	GY	4A	AX.89332213	7.41E+08	0.35	-3.24	5.28	0.08
E1	GY	4A	AX.89520254	7.26E+08	0.21	-3.01	3.55	0.05
E1	GY	4A	AX.89397291	7.34E+08	0.13	-3.69	3.67	0.06
E1	GY	4A	AX.89742680	7.31E+08	0.11	-3.99	3.53	0.05
E1	GY	4A	AX.89579660	7.26E+08	0.11	-3.69	3.19	0.05
E1	GY	4A	AX.89475393	7.28E+08	0.20	-3.47	4.41	0.07
E1	GY	4A	AX.89475393	7.28E+08	0.20	-3.47	4.41	0.07
E1	GY	4A	AX.89426288	6.61E+08	0.10	-3.77	3.10	0.05
E1	GY	4A	AX.89342690	7.26E+08	0.20	-2.95	3.27	0.05
E1	GY	4A	AX.89617069	7.28E+08	0.21	-3.37	4.31	0.07
E1	GY	4A	AX.86163366	7.41E+08	0.32	-2.97	4.32	0.07
E1	GY	4A	AX.89492172	6.17E+08	0.24	-2.63	3.02	0.05
E1	GY	4A	AX.89703275	7.22E+08	0.17	-3.09	3.15	0.06
E1	GY	4A	AX.89609557	7.26E+08	0.21	-3.35	4.20	0.06
E1	GY	4A	AX.89503717	7.10E+08	0.16	-3.16	3.15	0.06
E1	GY	4A	AX.89560573	7.26E+08	0.21	-3.47	4.48	0.07
E1	GY	4A	AX.89327540	7.26E+08	0.20	-2.95	3.27	0.05
E1	GY	4A	AX.89406498	6.61E+08	0.10	-3.77	3.10	0.05
E1	GY	4A	AX.89409546	7.23E+08	0.06	-5.17	3.53	0.06
E1	GY	4A	AX.89398873	7.21E+08	0.05	-5.43	3.36	0.05
E1	GY	4A	AX.89456312	7.20E+08	0.16	-3.14	3.18	0.06
E1	GY	4A	AX.89457592	7.26E+08	0.24	-3.39	4.64	0.07

E1	GY	4A	AX.89682510	7.08E+08	0.15	-3.31	3.26	0.06
E1	GY	4A	AX.89339840	7.33E+08	0.22	-3.03	3.67	0.06
E1	GY	4A	AX.89667062	7.22E+08	0.22	-2.89	3.35	0.06
E1	GY	4A	AX.89375502	7.40E+08	0.17	-3.55	4.08	0.06
E1	GY	4A	AX.89351599	7.30E+08	0.20	-3.47	4.41	0.07
E1	GY	4A	AX.89446059	7.40E+08	0.16	-3.59	4.00	0.06
E1	GY	4A	AX.89539186	7.43E+08	0.19	-3.14	3.54	0.05
E1	GY	4A	AX.89671253	7.22E+08	0.16	-3.31	3.48	0.06
E1	GY	4A	AX.89336177	4.06E+07	0.11	-3.73	3.15	0.05
E1	GY	4A	AX.89769007	7.41E+08	0.33	-2.93	4.29	0.06
E1	GY	4A	AX.89411907	7.27E+08	0.16	-3.43	3.63	0.06
E1	GY	4A	AX.89755912	7.33E+08	0.14	-3.62	3.64	0.06
E1	GY	4A	AX.89468920	7.22E+08	0.17	-3.14	3.25	0.06
E1	GY	4A	AX.89446608	7.25E+08	0.14	-3.51	3.54	0.05
E1	GY	4A	AX.89381828	7.32E+08	0.27	-3.43	5.05	0.08
E1	GY	4A	AX.89450476	7.26E+08	0.11	-4.20	3.87	0.06
E1	GY	4A	AX.89672103	6.02E+08	0.21	-2.94	3.36	0.05
E1	GY	4A	AX.89423359	7.24E+08	0.07	-5.67	4.40	0.07
E1	GY	4A	AX.89387177	7.33E+08	0.20	-2.98	3.39	0.05
E1	GY	4A	AX.89387177	7.33E+08	0.20	-2.98	3.39	0.05
E1	GY	4A	AX.89446861	7.33E+08	0.11	-4.31	4.18	0.07
E1	GY	4A	AX.89376728	7.27E+08	0.22	-3.52	4.72	0.07
E1	GY	4A	AX.89314907	7.22E+08	0.22	-2.94	3.44	0.06
E1	GY	4A	AX.89668236	7.33E+08	0.15	-3.82	4.19	0.07
E1	GY	4A	AX.86166405	7.26E+08	0.20	-2.95	3.27	0.05
E1	GY	4A	AX.89436017	7.25E+08	0.06	-5.45	3.63	0.06
E1	GY	4A	AX.89552078	7.09E+08	0.15	-3.35	3.34	0.06
E1	GY	4A	AX.89530069	7.40E+08	0.17	-3.58	4.06	0.06
E1	GY	4A	AX.89377182	7.27E+08	0.21	-3.53	4.68	0.07
E1	GY	4A	AX.89588174	7.34E+08	0.21	-3.02	3.56	0.05
E1	GY	4A	AX.89746072	4.09E+07	0.11	-3.73	3.15	0.05
E1	GY	4A	AX.89710159	7.24E+08	0.07	-5.27	4.32	0.07
E1	GY	4A	AX.89334174	7.22E+08	0.22	-2.75	3.11	0.05
E1	GY	4A	AX.89377454	7.19E+08	0.13	-3.52	3.39	0.06
E1	GY	4A	AX.89338248	7.40E+08	0.31	-3.26	5.00	0.08
E1	GY	4A	AX.89706134	7.42E+08	0.36	-3.26	5.34	0.08
E1	GY	4A	AX.89397291	7.34E+08	0.13	-3.69	3.67	0.06
E1	GY	4A	AX.89564765	7.39E+08	0.13	-3.60	3.51	0.05
E1	GY	4A	AX.89491474	7.28E+08	0.15	-3.40	3.42	0.05
E1	GY	4A	AX.89361955	7.39E+08	0.15	-3.46	3.52	0.05
E1	GY	4A	AX.89377870	7.24E+08	0.07	-5.67	4.40	0.07
E1	GY	4A	AX.86169711	7.41E+08	0.33	-2.72	3.73	0.05
E1	GY	4A	AX.89342690	7.26E+08	0.20	-2.95	3.27	0.05
E1	GY	4A	AX.89743266	4.09E+07	0.11	-3.73	3.15	0.05
E1	GY	4A	AX.89557030	7.39E+08	0.16	-3.67	4.16	0.07
E1	GY	4A	AX.89735433	7.24E+08	0.12	-3.97	3.74	0.06
E1	GY	4A	AX.89661835	7.33E+08	0.11	-3.63	3.02	0.04
E1	SPM2	4A	AX.89475843	6.17E+08	0.22	20.77	3.34	0.06
E1	GY	4A	AX.89425731	7.06E+08	0.16	-3.16	3.15	0.06
E1	GY	4A	AX.89617589	4.11E+07	0.11	-3.73	3.15	0.05

E1	GY	4A	AX.89456312	7.20E+08	0.16	-3.14	3.18	0.06
E1	GY	4A	AX.89367424	7.22E+08	0.17	-3.00	3.06	0.05
E1	GY	4A	AX.89367479	7.34E+08	0.22	-2.75	3.12	0.05
E1	GY	4A	AX.89367604	7.25E+08	0.18	-3.29	3.71	0.06
E1	GY	4A	AX.89712404	7.20E+08	0.17	-3.02	3.10	0.05
E1	GY	4A	AX.89328992	7.33E+08	0.19	-2.99	3.25	0.05
E1	GY	4A	AX.89735433	7.24E+08	0.12	-3.97	3.74	0.06
E1	GY	4A	AX.89761056	7.30E+08	0.15	-3.40	3.42	0.05
E1	GY	4A	AX.89310768	7.28E+08	0.21	-3.53	4.68	0.07
E1	GY	4A	AX.89310801	7.27E+08	0.13	-3.56	3.28	0.05
E1	GY	4A	AX.89566818	7.24E+08	0.07	-5.67	4.40	0.07
E1	GY	4A	AX.89376272	7.26E+08	0.18	-3.29	3.71	0.06
E1	GY	4A	AX.89380026	7.24E+08	0.12	-3.61	3.26	0.05
E1	GY	4A	AX.89423359	7.24E+08	0.07	-5.67	4.40	0.07
E1	GY	4A	AX.89376551	7.22E+08	0.22	-2.77	3.16	0.05
E1	GY	4A	AX.89517951	7.26E+08	0.20	-2.95	3.27	0.05
E1	GY	4A	AX.89517951	7.26E+08	0.20	-2.95	3.27	0.05
E1	GY	4A	AX.89578493	7.20E+08	0.17	-3.02	3.10	0.05
E1	GY	4A	AX.89615154	7.40E+08	0.25	-3.16	4.20	0.06
E1	GY	4A	AX.89369037	7.34E+08	0.13	-3.78	3.63	0.06
E1	GY	4A	AX.89611401	7.20E+08	0.22	-2.85	3.31	0.06
E1	GY	4A	AX.89532662	7.40E+08	0.27	-3.37	5.01	0.08
E1	GY	4A	AX.89369165	7.22E+08	0.16	-3.21	3.25	0.06
E1	GY	4A	AX.89369165	7.22E+08	0.16	-3.21	3.25	0.06
E1	GY	4A	AX.86166405	7.26E+08	0.20	-2.95	3.27	0.05
E1	GY	4A	AX.89392325	7.24E+08	0.12	-3.61	3.26	0.05
E1	GY	4A	AX.89623044	7.31E+08	0.20	-4.25	6.23	0.10
E1	GY	4A	AX.89615361	7.24E+08	0.06	-5.45	3.63	0.06
E1	GY	4A	AX.89544271	4.09E+07	0.11	-3.73	3.15	0.05
E1	GY	4A	AX.89428619	7.22E+08	0.22	-2.94	3.44	0.06
E1	GY	4A	AX.89726273	7.40E+08	0.35	-3.24	5.28	0.08
E1	GY	4A	AX.89384587	7.31E+08	0.06	-4.71	3.01	0.05
E1	GY	4A	AX.89334174	7.22E+08	0.22	-2.75	3.11	0.05
E1	GY	4A	AX.89680753	7.26E+08	0.14	-3.30	3.10	0.05
E1	GY	4A	AX.89349822	7.39E+08	0.16	-3.11	3.13	0.05
E1	GY	4A	AX.89349822	7.39E+08	0.16	-3.11	3.13	0.05
E1	GY	4A	AX.89568535	7.31E+08	0.27	-3.78	6.02	0.10
E1	GY	4A	AX.89776093	7.31E+08	0.27	-3.78	6.02	0.10
E1	GY	4A	AX.89429350	6.61E+08	0.09	-4.03	3.11	0.05
E1	GY	4A	AX.89377870	7.24E+08	0.07	-5.67	4.40	0.07
E1	GY	4A	AX.89421360	7.22E+08	0.17	-3.14	3.25	0.06
E1	GY	4A	AX.89568764	7.43E+08	0.20	-3.31	4.06	0.06
E1	GY	4A	AX.89334772	4.09E+07	0.11	-3.73	3.15	0.05
E1	GY	4A	AX.89335287	7.34E+08	0.14	-3.62	3.72	0.06
E1	GY	4A	AX.89321025	7.34E+08	0.14	-3.43	3.40	0.05
E1	GY	4A	AX.89328332	7.39E+08	0.11	-4.03	3.60	0.06
E1	GY	4A	AX.89425731	7.06E+08	0.16	-3.16	3.15	0.06
E1	GY	4A	AX.89313341	7.39E+08	0.12	-3.87	3.58	0.05
E1	GY	4A	AX.89406883	7.26E+08	0.18	-3.14	3.36	0.05
E1	GY	4A	AX.89666696	7.43E+08	0.10	-4.28	3.73	0.06

E1	GY	4A	AX.89343635	7.31E+08	0.15	-4.33	5.22	0.08
E1	GY	4A	AX.89343635	7.31E+08	0.15	-4.33	5.22	0.08
E1	GY	4A	AX.89716456	7.29E+08	0.22	-3.44	4.58	0.07
E1	GY	4A	AX.89367424	7.22E+08	0.17	-3.00	3.06	0.05
E1	GY	4A	AX.89654995	7.27E+08	0.07	-4.48	3.26	0.05
E1	GY	4A	AX.89712404	7.20E+08	0.17	-3.02	3.10	0.05
E1	GY	4A	AX.89602929	7.27E+08	0.20	-2.86	3.06	0.04
E1	GY	4A	AX.89690364	7.40E+08	0.36	-3.26	5.34	0.08
E1	GY	4A	AX.89744514	7.20E+08	0.21	-3.10	3.67	0.06
E1	GY	4A	AX.89594775	7.18E+08	0.26	-2.67	3.24	0.05
E1	GY	4A	AX.89554610	7.20E+08	0.22	-2.94	3.44	0.06
E1	GY	4A	AX.89360065	7.27E+08	0.27	-3.70	5.84	0.09
E1	GY	4A	AX.89382815	7.25E+08	0.09	-4.90	4.37	0.07
E1	GY	4A	AX.89336562	7.05E+08	0.15	-3.24	3.15	0.06
E1	GY	4A	AX.89450482	4.11E+07	0.11	-3.73	3.15	0.05
E1	GY	4A	AX.89477455	7.22E+08	0.22	-2.77	3.16	0.05
E1	GY	4A	AX.89356388	7.34E+08	0.20	-3.32	4.01	0.06
E1	GY	4A	AX.89559394	7.33E+08	0.12	-3.86	3.67	0.06
E1	GY	4A	AX.89395851	7.36E+08	0.14	-3.43	3.40	0.05
E1	GY	4A	AX.89376728	7.27E+08	0.22	-3.52	4.72	0.07
E1	GY	4A	AX.89383862	7.22E+08	0.17	-3.02	3.04	0.05
E1	GY	4A	AX.89501646	7.25E+08	0.21	-2.78	3.06	0.04
E1	GY	4A	AX.89432469	7.31E+08	0.20	-3.97	5.60	0.09
E1	GY	4A	AX.89404612	7.30E+08	0.19	-3.56	4.39	0.07
E1	GY	4A	AX.89718118	7.22E+08	0.07	-5.43	4.09	0.07
E1	GY	4A	AX.89336177	4.06E+07	0.11	-3.73	3.15	0.05
E1	GY	4A	AX.89465835	7.41E+08	0.35	-3.11	4.91	0.08
E1	GY	4A	AX.89726273	7.40E+08	0.35	-3.24	5.28	0.08
E1	GY	4A	AX.89396876	7.13E+08	0.20	-3.47	4.28	0.08
E1	GY	4A	AX.89611873	7.19E+08	0.22	-2.81	3.23	0.06
E1	GY	4A	AX.89327540	7.26E+08	0.20	-2.95	3.27	0.05
E1	GY	4A	AX.89644184	7.33E+08	0.21	-2.99	3.46	0.05
E1	GY	4A	AX.89526893	7.24E+08	0.06	-5.09	3.44	0.05
E1	GY	4A	AX.89462486	7.32E+08	0.27	-3.43	5.05	0.08
E1	GY	4A	AX.89462486	7.32E+08	0.27	-3.43	5.05	0.08
E1	GY	4A	AX.89706173	7.32E+08	0.27	-3.43	5.05	0.08
E1	GY	4A	AX.89466707	7.27E+08	0.13	-3.32	3.06	0.05
E1	GY	4A	AX.89491474	7.28E+08	0.15	-3.40	3.42	0.05
E1	GY	4A	AX.89338338	7.43E+08	0.21	-3.25	4.00	0.06
E1	GY	4A	AX.89537632	7.22E+08	0.21	-3.06	3.64	0.06
E1	GY	4A	AX.89556734	7.20E+08	0.17	-3.02	3.10	0.05
E1	GY	4A	AX.89534158	7.31E+08	0.15	-3.18	3.05	0.04
E1	GY	4A	AX.89393646	7.30E+08	0.23	-3.46	4.76	0.07
E1	GY	4A	AX.89557030	7.39E+08	0.16	-3.67	4.16	0.07
E1	GY	4A	AX.89677918	7.28E+08	0.27	-3.70	5.84	0.09
E1	GY	4A	AX.89545628	7.24E+08	0.12	-3.61	3.26	0.05
E1	GY	4A	AX.89480219	7.30E+08	0.13	-3.53	3.41	0.05
E1	GY	4A	AX.89499424	7.24E+08	0.10	-3.81	3.16	0.05
E1	GY	4A	AX.89516106	7.24E+08	0.19	-3.36	3.99	0.06
E1	GY	4A	AX.89503717	7.10E+08	0.16	-3.16	3.15	0.06

E1	GY	4A	AX.89534997	7.26E+08	0.18	-3.53	4.12	0.06
E1	GY	4A	AX.89328721	7.39E+08	0.16	-3.67	4.16	0.07
E1	GY	4A	AX.89682510	7.08E+08	0.15	-3.31	3.26	0.06
E1	GY	4A	AX.89610216	7.30E+08	0.20	-2.82	3.04	0.04
E1	GY	4A	AX.89610216	7.30E+08	0.20	-2.82	3.04	0.04
E1	GY	4A	AX.89351599	7.30E+08	0.20	-3.47	4.41	0.07
E1	GY	4A	AX.89482221	7.06E+08	0.16	-3.16	3.15	0.06
E1	GY	4A	AX.89482221	7.06E+08	0.16	-3.16	3.15	0.06
E1	SPM2	4A	AX.89722769	6.15E+08	0.34	17.54	3.17	0.06
E1	GY	4A	AX.89769007	7.41E+08	0.33	-2.93	4.29	0.06
E1	GY	4A	AX.86163267	7.06E+08	0.16	-3.16	3.15	0.06
E1	GY	4A	AX.89347832	7.22E+08	0.17	-3.14	3.25	0.06
E1	GY	4A	AX.89504759	7.25E+08	0.13	-3.35	3.11	0.05
E1	GY	4A	AX.89411907	7.27E+08	0.16	-3.43	3.63	0.06
E1	GY	4A	AX.89446608	7.25E+08	0.14	-3.51	3.54	0.05
E1	GY	4A	AX.89445680	7.31E+08	0.23	-3.78	5.58	0.09
E1	GY	4A	AX.89751035	7.27E+08	0.21	-3.46	4.45	0.07
E1	GY	4A	AX.89407940	7.23E+08	0.12	-3.61	3.26	0.05
E1	GY	4A	AX.89634915	7.24E+08	0.11	-3.60	3.06	0.05
E1	GY	4A	AX.89635144	7.31E+08	0.27	-3.78	6.02	0.10
E1	GY	4A	AX.89412618	7.34E+08	0.14	-3.62	3.72	0.06
E1	GY	4A	AX.89630821	7.24E+08	0.12	-3.61	3.26	0.05
E1	GY	4A	AX.89314907	7.22E+08	0.22	-2.94	3.44	0.06
E1	GY	4A	AX.89412766	7.37E+08	0.14	-3.43	3.40	0.05
E1	GY	4A	AX.89436017	7.25E+08	0.06	-5.45	3.63	0.06
E1	GY	4A	AX.89684381	7.40E+08	0.36	-3.26	5.34	0.08
E1	GY	4A	AX.89684381	7.40E+08	0.36	-3.26	5.34	0.08
E1	GY	4A	AX.86166488	7.41E+08	0.21	-3.10	3.73	0.06
E1	GY	4A	AX.89330690	7.34E+08	0.21	-2.83	3.20	0.05
E1	GY	4A	AX.89643739	7.24E+08	0.07	-5.67	4.40	0.07
E1	GY	4A	AX.89757043	7.24E+08	0.12	-3.61	3.26	0.05
E1	GY	4A	AX.89342097	7.20E+08	0.21	-2.82	3.16	0.05
E1	GY	4A	AX.89608735	7.39E+08	0.10	-4.10	3.59	0.06
E1	GY	4A	AX.89588539	7.22E+08	0.22	-2.75	3.11	0.05
E1	GY	4A	AX.89644184	7.33E+08	0.21	-2.99	3.46	0.05
E1	GY	4A	AX.89548890	7.25E+08	0.11	-4.13	3.87	0.06
E1	GY	4A	AX.89484604	7.40E+08	0.26	-3.62	5.45	0.09
E1	GY	4A	AX.89698578	7.20E+08	0.22	-2.85	3.31	0.06
E1	GY	4A	AX.89698578	7.20E+08	0.22	-2.85	3.31	0.06
E1	GY	4A	AX.89495274	7.22E+08	0.17	-3.14	3.25	0.06
E1	SPM2	4A	AX.89526889	6.15E+08	0.35	18.82	3.62	0.07
E1	GY	4A	AX.89499280	7.26E+08	0.15	-3.57	3.73	0.06
E1	GY	4A	AX.89499407	7.30E+08	0.15	-3.16	3.08	0.05
E1	GY	4A	AX.89703275	7.22E+08	0.17	-3.09	3.15	0.06
E1	GY	4A	AX.89609481	7.30E+08	0.20	-3.35	4.09	0.06
E1	GY	4A	AX.89398534	7.26E+08	0.09	-4.18	3.45	0.05
E1	GY	4A	AX.89764094	7.24E+08	0.12	-3.61	3.26	0.05
E1	GY	4A	AX.89661917	7.19E+08	0.23	-2.73	3.16	0.05
E1	GY	4A	AX.89313341	7.39E+08	0.12	-3.87	3.58	0.05
E1	GY	4A	AX.89414590	7.30E+08	0.17	-3.31	3.56	0.05

E1	GY	4A	AX.89602206	7.24E+08	0.06	-5.45	3.63	0.06
E1	GY	4A	AX.89520266	7.26E+08	0.20	-2.95	3.27	0.05
E1	GY	4A	AX.89321887	7.23E+08	0.06	-5.17	3.53	0.06
E1	GY	4A	AX.89674921	7.08E+08	0.16	-3.16	3.15	0.06
E1	GY	4A	AX.89674921	7.08E+08	0.16	-3.16	3.15	0.06
E1	GY	4A	AX.89712068	7.24E+08	0.07	-5.67	4.40	0.07
E1	GY	4A	AX.89759750	7.22E+08	0.16	-3.31	3.48	0.06
E1	GY	4A	AX.89563022	7.33E+08	0.22	-3.03	3.67	0.06
E1	GY	4A	AX.89761056	7.30E+08	0.15	-3.40	3.42	0.05
E1	GY	4A	AX.89566818	7.24E+08	0.07	-5.67	4.40	0.07
E1	GY	4A	AX.89555093	7.33E+08	0.22	-3.25	4.11	0.06
E1	GY	4A	AX.89578493	7.20E+08	0.17	-3.02	3.10	0.05
E1	GY	4A	AX.89672155	7.37E+08	0.14	-3.43	3.40	0.05
E1	GY	4A	AX.89668144	7.39E+08	0.15	-3.46	3.52	0.05
E1	GY	4A	AX.89345065	7.21E+08	0.22	-2.77	3.16	0.05
E1	GY	4A	AX.89404612	7.30E+08	0.19	-3.56	4.39	0.07
E1	GY	4A	AX.89652502	7.28E+08	0.22	-3.63	5.06	0.08
E1	GY	4A	AX.89652502	7.28E+08	0.22	-3.63	5.06	0.08
E1	GY	4A	AX.89664678	7.28E+08	0.15	-3.91	4.37	0.07
E1	GY	4A	AX.89522297	7.23E+08	0.12	-3.61	3.26	0.05
E1	GY	4A	AX.89522297	7.23E+08	0.12	-3.61	3.26	0.05
E1	GY	4A	AX.89522316	7.20E+08	0.22	-2.85	3.31	0.06
E1	GY	4A	AX.89639515	7.24E+08	0.07	-4.93	3.65	0.06
E1	GY	4A	AX.89462343	7.27E+08	0.27	-3.70	5.84	0.09
E1	GY	4A	AX.89684945	6.64E+08	0.20	-3.08	3.58	0.05
E1	GY	4A	AX.89369902	7.20E+08	0.17	-3.14	3.25	0.06
E1	GY	4A	AX.89498509	7.26E+08	0.18	-3.29	3.71	0.06
E1	GY	4A	AX.89484604	7.40E+08	0.26	-3.62	5.45	0.09
E1	GY	4A	AX.89312590	7.21E+08	0.22	-2.94	3.44	0.06
E1	GY	4A	AX.89440747	7.30E+08	0.22	-3.87	5.58	0.09
E1	GY	4A	AX.89440747	7.30E+08	0.22	-3.87	5.58	0.09
E1	GY	4A	AX.89616966	7.25E+08	0.11	-3.67	3.06	0.05
E1	GY	4A	AX.89417541	7.10E+08	0.14	-3.61	3.71	0.07
E1	GY	4A	AX.89523678	7.26E+08	0.18	-3.53	4.12	0.06
E1	GY	4A	AX.89346434	7.32E+08	0.26	-3.45	5.01	0.08
E1	GY	4A	AX.89335287	7.34E+08	0.14	-3.62	3.72	0.06
E1	GY	4A	AX.89531127	7.24E+08	0.11	-4.49	4.49	0.07
E1	GY	4A	AX.89328332	7.39E+08	0.11	-4.03	3.60	0.06
E1	GY	4A	AX.89680594	7.26E+08	0.18	-3.29	3.71	0.06
E1	GY	4A	AX.89739556	4.10E+07	0.11	-3.73	3.15	0.05
E1	GY	4A	AX.89471816	7.28E+08	0.20	-3.46	4.25	0.07
E1	GY	4A	AX.89731769	7.27E+08	0.22	-3.52	4.72	0.07
E1	GY	4A	AX.89452602	7.24E+08	0.09	-4.86	4.48	0.07
E1	GY	4A	AX.89617589	4.11E+07	0.11	-3.73	3.15	0.05
E1	GY	4A	AX.89557779	7.30E+08	0.15	-3.47	3.55	0.05
E1	GY	4A	AX.89629181	7.39E+08	0.16	-3.67	4.16	0.07
E1	GY	4A	AX.89347361	7.24E+08	0.06	-5.17	3.53	0.06
E1	GY	4A	AX.89524550	7.39E+08	0.11	-4.03	3.60	0.06
E1	GY	4A	AX.89594507	7.31E+08	0.27	-3.77	6.06	0.10
E1	GY	4A	AX.89594775	7.18E+08	0.26	-2.67	3.24	0.05

E1	GY	4A	AX.89554610	7.20E+08	0.22	-2.94	3.44	0.06
E1	GY	4A	AX.89347832	7.22E+08	0.17	-3.14	3.25	0.06
E1	GY	4A	AX.89360038	7.33E+08	0.22	-3.03	3.67	0.06
E1	GY	4A	AX.89314231	7.39E+08	0.16	-3.52	3.79	0.06
E1	GY	4A	AX.89360065	7.27E+08	0.27	-3.70	5.84	0.09
E1	GY	4A	AX.89704631	7.26E+08	0.13	-3.75	3.69	0.06
E1	GY	4A	AX.89707099	7.41E+08	0.33	-3.69	6.43	0.10
E1	GY	4A	AX.89707630	7.27E+08	0.07	-4.59	3.23	0.05
E1	GY	4A	AX.89369162	7.26E+08	0.20	-2.95	3.27	0.05
E1	GY	4A	AX.89420090	7.30E+08	0.16	-3.57	3.96	0.06
E1	GY	4A	AX.89749239	7.19E+08	0.17	-3.14	3.25	0.06
E1	GY	4A	AX.89454827	7.21E+08	0.17	-3.02	3.04	0.05
E1	GY	4A	AX.89384587	7.31E+08	0.06	-4.71	3.01	0.05
E1	GY	4A	AX.89409546	7.23E+08	0.06	-5.17	3.53	0.06
E1	GY	4A	AX.89592754	7.26E+08	0.12	-3.87	3.57	0.05
E1	GY	4A	AX.89455084	7.31E+08	0.27	-3.77	6.06	0.10
E1	GY	4A	AX.89706173	7.32E+08	0.27	-3.43	5.05	0.08
E1	GY	4A	AX.89775914	7.44E+08	0.07	-4.53	3.15	0.05
E1	GY	4A	AX.89549210	7.39E+08	0.15	-3.46	3.52	0.05
E1	GY	4A	AX.89644669	7.39E+08	0.16	-3.67	4.16	0.07
E1	GY	4A	AX.89644669	7.39E+08	0.16	-3.67	4.16	0.07
E1	GY	4A	AX.89767319	7.24E+08	0.06	-5.17	3.53	0.06
E1	GY	4A	AX.89499280	7.26E+08	0.15	-3.57	3.73	0.06
E1	GY	4A	AX.89743401	7.33E+08	0.19	-2.99	3.25	0.05
E1	GY	4A	AX.89515992	7.25E+08	0.21	-2.77	3.07	0.04
E1	GY	4A	AX.89515992	7.25E+08	0.21	-2.77	3.07	0.04
E1	GY	4A	AX.89452318	7.23E+08	0.06	-5.45	3.63	0.06
E1	GY	4A	AX.89480219	7.30E+08	0.13	-3.53	3.41	0.05
E1	GY	4A	AX.89328298	7.31E+08	0.19	-4.41	6.45	0.11
E1	GY	4A	AX.89321025	7.34E+08	0.14	-3.43	3.40	0.05
E1	GY	4A	AX.89422048	7.31E+08	0.20	-4.25	6.23	0.10
E1	GY	4A	AX.89422048	7.31E+08	0.20	-4.25	6.23	0.10
E1	GY	4A	AX.89768703	7.40E+08	0.27	-3.09	4.26	0.07
E1	GY	4A	AX.89744180	7.19E+08	0.22	-2.75	3.11	0.05
E1	GY	4A	AX.89351503	7.26E+08	0.18	-3.14	3.36	0.05
E1	GY	4A	AX.89710964	7.30E+08	0.26	-3.65	5.61	0.09
E1	GY	4A	AX.89719378	7.24E+08	0.12	-3.71	3.33	0.05
E1	GY	4A	AX.89618065	7.27E+08	0.17	-3.18	3.31	0.05
E1	GY	4A	AX.89744514	7.20E+08	0.21	-3.10	3.67	0.06
E1	GY	4A	AX.89599133	7.40E+08	0.34	-3.40	5.67	0.09
E1	GY	4A	AX.89504759	7.25E+08	0.13	-3.35	3.11	0.05
E1	GY	4A	AX.89727024	7.23E+08	0.12	-3.61	3.26	0.05
E1	GY	4A	AX.89529222	7.26E+08	0.24	-3.21	4.26	0.07
E1	GY	4A	AX.86163811	7.39E+08	0.09	-4.19	3.32	0.05
E1	GY	4A	AX.86163811	7.39E+08	0.09	-4.19	3.32	0.05
E1	GY	4A	AX.89482915	7.04E+08	0.16	-3.16	3.15	0.06
E1	GY	4A	AX.89486705	7.27E+08	0.23	-3.52	4.85	0.08
E1	GY	4A	AX.89412597	7.27E+08	0.13	-3.91	3.95	0.06
E1	GY	4A	AX.89663848	7.40E+08	0.21	-3.44	4.48	0.07
E1	GY	4A	AX.89611401	7.20E+08	0.22	-2.85	3.31	0.06

E1	GY	4A	AX.89536634	7.31E+08	0.15	-4.19	4.92	0.08
E1	GY	4A	AX.89552078	7.09E+08	0.15	-3.35	3.34	0.06
E1	GY	4A	AX.89483845	7.23E+08	0.07	-5.67	4.40	0.07
E1	GY	4A	AX.89530069	7.40E+08	0.17	-3.58	4.06	0.06
E1	GY	4A	AX.89377182	7.27E+08	0.21	-3.53	4.68	0.07
E1	GY	4A	AX.89610678	7.26E+08	0.20	-2.95	3.27	0.05
E1	GY	4A	AX.89757043	7.24E+08	0.12	-3.61	3.26	0.05
E1	GY	4A	AX.89608735	7.39E+08	0.10	-4.10	3.59	0.06
E1	GY	4A	AX.89680753	7.26E+08	0.14	-3.30	3.10	0.05
E1	GY	4A	AX.89588539	7.22E+08	0.22	-2.75	3.11	0.05
E1	GY	4A	AX.89518923	7.24E+08	0.12	-3.61	3.26	0.05
E1	GY	4A	AX.89518923	7.24E+08	0.12	-3.61	3.26	0.05
E1	GY	4A	AX.89455084	7.31E+08	0.27	-3.77	6.06	0.10
E1	GY	4A	AX.89767110	7.21E+08	0.23	-2.73	3.16	0.05
E1	GY	4A	AX.89564765	7.39E+08	0.13	-3.60	3.51	0.05
E1	GY	4A	AX.89421360	7.22E+08	0.17	-3.14	3.25	0.06
E1	GY	4A	AX.89491600	7.20E+08	0.21	-2.73	3.00	0.05
E1	GY	4A	AX.89495274	7.22E+08	0.17	-3.14	3.25	0.06
E1	GY	4A	AX.89565646	7.43E+08	0.21	-3.25	4.00	0.06
E1	GY	4A	AX.89495581	7.21E+08	0.23	-2.73	3.16	0.05
E1	GY	4A	AX.89495581	7.21E+08	0.23	-2.73	3.16	0.05
E1	GY	4A	AX.89448771	7.24E+08	0.06	-5.45	3.63	0.06
E1	GY	4A	AX.89448771	7.24E+08	0.06	-5.45	3.63	0.06
E1	GY	4A	AX.89577216	7.06E+08	0.16	-3.16	3.15	0.06
E1	GY	4A	AX.89735480	7.40E+08	0.36	-3.26	5.34	0.08
E1	GY	4A	AX.89636971	4.04E+07	0.11	-3.73	3.15	0.05
E1	GY	4A	AX.89576737	7.34E+08	0.21	-3.02	3.56	0.05
E1	GY	4A	AX.89459794	7.40E+08	0.14	-3.28	3.16	0.05
E1	GY	4A	AX.89747478	7.41E+08	0.36	-3.26	5.34	0.08
E1	GY	4A	AX.89772769	7.22E+08	0.17	-3.14	3.25	0.06
E1	GY	4A	AX.89354777	7.26E+08	0.09	-3.98	3.16	0.05
E1	GY	4A	AX.89343528	1.76E+07	0.27	3.13	4.32	0.08
E1	GY	4A	AX.89602206	7.24E+08	0.06	-5.45	3.63	0.06
E1	GY	4A	AX.89542047	7.24E+08	0.12	-3.61	3.26	0.05
E1	GY	4A	AX.89760308	7.21E+08	0.17	-3.14	3.25	0.06
E1	GY	4A	AX.89662356	7.26E+08	0.24	-2.96	3.67	0.05
E1	GY	4A	AX.89321874	7.26E+08	0.08	-4.16	3.15	0.05
E1	GY	4A	AX.89426288	6.61E+08	0.10	-3.77	3.10	0.05
E1	GY	4A	AX.89322015	7.39E+08	0.11	-4.03	3.60	0.06
E1	GY	4A	AX.89520523	7.24E+08	0.06	-5.45	3.63	0.06
E1	GY	4A	AX.89566336	7.27E+08	0.16	-4.09	4.93	0.08
E1	GY	4A	AX.89554375	7.27E+08	0.23	-3.49	4.78	0.08
E1	GY	4A	AX.89707063	7.39E+08	0.16	-3.67	4.16	0.07
E1	GY	4A	AX.89708349	7.31E+08	0.17	-3.41	3.82	0.06
E1	GY	4A	AX.89663245	7.19E+08	0.17	-3.14	3.25	0.06
E1	GY	4A	AX.89400318	7.31E+08	0.09	-4.19	3.46	0.05
E1	GY	4A	AX.89622319	7.41E+08	0.26	-3.58	5.42	0.09
E1	GY	4A	AX.89651575	7.24E+08	0.07	-5.67	4.40	0.07
E1	GY	4A	AX.89450913	7.27E+08	0.16	-4.17	5.12	0.08
E1	GY	4A	AX.89668144	7.39E+08	0.15	-3.46	3.52	0.05

E1	GY	4A	AX.89532662	7.40E+08	0.27	-3.37	5.01	0.08
E1	GY	4A	AX.89333861	7.26E+08	0.18	-3.29	3.71	0.06
E1	GY	4A	AX.89615361	7.24E+08	0.06	-5.45	3.63	0.06
E1	GY	4A	AX.89451460	6.47E+08	0.17	-3.10	3.18	0.05
E1	GY	4A	AX.89726325	7.29E+08	0.15	-3.40	3.42	0.05
E1	GY	4A	AX.89342097	7.20E+08	0.21	-2.82	3.16	0.05
E1	GY	4A	AX.89592754	7.26E+08	0.12	-3.87	3.57	0.05
E1	GY	4A	AX.89579660	7.26E+08	0.11	-3.69	3.19	0.05
E1	GY	4A	AX.89568535	7.31E+08	0.27	-3.78	6.02	0.10
E1	GY	4A	AX.89726883	7.41E+08	0.24	-3.40	4.67	0.07
E1	GY	4A	AX.89346010	7.32E+08	0.26	-3.45	5.05	0.08
E1	GY	4A	AX.89324248	7.43E+08	0.15	-3.54	3.67	0.06
E1	GY	4A	AX.89429351	7.27E+08	0.27	-3.70	5.84	0.09
E1	GY	4A	AX.89556734	7.20E+08	0.17	-3.02	3.10	0.05
E1	GY	4A	AX.89776312	7.22E+08	0.21	-3.06	3.64	0.06
E1	GY	4A	AX.89312590	7.21E+08	0.22	-2.94	3.44	0.06
E1	GY	4A	AX.89715796	7.04E+08	0.16	-3.16	3.15	0.06
E1	GY	4A	AX.89358742	7.10E+08	0.14	-3.24	3.08	0.05
E1	GY	4A	AX.89499679	7.26E+08	0.17	-3.23	3.41	0.05
E1	GY	4A	AX.89359076	7.28E+08	0.20	-3.47	4.41	0.07
E1	GY	4A	AX.89359076	7.28E+08	0.20	-3.47	4.41	0.07
E1	GY	4A	AX.89406883	7.26E+08	0.18	-3.14	3.36	0.05
E1	GY	4A	AX.89777164	7.28E+08	0.18	-3.08	3.32	0.05
E1	GY	4A	AX.89777164	7.28E+08	0.18	-3.08	3.32	0.05
E1	GY	4A	AX.89534997	7.26E+08	0.18	-3.53	4.12	0.06
E1	GY	4A	AX.89629079	7.28E+08	0.20	-3.09	3.55	0.05
E1	GY	4A	AX.89666696	7.43E+08	0.10	-4.28	3.73	0.06
E1	GY	4A	AX.89347361	7.24E+08	0.06	-5.17	3.53	0.06
E1	GY	4A	AX.89524550	7.39E+08	0.11	-4.03	3.60	0.06
E1	GY	4A	AX.89594507	7.31E+08	0.27	-3.77	6.06	0.10
E1	GY	4A	AX.89569986	7.24E+08	0.12	-3.61	3.26	0.05
E1	GY	4A	AX.89760720	1.76E+07	0.27	3.06	4.19	0.08
E1	GY	4A	AX.89618065	7.27E+08	0.17	-3.18	3.31	0.05
E1	GY	4A	AX.89535906	7.41E+08	0.21	-3.10	3.73	0.06
E1	GY	4A	AX.89704157	7.25E+08	0.12	-3.93	3.78	0.06
E1	GY	4A	AX.89704157	7.25E+08	0.12	-3.93	3.78	0.06
E1	GY	4A	AX.89711147	7.39E+08	0.18	-2.91	3.01	0.04
E1	GY	4A	AX.89431718	7.31E+08	0.14	-4.72	5.95	0.10
E1	GY	4A	AX.89431718	7.31E+08	0.14	-4.72	5.95	0.10
E1	GY	4A	AX.89559394	7.33E+08	0.12	-3.86	3.67	0.06
E1	GY	4A	AX.89753010	7.05E+08	0.16	-3.16	3.15	0.06
E1	GY	4A	AX.89707630	7.27E+08	0.07	-4.59	3.23	0.05
E1	GY	4A	AX.89668236	7.33E+08	0.15	-3.82	4.19	0.07
E1	GY	4A	AX.89741478	7.28E+08	0.13	-3.56	3.28	0.05
E1	GY	4A	AX.89749239	7.19E+08	0.17	-3.14	3.25	0.06
E1	GY	4A	AX.89718118	7.22E+08	0.07	-5.43	4.09	0.07
E1	GY	4A	AX.89348972	7.32E+08	0.27	-3.43	5.05	0.08
E1	GY	4A	AX.89705660	7.40E+08	0.48	-2.54	3.72	0.06
E1	GY	4A	AX.89327236	7.20E+08	0.18	-2.96	3.09	0.05
E1	GY	4A	AX.89327430	7.26E+08	0.28	-3.45	5.27	0.08

E1	GY	4A	AX.89515035	7.28E+08	0.26	-2.70	3.27	0.05
E1	GY	4A	AX.89742680	7.31E+08	0.11	-3.99	3.53	0.05
E1	GY	4A	AX.89466707	7.27E+08	0.13	-3.32	3.06	0.05
E1	GY	4A	AX.89537546	7.31E+08	0.16	-3.11	3.06	0.05
E1	GY	4A	AX.89349918	7.40E+08	0.18	-3.63	4.33	0.07
E1	GY	4A	AX.89361955	7.39E+08	0.15	-3.46	3.52	0.05
E1	GY	4A	AX.89763075	7.26E+08	0.18	-3.29	3.71	0.06
E1	GY	4A	AX.89549210	7.39E+08	0.15	-3.46	3.52	0.05
E1	GY	4A	AX.89584410	7.31E+08	0.27	-3.77	6.06	0.10
E1	GY	4A	AX.89584410	7.31E+08	0.27	-3.77	6.06	0.10
E1	GY	4A	AX.89433453	7.41E+08	0.23	-3.46	4.75	0.07
E1	GY	4A	AX.89743266	4.09E+07	0.11	-3.73	3.15	0.05
E1	GY	4A	AX.89743401	7.33E+08	0.19	-2.99	3.25	0.05
E1	GY	4A	AX.89385734	7.26E+08	0.15	-3.57	3.73	0.06
E1	GY	4A	AX.89492172	6.17E+08	0.24	-2.63	3.02	0.05
E1	GY	4A	AX.89499424	7.24E+08	0.10	-3.81	3.16	0.05
E1	GY	4A	AX.89645053	7.24E+08	0.08	-4.22	3.22	0.05
E1	GY	4A	AX.89637945	7.26E+08	0.14	-3.51	3.53	0.05
E1	GY	4A	AX.89707039	7.27E+08	0.15	-3.21	3.10	0.05
E1	GY	4A	AX.89328354	7.39E+08	0.16	-3.90	4.55	0.07
E1	GY	4A	AX.89398873	7.21E+08	0.05	-5.43	3.36	0.05
E1	GY	4A	AX.89468094	7.41E+08	0.24	-3.39	4.71	0.07
E1	GY	4A	AX.89598281	7.31E+08	0.17	-4.31	5.77	0.09
E1	GY	4A	AX.89434141	7.22E+08	0.22	-2.94	3.44	0.06
E1	GY	4A	AX.89621604	7.31E+08	0.27	-3.78	6.03	0.10
E1	GY	4A	AX.89621604	7.31E+08	0.27	-3.78	6.03	0.10
E1	GY	4A	AX.89710964	7.30E+08	0.26	-3.65	5.61	0.09
E1	GY	4A	AX.89539186	7.43E+08	0.19	-3.14	3.54	0.05
E1	GY	4A	AX.89328992	7.33E+08	0.19	-2.99	3.25	0.05
E1	GY	4A	AX.89376272	7.26E+08	0.18	-3.29	3.71	0.06
E1	GY	4A	AX.89672103	6.02E+08	0.21	-2.94	3.36	0.05
E1	GY	4A	AX.89634915	7.24E+08	0.11	-3.60	3.06	0.05
E1	GY	4A	AX.89400490	7.24E+08	0.06	-5.45	3.63	0.06
E1	GY	4A	AX.89446849	7.21E+08	0.22	-2.94	3.44	0.06
E1	GY	4A	AX.89423577	7.26E+08	0.13	-3.37	3.15	0.05
E1	GY	4A	AX.89387465	7.17E+08	0.06	-4.70	3.00	0.05
E1	GY	4A	AX.89352927	7.20E+08	0.17	-3.02	3.10	0.05
E1	GY	4A	AX.89600383	4.09E+07	0.11	-3.73	3.15	0.05
E1	GY	4A	AX.89506180	7.37E+08	0.10	-3.91	3.31	0.05
E1	GY	4A	AX.89665964	7.41E+08	0.23	-3.40	4.62	0.07
E1	GY	4A	AX.89643739	7.24E+08	0.07	-5.67	4.40	0.07
E1	GY	4A	AX.89611873	7.19E+08	0.22	-2.81	3.23	0.06
E1	GY	4A	AX.89775815	7.24E+08	0.07	-5.67	4.40	0.07
E1	GY	4A	AX.89767110	7.21E+08	0.23	-2.73	3.16	0.05
E1	GY	4A	AX.89556447	7.39E+08	0.15	-3.46	3.52	0.05
E1	GY	4A	AX.89776093	7.31E+08	0.27	-3.78	6.02	0.10
E1	GY	4A	AX.89735480	7.40E+08	0.36	-3.26	5.34	0.08
E1	GY	4A	AX.89531127	7.24E+08	0.11	-4.49	4.49	0.07
E1	GY	4A	AX.89699271	7.22E+08	0.22	-2.75	3.11	0.05
E1	GY	4A	AX.89747478	7.41E+08	0.36	-3.26	5.34	0.08

E1	GY	4A	AX.89772769	7.22E+08	0.17	-3.14	3.25	0.06
E1	GY	4A	AX.89614087	4.10E+07	0.11	-3.73	3.15	0.05
E1	GY	4A	AX.89614087	4.10E+07	0.11	-3.73	3.15	0.05
E1	GY	4A	AX.89712135	7.25E+08	0.06	-5.45	3.63	0.06
E1	GY	4A	AX.89700054	7.25E+08	0.21	-2.77	3.07	0.04
E1	GY	4A	AX.89391099	7.24E+08	0.12	-3.61	3.26	0.05
E1	GY	4A	AX.89391099	7.24E+08	0.12	-3.61	3.26	0.05
E1	GY	4A	AX.89637968	7.41E+08	0.35	-3.26	5.32	0.08
E1	GY	4A	AX.89450476	7.26E+08	0.11	-4.20	3.87	0.06
E1	GY	4A	AX.89380026	7.24E+08	0.12	-3.61	3.26	0.05
E1	GY	4A	AX.89622319	7.41E+08	0.26	-3.58	5.42	0.09
E1	GY	4A	AX.89322993	7.39E+08	0.15	-3.46	3.52	0.05
E1	GY	4A	AX.89725490	7.40E+08	0.30	-2.98	4.20	0.06
E1	GY	4A	AX.89664021	7.25E+08	0.12	-3.61	3.26	0.05
E1	GY	4A	AX.89345065	7.21E+08	0.22	-2.77	3.16	0.05
E1	GY	4A	AX.89544271	4.09E+07	0.11	-3.73	3.15	0.05
E1	GY	4A	AX.89428619	7.22E+08	0.22	-2.94	3.44	0.06
E1	GY	4A	AX.89652625	7.30E+08	0.07	-5.01	3.76	0.06
E1	GY	4A	AX.89652625	7.30E+08	0.07	-5.01	3.76	0.06
E1	GY	4A	AX.89697948	7.11E+08	0.14	-3.32	3.15	0.06
E1	GY	4A	AX.89697948	7.11E+08	0.14	-3.32	3.15	0.06
E1	GY	4A	AX.89604647	7.22E+08	0.20	-3.54	4.50	0.08
E1	GY	4A	AX.89579752	7.43E+08	0.20	-3.05	3.42	0.05
E1	GY	4A	AX.89689789	7.25E+08	0.21	-2.77	3.07	0.04
E1	GY	4A	AX.89324248	7.43E+08	0.15	-3.54	3.67	0.06
E1	GY	4A	AX.89429351	7.27E+08	0.27	-3.70	5.84	0.09
E1	GY	4A	AX.89334772	4.09E+07	0.11	-3.73	3.15	0.05
E1	GY	4A	AX.89545470	7.16E+08	0.19	-3.98	5.36	0.09
E1	GY	4A	AX.89393646	7.30E+08	0.23	-3.46	4.76	0.07
E1	GY	4A	AX.89593512	7.39E+08	0.16	-3.67	4.16	0.07
E1	GY	4A	AX.89593512	7.39E+08	0.16	-3.67	4.16	0.07
E1	GY	4A	AX.89680594	7.26E+08	0.18	-3.29	3.71	0.06
E1	GY	4A	AX.89739538	4.04E+07	0.11	-3.73	3.15	0.05
E1	GY	4A	AX.89371009	7.26E+08	0.15	-3.57	3.73	0.06
E1	GY	4A	AX.89452602	7.24E+08	0.09	-4.86	4.48	0.07
E1	GY	4A	AX.89654418	7.24E+08	0.12	-3.97	3.74	0.06
E1	GY	4A	AX.89430756	7.24E+08	0.06	-5.45	3.63	0.06
E1	GY	4A	AX.89686985	7.25E+08	0.18	-3.29	3.71	0.06
E1	GY	4A	AX.89629721	7.41E+08	0.27	-3.65	5.65	0.09
E1	GY	4A	AX.89654995	7.27E+08	0.07	-4.48	3.26	0.05
E1	GY	4A	AX.89776458	7.08E+08	0.16	-3.16	3.15	0.06
E1	GY	4A	AX.89371746	7.31E+08	0.27	-3.77	6.06	0.10
E1	GY	4A	AX.89558474	7.41E+08	0.30	-2.80	3.78	0.06
E1	GY	4A	AX.89680337	7.20E+08	0.22	-2.85	3.31	0.06
E1	GY	4A	AX.89535906	7.41E+08	0.21	-3.10	3.73	0.06
E1	GY	4A	AX.89711147	7.39E+08	0.18	-2.91	3.01	0.04
E1	GY	4A	AX.89582475	7.40E+08	0.17	-3.58	4.06	0.06
E1	GY	4A	AX.89336562	7.05E+08	0.15	-3.24	3.15	0.06
E1	GY	4A	AX.89442700	7.26E+08	0.18	-3.29	3.71	0.06
E1	GY	4A	AX.89326485	7.27E+08	0.15	-4.02	4.68	0.07

E1	GY	4A	AX.89326485	7.27E+08	0.15	-4.02	4.68	0.07
E1	GY	4A	AX.89638028	7.25E+08	0.14	-3.69	3.77	0.06
E1	GY	4A	AX.89751035	7.27E+08	0.21	-3.46	4.45	0.07
E1	GY	4A	AX.89709003	7.43E+08	0.09	-4.52	3.78	0.06
E1	GY	4A	AX.89709003	7.43E+08	0.09	-4.52	3.78	0.06
E1	GY	4A	AX.89432068	7.40E+08	0.20	-3.24	3.86	0.06
E1	GY	4A	AX.89635496	7.40E+08	0.17	-3.02	3.09	0.05
E1	GY	4A	AX.89775295	7.26E+08	0.20	-2.95	3.27	0.05
E1	GY	4A	AX.89501646	7.25E+08	0.21	-2.78	3.06	0.04
E1	GY	4A	AX.89515138	7.19E+08	0.22	-2.75	3.11	0.05
E1	GY	4A	AX.89515138	7.19E+08	0.22	-2.75	3.11	0.05
E1	GY	4A	AX.89479076	6.07E+07	0.11	-3.65	3.03	0.05
E1	GY	4A	AX.89680789	7.25E+08	0.18	-3.53	4.12	0.06
E1	GY	4A	AX.89680789	7.25E+08	0.18	-3.53	4.12	0.06
E1	GY	4A	AX.89775914	7.44E+08	0.07	-4.53	3.15	0.05
E1	GY	4A	AX.89503068	7.27E+08	0.15	-3.58	3.74	0.06
E1	GY	4A	AX.89612888	7.26E+08	0.20	-3.49	4.44	0.07
E1	GY	4A	AX.89612888	7.26E+08	0.20	-3.49	4.44	0.07
E1	GY	4A	AX.89445272	7.22E+08	0.22	-2.89	3.35	0.06
E1	GY	4A	AX.89452318	7.23E+08	0.06	-5.45	3.63	0.06
E1	GY	4A	AX.89516106	7.24E+08	0.19	-3.36	3.99	0.06
E1	GPS	4A	AX.89342370	7.28E+08	0.17	-1.52	3.25	0.05
E1	GY	4A	AX.89467705	7.34E+08	0.19	-3.71	4.66	0.07
E1	GY	4A	AX.89411387	7.39E+08	0.15	-3.29	3.24	0.05
E1	GY	4A	AX.89562467	4.09E+07	0.11	-3.73	3.15	0.05
E1	GY	4A	AX.89610369	7.30E+08	0.17	-3.80	4.60	0.07
E1	GY	4A	AX.86163366	7.41E+08	0.32	-2.97	4.32	0.07
E1	GY	4A	AX.89760946	7.38E+08	0.14	-3.31	3.20	0.05
E1	GY	4A	AX.89468920	7.22E+08	0.17	-3.14	3.25	0.06
E1	GY	4A	AX.89412597	7.27E+08	0.13	-3.91	3.95	0.06
E1	GY	4A	AX.89635144	7.31E+08	0.27	-3.78	6.02	0.10
E1	GY	4A	AX.89505951	7.26E+08	0.18	-3.29	3.71	0.06
E1	GY	4A	AX.86166488	7.41E+08	0.21	-3.10	3.73	0.06
E1	GY	4A	AX.89518458	7.33E+08	0.22	-3.03	3.67	0.06
E1	GY	4A	AX.89518458	7.33E+08	0.22	-3.03	3.67	0.06
E1	GY	4A	AX.89729945	7.34E+08	0.12	-3.55	3.08	0.05
E1	GY	4A	AX.89727342	7.23E+08	0.06	-5.17	3.53	0.06
E1	GY	4A	AX.89727342	7.23E+08	0.06	-5.17	3.53	0.06
E1	GY	4A	AX.89610678	7.26E+08	0.20	-2.95	3.27	0.05
E1	GY	4A	AX.89484147	7.39E+08	0.13	-3.69	3.68	0.06
E1	GY	4A	AX.89484147	7.39E+08	0.13	-3.69	3.68	0.06
E1	GY	4A	AX.89734387	7.37E+08	0.14	-3.43	3.40	0.05
E1	GY	4A	AX.89734387	7.37E+08	0.14	-3.43	3.40	0.05
E1	GY	4A	AX.89578346	7.27E+08	0.20	-2.86	3.06	0.04
E1	GY	4A	AX.89578346	7.27E+08	0.20	-2.86	3.06	0.04
E1	GY	4A	AX.89342370	7.28E+08	0.17	-3.95	4.93	0.08
E1	GY	4A	AX.89657236	7.25E+08	0.12	-3.61	3.26	0.05
E1	GY	4A	AX.89657236	7.25E+08	0.12	-3.61	3.26	0.05
E1	GY	4A	AX.89616966	7.25E+08	0.11	-3.67	3.06	0.05
E1	GY	4A	AX.89530946	7.22E+08	0.22	-2.75	3.11	0.05

E1	GY	4A	AX.89499407	7.30E+08	0.15	-3.16	3.08	0.05
E1	GY	4A	AX.89775360	7.33E+08	0.19	-2.99	3.25	0.05
E1	GY	4A	AX.89777958	7.24E+08	0.07	-5.57	4.27	0.07
E1	GY	4A	AX.89777958	7.24E+08	0.07	-5.57	4.27	0.07
E1	GY	4A	AX.89561531	6.61E+08	0.10	-3.77	3.10	0.05
E1	GY	4A	AX.89471816	7.28E+08	0.20	-3.46	4.25	0.07
E1	GY	4A	AX.89735722	6.64E+08	0.20	-3.08	3.58	0.05
E1	GY	4A	AX.89735722	6.64E+08	0.20	-3.08	3.58	0.05
E1	GY	4A	AX.89343528	1.76E+07	0.27	3.13	4.32	0.08
E1	GY	4A	AX.89776011	7.21E+08	0.22	-2.94	3.44	0.06
E1	GY	4A	AX.89776011	7.21E+08	0.22	-2.94	3.44	0.06
E1	GY	4A	AX.89378887	7.25E+08	0.12	-3.97	3.74	0.06
E1	GY	4A	AX.89378887	7.25E+08	0.12	-3.97	3.74	0.06
E1	GY	4A	AX.89321887	7.23E+08	0.06	-5.17	3.53	0.06
E1	GY	4A	AX.89520410	7.41E+08	0.24	-3.39	4.71	0.07
E1	GY	4A	AX.89520410	7.41E+08	0.24	-3.39	4.71	0.07
E1	GY	4A	AX.89712068	7.24E+08	0.07	-5.67	4.40	0.07
E1	GY	4A	AX.89520523	7.24E+08	0.06	-5.45	3.63	0.06
E1	GY	4A	AX.89727201	7.22E+08	0.22	-2.77	3.16	0.05
E1	GY	4A	AX.89486310	7.33E+08	0.22	-3.03	3.67	0.06
E1	GY	4A	AX.89486501	7.41E+08	0.35	-3.24	5.28	0.08
E1	GY	4A	AX.89486501	7.41E+08	0.35	-3.24	5.28	0.08
E1	GY	4A	AX.89333240	7.26E+08	0.18	-3.29	3.71	0.06
E1	GY	4A	AX.89555093	7.33E+08	0.22	-3.25	4.11	0.06
E1	GY	4A	AX.89497230	7.21E+08	0.22	-2.77	3.16	0.05
E1	GY	4A	AX.89400318	7.31E+08	0.09	-4.19	3.46	0.05
E1	GY	4A	AX.89525651	7.24E+08	0.07	-5.67	4.40	0.07
E1	GY	4A	AX.89525651	7.24E+08	0.07	-5.67	4.40	0.07
E1	GY	4A	AX.89598535	7.26E+08	0.15	-3.65	3.88	0.06
E1	GY	4A	AX.89450913	7.27E+08	0.16	-4.17	5.12	0.08
E1	GY	4A	AX.89766142	7.18E+08	0.27	-2.54	3.03	0.05
E1	GY	4A	AX.89766142	7.18E+08	0.27	-2.54	3.03	0.05
E1	GY	4A	AX.89775352	7.41E+08	0.24	-3.39	4.71	0.07
E1	GY	4A	AX.89664678	7.28E+08	0.15	-3.91	4.37	0.07
E1	GY	4A	AX.89474542	7.26E+08	0.24	-3.21	4.26	0.07
E1	GY	4A	AX.89558641	7.39E+08	0.15	-3.46	3.52	0.05
E1	GY	4A	AX.89558641	7.39E+08	0.15	-3.46	3.52	0.05
E1	GY	4A	AX.89474564	7.26E+08	0.11	-4.82	5.09	0.08
E1	GY	4A	AX.89451460	6.47E+08	0.17	-3.10	3.18	0.05
E1	GY	4A	AX.89639515	7.24E+08	0.07	-4.93	3.65	0.06
E1	GY	4A	AX.89380919	7.25E+08	0.06	-5.45	3.63	0.06
E1	GY	4A	AX.89380919	7.25E+08	0.06	-5.45	3.63	0.06
E1	GY	4A	AX.89369902	7.20E+08	0.17	-3.14	3.25	0.06
E1	GY	4A	AX.89639898	7.44E+08	0.21	-3.60	4.77	0.08
E1	GY	4A	AX.89639898	7.44E+08	0.21	-3.60	4.77	0.08
E1	GPS	4A	AX.89328298	7.31E+08	0.19	-1.39	3.01	0.05
E1	GY	4A	AX.89690177	7.26E+08	0.18	-3.29	3.71	0.06
E1	GY	4A	AX.89690177	7.26E+08	0.18	-3.29	3.71	0.06
E1	GY	4A	AX.89417541	7.10E+08	0.14	-3.61	3.71	0.07
E1	GY	4A	AX.89617069	7.28E+08	0.21	-3.37	4.31	0.07

E1	GY	4A	AX.89523678	7.26E+08	0.18	-3.53	4.12	0.06
E1	GY	4A	AX.89512247	7.24E+08	0.12	-3.61	3.26	0.05
E1	GY	4A	AX.89346434	7.32E+08	0.26	-3.45	5.01	0.08
E1	GY	4A	AX.89371009	7.26E+08	0.15	-3.57	3.73	0.06
E1	GY	4A	AX.89739538	4.04E+07	0.11	-3.73	3.15	0.05
E1	GY	4A	AX.89524010	7.32E+08	0.26	-3.45	5.01	0.08
E1	GY	4A	AX.89524010	7.32E+08	0.26	-3.45	5.01	0.08
E1	GY	4A	AX.89443687	7.27E+08	0.07	-4.41	3.02	0.05
E1	GY	4A	AX.89418224	7.24E+08	0.11	-4.10	3.82	0.06
E1	GY	4A	AX.89686985	7.25E+08	0.18	-3.29	3.71	0.06
E1	GY	4A	AX.89667062	7.22E+08	0.22	-2.89	3.35	0.06
E1	GY	4A	AX.89546685	7.39E+08	0.18	-3.24	3.62	0.05
E1	GY	4A	AX.89558474	7.41E+08	0.30	-2.80	3.78	0.06
E1	GPS	4A	AX.89440747	7.30E+08	0.22	-1.34	3.07	0.05
E1	GY	4A	AX.89476973	7.30E+08	0.20	-3.02	3.42	0.05
E1	GY	4A	AX.89704631	7.26E+08	0.13	-3.75	3.69	0.06
E1	GY	4A	AX.89707099	7.41E+08	0.33	-3.69	6.43	0.10
E1	GY	4A	AX.89420090	7.30E+08	0.16	-3.57	3.96	0.06
E1	GY	4A	AX.89775295	7.26E+08	0.20	-2.95	3.27	0.05
E1	GY	4A	AX.89348972	7.32E+08	0.27	-3.43	5.05	0.08
E1	GY	4A	AX.89454827	7.21E+08	0.17	-3.02	3.04	0.05
E1	GY	4A	AX.89373507	7.41E+08	0.30	-2.68	3.49	0.05
E1	GY	4A	AX.89526893	7.24E+08	0.06	-5.09	3.44	0.05
E1	GY	4A	AX.89349918	7.40E+08	0.18	-3.63	4.33	0.07
E1	GY	4A	AX.89338338	7.43E+08	0.21	-3.25	4.00	0.06
E1	GY	4A	AX.89640400	7.27E+08	0.13	-3.64	3.50	0.05
E1	GY	4A	AX.89492022	7.30E+08	0.20	-3.47	4.41	0.07
E1	GY	4A	AX.89385734	7.26E+08	0.15	-3.57	3.73	0.06
E1	GY	4A	AX.89385882	7.29E+08	0.22	-3.44	4.58	0.07
E1	GY	4A	AX.89637945	7.26E+08	0.14	-3.51	3.53	0.05
E1	GY	4A	AX.89597931	7.27E+08	0.15	-4.20	5.06	0.08
E1	GY	4A	AX.89597931	7.27E+08	0.15	-4.20	5.06	0.08
E1	GY	4A	AX.89670415	7.22E+08	0.17	-3.14	3.25	0.06
E1	GY	4A	AX.89719378	7.24E+08	0.12	-3.71	3.33	0.05
E1	GY	4A	AX.89375502	7.40E+08	0.17	-3.55	4.08	0.06
E1	GY	4A	AX.89599133	7.40E+08	0.34	-3.40	5.67	0.09
E1	GY	4A	AX.89727024	7.23E+08	0.12	-3.61	3.26	0.05
E1	GY	4A	AX.89446643	7.40E+08	0.36	-3.26	5.34	0.08
E1	GY	4A	AX.89729045	7.33E+08	0.07	-5.05	3.80	0.06
E1	GY	4A	AX.89407940	7.23E+08	0.12	-3.61	3.26	0.05
E1	GY	4A	AX.89663848	7.40E+08	0.21	-3.44	4.48	0.07
E1	GY	4A	AX.89571325	7.40E+08	0.26	-3.40	4.93	0.08
E1	GY	4A	AX.89387465	7.17E+08	0.06	-4.70	3.00	0.05
E1	GY	4A	AX.89600383	4.09E+07	0.11	-3.73	3.15	0.05
E1	GY	4A	AX.89665964	7.41E+08	0.23	-3.40	4.62	0.07
E1	GY	4A	AX.89483845	7.23E+08	0.07	-5.67	4.40	0.07
E1	GY	4A	AX.89719476	7.41E+08	0.27	-3.44	5.19	0.08
E1	GY	4A	AX.89707143	7.40E+08	0.25	-3.68	5.55	0.09
E1	GY	4A	AX.89681093	7.40E+08	0.25	-3.68	5.51	0.09
E1	GY	4A	AX.89681093	7.40E+08	0.25	-3.68	5.51	0.09

E1	GY	4A	AX.89685609	7.20E+08	0.17	-3.02	3.10	0.05
E1	GPS	4A	AX.89326485	7.27E+08	0.15	-1.62	3.36	0.06
E1	GY	4A	AX.89775360	7.33E+08	0.19	-2.99	3.25	0.05
E1	GY	4A	AX.89661917	7.19E+08	0.23	-2.73	3.16	0.05
E1	GY	4A	AX.89354777	7.26E+08	0.09	-3.98	3.16	0.05
E1	GY	4A	AX.89628286	7.20E+08	0.18	-3.05	3.20	0.06
E1	GY	4A	AX.89678810	7.25E+08	0.20	-2.98	3.40	0.05
E1	GY	4A	AX.89321874	7.26E+08	0.08	-4.16	3.15	0.05
E1	GY	4A	AX.89355306	7.41E+08	0.34	-3.80	6.90	0.11
E1	GY	4A	AX.89355306	7.41E+08	0.34	-3.80	6.90	0.11
E1	GY	4A	AX.89554375	7.27E+08	0.23	-3.49	4.78	0.08
E1	GY	4A	AX.89624256	7.44E+08	0.20	-3.20	3.77	0.06
E1	GY	4A	AX.89707063	7.39E+08	0.16	-3.67	4.16	0.07
E1	GY	4A	AX.89637772	7.21E+08	0.17	-3.14	3.25	0.06
E1	GY	4A	AX.89600590	7.27E+08	0.16	-3.28	3.37	0.05
E1	GY	4A	AX.89600590	7.27E+08	0.16	-3.28	3.37	0.05
E1	GY	4A	AX.89573514	7.34E+08	0.07	-4.45	3.06	0.05
E1	GY	4A	AX.89687921	7.25E+08	0.16	-3.47	3.70	0.06
E1	GY	4A	AX.89687921	7.25E+08	0.16	-3.47	3.70	0.06
E1	GY	4A	AX.89708349	7.31E+08	0.17	-3.41	3.82	0.06
E1	GY	4A	AX.89566817	7.26E+08	0.18	-3.14	3.36	0.05
E1	GY	4A	AX.89322782	7.24E+08	0.12	-3.61	3.26	0.05
E1	GY	4A	AX.89767812	7.31E+08	0.27	-3.65	5.66	0.09
E1	GY	4A	AX.89767812	7.31E+08	0.27	-3.65	5.66	0.09
E1	GY	4A	AX.89322806	7.39E+08	0.13	-3.42	3.15	0.05
E1	GY	4A	AX.89642948	7.41E+08	0.35	-3.41	5.77	0.09
E1	GY	4A	AX.89723261	7.33E+08	0.17	-3.49	3.89	0.06
E1	GY	4A	AX.89659280	7.39E+08	0.16	-3.67	4.16	0.07
E1	GY	4A	AX.89659280	7.39E+08	0.16	-3.67	4.16	0.07
E1	GY	4A	AX.89543701	6.61E+08	0.10	-3.77	3.10	0.05
E1	GY	4A	AX.89431866	7.34E+08	0.22	-2.82	3.22	0.05
E1	GY	4A	AX.89431866	7.34E+08	0.22	-2.82	3.22	0.05
E1	GY	4A	AX.89611606	4.10E+07	0.11	-3.73	3.15	0.05
E1	GY	4A	AX.89392325	7.24E+08	0.12	-3.61	3.26	0.05
E1	GY	4A	AX.89432804	7.25E+08	0.13	-4.07	4.12	0.06
E1	GY	4A	AX.89604647	7.22E+08	0.20	-3.54	4.50	0.08
E1	GY	4A	AX.89498509	7.26E+08	0.18	-3.29	3.71	0.06
E1	GY	4A	AX.89677918	7.28E+08	0.27	-3.70	5.84	0.09
E1	GY	4A	AX.89572745	7.27E+08	0.13	-3.56	3.28	0.05
E1	GY	4A	AX.89572745	7.27E+08	0.13	-3.56	3.28	0.05
E1	GY	4A	AX.89358742	7.10E+08	0.14	-3.24	3.08	0.05
E1	GY	4A	AX.89739556	4.10E+07	0.11	-3.73	3.15	0.05
E1	GY	4A	AX.89499679	7.26E+08	0.17	-3.23	3.41	0.05
E1	GY	4A	AX.89629079	7.28E+08	0.20	-3.09	3.55	0.05
E1	GY	4A	AX.89716456	7.29E+08	0.22	-3.44	4.58	0.07
E1	GY	4A	AX.89629721	7.41E+08	0.27	-3.65	5.65	0.09
E1	GY	4A	AX.89569986	7.24E+08	0.12	-3.61	3.26	0.05
E1	GY	4A	AX.89752209	7.24E+08	0.12	-3.61	3.26	0.05
E1	GY	4A	AX.89582475	7.40E+08	0.17	-3.58	4.06	0.06
E1	GY	4A	AX.89753010	7.05E+08	0.16	-3.16	3.15	0.06

E1	GY	4A	AX.89705660	7.40E+08	0.48	-2.54	3.72	0.06
E1	GY	4A	AX.89537546	7.31E+08	0.16	-3.11	3.06	0.05
E1	GY	4A	AX.89763075	7.26E+08	0.18	-3.29	3.71	0.06
E1	GY	4A	AX.89433453	7.41E+08	0.23	-3.46	4.75	0.07
E1	GY	4A	AX.89572712	4.05E+07	0.11	-3.73	3.15	0.05
E1	GY	4A	AX.89572712	4.05E+07	0.11	-3.73	3.15	0.05
E1	GY	4A	AX.89545628	7.24E+08	0.12	-3.61	3.26	0.05
E1	GY	4A	AX.89503631	7.28E+08	0.14	-3.41	3.29	0.05
E1	GY	4A	AX.89503631	7.28E+08	0.14	-3.41	3.29	0.05
E1	GY	4A	AX.89328298	7.31E+08	0.19	-4.41	6.45	0.11
E1	GY	4A	AX.89707039	7.27E+08	0.15	-3.21	3.10	0.05
E1	GY	4A	AX.89362942	7.26E+08	0.23	-3.14	4.02	0.06
E1	GY	4A	AX.89328354	7.39E+08	0.16	-3.90	4.55	0.07
E1	GY	4A	AX.89468094	7.41E+08	0.24	-3.39	4.71	0.07
E1	GY	4A	AX.89328721	7.39E+08	0.16	-3.67	4.16	0.07
E1	GY	4A	AX.89482485	7.24E+08	0.12	-4.03	3.83	0.06
E1	GY	4A	AX.89759750	7.22E+08	0.16	-3.31	3.48	0.06
E1	GY	4A	AX.89329174	7.22E+08	0.18	-3.24	3.54	0.06
E1	GY	4A	AX.89329174	7.22E+08	0.18	-3.24	3.54	0.06
E1	GY	4A	AX.89755912	7.33E+08	0.14	-3.62	3.64	0.06
E1	GY	4A	AX.89318590	7.24E+08	0.11	-3.67	3.07	0.05
E1	GY	4A	AX.89517532	7.24E+08	0.12	-3.61	3.26	0.05
E1	GY	4A	AX.89400490	7.24E+08	0.06	-5.45	3.63	0.06
E1	GY	4A	AX.89486705	7.27E+08	0.23	-3.52	4.85	0.08
E1	GY	4A	AX.89469231	7.30E+08	0.20	-2.88	3.19	0.05
E1	GY	4A	AX.89457592	7.26E+08	0.24	-3.39	4.64	0.07
E1	GY	4A	AX.89364766	7.26E+08	0.24	-2.96	3.67	0.05
E1	GY	4A	AX.89630821	7.24E+08	0.12	-3.61	3.26	0.05
E1	GY	4A	AX.89559764	7.22E+08	0.17	-3.14	3.25	0.06
E1	GY	4A	AX.89423999	7.24E+08	0.06	-5.48	3.66	0.06
E1	GY	4A	AX.89423999	7.24E+08	0.06	-5.48	3.66	0.06
E1	GY	4A	AX.89506180	7.37E+08	0.10	-3.91	3.31	0.05
E1	GY	4A	AX.89588174	7.34E+08	0.21	-3.02	3.56	0.05
E1	GY	4A	AX.89330690	7.34E+08	0.21	-2.83	3.20	0.05
E1	GY	4A	AX.89710159	7.24E+08	0.07	-5.27	4.32	0.07
E1	GPM2	4A	AX.89677538	4.06E+07	0.10	-1141.22	3.98	0.07
E1	GY	4A	AX.89684945	6.64E+08	0.20	-3.08	3.58	0.05
E1	GY	4A	AX.89775815	7.24E+08	0.07	-5.67	4.40	0.07
E1	GY	4A	AX.89712135	7.25E+08	0.06	-5.45	3.63	0.06
E1	GY	4A	AX.89367604	7.25E+08	0.18	-3.29	3.71	0.06
E1	GPM2	4A	AX.89484604	7.40E+08	0.26	-679.49	3.16	0.05
E1	GY	4A	AX.89497176	7.40E+08	0.33	-3.53	5.99	0.10
E1	GY	4A	AX.89333240	7.26E+08	0.18	-3.29	3.71	0.06
E1	GY	4A	AX.89723261	7.33E+08	0.17	-3.49	3.89	0.06
E1	GY	4A	AX.89651575	7.24E+08	0.07	-5.67	4.40	0.07
E1	GY	4A	AX.89672155	7.37E+08	0.14	-3.43	3.40	0.05
E1	GY	4A	AX.89725490	7.40E+08	0.30	-2.98	4.20	0.06
E1	GY	4A	AX.89369037	7.34E+08	0.13	-3.78	3.63	0.06
E1	GY	4A	AX.89611606	4.10E+07	0.11	-3.73	3.15	0.05
E1	GY	4A	AX.89474035	7.26E+08	0.13	-3.96	4.16	0.07

E1	GY	4A	AX.89631080	7.25E+08	0.11	-3.78	3.33	0.05
E1	GY	4A	AX.89631080	7.25E+08	0.11	-3.78	3.33	0.05
E1	GY	4A	AX.89775352	7.41E+08	0.24	-3.39	4.71	0.07
E1	GY	4A	AX.89462343	7.27E+08	0.27	-3.70	5.84	0.09
E1	GY	4A	AX.89537376	7.24E+08	0.07	-4.93	3.65	0.06
E1	GY	4A	AX.89579752	7.43E+08	0.20	-3.05	3.42	0.05
E1	GY	4A	AX.89545470	7.16E+08	0.19	-3.98	5.36	0.09
E1	GY	4A	AX.89690364	7.40E+08	0.36	-3.26	5.34	0.08
E1	GPM2	4A	AX.89469366	4.04E+07	0.10	-1141.22	3.98	0.07
E1	GPM2	4A	AX.89448965	4.19E+07	0.10	-949.30	3.01	0.05
E1	GY	4A	AX.89654418	7.24E+08	0.12	-3.97	3.74	0.06
E1	GPM2	4A	AX.89612127	4.11E+07	0.11	-1001.42	3.40	0.05
E1	GPM2	4A	AX.89743266	4.09E+07	0.11	-1001.42	3.40	0.05
E1	GY	4A	AX.89680337	7.20E+08	0.22	-2.85	3.31	0.06
E1	GY	4A	AX.89752209	7.24E+08	0.12	-3.61	3.26	0.05
E1	GY	4A	AX.89382815	7.25E+08	0.09	-4.90	4.37	0.07
E1	GY	4A	AX.89442700	7.26E+08	0.18	-3.29	3.71	0.06
E1	GY	4A	AX.89638028	7.25E+08	0.14	-3.69	3.77	0.06
E1	GY	4A	AX.89570705	7.31E+08	0.19	-3.04	3.35	0.05
E1	GY	4A	AX.89692841	7.29E+08	0.22	-3.44	4.58	0.07
E1	GY	4A	AX.89692841	7.29E+08	0.22	-3.44	4.58	0.07
E1	GY	4A	AX.89395851	7.36E+08	0.14	-3.43	3.40	0.05
E1	GPM2	4A	AX.89600383	4.09E+07	0.11	-1001.42	3.40	0.05
E1	GY	4A	AX.89432068	7.40E+08	0.20	-3.24	3.86	0.06
E1	GPM2	4A	AX.89600862	6.04E+08	0.32	626.11	3.09	0.05
E1	GY	4A	AX.89537632	7.22E+08	0.21	-3.06	3.64	0.06
E1	GY	4A	AX.89591500	7.27E+08	0.18	-4.23	5.68	0.09
E1	GY	4A	AX.89591500	7.27E+08	0.18	-4.23	5.68	0.09
E1	GY	4A	AX.89444767	7.26E+08	0.24	-3.38	4.68	0.07
E1	GY	4A	AX.89503068	7.27E+08	0.15	-3.58	3.74	0.06
E1	GY	4A	AX.89767319	7.24E+08	0.06	-5.17	3.53	0.06
E1	GPM2	4A	AX.89431718	7.31E+08	0.14	-850.46	3.20	0.05
E1	GY	4A	AX.89445272	7.22E+08	0.22	-2.89	3.35	0.06
E1	GPM2	4A	AX.89611606	4.10E+07	0.11	-1001.42	3.40	0.05
E1	GY	4A	AX.89467705	7.34E+08	0.19	-3.71	4.66	0.07
E1	GY	4A	AX.89695704	7.21E+08	0.07	-4.97	3.89	0.06
E1	GY	4A	AX.89695704	7.21E+08	0.07	-4.97	3.89	0.06
E1	GY	4A	AX.89755246	7.27E+08	0.12	-3.57	3.20	0.05
E1	GY	4A	AX.89755246	7.27E+08	0.12	-3.57	3.20	0.05
E1	GPM2	4A	AX.89580470	4.05E+07	0.10	-1141.22	3.98	0.07
E1	GY	4A	AX.89411387	7.39E+08	0.15	-3.29	3.24	0.05
E1	GY	4A	AX.89610369	7.30E+08	0.17	-3.80	4.60	0.07
E1	GY	4A	AX.89482485	7.24E+08	0.12	-4.03	3.83	0.06
E1	GY	4A	AX.89760946	7.38E+08	0.14	-3.31	3.20	0.05
E1	GY	4A	AX.89445680	7.31E+08	0.23	-3.78	5.58	0.09
E1	GY	4A	AX.89364766	7.26E+08	0.24	-2.96	3.67	0.05
E1	GY	4A	AX.86164644	7.22E+08	0.22	-2.70	3.02	0.05
E1	GY	4A	AX.86164644	7.22E+08	0.22	-2.70	3.02	0.05
E1	GY	4A	AX.89559764	7.22E+08	0.17	-3.14	3.25	0.06
E1	GY	4A	AX.89505951	7.26E+08	0.18	-3.29	3.71	0.06

E1	GPM2	4A	AX.89312465	4.05E+07	0.10	-1141.22	3.98	0.07
E1	GY	4A	AX.89486698	7.42E+07	0.07	-4.73	3.22	0.05
E1	GY	4A	AX.89486698	7.42E+07	0.07	-4.73	3.22	0.05
E1	GY	4A	AX.89548890	7.25E+08	0.11	-4.13	3.87	0.06
E1	GPM2	4A	AX.89509690	4.32E+07	0.09	-1091.74	3.55	0.06
E1	GY	4A	AX.89530946	7.22E+08	0.22	-2.75	3.11	0.05
E1	GY	4A	AX.89561531	6.61E+08	0.10	-3.77	3.10	0.05
E1	GY	4A	AX.89609481	7.30E+08	0.20	-3.35	4.09	0.06
E1	GY	4A	AX.89764094	7.24E+08	0.12	-3.61	3.26	0.05
E1	GY	4A	AX.89661835	7.33E+08	0.11	-3.63	3.02	0.04
E1	GY	4A	AX.89731769	7.27E+08	0.22	-3.52	4.72	0.07
E1	GY	4A	AX.89760308	7.21E+08	0.17	-3.14	3.25	0.06
E1	GY	4A	AX.89625179	7.22E+08	0.17	-3.14	3.25	0.06
E1	GY	4A	AX.89625179	7.22E+08	0.17	-3.14	3.25	0.06
E1	GPS	4A	AX.89412597	7.27E+08	0.13	-1.63	3.03	0.05
E1	GY	4A	AX.89727201	7.22E+08	0.22	-2.77	3.16	0.05
E1	GPM2	4A	AX.89371505	4.04E+07	0.10	-1141.22	3.98	0.07
E1	GY	4A	AX.89663245	7.19E+08	0.17	-3.14	3.25	0.06
E1	GY	4A	AX.89683437	7.33E+08	0.13	-3.67	3.64	0.06
E1	GY	4A	AX.89683437	7.33E+08	0.13	-3.67	3.64	0.06
E1	GY	4A	AX.89642948	7.41E+08	0.35	-3.41	5.77	0.09
E1	GY	4A	AX.89632750	7.41E+08	0.23	-3.46	4.76	0.07
E1	GPM2	4A	AX.89396292	4.05E+07	0.10	-1141.22	3.98	0.07
E1	GY	4A	AX.89598535	7.26E+08	0.15	-3.65	3.88	0.06
E1	GPM2	4A	AX.89544271	4.09E+07	0.11	-1001.42	3.40	0.05
E1	GPM2	4A	AX.89568216	4.19E+07	0.09	-1091.74	3.55	0.06
E1	GY	4A	AX.89474564	7.26E+08	0.11	-4.82	5.09	0.08
E1	GY	4A	AX.89726325	7.29E+08	0.15	-3.40	3.42	0.05
E1	GPM2	4A	AX.89666039	4.04E+07	0.10	-1014.16	3.36	0.05
E1	GY	4A	AX.89760389	7.40E+08	0.12	-4.27	4.24	0.07
E1	GY	4A	AX.89760389	7.40E+08	0.12	-4.27	4.24	0.07
E1	GY	4A	AX.89737070	7.09E+08	0.15	-3.47	3.62	0.06
E1	GY	4A	AX.89737070	7.09E+08	0.15	-3.47	3.62	0.06
E1	GPM2	4A	AX.89562467	4.09E+07	0.11	-1001.42	3.40	0.05
E1	GPM2	4A	AX.89748168	4.09E+07	0.10	-1141.22	3.98	0.07
E1	GPM2	4A	AX.89458101	4.09E+07	0.10	-1141.22	3.98	0.07
E1	GY	4A	AX.89715796	7.04E+08	0.16	-3.16	3.15	0.06
E1	GY	4A	AX.89381828	7.32E+08	0.27	-3.43	5.05	0.08
E1	GY	4A	AX.89557779	7.30E+08	0.15	-3.47	3.55	0.05
E1	GPM2	4A	AX.89564666	4.09E+07	0.10	-1141.22	3.98	0.07
E1	GY	4A	AX.89418224	7.24E+08	0.11	-4.10	3.82	0.06
E1	GY	4A	AX.89546685	7.39E+08	0.18	-3.24	3.62	0.05
E1	GY	4A	AX.89692225	7.33E+08	0.19	-3.40	4.06	0.06
E1	GY	4A	AX.89692225	7.33E+08	0.19	-3.40	4.06	0.06
E1	GY	4A	AX.89663455	7.22E+08	0.17	-3.27	3.55	0.06
E1	GPM2	4A	AX.89745300	4.19E+07	0.10	-1141.22	3.98	0.07
E1	GY	4A	AX.89741478	7.28E+08	0.13	-3.56	3.28	0.05
E1	GPM2	4A	AX.89608513	3.28E+08	0.11	-991.09	3.45	0.05
E1	GPM2	4A	AX.89640472	1.73E+07	0.08	-1105.70	3.20	0.06
E1	GPM2	4A	AX.89421899	4.19E+07	0.09	-1091.74	3.55	0.06

E1	GY	4A	AX.89479076	6.07E+07	0.11	-3.65	3.03	0.05
E1	GPM2	4A	AX.89572712	4.05E+07	0.11	-1001.42	3.40	0.05
E1	GY	4A	AX.89492022	7.30E+08	0.20	-3.47	4.41	0.07
E1	GPM2	4A	AX.89564368	4.19E+07	0.10	-949.30	3.01	0.05
E1	GY	4A	AX.89612127	4.11E+07	0.11	-3.73	3.15	0.05
E1	GY	4A	AX.89650128	7.20E+08	0.22	-2.75	3.11	0.05
E1	GY	4A	AX.89650128	7.20E+08	0.22	-2.75	3.11	0.05
E1	GY	4A	AX.89492910	7.41E+08	0.24	-3.29	4.42	0.07
E1	GY	4A	AX.89492910	7.41E+08	0.24	-3.29	4.42	0.07
E1	GY	4A	AX.89750215	7.31E+08	0.20	-3.97	5.60	0.09
E1	GY	4A	AX.89750215	7.31E+08	0.20	-3.97	5.60	0.09
E1	GY	4A	AX.89563022	7.33E+08	0.22	-3.03	3.67	0.06
E1	GY	4A	AX.89529222	7.26E+08	0.24	-3.21	4.26	0.07
E1	GPM2	4A	AX.89688644	4.04E+07	0.10	-1141.22	3.98	0.07
E1	GY	4A	AX.89692684	7.27E+08	0.08	-4.55	3.52	0.05
E1	GY	4A	AX.89692684	7.27E+08	0.08	-4.55	3.52	0.05
E1	GY	4A	AX.89571325	7.40E+08	0.26	-3.40	4.93	0.08
E1	GY	4A	AX.89536634	7.31E+08	0.15	-4.19	4.92	0.08
E1	GY	4A	AX.89707143	7.40E+08	0.25	-3.68	5.55	0.09
E1	GY	4A	AX.89685609	7.20E+08	0.17	-3.02	3.10	0.05
E1	GY	4A	AX.89491600	7.20E+08	0.21	-2.73	3.00	0.05
E1	GY	4A	AX.89565646	7.43E+08	0.21	-3.25	4.00	0.06
E1	GY	4A	AX.89577216	7.06E+08	0.16	-3.16	3.15	0.06
E1	GY	4A	AX.89636971	4.04E+07	0.11	-3.73	3.15	0.05
E1	GY	4A	AX.89576737	7.34E+08	0.21	-3.02	3.56	0.05
E1	GPM2	4A	AX.89739538	4.04E+07	0.11	-1001.42	3.40	0.05
E1	GPM2	4A	AX.89343528	1.76E+07	0.27	767.59	3.98	0.07
E1	GY	4A	AX.89628286	7.20E+08	0.18	-3.05	3.20	0.06
E1	GY	4A	AX.89542047	7.24E+08	0.12	-3.61	3.26	0.05
E1	GY	4A	AX.89566336	7.27E+08	0.16	-4.09	4.93	0.08
E1	GY	4A	AX.89624256	7.44E+08	0.20	-3.20	3.77	0.06
E1	GY	4A	AX.89573514	7.34E+08	0.07	-4.45	3.06	0.05
E1	GY	4A	AX.89566817	7.26E+08	0.18	-3.14	3.36	0.05
E1	GY	4A	AX.89322782	7.24E+08	0.12	-3.61	3.26	0.05
E1	GY	4A	AX.89432804	7.25E+08	0.13	-4.07	4.12	0.06
E1	GY	4A	AX.89726883	7.41E+08	0.24	-3.40	4.67	0.07
E1	GY	4A	AX.89534158	7.31E+08	0.15	-3.18	3.05	0.04
E1	GY	4A	AX.89443687	7.27E+08	0.07	-4.41	3.02	0.05
E1	GY	4A	AX.89430756	7.24E+08	0.06	-5.45	3.63	0.06
E1	GY	4A	AX.89760720	1.76E+07	0.27	3.06	4.19	0.08
E1	GY	4A	AX.89515035	7.28E+08	0.26	-2.70	3.27	0.05
E1	GY	4A	AX.89640400	7.27E+08	0.13	-3.64	3.50	0.05
E1	GY	4A	AX.89645053	7.24E+08	0.08	-4.22	3.22	0.05
E1	GY	4A	AX.89362942	7.26E+08	0.23	-3.14	4.02	0.06
E1	GY	4A	AX.89434141	7.22E+08	0.22	-2.94	3.44	0.06
E1	GY	4A	AX.89634490	4.04E+07	0.11	-3.73	3.15	0.05
E1	GY	4A	AX.89634490	4.04E+07	0.11	-3.73	3.15	0.05
E1	GY	4A	AX.89517532	7.24E+08	0.12	-3.61	3.26	0.05
E1	GY	4A	AX.89556447	7.39E+08	0.15	-3.46	3.52	0.05
E1	GPM2	4A	AX.89634490	4.04E+07	0.11	-1001.42	3.40	0.05

E1	GPM2	4A	AX.89548225	4.19E+07	0.09	-1091.74	3.55	0.06
E1	GY	4A	AX.89637968	7.41E+08	0.35	-3.26	5.32	0.08
E1	GY	4A	AX.89497176	7.40E+08	0.33	-3.53	5.99	0.10
E1	GY	4A	AX.89497230	7.21E+08	0.22	-2.77	3.16	0.05
E1	GY	4A	AX.89543701	6.61E+08	0.10	-3.77	3.10	0.05
E1	GY	4A	AX.89664021	7.25E+08	0.12	-3.61	3.26	0.05
E1	GY	4A	AX.89474035	7.26E+08	0.13	-3.96	4.16	0.07
E1	GY	4A	AX.89333861	7.26E+08	0.18	-3.29	3.71	0.06
E1	GPM2	4A	AX.89309778	4.04E+07	0.10	-1141.22	3.98	0.07
E1	GPM2	4A	AX.89647014	4.05E+07	0.10	-1141.22	3.98	0.07
E1	GY	4A	AX.89568764	7.43E+08	0.20	-3.31	4.06	0.06
E1	GPM2	4A	AX.89672103	6.02E+08	0.21	-715.71	3.07	0.05
E1	GY	4A	AX.89512247	7.24E+08	0.12	-3.61	3.26	0.05
E1	GPM2	4A	AX.89334772	4.09E+07	0.11	-1001.42	3.40	0.05
E1	GY	4A	AX.89477455	7.22E+08	0.22	-2.77	3.16	0.05
E1	GY	4A	AX.89570705	7.31E+08	0.19	-3.04	3.35	0.05
E1	GY	4A	AX.89635496	7.40E+08	0.17	-3.02	3.09	0.05
E1	GY	4A	AX.89444767	7.26E+08	0.24	-3.38	4.68	0.07
E1	GPM2	4A	AX.89521160	2.03E+08	0.11	-991.09	3.45	0.05
E1	GY	4A	AX.89729945	7.34E+08	0.12	-3.55	3.08	0.05
E1	GPM2	4A	AX.89415139	4.11E+07	0.10	-1141.22	3.98	0.07
E1	GY	4A	AX.89662356	7.26E+08	0.24	-2.96	3.67	0.05
E1	GY	4A	AX.89486310	7.33E+08	0.22	-3.03	3.67	0.06
E1	GPM2	4A	AX.89373580	2.24E+08	0.11	-991.09	3.45	0.05
E1	GY	4A	AX.89632750	7.41E+08	0.23	-3.46	4.76	0.07
E1	GY	4A	AX.89474542	7.26E+08	0.24	-3.21	4.26	0.07
E1	GY	4A	AX.89778290	7.24E+08	0.06	-5.17	3.53	0.06
E1	GY	4A	AX.89778290	7.24E+08	0.06	-5.17	3.53	0.06
E1	GPM2	4A	AX.89336177	4.06E+07	0.11	-1001.42	3.40	0.05
E1	GPS	4A	AX.89420090	7.30E+08	0.16	-1.51	3.12	0.05
E1	GPM2	4A	AX.89701606	4.09E+07	0.10	-1141.22	3.98	0.07
E1	TKW	4A	AX.89740430	7.27E+08	0.14	-1.30	3.49	0.06
E1	TKW	4A	AX.89706134	7.42E+08	0.36	-0.91	3.29	0.05
E1	TKW	4A	AX.89714548	5.97E+08	0.48	-0.85	3.12	0.05
E1	GPM2	4A	AX.89536695	4.19E+07	0.09	-1091.74	3.55	0.06
E1	TKW	4A	AX.89351946	7.42E+08	0.10	-1.37	3.00	0.05
E1	TKW	4A	AX.89637968	7.41E+08	0.35	-0.95	3.51	0.06
E1	GY	4A	AX.89663455	7.22E+08	0.17	-3.27	3.55	0.06
E1	GPM2	4A	AX.89372837	4.11E+07	0.10	-1141.22	3.98	0.07
E1	TKW	4A	AX.89444767	7.26E+08	0.24	-1.08	3.64	0.06
E1	TKW	4A	AX.89376728	7.27E+08	0.22	-1.18	3.97	0.07
E1	TKW	4A	AX.89594656	7.27E+08	0.14	-1.30	3.49	0.06
E1	TKW	4A	AX.89338338	7.43E+08	0.21	-1.12	3.56	0.06
E1	GPM2	4A	AX.89715843	4.04E+07	0.10	-1141.22	3.98	0.07
E1	TKW	4A	AX.89644184	7.33E+08	0.21	-1.03	3.08	0.05
E1	TKW	4A	AX.89584410	7.31E+08	0.27	-1.00	3.41	0.06
E1	TKW	4A	AX.89339840	7.33E+08	0.22	-1.03	3.17	0.05
E1	TKW	4A	AX.89566817	7.26E+08	0.18	-1.13	3.23	0.05
E1	GY	4A	AX.89612127	4.11E+07	0.11	-3.73	3.15	0.05
E1	TKW	4A	AX.89360038	7.33E+08	0.22	-1.03	3.17	0.05

E1	TKW	4A	AX.89661925	6.04E+08	0.39	-0.94	3.58	0.06
E1	TKW	4A	AX.89517951	7.26E+08	0.20	-1.04	3.01	0.05
E1	TKW	4A	AX.89684381	7.40E+08	0.36	-0.91	3.29	0.05
E1	TKW	4A	AX.89442700	7.26E+08	0.18	-1.08	3.01	0.05
E1	GPM2	4A	AX.89614087	4.10E+07	0.11	-1001.42	3.40	0.05
E1	GY	4A	AX.89318590	7.24E+08	0.11	-3.67	3.07	0.05
E1	GY	4A	AX.89729045	7.33E+08	0.07	-5.05	3.80	0.06
E1	GPM2	4A	AX.89739556	4.10E+07	0.11	-1001.42	3.40	0.05
E1	GPM2	4A	AX.89617589	4.11E+07	0.11	-1001.42	3.40	0.05
E1	GY	4A	AX.89446849	7.21E+08	0.22	-2.94	3.44	0.06
E1	TKW	4A	AX.89642948	7.41E+08	0.35	-0.87	3.04	0.05
E1	GY	4A	AX.89352927	7.20E+08	0.17	-3.02	3.10	0.05
E1	TKW	4A	AX.89376272	7.26E+08	0.18	-1.08	3.01	0.05
E1	TKW	4A	AX.89467077	7.42E+08	0.12	-1.33	3.16	0.05
E1	TKW	4A	AX.89459794	7.40E+08	0.14	-1.36	3.84	0.06
E1	GY	4A	AX.89719476	7.41E+08	0.27	-3.44	5.19	0.08
E1	TKW	4A	AX.89317315	7.34E+08	0.14	-1.47	4.31	0.07
E1	GPM2	4A	AX.89622027	4.10E+07	0.10	-1141.22	3.98	0.07
E1	TKW	4A	AX.89426124	6.04E+08	0.43	-0.89	3.32	0.06
E1	GPM2	4A	AX.89450482	4.11E+07	0.11	-1001.42	3.40	0.05
E1	TKW	4A	AX.89310768	7.28E+08	0.21	-1.07	3.30	0.05
E1	TKW	4A	AX.89333240	7.26E+08	0.18	-1.08	3.01	0.05
E1	TKW	4A	AX.89395605	6.04E+08	0.43	-0.90	3.44	0.06
E1	GPM2	4A	AX.89602523	6.04E+08	0.32	626.11	3.09	0.05
E1	TKW	4A	AX.89336009	7.40E+08	0.08	-1.56	3.23	0.05
E1	GY	4A	AX.89678810	7.25E+08	0.20	-2.98	3.40	0.05
E1	TKW	4A	AX.89446643	7.40E+08	0.36	-0.91	3.29	0.05
E1	GPM2	4A	AX.89636971	4.04E+07	0.11	-1001.42	3.40	0.05
E1	TKW	4A	AX.89674472	7.42E+08	0.10	-1.37	3.00	0.05
E1	GPM2	4A	AX.89565275	4.09E+07	0.10	-1141.22	3.98	0.07
E1	TKW	4A	AX.89387177	7.33E+08	0.20	-1.14	3.60	0.06
E1	GY	4A	AX.89322806	7.39E+08	0.13	-3.42	3.15	0.05
E1	TKW	4A	AX.89554375	7.27E+08	0.23	-1.15	3.92	0.07
E1	TKW	4A	AX.89455084	7.31E+08	0.27	-1.00	3.41	0.06
E1	TKW	4A	AX.89470934	7.33E+08	0.11	-1.39	3.21	0.05
E1	GY	4A	AX.89776312	7.22E+08	0.21	-3.06	3.64	0.06
E1	TKW	4A	AX.89469231	7.30E+08	0.20	-1.04	3.06	0.05
E1	TKW	4A	AX.89686985	7.25E+08	0.18	-1.08	3.01	0.05
E1	TKW	4A	AX.89560573	7.26E+08	0.21	-1.16	3.76	0.06
E1	TKW	4A	AX.89349918	7.40E+08	0.18	-1.42	4.78	0.08
E1	TKW	4A	AX.89763075	7.26E+08	0.18	-1.08	3.01	0.05
E1	TKW	4A	AX.89342690	7.26E+08	0.20	-1.04	3.01	0.05
E1	TKW	4A	AX.89561620	6.04E+08	0.39	-0.94	3.58	0.06
E1	TKW	4A	AX.89476261	7.34E+08	0.14	-1.32	3.66	0.06
E1	TKW	4A	AX.89446059	7.40E+08	0.16	-1.40	4.40	0.08
E1	TKW	4A	AX.89563022	7.33E+08	0.22	-1.03	3.17	0.05
E1	TKW	4A	AX.89353399	7.26E+08	0.20	-1.04	3.01	0.05
E1	TKW	4A	AX.89462343	7.27E+08	0.27	-0.94	3.06	0.05
E1	TKW	4A	AX.89370040	7.42E+08	0.10	-1.37	3.00	0.05
E1	TKW	4A	AX.89362942	7.26E+08	0.23	-0.99	3.08	0.05

E1	TKW	4A	AX.89751035	7.27E+08	0.21	-1.07	3.25	0.05
E1	TKW	4A	AX.89327430	7.26E+08	0.28	-1.00	3.43	0.06
E1	TKW	4A	AX.89327540	7.26E+08	0.20	-1.04	3.01	0.05
E1	TKW	4A	AX.89595323	7.26E+08	0.20	-1.09	3.22	0.05
E1	TKW	4A	AX.89537546	7.31E+08	0.16	-1.16	3.11	0.05
E1	TKW	4A	AX.89335287	7.34E+08	0.14	-1.18	3.03	0.05
E1	TKW	4A	AX.89682870	7.40E+08	0.12	-1.40	3.44	0.06
E1	TKW	4A	AX.89453985	7.34E+08	0.18	-1.20	3.65	0.06
E1	TKW	4A	AX.89310426	7.41E+08	0.35	-0.94	3.48	0.06
E1	TKW	4A	AX.89333861	7.26E+08	0.18	-1.08	3.01	0.05
E1	TKW	4A	AX.89770697	7.26E+08	0.20	-1.06	3.08	0.05
E1	TKW	4A	AX.89429351	7.27E+08	0.27	-0.94	3.06	0.05
E1	TKW	4A	AX.89738968	7.34E+08	0.19	-1.32	4.39	0.08
E1	GPM2	4A	AX.89428920	4.09E+07	0.10	-1141.22	3.98	0.07
E1	TKW	4A	AX.89518458	7.33E+08	0.22	-1.03	3.17	0.05
E1	TKW	4A	AX.89731769	7.27E+08	0.22	-1.18	3.97	0.07
E1	TKW	4A	AX.89594507	7.31E+08	0.27	-1.00	3.41	0.06
E1	TKW	4A	AX.89375502	7.40E+08	0.17	-1.46	4.95	0.09
E1	TKW	4A	AX.89486310	7.33E+08	0.22	-1.03	3.17	0.05
E1	TKW	4A	AX.89360065	7.27E+08	0.27	-0.94	3.06	0.05
E1	GPM2	4A	AX.89544078	4.11E+07	0.10	-1141.22	3.98	0.07
E1	TKW	4A	AX.89377182	7.27E+08	0.21	-1.07	3.30	0.05
E1	TKW	4A	AX.89747478	7.41E+08	0.36	-0.91	3.29	0.05
E1	TKW	4A	AX.89612888	7.26E+08	0.20	-1.05	3.10	0.05
E1	TKW	4A	AX.89686188	7.40E+08	0.10	-1.60	3.95	0.07
E1	TKW	4A	AX.89662356	7.26E+08	0.24	-1.04	3.34	0.05
E1	TKW	4A	AX.89346010	7.32E+08	0.26	-0.98	3.22	0.05
E1	GY	4A	AX.89776458	7.08E+08	0.16	-3.16	3.15	0.06
E1	GY	4A	AX.89371746	7.31E+08	0.27	-3.77	6.06	0.10
E1	TKW	4A	AX.89589414	7.30E+08	0.19	-1.13	3.31	0.05
E1	TKW	4A	AX.89775295	7.26E+08	0.20	-1.04	3.01	0.05
E1	GPM2	4A	AX.89760720	1.76E+07	0.27	803.14	4.35	0.08
E1	GY	4A	AX.89373507	7.41E+08	0.30	-2.68	3.49	0.05
E1	TKW	4A	AX.89341689	7.42E+08	0.15	-1.43	4.28	0.07
E1	TKW	4A	AX.89564765	7.39E+08	0.13	-1.43	3.99	0.07
E1	TKW	4A	AX.89417560	6.04E+08	0.43	-0.90	3.44	0.06
E1	TKW	4A	AX.89579752	7.43E+08	0.20	-1.14	3.48	0.06
E1	TKW	4A	AX.89328354	7.39E+08	0.16	-1.35	4.05	0.07
E1	TKW	4A	AX.89474542	7.26E+08	0.24	-1.02	3.28	0.05
E1	TKW	4A	AX.89565646	7.43E+08	0.21	-1.12	3.56	0.06
E1	TKW	4A	AX.89624256	7.44E+08	0.20	-1.11	3.36	0.06
E1	TKW	4A	AX.89465835	7.41E+08	0.35	-0.88	3.07	0.05
E1	TKW	4A	AX.89499407	7.30E+08	0.15	-1.16	3.07	0.05
E1	TKW	4A	AX.89457592	7.26E+08	0.24	-0.98	3.02	0.05
E1	TKW	4A	AX.89364766	7.26E+08	0.24	-1.04	3.34	0.05
E1	TKW	4A	AX.89741910	7.39E+08	0.09	-1.47	3.05	0.05
E1	TKW	4A	AX.89610216	7.30E+08	0.20	-1.18	3.75	0.06
E1	TKW	4A	AX.89639898	7.44E+08	0.21	-1.17	3.81	0.06
E1	TKW	4A	AX.89405213	7.39E+08	0.14	-1.23	3.16	0.05
E1	TKW	4A	AX.89690364	7.40E+08	0.36	-0.91	3.29	0.05

E1	TKW	4A	AX.89599133	7.40E+08	0.34	-0.91	3.24	0.05
E1	GPM2	4A	AX.89746072	4.09E+07	0.11	-1001.42	3.40	0.05
E1	TKW	4A	AX.89439391	7.42E+08	0.10	-1.37	3.00	0.05
E1	TKW	4A	AX.89371746	7.31E+08	0.27	-1.00	3.41	0.06
E1	GPM2	4A	AX.89598986	4.11E+07	0.10	-1141.22	3.98	0.07
E1	TKW	4A	AX.89448363	7.40E+08	0.13	-1.55	4.57	0.08
E1	TKW	4A	AX.89582475	7.40E+08	0.17	-1.34	4.15	0.07
E1	TKW	4A	AX.89529222	7.26E+08	0.24	-1.02	3.28	0.05
E1	TKW	4A	AX.89677918	7.28E+08	0.27	-0.94	3.06	0.05
E1	TKW	4A	AX.89423722	6.04E+08	0.38	-0.92	3.41	0.06
E1	TKW	4A	AX.89372187	7.26E+08	0.15	-1.29	3.60	0.06
E1	TKW	4A	AX.89380583	7.27E+08	0.15	-1.21	3.23	0.05
E1	TKW	4A	AX.89367604	7.25E+08	0.18	-1.08	3.01	0.05
E1	TKW	4A	AX.89539186	7.43E+08	0.19	-1.10	3.23	0.05
E1	TKW	4A	AX.89363602	6.04E+08	0.43	-0.90	3.44	0.06
E1	TKW	4A	AX.89621604	7.31E+08	0.27	-1.10	3.97	0.07
E1	TKW	4A	AX.89369162	7.26E+08	0.20	-1.04	3.01	0.05
E1	TKW	4A	AX.89530069	7.40E+08	0.17	-1.34	4.15	0.07
E1	TKW	4A	AX.89680594	7.26E+08	0.18	-1.08	3.01	0.05
E1	TKW	4A	AX.89537167	7.39E+08	0.13	-1.33	3.51	0.06
E1	TKW	4A	AX.89610678	7.26E+08	0.20	-1.04	3.01	0.05
E1	TKW	4A	AX.89412618	7.34E+08	0.14	-1.18	3.03	0.05
E1	TKW	4A	AX.89594512	5.97E+08	0.48	-0.85	3.12	0.05
E1	GY	4A	AX.89537376	7.24E+08	0.07	-4.93	3.65	0.06
E1	TKW	4A	AX.89522321	7.42E+08	0.10	-1.37	3.00	0.05
E1	TKW	4A	AX.89744361	7.26E+08	0.20	-1.09	3.22	0.05
E1	TKW	4A	AX.89483630	7.41E+08	0.36	-0.91	3.29	0.05
E1	TKW	4A	AX.89520266	7.26E+08	0.20	-1.04	3.01	0.05
E1	TKW	4A	AX.89310663	7.28E+08	0.09	-1.51	3.32	0.05
E1	TKW	4A	AX.89415948	7.27E+08	0.26	-1.19	4.44	0.08
E1	TKW	4A	AX.86166405	7.26E+08	0.20	-1.04	3.01	0.05
E1	TKW	4A	AX.89762249	7.30E+08	0.16	-1.21	3.36	0.06
E1	TKW	4A	AX.89721231	7.33E+08	0.11	-1.45	3.54	0.06
E1	TKW	4A	AX.89505951	7.26E+08	0.18	-1.08	3.01	0.05
E1	TKW	4A	AX.89735480	7.40E+08	0.36	-0.91	3.29	0.05
E1	TKW	4A	AX.89690177	7.26E+08	0.18	-1.08	3.01	0.05
E1	TKW	4A	AX.89767812	7.31E+08	0.27	-1.04	3.60	0.06
E2	GY	4A	AX.89755668	5.95E+07	0.08	-3.38	3.02	0.04
E2	SPM2	4A	AX.89611126	7.30E+08	0.17	15.54	3.07	0.05
E2	GY	4A	AX.89329174	7.22E+08	0.18	-3.01	4.31	0.07
E2	GY	4A	AX.89400490	7.24E+08	0.06	-4.62	3.75	0.05
E2	GPS	4A	AX.89632483	5.45E+08	0.33	1.49	3.34	0.06
E2	GY	4A	AX.89423999	7.24E+08	0.06	-4.92	4.13	0.06
E2	GY	4A	AX.89330373	7.26E+08	0.11	-3.28	3.45	0.05
E2	GY	4A	AX.89401350	7.21E+08	0.15	-2.66	3.14	0.05
E2	GY	4A	AX.86168551	7.20E+08	0.18	-2.43	3.01	0.05
E2	GY	4A	AX.89437107	7.21E+08	0.15	-2.66	3.14	0.05
E2	GY	4A	AX.89437107	7.21E+08	0.15	-2.66	3.14	0.05
E2	GY	4A	AX.89727342	7.23E+08	0.06	-4.05	3.18	0.04
E2	SPM2	4A	AX.89498000	5.84E+08	0.07	25.13	3.76	0.06

E2	GY	4A	AX.89331953	7.19E+08	0.19	-2.50	3.20	0.05
E2	GY	4A	AX.89592533	7.26E+08	0.10	-3.21	3.19	0.04
E2	GY	4A	AX.89332573	7.26E+08	0.13	-3.18	3.67	0.05
E2	GY	4A	AX.89332573	7.26E+08	0.13	-3.18	3.67	0.05
E2	GY	4A	AX.89332687	7.34E+08	0.40	-2.07	3.42	0.07
E2	SPM2	4A	AX.89777494	6.10E+08	0.31	-14.27	3.77	0.06
E2	GY	4A	AX.89333298	7.26E+08	0.13	-3.18	3.67	0.05
E2	GY	4A	AX.89473221	7.34E+08	0.40	-2.01	3.23	0.06
E2	SPM2	4A	AX.89641499	6.14E+08	0.10	24.05	4.31	0.08
E2	SPM2	4A	AX.89691321	7.30E+08	0.13	18.37	3.36	0.06
E2	GY	4A	AX.89404258	7.16E+08	0.08	-3.48	3.03	0.04
E2	GY	4A	AX.89404258	7.16E+08	0.08	-3.48	3.03	0.04
E2	GY	4A	AX.89474564	7.26E+08	0.11	-3.57	4.05	0.05
E2	GY	4A	AX.89639515	7.24E+08	0.07	-4.12	3.67	0.05
E2	GY	4A	AX.89380919	7.25E+08	0.06	-4.62	3.75	0.05
E2	GY	4A	AX.89579660	7.26E+08	0.11	-3.98	4.98	0.08
E2	GY	4A	AX.89579660	7.26E+08	0.11	-3.98	4.98	0.08
E2	SPM2	4A	AX.89589414	7.30E+08	0.19	14.92	3.02	0.05
E2	TKW	4A	AX.89483630	7.41E+08	0.36	-1.05	3.76	0.06
E2	GPS	4A	AX.89702776	5.44E+08	0.33	1.50	3.41	0.06
E2	GY	4A	AX.89336841	7.25E+08	0.07	-4.53	4.31	0.06
E2	GPS	4A	AX.89601326	5.46E+08	0.33	1.49	3.34	0.06
E2	GPS	4A	AX.89573483	5.66E+08	0.44	1.40	3.29	0.06
E2	GPS	4A	AX.89445978	5.45E+08	0.33	1.49	3.34	0.06
E2	SPM2	4A	AX.89439836	7.30E+08	0.09	21.03	3.17	0.05
E2	GPS	4A	AX.89316300	5.66E+08	0.45	1.35	3.09	0.06
E2	GY	4A	AX.89409546	7.23E+08	0.06	-4.05	3.18	0.04
E2	GY	4A	AX.89526893	7.24E+08	0.06	-4.68	4.08	0.06
E2	GY	4A	AX.89548890	7.25E+08	0.11	-3.34	3.64	0.05
E2	GPS	4A	AX.89379588	5.95E+08	0.35	1.55	3.67	0.06
E2	GPS	4A	AX.89754071	5.44E+08	0.34	1.47	3.29	0.06
E2	GPS	4A	AX.89442064	5.48E+08	0.33	1.59	3.73	0.07
E2	GPS	4A	AX.89692052	5.46E+08	0.33	1.49	3.34	0.06
E2	GPS	4A	AX.89504360	5.95E+08	0.35	1.55	3.67	0.06
E2	SPM2	4A	AX.89333648	7.30E+08	0.10	19.75	3.07	0.05
E2	GPS	4A	AX.89627932	5.94E+08	0.35	1.55	3.67	0.06
E2	GY	4A	AX.89491600	7.20E+08	0.21	-2.60	3.75	0.06
E2	GY	4A	AX.89491600	7.20E+08	0.21	-2.60	3.75	0.06
E2	GY	4A	AX.89452318	7.23E+08	0.06	-4.62	3.75	0.05
E2	SPM2	4A	AX.89777321	7.30E+08	0.13	17.20	3.00	0.05
E2	GPS	4A	AX.89648991	5.45E+08	0.33	1.43	3.11	0.06
E2	GY	4A	AX.89749239	7.19E+08	0.17	-2.94	3.97	0.07
E2	GY	4A	AX.86160988	7.42E+08	0.14	-2.80	3.28	0.04
E2	GY	4A	AX.86160988	7.42E+08	0.14	-2.80	3.28	0.04
E2	GPS	4A	AX.89702579	5.46E+08	0.33	1.66	4.02	0.07
E2	GPS	4A	AX.89557186	5.46E+08	0.32	1.61	3.77	0.07
E2	GY	4A	AX.89637772	7.21E+08	0.17	-2.94	3.97	0.07
E2	GPS	4A	AX.89453672	5.67E+08	0.45	1.35	3.09	0.06
E2	GY	4A	AX.89468920	7.22E+08	0.17	-2.94	3.97	0.07
E2	GY	4A	AX.89468920	7.22E+08	0.17	-2.94	3.97	0.07

E2	GPS	4A	AX.89408403	5.95E+08	0.35	1.55	3.67	0.06
E2	GY	4A	AX.89376538	7.20E+08	0.19	-2.71	3.70	0.06
E2	GY	4A	AX.89376538	7.20E+08	0.19	-2.71	3.70	0.06
E2	GPS	4A	AX.89684700	5.45E+08	0.33	1.49	3.34	0.06
E2	GY	4A	AX.89446861	7.33E+08	0.11	-4.25	5.55	0.07
E2	GY	4A	AX.89446861	7.33E+08	0.11	-4.25	5.55	0.07
E2	GY	4A	AX.89435512	7.20E+08	0.15	-2.76	3.29	0.05
E2	GY	4A	AX.89435512	7.20E+08	0.15	-2.76	3.29	0.05
E2	GPS	4A	AX.89478975	5.62E+08	0.48	1.39	3.29	0.06
E2	GY	4A	AX.86165449	7.26E+08	0.10	-3.61	3.93	0.06
E2	GY	4A	AX.86165449	7.26E+08	0.10	-3.61	3.93	0.06
E2	GY	4A	AX.89447138	7.20E+08	0.17	-2.94	3.97	0.07
E2	GY	4A	AX.89447138	7.20E+08	0.17	-2.94	3.97	0.07
E2	GY	4A	AX.89587577	7.19E+08	0.22	-2.76	4.21	0.07
E2	GY	4A	AX.89587577	7.19E+08	0.22	-2.76	4.21	0.07
E2	GPS	4A	AX.89324372	5.95E+08	0.42	1.35	3.08	0.04
E2	GY	4A	AX.89529986	7.37E+08	0.08	-3.56	3.08	0.04
E2	GY	4A	AX.89483845	7.23E+08	0.07	-4.83	4.58	0.06
E2	GY	4A	AX.89483845	7.23E+08	0.07	-4.83	4.58	0.06
E2	GPS	4A	AX.89480796	5.44E+08	0.33	1.50	3.41	0.06
E2	GY	4A	AX.86168547	7.20E+08	0.18	-2.43	3.01	0.05
E2	GY	4A	AX.86168547	7.20E+08	0.18	-2.43	3.01	0.05
E2	GY	4A	AX.86168548	7.20E+08	0.18	-2.43	3.01	0.05
E2	GY	4A	AX.86168548	7.20E+08	0.18	-2.43	3.01	0.05
E2	GY	4A	AX.86168551	7.20E+08	0.18	-2.43	3.01	0.05
E2	GY	4A	AX.86168553	7.20E+08	0.18	-2.43	3.01	0.05
E2	GY	4A	AX.86168553	7.20E+08	0.18	-2.43	3.01	0.05
E2	GY	4A	AX.89685609	7.20E+08	0.17	-2.84	3.81	0.06
E2	GY	4A	AX.89685609	7.20E+08	0.17	-2.84	3.81	0.06
E2	GPS	4A	AX.89409886	5.44E+08	0.34	1.47	3.29	0.06
E2	GPS	4A	AX.89435499	5.95E+08	0.35	1.55	3.67	0.06
E2	GY	4A	AX.89377870	7.24E+08	0.07	-4.83	4.58	0.06
E2	GY	4A	AX.89377870	7.24E+08	0.07	-4.83	4.58	0.06
E2	GY	4A	AX.89495274	7.22E+08	0.17	-2.94	3.97	0.07
E2	GY	4A	AX.89495274	7.22E+08	0.17	-2.94	3.97	0.07
E2	GPS	4A	AX.89335784	5.95E+08	0.35	1.55	3.67	0.06
E2	GY	4A	AX.89448771	7.24E+08	0.06	-4.62	3.75	0.05
E2	GY	4A	AX.89448771	7.24E+08	0.06	-4.62	3.75	0.05
E2	GPS	4A	AX.89560286	5.45E+08	0.33	1.43	3.11	0.06
E2	GY	4A	AX.89331953	7.19E+08	0.19	-2.50	3.20	0.05
E2	GPS	4A	AX.89400740	5.44E+08	0.34	1.47	3.29	0.06
E2	GPS	4A	AX.89513529	5.45E+08	0.33	1.43	3.11	0.06
E2	GPS	4A	AX.89548503	5.45E+08	0.33	1.49	3.34	0.06
E2	GY	4A	AX.89310232	1.05E+08	0.19	-2.61	3.38	0.04
E2	GY	4A	AX.89310232	1.05E+08	0.19	-2.61	3.38	0.04
E2	GPS	4A	AX.89369893	5.66E+08	0.45	1.35	3.09	0.06
E2	GY	4A	AX.89322015	7.39E+08	0.11	-3.11	3.12	0.04
E2	GY	4A	AX.89663245	7.19E+08	0.17	-2.94	3.97	0.07
E2	GY	4A	AX.89663245	7.19E+08	0.17	-2.94	3.97	0.07
E2	GY	4A	AX.89521160	2.03E+08	0.11	-3.00	3.05	0.04

E2	GY	4A	AX.89521160	2.03E+08	0.11	-3.00	3.05	0.04
E2	GY	4A	AX.89712135	7.25E+08	0.06	-4.62	3.75	0.05
E2	GY	4A	AX.89566818	7.24E+08	0.07	-4.83	4.58	0.06
E2	GY	4A	AX.89566818	7.24E+08	0.07	-4.83	4.58	0.06
E2	GPS	4A	AX.89578800	5.45E+08	0.33	1.49	3.34	0.06
E2	GY	4A	AX.89486698	7.42E+07	0.07	-3.81	3.05	0.04
E2	GY	4A	AX.89486698	7.42E+07	0.07	-3.81	3.05	0.04
E2	GPS	4A	AX.89401469	5.67E+08	0.45	1.35	3.09	0.06
E2	GPS	4A	AX.89321081	5.45E+08	0.33	1.49	3.34	0.06
E2	GPS	4A	AX.89324041	5.67E+08	0.45	1.35	3.09	0.06
E2	GPS	4A	AX.89448692	5.67E+08	0.45	1.35	3.09	0.06
E2	GY	4A	AX.89311564	7.21E+08	0.15	-2.66	3.14	0.05
E2	GY	4A	AX.89311564	7.21E+08	0.15	-2.66	3.14	0.05
E2	GY	4A	AX.89738343	1.03E+08	0.19	-2.47	3.08	0.04
E2	GY	4A	AX.89738343	1.03E+08	0.19	-2.47	3.08	0.04
E2	GPS	4A	AX.89555317	5.45E+08	0.33	1.49	3.34	0.06
E2	GPS	4A	AX.89720617	5.95E+08	0.35	1.55	3.67	0.06
E2	GY	4A	AX.89380919	7.25E+08	0.06	-4.62	3.75	0.05
E2	GPM2	4A	AX.89602523	6.04E+08	0.32	569.05	3.47	0.07
E2	GPS	4A	AX.89514361	5.66E+08	0.46	1.34	3.07	0.06
E2	GY	4A	AX.89523678	7.26E+08	0.18	-2.54	3.12	0.04
E2	GPS	4A	AX.89517350	5.45E+08	0.33	1.49	3.34	0.06
E2	GY	4A	AX.89346268	7.34E+08	0.13	-2.88	3.17	0.04
E2	GY	4A	AX.89346268	7.34E+08	0.13	-2.88	3.17	0.04
E2	GPS	4A	AX.89645150	5.47E+08	0.33	1.65	3.98	0.07
E2	GPS	4A	AX.89667681	5.66E+08	0.45	1.42	3.38	0.06
E2	GPS	4A	AX.89752513	5.95E+08	0.35	1.55	3.67	0.06
E2	GY	4A	AX.89313341	7.39E+08	0.12	-3.59	4.26	0.06
E2	GY	4A	AX.89313341	7.39E+08	0.12	-3.59	4.26	0.06
E2	GPS	4A	AX.89428154	5.45E+08	0.33	1.49	3.34	0.06
E2	GY	4A	AX.89524550	7.39E+08	0.11	-3.11	3.12	0.04
E2	GY	4A	AX.89524550	7.39E+08	0.11	-3.11	3.12	0.04
E2	GPS	4A	AX.89731999	5.44E+08	0.34	1.47	3.29	0.06
E2	GY	4A	AX.89430756	7.24E+08	0.06	-4.62	3.75	0.05
E2	GPS	4A	AX.89696836	5.45E+08	0.33	1.49	3.34	0.06
E2	GPS	4A	AX.89349328	5.95E+08	0.35	1.55	3.67	0.06
E2	GY	4A	AX.89525651	7.24E+08	0.07	-4.83	4.58	0.06
E2	GY	4A	AX.89525651	7.24E+08	0.07	-4.83	4.58	0.06
E2	GPS	4A	AX.89693645	5.95E+08	0.35	1.55	3.67	0.06
E2	GY	4A	AX.89383862	7.22E+08	0.17	-2.85	3.77	0.07
E2	GY	4A	AX.89383862	7.22E+08	0.17	-2.85	3.77	0.07
E2	GY	4A	AX.89718118	7.22E+08	0.07	-3.91	3.18	0.04
E2	GY	4A	AX.89718118	7.22E+08	0.07	-3.91	3.18	0.04
E2	GY	4A	AX.89454827	7.21E+08	0.17	-2.85	3.77	0.07
E2	GY	4A	AX.89454827	7.21E+08	0.17	-2.85	3.77	0.07
E2	GY	4A	AX.89421360	7.22E+08	0.17	-2.94	3.97	0.07
E2	GY	4A	AX.89421360	7.22E+08	0.17	-2.94	3.97	0.07
E2	GY	4A	AX.89316812	8.74E+07	0.10	-3.32	3.30	0.05
E2	GY	4A	AX.89316812	8.74E+07	0.10	-3.32	3.30	0.05
E2	GY	4A	AX.89328332	7.39E+08	0.11	-3.11	3.12	0.04

E2	GY	4A	AX.89597931	7.27E+08	0.15	-2.62	3.03	0.04
E2	GY	4A	AX.89597931	7.27E+08	0.15	-2.62	3.03	0.04
E2	GY	4A	AX.89456312	7.20E+08	0.16	-3.13	4.34	0.07
E2	GY	4A	AX.89456312	7.20E+08	0.16	-3.13	4.34	0.07
E2	GY	4A	AX.89749239	7.19E+08	0.17	-2.94	3.97	0.07
E2	GY	4A	AX.89422337	7.21E+08	0.17	-2.66	3.40	0.06
E2	GY	4A	AX.89422337	7.21E+08	0.17	-2.66	3.40	0.06
E2	GY	4A	AX.89671253	7.22E+08	0.16	-3.03	4.11	0.07
E2	GY	4A	AX.89671253	7.22E+08	0.16	-3.03	4.11	0.07
E2	GY	4A	AX.89637772	7.21E+08	0.17	-2.94	3.97	0.07
E2	GY	4A	AX.89602206	7.24E+08	0.06	-4.62	3.75	0.05
E2	GY	4A	AX.89602206	7.24E+08	0.06	-4.62	3.75	0.05
E2	GY	4A	AX.89423359	7.24E+08	0.07	-4.83	4.58	0.06
E2	GY	4A	AX.89423359	7.24E+08	0.07	-4.83	4.58	0.06
E2	GY	4A	AX.89352927	7.20E+08	0.17	-2.84	3.81	0.06
E2	GY	4A	AX.89352927	7.20E+08	0.17	-2.84	3.81	0.06
E2	GY	4A	AX.89529986	7.37E+08	0.08	-3.56	3.08	0.04
E2	GY	4A	AX.89458917	7.35E+07	0.11	-3.11	3.15	0.04
E2	GY	4A	AX.89458917	7.35E+07	0.11	-3.11	3.15	0.04
E2	GY	4A	AX.89459056	7.17E+08	0.23	-2.29	3.18	0.05
E2	GY	4A	AX.89459056	7.17E+08	0.23	-2.29	3.18	0.05
E2	TKW	4A	AX.89426124	6.04E+08	0.44	-0.98	3.51	0.06
E2	GY	4A	AX.89746890	7.25E+08	0.07	-4.38	4.27	0.06
E2	GY	4A	AX.89746890	7.25E+08	0.07	-4.38	4.27	0.06
E2	GY	4A	AX.89320706	7.16E+08	0.08	-3.48	3.03	0.04
E2	GY	4A	AX.89320706	7.16E+08	0.08	-3.48	3.03	0.04
E2	GY	4A	AX.89519742	7.26E+08	0.06	-3.95	3.07	0.04
E2	GY	4A	AX.89519742	7.26E+08	0.06	-3.95	3.07	0.04
E2	GY	4A	AX.89755151	7.19E+08	0.19	-2.50	3.20	0.05
E2	GY	4A	AX.89755151	7.19E+08	0.19	-2.50	3.20	0.05
E2	GPS	4A	AX.89744175	5.44E+08	0.34	1.47	3.29	0.06
E2	GPS	4A	AX.89530586	5.46E+08	0.33	1.49	3.34	0.06
E2	GY	4A	AX.89703275	7.22E+08	0.17	-2.75	3.52	0.06
E2	GY	4A	AX.89703275	7.22E+08	0.17	-2.75	3.52	0.06
E2	GY	4A	AX.89760308	7.21E+08	0.17	-2.94	3.97	0.07
E2	GY	4A	AX.89699439	7.20E+08	0.15	-2.76	3.29	0.05
E2	GY	4A	AX.89699439	7.20E+08	0.15	-2.76	3.29	0.05
E2	GY	4A	AX.89321887	7.23E+08	0.06	-4.05	3.18	0.04
E2	GY	4A	AX.89321887	7.23E+08	0.06	-4.05	3.18	0.04
E2	GY	4A	AX.89625179	7.22E+08	0.17	-2.94	3.97	0.07
E2	GY	4A	AX.89367424	7.22E+08	0.17	-2.92	4.01	0.07
E2	GY	4A	AX.89322015	7.39E+08	0.11	-3.11	3.12	0.04
E2	GY	4A	AX.89520523	7.24E+08	0.06	-4.62	3.75	0.05
E2	GY	4A	AX.89520523	7.24E+08	0.06	-4.62	3.75	0.05
E2	GY	4A	AX.89712135	7.25E+08	0.06	-4.62	3.75	0.05
E2	GY	4A	AX.89729945	7.34E+08	0.12	-3.05	3.20	0.04
E2	GY	4A	AX.89729945	7.34E+08	0.12	-3.05	3.20	0.04
E2	GY	4A	AX.89473221	7.34E+08	0.40	-2.01	3.23	0.06
E2	GY	4A	AX.89404144	7.20E+08	0.19	-2.71	3.70	0.06
E2	GY	4A	AX.89652625	7.30E+08	0.07	-3.78	3.13	0.04

E2	GY	4A	AX.89652625	7.30E+08	0.07	-3.78	3.13	0.04
E2	GY	4A	AX.89756968	7.53E+07	0.11	-3.11	3.15	0.04
E2	GY	4A	AX.89756968	7.53E+07	0.11	-3.11	3.15	0.04
E2	GY	4A	AX.89474564	7.26E+08	0.11	-3.57	4.05	0.05
E2	GY	4A	AX.89392892	4.50E+08	0.12	-2.98	3.11	0.04
E2	GY	4A	AX.89392892	4.50E+08	0.12	-2.98	3.11	0.04
E2	GY	4A	AX.89377454	7.19E+08	0.13	-2.98	3.47	0.06
E2	GY	4A	AX.89592944	7.16E+08	0.08	-3.48	3.03	0.04
E2	GY	4A	AX.89592944	7.16E+08	0.08	-3.48	3.03	0.04
E2	GY	4A	AX.89666696	7.43E+08	0.10	-3.19	3.07	0.04
E2	GY	4A	AX.89324248	7.43E+08	0.15	-2.90	3.55	0.05
E2	GY	4A	AX.89324248	7.43E+08	0.15	-2.90	3.55	0.05
E2	GY	4A	AX.89556734	7.20E+08	0.17	-2.84	3.81	0.06
E2	GY	4A	AX.89556734	7.20E+08	0.17	-2.84	3.81	0.06
E2	GY	4A	AX.89475409	7.26E+08	0.11	-3.50	3.85	0.05
E2	GY	4A	AX.89475409	7.26E+08	0.11	-3.50	3.85	0.05
E2	GY	4A	AX.89523678	7.26E+08	0.18	-2.54	3.12	0.04
E2	GY	4A	AX.89606149	7.26E+08	0.13	-3.18	3.67	0.05
E2	GY	4A	AX.89606149	7.26E+08	0.13	-3.18	3.67	0.05
E2	GY	4A	AX.89325572	7.26E+08	0.08	-3.76	3.43	0.05
E2	GY	4A	AX.89325572	7.26E+08	0.08	-3.76	3.43	0.05
E2	GY	4A	AX.89430756	7.24E+08	0.06	-4.62	3.75	0.05
E2	GY	4A	AX.89382815	7.25E+08	0.09	-5.24	6.78	0.10
E2	GY	4A	AX.89382815	7.25E+08	0.09	-5.24	6.78	0.10
E2	GY	4A	AX.89326464	7.17E+08	0.07	-3.94	3.57	0.05
E2	GY	4A	AX.89326464	7.17E+08	0.07	-3.94	3.57	0.05
E2	GY	4A	AX.89464835	1.48E+08	0.19	-2.55	3.35	0.04
E2	GY	4A	AX.89464835	1.48E+08	0.19	-2.55	3.35	0.04
E2	GY	4A	AX.89752763	7.21E+08	0.15	-2.66	3.14	0.05
E2	GY	4A	AX.89752763	7.21E+08	0.15	-2.66	3.14	0.05
E2	GY	4A	AX.89336841	7.25E+08	0.07	-4.53	4.31	0.06
E2	GY	4A	AX.89643739	7.24E+08	0.07	-4.83	4.58	0.06
E2	GY	4A	AX.89643739	7.24E+08	0.07	-4.83	4.58	0.06
E2	GY	4A	AX.89327236	7.20E+08	0.18	-2.76	3.76	0.06
E2	GY	4A	AX.89327236	7.20E+08	0.18	-2.76	3.76	0.06
E2	GY	4A	AX.89608614	7.26E+08	0.07	-4.54	4.37	0.06
E2	GY	4A	AX.89608614	7.26E+08	0.07	-4.54	4.37	0.06
E2	GY	4A	AX.89361375	7.21E+08	0.13	-3.10	3.76	0.06
E2	GY	4A	AX.89361375	7.21E+08	0.13	-3.10	3.76	0.06
E2	GY	4A	AX.89514865	7.20E+08	0.15	-2.76	3.29	0.05
E2	GY	4A	AX.89514865	7.20E+08	0.15	-2.76	3.29	0.05
E2	GPS	4A	AX.89375980	5.45E+08	0.33	1.49	3.34	0.06
E2	GY	4A	AX.89766987	7.33E+08	0.13	-3.18	3.73	0.04
E2	GY	4A	AX.89766987	7.33E+08	0.13	-3.18	3.73	0.04
E2	GY	4A	AX.89479076	6.07E+07	0.11	-3.18	3.26	0.04
E2	GY	4A	AX.89479076	6.07E+07	0.11	-3.18	3.26	0.04
E2	GY	4A	AX.89681679	7.19E+08	0.24	-2.49	3.75	0.06
E2	GY	4A	AX.89681679	7.19E+08	0.24	-2.49	3.75	0.06
E2	GY	4A	AX.89328332	7.39E+08	0.11	-3.11	3.12	0.04
E2	GPS	4A	AX.89738697	5.44E+08	0.33	1.50	3.41	0.06

E2	GY	4A	AX.89398873	7.21E+08	0.05	-5.30	4.42	0.06
E2	GY	4A	AX.89398873	7.21E+08	0.05	-5.30	4.42	0.06
E2	GY	4A	AX.89755668	5.95E+07	0.08	-3.38	3.02	0.04
E2	GY	4A	AX.89329174	7.22E+08	0.18	-3.01	4.31	0.07
E2	GY	4A	AX.89729045	7.33E+08	0.07	-4.11	3.63	0.04
E2	GPS	4A	AX.89763642	5.67E+08	0.45	1.35	3.09	0.06
E2	GY	4A	AX.89400490	7.24E+08	0.06	-4.62	3.75	0.05
E2	GY	4A	AX.89423999	7.24E+08	0.06	-4.92	4.13	0.06
E2	GY	4A	AX.89330373	7.26E+08	0.11	-3.28	3.45	0.05
E2	GY	4A	AX.89401350	7.21E+08	0.15	-2.66	3.14	0.05
E2	GY	4A	AX.89722710	7.26E+08	0.13	-3.18	3.67	0.05
E2	GY	4A	AX.89746453	7.21E+08	0.17	-2.66	3.40	0.06
E2	GY	4A	AX.89703146	7.22E+08	0.16	-2.64	3.18	0.05
E2	GY	4A	AX.89727342	7.23E+08	0.06	-4.05	3.18	0.04
E2	GY	4A	AX.89710159	7.24E+08	0.07	-4.22	4.00	0.05
E2	GY	4A	AX.89592533	7.26E+08	0.10	-3.21	3.19	0.04
E2	GY	4A	AX.89760308	7.21E+08	0.17	-2.94	3.97	0.07
E2	GPS	4A	AX.89452428	5.95E+08	0.35	1.55	3.67	0.06
E2	GY	4A	AX.89625179	7.22E+08	0.17	-2.94	3.97	0.07
E2	GY	4A	AX.89367424	7.22E+08	0.17	-2.92	4.01	0.07
E2	GY	4A	AX.89332687	7.34E+08	0.40	-2.07	3.42	0.07
E2	GY	4A	AX.89748469	7.21E+08	0.17	-2.66	3.40	0.06
E2	GY	4A	AX.89711147	7.39E+08	0.18	-2.73	3.66	0.05
E2	GY	4A	AX.89711147	7.39E+08	0.18	-2.73	3.66	0.05
E2	GY	4A	AX.89704631	7.26E+08	0.13	-3.82	5.21	0.08
E2	GY	4A	AX.89778290	7.24E+08	0.06	-4.05	3.18	0.04
E2	GY	4A	AX.89778290	7.24E+08	0.06	-4.05	3.18	0.04
E2	GY	4A	AX.89333298	7.26E+08	0.13	-3.18	3.67	0.05
E2	GPS	4A	AX.89327978	5.94E+08	0.42	1.48	3.60	0.06
E2	GPS	4A	AX.89323805	5.95E+08	0.34	1.69	4.21	0.07
E2	GY	4A	AX.89709003	7.43E+08	0.09	-3.29	3.01	0.04
E2	GY	4A	AX.89404144	7.20E+08	0.19	-2.71	3.70	0.06
E2	GPS	4A	AX.89333453	5.45E+08	0.33	1.49	3.34	0.06
E2	GY	4A	AX.89369165	7.22E+08	0.16	-3.05	4.07	0.07
E2	GY	4A	AX.89369165	7.22E+08	0.16	-3.05	4.07	0.07
E2	GY	4A	AX.89486973	7.18E+08	0.24	-2.30	3.25	0.05
E2	GY	4A	AX.89404874	7.25E+08	0.16	-2.93	3.83	0.05
E2	GY	4A	AX.89404874	7.25E+08	0.16	-2.93	3.83	0.05
E2	GPS	4A	AX.89329894	5.45E+08	0.33	1.49	3.34	0.06
E2	GY	4A	AX.89639515	7.24E+08	0.07	-4.12	3.67	0.05
E2	GY	4A	AX.89775815	7.24E+08	0.07	-4.83	4.58	0.06
E2	GY	4A	AX.89369902	7.20E+08	0.17	-2.94	3.97	0.07
E2	GY	4A	AX.89369902	7.20E+08	0.17	-2.94	3.97	0.07
E2	GY	4A	AX.89666696	7.43E+08	0.10	-3.19	3.07	0.04
E2	TKW	4A	AX.89565646	7.43E+08	0.21	-1.26	3.85	0.07
E2	GPS	4A	AX.89511314	5.67E+08	0.45	1.35	3.09	0.06
E2	GY	4A	AX.89462896	7.39E+08	0.08	-3.63	3.30	0.04
E2	GPS	4A	AX.89514281	5.71E+08	0.31	1.70	4.06	0.07
E2	GY	4A	AX.89618055	7.25E+08	0.08	-4.54	4.79	0.07
E2	GY	4A	AX.89608513	3.28E+08	0.11	-3.00	3.05	0.04

E2	GPS	4A	AX.89651803	5.46E+08	0.33	1.49	3.34	0.06
E2	TKW	4A	AX.89622319	7.41E+08	0.26	-1.04	3.20	0.05
E2	GPS	4A	AX.89533645	5.95E+08	0.42	1.35	3.08	0.04
E2	GPS	4A	AX.89606898	5.67E+08	0.45	1.35	3.09	0.06
E2	GY	4A	AX.89347361	7.24E+08	0.06	-4.05	3.18	0.04
E2	GPS	4A	AX.89630921	5.44E+08	0.34	1.47	3.29	0.06
E2	GPS	4A	AX.89334966	5.45E+08	0.33	1.43	3.11	0.06
E2	TKW	4A	AX.89561620	6.04E+08	0.39	-0.96	3.28	0.06
E2	GPS	4A	AX.89665310	5.52E+08	0.33	1.65	3.98	0.07
E2	GY	4A	AX.89450476	7.26E+08	0.11	-4.41	5.79	0.09
E2	GY	4A	AX.89450476	7.26E+08	0.11	-4.41	5.79	0.09
E2	GPS	4A	AX.89724741	5.95E+08	0.42	1.35	3.08	0.04
E2	GY	4A	AX.89663455	7.22E+08	0.17	-2.85	3.84	0.06
E2	GY	4A	AX.89663455	7.22E+08	0.17	-2.85	3.84	0.06
E2	GPS	4A	AX.89639961	6.04E+08	0.45	-1.53	3.88	0.06
E2	GPS	4A	AX.89389750	5.95E+08	0.35	1.55	3.67	0.06
E2	GPS	4A	AX.89584905	5.45E+08	0.33	1.43	3.11	0.06
E2	GPS	4A	AX.89537438	5.50E+08	0.34	1.39	3.01	0.05
E2	GPS	4A	AX.89673844	5.67E+08	0.45	1.35	3.09	0.06
E2	GPS	4A	AX.89768355	5.95E+08	0.42	1.35	3.08	0.04
E2	GPS	4A	AX.89520114	5.67E+08	0.45	1.35	3.09	0.06
E2	GY	4A	AX.89373580	2.24E+08	0.11	-3.00	3.05	0.04
E2	GY	4A	AX.89409546	7.23E+08	0.06	-4.05	3.18	0.04
E2	GPS	4A	AX.89443410	5.66E+08	0.45	1.35	3.09	0.06
E2	GPS	4A	AX.89551317	5.95E+08	0.35	1.55	3.67	0.06
E2	GPS	4A	AX.89501788	5.42E+08	0.30	1.46	3.08	0.05
E2	GY	4A	AX.89526893	7.24E+08	0.06	-4.68	4.08	0.06
E2	GPS	4A	AX.89377059	5.45E+08	0.33	1.49	3.34	0.06
E2	GY	4A	AX.89548890	7.25E+08	0.11	-3.34	3.64	0.05
E2	GPS	4A	AX.89604256	5.50E+08	0.33	1.59	3.73	0.07
E2	GPS	4A	AX.89650725	5.62E+08	0.45	1.33	3.01	0.06
E2	GPS	4A	AX.89421235	5.95E+08	0.35	1.55	3.67	0.06
E2	GPS	4A	AX.89739123	5.44E+08	0.33	1.50	3.41	0.06
E2	GPS	4A	AX.89591615	5.95E+08	0.35	1.55	3.67	0.06
E2	GY	4A	AX.89452318	7.23E+08	0.06	-4.62	3.75	0.05
E2	GY	4A	AX.89565539	7.19E+08	0.22	-2.57	3.74	0.06
E2	GY	4A	AX.89565539	7.19E+08	0.22	-2.57	3.74	0.06
E2	GY	4A	AX.89516106	7.24E+08	0.19	-2.42	3.06	0.04
E2	GY	4A	AX.89516106	7.24E+08	0.19	-2.42	3.06	0.04
E2	GY	4A	AX.89777958	7.24E+08	0.07	-4.43	3.93	0.05
E2	GPS	4A	AX.89673466	5.50E+08	0.33	1.65	3.98	0.07
E2	GY	4A	AX.89422048	7.31E+08	0.20	-2.44	3.18	0.04
E2	GPS	4A	AX.89473367	5.95E+08	0.35	1.55	3.67	0.06
E2	GY	4A	AX.89777038	7.26E+08	0.15	-2.88	3.50	0.05
E2	GY	4A	AX.89777038	7.26E+08	0.15	-2.88	3.50	0.05
E2	GY	4A	AX.89695704	7.21E+08	0.07	-3.67	3.12	0.05
E2	GY	4A	AX.89695704	7.21E+08	0.07	-3.67	3.12	0.05
E2	TKW	4A	AX.89568764	7.43E+08	0.20	-1.15	3.24	0.05
E2	GPS	4A	AX.89573595	5.45E+08	0.33	1.49	3.34	0.06
E2	GPS	4A	AX.89639038	5.66E+08	0.42	1.46	3.53	0.07

E2	TKW	4A	AX.89637968	7.41E+08	0.35	-1.12	4.17	0.07
E2	GPS	4A	AX.89505471	5.52E+08	0.33	1.65	3.98	0.07
E2	GPS	4A	AX.89376001	5.45E+08	0.33	1.43	3.11	0.06
E2	GY	4A	AX.89487527	7.33E+08	0.13	-2.86	3.11	0.04
E2	GY	4A	AX.89487527	7.33E+08	0.13	-2.86	3.11	0.04
E2	GPS	4A	AX.89570818	5.95E+08	0.35	1.55	3.67	0.06
E2	GPS	4A	AX.89606973	5.45E+08	0.33	1.43	3.11	0.06
E2	GPS	4A	AX.89489530	5.44E+08	0.34	1.47	3.29	0.06
E2	GPS	4A	AX.89327806	6.04E+08	0.34	1.47	3.31	0.05
E2	GPS	4A	AX.89583512	5.67E+08	0.44	1.47	3.57	0.07
E2	GPS	4A	AX.89741774	5.94E+08	0.42	1.48	3.60	0.06
E2	GPS	4A	AX.89733776	5.66E+08	0.45	1.35	3.09	0.06
E2	GPS	4A	AX.89408916	5.45E+08	0.33	1.49	3.34	0.06
E2	GY	4A	AX.89595328	7.33E+08	0.15	-2.74	3.20	0.04
E2	GY	4A	AX.89595328	7.33E+08	0.15	-2.74	3.20	0.04
E2	GY	4A	AX.89670415	7.22E+08	0.17	-2.94	3.97	0.07
E2	GPS	4A	AX.89591247	5.51E+08	0.30	1.47	3.10	0.06
E2	GPS	4A	AX.89465166	5.95E+08	0.42	1.35	3.08	0.04
E2	GPS	4A	AX.89694564	5.45E+08	0.33	1.49	3.34	0.06
E2	GPS	4A	AX.89486050	5.45E+08	0.33	1.43	3.11	0.06
E2	GPS	4A	AX.89714181	5.46E+08	0.33	1.49	3.34	0.06
E2	GY	4A	AX.89771229	7.20E+08	0.18	-2.56	3.26	0.05
E2	GY	4A	AX.89771229	7.20E+08	0.18	-2.56	3.26	0.05
E2	GPS	4A	AX.89413506	5.67E+08	0.45	1.35	3.09	0.06
E2	GY	4A	AX.89746453	7.21E+08	0.17	-2.66	3.40	0.06
E2	GY	4A	AX.89342418	7.18E+08	0.22	-2.29	3.10	0.05
E2	GY	4A	AX.89342418	7.18E+08	0.22	-2.29	3.10	0.05
E2	GY	4A	AX.89698306	7.14E+08	0.09	-3.24	3.08	0.04
E2	GY	4A	AX.89698306	7.14E+08	0.09	-3.24	3.08	0.04
E2	GY	4A	AX.89576142	7.21E+08	0.15	-2.66	3.14	0.05
E2	GPS	4A	AX.89546298	5.45E+08	0.33	1.49	3.34	0.06
E2	GPS	4A	AX.89672169	5.46E+08	0.32	1.61	3.77	0.07
E2	GPS	4A	AX.89677528	5.45E+08	0.33	1.49	3.34	0.06
E2	GPS	4A	AX.89522052	5.66E+08	0.45	1.35	3.09	0.06
E2	GPS	4A	AX.89762751	5.49E+08	0.34	1.45	3.23	0.06
E2	GPS	4A	AX.89583948	5.45E+08	0.33	1.49	3.34	0.06
E2	GY	4A	AX.89710159	7.24E+08	0.07	-4.22	4.00	0.05
E2	GY	4A	AX.89450913	7.27E+08	0.16	-2.86	3.58	0.05
E2	GY	4A	AX.89772769	7.22E+08	0.17	-2.94	3.97	0.07
E2	GY	4A	AX.89772769	7.22E+08	0.17	-2.94	3.97	0.07
E2	GPS	4A	AX.89579142	5.45E+08	0.33	1.43	3.11	0.06
E2	GPS	4A	AX.89677350	5.40E+08	0.31	1.52	3.38	0.06
E2	GPS	4A	AX.89774414	5.45E+08	0.33	1.49	3.34	0.06
E2	GY	4A	AX.89566336	7.27E+08	0.16	-2.80	3.47	0.05
E2	GPS	4A	AX.89324371	5.45E+08	0.33	1.49	3.34	0.06
E2	GPS	4A	AX.89697610	5.45E+08	0.33	1.49	3.34	0.06
E2	GY	4A	AX.89712404	7.20E+08	0.17	-2.84	3.81	0.06
E2	GY	4A	AX.89712404	7.20E+08	0.17	-2.84	3.81	0.06
E2	GY	4A	AX.89623044	7.31E+08	0.20	-2.44	3.18	0.04
E2	GY	4A	AX.89623044	7.31E+08	0.20	-2.44	3.18	0.04

E2	GPS	4A	AX.89557092	5.45E+08	0.33	1.49	3.34	0.06
E2	GPS	4A	AX.89474655	5.44E+08	0.33	1.52	3.47	0.06
E2	GPS	4A	AX.89378527	5.45E+08	0.33	1.49	3.34	0.06
E2	GY	4A	AX.89637945	7.26E+08	0.14	-3.77	5.48	0.08
E2	GY	4A	AX.89637945	7.26E+08	0.14	-3.77	5.48	0.08
E2	GPS	4A	AX.89452039	5.44E+08	0.34	1.47	3.29	0.06
E2	GPS	4A	AX.89611642	5.66E+08	0.45	1.35	3.09	0.06
E2	GPS	4A	AX.89709809	5.46E+08	0.33	1.49	3.34	0.06
E2	GY	4A	AX.89586918	7.28E+08	0.15	-3.08	3.93	0.05
E2	GPS	4A	AX.89519994	5.95E+08	0.42	1.35	3.08	0.04
E2	GY	4A	AX.89591500	7.27E+08	0.18	-2.78	3.67	0.05
E2	GY	4A	AX.89591500	7.27E+08	0.18	-2.78	3.67	0.05
E2	GY	4A	AX.89486973	7.18E+08	0.24	-2.30	3.25	0.05
E2	GY	4A	AX.89593836	7.16E+08	0.08	-3.50	3.05	0.04
E2	GPS	4A	AX.89349938	5.45E+08	0.33	1.49	3.34	0.06
E2	GY	4A	AX.89555897	7.19E+08	0.20	-2.41	3.11	0.05
E2	GY	4A	AX.89555897	7.19E+08	0.20	-2.41	3.11	0.05
E2	GPS	4A	AX.89735458	5.66E+08	0.45	1.35	3.09	0.06
E2	GPS	4A	AX.89344398	5.66E+08	0.45	1.35	3.09	0.06
E2	GPS	4A	AX.89491129	5.67E+08	0.45	1.35	3.09	0.06
E2	GY	4A	AX.89498055	7.28E+08	0.10	-3.46	3.53	0.04
E2	GY	4A	AX.89498055	7.28E+08	0.10	-3.46	3.53	0.04
E2	GPS	4A	AX.89643263	5.66E+08	0.45	1.35	3.09	0.06
E2	GY	4A	AX.89775815	7.24E+08	0.07	-4.83	4.58	0.06
E2	GPS	4A	AX.89326818	5.45E+08	0.33	1.49	3.34	0.06
E2	GPS	4A	AX.89612424	5.45E+08	0.33	1.49	3.34	0.06
E2	GPS	4A	AX.89536982	5.45E+08	0.33	1.43	3.11	0.06
E2	GY	4A	AX.89462896	7.39E+08	0.08	-3.63	3.30	0.04
E2	GPS	4A	AX.89727870	5.62E+08	0.44	1.40	3.29	0.06
E2	GY	4A	AX.89618055	7.25E+08	0.08	-4.54	4.79	0.07
E2	GY	4A	AX.89608513	3.28E+08	0.11	-3.00	3.05	0.04
E2	GY	4A	AX.89524087	6.07E+07	0.20	-2.42	3.22	0.05
E2	GY	4A	AX.89524087	6.07E+07	0.20	-2.42	3.22	0.05
E2	GY	4A	AX.89680753	7.26E+08	0.14	-3.80	5.43	0.08
E2	GY	4A	AX.89680753	7.26E+08	0.14	-3.80	5.43	0.08
E2	GPS	4A	AX.89752156	5.95E+08	0.35	1.44	3.24	0.05
E2	GPS	4A	AX.89598367	5.95E+08	0.35	1.55	3.67	0.06
E2	GY	4A	AX.89347361	7.24E+08	0.06	-4.05	3.18	0.04
E2	GPS	4A	AX.89504124	5.38E+08	0.31	1.52	3.38	0.06
E2	GPS	4A	AX.89628375	6.13E+08	0.23	-1.65	3.29	0.06
E2	GPS	4A	AX.89435121	5.45E+08	0.33	1.49	3.34	0.06
E2	GPS	4A	AX.89557393	5.45E+08	0.33	1.43	3.11	0.06
E2	GPS	4A	AX.89637306	5.46E+08	0.33	1.49	3.34	0.06
E2	GPS	4A	AX.89414093	5.77E+08	0.36	1.39	3.05	0.06
E2	GY	4A	AX.89347832	7.22E+08	0.17	-2.94	3.97	0.07
E2	GY	4A	AX.89347832	7.22E+08	0.17	-2.94	3.97	0.07
E2	GPS	4A	AX.89329697	5.95E+08	0.36	1.46	3.33	0.05
E2	TKW	4A	AX.89338338	7.43E+08	0.21	-1.26	3.85	0.07
E2	GPS	4A	AX.89330631	5.45E+08	0.33	1.49	3.34	0.06
E2	TKW	4A	AX.89417560	6.04E+08	0.44	-0.94	3.28	0.06

E2	GY	4A	AX.89391456	7.20E+08	0.19	-2.71	3.70	0.06
E2	GY	4A	AX.89391456	7.20E+08	0.19	-2.71	3.70	0.06
E2	GPS	4A	AX.89715936	5.45E+08	0.33	1.49	3.34	0.06
E2	GPS	4A	AX.89522378	5.44E+08	0.34	1.57	3.72	0.07
E2	GY	4A	AX.89559764	7.22E+08	0.17	-2.94	3.97	0.07
E2	GY	4A	AX.89559764	7.22E+08	0.17	-2.94	3.97	0.07
E2	GY	4A	AX.89593836	7.16E+08	0.08	-3.50	3.05	0.04
E2	GY	4A	AX.89755912	7.33E+08	0.14	-3.24	4.05	0.05
E2	GY	4A	AX.89755912	7.33E+08	0.14	-3.24	4.05	0.05
E2	GY	4A	AX.89578493	7.20E+08	0.17	-2.84	3.81	0.06
E2	GY	4A	AX.89578493	7.20E+08	0.17	-2.84	3.81	0.06
E2	GY	4A	AX.89767319	7.24E+08	0.06	-4.05	3.18	0.04
E2	GY	4A	AX.89767319	7.24E+08	0.06	-4.05	3.18	0.04
E2	GY	4A	AX.89385754	7.17E+08	0.08	-3.48	3.03	0.04
E2	GY	4A	AX.89385754	7.17E+08	0.08	-3.48	3.03	0.04
E2	GY	4A	AX.89777958	7.24E+08	0.07	-4.43	3.93	0.05
E2	GY	4A	AX.89422048	7.31E+08	0.20	-2.44	3.18	0.04
E2	GPS	4A	AX.89666003	5.91E+08	0.27	1.60	3.44	0.06
E2	GPS	4A	AX.89617814	5.46E+08	0.33	1.49	3.34	0.06
E2	GY	4A	AX.89651575	7.24E+08	0.07	-4.83	4.58	0.06
E2	GY	4A	AX.89651575	7.24E+08	0.07	-4.83	4.58	0.06
E2	GY	4A	AX.89704157	7.25E+08	0.12	-3.59	4.39	0.07
E2	GY	4A	AX.89704157	7.25E+08	0.12	-3.59	4.39	0.07
E2	TKW	4A	AX.89395605	6.04E+08	0.44	-0.94	3.28	0.06
E2	GPS	4A	AX.89509866	5.45E+08	0.33	1.49	3.34	0.06
E2	GY	4A	AX.89729045	7.33E+08	0.07	-4.11	3.63	0.04
E2	TKW	4A	AX.89375502	7.40E+08	0.17	-1.19	3.06	0.05
E2	GY	4A	AX.89712068	7.24E+08	0.07	-4.83	4.58	0.06
E2	GY	4A	AX.89709003	7.43E+08	0.09	-3.29	3.01	0.04
E2	GY	4A	AX.89670415	7.22E+08	0.17	-2.94	3.97	0.07
E2	GPS	4A	AX.89511230	5.45E+08	0.33	1.43	3.11	0.06
E2	GY	4A	AX.89722710	7.26E+08	0.13	-3.18	3.67	0.05
E2	GPS	4A	AX.89687879	5.95E+08	0.42	1.35	3.08	0.04
E2	GY	4A	AX.89636320	7.20E+08	0.18	-2.46	3.09	0.05
E2	GY	4A	AX.89636320	7.20E+08	0.18	-2.46	3.09	0.05
E2	TKW	4A	AX.89661925	6.04E+08	0.39	-0.96	3.28	0.06
E2	TKW	4A	AX.89332213	7.41E+08	0.35	-0.99	3.40	0.06
E2	GY	4A	AX.89576142	7.21E+08	0.15	-2.66	3.14	0.05
E2	GY	4A	AX.89664678	7.28E+08	0.15	-2.78	3.29	0.04
E2	GY	4A	AX.89664678	7.28E+08	0.15	-2.78	3.29	0.04
E2	TKW	4A	AX.89497176	7.40E+08	0.34	-0.96	3.13	0.05
E2	GY	4A	AX.89450913	7.27E+08	0.16	-2.86	3.58	0.05
E2	TKW	4A	AX.89323121	7.34E+08	0.08	-1.69	3.04	0.05
E2	GPS	4A	AX.89578201	5.45E+08	0.33	1.43	3.11	0.06
E2	GY	4A	AX.89566336	7.27E+08	0.16	-2.80	3.47	0.05
E2	GY	4A	AX.89436017	7.25E+08	0.06	-4.62	3.75	0.05
E2	GPS	4A	AX.89699003	5.95E+08	0.35	1.55	3.67	0.06
E2	TKW	4A	AX.89690364	7.40E+08	0.36	-1.05	3.76	0.06
E2	GY	4A	AX.89748469	7.21E+08	0.17	-2.66	3.40	0.06
E2	GPS	4A	AX.89547232	5.46E+08	0.33	1.66	4.02	0.07

E2	GY	4A	AX.89712068	7.24E+08	0.07	-4.83	4.58	0.06
E2	GY	4A	AX.89586918	7.28E+08	0.15	-3.08	3.93	0.05
E2	GPM2	4A	AX.89529538	6.04E+08	0.34	604.67	3.95	0.08
E2	GY	4A	AX.89501499	7.26E+08	0.15	-2.82	3.38	0.04
E2	GPM2	4A	AX.89600862	6.04E+08	0.32	569.05	3.47	0.07
E2	GY	4A	AX.89432804	7.25E+08	0.13	-3.43	4.18	0.06
E2	GY	4A	AX.89432804	7.25E+08	0.13	-3.43	4.18	0.06
E2	GPS	4A	AX.89761832	5.45E+08	0.33	1.49	3.34	0.06
E2	GY	4A	AX.89628286	7.20E+08	0.18	-2.74	3.65	0.06
E2	GY	4A	AX.89628286	7.20E+08	0.18	-2.74	3.65	0.06
E2	GPS	4A	AX.89517100	5.95E+08	0.36	1.48	3.44	0.06
E2	GY	4A	AX.89534997	7.26E+08	0.18	-2.54	3.12	0.04
E2	GY	4A	AX.89534997	7.26E+08	0.18	-2.54	3.12	0.04
E2	GPS	4A	AX.89687555	5.45E+08	0.33	1.49	3.34	0.06
E2	GPS	4A	AX.89522772	5.45E+08	0.33	1.49	3.34	0.06
E2	GPS	4A	AX.89479914	5.95E+08	0.42	1.35	3.08	0.04
E2	GPS	4A	AX.89590295	5.90E+08	0.44	-1.38	3.22	0.05
E2	GY	4A	AX.89501499	7.26E+08	0.15	-2.82	3.38	0.04
E2	GPM2	4A	AX.89327806	6.04E+08	0.34	604.67	3.95	0.08
E2	TKW	4A	AX.89579752	7.43E+08	0.19	-1.24	3.58	0.06
E2	GY	4A	AX.89680789	7.25E+08	0.18	-2.54	3.12	0.04
E2	GY	4A	AX.89680789	7.25E+08	0.18	-2.54	3.12	0.04
E2	TKW	4A	AX.89735480	7.40E+08	0.36	-1.05	3.76	0.06
E2	GPS	4A	AX.89407686	5.44E+08	0.34	1.47	3.29	0.06
E2	GPS	4A	AX.89494450	5.46E+08	0.33	1.43	3.11	0.06
E2	GPS	4A	AX.89720099	5.95E+08	0.42	1.35	3.08	0.04
E2	TKW	4A	AX.89511322	3.03E+07	0.07	-1.81	3.09	0.05
E2	GPS	4A	AX.89653465	5.95E+08	0.35	1.55	3.67	0.06
E2	TKW	4A	AX.89738968	7.34E+08	0.18	-1.15	3.02	0.05
E2	GPS	4A	AX.89754240	5.95E+08	0.35	1.55	3.67	0.06
E2	GPS	4A	AX.89407470	5.67E+08	0.48	1.39	3.29	0.06
E2	TKW	4A	AX.89624256	7.44E+08	0.20	-1.23	3.60	0.06
E2	GY	4A	AX.89668236	7.33E+08	0.15	-3.57	5.04	0.06
E2	GY	4A	AX.89668236	7.33E+08	0.15	-3.57	5.04	0.06
E2	GY	4A	AX.89615361	7.24E+08	0.06	-4.62	3.75	0.05
E2	GPS	4A	AX.89614897	5.46E+08	0.33	1.49	3.34	0.06
E2	GPS	4A	AX.89468874	5.95E+08	0.42	1.35	3.08	0.04
E2	GPS	4A	AX.89463488	5.45E+08	0.33	1.49	3.34	0.06
E2	GY	4A	AX.89377454	7.19E+08	0.13	-2.98	3.47	0.06
E2	GPS	4A	AX.89576809	5.45E+08	0.33	1.43	3.11	0.06
E2	TKW	4A	AX.89355306	7.41E+08	0.34	-1.02	3.49	0.06
E2	GPS	4A	AX.89429223	5.66E+08	0.45	1.35	3.09	0.06
E2	GY	4A	AX.89703146	7.22E+08	0.16	-2.64	3.18	0.05
E2	GPS	4A	AX.89560947	5.44E+08	0.34	1.47	3.29	0.06
E2	GY	4A	AX.89436017	7.25E+08	0.06	-4.62	3.75	0.05
E2	GY	4A	AX.89723261	7.33E+08	0.17	-3.21	4.57	0.06
E2	GY	4A	AX.89704631	7.26E+08	0.13	-3.82	5.21	0.08
E2	TKW	4A	AX.89539186	7.43E+08	0.19	-1.34	4.02	0.07
E2	GPS	4A	AX.89452952	5.45E+08	0.33	1.49	3.34	0.06
E2	GY	4A	AX.89494292	7.21E+08	0.17	-2.61	3.23	0.05

E2	GPS	4A	AX.89658575	5.45E+08	0.33	1.49	3.34	0.06
E2	GPS	4A	AX.89382686	5.45E+08	0.32	1.51	3.39	0.06
E2	GY	4A	AX.89615361	7.24E+08	0.06	-4.62	3.75	0.05
E2	GPS	4A	AX.89436427	5.95E+08	0.35	1.55	3.67	0.06
E2	GPS	4A	AX.89582191	5.95E+08	0.35	1.55	3.67	0.06
E2	TKW	4A	AX.89446643	7.40E+08	0.36	-1.05	3.76	0.06
E2	GPS	4A	AX.89663898	5.65E+08	0.32	1.43	3.06	0.05
E2	GY	4A	AX.89686634	7.12E+07	0.11	-3.11	3.15	0.04
E2	GPS	4A	AX.89312729	5.46E+08	0.33	1.49	3.34	0.06
E2	GPM2	4A	AX.89333422	6.04E+08	0.34	604.67	3.95	0.08
E2	GPS	4A	AX.89499788	5.44E+08	0.34	1.47	3.29	0.06
E2	GPS	4A	AX.89430644	5.63E+08	0.44	1.40	3.29	0.06
E2	GPS	4A	AX.89620966	5.66E+08	0.45	1.35	3.09	0.06
E2	GY	4A	AX.89546685	7.39E+08	0.18	-3.18	4.76	0.06
E2	GY	4A	AX.89546685	7.39E+08	0.18	-3.18	4.76	0.06
E2	GPS	4A	AX.89538080	5.45E+08	0.33	1.49	3.34	0.06
E2	TKW	4A	AX.89599133	7.40E+08	0.34	-1.08	3.87	0.07
E2	TKW	4A	AX.89363602	6.04E+08	0.44	-0.94	3.28	0.06
E2	TKW	4A	AX.89747478	7.41E+08	0.36	-1.05	3.76	0.06
E2	GPS	4A	AX.89574815	5.44E+08	0.34	1.47	3.29	0.06
E2	GPS	4A	AX.89603692	5.95E+08	0.42	1.35	3.08	0.04
E2	GPS	4A	AX.89624314	5.38E+08	0.31	1.52	3.38	0.06
E2	TKW	4A	AX.89324248	7.43E+08	0.15	-1.26	3.12	0.05
E2	GPS	4A	AX.89484427	5.45E+08	0.34	1.47	3.29	0.06
E2	GPS	4A	AX.89516218	5.95E+08	0.36	1.49	3.47	0.06
E2	GPS	4A	AX.89772491	5.95E+08	0.35	1.55	3.67	0.06
E2	TKW	4A	AX.89586557	7.43E+08	0.20	-1.19	3.45	0.06
E2	TKW	4A	AX.89431866	7.34E+08	0.22	-1.09	3.05	0.05
E2	TKW	4A	AX.89726273	7.40E+08	0.35	-0.99	3.40	0.06
E2	GY	4A	AX.89759750	7.22E+08	0.16	-3.03	4.11	0.07
E2	GPS	4A	AX.89760301	6.07E+08	0.40	-1.37	3.12	0.05
E2	GPS	4A	AX.89723951	5.67E+08	0.45	1.35	3.09	0.06
E2	GPS	4A	AX.89777494	6.10E+08	0.31	1.46	3.15	0.06
E2	GY	4A	AX.89373580	2.24E+08	0.11	-3.00	3.05	0.04
E2	GPS	4A	AX.89550641	5.45E+08	0.33	1.49	3.34	0.06
E2	GPS	4A	AX.89637984	5.46E+08	0.33	1.49	3.34	0.06
E2	GPS	4A	AX.89457395	5.45E+08	0.33	1.49	3.34	0.06
E2	GPS	4A	AX.89768942	5.45E+08	0.33	1.49	3.34	0.06
E2	TKW	4A	AX.89639898	7.44E+08	0.21	-1.27	3.89	0.07
E2	GPS	4A	AX.89429683	5.44E+08	0.34	1.47	3.29	0.06
E2	TKW	4A	AX.89349918	7.40E+08	0.17	-1.22	3.23	0.05
E2	TKW	4A	AX.89423722	6.04E+08	0.38	-0.95	3.23	0.06
E2	GPS	4A	AX.89749915	5.70E+08	0.35	1.53	3.57	0.07
E2	GPS	4A	AX.89539135	5.66E+08	0.45	1.35	3.09	0.06
E2	GPS	4A	AX.89641774	5.66E+08	0.45	1.35	3.09	0.06
E2	GPS	4A	AX.89711000	5.46E+08	0.33	1.66	4.02	0.07
E2	GPS	4A	AX.89491834	6.12E+08	0.47	1.35	3.14	0.05
E2	GY	4A	AX.89723261	7.33E+08	0.17	-3.21	4.57	0.06
E2	GY	4A	AX.89665964	7.41E+08	0.23	-2.79	4.41	0.05
E2	TKW	4A	AX.89372934	7.17E+08	0.20	-1.12	3.11	0.05

E2	TKW	4A	AX.89642948	7.41E+08	0.35	-1.03	3.60	0.06
E2	GY	4A	AX.89494292	7.21E+08	0.17	-2.61	3.23	0.05
E2	GPS	4A	AX.89415141	5.45E+08	0.33	1.49	3.34	0.06
E2	TKW	4A	AX.89338248	7.40E+08	0.31	-0.99	3.17	0.05
E2	GPS	4A	AX.89695940	5.45E+08	0.33	1.49	3.34	0.06
E2	GPS	4A	AX.89529538	6.04E+08	0.34	1.47	3.31	0.05
E2	TKW	4A	AX.89621604	7.31E+08	0.26	-1.08	3.42	0.06
E2	GPS	4A	AX.89463074	5.45E+08	0.33	1.49	3.34	0.06
E2	GPS	4A	AX.89317294	5.52E+08	0.33	1.59	3.73	0.07
E2	GPS	4A	AX.89489375	1.68E+07	0.43	1.33	3.03	0.06
E2	GY	4A	AX.89686634	7.12E+07	0.11	-3.11	3.15	0.04
E2	GPS	4A	AX.89606650	5.44E+08	0.33	1.41	3.06	0.06
E2	TKW	4A	AX.89415948	7.27E+08	0.25	-1.05	3.21	0.05
E2	GPS	4A	AX.89617217	5.65E+08	0.44	1.40	3.29	0.06
E2	GPS	4A	AX.89320878	5.66E+08	0.45	1.35	3.09	0.06
E2	GPS	4A	AX.89686935	5.67E+08	0.45	1.35	3.09	0.06
E2	GPS	4A	AX.89654837	5.46E+08	0.33	1.49	3.34	0.06
E2	TKW	4A	AX.89465835	7.41E+08	0.35	-1.01	3.52	0.06
E2	GPS	4A	AX.89566991	5.49E+08	0.30	1.47	3.10	0.06
E2	GPS	4A	AX.89342508	5.67E+08	0.45	1.35	3.09	0.06
E2	TKW	4A	AX.89684381	7.40E+08	0.36	-1.05	3.76	0.06
E2	GPS	4A	AX.89597478	5.45E+08	0.33	1.49	3.34	0.06
E2	TKW	4A	AX.89387177	7.33E+08	0.20	-1.11	3.06	0.05
E2	GPS	4A	AX.89485438	5.45E+08	0.33	1.49	3.34	0.06
E2	GPS	4A	AX.89703903	5.45E+08	0.33	1.49	3.34	0.06
E2	TKW	4A	AX.89706134	7.42E+08	0.36	-1.05	3.76	0.06
E2	GPS	4A	AX.89557552	6.04E+08	0.36	-1.57	3.78	0.06
E2	GPS	4A	AX.89694514	5.45E+08	0.33	1.49	3.34	0.06
E2	GPS	4A	AX.89614652	5.95E+08	0.35	1.55	3.67	0.06
E2	GPS	4A	AX.89380992	6.12E+08	0.24	-1.67	3.43	0.06
E2	GPS	4A	AX.89540224	5.95E+08	0.36	1.49	3.47	0.06
E2	TKW	4A	AX.89486501	7.41E+08	0.35	-0.99	3.40	0.06
E2	TKW	4A	AX.89310426	7.41E+08	0.35	-1.04	3.68	0.06
E2	GPS	4A	AX.89547782	5.46E+08	0.33	1.66	4.02	0.07
E2	GPS	4A	AX.89333422	6.04E+08	0.34	1.47	3.31	0.05
E2	TKW	4A	AX.89373507	7.41E+08	0.30	-1.00	3.21	0.05
E2	GY	4A	AX.89759750	7.22E+08	0.16	-3.03	4.11	0.07
E2	GPS	4A	AX.89733116	5.45E+08	0.33	1.49	3.34	0.06
E2	GPS	4A	AX.89541821	5.45E+08	0.33	1.49	3.34	0.06
E2	GY	4A	AX.89665964	7.41E+08	0.23	-2.79	4.41	0.05
E2	GPS	4A	AX.89511520	5.71E+08	0.31	1.45	3.12	0.06
E2	GPS	4A	AX.89746916	5.66E+08	0.45	1.35	3.09	0.06
E2	GPS	4A	AX.89499197	5.46E+08	0.33	1.43	3.11	0.06
E2	GPS	4A	AX.89734417	5.45E+08	0.33	1.49	3.34	0.06
E3	GPM2	4A	X.89325402_OT	6.69E+08	0.44	-913.03	3.43	0.07
E3	GPM2	4A	X.89674491_OT	6.69E+08	0.46	-958.08	3.72	0.07
E3	GPM2	4A	AX.89459824	6.69E+08	0.47	-1097.28	4.70	0.09
E3	GPM2	4A	X.89704963_OT	6.69E+08	0.46	-933.00	3.57	0.08
E3	GPM2	4A	AX.89473133	7.01E+08	0.33	-1120.06	4.14	0.04
E3	GPM2	4A	AX.89345173	7.02E+08	0.28	-1003.86	3.08	0.07

E3	GPM2	4A	AX.89575491	7.05E+08	0.42	-973.04	3.67	0.08
E3	GPM2	4A	AX.89509899	7.05E+08	0.42	-973.04	3.67	0.08
E3	GPM2	4A	AX.89629538	7.05E+08	0.40	-898.86	3.17	0.07
E3	GPM2	4A	X.89471294_OT	7.09E+08	0.37	-890.86	3.22	0.07
E3	GPM2	4A	X.89420240_OT	7.09E+08	0.38	-973.18	3.73	0.08
E3	GPM2	4A	X.89364662_OT	7.09E+08	0.38	-899.63	3.21	0.08
E3	GPM2	4A	X.89751487_OT	7.12E+08	0.17	-1103.89	3.11	0.03
E3	GPM2	4A	AX.89699790	7.13E+08	0.47	-1106.26	4.63	0.10
E3	GPM2	4A	X.89703546_OT	7.14E+08	0.33	-926.03	3.00	0.08
E3	GPM2	4A	AX.89576549	7.34E+08	0.35	-1082.11	3.71	0.11
E3	GPS	4A	AX.89417750	5.98E+08	0.08	3.99	3.75	0.10
E3	GPS	4A	AX.89669181	7.13E+08	0.41	2.50	3.79	0.11
E3	GPS	4A	AX.89423003	7.13E+08	0.42	2.41	3.56	0.10
E3	GPS	4A	AX.89582784	7.13E+08	0.42	2.38	3.51	0.10
E3	GPS	4A	AX.89639950	7.13E+08	0.41	2.50	3.79	0.11
E3	GPS	4A	AX.89699790	7.13E+08	0.48	-2.11	3.17	0.07
E3	GPS	4A	AX.89678654	7.33E+08	0.39	-2.19	3.05	0.09
E3	GPS	4A	AX.89576549	7.34E+08	0.36	-2.23	3.09	0.09
E3	GPS	4A	AX.89449191	7.34E+08	0.34	-2.30	3.04	0.09
E3	GPS	4A	X.89368493_OT	7.39E+08	0.33	-2.47	3.30	0.08
E3	GPS	4A	AX.89423354	7.39E+08	0.33	-2.47	3.30	0.08
E3	GPS	4A	X.89443062_OT	7.39E+08	0.31	-2.70	3.94	0.09
E3	GPS	4A	X.89691446_OT	7.39E+08	0.32	-2.58	3.47	0.08
E3	GY	4A	AX.89482533	3.87E+07	0.26	-3.51	3.52	0.05
E3	GY	4A	AX.89649145	5.97E+08	0.09	5.42	3.56	0.03
E3	GY	4A	AX.89619129	6.15E+08	0.22	-3.77	3.16	0.03
E3	GY	4A	AX.89724746	6.41E+08	0.30	-3.19	3.14	0.12
E3	GY	4A	AX.89340378	6.41E+08	0.30	-3.19	3.14	0.12
E3	GY	4A	AX.89528746	6.41E+08	0.30	-3.19	3.14	0.12
E3	GY	4A	AX.89433709	6.41E+08	0.29	-3.41	3.45	0.12
E3	GY	4A	AX.89459824	6.69E+08	0.47	-2.92	3.18	0.04
E3	GY	4A	AX.89608694	7.12E+08	0.31	3.51	3.02	0.09
E3	GY	4A	X.89741178_OT	7.12E+08	0.16	-3.98	3.26	0.04
E3	GY	4A	AX.89428823	7.12E+08	0.32	3.46	3.04	0.09
E3	GY	4A	X.89751487_OT	7.12E+08	0.17	-3.73	3.18	0.05
E3	GY	4A	AX.89658868	7.25E+08	0.36	3.42	3.04	0.08
E3	SPM2	4A	AX.89514281	5.71E+08	0.10	-32.27	3.22	0.05
E3	TKW	4A	AX.89743733	2.98E+06	0.43	0.85	3.05	0.10
E3	TKW	4A	AX.89558480	1.22E+07	0.35	0.92	3.38	0.01
E3	TKW	4A	AX.89541289	1.22E+07	0.35	0.88	3.10	0.01
E3	TKW	4A	AX.89489876	1.22E+07	0.35	0.92	3.38	0.01
E3	TKW	4A	AX.89682277	1.22E+07	0.35	0.92	3.38	0.01
E3	TKW	4A	AX.89395097	1.22E+07	0.35	0.88	3.10	0.01
E3	TKW	4A	AX.89384438	1.22E+07	0.48	-0.86	3.35	0.00
E3	TKW	4A	AX.89772643	1.24E+07	0.48	-0.85	3.27	0.00
E3	TKW	4A	AX.89485967	2.36E+08	0.33	1.04	3.10	0.11
E3	TKW	4A	AX.89600822	3.51E+08	0.33	1.04	3.10	0.11
E3	TKW	4A	AX.89516967	3.79E+08	0.34	1.06	3.24	0.11
E3	TKW	4A	AX.89463640	4.08E+08	0.34	1.06	3.24	0.11
E3	TKW	4A	X.89383862_OT	7.22E+08	0.25	1.09	3.07	0.16

E4	GPS	4A	AX.89493898	1.14E+07	0.24	1.91	3.22	0.09
E4	GPS	4A	AX.89678741	5.94E+08	0.24	1.76	3.03	0.06
E4	GPS	4A	AX.89366134	5.94E+08	0.24	1.78	3.10	0.06
E4	GPS	4A	AX.89465023	6.62E+08	0.36	1.70	3.35	0.08
E4	GPS	4A	AX.89426251	6.63E+08	0.36	1.70	3.35	0.08
E4	GPS	4A	AX.89586524	6.63E+08	0.39	1.89	4.13	0.09
E4	GPM2	4A	AX.89720401	6.41E+08	0.25	-500.04	3.03	0.06
E4	GPM2	4A	AX.89356961	6.41E+08	0.25	-522.41	3.39	0.07
E4	GPM2	4A	AX.89376969	6.41E+08	0.25	-522.41	3.39	0.07
E4	GPM2	4A	AX.89543865	6.41E+08	0.26	-521.89	3.41	0.07
E4	GPM2	4A	AX.89398574	6.41E+08	0.30	-493.02	3.19	0.09
E4	GPM2	4A	AX.89330535	6.68E+08	0.24	-523.60	3.23	0.06
E4	GPM2	4A	AX.89459824	6.69E+08	0.47	-531.19	4.22	0.08
E4	GPM2	4A	AX.89749515	6.81E+08	0.05	-1077.23	3.73	0.06
E4	GPM2	4A	AX.89531132	6.85E+08	0.28	-542.91	3.20	0.07
E4	GPM2	4A	AX.89699790	7.13E+08	0.47	-524.02	4.03	0.08
E4	GPS	4A	AX.89443183	6.68E+08	0.25	-1.78	3.33	0.08
E4	GPS	4A	AX.89330535	6.68E+08	0.24	-1.87	3.57	0.08
E4	GPS	4A	X.89674491_OT	6.69E+08	0.46	-1.49	3.09	0.03
E4	GPS	4A	AX.89531132	6.85E+08	0.28	-2.02	3.85	0.11
E4	GPS	4A	AX.89733479	6.86E+08	0.28	-1.86	3.33	0.10
E4	GY	4A	AX.89651410	6.33E+08	0.28	-1.65	3.20	0.03
E4	GY	4A	AX.89436511	6.33E+08	0.27	-1.77	3.60	0.04
E4	GY	4A	AX.89693822	6.33E+08	0.28	-1.80	3.71	0.04
E4	GY	4A	AX.89493396	6.33E+08	0.28	-1.67	3.29	0.04
E4	GY	4A	AX.89562504	6.33E+08	0.28	-1.80	3.71	0.04
E4	GY	4A	AX.89481375	6.33E+08	0.28	-1.66	3.23	0.04
E4	GY	4A	AX.89495210	6.33E+08	0.29	-1.63	3.14	0.04
E4	GY	4A	AX.89689438	6.33E+08	0.27	-1.79	3.65	0.04
E4	GY	4A	AX.89730528	6.33E+08	0.27	-1.78	3.65	0.04
E4	GY	4A	AX.89428257	6.35E+08	0.27	-1.70	3.31	0.04
E4	GY	4A	AX.89330535	6.68E+08	0.25	-1.80	3.35	0.07
E4	GY	4A	AX.89531132	6.85E+08	0.29	-1.88	3.43	0.08
E4	GY	4A	AX.89733479	6.86E+08	0.28	-1.77	3.06	0.08
E4	GY	4A	X.89751487_OT	7.12E+08	0.17	-2.04	3.40	0.05
E4	SPM2	4A	AX.89347196	5.93E+07	0.22	21.60	3.47	0.09
E4	SPM2	4A	AX.89593037	5.93E+07	0.22	21.26	3.40	0.09
E4	SPM2	4A	AX.89377280	5.93E+07	0.21	23.47	3.95	0.09
E4	SPM2	4A	AX.89641336	5.93E+07	0.22	21.60	3.47	0.09
E4	SPM2	4A	AX.89340695	5.93E+07	0.21	22.97	3.83	0.09
E4	SPM2	4A	AX.89499680	5.93E+07	0.22	21.26	3.40	0.09
E4	SPM2	4A	AX.89622341	5.93E+07	0.21	22.97	3.83	0.09
E4	SPM2	4A	AX.89679002	5.93E+07	0.21	22.97	3.83	0.09
E4	SPM2	4A	AX.89702938	5.93E+07	0.21	22.97	3.83	0.09
E4	SPM2	4A	AX.89767322	5.93E+07	0.22	21.26	3.40	0.09
E4	SPM2	4A	AX.89350995	5.93E+07	0.21	23.58	3.95	0.09
E4	SPM2	4A	AX.89428279	5.93E+07	0.21	22.97	3.83	0.09
E4	SPM2	4A	AX.89409621	5.93E+07	0.21	22.97	3.83	0.09
E4	SPM2	4A	AX.89550625	5.93E+07	0.22	21.26	3.40	0.09
E4	SPM2	4A	AX.89497321	5.93E+07	0.22	21.26	3.40	0.09

E4	SPM2	4A	AX.89610582	5.94E+07	0.16	29.17	4.85	0.11
E4	SPM2	4A	AX.89675862	5.95E+07	0.16	29.17	4.85	0.11
E4	SPM2	4A	AX.89374032	5.97E+07	0.17	27.70	4.50	0.11
E4	SPM2	4A	AX.89423661	5.97E+07	0.17	27.70	4.50	0.11
E4	SPM2	4A	AX.89529011	6.00E+07	0.17	27.70	4.50	0.11
E4	SPM2	4A	AX.89661494	6.00E+07	0.17	27.70	4.50	0.11
E4	SPM2	4A	AX.89346727	6.00E+07	0.17	27.70	4.50	0.11
E4	SPM2	4A	AX.89734528	6.00E+07	0.17	27.70	4.50	0.11
E4	SPM2	4A	AX.89634693	6.00E+07	0.17	27.70	4.50	0.11
E4	SPM2	4A	AX.89726762	6.00E+07	0.17	27.70	4.50	0.11
E4	GY	4A	AX.89737326	6.32E+08	0.23	-1.74	3.25	0.04
E4	GY	4A	AX.89658540	6.32E+08	0.19	-1.82	3.11	0.03
E4	GY	4A	AX.89377442	6.32E+08	0.19	-1.88	3.29	0.03
E4	GY	4A	AX.89538574	6.33E+08	0.27	-1.77	3.60	0.04
E4	SPM2	4A	AX.89728973	6.00E+07	0.17	26.08	4.08	0.11
E4	SPM2	4A	AX.89477053	6.00E+07	0.17	27.70	4.50	0.11
E4	SPM2	4A	AX.89380263	6.00E+07	0.17	27.70	4.50	0.11
E4	SPM2	4A	AX.89549134	6.01E+07	0.17	27.70	4.50	0.11
E4	SPM2	4A	AX.89587224	6.01E+07	0.17	27.70	4.50	0.11
E4	SPM2	4A	AX.89389399	6.01E+07	0.16	29.42	4.76	0.11
E4	SPM2	4A	AX.89410827	6.01E+07	0.17	27.70	4.50	0.11
E4	SPM2	4A	AX.89428290	6.01E+07	0.17	27.70	4.50	0.11
E4	SPM2	4A	AX.89666804	6.03E+07	0.17	28.30	4.64	0.12
E4	SPM2	4A	AX.89403111	6.04E+07	0.16	27.52	4.38	0.10
E4	SPM2	4A	AX.89711066	6.06E+07	0.16	29.34	4.92	0.11
E4	SPM2	4A	AX.89640473	6.07E+07	0.16	26.22	4.01	0.10
E4	SPM2	4A	AX.89702651	6.07E+07	0.16	26.22	4.01	0.10
E4	SPM2	4A	AX.89338941	6.07E+07	0.16	26.22	4.01	0.10
E4	SPM2	4A	AX.89629648	6.07E+07	0.17	24.59	3.62	0.10
E4	SPM2	4A	AX.89665341	6.07E+07	0.16	26.22	4.01	0.10
E4	SPM2	4A	AX.89594759	6.07E+07	0.16	26.22	4.01	0.10
E4	SPM2	4A	AX.89502015	6.07E+07	0.17	24.59	3.62	0.10
E4	SPM2	4A	AX.89541830	6.07E+07	0.16	26.22	4.01	0.10
E4	SPM2	4A	AX.89369254	6.07E+07	0.16	26.22	4.01	0.10
E4	SPM2	4A	AX.89738592	6.07E+07	0.16	26.22	4.01	0.10
E4	SPM2	4A	AX.89390212	6.07E+07	0.16	26.22	4.01	0.10
E4	SPM2	4A	AX.89380201	6.07E+07	0.16	27.73	4.34	0.10
E4	SPM2	4A	AX.89671748	6.07E+07	0.16	25.23	3.69	0.10
E4	SPM2	4A	AX.89470893	6.07E+07	0.17	24.59	3.62	0.10
E4	SPM2	4A	AX.89656158	6.07E+07	0.17	24.59	3.62	0.10
E4	SPM2	4A	AX.89347048	6.07E+07	0.17	24.59	3.62	0.10
E4	SPM2	4A	AX.89533264	6.07E+07	0.16	25.30	3.74	0.10
E4	SPM2	4A	AX.89323460	6.07E+07	0.16	26.07	3.93	0.10
E4	SPM2	4A	AX.89729754	6.07E+07	0.17	24.59	3.62	0.10
E4	SPM2	4A	AX.89525947	6.07E+07	0.19	22.39	3.36	0.09
E4	SPM2	4A	AX.89642875	6.09E+07	0.19	22.39	3.36	0.09
E4	SPM2	4A	AX.89336846	6.09E+07	0.18	22.45	3.34	0.09
E4	SPM2	4A	AX.89617643	6.09E+07	0.19	22.39	3.36	0.09
E4	SPM2	4A	AX.89632908	6.09E+07	0.19	22.39	3.36	0.09
E4	SPM2	4A	AX.89725238	6.09E+07	0.19	22.39	3.36	0.09

E4	SPM2	4A	AX.89535950	6.09E+07	0.19	22.39	3.36	0.09
E4	SPM2	4A	AX.89638765	1.53E+08	0.06	32.88	3.14	0.05
E4	SPM2	4A	AX.89669974	5.70E+08	0.43	19.53	3.94	0.09
E4	SPM2	4A	AX.89328975	5.96E+08	0.06	33.02	3.44	0.06
E4	TKW	4A	AX.89629988	7.01E+08	0.17	1.17	3.37	0.06
E4	TKW	4A	AX.89473133	7.01E+08	0.33	0.90	3.00	0.03
E1	GY	4B	AX.89426986	6.58E+08	0.09	-4.07	3.15	0.05
E1	GY	4B	AX.89378611	1.60E+08	0.39	-2.38	3.17	0.04
E1	GY	4B	AX.86162512	5.32E+08	0.32	2.53	3.26	0.06
E1	GY	4B	AX.89380326	5.62E+08	0.25	2.71	3.24	0.06
E1	GY	4B	AX.89606985	5.25E+08	0.16	-3.11	3.12	0.07
E1	GY	4B	AX.89449444	5.62E+08	0.27	2.82	3.61	0.06
E1	GY	4B	AX.89336970	5.32E+08	0.32	2.53	3.26	0.06
E1	GY	4B	AX.89586334	6.58E+08	0.08	-4.21	3.19	0.05
E1	SPM2	4B	AX.89582795	6.43E+08	0.32	18.63	3.39	0.05
E1	GY	4B	AX.89599985	5.62E+08	0.28	2.57	3.15	0.06
E1	SPM2	4B	AX.89378745	6.43E+08	0.33	17.15	3.02	0.04
E1	GY	4B	AX.89407311	5.62E+08	0.27	2.94	3.87	0.07
E1	GY	4B	AX.89433824	6.58E+08	0.08	-5.05	4.38	0.07
E1	GY	4B	AX.89426986	6.58E+08	0.09	-4.07	3.15	0.05
E1	GY	4B	AX.89705801	6.58E+08	0.09	-4.37	3.56	0.06
E1	GY	4B	AX.89449444	5.62E+08	0.27	2.82	3.61	0.06
E1	GY	4B	AX.89606985	5.25E+08	0.16	-3.11	3.12	0.07
E1	GY	4B	AX.89599985	5.62E+08	0.28	2.57	3.15	0.06
E1	GY	4B	AX.89376848	2.15E+08	0.28	2.73	3.52	0.06
E1	GY	4B	AX.89743825	5.32E+08	0.32	2.53	3.26	0.06
E1	SPM2	4B	AX.89753580	6.43E+08	0.33	17.15	3.02	0.04
E1	GY	4B	AX.89344896	1.62E+08	0.38	-2.50	3.42	0.05
E1	GY	4B	AX.89643271	5.33E+08	0.32	2.53	3.26	0.06
E1	GY	4B	AX.89588328	5.62E+08	0.28	2.57	3.15	0.06
E1	SPM2	4B	AX.89689341	6.43E+08	0.32	18.63	3.39	0.05
E1	GY	4B	AX.89349390	5.88E+08	0.25	2.92	3.69	0.07
E1	GY	4B	AX.89476043	6.58E+08	0.09	-4.86	4.45	0.07
E1	GY	4B	AX.89388981	1.69E+08	0.39	-2.31	3.02	0.04
E1	GY	4B	AX.89377927	5.32E+08	0.32	2.53	3.26	0.06
E1	GY	4B	AX.89389357	6.58E+08	0.08	-5.05	4.38	0.07
E1	GY	4B	AX.89354374	5.60E+08	0.26	2.82	3.55	0.06
E1	GY	4B	AX.89565967	5.62E+08	0.27	2.94	3.87	0.07
E1	GY	4B	AX.89317811	5.31E+08	0.33	2.56	3.37	0.06
E1	GY	4B	AX.89607271	1.25E+07	0.06	-4.78	3.08	0.06
E1	GY	4B	AX.89586334	6.58E+08	0.08	-4.21	3.19	0.05
E1	SPM2	4B	AX.89436818	6.43E+08	0.32	18.84	3.48	0.05
E1	GY	4B	AX.89364224	6.58E+08	0.09	-4.37	3.56	0.06
E1	GY	4B	AX.89512983	5.34E+08	0.32	2.53	3.26	0.06
E1	GY	4B	AX.89551942	4.44E+08	0.18	2.97	3.10	0.05
E1	SPM2	4B	AX.89355046	6.43E+08	0.32	18.63	3.39	0.05
E1	GY	4B	AX.89319673	5.32E+08	0.32	2.53	3.26	0.06
E1	GY	4B	AX.89323722	5.60E+08	0.26	2.82	3.55	0.06
E1	GY	4B	AX.89315775	5.93E+08	0.21	-2.92	3.30	0.05
E1	GY	4B	AX.89462202	5.61E+08	0.28	2.57	3.15	0.06

E1	GY	4B	AX.89557593	5.62E+08	0.28	2.57	3.15	0.06
E1	SPM2	4B	AX.89630537	6.43E+08	0.32	18.63	3.39	0.05
E1	SPM2	4B	AX.89548012	6.43E+08	0.32	18.63	3.39	0.05
E1	GY	4B	AX.89719542	6.58E+08	0.08	-5.05	4.38	0.07
E1	GY	4B	AX.89389357	6.58E+08	0.08	-5.05	4.38	0.07
E1	GY	4B	AX.89433824	6.58E+08	0.08	-5.05	4.38	0.07
E1	GY	4B	AX.89354461	5.33E+08	0.32	2.53	3.26	0.06
E1	GY	4B	AX.89678724	6.58E+08	0.09	-4.37	3.56	0.06
E1	SPM2	4B	AX.89331813	6.43E+08	0.32	18.63	3.39	0.05
E1	GY	4B	AX.86162512	5.32E+08	0.32	2.53	3.26	0.06
E1	SPM2	4B	AX.89419542	6.43E+08	0.32	18.84	3.48	0.05
E1	GY	4B	AX.89531900	5.62E+08	0.27	2.94	3.87	0.07
E1	GY	4B	AX.89531900	5.62E+08	0.27	2.94	3.87	0.07
E1	GY	4B	AX.89450047	6.58E+08	0.09	-4.37	3.56	0.06
E1	SPM2	4B	AX.89441361	6.43E+08	0.29	18.63	3.26	0.06
E1	GY	4B	AX.89667185	6.58E+08	0.08	-5.05	4.38	0.07
E1	GY	4B	AX.89344272	4.34E+08	0.18	2.97	3.10	0.05
E1	GY	4B	AX.89397095	5.33E+08	0.32	2.53	3.26	0.06
E1	GY	4B	AX.89352705	5.31E+08	0.33	2.56	3.37	0.06
E1	GY	4B	AX.89611300	1.67E+08	0.39	-2.38	3.17	0.04
E1	GY	4B	AX.89365086	5.93E+08	0.21	-2.92	3.30	0.05
E1	GY	4B	AX.89571745	5.32E+08	0.32	2.53	3.26	0.06
E1	GY	4B	AX.89588328	5.62E+08	0.28	2.57	3.15	0.06
E1	GY	4B	AX.89397095	5.33E+08	0.32	2.53	3.26	0.06
E1	GY	4B	AX.89526867	6.57E+08	0.07	-4.60	3.23	0.05
E1	GY	4B	AX.89388981	1.69E+08	0.39	-2.31	3.02	0.04
E1	GY	4B	AX.89448454	4.28E+08	0.45	2.31	3.11	0.05
E1	GY	4B	AX.89331310	6.57E+08	0.07	-4.60	3.23	0.05
E1	GY	4B	AX.89673916	5.32E+08	0.32	2.53	3.26	0.06
E1	SPM2	4B	AX.89517394	6.43E+08	0.32	18.63	3.39	0.05
E1	SPM2	4B	AX.86164394	6.43E+08	0.32	18.63	3.39	0.05
E1	GY	4B	AX.89492174	1.26E+07	0.05	-6.67	4.41	0.09
E1	GY	4B	AX.89331903	6.58E+08	0.09	-4.37	3.56	0.06
E1	GY	4B	AX.89669977	1.65E+08	0.39	-2.38	3.17	0.04
E1	GY	4B	AX.89695675	5.32E+08	0.33	2.44	3.12	0.06
E1	GY	4B	AX.89508190	5.61E+08	0.26	2.81	3.48	0.06
E1	GY	4B	AX.89553918	1.68E+08	0.39	-2.38	3.17	0.04
E1	GY	4B	AX.89456967	5.62E+08	0.28	2.57	3.15	0.06
E1	GY	4B	AX.89642522	5.32E+08	0.32	2.53	3.26	0.06
E1	GY	4B	AX.89355730	5.32E+08	0.32	2.53	3.26	0.06
E1	GY	4B	AX.89477195	5.32E+08	0.32	2.53	3.26	0.06
E1	GY	4B	AX.89748798	5.62E+08	0.27	2.94	3.87	0.07
E1	GY	4B	AX.89344896	1.62E+08	0.38	-2.50	3.42	0.05
E1	GY	4B	AX.89626539	5.31E+08	0.33	2.56	3.37	0.06
E1	GY	4B	AX.89643271	5.33E+08	0.32	2.53	3.26	0.06
E1	GY	4B	AX.89486798	5.62E+08	0.28	2.57	3.15	0.06
E1	GY	4B	AX.89365086	5.93E+08	0.21	-2.92	3.30	0.05
E1	SPM2	4B	AX.89675859	6.43E+08	0.31	18.99	3.48	0.05
E1	GY	4B	AX.89323722	5.60E+08	0.26	2.82	3.55	0.06
E1	GY	4B	AX.89746602	5.65E+08	0.29	2.75	3.58	0.06

E1	GY	4B	AX.89381343	5.60E+08	0.27	2.94	3.87	0.07
E1	GY	4B	AX.89354461	5.33E+08	0.32	2.53	3.26	0.06
E1	GY	4B	AX.89650100	1.31E+07	0.07	-5.03	3.96	0.08
E1	GY	4B	AX.89476160	1.72E+08	0.39	-2.38	3.17	0.04
E1	GY	4B	AX.89512983	5.34E+08	0.32	2.53	3.26	0.06
E1	GY	4B	AX.89407311	5.62E+08	0.27	2.94	3.87	0.07
E1	GY	4B	AX.89496758	1.65E+08	0.39	-2.38	3.17	0.04
E1	GY	4B	AX.89729866	6.58E+08	0.09	-4.37	3.56	0.06
E1	SPM2	4B	AX.89540061	6.43E+08	0.31	19.20	3.54	0.05
E1	GY	4B	AX.89683137	6.58E+08	0.09	-4.37	3.56	0.06
E1	GY	4B	AX.89594799	4.45E+08	0.18	2.97	3.10	0.05
E1	GY	4B	AX.89372260	5.33E+08	0.32	2.53	3.26	0.06
E1	GY	4B	AX.89497398	5.32E+08	0.32	2.53	3.26	0.06
E1	GY	4B	AX.89336970	5.32E+08	0.32	2.53	3.26	0.06
E1	SPM2	4B	AX.89354015	6.43E+08	0.33	17.68	3.15	0.05
E1	GY	4B	AX.89494121	5.33E+08	0.32	2.53	3.26	0.06
E1	GY	4B	AX.89404249	5.32E+08	0.32	2.53	3.26	0.06
E1	GY	4B	AX.89730387	1.72E+08	0.39	-2.38	3.17	0.04
E1	GY	4B	AX.89746405	5.42E+08	0.23	3.00	3.70	0.07
E1	GY	4B	AX.89361958	5.31E+08	0.33	2.56	3.37	0.06
E1	GY	4B	AX.89589070	5.61E+08	0.28	2.57	3.15	0.06
E1	GY	4B	AX.89327870	5.62E+08	0.28	2.57	3.15	0.06
E1	GY	4B	AX.89719542	6.58E+08	0.08	-5.05	4.38	0.07
E1	GY	4B	AX.89557504	6.58E+08	0.08	-5.05	4.38	0.07
E1	GY	4B	AX.89678724	6.58E+08	0.09	-4.37	3.56	0.06
E1	GY	4B	AX.89516995	2.86E+08	0.35	-2.37	3.04	0.04
E1	GY	4B	AX.89516995	2.86E+08	0.35	-2.37	3.04	0.04
E1	GY	4B	AX.89400160	5.92E+08	0.26	2.86	3.60	0.06
E1	GY	4B	AX.89330007	6.58E+08	0.08	-4.18	3.16	0.05
E1	GY	4B	AX.89571745	5.32E+08	0.32	2.53	3.26	0.06
E1	GY	4B	AX.89384581	1.31E+07	0.07	-5.40	4.25	0.08
E1	GY	4B	AX.89541555	1.31E+07	0.05	-6.46	4.17	0.08
E1	GY	4B	AX.89331310	6.57E+08	0.07	-4.60	3.23	0.05
E1	GY	4B	AX.89660931	1.55E+08	0.38	-2.33	3.02	0.04
E1	GY	4B	AX.89661370	5.62E+08	0.27	3.06	4.17	0.08
E1	GY	4B	AX.89414000	5.62E+08	0.27	2.94	3.87	0.07
E1	GY	4B	AX.89492174	1.26E+07	0.05	-6.67	4.41	0.09
E1	GY	4B	AX.89555995	5.93E+08	0.21	-2.92	3.30	0.05
E1	GY	4B	AX.89331903	6.58E+08	0.09	-4.37	3.56	0.06
E1	GY	4B	AX.89650100	1.31E+07	0.07	-5.03	3.96	0.08
E1	GY	4B	AX.89508190	5.61E+08	0.26	2.81	3.48	0.06
E1	GY	4B	AX.89508272	5.62E+08	0.28	2.57	3.15	0.06
E1	GY	4B	AX.89508272	5.62E+08	0.28	2.57	3.15	0.06
E1	SPM2	4B	AX.89514174	6.43E+08	0.32	18.63	3.39	0.05
E1	GY	4B	AX.89472483	5.62E+08	0.27	2.84	3.70	0.07
E1	GY	4B	AX.89752270	5.31E+08	0.33	2.56	3.37	0.06
E1	GY	4B	AX.89622164	3.44E+08	0.28	2.73	3.52	0.06
E1	GY	4B	AX.89380326	5.62E+08	0.25	2.71	3.24	0.06
E1	GY	4B	AX.89330007	6.58E+08	0.08	-4.18	3.16	0.05
E1	GY	4B	AX.89486798	5.62E+08	0.28	2.57	3.15	0.06

E1	GY	4B	AX.89551942	4.44E+08	0.18	2.97	3.10	0.05
E1	GY	4B	AX.89487605	5.62E+08	0.32	3.37	5.35	0.10
E1	SPM2	4B	AX.89363554	6.43E+08	0.32	18.63	3.39	0.05
E1	GY	4B	AX.89776240	5.31E+08	0.33	2.56	3.37	0.06
E1	GY	4B	AX.89381343	5.60E+08	0.27	2.94	3.87	0.07
E1	GY	4B	AX.89475860	5.32E+08	0.32	2.53	3.26	0.06
E1	GY	4B	AX.89557593	5.62E+08	0.28	2.57	3.15	0.06
E1	GY	4B	AX.89678567	5.32E+08	0.32	2.53	3.26	0.06
E1	GY	4B	AX.89476160	1.72E+08	0.39	-2.38	3.17	0.04
E1	GY	4B	AX.89371502	5.37E+08	0.23	3.00	3.70	0.07
E1	GY	4B	AX.89313869	4.44E+08	0.18	2.97	3.10	0.05
E1	GY	4B	AX.89313869	4.44E+08	0.18	2.97	3.10	0.05
E1	GY	4B	AX.89667145	1.68E+08	0.39	-2.38	3.17	0.04
E1	GY	4B	AX.89667145	1.68E+08	0.39	-2.38	3.17	0.04
E1	GY	4B	AX.89430932	6.58E+08	0.09	-4.90	4.33	0.07
E1	GY	4B	AX.89430932	6.58E+08	0.09	-4.90	4.33	0.07
E1	GY	4B	AX.89642522	5.32E+08	0.32	2.53	3.26	0.06
E1	GY	4B	AX.89594799	4.45E+08	0.18	2.97	3.10	0.05
E1	GY	4B	AX.89347833	5.62E+08	0.28	2.57	3.15	0.06
E1	GY	4B	AX.89626357	5.33E+08	0.32	2.53	3.26	0.06
E1	GY	4B	AX.89626371	1.66E+08	0.39	-2.38	3.17	0.04
E1	GY	4B	AX.89647330	5.61E+08	0.26	2.82	3.55	0.06
E1	GY	4B	AX.89361217	5.33E+08	0.32	2.53	3.26	0.06
E1	GY	4B	AX.89361217	5.33E+08	0.32	2.53	3.26	0.06
E1	GY	4B	AX.89315775	5.93E+08	0.21	-2.92	3.30	0.05
E1	GY	4B	AX.89349390	5.88E+08	0.25	2.92	3.69	0.07
E1	GY	4B	AX.89705801	6.58E+08	0.09	-4.37	3.56	0.06
E1	GY	4B	AX.89533727	5.33E+08	0.32	2.53	3.26	0.06
E1	GY	4B	AX.89316538	5.62E+08	0.27	3.00	3.99	0.07
E1	GY	4B	AX.89316538	5.62E+08	0.27	3.00	3.99	0.07
E1	GY	4B	AX.89361958	5.31E+08	0.33	2.56	3.37	0.06
E1	GY	4B	AX.89467069	5.33E+08	0.32	2.59	3.41	0.06
E1	SPM2	4B	AX.89621268	6.43E+08	0.32	18.63	3.39	0.05
E1	GY	4B	AX.89327870	5.62E+08	0.28	2.57	3.15	0.06
E1	GY	4B	AX.89669348	5.60E+08	0.26	2.82	3.55	0.06
E1	GY	4B	AX.89743825	5.32E+08	0.32	2.53	3.26	0.06
E1	GY	4B	AX.89598047	1.68E+08	0.39	-2.38	3.17	0.04
E1	GY	4B	AX.89647006	5.61E+08	0.23	2.91	3.50	0.06
E1	GY	4B	AX.89754106	5.32E+08	0.32	2.53	3.26	0.06
E1	GY	4B	AX.89319673	5.32E+08	0.32	2.53	3.26	0.06
E1	GY	4B	AX.89564249	5.33E+08	0.32	2.53	3.26	0.06
E1	GY	4B	AX.89698141	1.51E+08	0.38	-2.50	3.42	0.05
E1	GY	4B	AX.89541555	1.31E+07	0.05	-6.46	4.17	0.08
E1	GY	4B	AX.89448454	4.28E+08	0.45	2.31	3.11	0.05
E1	GY	4B	AX.89673916	5.32E+08	0.32	2.53	3.26	0.06
E1	GY	4B	AX.89760506	5.32E+08	0.32	2.53	3.26	0.06
E1	GY	4B	AX.89624445	5.33E+08	0.32	2.53	3.26	0.06
E1	GY	4B	AX.89772648	5.21E+08	0.23	3.08	3.85	0.07
E1	GY	4B	AX.89378611	1.60E+08	0.39	-2.38	3.17	0.04
E1	GY	4B	AX.89529379	5.33E+08	0.32	2.53	3.26	0.06

E1	GY	4B	AX.89529379	5.33E+08	0.32	2.53	3.26	0.06
E1	GY	4B	AX.89496758	1.65E+08	0.39	-2.38	3.17	0.04
E1	GY	4B	AX.89450047	6.58E+08	0.09	-4.37	3.56	0.06
E1	GY	4B	AX.89344272	4.34E+08	0.18	2.97	3.10	0.05
E1	GY	4B	AX.89672975	5.60E+08	0.26	2.82	3.55	0.06
E1	GY	4B	AX.89497126	5.32E+08	0.32	2.53	3.26	0.06
E1	GY	4B	AX.89497126	5.32E+08	0.32	2.53	3.26	0.06
E1	SPM2	4B	AX.89459544	6.43E+08	0.33	18.63	3.48	0.05
E1	GY	4B	AX.89578164	5.33E+08	0.32	2.53	3.26	0.06
E1	GY	4B	AX.89599903	4.44E+08	0.18	2.97	3.10	0.05
E1	GY	4B	AX.89733183	5.93E+08	0.21	-2.92	3.30	0.05
E1	GY	4B	AX.89628533	5.32E+08	0.32	2.53	3.26	0.06
E1	GY	4B	AX.89512685	5.87E+08	0.25	2.92	3.69	0.07
E1	GY	4B	AX.89462202	5.61E+08	0.28	2.57	3.15	0.06
E1	GY	4B	AX.89556208	1.26E+07	0.05	-6.12	4.11	0.08
E1	GY	4B	AX.89753877	1.26E+07	0.07	-5.03	3.96	0.08
E1	GY	4B	AX.89433900	5.33E+08	0.32	2.53	3.26	0.06
E1	SPM2	4B	AX.89310409	6.43E+08	0.32	18.13	3.25	0.05
E1	GY	4B	AX.89535038	5.58E+08	0.26	2.89	3.66	0.07
E1	GY	4B	AX.89607271	1.25E+07	0.06	-4.78	3.08	0.06
E1	GY	4B	AX.89594770	5.95E+07	0.36	2.58	3.56	0.06
E1	GY	4B	AX.89717411	1.28E+07	0.07	-5.69	4.66	0.09
E1	GY	4B	AX.89717411	1.28E+07	0.07	-5.69	4.66	0.09
E1	GY	4B	AX.89419922	5.32E+08	0.32	2.53	3.26	0.06
E1	GY	4B	AX.89419922	5.32E+08	0.32	2.53	3.26	0.06
E1	GY	4B	AX.89718135	5.31E+08	0.33	2.56	3.37	0.06
E1	GY	4B	AX.89384581	1.31E+07	0.07	-5.40	4.25	0.08
E1	GY	4B	AX.89604472	6.58E+08	0.09	-4.26	3.54	0.06
E1	GY	4B	AX.89526867	6.57E+08	0.07	-4.60	3.23	0.05
E1	GY	4B	AX.89730387	1.72E+08	0.39	-2.38	3.17	0.04
E1	GY	4B	AX.89718911	6.58E+08	0.09	-4.37	3.56	0.06
E1	GY	4B	AX.89604960	5.31E+08	0.33	2.56	3.37	0.06
E1	GY	4B	AX.89669348	5.60E+08	0.26	2.82	3.55	0.06
E1	GY	4B	AX.89584687	5.63E+08	0.29	2.75	3.58	0.06
E1	GY	4B	AX.89763483	6.58E+08	0.08	-5.05	4.38	0.07
E1	GY	4B	AX.89669977	1.65E+08	0.39	-2.38	3.17	0.04
E1	GY	4B	AX.89682209	5.33E+08	0.32	2.53	3.26	0.06
E1	GY	4B	AX.89422596	3.75E+08	0.28	2.73	3.52	0.06
E1	GY	4B	AX.89400160	5.92E+08	0.26	2.86	3.60	0.06
E1	GY	4B	AX.89352705	5.31E+08	0.33	2.56	3.37	0.06
E1	GY	4B	AX.89753877	1.26E+07	0.07	-5.03	3.96	0.08
E1	GY	4B	AX.89589070	5.61E+08	0.28	2.57	3.15	0.06
E1	GY	4B	AX.89377927	5.32E+08	0.32	2.53	3.26	0.06
E1	GY	4B	AX.89507152	5.30E+08	0.16	-3.12	3.08	0.07
E1	GY	4B	AX.89354374	5.60E+08	0.26	2.82	3.55	0.06
E1	GY	4B	AX.89422024	5.33E+08	0.32	2.53	3.26	0.06
E1	SPM2	4B	AX.89420848	6.43E+08	0.32	18.63	3.39	0.05
E1	GY	4B	AX.89712333	5.93E+08	0.21	-2.92	3.30	0.05
E1	GY	4B	AX.89712333	5.93E+08	0.21	-2.92	3.30	0.05
E1	GY	4B	AX.89355602	6.58E+08	0.08	-5.05	4.38	0.07

E1	GY	4B	AX.89355730	5.32E+08	0.32	2.53	3.26	0.06
E1	GY	4B	AX.89626371	1.66E+08	0.39	-2.38	3.17	0.04
E1	GY	4B	AX.89497398	5.32E+08	0.32	2.53	3.26	0.06
E1	GY	4B	AX.89404249	5.32E+08	0.32	2.53	3.26	0.06
E1	GY	4B	AX.89462337	5.89E+07	0.37	2.38	3.11	0.06
E1	GY	4B	AX.89642919	5.33E+08	0.32	2.53	3.26	0.06
E1	GY	4B	AX.89616304	5.60E+08	0.26	2.82	3.55	0.06
E1	GY	4B	AX.89771775	6.58E+08	0.09	-4.37	3.56	0.06
E1	GY	4B	AX.89616929	5.71E+08	0.28	2.57	3.14	0.06
E1	GY	4B	AX.89723602	5.31E+08	0.33	2.56	3.37	0.06
E1	GY	4B	AX.89476043	6.58E+08	0.09	-4.86	4.45	0.07
E1	GY	4B	AX.89678567	5.32E+08	0.32	2.53	3.26	0.06
E1	GY	4B	AX.89667185	6.58E+08	0.08	-5.05	4.38	0.07
E1	GY	4B	AX.89347833	5.62E+08	0.28	2.57	3.15	0.06
E1	SPM2	4B	AX.89566393	6.44E+08	0.47	16.20	3.01	0.05
E1	GY	4B	AX.89729025	4.39E+08	0.18	2.97	3.10	0.05
E1	GY	4B	AX.89729025	4.39E+08	0.18	2.97	3.10	0.05
E1	GY	4B	AX.89683540	5.62E+08	0.26	2.75	3.39	0.06
E1	GY	4B	AX.89344798	5.33E+08	0.32	2.53	3.26	0.06
E1	GY	4B	AX.89372260	5.33E+08	0.32	2.53	3.26	0.06
E1	GY	4B	AX.89655632	1.60E+08	0.44	-2.29	3.06	0.04
E1	GY	4B	AX.89701032	6.58E+08	0.07	-4.88	3.76	0.06
E1	GY	4B	AX.89439700	5.31E+08	0.33	2.56	3.37	0.06
E1	GPS	4B	AX.89487605	5.62E+08	0.32	1.33	3.78	0.07
E1	GY	4B	AX.89478754	5.31E+08	0.33	2.56	3.37	0.06
E1	GY	4B	AX.89514930	5.33E+08	0.32	2.53	3.26	0.06
E1	GY	4B	AX.89514930	5.33E+08	0.32	2.53	3.26	0.06
E1	GY	4B	AX.89681180	5.31E+08	0.33	2.56	3.37	0.06
E1	GY	4B	AX.89681180	5.31E+08	0.33	2.56	3.37	0.06
E1	GY	4B	AX.89549282	5.33E+08	0.32	2.53	3.26	0.06
E1	GY	4B	AX.89483878	5.93E+08	0.21	-2.92	3.30	0.05
E1	GY	4B	AX.89557504	6.58E+08	0.08	-5.05	4.38	0.07
E1	GY	4B	AX.89580725	5.33E+08	0.32	2.53	3.26	0.06
E1	GY	4B	AX.89695675	5.32E+08	0.33	2.44	3.12	0.06
E1	GY	4B	AX.89682209	5.33E+08	0.32	2.53	3.26	0.06
E1	GPS	4B	AX.89331813	6.43E+08	0.32	-1.19	3.13	0.05
E1	GY	4B	AX.89637611	5.31E+08	0.33	2.56	3.37	0.06
E1	GPS	4B	AX.89675859	6.43E+08	0.31	-1.22	3.26	0.06
E1	GY	4B	AX.89364224	6.58E+08	0.09	-4.37	3.56	0.06
E1	GY	4B	AX.89769229	5.31E+08	0.33	2.56	3.37	0.06
E1	GY	4B	AX.89611300	1.67E+08	0.39	-2.38	3.17	0.04
E1	GY	4B	AX.89401081	5.31E+08	0.33	2.56	3.37	0.06
E1	GY	4B	AX.89376848	2.15E+08	0.28	2.73	3.52	0.06
E1	GY	4B	AX.89757114	5.31E+08	0.33	2.56	3.37	0.06
E1	GY	4B	AX.89757114	5.31E+08	0.33	2.56	3.37	0.06
E1	GY	4B	AX.89648611	6.57E+08	0.07	-4.60	3.23	0.05
E1	GY	4B	AX.89576163	5.31E+08	0.33	2.56	3.40	0.06
E1	GY	4B	AX.89576163	5.31E+08	0.33	2.56	3.40	0.06
E1	GY	4B	AX.89661370	5.62E+08	0.27	3.06	4.17	0.08
E1	GY	4B	AX.89414000	5.62E+08	0.27	2.94	3.87	0.07

E1	GPS	4B	AX.89310409	6.43E+08	0.32	-1.19	3.15	0.05
E1	GY	4B	AX.89472483	5.62E+08	0.27	2.84	3.70	0.07
E1	GY	4B	AX.89672975	5.60E+08	0.26	2.82	3.55	0.06
E1	GY	4B	AX.89415532	5.32E+08	0.32	2.53	3.26	0.06
E1	GY	4B	AX.89415532	5.32E+08	0.32	2.53	3.26	0.06
E1	GY	4B	AX.89578164	5.33E+08	0.32	2.53	3.26	0.06
E1	GY	4B	AX.89512685	5.87E+08	0.25	2.92	3.69	0.07
E1	GY	4B	AX.89725528	5.88E+08	0.25	2.92	3.69	0.07
E1	GY	4B	AX.89725528	5.88E+08	0.25	2.92	3.69	0.07
E1	GY	4B	AX.89487605	5.62E+08	0.32	3.37	5.35	0.10
E1	GY	4B	AX.89556208	1.26E+07	0.05	-6.12	4.11	0.08
E1	GY	4B	AX.89776240	5.31E+08	0.33	2.56	3.37	0.06
E1	GY	4B	AX.89653815	5.60E+08	0.26	2.74	3.37	0.06
E1	GY	4B	AX.89653815	5.60E+08	0.26	2.74	3.37	0.06
E1	GY	4B	AX.89512210	6.57E+08	0.07	-4.60	3.23	0.05
E1	GPS	4B	AX.89363554	6.43E+08	0.32	-1.19	3.13	0.05
E1	GY	4B	AX.89430244	5.31E+08	0.33	2.56	3.37	0.06
E1	GY	4B	AX.89561827	5.32E+08	0.32	2.53	3.26	0.06
E1	GY	4B	AX.89452772	5.62E+08	0.27	2.99	4.04	0.07
E1	GY	4B	AX.89371502	5.37E+08	0.23	3.00	3.70	0.07
E1	GY	4B	AX.89586202	6.58E+08	0.09	-4.37	3.56	0.06
E1	GY	4B	AX.89657621	5.32E+08	0.32	2.53	3.26	0.06
E1	GPS	4B	AX.89354015	6.43E+08	0.33	-1.17	3.12	0.05
E1	GY	4B	AX.89683137	6.58E+08	0.09	-4.37	3.56	0.06
E1	GY	4B	AX.89594770	5.95E+07	0.36	2.58	3.56	0.06
E1	GPS	4B	AX.89621268	6.43E+08	0.32	-1.19	3.13	0.05
E1	GPS	4B	AX.89514174	6.43E+08	0.32	-1.19	3.13	0.05
E1	GY	4B	AX.89626357	5.33E+08	0.32	2.53	3.26	0.06
E1	GY	4B	AX.89344798	5.33E+08	0.32	2.53	3.26	0.06
E1	GY	4B	AX.89655632	1.60E+08	0.44	-2.29	3.06	0.04
E1	GY	4B	AX.89566116	1.60E+08	0.38	-2.33	3.02	0.04
E1	GY	4B	AX.89762659	5.93E+07	0.36	2.58	3.56	0.06
E1	GY	4B	AX.89604472	6.58E+08	0.09	-4.26	3.54	0.06
E1	GY	4B	AX.89533727	5.33E+08	0.32	2.53	3.26	0.06
E1	GY	4B	AX.89316572	1.33E+07	0.05	-5.41	3.32	0.05
E1	GY	4B	AX.89338729	5.33E+08	0.32	2.53	3.26	0.06
E1	GY	4B	AX.89641200	6.58E+08	0.12	-3.58	3.12	0.06
E1	GY	4B	AX.89445900	6.58E+08	0.09	-4.26	3.54	0.06
E1	GY	4B	AX.89445900	6.58E+08	0.09	-4.26	3.54	0.06
E1	GY	4B	AX.89671108	6.57E+08	0.07	-4.60	3.23	0.05
E1	GY	4B	AX.89671108	6.57E+08	0.07	-4.60	3.23	0.05
E1	GY	4B	AX.89422596	3.75E+08	0.28	2.73	3.52	0.06
E1	GY	4B	AX.89456967	5.62E+08	0.28	2.57	3.15	0.06
E1	GY	4B	AX.89636790	6.58E+08	0.09	-4.37	3.56	0.06
E1	GY	4B	AX.89769229	5.31E+08	0.33	2.56	3.37	0.06
E1	GY	4B	AX.89733183	5.93E+08	0.21	-2.92	3.30	0.05
E1	GY	4B	AX.89529468	5.32E+08	0.32	2.53	3.26	0.06
E1	GY	4B	AX.89647006	5.61E+08	0.23	2.91	3.50	0.06
E1	GY	4B	AX.89454237	5.62E+08	0.28	2.57	3.15	0.06
E1	GY	4B	AX.89570782	5.32E+08	0.32	2.53	3.26	0.06

E1	GY	4B	AX.89754106	5.32E+08	0.32	2.53	3.26	0.06
E1	GPS	4B	AX.89689341	6.43E+08	0.32	-1.19	3.13	0.05
E1	GY	4B	AX.89564249	5.33E+08	0.32	2.53	3.26	0.06
E1	GY	4B	AX.89436365	5.32E+08	0.32	2.53	3.26	0.06
E1	GY	4B	AX.89706082	5.33E+08	0.32	2.53	3.26	0.06
E1	GY	4B	AX.89698141	1.51E+08	0.38	-2.50	3.42	0.05
E1	GY	4B	AX.89711784	6.58E+08	0.09	-4.37	3.56	0.06
E1	GY	4B	AX.89746405	5.42E+08	0.23	3.00	3.70	0.07
E1	GY	4B	AX.89757410	5.62E+08	0.27	2.94	3.87	0.07
E1	GY	4B	AX.89757410	5.62E+08	0.27	2.94	3.87	0.07
E1	GY	4B	AX.89760506	5.32E+08	0.32	2.53	3.26	0.06
E1	GY	4B	AX.89624445	5.33E+08	0.32	2.53	3.26	0.06
E1	GY	4B	AX.89681781	5.31E+08	0.33	2.56	3.37	0.06
E1	GY	4B	AX.89772648	5.21E+08	0.23	3.08	3.85	0.07
E1	GY	4B	AX.89565967	5.62E+08	0.27	2.94	3.87	0.07
E1	GY	4B	AX.89624995	6.13E+08	0.48	-2.38	3.31	0.06
E1	GY	4B	AX.89764432	5.33E+08	0.32	2.53	3.26	0.06
E1	GY	4B	AX.89764432	5.33E+08	0.32	2.53	3.26	0.06
E1	GY	4B	AX.89493063	6.57E+08	0.07	-4.60	3.23	0.05
E1	GY	4B	AX.89599903	4.44E+08	0.18	2.97	3.10	0.05
E1	GY	4B	AX.89628533	5.32E+08	0.32	2.53	3.26	0.06
E1	GY	4B	AX.89680445	5.32E+08	0.32	2.53	3.26	0.06
E1	GY	4B	AX.89748792	5.31E+08	0.33	2.56	3.37	0.06
E1	GY	4B	AX.89748798	5.62E+08	0.27	2.94	3.87	0.07
E1	GY	4B	AX.89626539	5.31E+08	0.33	2.56	3.37	0.06
E1	GY	4B	AX.89462337	5.89E+07	0.37	2.38	3.11	0.06
E1	GY	4B	AX.89688994	5.33E+08	0.32	2.53	3.26	0.06
E1	GY	4B	AX.89433900	5.33E+08	0.32	2.53	3.26	0.06
E1	GY	4B	AX.89534566	5.62E+08	0.28	2.64	3.31	0.06
E1	GY	4B	AX.89637901	6.58E+08	0.08	-4.98	4.09	0.07
E1	GY	4B	AX.89637901	6.58E+08	0.08	-4.98	4.09	0.07
E1	GY	4B	AX.89465158	6.58E+08	0.09	-4.37	3.56	0.06
E1	GY	4B	AX.89497508	5.32E+08	0.32	2.53	3.26	0.06
E1	GY	4B	AX.89762659	5.93E+07	0.36	2.58	3.56	0.06
E1	GY	4B	AX.89718911	6.58E+08	0.09	-4.37	3.56	0.06
E1	GY	4B	AX.89467069	5.33E+08	0.32	2.59	3.41	0.06
E1	GY	4B	AX.89604960	5.31E+08	0.33	2.56	3.37	0.06
E1	GY	4B	AX.89584687	5.63E+08	0.29	2.75	3.58	0.06
E1	GY	4B	AX.89598047	1.68E+08	0.39	-2.38	3.17	0.04
E1	GY	4B	AX.89667528	1.68E+08	0.39	-2.38	3.17	0.04
E1	GY	4B	AX.89667528	1.68E+08	0.39	-2.38	3.17	0.04
E1	GY	4B	AX.89529468	5.32E+08	0.32	2.53	3.26	0.06
E1	GY	4B	AX.89570782	5.32E+08	0.32	2.53	3.26	0.06
E1	GY	4B	AX.89494121	5.33E+08	0.32	2.53	3.26	0.06
E1	GY	4B	AX.89401081	5.31E+08	0.33	2.56	3.37	0.06
E1	GPM2	4B	AX.89375117	9.26E+07	0.43	-706.57	4.20	0.07
E1	GPM2	4B	AX.89414452	5.34E+08	0.23	-779.68	3.73	0.06
E1	GPM2	4B	AX.89647006	5.61E+08	0.23	759.59	3.61	0.06
E1	GPM2	4B	AX.89354231	9.02E+07	0.43	-706.57	4.20	0.07
E1	GPM2	4B	AX.89588328	5.62E+08	0.28	742.19	3.85	0.07

E1	GPM2	4B	AX.89690592	5.34E+08	0.23	-779.68	3.73	0.06
E1	GPM2	4B	AX.89719857	5.35E+08	0.28	-677.27	3.31	0.06
E1	GY	4B	AX.89746602	5.65E+08	0.29	2.75	3.58	0.06
E1	GPM2	4B	AX.89589070	5.61E+08	0.28	742.19	3.85	0.07
E1	GY	4B	AX.89507152	5.30E+08	0.16	-3.12	3.08	0.07
E1	GPM2	4B	AX.89337612	5.34E+08	0.23	-779.68	3.73	0.06
E1	GPM2	4B	AX.89726318	5.34E+08	0.23	-779.68	3.73	0.06
E1	GPM2	4B	AX.89436792	2.23E+08	0.35	-669.91	3.60	0.06
E1	GPM2	4B	AX.89512685	5.87E+08	0.25	704.39	3.32	0.06
E1	GPM2	4B	AX.89761656	5.33E+08	0.32	630.14	3.11	0.05
E1	GPM2	4B	AX.89610360	9.33E+07	0.44	-665.91	3.80	0.06
E1	GPM2	4B	AX.89612979	5.34E+08	0.23	-779.68	3.73	0.06
E1	GPM2	4B	AX.89316538	5.62E+08	0.27	801.15	4.27	0.08
E1	GY	4B	AX.89355602	6.58E+08	0.08	-5.05	4.38	0.07
E1	GPM2	4B	AX.89598047	1.68E+08	0.39	-796.56	5.03	0.08
E1	GY	4B	AX.89752270	5.31E+08	0.33	2.56	3.37	0.06
E1	GPM2	4B	AX.89688994	5.33E+08	0.32	630.14	3.11	0.05
E1	GPM2	4B	AX.89706650	5.61E+08	0.27	729.99	3.67	0.07
E1	GY	4B	AX.89752585	1.97E+08	0.28	2.73	3.52	0.06
E1	GPM2	4B	AX.89762659	5.93E+07	0.36	624.92	3.22	0.06
E1	GPM2	4B	AX.89473460	5.62E+08	0.32	631.70	3.12	0.05
E1	GPM2	4B	AX.89422596	3.75E+08	0.28	739.81	3.86	0.07
E1	GY	4B	AX.89647330	5.61E+08	0.26	2.82	3.55	0.06
E1	GPM2	4B	AX.89349390	5.88E+08	0.25	704.39	3.32	0.06
E1	GPM2	4B	AX.89449412	5.37E+08	0.34	649.05	3.35	0.06
E1	GPM2	4B	AX.89327870	5.62E+08	0.28	742.19	3.85	0.07
E1	GPM2	4B	AX.89502783	1.48E+08	0.39	623.21	3.29	0.05
E1	GPM2	4B	AX.89400160	5.92E+08	0.26	662.79	3.02	0.05
E1	GPM2	4B	AX.89456967	5.62E+08	0.28	742.19	3.85	0.07
E1	GPM2	4B	AX.89358319	5.34E+08	0.23	-779.68	3.73	0.06
E1	GY	4B	AX.89642919	5.33E+08	0.32	2.53	3.26	0.06
E1	GPM2	4B	AX.89599985	5.62E+08	0.28	742.19	3.85	0.07
E1	GY	4B	AX.89771775	6.58E+08	0.09	-4.37	3.56	0.06
E1	GPM2	4B	AX.89516995	2.86E+08	0.35	-716.14	4.02	0.06
E1	GPM2	4B	AX.89373348	8.99E+07	0.47	-621.30	3.40	0.06
E1	GY	4B	AX.89616929	5.71E+08	0.28	2.57	3.14	0.06
E1	GPM2	4B	AX.89493700	5.34E+08	0.23	-779.68	3.73	0.06
E1	GPM2	4B	AX.89698141	1.51E+08	0.38	-840.80	5.51	0.09
E1	GY	4B	AX.89534566	5.62E+08	0.28	2.64	3.31	0.06
E1	GPM2	4B	AX.89361217	5.33E+08	0.32	630.14	3.11	0.05
E1	GY	4B	AX.89569510	5.62E+08	0.27	2.94	3.87	0.07
E1	GY	4B	AX.89569510	5.62E+08	0.27	2.94	3.87	0.07
E1	GPM2	4B	AX.89412641	8.99E+07	0.41	-592.31	3.07	0.05
E1	GPM2	4B	AX.86162512	5.32E+08	0.32	630.14	3.11	0.05
E1	GY	4B	AX.89476307	1.95E+08	0.27	2.62	3.22	0.06
E1	GY	4B	AX.89476307	1.95E+08	0.27	2.62	3.22	0.06
E1	GY	4B	AX.89586202	6.58E+08	0.09	-4.37	3.56	0.06
E1	GY	4B	AX.89729866	6.58E+08	0.09	-4.37	3.56	0.06
E1	SPM2	4B	AX.89575951	6.42E+08	0.32	18.92	3.47	0.06
E1	GY	4B	AX.89477195	5.32E+08	0.32	2.53	3.26	0.06

E1	GPM2	4B	AX.89637901	6.58E+08	0.08	-1286.22	4.14	0.07
E1	GY	4B	AX.89683540	5.62E+08	0.26	2.75	3.39	0.06
E1	GPM2	4B	AX.89737295	5.34E+08	0.23	-779.68	3.73	0.06
E1	GPM2	4B	AX.89465158	6.58E+08	0.09	-1103.87	3.46	0.06
E1	GPM2	4B	AX.89604997	1.43E+08	0.39	623.21	3.29	0.05
E1	GPM2	4B	AX.89667145	1.68E+08	0.39	-796.56	5.03	0.08
E1	GPM2	4B	AX.89708109	5.61E+08	0.26	740.31	3.69	0.07
E1	GPM2	4B	AX.89692741	5.31E+08	0.33	624.61	3.08	0.05
E1	GPM2	4B	AX.89681213	5.61E+08	0.27	729.99	3.67	0.07
E1	GY	4B	AX.89478754	5.31E+08	0.33	2.56	3.37	0.06
E1	GPM2	4B	AX.89414000	5.62E+08	0.27	836.33	4.65	0.09
E1	GPM2	4B	AX.89624445	5.33E+08	0.32	630.14	3.11	0.05
E1	GPM2	4B	AX.89476043	6.58E+08	0.09	-1000.58	3.05	0.05
E1	GPM2	4B	AX.89633674	9.35E+07	0.43	-649.05	3.62	0.06
E1	GY	4B	AX.89549282	5.33E+08	0.32	2.53	3.26	0.06
E1	GPM2	4B	AX.89667528	1.68E+08	0.39	-796.56	5.03	0.08
E1	GPM2	4B	AX.89578164	5.33E+08	0.32	630.14	3.11	0.05
E1	GPS	4B	AX.89582795	6.43E+08	0.32	-1.19	3.13	0.05
E1	GY	4B	AX.89483878	5.93E+08	0.21	-2.92	3.30	0.05
E1	GPM2	4B	AX.89486798	5.62E+08	0.28	742.19	3.85	0.07
E1	GPM2	4B	AX.89571745	5.32E+08	0.32	630.14	3.11	0.05
E1	GPM2	4B	AX.89518901	1.41E+08	0.34	641.16	3.28	0.05
E1	GPM2	4B	AX.89628533	5.32E+08	0.32	630.14	3.11	0.05
E1	GPM2	4B	AX.89765585	9.29E+07	0.43	-706.57	4.20	0.07
E1	GPM2	4B	AX.89354374	5.60E+08	0.26	826.66	4.47	0.08
E1	GPM2	4B	AX.89634563	2.05E+08	0.38	-717.14	4.16	0.07
E1	GY	4B	AX.89446221	1.72E+08	0.39	-2.38	3.17	0.04
E1	GPM2	4B	AX.89371502	5.37E+08	0.23	742.53	3.47	0.06
E1	GPM2	4B	AX.89512983	5.34E+08	0.32	630.14	3.11	0.05
E1	GPM2	4B	AX.89769229	5.31E+08	0.33	624.61	3.08	0.05
E1	GPS	4B	AX.89548012	6.43E+08	0.32	-1.19	3.13	0.05
E1	GPM2	4B	AX.89697036	1.66E+08	0.38	-751.74	4.50	0.07
E1	GY	4B	AX.89761656	5.33E+08	0.32	2.53	3.26	0.06
E1	GY	4B	AX.89761656	5.33E+08	0.32	2.53	3.26	0.06
E1	GY	4B	AX.89454237	5.62E+08	0.28	2.57	3.15	0.06
E1	GPM2	4B	AX.89380326	5.62E+08	0.25	785.31	4.00	0.07
E1	GPM2	4B	AX.89497508	5.32E+08	0.32	630.14	3.11	0.05
E1	GPM2	4B	AX.89356943	5.60E+08	0.27	754.72	3.85	0.07
E1	GPM2	4B	AX.89397095	5.33E+08	0.32	630.14	3.11	0.05
E1	GPM2	4B	AX.89623664	9.29E+07	0.43	-706.57	4.20	0.07
E1	GPM2	4B	AX.89449444	5.62E+08	0.27	797.52	4.28	0.08
E1	GPS	4B	AX.89420848	6.43E+08	0.32	-1.19	3.13	0.05
E1	GPM2	4B	AX.89674519	5.60E+08	0.27	729.99	3.67	0.07
E1	GPM2	4B	AX.89430244	5.31E+08	0.33	624.61	3.08	0.05
E1	GPM2	4B	AX.89649978	5.61E+08	0.27	729.99	3.67	0.07
E1	GPM2	4B	AX.89452772	5.62E+08	0.27	843.59	4.77	0.09
E1	GY	4B	AX.89660931	1.55E+08	0.38	-2.33	3.02	0.04
E1	GPM2	4B	AX.89465925	8.99E+07	0.47	-621.30	3.40	0.06
E1	GPM2	4B	AX.89360130	5.34E+08	0.23	-779.68	3.73	0.06
E1	GPM2	4B	AX.89626539	5.31E+08	0.33	624.61	3.08	0.05

E1	GPM2	4B	AX.89352705	5.31E+08	0.33	624.61	3.08	0.05
E1	GY	4B	AX.89555995	5.93E+08	0.21	-2.92	3.30	0.05
E1	GPS	4B	AX.89540061	6.43E+08	0.31	-1.21	3.17	0.05
E1	GPM2	4B	AX.89578890	2.98E+08	0.34	-664.15	3.49	0.05
E1	GY	4B	AX.89422024	5.33E+08	0.32	2.53	3.26	0.06
E1	GPM2	4B	AX.89775585	5.34E+08	0.23	-779.68	3.73	0.06
E1	GPM2	4B	AX.89757114	5.31E+08	0.33	624.61	3.08	0.05
E1	GPM2	4B	AX.89604621	5.34E+08	0.23	-779.68	3.73	0.06
E1	GY	4B	AX.89624995	6.13E+08	0.48	-2.38	3.31	0.06
E1	GPM2	4B	AX.89669348	5.60E+08	0.26	826.66	4.47	0.08
E1	GPM2	4B	AX.89636790	6.58E+08	0.09	-1103.87	3.46	0.06
E1	GPM2	4B	AX.89534566	5.62E+08	0.28	750.60	3.96	0.07
E1	GPM2	4B	AX.89378611	1.60E+08	0.39	-796.56	5.03	0.08
E1	GPM2	4B	AX.89757410	5.62E+08	0.27	836.33	4.65	0.09
E1	GPS	4B	AX.86164394	6.43E+08	0.32	-1.19	3.13	0.05
E1	GY	4B	AX.89622164	3.44E+08	0.28	2.73	3.52	0.06
E1	GPM2	4B	AX.89427218	1.44E+08	0.39	623.21	3.29	0.05
E1	GY	4B	AX.89692741	5.31E+08	0.33	2.56	3.37	0.06
E1	GY	4B	AX.89692741	5.31E+08	0.33	2.56	3.37	0.06
E1	GPM2	4B	AX.89583019	5.34E+08	0.23	-779.68	3.73	0.06
E1	GPM2	4B	AX.89322090	5.34E+08	0.23	-779.68	3.73	0.06
E1	GPS	4B	AX.89630537	6.43E+08	0.32	-1.19	3.13	0.05
E1	GPM2	4B	AX.89580725	5.33E+08	0.32	630.14	3.11	0.05
E1	GPM2	4B	AX.89436365	5.32E+08	0.32	630.14	3.11	0.05
E1	GPM2	4B	AX.89748792	5.31E+08	0.33	624.61	3.08	0.05
E1	GPS	4B	AX.89517394	6.43E+08	0.32	-1.19	3.13	0.05
E1	GY	4B	AX.89726177	5.33E+08	0.32	2.53	3.26	0.06
E1	GPM2	4B	AX.89718135	5.31E+08	0.33	624.61	3.08	0.05
E1	GY	4B	AX.89518901	1.41E+08	0.34	2.40	3.07	0.05
E1	GY	4B	AX.89616304	5.60E+08	0.26	2.82	3.55	0.06
E1	GPM2	4B	AX.89407311	5.62E+08	0.27	836.33	4.65	0.09
E1	GPM2	4B	AX.89430932	6.58E+08	0.09	-1072.67	3.30	0.05
E1	GPM2	4B	AX.89608524	5.35E+08	0.28	-677.27	3.31	0.06
E1	GY	4B	AX.89694641	5.32E+08	0.32	2.53	3.26	0.06
E1	GY	4B	AX.89694641	5.32E+08	0.32	2.53	3.26	0.06
E1	GPM2	4B	AX.89368975	5.34E+08	0.23	-779.68	3.73	0.06
E1	GPM2	4B	AX.89566116	1.60E+08	0.38	-825.48	5.31	0.09
E1	GPM2	4B	AX.89439700	5.31E+08	0.33	624.61	3.08	0.05
E1	GY	4B	AX.89430244	5.31E+08	0.33	2.56	3.37	0.06
E1	GY	4B	AX.89452772	5.62E+08	0.27	2.99	4.04	0.07
E1	GPM2	4B	AX.89604806	9.20E+07	0.44	-684.50	3.98	0.07
E1	GPM2	4B	AX.89677849	1.40E+08	0.40	-786.27	4.99	0.09
E1	GPM2	4B	AX.89667185	6.58E+08	0.08	-1253.68	4.14	0.07
E1	GPM2	4B	AX.89410817	5.34E+08	0.23	-779.68	3.73	0.06
E1	GPM2	4B	AX.89743825	5.32E+08	0.32	630.14	3.11	0.05
E1	GY	4B	AX.89657621	5.32E+08	0.32	2.53	3.26	0.06
E1	GPS	4B	AX.89355046	6.43E+08	0.32	-1.19	3.13	0.05
E1	GPM2	4B	AX.89752585	1.97E+08	0.28	739.81	3.86	0.07
E1	GY	4B	AX.89566116	1.60E+08	0.38	-2.33	3.02	0.04
E1	GPM2	4B	AX.89490930	5.34E+08	0.23	-779.68	3.73	0.06

E1	GPM2	4B	AX.89634709	5.34E+08	0.23	-779.68	3.73	0.06
E1	GPM2	4B	AX.89746405	5.42E+08	0.23	742.53	3.47	0.06
E1	GPM2	4B	AX.89678567	5.32E+08	0.32	630.14	3.11	0.05
E1	GPM2	4B	AX.89611300	1.67E+08	0.39	-796.56	5.03	0.08
E1	GPM2	4B	AX.89776240	5.31E+08	0.33	624.61	3.08	0.05
E1	GY	4B	AX.89316572	1.33E+07	0.05	-5.41	3.32	0.05
E1	GPM2	4B	AX.89708001	5.35E+08	0.28	-677.27	3.31	0.06
E1	GPM2	4B	AX.89458124	5.34E+08	0.23	-779.68	3.73	0.06
E1	GPM2	4B	AX.89595080	1.45E+08	0.39	-768.35	4.74	0.08
E1	GPM2	4B	AX.89642919	5.33E+08	0.32	630.14	3.11	0.05
E1	GPM2	4B	AX.89626371	1.66E+08	0.39	-796.56	5.03	0.08
E1	GY	4B	AX.89338729	5.33E+08	0.32	2.53	3.26	0.06
E1	GY	4B	AX.89736658	5.22E+08	0.24	2.96	3.65	0.07
E1	GPM2	4B	AX.89357532	1.40E+08	0.40	-771.09	4.82	0.08
E1	GY	4B	AX.89734003	6.58E+08	0.09	-4.37	3.56	0.06
E1	GY	4B	AX.89734003	6.58E+08	0.09	-4.37	3.56	0.06
E1	GPM2	4B	AX.89637611	5.31E+08	0.33	624.61	3.08	0.05
E1	GPM2	4B	AX.89564249	5.33E+08	0.32	630.14	3.11	0.05
E1	GPM2	4B	AX.89462337	5.89E+07	0.37	602.01	3.04	0.05
E1	GPM2	4B	AX.89561827	5.32E+08	0.32	630.14	3.11	0.05
E1	GY	4B	AX.89641200	6.58E+08	0.12	-3.58	3.12	0.06
E1	GPM2	4B	AX.89562245	5.33E+08	0.32	630.14	3.11	0.05
E1	GPM2	4B	AX.89694641	5.32E+08	0.32	630.14	3.11	0.05
E1	GPM2	4B	AX.89535038	5.58E+08	0.26	790.92	4.09	0.07
E1	GPM2	4B	AX.89519378	5.61E+08	0.27	688.02	3.28	0.06
E1	GY	4B	AX.89317811	5.31E+08	0.33	2.56	3.37	0.06
E1	GPM2	4B	AX.89338729	5.33E+08	0.32	630.14	3.11	0.05
E1	GPM2	4B	AX.89331903	6.58E+08	0.09	-1103.87	3.46	0.06
E1	GPM2	4B	AX.89616304	5.60E+08	0.26	826.66	4.47	0.08
E1	GPM2	4B	AX.89390027	5.34E+08	0.23	-779.68	3.73	0.06
E1	GPM2	4B	AX.89507152	5.30E+08	0.16	-817.26	3.18	0.06
E1	GPM2	4B	AX.89347833	5.62E+08	0.28	742.19	3.85	0.07
E1	GPM2	4B	AX.89638092	3.79E+08	0.34	-700.55	3.85	0.06
E1	GPM2	4B	AX.89759725	5.33E+08	0.32	630.14	3.11	0.05
E1	GY	4B	AX.89768552	5.31E+08	0.33	2.56	3.37	0.06
E1	GY	4B	AX.89768552	5.31E+08	0.33	2.56	3.37	0.06
E1	GPM2	4B	AX.89578373	1.40E+08	0.41	-744.98	4.56	0.07
E1	GY	4B	AX.89436365	5.32E+08	0.32	2.53	3.26	0.06
E1	GPM2	4B	AX.89764432	5.33E+08	0.32	630.14	3.11	0.05
E1	GPM2	4B	AX.89381343	5.60E+08	0.27	836.33	4.65	0.09
E1	GPM2	4B	AX.89586202	6.58E+08	0.09	-1103.87	3.46	0.06
E1	GY	4B	AX.89706082	5.33E+08	0.32	2.53	3.26	0.06
E1	GY	4B	AX.89759725	5.33E+08	0.32	2.53	3.26	0.06
E1	GPM2	4B	AX.89389357	6.58E+08	0.08	-1253.68	4.14	0.07
E1	GY	4B	AX.89681781	5.31E+08	0.33	2.56	3.37	0.06
E1	GPM2	4B	AX.89319673	5.32E+08	0.32	630.14	3.11	0.05
E1	GPM2	4B	AX.89489081	5.35E+08	0.23	-779.68	3.73	0.06
E1	GPM2	4B	AX.89476307	1.95E+08	0.27	649.01	3.03	0.05
E1	GY	4B	AX.89553918	1.68E+08	0.39	-2.38	3.17	0.04
E1	GPM2	4B	AX.89404249	5.32E+08	0.32	630.14	3.11	0.05

E1	GPM2	4B	AX.89354461	5.33E+08	0.32	630.14	3.11	0.05
E1	GPM2	4B	AX.89672975	5.60E+08	0.26	826.66	4.47	0.08
E1	GPM2	4B	AX.89565967	5.62E+08	0.27	836.33	4.65	0.09
E1	GPM2	4B	AX.89757603	5.35E+08	0.28	-677.27	3.31	0.06
E1	GPM2	4B	AX.89433900	5.33E+08	0.32	630.14	3.11	0.05
E1	GY	4B	AX.89680445	5.32E+08	0.32	2.53	3.26	0.06
E1	GY	4B	AX.89748792	5.31E+08	0.33	2.56	3.37	0.06
E1	GY	4B	AX.89518901	1.41E+08	0.34	2.40	3.07	0.05
E1	GPM2	4B	AX.89446221	1.72E+08	0.39	-796.56	5.03	0.08
E1	GY	4B	AX.89535038	5.58E+08	0.26	2.89	3.66	0.07
E1	GY	4B	AX.89465158	6.58E+08	0.09	-4.37	3.56	0.06
E1	GY	4B	AX.89497508	5.32E+08	0.32	2.53	3.26	0.06
E1	GY	4B	AX.89562245	5.33E+08	0.32	2.53	3.26	0.06
E1	GY	4B	AX.89718135	5.31E+08	0.33	2.56	3.37	0.06
E1	GY	4B	AX.89763483	6.58E+08	0.08	-5.05	4.38	0.07
E1	GPM2	4B	AX.89381638	5.34E+08	0.23	-779.68	3.73	0.06
E1	GPM2	4B	AX.89482598	5.34E+08	0.23	-779.68	3.73	0.06
E1	GPM2	4B	AX.89487605	5.62E+08	0.32	928.97	6.10	0.12
E1	GPM2	4B	AX.89475860	5.32E+08	0.32	630.14	3.11	0.05
E1	GPM2	4B	AX.89641200	6.58E+08	0.12	-910.15	3.07	0.05
E1	GPM2	4B	AX.89519558	5.34E+08	0.22	-841.11	4.20	0.07
E1	GPM2	4B	AX.89763140	5.34E+08	0.23	-779.68	3.73	0.06
E1	GPM2	4B	AX.89478754	5.31E+08	0.33	624.61	3.08	0.05
E1	GPM2	4B	AX.89594770	5.95E+07	0.36	624.92	3.22	0.06
E1	GPM2	4B	AX.89681781	5.31E+08	0.33	624.61	3.08	0.05
E1	GPM2	4B	AX.89364224	6.58E+08	0.09	-1103.87	3.46	0.06
E1	GPM2	4B	AX.89548773	5.61E+08	0.27	729.99	3.67	0.07
E1	GPM2	4B	AX.89462202	5.61E+08	0.28	742.19	3.85	0.07
E1	GPM2	4B	AX.89384733	9.48E+07	0.43	-649.05	3.62	0.06
E1	GY	4B	AX.89752585	1.97E+08	0.28	2.73	3.52	0.06
E1	GY	4B	AX.89473827	4.28E+08	0.45	2.31	3.11	0.05
E1	GY	4B	AX.89473827	4.28E+08	0.45	2.31	3.11	0.05
E1	GPM2	4B	AX.89436755	1.98E+06	0.25	694.04	3.23	0.06
E1	GPM2	4B	AX.89355602	6.58E+08	0.08	-1253.68	4.14	0.07
E1	GPM2	4B	AX.89415532	5.32E+08	0.32	630.14	3.11	0.05
E1	GPM2	4B	AX.89457648	5.61E+08	0.27	729.99	3.67	0.07
E1	GPM2	4B	AX.89349594	5.61E+08	0.27	729.99	3.67	0.07
E1	GPM2	4B	AX.89514930	5.33E+08	0.32	630.14	3.11	0.05
E1	GPM2	4B	AX.89706082	5.33E+08	0.32	630.14	3.11	0.05
E1	GY	4B	AX.89512210	6.57E+08	0.07	-4.60	3.23	0.05
E1	GY	4B	AX.89475860	5.32E+08	0.32	2.53	3.26	0.06
E1	GY	4B	AX.89561827	5.32E+08	0.32	2.53	3.26	0.06
E1	GPM2	4B	AX.89769498	1.59E+08	0.39	623.21	3.29	0.05
E1	GPM2	4B	AX.89533727	5.33E+08	0.32	630.14	3.11	0.05
E1	GPM2	4B	AX.89661370	5.62E+08	0.27	803.14	4.33	0.08
E1	GPM2	4B	AX.89644343	5.34E+08	0.23	-779.68	3.73	0.06
E1	GPM2	4B	AX.89409367	5.34E+08	0.23	-740.69	3.46	0.06
E1	GY	4B	AX.89439700	5.31E+08	0.33	2.56	3.37	0.06
E1	GPM2	4B	AX.89711784	6.58E+08	0.09	-1103.87	3.46	0.06
E1	GPM2	4B	AX.89681180	5.31E+08	0.33	624.61	3.08	0.05

E1	GPM2	4B	AX.89746602	5.65E+08	0.29	752.47	4.01	0.07
E1	GPM2	4B	AX.89706716	5.34E+08	0.23	-779.68	3.73	0.06
E1	GPM2	4B	AX.89569510	5.62E+08	0.27	836.33	4.65	0.09
E1	GPM2	4B	AX.89477195	5.32E+08	0.32	630.14	3.11	0.05
E1	GPM2	4B	AX.89344896	1.62E+08	0.38	-840.80	5.51	0.09
E1	GY	4B	AX.89580725	5.33E+08	0.32	2.53	3.26	0.06
E1	GPM2	4B	AX.89376848	2.15E+08	0.28	739.81	3.86	0.07
E1	GPM2	4B	AX.89364679	5.35E+08	0.23	-779.68	3.73	0.06
E1	GPM2	4B	AX.89718911	6.58E+08	0.09	-1103.87	3.46	0.06
E1	GPM2	4B	AX.89604960	5.31E+08	0.33	624.61	3.08	0.05
E1	GPM2	4B	AX.89767527	5.34E+08	0.23	-779.68	3.73	0.06
E1	GY	4B	AX.89637611	5.31E+08	0.33	2.56	3.37	0.06
E1	GY	4B	AX.89446221	1.72E+08	0.39	-2.38	3.17	0.04
E1	GPM2	4B	AX.89565835	5.34E+08	0.23	-779.68	3.73	0.06
E1	GPM2	4B	AX.89606985	5.25E+08	0.16	-834.73	3.37	0.07
E1	GPM2	4B	AX.89760506	5.32E+08	0.32	630.14	3.11	0.05
E1	GPM2	4B	AX.89637662	9.30E+07	0.42	-623.21	3.35	0.06
E1	GPM2	4B	AX.89736658	5.22E+08	0.24	694.11	3.13	0.05
E1	GPM2	4B	AX.89529379	5.33E+08	0.32	630.14	3.11	0.05
E1	GPM2	4B	AX.89497398	5.32E+08	0.32	630.14	3.11	0.05
E1	GPM2	4B	AX.89713958	5.34E+08	0.24	-732.33	3.47	0.06
E1	GPM2	4B	AX.89438370	5.34E+08	0.23	-779.68	3.73	0.06
E1	GPM2	4B	AX.89705801	6.58E+08	0.09	-1103.87	3.46	0.06
E1	GPM2	4B	AX.89639885	1.60E+08	0.39	-738.80	4.40	0.07
E1	GPM2	4B	AX.89746587	1.40E+08	0.41	-728.72	4.37	0.07
E1	GPM2	4B	AX.89496758	1.65E+08	0.39	-796.56	5.03	0.08
E1	GPM2	4B	AX.89657621	5.32E+08	0.32	630.14	3.11	0.05
E1	GPM2	4B	AX.89723602	5.31E+08	0.33	624.61	3.08	0.05
E1	GPM2	4B	AX.89323722	5.60E+08	0.26	826.66	4.47	0.08
E1	GY	4B	AX.89648611	6.57E+08	0.07	-4.60	3.23	0.05
E1	GPM2	4B	AX.89531900	5.62E+08	0.27	836.33	4.65	0.09
E1	GPM2	4B	AX.89344317	5.34E+08	0.23	-779.68	3.73	0.06
E1	GPM2	4B	AX.89586718	5.35E+08	0.23	-779.68	3.73	0.06
E1	GPM2	4B	AX.89494121	5.33E+08	0.32	630.14	3.11	0.05
E1	GPM2	4B	AX.89643271	5.33E+08	0.32	630.14	3.11	0.05
E1	GPM2	4B	AX.89450047	6.58E+08	0.09	-1103.87	3.46	0.06
E1	GPM2	4B	AX.89662922	1.40E+08	0.40	-771.09	4.82	0.08
E1	GPM2	4B	AX.89503045	5.34E+08	0.23	-779.68	3.73	0.06
E1	GPM2	4B	AX.89719542	6.58E+08	0.08	-1253.68	4.14	0.07
E1	GPM2	4B	AX.89331387	5.60E+08	0.27	729.99	3.67	0.07
E1	GPM2	4B	AX.89317768	5.34E+08	0.23	-779.68	3.73	0.06
E1	GPM2	4B	AX.89678724	6.58E+08	0.09	-1103.87	3.46	0.06
E1	GPM2	4B	AX.89559622	5.34E+08	0.23	-779.68	3.73	0.06
E1	GPM2	4B	AX.89647330	5.61E+08	0.26	826.66	4.47	0.08
E1	GPM2	4B	AX.89738577	5.34E+08	0.22	-841.11	4.20	0.07
E1	GPM2	4B	AX.89683540	5.62E+08	0.26	836.64	4.57	0.08
E1	GPM2	4B	AX.89656772	5.34E+08	0.23	-779.68	3.73	0.06
E1	GPM2	4B	AX.89754106	5.32E+08	0.32	630.14	3.11	0.05
E1	GPM2	4B	AX.89336970	5.32E+08	0.32	630.14	3.11	0.05
E1	GPM2	4B	AX.89771775	6.58E+08	0.09	-1103.87	3.46	0.06

E1	GY	4B	AX.89726177	5.33E+08	0.32	2.53	3.26	0.06
E1	GPM2	4B	AX.89673916	5.32E+08	0.32	630.14	3.11	0.05
E1	GPM2	4B	AX.89445465	1.40E+08	0.40	-771.09	4.82	0.08
E1	GPM2	4B	AX.89553918	1.68E+08	0.39	-796.56	5.03	0.08
E1	GPM2	4B	AX.89508190	5.61E+08	0.26	782.79	4.02	0.07
E1	GPM2	4B	AX.89508272	5.62E+08	0.28	742.19	3.85	0.07
E1	GPM2	4B	AX.89422294	5.34E+08	0.23	-779.68	3.73	0.06
E1	GPM2	4B	AX.89650641	5.61E+08	0.27	729.99	3.67	0.07
E1	GPM2	4B	AX.89748798	5.62E+08	0.27	836.33	4.65	0.09
E1	GPM2	4B	AX.89401081	5.31E+08	0.33	624.61	3.08	0.05
E1	TKW	4B	AX.89624776	8.26E+05	0.20	1.30	4.52	0.08
E1	GPM2	4B	AX.89377927	5.32E+08	0.32	630.14	3.11	0.05
E1	GPM2	4B	AX.89354183	9.20E+07	0.43	-706.57	4.20	0.07
E1	GPM2	4B	AX.89729866	6.58E+08	0.09	-1103.87	3.46	0.06
E1	GPM2	4B	AX.89642522	5.32E+08	0.32	630.14	3.11	0.05
E1	GPM2	4B	AX.89426215	8.99E+07	0.41	-592.31	3.07	0.05
E1	GPM2	4B	AX.89622164	3.44E+08	0.28	739.81	3.86	0.07
E1	GPM2	4B	AX.89372260	5.33E+08	0.32	630.14	3.11	0.05
E1	GPM2	4B	AX.89655632	1.60E+08	0.44	-748.67	4.66	0.07
E1	GPM2	4B	AX.89419922	5.32E+08	0.32	630.14	3.11	0.05
E1	GY	4B	AX.89562245	5.33E+08	0.32	2.53	3.26	0.06
E1	GPM2	4B	AX.89595313	5.34E+08	0.23	-779.68	3.73	0.06
E1	GPM2	4B	AX.89730387	1.72E+08	0.39	-796.56	5.03	0.08
E1	GPM2	4B	AX.89640443	5.34E+08	0.23	-779.68	3.73	0.06
E1	GPM2	4B	AX.89366415	5.34E+08	0.23	-779.68	3.73	0.06
E1	GPM2	4B	AX.89467293	9.37E+07	0.43	-649.05	3.62	0.06
E1	GPM2	4B	AX.89752270	5.31E+08	0.33	624.61	3.08	0.05
E1	GPM2	4B	AX.89467518	2.19E+08	0.40	-655.47	3.63	0.06
E1	GPM2	4B	AX.89609574	5.61E+08	0.27	729.99	3.67	0.07
E1	GPM2	4B	AX.89682209	5.33E+08	0.32	630.14	3.11	0.05
E1	GPM2	4B	AX.89654105	5.35E+08	0.23	-779.68	3.73	0.06
E1	GPM2	4B	AX.89441425	5.34E+08	0.23	-779.68	3.73	0.06
E1	GPM2	4B	AX.89673378	5.34E+08	0.23	-779.68	3.73	0.06
E1	GY	4B	AX.89736658	5.22E+08	0.24	2.96	3.65	0.07
E1	GPM2	4B	AX.89368909	5.34E+08	0.23	-742.38	3.43	0.06
E1	GPM2	4B	AX.89497126	5.32E+08	0.32	630.14	3.11	0.05
E1	GPM2	4B	AX.89476160	1.72E+08	0.39	-796.56	5.03	0.08
E1	TKW	4B	AX.89311841	1.09E+06	0.20	1.30	4.52	0.08
E1	GPM2	4B	AX.89361958	5.31E+08	0.33	624.61	3.08	0.05
E1	GPM2	4B	AX.89514433	5.34E+08	0.23	-779.68	3.73	0.06
E1	GPM2	4B	AX.89433824	6.58E+08	0.08	-1253.68	4.14	0.07
E1	GPM2	4B	AX.89557504	6.58E+08	0.08	-1253.68	4.14	0.07
E1	GY	4B	AX.89636790	6.58E+08	0.09	-4.37	3.56	0.06
E1	GPM2	4B	AX.89701032	6.58E+08	0.07	-1259.99	3.80	0.06
E1	GPM2	4B	AX.89687239	9.26E+07	0.41	-592.31	3.07	0.05
E1	GPM2	4B	AX.89763483	6.58E+08	0.08	-1253.68	4.14	0.07
E1	GPM2	4B	AX.89723548	9.11E+07	0.42	-677.82	3.89	0.07
E1	GPM2	4B	AX.89604119	5.34E+08	0.23	-779.68	3.73	0.06
E1	TKW	4B	AX.89688587	1.06E+06	0.13	1.45	3.98	0.06
E1	GPM2	4B	AX.89472483	5.62E+08	0.27	838.10	4.72	0.09

E1	GPM2	4B	AX.89616929	5.71E+08	0.28	681.65	3.32	0.06
E1	GPM2	4B	AX.89557593	5.62E+08	0.28	742.19	3.85	0.07
E1	TKW	4B	AX.89316017	1.05E+06	0.16	1.19	3.25	0.05
E1	GPM2	4B	AX.89584687	5.63E+08	0.29	752.47	4.01	0.07
E1	GPM2	4B	AX.89768552	5.31E+08	0.33	624.61	3.08	0.05
E1	GPM2	4B	AX.89669977	1.65E+08	0.39	-796.56	5.03	0.08
E1	GY	4B	AX.89711784	6.58E+08	0.09	-4.37	3.56	0.06
E1	GY	4B	AX.89759725	5.33E+08	0.32	2.53	3.26	0.06
E1	GPM2	4B	AX.89388981	1.69E+08	0.39	-793.07	4.99	0.08
E1	GPM2	4B	AX.89437061	5.34E+08	0.23	-744.02	3.48	0.06
E1	GPM2	4B	AX.89422024	5.33E+08	0.32	630.14	3.11	0.05
E1	GPM2	4B	AX.89344798	5.33E+08	0.32	630.14	3.11	0.05
E1	GPM2	4B	AX.89677795	5.60E+08	0.27	729.99	3.67	0.07
E1	GPM2	4B	AX.89777674	5.35E+08	0.37	619.71	3.19	0.06
E1	GPM2	4B	AX.89757598	5.34E+08	0.23	-707.24	3.19	0.05
E1	GPM2	4B	AX.89726177	5.33E+08	0.32	630.14	3.11	0.05
E1	GY	4B	AX.89493063	6.57E+08	0.07	-4.60	3.23	0.05
E1	GPM2	4B	AX.89576163	5.31E+08	0.33	610.66	3.00	0.05
E1	GPM2	4B	AX.89680445	5.32E+08	0.32	630.14	3.11	0.05
E1	TKW	4B	AX.89516841	1.06E+06	0.13	1.45	3.98	0.06
E1	GY	4B	AX.89688994	5.33E+08	0.32	2.53	3.26	0.06
E1	TKW	4B	AX.89405164	1.05E+06	0.20	1.30	4.52	0.08
E1	TKW	4B	AX.89603801	8.23E+05	0.15	1.39	4.15	0.07
E1	TKW	4B	AX.89598155	1.09E+06	0.13	1.45	3.98	0.06
E1	GPM2	4B	AX.89653815	5.60E+08	0.26	803.67	4.25	0.08
E1	GPM2	4B	AX.89429904	5.34E+08	0.23	-779.68	3.73	0.06
E1	GPM2	4B	AX.89529468	5.32E+08	0.32	630.14	3.11	0.05
E1	GPM2	4B	AX.89604722	5.34E+08	0.23	-779.68	3.73	0.06
E1	GPM2	4B	AX.89317811	5.31E+08	0.33	624.61	3.08	0.05
E1	GPM2	4B	AX.89683137	6.58E+08	0.09	-1103.87	3.46	0.06
E1	GPM2	4B	AX.89626357	5.33E+08	0.32	630.14	3.11	0.05
E1	GY	4B	AX.89723602	5.31E+08	0.33	2.56	3.37	0.06
E1	GPM2	4B	AX.89570782	5.32E+08	0.32	630.14	3.11	0.05
E1	GPM2	4B	AX.89422233	9.51E+07	0.43	-649.05	3.62	0.06
E1	GPM2	4B	AX.89432437	5.60E+08	0.33	611.96	3.01	0.05
E1	GPM2	4B	AX.89502649	5.34E+08	0.23	-779.68	3.73	0.06
E1	TKW	4B	AX.89700283	1.05E+06	0.15	1.39	4.15	0.07
E1	GY	4B	AX.89701032	6.58E+08	0.07	-4.88	3.76	0.06
E1	GPM2	4B	AX.89454237	5.62E+08	0.28	742.19	3.85	0.07
E1	GPM2	4B	AX.89734223	9.35E+07	0.34	-643.62	3.32	0.06
E1	GPM2	4B	AX.89660931	1.55E+08	0.38	-825.48	5.31	0.09
E1	GPM2	4B	AX.89330007	6.58E+08	0.08	-1121.40	3.41	0.05
E1	GPM2	4B	AX.89355730	5.32E+08	0.32	630.14	3.11	0.05
E1	GPM2	4B	AX.89753097	9.30E+07	0.44	-665.91	3.80	0.06
E1	GPM2	4B	AX.89467069	5.33E+08	0.32	707.71	3.80	0.07
E1	GPM2	4B	AX.89731604	9.37E+07	0.43	-649.05	3.62	0.06
E1	TKW	4B	AX.89470538	1.09E+06	0.18	1.32	4.27	0.07
E1	TKW	4B	AX.89472953	1.09E+06	0.13	1.45	3.98	0.06
E1	TKW	4B	AX.89326548	8.15E+05	0.11	1.44	3.41	0.05
E1	TKW	4B	AX.89376768	1.06E+06	0.13	1.45	3.98	0.06

E1	GPM2	4B	AX.89381203	5.34E+08	0.23	-779.68	3.73	0.06
E1	GPM2	4B	AX.89734003	6.58E+08	0.09	-1103.87	3.46	0.06
E1	TKW	4B	AX.89511155	1.05E+06	0.18	1.32	4.27	0.07
E1	TKW	4B	AX.89582667	1.06E+06	0.16	1.19	3.27	0.05
E1	GPM2	4B	AX.89725528	5.88E+08	0.25	704.39	3.32	0.06
E1	GPM2	4B	AX.89549282	5.33E+08	0.32	630.14	3.11	0.05
E1	GPM2	4B	AX.89772648	5.21E+08	0.23	716.42	3.27	0.06
E1	GPM2	4B	AX.89434819	5.62E+08	0.32	631.70	3.12	0.05
E1	TKW	4B	AX.89608391	1.05E+06	0.18	1.32	4.27	0.07
E1	TKW	4B	AX.89330961	1.05E+06	0.13	1.45	3.98	0.06
E1	TKW	4B	AX.89673766	1.08E+06	0.13	1.45	3.98	0.06
E1	TKW	4B	AX.89501151	1.08E+06	0.13	1.45	3.98	0.06
E1	TKW	4B	AX.89732228	1.06E+06	0.13	1.45	3.98	0.06
E1	TKW	4B	AX.89398014	1.12E+06	0.10	1.46	3.35	0.05
E2	GY	4B	AX.89574404	1.10E+06	0.05	-4.27	3.01	0.04
E2	GY	4B	AX.89330007	6.58E+08	0.08	-3.48	3.16	0.04
E2	SPM2	4B	AX.89618624	6.53E+08	0.08	24.35	3.92	0.07
E2	GY	4B	AX.89541555	1.31E+07	0.05	-6.74	6.24	0.12
E2	GY	4B	AX.89552019	1.22E+07	0.07	-3.78	3.19	0.04
E2	GY	4B	AX.89486157	6.39E+08	0.08	-4.25	4.36	0.09
E2	GY	4B	AX.89486157	6.39E+08	0.08	-4.25	4.36	0.09
E2	SPM2	4B	AX.89624995	6.13E+08	0.48	-12.21	3.30	0.06
E2	GPM2	4B	AX.89338045	1.26E+06	0.31	-562.81	3.46	0.05
E2	GPM2	4B	AX.89342325	1.25E+06	0.36	-645.24	4.66	0.08
E2	SPM2	4B	AX.89398014	1.12E+06	0.10	-20.07	3.28	0.05
E2	SPM2	4B	AX.89544391	6.43E+08	0.25	13.70	3.15	0.05
E2	GY	4B	AX.89594641	1.24E+07	0.07	-3.90	3.18	0.04
E2	SPM2	4B	AX.89730025	6.43E+08	0.26	13.86	3.25	0.06
E2	SPM2	4B	AX.89441361	6.43E+08	0.29	13.36	3.28	0.06
E2	SPM2	4B	AX.89480256	6.37E+08	0.10	23.25	4.23	0.07
E2	GY	4B	AX.89602483	6.40E+08	0.17	-2.90	3.85	0.07
E2	GPS	4B	AX.89648012	6.43E+08	0.29	-1.63	3.72	0.05
E2	GPM2	4B	AX.89555854	1.11E+06	0.35	-638.02	4.56	0.08
E2	GY	4B	AX.89550402	6.40E+08	0.17	-2.90	3.85	0.07
E2	GY	4B	AX.89550402	6.40E+08	0.17	-2.90	3.85	0.07
E2	GPS	4B	AX.89570204	6.43E+08	0.29	-1.63	3.72	0.05
E2	GY	4B	AX.89400118	1.23E+07	0.07	-3.90	3.18	0.04
E2	GY	4B	AX.89400118	1.23E+07	0.07	-3.90	3.18	0.04
E2	GPM2	4B	AX.89470538	1.09E+06	0.18	-763.15	4.20	0.08
E2	GPM2	4B	AX.89704777	1.32E+06	0.07	-1085.62	3.58	0.06
E2	GPS	4B	AX.89417461	6.43E+08	0.29	-1.63	3.72	0.05
E2	GPS	4B	AX.89743600	6.43E+08	0.29	-1.63	3.72	0.05
E2	GPS	4B	AX.89730025	6.43E+08	0.25	-1.52	3.07	0.04
E2	GY	4B	AX.89518627	1.26E+07	0.17	-2.49	3.01	0.05
E2	GY	4B	AX.89518627	1.26E+07	0.17	-2.49	3.01	0.05
E2	GY	4B	AX.89365524	6.41E+08	0.17	-2.90	3.85	0.07
E2	GY	4B	AX.89365524	6.41E+08	0.17	-2.90	3.85	0.07
E2	GPS	4B	AX.89411480	6.43E+08	0.30	-1.63	3.76	0.06
E2	GPS	4B	AX.89607271	1.25E+07	0.06	-2.77	3.08	0.06
E2	GY	4B	AX.89378503	1.23E+07	0.07	-3.90	3.18	0.04

E2	GY	4B	AX.89378503	1.23E+07	0.07	-3.90	3.18	0.04
E2	GPS	4B	AX.89447880	6.43E+08	0.29	-1.63	3.72	0.05
E2	GPM2	4B	AX.89536017	1.10E+06	0.09	-1000.28	4.18	0.07
E2	GPS	4B	AX.89700585	6.43E+08	0.29	-1.63	3.72	0.05
E2	GY	4B	AX.89591455	1.10E+06	0.05	-4.27	3.01	0.04
E2	GY	4B	AX.89591455	1.10E+06	0.05	-4.27	3.01	0.04
E2	GPS	4B	AX.89592689	6.43E+08	0.29	-1.63	3.72	0.05
E2	GY	4B	AX.89522924	1.23E+07	0.07	-3.90	3.18	0.04
E2	GY	4B	AX.89522924	1.23E+07	0.07	-3.90	3.18	0.04
E2	GPM2	4B	AX.89424041	1.26E+06	0.36	-645.24	4.66	0.08
E2	GPM2	4B	AX.89556208	1.26E+07	0.05	-1147.16	3.32	0.07
E2	GY	4B	AX.89384581	1.31E+07	0.07	-5.60	6.33	0.13
E2	GY	4B	AX.89384581	1.31E+07	0.07	-5.60	6.33	0.13
E2	GY	4B	AX.89316572	1.33E+07	0.05	-4.25	3.01	0.04
E2	GY	4B	AX.89316572	1.33E+07	0.05	-4.25	3.01	0.04
E2	GY	4B	AX.89717411	1.28E+07	0.07	-5.24	5.59	0.11
E2	GY	4B	AX.89717411	1.28E+07	0.07	-5.24	5.59	0.11
E2	GY	4B	AX.89503748	6.40E+08	0.17	-2.90	3.85	0.07
E2	GY	4B	AX.89732714	1.23E+07	0.07	-3.90	3.18	0.04
E2	GY	4B	AX.89732714	1.23E+07	0.07	-3.90	3.18	0.04
E2	GPM2	4B	AX.89318677	1.27E+06	0.36	-645.24	4.66	0.08
E2	GPM2	4B	AX.89393751	1.40E+07	0.12	-746.66	3.01	0.04
E2	GY	4B	AX.89387392	1.22E+07	0.07	-3.78	3.19	0.04
E2	GY	4B	AX.89387392	1.22E+07	0.07	-3.78	3.19	0.04
E2	TKW	4B	AX.89508279	1.32E+06	0.36	0.99	3.36	0.06
E2	GY	4B	AX.89704777	1.32E+06	0.07	-4.04	3.28	0.05
E2	GY	4B	AX.89704777	1.32E+06	0.07	-4.04	3.28	0.05
E2	GPM2	4B	AX.89414530	1.25E+06	0.31	-562.81	3.46	0.05
E2	TKW	4B	AX.89472953	1.09E+06	0.13	1.56	3.93	0.07
E2	GPM2	4B	AX.89751732	1.07E+06	0.35	-655.75	4.75	0.08
E2	GY	4B	AX.89650100	1.31E+07	0.07	-5.00	5.45	0.11
E2	GY	4B	AX.89650100	1.31E+07	0.07	-5.00	5.45	0.11
E2	GY	4B	AX.89426986	6.58E+08	0.09	-3.42	3.20	0.04
E2	GY	4B	AX.89426986	6.58E+08	0.09	-3.42	3.20	0.04
E2	TKW	4B	AX.89516841	1.06E+06	0.13	1.56	3.93	0.07
E2	GPM2	4B	AX.89648033	1.27E+06	0.09	-932.04	3.56	0.06
E2	GY	4B	AX.89563677	1.31E+07	0.07	-3.96	3.62	0.07
E2	GY	4B	AX.89563677	1.31E+07	0.07	-3.96	3.62	0.07
E2	GY	4B	AX.89604837	1.25E+07	0.07	-3.90	3.18	0.04
E2	GY	4B	AX.89604837	1.25E+07	0.07	-3.90	3.18	0.04
E2	TKW	4B	AX.89558314	5.59E+05	0.32	0.97	3.13	0.05
E2	GY	4B	AX.89607271	1.25E+07	0.06	-6.07	6.49	0.13
E2	GY	4B	AX.89607271	1.25E+07	0.06	-6.07	6.49	0.13
E2	GPM2	4B	AX.89442816	1.18E+06	0.31	-562.81	3.46	0.05
E2	GPM2	4B	AX.89348247	1.27E+06	0.36	-645.24	4.66	0.08
E2	TKW	4B	AX.89603812	1.06E+06	0.08	1.90	3.67	0.06
E2	GY	4B	AX.89492174	1.26E+07	0.05	-6.55	5.90	0.12
E2	GY	4B	AX.89492174	1.26E+07	0.05	-6.55	5.90	0.12
E2	GY	4B	AX.89503748	6.40E+08	0.17	-2.90	3.85	0.07
E2	TKW	4B	AX.89470538	1.09E+06	0.18	1.41	4.22	0.08

E2	GY	4B	AX.89574404	1.10E+06	0.05	-4.27	3.01	0.04
E2	GY	4B	AX.89330007	6.58E+08	0.08	-3.48	3.16	0.04
E2	GPS	4B	AX.89702761	6.43E+08	0.29	-1.63	3.72	0.05
E2	GY	4B	AX.89541555	1.31E+07	0.05	-6.74	6.24	0.12
E2	GPM2	4B	AX.89525362	1.20E+06	0.10	-908.75	3.68	0.06
E2	GY	4B	AX.89552019	1.22E+07	0.07	-3.78	3.19	0.04
E2	GPM2	4B	AX.89511155	1.05E+06	0.18	-763.15	4.20	0.08
E2	GPM2	4B	AX.89574404	1.10E+06	0.05	-1169.16	3.39	0.05
E2	GPM2	4B	AX.89340924	1.22E+06	0.10	-908.75	3.68	0.06
E2	TKW	4B	AX.89420815	5.61E+05	0.32	0.97	3.13	0.05
E2	GY	4B	AX.89590964	1.25E+06	0.12	-3.26	3.58	0.05
E2	GY	4B	AX.89590964	1.25E+06	0.12	-3.26	3.58	0.05
E2	GPM2	4B	AX.89736803	1.26E+06	0.31	-562.81	3.46	0.05
E2	GPM2	4B	AX.89590964	1.25E+06	0.11	-1035.84	5.10	0.09
E2	GY	4B	AX.89438962	1.31E+07	0.13	-3.86	5.13	0.10
E2	GY	4B	AX.89438962	1.31E+07	0.13	-3.86	5.13	0.10
E2	GPS	4B	AX.89407510	6.43E+08	0.30	-1.49	3.24	0.05
E2	GY	4B	AX.89766498	1.23E+07	0.07	-3.90	3.18	0.04
E2	GY	4B	AX.89766498	1.23E+07	0.07	-3.90	3.18	0.04
E2	GPS	4B	AX.89489650	6.43E+08	0.29	-1.63	3.72	0.05
E2	GPS	4B	AX.89373069	6.44E+08	0.32	-1.48	3.31	0.05
E2	GPM2	4B	AX.89477576	1.12E+06	0.06	-1254.49	4.10	0.07
E2	GPM2	4B	AX.89670164	1.26E+06	0.36	-645.24	4.66	0.08
E2	GPM2	4B	AX.89607271	1.25E+07	0.06	-1148.11	3.82	0.08
E2	GPM2	4B	AX.89710094	1.09E+06	0.35	-627.33	4.40	0.07
E2	GPS	4B	AX.89624860	6.43E+08	0.29	-1.63	3.72	0.05
E2	GPS	4B	AX.89353743	6.43E+08	0.32	-1.40	3.02	0.04
E2	GY	4B	AX.89751657	6.41E+08	0.17	-2.90	3.85	0.07
E2	GY	4B	AX.89751657	6.41E+08	0.17	-2.90	3.85	0.07
E2	TKW	4B	AX.89598155	1.09E+06	0.13	1.56	3.93	0.07
E2	GY	4B	AX.89594641	1.24E+07	0.07	-3.90	3.18	0.04
E2	GPM2	4B	AX.89354656	1.20E+06	0.10	-908.75	3.68	0.06
E2	GPS	4B	AX.89312097	6.43E+08	0.30	-1.49	3.24	0.05
E2	GPS	4B	AX.89477202	6.43E+08	0.30	-1.49	3.24	0.05
E2	GPM2	4B	AX.89777442	1.06E+06	0.35	-655.75	4.75	0.08
E2	TKW	4B	AX.89356251	6.73E+08	0.38	-0.95	3.19	0.05
E2	GPM2	4B	AX.89482262	1.25E+06	0.31	-562.81	3.46	0.05
E2	GPM2	4B	AX.89704154	1.06E+06	0.35	-655.75	4.75	0.08
E2	GY	4B	AX.89372215	6.41E+08	0.17	-2.90	3.85	0.07
E2	GY	4B	AX.89372215	6.41E+08	0.17	-2.90	3.85	0.07
E2	GY	4B	AX.89477576	1.12E+06	0.06	-4.30	3.26	0.05
E2	GY	4B	AX.89477576	1.12E+06	0.06	-4.30	3.26	0.05
E2	GPS	4B	AX.89649782	6.43E+08	0.29	-1.63	3.72	0.05
E2	GPS	4B	AX.89328165	6.43E+08	0.29	-1.63	3.72	0.05
E2	GPS	4B	AX.89689786	6.43E+08	0.29	-1.63	3.72	0.05
E2	GPM2	4B	AX.89311841	1.09E+06	0.20	-770.05	4.68	0.09
E2	TKW	4B	AX.89545685	1.25E+06	0.36	0.99	3.36	0.06
E2	GPS	4B	AX.89420985	6.43E+08	0.29	-1.63	3.72	0.05
E2	GPS	4B	AX.89577670	6.43E+08	0.28	-1.63	3.64	0.05
E2	GY	4B	AX.89556208	1.26E+07	0.05	-6.07	5.60	0.11

E2	TKW	4B	AX.89777442	1.06E+06	0.35	0.98	3.30	0.06
E2	GY	4B	AX.89602483	6.40E+08	0.17	-2.90	3.85	0.07
E2	GPS	4B	AX.89607742	6.43E+08	0.29	-1.63	3.72	0.05
E2	GPS	4B	AX.89735718	6.43E+08	0.30	-1.64	3.80	0.06
E2	TKW	4B	AX.89311841	1.09E+06	0.20	1.37	4.34	0.08
E2	GPS	4B	AX.89379339	6.43E+08	0.33	-1.40	3.02	0.04
E2	GPM2	4B	AX.89310301	2.38E+05	0.31	-555.75	3.37	0.05
E2	GPM2	4B	AX.89558638	8.22E+05	0.35	-655.75	4.75	0.08
E2	TKW	4B	AX.89501151	1.08E+06	0.13	1.56	3.93	0.07
E2	GPS	4B	AX.89481614	6.43E+08	0.29	-1.63	3.72	0.05
E2	GPM2	4B	AX.89486157	6.39E+08	0.08	-1000.04	3.78	0.09
E2	GPM2	4B	AX.89398310	1.31E+06	0.09	-932.04	3.56	0.06
E2	GPM2	4B	AX.89510255	3.34E+06	0.06	-1007.91	3.02	0.04
E2	GPM2	4B	AX.89541555	1.31E+07	0.05	-1333.51	3.98	0.09
E2	GPS	4B	AX.89390122	6.43E+08	0.33	-1.40	3.02	0.04
E2	GPS	4B	AX.89313935	6.43E+08	0.33	-1.40	3.02	0.04
E2	GPS	4B	AX.89458144	6.43E+08	0.29	-1.63	3.72	0.05
E2	GY	4B	AX.89514498	6.39E+08	0.08	-4.25	4.36	0.09
E2	GY	4B	AX.89514498	6.39E+08	0.08	-4.25	4.36	0.09
E2	GPS	4B	AX.89717455	6.43E+08	0.30	-1.49	3.24	0.05
E2	GPM2	4B	AX.89624776	8.26E+05	0.20	-770.05	4.68	0.09
E2	TKW	4B	AX.89385922	5.61E+05	0.32	0.97	3.13	0.05
E2	TKW	4B	AX.89704154	1.06E+06	0.35	0.98	3.30	0.06
E2	GPS	4B	AX.89405013	6.14E+08	0.39	1.40	3.22	0.05
E2	TKW	4B	AX.89376768	1.06E+06	0.13	1.56	3.93	0.07
E2	TKW	4B	AX.89550959	2.38E+05	0.35	1.00	3.39	0.06
E2	GPM2	4B	AX.89439714	1.26E+06	0.36	-645.24	4.66	0.08
E2	TKW	4B	AX.89325823	1.32E+06	0.34	1.02	3.44	0.06
E2	GPS	4B	AX.89642726	6.60E+08	0.28	1.59	3.52	0.05
E2	GPM2	4B	AX.89316443	6.54E+08	0.07	-985.39	3.08	0.05
E2	GPS	4B	AX.89729026	6.43E+08	0.29	-1.63	3.72	0.05
E2	TKW	4B	AX.89732228	1.06E+06	0.13	1.56	3.93	0.07
E2	TKW	4B	AX.89700283	1.05E+06	0.15	1.46	3.96	0.07
E2	GPM2	4B	AX.89672636	1.20E+06	0.09	-1000.28	4.18	0.07
E2	TKW	4B	AX.89405164	1.05E+06	0.20	1.37	4.34	0.08
E2	TKW	4B	AX.89342325	1.25E+06	0.36	0.99	3.36	0.06
E2	TKW	4B	AX.89424259	1.06E+06	0.35	0.98	3.30	0.06
E2	GPM2	4B	AX.89452063	1.25E+06	0.36	-645.24	4.66	0.08
E2	GPS	4B	AX.89481722	6.43E+08	0.29	-1.63	3.72	0.05
E2	TKW	4B	AX.89688587	1.06E+06	0.13	1.56	3.93	0.07
E2	TKW	4B	AX.89673766	1.08E+06	0.13	1.56	3.93	0.07
E2	GPM2	4B	AX.89678237	1.26E+06	0.31	-562.81	3.46	0.05
E2	TKW	4B	AX.89651222	5.60E+05	0.32	0.97	3.13	0.05
E2	TKW	4B	AX.89603801	8.23E+05	0.15	1.46	3.96	0.07
E2	GY	4B	AX.89753877	1.26E+07	0.07	-5.00	5.45	0.11
E2	GY	4B	AX.89753877	1.26E+07	0.07	-5.00	5.45	0.11
E2	GY	4B	AX.89746323	6.51E+08	0.13	-2.80	3.13	0.06
E2	GPM2	4B	AX.89679243	1.32E+06	0.34	-588.12	3.85	0.06
E2	TKW	4B	AX.89760754	5.59E+05	0.32	0.97	3.13	0.05
E2	GPM2	4B	AX.89438962	1.31E+07	0.13	-904.69	4.42	0.09

E2	TKW	4B	AX.89326548	8.15E+05	0.10	1.62	3.60	0.06
E2	TKW	4B	AX.89655662	1.08E+06	0.35	0.98	3.30	0.06
E2	TKW	4B	AX.89624776	8.26E+05	0.20	1.37	4.34	0.08
E2	TKW	4B	AX.89500369	1.20E+06	0.36	0.99	3.36	0.06
E2	GPS	4B	AX.89506257	6.43E+08	0.29	-1.63	3.72	0.05
E2	TKW	4B	AX.89623298	5.62E+05	0.32	0.97	3.13	0.05
E2	GY	4B	AX.89356483	6.41E+08	0.17	-2.90	3.85	0.07
E2	GY	4B	AX.89356483	6.41E+08	0.17	-2.90	3.85	0.07
E2	GY	4B	AX.89555692	1.23E+07	0.07	-3.90	3.18	0.04
E2	GY	4B	AX.89555692	1.23E+07	0.07	-3.90	3.18	0.04
E2	TKW	4B	AX.89725766	6.73E+08	0.38	-0.92	3.02	0.05
E2	GPM2	4B	AX.89655662	1.08E+06	0.35	-655.75	4.75	0.08
E2	GPM2	4B	AX.89384581	1.31E+07	0.07	-1031.27	3.56	0.07
E2	GPS	4B	AX.89600316	6.43E+08	0.30	-1.49	3.24	0.05
E2	GPS	4B	AX.89542249	6.43E+08	0.29	-1.63	3.72	0.05
E2	TKW	4B	AX.89577839	5.57E+05	0.32	0.97	3.13	0.05
E2	GPM2	4B	AX.89311787	1.27E+06	0.36	-645.24	4.66	0.08
E2	GPM2	4B	AX.89405164	1.05E+06	0.20	-770.05	4.68	0.09
E2	GPS	4B	AX.89407767	6.43E+08	0.29	-1.63	3.72	0.05
E2	GY	4B	AX.89556208	1.26E+07	0.05	-6.07	5.60	0.11
E2	GPM2	4B	AX.89698426	1.10E+06	0.09	-1000.28	4.18	0.07
E2	GPS	4B	AX.89692924	6.43E+08	0.29	-1.63	3.72	0.05
E2	GY	4B	AX.89530566	1.22E+07	0.07	-3.78	3.19	0.04
E2	GY	4B	AX.89696663	6.51E+08	0.13	-2.86	3.24	0.06
E2	TKW	4B	AX.89531200	5.61E+05	0.32	0.97	3.13	0.05
E2	GPS	4B	AX.89332326	6.43E+08	0.29	-1.63	3.72	0.05
E2	TKW	4B	AX.89777133	1.13E+06	0.36	0.99	3.36	0.06
E2	GPS	4B	AX.89701639	6.43E+08	0.33	-1.40	3.02	0.04
E2	TKW	4B	AX.89438749	5.55E+05	0.33	0.97	3.12	0.05
E2	GPM2	4B	AX.89507664	2.39E+05	0.31	-555.75	3.37	0.05
E2	GY	4B	AX.89746323	6.51E+08	0.13	-2.80	3.13	0.06
E2	GPM2	4B	AX.89340425	1.26E+06	0.36	-645.24	4.66	0.08
E2	GPM2	4B	AX.89753398	1.32E+06	0.36	-645.24	4.66	0.08
E2	GPM2	4B	AX.89481031	1.05E+06	0.35	-655.75	4.75	0.08
E2	GY	4B	AX.89582539	1.26E+06	0.07	-4.04	3.28	0.05
E2	GPM2	4B	AX.89584959	1.32E+06	0.36	-645.24	4.66	0.08
E2	GPM2	4B	AX.89424259	1.06E+06	0.35	-655.75	4.75	0.08
E2	GPS	4B	AX.89661499	6.43E+08	0.29	-1.63	3.72	0.05
E2	GPM2	4B	AX.89777133	1.13E+06	0.36	-645.24	4.66	0.08
E2	TKW	4B	AX.89485400	5.58E+05	0.32	0.97	3.13	0.05
E2	GPS	4B	AX.89700923	6.43E+08	0.29	-1.63	3.72	0.05
E2	GPM2	4B	AX.89582539	1.26E+06	0.07	-1085.62	3.58	0.06
E2	GPM2	4B	AX.89550959	2.38E+05	0.35	-638.02	4.56	0.08
E2	TKW	4B	AX.89586227	1.32E+06	0.36	0.99	3.36	0.06
E2	GPM2	4B	AX.89357689	6.22E+08	0.11	-787.21	3.09	0.05
E2	GY	4B	AX.89611914	6.47E+08	0.23	-2.60	3.95	0.07
E2	GPS	4B	AX.89403094	6.43E+08	0.32	-1.40	3.02	0.04
E2	GPS	4B	AX.89535815	6.43E+08	0.29	-1.63	3.72	0.05
E2	GPM2	4B	AX.89508279	1.32E+06	0.36	-645.24	4.66	0.08
E2	TKW	4B	AX.89532492	5.61E+05	0.32	0.97	3.13	0.05

E2	GPS	4B	AX.89441361	6.43E+08	0.29	-1.49	3.18	0.05
E2	GPM2	4B	AX.89754574	1.12E+06	0.35	-608.47	4.19	0.07
E2	GY	4B	AX.89582539	1.26E+06	0.07	-4.04	3.28	0.05
E2	TKW	4B	AX.89753398	1.32E+06	0.36	0.99	3.36	0.06
E2	GPM2	4B	AX.89669729	1.20E+06	0.09	-1000.28	4.18	0.07
E2	GY	4B	AX.89696663	6.51E+08	0.13	-2.86	3.24	0.06
E2	TKW	4B	AX.89316017	1.05E+06	0.15	1.32	3.39	0.06
E2	GPM2	4B	AX.89678902	1.20E+06	0.31	-562.81	3.46	0.05
E2	GPS	4B	AX.89520936	6.43E+08	0.29	-1.63	3.72	0.05
E2	GPM2	4B	AX.89591455	1.10E+06	0.05	-1169.16	3.39	0.05
E2	GPM2	4B	AX.89711166	1.13E+06	0.35	-557.87	3.57	0.06
E2	TKW	4B	AX.89558638	8.22E+05	0.35	0.98	3.30	0.06
E2	GPS	4B	AX.89411200	6.43E+08	0.29	-1.63	3.72	0.05
E2	GPS	4B	AX.89379401	6.43E+08	0.30	-1.59	3.61	0.05
E2	GPM2	4B	AX.89317719	1.24E+06	0.09	-1000.28	4.18	0.07
E2	GPS	4B	AX.89506443	6.43E+08	0.29	-1.63	3.72	0.05
E2	GPS	4B	AX.89684241	6.43E+08	0.29	-1.63	3.72	0.05
E2	TKW	4B	AX.89608391	1.05E+06	0.18	1.41	4.22	0.08
E2	GPM2	4B	AX.89433236	1.27E+06	0.36	-645.24	4.66	0.08
E2	GPM2	4B	AX.89767218	1.26E+06	0.36	-645.24	4.66	0.08
E2	GPM2	4B	AX.89500369	1.20E+06	0.36	-645.24	4.66	0.08
E2	TKW	4B	AX.89330961	1.05E+06	0.13	1.56	3.93	0.07
E2	GPS	4B	AX.89644812	6.43E+08	0.30	-1.49	3.24	0.05
E2	TKW	4B	AX.89715931	5.62E+05	0.32	0.97	3.13	0.05
E2	GPM2	4B	AX.89722528	1.10E+06	0.07	-1101.78	3.91	0.07
E2	TKW	4B	AX.89481031	1.05E+06	0.35	0.98	3.30	0.06
E2	GPS	4B	AX.89720444	6.43E+08	0.29	-1.63	3.72	0.05
E2	TKW	4B	AX.89767218	1.26E+06	0.36	0.99	3.36	0.06
E2	GPS	4B	AX.89530747	6.43E+08	0.29	-1.63	3.72	0.05
E2	GPS	4B	AX.89386120	6.43E+08	0.29	-1.63	3.72	0.05
E2	GPM2	4B	AX.89546587	1.10E+06	0.09	-1000.28	4.18	0.07
E2	GPM2	4B	AX.89376620	1.13E+06	0.31	-527.34	3.12	0.04
E2	GPM2	4B	AX.89711023	2.38E+05	0.31	-555.75	3.37	0.05
E2	TKW	4B	AX.89511155	1.05E+06	0.18	1.41	4.22	0.08
E2	TKW	4B	AX.89751732	1.07E+06	0.35	0.98	3.30	0.06
E2	TKW	4B	AX.89584959	1.32E+06	0.36	0.99	3.36	0.06
E2	TKW	4B	AX.89679243	1.32E+06	0.34	1.02	3.44	0.06
E2	GPM2	4B	AX.89514498	6.39E+08	0.08	-1000.04	3.78	0.09
E2	GPM2	4B	AX.89387141	1.21E+06	0.09	-1000.28	4.18	0.07
E2	GPS	4B	AX.89594400	6.43E+08	0.29	-1.63	3.72	0.05
E2	GPM2	4B	AX.89385325	1.22E+06	0.09	-1000.28	4.18	0.07
E2	TKW	4B	AX.89452063	1.25E+06	0.36	0.99	3.36	0.06
E2	GY	4B	AX.89530566	1.22E+07	0.07	-3.78	3.19	0.04
E2	GPM2	4B	AX.89676110	1.26E+06	0.31	-562.81	3.46	0.05
E2	GPS	4B	AX.89697958	6.43E+08	0.29	-1.63	3.72	0.05
E2	GPM2	4B	AX.89325823	1.32E+06	0.34	-588.12	3.85	0.06
E2	GY	4B	AX.89611914	6.47E+08	0.23	-2.60	3.95	0.07
E2	TKW	4B	AX.89313669	5.60E+05	0.32	0.97	3.13	0.05
E2	GPM2	4B	AX.89330007	6.58E+08	0.08	-876.79	3.09	0.04
E2	TKW	4B	AX.89490058	5.60E+05	0.32	0.97	3.13	0.05

E2	GPS	4B	AX.89438962	1.31E+07	0.13	-2.14	3.48	0.07
E2	TKW	4B	AX.89405169	5.61E+05	0.32	0.97	3.13	0.05
E2	TKW	4B	AX.89555854	1.11E+06	0.35	1.00	3.39	0.06
E2	TKW	4B	AX.89450204	1.27E+06	0.36	0.99	3.36	0.06
E2	GPM2	4B	AX.89693470	1.25E+06	0.31	-562.81	3.46	0.05
E2	TKW	4B	AX.89348247	1.27E+06	0.36	0.99	3.36	0.06
E2	GPM2	4B	AX.89450204	1.27E+06	0.36	-645.24	4.66	0.08
E2	GPS	4B	AX.89311940	6.43E+08	0.29	-1.63	3.72	0.05
E2	GPS	4B	AX.89604454	6.43E+08	0.29	-1.63	3.72	0.05
E2	GPM2	4B	AX.89545685	1.25E+06	0.36	-645.24	4.66	0.08
E2	TKW	4B	AX.89340425	1.26E+06	0.36	0.99	3.36	0.06
E2	TKW	4B	AX.89670164	1.26E+06	0.36	0.99	3.36	0.06
E2	GPS	4B	AX.89728605	6.43E+08	0.29	-1.63	3.72	0.05
E2	TKW	4B	AX.89318677	1.27E+06	0.36	0.99	3.36	0.06
E2	GPS	4B	AX.89430679	6.43E+08	0.29	-1.63	3.72	0.05
E2	TKW	4B	AX.89650899	5.61E+05	0.32	0.97	3.13	0.05
E2	TKW	4B	AX.89407895	5.60E+05	0.32	0.97	3.13	0.05
E2	TKW	4B	AX.89582667	1.06E+06	0.15	1.34	3.48	0.06
E2	TKW	4B	AX.89311787	1.27E+06	0.36	0.99	3.36	0.06
E2	TKW	4B	AX.89439714	1.26E+06	0.36	0.99	3.36	0.06
E2	GPM2	4B	AX.89608391	1.05E+06	0.18	-763.15	4.20	0.08
E2	GPS	4B	AX.89705325	6.43E+08	0.29	-1.63	3.72	0.05
E2	TKW	4B	AX.89424041	1.26E+06	0.36	0.99	3.36	0.06
E2	TKW	4B	AX.89500414	5.60E+05	0.32	0.97	3.13	0.05
E2	GPM2	4B	AX.89482708	2.37E+05	0.31	-555.75	3.37	0.05
E2	GPM2	4B	AX.89492174	1.26E+07	0.05	-1173.39	3.19	0.07
E2	GPM2	4B	AX.89586227	1.32E+06	0.36	-645.24	4.66	0.08
E2	GPM2	4B	AX.89764813	2.39E+08	0.08	-880.19	3.04	0.04
E2	TKW	4B	AX.89433236	1.27E+06	0.36	0.99	3.36	0.06
E2	GPS	4B	AX.89507497	6.43E+08	0.29	-1.63	3.72	0.05
E3	GPM2	4B	AX.89581777	3.80E+07	0.39	948.31	3.41	0.00
E3	GPM2	4B	AX.89574801	3.80E+07	0.40	996.01	3.79	0.00
E3	GPM2	4B	AX.89331938	3.80E+07	0.40	1023.29	3.93	0.00
E3	GPM2	4B	AX.89319283	3.80E+07	0.40	905.99	3.19	0.00
E3	GPM2	4B	AX.89462446	3.80E+07	0.39	958.97	3.51	0.00
E3	GPM2	4B	AX.89440374	3.80E+07	0.39	925.01	3.30	0.00
E3	GPM2	4B	AX.89716019	3.80E+07	0.39	958.97	3.51	0.00
E3	GPM2	4B	AX.89484647	3.82E+07	0.39	958.97	3.51	0.00
E3	GPM2	4B	AX.89313304	3.83E+07	0.40	923.87	3.31	0.00
E3	GPM2	4B	AX.89593486	3.83E+07	0.40	1023.29	3.93	0.00
E3	GPM2	4B	AX.89607364	3.83E+07	0.40	1023.29	3.93	0.00
E3	GPM2	4B	AX.89645483	1.07E+07	0.33	-949.88	3.13	0.06
E3	GPM2	4B	AX.89638915	1.07E+07	0.32	-1013.42	3.30	0.07
E3	GPM2	4B	AX.89609811	1.08E+07	0.32	-1004.06	3.20	0.07
E3	GPM2	4B	AX.89654556	1.08E+07	0.33	-1018.46	3.29	0.07
E3	GPM2	4B	AX.89575125	1.08E+07	0.33	-1018.46	3.29	0.07
E3	GPM2	4B	AX.89691011	3.66E+07	0.42	867.11	3.12	0.00
E3	GPM2	4B	AX.89544790	3.70E+07	0.42	855.35	3.07	0.00
E3	GPM2	4B	AX.89771160	3.70E+07	0.42	888.30	3.26	0.00
E3	GPM2	4B	AX.89403459	3.71E+07	0.42	888.30	3.26	0.00

E3	GPM2	4B	AX.89611058	3.71E+07	0.42	888.30	3.26	0.00
E3	GPM2	4B	AX.89740017	3.71E+07	0.42	886.91	3.25	0.00
E3	GPM2	4B	AX.89447599	3.71E+07	0.42	899.36	3.31	0.00
E3	GPM2	4B	AX.89560106	3.75E+07	0.39	956.90	3.52	0.00
E3	GPM2	4B	AX.89508296	3.77E+07	0.50	949.45	3.37	0.00
E3	GPM2	4B	AX.89349947	3.78E+07	0.49	1009.30	3.74	0.00
E3	GPM2	4B	AX.89313597	3.78E+07	0.49	1009.30	3.74	0.00
E3	GPM2	4B	AX.89412368	3.78E+07	0.49	1009.30	3.74	0.00
E3	GPM2	4B	AX.89459443	3.78E+07	0.50	937.47	3.27	0.00
E3	GPM2	4B	AX.89511248	3.78E+07	0.38	991.01	3.70	0.00
E3	GPM2	4B	AX.89707434	3.79E+07	0.49	971.45	3.48	0.00
E3	GPM2	4B	AX.89505881	3.79E+07	0.50	937.47	3.27	0.00
E3	GPM2	4B	AX.89766617	3.79E+07	0.39	958.97	3.51	0.00
E3	GPM2	4B	AX.89391627	3.79E+07	0.50	937.47	3.27	0.00
E3	GPM2	4B	AX.89552410	3.79E+07	0.39	958.97	3.51	0.00
E3	GPM2	4B	AX.89526525	3.79E+07	0.50	937.47	3.27	0.00
E3	GPM2	4B	AX.89669200	3.79E+07	0.39	958.97	3.51	0.00
E3	GPM2	4B	AX.89410142	3.79E+07	0.39	958.97	3.51	0.00
E3	GPM2	4B	AX.89486339	3.79E+07	0.40	928.69	3.35	0.00
E3	GPM2	4B	AX.89698371	3.79E+07	0.39	958.97	3.51	0.00
E3	GPM2	4B	AX.89495103	3.79E+07	0.39	958.97	3.51	0.00
E3	GPM2	4B	AX.89627885	3.79E+07	0.39	880.75	3.07	0.00
E3	GPM2	4B	AX.89478813	3.79E+07	0.39	958.97	3.51	0.00
E3	GPM2	4B	AX.89546302	3.79E+07	0.39	958.97	3.51	0.00
E3	GPM2	4B	AX.89542473	3.79E+07	0.40	909.03	3.20	0.00
E3	GPM2	4B	AX.89400517	3.79E+07	0.39	895.77	3.14	0.00
E3	GPM2	4B	AX.89425228	3.80E+07	0.39	958.97	3.51	0.00
E3	GPM2	4B	AX.89378460	3.80E+07	0.41	894.95	3.17	0.00
E3	GPM2	4B	AX.89506349	3.80E+07	0.38	900.28	3.20	0.00
E3	GPM2	4B	AX.89314466	3.80E+07	0.40	928.69	3.35	0.00
E3	GPM2	4B	AX.89689545	3.80E+07	0.40	928.69	3.35	0.00
E3	GPM2	4B	AX.89656295	3.80E+07	0.40	970.77	3.60	0.00
E3	GPM2	4B	AX.89599240	3.80E+07	0.40	928.69	3.35	0.00
E3	GPM2	4B	AX.89677554	3.80E+07	0.40	923.87	3.31	0.00
E3	GPM2	4B	AX.89356176	3.80E+07	0.40	928.69	3.35	0.00
E3	GPM2	4B	AX.89504489	3.80E+07	0.39	998.36	3.79	0.00
E3	GPM2	4B	AX.89706774	3.83E+07	0.40	928.69	3.35	0.00
E3	GPM2	4B	AX.89768844	3.83E+07	0.41	944.37	3.49	0.00
E3	GPM2	4B	AX.89677774	3.83E+07	0.40	987.72	3.74	0.00
E3	GPM2	4B	AX.89709952	3.83E+07	0.40	922.80	3.33	0.00
E3	GPM2	4B	AX.89686862	3.83E+07	0.40	966.17	3.60	0.00
E3	GPM2	4B	AX.89571155	3.83E+07	0.41	950.36	3.50	0.00
E3	GPM2	4B	AX.89601831	3.84E+07	0.41	900.57	3.20	0.00
E3	GPM2	4B	AX.89668547	3.84E+07	0.40	914.96	3.24	0.00
E3	GPM2	4B	AX.89590856	3.84E+07	0.41	892.10	3.16	0.00
E3	GPM2	4B	AX.89577229	3.85E+07	0.41	1042.48	4.04	0.00
E3	GPM2	4B	AX.89324506	3.86E+07	0.41	915.37	3.30	0.00
E3	GPM2	4B	AX.89472850	3.86E+07	0.41	870.96	3.01	0.00
E3	GPM2	4B	AX.89677501	3.86E+07	0.40	892.06	3.14	0.00
E3	GPM2	4B	AX.89333449	3.86E+07	0.38	914.61	3.29	0.00

E3	GPM2	4B	AX.89460204	3.86E+07	0.41	935.67	3.40	0.00
E3	GPM2	4B	AX.89382467	3.86E+07	0.40	928.68	3.35	0.00
E3	GPM2	4B	AX.89614135	3.86E+07	0.41	950.36	3.50	0.00
E3	GPM2	4B	AX.89344066	3.86E+07	0.41	950.36	3.50	0.00
E3	GPM2	4B	AX.89603525	3.86E+07	0.41	950.36	3.50	0.00
E3	GPM2	4B	AX.89762834	3.87E+07	0.41	885.68	3.11	0.00
E3	GPM2	4B	AX.89683291	3.87E+07	0.41	885.68	3.11	0.00
E3	GPM2	4B	AX.89328964	3.87E+07	0.41	885.68	3.11	0.00
E3	GPM2	4B	AX.89741879	3.88E+07	0.42	898.22	3.22	0.00
E3	GPM2	4B	AX.89702921	3.88E+07	0.42	864.37	3.03	0.00
E3	GPM2	4B	AX.89680320	6.60E+08	0.34	1010.11	3.32	0.10
E3	GPS	4B	AX.89343363	2.96E+07	0.30	-2.38	3.79	0.02
E3	GPS	4B	AX.89382876	3.06E+07	0.28	-2.41	3.69	0.01
E3	GY	4B	AX.89645483	1.07E+07	0.33	-3.51	3.75	0.05
E3	GY	4B	AX.89638915	1.07E+07	0.32	-3.46	3.46	0.04
E3	GY	4B	AX.89609811	1.08E+07	0.32	-3.57	3.59	0.04
E3	GY	4B	AX.89654556	1.08E+07	0.33	-3.52	3.52	0.04
E3	GY	4B	AX.89575125	1.08E+07	0.33	-3.52	3.52	0.04
E3	TKW	4B	AX.89743794	1.67E+07	0.25	-1.05	3.42	0.05
E3	TKW	4B	AX.89563300	1.67E+07	0.25	-1.05	3.42	0.05
E3	TKW	4B	AX.89363633	1.68E+07	0.24	-1.03	3.33	0.05
E3	TKW	4B	AX.89752007	2.40E+07	0.09	-1.44	3.01	0.02
E3	TKW	4B	AX.89578058	2.40E+07	0.10	-1.43	3.04	0.01
E3	TKW	4B	AX.89678773	6.44E+08	0.30	0.93	3.01	0.11
E3	TKW	4B	AX.89613001	6.44E+08	0.30	0.96	3.19	0.11
E3	TKW	4B	AX.89360797	6.44E+08	0.31	0.96	3.24	0.09
E3	TKW	4B	AX.89439656	6.44E+08	0.31	0.99	3.38	0.10
E3	TKW	4B	AX.89520658	6.44E+08	0.31	0.96	3.24	0.09
E3	TKW	4B	AX.89722219	6.44E+08	0.32	1.06	3.87	0.11
E3	TKW	4B	X.89621181_OT	6.45E+08	0.26	1.05	3.58	0.11
E3	TKW	4B	X.89773162_OT	6.45E+08	0.26	1.01	3.29	0.11
E3	TKW	4B	AX.89639340	6.45E+08	0.31	0.96	3.24	0.09
E3	TKW	4B	X.89718419_OT	6.45E+08	0.26	0.96	3.01	0.11
E3	TKW	4B	AX.89568959	6.45E+08	0.31	0.96	3.24	0.09
E3	TKW	4B	X.89732621_OT	6.45E+08	0.26	1.07	3.66	0.11
E3	TKW	4B	AX.89664022	6.45E+08	0.30	0.93	3.07	0.10
E3	TKW	4B	AX.89728470	6.45E+08	0.27	1.05	3.50	0.12
E3	TKW	4B	AX.89710722	6.45E+08	0.30	0.94	3.20	0.10
E3	TKW	4B	AX.89642563	6.45E+08	0.30	0.91	3.01	0.10
E3	TKW	4B	AX.89524337	6.54E+08	0.32	0.88	3.01	0.08
E3	TKW	4B	AX.89415046	6.54E+08	0.32	0.88	3.01	0.08
E3	TKW	4B	AX.89327826	6.54E+08	0.32	0.88	3.01	0.08
E3	TKW	4B	AX.89636167	6.54E+08	0.32	0.88	3.01	0.08
E3	TKW	4B	AX.89459222	6.54E+08	0.32	0.88	3.01	0.08
E3	TKW	4B	AX.89390456	6.54E+08	0.32	0.88	3.01	0.08
E3	TKW	4B	AX.89701603	6.54E+08	0.32	0.88	3.01	0.08
E3	TKW	4B	AX.89651761	6.54E+08	0.32	0.88	3.01	0.08
E3	TKW	4B	AX.89448796	6.54E+08	0.32	0.88	3.01	0.08
E3	TKW	4B	AX.89546654	6.61E+08	0.26	1.03	3.57	0.15
E3	TKW	4B	AX.89428631	6.61E+08	0.26	1.04	3.62	0.15

E3	TKW	4B	AX.89681705	6.61E+08	0.26	1.03	3.57	0.15
E3	TKW	4B	AX.89386305	6.61E+08	0.26	1.08	3.82	0.16
E3	TKW	4B	AX.89383901	6.61E+08	0.26	1.08	3.82	0.16
E3	TKW	4B	AX.89642301	6.61E+08	0.26	1.08	3.82	0.16
E3	TKW	4B	AX.89764222	6.61E+08	0.26	1.08	3.82	0.16
E3	TKW	4B	AX.89563454	6.61E+08	0.26	1.08	3.82	0.16
E3	SPM2	4B	AX.89731825	1.40E+07	0.33	20.07	3.17	0.05
E3	TKW	4B	AX.89527670	6.61E+08	0.47	-0.85	3.24	0.05
E3	TKW	4B	AX.89664514	6.61E+08	0.28	-0.92	3.14	0.01
E3	TKW	4B	AX.89664380	6.61E+08	0.26	1.08	3.82	0.16
E3	TKW	4B	AX.89605438	6.61E+08	0.26	1.08	3.82	0.16
E3	TKW	4B	AX.89461756	6.61E+08	0.26	0.96	3.19	0.16
E3	TKW	4B	AX.89482856	6.61E+08	0.26	1.08	3.82	0.16
E3	TKW	4B	AX.89726222	6.61E+08	0.26	1.08	3.82	0.16
E3	TKW	4B	AX.89722985	6.61E+08	0.26	1.03	3.51	0.15
E3	TKW	4B	AX.89534747	6.61E+08	0.26	1.08	3.82	0.16
E3	TKW	4B	AX.89575759	6.61E+08	0.26	1.08	3.82	0.16
E3	TKW	4B	AX.89447537	6.61E+08	0.47	-0.88	3.35	0.06
E4	GPS	4B	AX.89731957	6.69E+08	0.17	-1.93	3.01	0.07
E4	TKW	4B	AX.89743794	1.67E+07	0.25	-0.97	3.06	0.03
E4	TKW	4B	AX.89563300	1.67E+07	0.25	-0.97	3.06	0.03
E4	TKW	4B	AX.89309886	1.68E+07	0.29	-0.90	3.03	0.05
E1	SPM2	4D	AX.89666096	2.48E+07	0.44	17.17	3.28	0.05
E1	GY	4D	AX.89484971	3.49E+08	0.09	-4.28	3.39	0.05
E1	SPM2	4D	AX.89395719	6.72E+07	0.14	26.69	3.77	0.07
E1	SPM2	4D	AX.89582063	1.13E+08	0.38	17.73	3.34	0.06
E1	SPM2	4D	AX.89577388	3.54E+08	0.28	18.17	3.08	0.05
E1	GY	4D	AX.89310874	3.40E+08	0.09	-4.28	3.39	0.05
E1	SPM2	4D	AX.89523903	2.06E+07	0.34	18.67	3.51	0.06
E1	SPM2	4D	AX.89474282	1.12E+08	0.39	17.11	3.15	0.06
E1	GY	4D	AX.89714193	1.08E+08	0.38	2.41	3.16	0.06
E1	SPM2	4D	AX.89648476	6.90E+07	0.11	31.71	4.31	0.08
E1	SPM2	4D	AX.89348065	2.17E+07	0.33	17.67	3.11	0.05
E1	SPM2	4D	AX.89705815	2.54E+07	0.44	16.40	3.04	0.05
E1	SPM2	4D	AX.89526164	2.05E+07	0.33	17.67	3.11	0.05
E1	GY	4D	AX.89363319	3.46E+08	0.09	-4.28	3.39	0.05
E1	GY	4D	AX.89363319	3.46E+08	0.09	-4.28	3.39	0.05
E1	SPM2	4D	AX.89695623	6.72E+07	0.14	26.69	3.77	0.07
E1	SPM2	4D	AX.89319213	2.01E+07	0.31	20.09	3.78	0.06
E1	GY	4D	AX.89484971	3.49E+08	0.09	-4.28	3.39	0.05
E1	GY	4D	AX.89310874	3.40E+08	0.09	-4.28	3.39	0.05
E1	GY	4D	AX.89454347	2.38E+08	0.07	-4.86	3.51	0.07
E1	SPM2	4D	AX.89317994	8.95E+07	0.35	17.27	3.09	0.05
E1	GY	4D	AX.89568572	1.08E+08	0.38	2.41	3.16	0.06
E1	GY	4D	AX.89348065	2.17E+07	0.33	2.74	3.72	0.06
E1	SPM2	4D	AX.89317557	4.23E+07	0.13	27.18	3.60	0.06
E1	GY	4D	AX.89721792	4.39E+08	0.09	-4.44	3.76	0.06
E1	GY	4D	AX.89348065	2.17E+07	0.33	2.74	3.72	0.06
E1	GY	4D	AX.89672119	3.44E+08	0.09	-4.28	3.39	0.05
E1	GY	4D	AX.89412785	4.36E+08	0.47	2.37	3.23	0.06

E1	SPM2	4D	AX.89686896	3.52E+08	0.32	18.55	3.38	0.06
E1	GY	4D	AX.89523903	2.06E+07	0.34	2.56	3.39	0.05
E1	GY	4D	AX.89630243	4.39E+08	0.09	-4.44	3.76	0.06
E1	GY	4D	AX.89555421	3.37E+08	0.05	-5.91	3.82	0.06
E1	GY	4D	AX.89454347	2.38E+08	0.07	-4.86	3.51	0.07
E1	SPM2	4D	AX.89558809	4.99E+07	0.11	30.70	4.07	0.07
E1	GY	4D	AX.89721792	4.39E+08	0.09	-4.44	3.76	0.06
E1	GY	4D	AX.89672119	3.44E+08	0.09	-4.28	3.39	0.05
E1	GY	4D	AX.89412785	4.36E+08	0.47	2.37	3.23	0.06
E1	GY	4D	AX.89767153	3.37E+08	0.05	-5.91	3.82	0.06
E1	GY	4D	AX.89767153	3.37E+08	0.05	-5.91	3.82	0.06
E1	SPM2	4D	AX.89405319	1.92E+07	0.43	18.56	3.73	0.06
E1	GY	4D	AX.89473091	3.35E+08	0.05	-5.91	3.82	0.06
E1	GPS	4D	AX.89617408	3.80E+08	0.20	-1.44	3.30	0.06
E1	GY	4D	AX.89555421	3.37E+08	0.05	-5.91	3.82	0.06
E1	GY	4D	AX.89526164	2.05E+07	0.33	2.74	3.72	0.06
E1	GY	4D	AX.89489620	3.46E+08	0.09	-3.99	3.12	0.05
E1	GY	4D	AX.89505725	3.46E+07	0.17	3.49	3.90	0.07
E1	GY	4D	AX.89714193	1.08E+08	0.38	2.41	3.16	0.06
E1	GY	4D	AX.89523903	2.06E+07	0.34	2.56	3.39	0.05
E1	GPS	4D	AX.89769177	3.85E+08	0.08	-2.32	3.93	0.07
E1	GY	4D	AX.89575836	3.59E+06	0.49	2.37	3.25	0.06
E1	GY	4D	AX.89669740	3.59E+06	0.49	2.37	3.25	0.06
E1	GY	4D	AX.89669740	3.59E+06	0.49	2.37	3.25	0.06
E1	GY	4D	AX.89526164	2.05E+07	0.33	2.74	3.72	0.06
E1	SPM2	4D	AX.89329068	6.92E+07	0.13	28.22	3.95	0.07
E1	SPM2	4D	AX.89484618	2.30E+07	0.45	17.66	3.46	0.05
E1	GY	4D	AX.89542428	3.51E+08	0.09	-4.28	3.39	0.05
E1	GPM2	4D	AX.89342620	3.49E+08	0.07	-1227.45	3.40	0.06
E1	GY	4D	AX.89473091	3.35E+08	0.05	-5.91	3.82	0.06
E1	GPM2	4D	AX.89767153	3.37E+08	0.05	-1574.91	4.07	0.07
E1	GPM2	4D	AX.89672119	3.44E+08	0.09	-1235.53	4.14	0.07
E1	GPS	4D	AX.89413533	4.42E+08	0.13	-1.69	3.28	0.06
E1	GPM2	4D	AX.89630243	4.39E+08	0.09	-1084.67	3.44	0.06
E1	GY	4D	AX.89505725	3.46E+07	0.17	3.49	3.90	0.07
E1	GPM2	4D	AX.89327725	3.43E+08	0.07	-1206.55	3.47	0.06
E1	GPM2	4D	AX.89484971	3.49E+08	0.09	-1235.53	4.14	0.07
E1	GPM2	4D	AX.89454347	2.38E+08	0.07	-1327.78	3.89	0.07
E1	GPM2	4D	AX.89449157	3.54E+08	0.07	-1206.55	3.47	0.06
E1	GPM2	4D	AX.89542428	3.51E+08	0.09	-1235.53	4.14	0.07
E1	GY	4D	AX.89542428	3.51E+08	0.09	-4.28	3.39	0.05
E1	GPM2	4D	AX.89621529	5.35E+07	0.15	-841.63	3.16	0.05
E1	GPM2	4D	AX.89669740	3.59E+06	0.49	717.70	4.31	0.08
E1	GY	4D	AX.89575836	3.59E+06	0.49	2.37	3.25	0.06
E1	GPM2	4D	AX.89617408	3.80E+08	0.20	-721.47	3.01	0.05
E1	GY	4D	AX.89630243	4.39E+08	0.09	-4.44	3.76	0.06
E1	GPM2	4D	AX.89489620	3.46E+08	0.09	-1172.17	3.94	0.07
E1	GPM2	4D	AX.89715863	4.55E+08	0.35	-631.10	3.20	0.05
E1	GPM2	4D	AX.89596400	4.55E+08	0.33	-616.22	3.00	0.05
E1	GY	4D	AX.89568572	1.08E+08	0.38	2.41	3.16	0.06

E1	GPM2	4D	AX.89479312	3.46E+08	0.07	-1206.55	3.47	0.06
E1	GPM2	4D	AX.89766381	4.55E+08	0.34	-668.50	3.47	0.06
E1	GPM2	4D	AX.89661082	3.48E+08	0.07	-1206.55	3.47	0.06
E1	GY	4D	AX.89489620	3.46E+08	0.09	-3.99	3.12	0.05
E1	GPM2	4D	AX.89575836	3.59E+06	0.49	717.70	4.31	0.08
E1	GPM2	4D	AX.89363319	3.46E+08	0.09	-1235.53	4.14	0.07
E1	GPM2	4D	AX.89525279	4.55E+08	0.35	-631.10	3.20	0.05
E1	GPM2	4D	AX.89473091	3.35E+08	0.05	-1574.91	4.07	0.07
E1	GPM2	4D	AX.89310874	3.40E+08	0.09	-1235.53	4.14	0.07
E1	GPM2	4D	AX.89721792	4.39E+08	0.09	-1084.67	3.44	0.06
E1	GPM2	4D	AX.89481823	3.53E+08	0.07	-1206.55	3.47	0.06
E1	GPM2	4D	AX.89692908	4.55E+08	0.35	-612.41	3.02	0.05
E1	GPM2	4D	AX.89700742	3.49E+08	0.07	-1206.55	3.47	0.06
E1	TKW	4D	AX.89353662	4.64E+08	0.43	-0.92	3.55	0.06
E1	GPM2	4D	AX.89492683	5.25E+07	0.15	-841.63	3.16	0.05
E1	GPM2	4D	AX.89555421	3.37E+08	0.05	-1574.91	4.07	0.07
E1	GPM2	4D	AX.89504136	3.48E+08	0.07	-1206.55	3.47	0.06
E2	SPM2	4D	AX.89643657	2.84E+07	0.14	-17.07	3.12	0.05
E2	SPM2	4D	AX.89499126	1.46E+07	0.12	18.18	3.13	0.05
E2	GPS	4D	AX.89721792	4.39E+08	0.09	-2.39	3.40	0.05
E2	GPS	4D	AX.89630243	4.39E+08	0.09	-2.39	3.40	0.05
E2	GY	4D	AX.89454347	2.38E+08	0.07	-3.92	3.30	0.07
E2	GY	4D	AX.89454347	2.38E+08	0.07	-3.92	3.30	0.07
E2	GPS	4D	AX.89688246	4.53E+08	0.15	-1.83	3.07	0.05
E2	GPM2	4D	AX.89454347	2.38E+08	0.07	-1023.71	3.42	0.08
E3	GPM2	4D	AX.89743408	3.25E+08	0.25	-1019.66	3.15	0.10
E3	GPS	4D	AX.89630528	3.73E+08	0.32	2.59	3.99	0.11
E3	GPS	4D	AX.89540050	3.73E+08	0.32	2.62	4.07	0.11
E3	GPS	4D	AX.89723304	3.73E+08	0.32	2.59	3.99	0.11
E3	GPS	4D	AX.89405551	4.68E+08	0.38	2.28	3.23	0.11
E3	GPS	4D	AX.89326283	2.62E+07	0.45	2.02	3.04	0.07
E3	GPS	4D	AX.89755769	5.65E+07	0.17	3.17	4.12	0.07
E3	GPS	4D	AX.89639810	5.68E+07	0.17	3.17	4.12	0.07
E3	GPS	4D	AX.89532742	6.94E+07	0.18	2.71	3.21	0.05
E3	GPS	4D	AX.89408379	6.96E+07	0.18	2.71	3.21	0.05
E3	GPS	4D	AX.89547896	3.33E+08	0.37	2.00	3.10	0.07
E3	GPS	4D	AX.89743341	3.42E+08	0.07	4.11	3.07	0.09
E3	GPS	4D	AX.89419420	3.63E+08	0.21	2.99	4.54	0.09
E3	GPS	4D	AX.89674152	3.64E+08	0.18	3.22	4.63	0.10
E3	GPS	4D	AX.89604367	3.72E+08	0.33	2.70	4.33	0.12
E3	TKW	4D	AX.89326283	2.62E+07	0.45	-0.84	3.02	0.05
E3	TKW	4D	AX.89502911	2.86E+07	0.48	-0.88	3.09	0.03
E3	GY	4D	AX.89711898	6.25E+07	0.15	-4.35	3.76	0.07
E4	SPM2	4D	AX.89696241	3.47E+07	0.32	18.63	3.39	0.07
E4	SPM2	4D	AX.89650900	6.28E+07	0.30	17.95	3.16	0.07
E4	SPM2	4D	AX.89671864	8.23E+07	0.09	29.18	3.13	0.03
E4	SPM2	4D	AX.89658806	8.31E+07	0.48	16.93	3.38	0.04
E4	SPM2	4D	AX.89408983	8.75E+07	0.09	29.72	3.21	0.03
E4	SPM2	4D	AX.89473487	9.28E+07	0.32	18.62	3.66	0.05
E4	SPM2	4D	AX.89406343	3.43E+08	0.15	26.27	4.23	0.08

E1	GY	5A	AX.89573968	4.79E+08	0.35	2.57	3.48	0.06
E1	GY	5A	AX.89529755	5.69E+08	0.21	-2.78	3.08	0.04
E1	SPM2	5A	AX.89746268	4.82E+08	0.27	-18.59	3.12	0.05
E1	SPM2	5A	AX.89443794	1.23E+06	0.32	18.41	3.36	0.05
E1	GY	5A	AX.89365450	5.79E+08	0.29	-2.59	3.24	0.05
E1	GY	5A	AX.89674761	4.74E+08	0.07	-6.37	5.70	0.10
E1	GY	5A	AX.89338520	5.70E+08	0.21	-2.96	3.37	0.05
E1	GY	5A	AX.89401228	5.79E+08	0.28	-2.95	3.99	0.06
E1	GY	5A	AX.89328890	4.55E+08	0.09	-4.89	4.50	0.07
E1	GY	5A	AX.89416052	5.70E+08	0.21	-2.78	3.08	0.04
E1	GY	5A	AX.89573968	4.79E+08	0.35	2.57	3.48	0.06
E1	GY	5A	AX.86184719	4.74E+08	0.07	-6.37	5.70	0.10
E1	GY	5A	AX.89662391	5.81E+08	0.26	-3.06	4.09	0.06
E1	GY	5A	AX.89577447	5.81E+08	0.36	-2.86	4.22	0.06
E1	GY	5A	AX.89416052	5.70E+08	0.21	-2.78	3.08	0.04
E1	GY	5A	AX.89674171	5.79E+08	0.28	-2.77	3.57	0.05
E1	GY	5A	AX.89647546	5.79E+08	0.29	-2.59	3.24	0.05
E1	GY	5A	AX.89390857	5.78E+08	0.29	-2.59	3.24	0.05
E1	GY	5A	AX.86167542	4.79E+08	0.35	2.57	3.48	0.06
E1	GY	5A	AX.89532176	5.69E+08	0.21	-2.78	3.08	0.04
E1	GY	5A	AX.89487661	5.79E+08	0.29	-2.59	3.24	0.05
E1	GY	5A	AX.89560591	4.73E+08	0.07	-5.29	4.33	0.07
E1	GY	5A	AX.89424196	5.65E+08	0.13	-3.93	4.08	0.06
E1	GY	5A	AX.89332217	5.70E+08	0.20	-3.18	3.77	0.06
E1	GY	5A	AX.89461701	5.79E+08	0.28	-2.74	3.52	0.05
E1	GY	5A	AX.89593694	5.69E+08	0.21	-2.82	3.11	0.05
E1	GY	5A	AX.89394142	4.72E+08	0.07	-5.65	4.36	0.07
E1	GY	5A	AX.89500280	6.81E+08	0.13	-4.20	4.34	0.07
E1	GY	5A	AX.89351708	5.81E+08	0.37	-2.77	4.05	0.06
E1	GY	5A	AX.89532176	5.69E+08	0.21	-2.78	3.08	0.04
E1	GY	5A	AX.89332916	4.73E+08	0.08	-5.83	5.40	0.09
E1	GY	5A	AX.89494818	5.79E+08	0.28	-2.74	3.52	0.05
E1	GY	5A	AX.89419923	4.79E+08	0.35	2.57	3.48	0.06
E1	GY	5A	AX.89419923	4.79E+08	0.35	2.57	3.48	0.06
E1	GY	5A	AX.89396437	3.66E+08	0.08	-4.30	3.17	0.06
E1	GY	5A	AX.89381438	5.70E+08	0.20	-2.86	3.10	0.05
E1	GY	5A	AX.89749828	4.73E+08	0.07	-5.51	4.41	0.07
E1	GY	5A	AX.89550132	3.70E+08	0.09	-4.47	3.70	0.07
E1	GY	5A	AX.89367689	5.74E+08	0.25	-2.88	3.57	0.05
E1	GY	5A	AX.89358390	5.79E+08	0.28	-2.74	3.52	0.05
E1	GY	5A	AX.89468147	5.80E+08	0.28	-2.74	3.52	0.05
E1	GY	5A	AX.89461701	5.79E+08	0.28	-2.74	3.52	0.05
E1	GY	5A	AX.89351708	5.81E+08	0.37	-2.77	4.05	0.06
E1	GY	5A	AX.89347781	5.70E+08	0.20	-2.86	3.10	0.05
E1	GY	5A	AX.89744768	5.80E+08	0.29	-2.59	3.24	0.05
E1	SPM2	5A	AX.89574822	2.40E+06	0.25	19.80	3.33	0.06
E1	GY	5A	AX.89339851	5.70E+08	0.21	-2.78	3.08	0.04
E1	GY	5A	AX.89373213	6.81E+08	0.13	-3.88	3.89	0.06
E1	GY	5A	AX.89470458	6.81E+08	0.14	-3.31	3.18	0.05
E1	GY	5A	AX.89749828	4.73E+08	0.07	-5.51	4.41	0.07

E1	GY	5A	AX.89498567	5.69E+08	0.21	-2.78	3.08	0.04
E1	GY	5A	AX.89776590	5.78E+08	0.29	-2.59	3.24	0.05
E1	SPM2	5A	AX.89327629	1.87E+07	0.06	35.51	3.29	0.05
E1	GY	5A	AX.86167542	4.79E+08	0.35	2.57	3.48	0.06
E1	GY	5A	AX.89666351	5.79E+08	0.28	-2.74	3.52	0.05
E1	GY	5A	AX.89613621	5.79E+08	0.27	-2.74	3.46	0.05
E1	GY	5A	AX.89468147	5.80E+08	0.28	-2.74	3.52	0.05
E1	GY	5A	AX.89662391	5.81E+08	0.26	-3.06	4.09	0.06
E1	GY	5A	AX.89712014	4.77E+08	0.14	-3.89	4.11	0.06
E1	GY	5A	AX.89712014	4.77E+08	0.14	-3.89	4.11	0.06
E1	GY	5A	AX.89418657	4.73E+08	0.08	-5.83	5.40	0.09
E1	GY	5A	AX.89528830	5.79E+08	0.28	-2.74	3.52	0.05
E1	GY	5A	AX.89367689	5.74E+08	0.25	-2.88	3.57	0.05
E1	GY	5A	AX.89554532	4.56E+08	0.08	-5.00	4.12	0.07
E1	GY	5A	AX.89539733	4.75E+08	0.05	-6.87	5.04	0.08
E1	GY	5A	AX.89348019	5.78E+08	0.29	-2.59	3.24	0.05
E1	GY	5A	AX.89412668	5.69E+08	0.21	-2.78	3.08	0.04
E1	GY	5A	AX.89412871	5.79E+08	0.28	-2.74	3.52	0.05
E1	GY	5A	AX.89353357	4.75E+08	0.05	-6.87	5.04	0.08
E1	GY	5A	AX.89762948	5.79E+08	0.29	-2.59	3.24	0.05
E1	GY	5A	AX.89401627	5.80E+08	0.30	-2.78	3.74	0.06
E1	GY	5A	AX.89312208	5.79E+08	0.28	-2.74	3.52	0.05
E1	GY	5A	AX.89674148	5.69E+08	0.21	-2.78	3.08	0.04
E1	GY	5A	AX.89358390	5.79E+08	0.28	-2.74	3.52	0.05
E1	SPM2	5A	AX.89550971	4.82E+08	0.19	-22.04	3.37	0.06
E1	GY	5A	AX.89437997	3.69E+08	0.09	-4.47	3.70	0.07
E1	SPM2	5A	AX.89642695	5.13E+08	0.27	18.09	3.03	0.05
E1	GY	5A	AX.89355726	4.55E+08	0.07	-4.74	3.40	0.05
E1	GY	5A	AX.89360077	5.80E+08	0.29	-2.59	3.24	0.05
E1	GY	5A	AX.89337095	6.81E+08	0.14	-3.57	3.55	0.05
E1	GY	5A	AX.89348859	5.78E+08	0.28	-2.74	3.52	0.05
E1	GY	5A	AX.89315905	5.70E+08	0.21	-2.78	3.08	0.04
E1	GY	5A	AX.89767302	5.78E+08	0.29	-2.59	3.24	0.05
E1	GY	5A	AX.89338520	5.70E+08	0.21	-2.96	3.37	0.05
E1	GY	5A	AX.89407027	5.79E+08	0.28	-2.74	3.52	0.05
E1	SPM2	5A	AX.89347506	1.22E+06	0.32	18.62	3.45	0.05
E1	GY	5A	AX.89411073	4.55E+08	0.09	-4.89	4.50	0.07
E1	GY	5A	AX.89492912	5.69E+08	0.20	-3.00	3.41	0.05
E1	GY	5A	AX.89492912	5.69E+08	0.20	-3.00	3.41	0.05
E1	GY	5A	AX.89328890	4.55E+08	0.09	-4.89	4.50	0.07
E1	GY	5A	AX.89356069	5.69E+08	0.21	-2.78	3.08	0.04
E1	GY	5A	AX.89348019	5.78E+08	0.29	-2.59	3.24	0.05
E1	GY	5A	AX.89529755	5.69E+08	0.21	-2.78	3.08	0.04
E1	GY	5A	AX.89412871	5.79E+08	0.28	-2.74	3.52	0.05
E1	GY	5A	AX.89341791	5.78E+08	0.29	-2.59	3.24	0.05
E1	GY	5A	AX.89341791	5.78E+08	0.29	-2.59	3.24	0.05
E1	GY	5A	AX.89401627	5.80E+08	0.30	-2.78	3.74	0.06
E1	GY	5A	AX.89401844	6.64E+08	0.22	-2.88	3.32	0.06
E1	GY	5A	AX.89401844	6.64E+08	0.22	-2.88	3.32	0.06
E1	GY	5A	AX.89378039	5.69E+08	0.21	-2.78	3.08	0.04

E1	GY	5A	AX.89378039	5.69E+08	0.21	-2.78	3.08	0.04
E1	GY	5A	AX.86184719	4.74E+08	0.07	-6.37	5.70	0.10
E1	GY	5A	AX.89378499	5.70E+08	0.21	-2.78	3.08	0.04
E1	GY	5A	AX.89557608	5.69E+08	0.21	-2.78	3.08	0.04
E1	GY	5A	AX.89613621	5.79E+08	0.27	-2.74	3.46	0.05
E1	GY	5A	AX.89542560	4.73E+08	0.08	-5.83	5.40	0.09
E1	GY	5A	AX.89367671	5.69E+08	0.21	-2.78	3.08	0.04
E1	GY	5A	AX.89472857	5.69E+08	0.21	-2.78	3.08	0.04
E1	GY	5A	AX.89332916	4.73E+08	0.08	-5.83	5.40	0.09
E1	GY	5A	AX.89712955	4.73E+08	0.08	-5.83	5.40	0.09
E1	GY	5A	AX.89415710	4.72E+08	0.07	-5.65	4.36	0.07
E1	GY	5A	AX.89415710	4.72E+08	0.07	-5.65	4.36	0.07
E1	GY	5A	AX.89436006	5.69E+08	0.21	-2.78	3.08	0.04
E1	GY	5A	AX.89545387	5.76E+07	0.21	-2.82	3.15	0.05
E1	GY	5A	AX.89381438	5.70E+08	0.20	-2.86	3.10	0.05
E1	GY	5A	AX.89628759	5.79E+08	0.28	-2.74	3.52	0.05
E1	GY	5A	AX.89577026	5.69E+08	0.21	-2.78	3.08	0.04
E1	GY	5A	AX.89666351	5.79E+08	0.28	-2.74	3.52	0.05
E1	GY	5A	AX.89418657	4.73E+08	0.08	-5.83	5.40	0.09
E1	GY	5A	AX.89337095	6.81E+08	0.14	-3.57	3.55	0.05
E1	GY	5A	AX.89766154	4.79E+08	0.33	2.68	3.65	0.07
E1	GY	5A	AX.89396437	3.66E+08	0.08	-4.30	3.17	0.06
E1	GY	5A	AX.89603939	3.79E+08	0.08	-4.10	3.06	0.06
E1	GY	5A	AX.89348859	5.78E+08	0.28	-2.74	3.52	0.05
E1	GY	5A	AX.89373213	6.81E+08	0.13	-3.88	3.89	0.06
E1	SPM2	5A	AX.89463191	4.77E+08	0.31	-17.74	3.10	0.06
E1	GY	5A	AX.89705726	5.81E+08	0.37	-2.77	4.05	0.06
E1	GY	5A	AX.89315905	5.70E+08	0.21	-2.78	3.08	0.04
E1	GY	5A	AX.89560591	4.73E+08	0.07	-5.29	4.33	0.07
E1	GY	5A	AX.89757215	6.81E+08	0.14	-3.31	3.18	0.05
E1	GY	5A	AX.89754412	5.65E+08	0.14	-3.78	3.91	0.06
E1	GY	5A	AX.89358289	5.79E+08	0.29	-2.59	3.24	0.05
E1	GY	5A	AX.89467320	5.69E+08	0.21	-2.78	3.08	0.04
E1	GY	5A	AX.89490823	3.68E+08	0.09	-4.47	3.70	0.07
E1	GY	5A	AX.89739451	5.69E+08	0.21	-2.78	3.08	0.04
E1	GY	5A	AX.89351383	5.80E+08	0.29	-2.59	3.24	0.05
E1	GY	5A	AX.89752925	5.70E+08	0.21	-2.78	3.08	0.04
E1	GY	5A	AX.89550132	3.70E+08	0.09	-4.47	3.70	0.07
E1	GY	5A	AX.89708021	5.80E+08	0.29	-2.59	3.24	0.05
E1	GY	5A	AX.89708021	5.80E+08	0.29	-2.59	3.24	0.05
E1	GY	5A	AX.89671282	4.79E+08	0.35	2.57	3.48	0.06
E1	GY	5A	AX.89671282	4.79E+08	0.35	2.57	3.48	0.06
E1	GY	5A	AX.89504966	5.80E+08	0.29	-2.59	3.24	0.05
E1	GY	5A	AX.89513709	4.74E+08	0.07	-6.06	5.51	0.09
E1	GY	5A	AX.89514702	5.80E+08	0.29	-2.59	3.24	0.05
E1	GY	5A	AX.89458875	5.69E+08	0.21	-2.78	3.08	0.04
E1	GY	5A	AX.89458875	5.69E+08	0.21	-2.78	3.08	0.04
E1	GY	5A	AX.89674058	5.70E+08	0.20	-3.18	3.77	0.06
E1	GY	5A	AX.89747446	4.79E+08	0.35	2.57	3.48	0.06
E1	GY	5A	AX.89609633	5.79E+08	0.29	-2.59	3.24	0.05

E1	GY	5A	AX.89674761	4.74E+08	0.07	-6.37	5.70	0.10
E1	GY	5A	AX.89542560	4.73E+08	0.08	-5.83	5.40	0.09
E1	GY	5A	AX.89531737	5.78E+08	0.29	-2.59	3.24	0.05
E1	GY	5A	AX.89390857	5.78E+08	0.29	-2.59	3.24	0.05
E1	GY	5A	AX.89472857	5.69E+08	0.21	-2.78	3.08	0.04
E1	GY	5A	AX.89554603	5.70E+08	0.21	-2.78	3.08	0.04
E1	GY	5A	AX.89554603	5.70E+08	0.21	-2.78	3.08	0.04
E1	GY	5A	AX.89355726	4.55E+08	0.07	-4.74	3.40	0.05
E1	GY	5A	AX.89532286	6.81E+08	0.13	-4.20	4.34	0.07
E1	GY	5A	AX.89355896	5.70E+08	0.21	-2.78	3.08	0.04
E1	GY	5A	AX.89461065	5.79E+08	0.29	-2.59	3.24	0.05
E1	GY	5A	AX.89487661	5.79E+08	0.29	-2.59	3.24	0.05
E1	GY	5A	AX.89365633	5.79E+08	0.28	-2.55	3.12	0.04
E1	GY	5A	AX.89498567	5.69E+08	0.21	-2.78	3.08	0.04
E1	GY	5A	AX.89572674	5.78E+08	0.29	-2.59	3.24	0.05
E1	GY	5A	AX.89593694	5.69E+08	0.21	-2.82	3.11	0.05
E1	GY	5A	AX.89628759	5.79E+08	0.28	-2.74	3.52	0.05
E1	GY	5A	AX.89394142	4.72E+08	0.07	-5.65	4.36	0.07
E1	GY	5A	AX.89740114	4.72E+08	0.07	-5.65	4.36	0.07
E1	GY	5A	AX.89569902	5.69E+08	0.21	-2.78	3.08	0.04
E1	GY	5A	AX.89453604	5.69E+08	0.21	-2.78	3.08	0.04
E1	GY	5A	AX.89453604	5.69E+08	0.21	-2.78	3.08	0.04
E1	GY	5A	AX.89724824	5.69E+08	0.21	-2.78	3.08	0.04
E1	GY	5A	AX.89740805	5.95E+08	0.33	-2.62	3.49	0.06
E1	GY	5A	AX.89360516	5.78E+08	0.29	-2.59	3.24	0.05
E1	GY	5A	AX.89730190	4.55E+08	0.09	-4.89	4.50	0.07
E1	GY	5A	AX.89730190	4.55E+08	0.09	-4.89	4.50	0.07
E1	GY	5A	AX.89515116	5.80E+08	0.28	-2.74	3.52	0.05
E1	GY	5A	AX.89742725	5.70E+08	0.21	-2.78	3.08	0.04
E1	GY	5A	AX.89362731	5.69E+08	0.21	-2.78	3.08	0.04
E1	GY	5A	AX.89434232	5.79E+08	0.28	-2.74	3.52	0.05
E1	GY	5A	AX.89528830	5.79E+08	0.28	-2.74	3.52	0.05
E1	GY	5A	AX.89644722	6.81E+08	0.13	-4.20	4.34	0.07
E1	GY	5A	AX.89644722	6.81E+08	0.13	-4.20	4.34	0.07
E1	GY	5A	AX.89434678	4.73E+08	0.08	-5.83	5.40	0.09
E1	GY	5A	AX.89434678	4.73E+08	0.08	-5.83	5.40	0.09
E1	GY	5A	AX.89371970	3.68E+08	0.09	-4.47	3.70	0.07
E1	GY	5A	AX.89371970	3.68E+08	0.09	-4.47	3.70	0.07
E1	GY	5A	AX.89563193	5.70E+08	0.20	-2.86	3.10	0.05
E1	GY	5A	AX.89539733	4.75E+08	0.05	-6.87	5.04	0.08
E1	GY	5A	AX.89493604	4.79E+08	0.35	2.57	3.48	0.06
E1	GY	5A	AX.89683745	5.80E+08	0.28	-2.74	3.52	0.05
E1	GY	5A	AX.89741034	1.87E+07	0.18	-3.25	3.56	0.05
E1	GY	5A	AX.89741034	1.87E+07	0.18	-3.25	3.56	0.05
E1	GY	5A	AX.89635430	5.70E+08	0.21	-2.78	3.08	0.04
E1	GY	5A	AX.89611595	5.69E+08	0.21	-2.78	3.08	0.04
E1	GY	5A	AX.89436006	5.69E+08	0.21	-2.78	3.08	0.04
E1	GY	5A	AX.89401228	5.79E+08	0.28	-2.95	3.99	0.06
E1	GY	5A	AX.89365450	5.79E+08	0.29	-2.59	3.24	0.05
E1	GY	5A	AX.89424196	5.65E+08	0.13	-3.93	4.08	0.06

E1	GY	5A	AX.89320286	5.70E+08	0.21	-2.78	3.08	0.04
E1	SPM2	5A	AX.89651413	1.61E+06	0.15	23.06	3.18	0.06
E1	GY	5A	AX.89519811	3.77E+08	0.08	-4.30	3.17	0.06
E1	GY	5A	AX.89756768	5.65E+08	0.13	-3.93	4.08	0.06
E1	GY	5A	AX.89528643	5.80E+08	0.29	-2.59	3.24	0.05
E1	GY	5A	AX.89472573	6.81E+08	0.13	-4.20	4.34	0.07
E1	GY	5A	AX.89472573	6.81E+08	0.13	-4.20	4.34	0.07
E1	GY	5A	AX.89554532	4.56E+08	0.08	-5.00	4.12	0.07
E1	GY	5A	AX.89356069	5.69E+08	0.21	-2.78	3.08	0.04
E1	GY	5A	AX.89427323	5.65E+08	0.13	-3.93	4.08	0.06
E1	GY	5A	AX.89369030	6.88E+07	0.23	-2.99	3.67	0.06
E1	GY	5A	AX.89762948	5.79E+08	0.29	-2.59	3.24	0.05
E1	GY	5A	AX.89370346	5.40E+08	0.16	-3.04	3.00	0.04
E1	GY	5A	AX.89739451	5.69E+08	0.21	-2.78	3.08	0.04
E1	GY	5A	AX.89417785	5.79E+08	0.28	-2.74	3.52	0.05
E1	GY	5A	AX.89407027	5.79E+08	0.28	-2.74	3.52	0.05
E1	GY	5A	AX.89691016	5.69E+08	0.21	-2.78	3.08	0.04
E1	GY	5A	AX.89691016	5.69E+08	0.21	-2.78	3.08	0.04
E1	GY	5A	AX.89569902	5.69E+08	0.21	-2.78	3.08	0.04
E1	GPS	5A	AX.89664012	5.88E+08	0.12	1.73	3.03	0.05
E1	GY	5A	AX.89693326	4.55E+08	0.09	-4.89	4.50	0.07
E1	GY	5A	AX.89570035	5.79E+08	0.24	-2.80	3.31	0.05
E1	GY	5A	AX.89570035	5.79E+08	0.24	-2.80	3.31	0.05
E1	GY	5A	AX.89741852	5.79E+08	0.29	-2.59	3.24	0.05
E1	GY	5A	AX.89766534	4.72E+08	0.09	-3.98	3.03	0.05
E1	GY	5A	AX.89766534	4.72E+08	0.09	-3.98	3.03	0.05
E1	GPS	5A	AX.89577447	5.81E+08	0.36	-1.15	3.00	0.05
E1	GY	5A	AX.89705726	5.81E+08	0.37	-2.77	4.05	0.06
E1	GPS	5A	AX.89446700	5.88E+08	0.12	1.73	3.03	0.05
E1	SPM2	5A	AX.89536548	1.22E+06	0.31	18.46	3.33	0.05
E1	GY	5A	AX.89730271	5.78E+08	0.29	-2.59	3.24	0.05
E1	GY	5A	AX.89730271	5.78E+08	0.29	-2.59	3.24	0.05
E1	GY	5A	AX.89397135	6.81E+08	0.14	-3.31	3.18	0.05
E1	GY	5A	AX.89515116	5.80E+08	0.28	-2.74	3.52	0.05
E1	GY	5A	AX.89361677	4.74E+08	0.07	-6.06	5.51	0.09
E1	GY	5A	AX.89757215	6.81E+08	0.14	-3.31	3.18	0.05
E1	GY	5A	AX.89361971	4.73E+08	0.07	-6.10	5.57	0.09
E1	GY	5A	AX.89706227	5.78E+08	0.29	-2.59	3.24	0.05
E1	GY	5A	AX.89706227	5.78E+08	0.29	-2.59	3.24	0.05
E1	GY	5A	AX.89754412	5.65E+08	0.14	-3.78	3.91	0.06
E1	GY	5A	AX.89767302	5.78E+08	0.29	-2.59	3.24	0.05
E1	GY	5A	AX.89514415	5.69E+08	0.21	-2.78	3.08	0.04
E1	GPS	5A	AX.89538290	5.87E+08	0.12	1.73	3.03	0.05
E1	GY	5A	AX.89621569	4.55E+08	0.09	-4.89	4.50	0.07
E1	GPS	5A	AX.89624050	5.86E+08	0.10	1.85	3.06	0.05
E1	GY	5A	AX.89610177	6.64E+08	0.22	-2.88	3.32	0.06
E1	GPS	5A	AX.89554649	5.88E+08	0.12	1.73	3.03	0.05
E1	GY	5A	AX.89744768	5.80E+08	0.29	-2.59	3.24	0.05
E1	GPS	5A	AX.89498504	5.88E+08	0.12	1.73	3.03	0.05
E1	GY	5A	AX.89586923	5.69E+08	0.21	-2.78	3.08	0.04

E1	GY	5A	AX.89469235	5.78E+08	0.29	-2.59	3.24	0.05
E1	GY	5A	AX.89469235	5.78E+08	0.29	-2.59	3.24	0.05
E1	GY	5A	AX.89635430	5.70E+08	0.21	-2.78	3.08	0.04
E1	GY	5A	AX.89627247	4.74E+08	0.07	-6.06	5.51	0.09
E1	GY	5A	AX.89627247	4.74E+08	0.07	-6.06	5.51	0.09
E1	GPS	5A	AX.89514885	5.86E+08	0.13	1.67	3.01	0.05
E1	GY	5A	AX.89626211	4.79E+08	0.35	2.57	3.48	0.06
E1	GPS	5A	AX.89378909	5.87E+08	0.12	1.73	3.03	0.05
E1	GY	5A	AX.89470458	6.81E+08	0.14	-3.31	3.18	0.05
E1	GPS	5A	AX.89676472	5.87E+08	0.12	1.73	3.03	0.05
E1	GPS	5A	AX.89588397	5.87E+08	0.10	1.86	3.09	0.05
E1	GY	5A	AX.89378499	5.70E+08	0.21	-2.78	3.08	0.04
E1	GY	5A	AX.89632555	5.70E+08	0.21	-2.78	3.08	0.04
E1	GY	5A	AX.89519811	3.77E+08	0.08	-4.30	3.17	0.06
E1	GPS	5A	AX.89481347	5.87E+08	0.12	1.73	3.03	0.05
E1	GPS	5A	AX.89685111	5.87E+08	0.12	1.73	3.03	0.05
E1	GPS	5A	AX.89360530	5.75E+08	0.10	1.85	3.07	0.05
E1	GPS	5A	AX.89754412	5.65E+08	0.14	-1.71	3.39	0.06
E1	GY	5A	AX.89427323	5.65E+08	0.13	-3.93	4.08	0.06
E1	GPS	5A	AX.89603589	5.87E+08	0.12	1.73	3.03	0.05
E1	GY	5A	AX.89751422	6.64E+08	0.22	-2.88	3.32	0.06
E1	GY	5A	AX.89751422	6.64E+08	0.22	-2.88	3.32	0.06
E1	GY	5A	AX.89665160	5.48E+08	0.08	-4.54	3.49	0.07
E1	GY	5A	AX.89312208	5.79E+08	0.28	-2.74	3.52	0.05
E1	GPS	5A	AX.89427323	5.65E+08	0.13	-1.79	3.57	0.06
E1	GY	5A	AX.89776327	5.78E+08	0.29	-2.59	3.24	0.05
E1	GY	5A	AX.89572674	5.78E+08	0.29	-2.59	3.24	0.05
E1	GY	5A	AX.89674171	5.79E+08	0.28	-2.77	3.57	0.05
E1	GY	5A	AX.89546156	4.75E+08	0.05	-6.87	5.04	0.08
E1	GY	5A	AX.89546156	4.75E+08	0.05	-6.87	5.04	0.08
E1	GY	5A	AX.89476132	4.73E+08	0.08	-5.83	5.40	0.09
E1	GY	5A	AX.89476132	4.73E+08	0.08	-5.83	5.40	0.09
E1	GY	5A	AX.89740114	4.72E+08	0.07	-5.65	4.36	0.07
E1	GY	5A	AX.89347579	4.79E+08	0.35	2.57	3.48	0.06
E1	GY	5A	AX.89347579	4.79E+08	0.35	2.57	3.48	0.06
E1	GY	5A	AX.89347781	5.70E+08	0.20	-2.86	3.10	0.05
E1	GY	5A	AX.89693874	5.74E+08	0.25	-2.88	3.57	0.05
E1	GY	5A	AX.89693874	5.74E+08	0.25	-2.88	3.57	0.05
E1	GY	5A	AX.89740805	5.95E+08	0.33	-2.62	3.49	0.06
E1	GY	5A	AX.89650222	4.74E+08	0.06	-6.62	5.43	0.09
E1	GY	5A	AX.89766154	4.79E+08	0.33	2.68	3.65	0.07
E1	GY	5A	AX.89603939	3.79E+08	0.08	-4.10	3.06	0.06
E1	GY	5A	AX.89611730	5.80E+08	0.29	-2.59	3.24	0.05
E1	GY	5A	AX.89776633	5.78E+08	0.29	-2.59	3.24	0.05
E1	GPS	5A	AX.89681948	5.89E+08	0.10	1.85	3.06	0.05
E1	GY	5A	AX.89561103	4.75E+08	0.06	-5.85	4.09	0.07
E1	GY	5A	AX.89561103	4.75E+08	0.06	-5.85	4.09	0.07
E1	GY	5A	AX.89556661	6.64E+08	0.22	-2.88	3.32	0.06
E1	GY	5A	AX.89694942	3.68E+08	0.08	-4.30	3.17	0.06
E1	GPS	5A	AX.89512067	5.85E+08	0.10	1.87	3.02	0.05

E1	GY	5A	AX.89744074	5.81E+08	0.37	-2.77	4.04	0.06
E1	GY	5A	AX.89744074	5.81E+08	0.37	-2.77	4.04	0.06
E1	GY	5A	AX.89621656	4.73E+08	0.08	-5.83	5.40	0.09
E1	GY	5A	AX.89513709	4.74E+08	0.07	-6.06	5.51	0.09
E1	GY	5A	AX.89774371	4.74E+08	0.07	-6.06	5.51	0.09
E1	GY	5A	AX.89647546	5.79E+08	0.29	-2.59	3.24	0.05
E1	GY	5A	AX.89353357	4.75E+08	0.05	-6.87	5.04	0.08
E1	GY	5A	AX.89514702	5.80E+08	0.29	-2.59	3.24	0.05
E1	GY	5A	AX.89689921	5.69E+08	0.21	-2.78	3.08	0.04
E1	GY	5A	AX.89689921	5.69E+08	0.21	-2.78	3.08	0.04
E1	GY	5A	AX.89747446	4.79E+08	0.35	2.57	3.48	0.06
E1	GY	5A	AX.89609633	5.79E+08	0.29	-2.59	3.24	0.05
E1	GY	5A	AX.89577447	5.81E+08	0.36	-2.86	4.22	0.06
E1	GY	5A	AX.89531737	5.78E+08	0.29	-2.59	3.24	0.05
E1	GY	5A	AX.89367671	5.69E+08	0.21	-2.78	3.08	0.04
E1	GY	5A	AX.89773817	5.80E+08	0.28	-2.74	3.52	0.05
E1	GY	5A	AX.89773817	5.80E+08	0.28	-2.74	3.52	0.05
E1	GY	5A	AX.89566490	4.73E+08	0.07	-6.10	5.57	0.09
E1	GY	5A	AX.89566490	4.73E+08	0.07	-6.10	5.57	0.09
E1	GY	5A	AX.89712955	4.73E+08	0.08	-5.83	5.40	0.09
E1	GY	5A	AX.89355896	5.70E+08	0.21	-2.78	3.08	0.04
E1	GY	5A	AX.89461065	5.79E+08	0.29	-2.59	3.24	0.05
E1	GY	5A	AX.89555729	5.78E+08	0.29	-2.59	3.24	0.05
E1	GY	5A	AX.89665160	5.48E+08	0.08	-4.54	3.49	0.07
E1	GY	5A	AX.89365633	5.79E+08	0.28	-2.55	3.12	0.04
E1	GPS	5A	AX.89318562	5.86E+08	0.12	1.73	3.03	0.05
E1	GY	5A	AX.89545387	5.76E+07	0.21	-2.82	3.15	0.05
E1	GY	5A	AX.89358289	5.79E+08	0.29	-2.59	3.24	0.05
E1	GY	5A	AX.89417785	5.79E+08	0.28	-2.74	3.52	0.05
E1	GY	5A	AX.89500280	6.81E+08	0.13	-4.20	4.34	0.07
E1	GY	5A	AX.89360077	5.80E+08	0.29	-2.59	3.24	0.05
E1	GY	5A	AX.89521854	6.81E+08	0.13	-4.20	4.34	0.07
E1	GY	5A	AX.89360516	5.78E+08	0.29	-2.59	3.24	0.05
E1	GY	5A	AX.89776633	5.78E+08	0.29	-2.59	3.24	0.05
E1	GY	5A	AX.89361677	4.74E+08	0.07	-6.06	5.51	0.09
E1	GY	5A	AX.89742725	5.70E+08	0.21	-2.78	3.08	0.04
E1	GY	5A	AX.89361971	4.73E+08	0.07	-6.10	5.57	0.09
E1	GY	5A	AX.89467320	5.69E+08	0.21	-2.78	3.08	0.04
E1	GY	5A	AX.89362731	5.69E+08	0.21	-2.78	3.08	0.04
E1	GY	5A	AX.89682075	4.76E+08	0.05	-6.87	5.04	0.08
E1	GY	5A	AX.89755013	4.72E+08	0.07	-5.65	4.36	0.07
E1	GY	5A	AX.89610177	6.64E+08	0.22	-2.88	3.32	0.06
E1	GY	5A	AX.89504966	5.80E+08	0.29	-2.59	3.24	0.05
E1	GY	5A	AX.89700666	5.70E+08	0.21	-2.78	3.08	0.04
E1	GY	5A	AX.89683745	5.80E+08	0.28	-2.74	3.52	0.05
E1	GPM2	5A	AX.89363354	4.45E+08	0.09	-1005.28	3.10	0.05
E1	GPM2	5A	AX.89407707	5.74E+08	0.31	658.52	3.31	0.04
E1	GPM2	5A	AX.89776590	5.78E+08	0.29	-695.74	3.53	0.05
E1	GPM2	5A	AX.89341262	5.81E+08	0.26	891.63	5.10	0.08
E1	GPM2	5A	AX.89355633	5.74E+08	0.30	737.46	3.97	0.06

E1	GPM2	5A	AX.89703618	4.55E+08	0.06	-1324.30	3.32	0.05
E1	GPM2	5A	AX.89612205	3.93E+08	0.17	-780.65	3.09	0.05
E1	GPM2	5A	AX.89709901	3.92E+08	0.18	-864.36	3.81	0.07
E1	GPM2	5A	AX.89365633	5.79E+08	0.28	-742.30	3.91	0.06
E1	GPM2	5A	AX.89434678	4.73E+08	0.08	-1266.73	4.06	0.07
E1	GPM2	5A	AX.89447750	5.74E+08	0.30	737.46	3.97	0.06
E1	GPM2	5A	AX.89463676	5.81E+08	0.34	-727.57	4.12	0.06
E1	GPM2	5A	AX.89706227	5.78E+08	0.29	-695.74	3.53	0.05
E1	GY	5A	AX.89613013	4.73E+08	0.08	-6.05	5.77	0.10
E1	GPM2	5A	AX.89361677	4.74E+08	0.07	-1480.77	5.09	0.09
E1	GY	5A	AX.89332217	5.70E+08	0.20	-3.18	3.77	0.06
E1	GY	5A	AX.89437997	3.69E+08	0.09	-4.47	3.70	0.07
E1	GPM2	5A	AX.89776327	5.78E+08	0.29	-695.74	3.53	0.05
E1	GY	5A	AX.89756768	5.65E+08	0.13	-3.93	4.08	0.06
E1	GY	5A	AX.89528643	5.80E+08	0.29	-2.59	3.24	0.05
E1	GPM2	5A	AX.89634384	4.45E+08	0.09	-1005.28	3.10	0.05
E1	GPM2	5A	AX.89630592	4.73E+08	0.08	-1266.73	4.06	0.07
E1	GY	5A	AX.89369030	6.88E+07	0.23	-2.99	3.67	0.06
E1	GPM2	5A	AX.89602954	5.82E+08	0.34	-727.57	4.12	0.06
E1	SPM2	5A	AX.89462512	1.22E+06	0.32	18.62	3.45	0.05
E1	GPM2	5A	AX.89560591	4.73E+08	0.07	-1160.25	3.33	0.05
E1	GPM2	5A	AX.89459385	5.79E+08	0.28	-702.14	3.55	0.05
E1	GPM2	5A	AX.89361971	4.73E+08	0.07	-1319.55	4.15	0.07
E1	GPM2	5A	AX.89438235	3.92E+08	0.18	-864.36	3.81	0.07
E1	GPM2	5A	AX.89322245	3.92E+08	0.17	-780.65	3.09	0.05
E1	GY	5A	AX.89370346	5.40E+08	0.16	-3.04	3.00	0.04
E1	GPM2	5A	AX.89365450	5.79E+08	0.29	-695.74	3.53	0.05
E1	GY	5A	AX.89776590	5.78E+08	0.29	-2.59	3.24	0.05
E1	GPM2	5A	AX.89683745	5.80E+08	0.28	-737.77	3.87	0.06
E1	GPM2	5A	AX.89317782	3.92E+08	0.18	-864.36	3.81	0.07
E1	GPM2	5A	AX.89401228	5.79E+08	0.28	-767.01	4.11	0.06
E1	GPM2	5A	AX.89353357	4.75E+08	0.05	-1626.14	4.41	0.07
E1	GPM2	5A	AX.89627247	4.74E+08	0.07	-1480.77	5.09	0.09
E1	GPM2	5A	AX.89571890	5.81E+08	0.34	-727.57	4.12	0.06
E1	GPM2	5A	AX.89536487	3.92E+08	0.18	-864.36	3.81	0.07
E1	GPM2	5A	AX.89750176	3.92E+08	0.18	-794.46	3.36	0.06
E1	GPM2	5A	AX.89393436	5.91E+08	0.28	648.13	3.10	0.05
E1	GPM2	5A	AX.89743768	5.55E+08	0.27	-682.98	3.27	0.05
E1	GPM2	5A	AX.89621569	4.55E+08	0.09	-1245.18	4.48	0.07
E1	GPM2	5A	AX.89461701	5.79E+08	0.28	-737.77	3.87	0.06
E1	GPM2	5A	AX.89756768	5.65E+08	0.13	-872.04	3.20	0.05
E1	GPM2	5A	AX.89744481	3.92E+08	0.18	-864.36	3.81	0.07
E1	GPM2	5A	AX.89646475	3.93E+08	0.17	-780.65	3.09	0.05
E1	GY	5A	AX.89630592	4.73E+08	0.08	-5.83	5.40	0.09
E1	GY	5A	AX.89630592	4.73E+08	0.08	-5.83	5.40	0.09
E1	GPM2	5A	AX.89737612	5.81E+08	0.33	-771.62	4.51	0.07
E1	GPM2	5A	AX.89348859	5.78E+08	0.28	-737.77	3.87	0.06
E1	GPM2	5A	AX.89759673	5.78E+08	0.30	-633.40	3.08	0.05
E1	GY	5A	AX.89693326	4.55E+08	0.09	-4.89	4.50	0.07
E1	GPM2	5A	AX.89680671	3.92E+08	0.17	-780.65	3.09	0.05

E1	GPM2	5A	AX.89428853	3.92E+08	0.18	-864.36	3.81	0.07
E1	GPM2	5A	AX.89665160	5.48E+08	0.08	-1301.34	4.26	0.08
E1	GY	5A	AX.89741852	5.79E+08	0.29	-2.59	3.24	0.05
E1	GPM2	5A	AX.89618816	3.92E+08	0.17	-780.65	3.09	0.05
E1	GPM2	5A	AX.89413528	5.81E+08	0.33	-771.62	4.51	0.07
E1	GPM2	5A	AX.89545492	5.78E+08	0.29	-695.74	3.53	0.05
E1	GY	5A	AX.89397135	6.81E+08	0.14	-3.31	3.18	0.05
E1	GPM2	5A	AX.89504966	5.80E+08	0.29	-695.74	3.53	0.05
E1	GPM2	5A	AX.89468147	5.80E+08	0.28	-737.77	3.87	0.06
E1	GPM2	5A	AX.89621454	3.92E+08	0.18	-864.36	3.81	0.07
E1	GY	5A	AX.89514415	5.69E+08	0.21	-2.78	3.08	0.04
E1	GPM2	5A	AX.89635131	3.92E+08	0.17	-780.65	3.09	0.05
E1	GPM2	5A	AX.89611730	5.80E+08	0.29	-695.74	3.53	0.05
E1	GPM2	5A	AX.89579126	4.45E+08	0.09	-1005.28	3.10	0.05
E1	GY	5A	AX.89411073	4.55E+08	0.09	-4.89	4.50	0.07
E1	GY	5A	AX.89621569	4.55E+08	0.09	-4.89	4.50	0.07
E1	GPM2	5A	AX.89495470	3.93E+08	0.17	-783.00	3.16	0.05
E1	GY	5A	AX.89339851	5.70E+08	0.21	-2.78	3.08	0.04
E1	GY	5A	AX.89621656	4.73E+08	0.08	-5.83	5.40	0.09
E1	GPM2	5A	AX.89453765	3.92E+08	0.17	-780.65	3.09	0.05
E1	GPM2	5A	AX.89628759	5.79E+08	0.28	-737.77	3.87	0.06
E1	GPM2	5A	AX.89637689	5.81E+08	0.34	-727.57	4.12	0.06
E1	GPS	5A	AX.89629472	5.87E+08	0.12	1.73	3.03	0.05
E1	GPM2	5A	AX.89419288	5.80E+08	0.31	-647.65	3.22	0.05
E1	GPM2	5A	AX.89340573	3.92E+08	0.18	-864.36	3.81	0.07
E1	GY	5A	AX.89586923	5.69E+08	0.21	-2.78	3.08	0.04
E1	GPS	5A	AX.89322114	4.79E+08	0.13	-1.72	3.33	0.06
E1	GPM2	5A	AX.89358289	5.79E+08	0.29	-695.74	3.53	0.05
E1	GPS	5A	AX.89424196	5.65E+08	0.13	-1.79	3.57	0.06
E1	GPM2	5A	AX.89328890	4.55E+08	0.09	-1245.18	4.48	0.07
E1	GPM2	5A	AX.89776633	5.78E+08	0.29	-695.74	3.53	0.05
E1	GPM2	5A	AX.89360077	5.80E+08	0.29	-695.74	3.53	0.05
E1	GPM2	5A	AX.89382886	4.45E+08	0.09	-1005.28	3.10	0.05
E1	GPM2	5A	AX.89461065	5.79E+08	0.29	-695.74	3.53	0.05
E1	GPM2	5A	AX.89432062	4.83E+08	0.31	-654.67	3.30	0.05
E1	GY	5A	AX.89632555	5.70E+08	0.21	-2.78	3.08	0.04
E1	GY	5A	AX.89733452	5.78E+08	0.29	-2.59	3.24	0.05
E1	GY	5A	AX.89733452	5.78E+08	0.29	-2.59	3.24	0.05
E1	GPM2	5A	AX.89454851	5.91E+08	0.28	648.13	3.10	0.05
E1	GPM2	5A	AX.89572674	5.78E+08	0.29	-695.74	3.53	0.05
E1	GPM2	5A	AX.89341791	5.78E+08	0.29	-695.74	3.53	0.05
E1	GPM2	5A	AX.89550400	4.45E+08	0.09	-1005.28	3.10	0.05
E1	GPM2	5A	AX.89551301	5.81E+08	0.34	-727.57	4.12	0.06
E1	GPM2	5A	AX.89490699	3.92E+08	0.18	-864.36	3.81	0.07
E1	GPM2	5A	AX.89615359	3.92E+08	0.18	-864.36	3.81	0.07
E1	GPM2	5A	AX.89412871	5.79E+08	0.28	-737.77	3.87	0.06
E1	GPM2	5A	AX.89577998	4.45E+08	0.09	-1005.28	3.10	0.05
E1	GPM2	5A	AX.89730271	5.78E+08	0.29	-695.74	3.53	0.05
E1	GPS	5A	AX.89446653	5.87E+08	0.12	1.73	3.03	0.05
E1	GPM2	5A	AX.89545444	5.82E+08	0.34	-727.57	4.12	0.06

E1	GPM2	5A	AX.89358390	5.79E+08	0.28	-737.77	3.87	0.06
E1	GPS	5A	AX.89751484	5.86E+08	0.14	1.73	3.52	0.06
E1	GPS	5A	AX.89485888	5.87E+08	0.12	1.73	3.03	0.05
E1	GY	5A	AX.89776327	5.78E+08	0.29	-2.59	3.24	0.05
E1	GPM2	5A	AX.89714035	3.93E+08	0.17	-780.65	3.09	0.05
E1	GPM2	5A	AX.89555729	5.78E+08	0.29	-695.74	3.53	0.05
E1	GY	5A	AX.89557608	5.69E+08	0.21	-2.78	3.08	0.04
E1	GPS	5A	AX.89510976	5.88E+08	0.12	1.73	3.03	0.05
E1	GPM2	5A	AX.89620526	5.78E+08	0.29	-695.74	3.53	0.05
E1	GPM2	5A	AX.89644722	6.81E+08	0.13	-881.44	3.10	0.05
E1	GPM2	5A	AX.89385525	3.92E+08	0.17	-780.65	3.09	0.05
E1	GPM2	5A	AX.89714222	3.93E+08	0.17	-780.65	3.09	0.05
E1	GPS	5A	AX.89317837	5.87E+08	0.12	1.73	3.03	0.05
E1	GY	5A	AX.89545492	5.78E+08	0.29	-2.59	3.24	0.05
E1	GY	5A	AX.89724824	5.69E+08	0.21	-2.78	3.08	0.04
E1	GY	5A	AX.89521854	6.81E+08	0.13	-4.20	4.34	0.07
E1	GPM2	5A	AX.89355726	4.55E+08	0.07	-1413.22	4.45	0.07
E1	GPM2	5A	AX.89566490	4.73E+08	0.07	-1319.55	4.15	0.07
E1	GY	5A	AX.89650222	4.74E+08	0.06	-6.62	5.43	0.09
E1	GPM2	5A	AX.89351383	5.80E+08	0.29	-695.74	3.53	0.05
E1	GPM2	5A	AX.89570035	5.79E+08	0.24	-716.96	3.33	0.05
E1	GY	5A	AX.89611730	5.80E+08	0.29	-2.59	3.24	0.05
E1	GY	5A	AX.89635689	5.79E+08	0.28	-2.74	3.52	0.05
E1	GY	5A	AX.89635689	5.79E+08	0.28	-2.74	3.52	0.05
E1	GPM2	5A	AX.89674171	5.79E+08	0.28	-688.45	3.41	0.05
E1	GPM2	5A	AX.89609633	5.79E+08	0.29	-695.74	3.53	0.05
E1	GPM2	5A	AX.89666351	5.79E+08	0.28	-737.77	3.87	0.06
E1	GPM2	5A	AX.89749828	4.73E+08	0.07	-1381.73	4.27	0.07
E1	GPM2	5A	AX.89493117	5.81E+08	0.34	-729.51	4.14	0.06
E1	GY	5A	AX.89479761	6.64E+08	0.22	-2.88	3.32	0.06
E1	GY	5A	AX.89479761	6.64E+08	0.22	-2.88	3.32	0.06
E1	GY	5A	AX.89490823	3.68E+08	0.09	-4.47	3.70	0.07
E1	GY	5A	AX.89694942	3.68E+08	0.08	-4.30	3.17	0.06
E1	GPM2	5A	AX.89733452	5.78E+08	0.29	-695.74	3.53	0.05
E1	GPM2	5A	AX.89603908	5.81E+08	0.34	-729.51	4.14	0.06
E1	GPM2	5A	AX.89427323	5.65E+08	0.13	-872.04	3.20	0.05
E1	GY	5A	AX.89752925	5.70E+08	0.21	-2.78	3.08	0.04
E1	GPM2	5A	AX.89600440	3.92E+08	0.18	-864.36	3.81	0.07
E1	GPM2	5A	AX.89647546	5.79E+08	0.29	-695.74	3.53	0.05
E1	GPM2	5A	AX.89418657	4.73E+08	0.08	-1266.73	4.06	0.07
E1	GY	5A	AX.89774371	4.74E+08	0.07	-6.06	5.51	0.09
E1	GY	5A	AX.89493604	4.79E+08	0.35	2.57	3.48	0.06
E1	GY	5A	AX.89596246	5.78E+08	0.29	-2.59	3.24	0.05
E1	GPM2	5A	AX.89577447	5.81E+08	0.36	-835.43	5.37	0.09
E1	GY	5A	AX.89412668	5.69E+08	0.21	-2.78	3.08	0.04
E1	GPM2	5A	AX.89585065	5.78E+08	0.30	-633.40	3.08	0.05
E1	GY	5A	AX.89494818	5.79E+08	0.28	-2.74	3.52	0.05
E1	GPM2	5A	AX.89445986	5.81E+08	0.34	-727.57	4.12	0.06
E1	GPM2	5A	AX.89531737	5.78E+08	0.29	-695.74	3.53	0.05
E1	GPM2	5A	AX.89487661	5.79E+08	0.29	-695.74	3.53	0.05

E1	GPM2	5A	AX.89476132	4.73E+08	0.08	-1266.73	4.06	0.07
E1	GY	5A	AX.89320286	5.70E+08	0.21	-2.78	3.08	0.04
E1	GPM2	5A	AX.89767420	5.82E+08	0.34	-727.57	4.12	0.06
E1	GPM2	5A	AX.89767302	5.78E+08	0.29	-695.74	3.53	0.05
E1	GPM2	5A	AX.89712955	4.73E+08	0.08	-1266.73	4.06	0.07
E1	GY	5A	AX.89495938	1.87E+07	0.18	-3.25	3.56	0.05
E1	GY	5A	AX.89495938	1.87E+07	0.18	-3.25	3.56	0.05
E1	GPM2	5A	AX.89650222	4.74E+08	0.06	-1484.86	4.31	0.07
E1	GPM2	5A	AX.89542560	4.73E+08	0.08	-1266.73	4.06	0.07
E1	GPM2	5A	AX.89596246	5.78E+08	0.29	-695.74	3.53	0.05
E1	GPM2	5A	AX.89678214	3.92E+08	0.18	-864.36	3.81	0.07
E1	GPM2	5A	AX.89621656	4.73E+08	0.08	-1266.73	4.06	0.07
E1	GPM2	5A	AX.89325026	3.92E+08	0.18	-864.36	3.81	0.07
E1	GPM2	5A	AX.89500280	6.81E+08	0.13	-881.44	3.10	0.05
E1	GY	5A	AX.89532286	6.81E+08	0.13	-4.20	4.34	0.07
E1	GPM2	5A	AX.89503150	5.91E+08	0.28	648.13	3.10	0.05
E1	GY	5A	AX.89555729	5.78E+08	0.29	-2.59	3.24	0.05
E1	GY	5A	AX.89755013	4.72E+08	0.07	-5.65	4.36	0.07
E1	GY	5A	AX.89351383	5.80E+08	0.29	-2.59	3.24	0.05
E1	GY	5A	AX.89434232	5.79E+08	0.28	-2.74	3.52	0.05
E1	GY	5A	AX.89700666	5.70E+08	0.21	-2.78	3.08	0.04
E1	GY	5A	AX.89611595	5.69E+08	0.21	-2.78	3.08	0.04
E1	GPM2	5A	AX.89469235	5.78E+08	0.29	-695.74	3.53	0.05
E1	GPM2	5A	AX.89373733	5.81E+08	0.34	-729.51	4.14	0.06
E1	GPM2	5A	AX.89390857	5.78E+08	0.29	-695.74	3.53	0.05
E1	GPM2	5A	AX.89378760	3.92E+08	0.18	-864.36	3.81	0.07
E1	GPM2	5A	AX.89711068	5.78E+08	0.29	-659.02	3.24	0.05
E1	GY	5A	AX.89613013	4.73E+08	0.08	-6.05	5.77	0.10
E1	GPM2	5A	AX.89702845	4.45E+08	0.09	-1005.28	3.10	0.05
E1	GPM2	5A	AX.89378056	3.92E+08	0.18	-864.36	3.81	0.07
E1	GPM2	5A	AX.89493254	5.81E+08	0.34	-727.57	4.12	0.06
E1	GPM2	5A	AX.89644166	4.45E+08	0.09	-1005.28	3.10	0.05
E1	GPM2	5A	AX.89662391	5.81E+08	0.26	-732.06	3.65	0.06
E1	GPM2	5A	AX.89312208	5.79E+08	0.28	-737.77	3.87	0.06
E1	GPM2	5A	AX.89460465	5.81E+08	0.35	-683.36	3.73	0.06
E1	GPM2	5A	AX.89380790	3.93E+08	0.17	-780.65	3.09	0.05
E1	GPM2	5A	AX.89555957	5.81E+08	0.34	-700.67	3.88	0.06
E1	GPM2	5A	AX.89513709	4.74E+08	0.07	-1480.77	5.09	0.09
E1	GPM2	5A	AX.89708021	5.80E+08	0.29	-695.74	3.53	0.05
E1	GPM2	5A	AX.89399742	5.30E+08	0.12	-913.36	3.11	0.05
E1	GY	5A	AX.89674148	5.69E+08	0.21	-2.78	3.08	0.04
E1	GPM2	5A	AX.89401647	5.82E+08	0.34	-727.57	4.12	0.06
E1	GPM2	5A	AX.89324659	4.55E+08	0.10	-1069.71	3.57	0.06
E1	GPM2	5A	AX.89532286	6.81E+08	0.13	-881.44	3.10	0.05
E1	GPM2	5A	AX.89360516	5.78E+08	0.29	-695.74	3.53	0.05
E1	GPM2	5A	AX.89736562	3.91E+08	0.18	-864.36	3.81	0.07
E1	GPM2	5A	AX.89560177	5.81E+08	0.25	864.44	4.78	0.08
E1	GPM2	5A	AX.89528830	5.79E+08	0.28	-737.77	3.87	0.06
E1	GPM2	5A	AX.89561103	4.75E+08	0.06	-1316.29	3.28	0.05
E1	GPM2	5A	AX.89494818	5.79E+08	0.28	-737.77	3.87	0.06

E1	GPM2	5A	AX.89641527	3.92E+08	0.18	-864.36	3.81	0.07
E1	GPM2	5A	AX.89351708	5.81E+08	0.37	-804.94	5.08	0.08
E1	GPM2	5A	AX.89486573	3.92E+08	0.18	-864.36	3.81	0.07
E1	GPS	5A	AX.89320056	5.88E+08	0.12	1.73	3.03	0.05
E1	GPM2	5A	AX.89401627	5.80E+08	0.30	-754.07	4.15	0.07
E1	GPM2	5A	AX.89331314	3.92E+08	0.18	-864.36	3.81	0.07
E1	GPM2	5A	AX.89751458	5.81E+08	0.34	-729.51	4.14	0.06
E1	GPM2	5A	AX.89434232	5.79E+08	0.28	-737.77	3.87	0.06
E1	GPM2	5A	AX.89622904	5.78E+08	0.30	-633.40	3.08	0.05
E1	GPS	5A	AX.89756768	5.65E+08	0.13	-1.79	3.57	0.06
E1	GPM2	5A	AX.86184719	4.74E+08	0.07	-1550.79	5.24	0.09
E1	GPM2	5A	AX.89391038	5.81E+08	0.25	864.44	4.78	0.08
E1	GPM2	5A	AX.89705726	5.81E+08	0.37	-804.94	5.08	0.08
E1	GPM2	5A	AX.89616559	5.78E+08	0.30	-673.27	3.39	0.05
E1	GPM2	5A	AX.89640535	3.95E+08	0.19	-730.94	3.01	0.05
E1	GY	5A	AX.89626211	4.79E+08	0.35	2.57	3.48	0.06
E1	GPM2	5A	AX.89531245	5.81E+08	0.34	-727.57	4.12	0.06
E1	GPM2	5A	AX.89407027	5.79E+08	0.28	-737.77	3.87	0.06
E1	GPM2	5A	AX.89650252	4.45E+08	0.09	-1005.28	3.10	0.05
E1	GPM2	5A	AX.89594418	4.45E+08	0.09	-1005.28	3.10	0.05
E1	GPM2	5A	AX.89708510	5.81E+08	0.34	-727.57	4.12	0.06
E1	GPM2	5A	AX.89656640	4.45E+08	0.09	-1005.28	3.10	0.05
E1	GPM2	5A	AX.89400581	4.45E+08	0.09	-1005.28	3.10	0.05
E1	GPS	5A	AX.89616368	5.86E+08	0.12	1.73	3.03	0.05
E1	GY	5A	AX.89577026	5.69E+08	0.21	-2.78	3.08	0.04
E1	GPM2	5A	AX.89424196	5.65E+08	0.13	-872.04	3.20	0.05
E1	GPM2	5A	AX.89411073	4.55E+08	0.09	-1245.18	4.48	0.07
E1	GY	5A	AX.89750847	5.70E+08	0.20	-2.86	3.10	0.05
E1	GY	5A	AX.89750847	5.70E+08	0.20	-2.86	3.10	0.05
E1	GPM2	5A	AX.89419085	3.92E+08	0.18	-864.36	3.81	0.07
E1	GPM2	5A	AX.89532578	5.81E+08	0.34	-727.57	4.12	0.06
E1	GPM2	5A	AX.89475807	3.92E+08	0.17	-780.65	3.09	0.05
E1	GPM2	5A	AX.89589337	3.92E+08	0.18	-864.36	3.81	0.07
E1	GPM2	5A	AX.89344319	5.81E+08	0.28	691.33	3.43	0.05
E1	GPM2	5A	AX.89360663	5.78E+08	0.30	-673.27	3.39	0.05
E1	GPM2	5A	AX.89635689	5.79E+08	0.28	-737.77	3.87	0.06
E1	GPM2	5A	AX.89435020	5.81E+08	0.34	-729.51	4.14	0.06
E1	GPM2	5A	AX.89775769	4.45E+08	0.09	-1005.28	3.10	0.05
E1	GPM2	5A	AX.89539733	4.75E+08	0.05	-1626.14	4.41	0.07
E1	GPM2	5A	AX.89410951	3.92E+08	0.18	-864.36	3.81	0.07
E1	TKW	5A	AX.89530244	5.72E+08	0.36	0.90	3.22	0.06
E1	GPM2	5A	AX.89417785	5.79E+08	0.28	-737.77	3.87	0.06
E1	GY	5A	AX.89545492	5.78E+08	0.29	-2.59	3.24	0.05
E1	GPM2	5A	AX.89498534	3.92E+08	0.18	-864.36	3.81	0.07
E1	TKW	5A	AX.89740372	6.10E+08	0.14	-1.23	3.16	0.05
E1	TKW	5A	AX.89451889	5.93E+08	0.15	-1.29	3.69	0.06
E1	GPM2	5A	AX.89332916	4.73E+08	0.08	-1266.73	4.06	0.07
E1	GPM2	5A	AX.89546156	4.75E+08	0.05	-1626.14	4.41	0.07
E1	GPM2	5A	AX.89319533	5.91E+08	0.28	648.13	3.10	0.05
E1	GPM2	5A	AX.89567885	3.92E+08	0.17	-780.65	3.09	0.05

E1	GPM2	5A	AX.89514702	5.80E+08	0.29	-695.74	3.53	0.05
E1	GPM2	5A	AX.89730190	4.55E+08	0.09	-1245.18	4.48	0.07
E1	GPM2	5A	AX.89515116	5.80E+08	0.28	-737.77	3.87	0.06
E1	TKW	5A	AX.89328335	5.93E+08	0.15	-1.26	3.54	0.06
E1	GPM2	5A	AX.89741852	5.79E+08	0.29	-695.74	3.53	0.05
E1	GPM2	5A	AX.89540951	4.45E+08	0.09	-1005.28	3.10	0.05
E1	GPM2	5A	AX.89763670	5.81E+08	0.34	-729.51	4.14	0.06
E1	GPM2	5A	AX.89693874	5.74E+08	0.25	-695.53	3.24	0.05
E1	GPM2	5A	AX.89682075	4.76E+08	0.05	-1626.14	4.41	0.07
E1	GPM2	5A	AX.89486014	5.81E+08	0.34	-729.51	4.14	0.06
E1	GPM2	5A	AX.89641969	5.91E+08	0.28	648.13	3.10	0.05
E1	GY	5A	AX.89556661	6.64E+08	0.22	-2.88	3.32	0.06
E1	GPM2	5A	AX.89756297	5.78E+08	0.30	-633.40	3.08	0.05
E1	GPM2	5A	AX.89368848	5.81E+08	0.34	-729.51	4.14	0.06
E1	GPM2	5A	AX.89415710	4.72E+08	0.07	-1195.26	3.15	0.05
E1	GPM2	5A	AX.89528643	5.80E+08	0.29	-695.74	3.53	0.05
E1	GPM2	5A	AX.89447670	4.45E+08	0.09	-1005.28	3.10	0.05
E1	TKW	5A	AX.89393406	6.56E+08	0.07	-1.66	3.26	0.05
E1	TKW	5A	AX.89592042	5.92E+08	0.15	-1.28	3.52	0.06
E1	GPM2	5A	AX.89674761	4.74E+08	0.07	-1550.79	5.24	0.09
E1	TKW	5A	AX.89401724	5.92E+08	0.14	-1.21	3.06	0.05
E1	GPM2	5A	AX.89589560	4.45E+08	0.09	-1005.28	3.10	0.05
E1	GY	5A	AX.89596246	5.78E+08	0.29	-2.59	3.24	0.05
E1	GPM2	5A	AX.89725338	4.55E+08	0.09	-1122.57	3.74	0.06
E1	GPM2	5A	AX.89613013	4.73E+08	0.08	-1308.54	4.30	0.07
E1	GY	5A	AX.89620526	5.78E+08	0.29	-2.59	3.24	0.05
E1	GPM2	5A	AX.89656992	5.81E+08	0.34	-727.57	4.12	0.06
E1	TKW	5A	AX.89736983	6.19E+08	0.06	-1.79	3.18	0.05
E1	GPM2	5A	AX.89394142	4.72E+08	0.07	-1195.26	3.15	0.05
E1	GPM2	5A	AX.89755013	4.72E+08	0.07	-1195.26	3.15	0.05
E1	GY	5A	AX.89674058	5.70E+08	0.20	-3.18	3.77	0.06
E1	GPM2	5A	AX.89337299	5.81E+08	0.33	-771.62	4.51	0.07
E1	GPM2	5A	AX.89711790	4.45E+08	0.09	-1005.28	3.10	0.05
E1	GPM2	5A	AX.89744074	5.81E+08	0.37	-794.54	4.96	0.08
E1	GPM2	5A	AX.89774371	4.74E+08	0.07	-1480.77	5.09	0.09
E1	GPM2	5A	AX.89472573	6.81E+08	0.13	-881.44	3.10	0.05
E1	GPM2	5A	AX.89521854	6.81E+08	0.13	-881.44	3.10	0.05
E1	TKW	5A	AX.89506897	5.93E+08	0.15	-1.26	3.54	0.06
E1	GPM2	5A	AX.89754412	5.65E+08	0.14	-843.37	3.10	0.05
E1	TKW	5A	AX.89501141	5.92E+08	0.15	-1.29	3.69	0.06
E1	TKW	5A	AX.89448049	5.93E+08	0.15	-1.26	3.54	0.06
E1	TKW	5A	AX.89726776	5.93E+08	0.15	-1.28	3.60	0.06
E1	TKW	5A	AX.89339102	5.92E+08	0.15	-1.29	3.69	0.06
E1	TKW	5A	AX.89376560	5.92E+08	0.15	-1.29	3.69	0.06
E1	TKW	5A	AX.89590662	5.92E+08	0.15	-1.29	3.69	0.06
E1	TKW	5A	AX.89521768	5.93E+08	0.15	-1.26	3.54	0.06
E1	TKW	5A	AX.89664402	5.91E+08	0.15	-1.29	3.69	0.06
E1	TKW	5A	AX.89648813	6.99E+08	0.30	0.98	3.46	0.06
E1	TKW	5A	AX.89552993	6.99E+08	0.31	0.97	3.46	0.06
E1	TKW	5A	AX.89698761	5.92E+08	0.15	-1.29	3.69	0.06

E1	TKW	5A	AX.89569078	5.93E+08	0.16	-1.45	4.60	0.08
E1	TKW	5A	AX.89343248	5.92E+08	0.15	-1.28	3.56	0.06
E1	TKW	5A	AX.89328677	6.19E+08	0.06	-1.79	3.18	0.05
E1	TKW	5A	AX.89609993	5.92E+08	0.15	-1.29	3.69	0.06
E1	TKW	5A	AX.89431815	5.93E+08	0.15	-1.26	3.54	0.06
E1	TKW	5A	AX.89401313	5.92E+08	0.15	-1.29	3.58	0.06
E1	TKW	5A	AX.89572135	6.99E+08	0.30	0.98	3.46	0.06
E1	TKW	5A	AX.89572605	5.93E+08	0.15	-1.21	3.23	0.05
E1	TKW	5A	AX.89727760	5.92E+08	0.15	-1.29	3.69	0.06
E1	TKW	5A	AX.89744415	5.93E+08	0.09	-1.74	4.25	0.07
E1	TKW	5A	AX.89513782	5.92E+08	0.15	-1.29	3.69	0.06
E1	TKW	5A	AX.89607657	5.92E+08	0.15	-1.28	3.56	0.06
E1	TKW	5A	AX.89482956	6.99E+08	0.31	0.97	3.46	0.06
E1	GY	5A	AX.89682075	4.76E+08	0.05	-6.87	5.04	0.08
E1	TKW	5A	AX.89319302	5.93E+08	0.15	-1.26	3.54	0.06
E1	TKW	5A	AX.89762465	5.93E+08	0.15	-1.25	3.37	0.06
E1	TKW	5A	AX.89705732	5.93E+08	0.15	-1.26	3.54	0.06
E1	TKW	5A	AX.89411023	5.93E+08	0.15	-1.26	3.54	0.06
E1	GY	5A	AX.89563193	5.70E+08	0.20	-2.86	3.10	0.05
E1	TKW	5A	AX.89538796	5.91E+08	0.15	-1.29	3.69	0.06
E1	TKW	5A	AX.89351345	5.93E+08	0.15	-1.24	3.37	0.06
E1	TKW	5A	AX.89547739	5.92E+08	0.15	-1.29	3.69	0.06
E1	TKW	5A	AX.89596583	5.92E+08	0.15	-1.29	3.69	0.06
E1	GPM2	5A	AX.89348019	5.78E+08	0.29	-695.74	3.53	0.05
E1	TKW	5A	AX.89325223	5.92E+08	0.13	-1.36	3.58	0.06
E1	TKW	5A	AX.89525486	5.93E+08	0.15	-1.26	3.54	0.06
E1	TKW	5A	AX.89402694	4.76E+08	0.07	-1.78	3.69	0.06
E1	TKW	5A	AX.89730783	6.19E+08	0.06	-1.79	3.18	0.05
E1	TKW	5A	AX.89340018	5.92E+08	0.15	-1.29	3.69	0.06
E1	GPM2	5A	AX.89459954	5.81E+08	0.34	-727.57	4.12	0.06
E1	TKW	5A	AX.89450449	5.91E+08	0.15	-1.29	3.69	0.06
E1	GPM2	5A	AX.89367689	5.74E+08	0.25	-695.53	3.24	0.05
E1	GPM2	5A	AX.89773817	5.80E+08	0.28	-737.77	3.87	0.06
E1	TKW	5A	AX.89360530	5.75E+08	0.10	-1.51	3.57	0.06
E1	TKW	5A	AX.89568865	5.93E+08	0.15	-1.26	3.54	0.06
E1	TKW	5A	AX.89549497	7.00E+08	0.29	0.91	3.01	0.05
E1	TKW	5A	AX.89324893	5.92E+08	0.15	-1.29	3.69	0.06
E1	TKW	5A	AX.89654262	5.92E+08	0.13	-1.40	3.84	0.06
E1	GPM2	5A	AX.89566557	3.92E+08	0.18	-864.36	3.81	0.07
E1	GPM2	5A	AX.89490507	3.92E+08	0.18	-864.36	3.81	0.07
E1	TKW	5A	AX.89417671	6.19E+08	0.10	-1.59	3.78	0.07
E1	TKW	5A	AX.89394192	5.92E+08	0.15	-1.29	3.69	0.06
E1	GPM2	5A	AX.89554532	4.56E+08	0.08	-1230.47	3.86	0.06
E1	TKW	5A	AX.89491555	5.92E+08	0.15	-1.29	3.69	0.06
E1	TKW	5A	AX.89529513	5.92E+08	0.15	-1.29	3.69	0.06
E1	TKW	5A	AX.89700655	5.93E+08	0.15	-1.26	3.54	0.06
E1	TKW	5A	AX.89366464	5.93E+08	0.14	-1.36	3.74	0.06
E1	TKW	5A	AX.89659132	5.92E+08	0.15	-1.29	3.69	0.06
E1	GPM2	5A	AX.89436972	4.56E+08	0.07	-1192.54	3.49	0.06
E1	GPM2	5A	AX.89693326	4.55E+08	0.09	-1245.18	4.48	0.07

E1	GPM2	5A	AX.89744768	5.80E+08	0.29	-695.74	3.53	0.05
E1	TKW	5A	AX.89740910	6.19E+08	0.06	-1.79	3.18	0.05
E1	GPM2	5A	AX.89711199	5.81E+08	0.33	-723.24	4.05	0.06
E1	TKW	5A	AX.89547534	5.92E+08	0.15	-1.29	3.69	0.06
E1	GPM2	5A	AX.89762948	5.79E+08	0.29	-695.74	3.53	0.05
E1	TKW	5A	AX.89595049	5.93E+08	0.15	-1.24	3.37	0.06
E1	TKW	5A	AX.89556011	5.93E+08	0.16	-1.27	3.66	0.06
E1	GPM2	5A	AX.89622353	4.45E+08	0.09	-1005.28	3.10	0.05
E1	TKW	5A	AX.89352690	5.93E+08	0.15	-1.26	3.54	0.06
E1	TKW	5A	AX.89570517	5.92E+08	0.15	-1.29	3.69	0.06
E1	TKW	5A	AX.89460348	5.92E+08	0.15	-1.29	3.69	0.06
E1	TKW	5A	AX.89727386	5.92E+08	0.15	-1.29	3.69	0.06
E1	TKW	5A	AX.89651928	5.92E+08	0.15	-1.29	3.69	0.06
E1	GPM2	5A	AX.89384981	4.45E+08	0.09	-1005.28	3.10	0.05
E1	TKW	5A	AX.89611087	5.93E+08	0.13	-1.24	3.13	0.05
E1	TKW	5A	AX.89425333	5.92E+08	0.15	-1.28	3.56	0.06
E1	TKW	5A	AX.89499595	5.92E+08	0.15	-1.24	3.33	0.05
E1	TKW	5A	AX.89332451	5.93E+08	0.15	-1.29	3.69	0.06
E1	TKW	5A	AX.89488830	6.19E+08	0.09	-1.77	4.37	0.08
E1	GPM2	5A	AX.89613621	5.79E+08	0.27	-709.06	3.55	0.05
E1	GPM2	5A	AX.89696551	4.56E+08	0.07	-1192.54	3.49	0.06
E1	GPM2	5A	AX.89704030	5.81E+08	0.33	-734.09	4.16	0.07
E1	TKW	5A	AX.89348621	5.93E+08	0.15	-1.24	3.37	0.06
E1	TKW	5A	AX.89403916	5.93E+08	0.15	-1.26	3.54	0.06
E1	TKW	5A	AX.89618348	5.92E+08	0.15	-1.29	3.69	0.06
E1	GPM2	5A	AX.89606410	3.93E+08	0.17	-780.65	3.09	0.05
E1	TKW	5A	AX.89406356	6.19E+08	0.06	-1.79	3.18	0.05
E1	GY	5A	AX.89620526	5.78E+08	0.29	-2.59	3.24	0.05
E1	GPM2	5A	AX.89740114	4.72E+08	0.07	-1195.26	3.15	0.05
E1	GPM2	5A	AX.89554323	3.92E+08	0.18	-864.36	3.81	0.07
E1	TKW	5A	AX.89632277	5.92E+08	0.15	-1.29	3.69	0.06
E1	GPM2	5A	AX.89370748	3.95E+08	0.19	-756.14	3.19	0.05
E1	TKW	5A	AX.89399161	5.92E+08	0.15	-1.29	3.69	0.06
E1	TKW	5A	AX.89738281	5.92E+08	0.15	-1.29	3.69	0.06
E1	TKW	5A	AX.89549707	6.19E+08	0.06	-1.79	3.18	0.05
E1	TKW	5A	AX.89661979	5.93E+08	0.16	-1.27	3.66	0.06
E1	TKW	5A	AX.89733618	5.92E+08	0.15	-1.29	3.69	0.06
E1	TKW	5A	AX.89663074	5.93E+08	0.16	-1.27	3.66	0.06
E1	TKW	5A	AX.89744976	5.92E+08	0.15	-1.28	3.56	0.06
E1	TKW	5A	AX.89496362	5.93E+08	0.16	-1.27	3.66	0.06
E1	TKW	5A	AX.89609628	5.92E+08	0.15	-1.29	3.69	0.06
E1	TKW	5A	AX.89593804	5.92E+08	0.15	-1.29	3.69	0.06
E1	TKW	5A	AX.89611555	5.93E+08	0.15	-1.29	3.69	0.06
E1	TKW	5A	AX.89549846	5.91E+08	0.15	-1.29	3.69	0.06
E1	TKW	5A	AX.89522203	5.91E+08	0.15	-1.18	3.06	0.05
E2	SPM2	5A	AX.89329110	5.91E+08	0.16	16.53	3.30	0.06
E2	SPM2	5A	AX.89503606	4.57E+08	0.30	-12.81	3.07	0.05
E2	SPM2	5A	AX.89422654	5.91E+08	0.14	17.36	3.29	0.06
E2	SPM2	5A	AX.89446349	5.91E+08	0.14	18.42	3.56	0.06
E2	SPM2	5A	AX.89654471	5.82E+08	0.19	17.90	4.22	0.07

E2	SPM2	5A	AX.89675525	5.91E+08	0.14	18.42	3.56	0.06
E2	SPM2	5A	AX.89309938	5.91E+08	0.13	18.23	3.41	0.06
E2	SPM2	5A	AX.89605708	4.57E+08	0.24	-13.70	3.08	0.05
E2	SPM2	5A	AX.89455246	5.91E+08	0.18	15.43	3.10	0.05
E2	SPM2	5A	AX.89644722	6.81E+08	0.13	-18.44	3.30	0.05
E2	GY	5A	AX.89613013	4.73E+08	0.08	-3.60	3.18	0.04
E2	SPM2	5A	AX.89479046	5.91E+08	0.18	15.43	3.10	0.05
E2	SPM2	5A	AX.89723955	4.57E+08	0.27	-14.74	3.75	0.06
E2	SPM2	5A	AX.89459492	4.56E+08	0.22	-14.42	3.21	0.05
E2	SPM2	5A	AX.89498484	4.57E+08	0.24	-13.70	3.08	0.05
E2	GY	5A	AX.89486715	5.65E+08	0.10	-3.24	3.18	0.04
E2	GY	5A	AX.89486715	5.65E+08	0.10	-3.24	3.18	0.04
E2	SPM2	5A	AX.89432626	5.91E+08	0.17	17.23	3.61	0.06
E2	SPM2	5A	AX.89475722	5.91E+08	0.14	17.36	3.29	0.06
E2	GY	5A	AX.89741234	3.81E+08	0.08	-3.57	3.17	0.06
E2	SPM2	5A	AX.89570034	5.91E+08	0.14	18.42	3.56	0.06
E2	SPM2	5A	AX.89750309	4.57E+08	0.27	-14.28	3.52	0.05
E2	SPM2	5A	AX.89640894	5.91E+08	0.13	18.23	3.41	0.06
E2	SPM2	5A	AX.89607655	5.91E+08	0.14	18.42	3.56	0.06
E2	SPM2	5A	AX.89469921	5.91E+08	0.14	18.42	3.56	0.06
E2	SPM2	5A	AX.89396115	5.91E+08	0.14	18.42	3.56	0.06
E2	SPM2	5A	AX.89689348	5.91E+08	0.14	17.36	3.29	0.06
E2	SPM2	5A	AX.89655112	5.91E+08	0.18	15.43	3.10	0.05
E2	GY	5A	AX.89518533	5.68E+08	0.06	-4.28	3.32	0.04
E2	SPM2	5A	AX.89425879	5.91E+08	0.14	17.36	3.29	0.06
E2	SPM2	5A	AX.89355605	5.91E+08	0.14	18.16	3.48	0.06
E2	GY	5A	AX.89330863	3.81E+08	0.08	-3.57	3.17	0.06
E2	SPM2	5A	AX.89590910	5.91E+08	0.14	18.42	3.56	0.06
E2	SPM2	5A	AX.89485068	5.91E+08	0.18	15.43	3.10	0.05
E2	SPM2	5A	AX.89405358	5.91E+08	0.18	15.43	3.10	0.05
E2	SPM2	5A	AX.89646271	5.91E+08	0.18	15.43	3.10	0.05
E2	SPM2	5A	AX.89600246	5.91E+08	0.14	18.42	3.56	0.06
E2	SPM2	5A	AX.89639104	5.91E+08	0.14	18.42	3.56	0.06
E2	SPM2	5A	AX.89446239	5.91E+08	0.18	15.43	3.10	0.05
E2	SPM2	5A	AX.89393657	5.91E+08	0.18	15.43	3.10	0.05
E2	SPM2	5A	AX.89415579	4.57E+08	0.25	-14.10	3.31	0.05
E2	SPM2	5A	AX.89354699	5.81E+08	0.19	17.90	4.22	0.07
E2	SPM2	5A	AX.89592825	5.91E+08	0.17	17.45	3.76	0.07
E2	SPM2	5A	AX.89432182	5.91E+08	0.14	18.42	3.56	0.06
E2	SPM2	5A	AX.89337095	6.81E+08	0.14	-16.73	3.02	0.05
E2	SPM2	5A	AX.89405076	4.57E+08	0.27	-14.28	3.52	0.05
E2	SPM2	5A	AX.89487227	5.91E+08	0.14	18.42	3.56	0.06
E2	SPM2	5A	AX.89475870	4.55E+08	0.09	25.12	4.32	0.08
E2	SPM2	5A	AX.89356136	5.91E+08	0.14	18.42	3.56	0.06
E2	SPM2	5A	AX.89511267	5.91E+08	0.14	18.42	3.56	0.06
E2	SPM2	5A	AX.89688283	5.91E+08	0.14	18.42	3.56	0.06
E2	SPM2	5A	AX.89566356	5.91E+08	0.14	18.42	3.56	0.06
E2	SPM2	5A	AX.89450821	4.57E+08	0.30	-12.81	3.07	0.05
E2	GY	5A	AX.89437997	3.69E+08	0.09	-4.50	5.15	0.10
E2	SPM2	5A	AX.89390499	5.91E+08	0.14	18.42	3.56	0.06

E2	SPM2	5A	AX.89722106	5.91E+08	0.14	18.16	3.48	0.06
E2	SPM2	5A	AX.89443602	5.91E+08	0.17	16.77	3.45	0.06
E2	SPM2	5A	AX.89546715	5.91E+08	0.14	18.42	3.56	0.06
E2	SPM2	5A	AX.89500280	6.81E+08	0.13	-18.44	3.30	0.05
E2	GY	5A	AX.89712014	4.77E+08	0.14	-2.93	3.44	0.05
E2	GY	5A	AX.89712014	4.77E+08	0.14	-2.93	3.44	0.05
E2	SPM2	5A	AX.89773731	4.56E+08	0.22	-14.42	3.21	0.05
E2	SPM2	5A	AX.89660827	5.91E+08	0.14	17.36	3.29	0.06
E2	GY	5A	AX.89566490	4.73E+08	0.07	-3.89	3.47	0.05
E2	GY	5A	AX.89566490	4.73E+08	0.07	-3.89	3.47	0.05
E2	SPM2	5A	AX.89411312	4.56E+08	0.22	-14.42	3.21	0.05
E2	SPM2	5A	AX.89657151	5.91E+08	0.18	15.43	3.10	0.05
E2	SPM2	5A	AX.89549067	5.91E+08	0.17	18.12	3.94	0.07
E2	GY	5A	AX.89332916	4.73E+08	0.08	-3.71	3.34	0.05
E2	SPM2	5A	AX.89498270	5.91E+08	0.13	17.83	3.29	0.06
E2	SPM2	5A	AX.89739131	5.69E+08	0.09	21.63	3.47	0.06
E2	SPM2	5A	AX.89733858	4.57E+08	0.30	-12.81	3.07	0.05
E2	SPM2	5A	AX.89696752	5.91E+08	0.14	18.42	3.56	0.06
E2	SPM2	5A	AX.89576830	5.91E+08	0.14	18.42	3.56	0.06
E2	SPM2	5A	AX.89755253	5.91E+08	0.14	18.42	3.56	0.06
E2	SPM2	5A	AX.89359545	5.81E+08	0.19	17.90	4.22	0.07
E2	GY	5A	AX.89439613	3.81E+08	0.08	-3.57	3.17	0.06
E2	SPM2	5A	AX.89461566	6.83E+08	0.12	-18.29	3.17	0.05
E2	SPM2	5A	AX.89383545	5.91E+08	0.14	18.42	3.56	0.06
E2	GY	5A	AX.89413159	5.49E+08	0.09	-4.21	4.62	0.09
E2	SPM2	5A	AX.89629830	5.91E+08	0.14	18.42	3.56	0.06
E2	SPM2	5A	AX.89439755	5.91E+08	0.14	17.36	3.29	0.06
E2	GY	5A	AX.89714237	3.80E+08	0.08	-3.57	3.17	0.06
E2	SPM2	5A	AX.89526576	5.91E+08	0.14	18.42	3.56	0.06
E2	SPM2	5A	AX.89764051	5.91E+08	0.14	18.42	3.56	0.06
E2	SPM2	5A	AX.89611987	5.91E+08	0.17	17.45	3.76	0.07
E2	SPM2	5A	AX.89651257	5.91E+08	0.14	18.42	3.56	0.06
E2	SPM2	5A	AX.89455252	5.91E+08	0.14	18.42	3.56	0.06
E2	SPM2	5A	AX.89521854	6.81E+08	0.13	-18.44	3.30	0.05
E2	SPM2	5A	AX.89388125	5.81E+08	0.19	17.90	4.22	0.07
E2	SPM2	5A	AX.89452857	5.91E+08	0.14	18.42	3.56	0.06
E2	SPM2	5A	AX.89714178	5.91E+08	0.14	18.42	3.56	0.06
E2	SPM2	5A	AX.89358223	5.82E+08	0.19	17.90	4.22	0.07
E2	SPM2	5A	AX.89392497	6.88E+08	0.07	27.18	4.32	0.08
E2	SPM2	5A	AX.89566662	5.91E+08	0.14	18.42	3.56	0.06
E2	SPM2	5A	AX.89651661	4.57E+08	0.30	-12.81	3.07	0.05
E2	GY	5A	AX.89546156	4.75E+08	0.05	-5.53	4.73	0.07
E2	SPM2	5A	AX.89740428	5.91E+08	0.14	18.42	3.56	0.06
E2	SPM2	5A	AX.89517401	5.91E+08	0.17	15.46	3.00	0.05
E2	GY	5A	AX.89650222	4.74E+08	0.06	-4.40	3.64	0.05
E2	SPM2	5A	AX.89764773	5.91E+08	0.17	17.45	3.76	0.07
E2	SPM2	5A	AX.89321995	5.91E+08	0.14	18.42	3.56	0.06
E2	SPM2	5A	AX.89319421	5.91E+08	0.14	18.42	3.56	0.06
E2	SPM2	5A	AX.89644682	5.91E+08	0.14	18.42	3.56	0.06
E2	SPM2	5A	AX.89328375	5.91E+08	0.13	18.98	3.66	0.06

E2	SPM2	5A	AX.89406602	5.81E+08	0.19	17.90	4.22	0.07
E2	SPM2	5A	AX.89436077	5.69E+08	0.09	20.41	3.14	0.05
E2	SPM2	5A	AX.89486409	5.91E+08	0.14	17.36	3.29	0.06
E2	SPM2	5A	AX.89361782	4.57E+08	0.30	-12.81	3.07	0.05
E2	SPM2	5A	AX.89739930	5.91E+08	0.14	18.42	3.56	0.06
E2	SPM2	5A	AX.89578026	5.91E+08	0.18	15.43	3.10	0.05
E2	SPM2	5A	AX.89744654	3.96E+08	0.21	15.75	3.58	0.06
E2	SPM2	5A	AX.89774599	5.91E+08	0.17	16.77	3.45	0.06
E2	SPM2	5A	AX.89392107	5.91E+08	0.17	15.72	3.14	0.05
E2	GY	5A	AX.89513709	4.74E+08	0.07	-3.79	3.31	0.05
E2	SPM2	5A	AX.89699773	4.57E+08	0.30	-12.81	3.07	0.05
E2	SPM2	5A	AX.89350670	5.91E+08	0.14	17.14	3.15	0.05
E2	SPM2	5A	AX.89668680	4.57E+08	0.25	-14.10	3.31	0.05
E2	SPM2	5A	AX.89415719	5.91E+08	0.13	18.23	3.41	0.06
E2	SPM2	5A	AX.89559978	5.91E+08	0.16	16.53	3.30	0.06
E2	SPM2	5A	AX.89713998	5.91E+08	0.14	18.42	3.56	0.06
E2	SPM2	5A	AX.89577272	4.56E+08	0.25	-13.99	3.26	0.05
E2	SPM2	5A	AX.89692604	4.57E+08	0.22	-14.11	3.09	0.05
E2	SPM2	5A	AX.89455496	5.91E+08	0.13	18.23	3.41	0.06
E2	SPM2	5A	AX.89736318	4.57E+08	0.30	-12.81	3.07	0.05
E2	SPM2	5A	AX.89532286	6.81E+08	0.13	-18.44	3.30	0.05
E2	SPM2	5A	AX.89564241	5.91E+08	0.14	18.42	3.56	0.06
E2	SPM2	5A	AX.89580173	4.57E+08	0.27	-14.28	3.52	0.05
E2	SPM2	5A	AX.89506778	5.91E+08	0.14	18.42	3.56	0.06
E2	GY	5A	AX.89627247	4.74E+08	0.07	-3.79	3.31	0.05
E2	GY	5A	AX.89627247	4.74E+08	0.07	-3.79	3.31	0.05
E2	SPM2	5A	AX.89689096	4.57E+08	0.30	-12.81	3.07	0.05
E2	SPM2	5A	AX.89434282	4.56E+08	0.22	-14.42	3.21	0.05
E2	SPM2	5A	AX.89571547	5.91E+08	0.18	15.43	3.10	0.05
E2	SPM2	5A	AX.89747789	5.91E+08	0.14	18.42	3.56	0.06
E2	SPM2	5A	AX.89744840	5.81E+08	0.19	17.90	4.22	0.07
E2	SPM2	5A	AX.89314318	5.94E+08	0.13	18.10	3.20	0.05
E2	SPM2	5A	AX.89557528	5.91E+08	0.14	18.42	3.56	0.06
E2	SPM2	5A	AX.89504161	5.91E+08	0.14	18.42	3.56	0.06
E2	SPM2	5A	AX.89474520	4.57E+08	0.28	-13.24	3.15	0.05
E2	GY	5A	AX.89397690	3.80E+08	0.08	-3.57	3.17	0.06
E2	SPM2	5A	AX.89363819	5.91E+08	0.14	18.42	3.56	0.06
E2	SPM2	5A	AX.89411071	5.91E+08	0.13	18.23	3.41	0.06
E2	GY	5A	AX.89561103	4.75E+08	0.06	-4.56	3.63	0.05
E2	SPM2	5A	AX.89472573	6.81E+08	0.13	-18.44	3.30	0.05
E2	SPM2	5A	AX.89569627	5.91E+08	0.14	18.99	3.75	0.06
E2	SPM2	5A	AX.89754487	5.91E+08	0.18	15.43	3.10	0.05
E2	SPM2	5A	AX.89763298	5.91E+08	0.14	18.42	3.56	0.06
E2	SPM2	5A	AX.89332492	5.91E+08	0.18	15.43	3.10	0.05
E2	GY	5A	AX.89755013	4.72E+08	0.07	-5.65	6.01	0.10
E2	GY	5A	AX.89755013	4.72E+08	0.07	-5.65	6.01	0.10
E2	SPM2	5A	AX.89746639	5.91E+08	0.14	17.36	3.29	0.06
E2	SPM2	5A	AX.89648926	5.91E+08	0.14	18.42	3.56	0.06
E2	SPM2	5A	AX.89425021	5.91E+08	0.14	18.42	3.56	0.06
E2	SPM2	5A	AX.89542662	5.91E+08	0.18	15.43	3.10	0.05

E2	SPM2	5A	AX.89749149	5.91E+08	0.18	15.43	3.10	0.05
E2	SPM2	5A	AX.89770722	5.91E+08	0.13	18.81	3.51	0.06
E2	SPM2	5A	AX.89520608	5.91E+08	0.14	18.42	3.56	0.06
E2	GY	5A	AX.89550132	3.70E+08	0.09	-4.50	5.15	0.10
E2	GY	5A	AX.89550132	3.70E+08	0.09	-4.50	5.15	0.10
E2	GY	5A	AX.89375487	1.96E+07	0.06	-4.28	3.23	0.07
E2	GY	5A	AX.89375487	1.96E+07	0.06	-4.28	3.23	0.07
E2	GY	5A	AX.89539733	4.75E+08	0.05	-5.53	4.73	0.07
E2	GY	5A	AX.89539733	4.75E+08	0.05	-5.53	4.73	0.07
E2	GPM2	5A	AX.89600440	3.92E+08	0.18	-719.55	3.80	0.07
E2	GY	5A	AX.89377150	3.81E+08	0.08	-3.57	3.17	0.06
E2	GY	5A	AX.89377150	3.81E+08	0.08	-3.57	3.17	0.06
E2	GY	5A	AX.89413692	3.82E+08	0.08	-3.57	3.17	0.06
E2	GY	5A	AX.89413692	3.82E+08	0.08	-3.57	3.17	0.06
E2	GY	5A	AX.86184719	4.74E+08	0.07	-4.00	3.45	0.05
E2	GY	5A	AX.86184719	4.74E+08	0.07	-4.00	3.45	0.05
E2	GY	5A	AX.89736061	5.49E+08	0.11	-3.44	3.74	0.07
E2	GY	5A	AX.89736061	5.49E+08	0.11	-3.44	3.74	0.07
E2	GY	5A	AX.89736747	3.82E+08	0.08	-3.57	3.17	0.06
E2	GY	5A	AX.89736747	3.82E+08	0.08	-3.57	3.17	0.06
E2	GY	5A	AX.89510352	3.80E+08	0.08	-3.57	3.17	0.06
E2	GY	5A	AX.89510352	3.80E+08	0.08	-3.57	3.17	0.06
E2	GY	5A	AX.89738855	3.81E+08	0.08	-3.57	3.17	0.06
E2	GY	5A	AX.89738855	3.81E+08	0.08	-3.57	3.17	0.06
E2	GY	5A	AX.89312466	5.65E+08	0.10	-3.19	3.01	0.04
E2	GY	5A	AX.89312466	5.65E+08	0.10	-3.19	3.01	0.04
E2	GY	5A	AX.89776986	5.49E+08	0.10	-3.24	3.28	0.07
E2	GY	5A	AX.89776986	5.49E+08	0.10	-3.24	3.28	0.07
E2	GY	5A	AX.89703618	4.55E+08	0.06	-4.65	3.73	0.06
E2	GY	5A	AX.89703618	4.55E+08	0.06	-4.65	3.73	0.06
E2	GPM2	5A	AX.89440555	5.91E+08	0.28	543.62	3.11	0.06
E2	GY	5A	AX.89740114	4.72E+08	0.07	-5.65	6.01	0.10
E2	GY	5A	AX.89740114	4.72E+08	0.07	-5.65	6.01	0.10
E2	GY	5A	AX.89712955	4.73E+08	0.08	-3.71	3.34	0.05
E2	GY	5A	AX.89712955	4.73E+08	0.08	-3.71	3.34	0.05
E2	GY	5A	AX.89418657	4.73E+08	0.08	-3.71	3.34	0.05
E2	GY	5A	AX.89418657	4.73E+08	0.08	-3.71	3.34	0.05
E2	GY	5A	AX.89453773	5.68E+08	0.06	-4.28	3.32	0.04
E2	GY	5A	AX.89453773	5.68E+08	0.06	-4.28	3.32	0.04
E2	GY	5A	AX.89431791	5.49E+08	0.10	-3.19	3.07	0.06
E2	GY	5A	AX.89386393	3.82E+08	0.07	-3.90	3.34	0.04
E2	GY	5A	AX.89386393	3.82E+08	0.07	-3.90	3.34	0.04
E2	GY	5A	AX.89434678	4.73E+08	0.08	-3.71	3.34	0.05
E2	GY	5A	AX.89434678	4.73E+08	0.08	-3.71	3.34	0.05
E2	GPM2	5A	AX.89566557	3.92E+08	0.18	-719.55	3.80	0.07
E2	GY	5A	AX.89775045	1.93E+07	0.15	-2.81	3.43	0.06
E2	GY	5A	AX.89775045	1.93E+07	0.15	-2.81	3.43	0.06
E2	GY	5A	AX.89682075	4.76E+08	0.05	-5.53	4.73	0.07
E2	GY	5A	AX.89682075	4.76E+08	0.05	-5.53	4.73	0.07
E2	GY	5A	AX.89603939	3.79E+08	0.08	-4.11	4.23	0.08

E2	GY	5A	AX.89603939	3.79E+08	0.08	-4.11	4.23	0.08
E2	GY	5A	AX.89710892	6.66E+08	0.37	-1.95	3.05	0.04
E2	GY	5A	AX.89710892	6.66E+08	0.37	-1.95	3.05	0.04
E2	GY	5A	AX.89605507	6.66E+08	0.39	-1.98	3.17	0.04
E2	GY	5A	AX.89605507	6.66E+08	0.39	-1.98	3.17	0.04
E2	GY	5A	AX.89546156	4.75E+08	0.05	-5.53	4.73	0.07
E2	GY	5A	AX.89395291	3.82E+08	0.08	-3.57	3.17	0.06
E2	GY	5A	AX.89395291	3.82E+08	0.08	-3.57	3.17	0.06
E2	GY	5A	AX.89431791	5.49E+08	0.10	-3.19	3.07	0.06
E2	GY	5A	AX.89396437	3.66E+08	0.08	-4.11	4.01	0.08
E2	GY	5A	AX.89396437	3.66E+08	0.08	-4.11	4.01	0.08
E2	GY	5A	AX.89490823	3.68E+08	0.09	-4.50	5.15	0.10
E2	GY	5A	AX.89490823	3.68E+08	0.09	-4.50	5.15	0.10
E2	GY	5A	AX.89361677	4.74E+08	0.07	-3.79	3.31	0.05
E2	GY	5A	AX.89361677	4.74E+08	0.07	-3.79	3.31	0.05
E2	GY	5A	AX.89613013	4.73E+08	0.08	-3.60	3.18	0.04
E2	GY	5A	AX.89371970	3.68E+08	0.09	-4.50	5.15	0.10
E2	GY	5A	AX.89371970	3.68E+08	0.09	-4.50	5.15	0.10
E2	GY	5A	AX.89741234	3.81E+08	0.08	-3.57	3.17	0.06
E2	GY	5A	AX.89518533	5.68E+08	0.06	-4.28	3.32	0.04
E2	GPM2	5A	AX.89328022	7.09E+08	0.30	-577.44	3.50	0.07
E2	GY	5A	AX.89330863	3.81E+08	0.08	-3.57	3.17	0.06
E2	GY	5A	AX.89699280	5.49E+08	0.10	-3.19	3.07	0.06
E2	GPM2	5A	AX.89744481	3.92E+08	0.18	-719.55	3.80	0.07
E2	GY	5A	AX.89437997	3.69E+08	0.09	-4.50	5.15	0.10
E2	GY	5A	AX.89332916	4.73E+08	0.08	-3.71	3.34	0.05
E2	GY	5A	AX.89368511	5.49E+08	0.10	-3.24	3.28	0.07
E2	GY	5A	AX.89368511	5.49E+08	0.10	-3.24	3.28	0.07
E2	GPM2	5A	AX.89468532	5.91E+08	0.28	543.62	3.11	0.06
E2	GPM2	5A	AX.89694788	7.08E+08	0.21	594.72	3.03	0.06
E2	GPM2	5A	AX.89615359	3.92E+08	0.18	-719.55	3.80	0.07
E2	GY	5A	AX.89439613	3.81E+08	0.08	-3.57	3.17	0.06
E2	GY	5A	AX.89413159	5.49E+08	0.09	-4.21	4.62	0.09
E2	GY	5A	AX.89714237	3.80E+08	0.08	-3.57	3.17	0.06
E2	GY	5A	AX.89650222	4.74E+08	0.06	-4.40	3.64	0.05
E2	GPM2	5A	AX.89722866	6.96E+08	0.24	-584.81	3.25	0.05
E2	GY	5A	AX.89513709	4.74E+08	0.07	-3.79	3.31	0.05
E2	GY	5A	AX.89560047	5.49E+08	0.10	-3.19	3.07	0.06
E2	GPM2	5A	AX.89641527	3.92E+08	0.18	-719.55	3.80	0.07
E2	GPM2	5A	AX.89438235	3.92E+08	0.18	-719.55	3.80	0.07
E2	GY	5A	AX.89397690	3.80E+08	0.08	-3.57	3.17	0.06
E2	GY	5A	AX.89561103	4.75E+08	0.06	-4.56	3.63	0.05
E2	GY	5A	AX.89340846	5.49E+08	0.09	-4.21	4.62	0.09
E2	GY	5A	AX.89340846	5.49E+08	0.09	-4.21	4.62	0.09
E2	GPM2	5A	AX.89490507	3.92E+08	0.18	-719.55	3.80	0.07
E2	GPM2	5A	AX.89428853	3.92E+08	0.18	-719.55	3.80	0.07
E2	GY	5A	AX.89353357	4.75E+08	0.05	-5.53	4.73	0.07
E2	GPM2	5A	AX.89552993	6.99E+08	0.31	-660.32	4.48	0.09
E2	GY	5A	AX.89674761	4.74E+08	0.07	-4.00	3.45	0.05
E2	GY	5A	AX.89694942	3.68E+08	0.08	-4.11	4.01	0.08

E2	GY	5A	AX.89694942	3.68E+08	0.08	-4.11	4.01	0.08
E2	GPM2	5A	AX.89709901	3.92E+08	0.18	-719.55	3.80	0.07
E2	GY	5A	AX.89542560	4.73E+08	0.08	-3.71	3.34	0.05
E2	GPM2	5A	AX.89589337	3.92E+08	0.18	-719.55	3.80	0.07
E2	GPM2	5A	AX.89317782	3.92E+08	0.18	-719.55	3.80	0.07
E2	GPM2	5A	AX.89668189	3.92E+08	0.19	-666.41	3.45	0.06
E2	GY	5A	AX.89415710	4.72E+08	0.07	-5.65	6.01	0.10
E2	GY	5A	AX.89415710	4.72E+08	0.07	-5.65	6.01	0.10
E2	GY	5A	AX.89394142	4.72E+08	0.07	-5.65	6.01	0.10
E2	GY	5A	AX.89476132	4.73E+08	0.08	-3.71	3.34	0.05
E2	GY	5A	AX.89476132	4.73E+08	0.08	-3.71	3.34	0.05
E2	GY	5A	AX.89630592	4.73E+08	0.08	-3.71	3.34	0.05
E2	GY	5A	AX.89630592	4.73E+08	0.08	-3.71	3.34	0.05
E2	GPM2	5A	AX.89536487	3.92E+08	0.18	-719.55	3.80	0.07
E2	GY	5A	AX.89560047	5.49E+08	0.10	-3.19	3.07	0.06
E2	GY	5A	AX.89576291	3.82E+08	0.08	-3.57	3.17	0.06
E2	GY	5A	AX.89576291	3.82E+08	0.08	-3.57	3.17	0.06
E2	GY	5A	AX.89599577	3.81E+08	0.08	-3.57	3.17	0.06
E2	GY	5A	AX.89599577	3.81E+08	0.08	-3.57	3.17	0.06
E2	GPM2	5A	AX.89621454	3.92E+08	0.18	-719.55	3.80	0.07
E2	GY	5A	AX.89672548	3.82E+08	0.07	-3.71	3.22	0.06
E2	GY	5A	AX.89672548	3.82E+08	0.07	-3.71	3.22	0.06
E2	GY	5A	AX.89353357	4.75E+08	0.05	-5.53	4.73	0.07
E2	GY	5A	AX.89774371	4.74E+08	0.07	-3.79	3.31	0.05
E2	GY	5A	AX.89674761	4.74E+08	0.07	-4.00	3.45	0.05
E2	GPM2	5A	AX.89490699	3.92E+08	0.18	-719.55	3.80	0.07
E2	GPM2	5A	AX.89563433	5.91E+08	0.28	543.62	3.11	0.06
E2	GY	5A	AX.89542560	4.73E+08	0.08	-3.71	3.34	0.05
E2	GY	5A	AX.89621656	4.73E+08	0.08	-3.71	3.34	0.05
E2	GY	5A	AX.89621656	4.73E+08	0.08	-3.71	3.34	0.05
E2	GPM2	5A	AX.89482956	6.99E+08	0.31	-660.32	4.48	0.09
E2	GY	5A	AX.89394142	4.72E+08	0.07	-5.65	6.01	0.10
E2	GPM2	5A	AX.89331314	3.92E+08	0.18	-719.55	3.80	0.07
E2	GY	5A	AX.89361971	4.73E+08	0.07	-3.89	3.47	0.05
E2	GY	5A	AX.89361971	4.73E+08	0.07	-3.89	3.47	0.05
E2	GPM2	5A	AX.89378056	3.92E+08	0.18	-719.55	3.80	0.07
E2	GY	5A	AX.89699280	5.49E+08	0.10	-3.19	3.07	0.06
E2	GPM2	5A	AX.89736562	3.91E+08	0.18	-719.55	3.80	0.07
E2	GPM2	5A	AX.89699298	6.94E+08	0.24	-607.79	3.42	0.06
E2	GY	5A	AX.89774371	4.74E+08	0.07	-3.79	3.31	0.05
E2	GPM2	5A	AX.89562008	5.91E+08	0.28	543.62	3.11	0.06
E2	GPM2	5A	AX.89549497	7.00E+08	0.29	-531.05	3.03	0.05
E2	GPM2	5A	AX.89678214	3.92E+08	0.18	-719.55	3.80	0.07
E2	GPM2	5A	AX.89486573	3.92E+08	0.18	-719.55	3.80	0.07
E2	GPM2	5A	AX.89588551	5.91E+08	0.28	543.62	3.11	0.06
E2	GPM2	5A	AX.89716248	6.94E+08	0.24	-584.81	3.25	0.05
E2	GPM2	5A	AX.89648813	6.99E+08	0.30	-602.10	3.79	0.07
E2	GPM2	5A	AX.89318849	5.91E+08	0.28	543.62	3.11	0.06
E2	GY	5A	AX.89519811	3.77E+08	0.08	-4.11	4.01	0.08
E2	GY	5A	AX.89519811	3.77E+08	0.08	-4.11	4.01	0.08

E2	GY	5A	AX.89585803	3.82E+08	0.08	-3.57	3.17	0.06
E2	GY	5A	AX.89585803	3.82E+08	0.08	-3.57	3.17	0.06
E2	GPM2	5A	AX.89346281	5.91E+08	0.28	543.62	3.11	0.06
E2	GPM2	5A	AX.89419085	3.92E+08	0.18	-719.55	3.80	0.07
E2	GY	5A	AX.89573776	3.43E+08	0.08	-3.77	3.46	0.07
E2	GPM2	5A	AX.89340573	3.92E+08	0.18	-719.55	3.80	0.07
E2	GPS	5A	AX.89564336	5.49E+08	0.48	-1.38	3.28	0.06
E2	GPM2	5A	AX.89410951	3.92E+08	0.18	-719.55	3.80	0.07
E2	GPM2	5A	AX.89378760	3.92E+08	0.18	-719.55	3.80	0.07
E2	GY	5A	AX.89573776	3.43E+08	0.08	-3.77	3.46	0.07
E2	GPM2	5A	AX.89572135	6.99E+08	0.30	-602.10	3.79	0.07
E2	GPM2	5A	AX.89554323	3.92E+08	0.18	-719.55	3.80	0.07
E2	GPM2	5A	AX.89498534	3.92E+08	0.18	-719.55	3.80	0.07
E2	GPM2	5A	AX.89703618	4.55E+08	0.06	-1069.10	3.10	0.05
E2	GPM2	5A	AX.89750176	3.92E+08	0.18	-687.00	3.58	0.07
E2	GPM2	5A	AX.89325026	3.92E+08	0.18	-719.55	3.80	0.07
E2	GPM2	5A	AX.89543907	6.94E+08	0.25	-623.48	3.72	0.06
E3	GPM2	5A	AX.89661920	4.49E+08	0.14	-1360.77	3.72	0.02
E3	GPM2	5A	AX.89760616	4.49E+08	0.14	-1423.43	3.95	0.02
E3	GPM2	5A	AX.89617171	4.49E+08	0.15	-1414.30	4.04	0.03
E3	GPM2	5A	AX.89711415	4.49E+08	0.14	-1392.65	3.91	0.02
E3	GPM2	5A	AX.89358661	4.50E+08	0.14	-1340.72	3.60	0.02
E3	GPM2	5A	AX.89757084	5.21E+08	0.28	1092.61	3.48	0.11
E3	GPM2	5A	AX.89563032	6.63E+08	0.11	-1341.26	3.07	0.02
E3	GPS	5A	AX.89773683	6.76E+08	0.10	3.25	3.32	0.07
E3	GY	5A	AX.89617171	4.49E+08	0.15	-4.00	3.04	0.02
E3	GY	5A	AX.89711415	4.49E+08	0.14	-4.07	3.10	0.02
E3	GY	5A	AX.89406770	4.55E+08	0.09	-5.02	3.22	0.04
E3	GY	5A	AX.89714529	4.55E+08	0.08	-4.98	3.11	0.03
E3	GY	5A	X.89671700_OT	4.71E+08	0.14	4.01	3.37	0.05
E3	GY	5A	X.89317841_OT	4.71E+08	0.12	4.28	3.41	0.05
E3	GY	5A	AX.89380043	4.71E+08	0.09	4.56	3.02	0.04
E3	GY	5A	AX.89757084	5.21E+08	0.28	3.34	3.00	0.09
E3	GY	5A	AX.89373913	5.36E+08	0.07	-6.08	3.63	0.06
E3	GY	5A	AX.89563032	6.63E+08	0.11	-4.63	3.27	0.02
E3	GY	5A	AX.89679169	6.86E+08	0.05	6.10	3.01	0.05
E3	SPM2	5A	AX.89536153	1.31E+06	0.34	21.24	3.01	0.03
E3	SPM2	5A	AX.89450454	1.34E+06	0.17	27.34	3.43	0.07
E3	TKW	5A	AX.89547274	6.69E+05	0.15	-1.11	3.00	0.08
E3	TKW	5A	AX.89680071	1.23E+06	0.19	-1.07	3.31	0.06
E3	TKW	5A	AX.89661507	5.49E+08	0.47	0.84	3.06	0.00
E3	TKW	5A	AX.89491020	5.49E+08	0.46	0.85	3.18	0.00
E3	TKW	5A	AX.89530506	5.49E+08	0.46	0.85	3.18	0.00
E4	GPM2	5A	AX.89699188	5.92E+08	0.08	-807.62	3.33	0.04
E4	GPM2	5A	AX.89709891	5.93E+08	0.08	-798.50	3.13	0.04
E4	GPM2	5A	AX.89473061	5.92E+08	0.17	-597.66	3.00	0.05
E4	GPM2	5A	AX.89578773	5.93E+08	0.08	-798.50	3.13	0.04
E4	GPM2	5A	AX.89551875	5.93E+08	0.08	-798.50	3.13	0.04
E4	GPM2	5A	AX.89349516	5.92E+08	0.08	-807.62	3.33	0.04
E4	GPM2	5A	AX.89589500	5.92E+08	0.08	-807.62	3.33	0.04

E4	GPM2	5A	AX.89544655	5.92E+08	0.08	-807.62	3.33	0.04
E4	GPM2	5A	AX.89546901	5.93E+08	0.08	-798.50	3.13	0.04
E4	GPM2	5A	AX.89522733	5.93E+08	0.08	-903.67	3.68	0.05
E4	GPM2	5A	AX.89330508	5.93E+08	0.08	-798.50	3.13	0.04
E4	GPM2	5A	AX.89401724	5.92E+08	0.08	-807.62	3.33	0.04
E4	GPM2	5A	AX.89524839	5.93E+08	0.08	-903.67	3.68	0.05
E4	GPM2	5A	AX.89489270	5.93E+08	0.08	-903.67	3.68	0.05
E4	GPM2	5A	AX.89462533	5.92E+08	0.08	-825.52	3.36	0.05
E4	GPM2	5A	AX.89321652	5.92E+08	0.08	-807.62	3.33	0.04
E4	GPM2	5A	AX.89478035	5.92E+08	0.08	-843.80	3.33	0.04
E4	GPM2	5A	AX.89658872	5.92E+08	0.08	-807.62	3.33	0.04
E4	GPM2	5A	AX.89752642	5.92E+08	0.08	-807.62	3.33	0.04
E4	GPM2	5A	AX.89507566	5.93E+08	0.08	-807.62	3.33	0.04
E4	GPM2	5A	AX.89717890	5.93E+08	0.08	-807.62	3.33	0.04
E4	GPM2	5A	AX.89502057	5.93E+08	0.08	-868.44	3.48	0.05
E4	GPM2	5A	AX.89681189	5.93E+08	0.08	-798.50	3.13	0.04
E4	GPM2	5A	AX.89358620	5.93E+08	0.08	-798.50	3.13	0.04
E4	GPM2	5A	AX.89398585	5.92E+08	0.08	-843.32	3.28	0.05
E4	GPM2	5A	AX.89421365	5.92E+08	0.09	-791.91	3.54	0.05
E4	GPM2	5A	AX.89431839	5.93E+08	0.08	-798.50	3.13	0.04
E4	GPM2	5A	AX.89763927	5.93E+08	0.08	-903.67	3.68	0.05
E4	GPM2	5A	AX.89735430	5.93E+08	0.08	-903.67	3.68	0.05
E4	GPM2	5A	AX.89622712	5.93E+08	0.08	-903.67	3.68	0.05
E4	GPM2	5A	AX.89564282	5.95E+08	0.33	-470.07	3.20	0.05
E4	GPM2	5A	AX.89652899	5.92E+08	0.08	-847.22	3.22	0.05
E4	GPM2	5A	AX.89486445	5.92E+08	0.07	-839.38	3.05	0.04
E4	GPM2	5A	AX.89680165	5.92E+08	0.08	-807.62	3.33	0.04
E4	GPM2	5A	AX.89729633	5.92E+08	0.08	-807.62	3.33	0.04
E4	GPM2	5A	AX.89736080	5.92E+08	0.08	-807.62	3.33	0.04
E4	GPM2	5A	AX.89738574	5.92E+08	0.08	-807.62	3.33	0.04
E4	GPM2	5A	AX.89682323	5.92E+08	0.08	-807.62	3.33	0.04
E4	GPM2	5A	AX.89604407	5.92E+08	0.09	-784.22	3.26	0.04
E4	GPM2	5A	AX.89736516	5.92E+08	0.08	-807.62	3.33	0.04
E4	GPM2	5A	AX.89687552	5.91E+08	0.17	-618.12	3.28	0.06
E4	GPM2	5A	AX.89554510	5.91E+08	0.17	-612.39	3.13	0.05
E4	GPM2	5A	AX.89527897	5.91E+08	0.17	-618.12	3.28	0.06
E4	GPM2	5A	AX.89537171	5.91E+08	0.17	-629.99	3.34	0.06
E4	GPM2	5A	AX.89606663	3.81E+07	0.23	-531.52	3.10	0.03
E4	GPM2	5A	AX.89454674	2.95E+08	0.16	600.64	3.12	0.07
E4	GPM2	5A	AX.89570321	3.09E+08	0.16	628.19	3.23	0.07
E4	GPM2	5A	AX.89522473	5.12E+08	0.10	-739.04	3.31	0.03
E4	GPM2	5A	AX.89776907	5.91E+08	0.07	-984.18	3.99	0.06
E4	GPM2	5A	AX.89654141	5.91E+08	0.08	-810.93	3.24	0.04
E4	GPM2	5A	AX.89654699	5.91E+08	0.08	-810.93	3.24	0.04
E4	GPM2	5A	AX.89313176	5.91E+08	0.08	-810.93	3.24	0.04
E4	GPM2	5A	AX.89550470	5.91E+08	0.07	-984.18	3.99	0.06
E4	GPM2	5A	AX.89604111	5.91E+08	0.07	-984.18	3.99	0.06
E4	GPM2	5A	AX.89522203	5.91E+08	0.08	-829.76	3.27	0.04
E4	GY	5A	AX.89529513	5.92E+08	0.18	-2.22	3.77	0.06
E4	GY	5A	AX.89570517	5.92E+08	0.18	-2.22	3.77	0.06

E4	GY	5A	AX.89609993	5.92E+08	0.18	-2.22	3.77	0.06
E4	GY	5A	AX.89343248	5.92E+08	0.18	-2.23	3.88	0.06
E4	GY	5A	AX.89607657	5.92E+08	0.18	-2.23	3.88	0.06
E4	GY	5A	AX.89744976	5.92E+08	0.18	-2.19	3.83	0.06
E4	GY	5A	AX.89332451	5.93E+08	0.18	-2.23	3.88	0.06
E4	GY	5A	AX.89564282	5.95E+08	0.33	-1.66	3.47	0.05
E4	GY	5A	AX.89730631	6.81E+08	0.24	-1.79	3.26	0.05
E4	GY	5A	AX.89669520	6.81E+08	0.24	-1.74	3.12	0.05
E4	GY	5A	AX.89615829	6.82E+08	0.24	-1.79	3.26	0.05
E4	GY	5A	AX.89591696	6.82E+08	0.24	-1.74	3.12	0.05
E4	GY	5A	AX.89399967	6.82E+08	0.24	-1.77	3.19	0.05
E4	GY	5A	AX.89323718	6.82E+08	0.24	-1.79	3.26	0.05
E4	GY	5A	AX.89353663	6.82E+08	0.24	-1.79	3.26	0.05
E4	GY	5A	AX.89492854	6.82E+08	0.24	-1.79	3.26	0.05
E4	GY	5A	AX.89726154	6.83E+08	0.24	-1.79	3.26	0.05
E4	GY	5A	AX.89315375	6.83E+08	0.24	-1.85	3.43	0.05
E4	GY	5A	AX.89677477	6.83E+08	0.24	-1.74	3.12	0.05
E4	GY	5A	AX.89665828	6.83E+08	0.25	-1.79	3.38	0.05
E4	GY	5A	AX.89478190	6.83E+08	0.25	-1.73	3.17	0.05
E4	GY	5A	AX.89767529	6.83E+08	0.29	-1.60	3.03	0.04
E4	GY	5A	AX.89376560	5.92E+08	0.17	-2.26	3.89	0.06
E4	GY	5A	AX.89592042	5.92E+08	0.17	-2.26	3.89	0.06
E4	GY	5A	AX.89340018	5.92E+08	0.17	-2.26	3.89	0.06
E4	GY	5A	AX.89601577	5.92E+08	0.17	-2.26	3.89	0.06
E4	GY	5A	AX.89738281	5.92E+08	0.20	-2.15	3.93	0.06
E4	GY	5A	AX.89733618	5.92E+08	0.18	-2.23	3.88	0.06
E4	GY	5A	AX.89399161	5.92E+08	0.18	-2.03	3.36	0.05
E4	GY	5A	AX.89425333	5.92E+08	0.18	-2.23	3.88	0.06
E4	GY	5A	AX.89366839	2.09E+06	0.12	-2.32	3.11	0.05
E4	GY	5A	AX.89687552	5.91E+08	0.17	-2.42	4.26	0.07
E4	GY	5A	AX.89554510	5.91E+08	0.17	-2.42	4.17	0.07
E4	GY	5A	AX.89527897	5.91E+08	0.17	-2.42	4.26	0.07
E4	GY	5A	AX.89537171	5.91E+08	0.17	-2.45	4.32	0.07
E4	GY	5A	AX.89549846	5.91E+08	0.18	-2.23	3.87	0.06
E4	GY	5A	AX.89450449	5.91E+08	0.18	-2.23	3.87	0.06
E4	GY	5A	AX.89491555	5.92E+08	0.18	-2.22	3.77	0.06
E4	GY	5A	AX.89501141	5.92E+08	0.18	-2.22	3.77	0.06
E4	GY	5A	AX.89547739	5.92E+08	0.17	-1.98	3.06	0.05
E4	GY	5A	AX.89659132	5.92E+08	0.18	-2.22	3.77	0.06
E4	GY	5A	AX.89394192	5.92E+08	0.18	-2.22	3.77	0.06
E4	GY	5A	AX.89324893	5.92E+08	0.18	-2.22	3.77	0.06
E4	GY	5A	AX.89727386	5.92E+08	0.18	-2.22	3.77	0.06
E4	GY	5A	AX.89727760	5.92E+08	0.18	-2.23	3.88	0.06
E4	GY	5A	AX.89339102	5.92E+08	0.18	-2.23	3.88	0.06
E4	GY	5A	AX.89499595	5.92E+08	0.18	-2.23	3.88	0.06
E4	GY	5A	AX.89632277	5.92E+08	0.18	-2.23	3.88	0.06
E4	GY	5A	AX.89712525	5.92E+08	0.18	-2.23	3.88	0.06
E4	GY	5A	AX.89513782	5.92E+08	0.18	-2.23	3.88	0.06
E4	GY	5A	AX.89473061	5.92E+08	0.17	-2.47	4.31	0.07
E4	GY	5A	AX.89596583	5.92E+08	0.18	-2.23	3.88	0.06

E4	GY	5A	AX.89547534	5.92E+08	0.18	-2.23	3.88	0.06
E4	GY	5A	AX.89401313	5.92E+08	0.18	-2.23	3.88	0.06
E4	GY	5A	AX.89593804	5.92E+08	0.18	-2.22	3.77	0.06
E4	GY	5A	AX.89590662	5.92E+08	0.18	-2.23	3.88	0.06
E4	SPM2	5A	X.89551039_OT	4.62E+08	0.32	17.92	3.15	0.06
E4	SPM2	5A	AX.89411487	4.73E+08	0.14	24.51	3.17	0.09
E4	TKW	5A	AX.89447572	5.49E+08	0.11	1.43	3.64	0.02
E4	TKW	5A	AX.89581028	5.49E+08	0.11	1.33	3.18	0.01
E4	TKW	5A	AX.89773652	5.49E+08	0.11	1.33	3.18	0.01
E4	TKW	5A	AX.89476088	6.80E+08	0.38	-0.87	3.18	0.04
E4	TKW	5A	AX.89473320	6.80E+08	0.38	-0.87	3.18	0.04
E4	TKW	5A	AX.89510180	6.80E+08	0.37	-0.86	3.05	0.04
E4	TKW	5A	AX.89683744	6.80E+08	0.39	-0.86	3.07	0.05
E4	TKW	5A	AX.89325805	6.80E+08	0.42	-0.85	3.06	0.05
E1	GY	5B	AX.89383487	6.43E+08	0.11	-3.67	3.08	0.05
E1	SPM2	5B	AX.89454031	1.32E+07	0.30	-19.51	3.58	0.06
E1	GY	5B	AX.89545878	6.35E+08	0.27	3.03	4.09	0.06
E1	GY	5B	AX.89545878	6.35E+08	0.27	3.03	4.09	0.06
E1	GY	5B	AX.89342676	6.30E+08	0.23	2.90	3.51	0.06
E1	GY	5B	AX.89573412	6.36E+08	0.34	2.45	3.19	0.05
E1	GY	5B	AX.89554094	5.87E+08	0.31	2.47	3.09	0.04
E1	GY	5B	AX.89673663	6.36E+08	0.34	2.45	3.19	0.05
E1	GY	5B	AX.89437644	4.89E+08	0.05	-5.21	3.13	0.05
E1	GY	5B	AX.89335298	6.34E+08	0.28	2.62	3.28	0.05
E1	GY	5B	AX.86167810	5.81E+08	0.24	2.67	3.12	0.03
E1	GY	5B	AX.89760671	5.86E+08	0.33	2.51	3.29	0.04
E1	GY	5B	AX.89309378	5.88E+08	0.27	-2.66	3.31	0.06
E1	GY	5B	AX.89480538	6.34E+08	0.28	2.62	3.28	0.05
E1	GY	5B	AX.86167810	5.81E+08	0.24	2.67	3.12	0.03
E1	GY	5B	AX.89448319	6.37E+08	0.30	2.94	4.14	0.06
E1	GY	5B	AX.89390545	5.59E+08	0.28	2.61	3.27	0.05
E1	SPM2	5B	AX.89748868	1.32E+07	0.32	-17.72	3.14	0.05
E1	GY	5B	AX.89357083	6.21E+08	0.24	2.99	3.74	0.07
E1	GY	5B	AX.89380715	6.93E+08	0.24	-2.85	3.48	0.06
E1	GY	5B	AX.89668374	3.15E+08	0.21	-3.74	5.12	0.08
E1	GY	5B	AX.89458043	5.38E+08	0.08	4.16	3.15	0.05
E1	GY	5B	AX.89685979	6.32E+08	0.27	2.74	3.46	0.06
E1	GY	5B	AX.89624706	6.34E+08	0.28	2.62	3.28	0.05
E1	GY	5B	AX.89325031	1.98E+07	0.18	-3.25	3.58	0.05
E1	GY	5B	AX.89419340	6.43E+08	0.11	-3.72	3.15	0.05
E1	GY	5B	AX.89523813	6.37E+08	0.13	3.68	3.66	0.06
E1	GY	5B	AX.89357028	2.00E+07	0.18	-3.25	3.58	0.05
E1	GY	5B	AX.89380715	6.93E+08	0.24	-2.85	3.48	0.06
E1	GY	5B	AX.89471949	1.95E+07	0.18	-3.25	3.58	0.05
E1	GY	5B	AX.89627311	4.67E+08	0.20	-2.95	3.23	0.06
E1	GY	5B	AX.89624258	6.43E+08	0.11	-3.67	3.08	0.05
E1	GY	5B	AX.89760671	5.86E+08	0.33	2.51	3.29	0.04
E1	GY	5B	AX.89486250	6.43E+08	0.11	-3.67	3.08	0.05
E1	GY	5B	AX.89370717	7.04E+08	0.28	-2.76	3.61	0.06
E1	GY	5B	AX.89716468	6.43E+08	0.11	-3.67	3.08	0.05

E1	GY	5B	AX.89588192	5.86E+08	0.35	2.61	3.62	0.05
E1	GY	5B	AX.89386836	5.87E+08	0.34	2.54	3.40	0.04
E1	GY	5B	AX.89607377	6.35E+08	0.28	2.62	3.28	0.05
E1	GY	5B	AX.89480538	6.34E+08	0.28	2.62	3.28	0.05
E1	GY	5B	AX.89389594	6.36E+08	0.34	2.45	3.19	0.05
E1	GY	5B	AX.89331656	6.32E+08	0.27	2.74	3.46	0.06
E1	GY	5B	AX.89627311	4.67E+08	0.20	-2.95	3.23	0.06
E1	GY	5B	AX.89774767	6.37E+08	0.34	2.45	3.19	0.05
E1	GY	5B	AX.89340138	6.30E+08	0.23	2.90	3.51	0.06
E1	GY	5B	AX.89448752	6.37E+08	0.30	2.94	4.14	0.06
E1	GY	5B	AX.89371830	6.32E+08	0.27	2.74	3.46	0.06
E1	GY	5B	AX.89523813	6.37E+08	0.13	3.68	3.66	0.06
E1	GY	5B	AX.89410418	4.79E+08	0.05	-5.21	3.14	0.05
E1	GY	5B	AX.89384466	6.36E+08	0.34	2.45	3.21	0.05
E1	GY	5B	AX.89384466	6.36E+08	0.34	2.45	3.21	0.05
E1	GY	5B	AX.89734081	7.13E+08	0.06	-4.91	3.04	0.05
E1	GY	5B	AX.89600742	5.61E+08	0.25	2.66	3.15	0.05
E1	GY	5B	AX.89530183	4.80E+08	0.05	-5.21	3.14	0.05
E1	GY	5B	AX.89702097	6.35E+08	0.28	2.62	3.28	0.05
E1	SPM2	5B	AX.89388779	1.32E+07	0.29	-19.16	3.44	0.06
E1	GY	5B	AX.89746558	4.79E+08	0.05	-5.21	3.14	0.05
E1	GY	5B	AX.89390545	5.59E+08	0.28	2.61	3.27	0.05
E1	GY	5B	AX.89389198	6.32E+08	0.23	2.82	3.31	0.06
E1	GY	5B	AX.89424748	1.13E+07	0.17	-3.31	3.62	0.05
E1	GY	5B	AX.89448752	6.37E+08	0.30	2.94	4.14	0.06
E1	GY	5B	AX.89731133	6.35E+08	0.28	2.62	3.28	0.05
E1	GY	5B	AX.89385868	1.13E+07	0.17	-3.31	3.62	0.05
E1	GY	5B	AX.89533286	5.87E+08	0.13	-3.67	3.65	0.07
E1	GY	5B	AX.89335298	6.34E+08	0.28	2.62	3.28	0.05
E1	GY	5B	AX.89325031	1.98E+07	0.18	-3.25	3.58	0.05
E1	GY	5B	AX.89395633	5.86E+08	0.33	2.54	3.37	0.04
E1	GY	5B	AX.89332158	4.79E+08	0.05	-5.21	3.14	0.05
E1	GY	5B	AX.89403157	6.32E+08	0.27	2.74	3.46	0.06
E1	GY	5B	AX.89607377	6.35E+08	0.28	2.62	3.28	0.05
E1	GY	5B	AX.89594998	7.03E+08	0.28	-2.64	3.30	0.06
E1	GY	5B	AX.89406483	1.96E+07	0.18	-3.24	3.62	0.07
E1	GY	5B	AX.89440092	6.35E+08	0.28	2.62	3.28	0.05
E1	GY	5B	AX.89395633	5.86E+08	0.33	2.54	3.37	0.04
E1	GY	5B	AX.89322854	1.13E+07	0.17	-3.31	3.62	0.05
E1	GY	5B	AX.89454130	6.38E+08	0.49	-2.27	3.08	0.05
E1	GY	5B	AX.89439094	5.60E+08	0.25	2.66	3.15	0.05
E1	GY	5B	AX.89630809	4.80E+08	0.05	-5.21	3.14	0.05
E1	GY	5B	AX.89529948	2.00E+07	0.29	-2.53	3.15	0.05
E1	GY	5B	AX.89668374	3.15E+08	0.21	-3.74	5.12	0.08
E1	GY	5B	AX.89376899	5.87E+08	0.27	-2.66	3.31	0.06
E1	GY	5B	AX.89458043	5.38E+08	0.08	4.16	3.15	0.05
E1	GY	5B	AX.89357083	6.21E+08	0.24	2.99	3.74	0.07
E1	GY	5B	AX.89734081	7.13E+08	0.06	-4.91	3.04	0.05
E1	GY	5B	AX.89392715	6.43E+08	0.11	-3.67	3.08	0.05
E1	GY	5B	AX.89649925	4.79E+08	0.05	-5.21	3.14	0.05

E1	GY	5B	AX.89548636	1.96E+07	0.14	-3.52	3.56	0.05
E1	GY	5B	AX.89548636	1.96E+07	0.14	-3.52	3.56	0.05
E1	SPM2	5B	AX.89430959	5.19E+08	0.11	-29.48	3.71	0.06
E1	GY	5B	AX.86169102	5.42E+08	0.11	3.65	3.04	0.05
E1	GY	5B	AX.89354035	6.32E+08	0.27	2.74	3.46	0.06
E1	GY	5B	AX.89354035	6.32E+08	0.27	2.74	3.46	0.06
E1	GY	5B	AX.89327941	4.89E+08	0.06	-4.93	3.06	0.05
E1	GY	5B	AX.89327941	4.89E+08	0.06	-4.93	3.06	0.05
E1	GY	5B	AX.89538059	6.32E+08	0.27	2.74	3.46	0.06
E1	GY	5B	AX.89413887	1.97E+07	0.18	-3.25	3.58	0.05
E1	GY	5B	AX.89576361	6.36E+08	0.29	2.51	3.11	0.04
E1	GY	5B	AX.89503604	4.89E+08	0.05	-5.20	3.13	0.05
E1	GY	5B	AX.89542017	6.36E+08	0.31	2.87	4.05	0.06
E1	GY	5B	AX.89542017	6.36E+08	0.31	2.87	4.05	0.06
E1	GY	5B	AX.89350656	6.43E+08	0.10	-3.93	3.34	0.05
E1	GY	5B	AX.89358593	6.35E+08	0.28	2.62	3.28	0.05
E1	GY	5B	AX.86164400	4.69E+08	0.25	-2.61	3.06	0.05
E1	GY	5B	AX.89339124	5.86E+08	0.33	2.51	3.29	0.04
E1	GY	5B	AX.89594179	1.98E+07	0.18	-3.25	3.58	0.05
E1	GY	5B	AX.89764509	6.43E+08	0.11	-3.67	3.08	0.05
E1	GY	5B	AX.89682309	4.79E+08	0.05	-5.21	3.14	0.05
E1	GY	5B	AX.89456566	4.89E+08	0.06	-4.93	3.06	0.05
E1	GY	5B	AX.89367667	6.32E+08	0.24	2.80	3.33	0.06
E1	GY	5B	AX.89362470	4.79E+08	0.05	-5.21	3.14	0.05
E1	GY	5B	AX.89364126	4.79E+08	0.05	-5.21	3.14	0.05
E1	GY	5B	AX.89344695	6.35E+08	0.29	2.62	3.31	0.05
E1	GY	5B	AX.89309378	5.88E+08	0.27	-2.66	3.31	0.06
E1	GY	5B	AX.89318852	1.13E+07	0.17	-3.31	3.62	0.05
E1	GY	5B	AX.89387609	6.43E+08	0.11	-3.67	3.08	0.05
E1	GY	5B	AX.89387609	6.43E+08	0.11	-3.67	3.08	0.05
E1	GY	5B	AX.89364963	1.11E+07	0.17	-3.31	3.62	0.05
E1	GY	5B	AX.89529948	2.00E+07	0.29	-2.53	3.15	0.05
E1	GY	5B	AX.89530183	4.80E+08	0.05	-5.21	3.14	0.05
E1	GY	5B	AX.89392715	6.43E+08	0.11	-3.67	3.08	0.05
E1	GY	5B	AX.89544668	1.97E+07	0.18	-3.24	3.62	0.05
E1	GY	5B	AX.86169104	5.42E+08	0.11	3.73	3.25	0.06
E1	GY	5B	AX.89389198	6.32E+08	0.23	2.82	3.31	0.06
E1	GY	5B	AX.89624258	6.43E+08	0.11	-3.67	3.08	0.05
E1	GY	5B	AX.89342676	6.30E+08	0.23	2.90	3.51	0.06
E1	GY	5B	AX.89576361	6.36E+08	0.29	2.51	3.11	0.04
E1	GY	5B	AX.89662754	4.79E+08	0.05	-5.21	3.14	0.05
E1	GY	5B	AX.89727351	5.86E+08	0.33	2.51	3.29	0.04
E1	GY	5B	AX.89624706	6.34E+08	0.28	2.62	3.28	0.05
E1	GY	5B	AX.89437644	4.89E+08	0.05	-5.21	3.13	0.05
E1	GY	5B	AX.89406483	1.96E+07	0.18	-3.24	3.62	0.07
E1	GY	5B	AX.89332158	4.79E+08	0.05	-5.21	3.14	0.05
E1	GY	5B	AX.89531514	6.34E+08	0.28	2.62	3.28	0.05
E1	GY	5B	AX.89310027	4.79E+08	0.05	-5.21	3.14	0.05
E1	GY	5B	AX.89310027	4.79E+08	0.05	-5.21	3.14	0.05
E1	GY	5B	AX.89438140	4.79E+08	0.05	-5.21	3.14	0.05

E1	GY	5B	AX.89340138	6.30E+08	0.23	2.90	3.51	0.06
E1	GY	5B	AX.89704033	5.62E+08	0.27	2.58	3.10	0.05
E1	GY	5B	AX.89322373	5.86E+08	0.34	2.54	3.40	0.04
E1	GY	5B	AX.89508897	6.43E+08	0.11	-3.67	3.08	0.05
E1	GY	5B	AX.89426826	6.43E+08	0.11	-3.67	3.08	0.05
E1	GY	5B	AX.89755864	6.35E+08	0.28	2.62	3.28	0.05
E1	GY	5B	AX.89386836	5.87E+08	0.34	2.54	3.40	0.04
E1	GY	5B	AX.89582272	6.37E+08	0.34	2.45	3.19	0.05
E1	GY	5B	AX.89769947	6.37E+08	0.30	2.94	4.14	0.06
E1	GY	5B	AX.89400480	1.98E+07	0.18	-3.25	3.58	0.05
E1	GY	5B	AX.89704769	6.35E+08	0.28	2.62	3.28	0.05
E1	GY	5B	AX.89438995	2.00E+07	0.24	-2.79	3.31	0.05
E1	GY	5B	AX.89438995	2.00E+07	0.24	-2.79	3.31	0.05
E1	GY	5B	AX.89333600	6.36E+08	0.30	2.94	4.14	0.06
E1	GY	5B	AX.89428128	2.00E+07	0.19	-3.24	3.69	0.06
E1	GY	5B	AX.89647576	4.79E+08	0.05	-5.21	3.14	0.05
E1	GY	5B	AX.89652375	4.79E+08	0.05	-5.21	3.14	0.05
E1	GY	5B	AX.89497888	4.89E+08	0.05	-5.20	3.13	0.05
E1	GY	5B	AX.89369430	1.14E+07	0.17	-3.31	3.62	0.05
E1	GY	5B	AX.89311810	6.32E+08	0.27	2.67	3.35	0.06
E1	GY	5B	AX.89600742	5.61E+08	0.25	2.66	3.15	0.05
E1	GY	5B	AX.89726529	4.79E+08	0.05	-5.21	3.14	0.05
E1	GY	5B	AX.89440092	6.35E+08	0.28	2.62	3.28	0.05
E1	GY	5B	AX.89444392	6.37E+08	0.30	2.94	4.14	0.06
E1	GY	5B	AX.89673663	6.36E+08	0.34	2.45	3.19	0.05
E1	GY	5B	AX.89410418	4.79E+08	0.05	-5.21	3.14	0.05
E1	GY	5B	AX.89541991	6.34E+08	0.28	2.62	3.28	0.05
E1	GY	5B	AX.89541991	6.34E+08	0.28	2.62	3.28	0.05
E1	GY	5B	AX.89370717	7.04E+08	0.28	-2.76	3.61	0.06
E1	GY	5B	AX.89371209	6.37E+08	0.30	3.21	4.81	0.07
E1	GY	5B	AX.89394512	1.95E+07	0.19	-3.17	3.60	0.07
E1	GY	5B	AX.89764509	6.43E+08	0.11	-3.67	3.08	0.05
E1	GY	5B	AX.89654700	5.45E+08	0.13	3.37	3.07	0.05
E1	GY	5B	AX.89441811	1.98E+07	0.18	-3.25	3.58	0.05
E1	GY	5B	AX.89476252	1.98E+07	0.23	-2.70	3.09	0.06
E1	GY	5B	AX.89476252	1.98E+07	0.23	-2.70	3.09	0.06
E1	GY	5B	AX.89438140	4.79E+08	0.05	-5.21	3.14	0.05
E1	GY	5B	AX.89322373	5.86E+08	0.34	2.54	3.40	0.04
E1	GY	5B	AX.89563145	4.80E+08	0.05	-5.21	3.14	0.05
E1	GY	5B	AX.89547058	5.83E+08	0.27	2.65	3.23	0.05
E1	GY	5B	AX.89318852	1.13E+07	0.17	-3.31	3.62	0.05
E1	GY	5B	AX.89403758	6.31E+08	0.23	2.90	3.51	0.06
E1	GY	5B	AX.89380181	5.62E+08	0.32	2.61	3.45	0.05
E1	GY	5B	AX.89607893	3.73E+08	0.07	-4.48	3.26	0.05
E1	GY	5B	AX.89607893	3.73E+08	0.07	-4.48	3.26	0.05
E1	GY	5B	AX.89396386	1.14E+07	0.17	-3.31	3.62	0.05
E1	GY	5B	AX.89439777	2.00E+07	0.18	-3.25	3.58	0.05
E1	SPM2	5B	AX.89377670	1.32E+07	0.30	-19.51	3.58	0.06
E1	GY	5B	AX.89357144	6.30E+08	0.23	2.90	3.51	0.06
E1	GY	5B	AX.89327601	6.31E+08	0.23	2.90	3.51	0.06

E1	GY	5B	AX.89428861	5.87E+08	0.33	2.51	3.29	0.04
E1	SPM2	5B	AX.89741388	1.32E+07	0.32	-17.72	3.14	0.05
E1	GY	5B	AX.89338493	6.32E+08	0.27	2.81	3.65	0.07
E1	GY	5B	AX.89538059	6.32E+08	0.27	2.74	3.46	0.06
E1	GY	5B	AX.89385868	1.13E+07	0.17	-3.31	3.62	0.05
E1	GY	5B	AX.89350656	6.43E+08	0.10	-3.93	3.34	0.05
E1	GY	5B	AX.89371209	6.37E+08	0.30	3.21	4.81	0.07
E1	GY	5B	AX.89481691	4.79E+08	0.05	-5.21	3.14	0.05
E1	GY	5B	AX.89351668	4.89E+08	0.05	-5.20	3.13	0.05
E1	GY	5B	AX.89517273	6.43E+08	0.11	-3.67	3.08	0.05
E1	GY	5B	AX.86164400	4.69E+08	0.25	-2.61	3.06	0.05
E1	GY	5B	AX.89403758	6.31E+08	0.23	2.90	3.51	0.06
E1	GY	5B	AX.89536258	6.43E+08	0.11	-3.67	3.08	0.05
E1	GY	5B	AX.89447137	6.34E+08	0.28	2.69	3.41	0.05
E1	GY	5B	AX.89369103	1.95E+07	0.18	-3.25	3.58	0.05
E1	GY	5B	AX.89636730	6.26E+08	0.24	2.66	3.10	0.05
E1	GY	5B	AX.89701440	1.98E+07	0.18	-3.25	3.58	0.05
E1	GY	5B	AX.89474397	5.86E+08	0.33	2.51	3.29	0.04
E1	GY	5B	AX.89588192	5.86E+08	0.35	2.61	3.62	0.05
E1	GY	5B	AX.89734020	5.81E+08	0.24	2.67	3.12	0.03
E1	GY	5B	AX.89444392	6.37E+08	0.30	2.94	4.14	0.06
E1	GY	5B	AX.89757467	5.86E+08	0.34	2.54	3.40	0.04
E1	GY	5B	AX.86169104	5.42E+08	0.11	3.73	3.25	0.06
E1	SPM2	5B	AX.89333065	4.60E+08	0.20	-20.76	3.22	0.05
E1	GY	5B	AX.89685981	6.43E+08	0.11	-3.67	3.08	0.05
E1	GY	5B	AX.89685981	6.43E+08	0.11	-3.67	3.08	0.05
E1	GY	5B	AX.89649925	4.79E+08	0.05	-5.21	3.14	0.05
E1	GY	5B	AX.89471949	1.95E+07	0.18	-3.25	3.58	0.05
E1	GY	5B	AX.89367249	4.79E+08	0.05	-5.21	3.14	0.05
E1	GY	5B	AX.89367299	5.86E+08	0.34	2.54	3.40	0.04
E1	GY	5B	AX.89367299	5.86E+08	0.34	2.54	3.40	0.04
E1	GY	5B	AX.89554341	4.79E+08	0.05	-5.21	3.14	0.05
E1	GY	5B	AX.89554341	4.79E+08	0.05	-5.21	3.14	0.05
E1	GY	5B	AX.89662754	4.79E+08	0.05	-5.21	3.14	0.05
E1	GY	5B	AX.89355657	5.60E+08	0.25	2.66	3.15	0.05
E1	GY	5B	AX.89508910	6.32E+08	0.23	2.90	3.51	0.06
E1	GY	5B	AX.89379647	6.35E+08	0.28	2.62	3.28	0.05
E1	GY	5B	AX.89532223	6.32E+08	0.27	2.74	3.46	0.06
E1	GY	5B	AX.89415477	6.31E+08	0.23	2.90	3.51	0.06
E1	GY	5B	AX.89344695	6.35E+08	0.29	2.62	3.31	0.05
E1	GY	5B	AX.89400480	1.98E+07	0.18	-3.25	3.58	0.05
E1	GY	5B	AX.89380181	5.62E+08	0.32	2.61	3.45	0.05
E1	GY	5B	AX.89749327	5.87E+08	0.33	2.43	3.09	0.04
E1	GY	5B	AX.89474397	5.86E+08	0.33	2.51	3.29	0.04
E1	GY	5B	AX.89615514	1.98E+07	0.18	-3.25	3.58	0.05
E1	GY	5B	AX.89357028	2.00E+07	0.18	-3.25	3.58	0.05
E1	GY	5B	AX.89734020	5.81E+08	0.24	2.67	3.12	0.03
E1	GY	5B	AX.89413298	6.30E+08	0.23	2.90	3.51	0.06
E1	GY	5B	AX.89544668	1.97E+07	0.18	-3.24	3.62	0.05
E1	GY	5B	AX.89552577	4.79E+08	0.05	-5.21	3.14	0.05

E1	GY	5B	AX.89653121	2.00E+07	0.19	-3.33	3.92	0.06
E1	GY	5B	AX.89746558	4.79E+08	0.05	-5.21	3.14	0.05
E1	GY	5B	AX.89381160	6.93E+08	0.06	-4.93	3.26	0.07
E1	GY	5B	AX.86169102	5.42E+08	0.11	3.65	3.04	0.05
E1	GY	5B	AX.89405592	5.61E+08	0.25	2.66	3.15	0.05
E1	GY	5B	AX.89381592	4.80E+08	0.05	-5.21	3.14	0.05
E1	GY	5B	AX.89381592	4.80E+08	0.05	-5.21	3.14	0.05
E1	GY	5B	AX.89346742	6.31E+08	0.23	2.90	3.51	0.06
E1	GY	5B	AX.89546059	1.15E+07	0.18	-3.14	3.38	0.05
E1	GY	5B	AX.89313525	6.32E+08	0.27	2.74	3.46	0.06
E1	GY	5B	AX.89485770	1.13E+07	0.17	-3.31	3.62	0.05
E1	GY	5B	AX.89654700	5.45E+08	0.13	3.37	3.07	0.05
E1	GY	5B	AX.89641721	6.42E+08	0.11	-3.67	3.08	0.05
E1	GY	5B	AX.89382361	7.13E+08	0.08	-4.29	3.18	0.05
E1	GY	5B	AX.89382361	7.13E+08	0.08	-4.29	3.18	0.05
E1	GY	5B	AX.89590734	5.61E+08	0.25	2.66	3.15	0.05
E1	GY	5B	AX.89667390	1.97E+07	0.18	-3.25	3.58	0.05
E1	GY	5B	AX.89336425	6.29E+08	0.24	2.92	3.59	0.06
E1	GY	5B	AX.89594998	7.03E+08	0.28	-2.64	3.30	0.06
E1	GY	5B	AX.89547058	5.83E+08	0.27	2.65	3.23	0.05
E1	GY	5B	AX.89591274	4.55E+08	0.23	-2.80	3.26	0.06
E1	GY	5B	AX.89591274	4.55E+08	0.23	-2.80	3.26	0.06
E1	GY	5B	AX.89603441	6.31E+08	0.23	2.90	3.51	0.06
E1	GY	5B	AX.89454130	6.38E+08	0.49	-2.27	3.08	0.05
E1	GY	5B	AX.89617672	8.24E+06	0.13	-3.37	3.08	0.05
E1	GY	5B	AX.89383487	6.43E+08	0.11	-3.67	3.08	0.05
E1	GY	5B	AX.89766465	4.79E+08	0.05	-5.21	3.14	0.05
E1	GY	5B	AX.89619699	6.32E+08	0.28	2.53	3.08	0.05
E1	GY	5B	AX.89619699	6.32E+08	0.28	2.53	3.08	0.05
E1	GY	5B	AX.89619706	5.62E+08	0.33	2.49	3.23	0.05
E1	GY	5B	AX.89439777	2.00E+07	0.18	-3.25	3.58	0.05
E1	GY	5B	AX.89652622	7.04E+06	0.39	2.40	3.24	0.05
E1	SPM2	5B	AX.89423877	6.65E+06	0.20	-20.73	3.16	0.05
E1	GY	5B	AX.89730035	5.42E+08	0.11	3.73	3.25	0.06
E1	GY	5B	AX.89542131	6.36E+08	0.27	2.99	4.06	0.06
E1	GY	5B	AX.89428861	5.87E+08	0.33	2.51	3.29	0.04
E1	GY	5B	AX.89393248	6.65E+06	0.40	2.30	3.05	0.04
E1	GY	5B	AX.89503604	4.89E+08	0.05	-5.20	3.13	0.05
E1	GY	5B	AX.89456230	6.35E+08	0.29	2.57	3.21	0.05
E1	GY	5B	AX.89504076	7.03E+08	0.07	-4.77	3.45	0.07
E1	GY	5B	AX.89621509	4.55E+08	0.23	-2.80	3.26	0.06
E1	GY	5B	AX.89481691	4.79E+08	0.05	-5.21	3.14	0.05
E1	GY	5B	AX.89351668	4.89E+08	0.05	-5.20	3.13	0.05
E1	GY	5B	AX.89680483	4.80E+08	0.05	-5.21	3.14	0.05
E1	GY	5B	AX.89622545	6.36E+08	0.34	2.45	3.19	0.05
E1	GY	5B	AX.89769947	6.37E+08	0.30	2.94	4.14	0.06
E1	GY	5B	AX.89679650	5.86E+08	0.33	2.51	3.29	0.04
E1	GY	5B	AX.89679650	5.86E+08	0.33	2.51	3.29	0.04
E1	GY	5B	AX.89672140	6.35E+08	0.28	2.62	3.28	0.05
E1	GY	5B	AX.89319162	6.36E+08	0.33	2.70	3.75	0.06

E1	GY	5B	AX.89435584	4.79E+08	0.05	-5.21	3.14	0.05
E1	GY	5B	AX.89659582	6.34E+08	0.28	2.62	3.28	0.05
E1	GY	5B	AX.89447137	6.34E+08	0.28	2.69	3.41	0.05
E1	GY	5B	AX.89587535	6.36E+08	0.34	2.45	3.19	0.05
E1	GY	5B	AX.89587535	6.36E+08	0.34	2.45	3.19	0.05
E1	GY	5B	AX.89435827	6.34E+08	0.28	2.62	3.28	0.05
E1	GY	5B	AX.89672663	6.36E+08	0.34	2.45	3.19	0.05
E1	GY	5B	AX.89672663	6.36E+08	0.34	2.45	3.19	0.05
E1	GY	5B	AX.89575415	6.93E+08	0.06	-4.93	3.26	0.07
E1	GY	5B	AX.89319628	2.00E+07	0.18	-3.25	3.58	0.05
E1	GY	5B	AX.89447653	6.32E+08	0.23	2.90	3.51	0.06
E1	GY	5B	AX.89737044	1.13E+07	0.17	-3.31	3.62	0.05
E1	GY	5B	AX.89737044	1.13E+07	0.17	-3.31	3.62	0.05
E1	GY	5B	AX.89619826	4.79E+08	0.05	-5.21	3.14	0.05
E1	GY	5B	AX.89757467	5.86E+08	0.34	2.54	3.40	0.04
E1	GY	5B	AX.89714869	4.79E+08	0.05	-5.21	3.14	0.05
E1	GY	5B	AX.89516752	1.97E+07	0.18	-3.25	3.58	0.05
E1	GY	5B	AX.89662514	4.79E+08	0.05	-5.21	3.14	0.05
E1	GY	5B	AX.89654057	4.80E+08	0.05	-5.21	3.14	0.05
E1	GY	5B	AX.89685979	6.32E+08	0.27	2.74	3.46	0.06
E1	GY	5B	AX.89601860	7.03E+08	0.06	-5.33	3.49	0.07
E1	GY	5B	AX.89554094	5.87E+08	0.31	2.47	3.09	0.04
E1	GY	5B	AX.89508897	6.43E+08	0.11	-3.67	3.08	0.05
E1	GY	5B	AX.89675642	4.80E+08	0.05	-5.21	3.14	0.05
E1	GY	5B	AX.89415477	6.31E+08	0.23	2.90	3.51	0.06
E1	GY	5B	AX.89460759	4.79E+08	0.05	-5.21	3.14	0.05
E1	GY	5B	AX.89460759	4.79E+08	0.05	-5.21	3.14	0.05
E1	GY	5B	AX.89567072	1.14E+07	0.17	-3.31	3.62	0.05
E1	GY	5B	AX.89638418	4.79E+08	0.05	-5.21	3.14	0.05
E1	GY	5B	AX.89638729	1.15E+07	0.17	-3.31	3.62	0.05
E1	GY	5B	AX.89638729	1.15E+07	0.17	-3.31	3.62	0.05
E1	GY	5B	AX.89428128	2.00E+07	0.19	-3.24	3.69	0.06
E1	GY	5B	AX.89603853	6.32E+08	0.23	2.90	3.51	0.06
E1	GY	5B	AX.89603853	6.32E+08	0.23	2.90	3.51	0.06
E1	GY	5B	AX.89664468	4.79E+08	0.05	-5.21	3.14	0.05
E1	GY	5B	AX.89664468	4.79E+08	0.05	-5.21	3.14	0.05
E1	GY	5B	AX.89652375	4.79E+08	0.05	-5.21	3.14	0.05
E1	GY	5B	AX.89615514	1.98E+07	0.18	-3.25	3.58	0.05
E1	GY	5B	AX.89497888	4.89E+08	0.05	-5.20	3.13	0.05
E1	GY	5B	AX.89533286	5.87E+08	0.13	-3.67	3.65	0.07
E1	GY	5B	AX.89311810	6.32E+08	0.27	2.67	3.35	0.06
E1	GY	5B	AX.89702097	6.35E+08	0.28	2.62	3.28	0.05
E1	GY	5B	AX.89738550	6.37E+08	0.34	2.45	3.19	0.05
E1	GY	5B	AX.89494884	5.60E+08	0.25	2.66	3.15	0.05
E1	GY	5B	AX.89628012	4.79E+08	0.05	-5.21	3.14	0.05
E1	GY	5B	AX.89443122	5.87E+08	0.33	2.51	3.29	0.04
E1	GY	5B	AX.89462733	1.95E+07	0.19	-3.21	3.68	0.06
E1	GY	5B	AX.89429757	5.05E+07	0.47	-2.26	3.04	0.06
E1	GY	5B	AX.89429757	5.05E+07	0.47	-2.26	3.04	0.06
E1	GY	5B	AX.89605744	6.36E+08	0.30	2.94	4.14	0.06

E1	GY	5B	AX.89358593	6.35E+08	0.28	2.62	3.28	0.05
E1	GY	5B	AX.89617380	5.87E+08	0.33	2.45	3.15	0.04
E1	GY	5B	AX.89617380	5.87E+08	0.33	2.45	3.15	0.04
E1	GY	5B	AX.89546059	1.15E+07	0.18	-3.14	3.38	0.05
E1	GY	5B	AX.89371307	5.86E+08	0.33	2.52	3.31	0.04
E1	GY	5B	AX.89678376	2.00E+07	0.18	-3.14	3.43	0.05
E1	GY	5B	AX.89325602	6.93E+08	0.25	-2.85	3.51	0.06
E1	GY	5B	AX.89675139	6.32E+08	0.27	2.74	3.46	0.06
E1	GY	5B	AX.89359864	5.61E+08	0.25	2.66	3.15	0.05
E1	GY	5B	AX.89359864	5.61E+08	0.25	2.66	3.15	0.05
E1	GY	5B	AX.89336425	6.29E+08	0.24	2.92	3.59	0.06
E1	GY	5B	AX.89419340	6.43E+08	0.11	-3.72	3.15	0.05
E1	GY	5B	AX.89630366	7.04E+08	0.07	-4.78	3.46	0.07
E1	GY	5B	AX.89547536	4.79E+08	0.05	-5.21	3.14	0.05
E1	GY	5B	AX.89501084	1.97E+07	0.18	-3.25	3.58	0.05
E1	GY	5B	AX.89676214	6.37E+08	0.30	2.94	4.14	0.06
E1	GY	5B	AX.89630809	4.80E+08	0.05	-5.21	3.14	0.05
E1	GY	5B	AX.89396229	6.32E+08	0.27	2.67	3.35	0.06
E1	GY	5B	AX.89396386	1.14E+07	0.17	-3.31	3.62	0.05
E1	GY	5B	AX.89771453	4.79E+08	0.05	-5.21	3.14	0.05
E1	GY	5B	AX.89466410	6.43E+08	0.11	-3.67	3.08	0.05
E1	GY	5B	AX.89705205	5.83E+08	0.40	2.54	3.58	0.06
E1	GY	5B	AX.89537992	1.95E+07	0.17	-3.24	3.49	0.05
E1	GY	5B	AX.89463041	4.66E+08	0.06	-4.72	3.02	0.05
E1	GY	5B	AX.89328144	4.79E+08	0.05	-5.21	3.14	0.05
E1	GY	5B	AX.89524656	6.43E+08	0.11	-3.67	3.08	0.05
E1	GY	5B	AX.89768576	4.79E+08	0.05	-5.21	3.14	0.05
E1	GY	5B	AX.89682309	4.79E+08	0.05	-5.21	3.14	0.05
E1	GY	5B	AX.89573412	6.36E+08	0.34	2.45	3.19	0.05
E1	GY	5B	AX.89516768	4.79E+08	0.05	-5.21	3.14	0.05
E1	GY	5B	AX.89744337	6.37E+08	0.30	2.94	4.14	0.06
E1	GY	5B	AX.89744337	6.37E+08	0.30	2.94	4.14	0.06
E1	GY	5B	AX.89563145	4.80E+08	0.05	-5.21	3.14	0.05
E1	GY	5B	AX.89574029	5.86E+08	0.33	2.42	3.07	0.04
E1	GY	5B	AX.89574029	5.86E+08	0.33	2.42	3.07	0.04
E1	GY	5B	AX.89505018	4.79E+08	0.05	-5.21	3.14	0.05
E1	GY	5B	AX.89329613	1.97E+07	0.18	-3.25	3.58	0.05
E1	GY	5B	AX.89672140	6.35E+08	0.28	2.62	3.28	0.05
E1	GY	5B	AX.89319162	6.36E+08	0.33	2.70	3.75	0.06
E1	GY	5B	AX.89630915	5.62E+08	0.33	2.49	3.23	0.05
E1	GY	5B	AX.89364963	1.11E+07	0.17	-3.31	3.62	0.05
E1	GY	5B	AX.89388133	6.32E+08	0.27	2.74	3.46	0.06
E1	GY	5B	AX.89600633	1.15E+07	0.18	-3.14	3.38	0.05
E1	GY	5B	AX.89714869	4.79E+08	0.05	-5.21	3.14	0.05
E1	GY	5B	AX.89636584	6.31E+08	0.23	2.90	3.51	0.06
E1	GY	5B	AX.89437256	6.32E+08	0.23	2.90	3.51	0.06
E1	GY	5B	AX.89331656	6.32E+08	0.27	2.74	3.46	0.06
E1	GY	5B	AX.89623198	4.89E+08	0.05	-5.20	3.13	0.05
E1	GY	5B	AX.89437564	6.43E+08	0.11	-3.67	3.08	0.05
E1	GY	5B	AX.89437564	6.43E+08	0.11	-3.67	3.08	0.05

E1	GY	5B	AX.89354499	1.98E+07	0.18	-3.25	3.58	0.05
E1	GY	5B	AX.89354499	1.98E+07	0.18	-3.25	3.58	0.05
E1	SPM2	5B	AX.89439896	1.32E+07	0.29	-19.09	3.38	0.06
E1	GY	5B	AX.89711876	1.95E+07	0.18	-3.25	3.58	0.05
E1	GY	5B	AX.89542641	4.79E+08	0.05	-5.21	3.14	0.05
E1	GY	5B	AX.89542641	4.79E+08	0.05	-5.21	3.14	0.05
E1	GY	5B	AX.89496756	6.36E+08	0.34	2.45	3.19	0.05
E1	GY	5B	AX.89446263	6.37E+08	0.25	2.89	3.65	0.05
E1	GY	5B	AX.89614292	4.71E+08	0.28	-2.72	3.48	0.06
E1	GY	5B	AX.89614292	4.71E+08	0.28	-2.72	3.48	0.06
E1	GY	5B	AX.89461025	6.30E+08	0.23	2.90	3.51	0.06
E1	GY	5B	AX.89603441	6.31E+08	0.23	2.90	3.51	0.06
E1	GY	5B	AX.89543790	6.99E+06	0.44	2.27	3.03	0.05
E1	GY	5B	AX.89439094	5.60E+08	0.25	2.66	3.15	0.05
E1	GY	5B	AX.89333600	6.36E+08	0.30	2.94	4.14	0.06
E1	GY	5B	AX.89647576	4.79E+08	0.05	-5.21	3.14	0.05
E1	GY	5B	AX.89724585	2.16E+07	0.11	-3.74	3.27	0.05
E1	GY	5B	AX.89724585	2.16E+07	0.11	-3.74	3.27	0.05
E1	GY	5B	AX.89725988	1.98E+07	0.18	-3.25	3.58	0.05
E1	GY	5B	AX.89369430	1.14E+07	0.17	-3.31	3.62	0.05
E1	GY	5B	AX.89357144	6.30E+08	0.23	2.90	3.51	0.06
E1	GY	5B	AX.89702226	6.37E+08	0.34	2.65	3.65	0.05
E1	GY	5B	AX.89714399	1.15E+07	0.18	-3.14	3.38	0.05
E1	GY	5B	AX.89474866	4.79E+08	0.05	-5.21	3.14	0.05
E1	GY	5B	AX.89474866	4.79E+08	0.05	-5.21	3.14	0.05
E1	GY	5B	AX.89447968	4.79E+08	0.05	-5.21	3.14	0.05
E1	GY	5B	AX.89544766	5.60E+08	0.25	2.66	3.15	0.05
E1	GY	5B	AX.89498476	6.36E+08	0.30	2.94	4.14	0.06
E1	GY	5B	AX.89628012	4.79E+08	0.05	-5.21	3.14	0.05
E1	GY	5B	AX.89631907	6.36E+08	0.33	2.70	3.71	0.06
E1	GY	5B	AX.89405592	5.61E+08	0.25	2.66	3.15	0.05
E1	GY	5B	AX.89605744	6.36E+08	0.30	2.94	4.14	0.06
E1	GY	5B	AX.89727351	5.86E+08	0.33	2.51	3.29	0.04
E1	GY	5B	AX.89606400	6.31E+08	0.23	2.90	3.51	0.06
E1	GY	5B	AX.89608793	6.43E+08	0.11	-3.67	3.08	0.05
E1	GY	5B	AX.89678376	2.00E+07	0.18	-3.14	3.43	0.05
E1	GY	5B	AX.89394512	1.95E+07	0.19	-3.17	3.60	0.07
E1	GY	5B	AX.89325602	6.93E+08	0.25	-2.85	3.51	0.06
E1	GY	5B	AX.89641721	6.42E+08	0.11	-3.67	3.08	0.05
E1	GY	5B	AX.89675252	5.61E+08	0.25	2.66	3.15	0.05
E1	GY	5B	AX.89675252	5.61E+08	0.25	2.66	3.15	0.05
E1	GY	5B	AX.89476578	5.60E+08	0.25	2.66	3.15	0.05
E1	GY	5B	AX.89476578	5.60E+08	0.25	2.66	3.15	0.05
E1	GY	5B	AX.89371830	6.32E+08	0.27	2.74	3.46	0.06
E1	GY	5B	AX.89464183	6.36E+08	0.34	2.45	3.19	0.05
E1	GY	5B	AX.89476724	5.86E+08	0.33	2.45	3.15	0.04
E1	GY	5B	AX.89476724	5.86E+08	0.33	2.45	3.15	0.04
E1	GY	5B	AX.89655262	7.03E+08	0.29	-2.58	3.26	0.06
E1	GY	5B	AX.89594849	6.34E+08	0.28	2.62	3.28	0.05
E1	GY	5B	AX.89536172	1.98E+07	0.18	-3.14	3.43	0.05

E1	SPM2	5B	AX.89489374	1.32E+07	0.29	-19.88	3.63	0.06
E1	GY	5B	AX.89582777	4.79E+08	0.05	-5.21	3.14	0.05
E1	SPM2	5B	AX.89311748	1.33E+07	0.33	-18.37	3.36	0.06
E1	GY	5B	AX.89372325	6.34E+08	0.28	2.62	3.28	0.05
E1	GY	5B	AX.89501286	7.43E+07	0.47	2.31	3.16	0.06
E1	GY	5B	AX.89571058	1.95E+07	0.17	-3.18	3.39	0.05
E1	GPS	5B	AX.89377670	1.32E+07	0.30	1.57	4.74	0.09
E1	GY	5B	AX.89396229	6.32E+08	0.27	2.67	3.35	0.06
E1	GY	5B	AX.89337552	5.87E+08	0.27	-2.62	3.20	0.06
E1	GY	5B	AX.89619706	5.62E+08	0.33	2.49	3.23	0.05
E1	GY	5B	AX.89718577	5.86E+08	0.34	2.54	3.40	0.04
E1	GPS	5B	AX.89503604	4.89E+08	0.05	-2.55	3.09	0.05
E1	GY	5B	AX.89421217	1.13E+07	0.17	-3.31	3.62	0.05
E1	GY	5B	AX.89644485	1.98E+07	0.18	-3.25	3.58	0.05
E1	GY	5B	AX.89669309	4.79E+08	0.05	-5.21	3.14	0.05
E1	GY	5B	AX.89338493	6.32E+08	0.27	2.81	3.65	0.07
E1	GY	5B	AX.89731133	6.35E+08	0.28	2.62	3.28	0.05
E1	GY	5B	AX.89632393	5.86E+08	0.34	2.54	3.40	0.04
E1	GY	5B	AX.89338561	4.43E+08	0.44	2.64	3.95	0.07
E1	GY	5B	AX.89759768	5.62E+08	0.33	2.45	3.14	0.05
E1	GY	5B	AX.89609532	4.79E+08	0.05	-5.21	3.14	0.05
E1	GY	5B	AX.89609532	4.79E+08	0.05	-5.21	3.14	0.05
E1	GY	5B	AX.89703320	5.86E+08	0.33	2.51	3.29	0.04
E1	GY	5B	AX.89703320	5.86E+08	0.33	2.51	3.29	0.04
E1	GY	5B	AX.89535300	5.86E+08	0.34	2.54	3.40	0.04
E1	GY	5B	AX.89535300	5.86E+08	0.34	2.54	3.40	0.04
E1	GY	5B	AX.89364126	4.79E+08	0.05	-5.21	3.14	0.05
E1	GY	5B	AX.89422733	6.35E+08	0.28	2.62	3.28	0.05
E1	GY	5B	AX.89577810	5.81E+08	0.24	2.67	3.12	0.03
E1	GY	5B	AX.89586465	6.34E+08	0.28	2.62	3.28	0.05
E1	GY	5B	AX.89586465	6.34E+08	0.28	2.62	3.28	0.05
E1	GPS	5B	AX.89454031	1.32E+07	0.30	1.57	4.74	0.09
E1	GY	5B	AX.89505018	4.79E+08	0.05	-5.21	3.14	0.05
E1	GY	5B	AX.89708766	4.79E+08	0.05	-5.21	3.14	0.05
E1	GY	5B	AX.89704982	4.80E+08	0.05	-5.21	3.14	0.05
E1	GY	5B	AX.89704982	4.80E+08	0.05	-5.21	3.14	0.05
E1	GY	5B	AX.89659582	6.34E+08	0.28	2.62	3.28	0.05
E1	GY	5B	AX.89650284	6.34E+08	0.28	2.62	3.28	0.05
E1	GY	5B	AX.89692990	6.43E+08	0.11	-3.67	3.08	0.05
E1	GY	5B	AX.89692990	6.43E+08	0.11	-3.67	3.08	0.05
E1	GY	5B	AX.89435827	6.34E+08	0.28	2.62	3.28	0.05
E1	GY	5B	AX.89447298	2.00E+07	0.18	-3.25	3.58	0.05
E1	GY	5B	AX.89636730	6.26E+08	0.24	2.66	3.10	0.05
E1	GY	5B	AX.89575415	6.93E+08	0.06	-4.93	3.26	0.07
E1	GY	5B	AX.89447653	6.32E+08	0.23	2.90	3.51	0.06
E1	GY	5B	AX.89564342	1.96E+07	0.18	-3.25	3.58	0.05
E1	GY	5B	AX.89564342	1.96E+07	0.18	-3.25	3.58	0.05
E1	GY	5B	AX.89448319	6.37E+08	0.30	2.94	4.14	0.06
E1	GPS	5B	AX.89543476	6.85E+08	0.07	-2.37	3.32	0.06
E1	GY	5B	AX.89636584	6.31E+08	0.23	2.90	3.51	0.06

E1	GPS	5B	AX.89392333	6.85E+08	0.06	-2.37	3.12	0.05
E1	GY	5B	AX.89636160	4.89E+08	0.05	-5.20	3.13	0.05
E1	GY	5B	AX.89636160	4.89E+08	0.05	-5.20	3.13	0.05
E1	GY	5B	AX.89367249	4.79E+08	0.05	-5.21	3.14	0.05
E1	GY	5B	AX.89650371	4.79E+08	0.05	-5.21	3.14	0.05
E1	GY	5B	AX.89650371	4.79E+08	0.05	-5.21	3.14	0.05
E1	GY	5B	AX.89367667	6.32E+08	0.24	2.80	3.33	0.06
E1	GY	5B	AX.89598921	6.32E+08	0.26	2.75	3.42	0.06
E1	GY	5B	AX.89598921	6.32E+08	0.26	2.75	3.42	0.06
E1	GY	5B	AX.89426826	6.43E+08	0.11	-3.67	3.08	0.05
E1	GY	5B	AX.89653910	5.81E+08	0.24	2.67	3.12	0.03
E1	GY	5B	AX.89379647	6.35E+08	0.28	2.62	3.28	0.05
E1	GPS	5B	AX.89510887	1.32E+07	0.29	1.48	4.18	0.07
E1	GY	5B	AX.89500758	4.79E+08	0.05	-5.21	3.14	0.05
E1	GY	5B	AX.89500758	4.79E+08	0.05	-5.21	3.14	0.05
E1	GY	5B	AX.89582272	6.37E+08	0.34	2.45	3.19	0.05
E1	GY	5B	AX.89774767	6.37E+08	0.34	2.45	3.19	0.05
E1	GY	5B	AX.89510136	6.32E+08	0.27	2.59	3.15	0.06
E1	GY	5B	AX.89641096	4.79E+08	0.05	-5.21	3.14	0.05
E1	GY	5B	AX.89587402	6.43E+08	0.11	-3.67	3.08	0.05
E1	GY	5B	AX.89369103	1.95E+07	0.18	-3.25	3.58	0.05
E1	GY	5B	AX.89749327	5.87E+08	0.33	2.43	3.09	0.04
E1	GY	5B	AX.89649133	4.80E+08	0.05	-5.21	3.14	0.05
E1	GY	5B	AX.89639321	6.35E+08	0.28	2.62	3.28	0.05
E1	GY	5B	AX.89474842	4.79E+08	0.05	-5.21	3.14	0.05
E1	GY	5B	AX.89474842	4.79E+08	0.05	-5.21	3.14	0.05
E1	GY	5B	AX.89702287	6.20E+08	0.24	3.04	3.89	0.07
E1	GY	5B	AX.89447968	4.79E+08	0.05	-5.21	3.14	0.05
E1	GY	5B	AX.89648556	6.32E+08	0.27	2.74	3.46	0.06
E1	GY	5B	AX.89475197	5.81E+08	0.24	2.67	3.12	0.03
E1	GY	5B	AX.89653121	2.00E+07	0.19	-3.33	3.92	0.06
E1	GPS	5B	AX.89734563	6.85E+08	0.06	-2.37	3.12	0.05
E1	GPS	5B	AX.89600504	6.86E+08	0.06	-2.59	3.40	0.06
E1	GY	5B	AX.89655934	6.36E+08	0.34	2.45	3.19	0.05
E1	GPS	5B	AX.89479246	6.86E+08	0.06	-2.59	3.40	0.06
E1	GY	5B	AX.89346742	6.31E+08	0.23	2.90	3.51	0.06
E1	GY	5B	AX.89606400	6.31E+08	0.23	2.90	3.51	0.06
E1	GY	5B	AX.89371307	5.86E+08	0.33	2.52	3.31	0.04
E1	GY	5B	AX.89313525	6.32E+08	0.27	2.74	3.46	0.06
E1	GY	5B	AX.89594179	1.98E+07	0.18	-3.25	3.58	0.05
E1	GY	5B	AX.89716468	6.43E+08	0.11	-3.67	3.08	0.05
E1	GY	5B	AX.89755864	6.35E+08	0.28	2.62	3.28	0.05
E1	GY	5B	AX.89594849	6.34E+08	0.28	2.62	3.28	0.05
E1	GY	5B	AX.89679245	4.80E+08	0.05	-5.21	3.14	0.05
E1	GY	5B	AX.89547536	4.79E+08	0.05	-5.21	3.14	0.05
E1	GY	5B	AX.89617672	8.24E+06	0.13	-3.37	3.08	0.05
E1	GY	5B	AX.89676214	6.37E+08	0.30	2.94	4.14	0.06
E1	GY	5B	AX.89638955	7.03E+08	0.28	-3.27	4.80	0.09
E1	GY	5B	AX.89515226	6.35E+08	0.28	2.62	3.28	0.05
E1	GY	5B	AX.89532955	1.97E+07	0.18	-3.25	3.58	0.05

E1	GY	5B	AX.89766465	4.79E+08	0.05	-5.21	3.14	0.05
E1	SPM2	5B	AX.89772432	1.32E+07	0.30	-19.51	3.58	0.06
E1	GY	5B	AX.89599482	6.35E+08	0.28	2.62	3.28	0.05
E1	GY	5B	AX.89599482	6.35E+08	0.28	2.62	3.28	0.05
E1	GY	5B	AX.89652622	7.04E+06	0.39	2.40	3.24	0.05
E1	GY	5B	AX.89514611	6.37E+08	0.34	2.45	3.19	0.05
E1	GY	5B	AX.89774719	6.35E+08	0.29	2.57	3.21	0.05
E1	GY	5B	AX.89421217	1.13E+07	0.17	-3.31	3.62	0.05
E1	GY	5B	AX.89350220	4.89E+08	0.05	-5.20	3.13	0.05
E1	GY	5B	AX.89350220	4.89E+08	0.05	-5.20	3.13	0.05
E1	GY	5B	AX.89338561	4.43E+08	0.44	2.64	3.95	0.07
E1	GY	5B	AX.89561097	5.62E+08	0.27	2.62	3.21	0.05
E1	GY	5B	AX.89671443	4.80E+08	0.05	-5.21	3.14	0.05
E1	GY	5B	AX.89671443	4.80E+08	0.05	-5.21	3.14	0.05
E1	GY	5B	AX.89524656	6.43E+08	0.11	-3.67	3.08	0.05
E1	GY	5B	AX.89339124	5.86E+08	0.33	2.51	3.29	0.04
E1	GY	5B	AX.89768576	4.79E+08	0.05	-5.21	3.14	0.05
E1	GY	5B	AX.89678549	1.98E+07	0.18	-3.24	3.62	0.05
E1	GY	5B	AX.89577810	5.81E+08	0.24	2.67	3.12	0.03
E1	GY	5B	AX.89622545	6.36E+08	0.34	2.45	3.19	0.05
E1	GY	5B	AX.89622171	1.11E+07	0.17	-3.31	3.62	0.05
E1	GY	5B	AX.89708766	4.79E+08	0.05	-5.21	3.14	0.05
E1	GY	5B	AX.89518064	4.79E+08	0.05	-5.21	3.14	0.05
E1	GY	5B	AX.89709484	6.31E+08	0.23	2.90	3.51	0.06
E1	GY	5B	AX.89709484	6.31E+08	0.23	2.90	3.51	0.06
E1	GY	5B	AX.89376899	5.87E+08	0.27	-2.66	3.31	0.06
E1	GY	5B	AX.89319628	2.00E+07	0.18	-3.25	3.58	0.05
E1	GY	5B	AX.89388133	6.32E+08	0.27	2.74	3.46	0.06
E1	GY	5B	AX.89744387	4.64E+08	0.31	-2.59	3.37	0.06
E1	GY	5B	AX.89662514	4.79E+08	0.05	-5.21	3.14	0.05
E1	GY	5B	AX.89720865	4.79E+08	0.05	-5.21	3.14	0.05
E1	GY	5B	AX.89654057	4.80E+08	0.05	-5.21	3.14	0.05
E1	GY	5B	AX.89615841	2.00E+07	0.18	-3.25	3.58	0.05
E1	GY	5B	AX.89615841	2.00E+07	0.18	-3.25	3.58	0.05
E1	GY	5B	AX.89413887	1.97E+07	0.18	-3.25	3.58	0.05
E1	GY	5B	AX.89689229	6.37E+08	0.30	3.21	4.81	0.07
E1	GY	5B	AX.89389594	6.36E+08	0.34	2.45	3.19	0.05
E1	GY	5B	AX.89772404	4.79E+08	0.05	-5.21	3.14	0.05
E1	GY	5B	AX.89772404	4.79E+08	0.05	-5.21	3.14	0.05
E1	GY	5B	AX.89601860	7.03E+08	0.06	-5.33	3.49	0.07
E1	GY	5B	AX.89651787	4.89E+08	0.05	-5.20	3.13	0.05
E1	GPS	5B	AX.89403059	6.85E+08	0.06	-2.37	3.12	0.05
E1	GY	5B	AX.89680483	4.80E+08	0.05	-5.21	3.14	0.05
E1	GY	5B	AX.89711876	1.95E+07	0.18	-3.25	3.58	0.05
E1	GY	5B	AX.89531514	6.34E+08	0.28	2.62	3.28	0.05
E1	GY	5B	AX.89486250	6.43E+08	0.11	-3.67	3.08	0.05
E1	GY	5B	AX.89355657	5.60E+08	0.25	2.66	3.15	0.05
E1	GY	5B	AX.89675642	4.80E+08	0.05	-5.21	3.14	0.05
E1	GY	5B	AX.89461025	6.30E+08	0.23	2.90	3.51	0.06
E1	GY	5B	AX.89450464	5.60E+08	0.25	2.66	3.15	0.05

E1	GY	5B	AX.89486782	6.32E+08	0.23	2.90	3.51	0.06
E1	GY	5B	AX.89510136	6.32E+08	0.27	2.59	3.15	0.06
E1	GY	5B	AX.89392375	6.43E+08	0.11	-3.67	3.08	0.05
E1	GY	5B	AX.89649133	4.80E+08	0.05	-5.21	3.14	0.05
E1	GY	5B	AX.89725988	1.98E+07	0.18	-3.25	3.58	0.05
E1	GY	5B	AX.89439783	6.36E+08	0.34	2.65	3.65	0.05
E1	GY	5B	AX.89439783	6.36E+08	0.34	2.65	3.65	0.05
E1	GY	5B	AX.89639321	6.35E+08	0.28	2.62	3.28	0.05
E1	GY	5B	AX.89702226	6.37E+08	0.34	2.65	3.65	0.05
E1	GY	5B	AX.89702244	6.37E+08	0.30	2.94	4.14	0.06
E1	GY	5B	AX.89494884	5.60E+08	0.25	2.66	3.15	0.05
E1	GY	5B	AX.89475197	5.81E+08	0.24	2.67	3.12	0.03
E1	GY	5B	AX.89443122	5.87E+08	0.33	2.51	3.29	0.04
E1	GY	5B	AX.89751210	4.79E+08	0.05	-5.21	3.14	0.05
E1	GY	5B	AX.89751210	4.79E+08	0.05	-5.21	3.14	0.05
E1	GY	5B	AX.89703676	6.32E+08	0.23	2.90	3.51	0.06
E1	GY	5B	AX.89678593	6.42E+08	0.11	-3.67	3.08	0.05
E1	GY	5B	AX.89403157	6.32E+08	0.27	2.74	3.46	0.06
E1	GY	5B	AX.89464183	6.36E+08	0.34	2.45	3.19	0.05
E1	GY	5B	AX.89667390	1.97E+07	0.18	-3.25	3.58	0.05
E1	GY	5B	AX.89536172	1.98E+07	0.18	-3.14	3.43	0.05
E1	GY	5B	AX.89501084	1.97E+07	0.18	-3.25	3.58	0.05
E1	GY	5B	AX.89419694	6.93E+08	0.06	-4.93	3.26	0.07
E1	GY	5B	AX.89419694	6.93E+08	0.06	-4.93	3.26	0.07
E1	GY	5B	AX.89771453	4.79E+08	0.05	-5.21	3.14	0.05
E1	GY	5B	AX.89327601	6.31E+08	0.23	2.90	3.51	0.06
E1	GY	5B	AX.89466410	6.43E+08	0.11	-3.67	3.08	0.05
E1	GY	5B	AX.89537992	1.95E+07	0.17	-3.24	3.49	0.05
E1	GY	5B	AX.89632393	5.86E+08	0.34	2.54	3.40	0.04
E1	GY	5B	AX.89362470	4.79E+08	0.05	-5.21	3.14	0.05
E1	GY	5B	AX.89328144	4.79E+08	0.05	-5.21	3.14	0.05
E1	GY	5B	AX.89754983	5.62E+08	0.31	2.53	3.25	0.05
E1	GY	5B	AX.89754983	5.62E+08	0.31	2.53	3.25	0.05
E1	GY	5B	AX.89456566	4.89E+08	0.06	-4.93	3.06	0.05
E1	GY	5B	AX.89476512	1.95E+07	0.18	-3.25	3.58	0.05
E1	GY	5B	AX.89476512	1.95E+07	0.18	-3.25	3.58	0.05
E1	GY	5B	AX.89422733	6.35E+08	0.28	2.62	3.28	0.05
E1	GY	5B	AX.89756842	1.13E+07	0.17	-3.31	3.62	0.05
E1	GY	5B	AX.89457601	4.79E+08	0.05	-5.21	3.14	0.05
E1	GY	5B	AX.89435584	4.79E+08	0.05	-5.21	3.14	0.05
E1	GY	5B	AX.89635436	1.14E+07	0.17	-3.31	3.62	0.05
E1	GY	5B	AX.89635436	1.14E+07	0.17	-3.31	3.62	0.05
E1	GPM2	5B	AX.89405214	7.03E+08	0.27	-666.71	3.17	0.05
E1	GY	5B	AX.89619826	4.79E+08	0.05	-5.21	3.14	0.05
E1	GY	5B	AX.89413298	6.30E+08	0.23	2.90	3.51	0.06
E1	GY	5B	AX.89726529	4.79E+08	0.05	-5.21	3.14	0.05
E1	GY	5B	AX.89644522	6.32E+08	0.27	2.74	3.46	0.06
E1	GY	5B	AX.89437256	6.32E+08	0.23	2.90	3.51	0.06
E1	GY	5B	AX.89723687	6.32E+08	0.23	2.90	3.51	0.06
E1	GY	5B	AX.89723687	6.32E+08	0.23	2.90	3.51	0.06

E1	GPM2	5B	AX.89381160	6.93E+08	0.06	-1246.03	3.19	0.06
E1	GPM2	5B	AX.89543790	6.99E+06	0.44	608.45	3.29	0.05
E1	GY	5B	AX.89496756	6.36E+08	0.34	2.45	3.19	0.05
E1	GY	5B	AX.89704033	5.62E+08	0.27	2.58	3.10	0.05
E1	GPM2	5B	AX.89686833	7.13E+08	0.07	-1297.53	4.04	0.07
E1	GY	5B	AX.89446263	6.37E+08	0.25	2.89	3.65	0.05
E1	GPM2	5B	AX.89614903	7.12E+08	0.07	-1319.27	3.95	0.06
E1	GY	5B	AX.89532223	6.32E+08	0.27	2.74	3.46	0.06
E1	GPM2	5B	AX.89361075	4.35E+07	0.12	-883.86	3.03	0.05
E1	GY	5B	AX.89700614	6.32E+08	0.27	2.67	3.35	0.06
E1	GY	5B	AX.89704769	6.35E+08	0.28	2.62	3.28	0.05
E1	GPM2	5B	AX.89331179	6.85E+08	0.06	-1215.93	3.06	0.05
E1	GY	5B	AX.89543790	6.99E+06	0.44	2.27	3.03	0.05
E1	SPM2	5B	AX.89382950	1.32E+07	0.30	-19.51	3.58	0.06
E1	GY	5B	AX.89544766	5.60E+08	0.25	2.66	3.15	0.05
E1	GPM2	5B	AX.89382361	7.13E+08	0.08	-1228.47	3.85	0.06
E1	GPM2	5B	AX.89421361	7.12E+08	0.07	-1297.53	4.04	0.07
E1	GPM2	5B	AX.89593207	7.12E+08	0.07	-1319.27	3.95	0.06
E1	GPM2	5B	AX.89315634	6.85E+08	0.07	-1224.56	3.65	0.06
E1	GPM2	5B	AX.89588192	5.86E+08	0.35	606.79	3.06	0.04
E1	GY	5B	AX.89617598	6.35E+08	0.28	2.62	3.28	0.05
E1	GY	5B	AX.89485770	1.13E+07	0.17	-3.31	3.62	0.05
E1	GY	5B	AX.89441811	1.98E+07	0.18	-3.25	3.58	0.05
E1	GPS	5B	AX.89382950	1.32E+07	0.30	1.57	4.74	0.09
E1	GPM2	5B	AX.89573389	7.12E+08	0.07	-1319.27	3.95	0.06
E1	GPM2	5B	AX.89655262	7.03E+08	0.29	-662.60	3.27	0.06
E1	GY	5B	AX.89582777	4.79E+08	0.05	-5.21	3.14	0.05
E1	GY	5B	AX.89536258	6.43E+08	0.11	-3.67	3.08	0.05
E1	GY	5B	AX.89372325	6.34E+08	0.28	2.62	3.28	0.05
E1	GPS	5B	AX.89439896	1.32E+07	0.29	1.41	3.87	0.07
E1	GY	5B	AX.89571058	1.95E+07	0.17	-3.18	3.39	0.05
E1	GPM2	5B	AX.89390726	6.85E+08	0.06	-1215.93	3.06	0.05
E1	GPM2	5B	AX.89463027	7.03E+08	0.30	-710.32	3.72	0.06
E1	GY	5B	AX.89701440	1.98E+07	0.18	-3.25	3.58	0.05
E1	GY	5B	AX.89337552	5.87E+08	0.27	-2.62	3.20	0.06
E1	GY	5B	AX.89730035	5.42E+08	0.11	3.73	3.25	0.06
E1	GPS	5B	AX.86170130	6.90E+08	0.43	-1.18	3.29	0.06
E1	GPS	5B	AX.89532549	6.86E+08	0.06	-2.59	3.40	0.06
E1	GY	5B	AX.89652951	1.15E+07	0.18	-3.14	3.38	0.05
E1	GY	5B	AX.89705205	5.83E+08	0.40	2.54	3.58	0.06
E1	GPM2	5B	AX.89532549	6.86E+08	0.06	-1263.08	3.06	0.05
E1	GPS	5B	AX.89388779	1.32E+07	0.29	1.54	4.51	0.08
E1	GPM2	5B	AX.89353411	6.85E+08	0.07	-1224.56	3.65	0.06
E1	GY	5B	AX.89621509	4.55E+08	0.23	-2.80	3.26	0.06
E1	GPM2	5B	AX.89668970	6.84E+06	0.40	628.84	3.40	0.05
E1	GY	5B	AX.89481359	4.79E+08	0.05	-5.21	3.14	0.05
E1	GY	5B	AX.89481359	4.79E+08	0.05	-5.21	3.14	0.05
E1	GPM2	5B	AX.89433479	7.13E+08	0.07	-1319.27	3.95	0.06
E1	GY	5B	AX.89550379	5.60E+08	0.25	2.66	3.15	0.05
E1	GY	5B	AX.89550379	5.60E+08	0.25	2.66	3.15	0.05

E1	GY	5B	AX.89517273	6.43E+08	0.11	-3.67	3.08	0.05
E1	GPM2	5B	AX.89325602	6.93E+08	0.25	-863.09	4.71	0.09
E1	GPS	5B	AX.89631532	6.85E+08	0.06	-2.37	3.12	0.05
E1	GPS	5B	AX.89323712	1.32E+07	0.48	-1.23	3.62	0.06
E1	GY	5B	AX.89650284	6.34E+08	0.28	2.62	3.28	0.05
E1	GPS	5B	AX.89513907	1.32E+07	0.29	1.48	4.18	0.07
E1	GPM2	5B	AX.89638955	7.03E+08	0.28	-834.01	4.76	0.09
E1	GY	5B	AX.89630915	5.62E+08	0.33	2.49	3.23	0.05
E1	GY	5B	AX.89753098	4.79E+08	0.05	-5.21	3.14	0.05
E1	GPS	5B	AX.89651787	4.89E+08	0.05	-2.55	3.09	0.05
E1	GY	5B	AX.89542131	6.36E+08	0.27	2.99	4.06	0.06
E1	GY	5B	AX.89552577	4.79E+08	0.05	-5.21	3.14	0.05
E1	GPM2	5B	AX.89675505	7.12E+08	0.07	-1297.53	4.04	0.07
E1	GPS	5B	AX.89327121	6.85E+08	0.06	-2.37	3.12	0.05
E1	GPM2	5B	AX.89337872	7.13E+08	0.07	-1319.27	3.95	0.06
E1	GPS	5B	AX.86166457	6.85E+08	0.07	-2.37	3.32	0.06
E1	GY	5B	AX.89732001	6.36E+08	0.34	2.45	3.19	0.05
E1	GY	5B	AX.89732001	6.36E+08	0.34	2.45	3.19	0.05
E1	GPM2	5B	AX.89338561	4.43E+08	0.44	707.18	4.26	0.08
E1	GPS	5B	AX.89331179	6.85E+08	0.06	-2.37	3.12	0.05
E1	GY	5B	AX.89590734	5.61E+08	0.25	2.66	3.15	0.05
E1	GPS	5B	AX.89423781	1.32E+07	0.29	1.48	4.18	0.07
E1	GY	5B	AX.89508910	6.32E+08	0.23	2.90	3.51	0.06
E1	GY	5B	AX.89653910	5.81E+08	0.24	2.67	3.12	0.03
E1	GY	5B	AX.89646283	4.80E+08	0.05	-5.21	3.14	0.05
E1	GY	5B	AX.89646283	4.80E+08	0.05	-5.21	3.14	0.05
E1	GY	5B	AX.89700391	4.79E+08	0.05	-5.21	3.14	0.05
E1	GY	5B	AX.89700391	4.79E+08	0.05	-5.21	3.14	0.05
E1	GPM2	5B	AX.89608007	7.13E+08	0.07	-1319.27	3.95	0.06
E1	GY	5B	AX.89641096	4.79E+08	0.05	-5.21	3.14	0.05
E1	GPM2	5B	AX.89551934	4.49E+07	0.40	-594.78	3.10	0.05
E1	GPM2	5B	AX.89592632	6.65E+06	0.40	590.53	3.04	0.05
E1	GPM2	5B	AX.89344908	5.87E+08	0.09	-1029.36	3.23	0.05
E1	GY	5B	AX.89498476	6.36E+08	0.30	2.94	4.14	0.06
E1	GY	5B	AX.89648556	6.32E+08	0.27	2.74	3.46	0.06
E1	GY	5B	AX.89653037	6.32E+08	0.27	2.74	3.46	0.06
E1	GY	5B	AX.89653037	6.32E+08	0.27	2.74	3.46	0.06
E1	GPS	5B	AX.89638155	1.32E+07	0.29	1.48	4.18	0.07
E1	GPS	5B	AX.89623198	4.89E+08	0.05	-2.55	3.09	0.05
E1	GPM2	5B	AX.89751183	6.85E+08	0.07	-1224.56	3.65	0.06
E1	GPS	5B	AX.89489374	1.32E+07	0.29	1.49	4.26	0.08
E1	GY	5B	AX.89655934	6.36E+08	0.34	2.45	3.19	0.05
E1	GPS	5B	AX.89350220	4.89E+08	0.05	-2.55	3.09	0.05
E1	GPS	5B	AX.89497888	4.89E+08	0.05	-2.55	3.09	0.05
E1	GPS	5B	AX.89456923	6.86E+08	0.06	-2.59	3.40	0.06
E1	GPM2	5B	AX.89370717	7.04E+08	0.28	-876.57	5.25	0.10
E1	GPM2	5B	AX.89371209	6.37E+08	0.30	658.72	3.27	0.05
E1	GPM2	5B	AX.89695965	7.12E+08	0.07	-1319.27	3.95	0.06
E1	GY	5B	AX.89635785	6.99E+06	0.39	2.40	3.24	0.05
E1	GY	5B	AX.89635785	6.99E+06	0.39	2.40	3.24	0.05

E1	GPS	5B	AX.89412840	6.90E+08	0.38	-1.25	3.52	0.06
E1	GPM2	5B	AX.89752582	6.85E+08	0.06	-1215.93	3.06	0.05
E1	GPS	5B	AX.89454762	6.90E+08	0.42	-1.14	3.08	0.05
E1	GY	5B	AX.89638955	7.03E+08	0.28	-3.27	4.80	0.09
E1	GY	5B	AX.89532955	1.97E+07	0.18	-3.25	3.58	0.05
E1	GPM2	5B	AX.89504076	7.03E+08	0.07	-1329.59	4.00	0.08
E1	GY	5B	AX.89514611	6.37E+08	0.34	2.45	3.19	0.05
E1	GPM2	5B	AX.89627627	6.68E+06	0.40	628.84	3.40	0.05
E1	GY	5B	AX.89774719	6.35E+08	0.29	2.57	3.21	0.05
E1	GPM2	5B	AX.89764433	6.94E+08	0.05	-1358.81	3.23	0.05
E1	GPM2	5B	AX.89465129	7.03E+08	0.26	-667.56	3.12	0.05
E1	GPM2	5B	AX.89682564	7.13E+08	0.07	-1297.53	4.04	0.07
E1	GY	5B	AX.89561097	5.62E+08	0.27	2.62	3.21	0.05
E1	GPM2	5B	AX.89392333	6.85E+08	0.06	-1215.93	3.06	0.05
E1	GY	5B	AX.89456230	6.35E+08	0.29	2.57	3.21	0.05
E1	GY	5B	AX.89504076	7.03E+08	0.07	-4.77	3.45	0.07
E1	GPM2	5B	AX.89713196	4.32E+07	0.13	-894.74	3.35	0.06
E1	GPM2	5B	AX.89393248	6.65E+06	0.40	616.06	3.29	0.05
E1	GPM2	5B	AX.89420449	6.85E+08	0.07	-1226.80	3.48	0.06
E1	GY	5B	AX.89678549	1.98E+07	0.18	-3.24	3.62	0.05
E1	GPM2	5B	AX.89594998	7.03E+08	0.28	-824.79	4.67	0.08
E1	GPM2	5B	AX.89708564	6.85E+08	0.07	-1224.56	3.65	0.06
E1	GY	5B	AX.89680483	4.80E+08	0.05	-5.21	3.14	0.05
E1	GPM2	5B	AX.89456923	6.86E+08	0.06	-1263.08	3.06	0.05
E1	GY	5B	AX.89622171	1.11E+07	0.17	-3.31	3.62	0.05
E1	GY	5B	AX.89518064	4.79E+08	0.05	-5.21	3.14	0.05
E1	GPM2	5B	AX.89689229	6.37E+08	0.30	658.72	3.27	0.05
E1	GY	5B	AX.89600633	1.15E+07	0.18	-3.14	3.38	0.05
E1	GPM2	5B	AX.89593309	6.84E+06	0.41	592.86	3.09	0.05
E1	GPM2	5B	AX.89434733	4.28E+07	0.12	-883.86	3.03	0.05
E1	GPM2	5B	AX.89724475	7.12E+08	0.07	-1319.27	3.95	0.06
E1	GY	5B	AX.89744387	4.64E+08	0.31	-2.59	3.37	0.06
E1	GY	5B	AX.89516752	1.97E+07	0.18	-3.25	3.58	0.05
E1	GY	5B	AX.89654987	1.97E+07	0.18	-3.25	3.58	0.05
E1	GPM2	5B	AX.89756590	7.12E+08	0.05	-1303.20	3.01	0.05
E1	GY	5B	AX.89424748	1.13E+07	0.17	-3.31	3.62	0.05
E1	GY	5B	AX.89689229	6.37E+08	0.30	3.21	4.81	0.07
E1	GPM2	5B	AX.89776636	7.03E+08	0.26	-667.56	3.12	0.05
E1	GPM2	5B	AX.89728284	7.12E+08	0.07	-1297.53	4.04	0.07
E1	GY	5B	AX.89651787	4.89E+08	0.05	-5.20	3.13	0.05
E1	GPM2	5B	AX.89454797	7.12E+08	0.07	-1319.27	3.95	0.06
E1	GY	5B	AX.89680483	4.80E+08	0.05	-5.21	3.14	0.05
E1	GPM2	5B	AX.89658465	7.12E+08	0.07	-1362.37	3.95	0.06
E1	GPM2	5B	AX.89499258	5.87E+08	0.09	-1029.36	3.23	0.05
E1	GPM2	5B	AX.89600504	6.86E+08	0.06	-1263.08	3.06	0.05
E1	GY	5B	AX.89450464	5.60E+08	0.25	2.66	3.15	0.05
E1	GY	5B	AX.89322854	1.13E+07	0.17	-3.31	3.62	0.05
E1	GY	5B	AX.89638418	4.79E+08	0.05	-5.21	3.14	0.05
E1	GY	5B	AX.89392375	6.43E+08	0.11	-3.67	3.08	0.05
E1	GY	5B	AX.89702244	6.37E+08	0.30	2.94	4.14	0.06

E1	GY	5B	AX.89738550	6.37E+08	0.34	2.45	3.19	0.05
E1	GY	5B	AX.89714399	1.15E+07	0.18	-3.14	3.38	0.05
E1	GY	5B	AX.89702322	6.37E+08	0.34	2.99	4.54	0.07
E1	GY	5B	AX.89381160	6.93E+08	0.06	-4.93	3.26	0.07
E1	GY	5B	AX.89393248	6.65E+06	0.40	2.30	3.05	0.04
E1	GY	5B	AX.89463041	4.66E+08	0.06	-4.72	3.02	0.05
E1	GY	5B	AX.89703676	6.32E+08	0.23	2.90	3.51	0.06
E1	GY	5B	AX.89678593	6.42E+08	0.11	-3.67	3.08	0.05
E1	GY	5B	AX.89630366	7.04E+08	0.07	-4.78	3.46	0.07
E1	GY	5B	AX.89516768	4.79E+08	0.05	-5.21	3.14	0.05
E1	GY	5B	AX.89329613	1.97E+07	0.18	-3.25	3.58	0.05
E1	GY	5B	AX.89457601	4.79E+08	0.05	-5.21	3.14	0.05
E1	GPM2	5B	AX.89716929	6.85E+08	0.07	-1224.56	3.65	0.06
E1	GY	5B	AX.89623198	4.89E+08	0.05	-5.20	3.13	0.05
E1	GY	5B	AX.89689204	1.95E+07	0.22	-3.13	3.91	0.07
E1	GY	5B	AX.89689204	1.95E+07	0.22	-3.13	3.91	0.07
E1	GPM2	5B	AX.89763715	3.86E+07	0.25	-677.97	3.10	0.05
E1	GPM2	5B	AX.89734563	6.85E+08	0.06	-1215.93	3.06	0.05
E1	GPM2	5B	AX.89646040	7.12E+08	0.07	-1319.27	3.95	0.06
E1	GY	5B	AX.89700614	6.32E+08	0.27	2.67	3.35	0.06
E1	GPM2	5B	AX.89505750	7.03E+08	0.26	-667.56	3.12	0.05
E1	GPM2	5B	AX.89631532	6.85E+08	0.06	-1215.93	3.06	0.05
E1	GPM2	5B	AX.89421958	7.12E+08	0.07	-1319.27	3.95	0.06
E1	GPM2	5B	AX.89479246	6.86E+08	0.06	-1263.08	3.06	0.05
E1	GY	5B	AX.89631907	6.36E+08	0.33	2.70	3.71	0.06
E1	GPM2	5B	AX.89501784	7.12E+08	0.07	-1319.27	3.95	0.06
E1	GPM2	5B	AX.89734081	7.13E+08	0.06	-1571.45	4.47	0.07
E1	GY	5B	AX.89617598	6.35E+08	0.28	2.62	3.28	0.05
E1	GY	5B	AX.89655262	7.03E+08	0.29	-2.58	3.26	0.06
E1	GPS	5B	AX.89390726	6.85E+08	0.06	-2.37	3.12	0.05
E1	GPM2	5B	AX.89543476	6.85E+08	0.07	-1266.62	3.48	0.06
E1	GY	5B	AX.89501286	7.43E+07	0.47	2.31	3.16	0.06
E1	GY	5B	AX.89718577	5.86E+08	0.34	2.54	3.40	0.04
E1	GY	5B	AX.89652951	1.15E+07	0.18	-3.14	3.38	0.05
E1	GY	5B	AX.89644485	1.98E+07	0.18	-3.25	3.58	0.05
E1	GY	5B	AX.89759768	5.62E+08	0.33	2.45	3.14	0.05
E1	GPM2	5B	AX.86166457	6.85E+08	0.07	-1266.62	3.48	0.06
E1	GPS	5B	AX.89504388	1.32E+07	0.29	1.53	4.50	0.08
E1	GPS	5B	AX.89772432	1.32E+07	0.30	1.57	4.74	0.09
E1	GPS	5B	AX.89752582	6.85E+08	0.06	-2.37	3.12	0.05
E1	GPS	5B	AX.89351668	4.89E+08	0.05	-2.55	3.09	0.05
E1	GY	5B	AX.89658736	4.79E+08	0.05	-5.21	3.14	0.05
E1	GPM2	5B	AX.89403059	6.85E+08	0.06	-1215.93	3.06	0.05
E1	GPM2	5B	AX.89450444	6.67E+06	0.40	628.84	3.40	0.05
E1	GY	5B	AX.89753098	4.79E+08	0.05	-5.21	3.14	0.05
E1	GY	5B	AX.89447298	2.00E+07	0.18	-3.25	3.58	0.05
E1	GPM2	5B	AX.89469201	6.85E+08	0.07	-1224.56	3.65	0.06
E1	GPM2	5B	AX.89632104	4.10E+07	0.25	-737.37	3.58	0.06
E1	GPM2	5B	AX.89328074	7.12E+08	0.07	-1319.27	3.95	0.06
E1	GPM2	5B	AX.89747062	7.12E+08	0.07	-1297.53	4.04	0.07

E1	GPM2	5B	AX.89390507	7.12E+08	0.07	-1319.27	3.95	0.06
E1	GPM2	5B	AX.89494775	7.12E+08	0.07	-1297.53	4.04	0.07
E1	GPM2	5B	AX.89327121	6.85E+08	0.06	-1215.93	3.06	0.05
E1	GPM2	5B	AX.89525026	4.28E+07	0.12	-883.86	3.03	0.05
E1	GPS	5B	AX.89636160	4.89E+08	0.05	-2.55	3.09	0.05
E1	GY	5B	AX.89587402	6.43E+08	0.11	-3.67	3.08	0.05
E1	GPS	5B	AX.89420449	6.85E+08	0.07	-2.20	3.07	0.05
E1	GPM2	5B	AX.89763554	7.12E+08	0.07	-1319.27	3.95	0.06
E1	GY	5B	AX.89702287	6.20E+08	0.24	3.04	3.89	0.07
E1	GPM2	5B	AX.89516621	7.03E+08	0.26	-667.56	3.12	0.05
E1	GPM2	5B	AX.89415101	7.13E+08	0.07	-1319.27	3.95	0.06
E1	GPS	5B	AX.89365622	6.90E+08	0.38	-1.25	3.52	0.06
E1	GPM2	5B	AX.89756817	7.13E+08	0.07	-1319.27	3.95	0.06
E1	GY	5B	AX.89608793	6.43E+08	0.11	-3.67	3.08	0.05
E1	TKW	5B	AX.89532063	4.61E+08	0.08	-1.56	3.22	0.05
E1	GY	5B	AX.89675139	6.32E+08	0.27	2.74	3.46	0.06
E1	GPM2	5B	AX.89380715	6.93E+08	0.24	-833.76	4.39	0.08
E1	GPM2	5B	AX.89314809	7.12E+08	0.07	-1319.27	3.95	0.06
E1	GY	5B	AX.89679245	4.80E+08	0.05	-5.21	3.14	0.05
E1	GPM2	5B	AX.89419694	6.93E+08	0.06	-1246.03	3.19	0.06
E1	GY	5B	AX.89515226	6.35E+08	0.28	2.62	3.28	0.05
E1	TKW	5B	AX.89668374	3.15E+08	0.21	-1.02	3.03	0.05
E1	GPM2	5B	AX.89328007	7.03E+08	0.27	-666.71	3.17	0.05
E1	GPM2	5B	AX.89723531	7.12E+08	0.07	-1319.27	3.95	0.06
E1	GPM2	5B	AX.89462390	4.28E+07	0.12	-883.86	3.03	0.05
E1	TKW	5B	AX.89432218	6.23E+08	0.13	-1.26	3.23	0.05
E1	GPM2	5B	AX.89732027	7.03E+08	0.27	-666.71	3.17	0.05
E1	GY	5B	AX.89669309	4.79E+08	0.05	-5.21	3.14	0.05
E1	GPM2	5B	AX.89454370	7.12E+08	0.07	-1319.27	3.95	0.06
E1	GPM2	5B	AX.89313461	7.03E+08	0.05	-1306.05	3.02	0.06
E1	TKW	5B	AX.89506309	4.59E+08	0.07	-1.72	3.32	0.06
E1	GY	5B	AX.89756842	1.13E+07	0.17	-3.31	3.62	0.05
E1	GPM2	5B	AX.89739979	7.13E+08	0.07	-1319.27	3.95	0.06
E1	TKW	5B	AX.89746258	6.23E+08	0.13	-1.26	3.23	0.05
E1	GPM2	5B	AX.89635785	6.99E+06	0.39	615.51	3.25	0.05
E1	GPM2	5B	AX.89575415	6.93E+08	0.06	-1246.03	3.19	0.06
E1	TKW	5B	AX.89435338	4.60E+08	0.08	-1.56	3.22	0.05
E1	GY	5B	AX.89720865	4.79E+08	0.05	-5.21	3.14	0.05
E1	GY	5B	AX.89654987	1.97E+07	0.18	-3.25	3.58	0.05
E1	GPM2	5B	AX.89332853	4.23E+07	0.13	-918.80	3.42	0.06
E1	TKW	5B	AX.89497439	4.61E+08	0.08	-1.56	3.22	0.05
E1	TKW	5B	AX.89412840	6.90E+08	0.38	0.89	3.21	0.05
E1	GPM2	5B	AX.89697602	6.85E+08	0.08	-1091.09	3.29	0.05
E1	TKW	5B	AX.89425960	4.61E+08	0.08	-1.56	3.22	0.05
E1	GY	5B	AX.89567072	1.14E+07	0.17	-3.31	3.62	0.05
E1	GY	5B	AX.89486782	6.32E+08	0.23	2.90	3.51	0.06
E1	TKW	5B	AX.89314375	6.22E+08	0.13	-1.26	3.06	0.05
E1	GY	5B	AX.89702322	6.37E+08	0.34	2.99	4.54	0.07
E1	TKW	5B	AX.89386002	4.61E+08	0.08	-1.56	3.22	0.05
E1	TKW	5B	AX.89424190	4.61E+08	0.08	-1.56	3.22	0.05

E1	TKW	5B	AX.89415645	4.61E+08	0.08	-1.56	3.22	0.05
E1	GY	5B	AX.89644522	6.32E+08	0.27	2.74	3.46	0.06
E1	TKW	5B	AX.89650328	4.61E+08	0.08	-1.56	3.22	0.05
E1	TKW	5B	AX.89698901	4.61E+08	0.08	-1.56	3.22	0.05
E1	GPM2	5B	AX.89557890	5.87E+08	0.09	-1029.36	3.23	0.05
E1	GPM2	5B	AX.89356496	7.03E+08	0.27	-666.71	3.17	0.05
E1	TKW	5B	AX.89365622	6.90E+08	0.38	0.89	3.21	0.05
E1	GPM2	5B	AX.89501286	7.43E+07	0.47	659.41	3.80	0.07
E1	GY	5B	AX.89462733	1.95E+07	0.19	-3.21	3.68	0.06
E1	GPM2	5B	AX.89463041	4.66E+08	0.06	-1247.48	3.20	0.05
E1	TKW	5B	AX.89454762	6.90E+08	0.42	0.89	3.29	0.05
E1	TKW	5B	AX.89366900	4.61E+08	0.08	-1.56	3.22	0.05
E1	GPM2	5B	AX.89364659	7.12E+08	0.07	-1319.27	3.95	0.06
E1	GY	5B	AX.89658736	4.79E+08	0.05	-5.21	3.14	0.05
E1	GPM2	5B	AX.89756512	7.13E+08	0.07	-1319.27	3.95	0.06
E1	GPM2	5B	AX.89652622	7.04E+06	0.39	615.51	3.25	0.05
E1	TKW	5B	AX.89744863	4.60E+08	0.08	-1.56	3.22	0.05
E1	TKW	5B	AX.89331027	3.75E+07	0.06	-1.97	3.48	0.06
E1	GPM2	5B	AX.89565973	7.13E+08	0.07	-1319.27	3.95	0.06
E1	TKW	5B	AX.89602013	4.60E+08	0.08	-1.56	3.22	0.05
E1	GPM2	5B	AX.89601860	7.03E+08	0.06	-1436.94	3.82	0.07
E1	GPM2	5B	AX.89367647	6.67E+06	0.40	628.84	3.40	0.05
E2	GY	5B	AX.89499896	4.61E+08	0.07	-3.72	3.22	0.05
E2	SPM2	5B	AX.89655914	4.59E+08	0.11	18.77	3.02	0.05
E2	SPM2	5B	AX.89625953	4.59E+08	0.11	18.77	3.02	0.05
E2	SPM2	5B	AX.89420933	4.59E+08	0.11	18.77	3.02	0.05
E2	SPM2	5B	AX.89353786	4.59E+08	0.11	18.77	3.02	0.05
E2	SPM2	5B	AX.89421671	4.59E+08	0.11	18.77	3.02	0.05
E2	SPM2	5B	AX.89476039	4.59E+08	0.11	18.77	3.02	0.05
E2	SPM2	5B	AX.89364776	4.59E+08	0.11	18.77	3.02	0.05
E2	SPM2	5B	AX.89544142	4.59E+08	0.11	18.77	3.02	0.05
E2	GPM2	5B	AX.89532063	4.61E+08	0.08	1062.92	4.31	0.08
E2	SPM2	5B	AX.89549787	6.60E+08	0.30	-13.22	3.24	0.06
E2	GY	5B	AX.89614362	4.78E+08	0.06	-4.52	3.52	0.06
E2	SPM2	5B	AX.89570478	4.59E+08	0.11	18.77	3.02	0.05
E2	SPM2	5B	AX.89722558	4.59E+08	0.11	18.77	3.02	0.05
E2	SPM2	5B	AX.89416929	4.59E+08	0.11	18.77	3.02	0.05
E2	SPM2	5B	AX.89424256	4.59E+08	0.11	18.77	3.02	0.05
E2	SPM2	5B	AX.89500255	4.59E+08	0.11	18.77	3.02	0.05
E2	SPM2	5B	AX.89773672	4.59E+08	0.11	18.77	3.02	0.05
E2	GY	5B	AX.89514151	4.61E+08	0.07	-3.72	3.22	0.05
E2	SPM2	5B	AX.89322706	4.59E+08	0.11	18.77	3.02	0.05
E2	SPM2	5B	AX.89664209	6.94E+08	0.13	17.57	3.12	0.05
E2	SPM2	5B	AX.89541437	4.59E+08	0.11	18.77	3.02	0.05
E2	SPM2	5B	AX.89608876	4.59E+08	0.11	18.77	3.02	0.05
E2	GY	5B	AX.89657211	4.61E+08	0.22	-2.41	3.40	0.06
E2	GY	5B	AX.89434025	4.60E+08	0.23	-2.46	3.55	0.06
E2	GPM2	5B	AX.89331405	5.47E+08	0.36	-499.45	3.01	0.05
E2	GY	5B	AX.89376309	4.78E+08	0.06	-4.52	3.52	0.06
E2	GY	5B	AX.89376309	4.78E+08	0.06	-4.52	3.52	0.06

E2	GY	5B	AX.89586746	4.60E+08	0.23	-2.46	3.55	0.06
E2	GY	5B	AX.89586746	4.60E+08	0.23	-2.46	3.55	0.06
E2	GY	5B	AX.89661473	4.59E+08	0.17	-2.69	3.37	0.06
E2	GY	5B	AX.89661473	4.59E+08	0.17	-2.69	3.37	0.06
E2	GY	5B	AX.89590361	4.60E+08	0.23	-2.46	3.55	0.06
E2	GY	5B	AX.89590361	4.60E+08	0.23	-2.46	3.55	0.06
E2	GY	5B	AX.89509001	4.60E+08	0.23	-2.46	3.55	0.06
E2	GY	5B	AX.89509001	4.60E+08	0.23	-2.46	3.55	0.06
E2	GY	5B	AX.89461377	4.60E+08	0.23	-2.46	3.55	0.06
E2	GY	5B	AX.89704132	4.60E+08	0.23	-2.46	3.55	0.06
E2	GY	5B	AX.89704132	4.60E+08	0.23	-2.46	3.55	0.06
E2	GY	5B	AX.89346423	7.07E+08	0.16	-2.79	3.37	0.09
E2	GY	5B	AX.89346423	7.07E+08	0.16	-2.79	3.37	0.09
E2	GY	5B	AX.89381655	4.66E+08	0.05	-4.72	3.57	0.06
E2	GY	5B	AX.89381655	4.66E+08	0.05	-4.72	3.57	0.06
E2	GY	5B	AX.89346942	6.69E+08	0.08	-3.60	3.21	0.04
E2	GY	5B	AX.89346942	6.69E+08	0.08	-3.60	3.21	0.04
E2	GY	5B	AX.89516431	4.60E+08	0.23	-2.46	3.55	0.06
E2	GY	5B	AX.89516431	4.60E+08	0.23	-2.46	3.55	0.06
E2	GY	5B	AX.89567569	6.69E+08	0.12	-3.09	3.40	0.04
E2	GY	5B	AX.89567569	6.69E+08	0.12	-3.09	3.40	0.04
E2	GY	5B	AX.89455616	4.60E+08	0.23	-2.46	3.55	0.06
E2	GY	5B	AX.89455616	4.60E+08	0.23	-2.46	3.55	0.06
E2	GY	5B	AX.89422127	4.59E+08	0.17	-2.69	3.37	0.06
E2	GY	5B	AX.89422127	4.59E+08	0.17	-2.69	3.37	0.06
E2	GPM2	5B	AX.89435338	4.60E+08	0.08	1062.92	4.31	0.08
E2	GY	5B	AX.89718438	4.78E+08	0.06	-4.52	3.52	0.06
E2	TKW	5B	AX.89755554	2.42E+08	0.15	-1.38	3.15	0.06
E2	GY	5B	AX.89461377	4.60E+08	0.23	-2.46	3.55	0.06
E2	GY	5B	AX.89356716	6.69E+08	0.12	-3.09	3.40	0.04
E2	GY	5B	AX.89356716	6.69E+08	0.12	-3.09	3.40	0.04
E2	GY	5B	AX.89606286	4.78E+08	0.06	-4.52	3.52	0.06
E2	GY	5B	AX.89606286	4.78E+08	0.06	-4.52	3.52	0.06
E2	GY	5B	AX.89607893	3.73E+08	0.07	-3.65	3.12	0.05
E2	GY	5B	AX.89607893	3.73E+08	0.07	-3.65	3.12	0.05
E2	GY	5B	AX.89478037	4.60E+08	0.23	-2.46	3.55	0.06
E2	GY	5B	AX.89327642	4.62E+08	0.21	-2.70	4.02	0.07
E2	GY	5B	AX.89327642	4.62E+08	0.21	-2.70	4.02	0.07
E2	GY	5B	AX.89553321	4.62E+08	0.21	-2.70	4.02	0.07
E2	GY	5B	AX.89553321	4.62E+08	0.21	-2.70	4.02	0.07
E2	GY	5B	AX.89499896	4.61E+08	0.07	-3.72	3.22	0.05
E2	GY	5B	AX.89646594	4.62E+08	0.21	-2.70	4.02	0.07
E2	GY	5B	AX.89697318	6.69E+08	0.13	-2.99	3.49	0.04
E2	GY	5B	AX.89697318	6.69E+08	0.13	-2.99	3.49	0.04
E2	GY	5B	AX.89776155	4.60E+08	0.20	-2.59	3.60	0.05
E2	GY	5B	AX.89718438	4.78E+08	0.06	-4.52	3.52	0.06
E2	TKW	5B	AX.89683368	6.83E+08	0.37	1.00	3.01	0.06
E2	GY	5B	AX.89614362	4.78E+08	0.06	-4.52	3.52	0.06
E2	GY	5B	AX.89566726	4.60E+08	0.23	-2.46	3.55	0.06
E2	GY	5B	AX.89356446	4.60E+08	0.23	-2.46	3.55	0.06

E2	GY	5B	AX.89356446	4.60E+08	0.23	-2.46	3.55	0.06
E2	GY	5B	AX.89678817	4.60E+08	0.23	-2.22	3.02	0.05
E2	GY	5B	AX.89431248	4.59E+08	0.23	-2.46	3.55	0.06
E2	GY	5B	AX.89431248	4.59E+08	0.23	-2.46	3.55	0.06
E2	GY	5B	AX.89618438	4.61E+08	0.07	-3.72	3.22	0.05
E2	GY	5B	AX.89514151	4.61E+08	0.07	-3.72	3.22	0.05
E2	GY	5B	AX.89478037	4.60E+08	0.23	-2.46	3.55	0.06
E2	GPM2	5B	AX.89602013	4.60E+08	0.08	1062.92	4.31	0.08
E2	GY	5B	AX.89657211	4.61E+08	0.22	-2.41	3.40	0.06
E2	TKW	5B	AX.89777083	6.82E+08	0.31	1.12	3.44	0.07
E2	GY	5B	AX.89434025	4.60E+08	0.23	-2.46	3.55	0.06
E2	GY	5B	AX.89776155	4.60E+08	0.20	-2.59	3.60	0.05
E2	GPM2	5B	AX.89642352	5.47E+08	0.36	-500.33	3.02	0.05
E2	GY	5B	AX.89642265	4.61E+08	0.23	-2.46	3.55	0.06
E2	GY	5B	AX.89642265	4.61E+08	0.23	-2.46	3.55	0.06
E2	GY	5B	AX.89353414	4.60E+08	0.23	-2.46	3.55	0.06
E2	GY	5B	AX.89686617	4.61E+08	0.23	-2.50	3.65	0.07
E2	GY	5B	AX.89686617	4.61E+08	0.23	-2.50	3.65	0.07
E2	GPS	5B	AX.89454016	6.64E+08	0.15	-2.03	3.51	0.07
E2	GY	5B	AX.89427854	4.61E+08	0.24	-2.40	3.49	0.06
E2	GY	5B	AX.89427854	4.61E+08	0.24	-2.40	3.49	0.06
E2	GY	5B	AX.89678817	4.60E+08	0.23	-2.22	3.02	0.05
E2	GY	5B	AX.89752714	6.69E+08	0.12	-3.09	3.40	0.04
E2	GPM2	5B	AX.89465618	4.63E+08	0.06	1021.51	3.05	0.05
E2	GPM2	5B	AX.89713558	5.47E+08	0.36	-500.33	3.02	0.05
E2	GPM2	5B	AX.89365226	4.64E+08	0.07	1023.60	3.25	0.05
E2	GPM2	5B	AX.89373215	4.61E+08	0.08	1112.30	4.27	0.08
E2	GPM2	5B	AX.89754955	4.64E+08	0.06	1021.51	3.05	0.05
E2	GY	5B	AX.89764514	4.62E+08	0.21	-2.70	4.02	0.07
E2	TKW	5B	AX.89645403	1.39E+07	0.09	-1.66	3.05	0.06
E2	GY	5B	AX.89538284	4.60E+08	0.23	-2.46	3.55	0.06
E2	GPM2	5B	AX.89380376	5.47E+08	0.36	-500.33	3.02	0.05
E2	GPM2	5B	AX.89440851	5.47E+08	0.36	-499.45	3.01	0.05
E2	TKW	5B	AX.89523157	6.84E+08	0.39	1.02	3.19	0.07
E2	GPM2	5B	AX.89744863	4.60E+08	0.08	1062.92	4.31	0.08
E2	GY	5B	AX.89353414	4.60E+08	0.23	-2.46	3.55	0.06
E2	GY	5B	AX.89764514	4.62E+08	0.21	-2.70	4.02	0.07
E2	GPM2	5B	AX.89420230	5.47E+08	0.36	-500.33	3.02	0.05
E2	GY	5B	AX.89759980	4.78E+08	0.06	-4.52	3.52	0.06
E2	GY	5B	AX.89519958	4.60E+08	0.23	-2.46	3.55	0.06
E2	TKW	5B	AX.89365622	6.90E+08	0.38	1.00	3.08	0.06
E2	GPM2	5B	AX.89776439	5.47E+08	0.34	-539.22	3.39	0.05
E2	GY	5B	AX.89704665	4.78E+08	0.06	-4.52	3.52	0.06
E2	GY	5B	AX.89704665	4.78E+08	0.06	-4.52	3.52	0.06
E2	GY	5B	AX.89752714	6.69E+08	0.12	-3.09	3.40	0.04
E2	GPM2	5B	AX.89506309	4.59E+08	0.07	1160.06	4.37	0.08
E2	GPS	5B	AX.89484565	5.57E+08	0.39	1.42	3.32	0.05
E2	GY	5B	AX.89681314	4.61E+08	0.07	-3.72	3.22	0.05
E2	GY	5B	AX.89681314	4.61E+08	0.07	-3.72	3.22	0.05
E2	GY	5B	AX.89538284	4.60E+08	0.23	-2.46	3.55	0.06

E2	GY	5B	AX.89646594	4.62E+08	0.21	-2.70	4.02	0.07
E2	GPM2	5B	AX.89658797	5.47E+08	0.36	-500.33	3.02	0.05
E2	GY	5B	AX.89759980	4.78E+08	0.06	-4.52	3.52	0.06
E2	GY	5B	AX.89519958	4.60E+08	0.23	-2.46	3.55	0.06
E2	GPM2	5B	AX.89405571	4.64E+08	0.06	1021.51	3.05	0.05
E2	GPM2	5B	AX.89340142	5.47E+08	0.36	-500.33	3.02	0.05
E2	GY	5B	AX.89566726	4.60E+08	0.23	-2.46	3.55	0.06
E2	GPM2	5B	AX.89366900	4.61E+08	0.08	1062.92	4.31	0.08
E2	GPM2	5B	AX.89386002	4.61E+08	0.08	1062.92	4.31	0.08
E2	GPM2	5B	AX.89410892	4.63E+08	0.06	1021.51	3.05	0.05
E2	GPM2	5B	AX.89526452	5.47E+08	0.36	-500.33	3.02	0.05
E2	GPM2	5B	AX.89586180	5.47E+08	0.36	-500.33	3.02	0.05
E2	GPM2	5B	AX.89415645	4.61E+08	0.08	1062.92	4.31	0.08
E2	GY	5B	AX.89618438	4.61E+08	0.07	-3.72	3.22	0.05
E2	GPM2	5B	AX.89513465	4.63E+08	0.06	1021.51	3.05	0.05
E2	GY	5B	AX.89521262	4.59E+08	0.23	-2.46	3.55	0.06
E2	GY	5B	AX.89521262	4.59E+08	0.23	-2.46	3.55	0.06
E2	TKW	5B	AX.89412840	6.90E+08	0.38	1.00	3.08	0.06
E2	GPS	5B	AX.89560822	6.84E+08	0.22	-1.58	3.03	0.05
E2	GPM2	5B	AX.89698901	4.61E+08	0.08	1062.92	4.31	0.08
E2	GY	5B	AX.89619002	4.62E+08	0.21	-2.70	4.02	0.07
E2	GPM2	5B	AX.89771463	5.47E+08	0.35	-551.97	3.57	0.06
E2	GPM2	5B	AX.89652234	4.63E+08	0.06	1021.51	3.05	0.05
E2	GPM2	5B	AX.89402116	5.47E+08	0.36	-500.33	3.02	0.05
E2	GPM2	5B	AX.89686617	4.61E+08	0.23	-568.40	3.01	0.06
E2	GPM2	5B	AX.89650328	4.61E+08	0.08	1062.92	4.31	0.08
E2	GPM2	5B	AX.89497439	4.61E+08	0.08	1062.92	4.31	0.08
E2	GPM2	5B	AX.89555546	4.63E+08	0.06	1021.51	3.05	0.05
E2	GPM2	5B	AX.89553392	4.64E+08	0.06	1021.51	3.05	0.05
E2	GY	5B	AX.89738953	4.78E+08	0.06	-4.52	3.52	0.06
E2	GY	5B	AX.89738953	4.78E+08	0.06	-4.52	3.52	0.06
E2	GY	5B	AX.89619002	4.62E+08	0.21	-2.70	4.02	0.07
E2	GPM2	5B	AX.89465633	4.64E+08	0.06	1021.51	3.05	0.05
E2	GPM2	5B	AX.89425960	4.61E+08	0.08	1062.92	4.31	0.08
E2	GPM2	5B	AX.89424190	4.61E+08	0.08	1062.92	4.31	0.08
E2	GPM2	5B	AX.89591714	5.47E+08	0.36	-500.33	3.02	0.05
E2	GPM2	5B	AX.89501864	4.64E+08	0.06	1021.51	3.05	0.05
E3	GPM2	5B	AX.89444793	5.48E+07	0.06	2343.60	5.08	0.09
E3	GPM2	5B	AX.89569208	5.48E+07	0.06	2343.60	5.08	0.09
E3	GPM2	5B	AX.89498183	5.48E+07	0.06	2343.60	5.08	0.09
E3	GPM2	5B	AX.89649075	5.48E+07	0.06	2343.60	5.08	0.09
E3	GPM2	5B	AX.89598204	5.48E+07	0.06	2169.94	4.72	0.08
E3	GPM2	5B	AX.89641880	5.51E+07	0.06	2343.60	5.08	0.09
E3	GPM2	5B	AX.89426021	5.30E+07	0.06	2195.79	4.47	0.08
E3	GPM2	5B	AX.89447533	5.30E+07	0.06	2042.91	4.17	0.07
E3	GPM2	5B	X.89391178_OT	5.30E+07	0.06	2195.79	4.47	0.08
E3	GPM2	5B	AX.89531803	5.32E+07	0.06	2278.93	5.02	0.08
E3	GPM2	5B	AX.89622269	5.32E+07	0.06	2278.93	5.02	0.08
E3	GPM2	5B	AX.89378332	5.36E+07	0.06	2128.33	4.70	0.07
E3	GPM2	5B	AX.89563289	5.37E+07	0.07	2033.35	4.56	0.07

E3	GPM2	5B	AX.89527561	5.37E+07	0.06	2278.93	5.02	0.08
E3	GPM2	5B	AX.89717942	5.37E+07	0.06	2278.93	5.02	0.08
E3	GPM2	5B	AX.89592692	5.37E+07	0.07	2017.21	4.32	0.07
E3	GPM2	5B	AX.89417574	5.37E+07	0.06	2128.33	4.70	0.07
E3	GPM2	5B	AX.89616455	3.55E+07	0.46	-827.30	3.07	0.05
E3	GPM2	5B	AX.89326761	5.19E+07	0.05	2221.42	4.35	0.08
E3	GPM2	5B	AX.89575038	5.23E+07	0.06	2195.79	4.47	0.08
E3	GPM2	5B	AX.89325516	5.29E+07	0.05	2218.59	4.31	0.08
E3	GPM2	5B	AX.89755092	5.40E+07	0.06	2278.93	5.02	0.08
E3	GPM2	5B	AX.89487995	5.41E+07	0.06	2128.33	4.70	0.07
E3	GPM2	5B	AX.89503185	5.42E+07	0.06	2128.33	4.70	0.07
E3	GPM2	5B	AX.89717386	5.42E+07	0.06	2278.93	5.02	0.08
E3	GPM2	5B	AX.89561153	5.42E+07	0.06	2278.93	5.02	0.08
E3	GPM2	5B	AX.89733859	5.43E+07	0.05	1859.12	3.20	0.05
E3	GPM2	5B	AX.89391428	5.43E+07	0.06	2278.93	5.02	0.08
E3	GPM2	5B	AX.89561313	5.43E+07	0.06	2278.93	5.02	0.08
E3	GPM2	5B	AX.89695150	5.43E+07	0.06	2278.93	5.02	0.08
E3	GPM2	5B	AX.89310377	5.44E+07	0.06	2278.93	5.02	0.08
E3	GPM2	5B	AX.89575748	5.45E+07	0.07	2033.98	4.52	0.07
E3	GPM2	5B	AX.89382486	5.40E+07	0.07	2017.21	4.32	0.07
E3	GPM2	5B	AX.89630919	5.40E+07	0.06	2278.93	5.02	0.08
E3	GPM2	5B	AX.89618273	5.45E+07	0.07	2033.98	4.52	0.07
E3	GPM2	5B	AX.89357923	5.47E+07	0.06	2343.60	5.08	0.09
E3	GPM2	5B	AX.89388836	5.45E+07	0.06	2174.78	4.82	0.08
E3	GPM2	5B	AX.89570274	5.45E+07	0.06	2174.78	4.82	0.08
E3	GPS	5B	AX.89326761	5.19E+07	0.06	4.83	4.31	0.09
E3	GPS	5B	AX.89575038	5.23E+07	0.07	4.96	4.74	0.10
E3	GPS	5B	AX.89325516	5.29E+07	0.06	5.08	4.68	0.10
E3	GPS	5B	AX.89426021	5.30E+07	0.07	4.96	4.74	0.10
E3	GPS	5B	AX.89447533	5.30E+07	0.07	5.20	5.47	0.11
E3	GPS	5B	X.89391178_OT	5.30E+07	0.07	4.96	4.74	0.10
E3	GPS	5B	AX.89531803	5.32E+07	0.07	4.75	4.63	0.10
E3	GPS	5B	AX.89622269	5.32E+07	0.07	4.75	4.63	0.10
E3	GPS	5B	AX.89378332	5.36E+07	0.08	4.99	5.34	0.11
E3	GPS	5B	AX.89563289	5.37E+07	0.08	4.78	5.21	0.10
E3	GPS	5B	AX.89527561	5.37E+07	0.07	4.75	4.63	0.10
E3	GPS	5B	AX.89717942	5.37E+07	0.07	4.75	4.63	0.10
E3	GPS	5B	AX.89592692	5.37E+07	0.08	4.66	4.83	0.10
E3	GPS	5B	AX.89417574	5.37E+07	0.08	4.99	5.34	0.11
E3	GPS	5B	AX.89382486	5.40E+07	0.08	4.66	4.83	0.10
E3	GPS	5B	AX.89630919	5.40E+07	0.07	4.75	4.63	0.10
E3	GPS	5B	AX.89755092	5.40E+07	0.07	4.75	4.63	0.10
E3	GPS	5B	AX.89487995	5.41E+07	0.08	4.99	5.34	0.11
E3	GPS	5B	AX.89503185	5.42E+07	0.08	4.99	5.34	0.11
E3	GPS	5B	AX.89717386	5.42E+07	0.07	4.75	4.63	0.10
E3	GPS	5B	AX.89561153	5.42E+07	0.07	4.75	4.63	0.10
E3	GPS	5B	AX.89733859	5.43E+07	0.06	4.58	3.94	0.08
E3	GPS	5B	AX.89391428	5.43E+07	0.07	4.75	4.63	0.10
E3	GPS	5B	AX.89561313	5.43E+07	0.07	4.75	4.63	0.10
E3	GPS	5B	AX.89695150	5.43E+07	0.07	4.75	4.63	0.10

E3	GPS	5B	AX.89310377	5.44E+07	0.07	4.75	4.63	0.10
E3	GPS	5B	AX.89575748	5.45E+07	0.08	4.92	5.44	0.11
E3	GPS	5B	AX.89388836	5.45E+07	0.08	4.70	4.75	0.10
E3	GPS	5B	AX.89570274	5.45E+07	0.08	4.70	4.75	0.10
E3	GPS	5B	AX.89618273	5.45E+07	0.08	4.92	5.44	0.11
E3	GPS	5B	AX.89357923	5.47E+07	0.07	5.00	4.93	0.11
E3	GPS	5B	AX.89444793	5.48E+07	0.07	5.00	4.93	0.11
E3	GPS	5B	AX.89569208	5.48E+07	0.07	5.00	4.93	0.11
E3	GPS	5B	AX.89498183	5.48E+07	0.07	5.00	4.93	0.11
E3	GPS	5B	AX.89649075	5.48E+07	0.07	5.00	4.93	0.11
E3	GPS	5B	AX.89598204	5.48E+07	0.08	5.20	5.63	0.12
E3	GPS	5B	AX.89641880	5.51E+07	0.07	5.00	4.93	0.11
E3	GPS	5B	X.89555424_OT	5.30E+08	0.29	2.55	3.37	0.09
E3	GPS	5B	AX.89380190	5.35E+08	0.39	2.29	3.61	0.07
E3	GPS	5B	AX.89772266	5.36E+08	0.43	2.24	3.72	0.06
E3	GPS	5B	AX.89677997	5.36E+08	0.43	2.24	3.72	0.06
E3	GPS	5B	AX.89609004	5.36E+08	0.43	2.24	3.72	0.06
E3	GPS	5B	AX.89701209	5.36E+08	0.43	2.24	3.72	0.06
E3	GPS	5B	AX.89701460	5.36E+08	0.42	2.09	3.23	0.06
E3	GPS	5B	AX.89589110	5.36E+08	0.40	2.07	3.08	0.05
E3	GPS	5B	AX.89544678	5.38E+08	0.38	2.41	3.80	0.08
E3	GPS	5B	AX.89474094	5.38E+08	0.49	-2.24	3.74	0.07
E3	GPS	5B	AX.89707107	5.38E+08	0.48	-2.40	4.24	0.08
E3	GPS	5B	AX.89352707	5.38E+08	0.38	2.41	3.80	0.08
E3	GPS	5B	AX.89388848	5.39E+08	0.48	-2.30	3.90	0.07
E3	GPS	5B	AX.89680182	5.39E+08	0.38	2.41	3.80	0.08
E3	GPS	5B	AX.89548191	5.39E+08	0.38	2.41	3.80	0.08
E3	GPS	5B	AX.89694520	5.38E+08	0.38	2.41	3.80	0.08
E3	GPS	5B	AX.89704009	5.38E+08	0.38	2.41	3.80	0.08
E3	GPS	5B	AX.89537780	5.38E+08	0.38	2.41	3.80	0.08
E3	GPS	5B	AX.89335617	5.38E+08	0.49	-2.35	4.05	0.08
E3	GPS	5B	AX.89425489	5.38E+08	0.48	-2.40	4.24	0.08
E3	GPS	5B	AX.89604018	5.38E+08	0.38	2.41	3.80	0.08
E3	GPS	5B	AX.89772049	5.38E+08	0.48	-2.40	4.24	0.08
E3	GPS	5B	AX.89762142	5.38E+08	0.38	2.35	3.62	0.08
E3	GPS	5B	AX.89372247	5.38E+08	0.38	2.35	3.62	0.08
E3	GPS	5B	AX.89346029	5.39E+08	0.47	-2.38	4.18	0.07
E3	GPS	5B	AX.89651458	5.42E+08	0.26	2.53	3.39	0.07
E3	GPS	5B	AX.89672458	5.42E+08	0.27	2.49	3.36	0.07
E3	GPS	5B	AX.89550030	5.77E+08	0.11	-3.68	4.03	0.03
E3	GY	5B	AX.89326761	5.19E+07	0.05	7.23	4.12	0.08
E3	GY	5B	AX.89575038	5.23E+07	0.06	6.74	3.82	0.07
E3	GY	5B	AX.89325516	5.29E+07	0.05	6.87	3.74	0.07
E3	GY	5B	AX.89426021	5.30E+07	0.06	6.74	3.82	0.07
E3	GY	5B	AX.89447533	5.30E+07	0.06	6.18	3.48	0.07
E3	GY	5B	X.89391178_OT	5.30E+07	0.06	6.74	3.82	0.07
E3	GY	5B	AX.89531803	5.32E+07	0.06	6.85	4.14	0.08
E3	GY	5B	AX.89622269	5.32E+07	0.06	6.85	4.14	0.08
E3	GY	5B	AX.89378332	5.36E+07	0.06	6.31	3.79	0.07
E3	GY	5B	AX.89563289	5.37E+07	0.07	5.94	3.57	0.07

E3	GY	5B	AX.89527561	5.37E+07	0.06	6.85	4.14	0.08
E3	GY	5B	AX.89717942	5.37E+07	0.06	6.85	4.14	0.08
E3	GY	5B	AX.89592692	5.37E+07	0.07	5.97	3.48	0.07
E3	GY	5B	AX.89417574	5.37E+07	0.06	6.31	3.79	0.07
E3	GY	5B	AX.89382486	5.40E+07	0.07	5.97	3.48	0.07
E3	GY	5B	AX.89630919	5.40E+07	0.06	6.85	4.14	0.08
E3	GY	5B	AX.89755092	5.40E+07	0.06	6.85	4.14	0.08
E3	GY	5B	AX.89487995	5.41E+07	0.06	6.31	3.79	0.07
E3	GY	5B	AX.89503185	5.42E+07	0.06	6.31	3.79	0.07
E3	GY	5B	AX.89717386	5.42E+07	0.06	6.85	4.14	0.08
E3	GY	5B	AX.89561153	5.42E+07	0.06	6.85	4.14	0.08
E3	GY	5B	AX.89733859	5.43E+07	0.05	6.08	3.07	0.06
E3	GY	5B	AX.89391428	5.43E+07	0.06	6.85	4.14	0.08
E3	GY	5B	AX.89561313	5.43E+07	0.06	6.85	4.14	0.08
E3	GY	5B	AX.89695150	5.43E+07	0.06	6.85	4.14	0.08
E3	GY	5B	AX.89310377	5.44E+07	0.06	6.85	4.14	0.08
E3	GY	5B	AX.89575748	5.45E+07	0.07	6.28	3.91	0.07
E3	GY	5B	AX.89388836	5.45E+07	0.06	6.80	4.26	0.08
E3	GY	5B	AX.89570274	5.45E+07	0.06	6.80	4.26	0.08
E3	GY	5B	AX.89618273	5.45E+07	0.07	6.28	3.91	0.07
E3	GY	5B	AX.89357923	5.47E+07	0.06	7.32	4.49	0.08
E3	GY	5B	AX.89444793	5.48E+07	0.06	7.32	4.49	0.08
E3	GY	5B	AX.89569208	5.48E+07	0.06	7.32	4.49	0.08
E3	GY	5B	AX.89498183	5.48E+07	0.06	7.32	4.49	0.08
E3	GY	5B	AX.89649075	5.48E+07	0.06	7.32	4.49	0.08
E3	GY	5B	AX.89598204	5.48E+07	0.06	6.69	4.08	0.08
E3	GY	5B	AX.89641880	5.51E+07	0.06	7.32	4.49	0.08
E3	GY	5B	AX.89700382	6.66E+08	0.25	3.17	3.00	0.03
E3	SPM2	5B	AX.89438023	7.08E+07	0.49	-22.13	3.63	0.06
E3	SPM2	5B	AX.89338978	5.99E+08	0.53	-20.03	3.45	0.04
E3	SPM2	5B	AX.89447271	5.99E+08	0.43	18.79	3.02	0.04
E3	SPM2	5B	AX.89318860	6.00E+08	0.43	18.79	3.02	0.04
E3	SPM2	5B	AX.89416640	6.00E+08	0.44	19.28	3.20	0.05
E3	SPM2	5B	AX.89751387	6.87E+08	0.30	21.65	3.26	0.05
E4	GPS	5B	AX.89338284	5.81E+08	0.16	-2.00	3.09	0.06
E4	GPS	5B	AX.89353768	5.83E+08	0.20	-1.81	3.01	0.07
E4	GPS	5B	AX.89540649	5.53E+08	0.19	-1.98	3.11	0.07
E4	GPS	5B	AX.89365125	5.62E+08	0.22	-1.94	3.41	0.07
E4	SPM2	5B	AX.89616594	2.92E+08	0.06	44.21	4.46	0.11
E4	SPM2	5B	AX.89474859	6.81E+08	0.07	36.73	3.41	0.05
E4	TKW	5B	X.89705636_OT	6.97E+06	0.32	0.88	3.14	0.00
E4	TKW	5B	AX.89572203	4.91E+08	0.13	-1.16	3.04	0.04
E1	GY	5D	AX.89432860	2.24E+08	0.50	-2.63	3.94	0.06
E1	GY	5D	AX.89342736	4.39E+07	0.47	2.66	4.02	0.06
E1	GY	5D	AX.89668244	9.43E+07	0.48	2.29	3.11	0.05
E1	GY	5D	AX.86170182	2.44E+08	0.47	2.26	3.03	0.05
E1	GY	5D	AX.89742771	7.63E+07	0.50	-2.52	3.68	0.06
E1	GY	5D	AX.89697957	4.39E+07	0.47	2.66	4.02	0.06
E1	GY	5D	AX.89658445	1.69E+08	0.48	2.49	3.59	0.06
E1	GY	5D	AX.89735566	9.36E+07	0.49	-2.75	4.27	0.07

E1	GY	5D	AX.89742771	7.63E+07	0.50	-2.52	3.68	0.06
E1	GY	5D	AX.89388421	9.76E+07	0.49	-2.58	3.83	0.06
E1	GY	5D	AX.89697957	4.39E+07	0.47	2.66	4.02	0.06
E1	GY	5D	AX.89489167	2.19E+08	0.49	-2.69	4.10	0.07
E1	GY	5D	AX.89687953	5.01E+08	0.26	3.35	4.75	0.09
E1	GY	5D	AX.89448878	2.24E+08	0.50	-2.56	3.77	0.06
E1	GY	5D	AX.89755586	1.13E+08	0.49	-2.69	4.10	0.07
E1	GY	5D	AX.89508999	2.11E+08	0.49	-2.69	4.10	0.07
E1	GY	5D	AX.89357131	4.38E+07	0.47	2.66	4.02	0.06
E1	GY	5D	AX.89409385	1.69E+08	0.46	2.42	3.40	0.05
E1	GY	5D	AX.89452608	5.04E+08	0.27	2.74	3.45	0.06
E1	GY	5D	AX.89316308	2.33E+08	0.47	2.39	3.34	0.05
E1	GY	5D	AX.89757300	8.04E+07	0.47	-2.64	3.96	0.06
E1	GY	5D	AX.89342736	4.39E+07	0.47	2.66	4.02	0.06
E1	GY	5D	AX.89698977	4.38E+07	0.47	2.66	4.02	0.06
E1	GY	5D	AX.89454691	4.30E+07	0.16	-3.18	3.19	0.05
E1	GY	5D	AX.89542052	7.53E+07	0.50	-2.52	3.68	0.06
E1	GY	5D	AX.89658214	1.04E+08	0.49	-2.69	4.10	0.07
E1	GY	5D	AX.89525078	1.21E+08	0.44	2.36	3.24	0.06
E1	GY	5D	AX.89698977	4.38E+07	0.47	2.66	4.02	0.06
E1	GY	5D	AX.89336477	1.85E+08	0.49	-2.69	4.10	0.07
E1	GY	5D	AX.89425952	2.11E+08	0.49	-2.69	4.10	0.07
E1	GY	5D	AX.89380347	2.05E+08	0.47	2.51	3.65	0.06
E1	GY	5D	AX.89535294	1.38E+08	0.49	-2.69	4.10	0.07
E1	GY	5D	AX.89357131	4.38E+07	0.47	2.66	4.02	0.06
E1	GY	5D	AX.89330743	1.98E+08	0.46	2.78	4.34	0.07
E1	GY	5D	AX.89401495	4.39E+07	0.48	2.64	3.96	0.06
E1	GY	5D	AX.89323983	2.25E+08	0.47	2.39	3.34	0.05
E1	GY	5D	AX.89316308	2.33E+08	0.47	2.39	3.34	0.05
E1	GY	5D	AX.89495600	9.72E+07	0.50	-2.52	3.68	0.06
E1	GY	5D	AX.89495600	9.72E+07	0.50	-2.52	3.68	0.06
E1	GY	5D	AX.86170182	2.44E+08	0.47	2.26	3.03	0.05
E1	GY	5D	AX.89366425	1.75E+08	0.47	2.51	3.65	0.06
E1	GY	5D	AX.89366425	1.75E+08	0.47	2.51	3.65	0.06
E1	GY	5D	AX.89488601	4.38E+07	0.47	2.58	3.80	0.06
E1	GY	5D	AX.89384589	2.10E+08	0.48	2.49	3.59	0.06
E1	GY	5D	AX.89456254	1.29E+08	0.49	-2.69	4.10	0.07
E1	GY	5D	AX.89425952	2.11E+08	0.49	-2.69	4.10	0.07
E1	GY	5D	AX.89755586	1.13E+08	0.49	-2.69	4.10	0.07
E1	GY	5D	AX.89325957	4.38E+07	0.47	2.66	4.02	0.06
E1	GY	5D	AX.89721683	1.13E+08	0.49	-2.69	4.10	0.07
E1	GY	5D	AX.89313623	4.37E+07	0.47	2.66	4.02	0.06
E1	GY	5D	AX.89391478	1.84E+08	0.49	-2.65	4.00	0.06
E1	GY	5D	AX.89540252	8.03E+07	0.50	2.39	3.35	0.05
E1	GY	5D	AX.89435838	2.12E+08	0.47	2.51	3.65	0.06
E1	GY	5D	AX.89323827	1.14E+08	0.49	-2.69	4.10	0.07
E1	GY	5D	AX.89552613	1.16E+08	0.48	-2.62	3.93	0.06
E1	GY	5D	AX.89320147	4.38E+07	0.47	2.58	3.80	0.06
E1	GY	5D	AX.89770647	1.80E+08	0.49	-2.69	4.10	0.07
E1	GY	5D	AX.89448878	2.24E+08	0.50	-2.56	3.77	0.06

E1	GY	5D	AX.89542052	7.53E+07	0.50	-2.52	3.68	0.06
E1	GY	5D	AX.89489167	2.19E+08	0.49	-2.69	4.10	0.07
E1	GY	5D	AX.89472078	1.15E+08	0.49	-2.69	4.10	0.07
E1	GY	5D	AX.89768531	2.15E+08	0.47	2.51	3.65	0.06
E1	GY	5D	AX.89437950	9.29E+07	0.48	-2.67	4.06	0.06
E1	GY	5D	AX.89721683	1.13E+08	0.49	-2.69	4.10	0.07
E1	GY	5D	AX.89486678	4.41E+07	0.47	2.48	3.55	0.06
E1	GY	5D	AX.89486678	4.41E+07	0.47	2.48	3.55	0.06
E1	GY	5D	AX.89391478	1.84E+08	0.49	-2.65	4.00	0.06
E1	GY	5D	AX.89469909	2.42E+08	0.46	2.34	3.21	0.05
E1	GY	5D	AX.89544499	8.67E+07	0.49	-2.75	4.27	0.07
E1	GY	5D	AX.89544499	8.67E+07	0.49	-2.75	4.27	0.07
E1	GY	5D	AX.89424238	4.39E+07	0.47	2.58	3.80	0.06
E1	GY	5D	AX.89357500	4.39E+07	0.47	2.58	3.80	0.06
E1	GY	5D	AX.89346059	1.84E+08	0.49	-2.65	4.00	0.06
E1	GY	5D	AX.89471124	2.21E+08	0.49	-2.52	3.67	0.06
E1	GY	5D	AX.89568920	1.15E+08	0.49	-2.65	4.00	0.06
E1	GY	5D	AX.89723970	2.30E+08	0.47	2.39	3.34	0.05
E1	GY	5D	AX.89569343	1.02E+08	0.49	-2.69	4.10	0.07
E1	GY	5D	AX.89553654	7.76E+07	0.50	-2.52	3.68	0.06
E1	GY	5D	AX.89313623	4.37E+07	0.47	2.66	4.02	0.06
E1	GY	5D	AX.89594372	8.94E+07	0.49	-2.37	3.29	0.05
E1	GY	5D	AX.89546668	9.79E+07	0.49	-2.58	3.83	0.06
E1	GY	5D	AX.89562719	7.68E+07	0.50	-2.52	3.68	0.06
E1	GY	5D	AX.89460527	9.83E+07	0.48	2.38	3.33	0.05
E1	GY	5D	AX.89336477	1.85E+08	0.49	-2.69	4.10	0.07
E1	GY	5D	AX.89382937	8.82E+07	0.50	-2.52	3.68	0.06
E1	GY	5D	AX.89369146	8.05E+07	0.50	-2.52	3.68	0.06
E1	GY	5D	AX.89478204	1.04E+08	0.49	-2.69	4.10	0.07
E1	GY	5D	AX.89443733	1.85E+08	0.48	2.49	3.59	0.06
E1	GY	5D	AX.89454691	4.30E+07	0.16	-3.18	3.19	0.05
E1	GY	5D	AX.89536739	7.82E+07	0.50	-2.52	3.68	0.06
E1	GY	5D	AX.89384589	2.10E+08	0.48	2.49	3.59	0.06
E1	GY	5D	AX.89552613	1.16E+08	0.48	-2.62	3.93	0.06
E1	GY	5D	AX.89433215	1.19E+08	0.49	-2.69	4.10	0.07
E1	GY	5D	AX.89406002	1.66E+08	0.49	-2.69	4.10	0.07
E1	GY	5D	AX.89720229	1.09E+08	0.49	-2.65	4.00	0.06
E1	GY	5D	AX.89735566	9.36E+07	0.49	-2.75	4.27	0.07
E1	GY	5D	AX.89731768	4.37E+07	0.48	2.70	4.13	0.07
E1	GY	5D	AX.89633037	8.68E+07	0.50	-2.52	3.68	0.06
E1	GY	5D	AX.89658214	1.04E+08	0.49	-2.69	4.10	0.07
E1	GY	5D	AX.89648256	2.12E+08	0.47	2.51	3.65	0.06
E1	GY	5D	AX.89682193	4.39E+07	0.47	2.58	3.80	0.06
E1	GY	5D	AX.89375308	4.37E+07	0.47	2.66	4.02	0.06
E1	GY	5D	AX.89382301	5.20E+08	0.28	-2.49	3.01	0.05
E1	GY	5D	AX.89438522	4.39E+07	0.47	2.58	3.80	0.06
E1	GY	5D	AX.89400139	2.17E+08	0.47	2.51	3.65	0.06
E1	GY	5D	AX.89408003	2.29E+08	0.49	2.58	3.83	0.06
E1	GY	5D	AX.89540252	8.03E+07	0.50	2.39	3.35	0.05
E1	GY	5D	AX.89380347	2.05E+08	0.47	2.51	3.65	0.06

E1	GY	5D	AX.89369146	8.05E+07	0.50	-2.52	3.68	0.06
E1	GY	5D	AX.89387650	4.41E+07	0.47	2.50	3.61	0.06
E1	GY	5D	AX.89401087	9.25E+07	0.50	-2.52	3.68	0.06
E1	GY	5D	AX.89401495	4.39E+07	0.48	2.64	3.96	0.06
E1	GY	5D	AX.89470370	2.44E+08	0.47	2.26	3.03	0.05
E1	GY	5D	AX.89734213	4.38E+07	0.47	2.66	4.02	0.06
E1	GY	5D	AX.89734213	4.38E+07	0.47	2.66	4.02	0.06
E1	GY	5D	AX.89575695	1.57E+08	0.49	-2.69	4.10	0.07
E1	GY	5D	AX.89588502	4.38E+07	0.47	2.58	3.80	0.06
E1	GY	5D	AX.89320147	4.38E+07	0.47	2.58	3.80	0.06
E1	GY	5D	AX.89495145	4.41E+07	0.47	2.66	4.02	0.06
E1	GY	5D	AX.89471124	2.21E+08	0.49	-2.52	3.67	0.06
E1	GY	5D	AX.89661655	4.38E+07	0.47	2.48	3.55	0.06
E1	GY	5D	AX.89553654	7.76E+07	0.50	-2.52	3.68	0.06
E1	GY	5D	AX.89768531	2.15E+08	0.47	2.51	3.65	0.06
E1	GY	5D	AX.89508999	2.11E+08	0.49	-2.69	4.10	0.07
E1	GY	5D	AX.89395350	1.68E+08	0.49	-2.69	4.10	0.07
E1	GY	5D	AX.89382937	8.82E+07	0.50	-2.52	3.68	0.06
E1	GY	5D	AX.89404149	7.38E+07	0.50	-2.52	3.68	0.06
E1	GY	5D	AX.89388421	9.76E+07	0.49	-2.58	3.83	0.06
E1	GY	5D	AX.89673057	2.05E+08	0.49	-2.69	4.10	0.07
E1	GY	5D	AX.89416752	1.22E+08	0.49	-2.69	4.10	0.07
E1	GY	5D	AX.89710686	4.39E+07	0.47	2.59	3.83	0.06
E1	GY	5D	AX.89762986	1.06E+08	0.48	2.49	3.59	0.06
E1	GY	5D	AX.89511658	7.41E+07	0.50	-2.52	3.68	0.06
E1	GY	5D	AX.89568887	9.40E+07	0.49	-2.50	3.61	0.06
E1	GY	5D	AX.89569343	1.02E+08	0.49	-2.69	4.10	0.07
E1	GY	5D	AX.89727907	9.25E+07	0.43	2.49	3.53	0.05
E1	GY	5D	AX.89727907	9.25E+07	0.43	2.49	3.53	0.05
E1	GY	5D	AX.89325677	5.56E+07	0.49	2.42	3.42	0.05
E1	GY	5D	AX.89739353	4.41E+07	0.47	2.52	3.66	0.06
E1	GY	5D	AX.89570601	1.52E+08	0.49	-2.69	4.10	0.07
E1	GY	5D	AX.89774939	4.38E+07	0.47	2.58	3.80	0.06
E1	GY	5D	AX.89774939	4.38E+07	0.47	2.58	3.80	0.06
E1	GY	5D	AX.89664265	4.38E+07	0.47	2.58	3.80	0.06
E1	GY	5D	AX.89668244	9.43E+07	0.48	2.29	3.11	0.05
E1	GY	5D	AX.89664840	8.82E+07	0.50	-2.52	3.68	0.06
E1	GY	5D	AX.89443965	1.94E+08	0.49	-2.69	4.10	0.07
E1	GY	5D	AX.89432860	2.24E+08	0.50	-2.63	3.94	0.06
E1	GY	5D	AX.89494925	4.39E+07	0.47	2.58	3.80	0.06
E1	GY	5D	AX.89657536	4.38E+07	0.47	2.58	3.80	0.06
E1	GY	5D	AX.89445109	5.04E+08	0.27	2.74	3.45	0.06
E1	GY	5D	AX.89743508	1.11E+08	0.48	-2.61	3.90	0.06
E1	GY	5D	AX.89707797	4.41E+07	0.47	2.43	3.43	0.05
E1	GY	5D	AX.89731768	4.37E+07	0.48	2.70	4.13	0.07
E1	GY	5D	AX.89351168	2.42E+08	0.46	2.31	3.14	0.05
E1	GY	5D	AX.89707299	7.55E+07	0.50	-2.52	3.68	0.06
E1	GY	5D	AX.89434036	1.24E+08	0.49	-2.69	4.10	0.07
E1	GY	5D	AX.89382301	5.20E+08	0.28	-2.49	3.01	0.05
E1	GY	5D	AX.89562719	7.68E+07	0.50	-2.52	3.68	0.06

E1	GY	5D	AX.89558521	2.02E+08	0.47	2.51	3.65	0.06
E1	GY	5D	AX.89401087	9.25E+07	0.50	-2.52	3.68	0.06
E1	GY	5D	AX.89420794	9.82E+07	0.49	-2.58	3.83	0.06
E1	GY	5D	AX.89424238	4.39E+07	0.47	2.58	3.80	0.06
E1	GY	5D	AX.89666291	2.33E+08	0.49	-2.66	4.03	0.07
E1	GY	5D	AX.89424526	7.67E+07	0.48	-2.61	3.90	0.06
E1	GY	5D	AX.89320362	1.03E+08	0.49	-2.69	4.10	0.07
E1	GY	5D	AX.89576275	2.49E+08	0.49	-2.40	3.38	0.05
E1	GY	5D	AX.89661655	4.38E+07	0.47	2.48	3.55	0.06
E1	GY	5D	AX.89723970	2.30E+08	0.47	2.39	3.34	0.05
E1	GY	5D	AX.89554039	4.37E+07	0.47	2.66	4.02	0.06
E1	GY	5D	AX.89403223	1.20E+08	0.49	-2.69	4.10	0.07
E1	GY	5D	AX.89403223	1.20E+08	0.49	-2.69	4.10	0.07
E1	GY	5D	AX.89700339	1.25E+08	0.49	-2.69	4.10	0.07
E1	GY	5D	AX.89700339	1.25E+08	0.49	-2.69	4.10	0.07
E1	GY	5D	AX.89395350	1.68E+08	0.49	-2.69	4.10	0.07
E1	GY	5D	AX.89687953	5.01E+08	0.26	3.35	4.75	0.09
E1	GY	5D	AX.89333326	2.32E+08	0.47	2.39	3.34	0.05
E1	GY	5D	AX.89676090	2.00E+08	0.47	2.51	3.65	0.06
E1	GY	5D	AX.89428535	1.12E+08	0.49	-2.69	4.10	0.07
E1	GY	5D	AX.89544327	2.16E+08	0.49	-2.68	4.09	0.07
E1	GY	5D	AX.89323983	2.25E+08	0.47	2.39	3.34	0.05
E1	GY	5D	AX.89462453	1.94E+08	0.47	2.51	3.65	0.06
E1	GY	5D	AX.89677346	2.10E+08	0.49	-2.69	4.10	0.07
E1	GY	5D	AX.89523007	4.37E+07	0.47	2.58	3.80	0.06
E1	GY	5D	AX.89346059	1.84E+08	0.49	-2.65	4.00	0.06
E1	GY	5D	AX.89429373	2.31E+08	0.47	2.39	3.34	0.05
E1	GY	5D	AX.89417529	2.25E+08	0.49	2.58	3.83	0.06
E1	GY	5D	AX.89417529	2.25E+08	0.49	2.58	3.83	0.06
E1	GY	5D	AX.89346238	2.15E+08	0.47	2.51	3.65	0.06
E1	GY	5D	AX.89346238	2.15E+08	0.47	2.51	3.65	0.06
E1	GY	5D	AX.89335224	4.41E+07	0.47	2.58	3.80	0.06
E1	GY	5D	AX.89452608	5.04E+08	0.27	2.74	3.45	0.06
E1	GY	5D	AX.89716222	1.20E+08	0.49	-2.69	4.10	0.07
E1	GY	5D	AX.89766690	2.29E+08	0.50	-2.56	3.77	0.06
E1	GY	5D	AX.89325677	5.56E+07	0.49	2.42	3.42	0.05
E1	GY	5D	AX.89654927	2.00E+08	0.47	2.51	3.65	0.06
E1	GY	5D	AX.89325957	4.38E+07	0.47	2.66	4.02	0.06
E1	GY	5D	AX.89459312	1.30E+08	0.50	-2.55	3.75	0.06
E1	GY	5D	AX.89570601	1.52E+08	0.49	-2.69	4.10	0.07
E1	GY	5D	AX.89360590	8.89E+07	0.49	-2.61	3.90	0.06
E1	GY	5D	AX.89420287	4.41E+07	0.47	2.59	3.83	0.06
E1	GY	5D	AX.89396564	9.74E+07	0.48	-2.34	3.23	0.05
E1	GY	5D	AX.89433215	1.19E+08	0.49	-2.69	4.10	0.07
E1	GY	5D	AX.89498706	4.41E+07	0.47	2.56	3.76	0.06
E1	GY	5D	AX.89498706	4.41E+07	0.47	2.56	3.76	0.06
E1	GY	5D	AX.89406002	1.66E+08	0.49	-2.69	4.10	0.07
E1	GY	5D	AX.89743508	1.11E+08	0.48	-2.61	3.90	0.06
E1	GY	5D	AX.89488601	4.38E+07	0.47	2.58	3.80	0.06
E1	GY	5D	AX.89351168	2.42E+08	0.46	2.31	3.14	0.05

E1	GY	5D	AX.89328741	4.39E+07	0.47	2.58	3.80	0.06
E1	GY	5D	AX.89558521	2.02E+08	0.47	2.51	3.65	0.06
E1	GY	5D	AX.89364151	1.18E+08	0.49	-2.69	4.10	0.07
E1	GY	5D	AX.89364151	1.18E+08	0.49	-2.69	4.10	0.07
E1	GY	5D	AX.89622218	9.79E+07	0.49	-2.58	3.83	0.06
E1	GY	5D	AX.89469355	5.15E+08	0.06	-5.39	3.80	0.06
E1	GY	5D	AX.89387650	4.41E+07	0.47	2.50	3.61	0.06
E1	GY	5D	AX.89352946	4.38E+07	0.47	2.58	3.80	0.06
E1	GY	5D	AX.89469909	2.42E+08	0.46	2.34	3.21	0.05
E1	GY	5D	AX.89330743	1.98E+08	0.46	2.78	4.34	0.07
E1	GY	5D	AX.89494925	4.39E+07	0.47	2.58	3.80	0.06
E1	GY	5D	AX.89710686	4.39E+07	0.47	2.59	3.83	0.06
E1	GY	5D	AX.89757300	8.04E+07	0.47	-2.64	3.96	0.06
E1	GY	5D	AX.89495145	4.41E+07	0.47	2.66	4.02	0.06
E1	GY	5D	AX.89564905	1.71E+08	0.49	-2.77	4.33	0.07
E1	GY	5D	AX.89424526	7.67E+07	0.48	-2.61	3.90	0.06
E1	GY	5D	AX.89479940	4.38E+07	0.47	2.66	4.02	0.06
E1	GY	5D	AX.89479940	4.38E+07	0.47	2.66	4.02	0.06
E1	GY	5D	AX.89770647	1.80E+08	0.49	-2.69	4.10	0.07
E1	GY	5D	AX.89711492	1.34E+08	0.49	-2.69	4.10	0.07
E1	GY	5D	AX.89554039	4.37E+07	0.47	2.66	4.02	0.06
E1	GY	5D	AX.89460527	9.83E+07	0.48	2.38	3.33	0.05
E1	GY	5D	AX.89566469	9.25E+07	0.48	-2.67	4.06	0.06
E1	GY	5D	AX.89566469	9.25E+07	0.48	-2.67	4.06	0.06
E1	GY	5D	AX.89438522	4.39E+07	0.47	2.58	3.80	0.06
E1	GY	5D	AX.89473397	4.37E+07	0.47	2.66	4.02	0.06
E1	GY	5D	AX.89404149	7.38E+07	0.50	-2.52	3.68	0.06
E1	GY	5D	AX.89725494	1.84E+08	0.49	-2.65	4.00	0.06
E1	GY	5D	AX.89521924	8.85E+07	0.47	-2.64	3.96	0.06
E1	GY	5D	AX.89578902	1.33E+08	0.49	-2.72	4.20	0.07
E1	GY	5D	AX.89544327	2.16E+08	0.49	-2.68	4.09	0.07
E1	GY	5D	AX.89568887	9.40E+07	0.49	-2.50	3.61	0.06
E1	GY	5D	AX.89593351	1.75E+08	0.47	2.51	3.65	0.06
E1	GY	5D	AX.89452264	1.71E+08	0.49	-2.69	4.10	0.07
E1	GY	5D	AX.89601604	4.41E+07	0.47	2.58	3.80	0.06
E1	GY	5D	AX.89601604	4.41E+07	0.47	2.58	3.80	0.06
E1	GY	5D	AX.89633037	8.68E+07	0.50	-2.52	3.68	0.06
E1	GY	5D	AX.89633560	7.67E+07	0.47	-2.64	3.97	0.06
E1	GY	5D	AX.89546668	9.79E+07	0.49	-2.58	3.83	0.06
E1	GY	5D	AX.89767889	1.94E+08	0.48	2.49	3.59	0.06
E1	GY	5D	AX.89595297	1.10E+08	0.49	-2.65	4.00	0.06
E1	GY	5D	AX.89408003	2.29E+08	0.49	2.58	3.83	0.06
E1	GY	5D	AX.89360590	8.89E+07	0.49	-2.61	3.90	0.06
E1	GY	5D	AX.89443733	1.85E+08	0.48	2.49	3.59	0.06
E1	GPS	5D	AX.89443998	1.88E+08	0.49	-1.11	3.01	0.05
E1	GY	5D	AX.89426929	4.38E+07	0.47	2.66	4.02	0.06
E1	GY	5D	AX.89568303	7.60E+07	0.50	-2.52	3.68	0.06
E1	GY	5D	AX.89568303	7.60E+07	0.50	-2.52	3.68	0.06
E1	GY	5D	AX.89373750	1.09E+08	0.49	-2.69	4.10	0.07
E1	GY	5D	AX.89375041	2.26E+08	0.47	2.39	3.34	0.05

E1	GPS	5D	AX.89720229	1.09E+08	0.49	-1.11	3.01	0.05
E1	GY	5D	AX.89658445	1.69E+08	0.48	2.49	3.59	0.06
E1	GPS	5D	AX.89424526	7.67E+07	0.48	-1.12	3.10	0.05
E1	GY	5D	AX.89400139	2.17E+08	0.47	2.51	3.65	0.06
E1	GY	5D	AX.89412202	4.41E+07	0.50	-2.25	3.02	0.05
E1	GY	5D	AX.89679562	7.44E+07	0.50	-2.52	3.68	0.06
E1	GY	5D	AX.89679562	7.44E+07	0.50	-2.52	3.68	0.06
E1	GY	5D	AX.89352946	4.38E+07	0.47	2.58	3.80	0.06
E1	GY	5D	AX.89575695	1.57E+08	0.49	-2.69	4.10	0.07
E1	GY	5D	AX.89730596	1.08E+08	0.47	2.35	3.24	0.05
E1	GY	5D	AX.89666291	2.33E+08	0.49	-2.66	4.03	0.07
E1	GY	5D	AX.89553085	2.28E+08	0.50	-2.79	4.38	0.07
E1	GY	5D	AX.89416417	4.37E+07	0.47	2.58	3.80	0.06
E1	GY	5D	AX.89731614	2.05E+08	0.47	2.51	3.65	0.06
E1	GY	5D	AX.89724236	1.37E+08	0.49	-2.69	4.10	0.07
E1	GY	5D	AX.89724236	1.37E+08	0.49	-2.69	4.10	0.07
E1	GPS	5D	AX.89459312	1.30E+08	0.50	-1.13	3.11	0.05
E1	GY	5D	AX.89680171	1.17E+08	0.49	-2.69	4.10	0.07
E1	GY	5D	AX.89680171	1.17E+08	0.49	-2.69	4.10	0.07
E1	GY	5D	AX.89662378	1.04E+08	0.49	-2.69	4.10	0.07
E1	GY	5D	AX.89662378	1.04E+08	0.49	-2.69	4.10	0.07
E1	GY	5D	AX.89333326	2.32E+08	0.47	2.39	3.34	0.05
E1	GY	5D	AX.89676090	2.00E+08	0.47	2.51	3.65	0.06
E1	GPS	5D	AX.89766690	2.29E+08	0.50	-1.12	3.08	0.05
E1	GY	5D	AX.89428535	1.12E+08	0.49	-2.69	4.10	0.07
E1	GY	5D	AX.89578902	1.33E+08	0.49	-2.72	4.20	0.07
E1	GPS	5D	AX.89544327	2.16E+08	0.49	-1.10	3.00	0.05
E1	GY	5D	AX.89664840	8.82E+07	0.50	-2.52	3.68	0.06
E1	GPS	5D	AX.89391478	1.84E+08	0.49	-1.11	3.01	0.05
E1	GY	5D	AX.89673057	2.05E+08	0.49	-2.69	4.10	0.07
E1	GY	5D	AX.89526837	1.14E+08	0.49	-2.69	4.10	0.07
E1	GY	5D	AX.89660434	4.38E+07	0.47	2.58	3.80	0.06
E1	GY	5D	AX.89416752	1.22E+08	0.49	-2.69	4.10	0.07
E1	GY	5D	AX.89462453	1.94E+08	0.47	2.51	3.65	0.06
E1	GY	5D	AX.89357500	4.39E+07	0.47	2.58	3.80	0.06
E1	GY	5D	AX.89523007	4.37E+07	0.47	2.58	3.80	0.06
E1	GY	5D	AX.89762986	1.06E+08	0.48	2.49	3.59	0.06
E1	GPS	5D	AX.89666291	2.33E+08	0.49	-1.16	3.27	0.06
E1	GPS	5D	AX.89409385	1.69E+08	0.46	1.13	3.10	0.05
E1	GY	5D	AX.89511658	7.41E+07	0.50	-2.52	3.68	0.06
E1	GY	5D	AX.89568606	1.06E+08	0.49	-2.69	4.10	0.07
E1	GPS	5D	AX.89576275	2.49E+08	0.49	-1.19	3.40	0.06
E1	GPS	5D	AX.89725494	1.84E+08	0.49	-1.11	3.01	0.05
E1	GY	5D	AX.89766690	2.29E+08	0.50	-2.56	3.77	0.06
E1	GY	5D	AX.89716818	1.19E+08	0.49	-2.69	4.10	0.07
E1	GY	5D	AX.89420375	7.44E+07	0.50	-2.52	3.68	0.06
E1	GY	5D	AX.89420375	7.44E+07	0.50	-2.52	3.68	0.06
E1	GY	5D	AX.89657536	4.38E+07	0.47	2.58	3.80	0.06
E1	GY	5D	AX.89445109	5.04E+08	0.27	2.74	3.45	0.06
E1	GY	5D	AX.89707797	4.41E+07	0.47	2.43	3.43	0.05

E1	GY	5D	AX.89480523	4.41E+07	0.47	2.66	4.02	0.06
E1	GY	5D	AX.89480523	4.41E+07	0.47	2.66	4.02	0.06
E1	GY	5D	AX.89456254	1.29E+08	0.49	-2.69	4.10	0.07
E1	GY	5D	AX.89711519	4.41E+07	0.47	2.58	3.80	0.06
E1	GY	5D	AX.89434036	1.24E+08	0.49	-2.69	4.10	0.07
E1	GY	5D	AX.89412202	4.41E+07	0.50	-2.25	3.02	0.05
E1	GY	5D	AX.89565036	1.58E+08	0.47	2.51	3.65	0.06
E1	GY	5D	AX.89565036	1.58E+08	0.47	2.51	3.65	0.06
E1	GY	5D	AX.89638076	1.31E+08	0.49	-2.69	4.10	0.07
E1	GY	5D	AX.89420794	9.82E+07	0.49	-2.58	3.83	0.06
E1	GY	5D	AX.89548887	1.28E+08	0.49	-2.33	3.21	0.05
E1	GY	5D	AX.89588502	4.38E+07	0.47	2.58	3.80	0.06
E1	GY	5D	AX.89353576	9.16E+07	0.49	-2.75	4.27	0.07
E1	GY	5D	AX.89320362	1.03E+08	0.49	-2.69	4.10	0.07
E1	GY	5D	AX.89416417	4.37E+07	0.47	2.58	3.80	0.06
E1	GPS	5D	AX.89573386	2.22E+08	0.47	-1.18	3.36	0.06
E1	GY	5D	AX.89565647	2.19E+08	0.47	2.47	3.54	0.06
E1	GY	5D	AX.89772754	4.38E+07	0.47	2.58	3.80	0.06
E1	GY	5D	AX.89725494	1.84E+08	0.49	-2.65	4.00	0.06
E1	GY	5D	AX.89521924	8.85E+07	0.47	-2.64	3.96	0.06
E1	GY	5D	AX.89762284	8.25E+07	0.47	-2.64	3.96	0.06
E1	GY	5D	AX.89660113	4.38E+07	0.47	2.66	4.02	0.06
E1	GY	5D	AX.89660113	4.38E+07	0.47	2.66	4.02	0.06
E1	GY	5D	AX.89323827	1.14E+08	0.49	-2.69	4.10	0.07
E1	GY	5D	AX.89689324	1.84E+08	0.49	-2.65	4.00	0.06
E1	GY	5D	AX.89677346	2.10E+08	0.49	-2.69	4.10	0.07
E1	GY	5D	AX.89511566	1.12E+08	0.47	2.51	3.65	0.06
E1	GY	5D	AX.89568920	1.15E+08	0.49	-2.65	4.00	0.06
E1	GY	5D	AX.89429887	4.38E+07	0.47	2.66	4.02	0.06
E1	GY	5D	AX.89429887	4.38E+07	0.47	2.66	4.02	0.06
E1	GY	5D	AX.89716222	1.20E+08	0.49	-2.69	4.10	0.07
E1	GY	5D	AX.89535294	1.38E+08	0.49	-2.69	4.10	0.07
E1	GY	5D	AX.89394993	4.39E+07	0.47	2.58	3.80	0.06
E1	GY	5D	AX.89394993	4.39E+07	0.47	2.58	3.80	0.06
E1	GY	5D	AX.89359879	1.55E+08	0.47	2.51	3.65	0.06
E1	GY	5D	AX.89359879	1.55E+08	0.47	2.51	3.65	0.06
E1	GPS	5D	AX.89346059	1.84E+08	0.49	-1.11	3.01	0.05
E1	GY	5D	AX.89536693	1.51E+08	0.49	-2.69	4.10	0.07
E1	GY	5D	AX.89536693	1.51E+08	0.49	-2.69	4.10	0.07
E1	GY	5D	AX.89420287	4.41E+07	0.47	2.59	3.83	0.06
E1	GY	5D	AX.89396564	9.74E+07	0.48	-2.34	3.23	0.05
E1	GY	5D	AX.89681465	2.03E+08	0.47	2.51	3.63	0.06
E1	GY	5D	AX.89328741	4.39E+07	0.47	2.58	3.80	0.06
E1	GY	5D	AX.89617933	1.84E+08	0.48	2.45	3.49	0.05
E1	GY	5D	AX.89617933	1.84E+08	0.48	2.45	3.49	0.05
E1	GY	5D	AX.89755860	4.41E+07	0.47	2.58	3.80	0.06
E1	GY	5D	AX.89469355	5.15E+08	0.06	-5.39	3.80	0.06
E1	GY	5D	AX.89721992	4.41E+07	0.47	2.58	3.80	0.06
E1	GY	5D	AX.89721992	4.41E+07	0.47	2.58	3.80	0.06
E1	GY	5D	AX.89435838	2.12E+08	0.47	2.51	3.65	0.06

E1	GY	5D	AX.89564905	1.71E+08	0.49	-2.77	4.33	0.07
E1	GY	5D	AX.89507780	8.85E+07	0.50	2.47	3.56	0.06
E1	GY	5D	AX.89507780	8.85E+07	0.50	2.47	3.56	0.06
E1	GY	5D	AX.89711492	1.34E+08	0.49	-2.69	4.10	0.07
E1	GY	5D	AX.89472078	1.15E+08	0.49	-2.69	4.10	0.07
E1	GY	5D	AX.89739353	4.41E+07	0.47	2.52	3.66	0.06
E1	GPM2	5D	AX.89426929	4.38E+07	0.47	583.37	3.05	0.05
E1	GY	5D	AX.89473397	4.37E+07	0.47	2.66	4.02	0.06
E1	GY	5D	AX.89623625	4.41E+07	0.47	2.48	3.55	0.06
E1	GY	5D	AX.89623625	4.41E+07	0.47	2.48	3.55	0.06
E1	GY	5D	AX.89511566	1.12E+08	0.47	2.51	3.65	0.06
E1	GY	5D	AX.89593351	1.75E+08	0.47	2.51	3.65	0.06
E1	GY	5D	AX.89335224	4.41E+07	0.47	2.58	3.80	0.06
E1	GPM2	5D	AX.89473397	4.37E+07	0.47	583.37	3.05	0.05
E1	GY	5D	AX.89594372	8.94E+07	0.49	-2.37	3.29	0.05
E1	GY	5D	AX.89633560	7.67E+07	0.47	-2.64	3.97	0.06
E1	GY	5D	AX.89654927	2.00E+08	0.47	2.51	3.65	0.06
E1	GY	5D	AX.89767889	1.94E+08	0.48	2.49	3.59	0.06
E1	GPS	5D	AX.89361116	2.76E+08	0.25	1.30	3.07	0.05
E1	GPS	5D	AX.89552613	1.16E+08	0.48	-1.13	3.12	0.05
E1	GPS	5D	AX.89595297	1.10E+08	0.49	-1.11	3.01	0.05
E1	GY	5D	AX.89536739	7.82E+07	0.50	-2.52	3.68	0.06
E1	GPS	5D	AX.89578902	1.33E+08	0.49	-1.13	3.11	0.05
E1	GY	5D	AX.89373750	1.09E+08	0.49	-2.69	4.10	0.07
E1	GPS	5D	AX.89701251	2.96E+08	0.25	1.30	3.10	0.05
E1	GY	5D	AX.89720229	1.09E+08	0.49	-2.65	4.00	0.06
E1	GPM2	5D	AX.89660113	4.38E+07	0.47	583.37	3.05	0.05
E1	GY	5D	AX.89375041	2.26E+08	0.47	2.39	3.34	0.05
E1	GY	5D	AX.89648256	2.12E+08	0.47	2.51	3.65	0.06
E1	GY	5D	AX.89707299	7.55E+07	0.50	-2.52	3.68	0.06
E1	GY	5D	AX.89682193	4.39E+07	0.47	2.58	3.80	0.06
E1	GPS	5D	AX.89553085	2.28E+08	0.50	-1.19	3.42	0.06
E1	GY	5D	AX.89375308	4.37E+07	0.47	2.66	4.02	0.06
E1	GY	5D	AX.89525078	1.21E+08	0.44	2.36	3.24	0.06
E1	GPM2	5D	AX.89687953	5.01E+08	0.26	687.78	3.21	0.06
E1	GY	5D	AX.89443998	1.88E+08	0.49	-2.65	4.00	0.06
E1	GY	5D	AX.89443998	1.88E+08	0.49	-2.65	4.00	0.06
E1	GY	5D	AX.89470370	2.44E+08	0.47	2.26	3.03	0.05
E1	GY	5D	AX.89730596	1.08E+08	0.47	2.35	3.24	0.05
E1	GY	5D	AX.89576275	2.49E+08	0.49	-2.40	3.38	0.05
E1	GY	5D	AX.89553085	2.28E+08	0.50	-2.79	4.38	0.07
E1	GY	5D	AX.89731614	2.05E+08	0.47	2.51	3.65	0.06
E1	GPS	5D	AX.89568920	1.15E+08	0.49	-1.11	3.01	0.05
E1	GPS	5D	AX.89640713	2.74E+08	0.25	1.30	3.07	0.05
E1	GPS	5D	AX.89471124	2.21E+08	0.49	-1.13	3.11	0.05
E1	GPS	5D	AX.89689324	1.84E+08	0.49	-1.11	3.01	0.05
E1	GY	5D	AX.89526837	1.14E+08	0.49	-2.69	4.10	0.07
E1	GPM2	5D	AX.89554039	4.37E+07	0.47	583.37	3.05	0.05
E1	GY	5D	AX.89568606	1.06E+08	0.49	-2.69	4.10	0.07
E1	GY	5D	AX.89703697	2.19E+08	0.49	-2.69	4.10	0.07

E1	GY	5D	AX.89430604	2.30E+08	0.28	-3.39	5.12	0.09
E1	GPM2	5D	AX.89479940	4.38E+07	0.47	583.37	3.05	0.05
E1	GY	5D	AX.89716818	1.19E+08	0.49	-2.69	4.10	0.07
E1	GY	5D	AX.89459312	1.30E+08	0.50	-2.55	3.75	0.06
E1	GPM2	5D	AX.89325957	4.38E+07	0.47	583.37	3.05	0.05
E1	GY	5D	AX.89664265	4.38E+07	0.47	2.58	3.80	0.06
E1	GPM2	5D	AX.89480523	4.41E+07	0.47	583.37	3.05	0.05
E1	GY	5D	AX.89426929	4.38E+07	0.47	2.66	4.02	0.06
E1	GY	5D	AX.89573386	2.22E+08	0.47	-2.67	4.05	0.07
E1	GY	5D	AX.89711519	4.41E+07	0.47	2.58	3.80	0.06
E1	GY	5D	AX.89622218	9.79E+07	0.49	-2.58	3.83	0.06
E1	GY	5D	AX.89638076	1.31E+08	0.49	-2.69	4.10	0.07
E1	GY	5D	AX.89548887	1.28E+08	0.49	-2.33	3.21	0.05
E1	GY	5D	AX.89353576	9.16E+07	0.49	-2.75	4.27	0.07
E1	GY	5D	AX.89565647	2.19E+08	0.47	2.47	3.54	0.06
E1	GY	5D	AX.89772754	4.38E+07	0.47	2.58	3.80	0.06
E1	GY	5D	AX.89585184	4.38E+07	0.47	2.66	4.02	0.06
E1	GY	5D	AX.89585184	4.38E+07	0.47	2.66	4.02	0.06
E1	GY	5D	AX.89632186	2.17E+08	0.49	2.68	4.08	0.07
E1	GY	5D	AX.89762284	8.25E+07	0.47	-2.64	3.96	0.06
E1	GY	5D	AX.89429373	2.31E+08	0.47	2.39	3.34	0.05
E1	GY	5D	AX.89452264	1.71E+08	0.49	-2.69	4.10	0.07
E1	GY	5D	AX.89409385	1.69E+08	0.46	2.42	3.40	0.05
E1	GY	5D	AX.89681465	2.03E+08	0.47	2.51	3.63	0.06
E1	GPM2	5D	AX.89584091	4.53E+07	0.09	-1184.31	3.90	0.06
E1	GY	5D	AX.89437950	9.29E+07	0.48	-2.67	4.06	0.06
E1	GPM2	5D	AX.89375308	4.37E+07	0.47	583.37	3.05	0.05
E1	GPM2	5D	AX.89429887	4.38E+07	0.47	583.37	3.05	0.05
E1	GY	5D	AX.89595297	1.10E+08	0.49	-2.65	4.00	0.06
E1	GY	5D	AX.89478204	1.04E+08	0.49	-2.69	4.10	0.07
E1	GY	5D	AX.89443965	1.94E+08	0.49	-2.69	4.10	0.07
E1	GPM2	5D	AX.89313623	4.37E+07	0.47	583.37	3.05	0.05
E1	GPM2	5D	AX.89585184	4.38E+07	0.47	583.37	3.05	0.05
E1	GPS	5D	AX.89330743	1.98E+08	0.46	1.21	3.51	0.06
E1	GPM2	5D	AX.89457727	4.18E+07	0.19	742.99	3.06	0.05
E1	GPM2	5D	AX.89342736	4.39E+07	0.47	583.37	3.05	0.05
E1	GPM2	5D	AX.89697957	4.39E+07	0.47	583.37	3.05	0.05
E1	GPS	5D	AX.89448878	2.24E+08	0.50	-1.12	3.08	0.05
E1	GPM2	5D	AX.89734213	4.38E+07	0.47	583.37	3.05	0.05
E1	GPM2	5D	AX.89495145	4.41E+07	0.47	583.37	3.05	0.05
E1	GY	5D	AX.89703697	2.19E+08	0.49	-2.69	4.10	0.07
E1	GY	5D	AX.89430604	2.30E+08	0.28	-3.39	5.12	0.09
E1	GPM2	5D	AX.89357131	4.38E+07	0.47	583.37	3.05	0.05
E1	GY	5D	AX.89573386	2.22E+08	0.47	-2.67	4.05	0.07
E1	GY	5D	AX.89755860	4.41E+07	0.47	2.58	3.80	0.06
E1	GY	5D	AX.89632186	2.17E+08	0.49	2.68	4.08	0.07
E1	GY	5D	AX.89689324	1.84E+08	0.49	-2.65	4.00	0.06
E1	GY	5D	AX.89660434	4.38E+07	0.47	2.58	3.80	0.06
E1	GPM2	5D	AX.89401495	4.39E+07	0.48	581.36	3.03	0.05
E1	GPS	5D	AX.89591395	2.90E+08	0.27	1.31	3.25	0.06

E1	GPM2	5D	AX.89731768	4.37E+07	0.48	594.92	3.15	0.05
E1	GPM2	5D	AX.89698977	4.38E+07	0.47	583.37	3.05	0.05
E2	SPM2	5D	AX.89723525	2.79E+08	0.14	-17.65	3.31	0.05
E2	GY	5D	AX.89720258	4.65E+08	0.06	-5.09	4.34	0.07
E2	GY	5D	AX.89720258	4.65E+08	0.06	-5.09	4.34	0.07
E2	GY	5D	AX.89430604	2.30E+08	0.28	-2.19	3.28	0.05
E2	GY	5D	AX.89430604	2.30E+08	0.28	-2.19	3.28	0.05
E2	GPM2	5D	AX.89391799	4.38E+08	0.05	-1140.06	3.28	0.05
E2	GY	5D	AX.89651829	4.39E+08	0.08	-3.43	3.03	0.05
E2	GPS	5D	AX.89344085	4.73E+08	0.25	-1.84	4.24	0.06
E2	GY	5D	AX.89651829	4.39E+08	0.08	-3.43	3.03	0.05
E3	GPM2	5D	AX.89460631	5.03E+07	0.06	2179.97	3.99	0.08
E3	GPM2	5D	AX.89498457	5.55E+08	0.24	-987.34	3.04	0.01
E3	GPM2	5D	AX.89718196	5.56E+08	0.24	-1011.87	3.20	0.02
E3	GPM2	5D	AX.89502286	5.56E+08	0.23	-997.72	3.04	0.02
E3	GPM2	5D	AX.89688793	5.56E+08	0.24	-989.06	3.01	0.02
E3	GPS	5D	AX.89460631	5.03E+07	0.07	4.95	4.47	0.10
E3	GY	5D	AX.89493367	5.54E+08	0.22	-3.88	3.79	0.02
E3	GY	5D	AX.89482601	5.54E+08	0.22	-3.80	3.63	0.02
E3	GY	5D	AX.89322240	5.54E+08	0.23	-3.54	3.27	0.02
E3	GY	5D	AX.89525661	5.54E+08	0.22	-3.80	3.63	0.03
E3	GY	5D	AX.89682906	5.54E+08	0.22	-3.80	3.63	0.03
E3	GY	5D	AX.89730040	5.54E+08	0.23	-3.88	3.80	0.03
E3	GY	5D	AX.89418055	5.54E+08	0.23	-3.88	3.80	0.03
E3	GY	5D	AX.89591739	5.54E+08	0.23	-3.88	3.80	0.03
E3	GY	5D	AX.89746626	5.54E+08	0.22	-3.80	3.63	0.02
E3	GY	5D	AX.89696707	5.54E+08	0.22	-3.80	3.63	0.02
E3	GY	5D	AX.89748302	5.54E+08	0.22	-3.85	3.66	0.02
E3	GY	5D	AX.89725104	5.54E+08	0.24	-3.60	3.45	0.02
E3	GY	5D	AX.89535199	5.54E+08	0.24	-3.37	3.07	0.02
E3	GY	5D	AX.89730999	5.54E+08	0.24	-3.37	3.07	0.02
E3	GY	5D	AX.89617610	5.54E+08	0.24	-3.37	3.07	0.02
E3	GY	5D	AX.89487785	5.54E+08	0.24	-3.37	3.07	0.02
E3	GY	5D	AX.89474409	5.54E+08	0.24	-3.37	3.07	0.02
E3	GY	5D	AX.89646175	5.54E+08	0.24	-3.37	3.07	0.02
E3	GY	5D	AX.89647057	5.54E+08	0.24	-3.37	3.07	0.02
E3	GY	5D	AX.89450370	5.54E+08	0.24	-3.43	3.16	0.02
E3	GY	5D	AX.89410073	5.54E+08	0.24	-3.37	3.07	0.02
E3	GY	5D	AX.89593062	5.54E+08	0.24	-3.37	3.07	0.02
E3	GY	5D	AX.89527041	5.54E+08	0.24	-3.37	3.07	0.02
E3	GY	5D	AX.89676385	5.54E+08	0.24	-3.37	3.07	0.02
E3	GY	5D	AX.89635270	5.54E+08	0.24	-3.37	3.07	0.02
E3	GY	5D	AX.89681313	5.54E+08	0.24	-3.37	3.07	0.02
E3	GY	5D	AX.89461648	5.54E+08	0.24	-3.37	3.07	0.02
E3	GY	5D	AX.89634942	5.54E+08	0.24	-3.37	3.07	0.02
E3	GY	5D	AX.89691448	5.54E+08	0.24	-3.37	3.07	0.02
E3	GY	5D	AX.89482346	5.54E+08	0.24	-3.37	3.07	0.02
E3	GY	5D	AX.89565167	5.54E+08	0.24	-3.41	3.14	0.02
E3	GY	5D	AX.89588446	5.54E+08	0.25	-3.47	3.24	0.02
E3	GY	5D	AX.89621290	5.54E+08	0.24	-3.37	3.07	0.02

E3	GY	5D	AX.89399226	5.54E+08	0.25	-3.47	3.24	0.02
E3	GY	5D	AX.89559427	5.54E+08	0.24	-3.37	3.07	0.02
E3	GY	5D	AX.89405466	5.55E+08	0.24	-3.48	3.22	0.02
E3	GY	5D	AX.89408713	5.55E+08	0.24	-3.37	3.07	0.02
E3	GY	5D	AX.89722610	5.55E+08	0.24	-3.36	3.08	0.02
E3	GY	5D	AX.89411160	5.55E+08	0.24	-3.37	3.07	0.02
E3	GY	5D	AX.89691080	5.55E+08	0.24	-3.37	3.07	0.02
E3	GY	5D	AX.89509353	5.55E+08	0.24	-3.37	3.07	0.02
E3	GY	5D	AX.89348015	5.55E+08	0.24	-3.37	3.07	0.02
E3	GY	5D	AX.89319576	5.55E+08	0.24	-3.37	3.07	0.02
E3	GY	5D	AX.89373596	5.55E+08	0.25	-3.56	3.45	0.02
E3	GY	5D	AX.89756067	5.55E+08	0.25	-3.47	3.24	0.02
E3	GY	5D	AX.89475286	5.55E+08	0.25	-3.47	3.24	0.02
E3	GY	5D	AX.89508645	5.55E+08	0.24	-3.37	3.07	0.02
E3	GY	5D	AX.89633060	5.55E+08	0.24	-3.41	3.14	0.02
E3	GY	5D	AX.89742233	5.55E+08	0.24	-3.37	3.07	0.02
E3	GY	5D	AX.89742234	5.55E+08	0.25	-3.42	3.24	0.02
E3	GY	5D	AX.89472995	5.55E+08	0.24	-3.37	3.07	0.02
E3	GY	5D	AX.89559349	5.55E+08	0.24	-3.37	3.07	0.02
E3	GY	5D	AX.89654896	5.55E+08	0.24	-3.37	3.07	0.02
E3	GY	5D	AX.89632441	5.55E+08	0.25	-3.47	3.24	0.02
E3	GY	5D	AX.89378913	5.55E+08	0.25	-3.47	3.24	0.02
E3	GY	5D	AX.89374119	5.55E+08	0.25	-3.47	3.24	0.02
E3	GY	5D	AX.89575542	5.55E+08	0.25	-3.47	3.24	0.02
E3	GY	5D	AX.89558988	5.55E+08	0.24	-3.37	3.07	0.02
E3	GY	5D	AX.89604182	5.55E+08	0.25	-3.47	3.24	0.02
E3	GY	5D	AX.89421979	5.55E+08	0.25	-3.47	3.24	0.02
E3	GY	5D	AX.89588180	5.55E+08	0.25	-3.47	3.24	0.02
E3	GY	5D	AX.89655849	5.55E+08	0.25	-3.47	3.24	0.02
E3	GY	5D	AX.89679387	5.55E+08	0.25	-3.47	3.24	0.02
E3	GY	5D	AX.89628778	5.55E+08	0.25	-3.28	3.04	0.02
E3	GY	5D	AX.89362998	5.55E+08	0.25	-3.47	3.24	0.02
E3	GY	5D	AX.89663831	5.55E+08	0.25	-3.47	3.24	0.02
E3	GY	5D	AX.89633160	5.55E+08	0.25	-3.47	3.24	0.02
E3	GY	5D	AX.89662043	5.55E+08	0.25	-3.47	3.24	0.02
E3	GY	5D	AX.89596434	5.55E+08	0.25	-3.36	3.11	0.02
E3	GY	5D	AX.89356723	5.55E+08	0.24	-3.41	3.14	0.02
E3	GY	5D	AX.89361706	5.55E+08	0.25	-3.47	3.24	0.02
E3	GY	5D	AX.89635667	5.55E+08	0.24	-3.37	3.07	0.02
E3	GY	5D	AX.89524955	5.55E+08	0.24	-3.37	3.07	0.02
E3	GY	5D	AX.89628884	5.55E+08	0.24	-3.37	3.07	0.02
E3	GY	5D	AX.89486985	5.55E+08	0.24	-3.37	3.07	0.02
E3	GY	5D	AX.89602745	5.55E+08	0.24	-3.37	3.07	0.02
E3	GY	5D	AX.89709349	5.55E+08	0.24	-3.37	3.07	0.02
E3	GY	5D	AX.89498457	5.55E+08	0.24	-3.84	3.89	0.02
E3	GY	5D	AX.89765961	5.55E+08	0.23	-3.79	3.76	0.02
E3	GY	5D	AX.89423838	5.55E+08	0.25	-3.47	3.24	0.02
E3	GY	5D	AX.89585586	5.55E+08	0.25	-3.33	3.07	0.02
E3	GY	5D	AX.89733577	5.55E+08	0.24	-3.45	3.22	0.02
E3	GY	5D	AX.89621662	5.55E+08	0.25	-3.47	3.24	0.02

E3	GY	5D	AX.89717918	5.55E+08	0.25	-3.89	4.05	0.03
E3	GY	5D	AX.89695102	5.55E+08	0.25	-3.42	3.24	0.02
E3	GY	5D	AX.89336277	5.55E+08	0.25	-3.47	3.24	0.02
E3	GY	5D	AX.89512113	5.55E+08	0.24	-3.39	3.13	0.02
E3	GY	5D	AX.89444140	5.55E+08	0.25	-3.47	3.24	0.02
E3	GY	5D	AX.89329995	5.55E+08	0.25	-3.47	3.24	0.02
E3	GY	5D	AX.89682982	5.55E+08	0.25	-3.47	3.24	0.02
E3	GY	5D	AX.89616951	5.55E+08	0.25	-3.33	3.07	0.02
E3	GY	5D	AX.89487156	5.55E+08	0.25	-3.42	3.24	0.02
E3	GY	5D	AX.89633394	5.55E+08	0.25	-3.33	3.07	0.02
E3	GY	5D	AX.89465246	5.55E+08	0.24	-3.37	3.07	0.02
E3	GY	5D	AX.89686142	5.55E+08	0.24	-3.37	3.07	0.02
E3	GY	5D	AX.89324532	5.55E+08	0.25	-3.33	3.07	0.02
E3	GY	5D	AX.89602062	5.55E+08	0.25	-3.33	3.07	0.02
E3	GY	5D	AX.89495745	5.55E+08	0.25	-3.33	3.07	0.02
E3	GY	5D	AX.89331610	5.55E+08	0.25	-3.33	3.07	0.02
E3	GY	5D	AX.89527135	5.55E+08	0.25	-3.33	3.07	0.02
E3	GY	5D	AX.89695082	5.55E+08	0.24	-3.40	3.13	0.02
E3	GY	5D	AX.89724815	5.55E+08	0.24	-3.55	3.35	0.02
E3	GY	5D	AX.89465735	5.56E+08	0.24	-3.43	3.23	0.02
E3	GY	5D	AX.89575504	5.56E+08	0.24	-3.55	3.35	0.02
E3	GY	5D	AX.89696968	5.56E+08	0.24	-3.65	3.52	0.02
E3	GY	5D	AX.89708908	5.56E+08	0.24	-3.48	3.24	0.02
E3	GY	5D	AX.89447907	5.56E+08	0.25	-3.60	3.51	0.02
E3	GY	5D	AX.89597197	5.56E+08	0.24	-3.48	3.24	0.02
E3	GY	5D	AX.89515951	5.56E+08	0.24	-3.55	3.35	0.02
E3	GY	5D	AX.89389322	5.56E+08	0.24	-3.48	3.24	0.02
E3	GY	5D	AX.89410697	5.56E+08	0.24	-3.65	3.52	0.02
E3	GY	5D	AX.89489669	5.56E+08	0.24	-3.65	3.52	0.02
E3	GY	5D	AX.89699416	5.56E+08	0.24	-3.43	3.23	0.02
E3	GY	5D	AX.89543607	5.56E+08	0.24	-3.65	3.52	0.02
E3	GY	5D	AX.89424355	5.56E+08	0.24	-3.43	3.23	0.02
E3	GY	5D	AX.89423099	5.56E+08	0.24	-3.65	3.52	0.02
E3	GY	5D	AX.89710030	5.56E+08	0.19	-3.84	3.38	0.03
E3	GY	5D	AX.89544179	5.56E+08	0.24	-3.43	3.23	0.02
E3	GY	5D	AX.89505057	5.56E+08	0.24	-3.61	3.55	0.02
E3	GY	5D	AX.89718196	5.56E+08	0.24	-3.88	4.01	0.03
E3	GY	5D	AX.89327246	5.56E+08	0.17	-3.82	3.29	0.03
E3	GY	5D	AX.89393736	5.56E+08	0.17	-3.82	3.29	0.03
E3	GY	5D	AX.89610771	5.56E+08	0.17	-4.29	4.01	0.04
E3	GY	5D	AX.89652316	5.56E+08	0.17	-4.29	4.01	0.04
E3	GY	5D	AX.89379273	5.56E+08	0.17	-4.29	4.01	0.04
E3	GY	5D	AX.89632200	5.56E+08	0.18	-3.66	3.09	0.03
E3	GY	5D	AX.89684138	5.56E+08	0.19	-3.75	3.38	0.04
E3	GY	5D	AX.89502286	5.56E+08	0.23	-3.99	4.08	0.03
E3	GY	5D	AX.89344766	5.56E+08	0.24	-3.41	3.14	0.02
E3	GY	5D	AX.89696615	5.56E+08	0.23	-3.91	3.97	0.03
E3	GY	5D	AX.89688793	5.56E+08	0.24	-4.06	4.23	0.03
E3	TKW	5D	AX.89494435	3.88E+08	0.25	1.16	3.21	0.08
E3	GY	5D	AX.89460631	5.03E+07	0.06	6.58	3.34	0.07

E3	GY	5D	AX.89648993	5.42E+08	0.36	-3.00	3.01	0.05
E3	GY	5D	AX.89503888	5.51E+08	0.22	-3.37	3.10	0.05
E3	GY	5D	AX.89398084	5.51E+08	0.22	-3.49	3.25	0.05
E3	GY	5D	AX.89488459	5.51E+08	0.22	-3.49	3.25	0.05
E3	GY	5D	AX.89674679	5.51E+08	0.21	-3.48	3.18	0.05
E3	GY	5D	AX.89491527	5.51E+08	0.22	-3.43	3.22	0.05
E3	GY	5D	AX.89531362	5.53E+08	0.23	-3.88	3.80	0.03
E3	GY	5D	AX.89549579	5.53E+08	0.23	-3.88	3.80	0.03
E3	GY	5D	AX.89596564	5.53E+08	0.23	-3.88	3.80	0.03
E3	GY	5D	AX.89595142	5.53E+08	0.23	-3.88	3.80	0.03
E3	GY	5D	AX.89433989	5.53E+08	0.21	-3.73	3.42	0.02
E3	GY	5D	AX.89534586	5.53E+08	0.21	-3.84	3.67	0.02
E3	GY	5D	AX.89527557	5.53E+08	0.21	-3.84	3.67	0.02
E3	GY	5D	AX.89754254	5.53E+08	0.22	-3.92	3.82	0.03
E3	GY	5D	AX.89370928	5.53E+08	0.22	-3.45	3.03	0.02
E3	GY	5D	AX.89310331	5.53E+08	0.22	-3.71	3.48	0.02
E3	GY	5D	AX.89716843	5.53E+08	0.22	-3.80	3.63	0.03
E3	GY	5D	AX.89774314	5.53E+08	0.22	-3.80	3.63	0.03
E3	GY	5D	AX.89506758	5.53E+08	0.22	-3.80	3.63	0.03
E3	GY	5D	AX.89451982	5.53E+08	0.22	-3.80	3.63	0.03
E3	GY	5D	AX.89721418	5.54E+08	0.18	-3.72	3.13	0.02
E3	GY	5D	AX.89663996	5.54E+08	0.21	-3.71	3.44	0.03
E3	GY	5D	AX.89755947	5.54E+08	0.22	-3.80	3.63	0.03
E3	GY	5D	AX.89658343	5.54E+08	0.22	-3.80	3.63	0.03
E3	GY	5D	AX.89341726	5.54E+08	0.22	-3.74	3.52	0.03
E3	GY	5D	AX.89315561	5.54E+08	0.22	-3.80	3.63	0.03
E3	GY	5D	AX.89642259	5.54E+08	0.22	-3.74	3.52	0.03
E3	GY	5D	AX.89317384	5.54E+08	0.22	-3.74	3.52	0.03
E3	GY	5D	AX.89644074	5.54E+08	0.21	-3.77	3.56	0.03
E3	GY	5D	AX.89379598	5.54E+08	0.23	-3.88	3.80	0.03
E3	GY	5D	AX.89496107	5.54E+08	0.23	-3.88	3.80	0.03
E3	GY	5D	AX.89568359	5.54E+08	0.21	-3.78	3.57	0.02
E3	GY	5D	AX.89632265	5.54E+08	0.22	-3.79	3.64	0.03
E3	GY	5D	AX.89311973	5.54E+08	0.23	-3.53	3.29	0.02
E3	GY	5D	AX.89658664	5.54E+08	0.21	-3.78	3.57	0.02
E3	GY	5D	AX.89677074	5.54E+08	0.23	-3.88	3.80	0.03
E3	GY	5D	AX.89345377	5.54E+08	0.23	-3.88	3.80	0.03
E3	GY	5D	AX.89453386	5.54E+08	0.23	-3.88	3.80	0.03
E3	GY	5D	AX.89577888	5.54E+08	0.21	-3.78	3.57	0.02
E3	GY	5D	AX.89767142	5.54E+08	0.23	-3.88	3.80	0.03
E3	GY	5D	AX.89667245	5.54E+08	0.22	-3.71	3.48	0.02
E3	GY	5D	AX.89555202	5.54E+08	0.22	-3.71	3.48	0.02
E3	GY	5D	AX.89369615	5.54E+08	0.23	-3.88	3.80	0.03
E3	GY	5D	AX.89759518	5.54E+08	0.23	-3.88	3.80	0.03
E3	GY	5D	AX.89588258	5.54E+08	0.22	-3.86	3.74	0.03
E3	GY	5D	AX.89754049	5.54E+08	0.23	-3.88	3.80	0.03
E3	GY	5D	AX.89619784	5.54E+08	0.22	-3.80	3.63	0.02
E3	GY	5D	AX.89639008	5.54E+08	0.21	-3.63	3.26	0.02
E3	GY	5D	AX.89441040	5.54E+08	0.22	-3.80	3.63	0.03
E3	GY	5D	AX.89578188	5.54E+08	0.22	-3.84	3.69	0.03

E3	GY	5D	AX.89665780	5.54E+08	0.22	-3.80	3.63	0.03
E3	GY	5D	AX.89571916	5.54E+08	0.22	-3.62	3.31	0.03
E3	GY	5D	AX.89509820	5.54E+08	0.23	-3.88	3.80	0.03
E3	GY	5D	AX.89677610	5.54E+08	0.22	-3.80	3.63	0.03
E3	GY	5D	AX.89746134	5.54E+08	0.22	-3.80	3.63	0.03
E3	GY	5D	AX.89454409	5.54E+08	0.21	-3.84	3.73	0.03
E3	GY	5D	AX.89720445	5.54E+08	0.22	-3.80	3.63	0.03
E3	GY	5D	AX.89772832	5.54E+08	0.22	-3.80	3.63	0.03
E3	GY	5D	AX.89341879	5.54E+08	0.22	-3.80	3.63	0.03
E3	GY	5D	AX.89552617	5.54E+08	0.22	-3.80	3.63	0.03
E3	GY	5D	AX.89720944	5.54E+08	0.22	-3.52	3.18	0.02
E3	GY	5D	AX.89491813	5.54E+08	0.23	-3.88	3.80	0.03
E3	GY	5D	AX.89686143	5.54E+08	0.22	-3.80	3.63	0.03
E3	GY	5D	AX.89441663	5.54E+08	0.22	-3.80	3.63	0.03
E3	GY	5D	AX.89336024	5.54E+08	0.23	-3.88	3.80	0.03
E3	GY	5D	AX.89342095	5.54E+08	0.23	-3.75	3.62	0.02
E3	GY	5D	AX.89370597	5.54E+08	0.23	-3.66	3.46	0.02
E3	GY	5D	AX.89627494	5.54E+08	0.22	-3.80	3.63	0.03
E3	GY	5D	AX.89370230	5.54E+08	0.23	-3.66	3.46	0.02
E3	GY	5D	AX.89493083	5.54E+08	0.23	-3.75	3.62	0.02
E3	GY	5D	AX.89336525	5.54E+08	0.23	-3.66	3.46	0.02
E3	GY	5D	AX.89386339	5.54E+08	0.23	-3.66	3.46	0.02
E3	GY	5D	AX.89736893	5.54E+08	0.23	-3.66	3.46	0.02
E3	GY	5D	AX.89719901	5.54E+08	0.23	-3.66	3.46	0.02
E3	GY	5D	AX.89448742	5.54E+08	0.23	-3.66	3.46	0.02
E3	GY	5D	AX.89341837	5.54E+08	0.23	-3.33	3.00	0.02
E3	GY	5D	AX.89604858	5.54E+08	0.23	-3.33	3.00	0.02
E3	GY	5D	AX.89637165	5.54E+08	0.23	-3.33	3.00	0.02
E3	GY	5D	AX.89441387	5.54E+08	0.23	-3.33	3.00	0.02
E3	GY	5D	AX.89670745	5.54E+08	0.23	-3.33	3.00	0.02
E3	GY	5D	AX.89411065	5.54E+08	0.24	-3.60	3.45	0.02
E3	GY	5D	AX.89498805	5.54E+08	0.24	-3.60	3.45	0.02
E3	GY	5D	AX.89370004	5.55E+08	0.25	-3.42	3.24	0.02
E3	GY	5D	AX.89512336	5.55E+08	0.25	-3.42	3.24	0.02
E3	GY	5D	AX.89669450	5.55E+08	0.24	-3.37	3.07	0.02
E3	GY	5D	AX.89383924	5.55E+08	0.24	-3.52	3.35	0.02
E3	GY	5D	AX.89768392	5.55E+08	0.24	-3.39	3.10	0.02
E3	GY	5D	AX.89383120	5.55E+08	0.25	-3.47	3.24	0.02
E3	GY	5D	AX.89617428	5.55E+08	0.25	-3.47	3.24	0.02
E3	GY	5D	AX.89699498	5.55E+08	0.24	-3.37	3.07	0.02
E3	GY	5D	AX.89654440	5.55E+08	0.24	-3.37	3.07	0.02
E3	GY	5D	AX.89563389	5.55E+08	0.24	-3.37	3.07	0.02
E4	GPM2	5D	AX.89595049	4.74E+08	0.08	-890.98	3.38	0.05
E4	SPM2	5D	AX.89388589	3.68E+08	0.30	19.64	3.43	0.03
E4	SPM2	5D	AX.89512625	4.83E+08	0.50	16.13	3.07	0.07
E1	GY	6A	AX.89448704	1.21E+07	0.13	-4.16	4.42	0.08
E1	SPM2	6A	AX.89551676	3.78E+08	0.07	30.94	3.03	0.05
E1	GY	6A	AX.89327931	5.95E+08	0.13	3.75	3.79	0.07
E1	GY	6A	AX.89453452	5.95E+08	0.13	3.75	3.79	0.07
E1	GY	6A	AX.89399281	5.95E+08	0.13	3.75	3.79	0.07

E1	GY	6A	AX.89428100	5.95E+08	0.13	3.75	3.79	0.07
E1	GY	6A	AX.89327931	5.95E+08	0.13	3.75	3.79	0.07
E1	GY	6A	AX.89389958	5.60E+06	0.40	-2.91	4.54	0.08
E1	GY	6A	AX.89425314	7.15E+06	0.31	-2.91	4.14	0.06
E1	GY	6A	AX.89442899	6.60E+06	0.37	-2.79	4.13	0.07
E1	GY	6A	AX.89448704	1.21E+07	0.13	-4.16	4.42	0.08
E1	GY	6A	AX.89428100	5.95E+08	0.13	3.75	3.79	0.07
E1	SPM2	6A	AX.89712773	1.39E+07	0.10	-28.56	3.27	0.06
E1	GY	6A	AX.89442899	6.60E+06	0.37	-2.79	4.13	0.07
E1	GY	6A	AX.89437935	1.05E+07	0.11	-3.97	3.62	0.07
E1	GY	6A	AX.89448872	6.61E+06	0.37	-2.79	4.13	0.07
E1	GY	6A	AX.89661668	5.36E+06	0.38	-2.71	3.98	0.07
E1	GY	6A	AX.89399281	5.95E+08	0.13	3.75	3.79	0.07
E1	GY	6A	AX.89489584	1.05E+07	0.11	-3.97	3.62	0.07
E1	GY	6A	AX.89340229	5.95E+08	0.13	3.75	3.79	0.07
E1	GY	6A	AX.89437935	1.05E+07	0.11	-3.97	3.62	0.07
E1	GY	6A	AX.89489584	1.05E+07	0.11	-3.97	3.62	0.07
E1	GY	6A	AX.89453452	5.95E+08	0.13	3.75	3.79	0.07
E1	GY	6A	AX.89389958	5.60E+06	0.40	-2.91	4.54	0.08
E1	GY	6A	AX.89525311	1.06E+07	0.10	-4.22	3.77	0.07
E1	GY	6A	AX.89525311	1.06E+07	0.10	-4.22	3.77	0.07
E1	GY	6A	AX.89310997	5.95E+08	0.13	3.75	3.79	0.07
E1	GY	6A	AX.89324890	5.95E+08	0.13	3.75	3.79	0.07
E1	GY	6A	AX.89615289	1.23E+07	0.13	-4.16	4.42	0.08
E1	GY	6A	AX.89685917	1.05E+07	0.12	-3.72	3.34	0.06
E1	GY	6A	AX.89605663	5.95E+08	0.13	3.75	3.79	0.07
E1	GY	6A	AX.89727310	1.05E+07	0.12	-3.72	3.34	0.06
E1	GY	6A	AX.89743985	1.06E+07	0.12	-3.72	3.34	0.06
E1	GY	6A	AX.89539329	1.05E+07	0.11	-3.97	3.62	0.07
E1	GY	6A	AX.89368427	5.95E+08	0.13	3.75	3.79	0.07
E1	GY	6A	AX.89310997	5.95E+08	0.13	3.75	3.79	0.07
E1	GY	6A	AX.89360412	1.05E+07	0.12	-3.72	3.34	0.06
E1	GY	6A	AX.89393684	4.58E+06	0.37	-2.66	3.80	0.06
E1	GY	6A	AX.89341697	1.13E+07	0.06	-4.76	3.08	0.05
E1	GY	6A	AX.89388224	5.94E+08	0.44	-2.26	3.02	0.05
E1	GY	6A	AX.89362270	5.95E+08	0.13	3.75	3.79	0.07
E1	GY	6A	AX.89661668	5.36E+06	0.38	-2.71	3.98	0.07
E1	GY	6A	AX.89378676	1.05E+07	0.12	-3.72	3.34	0.06
E1	GY	6A	AX.89602780	5.33E+06	0.40	-2.85	4.38	0.07
E1	GY	6A	AX.89602780	5.33E+06	0.40	-2.85	4.38	0.07
E1	GY	6A	AX.89448872	6.61E+06	0.37	-2.79	4.13	0.07
E1	GY	6A	AX.89391096	6.73E+06	0.37	-2.79	4.13	0.07
E1	GY	6A	AX.89391096	6.73E+06	0.37	-2.79	4.13	0.07
E1	GY	6A	AX.89482931	1.16E+07	0.12	-3.81	3.59	0.07
E1	GY	6A	AX.89329955	6.32E+06	0.37	-2.79	4.13	0.07
E1	GY	6A	AX.89716829	5.32E+06	0.38	-2.71	3.98	0.07
E1	GY	6A	AX.89615589	5.95E+08	0.13	3.75	3.79	0.07
E1	GY	6A	AX.89404830	4.39E+06	0.37	-2.66	3.80	0.06
E1	GY	6A	AX.89533920	1.23E+07	0.15	-3.40	3.43	0.06
E1	GY	6A	AX.89533920	1.23E+07	0.15	-3.40	3.43	0.06

E1	GY	6A	AX.89640320	6.81E+06	0.38	-2.43	3.28	0.05
E1	GY	6A	AX.89324890	5.95E+08	0.13	3.75	3.79	0.07
E1	GY	6A	AX.89573183	6.60E+06	0.37	-2.79	4.13	0.07
E1	GY	6A	AX.89371581	6.61E+06	0.37	-2.79	4.13	0.07
E1	GY	6A	AX.89371581	6.61E+06	0.37	-2.79	4.13	0.07
E1	GY	6A	AX.89482931	1.16E+07	0.12	-3.81	3.59	0.07
E1	GY	6A	AX.89717492	7.39E+06	0.23	-2.99	3.69	0.06
E1	GY	6A	AX.89353043	5.96E+08	0.16	3.20	3.22	0.06
E1	GY	6A	AX.89361303	1.16E+07	0.12	-3.91	3.64	0.07
E1	GY	6A	AX.89714400	1.10E+07	0.12	-3.72	3.34	0.06
E1	GY	6A	AX.89480424	5.95E+08	0.13	3.75	3.79	0.07
E1	GY	6A	AX.89480424	5.95E+08	0.13	3.75	3.79	0.07
E1	GY	6A	AX.89378676	1.05E+07	0.12	-3.72	3.34	0.06
E1	GY	6A	AX.89682658	5.95E+08	0.13	3.75	3.79	0.07
E1	GY	6A	AX.89682658	5.95E+08	0.13	3.75	3.79	0.07
E1	GY	6A	AX.89539329	1.05E+07	0.11	-3.97	3.62	0.07
E1	GY	6A	AX.89340229	5.95E+08	0.13	3.75	3.79	0.07
E1	GY	6A	AX.89412160	5.36E+06	0.38	-2.71	3.98	0.07
E1	GY	6A	AX.89423567	5.95E+08	0.13	3.75	3.79	0.07
E1	GY	6A	AX.86165943	6.39E+06	0.37	-2.74	4.01	0.07
E1	GY	6A	AX.89341697	1.13E+07	0.06	-4.76	3.08	0.05
E1	GY	6A	AX.89676835	6.03E+08	0.43	-2.44	3.41	0.06
E1	GY	6A	AX.89342638	6.03E+08	0.42	-2.38	3.26	0.05
E1	GY	6A	AX.89342638	6.03E+08	0.42	-2.38	3.26	0.05
E1	GY	6A	AX.89342656	6.61E+06	0.37	-2.79	4.13	0.07
E1	GY	6A	AX.89342656	6.61E+06	0.37	-2.79	4.13	0.07
E1	GY	6A	AX.89448954	5.36E+06	0.38	-2.71	3.98	0.07
E1	GY	6A	AX.89448954	5.36E+06	0.38	-2.71	3.98	0.07
E1	GY	6A	AX.89378771	5.95E+08	0.13	3.75	3.79	0.07
E1	GY	6A	AX.89625041	1.23E+07	0.14	-3.23	3.07	0.05
E1	GY	6A	AX.89554396	1.13E+07	0.06	-4.76	3.08	0.05
E1	GY	6A	AX.89379391	5.95E+08	0.13	3.75	3.79	0.07
E1	GY	6A	AX.89591547	5.95E+08	0.13	3.65	3.51	0.06
E1	GY	6A	AX.89591547	5.95E+08	0.13	3.65	3.51	0.06
E1	GY	6A	AX.89416311	5.95E+08	0.13	3.75	3.79	0.07
E1	GY	6A	AX.89714400	1.10E+07	0.12	-3.72	3.34	0.06
E1	GY	6A	AX.89640320	6.81E+06	0.38	-2.43	3.28	0.05
E1	GY	6A	AX.89648942	1.14E+07	0.10	-3.75	3.08	0.06
E1	GY	6A	AX.89738289	5.95E+08	0.13	3.75	3.79	0.07
E1	GY	6A	AX.89418722	1.24E+07	0.13	-3.48	3.24	0.06
E1	GY	6A	AX.89418722	1.24E+07	0.13	-3.48	3.24	0.06
E1	GY	6A	AX.89716829	5.32E+06	0.38	-2.71	3.98	0.07
E1	GY	6A	AX.89615289	1.23E+07	0.13	-4.16	4.42	0.08
E1	GY	6A	AX.89362270	5.95E+08	0.13	3.75	3.79	0.07
E1	GY	6A	AX.89716589	5.13E+06	0.40	2.30	3.03	0.04
E1	GY	6A	AX.89499648	1.16E+07	0.12	-3.91	3.64	0.07
E1	GY	6A	AX.89499648	1.16E+07	0.12	-3.91	3.64	0.07
E1	GY	6A	AX.89573181	1.05E+07	0.11	-3.97	3.62	0.07
E1	GY	6A	AX.89743985	1.06E+07	0.12	-3.72	3.34	0.06
E1	GY	6A	AX.89720313	1.05E+07	0.11	-3.76	3.30	0.06

E1	GY	6A	AX.89720313	1.05E+07	0.11	-3.76	3.30	0.06
E1	GY	6A	AX.89363729	1.21E+07	0.11	-3.97	3.63	0.07
E1	GY	6A	AX.89607340	5.95E+08	0.13	3.75	3.79	0.07
E1	GY	6A	AX.89353043	5.96E+08	0.16	3.20	3.22	0.06
E1	GY	6A	AX.89518811	5.95E+08	0.13	3.75	3.79	0.07
E1	GY	6A	AX.89681003	6.03E+08	0.43	-2.41	3.35	0.06
E1	GY	6A	AX.89685917	1.05E+07	0.12	-3.72	3.34	0.06
E1	GY	6A	AX.89549498	6.03E+08	0.43	-2.41	3.35	0.06
E1	GY	6A	AX.89589426	5.95E+08	0.13	3.75	3.79	0.07
E1	GY	6A	AX.89589426	5.95E+08	0.13	3.75	3.79	0.07
E1	GY	6A	AX.89425314	7.15E+06	0.31	-2.91	4.14	0.06
E1	GY	6A	AX.89625041	1.23E+07	0.14	-3.23	3.07	0.05
E1	GY	6A	AX.89379391	5.95E+08	0.13	3.75	3.79	0.07
E1	GY	6A	AX.89662817	5.95E+08	0.13	3.65	3.51	0.06
E1	GY	6A	AX.89603372	7.15E+06	0.30	-2.66	3.48	0.05
E1	GY	6A	AX.89451585	1.24E+07	0.13	-3.48	3.24	0.06
E1	GY	6A	AX.89498345	5.36E+06	0.39	-2.74	4.07	0.07
E1	GY	6A	AX.89498345	5.36E+06	0.39	-2.74	4.07	0.07
E1	GY	6A	AX.89605663	5.95E+08	0.13	3.75	3.79	0.07
E1	GY	6A	AX.89727310	1.05E+07	0.12	-3.72	3.34	0.06
E1	GY	6A	AX.89463872	6.37E+06	0.41	-2.66	3.92	0.06
E1	GY	6A	AX.89717192	5.95E+08	0.13	3.75	3.79	0.07
E1	GY	6A	AX.89360412	1.05E+07	0.12	-3.72	3.34	0.06
E1	GY	6A	AX.89466068	5.95E+08	0.13	3.75	3.79	0.07
E1	GY	6A	AX.89537846	5.95E+08	0.13	3.75	3.79	0.07
E1	GY	6A	AX.89537846	5.95E+08	0.13	3.75	3.79	0.07
E1	GY	6A	AX.89393684	4.58E+06	0.37	-2.66	3.80	0.06
E1	GY	6A	AX.89503668	1.05E+07	0.12	-3.72	3.34	0.06
E1	GY	6A	AX.89503668	1.05E+07	0.12	-3.72	3.34	0.06
E1	GY	6A	AX.89573181	1.05E+07	0.11	-3.97	3.62	0.07
E1	GY	6A	AX.89363729	1.21E+07	0.11	-3.97	3.63	0.07
E1	GY	6A	AX.89400057	1.09E+07	0.11	-3.97	3.62	0.07
E1	GY	6A	AX.89400057	1.09E+07	0.11	-3.97	3.62	0.07
E1	GY	6A	AX.89329955	6.32E+06	0.37	-2.79	4.13	0.07
E1	GY	6A	AX.86165943	6.39E+06	0.37	-2.74	4.01	0.07
E1	GY	6A	AX.89353239	5.95E+08	0.13	3.75	3.79	0.07
E1	GY	6A	AX.89353239	5.95E+08	0.13	3.75	3.79	0.07
E1	GY	6A	AX.89576276	5.43E+06	0.38	-2.71	3.98	0.07
E1	GY	6A	AX.89403262	1.21E+07	0.11	-4.37	4.15	0.08
E1	GY	6A	AX.89403262	1.21E+07	0.11	-4.37	4.15	0.08
E1	GY	6A	AX.89664596	5.36E+06	0.38	-2.71	3.98	0.07
E1	GY	6A	AX.89664596	5.36E+06	0.38	-2.71	3.98	0.07
E1	GY	6A	AX.89404830	4.39E+06	0.37	-2.66	3.80	0.06
E1	GY	6A	AX.89369831	5.55E+06	0.38	-2.58	3.64	0.06
E1	GY	6A	AX.89369831	5.55E+06	0.38	-2.58	3.64	0.06
E1	GY	6A	AX.89608826	1.05E+07	0.11	-4.04	3.62	0.07
E1	GY	6A	AX.89345775	6.03E+08	0.44	-2.39	3.31	0.05
E1	GY	6A	AX.89707653	1.21E+07	0.13	-3.62	3.38	0.06
E1	GY	6A	AX.89451821	5.95E+08	0.13	3.75	3.79	0.07
E1	GY	6A	AX.89684726	5.36E+06	0.38	-2.70	3.92	0.07

E1	GY	6A	AX.89684726	5.36E+06	0.38	-2.70	3.92	0.07
E1	GY	6A	AX.89608083	5.95E+08	0.13	3.75	3.79	0.07
E1	GY	6A	AX.89717492	7.39E+06	0.23	-2.99	3.69	0.06
E1	GY	6A	AX.89619173	1.16E+07	0.12	-3.91	3.64	0.07
E1	GY	6A	AX.89619173	1.16E+07	0.12	-3.91	3.64	0.07
E1	GY	6A	AX.89361303	1.16E+07	0.12	-3.91	3.64	0.07
E1	GY	6A	AX.89472795	6.03E+08	0.43	-2.41	3.35	0.06
E1	GY	6A	AX.89412160	5.36E+06	0.38	-2.71	3.98	0.07
E1	GY	6A	AX.89685133	5.95E+08	0.13	3.75	3.79	0.07
E1	GY	6A	AX.89541713	6.72E+06	0.37	-2.79	4.13	0.07
E1	GY	6A	AX.89541713	6.72E+06	0.37	-2.79	4.13	0.07
E1	GY	6A	AX.89549498	6.03E+08	0.43	-2.41	3.35	0.06
E1	GY	6A	AX.89607551	1.13E+07	0.06	-4.76	3.08	0.05
E1	GY	6A	AX.89521495	1.16E+07	0.12	-3.91	3.64	0.07
E1	GY	6A	AX.89412851	1.13E+07	0.06	-4.76	3.08	0.05
E1	GY	6A	AX.89615589	5.95E+08	0.13	3.75	3.79	0.07
E1	GY	6A	AX.89416311	5.95E+08	0.13	3.75	3.79	0.07
E1	GY	6A	AX.89345775	6.03E+08	0.44	-2.39	3.31	0.05
E1	GY	6A	AX.89707653	1.21E+07	0.13	-3.62	3.38	0.06
E1	GY	6A	AX.89445666	5.12E+06	0.40	-2.70	4.00	0.07
E1	GY	6A	AX.89723063	5.33E+06	0.38	-2.71	3.98	0.07
E1	GY	6A	AX.89646713	6.03E+08	0.44	-2.34	3.19	0.05
E1	GY	6A	AX.89740833	6.84E+06	0.41	-2.96	4.73	0.08
E1	GY	6A	AX.89608083	5.95E+08	0.13	3.75	3.79	0.07
E1	GY	6A	AX.89668076	1.16E+07	0.12	-3.91	3.64	0.07
E1	GY	6A	AX.89633871	1.21E+07	0.14	-3.51	3.45	0.06
E1	GY	6A	AX.89633871	1.21E+07	0.14	-3.51	3.45	0.06
E1	GY	6A	AX.89687785	1.23E+07	0.13	-4.16	4.42	0.08
E1	GY	6A	AX.89628477	1.05E+07	0.12	-3.72	3.34	0.06
E1	GY	6A	AX.89716589	5.13E+06	0.40	2.30	3.03	0.04
E1	GY	6A	AX.89590228	1.05E+07	0.11	-3.97	3.62	0.07
E1	GY	6A	AX.89590228	1.05E+07	0.11	-3.97	3.62	0.07
E1	GY	6A	AX.89607340	5.95E+08	0.13	3.75	3.79	0.07
E1	GY	6A	AX.89710974	5.95E+08	0.13	3.65	3.51	0.06
E1	GY	6A	AX.89710974	5.95E+08	0.13	3.65	3.51	0.06
E1	GY	6A	AX.89353279	6.57E+06	0.37	-2.79	4.13	0.07
E1	GY	6A	AX.89353279	6.57E+06	0.37	-2.79	4.13	0.07
E1	GY	6A	AX.89388224	5.94E+08	0.44	-2.26	3.02	0.05
E1	GY	6A	AX.89663215	6.60E+06	0.37	-2.79	4.13	0.07
E1	GY	6A	AX.89685133	5.95E+08	0.13	3.75	3.79	0.07
E1	GY	6A	AX.89624935	1.21E+07	0.13	-4.16	4.42	0.08
E1	GY	6A	AX.89624935	1.21E+07	0.13	-4.16	4.42	0.08
E1	GY	6A	AX.89603372	7.15E+06	0.30	-2.66	3.48	0.05
E1	GY	6A	AX.89521495	1.16E+07	0.12	-3.91	3.64	0.07
E1	GY	6A	AX.89451585	1.24E+07	0.13	-3.48	3.24	0.06
E1	GY	6A	AX.89573183	6.60E+06	0.37	-2.79	4.13	0.07
E1	GY	6A	AX.89463872	6.37E+06	0.41	-2.66	3.92	0.06
E1	GY	6A	AX.89717192	5.95E+08	0.13	3.75	3.79	0.07
E1	GY	6A	AX.89466068	5.95E+08	0.13	3.75	3.79	0.07
E1	GY	6A	AX.89417045	5.95E+08	0.13	3.65	3.51	0.06

E1	GPM2	6A	AX.89573181	1.05E+07	0.11	-1046.42	3.81	0.07
E1	GPM2	6A	AX.89724488	6.73E+06	0.22	-738.22	3.38	0.06
E1	GY	6A	AX.89576276	5.43E+06	0.38	-2.71	3.98	0.07
E1	GY	6A	AX.89554396	1.13E+07	0.06	-4.76	3.08	0.05
E1	GPM2	6A	AX.86164577	5.85E+08	0.06	1249.09	3.01	0.03
E1	GPM2	6A	AX.89720313	1.05E+07	0.11	-958.61	3.28	0.06
E1	GY	6A	AX.89368427	5.95E+08	0.13	3.75	3.79	0.07
E1	GPM2	6A	AX.89472795	6.03E+08	0.43	-587.37	3.08	0.05
E1	GY	6A	AX.89635128	6.03E+08	0.45	-2.25	3.00	0.05
E1	GPM2	6A	AX.89549498	6.03E+08	0.43	-587.37	3.08	0.05
E1	GPM2	6A	AX.89503668	1.05E+07	0.12	-980.58	3.52	0.06
E1	GY	6A	AX.89748354	1.05E+07	0.12	-3.72	3.34	0.06
E1	GY	6A	AX.89748354	1.05E+07	0.12	-3.72	3.34	0.06
E1	GPM2	6A	AX.89484543	1.16E+07	0.11	-989.97	3.47	0.06
E1	GY	6A	AX.89676835	6.03E+08	0.43	-2.44	3.41	0.06
E1	GPM2	6A	AX.89533920	1.23E+07	0.15	-867.76	3.41	0.06
E1	GPM2	6A	AX.89658761	6.73E+06	0.22	-738.22	3.38	0.06
E1	GY	6A	AX.89648942	1.14E+07	0.10	-3.75	3.08	0.06
E1	GPM2	6A	AX.89676835	6.03E+08	0.43	-585.11	3.05	0.05
E1	GY	6A	AX.89690923	1.14E+07	0.12	-3.91	3.64	0.07
E1	GY	6A	AX.89690923	1.14E+07	0.12	-3.91	3.64	0.07
E1	GPM2	6A	AX.89608826	1.05E+07	0.11	-1057.99	3.76	0.07
E1	GPM2	6A	AX.89514811	4.68E+08	0.05	-1328.91	3.11	0.05
E1	GPM2	6A	AX.89375285	5.85E+08	0.06	1249.09	3.01	0.03
E1	GPM2	6A	AX.89707653	1.21E+07	0.13	-963.16	3.61	0.06
E1	GPM2	6A	AX.89361303	1.16E+07	0.12	-1067.75	4.07	0.07
E1	GY	6A	AX.89472795	6.03E+08	0.43	-2.41	3.35	0.06
E1	GPM2	6A	AX.89619173	1.16E+07	0.12	-1067.75	4.07	0.07
E1	GPM2	6A	AX.89463872	6.37E+06	0.41	-593.97	3.10	0.05
E1	GPM2	6A	AX.89727310	1.05E+07	0.12	-980.58	3.52	0.06
E1	GY	6A	AX.89518811	5.95E+08	0.13	3.75	3.79	0.07
E1	GPM2	6A	AX.89521495	1.16E+07	0.12	-1067.75	4.07	0.07
E1	GPM2	6A	AX.89568607	6.03E+08	0.28	705.84	3.56	0.06
E1	GPM2	6A	AX.86165943	6.39E+06	0.37	-621.36	3.25	0.05
E1	GPM2	6A	AX.89353279	6.57E+06	0.37	-626.11	3.28	0.05
E1	GY	6A	AX.89607551	1.13E+07	0.06	-4.76	3.08	0.05
E1	GY	6A	AX.89378771	5.95E+08	0.13	3.75	3.79	0.07
E1	GPM2	6A	AX.89541713	6.72E+06	0.37	-626.11	3.28	0.05
E1	GPM2	6A	AX.89740833	6.84E+06	0.41	-651.81	3.63	0.06
E1	GY	6A	AX.89662817	5.95E+08	0.13	3.65	3.51	0.06
E1	GPM2	6A	AX.89371581	6.61E+06	0.37	-626.11	3.28	0.05
E1	GPM2	6A	AX.89442899	6.60E+06	0.37	-626.11	3.28	0.05
E1	GY	6A	AX.89412851	1.13E+07	0.06	-4.76	3.08	0.05
E1	GPM2	6A	AX.89321054	1.16E+07	0.11	-989.97	3.47	0.06
E1	GY	6A	AX.89564541	5.95E+08	0.13	3.75	3.79	0.07
E1	GPM2	6A	AX.89476085	6.03E+08	0.27	704.27	3.45	0.06
E1	GY	6A	AX.89451821	5.95E+08	0.13	3.75	3.79	0.07
E1	GY	6A	AX.89445666	5.12E+06	0.40	-2.70	4.00	0.07
E1	GY	6A	AX.89738289	5.95E+08	0.13	3.75	3.79	0.07
E1	GPM2	6A	AX.89563727	5.85E+08	0.06	1249.09	3.01	0.03

E1	GY	6A	AX.89723063	5.33E+06	0.38	-2.71	3.98	0.07
E1	GPM2	6A	AX.89403262	1.21E+07	0.11	-1065.97	3.81	0.07
E1	GY	6A	AX.89646713	6.03E+08	0.44	-2.34	3.19	0.05
E1	GPM2	6A	AX.89663215	6.60E+06	0.37	-626.11	3.28	0.05
E1	GY	6A	AX.89740833	6.84E+06	0.41	-2.96	4.73	0.08
E1	GY	6A	AX.89668076	1.16E+07	0.12	-3.91	3.64	0.07
E1	GPM2	6A	AX.89491145	6.73E+06	0.22	-738.22	3.38	0.06
E1	GY	6A	AX.89687785	1.23E+07	0.13	-4.16	4.42	0.08
E1	GPM2	6A	AX.89624935	1.21E+07	0.13	-1078.27	4.51	0.08
E1	GPM2	6A	AX.89690923	1.14E+07	0.12	-1067.75	4.07	0.07
E1	GY	6A	AX.89417045	5.95E+08	0.13	3.65	3.51	0.06
E1	GY	6A	AX.89534738	5.95E+08	0.13	3.75	3.79	0.07
E1	GPM2	6A	AX.89448704	1.21E+07	0.13	-1078.27	4.51	0.08
E1	GPM2	6A	AX.89329900	6.73E+06	0.22	-738.22	3.38	0.06
E1	GPM2	6A	AX.89539329	1.05E+07	0.11	-1046.42	3.81	0.07
E1	GY	6A	AX.89564135	5.95E+08	0.13	3.75	3.79	0.07
E1	GY	6A	AX.89663215	6.60E+06	0.37	-2.79	4.13	0.07
E1	GY	6A	AX.89681003	6.03E+08	0.43	-2.41	3.35	0.06
E1	GY	6A	AX.89665621	5.87E+06	0.38	-2.70	3.92	0.07
E1	GY	6A	AX.89515569	5.56E+06	0.38	-2.70	3.92	0.07
E1	GPM2	6A	AX.89648942	1.14E+07	0.10	-1015.67	3.39	0.06
E1	GPM2	6A	AX.89482931	1.16E+07	0.12	-1027.42	3.92	0.07
E1	GY	6A	AX.89394835	5.13E+06	0.38	-2.71	3.98	0.07
E1	GY	6A	AX.89394835	5.13E+06	0.38	-2.71	3.98	0.07
E1	GY	6A	AX.89607041	1.05E+07	0.07	-4.35	3.11	0.06
E1	GY	6A	AX.89607041	1.05E+07	0.07	-4.35	3.11	0.06
E1	GPM2	6A	AX.89332896	5.85E+08	0.06	1249.09	3.01	0.03
E1	GPM2	6A	AX.89743985	1.06E+07	0.12	-980.58	3.52	0.06
E1	GPM2	6A	AX.89687785	1.23E+07	0.13	-1078.27	4.51	0.08
E1	GPM2	6A	AX.89677272	4.51E+08	0.10	1023.59	3.32	0.06
E1	GPM2	6A	AX.89714400	1.10E+07	0.12	-980.58	3.52	0.06
E1	GPM2	6A	AX.89681003	6.03E+08	0.43	-587.37	3.08	0.05
E1	GPM2	6A	AX.89525311	1.06E+07	0.10	-1076.62	3.75	0.07
E1	GPM2	6A	AX.89541930	6.03E+08	0.31	660.99	3.36	0.06
E1	GY	6A	AX.89635128	6.03E+08	0.45	-2.25	3.00	0.05
E1	GPM2	6A	AX.89378676	1.05E+07	0.12	-980.58	3.52	0.06
E1	GPM2	6A	AX.89363729	1.21E+07	0.11	-1010.21	3.59	0.06
E1	GPM2	6A	AX.89499648	1.16E+07	0.12	-1067.75	4.07	0.07
E1	GY	6A	AX.89608826	1.05E+07	0.11	-4.04	3.62	0.07
E1	GPM2	6A	AX.89329955	6.32E+06	0.37	-626.11	3.28	0.05
E1	GPM2	6A	AX.89772670	2.88E+08	0.12	894.89	3.01	0.05
E1	GPM2	6A	AX.89342656	6.61E+06	0.37	-626.11	3.28	0.05
E1	GPM2	6A	AX.89434668	4.80E+08	0.05	-1328.91	3.11	0.05
E1	GPM2	6A	AX.89748354	1.05E+07	0.12	-980.58	3.52	0.06
E1	GPM2	6A	AX.89391096	6.73E+06	0.37	-626.11	3.28	0.05
E1	GPM2	6A	AX.89360412	1.05E+07	0.12	-980.58	3.52	0.06
E1	GPM2	6A	AX.89417186	8.70E+06	0.09	-1059.28	3.39	0.06
E1	GPM2	6A	AX.89628477	1.05E+07	0.12	-980.58	3.52	0.06
E1	GPM2	6A	AX.89356250	4.68E+08	0.05	-1328.91	3.11	0.05
E1	GY	6A	AX.89423567	5.95E+08	0.13	3.75	3.79	0.07

E1	GPM2	6A	AX.89633871	1.21E+07	0.14	-927.50	3.65	0.06
E1	GPM2	6A	AX.86164580	5.85E+08	0.06	1249.09	3.01	0.03
E1	GPM2	6A	AX.89671643	6.03E+08	0.27	704.27	3.45	0.06
E1	GPM2	6A	AX.89327067	6.73E+06	0.22	-738.22	3.38	0.06
E1	GPM2	6A	AX.89448872	6.61E+06	0.37	-626.11	3.28	0.05
E1	GY	6A	AX.89564541	5.95E+08	0.13	3.75	3.79	0.07
E1	GPM2	6A	AX.89590228	1.05E+07	0.11	-1046.42	3.81	0.07
E1	GPM2	6A	AX.89668076	1.16E+07	0.12	-1067.75	4.07	0.07
E1	GPM2	6A	AX.89573183	6.60E+06	0.37	-626.11	3.28	0.05
E1	GY	6A	AX.89628477	1.05E+07	0.12	-3.72	3.34	0.06
E1	GPM2	6A	AX.89717492	7.39E+06	0.23	-763.83	3.68	0.06
E1	TKW	6A	AX.89450530	5.85E+08	0.07	-1.64	3.05	0.05
E1	GY	6A	AX.89534738	5.95E+08	0.13	3.75	3.79	0.07
E1	GPM2	6A	AX.89685917	1.05E+07	0.12	-980.58	3.52	0.06
E1	GPM2	6A	AX.89630403	6.03E+08	0.32	702.15	3.75	0.07
E1	GY	6A	AX.89564135	5.95E+08	0.13	3.75	3.79	0.07
E1	GPM2	6A	AX.89437935	1.05E+07	0.11	-1046.42	3.81	0.07
E1	GY	6A	AX.89665621	5.87E+06	0.38	-2.70	3.92	0.07
E1	GY	6A	AX.89515569	5.56E+06	0.38	-2.70	3.92	0.07
E1	GPM2	6A	AX.89625041	1.23E+07	0.14	-864.50	3.31	0.06
E1	TKW	6A	AX.89403769	5.85E+08	0.07	-1.83	3.47	0.06
E1	TKW	6A	AX.89334479	5.85E+08	0.07	-1.83	3.47	0.06
E1	GPM2	6A	AX.89615289	1.23E+07	0.13	-1078.27	4.51	0.08
E1	TKW	6A	AX.89391018	8.16E+05	0.39	0.86	3.06	0.05
E1	TKW	6A	AX.89775066	5.85E+08	0.05	-2.01	3.36	0.06
E1	GPM2	6A	AX.89400057	1.09E+07	0.11	-1046.42	3.81	0.07
E1	TKW	6A	AX.89734907	5.85E+08	0.07	-1.83	3.47	0.06
E1	GPM2	6A	AX.86170193	6.73E+06	0.22	-738.22	3.38	0.06
E1	TKW	6A	AX.89419752	5.85E+08	0.06	-1.89	3.47	0.06
E1	TKW	6A	AX.89563727	5.85E+08	0.06	-1.85	3.14	0.06
E1	TKW	6A	AX.89332896	5.85E+08	0.06	-1.85	3.14	0.06
E1	GPM2	6A	AX.89773450	5.34E+08	0.07	-1208.34	3.21	0.05
E1	TKW	6A	AX.86164579	5.85E+08	0.07	-1.83	3.47	0.06
E1	TKW	6A	AX.86164577	5.85E+08	0.06	-1.85	3.14	0.06
E1	GPM2	6A	AX.89489584	1.05E+07	0.11	-1046.42	3.81	0.07
E1	TKW	6A	AX.89375285	5.85E+08	0.06	-1.85	3.14	0.06
E1	TKW	6A	AX.86164580	5.85E+08	0.06	-1.85	3.14	0.06
E1	TKW	6A	AX.89677520	5.85E+08	0.07	-1.83	3.47	0.06
E1	TKW	6A	AX.89725066	5.85E+08	0.07	-1.83	3.47	0.06
E2	SPM2	6A	AX.89392972	5.97E+08	0.15	-16.38	3.04	0.05
E2	GY	6A	AX.89722624	4.59E+06	0.39	2.59	5.01	0.08
E2	GY	6A	AX.89722624	4.59E+06	0.39	2.59	5.01	0.08
E2	GY	6A	AX.89734293	1.40E+07	0.05	-5.07	4.11	0.09
E2	SPM2	6A	AX.89524831	9.19E+07	0.18	-15.12	3.04	0.05
E2	GY	6A	AX.89687785	1.23E+07	0.13	-2.89	3.20	0.05
E2	GY	6A	AX.89687785	1.23E+07	0.13	-2.89	3.20	0.05
E2	GPM2	6A	AX.89491145	6.73E+06	0.23	-604.32	3.26	0.05
E2	GY	6A	AX.89404830	4.39E+06	0.37	-2.11	3.49	0.06
E2	GY	6A	AX.89404830	4.39E+06	0.37	-2.11	3.49	0.06
E2	GY	6A	AX.89640320	6.81E+06	0.38	-2.59	5.01	0.09

E2	GY	6A	AX.89640320	6.81E+06	0.38	-2.59	5.01	0.09
E2	GY	6A	AX.89312365	1.56E+07	0.06	-4.22	3.43	0.05
E2	GY	6A	AX.89593169	1.92E+07	0.13	-2.87	3.20	0.05
E2	GY	6A	AX.89593169	1.92E+07	0.13	-2.87	3.20	0.05
E2	GY	6A	AX.89417558	1.40E+07	0.05	-4.92	3.62	0.08
E2	GPS	6A	AX.89661491	5.69E+08	0.20	-1.70	3.22	0.05
E2	GY	6A	AX.89371566	1.40E+07	0.07	-4.94	5.05	0.10
E2	GPS	6A	AX.89729515	5.74E+08	0.33	-1.90	5.16	0.08
E2	GPS	6A	AX.89475169	5.74E+08	0.34	-1.82	4.82	0.07
E2	GY	6A	AX.89360250	1.40E+07	0.05	-4.92	3.62	0.08
E2	GY	6A	AX.89360250	1.40E+07	0.05	-4.92	3.62	0.08
E2	GPS	6A	AX.89450195	5.75E+08	0.39	-1.56	3.88	0.06
E2	SPM2	6A	AX.89491809	6.12E+08	0.41	-12.01	3.11	0.05
E2	GPS	6A	AX.89487900	6.03E+08	0.35	1.44	3.25	0.06
E2	SPM2	6A	AX.89588407	5.72E+08	0.15	16.53	3.15	0.05
E2	GPS	6A	AX.89596823	5.98E+08	0.33	1.60	3.78	0.06
E2	GPS	6A	AX.89670142	5.69E+08	0.20	-1.70	3.22	0.05
E2	GY	6A	AX.89361597	1.37E+07	0.05	-4.92	3.62	0.08
E2	GPS	6A	AX.89530982	5.74E+08	0.39	-1.53	3.75	0.05
E2	GY	6A	AX.89515569	5.56E+06	0.38	-2.01	3.23	0.05
E2	GY	6A	AX.89480000	1.55E+07	0.08	-4.33	4.45	0.09
E2	SPM2	6A	AX.89316743	5.97E+08	0.15	-16.30	3.08	0.05
E2	GPS	6A	AX.89623116	5.74E+08	0.39	-1.53	3.75	0.05
E2	GY	6A	AX.89363729	1.21E+07	0.11	-3.24	3.51	0.06
E2	GPS	6A	AX.89633608	5.75E+08	0.39	-1.41	3.27	0.05
E2	GY	6A	AX.86162594	1.56E+07	0.07	-3.98	3.66	0.05
E2	GY	6A	AX.86162594	1.56E+07	0.07	-3.98	3.66	0.05
E2	GY	6A	AX.89646266	1.05E+07	0.12	-2.99	3.25	0.05
E2	GY	6A	AX.89646266	1.05E+07	0.12	-2.99	3.25	0.05
E2	GPS	6A	AX.89725205	5.69E+08	0.36	-1.50	3.51	0.06
E2	GPS	6A	AX.89756239	5.99E+08	0.17	1.92	3.53	0.06
E2	GY	6A	AX.89340534	1.41E+07	0.05	-4.92	3.62	0.08
E2	GY	6A	AX.89340534	1.41E+07	0.05	-4.92	3.62	0.08
E2	GY	6A	AX.89412160	5.36E+06	0.38	-2.04	3.32	0.05
E2	GY	6A	AX.89412160	5.36E+06	0.38	-2.04	3.32	0.05
E2	GY	6A	AX.89340745	3.76E+06	0.43	-2.08	3.56	0.06
E2	GY	6A	AX.89340745	3.76E+06	0.43	-2.08	3.56	0.06
E2	GPS	6A	AX.89330835	5.72E+08	0.17	-1.91	3.49	0.05
E2	GPS	6A	AX.89410236	6.02E+08	0.34	1.45	3.25	0.06
E2	GY	6A	AX.89341428	5.36E+06	0.38	2.21	3.81	0.05
E2	GPS	6A	AX.89685955	6.01E+08	0.35	1.38	3.02	0.05
E2	GPS	6A	AX.89586345	5.74E+08	0.35	-1.78	4.66	0.07
E2	GPS	6A	AX.89541933	5.74E+08	0.39	-1.53	3.75	0.05
E2	GPS	6A	AX.89495941	2.47E+07	0.29	1.57	3.47	0.05
E2	GPS	6A	AX.89429569	5.74E+08	0.39	-1.53	3.75	0.05
E2	GY	6A	AX.89648510	3.76E+06	0.43	-2.08	3.56	0.06
E2	GY	6A	AX.89648510	3.76E+06	0.43	-2.08	3.56	0.06
E2	GPS	6A	AX.89457125	5.75E+08	0.39	-1.56	3.88	0.06
E2	GY	6A	AX.89723063	5.33E+06	0.38	-2.04	3.32	0.05
E2	GY	6A	AX.89723063	5.33E+06	0.38	-2.04	3.32	0.05

E2	GY	6A	AX.89568723	1.87E+07	0.11	-3.24	3.38	0.05
E2	GY	6A	AX.89568723	1.87E+07	0.11	-3.24	3.38	0.05
E2	GY	6A	AX.89448704	1.21E+07	0.13	-2.89	3.20	0.05
E2	GY	6A	AX.89448704	1.21E+07	0.13	-2.89	3.20	0.05
E2	GY	6A	AX.89576276	5.43E+06	0.38	-2.04	3.32	0.05
E2	GY	6A	AX.89576276	5.43E+06	0.38	-2.04	3.32	0.05
E2	GPS	6A	AX.89455710	5.74E+08	0.39	-1.53	3.75	0.05
E2	GPS	6A	AX.89642739	5.74E+08	0.34	-1.89	5.11	0.08
E2	GY	6A	AX.89448954	5.36E+06	0.38	-2.04	3.32	0.05
E2	GY	6A	AX.89448954	5.36E+06	0.38	-2.04	3.32	0.05
E2	GPS	6A	AX.89728123	2.48E+07	0.29	1.53	3.29	0.05
E2	GPM2	6A	AX.89734293	1.40E+07	0.05	-1162.89	3.41	0.07
E2	GPS	6A	AX.89517706	5.69E+08	0.20	-1.70	3.22	0.05
E2	GPS	6A	AX.89588407	5.72E+08	0.15	-1.88	3.18	0.05
E2	GY	6A	AX.89716589	5.13E+06	0.40	2.20	3.82	0.05
E2	GY	6A	AX.89716589	5.13E+06	0.40	2.20	3.82	0.05
E2	GPS	6A	AX.89628567	2.56E+07	0.38	1.37	3.09	0.05
E2	GY	6A	AX.89614453	6.81E+06	0.37	-2.71	5.40	0.09
E2	GY	6A	AX.89614453	6.81E+06	0.37	-2.71	5.40	0.09
E2	GPS	6A	AX.89399086	6.02E+08	0.35	1.44	3.25	0.06
E2	GY	6A	AX.89473894	3.77E+06	0.42	-2.04	3.46	0.06
E2	GY	6A	AX.89473894	3.77E+06	0.42	-2.04	3.46	0.06
E2	GY	6A	AX.89664596	5.36E+06	0.38	-2.04	3.32	0.05
E2	GPS	6A	AX.89410481	5.99E+08	0.17	1.92	3.53	0.06
E2	GY	6A	AX.89451585	1.24E+07	0.13	-3.64	4.87	0.08
E2	GY	6A	AX.89451585	1.24E+07	0.13	-3.64	4.87	0.08
E2	GY	6A	AX.89312365	1.56E+07	0.06	-4.22	3.43	0.05
E2	GY	6A	AX.89417558	1.40E+07	0.05	-4.92	3.62	0.08
E2	GY	6A	AX.89418722	1.24E+07	0.13	-3.64	4.87	0.08
E2	GY	6A	AX.89418722	1.24E+07	0.13	-3.64	4.87	0.08
E2	GPS	6A	AX.89614453	6.81E+06	0.37	-1.39	3.10	0.05
E2	GPS	6A	AX.89448675	2.48E+07	0.29	1.55	3.34	0.05
E2	GPS	6A	AX.89777656	6.03E+08	0.31	1.43	3.02	0.05
E2	GY	6A	AX.89525311	1.06E+07	0.10	-3.19	3.19	0.05
E2	GY	6A	AX.89525311	1.06E+07	0.10	-3.19	3.19	0.05
E2	GPS	6A	AX.89475441	5.74E+08	0.34	-1.83	4.83	0.07
E2	GY	6A	AX.89615289	1.23E+07	0.13	-2.89	3.20	0.05
E2	GY	6A	AX.89615289	1.23E+07	0.13	-2.89	3.20	0.05
E2	GY	6A	AX.89384579	3.75E+06	0.42	-2.04	3.46	0.06
E2	GY	6A	AX.89384579	3.75E+06	0.42	-2.04	3.46	0.06
E2	GY	6A	AX.89596776	3.76E+06	0.42	-2.04	3.46	0.06
E2	GY	6A	AX.89596776	3.76E+06	0.42	-2.04	3.46	0.06
E2	GY	6A	AX.89661668	5.36E+06	0.38	-2.04	3.32	0.05
E2	GY	6A	AX.89661668	5.36E+06	0.38	-2.04	3.32	0.05
E2	GY	6A	AX.89351001	1.55E+07	0.08	-4.33	4.45	0.09
E2	GY	6A	AX.89351001	1.55E+07	0.08	-4.33	4.45	0.09
E2	GY	6A	AX.89341428	5.36E+06	0.38	2.21	3.81	0.05
E2	GY	6A	AX.89599312	6.73E+06	0.40	1.94	3.10	0.04
E2	GY	6A	AX.89599312	6.73E+06	0.40	1.94	3.10	0.04
E2	GY	6A	AX.89530334	1.40E+07	0.05	-5.07	4.11	0.09

E2	GY	6A	AX.89530334	1.40E+07	0.05	-5.07	4.11	0.09
E2	GY	6A	AX.89730615	6.73E+06	0.40	1.94	3.10	0.04
E2	GY	6A	AX.89730615	6.73E+06	0.40	1.94	3.10	0.04
E2	GY	6A	AX.89459019	3.76E+06	0.43	-2.04	3.46	0.05
E2	GY	6A	AX.89459019	3.76E+06	0.43	-2.04	3.46	0.05
E2	GY	6A	AX.89320580	1.85E+07	0.10	-3.65	4.04	0.07
E2	GY	6A	AX.89320580	1.85E+07	0.10	-3.65	4.04	0.07
E2	GPS	6A	AX.89422901	5.74E+08	0.33	-1.93	5.31	0.08
E2	GY	6A	AX.89389958	5.60E+06	0.40	-2.07	3.45	0.05
E2	GY	6A	AX.89389958	5.60E+06	0.40	-2.07	3.45	0.05
E2	GY	6A	AX.89449758	5.13E+06	0.41	2.29	4.13	0.06
E2	GY	6A	AX.89449758	5.13E+06	0.41	2.29	4.13	0.06
E2	GY	6A	AX.89602780	5.33E+06	0.40	-2.15	3.68	0.06
E2	GY	6A	AX.89602780	5.33E+06	0.40	-2.15	3.68	0.06
E2	GY	6A	AX.89716829	5.32E+06	0.38	-2.04	3.32	0.05
E2	GY	6A	AX.89716829	5.32E+06	0.38	-2.04	3.32	0.05
E2	GY	6A	AX.89566655	1.86E+07	0.11	-3.82	4.52	0.08
E2	GY	6A	AX.89566655	1.86E+07	0.11	-3.82	4.52	0.08
E2	GY	6A	AX.89664596	5.36E+06	0.38	-2.04	3.32	0.05
E2	GY	6A	AX.89568464	5.39E+07	0.46	1.93	3.18	0.04
E2	GY	6A	AX.89568464	5.39E+07	0.46	1.93	3.18	0.04
E2	GY	6A	AX.89544771	5.12E+06	0.37	2.47	4.58	0.07
E2	GY	6A	AX.89544771	5.12E+06	0.37	2.47	4.58	0.07
E2	GY	6A	AX.89393684	4.58E+06	0.37	-2.11	3.49	0.06
E2	GY	6A	AX.89393684	4.58E+06	0.37	-2.11	3.49	0.06
E2	GY	6A	AX.89406931	7.50E+07	0.07	-4.08	3.44	0.07
E2	GY	6A	AX.89394835	5.13E+06	0.38	-2.04	3.32	0.05
E2	GY	6A	AX.89394835	5.13E+06	0.38	-2.04	3.32	0.05
E2	GPS	6A	AX.89635017	5.74E+08	0.34	-1.62	3.93	0.06
E2	GY	6A	AX.89465182	3.80E+06	0.43	-2.08	3.56	0.06
E2	GY	6A	AX.89465182	3.80E+06	0.43	-2.08	3.56	0.06
E2	GY	6A	AX.89327045	1.45E+07	0.06	-4.16	3.36	0.07
E2	GY	6A	AX.89327045	1.45E+07	0.06	-4.16	3.36	0.07
E2	GPS	6A	AX.89506887	5.73E+08	0.29	-1.72	4.04	0.06
E2	GY	6A	AX.89415699	3.76E+06	0.42	-2.04	3.46	0.06
E2	GY	6A	AX.89684726	5.36E+06	0.38	-2.01	3.23	0.05
E2	GY	6A	AX.89684726	5.36E+06	0.38	-2.01	3.23	0.05
E2	GPM2	6A	AX.89544771	5.12E+06	0.37	529.44	3.35	0.06
E2	GY	6A	AX.89734293	1.40E+07	0.05	-5.07	4.11	0.09
E2	GY	6A	AX.89734380	5.53E+06	0.39	2.19	3.76	0.05
E2	GY	6A	AX.89754513	1.56E+07	0.07	-3.98	3.66	0.05
E2	GY	6A	AX.89754513	1.56E+07	0.07	-3.98	3.66	0.05
E2	GY	6A	AX.89402721	5.48E+06	0.39	2.08	3.47	0.05
E2	GY	6A	AX.89402721	5.48E+06	0.39	2.08	3.47	0.05
E2	GPS	6A	AX.89767068	5.75E+08	0.38	-1.48	3.49	0.05
E2	GY	6A	AX.89581832	1.87E+07	0.10	-3.72	4.01	0.07
E2	GY	6A	AX.89581832	1.87E+07	0.10	-3.72	4.01	0.07
E2	GY	6A	AX.89403262	1.21E+07	0.11	-3.14	3.19	0.05
E2	GY	6A	AX.89403262	1.21E+07	0.11	-3.14	3.19	0.05
E2	GPM2	6A	AX.89717492	7.39E+06	0.23	-594.77	3.25	0.05

E2	GPS	6A	AX.89357456	5.74E+08	0.34	-1.89	5.11	0.08
E2	GPS	6A	AX.89671956	6.01E+08	0.35	1.44	3.25	0.06
E2	GPS	6A	AX.89614685	5.74E+08	0.34	-1.82	4.82	0.07
E2	GPS	6A	AX.89770182	5.74E+08	0.39	-1.56	3.88	0.06
E2	GY	6A	AX.89369831	5.55E+06	0.38	-1.98	3.16	0.05
E2	GY	6A	AX.89369831	5.55E+06	0.38	-1.98	3.16	0.05
E2	GY	6A	AX.89498345	5.36E+06	0.39	-2.04	3.34	0.05
E2	GY	6A	AX.89665621	5.87E+06	0.38	-2.01	3.23	0.05
E2	GPS	6A	AX.89323135	5.74E+08	0.34	-1.89	5.11	0.08
E2	GPS	6A	AX.86167599	5.74E+08	0.33	-1.86	4.95	0.08
E2	GY	6A	AX.89406931	7.50E+07	0.07	-4.08	3.44	0.07
E2	GY	6A	AX.89407050	4.10E+06	0.43	-2.09	3.60	0.06
E2	GY	6A	AX.89407050	4.10E+06	0.43	-2.09	3.60	0.06
E2	GY	6A	AX.89371566	1.40E+07	0.07	-4.94	5.05	0.10
E2	GPS	6A	AX.89762980	5.74E+08	0.39	-1.56	3.88	0.06
E2	GPS	6A	AX.89697696	5.74E+08	0.34	-1.85	4.95	0.08
E2	GPS	6A	AX.86164444	5.70E+08	0.21	-1.62	3.01	0.04
E2	GPS	6A	AX.89333624	5.74E+08	0.32	-1.78	4.53	0.07
E2	GPS	6A	AX.89678981	5.74E+08	0.34	-1.82	4.82	0.07
E2	GPS	6A	AX.89369691	6.02E+08	0.31	1.43	3.02	0.05
E2	GPS	6A	AX.86164450	5.70E+08	0.21	-1.62	3.01	0.04
E2	GPS	6A	AX.89551123	5.65E+08	0.36	-1.43	3.25	0.06
E2	GPS	6A	AX.89389161	5.74E+08	0.34	-1.86	5.00	0.08
E2	GPS	6A	AX.89344236	5.74E+08	0.34	-1.89	5.11	0.08
E2	GPS	6A	AX.89670104	5.70E+08	0.35	-1.56	3.72	0.07
E2	GPS	6A	AX.89537531	2.47E+07	0.29	1.55	3.34	0.05
E2	GY	6A	AX.89361597	1.37E+07	0.05	-4.92	3.62	0.08
E2	GY	6A	AX.89515569	5.56E+06	0.38	-2.01	3.23	0.05
E2	GPS	6A	AX.89318220	5.70E+08	0.21	-1.62	3.01	0.04
E2	GPS	6A	AX.89452196	5.74E+08	0.33	-1.88	5.06	0.08
E2	GPS	6A	AX.89431171	5.74E+08	0.39	-1.53	3.75	0.05
E2	GY	6A	AX.89480000	1.55E+07	0.08	-4.33	4.45	0.09
E2	GPS	6A	AX.89589873	5.74E+08	0.39	-1.53	3.75	0.05
E2	GPS	6A	AX.89504495	2.70E+07	0.37	1.38	3.05	0.05
E2	GPS	6A	AX.89450221	2.49E+07	0.38	1.37	3.09	0.05
E2	GPS	6A	AX.89514445	5.74E+08	0.34	-1.84	4.94	0.08
E2	GY	6A	AX.89363729	1.21E+07	0.11	-3.24	3.51	0.06
E2	GPS	6A	AX.89539113	5.82E+08	0.23	-1.57	3.01	0.04
E2	GPS	6A	AX.89531685	2.56E+07	0.38	1.37	3.09	0.05
E2	GPS	6A	AX.89757572	5.73E+08	0.17	-1.91	3.55	0.05
E2	GPS	6A	AX.89476581	4.66E+06	0.31	1.46	3.16	0.05
E2	GPS	6A	AX.89675413	5.74E+08	0.34	-1.86	5.00	0.08
E2	GPS	6A	AX.89338802	2.48E+07	0.29	1.49	3.13	0.05
E2	GPS	6A	AX.89506289	5.63E+08	0.18	-1.71	3.04	0.04
E2	GPS	6A	AX.89552426	5.74E+08	0.34	-1.89	5.11	0.08
E2	GPS	6A	AX.89334292	5.74E+08	0.34	-1.86	5.00	0.08
E2	GPS	6A	AX.89712898	2.48E+07	0.38	1.37	3.09	0.05
E2	GPS	6A	AX.89574596	5.74E+08	0.33	-1.90	5.16	0.08
E2	GPM2	6A	AX.89329900	6.73E+06	0.23	-604.32	3.26	0.05
E2	GPS	6A	AX.89600827	2.34E+07	0.49	-1.39	3.31	0.06

E2	GPM2	6A	AX.89340534	1.41E+07	0.05	-1225.42	3.47	0.07
E2	GY	6A	AX.89734380	5.53E+06	0.39	2.19	3.76	0.05
E2	GPS	6A	AX.89384690	5.82E+08	0.15	-1.98	3.40	0.05
E2	GPS	6A	AX.89666483	5.75E+08	0.38	-1.54	3.77	0.05
E2	GPS	6A	AX.89372359	5.74E+08	0.34	-1.81	4.77	0.07
E2	GPS	6A	AX.89615146	5.83E+08	0.23	-1.57	3.01	0.04
E2	GPS	6A	AX.89387258	5.69E+08	0.20	-1.70	3.22	0.05
E2	GPS	6A	AX.89529892	5.74E+08	0.34	-1.89	5.11	0.08
E2	GPS	6A	AX.89342065	5.74E+08	0.33	-1.87	5.00	0.08
E2	GPM2	6A	AX.89775066	5.85E+08	0.05	1103.92	3.08	0.02
E2	GPS	6A	AX.89709762	5.82E+08	0.23	-1.57	3.01	0.04
E2	GPS	6A	AX.89561094	6.03E+08	0.31	1.43	3.02	0.05
E2	GPS	6A	AX.89435259	5.70E+08	0.35	-1.56	3.72	0.07
E2	GPS	6A	AX.89351448	5.74E+08	0.34	-1.79	4.70	0.07
E2	GPS	6A	AX.89542231	2.78E+07	0.34	1.40	3.07	0.05
E2	GPS	6A	AX.89741579	5.74E+08	0.34	-1.89	5.11	0.08
E2	GY	6A	AX.89567056	1.57E+07	0.09	-4.02	4.44	0.06
E2	GPS	6A	AX.89479044	6.02E+08	0.35	1.44	3.25	0.06
E2	GPS	6A	AX.89633350	5.74E+08	0.34	-1.82	4.82	0.07
E2	GY	6A	AX.89451275	5.84E+07	0.07	-3.91	3.55	0.04
E2	GY	6A	AX.89451275	5.84E+07	0.07	-3.91	3.55	0.04
E2	GPS	6A	AX.89678625	5.83E+08	0.23	-1.57	3.01	0.04
E2	GY	6A	AX.89738527	1.87E+07	0.13	-2.87	3.16	0.05
E2	GY	6A	AX.89738527	1.87E+07	0.13	-2.87	3.16	0.05
E2	GPS	6A	AX.89741467	5.68E+08	0.36	-1.50	3.51	0.06
E2	GPS	6A	AX.89456764	2.57E+07	0.35	1.41	3.13	0.05
E2	GY	6A	AX.89665621	5.87E+06	0.38	-2.01	3.23	0.05
E2	GPS	6A	AX.89708700	6.03E+08	0.35	1.44	3.25	0.06
E2	GPS	6A	AX.89334703	2.63E+07	0.37	1.49	3.50	0.06
E2	GPS	6A	AX.89380311	2.48E+07	0.28	1.57	3.39	0.05
E2	GY	6A	AX.89760277	5.81E+07	0.07	-3.91	3.55	0.04
E2	GPS	6A	AX.89362676	5.73E+08	0.16	-2.00	3.70	0.06
E2	GPS	6A	AX.86162777	2.49E+07	0.38	1.37	3.09	0.05
E2	GPS	6A	AX.89701627	5.74E+08	0.39	-1.53	3.75	0.05
E2	GPS	6A	AX.89602100	5.74E+08	0.34	-1.86	5.00	0.08
E2	GPS	6A	AX.89495733	5.74E+08	0.34	-1.89	5.11	0.08
E2	GPS	6A	AX.89542055	5.73E+08	0.15	-2.06	3.65	0.06
E2	GY	6A	AX.89348394	4.60E+06	0.43	-2.19	3.90	0.06
E2	GY	6A	AX.89348394	4.60E+06	0.43	-2.19	3.90	0.06
E2	GPS	6A	AX.89439930	5.74E+08	0.35	-1.78	4.66	0.07
E2	GY	6A	AX.89717492	7.39E+06	0.23	-2.58	3.89	0.06
E2	GPS	6A	AX.89494618	5.74E+08	0.35	-1.78	4.66	0.07
E2	GY	6A	AX.89433276	5.48E+06	0.43	2.32	4.29	0.06
E2	GPM2	6A	AX.89724488	6.73E+06	0.23	-604.32	3.26	0.05
E2	GY	6A	AX.89743239	1.87E+07	0.10	-3.65	4.04	0.07
E2	GY	6A	AX.89743239	1.87E+07	0.10	-3.65	4.04	0.07
E2	GY	6A	AX.89492323	6.72E+06	0.40	2.04	3.38	0.04
E2	GY	6A	AX.89492323	6.72E+06	0.40	2.04	3.38	0.04
E2	GY	6A	AX.89595207	3.77E+06	0.43	-2.11	3.66	0.06
E2	GPS	6A	AX.89320670	2.47E+07	0.29	1.55	3.34	0.05

E2	GPS	6A	AX.89346075	5.73E+08	0.17	-1.91	3.49	0.05
E2	GPS	6A	AX.89668027	5.69E+08	0.20	-1.70	3.22	0.05
E2	GPM2	6A	AX.89327067	6.73E+06	0.23	-604.32	3.26	0.05
E2	GPS	6A	AX.89727124	4.67E+06	0.33	1.45	3.20	0.05
E2	GY	6A	AX.89753335	3.34E+06	0.22	2.32	3.10	0.07
E2	GY	6A	AX.89624935	1.21E+07	0.13	-2.89	3.20	0.05
E2	GY	6A	AX.89624935	1.21E+07	0.13	-2.89	3.20	0.05
E2	GPS	6A	AX.89658052	5.98E+08	0.17	1.92	3.53	0.06
E2	GY	6A	AX.89567056	1.57E+07	0.09	-4.02	4.44	0.06
E2	GPS	6A	AX.89679693	5.75E+08	0.39	-1.56	3.88	0.06
E2	GY	6A	AX.89498345	5.36E+06	0.39	-2.04	3.34	0.05
E2	GPS	6A	AX.89521580	2.48E+07	0.29	1.55	3.34	0.05
E2	GY	6A	AX.89760277	5.81E+07	0.07	-3.91	3.55	0.04
E2	GPS	6A	AX.89543599	3.12E+07	0.47	1.35	3.15	0.05
E2	GPS	6A	AX.89551569	5.74E+08	0.34	-1.86	5.00	0.08
E2	GPS	6A	AX.89496208	5.79E+08	0.15	-1.96	3.42	0.05
E2	GPS	6A	AX.89514128	5.79E+08	0.15	-1.92	3.17	0.05
E2	GY	6A	AX.89595207	3.77E+06	0.43	-2.11	3.66	0.06
E2	GPS	6A	AX.89344114	5.82E+08	0.20	-1.80	3.48	0.05
E2	GY	6A	AX.89717492	7.39E+06	0.23	-2.58	3.89	0.06
E2	GPS	6A	AX.89372687	6.02E+08	0.31	1.43	3.02	0.05
E2	GPS	6A	AX.89338412	5.74E+08	0.34	-1.89	5.11	0.08
E2	GPM2	6A	AX.89530334	1.40E+07	0.05	-1162.89	3.41	0.07
E2	GY	6A	AX.89433276	5.48E+06	0.43	2.32	4.29	0.06
E2	GPS	6A	AX.89380329	5.74E+08	0.34	-1.85	4.95	0.08
E2	GPS	6A	AX.89436826	6.03E+08	0.35	1.44	3.25	0.06
E2	GY	6A	AX.89561952	1.45E+07	0.09	-3.85	3.97	0.07
E2	GY	6A	AX.89561952	1.45E+07	0.09	-3.85	3.97	0.07
E2	GPS	6A	AX.89533325	5.74E+08	0.34	-1.86	5.00	0.08
E2	GPS	6A	AX.89632590	5.73E+08	0.17	-1.91	3.49	0.05
E2	GPS	6A	AX.89725443	5.74E+08	0.39	-1.53	3.75	0.05
E2	GPS	6A	AX.89369974	5.74E+08	0.39	-1.53	3.75	0.05
E2	GPS	6A	AX.89531960	5.74E+08	0.34	-1.87	5.05	0.08
E2	GY	6A	AX.89415699	3.76E+06	0.42	-2.04	3.46	0.06
E2	GPS	6A	AX.89500120	5.74E+08	0.35	-1.83	4.89	0.08
E2	GPS	6A	AX.89363090	5.74E+08	0.35	-1.83	4.89	0.08
E2	GPS	6A	AX.89712497	5.83E+08	0.23	-1.57	3.01	0.04
E2	GPM2	6A	AX.86170193	6.73E+06	0.23	-604.32	3.26	0.05
E2	GPS	6A	AX.89320322	5.98E+08	0.33	1.54	3.56	0.05
E2	GY	6A	AX.89753335	3.34E+06	0.22	2.32	3.10	0.07
E2	GPS	6A	AX.89675302	2.37E+07	0.36	1.49	3.47	0.06
E2	GPS	6A	AX.89618501	5.74E+08	0.33	-1.90	5.16	0.08
E2	GPS	6A	AX.89321660	5.70E+08	0.35	-1.60	3.86	0.07
E2	GPS	6A	AX.89770131	5.74E+08	0.34	-1.86	5.00	0.08
E2	GPS	6A	AX.89560637	5.74E+08	0.34	-1.86	5.00	0.08
E2	GPS	6A	AX.89458005	5.83E+08	0.23	-1.57	3.01	0.04
E2	GPS	6A	AX.89630899	5.83E+08	0.23	-1.57	3.01	0.04
E2	GPS	6A	AX.89531359	2.56E+07	0.36	1.61	3.97	0.06
E2	GPM2	6A	AX.89658761	6.73E+06	0.23	-604.32	3.26	0.05
E2	GPS	6A	AX.89364988	5.69E+08	0.30	-1.65	3.80	0.06

E2	GPS	6A	AX.89716670	5.69E+08	0.36	-1.50	3.51	0.06
E2	GPS	6A	AX.89631336	5.74E+08	0.39	-1.53	3.75	0.05
E2	GPS	6A	AX.89492509	5.75E+08	0.38	-1.57	3.85	0.06
E2	GPS	6A	AX.89465934	5.69E+08	0.20	-1.70	3.22	0.05
E2	GPS	6A	AX.89498743	5.74E+08	0.34	-1.86	5.00	0.08
E2	GPS	6A	AX.89764070	5.74E+08	0.33	-1.90	5.14	0.08
E2	GPS	6A	AX.89353374	6.02E+08	0.31	1.43	3.02	0.05
E2	GPS	6A	AX.89715660	5.82E+08	0.23	-1.57	3.01	0.04
E2	GPS	6A	AX.89617092	5.69E+08	0.17	-1.83	3.30	0.05
E2	GPS	6A	AX.89543860	5.74E+08	0.34	-1.86	5.00	0.08
E2	GPS	6A	AX.89506754	5.98E+08	0.18	1.88	3.51	0.06
E2	GPS	6A	AX.89641015	3.09E+07	0.47	1.35	3.15	0.05
E2	GPS	6A	AX.89312948	5.75E+08	0.38	-1.63	4.15	0.06
E2	GPS	6A	AX.89532249	6.03E+08	0.35	1.44	3.25	0.06
E2	GPS	6A	AX.89458593	6.02E+08	0.35	1.44	3.25	0.06
E2	GPS	6A	AX.89740694	5.82E+08	0.20	-1.80	3.48	0.05
E2	GPS	6A	AX.89504429	2.49E+07	0.38	1.37	3.09	0.05
E2	GPS	6A	AX.89432790	5.99E+08	0.23	1.67	3.36	0.06
E2	GPS	6A	AX.89589555	5.74E+08	0.39	-1.53	3.75	0.05
E2	GPS	6A	AX.89687959	5.74E+08	0.39	-1.53	3.75	0.05
E2	GPS	6A	AX.89563488	5.74E+08	0.34	-1.82	4.82	0.07
E2	GPS	6A	AX.89620291	2.39E+07	0.45	-1.33	3.03	0.05
E2	GPS	6A	AX.89558297	5.72E+08	0.19	-1.91	3.72	0.06
E2	GPM2	6A	AX.89417558	1.40E+07	0.05	-1225.42	3.47	0.07
E2	GPS	6A	AX.89441093	6.02E+08	0.35	1.44	3.25	0.06
E2	GPM2	6A	AX.89340253	7.15E+06	0.12	-781.58	3.20	0.05
E2	GPS	6A	AX.89671323	5.74E+08	0.34	-1.89	5.11	0.08
E2	GPS	6A	AX.89497354	5.72E+08	0.19	-1.91	3.72	0.06
E2	GPS	6A	AX.89527207	5.75E+08	0.38	-1.54	3.77	0.05
E2	GPM2	6A	AX.89722624	4.59E+06	0.39	543.29	3.53	0.06
E2	GPM2	6A	AX.89360250	1.40E+07	0.05	-1225.42	3.47	0.07
E2	GPS	6A	AX.89583112	2.49E+07	0.28	1.59	3.49	0.05
E2	GPM2	6A	AX.89361597	1.37E+07	0.05	-1225.42	3.47	0.07
E2	GPS	6A	AX.89728533	5.75E+08	0.30	-1.47	3.15	0.04
E2	GPS	6A	AX.89315916	5.73E+08	0.17	-2.00	3.77	0.06
E2	GPS	6A	AX.89712175	2.56E+07	0.38	1.37	3.09	0.05
E2	GPS	6A	AX.89470719	5.74E+08	0.34	-1.89	5.11	0.08
E2	GPS	6A	AX.89597469	5.74E+08	0.34	-1.89	5.11	0.08
E2	GPM2	6A	AX.89371566	1.40E+07	0.07	-984.16	3.28	0.06
E2	GPS	6A	AX.89549685	6.01E+08	0.35	1.44	3.25	0.06
E3	GPS	6A	AX.89325506	1.67E+06	0.08	3.77	3.17	0.02
E3	GPS	6A	AX.89627110	5.99E+08	0.11	3.21	3.25	0.04
E3	GPS	6A	AX.89465276	5.99E+08	0.11	3.21	3.25	0.04
E3	GPS	6A	AX.89358478	5.99E+08	0.11	3.21	3.25	0.04
E3	GPS	6A	AX.89722217	5.99E+08	0.11	3.21	3.25	0.04
E3	GPS	6A	AX.89484965	5.99E+08	0.11	3.21	3.25	0.04
E3	GPS	6A	AX.89755431	5.99E+08	0.11	3.21	3.25	0.04
E3	GY	6A	AX.89561657	6.03E+08	0.10	-4.78	3.35	0.02
E3	SPM2	6A	AX.89311728	6.14E+08	0.35	21.52	3.28	0.07
E3	SPM2	6A	AX.89354031	6.14E+08	0.33	28.14	5.18	0.11

E3	SPM2	6A	X.89411770_OT	6.14E+08	0.36	22.44	3.55	0.08
E3	SPM2	6A	AX.89337491	6.14E+08	0.37	21.30	3.27	0.08
E3	SPM2	6A	X.89511535_OT	6.14E+08	0.36	22.54	3.56	0.08
E3	SPM2	6A	X.89403264_OT	6.14E+08	0.36	22.54	3.56	0.08
E3	SPM2	6A	AX.89687446	6.14E+08	0.33	24.17	3.90	0.08
E3	SPM2	6A	AX.89728011	6.14E+08	0.34	27.03	4.87	0.10
E3	SPM2	6A	AX.89406500	6.14E+08	0.34	25.89	4.54	0.09
E3	SPM2	6A	AX.89664583	6.14E+08	0.35	25.76	4.53	0.10
E3	SPM2	6A	AX.89372252	6.14E+08	0.33	28.77	5.39	0.11
E3	SPM2	6A	AX.89528310	6.11E+08	0.11	-30.20	3.08	0.05
E3	SPM2	6A	AX.89473928	6.13E+08	0.37	21.56	3.33	0.07
E4	GPM2	6A	AX.89775895	1.19E+07	0.17	548.41	3.01	0.03
E4	GPM2	6A	AX.89399614	1.20E+07	0.18	606.82	3.80	0.04
E4	GPM2	6A	AX.89569211	1.20E+07	0.18	606.82	3.80	0.04
E4	GPM2	6A	AX.89333267	1.24E+07	0.21	622.87	4.14	0.03
E4	GPM2	6A	AX.89442264	1.19E+07	0.17	548.41	3.01	0.03
E4	GPM2	6A	AX.89342703	8.17E+06	0.21	526.50	3.03	0.02
E4	GPM2	6A	AX.89363939	8.17E+06	0.21	551.68	3.20	0.02
E4	GPM2	6A	AX.89554180	1.24E+07	0.21	602.14	3.87	0.03
E4	GPM2	6A	AX.89416807	1.24E+07	0.21	602.14	3.87	0.03
E4	GPM2	6A	AX.89513747	1.24E+07	0.20	544.38	3.18	0.02
E4	GPM2	6A	AX.89451585	1.24E+07	0.46	515.82	4.03	0.07
E4	GY	6A	AX.89767404	1.09E+07	0.45	1.49	3.10	0.04
E4	GY	6A	X.89481606_OT	1.16E+07	0.45	-1.45	3.01	0.03
E4	GPS	6A	AX.89650935	5.97E+08	0.25	-1.77	3.11	0.10
E4	GPS	6A	AX.89776944	5.97E+08	0.26	-1.74	3.21	0.09
E4	GPS	6A	AX.89664759	5.97E+08	0.27	-1.73	3.25	0.09
E4	GPS	6A	AX.89429065	5.97E+08	0.26	-1.80	3.38	0.09
E4	GPS	6A	AX.89713144	5.97E+08	0.26	-1.74	3.21	0.09
E4	GPS	6A	AX.89626879	5.97E+08	0.26	-1.74	3.21	0.09
E4	GPS	6A	AX.89684654	5.97E+08	0.27	-1.73	3.25	0.09
E4	GPS	6A	AX.89720272	5.97E+08	0.27	-1.73	3.25	0.09
E4	GPS	6A	AX.89462597	5.97E+08	0.27	-1.73	3.25	0.09
E4	GPS	6A	AX.89578558	5.97E+08	0.26	-1.74	3.21	0.09
E4	GPS	6A	AX.89469783	5.97E+08	0.27	-1.73	3.25	0.09
E4	GPS	6A	AX.89313811	5.97E+08	0.26	-1.78	3.39	0.09
E4	GPS	6A	AX.89540414	5.97E+08	0.27	-1.71	3.12	0.09
E4	GPS	6A	AX.89558123	5.97E+08	0.27	-1.75	3.27	0.09
E4	GPS	6A	AX.89416492	5.97E+08	0.26	-1.78	3.39	0.09
E4	GPS	6A	AX.89454374	5.97E+08	0.27	-1.73	3.25	0.09
E4	GPS	6A	AX.89712144	5.97E+08	0.27	-1.73	3.25	0.09
E4	GPS	6A	AX.89508072	5.98E+08	0.27	-1.73	3.25	0.09
E4	GPS	6A	AX.89776921	5.98E+08	0.27	-1.80	3.47	0.10
E4	GPS	6A	AX.89685931	5.98E+08	0.26	-1.78	3.39	0.09
E4	GPS	6A	AX.89562610	5.98E+08	0.25	-1.92	3.65	0.09
E4	GY	6A	AX.89399614	1.20E+07	0.19	1.84	3.09	0.03
E4	GY	6A	AX.89569211	1.20E+07	0.19	1.84	3.09	0.03
E4	GY	6A	AX.89333267	1.24E+07	0.21	1.89	3.41	0.02
E4	GY	6A	AX.89554180	1.24E+07	0.21	1.80	3.09	0.02
E4	GY	6A	AX.89416807	1.24E+07	0.21	1.80	3.09	0.02

E4	GY	6A	AX.89488628	5.21E+08	0.07	-2.81	3.03	0.04
E4	SPM2	6A	AX.89349891	1.44E+07	0.12	24.78	3.12	0.04
E4	SPM2	6A	AX.89751817	2.70E+07	0.26	19.87	3.50	0.06
E4	SPM2	6A	X.89603108_OT	5.80E+08	0.13	26.20	3.52	0.06
E4	SPM2	6A	X.89643197_OT	5.80E+08	0.12	25.11	3.18	0.05
E4	SPM2	6A	X.89571346_OT	6.10E+08	0.21	21.86	3.30	0.09
E4	TKW	6A	AX.89582899	3.50E+07	0.32	0.93	3.10	0.00
E1	SPM2	6B	AX.89685941	6.77E+08	0.07	33.60	3.17	0.06
E1	SPM2	6B	AX.89656381	6.77E+08	0.06	34.11	3.07	0.05
E1	GY	6B	AX.86166228	6.57E+07	0.15	-3.21	3.09	0.05
E1	GY	6B	AX.89446890	8.73E+07	0.09	-4.50	3.73	0.06
E1	GY	6B	AX.89517996	1.29E+08	0.07	-5.14	4.11	0.07
E1	GY	6B	AX.89350910	2.35E+08	0.16	-3.38	3.51	0.05
E1	GY	6B	AX.89350910	2.35E+08	0.16	-3.38	3.51	0.05
E1	GY	6B	AX.89568337	4.67E+08	0.21	-2.84	3.19	0.05
E1	GY	6B	AX.89660252	1.29E+08	0.07	-5.14	4.11	0.07
E1	SPM2	6B	AX.89519115	6.78E+08	0.07	33.60	3.17	0.06
E1	GY	6B	AX.89388402	1.25E+08	0.22	-2.78	3.15	0.05
E1	GY	6B	AX.89421263	1.28E+08	0.07	-5.42	4.28	0.07
E1	GY	6B	AX.89660252	1.29E+08	0.07	-5.14	4.11	0.07
E1	GY	6B	AX.89421263	1.28E+08	0.07	-5.42	4.28	0.07
E1	GY	6B	AX.89463681	4.65E+08	0.20	-2.86	3.09	0.05
E1	GY	6B	AX.89613195	2.34E+08	0.16	-3.06	3.02	0.04
E1	GY	6B	AX.89328519	4.65E+08	0.20	-2.86	3.09	0.05
E1	GY	6B	AX.89332352	4.65E+08	0.20	-2.86	3.09	0.05
E1	GY	6B	AX.89462797	1.29E+08	0.08	-5.25	4.48	0.07
E1	GY	6B	AX.89496663	2.37E+08	0.16	-3.06	3.02	0.04
E1	GY	6B	AX.89333381	6.10E+08	0.17	-3.14	3.29	0.06
E1	GY	6B	AX.89496663	2.37E+08	0.16	-3.06	3.02	0.04
E1	GY	6B	AX.89449986	4.67E+08	0.21	-2.84	3.19	0.05
E1	GY	6B	AX.89530519	6.11E+08	0.18	-3.09	3.26	0.06
E1	GY	6B	AX.89446890	8.73E+07	0.09	-4.50	3.73	0.06
E1	GY	6B	AX.89517996	1.29E+08	0.07	-5.14	4.11	0.07
E1	GY	6B	AX.86166228	6.57E+07	0.15	-3.21	3.09	0.05
E1	GY	6B	AX.89532953	4.63E+08	0.20	-2.86	3.09	0.05
E1	GY	6B	AX.89319508	1.29E+08	0.07	-5.21	4.21	0.07
E1	GY	6B	AX.89323627	1.30E+08	0.07	-5.52	4.65	0.08
E1	SPM2	6B	AX.89355439	6.77E+08	0.06	34.11	3.07	0.05
E1	GY	6B	AX.89384533	2.34E+08	0.16	-3.06	3.02	0.04
E1	GY	6B	AX.89329305	6.11E+08	0.18	-3.09	3.26	0.06
E1	GY	6B	AX.89477111	2.35E+08	0.16	-3.06	3.02	0.04
E1	GY	6B	AX.89454965	6.06E+08	0.13	-3.35	3.02	0.05
E1	GY	6B	AX.89342187	6.09E+08	0.13	-3.35	3.02	0.05
E1	GY	6B	AX.89620029	1.29E+08	0.07	-4.55	3.33	0.05
E1	GY	6B	AX.89312342	1.25E+08	0.22	-2.78	3.15	0.05
E1	GY	6B	AX.89357544	4.66E+08	0.20	-2.86	3.09	0.05
E1	GY	6B	AX.89413801	4.66E+08	0.20	-2.86	3.09	0.05
E1	GY	6B	AX.89417457	4.66E+08	0.21	-2.92	3.29	0.05
E1	GY	6B	AX.89388402	1.25E+08	0.22	-2.78	3.15	0.05
E1	GY	6B	AX.89593764	6.12E+08	0.17	-2.98	3.01	0.05

E1	GY	6B	AX.89318747	4.63E+08	0.20	-2.86	3.09	0.05
E1	GY	6B	AX.89325291	1.28E+08	0.07	-5.67	4.38	0.07
E1	GY	6B	AX.89402631	1.28E+08	0.07	-5.42	4.28	0.07
E1	GY	6B	AX.89430381	2.39E+08	0.16	-3.06	3.02	0.04
E1	GY	6B	AX.89332261	6.06E+08	0.13	-3.35	3.02	0.05
E1	GY	6B	AX.89382186	4.62E+08	0.20	-2.86	3.09	0.05
E1	SPM2	6B	AX.89691460	6.77E+08	0.06	36.11	3.38	0.06
E1	GY	6B	AX.89415017	4.64E+08	0.20	-2.86	3.09	0.05
E1	GY	6B	AX.89745770	1.28E+08	0.07	-5.14	4.11	0.07
E1	GY	6B	AX.89318747	4.63E+08	0.20	-2.86	3.09	0.05
E1	GY	6B	AX.89446764	4.65E+08	0.20	-2.86	3.09	0.05
E1	SPM2	6B	AX.89526757	6.77E+08	0.07	33.60	3.17	0.06
E1	GY	6B	AX.89347329	4.64E+08	0.20	-2.86	3.09	0.05
E1	GY	6B	AX.89356678	4.63E+08	0.21	-2.84	3.18	0.05
E1	GY	6B	AX.89356678	4.63E+08	0.21	-2.84	3.18	0.05
E1	GY	6B	AX.89478059	4.67E+08	0.21	-2.84	3.19	0.05
E1	GY	6B	AX.89327088	4.64E+08	0.20	-2.86	3.09	0.05
E1	GY	6B	AX.89560249	4.27E+07	0.36	-2.46	3.26	0.05
E1	GY	6B	AX.89402543	4.63E+08	0.20	-2.86	3.09	0.05
E1	GY	6B	AX.89342187	6.09E+08	0.13	-3.35	3.02	0.05
E1	GY	6B	AX.89573055	1.28E+08	0.07	-5.42	4.28	0.07
E1	GY	6B	AX.89336582	1.28E+08	0.08	-4.72	3.89	0.06
E1	GY	6B	AX.89430381	2.39E+08	0.16	-3.06	3.02	0.04
E1	GY	6B	AX.89382186	4.62E+08	0.20	-2.86	3.09	0.05
E1	GY	6B	AX.89504302	4.63E+08	0.20	-2.86	3.09	0.05
E1	GY	6B	AX.89696497	1.30E+08	0.08	-4.88	3.94	0.06
E1	GY	6B	AX.89539469	6.11E+08	0.17	-3.11	3.23	0.06
E1	GY	6B	AX.89658859	6.12E+08	0.17	-2.98	3.01	0.05
E1	GY	6B	AX.89329305	6.11E+08	0.18	-3.09	3.26	0.06
E1	GY	6B	AX.89336582	1.28E+08	0.08	-4.72	3.89	0.06
E1	GY	6B	AX.89328519	4.65E+08	0.20	-2.86	3.09	0.05
E1	GY	6B	AX.89352558	1.29E+08	0.07	-5.14	4.11	0.07
E1	GY	6B	AX.89622419	1.25E+08	0.22	-2.78	3.15	0.05
E1	GY	6B	AX.89622419	1.25E+08	0.22	-2.78	3.15	0.05
E1	GY	6B	AX.89463681	4.65E+08	0.20	-2.86	3.09	0.05
E1	GY	6B	AX.89563872	1.25E+08	0.22	-2.78	3.15	0.05
E1	GY	6B	AX.89327088	4.64E+08	0.20	-2.86	3.09	0.05
E1	GY	6B	AX.89409254	2.35E+08	0.16	-3.06	3.02	0.04
E1	GY	6B	AX.89424161	1.29E+08	0.08	-5.25	4.48	0.07
E1	SPM2	6B	AX.89384275	6.78E+08	0.06	34.11	3.07	0.05
E1	GY	6B	AX.89495282	4.66E+08	0.21	-2.84	3.19	0.05
E1	GY	6B	AX.89759921	8.60E+07	0.10	-3.95	3.23	0.05
E1	GY	6B	AX.89365639	6.41E+08	0.05	-5.88	3.55	0.07
E1	GY	6B	AX.89438340	6.42E+08	0.05	-5.88	3.55	0.07
E1	GY	6B	AX.89395311	2.35E+08	0.16	-3.06	3.02	0.04
E1	GY	6B	AX.89543445	4.65E+08	0.20	-2.86	3.09	0.05
E1	SPM2	6B	AX.89567570	6.78E+08	0.07	33.60	3.17	0.06
E1	GY	6B	AX.89532953	4.63E+08	0.20	-2.86	3.09	0.05
E1	GY	6B	AX.89510891	4.62E+08	0.20	-2.86	3.09	0.05
E1	GY	6B	AX.89568337	4.67E+08	0.21	-2.84	3.19	0.05

E1	GY	6B	AX.89437168	1.29E+08	0.07	-5.14	4.11	0.07
E1	GY	6B	AX.89475763	1.27E+08	0.07	-5.42	4.28	0.07
E1	GY	6B	AX.89593764	6.12E+08	0.17	-2.98	3.01	0.05
E1	SPM2	6B	AX.89491484	6.77E+08	0.06	36.11	3.38	0.06
E1	GY	6B	AX.89535702	1.29E+08	0.07	-5.14	4.11	0.07
E1	SPM2	6B	AX.89608992	6.78E+08	0.06	34.11	3.07	0.05
E1	GY	6B	AX.89630292	6.12E+08	0.17	-2.98	3.01	0.05
E1	GY	6B	AX.89477111	2.35E+08	0.16	-3.06	3.02	0.04
E1	GY	6B	AX.89419906	1.30E+08	0.07	-5.52	4.65	0.08
E1	GY	6B	AX.89559915	6.11E+08	0.18	-3.09	3.26	0.06
E1	GY	6B	AX.89454965	6.06E+08	0.13	-3.35	3.02	0.05
E1	GY	6B	AX.89488095	1.30E+08	0.07	-5.52	4.65	0.08
E1	GY	6B	AX.89334641	4.63E+08	0.20	-2.86	3.09	0.05
E1	GY	6B	AX.89437168	1.29E+08	0.07	-5.14	4.11	0.07
E1	GY	6B	AX.89669783	1.28E+08	0.07	-4.78	3.26	0.05
E1	GY	6B	AX.89398647	4.65E+08	0.20	-2.86	3.09	0.05
E1	GY	6B	AX.89463363	1.29E+08	0.07	-5.52	4.65	0.08
E1	GY	6B	AX.89493127	1.30E+08	0.07	-5.52	4.65	0.08
E1	SPM2	6B	AX.89421537	6.77E+08	0.07	33.60	3.17	0.06
E1	GY	6B	AX.89570602	4.67E+08	0.21	-2.96	3.43	0.05
E1	GY	6B	AX.89446764	4.65E+08	0.20	-2.86	3.09	0.05
E1	SPM2	6B	AX.89535590	6.77E+08	0.07	33.60	3.17	0.06
E1	GY	6B	AX.89587336	1.29E+08	0.08	-5.25	4.48	0.07
E1	GY	6B	AX.89491312	4.63E+08	0.20	-2.86	3.09	0.05
E1	GY	6B	AX.89620029	1.29E+08	0.07	-4.55	3.33	0.05
E1	GY	6B	AX.89710819	4.66E+08	0.21	-2.84	3.19	0.05
E1	GY	6B	AX.89612588	2.35E+08	0.16	-3.06	3.02	0.04
E1	GY	6B	AX.89757356	4.27E+07	0.35	-2.70	3.82	0.06
E1	GY	6B	AX.89616707	4.66E+08	0.20	-3.12	3.54	0.05
E1	GY	6B	AX.89572587	1.27E+08	0.07	-5.14	4.11	0.07
E1	GY	6B	AX.89471542	2.35E+08	0.16	-3.06	3.02	0.04
E1	GY	6B	AX.89699001	4.63E+08	0.20	-2.86	3.09	0.05
E1	GY	6B	AX.89320931	2.27E+08	0.16	-3.06	3.02	0.04
E1	GY	6B	AX.89354586	2.35E+08	0.16	-3.06	3.02	0.04
E1	GY	6B	AX.89402543	4.63E+08	0.20	-2.86	3.09	0.05
E1	SPM2	6B	AX.89414164	6.78E+08	0.07	33.60	3.17	0.06
E1	GY	6B	AX.89735832	8.02E+07	0.09	-4.32	3.63	0.06
E1	GY	6B	AX.89707549	1.28E+08	0.07	-5.14	4.11	0.07
E1	GY	6B	AX.89602743	4.46E+08	0.18	-3.11	3.36	0.05
E1	GY	6B	AX.89602743	4.46E+08	0.18	-3.11	3.36	0.05
E1	GY	6B	AX.89449986	4.67E+08	0.21	-2.84	3.19	0.05
E1	GY	6B	AX.89415017	4.64E+08	0.20	-2.86	3.09	0.05
E1	GY	6B	AX.89426492	6.06E+08	0.13	-3.35	3.02	0.05
E1	GY	6B	AX.89438340	6.42E+08	0.05	-5.88	3.55	0.07
E1	GY	6B	AX.89626004	1.29E+08	0.07	-5.58	4.26	0.07
E1	GY	6B	AX.89435189	6.06E+08	0.13	-3.35	3.02	0.05
E1	GY	6B	AX.89310882	4.63E+08	0.20	-2.86	3.09	0.05
E1	GY	6B	AX.89333381	6.10E+08	0.17	-3.14	3.29	0.06
E1	GY	6B	AX.89521559	1.29E+08	0.07	-5.14	4.11	0.07
E1	GY	6B	AX.89323225	1.29E+08	0.08	-5.25	4.48	0.07

E1	GY	6B	AX.89323627	1.30E+08	0.07	-5.52	4.65	0.08
E1	GY	6B	AX.89745770	1.28E+08	0.07	-5.14	4.11	0.07
E1	GY	6B	AX.89592228	4.65E+08	0.20	-2.86	3.09	0.05
E1	SPM2	6B	AX.89455042	6.77E+08	0.07	33.60	3.17	0.06
E1	GY	6B	AX.89762764	1.27E+08	0.07	-5.14	4.11	0.07
E1	GY	6B	AX.89541349	7.97E+07	0.09	-4.18	3.29	0.05
E1	GY	6B	AX.89470738	2.27E+08	0.16	-3.06	3.02	0.04
E1	GY	6B	AX.89579714	1.18E+08	0.21	-2.84	3.19	0.05
E1	GY	6B	AX.89545159	4.64E+08	0.20	-2.86	3.09	0.05
E1	GY	6B	AX.89776452	6.93E+08	0.23	-2.71	3.05	0.04
E1	SPM2	6B	AX.89520056	6.78E+08	0.06	34.11	3.07	0.05
E1	GY	6B	AX.89452242	2.37E+08	0.16	-3.06	3.02	0.04
E1	GY	6B	AX.89699001	4.63E+08	0.20	-2.86	3.09	0.05
E1	GY	6B	AX.89475763	1.27E+08	0.07	-5.42	4.28	0.07
E1	SPM2	6B	AX.89706708	6.77E+08	0.07	33.60	3.17	0.06
E1	GY	6B	AX.89768419	1.28E+08	0.07	-5.42	4.28	0.07
E1	GY	6B	AX.89628919	1.27E+08	0.07	-5.42	4.28	0.07
E1	GY	6B	AX.89628919	1.27E+08	0.07	-5.42	4.28	0.07
E1	GY	6B	AX.89488073	1.26E+07	0.40	-2.71	4.01	0.07
E1	GY	6B	AX.89347329	4.64E+08	0.20	-2.86	3.09	0.05
E1	GY	6B	AX.89703732	1.29E+08	0.08	-5.25	4.48	0.07
E1	SPM2	6B	AX.89377951	6.78E+08	0.07	33.60	3.17	0.06
E1	SPM2	6B	AX.89714461	6.78E+08	0.07	33.60	3.17	0.06
E1	GY	6B	AX.89497770	4.65E+08	0.20	-2.86	3.09	0.05
E1	GY	6B	AX.89497770	4.65E+08	0.20	-2.86	3.09	0.05
E1	GY	6B	AX.89626004	1.29E+08	0.07	-5.58	4.26	0.07
E1	GY	6B	AX.89547076	1.29E+08	0.07	-5.14	4.11	0.07
E1	GY	6B	AX.89680406	1.25E+08	0.22	-2.78	3.15	0.05
E1	GY	6B	AX.89384533	2.34E+08	0.16	-3.06	3.02	0.04
E1	GY	6B	AX.89560190	1.29E+08	0.08	-4.72	3.90	0.06
E1	GY	6B	AX.89556137	4.26E+07	0.31	-2.78	3.76	0.06
E1	GY	6B	AX.89491312	4.63E+08	0.20	-2.86	3.09	0.05
E1	GY	6B	AX.89730316	1.30E+08	0.08	-5.25	4.48	0.07
E1	GY	6B	AX.89545203	1.29E+08	0.07	-5.14	4.11	0.07
E1	GY	6B	AX.89374488	2.35E+08	0.16	-3.38	3.51	0.05
E1	GY	6B	AX.89740128	6.41E+08	0.05	-5.88	3.55	0.07
E1	GY	6B	AX.89328387	1.28E+08	0.07	-5.42	4.28	0.07
E1	SPM2	6B	AX.89487015	6.77E+08	0.07	33.60	3.17	0.06
E1	GY	6B	AX.89422288	4.63E+08	0.20	-2.86	3.09	0.05
E1	GY	6B	AX.89422288	4.63E+08	0.20	-2.86	3.09	0.05
E1	GY	6B	AX.89744302	1.29E+08	0.07	-5.14	4.11	0.07
E1	GY	6B	AX.89517207	2.36E+08	0.16	-3.16	3.14	0.05
E1	GY	6B	AX.89539469	6.11E+08	0.17	-3.11	3.23	0.06
E1	GY	6B	AX.89434922	4.64E+08	0.20	-2.86	3.09	0.05
E1	GY	6B	AX.89658859	6.12E+08	0.17	-2.98	3.01	0.05
E1	GY	6B	AX.89435189	6.06E+08	0.13	-3.35	3.02	0.05
E1	GY	6B	AX.89683690	4.65E+08	0.20	-2.86	3.09	0.05
E1	GY	6B	AX.89587336	1.29E+08	0.08	-5.25	4.48	0.07
E1	GY	6B	AX.89435693	4.63E+08	0.20	-2.86	3.09	0.05
E1	GY	6B	AX.89435693	4.63E+08	0.20	-2.86	3.09	0.05

E1	GY	6B	AX.89563872	1.25E+08	0.22	-2.78	3.15	0.05
E1	GY	6B	AX.89319508	1.29E+08	0.07	-5.21	4.21	0.07
E1	GY	6B	AX.89518322	1.29E+08	0.08	-4.96	4.06	0.06
E1	GY	6B	AX.89724500	4.65E+08	0.20	-2.86	3.09	0.05
E1	GY	6B	AX.89409254	2.35E+08	0.16	-3.06	3.02	0.04
E1	GY	6B	AX.89518587	1.30E+08	0.07	-5.52	4.65	0.08
E1	GY	6B	AX.89560383	4.26E+07	0.35	-2.53	3.40	0.05
E1	GY	6B	AX.89698227	4.64E+08	0.20	-2.86	3.09	0.05
E1	GY	6B	AX.89413801	4.66E+08	0.20	-2.86	3.09	0.05
E1	GY	6B	AX.89735832	8.02E+07	0.09	-4.32	3.63	0.06
E1	GY	6B	AX.89747766	6.10E+08	0.16	-3.24	3.35	0.06
E1	GY	6B	AX.89402631	1.28E+08	0.07	-5.42	4.28	0.07
E1	GY	6B	AX.89520699	2.33E+08	0.16	-3.06	3.02	0.04
E1	GY	6B	AX.89520699	2.33E+08	0.16	-3.06	3.02	0.04
E1	GY	6B	AX.89589396	1.27E+08	0.07	-5.14	4.11	0.07
E1	GY	6B	AX.89555450	2.27E+08	0.16	-3.06	3.02	0.04
E1	GY	6B	AX.89592228	4.65E+08	0.20	-2.86	3.09	0.05
E1	GY	6B	AX.89556137	4.26E+07	0.31	-2.78	3.76	0.06
E1	GY	6B	AX.89541349	7.97E+07	0.09	-4.18	3.29	0.05
E1	SPM2	6B	AX.89333474	6.78E+08	0.07	33.60	3.17	0.06
E1	GY	6B	AX.89612774	1.29E+08	0.08	-5.25	4.48	0.07
E1	GY	6B	AX.89545159	4.64E+08	0.20	-2.86	3.09	0.05
E1	GY	6B	AX.89370211	4.28E+07	0.36	-2.64	3.70	0.05
E1	GY	6B	AX.89393465	4.63E+08	0.20	-2.86	3.09	0.05
E1	GY	6B	AX.89393465	4.63E+08	0.20	-2.86	3.09	0.05
E1	GY	6B	AX.89417457	4.66E+08	0.21	-2.92	3.29	0.05
E1	GY	6B	AX.89767544	4.26E+07	0.36	-2.52	3.39	0.05
E1	GY	6B	AX.89452242	2.37E+08	0.16	-3.06	3.02	0.04
E1	GY	6B	AX.89325291	1.28E+08	0.07	-5.67	4.38	0.07
E1	GY	6B	AX.89347083	4.27E+07	0.31	-2.74	3.71	0.05
E1	GY	6B	AX.89347083	4.27E+07	0.31	-2.74	3.71	0.05
E1	GY	6B	AX.89359724	6.05E+08	0.13	-3.35	3.02	0.05
E1	GY	6B	AX.89359724	6.05E+08	0.13	-3.35	3.02	0.05
E1	GY	6B	AX.89765140	4.65E+08	0.20	-2.86	3.09	0.05
E1	GY	6B	AX.89395311	2.35E+08	0.16	-3.06	3.02	0.04
E1	GY	6B	AX.89419965	6.42E+08	0.05	-5.88	3.55	0.07
E1	GY	6B	AX.89717947	4.65E+08	0.20	-2.86	3.09	0.05
E1	GY	6B	AX.89571598	6.10E+08	0.18	-3.09	3.26	0.06
E1	GY	6B	AX.89571598	6.10E+08	0.18	-3.09	3.26	0.06
E1	GY	6B	AX.89510891	4.62E+08	0.20	-2.86	3.09	0.05
E1	GY	6B	AX.89608575	1.29E+08	0.07	-5.52	4.65	0.08
E1	GY	6B	AX.89753643	4.65E+08	0.20	-2.86	3.09	0.05
E1	GY	6B	AX.89409984	4.63E+08	0.20	-2.86	3.06	0.04
E1	GY	6B	AX.89409984	4.63E+08	0.20	-2.86	3.06	0.04
E1	GY	6B	AX.89669478	2.35E+08	0.16	-3.06	3.02	0.04
E1	GY	6B	AX.89669478	2.35E+08	0.16	-3.06	3.02	0.04
E1	GY	6B	AX.89398647	4.65E+08	0.20	-2.86	3.09	0.05
E1	GY	6B	AX.89328387	1.28E+08	0.07	-5.42	4.28	0.07
E1	GY	6B	AX.89707549	1.28E+08	0.07	-5.14	4.11	0.07
E1	GY	6B	AX.89645517	2.27E+08	0.16	-3.06	3.02	0.04

E1	GY	6B	AX.89696497	1.30E+08	0.08	-4.88	3.94	0.06
E1	GY	6B	AX.89570602	4.67E+08	0.21	-2.96	3.43	0.05
E1	SPM2	6B	AX.89406711	6.77E+08	0.07	33.60	3.17	0.06
E1	GY	6B	AX.89708689	2.37E+08	0.16	-3.06	3.02	0.04
E1	GY	6B	AX.89708689	2.37E+08	0.16	-3.06	3.02	0.04
E1	GY	6B	AX.89540091	1.29E+08	0.07	-5.52	4.65	0.08
E1	GY	6B	AX.89635486	4.65E+08	0.20	-2.86	3.09	0.05
E1	GY	6B	AX.89541016	4.65E+08	0.20	-2.86	3.09	0.05
E1	GY	6B	AX.89424161	1.29E+08	0.08	-5.25	4.48	0.07
E1	GY	6B	AX.89575719	1.29E+08	0.07	-5.14	4.11	0.07
E1	GY	6B	AX.89530519	6.11E+08	0.18	-3.09	3.26	0.06
E1	GY	6B	AX.89552916	4.65E+08	0.20	-2.86	3.09	0.05
E1	GY	6B	AX.89552916	4.65E+08	0.20	-2.86	3.09	0.05
E1	SPM2	6B	AX.89390819	6.78E+08	0.06	36.11	3.38	0.06
E1	GY	6B	AX.89495282	4.66E+08	0.21	-2.84	3.19	0.05
E1	GY	6B	AX.89402250	2.27E+08	0.16	-3.06	3.02	0.04
E1	GY	6B	AX.89402250	2.27E+08	0.16	-3.06	3.02	0.04
E1	GY	6B	AX.89747277	1.25E+08	0.22	-2.80	3.17	0.05
E1	GY	6B	AX.89759921	8.60E+07	0.10	-3.95	3.23	0.05
E1	GY	6B	AX.89332261	6.06E+08	0.13	-3.35	3.02	0.05
E1	GY	6B	AX.89332352	4.65E+08	0.20	-2.86	3.09	0.05
E1	GY	6B	AX.89688061	4.65E+08	0.20	-2.86	3.09	0.05
E1	GY	6B	AX.89688061	4.65E+08	0.20	-2.86	3.09	0.05
E1	GY	6B	AX.89652368	4.28E+07	0.31	-2.78	3.76	0.06
E1	GY	6B	AX.89544312	1.29E+08	0.07	-5.52	4.65	0.08
E1	GY	6B	AX.89762764	1.27E+08	0.07	-5.14	4.11	0.07
E1	GY	6B	AX.89776452	6.93E+08	0.23	-2.71	3.05	0.04
E1	GY	6B	AX.89430017	1.25E+08	0.22	-2.77	3.10	0.05
E1	GY	6B	AX.89488073	1.26E+07	0.40	-2.71	4.01	0.07
E1	GY	6B	AX.89547076	1.29E+08	0.07	-5.14	4.11	0.07
E1	GY	6B	AX.89326589	1.30E+08	0.08	-5.25	4.48	0.07
E1	GY	6B	AX.89582883	4.66E+08	0.21	-2.84	3.19	0.05
E1	GY	6B	AX.89757356	4.27E+07	0.35	-2.70	3.82	0.06
E1	GY	6B	AX.89665712	4.26E+07	0.35	-2.53	3.40	0.05
E1	GY	6B	AX.89694977	2.35E+08	0.16	-3.06	3.02	0.04
E1	GY	6B	AX.89669783	1.28E+08	0.07	-4.78	3.26	0.05
E1	GY	6B	AX.89597949	1.29E+08	0.07	-5.52	4.65	0.08
E1	GY	6B	AX.89633298	4.27E+07	0.36	-2.46	3.26	0.05
E1	GY	6B	AX.89504302	4.63E+08	0.20	-2.86	3.09	0.05
E1	GY	6B	AX.89709192	1.28E+08	0.06	-5.09	3.42	0.05
E1	GY	6B	AX.89678904	1.29E+08	0.08	-5.25	4.48	0.07
E1	GY	6B	AX.89517207	2.36E+08	0.16	-3.16	3.14	0.05
E1	GY	6B	AX.89646939	1.28E+08	0.07	-5.42	4.28	0.07
E1	GY	6B	AX.89469421	4.63E+08	0.20	-2.85	3.03	0.04
E1	GY	6B	AX.89469421	4.63E+08	0.20	-2.85	3.03	0.04
E1	GY	6B	AX.89518322	1.29E+08	0.08	-4.96	4.06	0.06
E1	GY	6B	AX.89724500	4.65E+08	0.20	-2.86	3.09	0.05
E1	GY	6B	AX.89506640	2.39E+08	0.16	-3.06	3.02	0.04
E1	GY	6B	AX.89506640	2.39E+08	0.16	-3.06	3.02	0.04
E1	GY	6B	AX.89710819	4.66E+08	0.21	-2.84	3.19	0.05

E1	GY	6B	AX.89475640	1.28E+08	0.07	-5.14	4.11	0.07
E1	GY	6B	AX.89475640	1.28E+08	0.07	-5.14	4.11	0.07
E1	GY	6B	AX.89613195	2.34E+08	0.16	-3.06	3.02	0.04
E1	GY	6B	AX.89508204	4.63E+08	0.20	-2.86	3.09	0.05
E1	GY	6B	AX.89310882	4.63E+08	0.20	-2.86	3.09	0.05
E1	GY	6B	AX.89521559	1.29E+08	0.07	-5.14	4.11	0.07
E1	GY	6B	AX.89765957	1.25E+08	0.22	-2.78	3.15	0.05
E1	GY	6B	AX.89765957	1.25E+08	0.22	-2.78	3.15	0.05
E1	GY	6B	AX.89555450	2.27E+08	0.16	-3.06	3.02	0.04
E1	SPM2	6B	AX.89605371	6.77E+08	0.06	36.11	3.38	0.06
E1	GY	6B	AX.89521985	4.66E+08	0.21	-2.84	3.19	0.05
E1	GY	6B	AX.89697805	1.30E+08	0.08	-5.25	4.48	0.07
E1	GY	6B	AX.89697805	1.30E+08	0.08	-5.25	4.48	0.07
E1	GY	6B	AX.89345848	1.29E+08	0.07	-5.14	4.11	0.07
E1	GY	6B	AX.89763125	4.66E+08	0.20	-2.86	3.09	0.05
E1	GY	6B	AX.89488095	1.30E+08	0.07	-5.52	4.65	0.08
E1	GY	6B	AX.89541623	2.38E+08	0.16	-3.06	3.02	0.04
E1	GY	6B	AX.89334641	4.63E+08	0.20	-2.86	3.09	0.05
E1	GY	6B	AX.89495986	4.66E+08	0.21	-2.84	3.19	0.05
E1	GY	6B	AX.89680666	4.65E+08	0.20	-2.86	3.09	0.05
E1	GY	6B	AX.89546236	1.29E+08	0.07	-5.14	4.11	0.07
E1	GY	6B	AX.89629774	4.63E+08	0.20	-2.88	3.09	0.05
E1	GY	6B	AX.89629774	4.63E+08	0.20	-2.88	3.09	0.05
E1	GY	6B	AX.89765140	4.65E+08	0.20	-2.86	3.09	0.05
E1	GY	6B	AX.89476655	1.29E+08	0.07	-5.14	4.11	0.07
E1	GY	6B	AX.89476655	1.29E+08	0.07	-5.14	4.11	0.07
E1	GY	6B	AX.89709910	4.66E+08	0.21	-2.84	3.19	0.05
E1	GY	6B	AX.89630292	6.12E+08	0.17	-2.98	3.01	0.05
E1	GY	6B	AX.89419906	1.30E+08	0.07	-5.52	4.65	0.08
E1	GY	6B	AX.89478059	4.67E+08	0.21	-2.84	3.19	0.05
E1	GY	6B	AX.89559915	6.11E+08	0.18	-3.09	3.26	0.06
E1	GY	6B	AX.89717947	4.65E+08	0.20	-2.86	3.09	0.05
E1	GY	6B	AX.89560190	1.29E+08	0.08	-4.72	3.90	0.06
E1	GY	6B	AX.89560249	4.27E+07	0.36	-2.46	3.26	0.05
E1	GY	6B	AX.89730316	1.30E+08	0.08	-5.25	4.48	0.07
E1	GY	6B	AX.89709076	2.27E+08	0.16	-3.06	3.02	0.04
E1	GY	6B	AX.89709076	2.27E+08	0.16	-3.06	3.02	0.04
E1	GY	6B	AX.89665712	4.26E+07	0.35	-2.53	3.40	0.05
E1	SPM2	6B	AX.89572067	6.77E+08	0.06	34.11	3.07	0.05
E1	GY	6B	AX.89597949	1.29E+08	0.07	-5.52	4.65	0.08
E1	GY	6B	AX.89678904	1.29E+08	0.08	-5.25	4.48	0.07
E1	GY	6B	AX.89583493	4.65E+08	0.20	-2.86	3.09	0.05
E1	GY	6B	AX.89644949	2.35E+08	0.16	-3.06	3.02	0.04
E1	GY	6B	AX.89708341	1.28E+08	0.07	-5.42	4.28	0.07
E1	GY	6B	AX.89352327	1.27E+08	0.07	-5.42	4.28	0.07
E1	GY	6B	AX.89497224	4.63E+08	0.20	-2.86	3.09	0.05
E1	GY	6B	AX.89646939	1.28E+08	0.07	-5.42	4.28	0.07
E1	GY	6B	AX.89647084	6.42E+08	0.05	-5.88	3.55	0.07
E1	GY	6B	AX.89647084	6.42E+08	0.05	-5.88	3.55	0.07
E1	GY	6B	AX.89670568	4.66E+08	0.21	-2.84	3.19	0.05

E1	GY	6B	AX.89670568	4.66E+08	0.21	-2.84	3.19	0.05
E1	GY	6B	AX.89460102	4.63E+08	0.20	-2.86	3.09	0.05
E1	GY	6B	AX.89460102	4.63E+08	0.20	-2.86	3.09	0.05
E1	GY	6B	AX.89518587	1.30E+08	0.07	-5.52	4.65	0.08
E1	GY	6B	AX.89470738	2.27E+08	0.16	-3.06	3.02	0.04
E1	GY	6B	AX.89698227	4.64E+08	0.20	-2.86	3.09	0.05
E1	GY	6B	AX.89744520	4.63E+08	0.20	-2.86	3.09	0.05
E1	GY	6B	AX.89744520	4.63E+08	0.20	-2.86	3.09	0.05
E1	GY	6B	AX.89543948	1.27E+08	0.07	-5.14	4.11	0.07
E1	GY	6B	AX.89543948	1.27E+08	0.07	-5.14	4.11	0.07
E1	GY	6B	AX.89647703	1.29E+08	0.07	-5.52	4.65	0.08
E1	GY	6B	AX.89647703	1.29E+08	0.07	-5.52	4.65	0.08
E1	GY	6B	AX.89320931	2.27E+08	0.16	-3.06	3.02	0.04
E1	GY	6B	AX.89747277	1.25E+08	0.22	-2.80	3.17	0.05
E1	GY	6B	AX.89354586	2.35E+08	0.16	-3.06	3.02	0.04
E1	GY	6B	AX.89747766	6.10E+08	0.16	-3.24	3.35	0.06
E1	GY	6B	AX.89505563	4.66E+08	0.19	-3.10	3.44	0.05
E1	GY	6B	AX.89508341	2.38E+08	0.16	-3.06	3.02	0.04
E1	GY	6B	AX.89508341	2.38E+08	0.16	-3.06	3.02	0.04
E1	GY	6B	AX.89759438	4.27E+07	0.36	-2.73	3.91	0.06
E1	GY	6B	AX.89684340	4.63E+08	0.20	-2.86	3.09	0.05
E1	GY	6B	AX.89631175	1.28E+08	0.07	-5.14	4.11	0.07
E1	GY	6B	AX.89652368	4.28E+07	0.31	-2.78	3.76	0.06
E1	GY	6B	AX.89357544	4.66E+08	0.20	-2.86	3.09	0.05
E1	GY	6B	AX.89612774	1.29E+08	0.08	-5.25	4.48	0.07
E1	GY	6B	AX.89462797	1.29E+08	0.08	-5.25	4.48	0.07
E1	GY	6B	AX.89767544	4.26E+07	0.36	-2.52	3.39	0.05
E1	GY	6B	AX.89463363	1.29E+08	0.07	-5.52	4.65	0.08
E1	GY	6B	AX.89535702	1.29E+08	0.07	-5.14	4.11	0.07
E1	GY	6B	AX.89326589	1.30E+08	0.08	-5.25	4.48	0.07
E1	GY	6B	AX.89419965	6.42E+08	0.05	-5.88	3.55	0.07
E1	GY	6B	AX.89680406	1.25E+08	0.22	-2.78	3.15	0.05
E1	GY	6B	AX.89608575	1.29E+08	0.07	-5.52	4.65	0.08
E1	GY	6B	AX.89753643	4.65E+08	0.20	-2.86	3.09	0.05
E1	GY	6B	AX.89722924	4.26E+07	0.31	-2.78	3.76	0.06
E1	GY	6B	AX.89534036	4.65E+08	0.20	-2.86	3.09	0.05
E1	GY	6B	AX.89534036	4.65E+08	0.20	-2.86	3.09	0.05
E1	GY	6B	AX.89620456	6.64E+08	0.07	-4.56	3.18	0.05
E1	GY	6B	AX.89545203	1.29E+08	0.07	-5.14	4.11	0.07
E1	GY	6B	AX.89573055	1.28E+08	0.07	-5.42	4.28	0.07
E1	GY	6B	AX.89385970	1.27E+08	0.06	-5.01	3.13	0.05
E1	GY	6B	AX.89493127	1.30E+08	0.07	-5.52	4.65	0.08
E1	GY	6B	AX.89434922	4.64E+08	0.20	-2.86	3.09	0.05
E1	GY	6B	AX.89540091	1.29E+08	0.07	-5.52	4.65	0.08
E1	GY	6B	AX.89352558	1.29E+08	0.07	-5.14	4.11	0.07
E1	GY	6B	AX.89635486	4.65E+08	0.20	-2.86	3.09	0.05
E1	GPM2	6B	AX.89756296	1.30E+08	0.07	-1291.54	3.98	0.07
E1	GY	6B	AX.89541016	4.65E+08	0.20	-2.86	3.09	0.05
E1	GPM2	6B	AX.89424161	1.29E+08	0.08	-1224.86	3.81	0.06
E1	GPM2	6B	AX.89402631	1.28E+08	0.07	-1365.71	4.17	0.07

E1	GPM2	6B	AX.89608831	1.20E+07	0.20	824.14	3.85	0.07
E1	GY	6B	AX.89365639	6.41E+08	0.05	-5.88	3.55	0.07
E1	GPM2	6B	AX.89704684	1.25E+08	0.15	-882.07	3.49	0.06
E1	SPM2	6B	AX.89441080	6.78E+08	0.07	32.01	3.08	0.05
E1	GPM2	6B	AX.89696497	1.30E+08	0.08	-1130.67	3.32	0.05
E1	GPM2	6B	AX.89525135	3.57E+06	0.27	863.77	4.94	0.09
E1	GPM2	6B	AX.89634365	1.30E+08	0.30	631.46	3.02	0.05
E1	GPM2	6B	AX.89428831	3.57E+06	0.13	881.05	3.08	0.05
E1	GY	6B	AX.89759438	4.27E+07	0.36	-2.73	3.91	0.06
E1	GY	6B	AX.89607302	1.30E+08	0.07	-5.52	4.65	0.08
E1	GY	6B	AX.89607302	1.30E+08	0.07	-5.52	4.65	0.08
E1	GPM2	6B	AX.89518322	1.29E+08	0.08	-1086.41	3.10	0.05
E1	GY	6B	AX.89323225	1.29E+08	0.08	-5.25	4.48	0.07
E1	GPM2	6B	AX.89385970	1.27E+08	0.06	-1374.75	3.52	0.06
E1	GPM2	6B	AX.89703604	1.70E+05	0.29	739.56	3.90	0.07
E1	GY	6B	AX.89544312	1.29E+08	0.07	-5.52	4.65	0.08
E1	GY	6B	AX.89579714	1.18E+08	0.21	-2.84	3.19	0.05
E1	GPM2	6B	AX.89400295	1.25E+08	0.14	-879.32	3.39	0.05
E1	GPM2	6B	AX.89493997	1.30E+08	0.30	631.46	3.02	0.05
E1	GY	6B	AX.89430017	1.25E+08	0.22	-2.77	3.10	0.05
E1	GPM2	6B	AX.89501645	1.29E+08	0.30	631.46	3.02	0.05
E1	GPM2	6B	AX.89561439	7.00E+05	0.19	810.77	3.57	0.06
E1	GPM2	6B	AX.89572587	1.27E+08	0.07	-1290.12	3.97	0.07
E1	GY	6B	AX.89703732	1.29E+08	0.08	-5.25	4.48	0.07
E1	GY	6B	AX.89716778	1.27E+08	0.07	-5.14	4.11	0.07
E1	GY	6B	AX.89716778	1.27E+08	0.07	-5.14	4.11	0.07
E1	GY	6B	AX.89765820	4.63E+08	0.20	-2.85	3.03	0.04
E1	GPM2	6B	AX.89535702	1.29E+08	0.07	-1290.12	3.97	0.07
E1	GY	6B	AX.89582883	4.66E+08	0.21	-2.84	3.19	0.05
E1	GPM2	6B	AX.89762764	1.27E+08	0.07	-1290.12	3.97	0.07
E1	GPM2	6B	AX.89703732	1.29E+08	0.08	-1224.86	3.81	0.06
E1	GPM2	6B	AX.89485244	1.16E+07	0.12	971.92	3.44	0.06
E1	GY	6B	AX.89722924	4.26E+07	0.31	-2.78	3.76	0.06
E1	GPM2	6B	AX.89445880	6.81E+08	0.34	610.64	3.03	0.05
E1	GPM2	6B	AX.89352327	1.27E+08	0.07	-1365.71	4.17	0.07
E1	GY	6B	AX.89694977	2.35E+08	0.16	-3.06	3.02	0.04
E1	GPM2	6B	AX.89518587	1.30E+08	0.07	-1291.54	3.98	0.07
E1	GY	6B	AX.89768419	1.28E+08	0.07	-5.42	4.28	0.07
E1	GY	6B	AX.89633298	4.27E+07	0.36	-2.46	3.26	0.05
E1	GPM2	6B	AX.89345848	1.29E+08	0.07	-1290.12	3.97	0.07
E1	GPM2	6B	AX.89540091	1.29E+08	0.07	-1291.54	3.98	0.07
E1	GPM2	6B	AX.89518214	1.66E+05	0.28	791.39	4.35	0.08
E1	GY	6B	AX.89729238	4.65E+08	0.20	-2.86	3.09	0.05
E1	GY	6B	AX.89729238	4.65E+08	0.20	-2.86	3.09	0.05
E1	GPM2	6B	AX.89545203	1.29E+08	0.07	-1290.12	3.97	0.07
E1	GPM2	6B	AX.89475763	1.27E+08	0.07	-1365.71	4.17	0.07
E1	GPM2	6B	AX.89546236	1.29E+08	0.07	-1290.12	3.97	0.07
E1	GY	6B	AX.89612588	2.35E+08	0.16	-3.06	3.02	0.04
E1	GPM2	6B	AX.89773654	3.57E+06	0.28	859.72	5.04	0.09
E1	GPM2	6B	AX.89716778	1.27E+08	0.07	-1290.12	3.97	0.07

E1	GPM2	6B	AX.89517996	1.29E+08	0.07	-1290.12	3.97	0.07
E1	GPM2	6B	AX.89541349	7.97E+07	0.09	-1062.96	3.26	0.05
E1	GPM2	6B	AX.89488095	1.30E+08	0.07	-1291.54	3.98	0.07
E1	GPM2	6B	AX.89615713	1.25E+08	0.14	-879.32	3.39	0.05
E1	GPM2	6B	AX.89388402	1.25E+08	0.22	-742.58	3.40	0.05
E1	GPM2	6B	AX.89708341	1.28E+08	0.07	-1365.71	4.17	0.07
E1	GY	6B	AX.89426492	6.06E+08	0.13	-3.35	3.02	0.05
E1	GPM2	6B	AX.89463363	1.29E+08	0.07	-1291.54	3.98	0.07
E1	GPM2	6B	AX.89768419	1.28E+08	0.07	-1365.71	4.17	0.07
E1	GPM2	6B	AX.89464476	1.25E+08	0.14	-879.32	3.39	0.05
E1	GPM2	6B	AX.89435297	6.81E+08	0.34	610.64	3.03	0.05
E1	GPM2	6B	AX.89443367	1.30E+08	0.08	-1224.86	3.81	0.06
E1	GY	6B	AX.89741367	4.66E+08	0.20	-2.86	3.09	0.05
E1	GY	6B	AX.89741367	4.66E+08	0.20	-2.86	3.09	0.05
E1	GY	6B	AX.89521985	4.66E+08	0.21	-2.84	3.19	0.05
E1	GPM2	6B	AX.89437168	1.29E+08	0.07	-1290.12	3.97	0.07
E1	GY	6B	AX.89345848	1.29E+08	0.07	-5.14	4.11	0.07
E1	GY	6B	AX.89312342	1.25E+08	0.22	-2.78	3.15	0.05
E1	GY	6B	AX.89541623	2.38E+08	0.16	-3.06	3.02	0.04
E1	GY	6B	AX.89370211	4.28E+07	0.36	-2.64	3.70	0.05
E1	GPM2	6B	AX.89417745	1.25E+08	0.14	-879.32	3.39	0.05
E1	GPM2	6B	AX.89618571	1.30E+08	0.30	631.46	3.02	0.05
E1	GY	6B	AX.89617086	1.27E+08	0.07	-5.14	4.11	0.07
E1	GY	6B	AX.89617086	1.27E+08	0.07	-5.14	4.11	0.07
E1	GY	6B	AX.89495986	4.66E+08	0.21	-2.84	3.19	0.05
E1	GY	6B	AX.89680666	4.65E+08	0.20	-2.86	3.09	0.05
E1	GPM2	6B	AX.89392775	4.17E+06	0.20	784.13	3.53	0.06
E1	GY	6B	AX.89756296	1.30E+08	0.07	-5.52	4.65	0.08
E1	GY	6B	AX.89756296	1.30E+08	0.07	-5.52	4.65	0.08
E1	GPM2	6B	AX.89628266	6.22E+07	0.08	-1150.32	3.42	0.05
E1	GPM2	6B	AX.89417457	4.66E+08	0.21	-713.98	3.05	0.05
E1	GPM2	6B	AX.89573055	1.28E+08	0.07	-1365.71	4.17	0.07
E1	GPM2	6B	AX.89331939	1.25E+07	0.20	778.56	3.44	0.06
E1	GPM2	6B	AX.89323225	1.29E+08	0.08	-1224.86	3.81	0.06
E1	GY	6B	AX.89709910	4.66E+08	0.21	-2.84	3.19	0.05
E1	GPM2	6B	AX.89678904	1.29E+08	0.08	-1224.86	3.81	0.06
E1	GPM2	6B	AX.89553125	1.30E+08	0.30	631.46	3.02	0.05
E1	GPS	6B	AX.89402646	4.89E+06	0.13	1.66	3.13	0.05
E1	GPM2	6B	AX.89521559	1.29E+08	0.07	-1290.12	3.97	0.07
E1	GY	6B	AX.89443367	1.30E+08	0.08	-5.25	4.48	0.07
E1	GY	6B	AX.89443367	1.30E+08	0.08	-5.25	4.48	0.07
E1	GPM2	6B	AX.89608575	1.29E+08	0.07	-1291.54	3.98	0.07
E1	GPM2	6B	AX.89621317	1.97E+05	0.20	782.46	3.52	0.06
E1	GPM2	6B	AX.89563387	6.87E+08	0.24	-717.47	3.32	0.06
E1	GPM2	6B	AX.89747277	1.25E+08	0.22	-752.52	3.43	0.05
E1	GPM2	6B	AX.89697805	1.30E+08	0.08	-1224.86	3.81	0.06
E1	GY	6B	AX.89613744	6.42E+08	0.05	-5.88	3.55	0.07
E1	GY	6B	AX.89572587	1.27E+08	0.07	-5.14	4.11	0.07
E1	GPM2	6B	AX.89713760	3.57E+06	0.29	811.61	4.63	0.08
E1	GPM2	6B	AX.89309973	1.69E+05	0.29	739.56	3.90	0.07

E1	GY	6B	AX.89740128	6.41E+08	0.05	-5.88	3.55	0.07
E1	GPM2	6B	AX.89526051	2.45E+05	0.27	839.88	4.70	0.09
E1	GY	6B	AX.89385970	1.27E+08	0.06	-5.01	3.13	0.05
E1	GPM2	6B	AX.89385433	1.29E+08	0.07	-1290.12	3.97	0.07
E1	GPM2	6B	AX.89409984	4.63E+08	0.20	-761.27	3.26	0.05
E1	GPM2	6B	AX.89505918	3.48E+08	0.06	-1291.18	3.16	0.05
E1	GY	6B	AX.89744302	1.29E+08	0.07	-5.14	4.11	0.07
E1	GPM2	6B	AX.89730316	1.30E+08	0.08	-1224.86	3.81	0.06
E1	GY	6B	AX.89644949	2.35E+08	0.16	-3.06	3.02	0.04
E1	GY	6B	AX.89708341	1.28E+08	0.07	-5.42	4.28	0.07
E1	GY	6B	AX.89352327	1.27E+08	0.07	-5.42	4.28	0.07
E1	GY	6B	AX.89683690	4.65E+08	0.20	-2.86	3.09	0.05
E1	GPM2	6B	AX.89589396	1.27E+08	0.07	-1290.12	3.97	0.07
E1	GPM2	6B	AX.89770202	3.57E+06	0.29	831.26	4.79	0.09
E1	GPM2	6B	AX.89547076	1.29E+08	0.07	-1290.12	3.97	0.07
E1	GPM2	6B	AX.89768266	2.51E+05	0.29	765.15	4.17	0.08
E1	GPM2	6B	AX.89580063	3.57E+06	0.19	773.78	3.30	0.06
E1	GPM2	6B	AX.89773606	1.29E+08	0.30	631.46	3.02	0.05
E1	GPM2	6B	AX.89765957	1.25E+08	0.22	-742.58	3.40	0.05
E1	GPM2	6B	AX.89646939	1.28E+08	0.07	-1365.71	4.17	0.07
E1	GPM2	6B	AX.89572428	1.24E+07	0.20	778.56	3.44	0.06
E1	GPM2	6B	AX.89552080	4.16E+06	0.20	784.13	3.53	0.06
E1	GY	6B	AX.89683318	6.10E+08	0.18	-3.09	3.26	0.06
E1	GY	6B	AX.89683318	6.10E+08	0.18	-3.09	3.26	0.06
E1	GY	6B	AX.89577920	4.65E+08	0.20	-2.86	3.09	0.05
E1	GPM2	6B	AX.89669783	1.28E+08	0.07	-1179.62	3.06	0.05
E1	GPM2	6B	AX.89409455	6.81E+08	0.34	610.64	3.03	0.05
E1	GPM2	6B	AX.89675809	1.25E+08	0.15	-882.07	3.49	0.06
E1	GY	6B	AX.89505563	4.66E+08	0.19	-3.10	3.44	0.05
E1	GPM2	6B	AX.89589691	4.71E+08	0.14	-944.40	3.75	0.06
E1	GY	6B	AX.89589396	1.27E+08	0.07	-5.14	4.11	0.07
E1	GY	6B	AX.89684340	4.63E+08	0.20	-2.86	3.09	0.05
E1	GY	6B	AX.89631175	1.28E+08	0.07	-5.14	4.11	0.07
E1	GY	6B	AX.89774402	2.36E+08	0.16	-3.06	3.02	0.04
E1	GY	6B	AX.89616707	4.66E+08	0.20	-3.12	3.54	0.05
E1	GY	6B	AX.89613744	6.42E+08	0.05	-5.88	3.55	0.07
E1	GY	6B	AX.89681269	2.38E+08	0.16	-3.06	3.02	0.04
E1	GY	6B	AX.89385433	1.29E+08	0.07	-5.14	4.11	0.07
E1	GY	6B	AX.89385433	1.29E+08	0.07	-5.14	4.11	0.07
E1	GY	6B	AX.89575719	1.29E+08	0.07	-5.14	4.11	0.07
E1	GPM2	6B	AX.89607302	1.30E+08	0.07	-1291.54	3.98	0.07
E1	GPM2	6B	AX.89612774	1.29E+08	0.08	-1224.86	3.81	0.06
E1	GPM2	6B	AX.89563872	1.25E+08	0.22	-742.58	3.40	0.05
E1	GPM2	6B	AX.89441447	1.69E+05	0.29	739.56	3.90	0.07
E1	GY	6B	AX.89543445	4.65E+08	0.20	-2.86	3.09	0.05
E1	GPM2	6B	AX.89543948	1.27E+08	0.07	-1290.12	3.97	0.07
E1	GPM2	6B	AX.89369961	1.29E+08	0.30	631.46	3.02	0.05
E1	GPM2	6B	AX.89436988	3.57E+06	0.29	831.26	4.79	0.09
E1	GPM2	6B	AX.89544388	1.66E+05	0.28	777.59	4.18	0.08
E1	GPM2	6B	AX.89446890	8.73E+07	0.09	-1129.96	3.62	0.06

E1	GPM2	6B	AX.89660252	1.29E+08	0.07	-1290.12	3.97	0.07
E1	GPM2	6B	AX.89462431	1.25E+08	0.15	-882.07	3.49	0.06
E1	GPM2	6B	AX.89519130	7.16E+08	0.40	614.84	3.26	0.06
E1	GPM2	6B	AX.89597949	1.29E+08	0.07	-1291.54	3.98	0.07
E1	GPM2	6B	AX.89433965	1.30E+08	0.30	631.46	3.02	0.05
E1	GY	6B	AX.89765820	4.63E+08	0.20	-2.85	3.03	0.04
E1	GPM2	6B	AX.89411984	6.87E+08	0.24	-717.47	3.32	0.06
E1	GPM2	6B	AX.89326589	1.30E+08	0.08	-1224.86	3.81	0.06
E1	GPM2	6B	AX.89536390	1.29E+08	0.30	631.46	3.02	0.05
E1	GPM2	6B	AX.89628919	1.27E+08	0.07	-1365.71	4.17	0.07
E1	GPM2	6B	AX.89587336	1.29E+08	0.08	-1224.86	3.81	0.06
E1	GPM2	6B	AX.89617086	1.27E+08	0.07	-1290.12	3.97	0.07
E1	GY	6B	AX.89620456	6.64E+08	0.07	-4.56	3.18	0.05
E1	GY	6B	AX.89374488	2.35E+08	0.16	-3.38	3.51	0.05
E1	GPM2	6B	AX.89434614	1.29E+08	0.30	631.46	3.02	0.05
E1	GPM2	6B	AX.89620456	6.64E+08	0.07	-1229.28	3.47	0.06
E1	GPM2	6B	AX.89597386	1.25E+08	0.14	-861.75	3.20	0.05
E1	GPM2	6B	AX.89622419	1.25E+08	0.22	-742.58	3.40	0.05
E1	GPM2	6B	AX.89735832	8.02E+07	0.09	-1081.70	3.49	0.06
E1	GPM2	6B	AX.89312342	1.25E+08	0.22	-742.58	3.40	0.05
E1	GPM2	6B	AX.89475640	1.28E+08	0.07	-1290.12	3.97	0.07
E1	GPM2	6B	AX.89493127	1.30E+08	0.07	-1291.54	3.98	0.07
E1	GY	6B	AX.89471542	2.35E+08	0.16	-3.06	3.02	0.04
E1	GY	6B	AX.89508204	4.63E+08	0.20	-2.86	3.09	0.05
E1	GPM2	6B	AX.89575719	1.29E+08	0.07	-1290.12	3.97	0.07
E1	GPM2	6B	AX.89525639	6.22E+06	0.35	625.28	3.21	0.06
E1	GPM2	6B	AX.89744302	1.29E+08	0.07	-1290.12	3.97	0.07
E1	GPM2	6B	AX.89419906	1.30E+08	0.07	-1291.54	3.98	0.07
E1	GPM2	6B	AX.89476655	1.29E+08	0.07	-1290.12	3.97	0.07
E1	GPM2	6B	AX.89421263	1.28E+08	0.07	-1365.71	4.17	0.07
E1	GPM2	6B	AX.89681545	4.69E+08	0.14	-848.70	3.19	0.05
E1	GPM2	6B	AX.89626004	1.29E+08	0.07	-1481.51	4.54	0.08
E1	GPM2	6B	AX.89325291	1.28E+08	0.07	-1333.87	3.78	0.06
E1	GPM2	6B	AX.89352558	1.29E+08	0.07	-1290.12	3.97	0.07
E1	GPM2	6B	AX.89323627	1.30E+08	0.07	-1291.54	3.98	0.07
E1	GY	6B	AX.89546236	1.29E+08	0.07	-5.14	4.11	0.07
E1	GPM2	6B	AX.89319508	1.29E+08	0.07	-1145.18	3.24	0.05
E1	GPM2	6B	AX.89338973	1.14E+05	0.30	803.64	4.59	0.08
E1	GPM2	6B	AX.89770307	6.81E+08	0.34	610.64	3.03	0.05
E1	GPM2	6B	AX.89745770	1.28E+08	0.07	-1290.12	3.97	0.07
E1	GPM2	6B	AX.89514422	4.69E+08	0.14	-848.70	3.19	0.05
E1	GPM2	6B	AX.89544312	1.29E+08	0.07	-1291.54	3.98	0.07
E1	GPM2	6B	AX.89492919	1.29E+08	0.30	631.46	3.02	0.05
E1	GPM2	6B	AX.89618250	1.29E+08	0.30	631.46	3.02	0.05
E1	GPM2	6B	AX.89611281	6.81E+08	0.34	610.64	3.03	0.05
E1	TKW	6B	AX.89521152	6.71E+08	0.32	0.90	3.03	0.05
E1	GPM2	6B	AX.89652273	1.67E+05	0.20	768.57	3.31	0.06
E1	GPM2	6B	AX.89727379	1.20E+07	0.20	783.10	3.52	0.06
E1	GY	6B	AX.89583493	4.65E+08	0.20	-2.86	3.09	0.05
E1	GPM2	6B	AX.89328387	1.28E+08	0.07	-1365.71	4.17	0.07

E1	GY	6B	AX.89497224	4.63E+08	0.20	-2.86	3.09	0.05
E1	GPM2	6B	AX.89430017	1.25E+08	0.22	-734.89	3.29	0.05
E1	GY	6B	AX.89560383	4.26E+07	0.35	-2.53	3.40	0.05
E1	GPM2	6B	AX.89604116	1.29E+08	0.30	631.46	3.02	0.05
E1	GPM2	6B	AX.89527251	6.34E+06	0.34	-635.94	3.25	0.06
E1	GY	6B	AX.89577920	4.65E+08	0.20	-2.86	3.09	0.05
E1	GPM2	6B	AX.89631175	1.28E+08	0.07	-1290.12	3.97	0.07
E1	GPM2	6B	AX.89647703	1.29E+08	0.07	-1291.54	3.98	0.07
E1	GY	6B	AX.89774402	2.36E+08	0.16	-3.06	3.02	0.04
E1	TKW	6B	AX.89740335	3.27E+07	0.07	-1.96	4.14	0.07
E1	TKW	6B	AX.89578722	6.70E+08	0.32	0.90	3.03	0.05
E1	GY	6B	AX.89681269	2.38E+08	0.16	-3.06	3.02	0.04
E1	GY	6B	AX.89645517	2.27E+08	0.16	-3.06	3.02	0.04
E1	TKW	6B	AX.89433241	6.70E+08	0.32	0.90	3.03	0.05
E1	TKW	6B	AX.89402449	4.94E+07	0.05	-1.90	3.06	0.05
E1	GPM2	6B	AX.89336582	1.28E+08	0.08	-1200.75	3.85	0.06
E1	GPM2	6B	AX.89462797	1.29E+08	0.08	-1224.86	3.81	0.06
E1	TKW	6B	AX.89772714	6.71E+08	0.32	0.90	3.03	0.05
E1	GPM2	6B	AX.89680406	1.25E+08	0.22	-742.58	3.40	0.05
E1	GY	6B	AX.89709192	1.28E+08	0.06	-5.09	3.42	0.05
E1	GPM2	6B	AX.89707549	1.28E+08	0.07	-1290.12	3.97	0.07
E1	GPM2	6B	AX.89745555	5.97E+07	0.07	-1366.69	3.95	0.06
E1	TKW	6B	AX.89738573	2.78E+07	0.15	-1.22	3.27	0.05
E1	TKW	6B	AX.89536469	3.26E+07	0.08	-1.64	3.36	0.06
E1	TKW	6B	AX.89746864	3.26E+07	0.08	-1.64	3.36	0.06
E1	GPM2	6B	AX.89370625	3.57E+06	0.28	794.98	4.39	0.08
E1	TKW	6B	AX.89512454	6.66E+08	0.37	0.86	3.04	0.05
E1	GY	6B	AX.89763125	4.66E+08	0.20	-2.86	3.09	0.05
E2	SPM2	6B	AX.89773950	6.67E+08	0.10	19.47	3.11	0.05
E2	SPM2	6B	AX.89415057	2.80E+07	0.29	13.83	3.45	0.06
E2	GY	6B	AX.89647084	6.42E+08	0.05	-5.15	3.87	0.08
E2	SPM2	6B	AX.89526185	6.65E+08	0.10	20.12	3.18	0.05
E2	SPM2	6B	AX.89737931	6.68E+08	0.10	20.12	3.18	0.05
E2	SPM2	6B	AX.89541004	2.80E+07	0.25	15.00	3.64	0.06
E2	GY	6B	AX.89365639	6.41E+08	0.05	-5.15	3.87	0.08
E2	GY	6B	AX.89365837	1.15E+07	0.28	2.26	3.47	0.04
E2	SPM2	6B	AX.89515773	2.80E+07	0.30	12.65	3.03	0.05
E2	SPM2	6B	AX.89518009	2.80E+07	0.28	14.28	3.61	0.06
E2	SPM2	6B	AX.89667976	6.65E+08	0.10	20.12	3.18	0.05
E2	SPM2	6B	AX.89522613	6.97E+08	0.23	13.98	3.08	0.05
E2	SPM2	6B	AX.89548133	6.67E+08	0.10	20.12	3.18	0.05
E2	GY	6B	AX.89368639	3.42E+07	0.06	-4.00	3.12	0.04
E2	GY	6B	AX.89368639	3.42E+07	0.06	-4.00	3.12	0.04
E2	SPM2	6B	AX.89405089	6.64E+08	0.09	20.28	3.10	0.05
E2	SPM2	6B	AX.89573293	6.65E+08	0.10	19.93	3.24	0.05
E2	SPM2	6B	AX.89775847	1.93E+08	0.05	27.22	3.20	0.03
E2	GY	6B	AX.89488073	1.26E+07	0.40	-2.02	3.33	0.05
E2	SPM2	6B	AX.89636583	6.65E+08	0.10	20.12	3.18	0.05
E2	SPM2	6B	AX.89481103	6.67E+08	0.11	19.55	3.35	0.06
E2	SPM2	6B	AX.89653179	6.66E+08	0.10	19.47	3.11	0.05

E2	SPM2	6B	AX.89700613	4.87E+08	0.07	25.80	3.54	0.06
E2	SPM2	6B	AX.89446956	6.65E+08	0.11	20.14	3.41	0.06
E2	SPM2	6B	AX.89651887	1.15E+08	0.08	21.27	3.10	0.05
E2	SPM2	6B	AX.89684723	6.65E+08	0.10	20.12	3.18	0.05
E2	SPM2	6B	AX.89456984	2.80E+07	0.28	14.28	3.61	0.06
E2	SPM2	6B	AX.89760593	6.68E+08	0.11	19.55	3.35	0.06
E2	SPM2	6B	AX.89659500	6.66E+08	0.10	20.12	3.18	0.05
E2	SPM2	6B	AX.89453079	6.67E+08	0.10	20.12	3.18	0.05
E2	SPM2	6B	AX.89370949	2.80E+07	0.29	13.33	3.26	0.06
E2	GPS	6B	AX.89390995	6.87E+08	0.34	-1.47	3.33	0.06
E2	SPM2	6B	AX.89363496	2.80E+07	0.31	12.57	3.02	0.05
E2	SPM2	6B	AX.89520467	2.32E+08	0.05	26.24	3.01	0.05
E2	SPM2	6B	AX.89527556	6.66E+08	0.11	19.55	3.35	0.06
E2	GY	6B	AX.89446890	8.73E+07	0.09	-3.30	3.03	0.04
E2	GY	6B	AX.89446890	8.73E+07	0.09	-3.30	3.03	0.04
E2	GPS	6B	AX.89702417	6.64E+08	0.37	1.66	4.27	0.07
E2	GPS	6B	AX.89772591	2.13E+07	0.31	1.46	3.20	0.05
E2	GPM2	6B	AX.89433241	6.70E+08	0.31	-602.64	3.82	0.06
E2	GY	6B	AX.89613744	6.42E+08	0.05	-5.15	3.87	0.08
E2	GY	6B	AX.89613744	6.42E+08	0.05	-5.15	3.87	0.08
E2	GPS	6B	AX.89748271	4.74E+08	0.43	1.34	3.09	0.04
E2	GY	6B	AX.89488073	1.26E+07	0.40	-2.02	3.33	0.05
E2	GPS	6B	AX.89456512	6.87E+08	0.31	-1.55	3.50	0.06
E2	GY	6B	AX.89438340	6.42E+08	0.05	-5.15	3.87	0.08
E2	GY	6B	AX.89438340	6.42E+08	0.05	-5.15	3.87	0.08
E2	GY	6B	AX.89419179	3.42E+07	0.06	-4.00	3.12	0.04
E2	GY	6B	AX.89419179	3.42E+07	0.06	-4.00	3.12	0.04
E2	GPS	6B	AX.89442646	6.64E+08	0.39	1.41	3.27	0.05
E2	GPS	6B	AX.89700474	4.74E+08	0.43	1.34	3.09	0.04
E2	GPS	6B	AX.89533411	4.74E+08	0.43	1.34	3.09	0.04
E2	GPS	6B	AX.89485699	6.87E+08	0.31	-1.50	3.33	0.06
E2	GY	6B	AX.89419965	6.42E+08	0.05	-5.15	3.87	0.08
E2	GY	6B	AX.89419965	6.42E+08	0.05	-5.15	3.87	0.08
E2	GPS	6B	AX.89538522	4.27E+07	0.47	-1.43	3.50	0.07
E2	TKW	6B	AX.89659495	1.00E+08	0.15	-1.38	3.12	0.06
E2	TKW	6B	AX.89344094	9.41E+07	0.15	-1.38	3.12	0.06
E2	GPS	6B	AX.89471658	6.84E+08	0.41	-1.39	3.24	0.05
E2	GY	6B	AX.89647084	6.42E+08	0.05	-5.15	3.87	0.08
E2	GY	6B	AX.89365639	6.41E+08	0.05	-5.15	3.87	0.08
E2	GY	6B	AX.89365837	1.15E+07	0.28	2.26	3.47	0.04
E2	GPS	6B	AX.89364761	6.65E+08	0.46	1.54	3.95	0.07
E2	GPS	6B	AX.89558971	6.87E+08	0.24	-1.54	3.06	0.05
E2	TKW	6B	AX.89689694	1.13E+08	0.15	-1.38	3.12	0.06
E2	GPS	6B	AX.89468332	6.65E+08	0.47	1.42	3.47	0.06
E2	GPS	6B	AX.89725372	6.66E+08	0.48	-1.34	3.14	0.04
E2	TKW	6B	AX.89511298	6.71E+08	0.32	1.13	3.51	0.07
E2	GPS	6B	AX.89386498	4.74E+08	0.43	1.34	3.09	0.04
E2	GPS	6B	AX.89328871	4.74E+08	0.43	1.34	3.09	0.04
E2	GPS	6B	AX.89463926	4.74E+08	0.43	1.34	3.09	0.04
E2	GPS	6B	AX.89710107	6.80E+08	0.32	1.52	3.45	0.05

E2	GY	6B	AX.89740128	6.41E+08	0.05	-5.15	3.87	0.08
E2	GY	6B	AX.89740128	6.41E+08	0.05	-5.15	3.87	0.08
E2	GPS	6B	AX.89474705	6.65E+08	0.46	1.48	3.70	0.06
E2	TKW	6B	AX.89772714	6.71E+08	0.31	1.22	3.95	0.08
E2	GPS	6B	AX.89494058	4.74E+08	0.43	1.34	3.09	0.04
E2	GPS	6B	AX.89715367	6.87E+08	0.31	-1.57	3.60	0.06
E2	GPM2	6B	AX.89772714	6.71E+08	0.31	-602.64	3.82	0.06
E2	GPS	6B	AX.89689408	4.74E+08	0.43	1.41	3.39	0.04
E2	GPS	6B	AX.89756587	6.65E+08	0.47	1.44	3.54	0.06
E2	GPS	6B	AX.89724994	6.91E+08	0.40	-1.46	3.50	0.06
E2	GPS	6B	AX.89718746	6.65E+08	0.47	1.40	3.36	0.05
E2	GPS	6B	AX.89614845	6.87E+08	0.30	-1.66	3.87	0.07
E2	GPS	6B	AX.89647238	6.87E+08	0.30	-1.66	3.87	0.07
E2	TKW	6B	AX.89550703	3.41E+07	0.46	0.97	3.02	0.06
E2	GPS	6B	AX.89609142	4.74E+08	0.43	1.34	3.09	0.04
E2	GPM2	6B	AX.89512454	6.66E+08	0.37	-524.19	3.20	0.04
E2	GPM2	6B	AX.89511298	6.71E+08	0.32	-561.31	3.43	0.05
E2	GPS	6B	AX.89448317	6.87E+08	0.30	-1.66	3.87	0.07
E2	GPM2	6B	AX.89672126	6.87E+08	0.29	-536.18	3.07	0.05
E2	GPM2	6B	AX.89550703	3.41E+07	0.46	-533.29	3.51	0.08
E2	GPS	6B	AX.89508888	6.94E+08	0.49	-1.34	3.13	0.05
E2	GPS	6B	AX.89672126	6.87E+08	0.29	-1.67	3.87	0.07
E2	GPS	6B	AX.89501409	6.87E+08	0.34	-1.42	3.16	0.05
E2	GPS	6B	AX.89581796	6.64E+08	0.46	1.44	3.54	0.06
E2	GPS	6B	AX.89637862	6.69E+08	0.38	1.57	3.90	0.06
E2	GPS	6B	AX.89742164	6.65E+08	0.46	1.48	3.70	0.06
E2	GPS	6B	AX.89636340	6.80E+08	0.32	1.52	3.45	0.05
E2	GPS	6B	AX.89545263	6.83E+08	0.29	-1.48	3.11	0.05
E2	GPS	6B	AX.89644130	6.80E+08	0.32	1.52	3.45	0.05
E2	GPS	6B	AX.89430100	6.80E+08	0.32	1.52	3.45	0.05
E2	GPS	6B	AX.89367463	4.74E+08	0.43	1.34	3.09	0.04
E2	GPS	6B	AX.89553995	4.74E+08	0.43	1.34	3.09	0.04
E2	GPS	6B	AX.89453195	6.65E+08	0.47	1.44	3.54	0.06
E2	GPS	6B	AX.89774584	6.87E+08	0.24	-1.54	3.06	0.05
E2	GPS	6B	AX.89502067	4.74E+08	0.43	1.34	3.09	0.04
E2	TKW	6B	AX.89578722	6.70E+08	0.31	1.22	3.95	0.08
E2	GPS	6B	AX.89572829	4.74E+08	0.42	1.42	3.38	0.04
E2	GPS	6B	AX.89406272	6.80E+08	0.32	1.52	3.45	0.05
E2	GPM2	6B	AX.89393124	6.65E+08	0.43	-504.00	3.13	0.06
E2	GPS	6B	AX.89582169	2.27E+08	0.07	-2.84	3.42	0.07
E2	GPM2	6B	AX.89653439	6.71E+08	0.32	-663.35	4.53	0.07
E2	TKW	6B	AX.89740335	3.27E+07	0.07	-1.91	3.07	0.06
E2	TKW	6B	AX.89593900	9.40E+07	0.15	-1.38	3.12	0.06
E2	GPM2	6B	AX.89521152	6.71E+08	0.31	-602.64	3.82	0.06
E2	TKW	6B	AX.89438823	1.01E+08	0.17	-1.34	3.22	0.06
E2	TKW	6B	AX.89378889	1.14E+08	0.15	-1.53	3.74	0.07
E2	GPM2	6B	AX.89496535	6.68E+08	0.39	-497.38	3.01	0.04
E2	GPS	6B	AX.89349314	6.60E+08	0.45	1.32	3.05	0.05
E2	GPS	6B	AX.89374842	4.41E+07	0.48	-1.37	3.26	0.06
E2	GPS	6B	AX.89428683	6.79E+08	0.35	1.43	3.23	0.05

E2	GPM2	6B	AX.89568332	6.65E+08	0.43	-504.00	3.13	0.06
E2	TKW	6B	AX.89769081	1.01E+08	0.15	-1.41	3.15	0.06
E2	GPS	6B	AX.89663521	6.65E+08	0.46	1.50	3.77	0.06
E2	GPS	6B	AX.89449580	6.65E+08	0.46	1.48	3.70	0.06
E2	GPM2	6B	AX.89448461	6.65E+08	0.43	-504.00	3.13	0.06
E2	TKW	6B	AX.89746313	1.14E+08	0.16	-1.43	3.47	0.07
E2	GPS	6B	AX.89666072	6.64E+08	0.46	1.41	3.39	0.06
E2	TKW	6B	AX.89309543	9.97E+07	0.15	-1.38	3.12	0.06
E2	GPS	6B	AX.89332019	4.74E+08	0.43	1.34	3.09	0.04
E2	GPM2	6B	AX.89324679	6.65E+08	0.43	-504.00	3.13	0.06
E2	GPM2	6B	AX.89691169	6.65E+08	0.43	-504.00	3.13	0.06
E2	GPS	6B	AX.89380836	6.83E+08	0.29	-1.52	3.26	0.06
E2	GPS	6B	AX.89520467	2.32E+08	0.05	-3.00	3.09	0.07
E2	TKW	6B	AX.89467761	1.13E+08	0.15	-1.38	3.12	0.06
E2	TKW	6B	AX.89521152	6.71E+08	0.31	1.22	3.95	0.08
E2	TKW	6B	AX.89382096	9.97E+07	0.15	-1.38	3.18	0.06
E2	GPS	6B	AX.89562354	6.94E+08	0.44	-1.52	3.85	0.07
E2	GPS	6B	AX.89338776	4.74E+08	0.43	1.34	3.09	0.04
E2	GPS	6B	AX.89421987	4.74E+08	0.43	1.34	3.09	0.04
E2	GPM2	6B	AX.86168932	6.65E+08	0.43	-504.00	3.13	0.06
E2	GPS	6B	AX.86165948	6.83E+08	0.28	-1.48	3.06	0.05
E2	TKW	6B	AX.89653439	6.71E+08	0.32	1.22	3.96	0.08
E2	TKW	6B	AX.89526232	3.25E+07	0.40	1.11	3.67	0.07
E2	TKW	6B	AX.89334263	1.00E+08	0.15	-1.38	3.12	0.06
E2	GPS	6B	AX.89541177	6.87E+08	0.31	-1.55	3.50	0.06
E2	TKW	6B	AX.89436730	1.00E+08	0.15	-1.38	3.12	0.06
E2	TKW	6B	AX.89421643	6.64E+08	0.36	1.12	3.60	0.08
E2	GPS	6B	AX.89511788	6.91E+08	0.41	-1.36	3.15	0.06
E2	GPM2	6B	AX.89471658	6.84E+08	0.41	-515.75	3.31	0.06
E2	TKW	6B	AX.89716617	9.39E+07	0.15	-1.38	3.12	0.06
E2	GPM2	6B	AX.89578722	6.70E+08	0.31	-602.64	3.82	0.06
E2	TKW	6B	AX.89745004	1.14E+08	0.14	-1.47	3.30	0.06
E2	GPS	6B	AX.89664855	4.27E+07	0.47	-1.43	3.50	0.07
E2	TKW	6B	AX.89512454	6.66E+08	0.37	1.11	3.57	0.08
E2	GPS	6B	AX.89569889	4.74E+08	0.43	1.34	3.09	0.04
E2	GPS	6B	AX.89489768	6.79E+08	0.37	1.44	3.34	0.05
E2	TKW	6B	AX.89556458	7.07E+08	0.14	-1.45	3.15	0.06
E2	TKW	6B	AX.89447727	9.69E+07	0.15	-1.38	3.12	0.06
E2	TKW	6B	AX.89648820	1.13E+08	0.15	-1.38	3.12	0.06
E2	GPS	6B	AX.89385386	6.44E+08	0.15	-1.87	3.19	0.05
E2	GPS	6B	AX.89469894	6.87E+08	0.31	-1.57	3.60	0.06
E2	GPS	6B	AX.89393176	4.74E+08	0.43	1.34	3.09	0.04
E2	TKW	6B	AX.89531862	1.13E+08	0.15	-1.38	3.12	0.06
E2	GPS	6B	AX.89558753	4.74E+08	0.43	1.34	3.09	0.04
E2	GPS	6B	AX.89392557	6.65E+08	0.46	1.48	3.70	0.06
E2	TKW	6B	AX.89496535	6.68E+08	0.39	1.01	3.11	0.06
E2	TKW	6B	AX.89335615	1.13E+08	0.15	-1.38	3.12	0.06
E2	GPS	6B	AX.89501850	6.65E+08	0.46	1.48	3.70	0.06
E2	GPS	6B	AX.89488497	6.64E+08	0.37	1.66	4.27	0.07
E2	TKW	6B	AX.89571729	1.13E+08	0.15	-1.38	3.12	0.06

E2	TKW	6B	AX.89503877	1.07E+08	0.15	-1.38	3.12	0.06
E2	GPS	6B	AX.89542642	6.64E+08	0.46	1.45	3.56	0.06
E2	TKW	6B	AX.89672605	1.13E+08	0.15	-1.38	3.12	0.06
E2	TKW	6B	AX.89433241	6.70E+08	0.31	1.22	3.95	0.08
E3	GPM2	6B	AX.89355088	1.36E+08	0.28	994.10	3.45	0.08
E3	GPM2	6B	AX.89682344	1.36E+08	0.28	994.10	3.45	0.08
E3	GPM2	6B	AX.89524481	1.41E+08	0.18	1127.41	3.08	0.08
E3	GPM2	6B	AX.89455872	1.45E+08	0.24	985.51	3.18	0.08
E3	GPM2	6B	AX.89497347	4.51E+08	0.11	1490.93	3.65	0.01
E3	GPM2	6B	AX.89412102	4.51E+08	0.11	1432.68	3.45	0.01
E3	GY	6B	AX.89455872	1.45E+08	0.24	3.22	3.05	0.07
E3	GY	6B	AX.89497347	4.51E+08	0.11	4.93	3.59	0.01
E3	GY	6B	AX.89412102	4.51E+08	0.11	4.55	3.16	0.00
E3	SPM2	6B	AX.89601903	5.24E+07	0.18	-25.69	3.16	0.06
E3	SPM2	6B	AX.89497347	4.51E+08	0.08	36.13	3.25	0.04
E3	SPM2	6B	AX.89412102	4.51E+08	0.08	36.13	3.25	0.04
E3	SPM2	6B	AX.89403331	5.08E+08	0.07	42.37	3.73	0.06
E3	SPM2	6B	AX.89756640	7.14E+08	0.33	28.06	4.86	0.11
E3	SPM2	6B	AX.89773057	5.20E+07	0.18	-25.49	3.10	0.06
E3	SPM2	6B	AX.89372971	5.20E+07	0.18	-25.76	3.13	0.06
E3	SPM2	6B	X.89648081_OT	5.22E+07	0.19	-25.85	3.34	0.06
E3	SPM2	6B	X.89359127_OT	5.22E+07	0.18	-25.76	3.23	0.06
E3	SPM2	6B	AX.89709218	5.23E+07	0.18	-26.75	3.39	0.06
E3	SPM2	6B	AX.89603387	5.23E+07	0.18	-25.76	3.23	0.06
E3	SPM2	6B	AX.89372993	5.23E+07	0.18	-25.76	3.23	0.06
E3	SPM2	6B	AX.89580813	5.23E+07	0.18	-25.76	3.23	0.06
E3	SPM2	6B	AX.89560830	5.23E+07	0.18	-25.76	3.23	0.06
E3	SPM2	6B	AX.89616345	5.23E+07	0.18	-25.76	3.23	0.06
E3	SPM2	6B	AX.89579689	5.23E+07	0.18	-25.76	3.23	0.06
E3	SPM2	6B	AX.89686356	5.24E+07	0.18	-25.76	3.23	0.06
E4	GPM2	6B	AX.89429070	6.62E+08	0.22	-533.51	3.11	0.02
E4	GPM2	6B	AX.89597650	6.62E+08	0.22	-558.65	3.37	0.03
E4	GPM2	6B	X.89432769_OT	6.62E+08	0.22	-555.01	3.27	0.02
E4	GPS	6B	X.89409922_OT	4.32E+08	0.05	3.24	3.15	0.04
E4	GY	6B	AX.89632096	1.86E+07	0.11	-2.32	3.13	0.04
E4	GY	6B	AX.89766141	1.87E+07	0.10	-2.36	3.16	0.04
E4	GY	6B	AX.89330905	1.87E+07	0.10	-2.36	3.16	0.04
E4	GY	6B	AX.89443188	1.89E+07	0.42	-1.63	3.77	0.06
E4	GY	6B	AX.89532455	1.92E+07	0.14	-2.14	3.22	0.04
E4	GY	6B	AX.89720051	1.92E+07	0.14	-2.14	3.22	0.04
E4	GY	6B	X.89499897_OT	1.92E+07	0.13	-2.22	3.37	0.05
E4	GY	6B	AX.89365823	1.98E+07	0.15	-2.04	3.16	0.05
E4	GY	6B	X.89487057_OT	7.13E+08	0.08	-2.59	3.03	0.03
E4	SPM2	6B	AX.89712211	1.92E+07	0.14	-23.53	3.30	0.03
E4	SPM2	6B	AX.89472341	2.00E+07	0.14	-21.82	3.01	0.03
E4	SPM2	6B	AX.89550024	1.20E+08	0.36	17.50	3.15	0.05
E4	SPM2	6B	AX.89369178	1.20E+08	0.36	18.42	3.40	0.06
E4	SPM2	6B	AX.89323226	1.20E+08	0.36	17.99	3.26	0.05
E4	SPM2	6B	AX.89497402	1.20E+08	0.36	18.14	3.31	0.06
E4	SPM2	6B	AX.89748300	1.20E+08	0.37	17.22	3.12	0.05

E4	SPM2	6B	AX.89672794	1.20E+08	0.36	17.67	3.17	0.05
E4	SPM2	6B	AX.89357581	1.20E+08	0.36	17.67	3.17	0.05
E4	SPM2	6B	AX.89772989	1.20E+08	0.36	17.67	3.17	0.05
E4	SPM2	6B	AX.89540060	1.20E+08	0.36	18.14	3.31	0.06
E4	SPM2	6B	AX.89339906	1.20E+08	0.35	18.10	3.27	0.05
E4	SPM2	6B	AX.89343765	1.20E+08	0.35	18.10	3.27	0.05
E4	SPM2	6B	AX.89406775	1.20E+08	0.35	18.10	3.27	0.05
E4	SPM2	6B	AX.89602703	1.20E+08	0.36	17.75	3.19	0.05
E4	SPM2	6B	AX.89420193	1.21E+08	0.36	17.65	3.20	0.06
E4	SPM2	6B	AX.89355697	1.24E+08	0.12	29.81	3.89	0.09
E4	SPM2	6B	AX.89628267	1.24E+08	0.11	30.10	3.94	0.09
E4	SPM2	6B	AX.89481650	1.24E+08	0.11	30.10	3.94	0.09
E4	SPM2	6B	AX.89700249	1.24E+08	0.13	29.20	4.23	0.09
E4	SPM2	6B	AX.89410732	1.24E+08	0.12	28.84	3.88	0.09
E4	SPM2	6B	AX.89659601	1.24E+08	0.12	28.84	3.88	0.09
E4	SPM2	6B	AX.89508956	1.24E+08	0.13	35.94	6.08	0.13
E4	SPM2	6B	AX.89459468	1.24E+08	0.13	35.94	6.08	0.13
E4	SPM2	6B	AX.89329918	1.24E+08	0.11	35.80	5.55	0.12
E4	SPM2	6B	AX.89699961	1.25E+08	0.13	35.94	6.08	0.13
E4	SPM2	6B	AX.89650296	1.25E+08	0.13	35.94	6.08	0.13
E4	SPM2	6B	AX.89539688	1.25E+08	0.08	32.33	3.42	0.09
E4	SPM2	6B	AX.89650362	1.25E+08	0.12	35.47	5.41	0.12
E4	SPM2	6B	AX.89750622	2.05E+08	0.31	18.61	3.45	0.05
E4	SPM2	6B	AX.89433742	6.34E+08	0.25	19.65	3.20	0.08
E4	SPM2	6B	AX.89714678	6.34E+08	0.25	19.65	3.20	0.08
E4	SPM2	6B	AX.89392781	6.34E+08	0.25	19.65	3.20	0.08
E4	SPM2	6B	AX.89761729	6.34E+08	0.25	19.65	3.20	0.08
E4	SPM2	6B	AX.89699701	6.34E+08	0.25	19.65	3.20	0.08
E4	SPM2	6B	AX.89693483	6.34E+08	0.25	19.65	3.20	0.08
E4	SPM2	6B	AX.89719016	6.34E+08	0.25	19.65	3.20	0.08
E4	SPM2	6B	AX.89718939	6.34E+08	0.25	19.65	3.20	0.08
E4	SPM2	6B	AX.89773356	6.35E+08	0.25	19.65	3.20	0.08
E4	SPM2	6B	AX.89567047	6.35E+08	0.25	19.65	3.20	0.08
E4	SPM2	6B	AX.89698453	6.35E+08	0.25	19.65	3.20	0.08
E4	SPM2	6B	AX.89383586	6.35E+08	0.25	19.65	3.20	0.08
E4	SPM2	6B	AX.89672632	6.35E+08	0.25	19.66	3.23	0.08
E4	SPM2	6B	AX.89620111	6.35E+08	0.26	18.78	3.02	0.08
E4	SPM2	6B	AX.89481153	6.35E+08	0.26	18.78	3.02	0.08
E4	SPM2	6B	AX.89581581	6.35E+08	0.25	19.66	3.23	0.08
E4	SPM2	6B	AX.89737989	6.35E+08	0.26	18.78	3.02	0.08
E4	SPM2	6B	AX.89321681	6.35E+08	0.25	19.66	3.23	0.08
E4	SPM2	6B	AX.89473888	6.35E+08	0.25	19.66	3.23	0.08
E4	SPM2	6B	AX.89356651	6.35E+08	0.26	18.78	3.02	0.08
E4	SPM2	6B	AX.89664327	6.37E+08	0.27	19.20	3.11	0.08
E4	SPM2	6B	AX.89531414	6.40E+08	0.27	18.91	3.07	0.08
E4	SPM2	6B	AX.89663095	6.44E+08	0.25	19.37	3.10	0.08
E4	SPM2	6B	AX.89392016	6.44E+08	0.25	20.10	3.29	0.09
E4	SPM2	6B	AX.89594816	6.44E+08	0.25	19.41	3.17	0.09
E4	SPM2	6B	AX.89350676	6.44E+08	0.24	20.39	3.30	0.09
E4	SPM2	6B	AX.89719516	6.44E+08	0.23	20.29	3.18	0.09

E4	SPM2	6B	AX.89628229	6.44E+08	0.22	21.40	3.40	0.09
E4	SPM2	6B	AX.89644015	6.44E+08	0.23	20.29	3.18	0.09
E4	SPM2	6B	AX.89342893	6.44E+08	0.23	20.37	3.16	0.09
E4	SPM2	6B	AX.89541400	6.44E+08	0.22	20.68	3.25	0.09
E4	SPM2	6B	AX.89372156	6.44E+08	0.22	20.68	3.25	0.09
E4	SPM2	6B	AX.89636004	6.44E+08	0.23	22.13	3.72	0.10
E4	SPM2	6B	AX.89311528	6.44E+08	0.23	20.60	3.27	0.09
E4	SPM2	6B	AX.89691296	6.44E+08	0.23	20.60	3.27	0.09
E4	SPM2	6B	AX.89633074	6.44E+08	0.23	22.42	3.79	0.10
E4	SPM2	6B	AX.89328418	6.44E+08	0.23	22.42	3.79	0.10
E4	SPM2	6B	AX.89708698	6.44E+08	0.23	22.42	3.79	0.10
E1	SPM2	6D	AX.89776468	4.57E+08	0.35	17.24	3.09	0.06
E1	SPM2	6D	AX.89332731	4.57E+08	0.34	18.20	3.36	0.06
E1	SPM2	6D	AX.89662783	4.57E+08	0.35	17.12	3.07	0.06
E1	GY	6D	AX.89337772	3.26E+08	0.05	-5.80	3.74	0.07
E1	SPM2	6D	AX.89623338	4.57E+08	0.35	17.24	3.09	0.06
E1	SPM2	6D	AX.89446230	4.57E+08	0.35	17.12	3.07	0.06
E1	GY	6D	AX.89315989	4.60E+08	0.30	2.46	3.03	0.05
E1	SPM2	6D	AX.89523086	4.57E+08	0.36	18.09	3.39	0.06
E1	GY	6D	AX.89380458	9.79E+07	0.07	-5.08	3.62	0.07
E1	SPM2	6D	AX.89534983	4.57E+08	0.23	19.52	3.12	0.06
E1	SPM2	6D	AX.89713937	4.57E+08	0.35	17.24	3.09	0.06
E1	SPM2	6D	AX.89775608	4.59E+08	0.22	19.73	3.09	0.06
E1	GY	6D	AX.89380458	9.79E+07	0.07	-5.08	3.62	0.07
E1	SPM2	6D	AX.89661747	4.57E+08	0.35	17.24	3.09	0.06
E1	SPM2	6D	AX.89748977	4.57E+08	0.36	17.61	3.26	0.06
E1	SPM2	6D	AX.89744758	4.58E+08	0.24	19.37	3.14	0.06
E1	GY	6D	AX.89757204	3.29E+08	0.05	-5.64	3.30	0.06
E1	SPM2	6D	AX.89522782	4.58E+08	0.33	17.52	3.09	0.06
E1	GY	6D	AX.89351558	3.28E+08	0.05	-5.80	3.74	0.07
E1	SPM2	6D	AX.89336925	4.58E+08	0.23	19.80	3.19	0.06
E1	SPM2	6D	AX.89323273	4.57E+08	0.24	20.74	3.54	0.06
E1	GY	6D	AX.89315989	4.60E+08	0.30	2.46	3.03	0.05
E1	GY	6D	AX.89523090	3.35E+08	0.05	-5.80	3.74	0.07
E1	SPM2	6D	AX.89351003	4.57E+08	0.35	17.12	3.07	0.06
E1	SPM2	6D	AX.89681366	4.57E+08	0.35	17.24	3.09	0.06
E1	SPM2	6D	AX.89454903	4.57E+08	0.35	17.12	3.07	0.06
E1	GY	6D	AX.89523090	3.35E+08	0.05	-5.80	3.74	0.07
E1	GY	6D	AX.89351558	3.28E+08	0.05	-5.80	3.74	0.07
E1	SPM2	6D	AX.89488442	4.58E+08	0.26	18.89	3.15	0.06
E1	GY	6D	AX.89757204	3.29E+08	0.05	-5.64	3.30	0.06
E1	SPM2	6D	AX.89490043	4.58E+08	0.23	21.24	3.61	0.07
E1	SPM2	6D	AX.89355985	4.57E+08	0.35	17.12	3.07	0.06
E1	GY	6D	AX.89484426	3.36E+08	0.05	-5.80	3.74	0.07
E1	GY	6D	AX.89584641	3.46E+08	0.05	-5.80	3.74	0.07
E1	SPM2	6D	AX.89390617	4.57E+08	0.35	17.16	3.09	0.06
E1	SPM2	6D	AX.89409271	4.57E+08	0.35	17.24	3.09	0.06
E1	SPM2	6D	AX.89361838	4.57E+08	0.35	17.24	3.09	0.06
E1	SPM2	6D	AX.89560134	4.57E+08	0.22	21.68	3.59	0.07
E1	SPM2	6D	AX.89776650	4.57E+08	0.35	17.24	3.09	0.06

E1	GY	6D	AX.89337772	3.26E+08	0.05	-5.80	3.74	0.07
E1	GY	6D	AX.89560792	1.23E+08	0.05	-5.79	3.74	0.06
E1	GY	6D	AX.89560792	1.23E+08	0.05	-5.79	3.74	0.06
E1	GY	6D	AX.89484426	3.36E+08	0.05	-5.80	3.74	0.07
E1	GPM2	6D	AX.89379693	8.56E+07	0.34	-618.22	3.09	0.05
E1	GPM2	6D	AX.89655415	2.05E+08	0.10	-1028.94	3.42	0.05
E1	GPM2	6D	AX.89479129	3.05E+06	0.12	1062.01	3.99	0.07
E1	GPM2	6D	AX.89366928	1.96E+08	0.18	-819.47	3.50	0.06
E1	GPM2	6D	AX.89650556	1.40E+08	0.19	-866.99	3.92	0.07
E1	GPM2	6D	AX.89313992	8.52E+07	0.36	-600.50	3.00	0.05
E1	GPS	6D	AX.89479129	3.05E+06	0.12	2.06	4.22	0.07
E1	GY	6D	AX.89610816	9.78E+07	0.05	-5.40	3.07	0.06
E1	GY	6D	AX.89610816	9.78E+07	0.05	-5.40	3.07	0.06
E1	GPM2	6D	AX.89560792	1.23E+08	0.05	-1333.06	3.09	0.05
E1	GPM2	6D	AX.89407176	1.43E+08	0.19	-866.99	3.92	0.07
E1	GPM2	6D	AX.89351558	3.28E+08	0.05	-1436.70	3.51	0.06
E1	GPM2	6D	AX.89652027	1.48E+08	0.10	-1028.94	3.42	0.05
E1	GPM2	6D	AX.89424112	1.81E+08	0.18	-819.47	3.50	0.06
E1	GPM2	6D	AX.89337772	3.26E+08	0.05	-1436.70	3.51	0.06
E1	GPM2	6D	AX.89756935	1.61E+08	0.10	-1028.94	3.42	0.05
E1	GPM2	6D	AX.89551296	8.73E+07	0.34	-627.79	3.16	0.05
E1	GPM2	6D	AX.89640677	1.52E+08	0.10	-1028.94	3.42	0.05
E1	GPM2	6D	AX.89496441	1.90E+08	0.10	-1028.94	3.42	0.05
E1	GPM2	6D	AX.89493775	1.77E+08	0.10	-1028.94	3.42	0.05
E1	GPM2	6D	AX.89725912	8.68E+07	0.34	-633.97	3.21	0.05
E1	GPM2	6D	AX.89578323	8.84E+07	0.33	-612.83	3.01	0.05
E1	GPM2	6D	AX.89692572	2.09E+08	0.10	-1028.94	3.42	0.05
E1	GPM2	6D	AX.89695027	8.56E+07	0.36	-600.50	3.00	0.05
E1	GPM2	6D	AX.89523090	3.35E+08	0.05	-1436.70	3.51	0.06
E1	GPM2	6D	AX.89380458	9.79E+07	0.07	-1338.57	3.78	0.07
E1	GPM2	6D	AX.89706862	2.63E+08	0.16	-824.58	3.29	0.06
E1	GPM2	6D	AX.89468803	8.75E+07	0.34	-618.04	3.09	0.05
E1	GPM2	6D	AX.89478543	2.05E+08	0.10	-1028.94	3.42	0.05
E1	GPM2	6D	AX.89583904	2.18E+08	0.10	-1028.94	3.42	0.05
E1	GPM2	6D	AX.89584641	3.46E+08	0.05	-1436.70	3.51	0.06
E1	GPM2	6D	AX.89485933	1.74E+08	0.10	-1028.94	3.42	0.05
E1	GY	6D	AX.89584641	3.46E+08	0.05	-5.80	3.74	0.07
E1	GPM2	6D	AX.89333917	2.09E+08	0.10	-1028.94	3.42	0.05
E1	GPM2	6D	AX.89359783	1.60E+08	0.10	-1028.94	3.42	0.05
E1	GPM2	6D	AX.89484426	3.36E+08	0.05	-1436.70	3.51	0.06
E1	GPM2	6D	AX.89562029	8.73E+07	0.33	-626.86	3.13	0.05
E2	GY	6D	AX.89351558	3.28E+08	0.05	-4.34	3.15	0.07
E2	GY	6D	AX.89399830	2.43E+06	0.14	-2.87	3.29	0.03
E2	GY	6D	AX.89539764	2.43E+06	0.14	-2.87	3.29	0.03
E2	GY	6D	AX.89560357	2.43E+06	0.14	-2.70	3.05	0.03
E2	GY	6D	AX.89718621	2.44E+06	0.14	-3.07	3.84	0.04
E2	GY	6D	AX.89718621	2.44E+06	0.14	-3.07	3.84	0.04
E2	GY	6D	AX.89719214	2.42E+06	0.14	-2.80	3.25	0.03
E2	GY	6D	AX.89637263	2.50E+06	0.14	-2.87	3.29	0.03
E2	GY	6D	AX.89637263	2.50E+06	0.14	-2.87	3.29	0.03

E2	GY	6D	AX.89442454	2.43E+06	0.15	-2.64	3.01	0.03
E2	SPM2	6D	AX.89641175	4.65E+08	0.09	21.73	3.35	0.06
E2	SPM2	6D	AX.89771467	4.65E+08	0.15	19.64	4.16	0.07
E2	GPS	6D	AX.89351558	3.28E+08	0.05	-3.14	3.32	0.07
E2	GY	6D	AX.89372070	2.49E+06	0.14	-2.80	3.25	0.03
E2	GY	6D	AX.89337772	3.26E+08	0.05	-4.34	3.15	0.07
E2	GY	6D	AX.89481312	2.48E+06	0.15	-2.64	3.01	0.03
E2	GY	6D	AX.89586205	2.47E+06	0.14	-2.80	3.25	0.03
E2	GY	6D	AX.89586205	2.47E+06	0.14	-2.80	3.25	0.03
E2	GY	6D	AX.89775376	2.49E+06	0.14	-2.80	3.25	0.03
E2	GY	6D	AX.89775376	2.49E+06	0.14	-2.80	3.25	0.03
E2	GY	6D	AX.89560357	2.43E+06	0.14	-2.70	3.05	0.03
E2	GY	6D	AX.89624623	9.99E+07	0.07	-4.38	3.88	0.08
E2	GY	6D	AX.89624623	9.99E+07	0.07	-4.38	3.88	0.08
E2	GPM2	6D	AX.89710620	1.17E+08	0.12	-753.50	3.06	0.07
E2	GY	6D	AX.89414992	2.49E+06	0.15	-2.64	3.01	0.03
E2	GY	6D	AX.89414992	2.49E+06	0.15	-2.64	3.01	0.03
E2	GY	6D	AX.89614756	2.49E+06	0.14	-2.80	3.25	0.03
E2	GY	6D	AX.89614756	2.49E+06	0.14	-2.80	3.25	0.03
E2	GY	6D	AX.89748670	2.49E+06	0.14	-2.87	3.29	0.03
E2	GY	6D	AX.89748670	2.49E+06	0.14	-2.87	3.29	0.03
E2	GPM2	6D	AX.89380458	9.79E+07	0.07	-1169.49	4.25	0.09
E2	GY	6D	AX.89380458	9.79E+07	0.07	-5.29	5.44	0.11
E2	GY	6D	AX.89380458	9.79E+07	0.07	-5.29	5.44	0.11
E2	GY	6D	AX.89523090	3.35E+08	0.05	-4.34	3.15	0.07
E2	GY	6D	AX.89523090	3.35E+08	0.05	-4.34	3.15	0.07
E2	GY	6D	AX.89312869	2.48E+06	0.14	-2.80	3.25	0.03
E2	GY	6D	AX.89312869	2.48E+06	0.14	-2.80	3.25	0.03
E2	GY	6D	AX.89313357	2.50E+06	0.14	-2.71	3.07	0.03
E2	GY	6D	AX.89313357	2.50E+06	0.14	-2.71	3.07	0.03
E2	GY	6D	AX.89382799	2.43E+06	0.13	-3.18	3.85	0.04
E2	GY	6D	AX.89382799	2.43E+06	0.13	-3.18	3.85	0.04
E2	GY	6D	AX.89490273	2.42E+06	0.15	-2.67	3.13	0.06
E2	GY	6D	AX.89490273	2.42E+06	0.15	-2.67	3.13	0.06
E2	GY	6D	AX.89619145	2.42E+06	0.14	-2.80	3.25	0.03
E2	GY	6D	AX.89619145	2.42E+06	0.14	-2.80	3.25	0.03
E2	GY	6D	AX.89454517	2.49E+06	0.15	-2.64	3.01	0.03
E2	GY	6D	AX.89454517	2.49E+06	0.15	-2.64	3.01	0.03
E2	GY	6D	AX.89778248	2.49E+06	0.15	-2.64	3.01	0.03
E2	GY	6D	AX.89778248	2.49E+06	0.15	-2.64	3.01	0.03
E2	GY	6D	AX.89657062	2.45E+06	0.14	-2.80	3.25	0.03
E2	GY	6D	AX.89657062	2.45E+06	0.14	-2.80	3.25	0.03
E2	GY	6D	AX.89538990	2.47E+06	0.13	-3.18	3.85	0.04
E2	GY	6D	AX.89672765	2.49E+06	0.14	-2.87	3.29	0.03
E2	GY	6D	AX.89672765	2.49E+06	0.14	-2.87	3.29	0.03
E2	GPM2	6D	AX.89428596	9.09E+07	0.15	-770.83	3.87	0.07
E2	GY	6D	AX.89389247	7.19E+06	0.43	2.09	3.58	0.05
E2	GY	6D	AX.89389247	7.19E+06	0.43	2.09	3.58	0.05
E2	GY	6D	AX.89626257	2.49E+06	0.14	-2.80	3.25	0.03
E2	GY	6D	AX.89391510	2.45E+06	0.14	-2.80	3.25	0.03

E2	GY	6D	AX.89391510	2.45E+06	0.14	-2.80	3.25	0.03
E2	GY	6D	AX.89535290	2.47E+06	0.14	-3.10	3.79	0.04
E2	GY	6D	AX.89535290	2.47E+06	0.14	-3.10	3.79	0.04
E2	GY	6D	AX.89464601	2.50E+06	0.14	-2.87	3.29	0.03
E2	GY	6D	AX.89464601	2.50E+06	0.14	-2.87	3.29	0.03
E2	GY	6D	AX.89607698	2.47E+06	0.14	-2.80	3.25	0.03
E2	GY	6D	AX.89607698	2.47E+06	0.14	-2.80	3.25	0.03
E2	GY	6D	AX.89608316	2.43E+06	0.14	-2.87	3.29	0.03
E2	GY	6D	AX.89608316	2.43E+06	0.14	-2.87	3.29	0.03
E2	GY	6D	AX.89433283	2.50E+06	0.14	-2.80	3.25	0.03
E2	GY	6D	AX.89433283	2.50E+06	0.14	-2.80	3.25	0.03
E2	GY	6D	AX.89397880	2.42E+06	0.14	-2.87	3.29	0.03
E2	GY	6D	AX.89397880	2.42E+06	0.14	-2.87	3.29	0.03
E2	GY	6D	AX.89538990	2.47E+06	0.13	-3.18	3.85	0.04
E2	GY	6D	AX.89351558	3.28E+08	0.05	-4.34	3.15	0.07
E2	GY	6D	AX.89399830	2.43E+06	0.14	-2.87	3.29	0.03
E2	GY	6D	AX.89539764	2.43E+06	0.14	-2.87	3.29	0.03
E2	GY	6D	AX.89365404	2.50E+06	0.13	-2.80	3.08	0.03
E2	GY	6D	AX.89365404	2.50E+06	0.13	-2.80	3.08	0.03
E2	GPM2	6D	AX.89459659	9.72E+07	0.17	-701.24	3.61	0.06
E2	GY	6D	AX.89719214	2.42E+06	0.14	-2.80	3.25	0.03
E2	GY	6D	AX.89442454	2.43E+06	0.15	-2.64	3.01	0.03
E2	GY	6D	AX.89626257	2.49E+06	0.14	-2.80	3.25	0.03
E2	GY	6D	AX.89721743	2.42E+06	0.14	-2.80	3.25	0.03
E2	GY	6D	AX.89721743	2.42E+06	0.14	-2.80	3.25	0.03
E2	GPM2	6D	AX.89657963	9.72E+07	0.16	-748.17	3.82	0.07
E2	GY	6D	AX.89372070	2.49E+06	0.14	-2.80	3.25	0.03
E2	GY	6D	AX.89337772	3.26E+08	0.05	-4.34	3.15	0.07
E2	GPS	6D	AX.89484426	3.36E+08	0.05	-3.14	3.32	0.07
E2	GY	6D	AX.89481312	2.48E+06	0.15	-2.64	3.01	0.03
E2	GPM2	6D	AX.89313751	1.09E+08	0.12	-753.50	3.06	0.07
E2	GY	6D	AX.89761530	2.49E+06	0.14	-2.80	3.25	0.03
E2	GY	6D	AX.89564244	2.50E+06	0.14	-2.79	3.15	0.03
E2	GY	6D	AX.89484426	3.36E+08	0.05	-4.34	3.15	0.07
E2	GY	6D	AX.89484426	3.36E+08	0.05	-4.34	3.15	0.07
E2	GY	6D	AX.89705597	2.43E+06	0.14	-2.80	3.25	0.03
E2	GY	6D	AX.89705597	2.43E+06	0.14	-2.80	3.25	0.03
E2	GY	6D	AX.89701981	2.50E+06	0.14	-2.80	3.25	0.03
E2	GY	6D	AX.89701981	2.50E+06	0.14	-2.80	3.25	0.03
E2	GPS	6D	AX.89523090	3.35E+08	0.05	-3.14	3.32	0.07
E2	GY	6D	AX.89584641	3.46E+08	0.05	-4.34	3.15	0.07
E2	GY	6D	AX.89584641	3.46E+08	0.05	-4.34	3.15	0.07
E2	TKW	6D	AX.89340531	4.38E+08	0.42	0.99	3.42	0.06
E2	GY	6D	AX.89761530	2.49E+06	0.14	-2.80	3.25	0.03
E2	GY	6D	AX.89564244	2.50E+06	0.14	-2.79	3.15	0.03
E2	GPM2	6D	AX.89624623	9.99E+07	0.07	-1029.68	3.39	0.07
E2	GY	6D	AX.89531675	2.44E+06	0.15	-2.75	3.22	0.03
E2	GY	6D	AX.89531675	2.44E+06	0.15	-2.75	3.22	0.03
E2	GY	6D	AX.89478700	2.43E+06	0.14	-2.87	3.29	0.03
E2	GY	6D	AX.89568442	2.44E+06	0.15	-2.64	3.01	0.03

E2	GY	6D	AX.89568442	2.44E+06	0.15	-2.64	3.01	0.03
E2	GPM2	6D	AX.89620167	1.15E+08	0.12	-753.50	3.06	0.07
E2	GPS	6D	AX.89337772	3.26E+08	0.05	-3.14	3.32	0.07
E2	GPS	6D	AX.89757204	3.29E+08	0.05	-3.17	3.12	0.07
E2	GPS	6D	AX.89584641	3.46E+08	0.05	-3.14	3.32	0.07
E2	GY	6D	AX.89478700	2.43E+06	0.14	-2.87	3.29	0.03
E2	GPM2	6D	AX.89417270	1.04E+08	0.12	-753.50	3.06	0.07
E2	GPM2	6D	AX.89477214	1.10E+08	0.12	-753.50	3.06	0.07
E2	GPM2	6D	AX.89436776	1.14E+08	0.12	-753.50	3.06	0.07
E2	GPM2	6D	AX.89393162	9.43E+07	0.17	-701.24	3.61	0.06
E3	GPS	6D	AX.89563340	2.71E+08	0.30	-2.79	3.63	0.09
E3	GPS	6D	AX.89460370	3.10E+08	0.29	-2.75	3.60	0.09
E3	GPS	6D	AX.89337772	3.26E+08	0.31	-2.37	3.11	0.09
E3	GPS	6D	AX.89455858	3.55E+08	0.32	-2.31	3.12	0.09
E3	TKW	6D	AX.89710114	4.23E+08	0.35	-0.95	3.33	0.11
E3	TKW	6D	AX.89694015	4.24E+08	0.33	-0.92	3.10	0.10
E3	TKW	6D	AX.89531483	4.36E+08	0.29	-0.93	3.32	0.08
E3	SPM2	6D	AX.89652981	4.70E+08	0.17	26.71	3.22	0.05
E4	GPS	6D	X.89700629_OT	4.70E+08	0.20	-1.88	3.07	0.06
E4	GY	6D	AX.89493553	1.04E+07	0.10	-2.59	3.24	0.04
E4	GY	6D	AX.89536933	1.09E+07	0.21	1.81	3.06	0.02
E4	GPM2	6D	AX.89536933	1.09E+07	0.21	606.73	3.71	0.03
E4	SPM2	6D	AX.89397790	4.21E+08	0.24	20.50	3.04	0.08
E4	SPM2	6D	X.89700629_OT	4.70E+08	0.20	21.98	3.41	0.07
E4	TKW	6D	AX.89424484	9.68E+07	0.49	1.13	4.58	0.13
E4	TKW	6D	AX.89459659	9.72E+07	0.43	-0.96	3.30	0.10
E4	TKW	6D	AX.89657963	9.72E+07	0.46	-0.91	3.20	0.10
E4	TKW	6D	AX.89710114	4.23E+08	0.35	-1.17	4.88	0.14
E4	TKW	6D	AX.89694015	4.24E+08	0.33	-1.11	4.34	0.12
E4	TKW	6D	AX.89347001	4.36E+08	0.24	-1.00	3.23	0.07
E4	TKW	6D	AX.89428596	9.09E+07	0.49	-1.08	4.11	0.14
E4	TKW	6D	AX.89393162	9.43E+07	0.42	-1.05	3.84	0.11
E4	TKW	6D	AX.89659214	4.70E+07	0.11	-1.45	3.96	0.07
E1	SPM2	7A	AX.89674449	8.52E+07	0.47	-16.75	3.19	0.06
E1	SPM2	7A	AX.89365387	8.53E+07	0.48	-18.52	3.80	0.07
E1	SPM2	7A	AX.89728059	8.53E+07	0.48	-18.52	3.80	0.07
E1	SPM2	7A	AX.89750129	8.52E+07	0.47	-16.61	3.15	0.06
E1	SPM2	7A	AX.89322489	8.52E+07	0.47	-16.93	3.25	0.06
E1	GY	7A	AX.89413105	6.89E+08	0.24	-2.63	3.05	0.04
E1	SPM2	7A	AX.89362135	8.53E+07	0.48	-18.52	3.80	0.07
E1	GY	7A	AX.89695175	6.92E+08	0.17	3.08	3.17	0.04
E1	SPM2	7A	AX.89691294	8.53E+07	0.48	-18.52	3.80	0.07
E1	GY	7A	AX.89407297	7.08E+08	0.36	-2.41	3.19	0.06
E1	SPM2	7A	AX.89437917	8.53E+07	0.48	-18.52	3.80	0.07
E1	SPM2	7A	AX.89427331	8.53E+07	0.48	-18.52	3.80	0.07
E1	SPM2	7A	AX.89358546	8.51E+07	0.47	-16.93	3.25	0.06
E1	SPM2	7A	AX.89389967	8.52E+07	0.47	-16.93	3.25	0.06
E1	SPM2	7A	AX.89389117	8.53E+07	0.48	-18.52	3.80	0.07
E1	SPM2	7A	AX.89310521	8.52E+07	0.47	-16.93	3.25	0.06
E1	SPM2	7A	AX.89669240	8.51E+07	0.49	16.79	3.22	0.06

E1	SPM2	7A	AX.89767940	8.51E+07	0.49	16.54	3.13	0.06
E1	GY	7A	AX.89402422	7.09E+08	0.32	-2.46	3.13	0.06
E1	SPM2	7A	AX.89477949	6.71E+08	0.14	24.02	3.24	0.06
E1	GY	7A	AX.89635020	6.92E+08	0.20	2.82	3.07	0.04
E1	SPM2	7A	AX.89496810	8.52E+07	0.47	-16.93	3.25	0.06
E1	GY	7A	AX.89576529	6.72E+08	0.17	-3.26	3.49	0.06
E1	SPM2	7A	AX.89571170	8.52E+07	0.47	-16.93	3.25	0.06
E1	SPM2	7A	AX.89676739	8.53E+07	0.48	-18.52	3.80	0.07
E1	GY	7A	AX.89338064	6.72E+08	0.15	-3.40	3.53	0.06
E1	GY	7A	AX.89358168	7.09E+08	0.36	-2.33	3.02	0.06
E1	SPM2	7A	AX.89766471	8.53E+07	0.48	-18.52	3.80	0.07
E1	GY	7A	AX.89407297	7.08E+08	0.36	-2.41	3.19	0.06
E1	SPM2	7A	AX.89525950	3.74E+07	0.45	16.42	3.06	0.06
E1	SPM2	7A	AX.89538956	8.52E+07	0.48	-16.15	3.00	0.05
E1	GY	7A	AX.89635020	6.92E+08	0.20	2.82	3.07	0.04
E1	SPM2	7A	AX.89610935	8.53E+07	0.48	-18.52	3.80	0.07
E1	SPM2	7A	AX.89561047	8.52E+07	0.47	-16.93	3.25	0.06
E1	SPM2	7A	AX.89635468	8.54E+07	0.48	-18.52	3.80	0.07
E1	GY	7A	AX.89695896	6.72E+08	0.17	-3.30	3.63	0.06
E1	GY	7A	AX.89768981	6.72E+08	0.16	-3.27	3.44	0.05
E1	SPM2	7A	AX.89416745	8.53E+07	0.48	-18.52	3.80	0.07
E1	SPM2	7A	AX.89347005	8.52E+07	0.47	-16.93	3.25	0.06
E1	SPM2	7A	AX.89342394	6.71E+08	0.14	24.02	3.24	0.06
E1	GY	7A	AX.89413105	6.89E+08	0.24	-2.63	3.05	0.04
E1	GY	7A	AX.89338064	6.72E+08	0.15	-3.40	3.53	0.06
E1	GY	7A	AX.89349756	6.72E+08	0.16	-3.27	3.44	0.05
E1	SPM2	7A	AX.89535619	8.51E+07	0.48	-16.74	3.19	0.06
E1	SPM2	7A	AX.89746781	8.53E+07	0.48	-18.52	3.80	0.07
E1	SPM2	7A	AX.89632073	8.52E+07	0.47	-16.61	3.15	0.06
E1	SPM2	7A	AX.89773032	8.53E+07	0.48	-18.52	3.80	0.07
E1	SPM2	7A	AX.89533985	8.52E+07	0.47	-16.93	3.25	0.06
E1	GY	7A	AX.89454617	6.90E+08	0.22	2.87	3.37	0.05
E1	GY	7A	AX.89455713	4.77E+07	0.07	-5.38	4.05	0.06
E1	GY	7A	AX.89695175	6.92E+08	0.17	3.08	3.17	0.04
E1	GY	7A	AX.89411001	6.92E+08	0.19	-3.79	4.96	0.09
E1	GY	7A	AX.89549505	1.77E+07	0.08	-4.56	3.72	0.06
E1	GY	7A	AX.89683408	1.15E+08	0.26	-2.55	3.00	0.06
E1	GY	7A	AX.89457417	6.92E+08	0.20	-3.81	5.18	0.09
E1	GY	7A	AX.89702366	5.61E+06	0.11	-3.90	3.43	0.05
E1	SPM2	7A	AX.89638466	8.52E+07	0.47	-16.61	3.15	0.06
E1	SPM2	7A	AX.89547147	8.52E+07	0.47	-17.71	3.51	0.06
E1	GY	7A	AX.89488417	6.72E+08	0.17	-3.30	3.63	0.06
E1	GY	7A	AX.89488417	6.72E+08	0.17	-3.30	3.63	0.06
E1	GY	7A	AX.89704060	1.81E+07	0.32	-2.90	4.20	0.06
E1	GY	7A	AX.89596072	6.66E+08	0.41	2.56	3.71	0.06
E1	GY	7A	AX.89349756	6.72E+08	0.16	-3.27	3.44	0.05
E1	GY	7A	AX.89754578	6.92E+08	0.21	-4.24	6.45	0.12
E1	GY	7A	AX.89455713	4.77E+07	0.07	-5.38	4.05	0.06
E1	SPM2	7A	AX.89549503	3.80E+07	0.23	20.47	3.33	0.06
E1	GY	7A	AX.89573163	6.91E+08	0.26	2.80	3.51	0.05

E1	GY	7A	AX.89411001	6.92E+08	0.19	-3.79	4.96	0.09
E1	GY	7A	AX.89529147	6.89E+08	0.22	3.20	4.02	0.06
E1	GY	7A	AX.89683408	1.15E+08	0.26	-2.55	3.00	0.06
E1	GY	7A	AX.89702366	5.61E+06	0.11	-3.90	3.43	0.05
E1	SPM2	7A	AX.89651685	8.51E+07	0.49	16.79	3.22	0.06
E1	SPM2	7A	AX.89393785	8.53E+07	0.48	-18.52	3.80	0.07
E1	SPM2	7A	AX.89698264	8.52E+07	0.47	-16.93	3.25	0.06
E1	SPM2	7A	AX.89322222	4.75E+07	0.16	22.66	3.19	0.05
E1	GPS	7A	AX.89385994	5.89E+08	0.50	1.11	3.01	0.05
E1	GPS	7A	AX.89488888	5.95E+08	0.50	1.11	3.01	0.05
E1	GPS	7A	AX.89759797	5.93E+08	0.50	1.10	3.00	0.05
E1	GPS	7A	AX.89609381	5.85E+08	0.50	1.11	3.01	0.05
E1	GPS	7A	AX.89692801	5.93E+08	0.50	1.10	3.00	0.05
E1	GPS	7A	AX.89565548	5.90E+08	0.50	1.10	3.00	0.05
E1	GPS	7A	AX.89611613	5.94E+08	0.50	1.11	3.01	0.05
E1	GPS	7A	AX.89649407	5.93E+08	0.50	1.11	3.01	0.05
E1	SPM2	7A	AX.89416656	8.51E+07	0.49	16.79	3.22	0.06
E1	GPS	7A	AX.89448058	5.95E+08	0.50	1.10	3.00	0.05
E1	GY	7A	AX.89645723	7.32E+08	0.16	-3.21	3.26	0.06
E1	GPS	7A	AX.89642256	5.94E+08	0.50	1.10	3.00	0.05
E1	GY	7A	AX.89768981	6.72E+08	0.16	-3.27	3.44	0.05
E1	GPS	7A	AX.89720693	5.92E+08	0.50	1.10	3.00	0.05
E1	GY	7A	AX.89729033	6.90E+08	0.22	2.87	3.37	0.05
E1	GPS	7A	AX.89618387	5.88E+08	0.49	1.13	3.14	0.06
E1	GPS	7A	AX.89635830	5.85E+08	0.50	1.11	3.01	0.05
E1	GPS	7A	AX.89310981	5.94E+08	0.50	1.11	3.01	0.05
E1	GY	7A	AX.89514228	6.89E+08	0.33	-2.38	3.05	0.04
E1	GPS	7A	AX.89660912	5.92E+08	0.50	1.10	3.00	0.05
E1	GPS	7A	AX.89605766	5.85E+08	0.50	1.11	3.01	0.05
E1	GPS	7A	AX.89777023	5.93E+08	0.50	1.10	3.00	0.05
E1	GPS	7A	AX.89725593	5.87E+08	0.50	1.11	3.01	0.05
E1	GPS	7A	AX.89637946	5.94E+08	0.50	1.11	3.01	0.05
E1	GPS	7A	AX.89685614	5.93E+08	0.50	1.10	3.00	0.05
E1	GPS	7A	AX.89713730	5.84E+08	0.50	1.10	3.00	0.05
E1	GY	7A	AX.89402422	7.09E+08	0.32	-2.46	3.13	0.06
E1	SPM2	7A	AX.89536189	8.52E+07	0.47	-16.93	3.25	0.06
E1	GPS	7A	AX.89646568	5.85E+08	0.50	1.11	3.01	0.05
E1	GPS	7A	AX.89497203	5.87E+08	0.50	1.11	3.01	0.05
E1	GPS	7A	AX.89694425	5.94E+08	0.50	1.11	3.01	0.05
E1	GPS	7A	AX.89672574	5.89E+08	0.50	1.11	3.01	0.05
E1	GPS	7A	AX.89515454	5.94E+08	0.50	1.10	3.00	0.05
E1	GPS	7A	AX.89753909	5.90E+08	0.50	1.11	3.01	0.05
E1	GPS	7A	AX.89443688	5.85E+08	0.50	1.11	3.01	0.05
E1	GPS	7A	AX.89349805	5.93E+08	0.50	1.10	3.00	0.05
E1	GPS	7A	AX.89332530	5.95E+08	0.50	1.11	3.01	0.05
E1	GPS	7A	AX.89545465	5.92E+08	0.50	1.11	3.01	0.05
E1	GPS	7A	AX.89734645	5.92E+08	0.50	1.10	3.00	0.05
E1	GPS	7A	AX.89429431	5.90E+08	0.50	1.10	3.00	0.05
E1	GY	7A	AX.89704060	1.81E+07	0.32	-2.90	4.20	0.06
E1	GY	7A	AX.89704079	2.99E+07	0.50	-2.43	3.47	0.05

E1	GPS	7A	AX.89351303	5.94E+08	0.50	1.11	3.01	0.05
E1	GPS	7A	AX.89365193	5.94E+08	0.50	1.11	3.01	0.05
E1	GPS	7A	AX.89451083	5.94E+08	0.50	1.11	3.01	0.05
E1	GPS	7A	AX.89516497	5.94E+08	0.50	1.11	3.01	0.05
E1	GY	7A	AX.89596072	6.66E+08	0.41	2.56	3.71	0.06
E1	GY	7A	AX.89495797	6.72E+08	0.17	-3.30	3.63	0.06
E1	GPS	7A	AX.89716275	5.95E+08	0.50	1.10	3.00	0.05
E1	GPS	7A	AX.89745144	5.89E+08	0.50	1.11	3.01	0.05
E1	GY	7A	AX.89529147	6.89E+08	0.22	3.20	4.02	0.06
E1	GY	7A	AX.89729033	6.90E+08	0.22	2.87	3.37	0.05
E1	GPS	7A	AX.89699633	5.89E+08	0.50	1.10	3.00	0.05
E1	GY	7A	AX.89514228	6.89E+08	0.33	-2.38	3.05	0.04
E1	GPS	7A	AX.89544267	5.94E+08	0.50	1.11	3.01	0.05
E1	GY	7A	AX.89354261	6.89E+08	0.26	-2.78	3.50	0.05
E1	GY	7A	AX.89354261	6.89E+08	0.26	-2.78	3.50	0.05
E1	GY	7A	AX.89650018	7.08E+08	0.36	-2.55	3.53	0.07
E1	GPS	7A	AX.89757807	5.84E+08	0.50	1.11	3.01	0.05
E1	GY	7A	AX.89358168	7.09E+08	0.36	-2.33	3.02	0.06
E1	GY	7A	AX.89614349	6.72E+08	0.20	-3.36	4.20	0.07
E1	GY	7A	AX.89454617	6.90E+08	0.22	2.87	3.37	0.05
E1	GY	7A	AX.89754578	6.92E+08	0.21	-4.24	6.45	0.12
E1	GY	7A	AX.89734863	6.72E+08	0.17	-3.30	3.63	0.06
E1	GY	7A	AX.89576529	6.72E+08	0.17	-3.26	3.49	0.06
E1	SPM2	7A	AX.89667694	8.53E+07	0.48	-18.52	3.80	0.07
E1	GPM2	7A	AX.89394325	2.33E+07	0.43	-591.75	3.08	0.05
E1	GPS	7A	AX.89706161	5.95E+08	0.50	1.11	3.01	0.05
E1	GY	7A	AX.89549505	1.77E+07	0.08	-4.56	3.72	0.06
E1	GY	7A	AX.89614349	6.72E+08	0.20	-3.36	4.20	0.07
E1	GPM2	7A	AX.89529147	6.89E+08	0.22	747.75	3.37	0.05
E1	GPS	7A	AX.89750175	5.89E+08	0.50	1.10	3.00	0.05
E1	GPS	7A	AX.89551470	5.94E+08	0.50	1.11	3.01	0.05
E1	GPS	7A	AX.89720788	5.90E+08	0.50	1.11	3.01	0.05
E1	GY	7A	AX.89655850	4.77E+07	0.07	-5.38	4.05	0.06
E1	GPS	7A	AX.89330229	5.93E+08	0.50	1.10	3.00	0.05
E1	GPS	7A	AX.89679799	5.84E+08	0.50	1.11	3.01	0.05
E1	GPS	7A	AX.89597433	5.95E+08	0.50	1.11	3.01	0.05
E1	GY	7A	AX.89548753	6.72E+08	0.18	-3.01	3.16	0.05
E1	GY	7A	AX.89548753	6.72E+08	0.18	-3.01	3.16	0.05
E1	GPS	7A	AX.89710877	5.95E+08	0.50	1.10	3.00	0.05
E1	GPS	7A	AX.89353390	5.94E+08	0.50	1.11	3.01	0.05
E1	GPS	7A	AX.89486315	5.84E+08	0.50	1.11	3.01	0.05
E1	GPS	7A	AX.89369950	5.87E+08	0.49	1.13	3.14	0.06
E1	GPS	7A	AX.89388880	5.93E+08	0.50	1.10	3.00	0.05
E1	GPS	7A	AX.89351810	5.94E+08	0.50	1.11	3.01	0.05
E1	GY	7A	AX.89695896	6.72E+08	0.17	-3.30	3.63	0.06
E1	GPS	7A	AX.89600212	5.90E+08	0.50	1.11	3.01	0.05
E1	GPS	7A	AX.89642992	5.92E+08	0.50	1.10	3.00	0.05
E1	GPS	7A	AX.89635005	5.84E+08	0.50	1.11	3.01	0.05
E1	GPS	7A	AX.89563942	5.92E+08	0.50	1.10	3.00	0.05
E1	GPS	7A	AX.89698062	5.94E+08	0.50	1.11	3.01	0.05

E1	GPS	7A	AX.89310664	5.94E+08	0.50	1.11	3.01	0.05
E1	GPS	7A	AX.89445589	5.84E+08	0.50	1.11	3.01	0.05
E1	GPS	7A	AX.89592062	5.93E+08	0.50	1.10	3.00	0.05
E1	GPS	7A	AX.89732799	5.93E+08	0.50	1.10	3.00	0.05
E1	GPS	7A	AX.89506490	5.85E+08	0.50	1.11	3.01	0.05
E1	GPS	7A	AX.89371382	5.94E+08	0.50	1.11	3.01	0.05
E1	GPS	7A	AX.89384576	5.90E+08	0.50	1.10	3.00	0.05
E1	GPM2	7A	AX.89691734	6.89E+08	0.22	747.75	3.37	0.05
E1	GPM2	7A	AX.89411001	6.92E+08	0.19	-859.96	3.93	0.07
E1	GPS	7A	AX.89644599	5.89E+08	0.50	1.11	3.01	0.05
E1	GPM2	7A	AX.89573163	6.91E+08	0.26	742.16	3.66	0.06
E1	GPS	7A	AX.89396521	5.93E+08	0.50	1.10	3.00	0.05
E1	GPS	7A	AX.89774333	5.86E+08	0.50	1.11	3.01	0.05
E1	GPM2	7A	AX.89704060	1.81E+07	0.32	-841.40	5.13	0.08
E1	GPS	7A	AX.89720748	5.94E+08	0.50	1.11	3.01	0.05
E1	GPM2	7A	AX.89457417	6.92E+08	0.20	-892.45	4.33	0.08
E1	GPS	7A	AX.89497669	5.94E+08	0.50	1.11	3.01	0.05
E1	GPS	7A	AX.89352041	5.95E+08	0.50	1.10	3.00	0.05
E1	GPS	7A	AX.89386099	5.85E+08	0.50	1.11	3.01	0.05
E1	GPS	7A	AX.89542688	5.92E+08	0.50	1.10	3.00	0.05
E1	GPM2	7A	AX.89383516	4.76E+07	0.09	-1032.22	3.08	0.05
E1	GPS	7A	AX.89660197	5.95E+08	0.46	1.20	3.43	0.06
E1	GPS	7A	AX.89374905	5.89E+08	0.50	1.11	3.01	0.05
E1	GPS	7A	AX.89658409	5.92E+08	0.50	1.10	3.00	0.05
E1	GPS	7A	AX.89346636	5.93E+08	0.50	1.10	3.00	0.05
E1	GY	7A	AX.89704079	2.99E+07	0.50	-2.43	3.47	0.05
E1	GPS	7A	AX.89362841	5.89E+08	0.50	1.11	3.01	0.05
E1	GPS	7A	AX.89767265	5.94E+08	0.50	1.10	3.00	0.05
E1	GPM2	7A	AX.89664843	1.53E+08	0.23	694.99	3.05	0.05
E1	GY	7A	AX.89495797	6.72E+08	0.17	-3.30	3.63	0.06
E1	GPS	7A	AX.89667879	5.95E+08	0.50	1.10	3.00	0.05
E1	GPM2	7A	AX.89409314	1.48E+08	0.35	702.91	3.88	0.07
E1	GPM2	7A	AX.89455713	4.77E+07	0.07	-1248.87	3.36	0.05
E1	GY	7A	AX.89457417	6.92E+08	0.20	-3.81	5.18	0.09
E1	GPM2	7A	AX.89655850	4.77E+07	0.07	-1248.87	3.36	0.05
E1	GY	7A	AX.89650018	7.08E+08	0.36	-2.55	3.53	0.07
E1	GPS	7A	AX.89766240	5.85E+08	0.50	1.11	3.01	0.05
E1	GY	7A	AX.89573163	6.91E+08	0.26	2.80	3.51	0.05
E1	GY	7A	AX.89691734	6.89E+08	0.22	3.20	4.02	0.06
E1	GY	7A	AX.89691734	6.89E+08	0.22	3.20	4.02	0.06
E1	GY	7A	AX.89734863	6.72E+08	0.17	-3.30	3.63	0.06
E1	GPM2	7A	AX.89512492	1.51E+08	0.34	696.43	3.77	0.07
E1	GPM2	7A	AX.89313296	6.80E+08	0.43	601.38	3.16	0.05
E1	GPM2	7A	AX.89754578	6.92E+08	0.21	-876.73	4.32	0.08
E1	GPS	7A	AX.89576658	5.89E+08	0.50	1.11	3.01	0.05
E1	GPS	7A	AX.89422707	5.90E+08	0.50	1.11	3.01	0.05
E1	GPM2	7A	AX.89722852	2.00E+07	0.23	-696.20	3.10	0.05
E1	GY	7A	AX.89655850	4.77E+07	0.07	-5.38	4.05	0.06
E1	GPS	7A	AX.89730860	5.87E+08	0.50	1.11	3.01	0.05
E1	GPS	7A	AX.89386665	5.94E+08	0.50	1.11	3.01	0.05

E1	GPS	7A	AX.89482517	5.95E+08	0.50	1.11	3.01	0.05
E1	GPS	7A	AX.89735190	5.89E+08	0.50	1.11	3.01	0.05
E1	GPS	7A	AX.89339548	5.92E+08	0.50	1.10	3.00	0.05
E1	GY	7A	AX.89645723	7.32E+08	0.16	-3.21	3.26	0.06
E1	GPS	7A	AX.89311431	5.94E+08	0.50	1.10	3.00	0.05
E1	GPM2	7A	AX.89391268	1.76E+08	0.31	672.08	3.39	0.06
E1	GPM2	7A	AX.89440082	1.87E+08	0.31	672.08	3.39	0.06
E1	GPS	7A	AX.89398740	5.85E+08	0.50	1.11	3.01	0.05
E1	GPS	7A	AX.89590533	5.95E+08	0.50	1.11	3.01	0.05
E1	GPS	7A	AX.89690732	5.89E+08	0.50	1.11	3.01	0.05
E1	GPS	7A	AX.89395568	5.92E+08	0.50	1.10	3.00	0.05
E1	TKW	7A	AX.89392964	1.64E+08	0.11	-1.50	3.78	0.06
E1	TKW	7A	AX.89357487	1.76E+08	0.10	-1.37	3.00	0.05
E1	TKW	7A	AX.89775856	1.56E+08	0.11	-1.50	3.78	0.06
E1	TKW	7A	AX.89747316	1.43E+08	0.11	-1.50	3.78	0.06
E1	TKW	7A	AX.89644863	1.30E+08	0.12	-1.53	4.01	0.07
E1	TKW	7A	AX.89660702	1.56E+08	0.10	-1.43	3.25	0.05
E1	TKW	7A	AX.89506329	9.31E+07	0.14	-1.56	4.76	0.08
E1	TKW	7A	AX.89467570	1.36E+08	0.12	-1.53	4.01	0.07
E1	TKW	7A	AX.89530390	1.66E+08	0.11	-1.50	3.78	0.06
E1	TKW	7A	AX.89456654	1.36E+08	0.11	-1.41	3.37	0.06
E1	GPS	7A	AX.89422146	5.84E+08	0.50	1.20	3.46	0.06
E1	TKW	7A	AX.89405523	1.67E+08	0.11	-1.50	3.78	0.06
E1	TKW	7A	AX.89764069	1.46E+08	0.11	-1.38	3.15	0.05
E1	TKW	7A	AX.89658297	1.54E+08	0.10	-1.44	3.19	0.05
E1	TKW	7A	AX.89493315	1.44E+08	0.11	-1.50	3.78	0.06
E1	TKW	7A	AX.89436797	1.54E+08	0.10	-1.43	3.25	0.05
E1	TKW	7A	AX.89485213	1.48E+08	0.11	-1.50	3.78	0.06
E1	TKW	7A	AX.89643651	1.53E+08	0.10	-1.43	3.25	0.05
E1	TKW	7A	AX.89767696	9.31E+07	0.14	-1.56	4.76	0.08
E1	TKW	7A	AX.89653836	1.30E+08	0.11	-1.46	3.48	0.06
E1	TKW	7A	AX.89336191	1.30E+08	0.11	-1.48	3.69	0.07
E1	TKW	7A	AX.89371906	1.76E+08	0.10	-1.37	3.00	0.05
E1	TKW	7A	AX.89366109	1.62E+08	0.11	-1.50	3.78	0.06
E1	TKW	7A	AX.89610878	1.61E+08	0.10	-1.43	3.25	0.05
E1	TKW	7A	AX.86163580	1.65E+08	0.11	-1.50	3.78	0.06
E1	TKW	7A	AX.89754400	1.56E+08	0.11	-1.50	3.78	0.06
E1	TKW	7A	AX.89374540	1.58E+08	0.11	-1.50	3.78	0.06
E1	TKW	7A	AX.89526473	1.45E+08	0.10	-1.44	3.29	0.05
E1	TKW	7A	AX.89315701	1.76E+08	0.11	-1.50	3.63	0.06
E1	TKW	7A	AX.89375093	1.51E+08	0.11	-1.56	3.90	0.07
E1	TKW	7A	AX.89731899	1.69E+08	0.11	-1.56	3.90	0.07
E1	TKW	7A	AX.89521547	1.51E+08	0.10	-1.43	3.25	0.05
E1	TKW	7A	AX.89738373	1.69E+08	0.11	-1.56	3.90	0.07
E1	TKW	7A	AX.89388443	1.63E+08	0.11	-1.50	3.78	0.06
E1	TKW	7A	AX.89432757	1.57E+08	0.11	-1.50	3.78	0.06
E1	TKW	7A	AX.89653048	2.29E+08	0.31	-0.93	3.17	0.05
E1	TKW	7A	AX.89340252	1.32E+08	0.11	-1.42	3.45	0.06
E1	TKW	7A	AX.89381481	1.46E+08	0.10	-1.44	3.29	0.05
E1	TKW	7A	AX.89475720	1.49E+08	0.11	-1.45	3.46	0.06

E1	TKW	7A	AX.89318490	1.68E+08	0.11	-1.50	3.78	0.06
E1	TKW	7A	AX.89545760	9.25E+07	0.09	-1.59	3.62	0.06
E1	GPM2	7A	AX.89690725	1.96E+07	0.24	-673.90	3.00	0.05
E1	TKW	7A	AX.89495746	1.28E+08	0.11	-1.45	3.44	0.06
E1	TKW	7A	AX.89314033	2.27E+08	0.28	-1.00	3.44	0.06
E1	TKW	7A	AX.89565462	9.25E+07	0.13	-1.75	5.70	0.10
E1	TKW	7A	AX.89442959	1.51E+08	0.11	-1.38	3.15	0.05
E1	TKW	7A	AX.89620228	1.43E+08	0.11	-1.50	3.78	0.06
E1	TKW	7A	AX.89634576	1.47E+08	0.10	-1.44	3.29	0.05
E1	TKW	7A	AX.89429619	1.66E+08	0.11	-1.50	3.78	0.06
E1	TKW	7A	AX.89428813	1.33E+08	0.12	-1.53	4.01	0.07
E1	TKW	7A	AX.89559779	1.45E+08	0.11	-1.38	3.15	0.05
E1	TKW	7A	AX.89376496	1.37E+08	0.09	-1.45	3.09	0.05
E1	TKW	7A	AX.89592759	2.38E+08	0.32	-0.90	3.03	0.05
E1	TKW	7A	AX.89405257	1.76E+08	0.10	-1.37	3.00	0.05
E1	TKW	7A	AX.89433774	1.43E+08	0.11	-1.50	3.78	0.06
E1	TKW	7A	AX.89481630	1.48E+08	0.11	-1.38	3.15	0.05
E1	TKW	7A	AX.89391855	1.57E+08	0.11	-1.50	3.78	0.06
E1	TKW	7A	AX.89431374	1.39E+08	0.11	-1.48	3.69	0.07
E1	TKW	7A	AX.89667558	1.63E+08	0.11	-1.50	3.78	0.06
E1	TKW	7A	AX.89364030	1.48E+08	0.10	-1.52	3.61	0.06
E1	TKW	7A	AX.89692675	2.33E+08	0.31	-0.93	3.24	0.05
E1	TKW	7A	AX.89478535	7.10E+08	0.35	-0.98	3.68	0.07
E1	TKW	7A	AX.89553153	1.63E+08	0.11	-1.38	3.15	0.05
E1	TKW	7A	AX.89699004	2.44E+08	0.47	-0.90	3.45	0.06
E1	TKW	7A	AX.89401398	1.36E+08	0.11	-1.48	3.69	0.07
E1	TKW	7A	AX.89673528	1.49E+08	0.10	-1.43	3.25	0.05
E1	TKW	7A	AX.89318703	1.44E+08	0.11	-1.50	3.78	0.06
E1	GPM2	7A	AX.89553054	1.47E+08	0.35	702.91	3.88	0.07
E1	TKW	7A	AX.89384804	1.73E+08	0.11	-1.50	3.63	0.06
E1	TKW	7A	AX.89326929	6.47E+08	0.24	-1.02	3.31	0.06
E1	TKW	7A	AX.89600026	1.45E+08	0.11	-1.50	3.78	0.06
E1	TKW	7A	AX.89337396	1.72E+08	0.10	-1.45	3.31	0.05
E1	TKW	7A	AX.89356497	1.59E+08	0.10	-1.43	3.25	0.05
E1	TKW	7A	AX.89490648	1.49E+08	0.11	-1.50	3.78	0.06
E1	TKW	7A	AX.89586120	1.39E+08	0.11	-1.48	3.69	0.07
E1	TKW	7A	AX.89350564	1.01E+08	0.09	-1.57	3.39	0.06
E1	TKW	7A	AX.89616482	1.47E+08	0.10	-1.44	3.29	0.05
E1	TKW	7A	AX.89658013	1.55E+08	0.11	-1.50	3.78	0.06
E1	TKW	7A	AX.89343125	1.50E+08	0.10	-1.43	3.25	0.05
E1	TKW	7A	AX.89653730	1.45E+08	0.11	-1.50	3.78	0.06
E1	TKW	7A	AX.89446261	1.48E+08	0.10	-1.44	3.29	0.05
E1	TKW	7A	AX.89658779	1.48E+08	0.10	-1.44	3.29	0.05
E1	TKW	7A	AX.89455107	1.47E+08	0.11	-1.50	3.78	0.06
E1	TKW	7A	AX.89459271	1.57E+08	0.11	-1.50	3.78	0.06
E1	TKW	7A	AX.89337397	1.69E+08	0.11	-1.56	3.90	0.07
E1	TKW	7A	AX.89538446	1.37E+08	0.11	-1.57	3.94	0.07
E1	TKW	7A	AX.89695896	6.72E+08	0.17	-1.15	3.28	0.06
E1	TKW	7A	AX.89312431	1.63E+08	0.10	-1.50	3.41	0.06
E1	TKW	7A	AX.89449879	1.63E+08	0.11	-1.38	3.15	0.05

E1	TKW	7A	AX.89547821	1.27E+08	0.10	-1.40	3.01	0.05
E1	TKW	7A	AX.89571322	1.74E+08	0.11	-1.34	3.10	0.05
E1	TKW	7A	AX.89737403	1.68E+08	0.11	-1.56	3.90	0.07
E1	TKW	7A	AX.89388449	1.31E+08	0.10	-1.41	3.16	0.06
E1	TKW	7A	AX.89753039	1.54E+08	0.10	-1.43	3.25	0.05
E1	TKW	7A	AX.89401450	1.40E+08	0.10	-1.57	3.67	0.07
E1	TKW	7A	AX.89689588	9.31E+07	0.09	-1.52	3.34	0.06
E1	TKW	7A	AX.89315926	1.49E+08	0.10	-1.43	3.25	0.05
E1	GPM2	7A	AX.89645723	7.32E+08	0.16	-899.19	3.74	0.07
E1	TKW	7A	AX.89548753	6.72E+08	0.18	-1.15	3.32	0.06
E1	TKW	7A	AX.89348017	1.76E+08	0.10	-1.37	3.00	0.05
E1	TKW	7A	AX.89559201	9.31E+07	0.14	-1.56	4.76	0.08
E1	TKW	7A	AX.89379993	1.75E+08	0.11	-1.50	3.63	0.06
E1	TKW	7A	AX.89622255	1.38E+08	0.10	-1.50	3.41	0.06
E1	TKW	7A	AX.89533854	9.30E+07	0.09	-1.53	3.39	0.06
E1	TKW	7A	AX.86168877	6.47E+08	0.22	-1.17	3.97	0.07
E1	TKW	7A	AX.89613023	1.63E+08	0.11	-1.38	3.15	0.05
E1	TKW	7A	AX.89427563	1.35E+08	0.10	-1.50	3.41	0.06
E1	TKW	7A	AX.89407230	1.15E+08	0.09	-1.68	3.83	0.06
E1	TKW	7A	AX.89364823	9.24E+07	0.13	-1.75	5.70	0.10
E1	TKW	7A	AX.89597147	9.76E+07	0.14	-1.49	4.48	0.08
E1	TKW	7A	AX.89600384	1.37E+08	0.11	-1.58	4.13	0.07
E1	TKW	7A	AX.89462997	1.70E+08	0.11	-1.50	3.78	0.06
E1	TKW	7A	AX.89639086	1.71E+08	0.11	-1.50	3.63	0.06
E1	TKW	7A	AX.89385641	1.57E+08	0.11	-1.50	3.78	0.06
E1	TKW	7A	AX.89527725	1.37E+08	0.12	-1.53	4.01	0.07
E1	TKW	7A	AX.89495040	1.76E+08	0.10	-1.37	3.00	0.05
E1	TKW	7A	AX.89603125	1.43E+08	0.11	-1.50	3.78	0.06
E1	TKW	7A	AX.89386536	1.37E+08	0.09	-1.45	3.09	0.05
E1	TKW	7A	AX.89333459	1.39E+08	0.11	-1.48	3.69	0.07
E1	TKW	7A	AX.89762890	1.64E+08	0.11	-1.50	3.78	0.06
E1	TKW	7A	AX.89489289	1.50E+08	0.11	-1.50	3.78	0.06
E1	TKW	7A	AX.89377964	1.48E+08	0.11	-1.50	3.78	0.06
E1	TKW	7A	AX.89665240	1.45E+08	0.11	-1.50	3.78	0.06
E1	TKW	7A	AX.89440867	1.40E+08	0.11	-1.41	3.37	0.06
E1	TKW	7A	AX.89449737	1.49E+08	0.11	-1.50	3.78	0.06
E1	TKW	7A	AX.89481492	1.43E+08	0.11	-1.50	3.78	0.06
E1	TKW	7A	AX.89635600	1.45E+08	0.11	-1.38	3.15	0.05
E1	TKW	7A	AX.89687577	1.46E+08	0.10	-1.44	3.29	0.05
E1	TKW	7A	AX.89379340	1.67E+08	0.11	-1.50	3.78	0.06
E1	TKW	7A	AX.89667906	1.63E+08	0.11	-1.50	3.78	0.06
E1	TKW	7A	AX.89366584	1.46E+08	0.11	-1.38	3.15	0.05
E1	TKW	7A	AX.89328096	2.32E+08	0.31	-0.93	3.17	0.05
E1	TKW	7A	AX.89381551	1.63E+08	0.11	-1.50	3.78	0.06
E1	TKW	7A	AX.89698137	1.76E+08	0.10	-1.37	3.00	0.05
E1	TKW	7A	AX.89655282	2.44E+08	0.47	-0.97	3.93	0.07
E1	TKW	7A	AX.89679467	2.32E+08	0.31	-0.93	3.17	0.05
E1	TKW	7A	AX.89594092	2.25E+08	0.30	-0.91	3.06	0.05
E1	TKW	7A	AX.89424255	1.38E+08	0.12	-1.44	3.72	0.07
E1	TKW	7A	AX.89328602	1.39E+08	0.10	-1.53	3.66	0.07

E1	TKW	7A	AX.89319052	1.57E+08	0.10	-1.43	3.25	0.05
E1	TKW	7A	AX.89744487	1.73E+08	0.11	-1.50	3.63	0.06
E1	TKW	7A	AX.89609344	1.63E+08	0.11	-1.50	3.78	0.06
E1	TKW	7A	AX.89739311	1.72E+08	0.10	-1.51	3.43	0.06
E1	TKW	7A	AX.89658640	1.28E+08	0.11	-1.50	3.76	0.07
E1	TKW	7A	AX.89737889	1.59E+08	0.11	-1.38	3.15	0.05
E1	TKW	7A	AX.89612988	1.75E+08	0.11	-1.50	3.63	0.06
E1	TKW	7A	AX.89436093	1.49E+08	0.11	-1.50	3.78	0.06
E1	TKW	7A	AX.89738372	9.29E+07	0.13	-1.75	5.70	0.10
E1	TKW	7A	AX.89616060	1.51E+08	0.11	-1.38	3.15	0.05
E1	TKW	7A	AX.89591464	1.46E+08	0.11	-1.50	3.78	0.06
E1	TKW	7A	AX.89688580	1.74E+08	0.11	-1.34	3.10	0.05
E1	TKW	7A	AX.89610842	1.38E+08	0.11	-1.48	3.69	0.07
E1	TKW	7A	AX.89717346	1.57E+08	0.11	-1.50	3.78	0.06
E1	TKW	7A	AX.89336822	1.61E+08	0.10	-1.43	3.25	0.05
E1	TKW	7A	AX.89408190	2.26E+08	0.30	-0.91	3.06	0.05
E1	TKW	7A	AX.89373952	1.65E+08	0.11	-1.38	3.15	0.05
E1	TKW	7A	AX.89748859	1.30E+08	0.12	-1.53	4.01	0.07
E1	TKW	7A	AX.89429278	1.39E+08	0.12	-1.53	4.01	0.07
E1	TKW	7A	AX.89739003	1.35E+08	0.10	-1.44	3.29	0.05
E1	TKW	7A	AX.89454546	1.65E+08	0.10	-1.44	3.29	0.05
E1	TKW	7A	AX.89408882	2.29E+08	0.31	-0.93	3.17	0.05
E1	TKW	7A	AX.89571802	1.73E+08	0.10	-1.37	3.00	0.05
E1	TKW	7A	AX.89494699	1.67E+08	0.11	-1.50	3.78	0.06
E1	TKW	7A	AX.89698983	1.62E+08	0.10	-1.43	3.25	0.05
E1	TKW	7A	AX.89668647	9.66E+07	0.12	-1.68	4.90	0.09
E1	TKW	7A	AX.89335196	2.33E+08	0.33	-0.90	3.12	0.05
E1	TKW	7A	AX.89684792	1.49E+08	0.11	-1.56	3.90	0.07
E1	TKW	7A	AX.89374946	1.58E+08	0.10	-1.43	3.25	0.05
E1	TKW	7A	AX.89358941	1.40E+08	0.12	-1.53	4.01	0.07
E1	TKW	7A	AX.89677238	1.72E+08	0.11	-1.53	3.77	0.06
E1	TKW	7A	AX.89604680	1.60E+08	0.11	-1.50	3.78	0.06
E1	TKW	7A	AX.89424299	2.11E+08	0.11	-1.45	3.46	0.06
E1	TKW	7A	AX.89408864	1.28E+08	0.10	-1.42	3.22	0.06
E1	TKW	7A	AX.89384986	1.45E+08	0.11	-1.50	3.78	0.06
E1	TKW	7A	AX.89519151	1.30E+08	0.12	-1.53	4.01	0.07
E1	TKW	7A	AX.89541663	1.55E+08	0.10	-1.43	3.25	0.05
E1	TKW	7A	AX.89424539	1.69E+08	0.11	-1.50	3.78	0.06
E1	TKW	7A	AX.89495220	1.39E+08	0.12	-1.53	4.01	0.07
E1	TKW	7A	AX.89518598	1.43E+08	0.11	-1.56	3.90	0.07
E1	TKW	7A	AX.89669608	1.40E+08	0.11	-1.46	3.48	0.06
E1	TKW	7A	AX.89495549	1.46E+08	0.11	-1.50	3.78	0.06
E1	TKW	7A	AX.89542021	1.60E+08	0.11	-1.45	3.46	0.06
E1	TKW	7A	AX.89759894	1.45E+08	0.11	-1.50	3.78	0.06
E1	TKW	7A	AX.89732950	1.67E+08	0.11	-1.50	3.78	0.06
E1	TKW	7A	AX.89570644	1.71E+08	0.11	-1.50	3.78	0.06
E1	TKW	7A	AX.89309786	1.51E+08	0.11	-1.38	3.15	0.05
E1	TKW	7A	AX.89581387	1.45E+08	0.11	-1.45	3.46	0.06
E1	TKW	7A	AX.89329909	1.37E+08	0.12	-1.53	4.01	0.07
E1	TKW	7A	AX.89733362	1.76E+08	0.11	-1.50	3.63	0.06

E1	TKW	7A	AX.89645712	1.28E+08	0.15	-1.34	3.85	0.07
E1	TKW	7A	AX.89325778	2.25E+08	0.30	-0.91	3.06	0.05
E1	TKW	7A	AX.89720833	1.68E+08	0.11	-1.50	3.78	0.06
E1	TKW	7A	AX.89571275	1.02E+08	0.08	-1.65	3.57	0.06
E1	TKW	7A	AX.89439595	1.54E+08	0.11	-1.38	3.15	0.05
E1	TKW	7A	AX.89573586	1.47E+08	0.11	-1.50	3.78	0.06
E1	TKW	7A	AX.89481987	1.56E+08	0.10	-1.43	3.25	0.05
E1	TKW	7A	AX.89687568	1.74E+08	0.11	-1.34	3.10	0.05
E1	TKW	7A	AX.89426544	1.30E+08	0.10	-1.41	3.16	0.06
E1	TKW	7A	AX.89687726	1.51E+08	0.10	-1.43	3.25	0.05
E1	TKW	7A	AX.89566474	1.43E+08	0.11	-1.38	3.15	0.05
E1	TKW	7A	AX.89447647	1.50E+08	0.10	-1.43	3.25	0.05
E1	TKW	7A	AX.89442492	1.53E+08	0.11	-1.38	3.15	0.05
E1	TKW	7A	AX.89582193	1.62E+08	0.10	-1.43	3.25	0.05
E1	TKW	7A	AX.89543397	1.62E+08	0.10	-1.43	3.25	0.05
E1	TKW	7A	AX.89379986	1.67E+08	0.11	-1.50	3.78	0.06
E1	TKW	7A	AX.89729094	1.63E+08	0.11	-1.50	3.78	0.06
E1	TKW	7A	AX.89541410	1.52E+08	0.11	-1.38	3.15	0.05
E1	TKW	7A	AX.89742694	1.75E+08	0.11	-1.50	3.63	0.06
E1	TKW	7A	AX.89638333	1.49E+08	0.11	-1.50	3.78	0.06
E1	TKW	7A	AX.89551275	1.71E+08	0.11	-1.50	3.78	0.06
E1	TKW	7A	AX.89396027	1.51E+08	0.10	-1.43	3.25	0.05
E1	TKW	7A	AX.89574852	1.43E+08	0.11	-1.50	3.78	0.06
E1	TKW	7A	AX.86162558	1.67E+08	0.11	-1.50	3.78	0.06
E1	TKW	7A	AX.89441107	1.63E+08	0.10	-1.50	3.41	0.06
E1	TKW	7A	AX.89311516	1.30E+08	0.10	-1.41	3.16	0.06
E1	TKW	7A	AX.89675586	1.50E+08	0.10	-1.43	3.25	0.05
E1	TKW	7A	AX.89476774	2.32E+08	0.31	-0.93	3.17	0.05
E1	TKW	7A	AX.89319864	1.69E+08	0.11	-1.50	3.78	0.06
E1	TKW	7A	AX.89771158	1.61E+08	0.11	-1.50	3.78	0.06
E1	TKW	7A	AX.89600748	1.73E+08	0.11	-1.50	3.63	0.06
E1	TKW	7A	AX.89471888	1.76E+08	0.10	-1.37	3.00	0.05
E1	TKW	7A	AX.89322760	1.63E+08	0.11	-1.50	3.78	0.06
E1	TKW	7A	AX.89665121	1.60E+08	0.11	-1.38	3.15	0.05
E1	TKW	7A	AX.89342100	1.67E+08	0.11	-1.50	3.78	0.06
E1	TKW	7A	AX.89506645	1.48E+08	0.11	-1.50	3.78	0.06
E1	TKW	7A	AX.89451632	1.43E+08	0.10	-1.43	3.25	0.05
E1	TKW	7A	AX.89357231	1.67E+08	0.11	-1.45	3.46	0.06
E1	TKW	7A	AX.89413407	1.43E+08	0.11	-1.38	3.15	0.05
E1	TKW	7A	AX.89472440	5.73E+08	0.22	-1.01	3.08	0.05
E1	TKW	7A	AX.89768645	1.37E+08	0.12	-1.53	4.01	0.07
E1	TKW	7A	AX.89623908	1.40E+08	0.11	-1.50	3.78	0.06
E1	TKW	7A	AX.89475303	1.60E+08	0.11	-1.50	3.78	0.06
E1	TKW	7A	AX.89690031	1.63E+08	0.11	-1.50	3.78	0.06
E1	TKW	7A	AX.89553054	1.47E+08	0.35	-0.90	3.17	0.05
E1	TKW	7A	AX.89561044	1.71E+08	0.11	-1.50	3.78	0.06
E1	TKW	7A	AX.89690160	1.56E+08	0.11	-1.50	3.78	0.06
E1	TKW	7A	AX.89358256	1.43E+08	0.11	-1.50	3.78	0.06
E1	TKW	7A	AX.89515937	1.54E+08	0.10	-1.43	3.25	0.05
E1	TKW	7A	AX.89763534	1.37E+08	0.12	-1.53	4.01	0.07

E1	TKW	7A	AX.89421557	1.28E+08	0.09	-1.47	3.16	0.05
E1	TKW	7A	AX.89492125	2.44E+08	0.47	-0.97	3.93	0.07
E1	TKW	7A	AX.89569029	1.46E+08	0.10	-1.44	3.29	0.05
E1	TKW	7A	AX.89731543	1.59E+08	0.11	-1.38	3.15	0.05
E1	TKW	7A	AX.89398419	1.63E+08	0.11	-1.50	3.78	0.06
E1	TKW	7A	AX.89507660	1.45E+08	0.10	-1.52	3.61	0.06
E1	TKW	7A	AX.89343242	1.48E+08	0.10	-1.44	3.29	0.05
E1	TKW	7A	AX.89414396	1.51E+08	0.10	-1.43	3.25	0.05
E1	TKW	7A	AX.89351281	1.32E+08	0.12	-1.53	4.01	0.07
E1	TKW	7A	AX.89524399	1.66E+08	0.11	-1.50	3.78	0.06
E1	TKW	7A	AX.89430696	1.29E+08	0.11	-1.58	4.13	0.07
E1	TKW	7A	AX.89722128	2.32E+08	0.31	-0.93	3.17	0.05
E1	TKW	7A	AX.86162557	1.67E+08	0.11	-1.50	3.78	0.06
E1	TKW	7A	AX.89400029	1.71E+08	0.10	-1.51	3.57	0.06
E1	TKW	7A	AX.89525055	1.39E+08	0.10	-1.50	3.41	0.06
E1	TKW	7A	AX.89390913	1.45E+08	0.10	-1.44	3.29	0.05
E1	TKW	7A	AX.89359898	1.41E+08	0.11	-1.38	3.15	0.05
E1	TKW	7A	AX.89531975	1.38E+08	0.11	-1.48	3.69	0.07
E1	TKW	7A	AX.89314112	1.43E+08	0.10	-1.44	3.29	0.05
E1	TKW	7A	AX.89530196	9.19E+07	0.13	-1.75	5.70	0.10
E1	TKW	7A	AX.89738505	1.55E+08	0.10	-1.43	3.25	0.05
E1	TKW	7A	AX.89670058	1.43E+08	0.11	-1.50	3.78	0.06
E1	TKW	7A	AX.89634786	2.27E+08	0.30	-0.91	3.06	0.05
E1	TKW	7A	AX.89700694	1.36E+08	0.11	-1.46	3.48	0.06
E1	TKW	7A	AX.86164540	1.59E+08	0.11	-1.38	3.15	0.05
E1	TKW	7A	AX.89466610	1.74E+08	0.11	-1.34	3.10	0.05
E1	TKW	7A	AX.89431964	1.66E+08	0.10	-1.44	3.29	0.05
E1	TKW	7A	AX.89756658	2.44E+08	0.47	-0.90	3.45	0.06
E1	TKW	7A	AX.89693107	1.38E+08	0.12	-1.53	4.01	0.07
E1	TKW	7A	AX.89593216	1.54E+08	0.11	-1.38	3.15	0.05
E1	TKW	7A	AX.89652041	1.49E+08	0.11	-1.50	3.78	0.06
E1	TKW	7A	AX.89701338	1.43E+08	0.10	-1.44	3.29	0.05
E1	TKW	7A	AX.89469876	1.72E+08	0.10	-1.45	3.31	0.05
E1	TKW	7A	AX.89668508	1.50E+08	0.11	-1.50	3.78	0.06
E1	TKW	7A	AX.89676641	1.39E+08	0.10	-1.50	3.41	0.06
E1	TKW	7A	AX.89596149	1.64E+08	0.11	-1.50	3.78	0.06
E1	TKW	7A	AX.89627312	1.69E+08	0.11	-1.50	3.78	0.06
E1	TKW	7A	AX.89361534	1.48E+08	0.11	-1.50	3.78	0.06
E1	TKW	7A	AX.89627704	1.71E+08	0.11	-1.50	3.78	0.06
E1	TKW	7A	AX.89468092	1.27E+08	0.10	-1.40	3.01	0.05
E1	TKW	7A	AX.89597222	1.61E+08	0.11	-1.50	3.78	0.06
E1	TKW	7A	AX.89324648	1.67E+08	0.11	-1.50	3.78	0.06
E1	TKW	7A	AX.89488417	6.72E+08	0.17	-1.15	3.28	0.06
E1	TKW	7A	AX.89471482	1.35E+08	0.12	-1.53	4.01	0.07
E1	TKW	7A	AX.89750570	1.28E+08	0.11	-1.50	3.76	0.07
E1	TKW	7A	AX.89512065	1.53E+08	0.11	-1.38	3.15	0.05
E1	TKW	7A	AX.89732679	1.36E+08	0.12	-1.53	4.01	0.07
E1	TKW	7A	AX.89346440	1.51E+08	0.12	-1.30	3.13	0.05
E1	TKW	7A	AX.89512410	1.48E+08	0.11	-1.50	3.78	0.06
E1	TKW	7A	AX.89480466	1.43E+08	0.11	-1.36	3.20	0.05

E1	TKW	7A	AX.89546999	9.24E+07	0.13	-1.75	5.70	0.10
E1	TKW	7A	AX.89600792	1.51E+08	0.11	-1.38	3.15	0.05
E1	TKW	7A	AX.89512482	2.44E+08	0.47	-0.97	3.93	0.07
E1	TKW	7A	AX.89776997	2.31E+08	0.31	-0.93	3.17	0.05
E1	TKW	7A	AX.89339065	1.63E+08	0.11	-1.38	3.15	0.05
E1	TKW	7A	AX.89441489	1.28E+08	0.15	-1.34	3.85	0.07
E1	TKW	7A	AX.89317646	1.73E+08	0.11	-1.50	3.63	0.06
E1	TKW	7A	AX.89418195	2.33E+08	0.31	-0.93	3.17	0.05
E1	TKW	7A	AX.89325743	1.73E+08	0.11	-1.50	3.63	0.06
E1	TKW	7A	AX.89418410	1.55E+08	0.11	-1.50	3.78	0.06
E1	TKW	7A	AX.89709285	1.57E+08	0.11	-1.50	3.78	0.06
E1	TKW	7A	AX.89326050	2.32E+08	0.31	-0.93	3.17	0.05
E1	TKW	7A	AX.89590737	1.31E+08	0.11	-1.48	3.69	0.07
E1	TKW	7A	AX.89364082	1.40E+08	0.11	-1.45	3.46	0.06
E1	TKW	7A	AX.89318327	1.38E+08	0.10	-1.50	3.41	0.06
E1	TKW	7A	AX.89386658	1.46E+08	0.11	-1.38	3.15	0.05
E1	TKW	7A	AX.89513597	1.71E+08	0.11	-1.50	3.78	0.06
E1	TKW	7A	AX.89513616	9.31E+07	0.14	-1.56	4.76	0.08
E1	TKW	7A	AX.89379708	2.33E+08	0.31	-0.93	3.17	0.05
E1	TKW	7A	AX.89482819	1.69E+08	0.11	-1.50	3.78	0.06
E1	TKW	7A	AX.89761515	1.30E+08	0.11	-1.46	3.48	0.06
E1	TKW	7A	AX.89769703	1.63E+08	0.11	-1.38	3.15	0.05
E1	TKW	7A	AX.89450524	1.63E+08	0.11	-1.50	3.78	0.06
E1	TKW	7A	AX.89409809	1.56E+08	0.11	-1.50	3.78	0.06
E1	TKW	7A	AX.89567196	2.38E+08	0.39	1.00	4.00	0.07
E1	TKW	7A	AX.89311179	1.33E+08	0.11	-1.46	3.48	0.06
E1	TKW	7A	AX.89396165	1.42E+08	0.11	-1.45	3.46	0.06
E1	TKW	7A	AX.89319382	1.45E+08	0.10	-1.44	3.29	0.05
E1	TKW	7A	AX.89551667	1.27E+08	0.11	-1.36	3.18	0.05
E1	TKW	7A	AX.89443611	1.69E+08	0.11	-1.50	3.78	0.06
E1	TKW	7A	AX.89404511	1.73E+08	0.11	-1.50	3.63	0.06
E1	TKW	7A	AX.89672639	1.62E+08	0.11	-1.50	3.78	0.06
E1	TKW	7A	AX.89311463	1.28E+08	0.11	-1.45	3.44	0.06
E1	TKW	7A	AX.89420379	1.44E+08	0.11	-1.50	3.78	0.06
E1	TKW	7A	AX.89714055	1.51E+08	0.10	-1.43	3.25	0.05
E1	TKW	7A	AX.89583672	1.37E+08	0.12	-1.53	4.01	0.07
E1	TKW	7A	AX.89357153	1.29E+08	0.10	-1.50	3.41	0.06
E1	TKW	7A	AX.89342122	2.26E+08	0.30	-0.91	3.06	0.05
E1	TKW	7A	AX.89311992	1.67E+08	0.11	-1.50	3.78	0.06
E1	TKW	7A	AX.89334619	2.32E+08	0.31	-0.93	3.17	0.05
E1	TKW	7A	AX.89342760	9.19E+07	0.10	-1.60	3.81	0.06
E1	TKW	7A	AX.89576527	1.45E+08	0.11	-1.38	3.15	0.05
E1	TKW	7A	AX.89666093	1.29E+08	0.11	-1.46	3.48	0.06
E1	TKW	7A	AX.89763747	1.73E+08	0.11	-1.50	3.63	0.06
E1	TKW	7A	AX.89569119	1.64E+08	0.11	-1.50	3.78	0.06
E1	TKW	7A	AX.89553419	9.29E+07	0.10	-1.55	3.58	0.06
E1	TKW	7A	AX.89723991	1.58E+08	0.10	-1.43	3.25	0.05
E1	TKW	7A	AX.89641122	1.56E+08	0.12	-1.36	3.28	0.05
E1	TKW	7A	AX.89585251	1.51E+08	0.10	-1.43	3.25	0.05
E1	TKW	7A	AX.89321294	1.37E+08	0.12	-1.53	4.01	0.07

E1	TKW	7A	AX.89731809	1.37E+08	0.11	-1.58	4.14	0.07
E1	TKW	7A	AX.89594062	1.39E+08	0.12	-1.53	4.01	0.07
E1	TKW	7A	AX.89333393	1.50E+08	0.11	-1.50	3.78	0.06
E1	TKW	7A	AX.89617748	1.49E+08	0.11	-1.56	3.90	0.07
E1	TKW	7A	AX.89430418	1.67E+08	0.11	-1.38	3.15	0.05
E1	TKW	7A	AX.89682294	1.63E+08	0.11	-1.38	3.15	0.05
E1	TKW	7A	AX.89577336	1.57E+08	0.11	-1.50	3.78	0.06
E1	TKW	7A	AX.89773333	1.38E+08	0.11	-1.48	3.69	0.07
E1	TKW	7A	AX.89724466	2.03E+08	0.10	-1.45	3.31	0.05
E1	TKW	7A	AX.89390586	1.42E+08	0.11	-1.45	3.46	0.06
E1	TKW	7A	AX.86162561	1.67E+08	0.11	-1.50	3.78	0.06
E1	TKW	7A	AX.89486274	1.69E+08	0.11	-1.50	3.78	0.06
E1	TKW	7A	AX.89453590	1.48E+08	0.10	-1.44	3.29	0.05
E1	TKW	7A	AX.89539683	1.02E+08	0.09	-1.57	3.39	0.06
E1	TKW	7A	AX.89334123	9.22E+07	0.13	-1.75	5.70	0.10
E1	TKW	7A	AX.89578150	1.63E+08	0.11	-1.38	3.15	0.05
E1	TKW	7A	AX.89376150	1.49E+08	0.11	-1.50	3.78	0.06
E1	TKW	7A	AX.89368776	1.54E+08	0.11	-1.50	3.78	0.06
E1	TKW	7A	AX.89407934	1.51E+08	0.11	-1.50	3.78	0.06
E1	TKW	7A	AX.89368845	1.65E+08	0.11	-1.44	3.51	0.06
E1	TKW	7A	AX.89421069	1.50E+08	0.11	-1.38	3.15	0.05
E1	TKW	7A	AX.89654496	1.66E+08	0.10	-1.52	3.61	0.06
E1	TKW	7A	AX.89421091	1.38E+08	0.10	-1.52	3.61	0.06
E1	TKW	7A	AX.89540208	1.48E+08	0.11	-1.38	3.15	0.05
E1	TKW	7A	AX.89368896	2.29E+08	0.31	-0.93	3.17	0.05
E1	TKW	7A	AX.89323490	1.45E+08	0.11	-1.45	3.46	0.06
E1	TKW	7A	AX.89717943	1.49E+08	0.11	-1.50	3.78	0.06
E1	TKW	7A	AX.89392397	2.11E+08	0.10	-1.43	3.25	0.05
E1	TKW	7A	AX.89369333	6.48E+08	0.22	-1.17	3.97	0.07
E1	TKW	7A	AX.89749362	1.64E+08	0.10	-1.63	4.07	0.07
E1	TKW	7A	AX.89330404	1.39E+08	0.12	-1.53	4.01	0.07
E1	TKW	7A	AX.89315748	1.51E+08	0.11	-1.38	3.15	0.05
E1	TKW	7A	AX.89487592	1.41E+08	0.11	-1.38	3.15	0.05
E1	TKW	7A	AX.89775586	1.63E+08	0.11	-1.38	3.15	0.05
E1	TKW	7A	AX.89454763	1.57E+08	0.11	-1.50	3.78	0.06
E1	TKW	7A	AX.89526730	1.36E+08	0.11	-1.54	3.80	0.07
E1	TKW	7A	AX.89470303	1.68E+08	0.10	-1.51	3.57	0.06
E1	TKW	7A	AX.89416760	1.63E+08	0.11	-1.50	3.78	0.06
E1	TKW	7A	AX.89726582	1.38E+08	0.09	-1.45	3.09	0.05
E1	TKW	7A	AX.89324138	1.73E+08	0.11	-1.50	3.63	0.06
E1	TKW	7A	AX.89644225	1.50E+08	0.10	-1.43	3.25	0.05
E1	TKW	7A	AX.89440469	1.66E+08	0.11	-1.50	3.78	0.06
E1	TKW	7A	AX.89495101	1.58E+08	0.10	-1.43	3.25	0.05
E1	TKW	7A	AX.89588814	1.74E+08	0.10	-1.37	3.00	0.05
E1	TKW	7A	AX.89316650	2.26E+08	0.30	-0.91	3.06	0.05
E1	TKW	7A	AX.89488132	1.65E+08	0.11	-1.50	3.78	0.06
E1	TKW	7A	AX.89519246	1.45E+08	0.10	-1.44	3.29	0.05
E1	TKW	7A	AX.89702672	1.66E+08	0.11	-1.50	3.78	0.06
E1	TKW	7A	AX.89324453	1.36E+08	0.12	-1.53	4.01	0.07
E1	TKW	7A	AX.89433471	1.38E+08	0.11	-1.48	3.69	0.07

E1	TKW	7A	AX.89402089	1.65E+08	0.11	-1.50	3.78	0.06
E1	TKW	7A	AX.89653490	1.62E+08	0.10	-1.43	3.25	0.05
E1	TKW	7A	AX.89402098	1.57E+08	0.10	-1.43	3.25	0.05
E1	TKW	7A	AX.89448757	1.63E+08	0.09	-1.45	3.09	0.05
E1	TKW	7A	AX.89527502	1.30E+08	0.12	-1.53	4.01	0.07
E1	TKW	7A	AX.89373097	1.55E+08	0.11	-1.56	3.90	0.07
E1	TKW	7A	AX.89519648	1.67E+08	0.11	-1.57	3.94	0.07
E1	TKW	7A	AX.89438626	1.37E+08	0.12	-1.53	4.01	0.07
E1	TKW	7A	AX.89740607	1.67E+08	0.11	-1.45	3.46	0.06
E1	TKW	7A	AX.89750928	1.76E+08	0.11	-1.50	3.63	0.06
E1	TKW	7A	AX.89678230	1.48E+08	0.11	-1.50	3.78	0.06
E1	TKW	7A	AX.89549723	1.44E+08	0.11	-1.50	3.78	0.06
E1	TKW	7A	AX.89534932	1.63E+08	0.11	-1.50	3.78	0.06
E1	TKW	7A	AX.89423557	1.37E+08	0.10	-1.50	3.41	0.06
E1	TKW	7A	AX.89662317	1.54E+08	0.11	-1.38	3.15	0.05
E1	TKW	7A	AX.89637385	1.50E+08	0.10	-1.43	3.25	0.05
E1	TKW	7A	AX.89566041	1.43E+08	0.10	-1.43	3.25	0.05
E1	TKW	7A	AX.89607110	1.30E+08	0.11	-1.46	3.48	0.06
E1	TKW	7A	AX.89655050	2.45E+08	0.47	-0.90	3.45	0.06
E1	TKW	7A	AX.89768981	6.72E+08	0.16	-1.15	3.14	0.05
E1	TKW	7A	AX.89637754	1.63E+08	0.11	-1.38	3.15	0.05
E1	TKW	7A	AX.89395282	1.59E+08	0.11	-1.38	3.15	0.05
E1	TKW	7A	AX.89528953	1.76E+08	0.11	-1.50	3.63	0.06
E1	TKW	7A	AX.89310681	1.57E+08	0.11	-1.50	3.78	0.06
E1	TKW	7A	AX.89464561	9.31E+07	0.14	-1.56	4.76	0.08
E1	TKW	7A	AX.89679312	1.71E+08	0.11	-1.45	3.46	0.06
E1	TKW	7A	AX.89412224	1.46E+08	0.10	-1.44	3.29	0.05
E1	TKW	7A	AX.89403578	1.43E+08	0.11	-1.45	3.46	0.06
E1	TKW	7A	AX.89713016	1.34E+08	0.11	-1.46	3.48	0.06
E1	TKW	7A	AX.89476104	1.48E+08	0.11	-1.50	3.78	0.06
E1	TKW	7A	AX.89567073	1.48E+08	0.11	-1.50	3.78	0.06
E1	TKW	7A	AX.89574663	2.25E+08	0.30	-0.91	3.06	0.05
E1	TKW	7A	AX.89356403	1.62E+08	0.11	-1.50	3.78	0.06
E1	TKW	7A	AX.89529655	1.48E+08	0.11	-1.50	3.78	0.06
E1	TKW	7A	AX.89343793	1.57E+08	0.11	-1.50	3.78	0.06
E1	TKW	7A	AX.89559659	1.48E+08	0.10	-1.44	3.29	0.05
E1	TKW	7A	AX.89559664	1.51E+08	0.10	-1.43	3.25	0.05
E1	TKW	7A	AX.89483519	2.31E+08	0.31	-0.93	3.17	0.05
E1	TKW	7A	AX.89722248	1.30E+08	0.11	-1.48	3.69	0.07
E1	TKW	7A	AX.89490773	1.39E+08	0.12	-1.53	4.01	0.07
E1	TKW	7A	AX.89560011	1.45E+08	0.10	-1.44	3.29	0.05
E1	TKW	7A	AX.89506095	1.39E+08	0.12	-1.53	4.01	0.07
E1	TKW	7A	AX.89474306	1.43E+08	0.11	-1.38	3.15	0.05
E1	TKW	7A	AX.89738283	1.65E+08	0.10	-1.58	3.87	0.07
E1	TKW	7A	AX.89341815	1.34E+08	0.12	-1.53	4.01	0.07
E1	TKW	7A	AX.89705794	1.63E+08	0.11	-1.50	3.78	0.06
E1	TKW	7A	AX.89680684	9.31E+07	0.09	-1.52	3.34	0.06
E1	TKW	7A	AX.89583849	2.44E+08	0.47	-0.97	3.93	0.07
E1	TKW	7A	AX.89552552	1.63E+08	0.11	-1.38	3.15	0.05
E1	TKW	7A	AX.89537443	2.28E+08	0.31	-0.93	3.17	0.05

E1	TKW	7A	AX.89373636	1.71E+08	0.11	-1.50	3.78	0.06
E1	TKW	7A	AX.89530468	1.66E+08	0.11	-1.38	3.15	0.05
E1	TKW	7A	AX.89656982	1.54E+08	0.10	-1.43	3.25	0.05
E1	TKW	7A	AX.89312333	1.56E+08	0.11	-1.50	3.78	0.06
E1	TKW	7A	AX.89374014	1.43E+08	0.11	-1.50	3.78	0.06
E1	TKW	7A	AX.89537763	1.62E+08	0.11	-1.50	3.78	0.06
E1	TKW	7A	AX.89609075	1.52E+08	0.11	-1.38	3.15	0.05
E1	TKW	7A	AX.89421438	1.47E+08	0.10	-1.44	3.29	0.05
E1	TKW	7A	AX.89515736	1.44E+08	0.11	-1.50	3.78	0.06
E1	TKW	7A	AX.89657558	1.46E+08	0.11	-1.38	3.15	0.05
E1	TKW	7A	AX.89445214	1.62E+08	0.10	-1.43	3.25	0.05
E1	TKW	7A	AX.89445248	1.41E+08	0.11	-1.38	3.15	0.05
E1	TKW	7A	AX.89561391	1.49E+08	0.11	-1.50	3.78	0.06
E1	TKW	7A	AX.89611753	1.60E+08	0.10	-1.43	3.25	0.05
E1	TKW	7A	AX.89518568	1.50E+08	0.10	-1.43	3.25	0.05
E1	TKW	7A	AX.89487600	1.33E+08	0.11	-1.46	3.48	0.06
E1	TKW	7A	AX.89437582	1.66E+08	0.11	-1.50	3.78	0.06
E1	TKW	7A	AX.89389944	1.57E+08	0.11	-1.50	3.78	0.06
E1	TKW	7A	AX.89593768	2.39E+08	0.32	-0.96	3.40	0.06
E1	TKW	7A	AX.89459716	1.31E+08	0.10	-1.52	3.60	0.06
E1	TKW	7A	AX.89569389	1.36E+08	0.12	-1.53	4.01	0.07
E1	TKW	7A	AX.89602055	1.44E+08	0.10	-1.52	3.61	0.06
E1	TKW	7A	AX.89585629	1.39E+08	0.11	-1.57	3.94	0.07
E1	TKW	7A	AX.89755224	1.56E+08	0.10	-1.43	3.25	0.05
E1	TKW	7A	AX.89367073	1.57E+08	0.11	-1.50	3.78	0.06
E1	TKW	7A	AX.89485900	1.70E+08	0.11	-1.45	3.46	0.06
E1	TKW	7A	AX.89739998	1.45E+08	0.10	-1.44	3.29	0.05
E1	TKW	7A	AX.89445892	1.38E+08	0.11	-1.48	3.69	0.07
E1	TKW	7A	AX.89691373	1.69E+08	0.11	-1.50	3.78	0.06
E1	TKW	7A	AX.89524542	1.35E+08	0.12	-1.53	4.01	0.07
E1	TKW	7A	AX.89546700	1.57E+08	0.11	-1.38	3.15	0.05
E1	TKW	7A	AX.89344105	1.55E+08	0.11	-1.45	3.46	0.06
E1	TKW	7A	AX.89554508	1.43E+08	0.11	-1.50	3.78	0.06
E1	TKW	7A	AX.89689052	1.29E+08	0.11	-1.46	3.48	0.06
E1	TKW	7A	AX.89468815	1.39E+08	0.10	-1.50	3.41	0.06
E1	TKW	7A	AX.89344288	1.28E+08	0.10	-1.60	3.82	0.06
E1	TKW	7A	AX.89675710	1.52E+08	0.16	-1.32	3.98	0.07
E1	TKW	7A	AX.89423328	2.11E+08	0.10	-1.43	3.25	0.05
E1	TKW	7A	AX.89634952	2.32E+08	0.31	-0.93	3.17	0.05
E1	TKW	7A	AX.89709135	2.32E+08	0.31	-0.93	3.17	0.05
E1	TKW	7A	AX.89432005	1.70E+08	0.11	-1.50	3.78	0.06
E1	TKW	7A	AX.89447098	1.67E+08	0.10	-1.63	4.07	0.07
E1	TKW	7A	AX.89323262	1.76E+08	0.11	-1.50	3.63	0.06
E1	TKW	7A	AX.89323535	1.45E+08	0.10	-1.52	3.61	0.06
E1	TKW	7A	AX.89540761	1.73E+08	0.12	-1.33	3.23	0.05
E1	TKW	7A	AX.89532761	2.30E+08	0.31	-0.93	3.17	0.05
E1	TKW	7A	AX.89532911	2.03E+08	0.10	-1.52	3.47	0.06
E1	TKW	7A	AX.89741785	1.76E+08	0.10	-1.37	3.00	0.05
E1	TKW	7A	AX.89371928	1.73E+08	0.12	-1.40	3.44	0.06
E1	TKW	7A	AX.89604136	2.11E+08	0.10	-1.43	3.25	0.05

E1	TKW	7A	AX.89548350	1.67E+08	0.11	-1.38	3.15	0.05
E1	TKW	7A	AX.89409314	1.48E+08	0.35	-0.90	3.17	0.05
E1	TKW	7A	AX.89636079	9.19E+07	0.15	-1.61	5.29	0.09
E1	TKW	7A	AX.89718731	1.49E+08	0.11	-1.50	3.78	0.06
E1	TKW	7A	AX.89684994	1.76E+08	0.10	-1.37	3.00	0.05
E1	TKW	7A	AX.89660543	1.48E+08	0.10	-1.44	3.29	0.05
E1	TKW	7A	AX.89333364	1.51E+08	0.11	-1.38	3.15	0.05
E1	TKW	7A	AX.89775885	1.40E+08	0.11	-1.50	3.78	0.06
E1	TKW	7A	AX.89694097	1.51E+08	0.11	-1.50	3.78	0.06
E1	TKW	7A	AX.89502947	1.97E+08	0.10	-1.45	3.31	0.05
E1	TKW	7A	AX.89417340	1.45E+08	0.11	-1.45	3.46	0.06
E1	TKW	7A	AX.89661146	1.62E+08	0.10	-1.43	3.25	0.05
E1	TKW	7A	AX.89527321	1.93E+08	0.45	0.92	3.55	0.06
E1	TKW	7A	AX.89661403	2.24E+08	0.30	-0.91	3.06	0.05
E1	TKW	7A	AX.89750454	1.76E+08	0.11	-1.50	3.63	0.06
E1	TKW	7A	AX.89727290	1.54E+08	0.11	-1.50	3.78	0.06
E1	TKW	7A	AX.89433593	1.55E+08	0.10	-1.43	3.25	0.05
E1	TKW	7A	AX.89620706	1.60E+08	0.11	-1.45	3.46	0.06
E1	TKW	7A	AX.89503637	1.64E+08	0.14	-1.35	3.79	0.06
E1	TKW	7A	AX.89743697	1.40E+08	0.11	-1.50	3.78	0.06
E1	TKW	7A	AX.89686253	1.66E+08	0.10	-1.39	3.01	0.05
E1	TKW	7A	AX.89362870	1.66E+08	0.11	-1.38	3.15	0.05
E1	TKW	7A	AX.89346719	1.42E+08	0.11	-1.38	3.15	0.05
E1	TKW	7A	AX.89695562	2.25E+08	0.30	-0.91	3.06	0.05
E1	TKW	7A	AX.89703421	1.76E+08	0.11	-1.50	3.63	0.06
E1	TKW	7A	AX.89425836	2.11E+08	0.10	-1.43	3.25	0.05
E1	TKW	7A	AX.89314613	9.66E+07	0.09	-1.57	3.39	0.06
E1	TKW	7A	AX.89777419	1.59E+08	0.11	-1.50	3.78	0.06
E1	TKW	7A	AX.89573384	1.67E+08	0.10	-1.63	4.07	0.07
E1	TKW	7A	AX.89481678	1.57E+08	0.10	-1.43	3.25	0.05
E1	TKW	7A	AX.89728136	1.49E+08	0.11	-1.50	3.78	0.06
E1	TKW	7A	AX.89550173	2.28E+08	0.30	-0.91	3.06	0.05
E1	TKW	7A	AX.89472576	2.00E+08	0.09	-1.58	3.59	0.06
E1	TKW	7A	AX.89379269	1.46E+08	0.11	-1.50	3.78	0.06
E1	TKW	7A	AX.89720892	1.39E+08	0.10	-1.50	3.41	0.06
E1	TKW	7A	AX.89355529	1.62E+08	0.10	-1.43	3.25	0.05
E1	TKW	7A	AX.89566441	1.63E+08	0.11	-1.38	3.15	0.05
E1	GPM2	7A	AX.89643578	1.99E+08	0.31	665.20	3.33	0.06
E1	TKW	7A	AX.89369485	1.63E+08	0.11	-1.50	3.78	0.06
E1	TKW	7A	AX.89582172	2.27E+08	0.30	-0.91	3.06	0.05
E1	TKW	7A	AX.89663097	1.72E+08	0.10	-1.45	3.31	0.05
E1	TKW	7A	AX.89744801	1.56E+08	0.10	-1.43	3.25	0.05
E1	TKW	7A	AX.89591031	1.69E+08	0.11	-1.45	3.46	0.06
E1	TKW	7A	AX.89559099	1.72E+08	0.11	-1.50	3.63	0.06
E1	TKW	7A	AX.89457362	1.51E+08	0.10	-1.43	3.25	0.05
E1	TKW	7A	AX.89569564	1.51E+08	0.11	-1.38	3.15	0.05
E1	TKW	7A	AX.89607869	1.51E+08	0.11	-1.36	3.20	0.05
E1	TKW	7A	AX.89326653	1.74E+08	0.11	-1.34	3.10	0.05
E1	TKW	7A	AX.89655516	2.03E+08	0.10	-1.52	3.47	0.06
E1	TKW	7A	AX.89745264	1.61E+08	0.10	-1.43	3.25	0.05

E1	TKW	7A	AX.89630617	1.33E+08	0.11	-1.41	3.37	0.06
E1	TKW	7A	AX.89650308	1.75E+08	0.11	-1.34	3.10	0.05
E1	TKW	7A	AX.89575195	1.59E+08	0.10	-1.43	3.25	0.05
E1	TKW	7A	AX.89664370	1.55E+08	0.10	-1.43	3.25	0.05
E1	TKW	7A	AX.89474147	1.67E+08	0.11	-1.45	3.46	0.06
E1	TKW	7A	AX.89544126	1.38E+08	0.12	-1.53	4.01	0.07
E1	TKW	7A	AX.89745832	1.35E+08	0.10	-1.44	3.29	0.05
E1	TKW	7A	AX.89373050	1.55E+08	0.11	-1.50	3.78	0.06
E1	TKW	7A	AX.89443993	1.66E+08	0.11	-1.50	3.78	0.06
E1	TKW	7A	AX.89639374	1.63E+08	0.11	-1.50	3.78	0.06
E1	TKW	7A	AX.89428744	1.45E+08	0.11	-1.50	3.78	0.06
E1	TKW	7A	AX.89664945	1.63E+08	0.11	-1.50	3.78	0.06
E1	TKW	7A	AX.89491168	1.67E+08	0.10	-1.63	4.07	0.07
E1	TKW	7A	AX.89600921	2.44E+08	0.47	-0.97	3.93	0.07
E1	TKW	7A	AX.89349756	6.72E+08	0.16	-1.15	3.14	0.05
E1	TKW	7A	AX.89397250	2.11E+08	0.10	-1.43	3.25	0.05
E1	TKW	7A	AX.89717422	1.47E+08	0.10	-1.37	3.03	0.05
E1	TKW	7A	AX.89327748	1.43E+08	0.11	-1.56	3.90	0.07
E1	TKW	7A	AX.89563839	6.48E+08	0.22	-1.17	3.97	0.07
E1	TKW	7A	AX.89689917	1.27E+08	0.10	-1.40	3.01	0.05
E1	TKW	7A	AX.89545256	1.68E+08	0.11	-1.50	3.78	0.06
E1	TKW	7A	AX.89452043	1.69E+08	0.11	-1.50	3.78	0.06
E1	TKW	7A	AX.89563909	1.30E+08	0.11	-1.48	3.69	0.07
E1	TKW	7A	AX.89568895	1.50E+08	0.11	-1.50	3.78	0.06
E1	TKW	7A	AX.89763564	1.66E+08	0.10	-1.44	3.29	0.05
E1	TKW	7A	AX.89513578	1.43E+08	0.11	-1.56	3.90	0.07
E1	TKW	7A	AX.89511123	1.39E+08	0.11	-1.57	3.94	0.07
E1	TKW	7A	AX.89406163	1.43E+08	0.11	-1.50	3.78	0.06
E1	TKW	7A	AX.89627562	1.39E+08	0.12	-1.53	4.01	0.07
E1	TKW	7A	AX.89475948	1.39E+08	0.12	-1.53	4.01	0.07
E1	TKW	7A	AX.89335527	1.67E+08	0.10	-1.63	4.07	0.07
E1	TKW	7A	AX.89707225	1.38E+08	0.11	-1.41	3.37	0.06
E1	TKW	7A	AX.89459897	1.57E+08	0.11	-1.50	3.78	0.06
E1	TKW	7A	AX.89369888	1.51E+08	0.11	-1.38	3.15	0.05
E1	TKW	7A	AX.89499809	9.31E+07	0.14	-1.56	4.76	0.08
E1	TKW	7A	AX.89524295	1.63E+08	0.11	-1.38	3.15	0.05
E1	TKW	7A	AX.89669263	1.50E+08	0.11	-1.56	3.90	0.07
E1	TKW	7A	AX.89508516	1.71E+08	0.11	-1.50	3.78	0.06
E1	TKW	7A	AX.89577598	1.48E+08	0.12	-1.35	3.25	0.05
E1	TKW	7A	AX.89551804	1.68E+08	0.11	-1.50	3.78	0.06
E1	TKW	7A	AX.89367738	1.62E+08	0.11	-1.50	3.78	0.06
E1	TKW	7A	AX.89675595	1.95E+08	0.10	-1.55	3.75	0.06
E1	TKW	7A	AX.89367776	1.30E+08	0.11	-1.41	3.37	0.06
E1	TKW	7A	AX.89480189	1.45E+08	0.10	-1.44	3.29	0.05
E1	TKW	7A	AX.89740641	1.41E+08	0.11	-1.50	3.78	0.06
E1	TKW	7A	AX.89645283	6.48E+08	0.22	-1.17	3.97	0.07
E1	TKW	7A	AX.89322960	1.48E+08	0.11	-1.38	3.15	0.05
E1	TKW	7A	AX.89368878	1.32E+08	0.11	-1.46	3.48	0.06
E1	TKW	7A	AX.89418127	1.43E+08	0.11	-1.50	3.78	0.06
E1	TKW	7A	AX.89659553	2.44E+08	0.47	-0.97	3.93	0.07

E1	TKW	7A	AX.89469383	1.73E+08	0.11	-1.50	3.63	0.06
E1	TKW	7A	AX.89408537	1.45E+08	0.07	-1.79	3.72	0.06
E1	TKW	7A	AX.89494213	1.64E+08	0.11	-1.50	3.78	0.06
E1	TKW	7A	AX.89571307	1.76E+08	0.10	-1.37	3.00	0.05
E1	TKW	7A	AX.89518189	1.62E+08	0.10	-1.43	3.25	0.05
E1	TKW	7A	AX.89423849	1.35E+08	0.11	-1.59	4.03	0.07
E1	TKW	7A	AX.89408717	2.11E+08	0.11	-1.50	3.78	0.06
E1	TKW	7A	AX.89696524	1.67E+08	0.11	-1.50	3.78	0.06
E1	TKW	7A	AX.89472880	1.37E+08	0.12	-1.53	4.01	0.07
E1	TKW	7A	AX.89710011	1.30E+08	0.11	-1.46	3.48	0.06
E1	TKW	7A	AX.89326328	1.43E+08	0.11	-1.50	3.78	0.06
E1	TKW	7A	AX.89513655	1.63E+08	0.11	-1.38	3.15	0.05
E1	TKW	7A	AX.89377277	9.29E+07	0.07	-1.90	4.14	0.07
E1	TKW	7A	AX.89439967	1.49E+08	0.10	-1.43	3.25	0.05
E1	TKW	7A	AX.89596485	1.46E+08	0.10	-1.44	3.29	0.05
E1	TKW	7A	AX.89340879	1.48E+08	0.10	-1.44	3.29	0.05
E1	TKW	7A	AX.89440500	1.74E+08	0.11	-1.50	3.63	0.06
E1	TKW	7A	AX.89361870	2.03E+08	0.15	-1.58	5.14	0.09
E1	TKW	7A	AX.89572652	1.62E+08	0.11	-1.38	3.15	0.05
E1	TKW	7A	AX.89737723	1.55E+08	0.12	-1.42	3.65	0.06
E1	TKW	7A	AX.89653667	9.31E+07	0.14	-1.56	4.76	0.08
E1	TKW	7A	AX.89490858	1.35E+08	0.12	-1.53	4.01	0.07
E1	TKW	7A	AX.86184384	2.27E+08	0.30	-0.91	3.06	0.05
E1	TKW	7A	AX.89720292	1.55E+08	0.11	-1.38	3.15	0.05
E1	TKW	7A	AX.89534609	1.49E+08	0.11	-1.50	3.78	0.06
E1	TKW	7A	AX.89554802	1.45E+08	0.11	-1.51	3.68	0.06
E1	TKW	7A	AX.89648084	1.63E+08	0.11	-1.38	3.15	0.05
E1	TKW	7A	AX.89746290	1.62E+08	0.11	-1.38	3.15	0.05
E1	TKW	7A	AX.89441396	1.67E+08	0.11	-1.50	3.78	0.06
E1	TKW	7A	AX.89327554	1.72E+06	0.25	-1.02	3.38	0.06
E1	TKW	7A	AX.89628948	2.44E+08	0.48	-0.95	3.80	0.06
E1	TKW	7A	AX.89698185	1.49E+08	0.11	-1.50	3.78	0.06
E1	TKW	7A	AX.89733438	2.33E+08	0.31	-0.91	3.09	0.05
E1	TKW	7A	AX.89629771	1.37E+08	0.12	-1.53	4.01	0.07
E1	TKW	7A	AX.89363891	1.73E+08	0.10	-1.41	3.17	0.05
E1	TKW	7A	AX.89768978	1.74E+08	0.11	-1.50	3.63	0.06
E1	TKW	7A	AX.89763557	1.32E+08	0.11	-1.48	3.69	0.07
E1	TKW	7A	AX.89555800	1.35E+08	0.11	-1.41	3.37	0.06
E1	TKW	7A	AX.89573739	1.67E+08	0.11	-1.38	3.15	0.05
E1	TKW	7A	AX.89518535	1.46E+08	0.10	-1.44	3.29	0.05
E1	TKW	7A	AX.89582185	1.30E+08	0.11	-1.45	3.46	0.06
E1	TKW	7A	AX.89347924	1.49E+08	0.11	-1.56	3.90	0.07
E1	TKW	7A	AX.89558931	2.44E+08	0.47	-0.97	3.93	0.07
E1	TKW	7A	AX.89505008	2.26E+08	0.30	-0.91	3.06	0.05
E1	TKW	7A	AX.89744846	1.52E+08	0.11	-1.38	3.15	0.05
E1	TKW	7A	AX.89355942	1.43E+08	0.11	-1.50	3.78	0.06
E1	TKW	7A	AX.89574177	1.63E+08	0.11	-1.50	3.78	0.06
E1	TKW	7A	AX.89582619	1.52E+08	0.11	-1.38	3.15	0.05
E1	TKW	7A	AX.89335559	1.39E+08	0.10	-1.50	3.41	0.06
E1	TKW	7A	AX.89382013	1.36E+08	0.12	-1.53	4.01	0.07

E1	TKW	7A	AX.89505562	1.63E+08	0.11	-1.50	3.78	0.06
E1	TKW	7A	AX.89641393	1.72E+08	0.11	-1.50	3.78	0.06
E1	TKW	7A	AX.89729445	1.71E+08	0.11	-1.50	3.78	0.06
E1	TKW	7A	AX.89414772	1.59E+08	0.11	-1.38	3.15	0.05
E1	TKW	7A	AX.89727583	2.25E+08	0.30	-0.91	3.06	0.05
E1	TKW	7A	AX.89729680	1.68E+08	0.10	-1.63	4.07	0.07
E1	TKW	7A	AX.89705453	1.56E+08	0.10	-1.43	3.25	0.05
E1	TKW	7A	AX.89697740	1.44E+08	0.11	-1.50	3.78	0.06
E1	TKW	7A	AX.89554236	1.48E+08	0.11	-1.57	3.94	0.07
E1	TKW	7A	AX.89680047	1.35E+08	0.10	-1.44	3.29	0.05
E1	TKW	7A	AX.89524884	1.76E+08	0.11	-1.50	3.63	0.06
E1	TKW	7A	AX.89506226	1.36E+08	0.11	-1.57	3.94	0.07
E1	TKW	7A	AX.89536720	1.49E+08	0.11	-1.50	3.78	0.06
E1	TKW	7A	AX.89466043	1.38E+08	0.11	-1.48	3.69	0.07
E1	TKW	7A	AX.89639432	1.74E+08	0.11	-1.50	3.63	0.06
E1	TKW	7A	AX.89425258	1.75E+08	0.10	-1.45	3.31	0.05
E1	TKW	7A	AX.89735671	1.49E+08	0.11	-1.50	3.78	0.06
E1	TKW	7A	AX.89738580	1.48E+08	0.11	-1.38	3.15	0.05
E1	TKW	7A	AX.89417939	1.33E+08	0.11	-1.46	3.48	0.06
E1	TKW	7A	AX.89725327	1.38E+08	0.12	-1.53	4.01	0.07
E1	TKW	7A	AX.89352685	1.45E+08	0.11	-1.50	3.78	0.06
E1	TKW	7A	AX.89394873	1.63E+08	0.11	-1.44	3.41	0.06
E1	TKW	7A	AX.89537751	1.63E+08	0.11	-1.50	3.78	0.06
E1	TKW	7A	AX.89439172	1.31E+08	0.12	-1.53	4.01	0.07
E1	TKW	7A	AX.89701002	1.45E+08	0.10	-1.44	3.29	0.05
E1	TKW	7A	AX.89459148	1.63E+08	0.11	-1.38	3.15	0.05
E1	TKW	7A	AX.89401078	1.33E+08	0.12	-1.53	4.01	0.07
E1	TKW	7A	AX.89601469	1.02E+08	0.09	-1.57	3.39	0.06
E1	TKW	7A	AX.89392172	1.49E+08	0.11	-1.50	3.78	0.06
E1	TKW	7A	AX.89510504	1.50E+08	0.10	-1.43	3.25	0.05
E1	TKW	7A	AX.89345107	1.43E+08	0.10	-1.43	3.25	0.05
E1	TKW	7A	AX.89578138	1.48E+08	0.10	-1.44	3.29	0.05
E1	TKW	7A	AX.89743153	9.25E+07	0.13	-1.75	5.70	0.10
E1	TKW	7A	AX.89461963	2.26E+08	0.30	-0.91	3.06	0.05
E1	TKW	7A	AX.89369711	1.66E+08	0.11	-1.50	3.78	0.06
E1	TKW	7A	AX.89541396	1.55E+08	0.10	-1.43	3.25	0.05
E1	TKW	7A	AX.89452691	1.44E+08	0.11	-1.50	3.78	0.06
E1	TKW	7A	AX.89460095	1.73E+08	0.11	-1.50	3.63	0.06
E1	TKW	7A	AX.89743151	1.76E+08	0.11	-1.50	3.63	0.06
E1	TKW	7A	AX.89757701	9.76E+07	0.12	-1.68	4.90	0.09
E1	TKW	7A	AX.89661348	1.58E+08	0.14	-1.45	4.18	0.07
E1	TKW	7A	AX.89650913	2.32E+08	0.31	-0.93	3.17	0.05
E1	TKW	7A	AX.89400115	9.19E+07	0.12	-1.75	5.09	0.09
E1	TKW	7A	AX.89560296	2.12E+08	0.11	-1.38	3.15	0.05
E1	TKW	7A	AX.89509226	1.45E+08	0.11	-1.38	3.15	0.05
E1	TKW	7A	AX.89725084	1.68E+08	0.11	-1.56	3.90	0.07
E1	TKW	7A	AX.86163778	1.41E+08	0.10	-1.43	3.25	0.05
E1	TKW	7A	AX.89418697	1.59E+08	0.10	-1.43	3.25	0.05
E1	TKW	7A	AX.89446661	1.28E+08	0.09	-1.47	3.16	0.05
E1	TKW	7A	AX.89325529	1.66E+08	0.11	-1.38	3.15	0.05

E1	TKW	7A	AX.89493915	1.76E+08	0.10	-1.37	3.00	0.05
E1	TKW	7A	AX.89711943	2.44E+08	0.47	-0.96	3.83	0.07
E1	TKW	7A	AX.89456715	1.38E+08	0.10	-1.50	3.41	0.06
E1	TKW	7A	AX.89510314	2.32E+08	0.31	-0.93	3.17	0.05
E1	TKW	7A	AX.89701284	1.38E+08	0.10	-1.50	3.41	0.06
E1	TKW	7A	AX.89687304	1.69E+08	0.11	-1.50	3.78	0.06
E1	TKW	7A	AX.89515695	1.76E+08	0.11	-1.50	3.63	0.06
E1	TKW	7A	AX.89777832	9.29E+07	0.13	-1.75	5.70	0.10
E1	TKW	7A	AX.89392694	1.39E+08	0.11	-1.41	3.37	0.06
E1	TKW	7A	AX.89352236	1.28E+08	0.11	-1.50	3.76	0.07
E1	TKW	7A	AX.89533208	1.63E+08	0.11	-1.50	3.78	0.06
E1	TKW	7A	AX.89566598	1.30E+08	0.10	-1.41	3.16	0.06
E1	TKW	7A	AX.89668798	2.38E+08	0.33	-0.92	3.20	0.05
E1	TKW	7A	AX.89403650	1.39E+08	0.11	-1.55	3.87	0.07
E1	TKW	7A	AX.89644044	9.19E+07	0.07	-1.86	3.79	0.06
E1	TKW	7A	AX.89526781	1.62E+08	0.11	-1.38	3.15	0.05
E1	TKW	7A	AX.89440021	1.39E+08	0.11	-1.58	4.13	0.07
E1	TKW	7A	AX.89353567	1.73E+08	0.12	-1.38	3.38	0.06
E1	TKW	7A	AX.89507903	1.68E+08	0.10	-1.63	4.07	0.07
E1	TKW	7A	AX.89747648	1.67E+08	0.10	-1.63	4.07	0.07
E1	TKW	7A	AX.89343295	1.54E+08	0.11	-1.38	3.15	0.05
E1	TKW	7A	AX.89658380	1.48E+08	0.11	-1.38	3.15	0.05
E1	TKW	7A	AX.89644324	1.62E+08	0.10	-1.43	3.25	0.05
E1	TKW	7A	AX.89638456	1.48E+08	0.11	-1.50	3.78	0.06
E1	TKW	7A	AX.89456317	1.29E+08	0.10	-1.63	4.07	0.07
E1	TKW	7A	AX.89750957	1.59E+08	0.11	-1.38	3.15	0.05
E1	TKW	7A	AX.89518578	1.63E+08	0.11	-1.38	3.15	0.05
E1	TKW	7A	AX.89383267	1.46E+08	0.11	-1.38	3.15	0.05
E1	TKW	7A	AX.89729776	1.38E+08	0.11	-1.48	3.69	0.07
E1	TKW	7A	AX.89385563	1.40E+08	0.12	-1.53	4.01	0.07
E1	TKW	7A	AX.89559022	1.71E+08	0.11	-1.50	3.78	0.06
E1	TKW	7A	AX.89676558	1.48E+08	0.11	-1.45	3.46	0.06
E1	TKW	7A	AX.89565485	1.43E+08	0.11	-1.38	3.15	0.05
E1	TKW	7A	AX.89497985	1.59E+08	0.10	-1.43	3.25	0.05
E1	TKW	7A	AX.89759971	1.63E+08	0.11	-1.50	3.78	0.06
E1	TKW	7A	AX.89657659	1.51E+08	0.11	-1.38	3.15	0.05
E1	TKW	7A	AX.89775549	1.76E+08	0.11	-1.50	3.63	0.06
E1	TKW	7A	AX.89419990	1.59E+08	0.10	-1.43	3.25	0.05
E1	TKW	7A	AX.89767293	1.52E+08	0.10	-1.43	3.25	0.05
E1	TKW	7A	AX.89737812	1.46E+08	0.10	-1.44	3.29	0.05
E1	TKW	7A	AX.89570703	1.49E+08	0.11	-1.56	3.90	0.07
E1	TKW	7A	AX.89720425	2.29E+08	0.31	-0.93	3.17	0.05
E1	TKW	7A	AX.89726540	1.65E+08	0.11	-1.50	3.78	0.06
E1	TKW	7A	AX.89679375	1.62E+08	0.11	-1.38	3.15	0.05
E1	TKW	7A	AX.89709005	1.72E+08	0.11	-1.50	3.63	0.06
E1	TKW	7A	AX.89516555	1.37E+08	0.12	-1.53	4.01	0.07
E1	TKW	7A	AX.89524740	1.57E+08	0.10	-1.43	3.25	0.05
E1	TKW	7A	AX.89350449	2.31E+08	0.31	-0.97	3.43	0.06
E1	TKW	7A	AX.89327973	1.62E+08	0.41	0.86	3.10	0.05
E1	TKW	7A	AX.89607233	2.02E+08	0.37	0.97	3.70	0.06

E1	TKW	7A	AX.89676560	1.50E+08	0.10	-1.43	3.25	0.05
E1	TKW	7A	AX.89624899	1.45E+08	0.11	-1.38	3.15	0.05
E1	TKW	7A	AX.89676721	1.44E+08	0.11	-1.50	3.78	0.06
E1	TKW	7A	AX.89551283	1.49E+08	0.11	-1.50	3.78	0.06
E1	TKW	7A	AX.89655678	1.63E+08	0.11	-1.38	3.15	0.05
E1	TKW	7A	AX.89490919	1.43E+08	0.11	-1.50	3.78	0.06
E1	TKW	7A	AX.89752260	1.33E+08	0.11	-1.46	3.48	0.06
E1	TKW	7A	AX.89553835	1.65E+08	0.12	-1.45	3.67	0.06
E1	TKW	7A	AX.89731965	1.30E+08	0.11	-1.48	3.69	0.07
E1	TKW	7A	AX.89716253	9.30E+07	0.10	-1.55	3.58	0.06
E1	TKW	7A	AX.89326454	1.58E+08	0.10	-1.43	3.25	0.05
E1	TKW	7A	AX.89633286	1.49E+08	0.11	-1.56	3.90	0.07
E1	TKW	7A	AX.89749810	1.55E+08	0.11	-1.50	3.78	0.06
E1	TKW	7A	AX.89661750	1.39E+08	0.10	-1.63	4.07	0.07
E1	TKW	7A	AX.89748513	1.59E+08	0.10	-1.43	3.25	0.05
E1	TKW	7A	AX.89516270	9.21E+07	0.13	-1.75	5.70	0.10
E1	TKW	7A	AX.89438156	9.31E+07	0.09	-1.52	3.34	0.06
E1	TKW	7A	AX.89679360	9.96E+07	0.08	-1.65	3.57	0.06
E1	TKW	7A	AX.89347950	1.72E+08	0.10	-1.51	3.57	0.06
E1	TKW	7A	AX.89410222	1.38E+08	0.11	-1.48	3.69	0.07
E1	TKW	7A	AX.89739831	9.31E+07	0.14	-1.56	4.76	0.08
E1	TKW	7A	AX.89398554	1.39E+08	0.10	-1.50	3.41	0.06
E1	TKW	7A	AX.89617663	1.39E+08	0.10	-1.50	3.41	0.06
E1	TKW	7A	AX.89678490	2.11E+08	0.10	-1.43	3.25	0.05
E1	TKW	7A	AX.89449940	1.48E+08	0.11	-1.57	3.94	0.07
E1	TKW	7A	AX.89536608	1.56E+08	0.11	-1.50	3.78	0.06
E1	TKW	7A	AX.89690818	1.69E+08	0.11	-1.50	3.78	0.06
E1	TKW	7A	AX.89734863	6.72E+08	0.17	-1.15	3.28	0.06
E1	TKW	7A	AX.89773120	2.25E+08	0.30	-0.91	3.06	0.05
E1	TKW	7A	AX.89552079	2.11E+08	0.10	-1.43	3.25	0.05
E1	TKW	7A	AX.89319483	1.75E+08	0.11	-1.50	3.63	0.06
E1	TKW	7A	AX.89569777	1.50E+08	0.11	-1.50	3.78	0.06
E1	TKW	7A	AX.89535969	1.49E+08	0.10	-1.43	3.25	0.05
E1	TKW	7A	AX.89581078	2.11E+08	0.10	-1.43	3.25	0.05
E1	TKW	7A	AX.89715143	1.63E+08	0.11	-1.50	3.78	0.06
E1	TKW	7A	AX.89490717	9.24E+07	0.12	-1.73	5.01	0.09
E1	TKW	7A	AX.89347387	1.64E+08	0.10	-1.50	3.41	0.06
E1	TKW	7A	AX.89457242	1.56E+08	0.11	-1.50	3.78	0.06
E1	TKW	7A	AX.89581652	1.43E+08	0.11	-1.45	3.46	0.06
E1	TKW	7A	AX.89637523	1.63E+08	0.11	-1.50	3.78	0.06
E1	TKW	7A	AX.89643934	1.45E+08	0.10	-1.44	3.29	0.05
E1	TKW	7A	AX.89704496	1.35E+08	0.11	-1.58	4.13	0.07
E1	TKW	7A	AX.89658338	2.26E+08	0.30	-0.91	3.06	0.05
E1	TKW	7A	AX.89585521	1.39E+08	0.11	-1.46	3.48	0.06
E1	TKW	7A	AX.89571232	1.67E+08	0.10	-1.63	4.07	0.07
E1	TKW	7A	AX.89738339	1.74E+08	0.11	-1.50	3.63	0.06
E1	TKW	7A	AX.89633191	1.45E+08	0.11	-1.50	3.78	0.06
E1	TKW	7A	AX.89744789	2.44E+08	0.47	-0.90	3.45	0.06
E1	TKW	7A	AX.89608997	1.54E+08	0.11	-1.50	3.78	0.06
E1	TKW	7A	AX.89500683	1.68E+08	0.11	-1.50	3.78	0.06

E1	TKW	7A	AX.89314049	1.66E+08	0.11	-1.50	3.78	0.06
E1	TKW	7A	AX.89391932	1.75E+08	0.10	-1.37	3.00	0.05
E1	TKW	7A	AX.89413488	1.28E+08	0.11	-1.50	3.76	0.07
E1	TKW	7A	AX.89387277	1.54E+08	0.11	-1.38	3.15	0.05
E1	TKW	7A	AX.89365511	1.73E+08	0.11	-1.50	3.63	0.06
E1	TKW	7A	AX.89747041	1.36E+08	0.12	-1.53	4.01	0.07
E1	TKW	7A	AX.89576048	1.48E+08	0.10	-1.44	3.29	0.05
E1	TKW	7A	AX.89763293	1.49E+08	0.10	-1.43	3.25	0.05
E1	TKW	7A	AX.89522975	1.58E+08	0.10	-1.43	3.25	0.05
E1	TKW	7A	AX.89351833	1.46E+08	0.10	-1.44	3.29	0.05
E1	TKW	7A	AX.89445402	1.63E+08	0.10	-1.50	3.41	0.06
E1	TKW	7A	AX.89586652	1.63E+08	0.11	-1.50	3.78	0.06
E1	TKW	7A	AX.89482447	1.46E+08	0.11	-1.38	3.15	0.05
E1	TKW	7A	AX.89620967	1.49E+08	0.11	-1.50	3.78	0.06
E1	TKW	7A	AX.89437456	1.01E+08	0.09	-1.57	3.39	0.06
E1	TKW	7A	AX.89585125	1.39E+08	0.12	-1.53	4.01	0.07
E1	TKW	7A	AX.89401779	1.64E+08	0.11	-1.50	3.78	0.06
E1	TKW	7A	AX.89624159	1.61E+08	0.11	-1.50	3.78	0.06
E1	TKW	7A	AX.89737422	1.46E+08	0.10	-1.44	3.29	0.05
E1	GPM2	7A	AX.89744118	6.80E+08	0.43	601.38	3.16	0.05
E1	TKW	7A	AX.89665188	1.39E+08	0.10	-1.50	3.41	0.06
E1	TKW	7A	AX.89662020	1.39E+08	0.10	-1.50	3.41	0.06
E1	TKW	7A	AX.89593799	1.37E+08	0.09	-1.45	3.09	0.05
E1	TKW	7A	AX.89340577	1.64E+08	0.11	-1.50	3.78	0.06
E1	TKW	7A	AX.89442360	2.45E+08	0.47	-0.97	3.93	0.07
E1	TKW	7A	AX.89564998	1.54E+08	0.10	-1.43	3.25	0.05
E1	TKW	7A	AX.89318239	1.56E+08	0.11	-1.50	3.78	0.06
E1	TKW	7A	AX.89454979	1.59E+08	0.10	-1.43	3.25	0.05
E1	GPS	7A	AX.89326437	5.90E+08	0.50	1.11	3.01	0.05
E1	TKW	7A	AX.89774268	1.58E+08	0.11	-1.35	3.15	0.05
E1	TKW	7A	AX.89435711	1.66E+08	0.11	-1.50	3.78	0.06
E1	TKW	7A	AX.89603381	1.46E+08	0.10	-1.44	3.29	0.05
E1	TKW	7A	AX.89463530	1.55E+08	0.11	-1.50	3.78	0.06
E1	TKW	7A	AX.89457168	1.44E+08	0.14	-1.45	4.18	0.07
E1	TKW	7A	AX.89339632	1.60E+08	0.11	-1.50	3.78	0.06
E1	TKW	7A	AX.89723217	9.19E+07	0.15	-1.64	5.61	0.10
E1	TKW	7A	AX.89311121	2.25E+08	0.30	-0.91	3.06	0.05
E1	TKW	7A	AX.89558625	1.45E+08	0.11	-1.50	3.78	0.06
E1	TKW	7A	AX.89450860	1.58E+08	0.10	-1.43	3.25	0.05
E1	TKW	7A	AX.89432963	1.76E+08	0.11	-1.50	3.63	0.06
E1	TKW	7A	AX.89668963	1.74E+08	0.11	-1.34	3.10	0.05
E1	TKW	7A	AX.89556304	2.25E+08	0.30	-0.91	3.06	0.05
E1	TKW	7A	AX.89395703	2.12E+08	0.10	-1.43	3.25	0.05
E1	TKW	7A	AX.89675768	1.62E+08	0.11	-1.38	3.15	0.05
E1	TKW	7A	AX.89505540	2.12E+08	0.11	-1.38	3.15	0.05
E1	TKW	7A	AX.89393659	1.30E+08	0.12	-1.53	4.01	0.07
E1	TKW	7A	AX.89776623	1.67E+08	0.11	-1.50	3.78	0.06
E1	TKW	7A	AX.89638213	1.45E+08	0.11	-1.50	3.78	0.06
E1	TKW	7A	AX.89634936	1.39E+08	0.12	-1.53	4.01	0.07
E1	TKW	7A	AX.89334270	1.43E+08	0.11	-1.45	3.46	0.06

E1	TKW	7A	AX.89645587	2.33E+08	0.31	-0.91	3.09	0.05
E1	TKW	7A	AX.89581578	6.48E+08	0.22	-1.17	3.97	0.07
E1	TKW	7A	AX.89693291	1.45E+08	0.10	-1.44	3.29	0.05
E1	TKW	7A	AX.89703238	1.66E+08	0.11	-1.38	3.15	0.05
E1	TKW	7A	AX.89326890	1.35E+08	0.12	-1.53	4.01	0.07
E1	TKW	7A	AX.89482524	1.74E+08	0.11	-1.34	3.10	0.05
E1	TKW	7A	AX.89352556	1.92E+08	0.11	-1.50	3.63	0.06
E1	TKW	7A	AX.89710443	2.11E+08	0.10	-1.43	3.25	0.05
E1	TKW	7A	AX.89473713	1.44E+08	0.10	-1.44	3.29	0.05
E1	TKW	7A	AX.89534236	1.43E+08	0.11	-1.50	3.78	0.06
E1	TKW	7A	AX.89767754	1.55E+08	0.11	-1.50	3.78	0.06
E1	TKW	7A	AX.89695114	1.69E+08	0.11	-1.50	3.78	0.06
E1	TKW	7A	AX.89674985	1.38E+08	0.10	-1.50	3.41	0.06
E1	TKW	7A	AX.89680783	1.55E+08	0.10	-1.43	3.25	0.05
E1	TKW	7A	AX.89616620	2.44E+08	0.47	-0.90	3.45	0.06
E1	TKW	7A	AX.89546002	2.44E+08	0.47	-0.97	3.93	0.07
E1	TKW	7A	AX.89561828	1.39E+08	0.12	-1.53	4.01	0.07
E1	TKW	7A	AX.89531283	1.63E+08	0.11	-1.50	3.78	0.06
E1	TKW	7A	AX.89457427	1.75E+08	0.10	-1.37	3.00	0.05
E1	TKW	7A	AX.89340926	1.43E+08	0.11	-1.50	3.78	0.06
E1	TKW	7A	AX.89559450	1.63E+08	0.11	-1.50	3.78	0.06
E1	TKW	7A	AX.89492869	1.31E+08	0.12	-1.53	4.01	0.07
E1	TKW	7A	AX.89382385	9.24E+07	0.13	-1.75	5.70	0.10
E1	TKW	7A	AX.89476869	1.46E+08	0.11	-1.38	3.15	0.05
E1	TKW	7A	AX.89663706	2.11E+08	0.11	-1.50	3.78	0.06
E1	TKW	7A	AX.89673202	1.45E+08	0.10	-1.44	3.29	0.05
E1	TKW	7A	AX.89525405	1.71E+08	0.11	-1.50	3.78	0.06
E1	TKW	7A	AX.89453355	2.11E+08	0.11	-1.38	3.15	0.05
E1	TKW	7A	AX.89314646	1.69E+08	0.11	-1.50	3.78	0.06
E1	TKW	7A	AX.89466930	1.71E+08	0.11	-1.50	3.78	0.06
E1	TKW	7A	AX.89486831	1.28E+08	0.11	-1.50	3.76	0.07
E1	TKW	7A	AX.89626767	1.73E+08	0.12	-1.38	3.38	0.06
E1	TKW	7A	AX.89518080	1.62E+08	0.11	-1.38	3.15	0.05
E1	TKW	7A	AX.89532221	1.39E+08	0.12	-1.53	4.01	0.07
E1	TKW	7A	AX.89315572	1.43E+08	0.11	-1.50	3.78	0.06
E1	TKW	7A	AX.89766982	2.31E+08	0.31	-0.93	3.17	0.05
E1	TKW	7A	AX.89324155	9.25E+07	0.13	-1.71	5.35	0.09
E1	TKW	7A	AX.89609923	1.51E+08	0.10	-1.43	3.25	0.05
E1	TKW	7A	AX.89694134	1.54E+08	0.11	-1.38	3.15	0.05
E1	TKW	7A	AX.89612658	1.56E+08	0.11	-1.32	3.02	0.05
E1	TKW	7A	AX.89382266	1.60E+08	0.10	-1.39	3.10	0.05
E1	TKW	7A	AX.89750440	1.46E+08	0.11	-1.50	3.78	0.06
E1	TKW	7A	AX.89367696	2.31E+08	0.31	-0.93	3.17	0.05
E1	TKW	7A	AX.89589491	1.64E+08	0.11	-1.38	3.15	0.05
E1	TKW	7A	AX.89658888	1.59E+08	0.10	-1.43	3.25	0.05
E1	TKW	7A	AX.89503793	1.73E+08	0.11	-1.50	3.63	0.06
E1	TKW	7A	AX.89309860	1.62E+08	0.11	-1.50	3.78	0.06
E1	TKW	7A	AX.89489343	1.67E+08	0.11	-1.57	3.94	0.07
E1	TKW	7A	AX.89365732	1.42E+08	0.11	-1.45	3.46	0.06
E1	TKW	7A	AX.89442420	1.46E+08	0.11	-1.38	3.15	0.05

E1	TKW	7A	AX.89559197	1.65E+08	0.11	-1.38	3.15	0.05
E1	TKW	7A	AX.89395679	1.54E+08	0.10	-1.43	3.25	0.05
E1	TKW	7A	AX.89364717	1.73E+08	0.10	-1.45	3.31	0.05
E1	TKW	7A	AX.89490664	1.39E+08	0.12	-1.53	4.01	0.07
E1	TKW	7A	AX.89490696	1.65E+08	0.11	-1.50	3.78	0.06
E1	TKW	7A	AX.89591960	1.69E+08	0.11	-1.56	3.90	0.07
E1	TKW	7A	AX.89664666	1.51E+08	0.11	-1.38	3.15	0.05
E1	TKW	7A	AX.86163578	1.65E+08	0.11	-1.50	3.78	0.06
E1	TKW	7A	AX.89738526	1.39E+08	0.11	-1.46	3.48	0.06
E1	TKW	7A	AX.89327721	1.63E+08	0.11	-1.50	3.78	0.06
E1	TKW	7A	AX.89423828	1.30E+08	0.11	-1.46	3.48	0.06
E1	TKW	7A	AX.89640632	1.43E+08	0.11	-1.38	3.15	0.05
E1	TKW	7A	AX.89715512	1.76E+08	0.10	-1.37	3.00	0.05
E1	TKW	7A	AX.89445359	9.30E+07	0.14	-1.56	4.76	0.08
E1	TKW	7A	AX.89445388	1.63E+08	0.10	-1.50	3.41	0.06
E1	TKW	7A	AX.89321190	9.68E+07	0.13	-1.43	3.98	0.07
E1	TKW	7A	AX.89524117	2.44E+08	0.47	-0.90	3.45	0.06
E1	TKW	7A	AX.89679566	1.36E+08	0.12	-1.53	4.01	0.07
E1	TKW	7A	AX.89641647	1.63E+08	0.11	-1.38	3.15	0.05
E1	TKW	7A	AX.89505905	1.36E+08	0.12	-1.53	4.01	0.07
E1	TKW	7A	AX.89570013	1.68E+08	0.11	-1.50	3.78	0.06
E1	TKW	7A	AX.89753271	1.39E+08	0.11	-1.48	3.69	0.07
E1	TKW	7A	AX.89577646	1.65E+08	0.11	-1.50	3.78	0.06
E1	TKW	7A	AX.89724963	1.64E+08	0.11	-1.50	3.78	0.06
E1	TKW	7A	AX.89748563	2.44E+08	0.47	-0.97	3.93	0.07
E1	TKW	7A	AX.89733212	1.39E+08	0.10	-1.50	3.41	0.06
E1	TKW	7A	AX.89312274	1.48E+08	0.10	-1.44	3.29	0.05
E1	TKW	7A	AX.89717446	2.11E+08	0.10	-1.43	3.25	0.05
E1	TKW	7A	AX.89659588	1.55E+08	0.11	-1.38	3.15	0.05
E1	TKW	7A	AX.89540791	2.11E+08	0.11	-1.38	3.15	0.05
E1	TKW	7A	AX.89578876	1.28E+08	0.09	-1.47	3.16	0.05
E1	TKW	7A	AX.89741905	1.69E+08	0.11	-1.56	3.90	0.07
E1	TKW	7A	AX.89384450	1.69E+08	0.11	-1.56	3.90	0.07
E1	TKW	7A	AX.89556024	9.24E+07	0.13	-1.75	5.70	0.10
E1	TKW	7A	AX.89627461	1.49E+08	0.11	-1.50	3.78	0.06
E1	TKW	7A	AX.89693975	1.63E+08	0.11	-1.38	3.15	0.05
E1	TKW	7A	AX.89377673	1.46E+08	0.10	-1.44	3.29	0.05
E1	TKW	7A	AX.89409921	2.12E+08	0.11	-1.38	3.15	0.05
E1	TKW	7A	AX.89479561	1.46E+08	0.10	-1.44	3.29	0.05
E1	TKW	7A	AX.89565819	1.67E+08	0.11	-1.50	3.78	0.06
E1	TKW	7A	AX.89598809	1.60E+08	0.11	-1.50	3.78	0.06
E1	TKW	7A	AX.89456953	9.31E+07	0.14	-1.56	4.76	0.08
E1	TKW	7A	AX.89696867	1.59E+08	0.09	-1.51	3.17	0.05
E1	TKW	7A	AX.89713138	1.63E+08	0.11	-1.50	3.78	0.06
E1	TKW	7A	AX.89513912	1.73E+08	0.11	-1.50	3.63	0.06
E1	TKW	7A	AX.89443261	1.39E+08	0.11	-1.54	3.80	0.07
E1	TKW	7A	AX.89591873	9.29E+07	0.13	-1.75	5.70	0.10
E1	TKW	7A	AX.89737852	1.43E+08	0.10	-1.43	3.25	0.05
E1	TKW	7A	AX.89440892	1.74E+08	0.11	-1.34	3.10	0.05
E1	TKW	7A	AX.89341483	1.64E+08	0.11	-1.50	3.78	0.06

E1	TKW	7A	AX.89774061	1.43E+08	0.11	-1.50	3.78	0.06
E1	TKW	7A	AX.89498031	2.12E+08	0.10	-1.43	3.25	0.05
E1	TKW	7A	AX.89314363	2.11E+08	0.11	-1.50	3.78	0.06
E1	TKW	7A	AX.89762809	6.47E+08	0.24	-1.02	3.31	0.06
E1	TKW	7A	AX.89522800	2.11E+08	0.10	-1.43	3.25	0.05
E1	TKW	7A	AX.89738666	6.48E+08	0.22	-1.17	3.97	0.07
E1	TKW	7A	AX.89570761	1.62E+08	0.10	-1.44	3.27	0.05
E1	TKW	7A	AX.89528414	1.30E+08	0.10	-1.41	3.16	0.06
E1	TKW	7A	AX.89349894	2.32E+08	0.31	-0.93	3.17	0.05
E1	TKW	7A	AX.89681143	1.30E+08	0.11	-1.46	3.48	0.06
E1	TKW	7A	AX.89693276	2.11E+08	0.11	-1.38	3.15	0.05
E1	TKW	7A	AX.89429742	1.55E+08	0.11	-1.38	3.15	0.05
E1	TKW	7A	AX.89320738	9.76E+07	0.08	-1.65	3.57	0.06
E1	TKW	7A	AX.89609374	1.45E+08	0.11	-1.38	3.15	0.05
E1	TKW	7A	AX.89594045	1.29E+08	0.10	-1.50	3.41	0.06
E1	TKW	7A	AX.89445885	2.11E+08	0.09	-1.45	3.09	0.05
E1	TKW	7A	AX.89691262	1.76E+08	0.10	-1.37	3.00	0.05
E1	TKW	7A	AX.89382322	1.49E+08	0.11	-1.38	3.15	0.05
E1	TKW	7A	AX.89618041	1.30E+08	0.11	-1.48	3.69	0.07
E1	TKW	7A	AX.89664943	1.45E+08	0.11	-1.45	3.46	0.06
E1	TKW	7A	AX.89746157	1.71E+08	0.11	-1.50	3.78	0.06
E1	TKW	7A	AX.89391234	1.48E+08	0.11	-1.57	3.94	0.07
E1	TKW	7A	AX.89727857	1.63E+08	0.11	-1.38	3.15	0.05
E1	TKW	7A	AX.89423656	1.48E+08	0.10	-1.44	3.29	0.05
E1	TKW	7A	AX.89733640	1.69E+08	0.11	-1.50	3.78	0.06
E1	TKW	7A	AX.89564148	1.56E+08	0.10	-1.43	3.25	0.05
E1	TKW	7A	AX.89766912	1.69E+08	0.10	-1.51	3.57	0.06
E1	TKW	7A	AX.89596449	9.22E+07	0.13	-1.75	5.70	0.10
E1	TKW	7A	AX.89549174	1.74E+08	0.10	-1.37	3.00	0.05
E1	TKW	7A	AX.89433904	1.32E+08	0.11	-1.41	3.37	0.06
E1	TKW	7A	AX.89645017	1.30E+08	0.11	-1.41	3.37	0.06
E1	TKW	7A	AX.89344375	2.99E+08	0.29	-0.94	3.15	0.06
E1	TKW	7A	AX.89616317	9.96E+07	0.09	-1.57	3.39	0.06
E1	TKW	7A	AX.89645691	1.62E+08	0.10	-1.43	3.25	0.05
E1	TKW	7A	AX.89482233	1.47E+08	0.10	-1.44	3.29	0.05
E1	TKW	7A	AX.89371886	1.69E+08	0.11	-1.50	3.78	0.06
E1	TKW	7A	AX.89679184	1.71E+08	0.11	-1.50	3.78	0.06
E1	TKW	7A	AX.89464243	1.63E+08	0.11	-1.57	3.94	0.07
E1	TKW	7A	AX.89472900	1.76E+08	0.11	-1.50	3.63	0.06
E1	TKW	7A	AX.89558892	1.39E+08	0.11	-1.48	3.69	0.07
E1	TKW	7A	AX.89582596	1.43E+08	0.11	-1.50	3.78	0.06
E1	TKW	7A	AX.89607924	1.71E+08	0.11	-1.45	3.46	0.06
E1	TKW	7A	AX.89529525	1.47E+08	0.11	-1.50	3.78	0.06
E1	TKW	7A	AX.89348695	1.33E+08	0.11	-1.46	3.48	0.06
E1	TKW	7A	AX.89514329	1.46E+08	0.10	-1.44	3.29	0.05
E1	TKW	7A	AX.89529919	1.36E+08	0.12	-1.53	4.01	0.07
E1	TKW	7A	AX.89567946	2.12E+08	0.11	-1.50	3.78	0.06
E1	TKW	7A	AX.89404679	1.45E+08	0.11	-1.38	3.15	0.05
E1	TKW	7A	AX.89480444	1.44E+08	0.11	-1.50	3.78	0.06
E1	TKW	7A	AX.89373311	1.39E+08	0.11	-1.48	3.69	0.07

E1	TKW	7A	AX.89320137	1.67E+08	0.10	-1.63	4.07	0.07
E1	TKW	7A	AX.89560870	2.12E+08	0.10	-1.43	3.25	0.05
E1	TKW	7A	AX.89584745	1.46E+08	0.11	-1.50	3.78	0.06
E1	TKW	7A	AX.89657752	1.51E+08	0.10	-1.43	3.25	0.05
E1	TKW	7A	AX.89414061	2.44E+08	0.47	-0.97	3.93	0.07
E1	TKW	7A	AX.89755312	1.69E+08	0.11	-1.50	3.78	0.06
E1	TKW	7A	AX.89742866	9.31E+07	0.07	-1.63	3.01	0.05
E1	TKW	7A	AX.89524570	1.69E+08	0.11	-1.56	3.90	0.07
E1	TKW	7A	AX.89625416	1.57E+08	0.11	-1.50	3.78	0.06
E1	TKW	7A	AX.89773714	1.63E+08	0.11	-1.50	3.78	0.06
E1	TKW	7A	AX.89748815	1.60E+08	0.11	-1.45	3.46	0.06
E1	TKW	7A	AX.89563993	1.01E+08	0.09	-1.57	3.39	0.06
E1	TKW	7A	AX.89676904	1.39E+08	0.11	-1.41	3.37	0.06
E1	TKW	7A	AX.89385468	1.58E+08	0.11	-1.50	3.78	0.06
E1	TKW	7A	AX.89770675	1.57E+08	0.11	-1.50	3.78	0.06
E1	TKW	7A	AX.89669837	9.19E+07	0.08	-1.99	4.71	0.08
E1	TKW	7A	AX.89471729	1.60E+08	0.11	-1.45	3.46	0.06
E1	TKW	7A	AX.89760158	1.48E+08	0.11	-1.45	3.46	0.06
E1	TKW	7A	AX.89566135	1.40E+08	0.10	-1.43	3.25	0.05
E1	TKW	7A	AX.89706709	1.73E+08	0.10	-1.37	3.00	0.05
E1	TKW	7A	AX.89369373	1.36E+08	0.09	-1.45	3.09	0.05
E1	TKW	7A	AX.89761067	1.53E+08	0.11	-1.38	3.15	0.05
E1	TKW	7A	AX.89747753	1.73E+08	0.10	-1.37	3.00	0.05
E1	TKW	7A	AX.89770557	1.02E+08	0.09	-1.57	3.39	0.06
E1	TKW	7A	AX.89623108	1.47E+08	0.10	-1.44	3.29	0.05
E1	TKW	7A	AX.89413022	1.66E+08	0.11	-1.50	3.78	0.06
E1	TKW	7A	AX.89552086	1.48E+08	0.11	-1.38	3.15	0.05
E1	TKW	7A	AX.89563099	1.63E+08	0.11	-1.50	3.78	0.06
E1	TKW	7A	AX.89311866	1.50E+08	0.10	-1.43	3.25	0.05
E1	TKW	7A	AX.89670257	1.65E+08	0.11	-1.50	3.78	0.06
E1	TKW	7A	AX.89595385	2.11E+08	0.11	-1.38	3.15	0.05
E1	TKW	7A	AX.89754191	1.37E+08	0.10	-1.63	4.07	0.07
E1	TKW	7A	AX.89588065	1.46E+08	0.11	-1.38	3.15	0.05
E1	TKW	7A	AX.89609800	1.74E+08	0.11	-1.50	3.63	0.06
E1	TKW	7A	AX.89594145	2.44E+08	0.47	-0.90	3.45	0.06
E1	TKW	7A	AX.89492663	1.32E+08	0.11	-1.46	3.48	0.06
E1	TKW	7A	AX.89359323	5.69E+08	0.25	1.01	3.32	0.06
E1	TKW	7A	AX.89569864	9.34E+07	0.07	-1.94	4.06	0.07
E1	TKW	7A	AX.89453447	2.12E+08	0.11	-1.38	3.15	0.05
E1	TKW	7A	AX.89692416	2.11E+08	0.10	-1.50	3.41	0.06
E1	TKW	7A	AX.89740580	1.66E+08	0.11	-1.38	3.15	0.05
E1	TKW	7A	AX.89725169	1.47E+08	0.11	-1.50	3.78	0.06
E1	TKW	7A	AX.89444163	2.32E+08	0.31	-0.93	3.17	0.05
E1	TKW	7A	AX.89549866	1.39E+08	0.12	-1.53	4.01	0.07
E1	TKW	7A	AX.89408775	2.32E+08	0.31	-0.93	3.17	0.05
E1	TKW	7A	AX.89749279	1.30E+08	0.12	-1.53	4.01	0.07
E1	TKW	7A	AX.89693849	1.74E+08	0.11	-1.50	3.63	0.06
E1	TKW	7A	AX.89482666	1.01E+08	0.09	-1.57	3.39	0.06
E1	TKW	7A	AX.89749770	1.28E+08	0.08	-1.60	3.37	0.06
E1	TKW	7A	AX.89697152	1.38E+08	0.09	-1.45	3.09	0.05

E1	TKW	7A	AX.89462609	1.47E+08	0.11	-1.38	3.15	0.05
E1	TKW	7A	AX.89757664	1.45E+08	0.11	-1.50	3.78	0.06
E1	TKW	7A	AX.89512143	1.28E+08	0.09	-1.47	3.16	0.05
E1	TKW	7A	AX.89512492	1.51E+08	0.34	-0.90	3.14	0.05
E1	TKW	7A	AX.89631557	9.19E+07	0.10	-1.55	3.74	0.06
E1	TKW	7A	AX.89418006	1.32E+08	0.11	-1.35	3.06	0.05
E1	TKW	7A	AX.89513304	1.64E+08	0.11	-1.38	3.15	0.05
E1	TKW	7A	AX.89746708	1.29E+08	0.10	-1.50	3.41	0.06
E1	TKW	7A	AX.89687920	1.56E+08	0.11	-1.38	3.15	0.05
E1	TKW	7A	AX.89523870	9.26E+07	0.13	-1.75	5.70	0.10
E1	TKW	7A	AX.89476138	1.67E+08	0.11	-1.50	3.78	0.06
E1	TKW	7A	AX.89551736	1.63E+08	0.11	-1.50	3.78	0.06
E1	TKW	7A	AX.89722293	1.75E+08	0.10	-1.37	3.00	0.05
E1	TKW	7A	AX.89559997	2.27E+08	0.30	-0.91	3.06	0.05
E1	TKW	7A	AX.89618132	1.72E+08	0.10	-1.45	3.31	0.05
E1	TKW	7A	AX.89380613	1.45E+08	0.10	-1.44	3.29	0.05
E1	TKW	7A	AX.89729956	1.51E+08	0.11	-1.38	3.15	0.05
E1	TKW	7A	AX.89683256	1.63E+08	0.11	-1.50	3.78	0.06
E1	TKW	7A	AX.89474753	1.43E+08	0.11	-1.50	3.78	0.06
E1	TKW	7A	AX.89373595	1.53E+08	0.10	-1.43	3.25	0.05
E1	TKW	7A	AX.89765922	9.31E+07	0.15	-1.55	4.92	0.09
E1	TKW	7A	AX.89368890	1.30E+08	0.11	-1.46	3.48	0.06
E1	GPM2	7A	AX.89486552	2.07E+08	0.31	665.20	3.33	0.06
E1	TKW	7A	AX.89717578	2.11E+08	0.10	-1.43	3.25	0.05
E1	TKW	7A	AX.89447882	1.76E+08	0.11	-1.50	3.63	0.06
E1	TKW	7A	AX.89686118	1.30E+08	0.11	-1.46	3.48	0.06
E1	TKW	7A	AX.89495797	6.72E+08	0.17	-1.15	3.28	0.06
E1	TKW	7A	AX.89461207	1.47E+08	0.10	-1.44	3.29	0.05
E1	TKW	7A	AX.89631561	1.47E+08	0.11	-1.40	3.24	0.05
E1	TKW	7A	AX.89339347	1.74E+08	0.11	-1.39	3.20	0.05
E1	TKW	7A	AX.89542429	9.76E+07	0.09	-1.57	3.39	0.06
E1	TKW	7A	AX.89652732	1.45E+08	0.10	-1.44	3.29	0.05
E1	TKW	7A	AX.89657479	1.39E+08	0.11	-1.49	3.59	0.06
E1	TKW	7A	AX.89369267	1.51E+08	0.11	-1.38	3.15	0.05
E1	TKW	7A	AX.89728582	1.70E+08	0.11	-1.50	3.78	0.06
E1	TKW	7A	AX.89438086	9.27E+07	0.13	-1.75	5.70	0.10
E1	TKW	7A	AX.89775656	1.51E+08	0.10	-1.43	3.25	0.05
E1	TKW	7A	AX.89455663	1.68E+08	0.11	-1.45	3.46	0.06
E1	TKW	7A	AX.89591152	2.25E+08	0.29	-0.98	3.40	0.06
E1	TKW	7A	AX.89327185	1.74E+08	0.10	-1.55	3.75	0.06
E1	TKW	7A	AX.89325179	1.47E+08	0.11	-1.38	3.15	0.05
E1	TKW	7A	AX.89576342	1.00E+08	0.09	-1.57	3.39	0.06
E1	TKW	7A	AX.89554314	9.65E+07	0.12	-1.68	4.90	0.09
E1	TKW	7A	AX.89631101	1.28E+08	0.09	-1.47	3.16	0.05
E1	TKW	7A	AX.89412894	1.64E+08	0.11	-1.50	3.78	0.06
E1	TKW	7A	AX.89760693	1.76E+08	0.10	-1.37	3.00	0.05
E1	TKW	7A	AX.89771979	1.38E+08	0.12	-1.53	4.01	0.07
E1	TKW	7A	AX.89708817	1.64E+08	0.11	-1.50	3.78	0.06
E1	TKW	7A	AX.89393704	1.36E+08	0.12	-1.53	4.01	0.07
E1	TKW	7A	AX.89694506	1.43E+08	0.11	-1.50	3.78	0.06

E1	TKW	7A	AX.89558631	1.49E+08	0.11	-1.45	3.46	0.06
E1	TKW	7A	AX.89512425	1.50E+08	0.10	-1.44	3.16	0.05
E1	TKW	7A	AX.89317218	1.49E+08	0.11	-1.45	3.46	0.06
E1	TKW	7A	AX.89607576	2.11E+08	0.11	-1.38	3.15	0.05
E1	TKW	7A	AX.89733424	2.02E+08	0.10	-1.45	3.31	0.05
E1	TKW	7A	AX.89516376	1.55E+08	0.10	-1.43	3.25	0.05
E1	TKW	7A	AX.89722286	1.72E+08	0.11	-1.50	3.78	0.06
E1	TKW	7A	AX.89666934	1.48E+08	0.10	-1.44	3.29	0.05
E1	TKW	7A	AX.89666498	1.45E+08	0.11	-1.57	3.94	0.07
E1	TKW	7A	AX.89626073	1.49E+08	0.11	-1.50	3.78	0.06
E1	TKW	7A	AX.89404941	1.76E+08	0.11	-1.50	3.63	0.06
E1	TKW	7A	AX.89576921	1.72E+08	0.10	-1.45	3.31	0.05
E1	TKW	7A	AX.89537853	1.29E+08	0.12	-1.53	4.01	0.07
E1	TKW	7A	AX.89674638	1.67E+08	0.11	-1.50	3.78	0.06
E1	TKW	7A	AX.89343150	1.71E+08	0.11	-1.50	3.78	0.06
E1	TKW	7A	AX.89614349	6.72E+08	0.20	-1.25	4.25	0.07
E2	SPM2	7A	AX.89338075	6.28E+08	0.25	14.23	3.33	0.06
E2	SPM2	7A	AX.89689513	6.40E+08	0.13	17.18	3.01	0.05
E2	SPM2	7A	AX.89497690	8.59E+07	0.21	14.93	3.32	0.06
E2	SPM2	7A	AX.89536189	8.52E+07	0.47	-11.71	3.07	0.05
E2	SPM2	7A	AX.89441511	8.56E+07	0.16	17.06	3.42	0.06
E2	SPM2	7A	AX.89533869	9.03E+07	0.10	26.41	5.31	0.10
E2	SPM2	7A	AX.89547147	8.52E+07	0.47	-12.49	3.43	0.06
E2	SPM2	7A	AX.89389967	8.52E+07	0.47	-11.71	3.07	0.05
E2	SPM2	7A	AX.89389117	8.53E+07	0.48	-12.62	3.50	0.06
E2	SPM2	7A	AX.89347005	8.52E+07	0.47	-11.71	3.07	0.05
E2	SPM2	7A	AX.89340453	8.59E+07	0.22	15.19	3.47	0.06
E2	SPM2	7A	AX.89562955	6.40E+08	0.17	17.34	3.73	0.06
E2	SPM2	7A	AX.89366635	9.03E+07	0.10	26.41	5.31	0.10
E2	SPM2	7A	AX.89328795	9.05E+07	0.05	28.43	3.45	0.06
E2	SPM2	7A	AX.89344728	8.38E+07	0.48	-12.14	3.27	0.06
E2	SPM2	7A	AX.89519524	9.14E+07	0.07	25.22	3.80	0.07
E2	GY	7A	AX.89655850	4.77E+07	0.07	-4.63	4.29	0.06
E2	SPM2	7A	AX.89399929	8.56E+07	0.16	16.34	3.18	0.05
E2	SPM2	7A	AX.89472739	6.40E+08	0.13	17.18	3.01	0.05
E2	SPM2	7A	AX.89310521	8.52E+07	0.47	-11.71	3.07	0.05
E2	SPM2	7A	AX.89592523	2.23E+08	0.20	15.88	3.59	0.06
E2	SPM2	7A	AX.89690492	8.38E+07	0.47	-11.76	3.09	0.06
E2	SPM2	7A	AX.89437917	8.53E+07	0.48	-12.62	3.50	0.06
E2	SPM2	7A	AX.89717792	6.27E+08	0.20	14.71	3.10	0.05
E2	SPM2	7A	AX.89362135	8.53E+07	0.48	-12.62	3.50	0.06
E2	SPM2	7A	AX.89446007	6.28E+08	0.20	14.98	3.25	0.06
E2	SPM2	7A	AX.89443663	9.19E+07	0.12	19.50	3.44	0.06
E2	SPM2	7A	AX.89322222	4.75E+07	0.16	17.16	3.52	0.06
E2	SPM2	7A	AX.89422899	6.28E+08	0.25	14.23	3.33	0.06
E2	SPM2	7A	AX.89360916	7.32E+08	0.10	21.32	3.52	0.06
E2	SPM2	7A	AX.89450088	1.49E+08	0.20	15.54	3.36	0.06
E2	SPM2	7A	AX.89613288	8.56E+07	0.16	16.58	3.32	0.06
E2	SPM2	7A	AX.89382961	8.38E+07	0.47	-11.76	3.09	0.06
E2	SPM2	7A	AX.89454239	6.28E+08	0.22	14.78	3.31	0.06

E2	SPM2	7A	AX.89728059	8.53E+07	0.48	-12.62	3.50	0.06
E2	SPM2	7A	AX.89495137	6.29E+08	0.25	14.23	3.33	0.06
E2	SPM2	7A	AX.89533985	8.52E+07	0.47	-11.71	3.07	0.05
E2	SPM2	7A	AX.89331101	7.36E+08	0.08	24.79	3.87	0.07
E2	SPM2	7A	AX.89359463	8.52E+07	0.47	-11.61	3.03	0.05
E2	SPM2	7A	AX.89722397	6.40E+08	0.13	17.18	3.01	0.05
E2	SPM2	7A	AX.89373046	6.14E+08	0.08	22.39	3.39	0.06
E2	SPM2	7A	AX.89607906	8.38E+07	0.47	-11.76	3.09	0.06
E2	SPM2	7A	AX.89589758	6.27E+08	0.20	14.71	3.10	0.05
E2	SPM2	7A	AX.89610935	8.53E+07	0.48	-12.62	3.50	0.06
E2	SPM2	7A	AX.89496810	8.52E+07	0.47	-11.71	3.07	0.05
E2	SPM2	7A	AX.89414275	8.99E+07	0.07	27.07	3.86	0.07
E2	SPM2	7A	AX.89561047	8.52E+07	0.47	-11.71	3.07	0.05
E2	SPM2	7A	AX.89691294	8.53E+07	0.48	-12.62	3.50	0.06
E2	SPM2	7A	AX.89694768	9.19E+07	0.09	20.36	3.01	0.05
E2	SPM2	7A	AX.89549336	8.38E+07	0.47	-11.76	3.09	0.06
E2	GY	7A	AX.89651931	6.87E+06	0.36	-1.96	3.02	0.06
E2	GY	7A	AX.89651931	6.87E+06	0.36	-1.96	3.02	0.06
E2	SPM2	7A	AX.89712434	9.14E+07	0.07	25.22	3.80	0.07
E2	SPM2	7A	AX.89560537	8.49E+07	0.45	12.27	3.30	0.06
E2	SPM2	7A	AX.89748541	8.38E+07	0.47	-11.76	3.09	0.06
E2	SPM2	7A	AX.89539906	8.38E+07	0.49	-12.18	3.29	0.06
E2	SPM2	7A	AX.89357222	9.03E+07	0.10	26.41	5.31	0.10
E2	GY	7A	AX.89474325	4.76E+07	0.05	-4.53	3.40	0.05
E2	SPM2	7A	AX.89701961	8.38E+07	0.47	-11.76	3.09	0.06
E2	SPM2	7A	AX.89757161	6.41E+08	0.12	17.90	3.06	0.05
E2	SPM2	7A	AX.89626055	6.40E+08	0.13	17.18	3.01	0.05
E2	SPM2	7A	AX.89365387	8.53E+07	0.48	-12.62	3.50	0.06
E2	SPM2	7A	AX.89773543	8.98E+07	0.07	25.10	3.77	0.06
E2	SPM2	7A	AX.89575300	8.56E+07	0.16	16.34	3.18	0.05
E2	TKW	7A	AX.89645283	6.48E+08	0.23	-1.32	4.20	0.07
E2	TKW	7A	AX.89711943	2.44E+08	0.47	-0.93	3.11	0.05
E2	SPM2	7A	AX.89659114	8.52E+07	0.47	-11.84	3.12	0.05
E2	SPM2	7A	AX.89427331	8.53E+07	0.48	-12.62	3.50	0.06
E2	SPM2	7A	AX.89438003	8.59E+07	0.21	14.93	3.32	0.06
E2	SPM2	7A	AX.89399345	8.59E+07	0.21	14.93	3.32	0.06
E2	SPM2	7A	AX.89565261	8.56E+07	0.16	16.34	3.18	0.05
E2	SPM2	7A	AX.89698264	8.52E+07	0.47	-11.71	3.07	0.05
E2	SPM2	7A	AX.89635468	8.54E+07	0.48	-12.62	3.50	0.06
E2	SPM2	7A	AX.89433942	8.56E+07	0.16	16.58	3.32	0.06
E2	SPM2	7A	AX.89740653	6.29E+08	0.22	15.90	3.80	0.07
E2	SPM2	7A	AX.89416745	8.53E+07	0.48	-12.62	3.50	0.06
E2	SPM2	7A	AX.89322489	8.52E+07	0.47	-11.71	3.07	0.05
E2	SPM2	7A	AX.89536861	6.28E+08	0.25	14.23	3.33	0.06
E2	SPM2	7A	AX.89426478	8.59E+07	0.21	14.93	3.32	0.06
E2	SPM2	7A	AX.89766471	8.53E+07	0.48	-12.62	3.50	0.06
E2	SPM2	7A	AX.89676739	8.53E+07	0.48	-12.62	3.50	0.06
E2	SPM2	7A	AX.89522144	6.40E+08	0.17	17.34	3.73	0.06
E2	SPM2	7A	AX.89569864	9.34E+07	0.07	22.74	3.02	0.05
E2	SPM2	7A	AX.89711930	6.27E+08	0.20	14.72	3.11	0.05

E2	SPM2	7A	AX.89373433	8.59E+07	0.21	14.93	3.32	0.06
E2	SPM2	7A	AX.89507244	8.49E+07	0.46	11.99	3.18	0.06
E2	SPM2	7A	AX.89651386	6.29E+08	0.24	14.24	3.30	0.06
E2	SPM2	7A	AX.89358546	8.51E+07	0.47	-11.71	3.07	0.05
E2	SPM2	7A	AX.89370059	6.27E+08	0.20	14.66	3.13	0.05
E2	SPM2	7A	AX.89752478	9.14E+07	0.12	17.97	3.08	0.05
E2	SPM2	7A	AX.89741339	8.38E+07	0.48	-12.14	3.27	0.06
E2	SPM2	7A	AX.89614860	6.40E+08	0.17	17.34	3.73	0.06
E2	SPM2	7A	AX.89508141	9.14E+07	0.08	23.71	3.58	0.06
E2	SPM2	7A	AX.89667694	8.53E+07	0.48	-12.62	3.50	0.06
E2	SPM2	7A	AX.89746781	8.53E+07	0.48	-12.62	3.50	0.06
E2	SPM2	7A	AX.89773032	8.53E+07	0.48	-12.62	3.50	0.06
E2	GPM2	7A	AX.89733210	6.21E+08	0.10	871.08	3.50	0.07
E2	TKW	7A	AX.89464638	8.54E+07	0.23	-1.14	3.31	0.06
E2	SPM2	7A	AX.89425039	9.14E+07	0.08	23.71	3.58	0.06
E2	SPM2	7A	AX.89490892	6.29E+08	0.25	14.23	3.33	0.06
E2	SPM2	7A	AX.89571170	8.52E+07	0.47	-11.71	3.07	0.05
E2	SPM2	7A	AX.89393785	8.53E+07	0.48	-12.62	3.50	0.06
E2	TKW	7A	AX.89738666	6.48E+08	0.23	-1.32	4.20	0.07
E2	TKW	7A	AX.89616620	2.44E+08	0.46	-0.92	3.03	0.05
E2	GY	7A	AX.89503581	6.86E+06	0.36	-1.96	3.02	0.06
E2	GY	7A	AX.89503581	6.86E+06	0.36	-1.96	3.02	0.06
E2	TKW	7A	AX.89324155	9.25E+07	0.13	-1.45	3.31	0.06
E2	TKW	7A	AX.89655050	2.45E+08	0.46	-0.92	3.03	0.05
E2	TKW	7A	AX.89607233	2.02E+08	0.38	1.02	3.45	0.06
E2	GY	7A	AX.89754726	1.13E+08	0.19	-2.47	3.18	0.05
E2	GY	7A	AX.89754726	1.13E+08	0.19	-2.47	3.18	0.05
E2	GPM2	7A	AX.89374570	6.21E+08	0.10	871.08	3.50	0.07
E2	GY	7A	AX.89393977	6.13E+08	0.41	-1.95	3.11	0.04
E2	GY	7A	AX.89393977	6.13E+08	0.41	-1.95	3.11	0.04
E2	TKW	7A	AX.89470936	7.11E+08	0.31	-1.03	3.23	0.06
E2	GY	7A	AX.89455713	4.77E+07	0.07	-4.63	4.29	0.06
E2	GY	7A	AX.89455713	4.77E+07	0.07	-4.63	4.29	0.06
E2	TKW	7A	AX.89565462	9.25E+07	0.13	-1.48	3.53	0.06
E2	TKW	7A	AX.89400115	9.19E+07	0.11	-1.51	3.26	0.06
E2	TKW	7A	AX.89659553	2.44E+08	0.47	-0.96	3.27	0.05
E2	GY	7A	AX.89655850	4.77E+07	0.07	-4.63	4.29	0.06
E2	TKW	7A	AX.89310792	8.54E+07	0.23	-1.15	3.30	0.06
E2	TKW	7A	AX.89762809	6.47E+08	0.24	-1.17	3.55	0.06
E2	TKW	7A	AX.89568365	6.48E+08	0.19	-1.23	3.28	0.05
E2	GY	7A	AX.89474325	4.76E+07	0.05	-4.53	3.40	0.05
E2	TKW	7A	AX.89558931	2.44E+08	0.47	-0.96	3.27	0.05
E2	TKW	7A	AX.89594145	2.44E+08	0.46	-0.92	3.03	0.05
E2	TKW	7A	AX.89327554	1.72E+06	0.25	-1.12	3.36	0.06
E2	TKW	7A	AX.89442360	2.45E+08	0.47	-0.96	3.27	0.05
E2	TKW	7A	AX.89516270	9.21E+07	0.13	-1.48	3.53	0.06
E2	TKW	7A	AX.89628948	2.44E+08	0.47	-0.94	3.19	0.05
E2	GPM2	7A	AX.89367673	1.35E+08	0.48	552.24	3.85	0.07
E2	GPM2	7A	AX.89532520	6.21E+08	0.10	871.08	3.50	0.07
E2	TKW	7A	AX.89726434	8.54E+07	0.23	-1.15	3.30	0.06

E2	TKW	7A	AX.89722718	8.54E+07	0.23	-1.14	3.31	0.06
E2	TKW	7A	AX.89456167	9.53E+06	0.19	-1.28	3.53	0.06
E2	GY	7A	AX.89383516	4.76E+07	0.09	-3.96	4.18	0.06
E2	TKW	7A	AX.89653667	9.31E+07	0.14	-1.33	3.02	0.05
E2	TKW	7A	AX.89513616	9.31E+07	0.14	-1.33	3.02	0.05
E2	TKW	7A	AX.89471369	7.13E+08	0.23	-1.09	3.02	0.05
E2	TKW	7A	AX.89600921	2.44E+08	0.47	-0.96	3.27	0.05
E2	TKW	7A	AX.89414061	2.44E+08	0.47	-0.96	3.27	0.05
E2	TKW	7A	AX.89668647	9.66E+07	0.12	-1.47	3.22	0.06
E2	TKW	7A	AX.89556024	9.24E+07	0.13	-1.48	3.53	0.06
E2	TKW	7A	AX.89445359	9.30E+07	0.14	-1.33	3.02	0.05
E2	TKW	7A	AX.89756658	2.44E+08	0.46	-0.92	3.03	0.05
E2	TKW	7A	AX.89563839	6.48E+08	0.23	-1.32	4.20	0.07
E2	TKW	7A	AX.86168039	6.47E+08	0.24	-1.07	3.06	0.05
E2	TKW	7A	AX.89317732	2.71E+07	0.41	-0.95	3.15	0.05
E2	TKW	7A	AX.89655079	8.54E+07	0.23	-1.14	3.31	0.06
E2	TKW	7A	AX.89546002	2.44E+08	0.47	-0.96	3.27	0.05
E2	GY	7A	AX.89383516	4.76E+07	0.09	-3.96	4.18	0.06
E2	TKW	7A	AX.89609258	6.47E+08	0.18	-1.21	3.09	0.05
E2	TKW	7A	AX.89777832	9.29E+07	0.13	-1.48	3.53	0.06
E2	TKW	7A	AX.89451059	7.24E+08	0.11	-1.44	3.03	0.05
E2	TKW	7A	AX.89567196	2.38E+08	0.39	1.00	3.42	0.06
E2	TKW	7A	AX.89505927	8.54E+07	0.23	-1.15	3.30	0.06
E2	TKW	7A	AX.89708661	2.71E+07	0.41	-0.95	3.15	0.05
E2	TKW	7A	AX.89528732	6.47E+08	0.19	-1.23	3.28	0.05
E2	TKW	7A	AX.89515531	8.54E+07	0.23	-1.14	3.31	0.06
E2	TKW	7A	AX.89739831	9.31E+07	0.14	-1.33	3.02	0.05
E2	TKW	7A	AX.89546999	9.24E+07	0.13	-1.48	3.53	0.06
E2	TKW	7A	AX.89369333	6.48E+08	0.23	-1.32	4.20	0.07
E2	TKW	7A	AX.89523870	9.26E+07	0.13	-1.48	3.53	0.06
E2	GY	7A	AX.89388162	1.20E+07	0.48	-1.89	3.01	0.06
E2	TKW	7A	AX.89490717	9.24E+07	0.11	-1.51	3.27	0.06
E2	TKW	7A	AX.89774808	7.12E+08	0.23	-1.11	3.16	0.05
E2	TKW	7A	AX.89744789	2.44E+08	0.46	-0.92	3.03	0.05
E2	TKW	7A	AX.89560087	2.09E+08	0.29	1.00	3.01	0.05
E2	TKW	7A	AX.89738372	9.29E+07	0.13	-1.48	3.53	0.06
E2	TKW	7A	AX.89530196	9.19E+07	0.13	-1.48	3.53	0.06
E2	TKW	7A	AX.89651291	2.71E+07	0.42	-0.93	3.06	0.05
E2	TKW	7A	AX.89743153	9.25E+07	0.13	-1.48	3.53	0.06
E2	TKW	7A	AX.89382385	9.24E+07	0.13	-1.48	3.53	0.06
E2	TKW	7A	AX.86163741	6.47E+08	0.19	-1.23	3.28	0.05
E2	TKW	7A	AX.89559201	9.31E+07	0.14	-1.33	3.02	0.05
E2	TKW	7A	AX.89527321	1.93E+08	0.46	0.97	3.29	0.05
E2	TKW	7A	AX.89577128	7.12E+08	0.31	-1.07	3.49	0.07
E2	GY	7A	AX.89597326	6.85E+06	0.38	-1.98	3.13	0.06
E2	TKW	7A	AX.89456953	9.31E+07	0.14	-1.33	3.02	0.05
E2	TKW	7A	AX.89655282	2.44E+08	0.47	-0.96	3.27	0.05
E2	TKW	7A	AX.89583849	2.44E+08	0.47	-0.96	3.27	0.05
E2	TKW	7A	AX.89492125	2.44E+08	0.47	-0.96	3.27	0.05
E2	TKW	7A	AX.89699004	2.44E+08	0.46	-0.92	3.03	0.05

E2	TKW	7A	AX.89499809	9.31E+07	0.14	-1.33	3.02	0.05
E2	TKW	7A	AX.86168877	6.47E+08	0.23	-1.32	4.20	0.07
E2	TKW	7A	AX.89748563	2.44E+08	0.47	-0.96	3.27	0.05
E2	GY	7A	AX.89388162	1.20E+07	0.48	-1.89	3.01	0.06
E2	TKW	7A	AX.89464561	9.31E+07	0.14	-1.33	3.02	0.05
E2	TKW	7A	AX.89636079	9.19E+07	0.15	-1.49	3.85	0.07
E2	TKW	7A	AX.89581578	6.48E+08	0.23	-1.32	4.20	0.07
E2	GY	7A	AX.89597326	6.85E+06	0.38	-1.98	3.13	0.06
E2	TKW	7A	AX.89596449	9.22E+07	0.13	-1.48	3.53	0.06
E2	TKW	7A	AX.89326929	6.47E+08	0.24	-1.17	3.55	0.06
E2	TKW	7A	AX.89506329	9.31E+07	0.14	-1.33	3.02	0.05
E2	TKW	7A	AX.89540561	8.54E+07	0.23	-1.14	3.31	0.06
E2	GPM2	7A	AX.89754263	6.21E+08	0.10	871.08	3.50	0.07
E2	TKW	7A	AX.89478535	7.10E+08	0.35	-0.98	3.16	0.06
E2	TKW	7A	AX.89597147	9.76E+07	0.14	-1.32	3.06	0.05
E2	TKW	7A	AX.89367673	1.35E+08	0.48	-0.95	3.24	0.06
E2	TKW	7A	AX.89695436	2.71E+07	0.42	-0.93	3.06	0.05
E2	TKW	7A	AX.89765922	9.31E+07	0.15	-1.37	3.33	0.06
E2	TKW	7A	AX.89548934	2.71E+07	0.41	-0.95	3.15	0.05
E2	GY	7A	AX.89754578	6.92E+08	0.21	-2.84	4.29	0.06
E2	TKW	7A	AX.89334123	9.22E+07	0.13	-1.48	3.53	0.06
E2	GPM2	7A	AX.89411187	6.21E+08	0.10	871.08	3.50	0.07
E2	TKW	7A	AX.89681811	8.54E+07	0.23	-1.14	3.31	0.06
E2	TKW	7A	AX.89591873	9.29E+07	0.13	-1.48	3.53	0.06
E2	TKW	7A	AX.89767696	9.31E+07	0.14	-1.33	3.02	0.05
E2	TKW	7A	AX.89364823	9.24E+07	0.13	-1.48	3.53	0.06
E2	TKW	7A	AX.89411491	8.54E+07	0.23	-1.15	3.30	0.06
E2	TKW	7A	AX.89524117	2.44E+08	0.46	-0.92	3.03	0.05
E2	TKW	7A	AX.89662707	6.47E+08	0.19	-1.23	3.28	0.05
E2	TKW	7A	AX.89723217	9.19E+07	0.15	-1.55	4.20	0.08
E2	TKW	7A	AX.89661348	1.58E+08	0.14	-1.43	3.39	0.06
E2	TKW	7A	AX.89460324	1.54E+08	0.28	1.10	3.46	0.06
E2	TKW	7A	AX.89457168	1.44E+08	0.14	-1.43	3.39	0.06
E2	TKW	7A	AX.89487442	7.12E+08	0.22	-1.17	3.34	0.06
E2	TKW	7A	AX.89554314	9.65E+07	0.12	-1.47	3.22	0.06
E2	TKW	7A	AX.89512482	2.44E+08	0.47	-0.96	3.27	0.05
E2	GY	7A	AX.89754578	6.92E+08	0.21	-2.84	4.29	0.06
E2	TKW	7A	AX.89438086	9.27E+07	0.13	-1.48	3.53	0.06
E2	TKW	7A	AX.89757701	9.76E+07	0.12	-1.47	3.22	0.06
E2	TKW	7A	AX.89739023	6.47E+08	0.19	-1.23	3.28	0.05
E2	TKW	7A	AX.89724295	6.48E+08	0.19	-1.23	3.28	0.05
E3	GPM2	7A	AX.89570857	5.38E+08	0.18	-1102.29	3.10	0.06
E3	GPM2	7A	AX.89485756	5.39E+08	0.15	-1134.83	3.18	0.00
E3	GPS	7A	AX.89722349	3.92E+07	0.39	2.07	3.01	0.10
E3	GY	7A	AX.89310828	5.64E+07	0.07	5.39	3.35	0.04
E3	GY	7A	AX.89393409	2.33E+08	0.12	-4.59	3.10	0.06
E3	GY	7A	AX.89589823	5.80E+08	0.26	-3.42	3.02	0.06
E3	TKW	7A	AX.89547571	6.18E+08	0.40	0.86	3.19	0.06
E3	TKW	7A	AX.89729033	6.90E+08	0.20	1.16	3.43	0.13
E3	TKW	7A	AX.89442921	6.95E+08	0.24	-0.98	3.31	0.04

E3	TKW	7A	AX.89462447	6.95E+08	0.24	-1.06	3.78	0.04
E3	TKW	7A	AX.89399371	6.95E+08	0.25	-0.94	3.11	0.04
E3	TKW	7A	AX.89526831	7.01E+08	0.33	0.92	3.03	0.14
E3	SPM2	7A	AX.89336359	5.50E+07	0.17	27.33	3.19	0.09
E3	SPM2	7A	AX.89355609	5.54E+07	0.18	29.77	3.99	0.08
E3	SPM2	7A	AX.89411886	5.54E+07	0.18	26.13	3.15	0.07
E3	TKW	7A	AX.89507777	4.82E+07	0.19	1.11	3.15	0.10
E3	TKW	7A	AX.89570857	5.38E+08	0.18	1.09	3.07	0.07
E4	GPM2	7A	AX.89657710	6.11E+08	0.25	521.44	3.48	0.05
E4	GPM2	7A	AX.89468430	6.11E+08	0.26	510.08	3.39	0.05
E4	GPM2	7A	AX.89368907	6.11E+08	0.25	528.28	3.59	0.05
E4	GPM2	7A	AX.89683691	6.11E+08	0.25	521.44	3.48	0.05
E4	GPM2	7A	AX.89527896	6.11E+08	0.25	521.44	3.48	0.05
E4	GPM2	7A	AX.89324393	6.11E+08	0.25	495.28	3.19	0.05
E4	GPM2	7A	AX.89592969	6.11E+08	0.26	510.08	3.39	0.05
E4	GPM2	7A	AX.89494441	6.11E+08	0.25	521.44	3.48	0.05
E4	GPS	7A	AX.89683923	7.30E+08	0.27	-1.60	3.05	0.03
E4	GPS	7A	AX.89327720	7.63E+07	0.38	-1.51	3.02	0.05
E4	GPS	7A	AX.89382787	7.63E+07	0.38	-1.51	3.02	0.05
E4	GY	7A	AX.89502546	6.26E+08	0.41	1.54	3.15	0.05
E4	GY	7A	AX.89577081	6.27E+08	0.44	-1.49	3.06	0.05
E4	GY	7A	AX.89376670	6.27E+08	0.47	-1.55	3.26	0.06
E4	GY	7A	AX.89474076	7.12E+08	0.41	-1.52	3.19	0.04
E4	GY	7A	AX.89575405	7.12E+08	0.44	-1.45	3.01	0.04
E4	GY	7A	AX.89682971	7.13E+08	0.35	-1.52	3.00	0.04
E4	GY	7A	AX.89566960	7.13E+08	0.35	-1.52	3.00	0.04
E4	GY	7A	AX.89498653	7.16E+08	0.38	1.52	3.03	0.04
E4	GPS	7A	AX.89401082	7.64E+07	0.38	-1.52	3.03	0.05
E4	GPS	7A	AX.89707942	7.65E+07	0.37	-1.60	3.31	0.06
E4	GPS	7A	AX.89430333	7.65E+07	0.38	-1.56	3.17	0.05
E4	GPS	7A	AX.89746821	7.64E+07	0.38	-1.56	3.17	0.05
E4	GPS	7A	AX.89390000	7.64E+07	0.38	-1.56	3.17	0.05
E4	GPS	7A	AX.89334823	7.29E+08	0.33	-1.63	3.31	0.04
E4	GPS	7A	AX.89761639	7.30E+08	0.32	-1.59	3.15	0.04
E4	GPS	7A	AX.89575998	7.65E+07	0.38	-1.56	3.17	0.05
E4	GPS	7A	AX.89330322	7.65E+07	0.38	-1.56	3.17	0.05
E4	GPS	7A	AX.89613698	7.63E+07	0.38	-1.56	3.17	0.05
E4	GPS	7A	AX.89562292	7.63E+07	0.38	-1.55	3.14	0.05
E4	GY	7A	AX.89406368	5.80E+08	0.23	-2.02	3.65	0.07
E4	GY	7A	AX.89637941	5.80E+08	0.20	-1.96	3.16	0.06
E4	GY	7A	AX.89374967	5.83E+08	0.20	-1.92	3.05	0.06
E4	GY	7A	AX.89622170	6.09E+08	0.22	-1.88	3.17	0.07
E4	GY	7A	AX.89459548	6.11E+08	0.47	1.59	3.41	0.06
E4	GY	7A	AX.89367435	6.11E+08	0.49	1.62	3.57	0.07
E4	GY	7A	AX.89454931	5.75E+08	0.21	-1.95	3.26	0.06
E4	GY	7A	AX.89698201	5.75E+08	0.20	-2.05	3.48	0.07
E4	GY	7A	AX.89395637	5.75E+08	0.20	-2.05	3.48	0.07
E4	GY	7A	X.89647087_OT	5.75E+08	0.20	-2.00	3.32	0.06
E4	GY	7A	X.89491322_OT	5.75E+08	0.20	-2.37	4.55	0.08
E4	GY	7A	AX.89317202	5.76E+08	0.21	-1.94	3.22	0.06

E4	GY	7A	AX.89310421	5.75E+08	0.21	-1.86	3.03	0.06
E4	GY	7A	AX.89666394	5.80E+08	0.22	-1.96	3.42	0.07
E4	GY	7A	AX.89525682	5.80E+08	0.21	-2.03	3.56	0.06
E4	SPM2	7A	AX.89511873	1.95E+07	0.14	-24.03	3.28	0.05
E4	SPM2	7A	AX.89557808	2.00E+07	0.18	-20.51	3.08	0.03
E4	SPM2	7A	AX.89345079	3.11E+07	0.31	-17.15	3.14	0.03
E4	SPM2	7A	AX.89607031	8.99E+07	0.07	30.26	3.04	0.03
E4	SPM2	7A	AX.89368186	1.46E+08	0.08	28.82	3.14	0.07
E4	SPM2	7A	AX.89558597	1.60E+08	0.06	40.03	3.98	0.09
E4	SPM2	7A	AX.89661967	2.05E+08	0.06	34.21	3.29	0.07
E4	SPM2	7A	AX.89321375	2.24E+08	0.07	33.43	3.42	0.07
E4	SPM2	7A	AX.89518487	2.33E+08	0.08	30.68	3.12	0.07
E4	SPM2	7A	AX.89444418	3.48E+08	0.09	30.74	3.72	0.07
E4	TKW	7A	AX.89539865	6.25E+07	0.33	-0.89	3.03	0.09
E4	TKW	7A	AX.89493102	5.73E+08	0.32	0.87	3.03	0.09
E4	TKW	7A	AX.89584122	5.76E+08	0.34	0.89	3.10	0.09
E4	TKW	7A	AX.89529897	6.66E+08	0.12	1.43	3.81	0.01
E4	TKW	7A	AX.89630231	6.69E+08	0.11	1.28	3.08	0.01
E4	TKW	7A	AX.89349756	6.72E+08	0.27	1.10	4.11	0.02
E4	TKW	7A	AX.89520446	6.72E+08	0.32	0.90	3.04	0.00
E4	TKW	7A	AX.89668053	6.74E+08	0.33	0.89	3.16	0.01
E4	TKW	7A	AX.89764215	6.74E+08	0.33	0.90	3.24	0.01
E4	TKW	7A	AX.89592875	7.10E+08	0.16	-1.03	3.04	0.02
E4	TKW	7A	AX.89433881	7.10E+08	0.16	-1.03	3.04	0.02
E4	TKW	7A	AX.89575405	7.12E+08	0.43	-0.84	3.29	0.06
E1	GY	7B	AX.89422790	7.09E+08	0.46	2.55	3.73	0.07
E1	GY	7B	AX.89326836	6.11E+08	0.08	-4.79	4.01	0.06
E1	GY	7B	AX.89637049	7.09E+08	0.43	2.38	3.27	0.06
E1	GY	7B	AX.89544298	7.09E+08	0.43	2.32	3.13	0.06
E1	GY	7B	AX.89402723	5.95E+08	0.13	-3.61	3.35	0.06
E1	GY	7B	AX.89544298	7.09E+08	0.43	2.32	3.13	0.06
E1	GY	7B	AX.89628690	3.39E+07	0.07	-4.52	3.31	0.06
E1	GY	7B	AX.89503859	3.39E+07	0.08	-4.35	3.25	0.06
E1	GY	7B	AX.89439688	3.18E+07	0.05	-5.94	3.62	0.07
E1	GY	7B	AX.89353636	7.09E+08	0.43	2.27	3.01	0.05
E1	GY	7B	AX.89463761	7.09E+08	0.44	2.48	3.53	0.07
E1	GY	7B	AX.89687662	4.90E+06	0.20	-2.83	3.01	0.05
E1	GY	7B	AX.89442633	7.22E+08	0.20	-3.24	3.79	0.06
E1	GY	7B	AX.89376058	7.09E+08	0.40	2.35	3.15	0.06
E1	GY	7B	AX.89732098	7.22E+08	0.28	-2.73	3.50	0.05
E1	GY	7B	AX.89422448	7.11E+08	0.34	-2.49	3.28	0.06
E1	GY	7B	AX.89535574	7.09E+08	0.15	-3.91	4.36	0.08
E1	GY	7B	AX.89610838	7.09E+08	0.43	2.38	3.27	0.06
E1	GY	7B	AX.89531154	7.09E+08	0.43	2.38	3.27	0.06
E1	GY	7B	AX.89637049	7.09E+08	0.43	2.38	3.27	0.06
E1	GY	7B	AX.89633557	7.14E+08	0.44	-2.35	3.22	0.05
E1	GY	7B	AX.89387103	7.23E+08	0.26	-2.63	3.16	0.05
E1	GY	7B	AX.89344849	7.22E+08	0.28	-2.74	3.55	0.05
E1	GY	7B	AX.89770281	7.22E+08	0.28	-2.73	3.50	0.05
E1	GY	7B	AX.89734790	3.39E+07	0.08	-4.35	3.25	0.06

E1	GY	7B	AX.89455659	6.78E+07	0.07	-5.35	3.99	0.06
E1	GY	7B	AX.89455659	6.78E+07	0.07	-5.35	3.99	0.06
E1	GY	7B	AX.89531154	7.09E+08	0.43	2.38	3.27	0.06
E1	GY	7B	AX.89594740	6.11E+08	0.08	-4.79	4.01	0.06
E1	GY	7B	AX.89525378	7.24E+08	0.20	-3.09	3.55	0.05
E1	GY	7B	AX.89356144	7.09E+08	0.43	2.38	3.27	0.06
E1	GY	7B	AX.89326836	6.11E+08	0.08	-4.79	4.01	0.06
E1	GY	7B	AX.89397065	7.09E+08	0.43	2.32	3.13	0.06
E1	GY	7B	AX.89744030	7.09E+08	0.43	2.38	3.27	0.06
E1	GY	7B	AX.89422373	3.38E+07	0.08	-4.53	3.49	0.07
E1	GY	7B	AX.89422373	3.38E+07	0.08	-4.53	3.49	0.07
E1	GY	7B	AX.89535574	7.09E+08	0.15	-3.91	4.36	0.08
E1	GY	7B	AX.89376058	7.09E+08	0.40	2.35	3.15	0.06
E1	GY	7B	AX.89539778	7.09E+08	0.43	2.38	3.27	0.06
E1	GY	7B	AX.89539778	7.09E+08	0.43	2.38	3.27	0.06
E1	GY	7B	AX.89439281	4.32E+06	0.20	-2.83	3.01	0.05
E1	GY	7B	AX.89365162	7.23E+08	0.20	-3.10	3.51	0.05
E1	GY	7B	AX.89541447	6.78E+07	0.07	-5.35	3.99	0.06
E1	GY	7B	AX.89541447	6.78E+07	0.07	-5.35	3.99	0.06
E1	GY	7B	AX.89389066	7.24E+08	0.20	-3.09	3.55	0.05
E1	GY	7B	AX.89438204	3.39E+07	0.08	-4.35	3.25	0.06
E1	GY	7B	AX.89713490	7.09E+08	0.11	-4.11	3.72	0.07
E1	GY	7B	AX.89450411	7.11E+08	0.34	-2.47	3.23	0.06
E1	GY	7B	AX.89450411	7.11E+08	0.34	-2.47	3.23	0.06
E1	GY	7B	AX.89344849	7.22E+08	0.28	-2.74	3.55	0.05
E1	GY	7B	AX.89651495	6.11E+08	0.08	-4.79	4.01	0.06
E1	GY	7B	AX.89770281	7.22E+08	0.28	-2.73	3.50	0.05
E1	GY	7B	AX.89439688	3.18E+07	0.05	-5.94	3.62	0.07
E1	GY	7B	AX.89653289	7.11E+08	0.34	-2.38	3.06	0.06
E1	GY	7B	AX.89439206	7.09E+08	0.29	-2.57	3.21	0.05
E1	GY	7B	AX.89537754	7.22E+08	0.28	-2.73	3.50	0.05
E1	GY	7B	AX.89628690	3.39E+07	0.07	-4.52	3.31	0.06
E1	GY	7B	AX.89496578	4.89E+06	0.20	-2.83	3.01	0.05
E1	GY	7B	AX.89629742	7.09E+08	0.43	2.38	3.27	0.06
E1	GY	7B	AX.89525378	7.24E+08	0.20	-3.09	3.55	0.05
E1	GY	7B	AX.89604264	6.11E+08	0.09	-4.69	4.03	0.06
E1	GY	7B	AX.89373466	3.38E+07	0.09	-4.14	3.26	0.06
E1	GY	7B	AX.89537754	7.22E+08	0.28	-2.73	3.50	0.05
E1	SPM2	7B	AX.89502259	1.26E+06	0.44	18.34	3.68	0.07
E1	GY	7B	AX.89744299	4.90E+06	0.20	-2.83	3.01	0.05
E1	GY	7B	AX.89721401	3.40E+07	0.08	-4.35	3.25	0.06
E1	GY	7B	AX.89539917	7.23E+08	0.18	-3.05	3.21	0.05
E1	GY	7B	AX.89356144	7.09E+08	0.43	2.38	3.27	0.06
E1	GY	7B	AX.89387103	7.23E+08	0.26	-2.63	3.16	0.05
E1	GY	7B	AX.89518585	7.24E+08	0.18	-2.97	3.06	0.04
E1	GY	7B	AX.89353636	7.09E+08	0.43	2.27	3.01	0.05
E1	GY	7B	AX.89636284	7.09E+08	0.43	2.38	3.27	0.06
E1	GY	7B	AX.89636284	7.09E+08	0.43	2.38	3.27	0.06
E1	GY	7B	AX.89711907	7.24E+08	0.20	-3.10	3.51	0.05
E1	GY	7B	AX.89402723	5.95E+08	0.13	-3.61	3.35	0.06

E1	GY	7B	AX.89507123	7.14E+08	0.46	-2.36	3.25	0.05
E1	GY	7B	AX.89355748	4.90E+06	0.20	-2.83	3.01	0.05
E1	GY	7B	AX.89638464	7.13E+08	0.43	-2.38	3.26	0.05
E1	GY	7B	AX.89638464	7.13E+08	0.43	-2.38	3.26	0.05
E1	GY	7B	AX.89532964	7.18E+08	0.37	-2.35	3.08	0.05
E1	GY	7B	AX.89532964	7.18E+08	0.37	-2.35	3.08	0.05
E1	GY	7B	AX.89749617	7.09E+08	0.43	2.38	3.27	0.06
E1	GY	7B	AX.89580565	7.09E+08	0.43	2.32	3.13	0.06
E1	GY	7B	AX.89463761	7.09E+08	0.44	2.48	3.53	0.07
E1	GY	7B	AX.89618270	4.40E+06	0.20	-2.83	3.01	0.05
E1	GY	7B	AX.89442633	7.22E+08	0.20	-3.24	3.79	0.06
E1	GY	7B	AX.89500899	7.22E+08	0.28	-2.74	3.55	0.05
E1	GY	7B	AX.89348599	1.11E+08	0.15	-3.85	4.34	0.07
E1	GY	7B	AX.89567542	2.43E+07	0.06	-5.02	3.15	0.06
E1	GY	7B	AX.89397065	7.09E+08	0.43	2.32	3.13	0.06
E1	GY	7B	AX.89385605	7.24E+08	0.20	-3.10	3.51	0.05
E1	GY	7B	AX.89621133	7.09E+08	0.34	-2.64	3.63	0.06
E1	GY	7B	AX.89621133	7.09E+08	0.34	-2.64	3.63	0.06
E1	GY	7B	AX.89375018	7.14E+08	0.44	-2.47	3.49	0.06
E1	GY	7B	AX.89503859	3.39E+07	0.08	-4.35	3.25	0.06
E1	GY	7B	AX.89433973	3.40E+07	0.08	-4.35	3.25	0.06
E1	GY	7B	AX.89433973	3.40E+07	0.08	-4.35	3.25	0.06
E1	GY	7B	AX.89755027	7.18E+08	0.25	-2.85	3.51	0.05
E1	GY	7B	AX.89633557	7.14E+08	0.44	-2.35	3.22	0.05
E1	GY	7B	AX.89744299	4.90E+06	0.20	-2.83	3.01	0.05
E1	GY	7B	AX.89646228	7.13E+08	0.44	-2.47	3.49	0.06
E1	GY	7B	AX.89529191	4.90E+06	0.20	-2.83	3.01	0.05
E1	GY	7B	AX.89529191	4.90E+06	0.20	-2.83	3.01	0.05
E1	SPM2	7B	AX.89389706	6.08E+07	0.05	36.94	3.04	0.05
E1	GY	7B	AX.89610838	7.09E+08	0.43	2.38	3.27	0.06
E1	GY	7B	AX.89365162	7.23E+08	0.20	-3.10	3.51	0.05
E1	GY	7B	AX.89389066	7.24E+08	0.20	-3.09	3.55	0.05
E1	GY	7B	AX.89734790	3.39E+07	0.08	-4.35	3.25	0.06
E1	GY	7B	AX.89732305	7.22E+08	0.28	-2.74	3.55	0.05
E1	GY	7B	AX.89355748	4.90E+06	0.20	-2.83	3.01	0.05
E1	GY	7B	AX.89656107	7.22E+08	0.28	-2.74	3.55	0.05
E1	GY	7B	AX.89677410	5.95E+08	0.14	-3.20	3.01	0.05
E1	GY	7B	AX.89653289	7.11E+08	0.34	-2.38	3.06	0.06
E1	SPM2	7B	AX.89398674	6.43E+05	0.38	-17.89	3.38	0.06
E1	GY	7B	AX.89431025	7.14E+08	0.49	-2.73	4.21	0.07
E1	GY	7B	AX.89500899	7.22E+08	0.28	-2.74	3.55	0.05
E1	GPS	7B	AX.89516048	1.45E+08	0.19	-1.41	3.07	0.05
E1	GY	7B	AX.89555322	7.09E+08	0.43	2.38	3.27	0.06
E1	GY	7B	AX.89432264	3.39E+07	0.08	-4.25	3.27	0.06
E1	SPM2	7B	AX.89540727	7.10E+08	0.30	-18.70	3.33	0.06
E1	GY	7B	AX.89694858	3.40E+07	0.08	-4.35	3.25	0.06
E1	GY	7B	AX.89694858	3.40E+07	0.08	-4.35	3.25	0.06
E1	GY	7B	AX.89375018	7.14E+08	0.44	-2.47	3.49	0.06
E1	GY	7B	AX.89622108	3.39E+07	0.08	-4.35	3.25	0.06
E1	GY	7B	AX.89622108	3.39E+07	0.08	-4.35	3.25	0.06

E1	GPS	7B	AX.89337128	1.46E+08	0.19	-1.41	3.07	0.05
E1	GY	7B	AX.89505290	7.14E+08	0.49	-2.66	4.04	0.07
E1	GY	7B	AX.89611802	7.24E+08	0.20	-3.08	3.59	0.05
E1	GY	7B	AX.89518585	7.24E+08	0.18	-2.97	3.06	0.04
E1	GY	7B	AX.89631359	7.09E+08	0.43	2.27	3.01	0.05
E1	GY	7B	AX.89713490	7.09E+08	0.11	-4.11	3.72	0.07
E1	GY	7B	AX.89687662	4.90E+06	0.20	-2.83	3.01	0.05
E1	GY	7B	AX.89542111	7.22E+08	0.20	-3.10	3.51	0.05
E1	GPS	7B	AX.89548310	6.31E+07	0.06	-2.40	3.04	0.05
E1	GY	7B	AX.89651495	6.11E+08	0.08	-4.79	4.01	0.06
E1	GY	7B	AX.89489151	3.39E+07	0.08	-4.35	3.25	0.06
E1	GY	7B	AX.89489151	3.39E+07	0.08	-4.35	3.25	0.06
E1	GPS	7B	AX.89333929	6.31E+07	0.06	-2.40	3.04	0.05
E1	GY	7B	AX.89629742	7.09E+08	0.43	2.38	3.27	0.06
E1	GY	7B	AX.89594740	6.11E+08	0.08	-4.79	4.01	0.06
E1	GY	7B	AX.89373466	3.38E+07	0.09	-4.14	3.26	0.06
E1	GY	7B	AX.89385605	7.24E+08	0.20	-3.10	3.51	0.05
E1	GY	7B	AX.89732098	7.22E+08	0.28	-2.73	3.50	0.05
E1	GY	7B	AX.89493054	7.13E+08	0.44	-2.47	3.49	0.06
E1	GY	7B	AX.89519751	3.39E+07	0.07	-4.89	3.79	0.07
E1	GY	7B	AX.89672581	6.11E+08	0.09	-4.38	3.59	0.06
E1	GY	7B	AX.89438204	3.39E+07	0.08	-4.35	3.25	0.06
E1	GY	7B	AX.89675811	3.38E+07	0.09	-4.02	3.22	0.06
E1	GY	7B	AX.89675811	3.38E+07	0.09	-4.02	3.22	0.06
E1	GY	7B	AX.89618270	4.40E+06	0.20	-2.83	3.01	0.05
E1	GY	7B	AX.89348599	1.11E+08	0.15	-3.85	4.34	0.07
E1	GY	7B	AX.89744030	7.09E+08	0.43	2.38	3.27	0.06
E1	GY	7B	AX.89422790	7.09E+08	0.46	2.55	3.73	0.07
E1	GY	7B	AX.89539917	7.23E+08	0.18	-3.05	3.21	0.05
E1	GY	7B	AX.89505290	7.14E+08	0.49	-2.66	4.04	0.07
E1	GY	7B	AX.89611802	7.24E+08	0.20	-3.08	3.59	0.05
E1	GPM2	7B	AX.89438204	3.39E+07	0.08	-1079.90	3.09	0.05
E1	GY	7B	AX.89507637	4.90E+06	0.20	-2.83	3.01	0.05
E1	GY	7B	AX.89507637	4.90E+06	0.20	-2.83	3.01	0.05
E1	GPM2	7B	AX.89567542	2.43E+07	0.06	-1446.14	3.86	0.07
E1	GPM2	7B	AX.89503859	3.39E+07	0.08	-1079.90	3.09	0.05
E1	GY	7B	AX.89677410	5.95E+08	0.14	-3.20	3.01	0.05
E1	GPM2	7B	AX.89455659	6.78E+07	0.07	-1399.10	4.13	0.07
E1	GY	7B	AX.89607120	7.09E+08	0.19	-3.24	3.67	0.06
E1	GY	7B	AX.89607120	7.09E+08	0.19	-3.24	3.67	0.06
E1	GY	7B	AX.89431025	7.14E+08	0.49	-2.73	4.21	0.07
E1	GPM2	7B	AX.89400804	7.13E+08	0.20	726.36	3.03	0.05
E1	GPM2	7B	AX.89628690	3.39E+07	0.07	-1105.99	3.07	0.05
E1	GY	7B	AX.89555322	7.09E+08	0.43	2.38	3.27	0.06
E1	GPM2	7B	AX.89489151	3.39E+07	0.08	-1079.90	3.09	0.05
E1	GPM2	7B	AX.89656107	7.22E+08	0.28	-641.29	3.05	0.05
E1	GY	7B	AX.89631359	7.09E+08	0.43	2.27	3.01	0.05
E1	GY	7B	AX.89439206	7.09E+08	0.29	-2.57	3.21	0.05
E1	GPM2	7B	AX.89431025	7.14E+08	0.49	-612.31	3.36	0.05
E1	GY	7B	AX.89542111	7.22E+08	0.20	-3.10	3.51	0.05

E1	GPM2	7B	AX.89422790	7.09E+08	0.46	604.11	3.26	0.06
E1	GPM2	7B	AX.89653289	7.11E+08	0.34	-642.87	3.35	0.06
E1	GY	7B	AX.89496578	4.89E+06	0.20	-2.83	3.01	0.05
E1	GPS	7B	AX.89603869	6.30E+07	0.06	-2.40	3.04	0.05
E1	GPM2	7B	AX.89739191	7.11E+08	0.27	-703.18	3.47	0.07
E1	GY	7B	AX.89567542	2.43E+07	0.06	-5.02	3.15	0.06
E1	GY	7B	AX.89604264	6.11E+08	0.09	-4.69	4.03	0.06
E1	GPM2	7B	AX.89614268	7.13E+08	0.17	763.96	3.03	0.05
E1	GPM2	7B	AX.89439688	3.18E+07	0.05	-1372.55	3.05	0.06
E1	GY	7B	AX.89493054	7.13E+08	0.44	-2.47	3.49	0.06
E1	GY	7B	AX.89519751	3.39E+07	0.07	-4.89	3.79	0.07
E1	GPM2	7B	AX.89721401	3.40E+07	0.08	-1079.90	3.09	0.05
E1	GY	7B	AX.89672581	6.11E+08	0.09	-4.38	3.59	0.06
E1	GPM2	7B	AX.89519751	3.39E+07	0.07	-1165.63	3.36	0.06
E1	GPM2	7B	AX.89501087	7.10E+08	0.27	-682.45	3.27	0.06
E1	GPM2	7B	AX.89463325	6.07E+08	0.35	-611.54	3.11	0.06
E1	GY	7B	AX.89711907	7.24E+08	0.20	-3.10	3.51	0.05
E1	GY	7B	AX.89507123	7.14E+08	0.46	-2.36	3.25	0.05
E1	GY	7B	AX.89749617	7.09E+08	0.43	2.38	3.27	0.06
E1	GY	7B	AX.89580565	7.09E+08	0.43	2.32	3.13	0.06
E1	GY	7B	AX.89755027	7.18E+08	0.25	-2.85	3.51	0.05
E1	GY	7B	AX.89646228	7.13E+08	0.44	-2.47	3.49	0.06
E1	GPM2	7B	AX.89505290	7.14E+08	0.49	-591.90	3.17	0.05
E1	GPM2	7B	AX.89734790	3.39E+07	0.08	-1079.90	3.09	0.05
E1	GPM2	7B	AX.89433973	3.40E+07	0.08	-1079.90	3.09	0.05
E1	GY	7B	AX.89656107	7.22E+08	0.28	-2.74	3.55	0.05
E1	GPM2	7B	AX.89524406	7.10E+08	0.27	-682.45	3.27	0.06
E1	GPM2	7B	AX.89368338	7.10E+08	0.32	-679.92	3.57	0.07
E1	GPM2	7B	AX.89535574	7.09E+08	0.15	-955.49	4.03	0.07
E1	GPM2	7B	AX.89450411	7.11E+08	0.34	-641.16	3.31	0.06
E1	GPM2	7B	AX.89622108	3.39E+07	0.08	-1079.90	3.09	0.05
E1	GY	7B	AX.89668000	4.90E+06	0.20	-2.83	3.01	0.05
E1	GY	7B	AX.89668000	4.90E+06	0.20	-2.83	3.01	0.05
E1	GY	7B	AX.89432264	3.39E+07	0.08	-4.25	3.27	0.06
E1	GPM2	7B	AX.89422825	6.02E+08	0.32	760.93	4.35	0.08
E1	GPM2	7B	AX.89413520	7.10E+08	0.27	-682.45	3.27	0.06
E1	GPM2	7B	AX.89542430	7.13E+08	0.26	698.55	3.33	0.06
E1	GPM2	7B	AX.89713490	7.09E+08	0.11	-1025.95	3.57	0.06
E1	GPM2	7B	AX.89568763	7.13E+08	0.17	763.96	3.03	0.05
E1	GPM2	7B	AX.89694858	3.40E+07	0.08	-1079.90	3.09	0.05
E1	GPM2	7B	AX.89456148	7.11E+08	0.27	-682.45	3.27	0.06
E1	GY	7B	AX.89732305	7.22E+08	0.28	-2.74	3.55	0.05
E1	GPM2	7B	AX.89489457	7.10E+08	0.27	-682.45	3.27	0.06
E1	GPM2	7B	AX.89442811	7.10E+08	0.26	-740.93	3.73	0.07
E1	GPM2	7B	AX.89376058	7.09E+08	0.40	586.38	3.01	0.05
E1	TKW	7B	AX.89333426	1.11E+08	0.11	-1.45	3.46	0.06
E1	TKW	7B	AX.89312200	6.42E+08	0.12	-1.30	3.03	0.05
E1	GPM2	7B	AX.89692589	7.10E+08	0.27	-682.45	3.27	0.06
E1	GY	7B	AX.89422448	7.11E+08	0.34	-2.49	3.28	0.06
E1	TKW	7B	AX.89601766	6.42E+08	0.12	-1.30	3.03	0.05

E1	GY	7B	AX.89721401	3.40E+07	0.08	-4.35	3.25	0.06
E1	GPM2	7B	AX.89732305	7.22E+08	0.28	-641.29	3.05	0.05
E1	TKW	7B	AX.89352844	6.42E+08	0.12	-1.30	3.03	0.05
E1	GPM2	7B	AX.89684378	7.11E+08	0.27	-682.45	3.27	0.06
E1	GPM2	7B	AX.89692354	7.11E+08	0.27	-682.45	3.27	0.06
E1	TKW	7B	AX.89479069	6.42E+08	0.12	-1.30	3.03	0.05
E1	TKW	7B	AX.89481912	4.49E+07	0.07	-1.64	3.07	0.05
E1	TKW	7B	AX.89357125	6.42E+08	0.12	-1.30	3.03	0.05
E1	GY	7B	AX.89439281	4.32E+06	0.20	-2.83	3.01	0.05
E1	GPM2	7B	AX.89344849	7.22E+08	0.28	-641.29	3.05	0.05
E1	TKW	7B	AX.89333210	6.42E+08	0.12	-1.30	3.14	0.05
E1	TKW	7B	AX.89575426	6.43E+08	0.12	-1.30	3.03	0.05
E1	TKW	7B	AX.89513518	6.43E+08	0.11	-1.43	3.49	0.06
E1	TKW	7B	AX.89462633	5.94E+08	0.05	-1.89	3.04	0.05
E1	TKW	7B	AX.89466097	6.43E+08	0.12	-1.30	3.03	0.05
E1	TKW	7B	AX.89540157	6.43E+08	0.13	-1.33	3.54	0.06
E1	TKW	7B	AX.89667167	6.43E+08	0.12	-1.34	3.29	0.05
E1	TKW	7B	AX.89468928	6.42E+08	0.12	-1.30	3.03	0.05
E1	TKW	7B	AX.89472431	6.43E+08	0.12	-1.30	3.03	0.05
E1	GPM2	7B	AX.89541447	6.78E+07	0.07	-1399.10	4.13	0.07
E1	GPM2	7B	AX.89532644	7.18E+08	0.07	1452.43	4.41	0.08
E1	TKW	7B	AX.89497058	6.43E+08	0.13	-1.33	3.54	0.06
E1	TKW	7B	AX.89436577	6.43E+08	0.12	-1.30	3.03	0.05
E1	GPM2	7B	AX.89422448	7.11E+08	0.34	-664.57	3.52	0.07
E1	TKW	7B	AX.89667248	6.43E+08	0.12	-1.30	3.03	0.05
E1	TKW	7B	AX.89731988	5.94E+08	0.05	-1.89	3.04	0.05
E1	TKW	7B	AX.89551431	6.43E+08	0.11	-1.43	3.49	0.06
E1	TKW	7B	AX.89712015	6.43E+08	0.12	-1.30	3.03	0.05
E1	TKW	7B	AX.89354142	6.43E+08	0.13	-1.33	3.54	0.06
E1	TKW	7B	AX.89654737	6.42E+08	0.12	-1.30	3.03	0.05
E1	TKW	7B	AX.89736925	6.43E+08	0.13	-1.33	3.35	0.06
E1	TKW	7B	AX.89705146	6.42E+08	0.12	-1.30	3.03	0.05
E1	TKW	7B	AX.89473885	6.43E+08	0.12	-1.30	3.03	0.05
E1	TKW	7B	AX.89545842	6.43E+08	0.12	-1.30	3.03	0.05
E2	GY	7B	AX.89399705	1.24E+08	0.20	-2.79	4.06	0.10
E2	GY	7B	AX.89476659	1.33E+08	0.19	-2.71	3.76	0.07
E2	GY	7B	AX.89329065	1.26E+08	0.21	-2.34	3.12	0.08
E2	GY	7B	AX.89573863	1.34E+08	0.22	-2.45	3.52	0.09
E2	GY	7B	AX.89721479	1.24E+08	0.20	-2.39	3.12	0.07
E2	GY	7B	AX.89610890	1.25E+08	0.20	-2.80	4.13	0.10
E2	GY	7B	AX.89352521	3.31E+07	0.28	-2.10	3.04	0.05
E2	GY	7B	AX.89435587	5.91E+07	0.06	-4.22	3.18	0.04
E2	SPM2	7B	AX.89715777	6.41E+08	0.07	25.80	3.53	0.06
E2	GY	7B	AX.89401143	1.24E+08	0.20	-2.79	4.06	0.10
E2	GY	7B	AX.89330300	1.24E+08	0.22	-2.49	3.56	0.08
E2	GY	7B	AX.89436335	1.34E+08	0.22	-2.45	3.52	0.09
E2	GY	7B	AX.89436335	1.34E+08	0.22	-2.45	3.52	0.09
E2	GY	7B	AX.89354117	1.25E+08	0.20	-2.80	4.13	0.10
E2	GY	7B	AX.89724012	1.25E+08	0.20	-2.69	3.85	0.09
E2	GY	7B	AX.89590117	1.24E+08	0.20	-2.79	4.06	0.10

E2	GY	7B	AX.89577587	1.33E+08	0.25	-2.21	3.12	0.08
E2	GY	7B	AX.89332784	1.25E+08	0.20	-2.44	3.22	0.08
E2	SPM2	7B	AX.89618651	6.76E+08	0.25	15.87	4.05	0.07
E2	GY	7B	AX.89439688	3.18E+07	0.05	-4.77	3.39	0.08
E2	GY	7B	AX.89312842	1.24E+08	0.20	-2.38	3.04	0.07
E2	GY	7B	AX.89653266	1.27E+08	0.21	-2.45	3.36	0.09
E2	GY	7B	AX.89546755	7.20E+08	0.06	-4.64	3.76	0.04
E2	GY	7B	AX.89337128	1.46E+08	0.19	-2.40	3.05	0.04
E2	GY	7B	AX.89409124	1.24E+08	0.20	-2.79	4.06	0.10
E2	GY	7B	AX.89409124	1.24E+08	0.20	-2.79	4.06	0.10
E2	GY	7B	AX.89548327	1.25E+08	0.20	-2.44	3.22	0.08
E2	GY	7B	AX.89548540	1.24E+08	0.20	-2.79	4.06	0.10
E2	GY	7B	AX.89572322	1.25E+08	0.20	-2.80	4.13	0.10
E2	SPM2	7B	AX.89739917	1.12E+08	0.10	19.73	3.07	0.05
E2	GPS	7B	AX.89385605	7.24E+08	0.20	-1.68	3.10	0.05
E2	GY	7B	AX.89329116	1.25E+08	0.20	-2.46	3.30	0.08
E2	GY	7B	AX.89329116	1.25E+08	0.20	-2.46	3.30	0.08
E2	GY	7B	AX.89732724	1.28E+08	0.19	-2.50	3.31	0.09
E2	GY	7B	AX.89732724	1.28E+08	0.19	-2.50	3.31	0.09
E2	GY	7B	AX.89517643	1.25E+08	0.20	-2.80	4.13	0.10
E2	GY	7B	AX.89517643	1.25E+08	0.20	-2.80	4.13	0.10
E2	GY	7B	AX.89752544	1.25E+08	0.20	-2.58	3.58	0.09
E2	GY	7B	AX.89737247	1.25E+08	0.20	-2.80	4.13	0.10
E2	GY	7B	AX.89737247	1.25E+08	0.20	-2.80	4.13	0.10
E2	GY	7B	AX.89531537	1.25E+08	0.20	-2.59	3.55	0.09
E2	GY	7B	AX.89341648	1.27E+08	0.21	-2.30	3.02	0.08
E2	GY	7B	AX.89341648	1.27E+08	0.21	-2.30	3.02	0.08
E2	GY	7B	AX.89377678	1.55E+07	0.05	-5.62	4.75	0.07
E2	GY	7B	AX.89377678	1.55E+07	0.05	-5.62	4.75	0.07
E2	GY	7B	AX.89519392	1.25E+08	0.20	-2.44	3.22	0.08
E2	GY	7B	AX.89519392	1.25E+08	0.20	-2.44	3.22	0.08
E2	GY	7B	AX.86184396	1.25E+08	0.20	-2.97	4.52	0.11
E2	GY	7B	AX.86184396	1.25E+08	0.20	-2.97	4.52	0.11
E2	GY	7B	AX.89699057	1.34E+08	0.22	-2.45	3.52	0.09
E2	GY	7B	AX.89699057	1.34E+08	0.22	-2.45	3.52	0.09
E2	GY	7B	AX.86184465	1.24E+08	0.20	-2.79	4.06	0.10
E2	GY	7B	AX.86184465	1.24E+08	0.20	-2.79	4.06	0.10
E2	GY	7B	AX.89587390	1.33E+08	0.25	-2.23	3.20	0.08
E2	GY	7B	AX.89366909	1.25E+08	0.20	-2.44	3.22	0.08
E2	GY	7B	AX.89366909	1.25E+08	0.20	-2.44	3.22	0.08
E2	GY	7B	AX.89390140	1.24E+08	0.20	-2.79	4.06	0.10
E2	GY	7B	AX.89520075	3.39E+07	0.09	-3.78	3.85	0.07
E2	GY	7B	AX.89520075	3.39E+07	0.09	-3.78	3.85	0.07
E2	GY	7B	AX.89590117	1.24E+08	0.20	-2.79	4.06	0.10
E2	GPS	7B	AX.89602085	5.50E+06	0.08	-2.40	3.09	0.05
E2	GY	7B	AX.89736611	1.25E+08	0.20	-2.80	4.13	0.10
E2	GY	7B	AX.89736611	1.25E+08	0.20	-2.80	4.13	0.10
E2	GY	7B	AX.89310532	1.34E+08	0.22	-2.45	3.52	0.09
E2	GY	7B	AX.89310532	1.34E+08	0.22	-2.45	3.52	0.09
E2	GY	7B	AX.89508992	1.43E+08	0.26	-2.23	3.24	0.08

E2	GY	7B	AX.89508992	1.43E+08	0.26	-2.23	3.24	0.08
E2	GY	7B	AX.89450310	1.25E+08	0.20	-2.44	3.22	0.08
E2	GY	7B	AX.89450310	1.25E+08	0.20	-2.44	3.22	0.08
E2	GY	7B	AX.89379822	1.34E+08	0.25	-2.21	3.12	0.08
E2	GY	7B	AX.89379822	1.34E+08	0.25	-2.21	3.12	0.08
E2	GY	7B	AX.89591697	1.24E+08	0.20	-2.38	3.04	0.07
E2	GY	7B	AX.89591697	1.24E+08	0.20	-2.38	3.04	0.07
E2	GY	7B	AX.89567542	2.43E+07	0.06	-5.18	4.54	0.09
E2	GY	7B	AX.89439563	1.24E+08	0.20	-2.79	4.06	0.10
E2	GY	7B	AX.89439563	1.24E+08	0.20	-2.79	4.06	0.10
E2	GY	7B	AX.89474156	1.34E+08	0.25	-2.21	3.12	0.08
E2	GY	7B	AX.89474156	1.34E+08	0.25	-2.21	3.12	0.08
E2	GY	7B	AX.89487687	1.34E+08	0.25	-2.21	3.12	0.08
E2	GY	7B	AX.89487687	1.34E+08	0.25	-2.21	3.12	0.08
E2	GY	7B	AX.89440438	1.34E+08	0.22	-2.45	3.47	0.09
E2	GY	7B	AX.89440438	1.34E+08	0.22	-2.45	3.47	0.09
E2	GY	7B	AX.89580183	1.34E+08	0.25	-2.21	3.12	0.08
E2	GY	7B	AX.89580183	1.34E+08	0.25	-2.21	3.12	0.08
E2	GPS	7B	AX.89631122	5.22E+06	0.18	-1.95	3.73	0.07
E2	GY	7B	AX.89312842	1.24E+08	0.20	-2.38	3.04	0.07
E2	GY	7B	AX.89370690	1.24E+08	0.20	-2.79	4.06	0.10
E2	GY	7B	AX.89370690	1.24E+08	0.20	-2.79	4.06	0.10
E2	GY	7B	AX.89620932	1.25E+08	0.20	-2.44	3.22	0.08
E2	GY	7B	AX.89452470	1.24E+08	0.20	-2.38	3.04	0.07
E2	GY	7B	AX.89452470	1.24E+08	0.20	-2.38	3.04	0.07
E2	GY	7B	AX.89546574	1.25E+08	0.20	-2.80	4.13	0.10
E2	GY	7B	AX.89546574	1.25E+08	0.20	-2.80	4.13	0.10
E2	GY	7B	AX.89524726	1.25E+08	0.20	-2.80	4.13	0.10
E2	GY	7B	AX.89524726	1.25E+08	0.20	-2.80	4.13	0.10
E2	GY	7B	AX.89581762	1.34E+08	0.22	-2.45	3.52	0.09
E2	GY	7B	AX.89581762	1.34E+08	0.22	-2.45	3.52	0.09
E2	GY	7B	AX.89525451	4.85E+07	0.07	-3.95	3.58	0.05
E2	GY	7B	AX.89525451	4.85E+07	0.07	-3.95	3.58	0.05
E2	GY	7B	AX.89348537	1.35E+08	0.26	-2.24	3.24	0.08
E2	GY	7B	AX.89348537	1.35E+08	0.26	-2.24	3.24	0.08
E2	GY	7B	AX.89348599	1.11E+08	0.15	-3.31	4.61	0.07
E2	GY	7B	AX.89664539	1.25E+08	0.20	-2.80	4.13	0.10
E2	GY	7B	AX.89664539	1.25E+08	0.20	-2.80	4.13	0.10
E2	GY	7B	AX.89619603	1.26E+08	0.21	-2.34	3.12	0.08
E2	GY	7B	AX.89491054	1.34E+08	0.24	-2.30	3.26	0.08
E2	GY	7B	AX.89491054	1.34E+08	0.24	-2.30	3.26	0.08
E2	GY	7B	AX.89373417	1.26E+08	0.21	-2.34	3.12	0.08
E2	GY	7B	AX.89373417	1.26E+08	0.21	-2.34	3.12	0.08
E2	GY	7B	AX.89631679	1.36E+08	0.18	-2.61	3.41	0.05
E2	GY	7B	AX.89631679	1.36E+08	0.18	-2.61	3.41	0.05
E2	GY	7B	AX.89560711	1.34E+08	0.23	-2.46	3.60	0.09
E2	GY	7B	AX.89560711	1.34E+08	0.23	-2.46	3.60	0.09
E2	GY	7B	AX.89596775	1.34E+08	0.22	-2.45	3.52	0.09
E2	GY	7B	AX.89596775	1.34E+08	0.22	-2.45	3.52	0.09
E2	GY	7B	AX.89669241	3.38E+07	0.09	-3.78	3.85	0.07

E2	GY	7B	AX.89669241	3.38E+07	0.09	-3.78	3.85	0.07
E2	GY	7B	AX.89316726	1.34E+08	0.25	-2.21	3.12	0.08
E2	GY	7B	AX.89316726	1.34E+08	0.25	-2.21	3.12	0.08
E2	GY	7B	AX.89580035	1.25E+08	0.19	-2.54	3.39	0.08
E2	GY	7B	AX.89580035	1.25E+08	0.19	-2.54	3.39	0.08
E2	GY	7B	AX.89561410	1.25E+08	0.20	-2.59	3.55	0.09
E2	GY	7B	AX.89561410	1.25E+08	0.20	-2.59	3.55	0.09
E2	GY	7B	AX.89422101	1.25E+08	0.20	-2.44	3.22	0.08
E2	GY	7B	AX.89422101	1.25E+08	0.20	-2.44	3.22	0.08
E2	GY	7B	AX.89599341	1.25E+08	0.20	-2.80	4.13	0.10
E2	GY	7B	AX.89599341	1.25E+08	0.20	-2.80	4.13	0.10
E2	GY	7B	AX.89386589	1.34E+08	0.22	-2.45	3.52	0.09
E2	GY	7B	AX.89386589	1.34E+08	0.22	-2.45	3.52	0.09
E2	GY	7B	AX.89329065	1.26E+08	0.21	-2.34	3.12	0.08
E2	GY	7B	AX.89457197	2.58E+06	0.05	-4.52	3.04	0.07
E2	GY	7B	AX.89457197	2.58E+06	0.05	-4.52	3.04	0.07
E2	GY	7B	AX.89671906	1.35E+08	0.17	-2.61	3.31	0.06
E2	GY	7B	AX.89671906	1.35E+08	0.17	-2.61	3.31	0.06
E2	GY	7B	AX.89752544	1.25E+08	0.20	-2.58	3.58	0.09
E2	GY	7B	AX.89341213	1.25E+08	0.20	-2.80	4.13	0.10
E2	GY	7B	AX.89341213	1.25E+08	0.20	-2.80	4.13	0.10
E2	GY	7B	AX.89531537	1.25E+08	0.20	-2.59	3.55	0.09
E2	GY	7B	AX.89724261	1.25E+08	0.20	-2.41	3.14	0.08
E2	GY	7B	AX.89424182	1.28E+08	0.18	-2.48	3.13	0.08
E2	GY	7B	AX.89424182	1.28E+08	0.18	-2.48	3.13	0.08
E2	GY	7B	AX.89600751	1.25E+08	0.20	-2.44	3.22	0.08
E2	GY	7B	AX.89600751	1.25E+08	0.20	-2.44	3.22	0.08
E2	GY	7B	AX.89673439	3.39E+07	0.09	-3.78	3.85	0.07
E2	GY	7B	AX.89673439	3.39E+07	0.09	-3.78	3.85	0.07
E2	GY	7B	AX.89575968	1.55E+07	0.05	-5.62	4.75	0.07
E2	GY	7B	AX.89575968	1.55E+07	0.05	-5.62	4.75	0.07
E2	GY	7B	AX.89698438	1.25E+08	0.20	-2.80	4.13	0.10
E2	GY	7B	AX.89698438	1.25E+08	0.20	-2.80	4.13	0.10
E2	GY	7B	AX.89641180	1.25E+08	0.20	-2.80	4.13	0.10
E2	GY	7B	AX.89641180	1.25E+08	0.20	-2.80	4.13	0.10
E2	GY	7B	AX.89320399	1.34E+08	0.22	-2.45	3.52	0.09
E2	GY	7B	AX.89320399	1.34E+08	0.22	-2.45	3.52	0.09
E2	GY	7B	AX.89320512	1.34E+08	0.22	-2.45	3.52	0.09
E2	GY	7B	AX.89320512	1.34E+08	0.22	-2.45	3.52	0.09
E2	GY	7B	AX.89485021	1.24E+08	0.20	-2.79	4.06	0.10
E2	GY	7B	AX.89485021	1.24E+08	0.20	-2.79	4.06	0.10
E2	GY	7B	AX.89674367	3.38E+07	0.09	-3.78	3.85	0.07
E2	GY	7B	AX.89674367	3.38E+07	0.09	-3.78	3.85	0.07
E2	GY	7B	AX.89587390	1.33E+08	0.25	-2.23	3.20	0.08
E2	GY	7B	AX.89390140	1.24E+08	0.20	-2.79	4.06	0.10
E2	GY	7B	AX.89611605	1.24E+08	0.20	-2.79	4.06	0.10
E2	GY	7B	AX.89611605	1.24E+08	0.20	-2.79	4.06	0.10
E2	GY	7B	AX.89675811	3.38E+07	0.09	-3.63	3.74	0.07
E2	GY	7B	AX.89675811	3.38E+07	0.09	-3.63	3.74	0.07
E2	GY	7B	AX.89603705	1.33E+08	0.22	-2.45	3.52	0.09

E2	GY	7B	AX.89603705	1.33E+08	0.22	-2.45	3.52	0.09
E2	GY	7B	AX.89567542	2.43E+07	0.06	-5.18	4.54	0.09
E2	GY	7B	AX.89688646	1.34E+08	0.25	-2.23	3.20	0.08
E2	GY	7B	AX.89688646	1.34E+08	0.25	-2.23	3.20	0.08
E2	GY	7B	AX.89738607	1.55E+07	0.05	-5.62	4.75	0.07
E2	GY	7B	AX.89738607	1.55E+07	0.05	-5.62	4.75	0.07
E2	GY	7B	AX.89440321	1.25E+08	0.20	-2.80	4.13	0.10
E2	GY	7B	AX.89440321	1.25E+08	0.20	-2.80	4.13	0.10
E2	GY	7B	AX.89357472	1.25E+08	0.20	-2.44	3.22	0.08
E2	GY	7B	AX.89357472	1.25E+08	0.20	-2.44	3.22	0.08
E2	GY	7B	AX.89605378	1.34E+08	0.25	-2.21	3.12	0.08
E2	GY	7B	AX.89605378	1.34E+08	0.25	-2.21	3.12	0.08
E2	GY	7B	AX.89620932	1.25E+08	0.20	-2.44	3.22	0.08
E2	GY	7B	AX.89739195	1.34E+08	0.22	-2.45	3.52	0.09
E2	GY	7B	AX.89430386	1.24E+08	0.20	-2.38	3.04	0.07
E2	GY	7B	AX.89430386	1.24E+08	0.20	-2.38	3.04	0.07
E2	GY	7B	AX.89752029	1.24E+08	0.21	-2.39	3.24	0.08
E2	GY	7B	AX.89752029	1.24E+08	0.21	-2.39	3.24	0.08
E2	GY	7B	AX.89607239	1.34E+08	0.22	-2.45	3.52	0.09
E2	GY	7B	AX.89607239	1.34E+08	0.22	-2.45	3.52	0.09
E2	GY	7B	AX.89500431	1.25E+08	0.20	-2.80	4.13	0.10
E2	GY	7B	AX.89500431	1.25E+08	0.20	-2.80	4.13	0.10
E2	GY	7B	AX.89348599	1.11E+08	0.15	-3.31	4.61	0.07
E2	GY	7B	AX.89465750	1.34E+08	0.22	-2.45	3.52	0.09
E2	GY	7B	AX.89465750	1.34E+08	0.22	-2.45	3.52	0.09
E2	GY	7B	AX.89619603	1.26E+08	0.21	-2.34	3.12	0.08
E2	GY	7B	AX.89548540	1.24E+08	0.20	-2.79	4.06	0.10
E2	GY	7B	AX.89723127	1.25E+08	0.20	-2.80	4.13	0.10
E2	GY	7B	AX.89723127	1.25E+08	0.20	-2.80	4.13	0.10
E2	GY	7B	AX.89433400	1.25E+08	0.20	-2.46	3.30	0.08
E2	GY	7B	AX.89433400	1.25E+08	0.20	-2.46	3.30	0.08
E2	GY	7B	AX.89609177	1.33E+08	0.25	-2.21	3.12	0.08
E2	GY	7B	AX.89609177	1.33E+08	0.25	-2.21	3.12	0.08
E2	GY	7B	AX.89467398	1.25E+08	0.20	-2.80	4.13	0.10
E2	GY	7B	AX.89467398	1.25E+08	0.20	-2.80	4.13	0.10
E2	GY	7B	AX.89523827	1.25E+08	0.20	-2.80	4.13	0.10
E2	GY	7B	AX.89523827	1.25E+08	0.20	-2.80	4.13	0.10
E2	GY	7B	AX.89328181	1.24E+08	0.20	-2.79	4.06	0.10
E2	GY	7B	AX.89328181	1.24E+08	0.20	-2.79	4.06	0.10
E2	GY	7B	AX.89621414	9.93E+07	0.05	-4.80	3.72	0.08
E2	GY	7B	AX.89399705	1.24E+08	0.20	-2.79	4.06	0.10
E2	GY	7B	AX.89691930	1.24E+08	0.20	-2.38	3.04	0.07
E2	GY	7B	AX.89691930	1.24E+08	0.20	-2.38	3.04	0.07
E2	GY	7B	AX.89476659	1.33E+08	0.19	-2.71	3.76	0.07
E2	GY	7B	AX.89573863	1.34E+08	0.22	-2.45	3.52	0.09
E2	GY	7B	AX.89658807	1.34E+08	0.25	-2.21	3.12	0.08
E2	GY	7B	AX.89658807	1.34E+08	0.25	-2.21	3.12	0.08
E2	GY	7B	AX.89721479	1.24E+08	0.20	-2.39	3.12	0.07
E2	GY	7B	AX.89696915	1.25E+08	0.20	-2.80	4.13	0.10
E2	GY	7B	AX.89610890	1.25E+08	0.20	-2.80	4.13	0.10

E2	GY	7B	AX.89574468	1.26E+08	0.21	-2.34	3.12	0.08
E2	GY	7B	AX.89352521	3.31E+07	0.28	-2.10	3.04	0.05
E2	GY	7B	AX.89435587	5.91E+07	0.06	-4.22	3.18	0.04
E2	GY	7B	AX.89401143	1.24E+08	0.20	-2.79	4.06	0.10
E2	GY	7B	AX.89330300	1.24E+08	0.22	-2.49	3.56	0.08
E2	GY	7B	AX.89709888	1.34E+08	0.22	-2.45	3.52	0.09
E2	GY	7B	AX.89709888	1.34E+08	0.22	-2.45	3.52	0.09
E2	GY	7B	AX.89470413	1.25E+08	0.20	-2.80	4.13	0.10
E2	GY	7B	AX.89484474	1.24E+08	0.20	-2.79	4.06	0.10
E2	GY	7B	AX.89484474	1.24E+08	0.20	-2.79	4.06	0.10
E2	TKW	7B	AX.89565708	5.97E+07	0.29	1.05	3.04	0.06
E2	GY	7B	AX.89354117	1.25E+08	0.20	-2.80	4.13	0.10
E2	GY	7B	AX.89516048	1.45E+08	0.19	-2.40	3.05	0.04
E2	GY	7B	AX.89516048	1.45E+08	0.19	-2.40	3.05	0.04
E2	GY	7B	AX.89366919	1.25E+08	0.20	-2.80	4.13	0.10
E2	GY	7B	AX.89724012	1.25E+08	0.20	-2.69	3.85	0.09
E2	GY	7B	AX.89577587	1.33E+08	0.25	-2.21	3.12	0.08
E2	GY	7B	AX.89332784	1.25E+08	0.20	-2.44	3.22	0.08
E2	GPS	7B	AX.89335596	7.01E+08	0.32	1.58	3.67	0.06
E2	GY	7B	AX.89380451	1.25E+08	0.20	-2.76	3.98	0.10
E2	GY	7B	AX.89439688	3.18E+07	0.05	-4.77	3.39	0.08
E2	GY	7B	AX.89440767	1.25E+08	0.20	-2.80	4.13	0.10
E2	GY	7B	AX.89593306	1.25E+08	0.20	-2.44	3.22	0.08
E2	GY	7B	AX.89739195	1.34E+08	0.22	-2.45	3.52	0.09
E2	GY	7B	AX.89512356	1.25E+08	0.20	-2.80	4.13	0.10
E2	GY	7B	AX.89512356	1.25E+08	0.20	-2.80	4.13	0.10
E2	GY	7B	AX.89496093	1.25E+08	0.20	-2.80	4.13	0.10
E2	GY	7B	AX.89371264	1.34E+08	0.22	-2.45	3.52	0.09
E2	GY	7B	AX.89653266	1.27E+08	0.21	-2.45	3.36	0.09
E2	GPS	7B	AX.89344849	7.22E+08	0.29	-1.55	3.37	0.05
E2	GY	7B	AX.89371848	1.25E+08	0.20	-2.80	4.13	0.10
E2	GY	7B	AX.89371848	1.25E+08	0.20	-2.80	4.13	0.10
E2	GY	7B	AX.89546755	7.20E+08	0.06	-4.64	3.76	0.04
E2	GY	7B	AX.89509174	1.25E+08	0.20	-2.80	4.13	0.10
E2	GY	7B	AX.89408294	1.25E+08	0.20	-2.44	3.22	0.08
E2	GY	7B	AX.89337128	1.46E+08	0.19	-2.40	3.05	0.04
E2	GY	7B	AX.89432264	3.39E+07	0.08	-4.19	4.43	0.08
E2	GY	7B	AX.89432264	3.39E+07	0.08	-4.19	4.43	0.08
E2	GY	7B	AX.89348742	1.24E+08	0.20	-2.79	4.06	0.10
E2	GY	7B	AX.89646582	1.34E+08	0.22	-2.45	3.52	0.09
E2	GY	7B	AX.89548327	1.25E+08	0.20	-2.44	3.22	0.08
E2	GY	7B	AX.89478960	3.39E+07	0.07	-5.05	5.01	0.10
E2	GY	7B	AX.89478960	3.39E+07	0.07	-5.05	5.01	0.10
E2	GY	7B	AX.89373466	3.38E+07	0.09	-3.64	3.60	0.07
E2	GY	7B	AX.89572322	1.25E+08	0.20	-2.80	4.13	0.10
E2	GY	7B	AX.89584578	1.43E+08	0.11	-3.43	3.69	0.07
E2	GY	7B	AX.89584578	1.43E+08	0.11	-3.43	3.69	0.07
E2	GY	7B	AX.89583303	1.25E+08	0.20	-2.80	4.13	0.10
E2	GY	7B	AX.89583303	1.25E+08	0.20	-2.80	4.13	0.10
E2	GY	7B	AX.89605701	1.24E+08	0.20	-2.79	4.06	0.10

E2	GY	7B	AX.89605701	1.24E+08	0.20	-2.79	4.06	0.10
E2	GPS	7B	AX.89387103	7.23E+08	0.26	-1.53	3.13	0.05
E2	GY	7B	AX.89445902	1.25E+08	0.20	-2.59	3.55	0.09
E2	GY	7B	AX.89445902	1.25E+08	0.20	-2.59	3.55	0.09
E2	GPS	7B	AX.89525378	7.24E+08	0.20	-1.69	3.17	0.05
E2	GPM2	7B	AX.89532713	4.19E+08	0.16	-665.07	3.13	0.05
E2	GY	7B	AX.89696915	1.25E+08	0.20	-2.80	4.13	0.10
E2	GY	7B	AX.89752529	1.25E+08	0.20	-2.80	4.13	0.10
E2	GY	7B	AX.89752529	1.25E+08	0.20	-2.80	4.13	0.10
E2	GY	7B	AX.89529615	1.24E+08	0.20	-2.38	3.04	0.07
E2	GPM2	7B	AX.89567542	2.43E+07	0.06	-1424.14	5.17	0.11
E2	GY	7B	AX.89724261	1.25E+08	0.20	-2.41	3.14	0.08
E2	GY	7B	AX.89470413	1.25E+08	0.20	-2.80	4.13	0.10
E2	GY	7B	AX.89342460	1.45E+08	0.27	-2.36	3.68	0.09
E2	GY	7B	AX.89342460	1.45E+08	0.27	-2.36	3.68	0.09
E2	GY	7B	AX.89402156	1.35E+08	0.17	-2.61	3.31	0.06
E2	GY	7B	AX.89402156	1.35E+08	0.17	-2.61	3.31	0.06
E2	GPS	7B	AX.89733307	7.23E+08	0.35	-1.40	3.08	0.04
E2	GY	7B	AX.89772656	1.25E+08	0.20	-2.80	4.13	0.10
E2	GY	7B	AX.89772656	1.25E+08	0.20	-2.80	4.13	0.10
E2	GY	7B	AX.89366919	1.25E+08	0.20	-2.80	4.13	0.10
E2	GY	7B	AX.89367004	3.38E+07	0.09	-3.78	3.84	0.07
E2	GY	7B	AX.89367004	3.38E+07	0.09	-3.78	3.84	0.07
E2	GY	7B	AX.89485700	1.26E+08	0.21	-2.34	3.12	0.08
E2	GY	7B	AX.89485700	1.26E+08	0.21	-2.34	3.12	0.08
E2	GY	7B	AX.89380451	1.25E+08	0.20	-2.76	3.98	0.10
E2	GPS	7B	AX.89500899	7.22E+08	0.29	-1.48	3.11	0.04
E2	GY	7B	AX.89496093	1.25E+08	0.20	-2.80	4.13	0.10
E2	GPM2	7B	AX.89572278	2.89E+07	0.37	-497.76	3.01	0.05
E2	GY	7B	AX.89551103	1.25E+08	0.20	-2.80	4.13	0.10
E2	GY	7B	AX.89551103	1.25E+08	0.20	-2.80	4.13	0.10
E2	GY	7B	AX.89490498	1.25E+08	0.20	-2.56	3.61	0.09
E2	GY	7B	AX.89490498	1.25E+08	0.20	-2.56	3.61	0.09
E2	GY	7B	AX.89348742	1.24E+08	0.20	-2.79	4.06	0.10
E2	GY	7B	AX.89428294	1.33E+08	0.25	-2.21	3.12	0.08
E2	GY	7B	AX.89646582	1.34E+08	0.22	-2.45	3.52	0.09
E2	GY	7B	AX.89607333	1.26E+08	0.21	-2.34	3.12	0.08
E2	GY	7B	AX.89607333	1.26E+08	0.21	-2.34	3.12	0.08
E2	GY	7B	AX.89584606	1.25E+08	0.20	-2.44	3.22	0.08
E2	GY	7B	AX.89584606	1.25E+08	0.20	-2.44	3.22	0.08
E2	GY	7B	AX.89467240	1.24E+08	0.20	-2.79	4.06	0.10
E2	GY	7B	AX.89467240	1.24E+08	0.20	-2.79	4.06	0.10
E2	GY	7B	AX.89621414	9.93E+07	0.05	-4.80	3.72	0.08
E2	GY	7B	AX.89768336	1.25E+08	0.20	-2.80	4.13	0.10
E2	GY	7B	AX.89768336	1.25E+08	0.20	-2.80	4.13	0.10
E2	GY	7B	AX.89422373	3.38E+07	0.08	-4.67	5.12	0.10
E2	GY	7B	AX.89422373	3.38E+07	0.08	-4.67	5.12	0.10
E2	GPS	7B	AX.89389066	7.24E+08	0.20	-1.69	3.17	0.05
E2	GY	7B	AX.89529615	1.24E+08	0.20	-2.38	3.04	0.07
E2	GY	7B	AX.89541032	3.38E+07	0.09	-3.78	3.85	0.07

E2	GY	7B	AX.89764916	1.28E+08	0.21	-2.46	3.47	0.09
E2	GY	7B	AX.89764916	1.28E+08	0.21	-2.46	3.47	0.09
E2	GY	7B	AX.89467037	1.25E+08	0.20	-2.59	3.55	0.09
E2	GY	7B	AX.89467037	1.25E+08	0.20	-2.59	3.55	0.09
E2	GPS	7B	AX.89627097	5.22E+06	0.17	-2.01	3.86	0.07
E2	GY	7B	AX.89652443	1.25E+08	0.20	-2.80	4.13	0.10
E2	GY	7B	AX.89652443	1.25E+08	0.20	-2.80	4.13	0.10
E2	GY	7B	AX.89616288	1.34E+08	0.25	-2.21	3.12	0.08
E2	GY	7B	AX.89440767	1.25E+08	0.20	-2.80	4.13	0.10
E2	GY	7B	AX.89593306	1.25E+08	0.20	-2.44	3.22	0.08
E2	GY	7B	AX.89573280	1.26E+08	0.21	-2.30	3.02	0.08
E2	GY	7B	AX.89504337	3.38E+07	0.24	-2.20	3.03	0.05
E2	GY	7B	AX.89740217	1.25E+08	0.20	-2.80	4.13	0.10
E2	GY	7B	AX.89740217	1.25E+08	0.20	-2.80	4.13	0.10
E2	GPM2	7B	AX.89596767	4.12E+08	0.16	-665.07	3.13	0.05
E2	GY	7B	AX.89428294	1.33E+08	0.25	-2.21	3.12	0.08
E2	GY	7B	AX.89668377	1.34E+08	0.23	-2.34	3.29	0.08
E2	GY	7B	AX.89668377	1.34E+08	0.23	-2.34	3.29	0.08
E2	GY	7B	AX.89373466	3.38E+07	0.09	-3.64	3.60	0.07
E2	GPS	7B	AX.89542111	7.22E+08	0.20	-1.68	3.10	0.05
E2	GY	7B	AX.89573280	1.26E+08	0.21	-2.30	3.02	0.08
E2	GY	7B	AX.89550633	1.25E+08	0.20	-2.60	3.63	0.09
E2	GY	7B	AX.89574468	1.26E+08	0.21	-2.34	3.12	0.08
E2	GY	7B	AX.89541032	3.38E+07	0.09	-3.78	3.85	0.07
E2	GY	7B	AX.89557410	1.34E+08	0.22	-2.45	3.47	0.09
E2	GY	7B	AX.89504337	3.38E+07	0.24	-2.20	3.03	0.05
E2	GPS	7B	AX.89365162	7.23E+08	0.20	-1.68	3.10	0.05
E2	GY	7B	AX.89616288	1.34E+08	0.25	-2.21	3.12	0.08
E2	GPS	7B	AX.89442633	7.22E+08	0.20	-1.71	3.20	0.05
E2	GPM2	7B	AX.89422373	3.38E+07	0.08	-945.60	3.42	0.07
E2	GY	7B	AX.89499678	1.37E+08	0.18	-2.61	3.41	0.05
E2	GY	7B	AX.89371264	1.34E+08	0.22	-2.45	3.52	0.09
E2	GY	7B	AX.89736261	1.26E+08	0.21	-2.34	3.12	0.08
E2	GPS	7B	AX.89732305	7.22E+08	0.29	-1.55	3.37	0.05
E2	GPS	7B	AX.89770281	7.22E+08	0.28	-1.54	3.29	0.05
E2	GPS	7B	AX.89732098	7.22E+08	0.28	-1.54	3.29	0.05
E2	GY	7B	AX.89509174	1.25E+08	0.20	-2.80	4.13	0.10
E2	GPM2	7B	AX.89457197	2.58E+06	0.05	-1191.72	3.20	0.08
E2	GY	7B	AX.89408294	1.25E+08	0.20	-2.44	3.22	0.08
E2	GY	7B	AX.89761603	1.28E+08	0.21	-2.31	3.10	0.08
E2	GY	7B	AX.89550633	1.25E+08	0.20	-2.60	3.63	0.09
E2	GY	7B	AX.89499678	1.37E+08	0.18	-2.61	3.41	0.05
E2	GY	7B	AX.89557410	1.34E+08	0.22	-2.45	3.47	0.09
E2	GY	7B	AX.89736261	1.26E+08	0.21	-2.34	3.12	0.08
E2	GPS	7B	AX.89400842	5.22E+06	0.08	-2.60	3.54	0.06
E2	GPS	7B	AX.89537754	7.22E+08	0.28	-1.54	3.29	0.05
E2	GY	7B	AX.89761603	1.28E+08	0.21	-2.31	3.10	0.08
E2	GPS	7B	AX.89711907	7.24E+08	0.20	-1.68	3.10	0.05
E2	GY	7B	AX.89630887	1.33E+08	0.25	-2.21	3.12	0.08
E2	GPM2	7B	AX.89478960	3.39E+07	0.07	-968.81	3.06	0.06

E2	GPS	7B	AX.89656107	7.22E+08	0.29	-1.55	3.37	0.05
E2	GY	7B	AX.89630887	1.33E+08	0.25	-2.21	3.12	0.08
E2	GPS	7B	AX.89572087	7.31E+08	0.27	-1.50	3.06	0.05
E3	GPM2	7B	AX.89668688	7.46E+06	0.15	1197.86	3.17	0.09
E3	GPM2	7B	AX.89763546	3.51E+07	0.15	-1304.09	3.68	0.07
E3	GPM2	7B	AX.89720905	3.51E+07	0.14	-1315.38	3.64	0.07
E3	GPM2	7B	AX.89558096	3.61E+07	0.14	-1278.98	3.49	0.06
E3	GPM2	7B	AX.89640625	4.92E+07	0.49	951.47	3.00	0.12
E3	GPM2	7B	AX.89748150	5.76E+07	0.49	954.97	3.15	0.12
E3	GPM2	7B	AX.89550945	6.30E+07	0.16	1219.89	3.01	0.10
E3	GPM2	7B	AX.89505337	6.49E+08	0.43	891.91	3.18	0.09
E3	GPM2	7B	AX.89699239	6.53E+08	0.38	897.14	3.03	0.12
E3	GPM2	7B	AX.89663338	6.88E+08	0.39	-900.82	3.02	0.08
E3	GPM2	7B	AX.89754941	6.88E+08	0.39	-900.82	3.02	0.08
E3	GPM2	7B	AX.89441665	6.88E+08	0.39	-900.82	3.02	0.08
E3	GPM2	7B	X.89739629_OT	7.11E+08	0.23	1143.21	3.62	0.09
E3	GPM2	7B	X.89397389_OT	7.11E+08	0.23	1143.21	3.62	0.09
E3	GPM2	7B	X.89505633_OT	7.11E+08	0.23	1143.21	3.62	0.09
E3	GPM2	7B	X.89708397_OT	7.11E+08	0.23	1145.46	3.63	0.09
E3	GPM2	7B	AX.89658728	7.21E+08	0.34	906.82	3.11	0.06
E3	GPM2	7B	AX.89697567	7.32E+08	0.37	855.34	3.03	0.01
E3	GPM2	7B	X.89561834_OT	7.34E+08	0.31	926.74	3.17	0.06
E3	GPM2	7B	X.89746395_OT	7.34E+08	0.33	903.58	3.08	0.06
E3	GPM2	7B	AX.89521287	7.40E+08	0.15	1196.88	3.17	0.04
E3	GPS	7B	AX.89653581	6.14E+08	0.29	2.60	3.40	0.11
E3	GPS	7B	AX.89591041	6.14E+08	0.54	2.04	3.17	0.08
E3	GPS	7B	AX.89607431	6.14E+08	0.29	2.62	3.51	0.11
E3	GPS	7B	AX.89496416	6.15E+08	0.53	2.41	4.27	0.11
E3	GPS	7B	AX.89661304	6.15E+08	0.51	2.18	3.57	0.10
E3	GPS	7B	AX.89335459	6.40E+08	0.33	2.08	3.16	0.07
E3	GPS	7B	AX.89416974	6.40E+08	0.33	2.08	3.16	0.07
E3	GPS	7B	AX.89416623	6.40E+08	0.32	2.13	3.30	0.07
E3	GPS	7B	AX.89635654	6.40E+08	0.33	2.03	3.05	0.07
E3	GPS	7B	AX.89645291	6.14E+08	0.29	2.70	3.60	0.11
E3	GPS	7B	AX.89768040	6.14E+08	0.28	2.57	3.28	0.11
E3	GPS	7B	AX.89492079	6.14E+08	0.28	2.78	3.72	0.12
E3	GPS	7B	AX.89440470	6.14E+08	0.28	2.57	3.28	0.11
E3	GPS	7B	AX.89702526	6.14E+08	0.53	2.26	3.82	0.10
E3	GPS	7B	AX.89483489	6.14E+08	0.29	2.69	3.63	0.11
E3	GPS	7B	AX.89514787	6.14E+08	0.29	2.56	3.33	0.11
E3	GPS	7B	AX.89470220	6.14E+08	0.29	2.82	3.97	0.12
E3	GPS	7B	AX.89469662	6.14E+08	0.29	2.54	3.27	0.11
E3	GPS	7B	AX.89610172	6.14E+08	0.28	2.64	3.39	0.11
E3	GPS	7B	AX.89596241	6.14E+08	0.28	2.57	3.28	0.11
E3	GPS	7B	AX.89727231	6.14E+08	0.29	2.70	3.60	0.11
E3	GPS	7B	AX.89641530	6.59E+07	0.39	-2.21	3.01	0.09
E3	GPS	7B	AX.89509563	6.64E+07	0.39	-2.25	3.10	0.10
E3	GPS	7B	AX.89347104	6.65E+07	0.40	-2.25	3.17	0.10
E3	GPS	7B	AX.89317429	5.48E+08	0.20	-2.61	3.16	0.07
E3	GPS	7B	AX.89481286	5.48E+08	0.26	-2.31	3.10	0.07

E3	GPS	7B	AX.89651804	5.51E+08	0.21	-2.58	3.16	0.07
E3	GPS	7B	AX.89652106	6.14E+08	0.47	2.04	3.06	0.09
E3	GPS	7B	AX.89700190	6.14E+08	0.28	2.57	3.28	0.11
E3	GPS	7B	AX.89721851	6.14E+08	0.28	2.78	3.72	0.12
E3	TKW	7B	AX.89718058	5.72E+07	0.25	0.98	3.29	0.08
E3	TKW	7B	X.89328028_OT	5.73E+07	0.25	0.95	3.11	0.07
E3	TKW	7B	AX.89347358	5.75E+07	0.10	1.58	3.89	0.14
E3	TKW	7B	AX.89356648	5.87E+07	0.26	0.96	3.17	0.08
E3	TKW	7B	AX.89649105	5.87E+07	0.23	1.15	3.80	0.11
E3	TKW	7B	AX.89556910	5.87E+07	0.23	1.15	3.80	0.11
E3	TKW	7B	AX.89708198	5.87E+07	0.23	1.15	3.80	0.11
E3	TKW	7B	AX.89608747	5.87E+07	0.23	1.15	3.80	0.11
E3	TKW	7B	AX.89585939	5.88E+07	0.23	1.15	3.80	0.11
E3	TKW	7B	AX.89750995	5.88E+07	0.23	1.15	3.80	0.11
E3	TKW	7B	AX.89380148	5.88E+07	0.23	1.15	3.80	0.11
E3	TKW	7B	AX.89523330	5.88E+07	0.23	1.15	3.80	0.11
E3	TKW	7B	AX.89668434	5.88E+07	0.22	1.18	3.94	0.11
E3	TKW	7B	AX.89493106	5.88E+07	0.23	1.15	3.80	0.11
E3	TKW	7B	AX.89721798	5.88E+07	0.23	1.15	3.80	0.11
E3	TKW	7B	AX.89722888	5.88E+07	0.23	1.15	3.80	0.11
E3	TKW	7B	AX.89318969	5.89E+07	0.22	1.21	4.16	0.12
E3	TKW	7B	AX.89352670	5.91E+07	0.22	1.21	4.16	0.12
E3	TKW	7B	AX.89734626	5.91E+07	0.22	1.21	4.16	0.12
E3	TKW	7B	AX.89405370	5.91E+07	0.22	1.19	3.98	0.12
E3	TKW	7B	AX.89397601	5.91E+07	0.22	1.19	3.98	0.12
E3	TKW	7B	AX.89332408	6.82E+07	0.10	-1.29	3.05	0.03
E3	TKW	7B	AX.89614057	5.93E+08	0.06	-1.70	3.17	0.03
E3	TKW	7B	AX.89365641	5.93E+08	0.06	-1.70	3.17	0.03
E3	TKW	7B	AX.89727940	5.93E+08	0.06	-1.70	3.17	0.03
E3	TKW	7B	AX.89597240	5.93E+08	0.06	-1.70	3.17	0.03
E3	TKW	7B	AX.89337309	5.94E+08	0.07	-1.55	3.07	0.02
E3	TKW	7B	AX.89619692	5.94E+08	0.07	-1.55	3.07	0.02
E3	TKW	7B	AX.89592310	5.94E+08	0.06	-1.69	3.17	0.03
E3	TKW	7B	AX.89511546	5.94E+08	0.07	-1.61	3.17	0.02
E3	TKW	7B	AX.89492490	5.94E+08	0.07	-1.50	3.03	0.01
E3	TKW	7B	AX.89752147	5.94E+08	0.08	-1.56	3.37	0.02
E3	TKW	7B	X.89413785_OT	6.04E+08	0.16	1.20	3.02	0.10
E3	TKW	7B	X.89667077_OT	6.04E+08	0.16	1.20	3.02	0.10
E3	TKW	7B	AX.89591239	6.12E+08	0.19	1.26	3.84	0.13
E3	TKW	7B	X.89421973_OT	6.12E+08	0.19	1.26	3.84	0.13
E3	TKW	7B	AX.89777369	6.91E+08	0.40	-0.83	3.04	0.03
E3	TKW	7B	AX.89476237	7.05E+08	0.09	-1.43	3.09	0.05
E3	TKW	7B	AX.89337734	7.06E+08	0.08	-1.71	3.47	0.05
E3	TKW	7B	AX.89490475	7.06E+08	0.08	-1.71	3.47	0.05
E3	TKW	7B	AX.89575481	7.06E+08	0.08	-1.71	3.47	0.05
E3	TKW	7B	AX.89344008	7.06E+08	0.07	-1.96	3.93	0.06
E3	TKW	7B	AX.89642094	7.07E+08	0.07	-1.72	3.27	0.05
E3	TKW	7B	AX.89606362	7.08E+08	0.07	-1.72	3.27	0.05
E3	TKW	7B	AX.89375450	7.22E+08	0.18	-1.03	3.01	0.01
E3	TKW	7B	AX.89646540	7.25E+08	0.09	-1.39	3.09	0.01

E3	TKW	7B	AX.89773642	7.25E+08	0.09	-1.45	3.26	0.01
E3	TKW	7B	AX.89624023	7.44E+08	0.26	0.93	3.04	0.09
E3	GY	7B	AX.89534547	1.17E+07	0.48	2.92	3.47	0.05
E3	GY	7B	AX.89578776	7.08E+08	0.40	-3.19	3.79	0.09
E3	GY	7B	AX.89621133	7.09E+08	0.48	-3.14	3.92	0.07
E3	GY	7B	X.89647317_OT	7.10E+08	0.26	3.24	3.06	0.05
E3	SPM2	7B	AX.89421417	8.53E+07	0.38	-20.53	3.25	0.07
E3	SPM2	7B	AX.89413060	8.53E+07	0.38	-20.53	3.25	0.07
E3	SPM2	7B	AX.89608641	8.55E+07	0.38	-20.53	3.25	0.07
E3	GY	7B	X.89739629_OT	7.11E+08	0.23	3.83	3.68	0.07
E3	GY	7B	X.89397389_OT	7.11E+08	0.23	3.83	3.68	0.07
E3	GY	7B	X.89505633_OT	7.11E+08	0.23	3.83	3.68	0.07
E3	GY	7B	X.89708397_OT	7.11E+08	0.23	3.95	3.88	0.07
E3	GY	7B	AX.89636895	7.15E+08	0.18	-3.64	3.07	0.05
E3	SPM2	7B	AX.89334838	8.58E+07	0.38	-20.53	3.25	0.07
E3	SPM2	7B	AX.89700399	8.70E+07	0.38	-20.53	3.25	0.07
E3	TKW	7B	AX.89763546	3.51E+07	0.15	1.28	3.68	0.09
E3	TKW	7B	AX.89720905	3.51E+07	0.14	1.29	3.63	0.09
E3	TKW	7B	AX.89567140	3.61E+07	0.09	1.45	3.17	0.12
E3	TKW	7B	AX.89558096	3.61E+07	0.14	1.25	3.47	0.09
E3	TKW	7B	AX.89570074	5.36E+07	0.25	0.99	3.29	0.08
E3	TKW	7B	AX.89653409	5.36E+07	0.25	0.97	3.15	0.08
E3	TKW	7B	AX.89476988	5.43E+07	0.25	0.99	3.29	0.08
E3	TKW	7B	AX.89484661	5.43E+07	0.25	0.99	3.29	0.08
E3	TKW	7B	AX.89373542	5.43E+07	0.25	0.99	3.29	0.08
E3	TKW	7B	AX.89376880	5.43E+07	0.25	0.99	3.29	0.08
E3	TKW	7B	AX.89544893	5.48E+07	0.25	0.99	3.29	0.08
E3	TKW	7B	AX.89479303	5.48E+07	0.25	0.93	3.01	0.08
E3	TKW	7B	AX.89523378	5.48E+07	0.25	0.98	3.26	0.08
E3	TKW	7B	AX.89707517	5.48E+07	0.25	0.98	3.26	0.08
E3	TKW	7B	AX.89774834	5.48E+07	0.25	0.98	3.26	0.08
E3	TKW	7B	AX.89509435	5.53E+07	0.25	0.96	3.15	0.08
E3	TKW	7B	AX.89356004	5.59E+07	0.25	1.08	3.88	0.09
E3	TKW	7B	X.89453230_OT	5.59E+07	0.25	1.01	3.38	0.08
E3	TKW	7B	X.89756228_OT	5.67E+07	0.23	0.99	3.21	0.08
E3	TKW	7B	X.89605256_OT	5.68E+07	0.25	0.99	3.31	0.07
E3	TKW	7B	X.89591686_OT	5.69E+07	0.23	0.96	3.10	0.08
E3	TKW	7B	X.89548248_OT	5.70E+07	0.25	0.98	3.29	0.08
E3	SPM2	7B	AX.89627785	8.70E+07	0.34	-21.05	3.14	0.07
E3	SPM2	7B	AX.89489197	8.70E+07	0.34	-21.05	3.14	0.07
E3	SPM2	7B	AX.89733748	7.42E+08	0.21	23.64	3.14	0.07
E4	GPM2	7B	AX.89573902	5.42E+08	0.43	472.34	3.15	0.05
E4	GPM2	7B	AX.89497580	6.06E+08	0.33	-481.30	3.12	0.08
E4	GPS	7B	AX.89761025	1.10E+06	0.09	2.64	3.51	0.04
E4	GPS	7B	AX.89390019	5.46E+08	0.27	1.73	3.01	0.05
E4	GPS	7B	AX.89473542	7.01E+08	0.37	1.60	3.39	0.04
E4	GPS	7B	X.89391320_OT	7.17E+08	0.08	-2.76	3.26	0.05
E4	GY	7B	AX.89699607	7.16E+08	0.16	2.05	3.30	0.05
E4	GY	7B	AX.89744325	7.16E+08	0.16	2.05	3.30	0.05
E4	GY	7B	AX.89615954	7.16E+08	0.17	1.96	3.12	0.05

E4	GY	7B	AX.89317860	7.16E+08	0.17	2.01	3.26	0.05
E4	GY	7B	AX.89558813	7.16E+08	0.16	2.05	3.30	0.05
E4	GY	7B	AX.89702387	7.16E+08	0.16	2.05	3.30	0.05
E4	GY	7B	AX.89660937	7.16E+08	0.14	2.23	3.44	0.05
E4	GY	7B	AX.89618851	7.16E+08	0.16	2.05	3.30	0.05
E4	GY	7B	AX.89740742	7.16E+08	0.16	2.05	3.30	0.05
E4	GY	7B	AX.89670129	7.17E+08	0.16	2.19	3.57	0.05
E4	GY	7B	AX.89392875	7.17E+08	0.16	2.05	3.30	0.05
E4	GY	7B	AX.89554739	7.17E+08	0.16	2.05	3.30	0.05
E4	GY	7B	AX.89317458	7.17E+08	0.16	2.05	3.30	0.05
E4	GY	7B	AX.89765675	7.16E+08	0.17	1.90	3.02	0.04
E4	GY	7B	AX.89588125	7.16E+08	0.17	1.90	3.02	0.04
E4	GY	7B	AX.89596685	7.16E+08	0.17	1.90	3.02	0.04
E4	GY	7B	AX.89649345	7.16E+08	0.17	1.90	3.02	0.04
E4	GY	7B	AX.89334868	7.16E+08	0.17	1.90	3.02	0.04
E4	GY	7B	AX.89593857	7.16E+08	0.17	1.90	3.02	0.04
E4	GY	7B	AX.89678787	7.16E+08	0.16	2.05	3.30	0.05
E4	SPM2	7B	AX.89379932	1.21E+08	0.31	-17.25	3.02	0.05
E4	SPM2	7B	AX.89591239	6.12E+08	0.19	-22.92	3.42	0.04
E4	SPM2	7B	X.89421973_OT	6.12E+08	0.19	-22.92	3.42	0.04
E4	SPM2	7B	AX.89599879	6.36E+08	0.20	19.29	3.10	0.07
E4	SPM2	7B	AX.89757077	6.80E+08	0.26	18.13	3.23	0.03
E4	SPM2	7B	AX.89635871	6.80E+08	0.26	18.13	3.23	0.03
E4	SPM2	7B	AX.89686954	6.80E+08	0.26	18.13	3.23	0.03
E4	SPM2	7B	AX.89723106	6.80E+08	0.22	18.44	3.02	0.03
E4	SPM2	7B	AX.89605954	6.80E+08	0.22	18.44	3.02	0.03
E4	SPM2	7B	AX.89403402	6.80E+08	0.26	18.13	3.23	0.03
E4	SPM2	7B	AX.89585439	6.80E+08	0.26	18.13	3.23	0.03
E4	SPM2	7B	AX.89433228	6.80E+08	0.24	19.86	3.66	0.04
E4	SPM2	7B	AX.89640891	6.80E+08	0.26	18.13	3.23	0.03
E4	SPM2	7B	AX.89375681	6.80E+08	0.26	18.13	3.23	0.03
E4	SPM2	7B	AX.89407406	6.80E+08	0.25	19.84	3.71	0.04
E4	SPM2	7B	AX.89651617	6.80E+08	0.25	19.84	3.71	0.04
E4	SPM2	7B	AX.89658160	6.80E+08	0.26	18.13	3.23	0.03
E4	SPM2	7B	AX.89625147	6.80E+08	0.26	18.13	3.23	0.03
E4	SPM2	7B	AX.89355415	6.80E+08	0.26	18.13	3.23	0.03
E4	SPM2	7B	AX.89440840	6.80E+08	0.26	18.13	3.23	0.03
E4	SPM2	7B	AX.89311971	6.80E+08	0.23	20.13	3.56	0.03
E4	SPM2	7B	AX.89611011	6.82E+08	0.30	-17.34	3.01	0.04
E4	SPM2	7B	X.89377909_OT	7.08E+08	0.09	31.73	3.98	0.07
E4	TKW	7B	AX.89709974	6.52E+08	0.14	1.32	3.50	0.05
E4	TKW	7B	AX.89531452	6.52E+08	0.13	1.31	3.23	0.06
E4	TKW	7B	AX.89312529	6.52E+08	0.13	1.31	3.23	0.06
E4	TKW	7B	AX.89563370	6.53E+08	0.24	1.05	3.58	0.06
E4	TKW	7B	AX.89742364	6.57E+08	0.43	0.83	3.08	0.07
E4	TKW	7B	AX.89480846	6.57E+08	0.47	0.83	3.09	0.04
E4	TKW	7B	AX.89423478	6.57E+08	0.47	0.83	3.09	0.04
E4	TKW	7B	AX.89777369	6.91E+08	0.40	-0.88	3.42	0.03
E4	TKW	7B	AX.89710206	7.05E+08	0.09	-1.42	3.12	0.04
E4	TKW	7B	AX.89337461	7.06E+08	0.13	-1.31	3.46	0.02

E4	TKW	7B	AX.89686472	7.06E+08	0.09	-1.54	3.59	0.04
E4	TKW	7B	AX.89534746	7.06E+08	0.13	-1.31	3.46	0.02
E4	TKW	7B	AX.89558471	7.06E+08	0.13	-1.31	3.46	0.02
E4	TKW	7B	AX.89636896	7.06E+08	0.13	-1.31	3.46	0.02
E4	TKW	7B	AX.89511616	7.06E+08	0.13	-1.31	3.46	0.02
E4	TKW	7B	AX.89703591	7.06E+08	0.13	-1.31	3.46	0.02
E4	TKW	7B	AX.89773610	7.06E+08	0.13	-1.31	3.46	0.02
E4	TKW	7B	AX.89711767	7.06E+08	0.13	-1.31	3.46	0.02
E4	TKW	7B	AX.89519084	7.06E+08	0.13	-1.31	3.46	0.02
E4	TKW	7B	AX.89447859	7.06E+08	0.13	-1.31	3.46	0.02
E4	TKW	7B	AX.89603717	7.06E+08	0.13	-1.31	3.46	0.02
E4	TKW	7B	AX.89638517	7.06E+08	0.13	-1.31	3.46	0.02
E4	TKW	7B	AX.89672290	7.06E+08	0.13	-1.31	3.46	0.02
E4	TKW	7B	AX.89685760	7.06E+08	0.13	-1.31	3.46	0.02
E4	TKW	7B	AX.89443247	7.06E+08	0.13	-1.31	3.46	0.02
E4	TKW	7B	AX.89458919	7.06E+08	0.13	-1.31	3.46	0.02
E4	TKW	7B	AX.89713023	7.06E+08	0.13	-1.31	3.46	0.02
E4	TKW	7B	AX.89454176	7.06E+08	0.13	-1.31	3.46	0.02
E4	TKW	7B	AX.89555837	7.06E+08	0.13	-1.31	3.46	0.02
E4	TKW	7B	AX.89545049	7.06E+08	0.13	-1.31	3.46	0.02
E4	TKW	7B	AX.89743598	7.06E+08	0.13	-1.31	3.46	0.02
E4	TKW	7B	AX.89432444	7.06E+08	0.09	-1.54	3.59	0.04
E4	TKW	7B	AX.89580997	7.06E+08	0.09	-1.54	3.59	0.04
E4	TKW	7B	AX.89495741	7.06E+08	0.09	-1.54	3.59	0.04
E4	TKW	7B	AX.89738534	7.06E+08	0.09	-1.54	3.59	0.04
E4	TKW	7B	AX.89759475	7.06E+08	0.09	-1.54	3.59	0.04
E4	TKW	7B	AX.89495772	7.06E+08	0.09	-1.54	3.59	0.04
E4	TKW	7B	AX.89507839	7.06E+08	0.09	-1.54	3.59	0.04
E4	TKW	7B	AX.89505673	7.06E+08	0.09	-1.54	3.59	0.04
E4	TKW	7B	AX.89337734	7.06E+08	0.08	-1.92	4.39	0.05
E4	TKW	7B	AX.89490475	7.06E+08	0.08	-1.92	4.39	0.05
E4	TKW	7B	AX.89575481	7.06E+08	0.08	-1.92	4.39	0.05
E4	TKW	7B	AX.89344008	7.06E+08	0.07	-2.05	4.35	0.05
E4	TKW	7B	AX.89642094	7.07E+08	0.07	-1.84	3.76	0.05
E4	TKW	7B	AX.89606362	7.08E+08	0.07	-1.84	3.76	0.05
E4	TKW	7B	AX.89528636	7.08E+08	0.08	-1.59	3.17	0.04
E4	TKW	7B	AX.89658728	7.21E+08	0.34	-0.97	3.72	0.04
E4	TKW	7B	AX.89772254	7.22E+08	0.29	-0.86	3.07	0.02
E4	TKW	7B	AX.89325530	7.24E+08	0.34	-1.03	4.49	0.03
E4	TKW	7B	AX.89663962	7.24E+08	0.17	-1.11	3.34	0.02
E4	TKW	7B	AX.89646540	7.25E+08	0.09	-1.38	3.13	0.01
E4	TKW	7B	AX.89629887	7.32E+08	0.09	1.40	3.05	0.04
E4	TKW	7B	AX.89362583	7.33E+08	0.09	1.40	3.05	0.04
E4	TKW	7B	AX.89343384	7.34E+08	0.14	1.52	5.05	0.07
E4	TKW	7B	AX.89557382	7.34E+08	0.17	1.28	4.13	0.06
E4	TKW	7B	AX.89564789	7.42E+08	0.08	1.62	3.98	0.07
E4	TKW	7B	AX.89624023	7.44E+08	0.26	0.98	3.37	0.09
E1	SPM2	7D	AX.89635835	1.24E+08	0.27	18.72	3.21	0.06
E1	SPM2	7D	AX.89366664	1.11E+08	0.22	20.49	3.32	0.06
E1	SPM2	7D	AX.89453861	7.07E+06	0.17	21.69	3.14	0.05

E1	SPM2	7D	AX.89749449	7.40E+06	0.19	20.58	3.08	0.05
E1	GY	7D	AX.89554204	1.00E+07	0.16	-3.79	4.42	0.07
E1	SPM2	7D	AX.89522121	6.58E+06	0.23	20.15	3.32	0.06
E1	SPM2	7D	AX.89564658	7.40E+06	0.19	20.80	3.09	0.05
E1	GY	7D	AX.89649617	6.21E+08	0.48	-2.52	3.67	0.07
E1	SPM2	7D	AX.89416780	6.59E+06	0.20	21.31	3.32	0.06
E1	SPM2	7D	AX.89336154	6.14E+06	0.21	19.50	3.02	0.05
E1	SPM2	7D	AX.89352255	7.40E+06	0.18	22.67	3.45	0.06
E1	SPM2	7D	AX.89692632	7.41E+06	0.18	21.26	3.14	0.05
E1	SPM2	7D	AX.89462288	6.33E+08	0.47	17.52	3.53	0.05
E1	SPM2	7D	AX.89382448	7.40E+06	0.18	22.09	3.30	0.06
E1	SPM2	7D	AX.89738802	7.40E+06	0.22	19.99	3.20	0.06
E1	GY	7D	AX.89554204	1.00E+07	0.16	-3.79	4.42	0.07
E1	GY	7D	AX.89532397	8.08E+07	0.48	2.84	4.52	0.09
E1	GY	7D	AX.89649617	6.21E+08	0.48	-2.52	3.67	0.07
E1	GY	7D	AX.89461697	1.01E+07	0.17	-3.21	3.44	0.05
E1	GY	7D	AX.89461697	1.01E+07	0.17	-3.21	3.44	0.05
E1	SPM2	7D	AX.89550211	1.12E+08	0.30	18.68	3.38	0.06
E1	SPM2	7D	AX.89314572	1.12E+08	0.29	17.80	3.09	0.05
E1	SPM2	7D	AX.89516643	6.18E+08	0.17	-22.21	3.21	0.05
E1	SPM2	7D	AX.89665942	6.49E+06	0.15	22.35	3.06	0.05
E1	SPM2	7D	AX.89314582	7.40E+06	0.20	20.21	3.12	0.05
E1	SPM2	7D	AX.89405910	1.23E+08	0.24	21.55	3.80	0.07
E1	GY	7D	AX.89532397	8.08E+07	0.48	2.84	4.52	0.09
E1	GPM2	7D	AX.89649617	6.21E+08	0.48	-696.83	4.15	0.08
E1	TKW	7D	AX.89427286	1.05E+07	0.27	-0.95	3.15	0.05
E1	TKW	7D	AX.89667192	7.55E+06	0.12	-1.29	3.07	0.05
E1	TKW	7D	AX.89487631	9.30E+06	0.08	-1.64	3.35	0.06
E1	TKW	7D	AX.89778237	5.00E+08	0.18	-1.18	3.45	0.06
E1	TKW	7D	AX.89723762	1.05E+07	0.28	-1.03	3.68	0.06
E2	SPM2	7D	AX.89446415	9.31E+06	0.10	19.73	3.08	0.05
E2	SPM2	7D	AX.89342989	9.31E+06	0.10	19.73	3.08	0.05
E2	SPM2	7D	AX.89469088	9.37E+06	0.10	19.73	3.08	0.05
E2	SPM2	7D	AX.89692131	9.38E+06	0.10	19.73	3.08	0.05
E2	GY	7D	AX.89399855	5.93E+08	0.06	-4.31	3.50	0.05
E2	SPM2	7D	AX.89674483	4.81E+08	0.08	22.13	3.33	0.06
E2	SPM2	7D	AX.89748353	8.62E+06	0.10	19.73	3.08	0.05
E2	SPM2	7D	AX.89557751	9.31E+06	0.10	19.73	3.08	0.05
E2	SPM2	7D	AX.89681082	8.74E+06	0.10	19.73	3.08	0.05
E2	SPM2	7D	AX.89675895	1.06E+07	0.10	19.73	3.08	0.05
E2	SPM2	7D	AX.89503214	9.31E+06	0.14	17.99	3.43	0.06
E2	SPM2	7D	AX.89336736	8.54E+06	0.10	19.73	3.08	0.05
E2	SPM2	7D	AX.89663921	8.82E+06	0.10	19.73	3.08	0.05
E2	SPM2	7D	AX.89462704	1.05E+07	0.10	19.73	3.08	0.05
E2	SPM2	7D	AX.89586022	1.06E+07	0.09	20.31	3.12	0.05
E2	SPM2	7D	AX.89341181	7.23E+07	0.08	23.09	3.42	0.06
E2	SPM2	7D	AX.89570399	8.81E+06	0.10	19.73	3.08	0.05
E2	SPM2	7D	AX.89576026	9.38E+06	0.07	24.02	3.32	0.06
E2	SPM2	7D	AX.89685073	9.30E+06	0.10	19.73	3.08	0.05
E2	SPM2	7D	AX.89772774	1.06E+07	0.10	19.73	3.08	0.05

E2	SPM2	7D	AX.89445045	5.76E+08	0.28	13.52	3.27	0.06
E2	SPM2	7D	AX.89757460	9.30E+06	0.10	19.73	3.08	0.05
E2	SPM2	7D	AX.89424922	9.30E+06	0.10	19.73	3.08	0.05
E2	SPM2	7D	AX.89618138	1.05E+07	0.10	19.73	3.08	0.05
E2	SPM2	7D	AX.89541512	8.60E+06	0.10	19.73	3.08	0.05
E2	SPM2	7D	AX.89539969	9.39E+06	0.10	19.73	3.08	0.05
E2	SPM2	7D	AX.89607855	8.62E+06	0.10	19.73	3.08	0.05
E2	SPM2	7D	AX.89540702	8.54E+06	0.10	19.73	3.08	0.05
E2	SPM2	7D	AX.89326407	8.48E+06	0.08	20.83	3.00	0.05
E2	SPM2	7D	AX.89621074	1.06E+07	0.10	19.73	3.08	0.05
E2	SPM2	7D	AX.89535367	8.75E+06	0.10	19.73	3.08	0.05
E2	SPM2	7D	AX.89583664	1.05E+07	0.10	19.73	3.08	0.05
E2	SPM2	7D	AX.89703385	9.35E+06	0.10	19.73	3.08	0.05
E2	SPM2	7D	AX.89504207	8.88E+06	0.10	19.73	3.08	0.05
E2	SPM2	7D	AX.89348006	1.06E+07	0.10	19.73	3.08	0.05
E2	SPM2	7D	AX.89585410	8.81E+06	0.08	21.25	3.11	0.05
E2	SPM2	7D	AX.89487402	1.06E+07	0.10	19.73	3.08	0.05
E2	SPM2	7D	AX.89723762	1.05E+07	0.28	15.16	4.02	0.07
E2	SPM2	7D	AX.89402693	9.31E+06	0.09	21.72	3.50	0.06
E2	SPM2	7D	AX.89424329	9.31E+06	0.10	19.73	3.08	0.05
E2	SPM2	7D	AX.89635792	8.63E+06	0.10	19.73	3.08	0.05
E2	SPM2	7D	AX.89761456	1.06E+07	0.10	19.73	3.08	0.05
E2	SPM2	7D	AX.89462288	6.33E+08	0.47	11.63	3.03	0.04
E2	SPM2	7D	AX.89427286	1.05E+07	0.27	16.48	4.57	0.08
E2	SPM2	7D	AX.89535442	9.38E+06	0.10	19.73	3.08	0.05
E2	SPM2	7D	AX.89350467	8.55E+06	0.10	19.73	3.08	0.05
E2	SPM2	7D	AX.89696917	8.54E+06	0.10	19.73	3.08	0.05
E2	SPM2	7D	AX.89497570	1.04E+07	0.10	19.73	3.08	0.05
E2	SPM2	7D	AX.89523493	6.33E+08	0.46	11.63	3.03	0.04
E2	SPM2	7D	AX.89711332	8.81E+06	0.10	19.73	3.08	0.05
E2	SPM2	7D	AX.89699716	9.38E+06	0.10	19.73	3.08	0.05
E2	SPM2	7D	AX.89451298	5.52E+08	0.13	19.57	3.86	0.07
E2	SPM2	7D	AX.89383710	8.48E+06	0.09	20.43	3.15	0.05
E2	SPM2	7D	AX.89720968	9.31E+06	0.10	19.73	3.08	0.05
E2	GY	7D	AX.89719591	5.78E+08	0.05	-4.76	3.65	0.08
E2	GY	7D	AX.89681358	5.94E+08	0.24	-2.35	3.35	0.04
E2	GY	7D	AX.89681358	5.94E+08	0.24	-2.35	3.35	0.04
E2	GY	7D	AX.89719591	5.78E+08	0.05	-4.76	3.65	0.08
E2	GY	7D	AX.89399855	5.93E+08	0.06	-4.31	3.50	0.05
E2	GY	7D	AX.89625992	5.78E+08	0.05	-4.76	3.65	0.08
E2	GY	7D	AX.89625992	5.78E+08	0.05	-4.76	3.65	0.08
E2	TKW	7D	AX.89427286	1.05E+07	0.27	-1.13	3.46	0.06
E2	TKW	7D	AX.89778237	5.00E+08	0.18	-1.28	3.30	0.06
E2	TKW	7D	AX.89723762	1.05E+07	0.28	-1.18	3.77	0.07
E2	TKW	7D	AX.89447242	7.45E+06	0.08	-1.72	3.03	0.05
E2	TKW	7D	AX.89596797	5.35E+08	0.25	-1.10	3.17	0.06
E2	GPS	7D	AX.89341474	5.77E+07	0.49	1.32	3.04	0.06
E3	GPS	7D	AX.89418550	2.12E+07	0.16	2.88	3.02	0.11
E3	GPS	7D	AX.89530418	9.45E+07	0.18	2.91	3.34	0.13
E3	TKW	7D	AX.89680463	5.32E+08	0.26	1.00	3.22	0.10

E3	SPM2	7D	AX.89743445	2.31E+08	0.24	-25.36	3.75	0.10
E3	SPM2	7D	AX.89480177	2.31E+08	0.24	-23.90	3.42	0.10
E3	SPM2	7D	AX.89717052	2.36E+08	0.17	-25.48	3.01	0.06
E3	SPM2	7D	AX.89454432	5.23E+08	0.33	23.17	3.03	0.03
E4	GPS	7D	AX.89638812	1.63E+08	0.48	1.45	3.03	0.07
E4	GPM2	7D	AX.89729164	5.07E+08	0.40	541.32	3.77	0.09
E4	GPM2	7D	AX.89606634	5.98E+08	0.10	725.23	3.02	0.04
E4	SPM2	7D	AX.89311376	1.62E+08	0.45	-16.63	3.28	0.06
E4	SPM2	7D	AX.89638812	1.63E+08	0.48	-18.19	3.81	0.07
E4	SPM2	7D	AX.89390893	4.45E+08	0.41	18.31	3.69	0.09
