# Supplementary material for: Wheat individual grain-size variance originates from crop development and from specific genetic determinism
Source: PLoS One. 2020 Mar 26;15(3):e0230689. doi: 10.1371/journal.pone.0230689 (PMC7098578; doi:10.1371/journal.pone.0230689)
Supplement: Attachment — Submitted filename: Response to Reviewers.docx [file pone.0230689.s011.docx]

Dear Aimin Zhang,

Please find attached our revised manuscript “Wheat individual grain-size variance originates from crop development and from specific genetic determinism” we submitted for review in PLoS ONE. As required the revised version is provided with apparent “track changes”.

As you requested, the phrase “data not shown” in the manuscript was deleted. Lines 250-252 were removed. Line 387 was replaced by reference to table S4. Lines 341-344 and lines 355 were replaced with respectively the exact p-values of the statistical test described in the Materials & Methods part (Lines 228-229) and the Pearson correlations coefficients.

We believe the points raised by the two reviewers led to a significant improvement of the manuscript. Below are the responses to specific reviewer comments. Reviewer comments are in italic.

*Reviewer #1: In this manuscript, authors used two large genetic wheat panels under two environments and investigated the individual grain size variances and yield components. They showed that the variance of individual grain size originates from the pre-flowering components of grain yield and also has a specific genetic basis. Moreover, they identified 64 QTLs with the effects on the variance of individual grain size, either dependently or dependently from the other yield components, suggesting that individual grain size variance could be used as a trait to be manipulated in wheat breeding practices.*

*Overall, this manuscript provides another layer of insight into the intra-genotypic variance of individual grain mass and its effect on TKW. The data is sufficient to support their conclusions. However, a few of minor points should be considered to improve the manuscript.*

*Specific comments:*

1. *Authors identified a total 64 OTLs for variance of individual grain size and listed their chromosome distribution but without their position. I think it is better to give a rough position in each chromosome either physically or genetically.*

**Authors response:** The physical positions of the 64 QTL were added on the table S4 as requested (Supplementary data, lines 872-875).

2. *Authors also mentioned they identified QTLs for yield components but did not show these QTLs. I think this information should be provided. Since extensive such QTLs have been reported, how many such QTLs are likely overlapping with reported?*

**Authors response:** The main objective of the present manuscript is to focus on GSV determinism. This subject has not been covered so far in the existing literature. To highlight the focus made on this trait, the choice was made not to identify QTL for other traits if they were not linked with GSV QTLs. The methodology used for QTL identification was therefore defined as follow: (i) identification of all QTL for GSV and (ii) identification of the previously identified GSV QTLs that were in colocalisation with yield components. Therefore, our results do not allow to make a comparison with existing literature about known genetic determinism of yield components, which could be the objective of a different manuscript. Nevertheless, the position of all SNPs associated with all traits (including yield components) are presented in Table S4 (see above). The Materials & Methods section was also improved in order to make the methodology used clearer for the reader (Line 254).

3. *Regarding to GSV, the 30 “driven” QTLs obviously contribute to the yield, how about 34 “specific” QTLs?*

**Authors response:** The main objective of the manuscript was to determine what are the determinants of GSV and not GSV effects on grain yield. Yet, grain yield, as an agronomic trait integrating the different yield components and fixed at the end of the crop cycle, cannot be considered as a putative biological determinant of GSV. We identified two types of contributions to GSV elaboration. First, the yield components that are fixed before the start of grain-filling (SPM2 and GPS) and thus before the construction of GSV. These two components are characteristics of the effects of the canopy structure on GSV. Second, TKW, although it is the final yield component, is only considered in our manuscript through the relationship between trait mean (TKW) and variance of the same trait (GSV) as discussed in the manuscript (Lines 530-540) (Wolc et al., 2012).

If the point is to understand the direct effects of GSV on grain yield, the only individual yield component that can be impacted through a causal relationship, will be the TKW. Following this argument, any relationship between GSV and grain yield, which is not triggered by TKW, should be purely artefactual. Therefore, looking for a colocalisation between specific QTL of GSV (that are following our definition, not in colocalisation with others yield components) and QTL of grain yield does not seem relevant since no biological meaning could be associated with. On the basis of the position of the significant SNPs presented in the added table S6, this argument is partially validated. Indeed, the search for significant SNPs related to grain yield within the positions of the 64 QTL has revealed that there was significant colocalisation with SNPs related with grain yield for only one specific QTL of GSV. In comparison, 14 out of the 30 “driven” QTL of GSV were in the same zone of interest as SNPs related of grain yield.

Considering that no direct effect of GSV on grain yield can be anticipated, the interest of the specific QTL of GSV goes beyond yield considerations. As discussed in the manuscript (Lines 582-588), specific QTL of GSV can be considered as a tool to manipulate grain quality without any impact on yield components. To clarify this point, a sentence was added in the discussion (Lines 564-568).

*The biological function of these specific QTLs should be discussed.*

**Authors response:** Following reviewer 1’s suggestion, we have conducted a gene ontology enrichment analysis, in order to identify the biological processes (BP) over-represented in the “specific” QTL compared to the “driven” QTL. The results are shown in the table 1 below. An enrichment of genes from six biological processes is observed in the “specific” QTL compare to the “driven” QTL. However, these coarsely described mechanism do not provide particular insight into the specific determinism of GSV. This observation is not surprising since causes of grain size variability are not elucidated even at a macroscopic ecophysiological scale. Among the putative mechanism, which could govern grain size variability within spike, the literature proposes (i) the delay of development between florets, (ii) architectural constraints or (iii) hormonal gradients within the spike. Gene ontology analysis does not provide sufficient insight into biological processes to link putative genes with such putative mechanism. Therefore, we do not think this analysis would add significant value to the manuscript and would bring us in this specific case to highly speculative discussion.

**Table 1. Gene ontology enrichment analysis**

| GO.ID | Biological process | Annotated number of genes in the specific QTL | Significant number of genes in the specific QTL different to the “driven” QTL | Fisher test (p-value) |
| --- | --- | --- | --- | --- |
| GO:0042545 | Cell wall modification | 49 | 8 | 5.70E-06 |
| GO:0006817 | Phosphate ion transport | 5 | 2 | 0.004 |
| GO:0006890 | Retrograde vesicle-mediated transport, Golgi to ER | 6 | 2 | 0.0059 |
| GO:0006801 | Superoxide metabolic processes | 12 | 2 | 0.0239 |
| GO:0000290 | Deadenylation-dependent decapping of nuclear-transcribed mRNA | 2 | 1 | 0.0404 |
| GO:0006379 | mRNA cleavage | 2 | 1 | 0.0404 |

Gene ontology (biological processes) was compared between specific QTL and “driven” QTL. Enrichment fisher test was performed with TopGO package on the proportion of biological processes presented in “specific” QTL compare to ‘driven” QTL. (Version: 2.34.0).

4. *The manuscript should be more concise, especially Abstract, Introduction and Discussion parts.*

**Authors response:** Improvement in the abstract, introduction and discussion parts were made, in order to avoid repetitions. Changes were made in the abstract (lines 27, 31-32, 35-40) and introduction (lines 106-124). To clarify some points, changes were made in the discussion (Lines 504-518, 564-568).

*Reviewer #2: Although this research seemed to be interesting, the manuscript should be further revised, especially the experiment desgin and data analysis, language, etc.*

*Introduction*

*p11: Line 108-113 and Line 114-122 seemed to be repetitive, so delete one of them or revise.*

**Authors response:** Changes in the introduction part were made (Lines 109-123) in accordance to this comment. More generally, the manuscript was checked again by the scientific editing service that proof checked the first version of the manuscript (www.rescript.co.nz)

*Experiment design:*

*The two different populations were used in two years under well-watered and water-deficit, in fact, each one population has been grown in one year under two treatments, so E1,E2 E3 and E4 should not be used to anlyze data. therefore recommend the authors to analyze the data under different treatment in one population, respcetively.*

**Authors response:** Considering the statistical methodologies used, two different populations of wheat genotypes were indeed used in two years under two distinct environmental conditions. In the analysis, we have annotated E1 and E2 as respectively the first population (Panel 2016) under two contrasting water conditions; and E3 and E4 as respectively the second population (Panel 2017) under two contrasting conditions. We analysed the two different populations separately in our statistical analysis. Reviewer’s 2 comment suggests discussing the QTL that are detected between the two environments of the same population or between the 2 populations would provide insight the robustness of these QTL. Following this suggestion, Table 4 was modified to emphasize the QTL found in common within each population for two environments and those found between two different populations. Results (lines 385-387) and Discussion sections (lines 504-518) were modified to provide an interpretation of the differences between common QTL found under an environmental (same population) or genotypic (two different populations) source of variation.

*Why there are no replicates? It is not clear.*

**Authors response:** Regarding the comment on the fact that we have no replicate in the 2016/2017 experiment, as described in the M&M section, the experimental design was based on an augmented design which is widely chosen when a large number of genotypes (312 genotypes) should be tested in a limited number of micro-plots (Elias *et al.,* 2016). Only four control genotypes were heavily replicated within the experimental setup in order to account for spatial heterogeneity, but the other genotypes were not replicated. The specificity of the design was considered in the spatial adjustment of the phenotypic data.

*Results*

*Many SNPs were found in different traits, but the significant SNP related trait was not pointed out.*

**Authors response:** The comment is similar to the second question addressed by the first reviewer, please find the complete results of all significant SNPs for GSV and yield components in the table S6 with the following information: Environment, Trait, Chromosome, Name of the SNP, Physical position, MAF, Effect, LOD score and Percentage of the total variance of each trait explained by the significant SNP (Supplementary data part, lines 892-899).

*The language should be further revised.*

**Authors response:** Language editing of the revised manuscript was again performed by a professional editor (Alexander Land, www.rescript.co.nz).

In conclusion, we believe the suggestions made by the reviewer allowed to improve the quality of our manuscript and help us to clarify some points that were not sufficiently described in the first version. We hope that the reviewers will consider that the manuscript in the present form will meet the requirements for publication in PLOS One.

Additional question to the editor:

Discussions during this revision process led us to the conclusion that the manuscript title could be improved. The present title is “Wheat individual grain-size variance originates from crop development and from specific genetic determinism” but would be clearer as:

**“Wheat individual grain-size variance originates from canopy structure and from specific genetic determinism”**

This proposition was not included in the present version of the manuscript to avoid confusion in the edition process but please give us also your opinion on this proposition.

Sincerely,

On behalf of the authors,

PhD Student Aurore Beral

Université Clermont Auvergne, INRAE, UMR 1095 GDEC

Clermont-Ferrand, France

References:

Elias, A.A., Robbins, K.R., Doerge, R.W., Tuinstra, M.R., 2016. Half a Century of Studying Genotype × Environment Interactions in Plant Breeding Experiments. Crop Science 56, 2090–2105. https://doi.org/10.2135/cropsci2015.01.0061

Wolc, A., Arango, J., Settar, P., Fulton, J.E., O’Sullivan, N.P., Preisinger, R., Habier, D., Fernando, R., Garrick, D.J., Hill, W.G., Dekkers, J.C.M., 2012. Genome-wide association analysis and genetic architecture of egg weight and egg uniformity in layer chickens: Genome-wide association analysis and genetic architecture of egg. Animal Genetics 43, 87–96. https://doi.org/10.1111/j.1365-2052.2012.02381.x
